# Physiology, Pathology and Relatedness of Human Tissues from Gene Expression Meta-Analysis

**DOI:** 10.1371/journal.pone.0001880

**Published:** 2008-04-02

**Authors:** Dario Greco, Panu Somervuo, Antonio Di Lieto, Tuomas Raitila, Lucio Nitsch, Eero Castrén, Petri Auvinen

**Affiliations:** 1 Institute of Biotechnology, University of Helsinki, Helsinki, Finland; 2 Neuroscience Center, University of Helsinki, Helsinki, Finland; 3 Dipartimento di Biologia e Patologia Cellulare e Molecolare, Università “Federico II”, Napoli, Italy; University of Giessen, Germany

## Abstract

**Background:**

Development and maintenance of the identity of tissues is of central importance for multicellular organisms. Based on gene expression profiles, it is possible to divide genes in housekeeping genes and those whose expression is preferential in one or a few tissues and which provide specialized functions that have a strong effect on the physiology of the whole organism.

**Results:**

We have surveyed the gene expression in 78 normal human tissues integrating publicly available microarray gene expression data. A total amount of 1601 genes were identified as selectively expressed in one or more tissues. The tissue-selective genes covered a wide range of cellular and molecular functions, and could be linked to 361 human diseases with Mendelian inheritance. Based on the gene expression profiles, we were able to form a network of tissues reflecting their functional relatedness and, to certain extent, their development. Using co-citation driven gene network technique and promoter analysis, we predicted a transcriptional module where the co-operation of the transcription factors E2F and NF-kappaB can possibly regulate a number of genes involved in the neurogenesis that takes place in the adult hippocampus.

**Conclusions:**

Here we propose that integration of gene expression data from Affymetrix GeneChip experiments is possible through re-annotation and commonly used pre-processing methods. We suggest that some functional aspects of the tissues can be explained by the co-operation of multiple transcription factors that regulate the expression of selected groups of genes.

## Introduction

The human body consists of numerous cell types that are highly organized into functional units constituting tissues and organs. Expression patterns of genes have been under selection for eons and, as a result, cell types and tissues differ from each other both morphologically and functionally. The mechanisms leading to the development, differentiation, and maintenance of tissues have been under intensive investigation by generations of scientists.

A generally accepted view of gene expression programs divides genes in two main categories: i) housekeeping genes that are virtually always expressed in every tissue and work to maintain basic cellular functions; and ii) genes whose expression is preferential in one or a few tissues and which provide specialized functions that have a strong effect on the physiology of the whole organism.

Compared to the housekeeping genes, tissue-specific genes have been described as longer [1], with longer introns [2], a lower GC content [3], and lower substitution rates at non synonymous sites [4]. Moreover, tissue-specific genes seem to evolve faster and they are more likely to be mutated in genetic diseases with Mendelian inheritance [5].

In terms of gene expression, tissue specificity can be addressed in strict terms of genes that are exclusively transcribed in only one particular tissue type, but there is evidence indicating that most tissues with similar function share many expression patterns. Therefore, the concept of tissue-selectivity, which considers those genes whose expression is enriched in one or more similar tissues [6], might be more useful. Affymetrix high-density oligonucleotide arrays [7] have been already used for investigating tissue-specific expression patterns [6], [8]. However, there are several problematic aspects in the GeneChip technology, related especially to the mis-annotation of many probes. Dai and collaborators [9] have observed that updating the probe annotation for all the Affymetrix chipsets affects a large number of the probe sets. More recently, it has been shown that updating the definitions of the Affymetrix probes leads to more precise and accurate results as compared with the original annotations provided by the manufacturer [10]. Re-annotation of the Affymetrix probes has been also shown to improve the cross-platform reproducibility and meta-analysis of independent microarray experiments [11].

The aim of this study was to investigate tissue-selective expression patterns, integrating publicly available gene expression data. A total of 195 images of Affymetrix GeneChips were collected from the GEO database (http://www.ncbi.nlm.nih.gov/geo/). All probes present on the chipset were re-annotated according to the latest release of the Entrez Gene database (http://www.ncbi.nlm.nih.gov/sites/entrezdbgene). After extended quality control and preprocessing, we explored the tissue-selective expression patterns.

## Results

### Identification of tissue-selective genes

We searched for genes expressed in a tissue-selective manner. A tissue-selectivity score was computed for each gene and used as a weight for the expression values. After permutation test we could identify 1601 genes selectively expressed in one or more tissues. About 35% of 1601 genes were selectively expressed only in one tissue, 20% were shared by two, and 13% by three tissues. Ten percent of the tissue-selective genes were shared by six or more tissues. The majority of tissue-selective genes shared by ten or more tissues were expressed in neural system tissues. The majority of the tissue selective genes were found in the immune system (32% of 1601), followed by central and peripheral nervous systems (17%), muscles (15%) and reproductive organs (9%). Altogether, the other categories accounted 27% of selective genes.

### Functions of tissue-selective genes

The 1601 tissue-selective genes covered a wide range of cellular and molecular functions as they could be annotated into 1694 distinct Biological Process, 1094 Molecular Function and 290 Cellular Component functional families from the three gene ontology classifications (File S1, tables 0.2, 0.3 and 0.4). The gene ontology classification revealed a suggestive distribution of the 1601 tissue-selective genes into functional families: 19% of them were classified in the Molecular Function family “signal transducer activity”, and about 8% in the group of “receptor binding” proteins. Moreover, when the same genes were grouped according to the Cellular Component ontology, about 18% were annotated under the family “extracellular region”.

The classification of the 1601 tissue-selective genes according to the Biological Process ontology also showed that about 16% were associated with the term “development”, and almost 14% to the term “immune response”.

The gene ontology annotation was also used to characterize the genes identified in each of the 78 tissues separately (details in File S1). For example, “blood coagulation”, “iron ion homeostasis”, “lipid metabolism”, and “gluconeogenesis” were found over-represented in liver with p<1.0E-4 (File S1, table 44.2). Similarly, “male gamete generation” and “spermatogenesis” were found in testis with p<1.0E-18 (File S1, table 55.2). Generally, the selective genes in all the analyzed tissues showed excellent correlation with the known physiological functions.

### Tissue-selective genes and human diseases

It has been suggested that slow-evolving housekeeping genes are underrepresented among disease genes, due to a higher chance of embryonic lethality when mutated [5]. The 1601 tissue-selective genes were enriched in disease-genes as they were linked to 361 diseases described in the OMIM database (http://www.ncbi.nlm.nih.gov/sites/entrezdbOMIM). We also observed that, in most of the cases, the 1601 tissue-selective genes are associated to pathological phenotype in the tissues or organs where they are found selectively expressed.

For instance, among the genes we found selectively expressed in fetal heart, GATA4 and NKX2.5 have been associated with Atrial Septal Defect 2 [12], [13]. In addition, mutations of the gene NKX2.5 have been described in Tetralogy of Fallot [14], [15] and in Atrial Septal Defect with Atrioventricular Conduction Defect [16], [17]. The placenta-selective gene RASA1 is reported in two diseases characterized by aberrations of blood vessels: the Parkes Weber Syndrome and Capillary Malformation-Arteriovenous Malformation [18]. Several muscle-selective genes have been described in a number of myopathies, as well as several gland-selective genes are associated with syndromes of the endocrine system and metabolic diseases. More extensive listing of diseases is available in the supplemental results (File S1, table 0.5).

### Tissue connectome

We wanted to investigate the hypothesis that tissues and organs sharing tissue-selective genes might present some degree of relatedness.

For this propose, we built a network of tissues that we named connectome. In the connectome, each node represented a tissue and the genes selectively expressed in two or more tissues formed the edges between the nodes. Each edge was thus associated with the number of shared genes.

**Figure 1:**
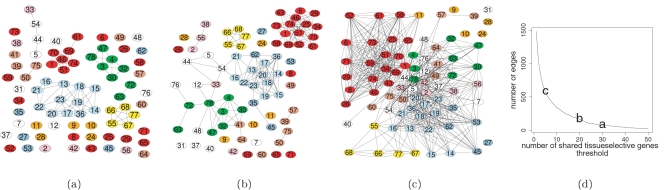
Tissue Connectome. Color code: Central Nervous System RED; Peripheral Nervous System ORANGE; Testis YELLOW; Muscles GREEN; immune cells LIGHT BLUE; Immune Organs DARK BLUE; Respiratory System PINK; Pancreas and Islets DARK PINK; Adrenal Gland and Adrenal Cortex LIGHT PINK; Thyroid and Fetal Thyroid SEPIA; Others WHITE. The edges have been drawn between tissue nodes sharing: a) 30 or more genes; b) 20 or more genes; c) 5 or more genes. d) the number of edges as a function of tissue-selective genes shared by two or more tissues. The tissue indexes are reported in Table 1.

The number of edges in the network was computed as a function of the number of shared genes between the tissues and three cutoff values (30 or more genes, 20 or more genes, and 5 or more genes) were chosen as representative of different degrees of connectivity (Figure 1d).

#### The 30 sharing genes connectome

It was possible to observe separate networks of seven central nervous system (CNS) tissues, five testis tissues, twelve immune cells, and six muscles (Figure 1a). Moreover, a chain-like connection was found between fetal lung – fetal liver – liver – kidney. The forebrain structures, consisting of telencephalic and diencephalic structures, clustered together. The central tissue in the CNS cluster was amygdala, which is consistent with the view that amygdala, rather than being a structural and developmental unit, is a collection of adjacent cell groups within the forebrain [19], [20]. Whole blood had extended connections with several circulating cells, such as T cells, B cells, natural killer cells, monocytes, and BDCA4-dendritic cells. On the other hand, tight connections were also formed between the cells resident in the bone marrow, such as early erythroid cells, B lymphoblasts, endothelial cells, and CD34 clones.

#### The 20 sharing genes connectome

Two larger sub-networks emerged, constituted respectively of CNS tissues, and immune cells and tissues, muscles, excretory organs, and thyroid (Figure 1b). Tonsil bridged the connection between immune cells and muscles. This makes sense as in the tonsil there are both myoepithelial and immune cells. Tonsil presented also connections with B cells, BDCA4-dendritic cells, and lymph node. Histologic studies of the tonsil showed that they are lymphoid structures consisting mainly of B-lymphocytes, but they are occupied also by T-lymphocytes, activated B-lymphocytes and other cells of the immune system. Tonsil shares histological features with lymph nodes as its cells are supported by a fine network of reticular fibers and high-endothelial venules function in the “homing” of circulating lymphocytes [21], [22]. During the fetal life massive erythropoiesis happens in several tissues such as the liver [23]. Fetal liver, in fact, connected early erythroid cells and CD105-endothelial cells that are precursors of circulating cells and which reside in the bone marrow after the birth. The testis tissues still appeared unconnected to any other tissue. The cluster of 15 CNS tissues included structures spanning from the spinal cord to telencephalic structures, forming the center of this network.

#### The 5 sharing genes connectome

All CNS tissues, including the olfactory bulb clustered together (Figure 1c). In addition, some peripheral nervous system structures joined this cluster. This sub-network was no longer distinct but shared links with other structures, particularly fetal tissues such as heart and lung. In addition, muscles shared links with the CNS cluster, which may be due to the innervation of the muscle samples as well as to the genes involved in ion homeostasis, which are expressed both in neurons and muscular cells. Fetal brain, hippocampus and olfactory bulb bridged the CNS cluster with other tissues. Neurogenesis, production of new neurons, continues in adult hippocampus and olfactory bulb and the genes expressed in these newborn neurons may give them immature characteristics, which are shared by fetal brain and other fetal tissues [24]. Hypothalamus and pituitary gland, which are anatomically connected, linked together.

### The hippocampus regulatory gene network

One of the goals of our analysis was to find clear correspondence between the tissue-selective gene expression programs and specific functions of tissues. Within the CNS, neurogenesis during adult life takes place in the hippocampus during normal and pathological conditions. The analysis of the connectome showed interesting links of the hippocampus with other anatomical structures where cell duplication and differentiation are known to happen. We wanted to test the hypothesis that genes selectively expressed in hippocampus would form a transcriptional network underlying this specific function. For this, we built a network of the hippocampus-selective genes based on their co-citation into the Pubmed database as well as the presence of specific transcription factor binding sites (TFBS) in their promoter regions ( Figure S1). The transcription factor NF-kappaB, which was not among the hippocampus-selective genes, presented an interesting topological position as it had connections with a number of hippocampus-selective genes (Figure S1). Detailed analysis of the promoters of the NF-kappaB interactors revealed the presence of a significantly conserved binding sequence for E2F and, 92–115 bp downstream, one for NF-kappaB (Figure S2). Screening the whole set of known human promoters, we found the E2F-NF-kappaB module in 1901 regulatory sequences, suggesting a common mechanism of transcriptional regulation. The gene ontology clustering of these genes showed significant over-representation of the families “nervous system development”, “cell adhesion”, “transmembrane receptor protein tyrosine kinase signaling pathway”, and “retinoic acid receptor activity” (details in File S2). In addition to the hippocampus-selective genes, also some fetal brain-selective, amygdala-selective, and prefrontal cortex-selective genes could be regulated by the E2F-NF-kappaB module. All these areas have been extensively investigated for neurogenesis [25].

## Discussion

We have integrated microarray data produced in several laboratories for exploring the tissue-selective expression patterns in 78 normal human tissues.

One of the interests about the tissue-specific genes concerns their functional role in normal and pathological conditions. We observed that the group of tissue-selective genes is enriched in “signal transducer activity”, “receptor binding”, and “extracellular region”, as well as “development” and “immune response” functional families. Our results are largely concordant with the findings of Winter and collaborators, who have reported that genes encoding secreted proteins highly correlate with tissue specificity [5]. Freilich and collaborators also reported that genes with tissue-specific expression patterns mainly encode for regulatory proteins involved in signal transduction activity [26]. In addition, they observed that the tissue-specific group is possibly enriched in transcription factors encoding genes [26]. We found only 59 transcription factor genes expressed in a tissue-selective manner. Accordingly, Yu and collaborators have reported that ubiquitously expressed transcription factors can combine with other factors contributing to tissue-specificity. In addition, they observed that individual transcription factors can participate in tissue-specific gene regulation by interacting with distinct partners in different tissues [27]. We propose that many transcription factors are evenly expressed in many tissues and that several stimuli mediated by tissue-specific signal transduction machineries mediate the functional activation of specific combinations of them at the protein level only in certain tissues and in defined temporal windows.

The connectome shows a novel and intriguing way to investigate the relatedness of human tissues and organs. While interpreting these results, it should be also taken into account that many human tissues have a complex architecture, as they consist of a certain number of specialized cells with variant transcriptional profiles. Microarrays can reliably detect cRNA species at the concentration of a few pico-molars, but it can be problematic to assign a certain gene expression event to the correct cell subpopulation of complex tissues. Nevertheless, we believe that the tissues should be always studied as functional entities and their global gene expression should be target of interest. When thinking of the liver, for instance, it should be considered that the identity of this organ is given by the combination of expression programs in several kinds of cells, more than just hepatocytes. Moreover, it is reasonable to think that in samples of many tissues some amount of blood cells are also present. This can easily explain the wide connections that circulating cells form with other kinds of anatomical structures in terms of shared gene expression. However, we observed a very tight intra-connectivity within the groups of nervous tissues, blood cells, testis tissues, and muscles, suggesting that the identity of these anatomical structures is determined by the differential expression of many genes. This is also evident from several clustering analyses we preformed on the data (details in File S3). The connectome of tissues also shows interesting interactions largely explained by the functional and morphological similarity of some tissues. It is the case, for instance, of the connection between the tonsil and lymph node. In other cases, the topological features of certain tissues in the network are suggestive of developmental mechanisms, as for the central position of the amygdala within the central nervous system connectome.

Increasing attention is being oriented to the inference of transcriptional regulatory networks from high throughput gene expression screenings. These approaches aim to link gene expression data to the activity of transcription factors in cause-effect models. Some effort has been already put also into the investigation of regulatory gene networks of tissue-specific genes having a central role in the physiology and development of specific anatomical structures [28], [29], [30]. We investigated in detail the expression patterns that might play a role in determining some functional aspect of the hippocampus. We computationally predicted a promoter module formed by conserved consensus motifs for the transcription factors E2F and NF-kappaB present on 1901 human known promoters. The gene ontology classification suggests that these genes are directly involved in neurogenesis and central nervous system development.

The E2F family of transcription factors, by interaction with several partners such as pRb, p107 or p130, are thought to regulate the cell cycle [31], [32] and trigger signals that also either promote cellular growth, cell cycle exit, or terminal differentiation in neurons [32], [33]. NF-kappaB has been described as playing an important role in synaptic activity and plasticity, neuroprotection, and in behavioral aspects of learning and memory [34]. Moreover, members of NF-kappaB family have been found to be expressed in areas of active neurogenesis in post-natal and adult mouse brains [35].

Altogether, these results expand our understanding of how gene expression programs determine the functional identity of human tissues.

## Methods

### Data collection

Total number of 195 Affymetrix HG-U133A CEL files was collected from the GEO database (http://www.ncbi.nlm.nih.gov/geo/) from 6 different data sets. All the gathered arrays had been hybridized to normal adult or fetal human tissues or cell types for a total of 78 different classes (Table 1). Data were selected according to the following criteria: i) all the experiments had been documented according to the MIAME standard; ii) all the arrays had been hybridized with samples isolated from human tissues and experiments were not carried out with cell lines; iii) all the samples came from healthy control subjects or from reference RNA samples; iv) the raw array images (CEL files) were available for download; and v) the Affymetrix chipset used for the hybridization was the human HGU-133A. A quality check of the data was performed using the recommendations of the manufacturer. Altogether, 195 sets of individual array data were used for further analysis.

**Table 1:** Summary of the data set analyzed.

tissue	category	Internal tissue code
Hippocampus	neural	1
BronchialEpitelia	respiratory	2
LimbMuscle	muscle	3
ExtraocularMuscle	muscle	4
Kidney	excretory	5
SubthalamicNucleus	neural	6
Skin	connective	7
GlobusPallidus	neural	8
CiliaryGanglion	neural	9
AtrioVentricularNode	neural	10
DRG	neural	11
Placenta	reproductive	12
721-BLymphoblasts	immune	13
PB-CD8TCells	immune	14
PB-CD4TCells	immune	15
BM-CD71EarlyErythroid	immune	16
PB-CD14Monocytes	immune	17
PB-CD56NKCells	immune	18
PB-CD19BCells	immune	19
BM-CD33Myeloid	immune	20
BM-CD105Endothelial	immune	21
BM-CD34	immune	22
PB-BDCA4DentriticCells	immune	23
SuperiorCervicalGanglion	neural	24
MedullaOblongata	neural	25
Pons	neural	26
Appendix	immune	27
TrigeminalGanglion	neural	28
TemporalLobe	neural	29
Tongue	muscle	30
UterusCorpus	reproductive	31
PsoasMuscle	muscle	32
FetalLung	respiratory	33
ParietalLobe	neural	34
Tonsil	immune	35
WholeBlood	circulatory	36
HBEC	circulatory	37
SalivaryGland	gland	38
AdrenalCortex	gland	39
Ovary	reproductive	40
Thyroid	gland	41
Lung	respiratory	42
FetalBrain	neural	43
Liver	excretory	44
LymphNode	immune	45
Amygdala	neural	46
Heart	muscle	47
Uterus	reproductive	48
Prostate	gland	49
Pancreas	gland	50
PrefrontalCortex	neural	51
CingulateCortex	neural	52
Thymus	immune	53
FetalLiver	excretory	54
Testis	reproductive	55
Trachea	respiratory	56
AdrenalGland	gland	57
SpinalCord	neural	58
Cerebellum	neural	59
PituitaryGland	gland	60
Thalamus	neural	61
BoneMarrow	immune	62
CardiacMyocytes	muscle	63
FetalThyroid	gland	64
OlfactoryBulb	neural	65
TestisGermCell	reproductive	66
TestisIntersitial	reproductive	67
TestisLeydigCell	reproductive	68
Hypothalamus	neural	69
OccipitalLobe	neural	70
CerebellumPeduncles	neural	71
SmoothMuscle	muscle	72
CaudateNucleus	neural	73
WholeBrain	neural	74
Islet	gland	75
Adipocytes	connective	76
TestiSeminiferousTubule	reproductive	77
FetalHeart	muscle	78

### Data Pre-processing

Sequence-based re-annotation of the Affymetrix probes on an HGU-133A chipset [9] according to the latest release of the Entrez Gene database was used (http://www.ncbi.nlm.nih.gov/sites/entrezdbgene). The expression values for each gene were calculated using the RMA algorithm [36].

### Tissue-selectivity analysis

A tissue-selectivity score was computed for each tissue-gene pair from the expression data matrix. Permutation test was performed to define a significance threshold. Details in Table S1.

### Gene ontology analysis

Fisher's exact test was performed in order to select over-represented gene ontology classes in the tissue-selective genes. The functional families presenting p<0.01 were considered as significantly represented.

### Gene network and promoter analysis

The hippocampus-selective genes were processed in the software Bibiosphere to build up gene networks based on their co-citation in the literature as well as the presence of TFBS for known transcription factors in their promoter regions (http://www.genomatix.de/products/BiblioSphere/). Because of the extensive connectivity of NF-KappaB within the network, the genes presenting a significant TFBS for NF-KappaB were selected for further analysis. The promoter sequences of these genes were retrieved using the Gene2Promoter software (http://www.genomatix.de/online help/help eldorado/Gene2Promoter Intro.html) and analyzed with FrameWorker (http://www.genomatix.de/online help/help gems/FrameWorker.html) to search for common models containing at least two TFBS. Finally, the significant model constituted by E2F and NF-KappaB was screened for in the whole set of known human promoters using ModelInspector (http://www.genomatix.de/online help/help fastm/modelinspector help.html).

## Supporting Information

Table S1 (0.06 MB DOC)

Figure S1 (0.12 MB PDF)

Figure S2 (0.04 MB PDF)

File S1 (7.50 MB PDF)

File S2 (0.82 MB PDF)

File S3 (0.17 MB PDF)
