# Supplementary material for: Physiology, Pathology and Relatedness of Human Tissues from Gene Expression Meta-Analysis
Source: PLoS One. 2008 Apr 2;3(4):e1880. doi: 10.1371/journal.pone.0001880 (PMC2268968; doi:10.1371/journal.pone.0001880)
Supplement: Table S1 — (0.06 MB DOC) [file pone.0001880.s001.doc]

**D. Greco et al. – SUPPLEMENTARY TEXT 1**

***Data collection***

The largest amount of raw data was retrieved from the GEO dataset GDS596 [2].

Retrieving microarray data from public repositories can present several difficulties, as many experiments have not been fully documented. Moreover, we could not use many datasets present in GEO database because only the tables with the normalized values are available. In these cases, the re-annotation of the probes is not possible and, in addition, outdated experiments are very often normalized using methods no longer considered reliable.

Integration of microarray data is increasingly becoming a need in the post-genomics era. For this reason we believe that sharing of raw data should be required for the publication of new microarray datasets. We retrieved the largest amount of raw data from the GEO dataset GDS596. A weakness of this dataset is given by the fact that each tissue is hybridized to two Affymetrix GeneChips. This dataset was originally reported by Su and collaborators, who have investigated the gene expression of human and mouse tissues (both healthy and diseased tissues), preprocessing the Affymetrix GeneChip data by using the MAS 5 algorithm [2]. This methodology presents some inaccuracies, especially because each array is normalized separately from the others, and because the information about the hybridization of MM probes is taken into account [3-6]. However, the GDS596 dataset is still considered a reliable tool for surveying tissue gene expression, as is currently used as a reference in several public databases [7, 8].

We believe that the bias given by the limited number of replicates per tissue can be largely corrected by processing the data using a model-based method that integrates all the data through the parameterization of the hybridization features into a linear model.

***Data Pre-processing***

There are several problematic aspects in the GeneChip technology, related especially to the mis-annotation of many probes. Dai and collaborators [9] have observed that updating the probe annotation for most of the Affymetrix human, mouse and rat genomes affects a large number of the probe sets. As a consequence, the genes identified as differentially expressed using the original and updated probe definition show only 50% overlap. Regarding the HG-U133A chipset used in this study, it has been shown that 14.4% of probe sets have an “unreliable representative public ID”, 34.2% present “UniGene redundancy”, and 36% of the probe sets contain “probe(s) with multiple UniGene hit”. Moreover, 16.3% have “probe(s) with multiple genome hits”, and 3.6% include “probe(s) with no known target”. These results convinced several researchers of the necessity to use some re-annotation of the HG-U133A chipset. More recently, it has been shown that updated definitions of the Affymetrix probes lead to more precise and accurate results as compared with the original annotations provided by the manufacturer [10]. Currently, several re-annotation methods are available allowing the probes to be mapped to genes, transcripts, or even exons sequences stored in public databases [11, 12]. However, exon-based re-annotation leads to decreased precision and increased variance in estimating gene expression, probably due to the smaller number of probes that map to each exon [10].

Sequence-based re-annotation of the Affymetrix probes on an HGU-133A chipset [13] according to the latest release of the Entrez Gene database was used [11]. During the re-annotation process, there is no loss of information, but each single oligonucleotide probe is re-assigned to the correct gene. A few probes are nevertheless eliminated because they do not recognize any transcript or they have been designed for matching the antisense sequence of a given transcript. However, re-annotation results in a reduction in the number of probe sets, as all the probes for a given transcript are grouped together. In the original set, the Affymetrix GeneChip HGU-133A contains 22283 probe sets. Of these, many are thought to hybridize with different portions of the same transcripts. After re-annotation, it is possible to count as many as 12201 new probe sets that contain more probes than the original ones, and are definitely more reliable and specific. Moreover, a meta-annotation package for the R environment was created using BioConductor facilities [14] in order to link the Entrez Gene IDs to the major biological databases, such us Geneontology [15], KEGG Pathways [16, 17], and PubMed [18]. The meta-annotation package is available from the authors upon request. Preprocessing was performed using the software R [19] and the package BioConductor [20]. The expression values for each gene were calculated using the RMA algorithm [20] implemented in the package Affy [21]. The RMA method as described originally by Irizarry et al. [20] allows robust estimation of inter-array variability. It uses information from multiple arrays for normalizing the dataset (through quantile normalization, the data are forced to have the same distribution) and fitting a linear model for each probe set across all the arrays of the dataset. More recently, the successful use of a set of biologically independent Affymetrix GeneChips for training the RMA model has been described [21].

We believe that estimation of the probe effect and the estimation of the array effect across our 195 arrays is accurate and that, through the RMA model, data integration becomes possible to a reasonable extent.

***Tissue-selectivity analysis***

A tissue-selectivity score *sij* was computed for each tissue-gene pair from the expression data matrix

(1),

where *xij* is the normalized expression value of the gene *i* in the tissue *j* and *wi* is a gene-specific weight. Normalization of expression values was performed gene-wise for RMA normalized expression values *yij* so that the maximum value from N tissues was set to 1 for each gene *i*:

(2),

The gene-specific weight *wi* measures the tissue selectivity of the gene according to Yanai et al. [24]:

(3).

The score s in Equation (1) is a combination of normalized expression value *xij* and the tissue-selectivity index of the gene with range of values between zero and one. In order to obtain a large value for a tissue-gene pair, it is required that the gene is highly expressed in a tissue (relative to the other tissues) as well being tissue-selective. A maximum value occurs if there is only one peak in the expression profile of a gene over all tissues. A permutation test was performed to define a significance threshold for the tissue-selectivity score. Samples of 78 random tissue-gene pairs were taken from the original data matrix.

The tissue-selectivity score was computed for each of these and the distribution of the scores was estimated based on 100.000 samples. The cutoff point was selected as the value above which 5% of the random scores. The genes having the original tissue-gene pairs above this cutoff were labeled as tissue-selective.

The identification of tissue-specific gene expression patterns has been the focus of a number of studies [24 – 31]. Hsiao et al. [25] examined 19 tissues selecting about 21% of 7000 tissue-specific genes, Saito-Hisaminato et al. [26] selected 17% of about 27000 tissue-specific genes in 29 tissues, and Liang et al. [27] selected 14% of about 27000 tissue-selective genes in 97 tissues. In this study, about 13% of all the genes analyzed emerged as selective in one or more of the 78 tissues screened. The similarity of the tissue-selective gene rate over all the genes is surprisingly consistent when comparing the results presented here with other reports cited above, considering the differences in the microarray platforms, in the preprocessing methodologies, and in the methods employed for pinpointing tissue-related genes. Certain publications using Shannon’s entropy measure [32] and Akaike’s information criterion (AIC)-based outlier detection method [33, 34] are discordant with our results in terms of the tissue-selectivity rate. In those publications, the proportion of tissue-selective genes reported was close to 70% of all the analyzed genes. The AIC-based outliers detection method resulted in 6636 tissue-selective genes out of 12201 (54%) in our dataset. Although the method [33] works well in clear-cut data sets (clear peaks observed in the expression profile), several problematic expression profiles were misinterpreted. However, 1521 out of 6636 genes were present in our group of 1601 tissue-selective genes, indicating the high consistency (95%) of the two sets of results.

***Gene ontology analysis***

Fisher’s exact test was performed in order to select over-represented gene ontology classes in the tissue-selective genes compared to all the genes represented on the re-annotated chipset. The functional families presenting p-value < 0.01 were considered as significantly represented. The R implementation of the algorithm, included in the GOstats package, was used [35].

***Gene network and promoter analysis***

The hippocampus-selective genes were processed in the software Bibiosphere to build up gene networks based on their co-citation in the literature as well as the presence of TFBS for known transcription factors in their promoter regions (http://www.genomatix.de/products/BiblioSphere/). Because of the extensive connectivity of NF-KappaB within the network, the genes presenting a significant TFBS for NF-KappaB were selected for further analysis. The promoter sequences of these genes were retrieved using the Gene2Promoter software (http://www.genomatix.de/online help/help eldorado/Gene2Promoter Intro.html) and analyzed with FrameWorker (http://www.genomatix.de/online help/help gems/FrameWorker.html) to search for common models containing at least two TFBS. Finally, the significant model constituted by E2F and NF-KappaB was screened for in the whole set of known human promoters using ModelInspector (http://www.genomatix.de/online help/help fastm/modelinspector help.html).

***Figure Legends***

***Supplemental Figure 1. Knowledge-based network of the hippocampus-selective genes.***

The nodes represent the genes. In BLUE, the hippocampus-selective genes; in WHITE, the transcription factors that interact with the hippocampus specific genes. The BLACK edges indicate co-citation of two genes in the PubMed database; the GREEN edges indicate the presence of a significant TFBS on the promoter of the given gene for the specific interacting transcription factor.

***Supplemental Figure 2. The E2F - NF-KappaB module.***

In A) a detailed description of the module is shown: the name of the transcription factor matrix, the DNA strand that it is predicted to bind, and the distance between the two elements of the module. In B) the module is shown as binding a set of four related promoters: in PINK and in BLUE are shown, respectively, the binding sites for E2F and for NF-KappaB and their position on each promoter. The PINK arrows indicate the transcription starting sites.

***References***

1. Barrett T, Edgar R: Mining microarray data at NCBI’s Gene Expression Omnibus (GEO)*. Methods in molecular biology (Clifton, N.J.) 2006, 338:175–190.
2. Su AI, Wiltshire T, Batalov S, Lapp H, Ching KA, Block D, Zhang J, Soden R, Hayakawa M, Kreiman G, Cooke MP, Walker JR, Hogenesch JB: A gene atlas of the mouse and human protein-encoding transcriptomes. Proceedings of the National Academy of Sciences of the United States of America 2004, 101(16):6062–6067.
3. Lee I, Dombkowski AA, Athey BD: Guidelines for incorporating non-perfectly matched oligonucleotides into target-specific hybridization probes for a DNA microarray. Nucleic acids research 2004, 32(2):681–690.
4. Naef F, Hacker CR, Patil N, Magnasco M: Empirical characterization of the expression ratio noise structure in high-density oligonucleotide arrays. Genome biology 2002, 3(4):RESEARCH0018.
5. Naef F, Hacker CR, Patil N, Magnasco M: Characterization of the expression ratio noise structure inhigh-density oligonucleotide arrays. Genome biology 2002, 3:PREPRINT0001.
6. Naef F, Lim DA, Patil N, Magnasco M: DNA hybridization to mismatched templates: a chip study. Physical review.E, Statistical, nonlinear, and soft matter physics 2002, 65(4 Pt 1):040902.
7. GNF SymAtlas. Database on gene function and structure, from the Genomics Institute of the Novartis Research Foundation. [http://symatlas.gnf.org/SymAtlas/].
8. BioMart Project [http://www.biomart.org/].
9. Dai M, Wang P, Boyd AD, Kostov G, Athey B, Jones EG, Bunney WE, Myers RM, Speed TP, Akil H, Watson SJ, Meng F: Evolving gene/transcript definitions significantly alter the interpretation of GeneChip data. Nucleic acids research 2005, 33(20):e175.
10. Sandberg R, Larsson O: Improved precision and accuracy for microarrays using updated probe set definitions. BMC Bioinformatics 2007, 8:48.
11. Entrez Gene [http://www.ncbi.nlm.nih.gov/sites/entrez?db=gene].
12. Ensembl [http://www.ensembl.org].
13. Custom CDFs [http://brainarray.mbni.med.umich.edu/Brainarray/Database/CustomCDF/CDF download.asp].
14. Zhang J: AnnBuilder: Bioconductor annotation data package builder 2006.
15. Harris MA, Clark J, Ireland A, Lomax J, Ashburner M, Foulger R, Eilbeck K, Lewis S, Marshall B, Mungall C, et al.: The Gene Ontology (GO) database and informatics resource. Nucleic acids research 2004, 32(Database issue):D258–61
16. Kanehisa M, Goto S, Hattori M, Aoki-Kinoshita KF, Itoh M, Kawashima S, Katayama T, Araki M, Hirakawa M: From genomics to chemical genomics: new developments in KEGG. Nucleic acids research 2006, 34(Database issue):D354–7
17. Arakawa K, Kono N, Yamada Y, Mori H, Tomita M: KEGG-based pathway visualization tool for complex omics data. In silico biology 2005, 5(4):419–423.
18. Wheeler DL, Barrett T, Benson DA, Bryant SH, Canese K, Chetvernin V, Church DM, DiCuccio M, Edgar R, Federhen S, et al.: Database resources of the National Center for Biotechnology Information. Nucleic acids research 2006, 34(Database issue):D173–80.
19. The Comprehensive R Archive Network [http://cran.r-project.org/].
20. The BioConductor Project [http://www.bioconductor.org/].
21. Irizarry RA, Hobbs B, Collin F, Beazer-Barclay YD, Antonellis KJ, Scherf U, Speed TP: Exploration, normalization, and summaries of high density oligonucleotide array probe level data. Biostatistics 2003, 4(2):249–264.
22. Irizarry RA, Gautier L, Bolstad BM, Miller C: affy: Methods for Affymetrix Oligonucleotide Arrays 2006.
23. Katz S, Irizarry RA, Lin X, Tripputi M, Porter MW: A summarization approach for Affymetrix GeneChip data using a reference training set from a large, biologically diverse database. BMC Bioinformatics 2006, 7(464).
24. Yanai I, Benjamin H, Shmoish M, Chalifa-Caspi V, Shklar M, Ophir R, Bar-Even A, Horn-Saban S, Safran M, Domany E, Lancet D, Shmueli O: Genome-wide midrange transcription profiles reveal expression level relationships in human tissue specification. Bioinformatics 2005, 21(5):650–659.
25. Hsiao LL, Dangond F, Yoshida T, Hong R, Jensen RV, Misra J, Dillon W, Lee KF, Clark KE, Haverty P, et al.: A compendium of gene expression in normal human tissues. Physiological genomics 2001, 7(2):97–104.
26. Saito-Hisaminato A, Katagiri T, Kakiuchi S, Nakamura T, Tsunoda T, Nakamura Y: Genome-wide profiling of gene expression in 29 normal human tissues with a cDNA microarray. DNA research : an international journal for rapid publication of reports on genes and genomes 2002, 9(2):35–45.
27. Liang S, Li Y, Be X, Howes S, Liu W: Detecting and profiling tissue-selective genes. Physiological genomics 2006, 26(2):158–162.
28. Schadt EE, Edwards SW, GuhaThakurta D, Holder D, Ying L, Svetnik V, Leonardson A, Hart KW, Russell A, Li G, Cavet G, Castle J, McDonagh P, et al.: A comprehensive transcript index of the human genome generated using microarrays and computational approaches. Genome biology 2004, 5(10):R73.
29. Shyamsundar R, Kim YH, Higgins JP, Montgomery K, Jorden M, Sethuraman A, van de Rijn M, Botstein D, Brown PO, Pollack JR: A DNA microarray survey of gene expression in normal human tissues. Genome biology 2005, 6(3):R22.
30. Amatschek S, Koenig U, Auer H, Steinlein P, Pacher M, Gruenfelder A, Dekan G, Vogl S, Kubista E, Heider KH, Stratowa C, Schreiber M, Sommergruber W: Tissue-wide expression profiling using cDNA subtraction and microarrays to identify tumor-specific genes. Cancer research 2004, 64(3):844–856.
31. Huminiecki L, Lloyd AT, Wolfe KH: Congruence of tissue expression profiles from Gene Expression Atlas, SAGEmap and TissueInfo databases. BMC genomics [electronic resource] 2003, 4:31.
32. Schug J, Schuller WP, Kappen C, Salbaum JM, Bucan M, Stoeckert J C J: Promoter features related to tissue specificity as measured by Shannon entropy. Genome biology 2005, 6(4):R33.
33. Kadota K, Nishimura S, Bono H, Nakamura S, Hayashizaki Y, Okazaki Y, Takahashi K: Detection of genes with tissue-specific expression patterns using Akaike’s information criterion procedure. Physiological genomics 2003, 12(3):251–259.
34. Kadota K, Ye J, Nakai Y, Terada T, Shimizu K: ROKU: a novel method for identification of tissue-specific genes. BMC bioinformatics 2006, 7:294.
35. Gentleman R: Using GO for statistical analyses, Heidelberg: Physica Verlag. Compstat 2004 Proceedings in Computational Statistics 2004 :171–180.
