# Supplementary figures and images for: Physiology, Pathology and Relatedness of Human Tissues from Gene Expression Meta-Analysis
Source: PLoS One. 2008 Apr 2;3(4):e1880. doi: 10.1371/journal.pone.0001880 (PMC2268968; doi:10.1371/journal.pone.0001880)

# Supplemental Figure 1

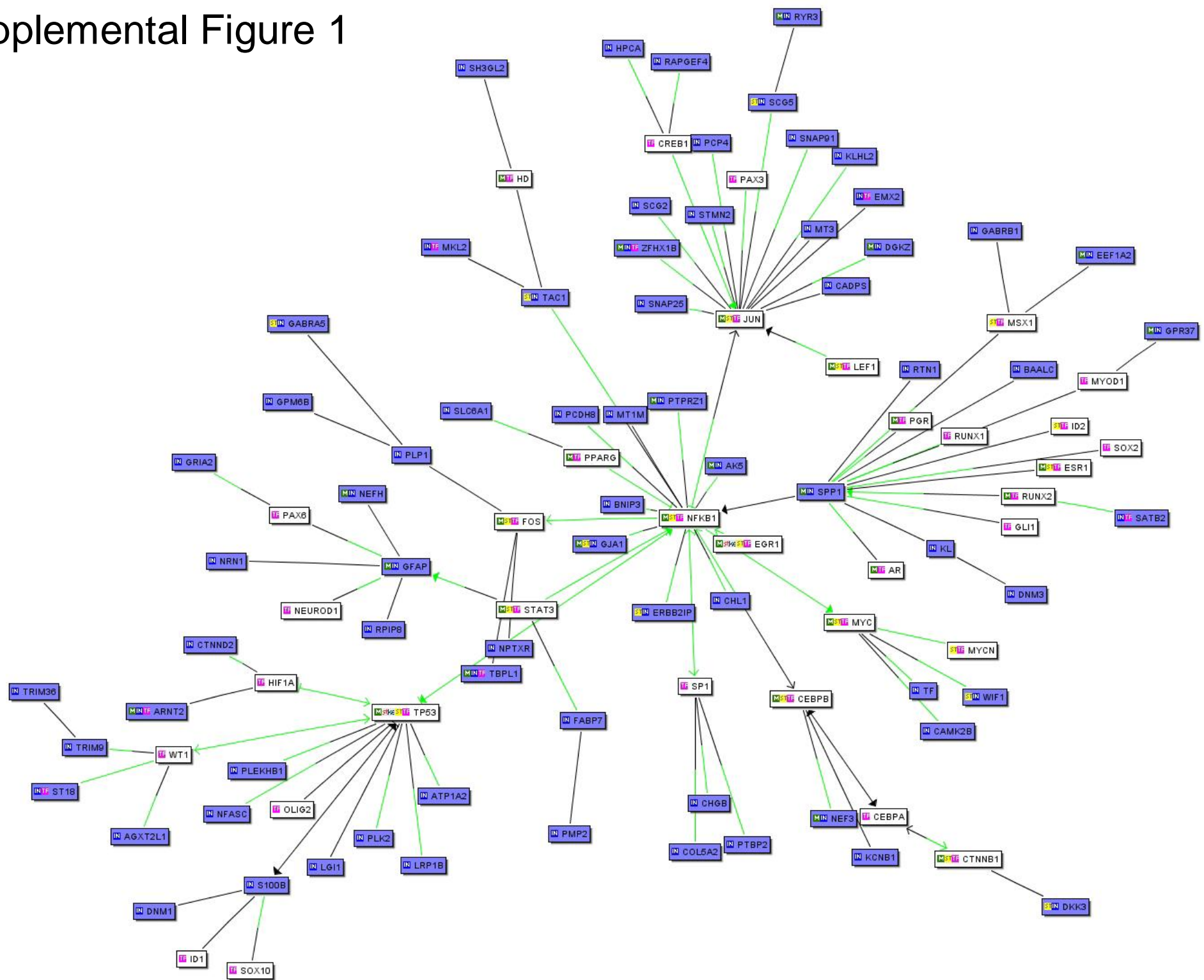

Supplement: Figure S1 — (0.12 MB PDF) [file pone.0001880.s002.pdf]
