# Supplementary material for: Physiology, Pathology and Relatedness of Human Tissues from Gene Expression Meta-Analysis
Source: PLoS One. 2008 Apr 2;3(4):e1880. doi: 10.1371/journal.pone.0001880 (PMC2268968; doi:10.1371/journal.pone.0001880)

Supplemental Figure 2

A

	Element type	Name	Strand	Parameters	Distance to next element
1	Matrix	V$E2FF	(+)	Min. core sim.: 0.750<br>Min. matrix sim.: optimized	92 to 115 bp
2	Matrix	V$NFKB	(-)	Min. core sim.: 0.750<br>Min. matrix sim.: optimized	---

B

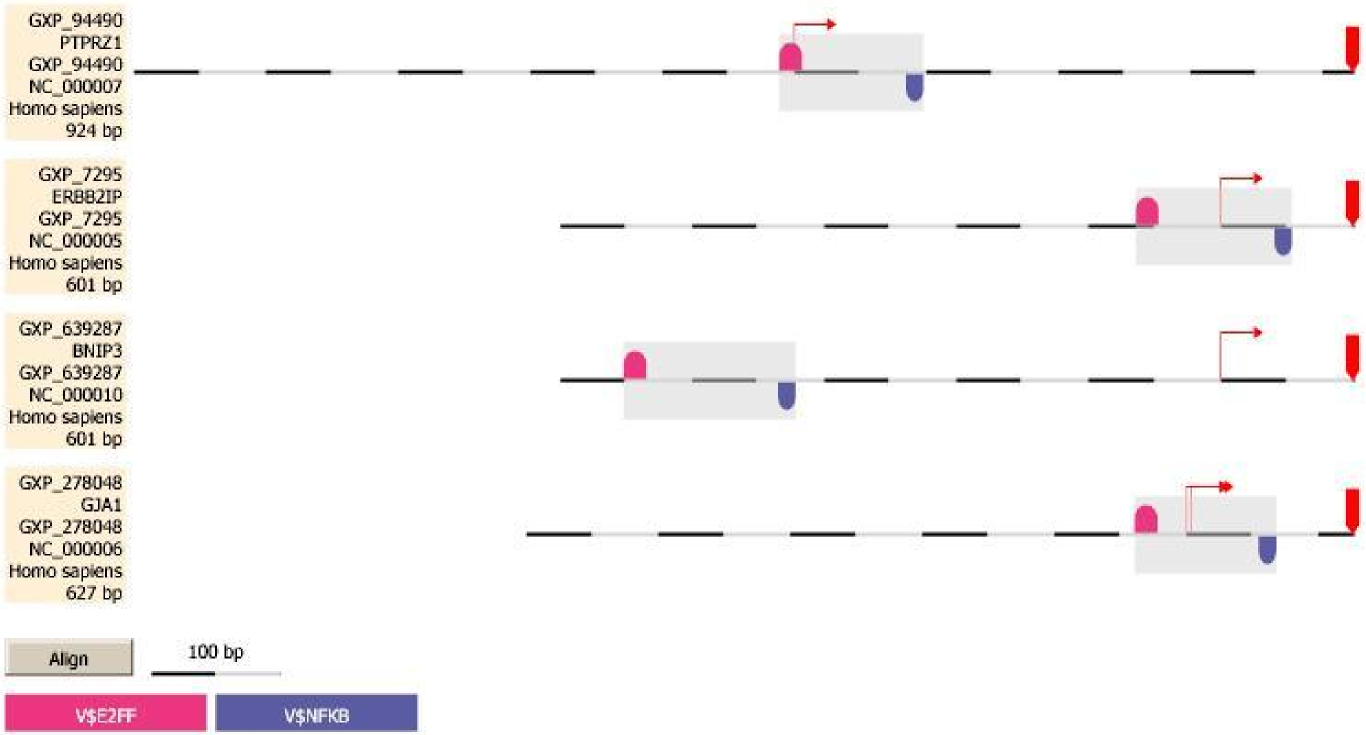

Supplement: Figure S2 — (0.04 MB PDF) [file pone.0001880.s003.pdf]
