# Supplementary material for: Physiology, Pathology and Relatedness of Human Tissues from Gene Expression Meta-Analysis
Source: PLoS One. 2008 Apr 2;3(4):e1880. doi: 10.1371/journal.pone.0001880 (PMC2268968; doi:10.1371/journal.pone.0001880)
Supplement: File S2 — (0.82 MB PDF) [file pone.0001880.s005.pdf]

GEMS Launcher Task: *ModelInspector*: Search for user-defined models
working on Human Promoters

Output overview of ModelInspector matches (1980 matches)

go to:[Output overview] [Detailed output] [Statistics]

ModelInspector Release 5.4.3 May 2007 **Thu Aug 9 12:21:50 2007**

Solution parameters:

**Sequence file:**	Human Promoters
**Models:**	User-defined/E2FF-NFKB.model
**Strand(s) searched:**	both strands
**Threshold for number of elements:**	100.0 % (2 of 2 elements)
**Output sorted by:**	match positions on the sequences
**Maximum number of matches:**	3000

Match List:

Sequence	Model Name	Position	Strand	Select Match
**GXP_4176** [GXP_4176] (1 - 749)<br>**MKNK2**, GXL_3345, GeneID: 2872, Homo sapiens chr. 19<br>MAP kinase interacting serine/threonine kinase 2	E2FF-NFKB	399 - 521	(+)	
**GXP_4179** [GXP_4179] (1 - 616)<br>**RFX2**, GXL_3347, GeneID: 5990, Homo sapiens chr. 19<br>regulatory factor X, 2 (influences HLA class II expression)	E2FF-NFKB	62 - 189	(+)	
	E2FF-NFKB	288 - 175	(-)	
**GXP_4188** [GXP_4188] (1 - 601)<br>**LSM7**, GXL_3352, GeneID: 51690, Homo sapiens chr. 19	E2FF-NFKB	344 - 216	(-)	

LSM7 homolog, U6 small nuclear RNA associated (S. cerevisiae)				
**GXP_912657** [GXP_912657] (1 - 601)<br>**LSM7**, GXL_3352, GeneID: 51690, Homo sapiens chr. 19<br>LSM7 homolog, U6 small nuclear RNA associated (S. cerevisiae)	E2FF-NFKB	246 - 118	(-)	
**GXP_912658** [GXP_912658] (1 - 619)<br>**LSM7**, GXL_3352, GeneID: 51690, Homo sapiens chr. 19<br>LSM7 homolog, U6 small nuclear RNA associated (S. cerevisiae)	E2FF-NFKB	377 - 249	(-)	
**GXP_912599** [GXP_912599] (1 - 601)<br>**PRG2,LOC729098**, GXL_3356, GeneID: 79948,729098, Homo sapiens chr. 19<br>plasticity-related gene 2; hypothetical protein LOC729098	E2FF-NFKB	293 - 416	(+)	
**GXP_4218** [GXP_4218] (1 - 1365)<br>**LONP1**, GXL_3370, GeneID: 9361, Homo sapiens chr. 19<br>lon peptidase 1, mitochondrial	E2FF-NFKB	620 - 508	(-)	
**GXP_4219** [GXP_4219] (1 - 620)<br>**LONP1**, GXL_3370, GeneID: 9361, Homo sapiens chr. 19<br>lon peptidase 1, mitochondrial	E2FF-NFKB	214 - 102	(-)	
**GXP_4229** [GXP_4229] (1 - 996)<br>**FARSA**, GXL_3377, GeneID: 2193, Homo sapiens chr. 19<br>phenylalanyl-tRNA synthetase, alpha subunit	E2FF-NFKB	423 - 535	(+)	
**GXP_648190** [GXP_648190] (1 - 601)<br>**FARSA**, GXL_3377, GeneID: 2193, Homo sapiens chr. 19<br>phenylalanyl-tRNA synthetase, alpha subunit	E2FF-NFKB	359 - 239	(-)	
**GXP_4236** [GXP_4236] (1 - 928)<br>**ZBTB7A**, GXL_3383, GeneID: 51341, Homo sapiens chr. 19<br>zinc finger and BTB domain containing 7A	E2FF-NFKB	52 - 162	(+)	
**GXP_648100** [GXP_648100] (1 - 601)<br>**CDC37**, GXL_3387, GeneID: 11140, Homo sapiens chr. 19<br>cell division cycle 37 homolog (S. cerevisiae)	E2FF-NFKB	232 - 126	(-)	
**GXP_4247** [GXP_4247] (1 - 601)<br>**TLE2**, GXL_3391, GeneID: 7089, Homo sapiens chr. 19<br>transducin-like enhancer of split 2 (E(sp1) homolog, Drosophila)	E2FF-NFKB	411 - 293	(-)	
**GXP_4249** [GXP_4249] (1 - 1144)<br>**MGC19604**, GXL_3392, GeneID: 112812, Homo sapiens chr. 19<br>similar to RIKEN cDNA B230118G17 gene	E2FF-NFKB	646 - 519	(-)	
**GXP_4251** [GXP_4251] (1 - 1089)<br>**MAN2B1**, GXL_3393, GeneID: 4125, Homo sapiens chr. 19<br>mannosidase, alpha, class 2B, member 1	E2FF-NFKB	961 - 845	(-)	
	E2FF-NFKB	1083 - 968	(-)	
**GXP_648168** [GXP_648168] (1 - 601)<br>**ZNF564,ZNF709**, GXL_3395, GeneID: 163050,163051, Homo sapiens chr. 19<br>zinc finger protein 564;zinc finger protein 709	E2FF-NFKB	347 - 236	(-)	

**GXP_4266** [GXP_4266] (1 - 881)<br>**CACNA1A**, GXL_3405, GeneID: 773, Homo sapiens chr. 19<br>calcium channel, voltage-dependent, P/Q type, alpha 1A subunit	E2FF-NFKB	521 - 410	(-)	
**GXP_4292** [GXP_4292] (1 - 778)<br>**MATK**, GXL_3420, GeneID: 4145, Homo sapiens chr. 19<br>megakaryocyte-associated tyrosine kinase	E2FF-NFKB	456 - 573	(+)	
**GXP_4334** [GXP_4334] (1 - 1333)<br>**PLEKHJ1**, GXL_3447, GeneID: 55111, Homo sapiens chr. 19<br>pleckstrin homology domain containing, family J member 1	E2FF-NFKB	644 - 525	(-)	
**GXP_4349** [GXP_4349] (1 - 629)<br>**ZNF442**, GXL_3454, GeneID: 79973, Homo sapiens chr. 19<br>zinc finger protein 442	E2FF-NFKB	353 - 240	(-)	
**GXP_4351** [GXP_4351] (1 - 665)<br>**FBXW9**, GXL_3456, GeneID: 84261, Homo sapiens chr. 19<br>F-box and WD repeat domain containing 9	E2FF-NFKB	471 - 577	(+)	
**GXP_4352** [GXP_4352] (1 - 821)<br>**RANBP3**, GXL_3457, GeneID: 8498, Homo sapiens chr. 19<br>RAN binding protein 3	E2FF-NFKB	463 - 345	(-)	
**GXP_912581** [GXP_912581] (1 - 601)<br>**SHC2,LOC732442**, GXL_3465, GeneID: 25759,732442, Homo sapiens chr. 19<br>SHC (Src homology 2 domain containing) transforming protein 2; similar to SHC transforming protein 2 (SH2 domain protein C2) (Src homology 2 domain-containing transforming protein C2) (Protein Sck)	E2FF-NFKB	275 - 152	(-)	
**GXP_4443** [GXP_4443] (1 - 698)<br>**COL5A3**, GXL_3518, GeneID: 50509, Homo sapiens chr. 19<br>collagen, type V, alpha 3	E2FF-NFKB	579 - 473	(-)	
**GXP_4449** [GXP_4449] (1 - 1147)<br>**TIMM13**, GXL_3523, GeneID: 26517, Homo sapiens chr. 19<br>translocase of inner mitochondrial membrane 13 homolog (yeast)	E2FF-NFKB	688 - 803	(+)	
**GXP_4488** [GXP_4488] (1 - 601)<br>**FLJ25758**, GXL_3551, GeneID: 497049, Homo sapiens chr. 19<br>hypothetical locus FLJ25758	E2FF-NFKB	445 - 559	(+)	
**GXP_4495** [GXP_4495] (1 - 945)<br>**NDUFA11**, GXL_3557, GeneID: 126328, Homo sapiens chr. 19<br>NADH dehydrogenase (ubiquinone) 1 alpha subcomplex, 11, 14.7kDa	E2FF-NFKB	25 - 143	(+)	
	E2FF-NFKB	762 - 883	(+)	
**GXP_912748** [GXP_912748] (1 - 603)<br>**MLLT1,LOC729433**, GXL_3558, GeneID: 4298,729433, Homo sapiens chr. 19<br>myeloid/lymphoid or mixed-lineage leukemia (trithorax homolog, Drosophila), translocated to, 1; similar to myeloid/lymphoid or mixed-lineage leukemia (trithorax homolog, Drosophila), translocated to, 1	E2FF-NFKB	346 - 453	(+)	
	E2FF-NFKB	546 - 439	(-)	
**GXP_4501** [GXP_4501] (1 - 668)<br>**POLRMT**, GXL_3563, GeneID: 5442, Homo sapiens chr. 19	E2FF-NFKB	543 - 664	(+)	

polymerase (RNA) mitochondrial (DNA directed)				
**GXP_4505** [GXP_4505] (1 - 601)<br>**REXO1**, GXL_3567, GeneID: 57455, Homo sapiens chr. 19<br>REX1, RNA exonuclease 1 homolog (S. cerevisiae)	E2FF-NFKB	217 - 324	(+)	
**GXP_4514** [GXP_4514] (1 - 601)<br>**MIER2,LOC644979,LOC650495**, GXL_3576, GeneID: 54531,644979,650495, Homo sapiens chr. 19<br>mesoderm induction early response 1, family member 2; hypothetical LOC644979;hypothetical protein LOC650495	E2FF-NFKB	573 - 466	(-)	
**GXP_912577** [GXP_912577] (1 - 715)<br>**MIER2,LOC644979,LOC650495**, GXL_3576, GeneID: 54531,644979,650495, Homo sapiens chr. 19<br>mesoderm induction early response 1, family member 2; hypothetical LOC644979;hypothetical protein LOC650495	E2FF-NFKB	700 - 593	(-)	
**GXP_5888** [GXP_5888] (1 - 1334)<br>**GCHFR**, GXL_4895, GeneID: 2644, Homo sapiens chr. 15<br>GTP cyclohydrolase I feedback regulator	E2FF-NFKB	1130 - 1240	(+)	
**GXP_909006** [GXP_909006] (1 - 941)<br>**NUT**, GXL_4902, GeneID: 256646, Homo sapiens chr. 15<br>nuclear protein in testis	E2FF-NFKB	253 - 147	(-)	
**GXP_908841** [GXP_908841] (1 - 601)<br>**FLJ35785**, GXL_4909, GeneID: 283796, Homo sapiens chr. 15<br>hypothetical protein FLJ35785	E2FF-NFKB	201 - 316	(+)	
**GXP_5957** [GXP_5957] (1 - 724)<br>**AP4E1**, GXL_4935, GeneID: 23431, Homo sapiens chr. 15<br>adaptor-related protein complex 4, epsilon 1 subunit	E2FF-NFKB	454 - 329	(-)	
**GXP_5979** [GXP_5979] (1 - 601)<br>**B2M**, GXL_4953, GeneID: 567, Homo sapiens chr. 15<br>beta-2-microglobulin	E2FF-NFKB	293 - 417	(+)	
**GXP_6015** [GXP_6015] (1 - 1050)<br>**SPINT1**, GXL_4973, GeneID: 6692, Homo sapiens chr. 15<br>serine peptidase inhibitor, Kunitz type 1	E2FF-NFKB	131 - 2	(-)	
**GXP_6026** [GXP_6026] (1 - 698)<br>**RAB8B**, GXL_4983, GeneID: 51762, Homo sapiens chr. 15<br>RAB8B, member RAS oncogene family	E2FF-NFKB	138 - 261	(+)	
**GXP_643916** [GXP_643916] (1 - 601)<br>**THBS1**, GXL_5013, GeneID: 7057, Homo sapiens chr. 15<br>thrombospondin 1	E2FF-NFKB	3 - 122	(+)	
**GXP_909033** [GXP_909033] (1 - 749)<br>**THBS1**, GXL_5013, GeneID: 7057, Homo sapiens chr. 15<br>thrombospondin 1	E2FF-NFKB	409 - 528	(+)	
**GXP_6068** [GXP_6068] (1 - 793)<br>**D4ST1**, GXL_5016, GeneID: 113189, Homo sapiens chr. 15<br>dermatan 4 sulfotransferase 1	E2FF-NFKB	472 - 581	(+)	

**GXP_6182** [GXP_6182] (1 - 623)<br>**MAPK6**, GXL_5117, GeneID: 5597, Homo sapiens chr. 15<br>mitogen-activated protein kinase 6	E2FF-NFKB	503 - 382	(-)	
**GXP_7295** [GXP_7295] (1 - 681)<br>**ERBB2IP**, GXL_5936, GeneID: 55914, Homo sapiens chr. 5<br>erbb2 interacting protein	E2FF-NFKB	517 - 634	(+)	
**GXP_7303** [GXP_7303] (1 - 782)<br>**XRCC4**, GXL_5942, GeneID: 7518, Homo sapiens chr. 5<br>X-ray repair complementing defective repair in Chinese hamster cells 4	E2FF-NFKB	501 - 391	(-)	
**GXP_7369** [GXP_7369] (1 - 705)<br>**DMXL1**, GXL_5982, GeneID: 1657, Homo sapiens chr. 5<br>Dmx-like 1	E2FF-NFKB	186 - 61	(-)	
**GXP_657037** [GXP_657037] (1 - 601)<br>**DMXL1**, GXL_5982, GeneID: 1657, Homo sapiens chr. 5<br>Dmx-like 1	E2FF-NFKB	366 - 241	(-)	
**GXP_7482** [GXP_7482] (1 - 655)<br>**COMMD10**, GXL_6058, GeneID: 51397, Homo sapiens chr. 5<br>COMM domain containing 10	E2FF-NFKB	542 - 652	(+)	
**GXP_922774** [GXP_922774] (1 - 601)<br>**COMMD10**, GXL_6058, GeneID: 51397, Homo sapiens chr. 5<br>COMM domain containing 10	E2FF-NFKB	59 - 169	(+)	
**GXP_7485** [GXP_7485] (1 - 601)<br>**MEGF10**, GXL_6060, GeneID: 84466, Homo sapiens chr. 5<br>multiple EGF-like-domains 10	E2FF-NFKB	150 - 29	(-)	
**GXP_922708** [GXP_922708] (1 - 716)<br>**CAST**, GXL_6082, GeneID: 831, Homo sapiens chr. 5<br>calpastatin	E2FF-NFKB	643 - 514	(-)	
**GXP_656704** [GXP_656704] (1 - 601)<br>**RNF180**, GXL_6083, GeneID: 285671, Homo sapiens chr. 5<br>ring finger protein 180	E2FF-NFKB	424 - 552	(+)	
**GXP_922629** [GXP_922629] (1 - 601)<br>**THBS4**, GXL_6180, GeneID: 7060, Homo sapiens chr. 5<br>thrombospondin 4	E2FF-NFKB	418 - 532	(+)	
**GXP_7674** [GXP_7674] (1 - 756)<br>**F2RL1**, GXL_6214, GeneID: 2150, Homo sapiens chr. 5<br>coagulation factor II (thrombin) receptor-like 1	E2FF-NFKB	386 - 270	(-)	
**GXP_10542** [GXP_10542] (1 - 1084)<br>**CACNA1G**, GXL_8620, GeneID: 8913, Homo sapiens chr. 17<br>calcium channel, voltage-dependent, T type, alpha 1G subunit	E2FF-NFKB	673 - 784	(+)	
	E2FF-NFKB	403 - 279	(-)	

**GXP_10601** [GXP_10601] (1 - 604)<br>**METTL2A**, GXL_8648, GeneID: 339175, Homo sapiens chr. 17<br>methyltransferase like 2A				
**GXP_10699** [GXP_10699] (1 - 770)<br>**WNT9B**, GXL_8708, GeneID: 7484, Homo sapiens chr. 17<br>wingless-type MMTV integration site family, member 9B	E2FF-NFKB	413 - 542	(+)	
**GXP_10703** [GXP_10703] (1 - 701)<br>**EPX**, GXL_8710, GeneID: 8288, Homo sapiens chr. 17<br>eosinophil peroxidase	E2FF-NFKB	225 - 340	(+)	
**GXP_10721** [GXP_10721] (1 - 613)<br>**CDK3**, GXL_8723, GeneID: 1018, Homo sapiens chr. 17<br>cyclin-dependent kinase 3	E2FF-NFKB	494 - 605	(+)	
**GXP_647113** [GXP_647113] (1 - 601)<br>**LLGL2,LOC652838**, GXL_8738, GeneID: 3993,652838, Homo sapiens chr. 17<br>lethal giant larvae homolog 2 (Drosophila); similar to lethal giant larvae homolog 2 isoform a	E2FF-NFKB	17 - 145	(+)	
**GXP_10761** [GXP_10761] (1 - 748)<br>**ICT1**, GXL_8747, GeneID: 3396, Homo sapiens chr. 17<br>immature colon carcinoma transcript 1	E2FF-NFKB	406 - 515	(+)	
**GXP_647026** [GXP_647026] (1 - 601)<br>**PITPNC1,LOC729822,LOC731962**, GXL_8748, GeneID: 26207,729822,731962, Homo sapiens chr. 17<br>phosphatidylinositol transfer protein, cytoplasmic 1; similar to phosphatidylinositol transfer protein, cytoplasmic 1; similar to phosphatidylinositol transfer protein, cytoplasmic 1	E2FF-NFKB	366 - 254	(-)	
**GXP_911735** [GXP_911735] (1 - 754)<br>**AKAP1**, GXL_8757, GeneID: 8165, Homo sapiens chr. 17<br>A kinase (PRKA) anchor protein 1	E2FF-NFKB	416 - 303	(-)	
**GXP_10837** [GXP_10837] (1 - 974)<br>**SOX9**, GXL_8809, GeneID: 6662, Homo sapiens chr. 17<br>SRY (sex determining region Y)-box 9 (campomelic dysplasia, autosomal sex-reversal)	E2FF-NFKB	155 - 261	(+)	
**GXP_10841** [GXP_10841] (1 - 1326)<br>**KCTD2,LOC643177,LOC732133**, GXL_8813, GeneID: 23510,643177,732133, Homo sapiens chr. 17<br>potassium channel tetramerisation domain containing 2; hypothetical LOC643177;hypothetical protein LOC732133	E2FF-NFKB	309 - 197	(-)	
**GXP_14262** [GXP_14262] (1 - 946)<br>**SLC25A43**, GXL_11805, GeneID: 203427, Homo sapiens chr. X<br>solute carrier family 25, member 43	E2FF-NFKB	834 - 716	(-)	
**GXP_14297** [GXP_14297] (1 - 1386)<br>**ZIC3**, GXL_11824, GeneID: 7547, Homo sapiens chr. X<br>Zic family member 3 heterotaxy 1 (odd-paired homolog, Drosophila)	E2FF-NFKB	886 - 766	(-)	
**GXP_14323** [GXP_14323] (1 - 601)<br>**MTMR1**, GXL_11840, GeneID: 8776, Homo sapiens chr. X<br>myotubularin related protein 1	E2FF-NFKB	132 - 7	(-)	

**GXP_14329** [GXP_14329] (1 - 836)<br>**VBP1**, GXL_11844, GeneID: 7411, Homo sapiens chr. X<br>von Hippel-Lindau binding protein 1	E2FF-NFKB	702 - 581	(-)	
**GXP_14396** [GXP_14396] (1 - 602)<br>**RPL10,SNORA70**, GXL_11885, GeneID: 6134,26778, Homo sapiens chr. X<br>ribosomal protein L10; small nucleolar RNA, H/ACA box 70	E2FF-NFKB	280 - 162	(-)	
**GXP_14407** [GXP_14407] (1 - 1183)<br>**SLC25A14**, GXL_11894, GeneID: 9016, Homo sapiens chr. X<br>solute carrier family 25 (mitochondrial carrier, brain), member 14	E2FF-NFKB	835 - 722	(-)	
**GXP_14514** [GXP_14514] (1 - 601)<br>**BIRC4**, GXL_11974, GeneID: 331, Homo sapiens chr. X<br>baculoviral IAP repeat-containing 4	E2FF-NFKB	423 - 546	(+)	
**GXP_14569** [GXP_14569] (1 - 601)<br>**DUSP9**, GXL_12026, GeneID: 1852, Homo sapiens chr. X<br>dual specificity phosphatase 9	E2FF-NFKB	515 - 405	(-)	
**GXP_14963** [GXP_14963] (1 - 659)<br>**ZFAND2A**, GXL_12387, GeneID: 90637, Homo sapiens chr. 7<br>zinc finger, AN1-type domain 2A	E2FF-NFKB	513 - 404	(-)	
**GXP_15000** [GXP_15000] (1 - 789)<br>**GNA12**, GXL_12402, GeneID: 2768, Homo sapiens chr. 7<br>guanine nucleotide binding protein (G protein) alpha 12	E2FF-NFKB	259 - 152	(-)	
**GXP_15021** [GXP_15021] (1 - 740)<br>**NDUFA4**, GXL_12415, GeneID: 4697, Homo sapiens chr. 7<br>NADH dehydrogenase (ubiquinone) 1 alpha subcomplex, 4, 9kDa	E2FF-NFKB	183 - 303	(+)	
**GXP_15052** [GXP_15052] (1 - 1002)<br>**CDCA7L**, GXL_12439, GeneID: 55536, Homo sapiens chr. 7<br>cell division cycle associated 7-like	E2FF-NFKB	674 - 556	(-)	
**GXP_924610** [GXP_924610] (1 - 752)<br>**FLJ14712**, GXL_12471, GeneID: 221806, Homo sapiens chr. 7<br>hypothetical protein FLJ14712	E2FF-NFKB	604 - 478	(-)	
**GXP_15109** [GXP_15109] (1 - 601)<br>**INTS1**, GXL_12475, GeneID: 26173, Homo sapiens chr. 7<br>integrator complex subunit 1	E2FF-NFKB	245 - 119	(-)	
**GXP_924809** [GXP_924809] (1 - 601)<br>**FLJ21767**, GXL_12484, GeneID: 401331, Homo sapiens chr. 7<br>hypothetical protein FLJ21767	E2FF-NFKB	186 - 303	(+)	
**GXP_15139** [GXP_15139] (1 - 657)<br>**SNX8**, GXL_12491, GeneID: 29886, Homo sapiens chr. 7<br>sorting nexin 8	E2FF-NFKB	514 - 640	(+)	
	E2FF-NFKB	365 - 485	(+)	

**GXP_659253** [GXP_659253] (1 - 601)<br>**HOXA10**, GXL_12514, GeneID: 3206, Homo sapiens chr. 7<br>homeobox A10				
**GXP_15191** [GXP_15191] (1 - 745)<br>**MYO1G**, GXL_12529, GeneID: 64005, Homo sapiens chr. 7<br>myosin IG	E2FF-NFKB	365 - 484	(+)	
**GXP_15256** [GXP_15256] (1 - 747)<br>**DPY19L1**, GXL_12584, GeneID: 23333, Homo sapiens chr. 7<br>dpy-19-like 1 (C. elegans)	E2FF-NFKB	150 - 26	(-)	
**GXP_924748** [GXP_924748] (1 - 601)<br>**DPY19L1**, GXL_12584, GeneID: 23333, Homo sapiens chr. 7<br>dpy-19-like 1 (C. elegans)	E2FF-NFKB	379 - 255	(-)	
**GXP_15263** [GXP_15263] (1 - 1084)<br>**TMED4**, GXL_12591, GeneID: 222068, Homo sapiens chr. 7<br>transmembrane emp24 protein transport domain containing 4	E2FF-NFKB	620 - 731	(+)	
**GXP_924507** [GXP_924507] (1 - 601)<br>**MGC11257**, GXL_12612, GeneID: 84310, Homo sapiens chr. 7<br>hypothetical protein MGC11257	E2FF-NFKB	430 - 321	(-)	
**GXP_15296** [GXP_15296] (1 - 601)<br>**SETX,LOC727989**, GXL_12617, GeneID: 23064,727989, Homo sapiens chr. 9<br>senataxin;hypothetical protein LOC727989	E2FF-NFKB	118 - 233	(+)	
**GXP_661785** [GXP_661785] (1 - 601)<br>**RUSC2,LOC646932**, GXL_12618, GeneID: 9853,646932, Homo sapiens chr. 9<br>RUN and SH3 domain containing 2; hypothetical LOC646932	E2FF-NFKB	93 - 212	(+)	
**GXP_926698** [GXP_926698] (1 - 926)<br>**RUSC2,LOC646932**, GXL_12618, GeneID: 9853,646932, Homo sapiens chr. 9<br>RUN and SH3 domain containing 2; hypothetical LOC646932	E2FF-NFKB	375 - 486	(+)	
**GXP_15349** [GXP_15349] (1 - 674)<br>**SH3GL2**, GXL_12645, GeneID: 6456, Homo sapiens chr. 9<br>SH3-domain GRB2-like 2	E2FF-NFKB	495 - 373	(-)	
**GXP_926622** [GXP_926622] (1 - 601)<br>**IFT74**, GXL_12656, GeneID: 80173, Homo sapiens chr. 9<br>intraflagellar transport 74 homolog (Chlamydomonas)	E2FF-NFKB	293 - 178	(-)	
**GXP_15377** [GXP_15377] (1 - 601)<br>**RALGDS**, GXL_12661, GeneID: 5900, Homo sapiens chr. 9<br>ral guanine nucleotide dissociation stimulator	E2FF-NFKB	468 - 352	(-)	
**GXP_927370** [GXP_927370] (1 - 643)<br>**RALGDS**, GXL_12661, GeneID: 5900, Homo sapiens chr. 9<br>ral guanine nucleotide dissociation stimulator	E2FF-NFKB	253 - 377	(+)	
	E2FF-NFKB	177 - 58	(-)	

**GXP_15396** [GXP_15396] (1 - 680)<br>**CIZ1**, GXL_12672, GeneID: 25792, Homo sapiens chr. 9<br>CDKN1A interacting zinc finger protein 1				
**GXP_927269** [GXP_927269] (1 - 601)<br>**CIZ1**, GXL_12672, GeneID: 25792, Homo sapiens chr. 9<br>CDKN1A interacting zinc finger protein 1	E2FF-NFKB	422 - 303	(-)	
**GXP_15402** [GXP_15402] (1 - 601)<br>**DNAI1**, GXL_12675, GeneID: 27019, Homo sapiens chr. 9<br>dynein, axonemal, intermediate chain 1	E2FF-NFKB	456 - 347	(-)	
**GXP_15404** [GXP_15404] (1 - 883)<br>**AGPAT2**, GXL_12676, GeneID: 10555, Homo sapiens chr. 9<br>1-acylglycerol-3-phosphate O-acyltransferase 2 (lysophosphatidic acid acyltransferase, beta)	E2FF-NFKB	288 - 417	(+)	
**GXP_926517** [GXP_926517] (1 - 601)<br>**SMARCA2**, GXL_12684, GeneID: 6595, Homo sapiens chr. 9<br>SWI/SNF related, matrix associated, actin dependent regulator of chromatin, subfamily a, member 2	E2FF-NFKB	148 - 263	(+)	
**GXP_927263** [GXP_927263] (1 - 1351)<br>**PTGES2**, GXL_12692, GeneID: 80142, Homo sapiens chr. 9<br>prostaglandin E synthase 2	E2FF-NFKB	412 - 541	(+)	
**GXP_15446** [GXP_15446] (1 - 613)<br>**JMJD2C**, GXL_12702, GeneID: 23081, Homo sapiens chr. 9<br>jumonji domain containing 2C	E2FF-NFKB	436 - 320	(-)	
**GXP_15457** [GXP_15457] (1 - 742)<br>**SH2D3C**, GXL_12708, GeneID: 10044, Homo sapiens chr. 9<br>SH2 domain containing 3C	E2FF-NFKB	378 - 492	(+)	
**GXP_15461** [GXP_15461] (1 - 1291)<br>**UCK1**, GXL_12710, GeneID: 83549, Homo sapiens chr. 9<br>uridine-cytidine kinase 1	E2FF-NFKB	1029 - 903	(-)	
**GXP_15476** [GXP_15476] (1 - 1175)<br>**DMRT1**, GXL_12723, GeneID: 1761, Homo sapiens chr. 9<br>doublesex and mab-3 related transcription factor 1	E2FF-NFKB	253 - 129	(-)	
**GXP_15491** [GXP_15491] (1 - 601)<br>**MGC61598**, GXL_12734, GeneID: 441478, Homo sapiens chr. 9<br>similar to ankyrin-repeat protein Nrarp	E2FF-NFKB	44 - 157	(+)	
**GXP_927295** [GXP_927295] (1 - 601)<br>**CCBL1**, GXL_12748, GeneID: 883, Homo sapiens chr. 9<br>cysteine conjugate-beta lyase, cytoplasmic (glutamine transaminase K, kyneurenine aminotransferase)	E2FF-NFKB	305 - 184	(-)	
**GXP_926510** [GXP_926510] (1 - 616)<br>**DOCK8**, GXL_12784, GeneID: 81704, Homo sapiens chr. 9<br>dedicator of cytokinesis 8	E2FF-NFKB	374 - 247	(-)	
	E2FF-NFKB	643 - 757	(+)	

**GXP_15564** [GXP_15564] (1 - 816)<br>**CDC37L1**, GXL_12789, GeneID: 55664, Homo sapiens chr. 9<br>cell division cycle 37 homolog (S. cerevisiae)-like 1				
**GXP_15605** [GXP_15605] (1 - 601)<br>**C9orf127**, GXL_12825, GeneID: 51754, Homo sapiens chr. 9<br>chromosome 9 open reading frame 127	E2FF-NFKB	193 - 75	(-)	
**GXP_662800** [GXP_662800] (1 - 601)<br>**LCN8**, GXL_12833, GeneID: 138307, Homo sapiens chr. 9<br>lipocalin 8	E2FF-NFKB	431 - 314	(-)	
**GXP_15636** [GXP_15636] (1 - 618)<br>**OBP2B**, GXL_12853, GeneID: 29989, Homo sapiens chr. 9<br>odorant binding protein 2B	E2FF-NFKB	272 - 380	(+)	
**GXP_16821** [GXP_16821] (1 - 604)<br>**METTL6**, GXL_13741, GeneID: 131965, Homo sapiens chr. 3<br>methyltransferase like 6	E2FF-NFKB	593 - 478	(-)	
**GXP_16838** [GXP_16838] (1 - 1129)<br>**HACL1**, GXL_13749, GeneID: 26061, Homo sapiens chr. 3<br>2-hydroxyacyl-CoA lyase 1	E2FF-NFKB	1103 - 987	(-)	
**GXP_919802** [GXP_919802] (1 - 601)<br>**HACL1**, GXL_13749, GeneID: 26061, Homo sapiens chr. 3<br>2-hydroxyacyl-CoA lyase 1	E2FF-NFKB	555 - 439	(-)	
**GXP_920051** [GXP_920051] (1 - 601)<br>**KIF9**, GXL_13760, GeneID: 64147, Homo sapiens chr. 3<br>kinesin family member 9	E2FF-NFKB	262 - 382	(+)	
**GXP_919698** [GXP_919698] (1 - 601)<br>**IL5RA**, GXL_13776, GeneID: 3568, Homo sapiens chr. 3<br>interleukin 5 receptor, alpha	E2FF-NFKB	353 - 475	(+)	
**GXP_653822** [GXP_653822] (1 - 661)<br>**AMT,NICN1**, GXL_13801, GeneID: 275,84276, Homo sapiens chr. 3<br>aminomethyltransferase;nicolin 1	E2FF-NFKB	361 - 489	(+)	
**GXP_920081** [GXP_920081] (1 - 601)<br>**SLC26A6**, GXL_13817, GeneID: 65010, Homo sapiens chr. 3<br>solute carrier family 26, member 6	E2FF-NFKB	320 - 198	(-)	
**GXP_16987** [GXP_16987] (1 - 601)<br>**FLJ42094**, GXL_13834, GeneID: 401050, Homo sapiens chr. 3<br>hypothetical gene supported by AK124088	E2FF-NFKB	253 - 370	(+)	
**GXP_919850** [GXP_919850] (1 - 601)<br>**NGLY1**, GXL_13858, GeneID: 55768, Homo sapiens chr. 3<br>N-glycanase 1	E2FF-NFKB	254 - 381	(+)	
	E2FF-NFKB	507 - 615	(+)	

**GXP_17081** [GXP_17081] (1 - 617)<br>**TSP50,TESSP5,LOC729280,LOC729752,LOC729756**, GXL_13905, GeneID: 29122,377047,729280,729752,729756, Homo sapiens chr. 3<br>testes-specific protease 50;testis serine protease 5; testis serine protease 6; similar to testis serine protease 2; similar to testis serine protease 2				
**GXP_18868** [GXP_18868] (1 - 1364)<br>**ATP6V1G1**, GXL_15185, GeneID: 9550, Homo sapiens chr. 9<br>ATPase, H+ transporting, lysosomal 13kDa, V1 subunit G1	E2FF-NFKB	709 - 828	(+)	
**GXP_18896** [GXP_18896] (1 - 601)<br>**VPS13A**, GXL_15208, GeneID: 23230, Homo sapiens chr. 9<br>vacuolar protein sorting 13 homolog A (S. cerevisiae)	E2FF-NFKB	168 - 278	(+)	
**GXP_18950** [GXP_18950] (1 - 601)<br>**FOXE1**, GXL_15242, GeneID: 2304, Homo sapiens chr. 9<br>forkhead box E1 (thyroid transcription factor 2)	E2FF-NFKB	182 - 302	(+)	
**GXP_18970** [GXP_18970] (1 - 947)<br>**ANP32B**, GXL_15260, GeneID: 10541, Homo sapiens chr. 9<br>acidic (leucine-rich) nuclear phosphoprotein 32 family, member B	E2FF-NFKB	254 - 369	(+)	
**GXP_19027** [GXP_19027] (1 - 601)<br>**CCIN**, GXL_15308, GeneID: 881, Homo sapiens chr. 9<br>calicin	E2FF-NFKB	307 - 418	(+)	
**GXP_19087** [GXP_19087] (1 - 1320)<br>**PGM5**, GXL_15363, GeneID: 5239, Homo sapiens chr. 9<br>phosphoglucomutase 5	E2FF-NFKB	1025 - 1133	(+)	
**GXP_926855** [GXP_926855] (1 - 601)<br>**PGM5**, GXL_15363, GeneID: 5239, Homo sapiens chr. 9<br>phosphoglucomutase 5	E2FF-NFKB	165 - 273	(+)	
**GXP_19091** [GXP_19091] (1 - 819)<br>**OSTF1**, GXL_15367, GeneID: 26578, Homo sapiens chr. 9<br>osteoclast stimulating factor 1	E2FF-NFKB	414 - 307	(-)	
**GXP_19873** [GXP_19873] (1 - 749)<br>**HDAC6**, GXL_16119, GeneID: 10013, Homo sapiens chr. X<br>histone deacetylase 6	E2FF-NFKB	422 - 547	(+)	
**GXP_19874** [GXP_19874] (1 - 601)<br>**HDAC6**, GXL_16119, GeneID: 10013, Homo sapiens chr. X<br>histone deacetylase 6	E2FF-NFKB	274 - 399	(+)	
**GXP_19893** [GXP_19893] (1 - 671)<br>**HNRPH2**, GXL_16131, GeneID: 3188, Homo sapiens chr. X<br>heterogeneous nuclear ribonucleoprotein H2 (H')	E2FF-NFKB	240 - 120	(-)	
**GXP_19900** [GXP_19900] (1 - 601)<br>**FLJ21687**, GXL_16136, GeneID: 79917, Homo sapiens chr. X<br>PDZ domain containing, X chromosome	E2FF-NFKB	381 - 258	(-)	

**GXP_19902** [GXP_19902] (1 - 601)<br>**FLJ21687**, GXL_16136, GeneID: 79917, Homo sapiens chr. X<br>PDZ domain containing, X chromosome	E2FF-NFKB	354 - 231	(-)	
**GXP_927669** [GXP_927669] (1 - 601)<br>**MID1IP1**, GXL_16149, GeneID: 58526, Homo sapiens chr. X<br>MID1 interacting protein 1 (gastrulation specific G12 homolog (zebrafish))	E2FF-NFKB	168 - 47	(-)	
	E2FF-NFKB	553 - 425	(-)	
**GXP_19968** [GXP_19968] (1 - 1464)<br>**NHS**, GXL_16181, GeneID: 4810, Homo sapiens chr. X<br>Nance-Horan syndrome (congenital cataracts and dental anomalies)	E2FF-NFKB	134 - 246	(+)	
**GXP_20013** [GXP_20013] (1 - 800)<br>**TSPAN7**, GXL_16214, GeneID: 7102, Homo sapiens chr. X<br>tetraspanin 7	E2FF-NFKB	583 - 460	(-)	
**GXP_663043** [GXP_663043] (1 - 601)<br>**PTCHD1**, GXL_16263, GeneID: 139411, Homo sapiens chr. X<br>patched domain containing 1	E2FF-NFKB	530 - 401	(-)	
**GXP_927603** [GXP_927603] (1 - 601)<br>**PTCHD1**, GXL_16263, GeneID: 139411, Homo sapiens chr. X<br>patched domain containing 1	E2FF-NFKB	327 - 438	(+)	
**GXP_20080** [GXP_20080] (1 - 660)<br>**UBE1**, GXL_16272, GeneID: 7317, Homo sapiens chr. X<br>ubiquitin-activating enzyme E1 (A1S9T and BN75 temperature sensitivity complementing)	E2FF-NFKB	398 - 284	(-)	
**GXP_20115** [GXP_20115] (1 - 853)<br>**RP2**, GXL_16300, GeneID: 6102, Homo sapiens chr. X<br>retinitis pigmentosa 2 (X-linked recessive)	E2FF-NFKB	569 - 684	(+)	
**GXP_20125** [GXP_20125] (1 - 601)<br>**SLC16A2**, GXL_16310, GeneID: 6567, Homo sapiens chr. X<br>solute carrier family 16, member 2 (monocarboxylic acid transporter 8)	E2FF-NFKB	6 - 129	(+)	
**GXP_927899** [GXP_927899] (1 - 1118)<br>**KIF4A**, GXL_16314, GeneID: 24137, Homo sapiens chr. X<br>kinesin family member 4A	E2FF-NFKB	684 - 565	(-)	
**GXP_927898** [GXP_927898] (1 - 601)<br>**ARR3**, GXL_16326, GeneID: 407, Homo sapiens chr. X<br>arrestin 3, retinal (X-arrestin)	E2FF-NFKB	431 - 303	(-)	
**GXP_663334** [GXP_663334] (1 - 601)<br>**NUDT10**, GXL_16357, GeneID: 170685, Homo sapiens chr. X<br>nudix (nucleoside diphosphate linked moiety X)-type motif 10	E2FF-NFKB	228 - 111	(-)	
**GXP_20200** [GXP_20200] (1 - 1391)<br>**EFNB1**, GXL_16372, GeneID: 1947, Homo sapiens chr. X<br>ephrin-B1	E2FF-NFKB	749 - 858	(+)	
	E2FF-NFKB	300 - 420	(+)	

**GXP_23325** [GXP_23325] (1 - 673)<br>**ARF1**, GXL_19246, GeneID: 375, Homo sapiens chr. 1<br>ADP-ribosylation factor 1				
**GXP_23353** [GXP_23353] (1 - 687)<br>**C1orf69**, GXL_19263, GeneID: 200205, Homo sapiens chr. 1<br>chromosome 1 open reading frame 69	E2FF-NFKB	504 - 377	(-)	
**GXP_638038** [GXP_638038] (1 - 601)<br>**COG2**, GXL_19270, GeneID: 22796, Homo sapiens chr. 1<br>component of oligomeric golgi complex 2	E2FF-NFKB	421 - 543	(+)	
**GXP_23394** [GXP_23394] (1 - 601)<br>**FLJ39095**, GXL_19286, GeneID: 400812, Homo sapiens chr. 1<br>hypothetical gene supported by AK096414	E2FF-NFKB	116 - 234	(+)	
**GXP_23422** [GXP_23422] (1 - 983)<br>**FAM36A**, GXL_19307, GeneID: 116228, Homo sapiens chr. 1<br>family with sequence similarity 36, member A	E2FF-NFKB	856 - 742	(-)	
**GXP_916281** [GXP_916281] (1 - 609)<br>**EPHX1**, GXL_19320, GeneID: 2052, Homo sapiens chr. 1<br>epoxide hydrolase 1, microsomal (xenobiotic)	E2FF-NFKB	559 - 430	(-)	
**GXP_23493** [GXP_23493] (1 - 747)<br>**SLC35F3**, GXL_19352, GeneID: 148641, Homo sapiens chr. 1<br>solute carrier family 35, member F3	E2FF-NFKB	194 - 306	(+)	
**GXP_916308** [GXP_916308] (1 - 638)<br>**C1orf142**, GXL_19357, GeneID: 116841, Homo sapiens chr. 1<br>chromosome 1 open reading frame 142	E2FF-NFKB	393 - 272	(-)	
**GXP_23557** [GXP_23557] (1 - 820)<br>**KIAA1804**, GXL_19408, GeneID: 84451, Homo sapiens chr. 1<br>mixed lineage kinase 4	E2FF-NFKB	339 - 463	(+)	
**GXP_32352** [GXP_32352] (1 - 601)<br>**GALNT10**, GXL_27380, GeneID: 55568, Homo sapiens chr. 5<br>UDP-N-acetyl-alpha-D-galactosamine:polypeptide N-acetylgalactosaminyltransferase 10 (GalNAc-T10)	E2FF-NFKB	481 - 362	(-)	
**GXP_32364** [GXP_32364] (1 - 647)<br>**FOXI1**, GXL_27384, GeneID: 2299, Homo sapiens chr. 5<br>forkhead box I1	E2FF-NFKB	244 - 355	(+)	
**GXP_922894** [GXP_922894] (1 - 601)<br>**TGFBI**, GXL_27386, GeneID: 7045, Homo sapiens chr. 5<br>transforming growth factor, beta-induced, 68kDa	E2FF-NFKB	352 - 469	(+)	
**GXP_657314** [GXP_657314] (1 - 1414)<br>**MST150**, GXL_27388, GeneID: 85027, Homo sapiens chr. 5<br>MSTP150	E2FF-NFKB	1206 - 1097	(-)	
	E2FF-NFKB	38 - 155	(+)	

**GXP_923022** [GXP_923022] (1 - 601)<br>**ABLIM3**, GXL_27402, GeneID: 22885, Homo sapiens chr. 5<br>actin binding LIM protein family, member 3				
**GXP_32409** [GXP_32409] (1 - 1133)<br>**PCDHA9,PCDHAC2,PCDHAC1,PCDHA13,PCDHA12,PCDHA11,PCDHA10,**, GXL_27407, GeneID: 9752,56134,56135,56136,56137,56138,56139,56140,56141, 56142,56143,56144,56145,56146,56147, Homo sapiens chr. 5<br>protocadherin alpha 9; protocadherin alpha subfamily C, 2; protocadherin alpha subfamily C, 1;protocadherin alpha 13; protocadherin alpha 12;protocadherin alpha 11; protocadherin alpha 10;protocadherin alpha 8; protocadherin alpha 7;protocadherin alpha 6; protocadherin alpha 5;protocadherin alpha 4; protocadherin alpha 3;protocadherin alpha 2; protocadherin alpha 1	E2FF-NFKB	672 - 558	(-)	
**GXP_657263** [GXP_657263] (1 - 601)<br>**RBM27**, GXL_27409, GeneID: 54439, Homo sapiens chr. 5<br>RNA binding motif protein 27	E2FF-NFKB	418 - 545	(+)	
**GXP_32430** [GXP_32430] (1 - 601)<br>**MFAP3**, GXL_27413, GeneID: 4238, Homo sapiens chr. 5<br>microfibrillar-associated protein 3	E2FF-NFKB	241 - 357	(+)	
**GXP_32433** [GXP_32433] (1 - 601)<br>**SGCD**, GXL_27414, GeneID: 6444, Homo sapiens chr. 5<br>sarcoglycan, delta (35kDa dystrophin-associated glycoprotein)	E2FF-NFKB	516 - 387	(-)	
**GXP_922880** [GXP_922880] (1 - 601)<br>**C5orf14**, GXL_27422, GeneID: 79770, Homo sapiens chr. 5<br>chromosome 5 open reading frame 14	E2FF-NFKB	462 - 575	(+)	
**GXP_32458** [GXP_32458] (1 - 871)<br>**PHF15**, GXL_27426, GeneID: 23338, Homo sapiens chr. 5<br>PHD finger protein 15	E2FF-NFKB	291 - 417	(+)	
**GXP_32472** [GXP_32472] (1 - 603)<br>**C5orf24**, GXL_27435, GeneID: 134553, Homo sapiens chr. 5<br>chromosome 5 open reading frame 24	E2FF-NFKB	101 - 222	(+)	
**GXP_32479** [GXP_32479] (1 - 601)<br>**EIF4EBP3,ANKHD1,MASK-BP3**, GXL_27439, GeneID: 8637,54882,404734, Homo sapiens chr. 5<br>eukaryotic translation initiation factor 4E binding protein 3;ankyrin repeat and KH domain containing 1; MASK-4E-BP3 alternate reading frame gene	E2FF-NFKB	178 - 57	(-)	
**GXP_35517** [GXP_35517] (1 - 601)<br>**SFRP1**, GXL_29754, GeneID: 6422, Homo sapiens chr. 8<br>secreted frizzled-related protein 1	E2FF-NFKB	212 - 100	(-)	
**GXP_492892** [GXP_492892] (1 - 1285)<br>**RDHE2**, GXL_29756, GeneID: 195814, Homo sapiens chr. 8<br>retinal short chain dehydrogenase reductase isoform 1	E2FF-NFKB	1093 - 980	(-)	
**GXP_925773** [GXP_925773] (1 - 1479)<br>**ChGn**, GXL_29774, GeneID: 55790, Homo sapiens chr. 8<br>chondroitin beta1,4 N-acetylgalactosaminyltransferase	E2FF-NFKB	1418 - 1299	(-)	

**GXP_660778** [GXP_660778] (1 - 1467)<br>**LONRF1**, GXL_29785, GeneID: 91694, Homo sapiens chr. 8<br>LON peptidase N-terminal domain and ring finger 1	E2FF-NFKB	149 - 27	(-)	
**GXP_925874** [GXP_925874] (1 - 670)<br>**ZNF395,FBXO16**, GXL_29789, GeneID: 55893,157574, Homo sapiens chr. 8<br>zinc finger protein 395;F-box protein 16	E2FF-NFKB	268 - 159	(-)	
**GXP_35604** [GXP_35604] (1 - 629)<br>**XKR6**, GXL_29797, GeneID: 286046, Homo sapiens chr. 8<br>XK, Kell blood group complex subunit-related family, member 6	E2FF-NFKB	481 - 364	(-)	
**GXP_35648** [GXP_35648] (1 - 612)<br>**NKX3-1**, GXL_29827, GeneID: 4824, Homo sapiens chr. 8<br>NK3 transcription factor related, locus 1 (Drosophila)	E2FF-NFKB	230 - 341	(+)	
**GXP_35660** [GXP_35660] (1 - 1095)<br>**RNF170**, GXL_29836, GeneID: 81790, Homo sapiens chr. 8<br>ring finger protein 170	E2FF-NFKB	480 - 374	(-)	
**GXP_35714** [GXP_35714] (1 - 632)<br>**PSD3**, GXL_29880, GeneID: 23362, Homo sapiens chr. 8<br>pleckstrin and Sec7 domain containing 3	E2FF-NFKB	142 - 34	(-)	
**GXP_35719** [GXP_35719] (1 - 602)<br>**FLJ43582**, GXL_29884, GeneID: 389649, Homo sapiens chr. 8<br>FLJ43582 protein	E2FF-NFKB	437 - 554	(+)	
**GXP_35843** [GXP_35843] (1 - 906)<br>**STC1**, GXL_29994, GeneID: 6781, Homo sapiens chr. 8<br>stanniocalcin 1	E2FF-NFKB	764 - 886	(+)	
**GXP_35883** [GXP_35883] (1 - 800)<br>**LYPLA1**, GXL_30030, GeneID: 10434, Homo sapiens chr. 8<br>lysophospholipase I	E2FF-NFKB	734 - 612	(-)	
**GXP_38059** [GXP_38059] (1 - 611)<br>**TRPM3**, GXL_31660, GeneID: 80036, Homo sapiens chr. 9<br>transient receptor potential cation channel, subfamily M, member 3	E2FF-NFKB	211 - 339	(+)	
**GXP_661742** [GXP_661742] (1 - 601)<br>**BAG1**, GXL_31666, GeneID: 573, Homo sapiens chr. 9<br>BCL2-associated athanogene	E2FF-NFKB	241 - 118	(-)	
**GXP_38073** [GXP_38073] (1 - 760)<br>**C9orf95**, GXL_31669, GeneID: 54981, Homo sapiens chr. 9<br>chromosome 9 open reading frame 95	E2FF-NFKB	329 - 436	(+)	
**GXP_38145** [GXP_38145] (1 - 1072)<br>**FAM29A**, GXL_31705, GeneID: 54801, Homo sapiens chr. 9<br>family with sequence similarity 29, member A	E2FF-NFKB	197 - 307	(+)	
	E2FF-NFKB	563 - 677	(+)	
	E2FF-NFKB	444 - 336	(-)	

**GXP_38171** [GXP_38171] (1 - 745)<br>**RP11-138L21.1**, GXL_31725, GeneID: 389722, Homo sapiens chr. 9<br>similar to cell recognition molecule CASPR3				
**GXP_38237** [GXP_38237] (1 - 601)<br>**C9orf25**, GXL_31768, GeneID: 203259, Homo sapiens chr. 9<br>chromosome 9 open reading frame 25	E2FF-NFKB	303 - 412	(+)	
**GXP_38240** [GXP_38240] (1 - 1025)<br>**CNTNAP3,LOC727745**, GXL_31770, GeneID: 79937,727745, Homo sapiens chr. 9<br>contactin associated protein-like 3; similar to cell recognition molecule CASPR3	E2FF-NFKB	460 - 570	(+)	
**GXP_926541** [GXP_926541] (1 - 601)<br>**KIAA1815**, GXL_31791, GeneID: 79956, Homo sapiens chr. 9<br>KIAA1815	E2FF-NFKB	465 - 343	(-)	
**GXP_661799** [GXP_661799] (1 - 601)<br>**GBA2**, GXL_31794, GeneID: 57704, Homo sapiens chr. 9<br>glucosidase, beta (bile acid) 2	E2FF-NFKB	241 - 370	(+)	
**GXP_38293** [GXP_38293] (1 - 601)<br>**C9orf72**, GXL_31809, GeneID: 203228, Homo sapiens chr. 9<br>chromosome 9 open reading frame 72	E2FF-NFKB	271 - 143	(-)	
**GXP_38294** [GXP_38294] (1 - 967)<br>**C9orf72**, GXL_31809, GeneID: 203228, Homo sapiens chr. 9<br>chromosome 9 open reading frame 72	E2FF-NFKB	271 - 143	(-)	
**GXP_926628** [GXP_926628] (1 - 601)<br>**C9orf72**, GXL_31809, GeneID: 203228, Homo sapiens chr. 9<br>chromosome 9 open reading frame 72	E2FF-NFKB	294 - 166	(-)	
**GXP_38340** [GXP_38340] (1 - 601)<br>**TLN1**, GXL_31848, GeneID: 7094, Homo sapiens chr. 9<br>talin 1	E2FF-NFKB	304 - 187	(-)	
**GXP_38367** [GXP_38367] (1 - 912)<br>**RASEF**, GXL_31872, GeneID: 158158, Homo sapiens chr. 9<br>RAS and EF-hand domain containing	E2FF-NFKB	606 - 726	(+)	
**GXP_926870** [GXP_926870] (1 - 601)<br>**APBA1**, GXL_31899, GeneID: 320, Homo sapiens chr. 9<br>amyloid beta (A4) precursor protein-binding, family A, member 1 (X11)	E2FF-NFKB	296 - 177	(-)	
**GXP_926651** [GXP_926651] (1 - 777)<br>**AQP7,LOC730908**, GXL_31901, GeneID: 364,730908, Homo sapiens chr. 9<br>aquaporin 7; similar to Aquaporin-7 (AQP-7) (Aquaporin-7-like) (Aquaporin adipose) (AQPap)	E2FF-NFKB	368 - 244	(-)	
**GXP_38420** [GXP_38420] (1 - 783)<br>**GNAQ**, GXL_31923, GeneID: 2776, Homo sapiens chr. 9<br>guanine nucleotide binding protein (G protein), q polypeptide	E2FF-NFKB	190 - 302	(+)	
	E2FF-NFKB	334 - 215	(-)	

**GXP_38438** [GXP_38438] (1 - 601)<br>**BCL2L12**, GXL_31938, GeneID: 83596, Homo sapiens chr. 19<br>BCL2-like 12 (proline rich)				
**GXP_38443** [GXP_38443] (1 - 816)<br>**ZNF324**, GXL_31941, GeneID: 25799, Homo sapiens chr. 19<br>zinc finger protein 324	E2FF-NFKB	492 - 375	(-)	
	E2FF-NFKB	655 - 765	(+)	
**GXP_38455** [GXP_38455] (1 - 1192)<br>**CACNG6**, GXL_31950, GeneID: 59285, Homo sapiens chr. 19<br>calcium channel, voltage-dependent, gamma subunit 6	E2FF-NFKB	139 - 15	(-)	
**GXP_38467** [GXP_38467] (1 - 644)<br>**ZNF324B**, GXL_31956, GeneID: 388569, Homo sapiens chr. 19<br>zinc finger protein 324B	E2FF-NFKB	633 - 511	(-)	
**GXP_38483** [GXP_38483] (1 - 612)<br>**PPFIA3**, GXL_31967, GeneID: 8541, Homo sapiens chr. 19<br>protein tyrosine phosphatase, receptor type, f polypeptide (PTPRF), interacting protein (liprin), alpha 3	E2FF-NFKB	452 - 564	(+)	
**GXP_913604** [GXP_913604] (1 - 601)<br>**FTL**, GXL_31968, GeneID: 2512, Homo sapiens chr. 19<br>ferritin, light polypeptide	E2FF-NFKB	458 - 334	(-)	
**GXP_38600** [GXP_38600] (1 - 1218)<br>**ZIK1**, GXL_32029, GeneID: 284307, Homo sapiens chr. 19<br>zinc finger protein interacting with K protein 1 homolog (mouse)	E2FF-NFKB	429 - 323	(-)	
**GXP_913607** [GXP_913607] (1 - 601)<br>**RUVBL2**, GXL_32034, GeneID: 10856, Homo sapiens chr. 19<br>RuvB-like 2 (E. coli)	E2FF-NFKB	32 - 140	(+)	
**GXP_38624** [GXP_38624] (1 - 601)<br>**LILRB1**, GXL_32046, GeneID: 10859, Homo sapiens chr. 19<br>leukocyte immunoglobulin-like receptor, subfamily B (with TM and ITIM domains), member 1	E2FF-NFKB	246 - 352	(+)	
**GXP_38668** [GXP_38668] (1 - 723)<br>**PRMT1**, GXL_32079, GeneID: 3276, Homo sapiens chr. 19<br>protein arginine methyltransferase 1	E2FF-NFKB	136 - 256	(+)	
**GXP_649047** [GXP_649047] (1 - 601)<br>**PRMT1**, GXL_32079, GeneID: 3276, Homo sapiens chr. 19<br>protein arginine methyltransferase 1	E2FF-NFKB	251 - 371	(+)	
**GXP_913761** [GXP_913761] (1 - 601)<br>**TSEN34**, GXL_32093, GeneID: 79042, Homo sapiens chr. 19<br>tRNA splicing endonuclease 34 homolog (S. cerevisiae)	E2FF-NFKB	178 - 292	(+)	
**GXP_913763** [GXP_913763] (1 - 601)<br>**TSEN34**, GXL_32093, GeneID: 79042, Homo sapiens chr. 19<br>tRNA splicing endonuclease 34 homolog (S. cerevisiae)	E2FF-NFKB	276 - 167	(-)	
	E2FF-NFKB	718 - 829	(+)	

**GXP_38712** [GXP_38712] (1 - 1156)<br>**CCDC106**, GXL_32117, GeneID: 29903, Homo sapiens chr. 19<br>coiled-coil domain containing 106	E2FF-NFKB<br>E2FF-NFKB<br>E2FF-NFKB	910 - 1021<br>925 - 816<br>1090 - 970	(+)<br>(-)<br>(-)	
**GXP_486215** [GXP_486215] (1 - 601)<br>**U2AF2**, GXL_32127, GeneID: 11338, Homo sapiens chr. 19<br>U2 small nuclear RNA auxiliary factor 2	E2FF-NFKB	525 - 419	(-)	
**GXP_913820** [GXP_913820] (1 - 621)<br>**U2AF2**, GXL_32127, GeneID: 11338, Homo sapiens chr. 19<br>U2 small nuclear RNA auxiliary factor 2	E2FF-NFKB	150 - 27	(-)	
**GXP_913821** [GXP_913821] (1 - 601)<br>**U2AF2**, GXL_32127, GeneID: 11338, Homo sapiens chr. 19<br>U2 small nuclear RNA auxiliary factor 2	E2FF-NFKB	298 - 192	(-)	
**GXP_913636** [GXP_913636] (1 - 608)<br>**PRRG2,PRR12**, GXL_32132, GeneID: 5639,57479, Homo sapiens chr. 19<br>proline rich Gla (G-carboxyglutamic acid) 2; proline rich 12	E2FF-NFKB	457 - 342	(-)	
**GXP_40284** [GXP_40284] (1 - 601)<br>**AUTS2**, GXL_33483, GeneID: 26053, Homo sapiens chr. 7<br>autism susceptibility candidate 2	E2FF-NFKB	305 - 424	(+)	
**GXP_40311** [GXP_40311] (1 - 601)<br>**UPP1**, GXL_33494, GeneID: 7378, Homo sapiens chr. 7<br>uridine phosphorylase 1	E2FF-NFKB	573 - 448	(-)	
**GXP_40409** [GXP_40409] (1 - 638)<br>**RCP9**, GXL_33547, GeneID: 27297, Homo sapiens chr. 7<br>calcitonin gene-related peptide-receptor component protein	E2FF-NFKB	566 - 448	(-)	
**GXP_40496** [GXP_40496] (1 - 822)<br>**LIMK1**, GXL_33601, GeneID: 3984, Homo sapiens chr. 7<br>LIM domain kinase 1	E2FF-NFKB	581 - 455	(-)	
**GXP_40501** [GXP_40501] (1 - 769)<br>**YKT6**, GXL_33603, GeneID: 10652, Homo sapiens chr. 7<br>YKT6 v-SNARE homolog (S. cerevisiae)	E2FF-NFKB	431 - 316	(-)	
**GXP_40535** [GXP_40535] (1 - 1097)<br>**DBNL**, GXL_33631, GeneID: 28988, Homo sapiens chr. 7<br>drebrin-like	E2FF-NFKB	948 - 1070	(+)	
**GXP_659412** [GXP_659412] (1 - 601)<br>**CCM2**, GXL_33633, GeneID: 83605, Homo sapiens chr. 7<br>cerebral cavernous malformation 2	E2FF-NFKB<br>E2FF-NFKB	168 - 281<br>526 - 419	(+)<br>(-)	
**GXP_924826** [GXP_924826] (1 - 601)<br>**RAMP3**, GXL_33637, GeneID: 10268, Homo sapiens chr. 7<br>receptor (G protein-coupled) activity modifying protein 3	E2FF-NFKB	225 - 116	(-)	
	E2FF-NFKB	593 - 703	(+)	

**GXP_40600** [GXP_40600] (1 - 930)<br>**SPATA18**, GXL_33688, GeneID: 132671, Homo sapiens chr. 4<br>spermatogenesis associated 18 homolog (rat)				
**GXP_40605** [GXP_40605] (1 - 619)<br>**SPRY1,LOC729345,LOC730525**, GXL_33692, GeneID: 10252,729345,730525, Homo sapiens chr. 4<br>sprouty homolog 1, antagonist of FGF signaling (Drosophila); hypothetical protein LOC729345; hypothetical protein LOC730525	E2FF-NFKB	266 - 392	(+)	
**GXP_40609** [GXP_40609] (1 - 1012)<br>**HSPA4L**, GXL_33695, GeneID: 22824, Homo sapiens chr. 4<br>heat shock 70kDa protein 4-like	E2FF-NFKB	589 - 698	(+)	
**GXP_40655** [GXP_40655] (1 - 1171)<br>**TMEM165**, GXL_33717, GeneID: 55858, Homo sapiens chr. 4<br>transmembrane protein 165	E2FF-NFKB	720 - 611	(-)	
**GXP_40678** [GXP_40678] (1 - 701)<br>**C4orf29**, GXL_33730, GeneID: 80167, Homo sapiens chr. 4<br>chromosome 4 open reading frame 29	E2FF-NFKB	360 - 468	(+)	
**GXP_40679** [GXP_40679] (1 - 1015)<br>**MAG1**, GXL_33731, GeneID: 84803, Homo sapiens chr. 4<br>lung cancer metastasis-associated protein	E2FF-NFKB	828 - 947	(+)	
**GXP_40684** [GXP_40684] (1 - 601)<br>**PCDH10**, GXL_33733, GeneID: 57575, Homo sapiens chr. 4<br>protocadherin 10	E2FF-NFKB	343 - 229	(-)	
**GXP_40759** [GXP_40759] (1 - 723)<br>**EXOC1**, GXL_33779, GeneID: 55763, Homo sapiens chr. 4<br>exocyst complex component 1	E2FF-NFKB	522 - 650	(+)	
**GXP_921524** [GXP_921524] (1 - 739)<br>**LPHN3**, GXL_33814, GeneID: 23284, Homo sapiens chr. 4<br>latrophilin 3	E2FF-NFKB	326 - 448	(+)	
**GXP_921914** [GXP_921914] (1 - 764)<br>**ZNF330**, GXL_33833, GeneID: 27309, Homo sapiens chr. 4<br>zinc finger protein 330	E2FF-NFKB	460 - 576	(+)	
**GXP_40908** [GXP_40908] (1 - 799)<br>**CXCL6**, GXL_33882, GeneID: 6372, Homo sapiens chr. 4<br>chemokine (C-X-C motif) ligand 6 (granulocyte chemotactic protein 2)	E2FF-NFKB	716 - 589	(-)	
**GXP_40992** [GXP_40992] (1 - 676)<br>**CXCL1**, GXL_33955, GeneID: 2919, Homo sapiens chr. 4<br>chemokine (C-X-C motif) ligand 1 (melanoma growth stimulating activity, alpha)	E2FF-NFKB	385 - 510	(+)	
**GXP_40998** [GXP_40998] (1 - 998)<br>**DCK**, GXL_33961, GeneID: 1633, Homo sapiens chr. 4<br>deoxycytidine kinase	E2FF-NFKB	531 - 408	(-)	
	E2FF-NFKB	261 - 380	(+)	

**GXP_41951** [GXP_41951] (1 - 601)<br>**LZIC**, GXL_34753, GeneID: 84328, Homo sapiens chr. 1<br>leucine zipper and CTNNBIP1 domain containing				
**GXP_41958** [GXP_41958] (1 - 624)<br>**MAD2L2**, GXL_34757, GeneID: 10459, Homo sapiens chr. 1<br>MAD2 mitotic arrest deficient-like 2 (yeast)	E2FF-NFKB	303 - 418	(+)	
**GXP_914066** [GXP_914066] (1 - 1103)<br>**MAD2L2**, GXL_34757, GeneID: 10459, Homo sapiens chr. 1<br>MAD2 mitotic arrest deficient-like 2 (yeast)	E2FF-NFKB	117 - 232	(+)	
	E2FF-NFKB	347 - 462	(+)	
	E2FF-NFKB	563 - 448	(-)	
**GXP_42036** [GXP_42036] (1 - 601)<br>**E2F2**, GXL_34791, GeneID: 1870, Homo sapiens chr. 1<br>E2F transcription factor 2	E2FF-NFKB	121 - 229	(+)	
**GXP_42069** [GXP_42069] (1 - 601)<br>**FLJ14100,C1orf86**, GXL_34809, GeneID: 80093,199990, Homo sapiens chr. 1<br>hypothetical protein FLJ14100; chromosome 1 open reading frame 86	E2FF-NFKB	430 - 317	(-)	
**GXP_42079** [GXP_42079] (1 - 1250)<br>**USP48**, GXL_34815, GeneID: 84196, Homo sapiens chr. 1<br>ubiquitin specific peptidase 48	E2FF-NFKB	1012 - 894	(-)	
**GXP_42156** [GXP_42156] (1 - 907)<br>**DDOST**, GXL_34858, GeneID: 1650, Homo sapiens chr. 1<br>dolichyl-diphosphooligosaccharide-protein glycosyltransferase	E2FF-NFKB	895 - 767	(-)	
**GXP_42201** [GXP_42201] (1 - 601)<br>**TNFRSF14**, GXL_34887, GeneID: 8764, Homo sapiens chr. 1<br>tumor necrosis factor receptor superfamily, member 14 (herpesvirus entry mediator)	E2FF-NFKB	549 - 433	(-)	
**GXP_42208** [GXP_42208] (1 - 680)<br>**C1orf117**, GXL_34894, GeneID: 348487, Homo sapiens chr. 1<br>chromosome 1 open reading frame 117	E2FF-NFKB	160 - 266	(+)	
**GXP_914167** [GXP_914167] (1 - 601)<br>**C1orf117**, GXL_34894, GeneID: 348487, Homo sapiens chr. 1<br>chromosome 1 open reading frame 117	E2FF-NFKB	355 - 476	(+)	
**GXP_914255** [GXP_914255] (1 - 601)<br>**RAP1GAP**, GXL_34941, GeneID: 5909, Homo sapiens chr. 1<br>RAP1 GTPase activating protein	E2FF-NFKB	180 - 304	(+)	
**GXP_914256** [GXP_914256] (1 - 625)<br>**RAP1GAP**, GXL_34941, GeneID: 5909, Homo sapiens chr. 1<br>RAP1 GTPase activating protein	E2FF-NFKB	380 - 255	(-)	
**GXP_42281** [GXP_42281] (1 - 1014)<br>**DHRS3**, GXL_34954, GeneID: 9249, Homo sapiens chr. 1<br>dehydrogenase/reductase (SDR family) member 3	E2FF-NFKB	541 - 651	(+)	
	E2FF-NFKB	317 - 424	(+)	

**GXP_42288** [GXP_42288] (1 - 1088)<br>**KIF17**, GXL_34960, GeneID: 57576, Homo sapiens chr. 1<br>kinesin family member 17				
**GXP_658982** [GXP_658982] (1 - 899)<br>**C7orf20**, GXL_34992, GeneID: 51608, Homo sapiens chr. 7<br>chromosome 7 open reading frame 20	E2FF-NFKB	400 - 286	(-)	
**GXP_924720** [GXP_924720] (1 - 963)<br>**C7orf41**, GXL_35036, GeneID: 222166, Homo sapiens chr. 7<br>chromosome 7 open reading frame 41	E2FF-NFKB	584 - 461	(-)	
**GXP_42444** [GXP_42444] (1 - 815)<br>**KIAA1706,LOC730171,LOC730371,LOC731418**, GXL_35050, GeneID: 80820,730171,730371,731418, Homo sapiens chr. 7<br>KIAA1706 protein;similar to KIAA1706 protein; similar to KIAA1706 protein;similar to KIAA1706 protein	E2FF-NFKB	680 - 792	(+)	
**GXP_42479** [GXP_42479] (1 - 1088)<br>**EVX1**, GXL_35068, GeneID: 2128, Homo sapiens chr. 7<br>even-skipped homeobox 1	E2FF-NFKB	865 - 742	(-)	
**GXP_42491** [GXP_42491] (1 - 657)<br>**ANLN**, GXL_35076, GeneID: 54443, Homo sapiens chr. 7<br>anillin, actin binding protein	E2FF-NFKB	245 - 365	(+)	
**GXP_659028** [GXP_659028] (1 - 674)<br>**LFNG**, GXL_35115, GeneID: 3955, Homo sapiens chr. 7<br>LFNG O-fucosylpeptide 3-beta-N-acetylglucosaminyltransferase	E2FF-NFKB	230 - 358	(+)	
**GXP_659175** [GXP_659175] (1 - 601)<br>**AHR,LOC729939,LOC730361,LOC730716**, GXL_35124, GeneID: 196,729939,730361,730716, Homo sapiens chr. 7<br>aryl hydrocarbon receptor; similar to aryl hydrocarbon receptor; similar to aryl hydrocarbon receptor; similar to aryl hydrocarbon receptor	E2FF-NFKB	595 - 475	(-)	
**GXP_42579** [GXP_42579] (1 - 664)<br>**C1GALT1**, GXL_35131, GeneID: 56913, Homo sapiens chr. 7<br>core 1 synthase, glycoprotein-N-acetylgalactosamine 3-beta-galactosyltransferase, 1	E2FF-NFKB	183 - 303	(+)	
**GXP_42584** [GXP_42584] (1 - 960)<br>**GARS**, GXL_35136, GeneID: 2617, Homo sapiens chr. 7<br>glycyl-tRNA synthetase	E2FF-NFKB	782 - 676	(-)	
**GXP_42585** [GXP_42585] (1 - 601)<br>**ITGB8,LOC729972,LOC730362,LOC730781**, GXL_35137, GeneID: 3696,729972,730362,730781, Homo sapiens chr. 7<br>integrin, beta 8;hypothetical protein LOC729972; hypothetical protein LOC730362; hypothetical protein LOC730781	E2FF-NFKB	297 - 424	(+)	
**GXP_924683** [GXP_924683] (1 - 601)<br>**SNX10**, GXL_35167, GeneID: 29887, Homo sapiens chr. 7<br>sorting nexin 10	E2FF-NFKB	208 - 84	(-)	
**GXP_491932** [GXP_491932] (1 - 601)<br>**KIAA0415**, GXL_35168, GeneID: 9907, Homo sapiens chr. 7<br>KIAA0415 protein	E2FF-NFKB	275 - 159	(-)	
	E2FF-NFKB	256 - 382	(+)	

**GXP_42645** [GXP_42645] (1 - 601)<br>**ZDHHC4**, GXL_35182, GeneID: 55146, Homo sapiens chr. 7<br>zinc finger, DHHC-type containing 4				
**GXP_42655** [GXP_42655] (1 - 951)<br>**PLEKHA8**, GXL_35191, GeneID: 84725, Homo sapiens chr. 7<br>pleckstrin homology domain containing, family A (phosphoinositide binding specific) member 8	E2FF-NFKB	481 - 365	(-)	
**GXP_42680** [GXP_42680] (1 - 938)<br>**JTV1**, GXL_35214, GeneID: 7965, Homo sapiens chr. 7<br>JTV1 gene	E2FF-NFKB	210 - 95	(-)	
	E2FF-NFKB	374 - 486	(+)	
**GXP_647125** [GXP_647125] (1 - 601)<br>**RECQL5**, GXL_35862, GeneID: 9400, Homo sapiens chr. 17<br>RecQ protein-like 5	E2FF-NFKB	475 - 349	(-)	
**GXP_911956** [GXP_911956] (1 - 601)<br>**RECQL5**, GXL_35862, GeneID: 9400, Homo sapiens chr. 17<br>RecQ protein-like 5	E2FF-NFKB	463 - 581	(+)	
**GXP_43374** [GXP_43374] (1 - 705)<br>**FAM104A**, GXL_35863, GeneID: 84923, Homo sapiens chr. 17<br>family with sequence similarity 104, member A	E2FF-NFKB	9 - 122	(+)	
**GXP_43402** [GXP_43402] (1 - 653)<br>**TEX2**, GXL_35876, GeneID: 55852, Homo sapiens chr. 17<br>testis expressed 2	E2FF-NFKB	134 - 11	(-)	
**GXP_43464** [GXP_43464] (1 - 1135)<br>**CHAD**, GXL_35915, GeneID: 1101, Homo sapiens chr. 17<br>chondroadherin	E2FF-NFKB	192 - 313	(+)	
**GXP_43483** [GXP_43483] (1 - 601)<br>**ABCA8**, GXL_35924, GeneID: 10351, Homo sapiens chr. 17<br>ATP-binding cassette, sub-family A (ABC1), member 8	E2FF-NFKB	144 - 259	(+)	
**GXP_43494** [GXP_43494] (1 - 601)<br>**RNF43**, GXL_35929, GeneID: 54894, Homo sapiens chr. 17<br>ring finger protein 43	E2FF-NFKB	410 - 526	(+)	
**GXP_43591** [GXP_43591] (1 - 601)<br>**SDK2**, GXL_35998, GeneID: 54549, Homo sapiens chr. 17<br>sidekick homolog 2 (chicken)	E2FF-NFKB	364 - 493	(+)	
**GXP_43606** [GXP_43606] (1 - 703)<br>**LYK5**, GXL_36009, GeneID: 92335, Homo sapiens chr. 17<br>protein kinase LYK5	E2FF-NFKB	63 - 172	(+)	
**GXP_43619** [GXP_43619] (1 - 1062)<br>**SFRS1**, GXL_36015, GeneID: 6426, Homo sapiens chr. 17<br>splicing factor, arginine/serine-rich 1 (splicing factor 2, alternate splicing factor)	E2FF-NFKB	293 - 414	(+)	
	E2FF-NFKB	882 - 991	(+)	

**GXP_43724** [GXP_43724] (1 - 1068)<br>**HOXB4**, GXL_36109, GeneID: 3214, Homo sapiens chr. 17<br>homeobox B4				
**GXP_43725** [GXP_43725] (1 - 867)<br>**SKAP1**, GXL_36110, GeneID: 8631, Homo sapiens chr. 17<br>src kinase associated phosphoprotein 1	E2FF-NFKB	629 - 504	(-)	
**GXP_44491** [GXP_44491] (1 - 856)<br>**SC65**, GXL_36739, GeneID: 10609, Homo sapiens chr. 17<br>synaptonemal complex protein SC65	E2FF-NFKB	675 - 556	(-)	
**GXP_44522** [GXP_44522] (1 - 974)<br>**HCRT**, GXL_36758, GeneID: 3060, Homo sapiens chr. 17<br>hypocretin (orexin) neuropeptide precursor	E2FF-NFKB	751 - 637	(-)	
**GXP_44526** [GXP_44526] (1 - 1378)<br>**PHF12**, GXL_36760, GeneID: 57649, Homo sapiens chr. 17<br>PHD finger protein 12	E2FF-NFKB	577 - 697	(+)	
**GXP_911401** [GXP_911401] (1 - 601)<br>**PLXDC1**, GXL_36768, GeneID: 57125, Homo sapiens chr. 17<br>plexin domain containing 1	E2FF-NFKB	148 - 260	(+)	
**GXP_44573** [GXP_44573] (1 - 625)<br>**SLFN12**, GXL_36787, GeneID: 55106, Homo sapiens chr. 17<br>schlafen family member 12	E2FF-NFKB	135 - 244	(+)	
**GXP_911313** [GXP_911313] (1 - 686)<br>**SLFN12**, GXL_36787, GeneID: 55106, Homo sapiens chr. 17<br>schlafen family member 12	E2FF-NFKB	82 - 207	(+)	
**GXP_44634** [GXP_44634] (1 - 1480)<br>**GJC1**, GXL_36830, GeneID: 125111, Homo sapiens chr. 17<br>gap junction protein, chi 1, 31.9kDa	E2FF-NFKB	155 - 274	(+)	
**GXP_44666** [GXP_44666] (1 - 601)<br>**SEZ6**, GXL_36845, GeneID: 124925, Homo sapiens chr. 17<br>seizure related 6 homolog (mouse)	E2FF-NFKB	12 - 128	(+)	
**GXP_911356** [GXP_911356] (1 - 601)<br>**ACACA**, GXL_36849, GeneID: 31, Homo sapiens chr. 17<br>acetyl-Coenzyme A carboxylase alpha	E2FF-NFKB	427 - 548	(+)	
**GXP_44724** [GXP_44724] (1 - 625)<br>**HAP1**, GXL_36890, GeneID: 9001, Homo sapiens chr. 17<br>huntingtin-associated protein 1 (neuroan 1)	E2FF-NFKB	309 - 203	(-)	
**GXP_44752** [GXP_44752] (1 - 601)<br>**ETV4**, GXL_36916, GeneID: 2118, Homo sapiens chr. 17<br>ets variant gene 4 (E1A enhancer binding protein, E1AF)	E2FF-NFKB	112 - 225	(+)	
	E2FF-NFKB	44 - 157	(+)	

**GXP_911525** [GXP_911525] (1 - 601)<br>**ETV4**, GXL_36916, GeneID: 2118, Homo sapiens chr. 17<br>ets variant gene 4 (E1A enhancer binding protein, E1AF)				
**GXP_911576** [GXP_911576] (1 - 601)<br>**PLCD3**, GXL_36928, GeneID: 113026, Homo sapiens chr. 17<br>phospholipase C, delta 3	E2FF-NFKB	387 - 260	(-)	
**GXP_44793** [GXP_44793] (1 - 690)<br>**NUFIP2**, GXL_36956, GeneID: 57532, Homo sapiens chr. 17<br>nuclear fragile X mental retardation protein interacting protein 2	E2FF-NFKB	87 - 196	(+)	
**GXP_911244** [GXP_911244] (1 - 601)<br>**NUFIP2**, GXL_36956, GeneID: 57532, Homo sapiens chr. 17<br>nuclear fragile X mental retardation protein interacting protein 2	E2FF-NFKB	420 - 529	(+)	
**GXP_46963** [GXP_46963] (1 - 601)<br>**SERPINH1**, GXL_38894, GeneID: 871, Homo sapiens chr. 11<br>serpin peptidase inhibitor, clade H (heat shock protein 47), member 1, (collagen binding protein 1)	E2FF-NFKB	31 - 156	(+)	
**GXP_46964** [GXP_46964] (1 - 1348)<br>**SERPINH1**, GXL_38894, GeneID: 871, Homo sapiens chr. 11<br>serpin peptidase inhibitor, clade H (heat shock protein 47), member 1, (collagen binding protein 1)	E2FF-NFKB	283 - 408	(+)	
**GXP_46983** [GXP_46983] (1 - 1061)<br>**PANX1,LOC728120,LOC730569**, GXL_38901, GeneID: 24145,728120,730569, Homo sapiens chr. 11<br>pannexin 1;hypothetical protein LOC728120; hypothetical protein LOC730569	E2FF-NFKB	509 - 385	(-)	
**GXP_46993** [GXP_46993] (1 - 602)<br>**PACS1**, GXL_38906, GeneID: 55690, Homo sapiens chr. 11<br>phosphofurin acidic cluster sorting protein 1	E2FF-NFKB	458 - 341	(-)	
**GXP_46997** [GXP_46997] (1 - 601)<br>**ZC3H12C**, GXL_38909, GeneID: 85463, Homo sapiens chr. 11<br>zinc finger CCCH-type containing 12C	E2FF-NFKB	365 - 479	(+)	
**GXP_905993** [GXP_905993] (1 - 601)<br>**ZC3H12C**, GXL_38909, GeneID: 85463, Homo sapiens chr. 11<br>zinc finger CCCH-type containing 12C	E2FF-NFKB	123 - 237	(+)	
**GXP_47024** [GXP_47024] (1 - 655)<br>**SF3B2**, GXL_38923, GeneID: 10992, Homo sapiens chr. 11<br>splicing factor 3b, subunit 2, 145kDa	E2FF-NFKB	290 - 404	(+)	
**GXP_47037** [GXP_47037] (1 - 766)<br>**CAPN5**, GXL_38932, GeneID: 726, Homo sapiens chr. 11<br>calpain 5	E2FF-NFKB	502 - 617	(+)	
**GXP_47057** [GXP_47057] (1 - 799)<br>**TMEM135**, GXL_38945, GeneID: 65084, Homo sapiens chr. 11<br>transmembrane protein 135	E2FF-NFKB	563 - 442	(-)	
	E2FF-NFKB	436 - 542	(+)	

**GXP_906023** [GXP_906023] (1 - 601)<br>**C11orf52**, GXL_38949, GeneID: 91894, Homo sapiens chr. 11<br>chromosome 11 open reading frame 52				
**GXP_47080** [GXP_47080] (1 - 601)<br>**NDUFV1**, GXL_38955, GeneID: 4723, Homo sapiens chr. 11<br>NADH dehydrogenase (ubiquinone) flavoprotein 1, 51kDa	E2FF-NFKB	298 - 413	(+)	
**GXP_47112** [GXP_47112] (1 - 937)<br>**PRSS23**, GXL_38976, GeneID: 11098, Homo sapiens chr. 11<br>protease, serine, 23	E2FF-NFKB	584 - 467	(-)	
**GXP_47124** [GXP_47124] (1 - 686)<br>**DGAT2**, GXL_38983, GeneID: 84649, Homo sapiens chr. 11<br>diacylglycerol O-acyltransferase homolog 2 (mouse)	E2FF-NFKB	419 - 306	(-)	
**GXP_47132** [GXP_47132] (1 - 601)<br>**SIPA1**, GXL_38989, GeneID: 6494, Homo sapiens chr. 11<br>signal-induced proliferation-associated gene 1	E2FF-NFKB	140 - 17	(-)	
**GXP_47173** [GXP_47173] (1 - 601)<br>**DIXDC1**, GXL_39013, GeneID: 85458, Homo sapiens chr. 11<br>DIX domain containing 1	E2FF-NFKB	265 - 377	(+)	
**GXP_480584** [GXP_480584] (1 - 601)<br>**ELMOD1**, GXL_39029, GeneID: 55531, Homo sapiens chr. 11<br>ELMO/CED-12 domain containing 1	E2FF-NFKB	103 - 209	(+)	
**GXP_905704** [GXP_905704] (1 - 601)<br>**CCND1**, GXL_39095, GeneID: 595, Homo sapiens chr. 11<br>cyclin D1	E2FF-NFKB	199 - 306	(+)	
**GXP_47296** [GXP_47296] (1 - 747)<br>**AIP**, GXL_39111, GeneID: 9049, Homo sapiens chr. 11<br>aryl hydrocarbon receptor interacting protein	E2FF-NFKB	512 - 388	(-)	
**GXP_48341** [GXP_48341] (1 - 995)<br>**MRPS18A**, GXL_39983, GeneID: 55168, Homo sapiens chr. 6<br>mitochondrial ribosomal protein S18A	E2FF-NFKB	736 - 611	(-)	
**GXP_48348** [GXP_48348] (1 - 911)<br>**CYP39A1**, GXL_39988, GeneID: 51302, Homo sapiens chr. 6<br>cytochrome P450, family 39, subfamily A, polypeptide 1	E2FF-NFKB	171 - 62	(-)	
**GXP_48351** [GXP_48351] (1 - 687)<br>**ASCC3**, GXL_39990, GeneID: 10973, Homo sapiens chr. 6<br>activating signal cointegrator 1 complex subunit 3	E2FF-NFKB	203 - 323	(+)	
**GXP_48450** [GXP_48450] (1 - 653)<br>**KHDRBS2**, GXL_40041, GeneID: 202559, Homo sapiens chr. 6<br>KH domain containing, RNA binding, signal transduction associated 2	E2FF-NFKB	255 - 141	(-)	
	E2FF-NFKB	613 - 730	(+)	

**GXP_48470** [GXP_48470] (1 - 771)<br>**HMGCLL1**, GXL_40054, GeneID: 54511, Homo sapiens chr. 6<br>3-hydroxymethyl-3-methylglutaryl-Coenzyme A lyase-like 1				
**GXP_48482** [GXP_48482] (1 - 602)<br>**TFEB**, GXL_40059, GeneID: 7942, Homo sapiens chr. 6<br>transcription factor EB	E2FF-NFKB	357 - 248	(-)	
**GXP_923737** [GXP_923737] (1 - 601)<br>**TFEB**, GXL_40059, GeneID: 7942, Homo sapiens chr. 6<br>transcription factor EB	E2FF-NFKB	539 - 430	(-)	
**GXP_48548** [GXP_48548] (1 - 612)<br>**RP11-444E17.2**, GXL_40101, GeneID: 202500, Homo sapiens chr. 6<br>hypothetical protein MGC33600	E2FF-NFKB	358 - 473	(+)	
**GXP_338414** [GXP_338414] (1 - 601)<br>**RP11-444E17.2**, GXL_40101, GeneID: 202500, Homo sapiens chr. 6<br>hypothetical protein MGC33600	E2FF-NFKB	64 - 179	(+)	
**GXP_48573** [GXP_48573] (1 - 602)<br>**MRPL14**, GXL_40117, GeneID: 64928, Homo sapiens chr. 6<br>mitochondrial ribosomal protein L14	E2FF-NFKB<br>E2FF-NFKB	133 - 17<br>479 - 599	(-)<br>(+)	
**GXP_923794** [GXP_923794] (1 - 601)<br>**MRPL14**, GXL_40117, GeneID: 64928, Homo sapiens chr. 6<br>mitochondrial ribosomal protein L14	E2FF-NFKB	331 - 451	(+)	
**GXP_923929** [GXP_923929] (1 - 601)<br>**B3GAT2**, GXL_40121, GeneID: 135152, Homo sapiens chr. 6<br>beta-1, 3-glucuronyltransferase 2 (glucuronosyltransferase S)	E2FF-NFKB	401 - 287	(-)	
**GXP_923743** [GXP_923743] (1 - 601)<br>**TRFP**, GXL_40127, GeneID: 9477, Homo sapiens chr. 6<br>Trf (TATA binding protein-related factor)-proximal homolog (Drosophila)	E2FF-NFKB	493 - 382	(-)	
**GXP_48626** [GXP_48626] (1 - 619)<br>**IBTK**, GXL_40159, GeneID: 25998, Homo sapiens chr. 6<br>inhibitor of Bruton agammaglobulinemia tyrosine kinase	E2FF-NFKB<br>E2FF-NFKB	359 - 235<br>587 - 480	(-)<br>(-)	
**GXP_924075** [GXP_924075] (1 - 706)<br>**ATG5**, GXL_40162, GeneID: 9474, Homo sapiens chr. 6<br>ATG5 autophagy related 5 homolog (S. cerevisiae)	E2FF-NFKB	332 - 440	(+)	
**GXP_48636** [GXP_48636] (1 - 656)<br>**CD164**, GXL_40164, GeneID: 8763, Homo sapiens chr. 6<br>CD164 molecule, sialomucin	E2FF-NFKB	148 - 271	(+)	
**GXP_48645** [GXP_48645] (1 - 694)<br>**C6orf66**, GXL_40171, GeneID: 29078, Homo sapiens chr. 6<br>chromosome 6 open reading frame 66	E2FF-NFKB	302 - 410	(+)	
	E2FF-NFKB	183 - 66	(-)	

**GXP_924070** [GXP_924070] (1 - 617)<br>**BVES**, GXL_40202, GeneID: 11149, Homo sapiens chr. 6<br>blood vessel epicardial substance				
**GXP_48691** [GXP_48691] (1 - 1083)<br>**PREP,LOC728180,LOC731921**, GXL_40210, GeneID: 5550,728180,731921, Homo sapiens chr. 6<br>prolyl endopeptidase;hypothetical protein LOC728180; hypothetical protein LOC731921	E2FF-NFKB	849 - 974	(+)	
**GXP_48698** [GXP_48698] (1 - 737)<br>**GTPBP2**, GXL_40217, GeneID: 54676, Homo sapiens chr. 6<br>GTP binding protein 2	E2FF-NFKB	298 - 415	(+)	
**GXP_923785** [GXP_923785] (1 - 601)<br>**GTPBP2**, GXL_40217, GeneID: 54676, Homo sapiens chr. 6<br>GTP binding protein 2	E2FF-NFKB	571 - 455	(-)	
**GXP_48709** [GXP_48709] (1 - 601)<br>**GSTA1**, GXL_40227, GeneID: 2938, Homo sapiens chr. 6<br>glutathione S-transferase A1	E2FF-NFKB	555 - 449	(-)	
**GXP_48716** [GXP_48716] (1 - 903)<br>**CGA**, GXL_40234, GeneID: 1081, Homo sapiens chr. 6<br>glycoprotein hormones, alpha polypeptide	E2FF-NFKB	606 - 730	(+)	
**GXP_924027** [GXP_924027] (1 - 601)<br>**BACH2**, GXL_40247, GeneID: 60468, Homo sapiens chr. 6<br>BTB and CNC homology 1, basic leucine zipper transcription factor 2	E2FF-NFKB	426 - 538	(+)	
**GXP_642733** [GXP_642733] (1 - 1144)<br>**SLAIN1**, GXL_40537, GeneID: 122060, Homo sapiens chr. 13<br>SLAIN motif family, member 1	E2FF-NFKB	115 - 235	(+)	
**GXP_49107** [GXP_49107] (1 - 601)<br>**RAP2A,LOC121906**, GXL_40549, GeneID: 5911,121906, Homo sapiens chr. 13<br>RAP2A, member of RAS oncogene family; similar to Proteasome subunit alpha type 6 (Proteasome iota chain) (Macropain iota chain) (Multicatalytic endopeptidase complex iota chain)	E2FF-NFKB	467 - 593	(+)	
**GXP_49141** [GXP_49141] (1 - 1182)<br>**PCID2**, GXL_40570, GeneID: 55795, Homo sapiens chr. 13<br>PCI domain containing 2	E2FF-NFKB	168 - 52	(-)	
**GXP_49294** [GXP_49294] (1 - 601)<br>**AKAP11**, GXL_40654, GeneID: 11215, Homo sapiens chr. 13<br>A kinase (PRKA) anchor protein 11	E2FF-NFKB	307 - 198	(-)	
**GXP_49335** [GXP_49335] (1 - 1018)<br>**SUGT1**, GXL_40681, GeneID: 10910, Homo sapiens chr. 13<br>SGT1, suppressor of G2 allele of SKP1 (S. cerevisiae)	E2FF-NFKB	305 - 196	(-)	
**GXP_49353** [GXP_49353] (1 - 787)<br>**ITM2B**, GXL_40696, GeneID: 9445, Homo sapiens chr. 13<br>integral membrane protein 2B	E2FF-NFKB	130 - 242	(+)	

**GXP_49458** [GXP_49458] (1 - 1186)<br>**C13orf24**, GXL_40782, GeneID: 10464, Homo sapiens chr. 13<br>chromosome 13 open reading frame 24	E2FF-NFKB	938 - 1049	(+)	
**GXP_908032** [GXP_908032] (1 - 601)<br>**FAM70B**, GXL_40785, GeneID: 348013, Homo sapiens chr. 13<br>family with sequence similarity 70, member B	E2FF-NFKB	143 - 17	(-)	
**GXP_905667** [GXP_905667] (1 - 601)<br>**NUDT8**, GXL_41455, GeneID: 254552, Homo sapiens chr. 11<br>nudix (nucleoside diphosphate linked moiety X)-type motif 8	E2FF-NFKB	178 - 49	(-)	
**GXP_905361** [GXP_905361] (1 - 1293)<br>**SLC43A3**, GXL_41489, GeneID: 29015, Homo sapiens chr. 11<br>solute carrier family 43, member 3	E2FF-NFKB	737 - 628	(-)	
**GXP_50338** [GXP_50338] (1 - 809)<br>**B3GAT3**, GXL_41542, GeneID: 26229, Homo sapiens chr. 11<br>beta-1, 3-glucuronyltransferase 3 (glucuronosyltransferase I)	E2FF-NFKB	439 - 325	(-)	
**GXP_50390** [GXP_50390] (1 - 1184)<br>**SHANK2**, GXL_41576, GeneID: 22941, Homo sapiens chr. 11<br>SH3 and multiple ankyrin repeat domains 2	E2FF-NFKB	438 - 318	(-)	
**GXP_480054** [GXP_480054] (1 - 845)<br>**LRP4**, GXL_41578, GeneID: 4038, Homo sapiens chr. 11<br>low density lipoprotein receptor-related protein 4	E2FF-NFKB	447 - 553	(+)	
	E2FF-NFKB	783 - 664	(-)	
**GXP_50398** [GXP_50398] (1 - 601)<br>**CDCA5**, GXL_41580, GeneID: 113130, Homo sapiens chr. 11<br>cell division cycle associated 5	E2FF-NFKB	504 - 391	(-)	
**GXP_480361** [GXP_480361] (1 - 601)<br>**PPP1CA**, GXL_41584, GeneID: 5499, Homo sapiens chr. 11<br>protein phosphatase 1, catalytic subunit, alpha isoform	E2FF-NFKB	221 - 335	(+)	
**GXP_50426** [GXP_50426] (1 - 936)<br>**MRGPRF**, GXL_41598, GeneID: 219928, Homo sapiens chr. 11<br>MAS-related GPR, member F	E2FF-NFKB	749 - 638	(-)	
**GXP_50438** [GXP_50438] (1 - 1159)<br>**HSPC152**, GXL_41609, GeneID: 51504, Homo sapiens chr. 11<br>hypothetical protein HSPC152	E2FF-NFKB	869 - 994	(+)	
**GXP_50446** [GXP_50446] (1 - 601)<br>**SLC15A3**, GXL_41617, GeneID: 51296, Homo sapiens chr. 11<br>solute carrier family 15, member 3	E2FF-NFKB	227 - 353	(+)	
**GXP_50471** [GXP_50471] (1 - 1185)<br>**SLC29A2**, GXL_41635, GeneID: 3177, Homo sapiens chr. 11<br>solute carrier family 29 (nucleoside transporters), member 2	E2FF-NFKB	1097 - 975	(-)	
	E2FF-NFKB	506 - 400	(-)	

**GXP_905679** [GXP_905679] (1 - 623)<br>**UNC93B1**, GXL_41690, GeneID: 81622, Homo sapiens chr. 11<br>unc-93 homolog B1 (C. elegans)				
**GXP_50547** [GXP_50547] (1 - 996)<br>**MAP4K2**, GXL_41705, GeneID: 5871, Homo sapiens chr. 11<br>mitogen-activated protein kinase kinase kinase kinase 2	E2FF-NFKB	633 - 515	(-)	
**GXP_50882** [GXP_50882] (1 - 694)<br>**PPM1B**, GXL_42020, GeneID: 5495, Homo sapiens chr. 2<br>protein phosphatase 1B (formerly 2C), magnesium-dependent, beta isoform	E2FF-NFKB	488 - 616	(+)	
**GXP_918185** [GXP_918185] (1 - 601)<br>**MTA3,hCG_1783907,LOC731342**, GXL_42030, GeneID: 57504,728786,731342, Homo sapiens chr. 2<br>metastasis associated 1 family, member 3; metastasis associated 1 family, member 3; similar to metastasis associated 1 family, member 3	E2FF-NFKB	379 - 260	(-)	
**GXP_50948** [GXP_50948] (1 - 602)<br>**ATP6V1C2**, GXL_42048, GeneID: 245973, Homo sapiens chr. 2<br>ATPase, H+ transporting, lysosomal 42kDa, V1 subunit C2	E2FF-NFKB	192 - 69	(-)	
**GXP_917930** [GXP_917930] (1 - 799)<br>**IAH1**, GXL_42050, GeneID: 285148, Homo sapiens chr. 2<br>isoamyl acetate-hydrolyzing esterase 1 homolog (S. cerevisiae)	E2FF-NFKB	726 - 612	(-)	
**GXP_51068** [GXP_51068] (1 - 793)<br>**RRM2**, GXL_42124, GeneID: 6241, Homo sapiens chr. 2<br>ribonucleotide reductase M2 polypeptide	E2FF-NFKB	306 - 416	(+)	
**GXP_917978** [GXP_917978] (1 - 601)<br>**VSNL1**, GXL_42131, GeneID: 7447, Homo sapiens chr. 2<br>visinin-like 1	E2FF-NFKB	392 - 270	(-)	
**GXP_51095** [GXP_51095] (1 - 663)<br>**MSH6**, GXL_42143, GeneID: 2956, Homo sapiens chr. 2<br>mutS homolog 6 (E. coli)	E2FF-NFKB	72 - 178	(+)	
**GXP_51097** [GXP_51097] (1 - 710)<br>**GRHL1**, GXL_42144, GeneID: 29841, Homo sapiens chr. 2<br>grainyhead-like 1 (Drosophila)	E2FF-NFKB	217 - 110	(-)	
**GXP_917935** [GXP_917935] (1 - 601)<br>**GRHL1**, GXL_42144, GeneID: 29841, Homo sapiens chr. 2<br>grainyhead-like 1 (Drosophila)	E2FF-NFKB	601 - 494	(-)	
**GXP_51149** [GXP_51149] (1 - 601)<br>**KCNF1**, GXL_42180, GeneID: 3754, Homo sapiens chr. 2<br>potassium voltage-gated channel, subfamily F, member 1	E2FF-NFKB	233 - 121	(-)	
**GXP_51164** [GXP_51164] (1 - 646)<br>**FLJ21839**, GXL_42191, GeneID: 60509, Homo sapiens chr. 2<br>hypothetical protein FLJ21839	E2FF-NFKB	326 - 204	(-)	
	E2FF-NFKB	384 - 262	(-)	

**GXP_918058** [GXP_918058] (1 - 601)<br>**FLJ21839**, GXL_42191, GeneID: 60509, Homo sapiens chr. 2<br>hypothetical protein FLJ21839				
**GXP_51292** [GXP_51292] (1 - 624)<br>**C8orf79**, GXL_42300, GeneID: 286032, Homo sapiens chr. 8<br>chromosome 8 open reading frame 79	E2FF-NFKB	514 - 623	(+)	
**GXP_660779** [GXP_660779] (1 - 1072)<br>**C8orf79**, GXL_42300, GeneID: 286032, Homo sapiens chr. 8<br>chromosome 8 open reading frame 79	E2FF-NFKB	202 - 311	(+)	
	E2FF-NFKB	677 - 800	(+)	
**GXP_660751** [GXP_660751] (1 - 885)<br>**GATA4**, GXL_42301, GeneID: 2626, Homo sapiens chr. 8<br>GATA binding protein 4	E2FF-NFKB	679 - 561	(-)	
**GXP_926503** [GXP_926503] (1 - 1549)<br>**ZNF251**, GXL_42306, GeneID: 90987, Homo sapiens chr. 8<br>zinc finger protein 251	E2FF-NFKB	1008 - 883	(-)	
**GXP_51321** [GXP_51321] (1 - 687)<br>**EPHX2**, GXL_42319, GeneID: 2053, Homo sapiens chr. 8<br>epoxide hydrolase 2, cytoplasmic	E2FF-NFKB	584 - 477	(-)	
**GXP_51325** [GXP_51325] (1 - 729)<br>**PLEC1,LOC652460**, GXL_42320, GeneID: 5339,652460, Homo sapiens chr. 8<br>plectin 1, intermediate filament binding protein 500kDa; similar to Plectin 1 (PLTN) (PCN) (Hemidesmosomal protein 1) (HD1)	E2FF-NFKB	462 - 355	(-)	
**GXP_926460** [GXP_926460] (1 - 601)<br>**PLEC1,LOC652460**, GXL_42320, GeneID: 5339,652460, Homo sapiens chr. 8<br>plectin 1, intermediate filament binding protein 500kDa; similar to Plectin 1 (PLTN) (PCN) (Hemidesmosomal protein 1) (HD1)	E2FF-NFKB	166 - 50	(-)	
**GXP_926461** [GXP_926461] (1 - 601)<br>**PLEC1,LOC652460**, GXL_42320, GeneID: 5339,652460, Homo sapiens chr. 8<br>plectin 1, intermediate filament binding protein 500kDa; similar to Plectin 1 (PLTN) (PCN) (Hemidesmosomal protein 1) (HD1)	E2FF-NFKB	226 - 110	(-)	
**GXP_51332** [GXP_51332] (1 - 602)<br>**POTE8**, GXL_42322, GeneID: 340441, Homo sapiens chr. 8<br>protein expressed in prostate, ovary, testis, and placenta 8	E2FF-NFKB	487 - 371	(-)	
**GXP_51350** [GXP_51350] (1 - 602)<br>**SLC25A37**, GXL_42330, GeneID: 51312, Homo sapiens chr. 8<br>solute carrier family 25, member 37	E2FF-NFKB	588 - 472	(-)	
**GXP_660940** [GXP_660940] (1 - 1094)<br>**NRG1**, GXL_42336, GeneID: 3084, Homo sapiens chr. 8<br>neuregulin 1	E2FF-NFKB	443 - 327	(-)	
**GXP_51374** [GXP_51374] (1 - 602)<br>**PTK2B**, GXL_42339, GeneID: 2185, Homo sapiens chr. 8<br>PTK2B protein tyrosine kinase 2 beta	E2FF-NFKB	458 - 565	(+)	
	E2FF-NFKB	295 - 169	(-)	

**GXP_925850** [GXP_925850] (1 - 602)<br>**PTK2B**, GXL_42339, GeneID: 2185, Homo sapiens chr. 8<br>PTK2B protein tyrosine kinase 2 beta				
**GXP_660931** [GXP_660931] (1 - 601)<br>**LEPROTL1**, GXL_42352, GeneID: 23484, Homo sapiens chr. 8<br>leptin receptor overlapping transcript-like 1	E2FF-NFKB	371 - 257	(-)	
**GXP_51425** [GXP_51425] (1 - 1460)<br>**BMP1**, GXL_42368, GeneID: 649, Homo sapiens chr. 8<br>bone morphogenetic protein 1	E2FF-NFKB	862 - 738	(-)	
**GXP_51439** [GXP_51439] (1 - 1154)<br>**FBXO25**, GXL_42376, GeneID: 26260, Homo sapiens chr. 8<br>F-box protein 25	E2FF-NFKB	491 - 617	(+)	
**GXP_660989** [GXP_660989] (1 - 633)<br>**PLEKHA2**, GXL_42387, GeneID: 59339, Homo sapiens chr. 8<br>pleckstrin homology domain containing, family A (phosphoinositide binding specific) member 2	E2FF-NFKB	300 - 418	(+)	
**GXP_51488** [GXP_51488] (1 - 624)<br>**CHMP7**, GXL_42411, GeneID: 91782, Homo sapiens chr. 8<br>CHMP family, member 7	E2FF-NFKB	302 - 185	(-)	
**GXP_51492** [GXP_51492] (1 - 844)<br>**HOOK3**, GXL_42414, GeneID: 84376, Homo sapiens chr. 8<br>hook homolog 3 (Drosophila)	E2FF-NFKB	729 - 835	(+)	
**GXP_51511** [GXP_51511] (1 - 774)<br>**SCARA3**, GXL_42429, GeneID: 51435, Homo sapiens chr. 8<br>scavenger receptor class A, member 3	E2FF-NFKB	734 - 624	(-)	
**GXP_51535** [GXP_51535] (1 - 680)<br>**FBXL6,C8ORFK29**, GXL_42446, GeneID: 26233,340393, Homo sapiens chr. 8<br>F-box and leucine-rich repeat protein 6; hypothetical LOC340393	E2FF-NFKB	301 - 192	(-)	
**GXP_51584** [GXP_51584] (1 - 786)<br>**ADAMDEC1**, GXL_42486, GeneID: 27299, Homo sapiens chr. 8<br>ADAM-like, decysin 1	E2FF-NFKB	551 - 666	(+)	
**GXP_51614** [GXP_51614] (1 - 834)<br>**EIF4EBP1**, GXL_42507, GeneID: 1978, Homo sapiens chr. 8<br>eukaryotic translation initiation factor 4E binding protein 1	E2FF-NFKB	418 - 312	(-)	
**GXP_51640** [GXP_51640] (1 - 963)<br>**RPL8**, GXL_42531, GeneID: 6132, Homo sapiens chr. 8<br>ribosomal protein L8	E2FF-NFKB	919 - 802	(-)	
**GXP_51662** [GXP_51662] (1 - 601)<br>**KBTBD11**, GXL_42552, GeneID: 9920, Homo sapiens chr. 8<br>kelch repeat and BTB (POZ) domain containing 11	E2FF-NFKB	319 - 204	(-)	
	E2FF-NFKB	236 - 354	(+)	

**GXP_52653** [GXP_52653] (1 - 601)<br>**HIST1H3I**, GXL_43372, GeneID: 8354, Homo sapiens chr. 6<br>histone cluster 1, H3i				
**GXP_52684** [GXP_52684] (1 - 651)<br>**MUTED,TXNDC5**, GXL_43395, GeneID: 63915,81567, Homo sapiens chr. 6<br>muted homolog (mouse);thioredoxin domain containing 5	E2FF-NFKB	286 - 410	(+)	
**GXP_52688** [GXP_52688] (1 - 663)<br>**DEK**, GXL_43398, GeneID: 7913, Homo sapiens chr. 6<br>DEK oncogene (DNA binding)	E2FF-NFKB	468 - 348	(-)	
**GXP_52691** [GXP_52691] (1 - 601)<br>**KIAA1949**, GXL_43401, GeneID: 170954, Homo sapiens chr. 6<br>KIAA1949	E2FF-NFKB	180 - 51	(-)	
**GXP_491063** [GXP_491063] (1 - 1157)<br>**TBC1D7**, GXL_43404, GeneID: 51256, Homo sapiens chr. 6<br>TBC1 domain family, member 7	E2FF-NFKB	330 - 224	(-)	
**GXP_923403** [GXP_923403] (1 - 699)<br>**TBC1D7**, GXL_43404, GeneID: 51256, Homo sapiens chr. 6<br>TBC1 domain family, member 7	E2FF-NFKB	262 - 156	(-)	
**GXP_52704** [GXP_52704] (1 - 644)<br>**SERPINB9**, GXL_43410, GeneID: 5272, Homo sapiens chr. 6<br>serpin peptidase inhibitor, clade B (ovalbumin), member 9	E2FF-NFKB	185 - 61	(-)	
**GXP_52710** [GXP_52710] (1 - 601)<br>**KIF13A**, GXL_43414, GeneID: 63971, Homo sapiens chr. 6<br>kinesin family member 13A	E2FF-NFKB	436 - 308	(-)	
**GXP_52766** [GXP_52766] (1 - 601)<br>**NEDD9**, GXL_43451, GeneID: 4739, Homo sapiens chr. 6<br>neural precursor cell expressed, developmentally down-regulated 9	E2FF-NFKB	396 - 522	(+)	
**GXP_52820** [GXP_52820] (1 - 792)<br>**NRM**, GXL_43486, GeneID: 11270, Homo sapiens chr. 6<br>nurim (nuclear envelope membrane protein)	E2FF-NFKB	756 - 642	(-)	
**GXP_923533** [GXP_923533] (1 - 601)<br>**NRM**, GXL_43486, GeneID: 11270, Homo sapiens chr. 6<br>nurim (nuclear envelope membrane protein)	E2FF-NFKB	317 - 203	(-)	
**GXP_923595** [GXP_923595] (1 - 601)<br>**PBX2**, GXL_43514, GeneID: 5089, Homo sapiens chr. 6<br>pre-B-cell leukemia homeobox 2	E2FF-NFKB	160 - 275	(+)	
**GXP_52858** [GXP_52858] (1 - 601)<br>**GMDS**, GXL_43515, GeneID: 2762, Homo sapiens chr. 6<br>GDP-mannose 4,6-dehydratase	E2FF-NFKB	153 - 37	(-)	
	E2FF-NFKB	490 - 596	(+)	

**GXP_52868** [GXP_52868] (1 - 705)<br>**SLC17A2**, GXL_43523, GeneID: 10246, Homo sapiens chr. 6<br>solute carrier family 17 (sodium phosphate), member 2				
**GXP_52901** [GXP_52901] (1 - 601)<br>**GNL1**, GXL_43548, GeneID: 2794, Homo sapiens chr. 6<br>guanine nucleotide binding protein-like 1	E2FF-NFKB	584 - 477	(-)	
**GXP_52909** [GXP_52909] (1 - 799)<br>**RXRB**, GXL_43556, GeneID: 6257, Homo sapiens chr. 6<br>retinoid X receptor, beta	E2FF-NFKB	724 - 608	(-)	
**GXP_52928** [GXP_52928] (1 - 750)<br>**SSR1**, GXL_43573, GeneID: 6745, Homo sapiens chr. 6<br>signal sequence receptor, alpha (translocon-associated protein alpha)	E2FF-NFKB	604 - 484	(-)	
**GXP_923462** [GXP_923462] (1 - 699)<br>**C6orf32**, GXL_43592, GeneID: 9750, Homo sapiens chr. 6<br>chromosome 6 open reading frame 32	E2FF-NFKB	275 - 159	(-)	
**GXP_52989** [GXP_52989] (1 - 943)<br>**WDR46**, GXL_43633, GeneID: 9277, Homo sapiens chr. 6<br>WD repeat domain 46	E2FF-NFKB	898 - 784	(-)	
**GXP_491044** [GXP_491044] (1 - 1380)<br>**TFAP2A**, GXL_43639, GeneID: 7020, Homo sapiens chr. 6<br>transcription factor AP-2 alpha (activating enhancer binding protein 2 alpha)	E2FF-NFKB	1190 - 1316	(+)	
**GXP_54898** [GXP_54898] (1 - 601)<br>**DOK3**, GXL_45230, GeneID: 79930, Homo sapiens chr. 5<br>docking protein 3	E2FF-NFKB	486 - 592	(+)	
**GXP_923228** [GXP_923228] (1 - 658)<br>**DOK3**, GXL_45230, GeneID: 79930, Homo sapiens chr. 5<br>docking protein 3	E2FF-NFKB	230 - 336	(+)	
**GXP_923229** [GXP_923229] (1 - 1132)<br>**DOK3**, GXL_45230, GeneID: 79930, Homo sapiens chr. 5<br>docking protein 3	E2FF-NFKB	1016 - 1122	(+)	
**GXP_54964** [GXP_54964] (1 - 843)<br>**GFPT2**, GXL_45265, GeneID: 9945, Homo sapiens chr. 5<br>glutamine-fructose-6-phosphate transaminase 2	E2FF-NFKB	259 - 384	(+)	
**GXP_54966** [GXP_54966] (1 - 1169)<br>**RAB24**, GXL_45266, GeneID: 53917, Homo sapiens chr. 5<br>RAB24, member RAS oncogene family	E2FF-NFKB	816 - 687	(-)	
**GXP_923215** [GXP_923215] (1 - 973)<br>**RAB24**, GXL_45266, GeneID: 53917, Homo sapiens chr. 5<br>RAB24, member RAS oncogene family	E2FF-NFKB	319 - 203	(-)	
	E2FF-NFKB	401 - 277	(-)	

**GXP_923099** [GXP_923099] (1 - 601)<br>**FLJ31951**, GXL_45270, GeneID: 153830, Homo sapiens chr. 5<br>hypothetical protein FLJ31951				
**GXP_923201** [GXP_923201] (1 - 995)<br>**SNCB,LOC652730**, GXL_45290, GeneID: 6620,652730, Homo sapiens chr. 5<br>synuclein, beta;hypothetical protein LOC652730	E2FF-NFKB	468 - 361	(-)	
**GXP_55039** [GXP_55039] (1 - 640)<br>**MGAT1**, GXL_45309, GeneID: 4245, Homo sapiens chr. 5<br>mannosyl (alpha-1,3-)-glycoprotein beta-1, 2-N-acetylglucosaminyltransferase	E2FF-NFKB	279 - 387	(+)	
**GXP_490998** [GXP_490998] (1 - 601)<br>**RASGEF1C,LOC729618**, GXL_45338, GeneID: 255426,729618, Homo sapiens chr. 5<br>RasGEF domain family, member 1C; hypothetical protein LOC729618	E2FF-NFKB	150 - 43	(-)	
**GXP_55087** [GXP_55087] (1 - 811)<br>**OSMR**, GXL_45342, GeneID: 9180, Homo sapiens chr. 5<br>oncostatin M receptor	E2FF-NFKB	639 - 526	(-)	
**GXP_922301** [GXP_922301] (1 - 601)<br>**SUB1**, GXL_45399, GeneID: 10923, Homo sapiens chr. 5<br>SUB1 homolog (S. cerevisiae)	E2FF-NFKB	382 - 496	(+)	
**GXP_912019** [GXP_912019] (1 - 601)<br>**HRNBP3**, GXL_45780, GeneID: 146713, Homo sapiens chr. 17<br>hypothetical protein LOC146713	E2FF-NFKB	547 - 422	(-)	
**GXP_55757** [GXP_55757] (1 - 926)<br>**ARRB2**, GXL_45800, GeneID: 409, Homo sapiens chr. 17<br>arrestin, beta 2	E2FF-NFKB	809 - 915	(+)	
**GXP_55762** [GXP_55762] (1 - 601)<br>**AATK,LOC651771**, GXL_45803, GeneID: 9625,651771, Homo sapiens chr. 17<br>apoptosis-associated tyrosine kinase; similar to apoptosis-associated tyrosine kinase	E2FF-NFKB	99 - 228	(+)	
**GXP_910922** [GXP_910922] (1 - 601)<br>**KIAA0523**, GXL_45804, GeneID: 23302, Homo sapiens chr. 17<br>KIAA0523 protein	E2FF-NFKB	15 - 132	(+)	
**GXP_55785** [GXP_55785] (1 - 1302)<br>**SIRT7,LOC644124**, GXL_45817, GeneID: 51547,644124, Homo sapiens chr. 17<br>sirtuin (silent mating type information regulation 2 homolog) 7 (S. cerevisiae); similar to NAD-dependent deacetylase sirtuin-7 (SIR2-like protein 7)	E2FF-NFKB	1140 - 1246	(+)	
**GXP_55786** [GXP_55786] (1 - 601)<br>**SIRT7,LOC644124**, GXL_45817, GeneID: 51547,644124, Homo sapiens chr. 17<br>sirtuin (silent mating type information regulation 2 homolog) 7 (S. cerevisiae); similar to NAD-dependent deacetylase sirtuin-7 (SIR2-like protein 7)	E2FF-NFKB	439 - 545	(+)	
**GXP_646176** [GXP_646176] (1 - 628)<br>**LRRC48**, GXL_45818, GeneID: 83450, Homo sapiens chr. 17	E2FF-NFKB	425 - 312	(-)	

leucine rich repeat containing 48				
**GXP_55821** [GXP_55821] (1 - 601)<br>**RNF167**, GXL_45838, GeneID: 26001, Homo sapiens chr. 17<br>ring finger protein 167	E2FF-NFKB	136 - 25	(-)	
**GXP_55832** [GXP_55832] (1 - 601)<br>**RUTBC1**, GXL_45846, GeneID: 9905, Homo sapiens chr. 17<br>RUN and TBC1 domain containing 1	E2FF-NFKB	13 - 140	(+)	
**GXP_911032** [GXP_911032] (1 - 601)<br>**USP43**, GXL_45847, GeneID: 124739, Homo sapiens chr. 17<br>ubiquitin specific peptidase 43	E2FF-NFKB	467 - 341	(-)	
**GXP_912109** [GXP_912109] (1 - 601)<br>**C17orf62**, GXL_45861, GeneID: 79415, Homo sapiens chr. 17<br>chromosome 17 open reading frame 62	E2FF-NFKB	220 - 94	(-)	
**GXP_55894** [GXP_55894] (1 - 668)<br>**C17orf56**, GXL_45879, GeneID: 146705, Homo sapiens chr. 17<br>chromosome 17 open reading frame 56	E2FF-NFKB	584 - 478	(-)	
**GXP_912056** [GXP_912056] (1 - 601)<br>**C17orf56**, GXL_45879, GeneID: 146705, Homo sapiens chr. 17<br>chromosome 17 open reading frame 56	E2FF-NFKB	245 - 139	(-)	
**GXP_55936** [GXP_55936] (1 - 1075)<br>**C17orf39**, GXL_45903, GeneID: 79018, Homo sapiens chr. 17<br>chromosome 17 open reading frame 39	E2FF-NFKB	964 - 849	(-)	
**GXP_55940** [GXP_55940] (1 - 879)<br>**PYCR1,LOC728891**, GXL_45906, GeneID: 5831,728891, Homo sapiens chr. 17<br>pyrroline-5-carboxylate reductase 1; similar to pyrroline-5-carboxylate reductase family, member 2	E2FF-NFKB	267 - 142	(-)	
	E2FF-NFKB	399 - 527	(+)	
**GXP_912081** [GXP_912081] (1 - 1084)<br>**PYCR1,LOC728891**, GXL_45906, GeneID: 5831,728891, Homo sapiens chr. 17<br>pyrroline-5-carboxylate reductase 1; similar to pyrroline-5-carboxylate reductase family, member 2	E2FF-NFKB	471 - 346	(-)	
	E2FF-NFKB	603 - 731	(+)	
**GXP_912012** [GXP_912012] (1 - 601)<br>**LGALS3BP**, GXL_45917, GeneID: 3959, Homo sapiens chr. 17<br>lectin, galactoside-binding, soluble, 3 binding protein	E2FF-NFKB	155 - 38	(-)	
**GXP_55958** [GXP_55958] (1 - 848)<br>**MIS12**, GXL_45918, GeneID: 79003, Homo sapiens chr. 17<br>MIS12, MIND kinetochore complex component, homolog (yeast)	E2FF-NFKB	128 - 5	(-)	
**GXP_55976** [GXP_55976] (1 - 616)<br>**ST6GALNAC2**, GXL_45932, GeneID: 10610, Homo sapiens chr. 17<br>ST6 (alpha-N-acetyl-neuraminyl-2,3-beta-galactosyl-1, 3)-N-acetylgalactosaminide alpha-2,6-sialyltransferase 2	E2FF-NFKB	442 - 567	(+)	
**GXP_56005** [GXP_56005] (1 - 601)<br>**GARNL4**, GXL_45955, GeneID: 23108, Homo sapiens chr. 17<br>GTPase activating Rap/RanGAP domain-like 4	E2FF-NFKB	252 - 143	(-)	

**GXP_56028** [GXP_56028] (1 - 926)<br>**JMJD6**, GXL_45974, GeneID: 23210, Homo sapiens chr. 17<br>jumonji domain containing 6	E2FF-NFKB	458 - 343	(-)	
**GXP_56033** [GXP_56033] (1 - 601)<br>**TIMP2**, GXL_45979, GeneID: 7077, Homo sapiens chr. 17<br>TIMP metallopeptidase inhibitor 2	E2FF-NFKB	227 - 337	(+)	
**GXP_57111** [GXP_57111] (1 - 1077)<br>**C1orf94**, GXL_46922, GeneID: 84970, Homo sapiens chr. 1<br>chromosome 1 open reading frame 94	E2FF-NFKB	293 - 410	(+)	
**GXP_57125** [GXP_57125] (1 - 628)<br>**ALG6**, GXL_46929, GeneID: 29929, Homo sapiens chr. 1<br>asparagine-linked glycosylation 6 homolog (S. cerevisiae, alpha-1,3-glucosyltransferase)	E2FF-NFKB	400 - 282	(-)	
**GXP_636013** [GXP_636013] (1 - 601)<br>**ALG6**, GXL_46929, GeneID: 29929, Homo sapiens chr. 1<br>asparagine-linked glycosylation 6 homolog (S. cerevisiae, alpha-1,3-glucosyltransferase)	E2FF-NFKB	289 - 171	(-)	
**GXP_57142** [GXP_57142] (1 - 601)<br>**CACHD1**, GXL_46933, GeneID: 57685, Homo sapiens chr. 1<br>cache domain containing 1	E2FF-NFKB	136 - 17	(-)	
**GXP_57221** [GXP_57221] (1 - 1187)<br>**LRRC42**, GXL_46971, GeneID: 115353, Homo sapiens chr. 1<br>leucine rich repeat containing 42	E2FF-NFKB	117 - 3	(-)	
	E2FF-NFKB	441 - 561	(+)	
**GXP_914766** [GXP_914766] (1 - 601)<br>**SCP2**, GXL_46975, GeneID: 6342, Homo sapiens chr. 1<br>sterol carrier protein 2	E2FF-NFKB	209 - 81	(-)	
**GXP_57266** [GXP_57266] (1 - 602)<br>**NSUN4**, GXL_46999, GeneID: 387338, Homo sapiens chr. 1<br>NOL1/NOP2/Sun domain family, member 4	E2FF-NFKB	252 - 365	(+)	
**GXP_635836** [GXP_635836] (1 - 601)<br>**SLC5A9**, GXL_47008, GeneID: 200010, Homo sapiens chr. 1<br>solute carrier family 5 (sodium/glucose cotransporter), member 9	E2FF-NFKB	252 - 127	(-)	
**GXP_635593** [GXP_635593] (1 - 831)<br>**CDCA8**, GXL_47019, GeneID: 55143, Homo sapiens chr. 1<br>cell division cycle associated 8	E2FF-NFKB	350 - 458	(+)	
**GXP_914542** [GXP_914542] (1 - 601)<br>**CDCA8**, GXL_47019, GeneID: 55143, Homo sapiens chr. 1<br>cell division cycle associated 8	E2FF-NFKB	220 - 328	(+)	
**GXP_914633** [GXP_914633] (1 - 601)<br>**PTPRF**, GXL_47021, GeneID: 5792, Homo sapiens chr. 1<br>protein tyrosine phosphatase, receptor type, F	E2FF-NFKB	387 - 275	(-)	
	E2FF-NFKB	513 - 636	(+)	

**GXP_57306** [GXP_57306] (1 - 847)<br>**AK3L1,AK3L2**, GXL_47028, GeneID: 205,387851, Homo sapiens chr. 1<br>adenylate kinase 3-like 1;adenylate kinase 3-like 2				
**GXP_57319** [GXP_57319] (1 - 801)<br>**B4GALT2**, GXL_47033, GeneID: 8704, Homo sapiens chr. 1<br>UDP-Gal:betaGlcNAc beta 1,4- galactosyltransferase, polypeptide 2	E2FF-NFKB	389 - 262	(-)	
**GXP_635633** [GXP_635633] (1 - 601)<br>**RLF**, GXL_47038, GeneID: 6018, Homo sapiens chr. 1<br>rearranged L-myc fusion	E2FF-NFKB	318 - 430	(+)	
**GXP_57342** [GXP_57342] (1 - 613)<br>**L1TD1**, GXL_47051, GeneID: 54596, Homo sapiens chr. 1<br>LINE-1 type transposase domain containing 1	E2FF-NFKB	560 - 434	(-)	
**GXP_635673** [GXP_635673] (1 - 601)<br>**YBX1**, GXL_47074, GeneID: 4904, Homo sapiens chr. 1<br>Y box binding protein 1	E2FF-NFKB	126 - 12	(-)	
**GXP_57388** [GXP_57388] (1 - 601)<br>**FOXD3**, GXL_47088, GeneID: 27022, Homo sapiens chr. 1<br>forkhead box D3	E2FF-NFKB	132 - 16	(-)	
**GXP_57481** [GXP_57481] (1 - 639)<br>**ASXL3**, GXL_47168, GeneID: 80816, Homo sapiens chr. 18<br>additional sex combs like 3 (Drosophila)	E2FF-NFKB	60 - 182	(+)	
**GXP_57486** [GXP_57486] (1 - 601)<br>**SERPINB10**, GXL_47171, GeneID: 5273, Homo sapiens chr. 18<br>serpin peptidase inhibitor, clade B (ovalbumin), member 10	E2FF-NFKB	185 - 305	(+)	
**GXP_57540** [GXP_57540] (1 - 601)<br>**C18orf23,RNF165**, GXL_47199, GeneID: 147341,494470, Homo sapiens chr. 18<br>chromosome 18 open reading frame 23; ring finger protein 165	E2FF-NFKB	182 - 301	(+)	
**GXP_57541** [GXP_57541] (1 - 601)<br>**C18orf23,RNF165**, GXL_47199, GeneID: 147341,494470, Homo sapiens chr. 18<br>chromosome 18 open reading frame 23; ring finger protein 165	E2FF-NFKB	133 - 16	(-)	
**GXP_57652** [GXP_57652] (1 - 644)<br>**RALBP1**, GXL_47270, GeneID: 10928, Homo sapiens chr. 18<br>ralA binding protein 1	E2FF-NFKB	276 - 147	(-)	
**GXP_58290** [GXP_58290] (1 - 1451)<br>**PIB5PA**, GXL_47700, GeneID: 27124, Homo sapiens chr. 22<br>phosphatidylinositol (4,5) bisphosphate 5-phosphatase, A	E2FF-NFKB	1005 - 885	(-)	
**GXP_917605** [GXP_917605] (1 - 601)<br>**HMG2L1**, GXL_47716, GeneID: 10042, Homo sapiens chr. 22<br>high-mobility group protein 2-like 1	E2FF-NFKB	518 - 390	(-)	
	E2FF-NFKB	330 - 218	(-)	

**GXP_58319** [GXP_58319] (1 - 721)<br>**TRIOBP**, GXL_47718, GeneID: 11078, Homo sapiens chr. 22<br>TRIO and F-actin binding protein				
**GXP_58345** [GXP_58345] (1 - 601)<br>**CBY1**, GXL_47735, GeneID: 25776, Homo sapiens chr. 22<br>chibby homolog 1 (Drosophila)	E2FF-NFKB	326 - 440	(+)	
**GXP_58359** [GXP_58359] (1 - 662)<br>**XRCC6**, GXL_47743, GeneID: 2547, Homo sapiens chr. 22<br>X-ray repair complementing defective repair in Chinese hamster cells 6 (Ku autoantigen, 70kDa)	E2FF-NFKB	242 - 130	(-)	
**GXP_58395** [GXP_58395] (1 - 927)<br>**FBXO7**, GXL_47768, GeneID: 25793, Homo sapiens chr. 22<br>F-box protein 7	E2FF-NFKB	531 - 421	(-)	
**GXP_917781** [GXP_917781] (1 - 603)<br>**TSPO**, GXL_47776, GeneID: 706, Homo sapiens chr. 22<br>translocator protein (18kDa)	E2FF-NFKB	406 - 535	(+)	
**GXP_917794** [GXP_917794] (1 - 900)<br>**PARVG**, GXL_47777, GeneID: 64098, Homo sapiens chr. 22<br>parvin, gamma	E2FF-NFKB	570 - 451	(-)	
**GXP_58413** [GXP_58413] (1 - 610)<br>**ACO2**, GXL_47782, GeneID: 50, Homo sapiens chr. 22<br>aconitase 2, mitochondrial	E2FF-NFKB	182 - 60	(-)	
**GXP_917684** [GXP_917684] (1 - 601)<br>**TOMM22**, GXL_47788, GeneID: 56993, Homo sapiens chr. 22<br>translocase of outer mitochondrial membrane 22 homolog (yeast)	E2FF-NFKB	272 - 386	(+)	
**GXP_917607** [GXP_917607] (1 - 601)<br>**HMOX1**, GXL_47792, GeneID: 3162, Homo sapiens chr. 22<br>heme oxygenase (decycling) 1	E2FF-NFKB	376 - 254	(-)	
**GXP_58485** [GXP_58485] (1 - 601)<br>**BIK**, GXL_47841, GeneID: 638, Homo sapiens chr. 22<br>BCL2-interacting killer (apoptosis-inducing)	E2FF-NFKB	125 - 239	(+)	
**GXP_925540** [GXP_925540] (1 - 601)<br>**ARHGEF5**, GXL_49412, GeneID: 7984, Homo sapiens chr. 7<br>Rho guanine nucleotide exchange factor (GEF) 5	E2FF-NFKB	149 - 272	(+)	
**GXP_60185** [GXP_60185] (1 - 694)<br>**CUL1**, GXL_49430, GeneID: 8454, Homo sapiens chr. 7<br>cullin 1	E2FF-NFKB	150 - 41	(-)	
**GXP_925554** [GXP_925554] (1 - 655)<br>**CUL1**, GXL_49430, GeneID: 8454, Homo sapiens chr. 7<br>cullin 1	E2FF-NFKB	566 - 457	(-)	
	E2FF-NFKB	390 - 281	(-)	

**GXP_925555** [GXP_925555] (1 - 665)<br>**CUL1**, GXL_49430, GeneID: 8454, Homo sapiens chr. 7<br>cullin 1				
**GXP_60209** [GXP_60209] (1 - 657)<br>**INSIG1**, GXL_49442, GeneID: 3638, Homo sapiens chr. 7<br>insulin induced gene 1	E2FF-NFKB	208 - 314	(+)	
**GXP_60247** [GXP_60247] (1 - 826)<br>**TMEM176A**, GXL_49467, GeneID: 55365, Homo sapiens chr. 7<br>transmembrane protein 176A	E2FF-NFKB	444 - 561	(+)	
**GXP_60250** [GXP_60250] (1 - 905)<br>**UBE3C**, GXL_49469, GeneID: 9690, Homo sapiens chr. 7<br>ubiquitin protein ligase E3C	E2FF-NFKB	620 - 494	(-)	
**GXP_68976** [GXP_68976] (1 - 771)<br>**ZBTB7B**, GXL_57572, GeneID: 51043, Homo sapiens chr. 1<br>zinc finger and BTB domain containing 7B	E2FF-NFKB	544 - 671	(+)	
**GXP_637136** [GXP_637136] (1 - 601)<br>**KIRREL**, GXL_57594, GeneID: 55243, Homo sapiens chr. 1<br>kin of IRRE like (Drosophila)	E2FF-NFKB	177 - 303	(+)	
**GXP_915449** [GXP_915449] (1 - 601)<br>**TARS2**, GXL_57597, GeneID: 80222, Homo sapiens chr. 1<br>threonyl-tRNA synthetase 2, mitochondrial (putative)	E2FF-NFKB	485 - 593	(+)	
**GXP_637295** [GXP_637295] (1 - 601)<br>**DUSP27**, GXL_57613, GeneID: 92235, Homo sapiens chr. 1<br>dual specificity phosphatase 27 (putative)	E2FF-NFKB	478 - 371	(-)	
**GXP_69059** [GXP_69059] (1 - 665)<br>**PSMD4**, GXL_57620, GeneID: 5710, Homo sapiens chr. 1<br>proteasome (prosome, macropain) 26S subunit, non-ATPase, 4	E2FF-NFKB	427 - 306	(-)	
**GXP_69159** [GXP_69159] (1 - 1151)<br>**MTX1**, GXL_57686, GeneID: 4580, Homo sapiens chr. 1<br>metaxin 1	E2FF-NFKB	227 - 119	(-)	
**GXP_69173** [GXP_69173] (1 - 691)<br>**EFNA3**, GXL_57696, GeneID: 1944, Homo sapiens chr. 1<br>ephrin-A3	E2FF-NFKB	501 - 381	(-)	
**GXP_69175** [GXP_69175] (1 - 1332)<br>**CA14**, GXL_57698, GeneID: 23632, Homo sapiens chr. 1<br>carbonic anhydrase XIV	E2FF-NFKB	1048 - 936	(-)	
**GXP_69180** [GXP_69180] (1 - 668)<br>**RAG1AP1**, GXL_57702, GeneID: 55974, Homo sapiens chr. 1<br>recombination activating gene 1 activating protein 1	E2FF-NFKB	458 - 342	(-)	
	E2FF-NFKB	62 - 191	(+)	

**GXP_69201** [GXP_69201] (1 - 1225)<br>**MRPS21**, GXL_57718, GeneID: 54460, Homo sapiens chr. 1<br>mitochondrial ribosomal protein S21				
**GXP_69248** [GXP_69248] (1 - 1111)<br>**ATP1B1**, GXL_57755, GeneID: 481, Homo sapiens chr. 1<br>ATPase, Na+/K+ transporting, beta 1 polypeptide	E2FF-NFKB	319 - 197	(-)	
**GXP_69251** [GXP_69251] (1 - 601)<br>**ATP8B2**, GXL_57758, GeneID: 57198, Homo sapiens chr. 1<br>ATPase, Class I, type 8B, member 2	E2FF-NFKB	349 - 224	(-)	
**GXP_69256** [GXP_69256] (1 - 1306)<br>**CREB3L4**, GXL_57761, GeneID: 148327, Homo sapiens chr. 1<br>cAMP responsive element binding protein 3-like 4	E2FF-NFKB	344 - 218	(-)	
**GXP_69376** [GXP_69376] (1 - 824)<br>**GYPC**, GXL_57862, GeneID: 2995, Homo sapiens chr. 2<br>glycophorin C (Gerbich blood group)	E2FF-NFKB	656 - 769	(+)	
**GXP_651035** [GXP_651035] (1 - 833)<br>**DNAJC10**, GXL_57864, GeneID: 54431, Homo sapiens chr. 2<br>DnaJ (Hsp40) homolog, subfamily C, member 10	E2FF-NFKB	134 - 262	(+)	
**GXP_650818** [GXP_650818] (1 - 601)<br>**TANK**, GXL_57870, GeneID: 10010, Homo sapiens chr. 2<br>TRAF family member-associated NFKB activator	E2FF-NFKB	5 - 112	(+)	
**GXP_918983** [GXP_918983] (1 - 745)<br>**TANK**, GXL_57870, GeneID: 10010, Homo sapiens chr. 2<br>TRAF family member-associated NFKB activator	E2FF-NFKB	434 - 541	(+)	
**GXP_69395** [GXP_69395] (1 - 601)<br>**LYPD6**, GXL_57873, GeneID: 130574, Homo sapiens chr. 2<br>LY6/PLAUR domain containing 6	E2FF-NFKB	57 - 170	(+)	
**GXP_69415** [GXP_69415] (1 - 602)<br>**ZAK**, GXL_57882, GeneID: 51776, Homo sapiens chr. 2<br>sterile alpha motif and leucine zipper containing kinase AZK	E2FF-NFKB	526 - 398	(-)	
**GXP_69424** [GXP_69424] (1 - 684)<br>**SSB**, GXL_57886, GeneID: 6741, Homo sapiens chr. 2<br>Sjogren syndrome antigen B (autoantigen La)	E2FF-NFKB	435 - 559	(+)	
**GXP_918762** [GXP_918762] (1 - 723)<br>**DPP10**, GXL_57887, GeneID: 57628, Homo sapiens chr. 2<br>dipeptidyl-peptidase 10	E2FF-NFKB	17 - 137	(+)	
**GXP_919044** [GXP_919044] (1 - 778)<br>**KLHL23,PHOSPHO2**, GXL_57903, GeneID: 151230,493911, Homo sapiens chr. 2<br>kelch-like 23 (Drosophila);phosphatase, orphan 2	E2FF-NFKB	88 - 203	(+)	
	E2FF-NFKB	859 - 752	(-)	

**GXP_650899** [GXP_650899] (1 - 1006)<br>**GAD1**, GXL_57933, GeneID: 2571, Homo sapiens chr. 2<br>glutamate decarboxylase 1 (brain, 67kDa)				
**GXP_919047** [GXP_919047] (1 - 601)<br>**MYO3B**, GXL_57940, GeneID: 140469, Homo sapiens chr. 2<br>myosin IIIB	E2FF-NFKB	477 - 355	(-)	
**GXP_919048** [GXP_919048] (1 - 601)<br>**MYO3B**, GXL_57940, GeneID: 140469, Homo sapiens chr. 2<br>myosin IIIB	E2FF-NFKB	308 - 186	(-)	
**GXP_650939** [GXP_650939] (1 - 601)<br>**CDCA7**, GXL_57946, GeneID: 83879, Homo sapiens chr. 2<br>cell division cycle associated 7	E2FF-NFKB	432 - 320	(-)	
**GXP_69549** [GXP_69549] (1 - 601)<br>**MYO1B**, GXL_57949, GeneID: 4430, Homo sapiens chr. 2<br>myosin IB	E2FF-NFKB	288 - 415	(+)	
**GXP_919039** [GXP_919039] (1 - 856)<br>**PPIG**, GXL_58070, GeneID: 9360, Homo sapiens chr. 2<br>peptidylprolyl isomerase G (cyclophilin G)	E2FF-NFKB	480 - 592	(+)	
**GXP_69724** [GXP_69724] (1 - 903)<br>**INPP1**, GXL_58083, GeneID: 3628, Homo sapiens chr. 2<br>inositol polyphosphate-1-phosphatase	E2FF-NFKB<br>E2FF-NFKB	114 - 241<br>632 - 525	(+)<br>(-)	
**GXP_69748** [GXP_69748] (1 - 668)<br>**PMS1**, GXL_58106, GeneID: 5378, Homo sapiens chr. 2<br>PMS1 postmeiotic segregation increased 1 (S. cerevisiae)	E2FF-NFKB	184 - 62	(-)	
**GXP_918933** [GXP_918933] (1 - 604)<br>**KIF5C**, GXL_58109, GeneID: 3800, Homo sapiens chr. 2<br>kinesin family member 5C	E2FF-NFKB	502 - 387	(-)	
**GXP_70929** [GXP_70929] (1 - 658)<br>**TMEM51**, GXL_58949, GeneID: 55092, Homo sapiens chr. 1<br>transmembrane protein 51	E2FF-NFKB	251 - 132	(-)	
**GXP_914006** [GXP_914006] (1 - 601)<br>**PER3**, GXL_58952, GeneID: 8863, Homo sapiens chr. 1<br>period homolog 3 (Drosophila)	E2FF-NFKB	340 - 225	(-)	
**GXP_914392** [GXP_914392] (1 - 601)<br>**RCC1,SNORA73A,SNHG3,SNHG3-RCC1**, GXL_58953, GeneID: 1104,6080,8420,751867, Homo sapiens chr. 1<br>regulator of chromosome condensation 1; small nucleolar RNA, H/ACA box 73A; small nucleolar RNA host gene (non-protein coding) 3; regulator of chromosome condensation 1	E2FF-NFKB	425 - 316	(-)	
**GXP_70941** [GXP_70941] (1 - 601)<br>**SESN2**, GXL_58955, GeneID: 83667, Homo sapiens chr. 1<br>sestrin 2	E2FF-NFKB	114 - 228	(+)	

**GXP_70947** [GXP_70947] (1 - 604)<br>**RPL11**, GXL_58957, GeneID: 6135, Homo sapiens chr. 1<br>ribosomal protein L11	E2FF-NFKB	165 - 282	(+)	
**GXP_70990** [GXP_70990] (1 - 601)<br>**VPS13D**, GXL_58980, GeneID: 55187, Homo sapiens chr. 1<br>vacuolar protein sorting 13 homolog D (S. cerevisiae)	E2FF-NFKB	216 - 322	(+)	
**GXP_71031** [GXP_71031] (1 - 601)<br>**EPB41**, GXL_59001, GeneID: 2035, Homo sapiens chr. 1<br>erythrocyte membrane protein band 4.1 (elliptocytosis 1, RH-linked)	E2FF-NFKB	595 - 472	(-)	
**GXP_71049** [GXP_71049] (1 - 1170)<br>**PDPN**, GXL_59014, GeneID: 10630, Homo sapiens chr. 1<br>podoplanin	E2FF-NFKB	344 - 459	(+)	
**GXP_71054** [GXP_71054] (1 - 1036)<br>**SH3BGRL3**, GXL_59017, GeneID: 83442, Homo sapiens chr. 1<br>SH3 domain binding glutamic acid-rich protein like 3	E2FF-NFKB	531 - 654	(+)	
**GXP_71068** [GXP_71068] (1 - 629)<br>**H6PD**, GXL_59027, GeneID: 9563, Homo sapiens chr. 1<br>hexose-6-phosphate dehydrogenase (glucose 1-dehydrogenase)	E2FF-NFKB	281 - 175	(-)	
**GXP_914032** [GXP_914032] (1 - 601)<br>**H6PD**, GXL_59027, GeneID: 9563, Homo sapiens chr. 1<br>hexose-6-phosphate dehydrogenase (glucose 1-dehydrogenase)	E2FF-NFKB	206 - 88	(-)	
**GXP_71071** [GXP_71071] (1 - 1132)<br>**XKR8**, GXL_59028, GeneID: 55113, Homo sapiens chr. 1<br>XK, Kell blood group complex subunit-related family, member 8	E2FF-NFKB	338 - 460	(+)	
**GXP_71084** [GXP_71084] (1 - 823)<br>**WDTC1**, GXL_59034, GeneID: 23038, Homo sapiens chr. 1<br>WD and tetratricopeptide repeats 1	E2FF-NFKB	350 - 473	(+)	
**GXP_71095** [GXP_71095] (1 - 1330)<br>**ZCCHC17**, GXL_59042, GeneID: 51538, Homo sapiens chr. 1<br>zinc finger, CCHC domain containing 17	E2FF-NFKB	855 - 971	(+)	
**GXP_71114** [GXP_71114] (1 - 685)<br>**ATPIF1**, GXL_59049, GeneID: 93974, Homo sapiens chr. 1<br>ATPase inhibitory factor 1	E2FF-NFKB	540 - 433	(-)	
**GXP_71117** [GXP_71117] (1 - 1099)<br>**YTHDF2**, GXL_59051, GeneID: 51441, Homo sapiens chr. 1<br>YTH domain family, member 2	E2FF-NFKB	200 - 316	(+)	
**GXP_914145** [GXP_914145] (1 - 601)<br>**DNAJC16**, GXL_59060, GeneID: 23341, Homo sapiens chr. 1<br>DnaJ (Hsp40) homolog, subfamily C, member 16	E2FF-NFKB	139 - 26	(-)	
	E2FF-NFKB	428 - 320	(-)	

**GXP_71142** [GXP_71142] (1 - 673)<br>**NPAL3**, GXL_59067, GeneID: 57185, Homo sapiens chr. 1<br>NIPA-like domain containing 3				
**GXP_71146** [GXP_71146] (1 - 756)<br>**STX12**, GXL_59069, GeneID: 23673, Homo sapiens chr. 1<br>syntaxin 12	E2FF-NFKB	512 - 405	(-)	
**GXP_71148** [GXP_71148] (1 - 650)<br>**EFHD2**, GXL_59070, GeneID: 79180, Homo sapiens chr. 1<br>EF-hand domain family, member D2	E2FF-NFKB	247 - 132	(-)	
**GXP_71177** [GXP_71177] (1 - 844)<br>**CAMTA1**, GXL_59091, GeneID: 23261, Homo sapiens chr. 1<br>calmodulin binding transcription activator 1	E2FF-NFKB	160 - 269	(+)	
	E2FF-NFKB	361 - 255	(-)	
**GXP_71185** [GXP_71185] (1 - 1087)<br>**EPHA8**, GXL_59095, GeneID: 2046, Homo sapiens chr. 1<br>EPH receptor A8	E2FF-NFKB	658 - 544	(-)	
**GXP_71198** [GXP_71198] (1 - 939)<br>**EPHB2**, GXL_59105, GeneID: 2048, Homo sapiens chr. 1<br>EPH receptor B2	E2FF-NFKB	452 - 346	(-)	
**GXP_71199** [GXP_71199] (1 - 1267)<br>**TSSK3**, GXL_59106, GeneID: 81629, Homo sapiens chr. 1<br>testis-specific serine kinase 3	E2FF-NFKB	307 - 427	(+)	
	E2FF-NFKB	534 - 413	(-)	
**GXP_71206** [GXP_71206] (1 - 689)<br>**SERINC2**, GXL_59113, GeneID: 347735, Homo sapiens chr. 1<br>serine incorporator 2	E2FF-NFKB	613 - 489	(-)	
**GXP_71236** [GXP_71236] (1 - 844)<br>**OPRD1**, GXL_59132, GeneID: 4985, Homo sapiens chr. 1<br>opioid receptor, delta 1	E2FF-NFKB	268 - 381	(+)	
**GXP_914081** [GXP_914081] (1 - 601)<br>**RP5-1077B9.4**, GXL_59134, GeneID: 60672, Homo sapiens chr. 1<br>invasion inhibitory protein 45	E2FF-NFKB	243 - 353	(+)	
**GXP_914141** [GXP_914141] (1 - 601)<br>**ELA2A**, GXL_59175, GeneID: 63036, Homo sapiens chr. 1<br>elastase 2A	E2FF-NFKB	468 - 591	(+)	
**GXP_71704** [GXP_71704] (1 - 1013)<br>**AQP5**, GXL_59530, GeneID: 362, Homo sapiens chr. 12<br>aquaporin 5	E2FF-NFKB	657 - 765	(+)	
**GXP_71706** [GXP_71706] (1 - 601)<br>**AEBP2**, GXL_59531, GeneID: 121536, Homo sapiens chr. 12<br>AE binding protein 2	E2FF-NFKB	381 - 254	(-)	
	E2FF-NFKB	201 - 83	(-)	

**GXP_71826** [GXP_71826] (1 - 601)<br>**APOLD1**, GXL_59610, GeneID: 81575, Homo sapiens chr. 12<br>apolipoprotein L domain containing 1				
**GXP_641091** [GXP_641091] (1 - 601)<br>**APOLD1**, GXL_59610, GeneID: 81575, Homo sapiens chr. 12<br>apolipoprotein L domain containing 1	E2FF-NFKB	199 - 81	(-)	
**GXP_71828** [GXP_71828] (1 - 1435)<br>**KLHDC5**, GXL_59611, GeneID: 57542, Homo sapiens chr. 12<br>kelch domain containing 5	E2FF-NFKB	813 - 926	(+)	
**GXP_71874** [GXP_71874] (1 - 719)<br>**CD9**, GXL_59643, GeneID: 928, Homo sapiens chr. 12<br>CD9 molecule	E2FF-NFKB	623 - 495	(-)	
**GXP_71946** [GXP_71946] (1 - 659)<br>**SURB7**, GXL_59706, GeneID: 9412, Homo sapiens chr. 12<br>SRB7 suppressor of RNA polymerase B homolog (yeast)	E2FF-NFKB	542 - 434	(-)	
**GXP_71949** [GXP_71949] (1 - 752)<br>**EMG1**, GXL_59709, GeneID: 10436, Homo sapiens chr. 12<br>EMG1 nucleolar protein homolog (S. cerevisiae)	E2FF-NFKB	367 - 489	(+)	
**GXP_79394** [GXP_79394] (1 - 722)<br>**CAMKK2**, GXL_66082, GeneID: 10645, Homo sapiens chr. 12<br>calcium/calmodulin-dependent protein kinase kinase 2, beta	E2FF-NFKB	222 - 95	(-)	
**GXP_642201** [GXP_642201] (1 - 601)<br>**CAMKK2**, GXL_66082, GeneID: 10645, Homo sapiens chr. 12<br>calcium/calmodulin-dependent protein kinase kinase 2, beta	E2FF-NFKB	386 - 494	(+)	
**GXP_79398** [GXP_79398] (1 - 648)<br>**DIABLO**, GXL_66085, GeneID: 56616, Homo sapiens chr. 12<br>diablo homolog (Drosophila)	E2FF-NFKB	224 - 346	(+)	
**GXP_79399** [GXP_79399] (1 - 601)<br>**DIABLO**, GXL_66085, GeneID: 56616, Homo sapiens chr. 12<br>diablo homolog (Drosophila)	E2FF-NFKB	14 - 136	(+)	
**GXP_641947** [GXP_641947] (1 - 601)<br>**SLC41A2**, GXL_66086, GeneID: 84102, Homo sapiens chr. 12<br>solute carrier family 41, member 2	E2FF-NFKB	134 - 22	(-)	
**GXP_642368** [GXP_642368] (1 - 601)<br>**GALNT9,LOC642141,LOC729185**, GXL_66099, GeneID: 50614,642141,729185, Homo sapiens chr. 12<br>UDP-N-acetyl-alpha-D-galactosamine:polypeptide N-acetylgalactosaminyltransferase 9 (GalNAc-T9); similar to Polypeptide N-acetylgalactosaminyltransferase 9 (Protein-UDP acetylgalactosaminyltransferase 9) (UDP-GalNAc:polypeptide N-acetylgalactosaminyltransferase 9) (Polypeptide GalNAc transferase 9) (GalNAc-T9) (pp-GaNTase 9); similar to Polypeptide N-acetylgalactosaminyltransferase 9 (Protein-UDP acetylgalactosaminyltransferase 9) (UDP-GalNAc:polypeptide N-acetylgalactosaminyltransferase 9) (Polypeptide GalNAc transferase 9) (GalNAc-T9) (pp-GaNTase 9)	E2FF-NFKB	383 - 263	(-)	

**GXP_79436** [GXP_79436] (1 - 601)<br>**CLIP1**, GXL_66107, GeneID: 6249, Homo sapiens chr. 12<br>CAP-GLY domain containing linker protein 1	E2FF-NFKB	41 - 166	(+)	
**GXP_907482** [GXP_907482] (1 - 601)<br>**CLIP1**, GXL_66107, GeneID: 6249, Homo sapiens chr. 12<br>CAP-GLY domain containing linker protein 1	E2FF-NFKB	488 - 366	(-)	
**GXP_79504** [GXP_79504] (1 - 872)<br>**TBX5**, GXL_66144, GeneID: 6910, Homo sapiens chr. 12<br>T-box 5	E2FF-NFKB	113 - 224	(+)	
**GXP_79530** [GXP_79530] (1 - 601)<br>**HPD**, GXL_66158, GeneID: 3242, Homo sapiens chr. 12<br>4-hydroxyphenylpyruvate dioxygenase	E2FF-NFKB	144 - 26	(-)	
**GXP_79534** [GXP_79534] (1 - 635)<br>**MSI1**, GXL_66160, GeneID: 4440, Homo sapiens chr. 12<br>musashi homolog 1 (Drosophila)	E2FF-NFKB	241 - 118	(-)	
**GXP_907298** [GXP_907298] (1 - 601)<br>**SSH1**, GXL_66189, GeneID: 54434, Homo sapiens chr. 12<br>slingshot homolog 1 (Drosophila)	E2FF-NFKB	201 - 314	(+)	
**GXP_79630** [GXP_79630] (1 - 601)<br>**RAB35**, GXL_66213, GeneID: 11021, Homo sapiens chr. 12<br>RAB35, member RAS oncogene family	E2FF-NFKB	259 - 153	(-)	
**GXP_79678** [GXP_79678] (1 - 601)<br>**FLJ45278**, GXL_66252, GeneID: 642797, Homo sapiens chr. 12<br>hypothetical protein LOC642797	E2FF-NFKB	392 - 519	(+)	
**GXP_79685** [GXP_79685] (1 - 602)<br>**GLT8D2**, GXL_66257, GeneID: 83468, Homo sapiens chr. 12<br>glycosyltransferase 8 domain containing 2	E2FF-NFKB	187 - 311	(+)	
**GXP_907379** [GXP_907379] (1 - 601)<br>**LHX5**, GXL_66285, GeneID: 64211, Homo sapiens chr. 12<br>LIM homeobox 5	E2FF-NFKB	519 - 403	(-)	
**GXP_80728** [GXP_80728] (1 - 803)<br>**EBAG9**, GXL_67198, GeneID: 9166, Homo sapiens chr. 8<br>estrogen receptor binding site associated, antigen, 9	E2FF-NFKB	564 - 457	(-)	
**GXP_80760** [GXP_80760] (1 - 760)<br>**CHD7**, GXL_67218, GeneID: 55636, Homo sapiens chr. 8<br>chromodomain helicase DNA binding protein 7	E2FF-NFKB	361 - 242	(-)	
**GXP_80797** [GXP_80797] (1 - 618)<br>**FAM92A1**, GXL_67242, GeneID: 137392, Homo sapiens chr. 8<br>family with sequence similarity 92, member A1	E2FF-NFKB	370 - 494	(+)	
	E2FF-NFKB	93 - 211	(+)	

**GXP_926098** [GXP_926098] (1 - 601)<br>**SULF1**, GXL_67246, GeneID: 23213, Homo sapiens chr. 8<br>sulfatase 1				
**GXP_80810** [GXP_80810] (1 - 991)<br>**LY6K**, GXL_67247, GeneID: 54742, Homo sapiens chr. 8<br>lymphocyte antigen 6 complex, locus K	E2FF-NFKB	717 - 845	(+)	
**GXP_492916** [GXP_492916] (1 - 601)<br>**SGK3**, GXL_67262, GeneID: 23678, Homo sapiens chr. 8<br>serum/glucocorticoid regulated kinase family, member 3	E2FF-NFKB	241 - 119	(-)	
**GXP_80834** [GXP_80834] (1 - 1471)<br>**TRIB1**, GXL_67263, GeneID: 10221, Homo sapiens chr. 8<br>tribbles homolog 1 (Drosophila)	E2FF-NFKB	437 - 556	(+)	
**GXP_80877** [GXP_80877] (1 - 1109)<br>**C8orf34**, GXL_67290, GeneID: 116328, Homo sapiens chr. 8<br>chromosome 8 open reading frame 34	E2FF-NFKB	246 - 374	(+)	
**GXP_80901** [GXP_80901] (1 - 868)<br>**CRISPLD1**, GXL_67306, GeneID: 83690, Homo sapiens chr. 8<br>cysteine-rich secretory protein LCCL domain containing 1	E2FF-NFKB	49 - 175	(+)	
**GXP_80920** [GXP_80920] (1 - 695)<br>**C8orf32**, GXL_67320, GeneID: 55093, Homo sapiens chr. 8<br>chromosome 8 open reading frame 32	E2FF-NFKB	308 - 422	(+)	
**GXP_80950** [GXP_80950] (1 - 944)<br>**SOX17**, GXL_67343, GeneID: 64321, Homo sapiens chr. 8<br>SRY (sex determining region Y)-box 17	E2FF-NFKB	846 - 721	(-)	
**GXP_80958** [GXP_80958] (1 - 1006)<br>**KCNS2**, GXL_67351, GeneID: 3788, Homo sapiens chr. 8<br>potassium voltage-gated channel, delayed-rectifier, subfamily S, member 2	E2FF-NFKB	307 - 179	(-)	
**GXP_81019** [GXP_81019] (1 - 887)<br>**CHMP4C**, GXL_67408, GeneID: 92421, Homo sapiens chr. 8<br>chromatin modifying protein 4C	E2FF-NFKB	410 - 530	(+)	
**GXP_661053** [GXP_661053] (1 - 601)<br>**MRPL15**, GXL_67416, GeneID: 29088, Homo sapiens chr. 8<br>mitochondrial ribosomal protein L15	E2FF-NFKB	138 - 260	(+)	
**GXP_85054** [GXP_85054] (1 - 601)<br>**ZFP161**, GXL_70830, GeneID: 7541, Homo sapiens chr. 18<br>zinc finger protein 161 homolog (mouse)	E2FF-NFKB	111 - 231	(+)	
**GXP_85060** [GXP_85060] (1 - 835)<br>**PQLC1,LOC729311,LOC731341**, GXL_70833, GeneID: 80148,729311,731341, Homo sapiens chr. 18<br>PQ loop repeat containing 1; hypothetical protein LOC729311; hypothetical protein LOC731341	E2FF-NFKB	482 - 353	(-)	
	E2FF-NFKB	808 - 685	(-)	

**GXP_85070** [GXP_85070] (1 - 925)<br>**MRCL3**, GXL_70836, GeneID: 10627, Homo sapiens chr. 18<br>myosin regulatory light chain MRCL3				
**GXP_85150** [GXP_85150] (1 - 825)<br>**NOL4**, GXL_70875, GeneID: 8715, Homo sapiens chr. 18<br>nucleolar protein 4	E2FF-NFKB	429 - 320	(-)	
**GXP_85153** [GXP_85153] (1 - 742)<br>**CYB5A**, GXL_70877, GeneID: 1528, Homo sapiens chr. 18<br>cytochrome b5 type A (microsomal)	E2FF-NFKB	441 - 563	(+)	
**GXP_85157** [GXP_85157] (1 - 767)<br>**PTPN2**, GXL_70880, GeneID: 5771, Homo sapiens chr. 18<br>protein tyrosine phosphatase, non-receptor type 2	E2FF-NFKB	394 - 278	(-)	
**GXP_912316** [GXP_912316] (1 - 601)<br>**DSC2**, GXL_70883, GeneID: 1824, Homo sapiens chr. 18<br>desmocollin 2	E2FF-NFKB	155 - 41	(-)	
**GXP_912459** [GXP_912459] (1 - 1093)<br>**TCF4**, GXL_70886, GeneID: 6925, Homo sapiens chr. 18<br>transcription factor 4	E2FF-NFKB	886 - 775	(-)	
**GXP_85236** [GXP_85236] (1 - 612)<br>**L3MBTL4**, GXL_70929, GeneID: 91133, Homo sapiens chr. 18<br>l(3)mbt-like 4 (Drosophila)	E2FF-NFKB	470 - 346	(-)	
**GXP_912159** [GXP_912159] (1 - 601)<br>**L3MBTL4**, GXL_70929, GeneID: 91133, Homo sapiens chr. 18<br>l(3)mbt-like 4 (Drosophila)	E2FF-NFKB	354 - 230	(-)	
**GXP_647736** [GXP_647736] (1 - 601)<br>**FBXO15**, GXL_70950, GeneID: 201456, Homo sapiens chr. 18<br>F-box protein 15	E2FF-NFKB	474 - 361	(-)	
**GXP_85309** [GXP_85309] (1 - 1263)<br>**ST8SIA5**, GXL_70981, GeneID: 29906, Homo sapiens chr. 18<br>ST8 alpha-N-acetyl-neuraminide alpha-2, 8-sialyltransferase 5	E2FF-NFKB	1017 - 911	(-)	
**GXP_85321** [GXP_85321] (1 - 602)<br>**FLJ45994**, GXL_70986, GeneID: 400645, Homo sapiens chr. 18<br>hypothetical gene supported by AK127888	E2FF-NFKB	127 - 234	(+)	
**GXP_85376** [GXP_85376] (1 - 848)<br>**ESCO1**, GXL_71032, GeneID: 114799, Homo sapiens chr. 18<br>establishment of cohesion 1 homolog 1 (S. cerevisiae)	E2FF-NFKB	580 - 703	(+)	
**GXP_85421** [GXP_85421] (1 - 601)<br>**FLJ44087**, GXL_71068, GeneID: 400649, Homo sapiens chr. 18<br>hypothetical gene supported by AK126075	E2FF-NFKB	133 - 23	(-)	
	E2FF-NFKB	559 - 432	(-)	

**GXP_85449** [GXP_85449] (1 - 602)<br>**hCG_38480,LOC730568**, GXL_71093, GeneID: 728606,730568, Homo sapiens chr. 18<br>potassium channel tetramerisation domain containing 1; hypothetical protein LOC730568				
**GXP_918377** [GXP_918377] (1 - 601)<br>**DUSP11**, GXL_72327, GeneID: 8446, Homo sapiens chr. 2<br>dual specificity phosphatase 11 (RNA/RNP complex 1-interacting)	E2FF-NFKB	126 - 249	(+)	
**GXP_918413** [GXP_918413] (1 - 1019)<br>**TMEM166**, GXL_72359, GeneID: 84141, Homo sapiens chr. 2<br>transmembrane protein 166	E2FF-NFKB	723 - 598	(-)	
**GXP_86815** [GXP_86815] (1 - 733)<br>**SEMA4C**, GXL_72361, GeneID: 54910, Homo sapiens chr. 2<br>sema domain, immunoglobulin domain (Ig), transmembrane domain (TM) and short cytoplasmic domain, (semaphorin) 4C	E2FF-NFKB	66 - 175	(+)	
**GXP_86879** [GXP_86879] (1 - 663)<br>**FLJ10081**, GXL_72398, GeneID: 55683, Homo sapiens chr. 2<br>hypothetical protein FLJ10081	E2FF-NFKB	465 - 348	(-)	
**GXP_86917** [GXP_86917] (1 - 1352)<br>**TMEM150**, GXL_72415, GeneID: 129303, Homo sapiens chr. 2<br>transmembrane protein 150	E2FF-NFKB	755 - 639	(-)	
**GXP_918370** [GXP_918370] (1 - 861)<br>**FBXO41**, GXL_72420, GeneID: 150726, Homo sapiens chr. 2<br>F-box protein 41	E2FF-NFKB	571 - 696	(+)	
**GXP_86956** [GXP_86956] (1 - 824)<br>**SUCLG1**, GXL_72436, GeneID: 8802, Homo sapiens chr. 2<br>succinate-CoA ligase, GDP-forming, alpha subunit	E2FF-NFKB<br>E2FF-NFKB	578 - 451<br>785 - 656	(-)<br>(-)	
**GXP_88403** [GXP_88403] (1 - 663)<br>**HMHA1**, GXL_73753, GeneID: 23526, Homo sapiens chr. 19<br>histocompatibility (minor) HA-1	E2FF-NFKB	558 - 443	(-)	
**GXP_88412** [GXP_88412] (1 - 749)<br>**EVI5L**, GXL_73760, GeneID: 115704, Homo sapiens chr. 19<br>ecotropic viral integration site 5-like	E2FF-NFKB	296 - 407	(+)	
**GXP_88415** [GXP_88415] (1 - 601)<br>**PTBP1**, GXL_73762, GeneID: 5725, Homo sapiens chr. 19<br>polypyrimidine tract binding protein 1	E2FF-NFKB	220 - 337	(+)	
**GXP_88432** [GXP_88432] (1 - 698)<br>**A1BG,ZNF497**, GXL_73774, GeneID: 1,162968, Homo sapiens chr. 19<br>alpha-1-B glycoprotein;zinc finger protein 497	E2FF-NFKB	235 - 351	(+)	
**GXP_88457** [GXP_88457] (1 - 601)<br>**LENG9**, GXL_73787, GeneID: 94059, Homo sapiens chr. 19<br>leukocyte receptor cluster (LRC) member 9	E2FF-NFKB	371 - 488	(+)	
	E2FF-NFKB	305 - 417	(+)	

**GXP_88475** [GXP_88475] (1 - 692)<br>**TMEM146**, GXL_73801, GeneID: 257062, Homo sapiens chr. 19<br>transmembrane protein 146				
**GXP_88482** [GXP_88482] (1 - 605)<br>**AZU1**, GXL_73808, GeneID: 566, Homo sapiens chr. 19<br>azurocidin 1 (cationic antimicrobial protein 37)	E2FF-NFKB	524 - 401	(-)	
**GXP_88497** [GXP_88497] (1 - 942)<br>**SF3A2**, GXL_73818, GeneID: 8175, Homo sapiens chr. 19<br>splicing factor 3a, subunit 2, 66kDa	E2FF-NFKB	815 - 934	(+)	
**GXP_88506** [GXP_88506] (1 - 728)<br>**HCN2**, GXL_73824, GeneID: 610, Homo sapiens chr. 19<br>hyperpolarization activated cyclic nucleotide-gated potassium channel 2	E2FF-NFKB	709 - 583	(-)	
**GXP_647783** [GXP_647783] (1 - 601)<br>**MADCAM1**, GXL_73834, GeneID: 8174, Homo sapiens chr. 19<br>mucosal vascular addressin cell adhesion molecule 1	E2FF-NFKB	290 - 167	(-)	
**GXP_88544** [GXP_88544] (1 - 601)<br>**SHD**, GXL_73853, GeneID: 56961, Homo sapiens chr. 19<br>Src homology 2 domain containing transforming protein D	E2FF-NFKB	189 - 311	(+)	
**GXP_88555** [GXP_88555] (1 - 726)<br>**MUM1**, GXL_73863, GeneID: 84939, Homo sapiens chr. 19<br>melanoma associated antigen (mutated) 1	E2FF-NFKB	208 - 91	(-)	
**GXP_912696** [GXP_912696] (1 - 601)<br>**ITGB1BP3**, GXL_73868, GeneID: 27231, Homo sapiens chr. 19<br>integrin beta 1 binding protein 3	E2FF-NFKB	280 - 389	(+)	
**GXP_88602** [GXP_88602] (1 - 885)<br>**VMAC**, GXL_73893, GeneID: 400673, Homo sapiens chr. 19<br>vimentin-type IF-associated coiled-coil protein	E2FF-NFKB	369 - 251	(-)	
**GXP_88672** [GXP_88672] (1 - 719)<br>**CHAF1A**, GXL_73949, GeneID: 10036, Homo sapiens chr. 19<br>chromatin assembly factor 1, subunit A (p150)	E2FF-NFKB	600 - 494	(-)	
**GXP_90062** [GXP_90062] (1 - 736)<br>**TMEM112B**, GXL_75058, GeneID: 91289, Homo sapiens chr. 22<br>transmembrane protein 112B	E2FF-NFKB	546 - 655	(+)	
**GXP_653376** [GXP_653376] (1 - 601)<br>**PLXNB2**, GXL_75087, GeneID: 23654, Homo sapiens chr. 22<br>plexin B2	E2FF-NFKB	314 - 187	(-)	
**GXP_917848** [GXP_917848] (1 - 601)<br>**PLXNB2**, GXL_75087, GeneID: 23654, Homo sapiens chr. 22<br>plexin B2	E2FF-NFKB	23 - 144	(+)	
	E2FF-NFKB	273 - 394	(+)	

**GXP_917468** [GXP_917468] (1 - 621)<br>**ADORA2A**, GXL_75130, GeneID: 135, Homo sapiens chr. 22<br>adenosine A2a receptor				
**GXP_90205** [GXP_90205] (1 - 601)<br>**PHF21B**, GXL_75135, GeneID: 112885, Homo sapiens chr. 22<br>PHD finger protein 21B	E2FF-NFKB	476 - 590	(+)	
**GXP_90206** [GXP_90206] (1 - 601)<br>**PHF21B**, GXL_75135, GeneID: 112885, Homo sapiens chr. 22<br>PHD finger protein 21B	E2FF-NFKB	248 - 362	(+)	
**GXP_90224** [GXP_90224] (1 - 601)<br>**C22orf27**, GXL_75144, GeneID: 150291, Homo sapiens chr. 22<br>chromosome 22 open reading frame 27	E2FF-NFKB	146 - 264	(+)	
**GXP_90227** [GXP_90227] (1 - 993)<br>**SNRPD3**, GXL_75147, GeneID: 6634, Homo sapiens chr. 22<br>small nuclear ribonucleoprotein D3 polypeptide 18kDa	E2FF-NFKB	221 - 347	(+)	
**GXP_90231** [GXP_90231] (1 - 601)<br>**MAPK12**, GXL_75150, GeneID: 6300, Homo sapiens chr. 22<br>mitogen-activated protein kinase 12	E2FF-NFKB<br>E2FF-NFKB	369 - 489<br>583 - 476	(+)<br>(-)	
**GXP_90732** [GXP_90732] (1 - 601)<br>**ITSN2**, GXL_75589, GeneID: 50618, Homo sapiens chr. 2<br>intersectin 2	E2FF-NFKB	165 - 280	(+)	
**GXP_649531** [GXP_649531] (1 - 601)<br>**OTOF**, GXL_75605, GeneID: 9381, Homo sapiens chr. 2<br>otoferlin	E2FF-NFKB	566 - 460	(-)	
**GXP_649654** [GXP_649654] (1 - 601)<br>**HEATR5B**, GXL_75606, GeneID: 54497, Homo sapiens chr. 2<br>HEAT repeat containing 5B	E2FF-NFKB	461 - 582	(+)	
**GXP_90795** [GXP_90795] (1 - 601)<br>**ROCK2**, GXL_75618, GeneID: 9475, Homo sapiens chr. 2<br>Rho-associated, coiled-coil containing protein kinase 2	E2FF-NFKB	50 - 177	(+)	
**GXP_90892** [GXP_90892] (1 - 601)<br>**GPR113**, GXL_75669, GeneID: 165082, Homo sapiens chr. 2<br>G protein-coupled receptor 113	E2FF-NFKB	375 - 481	(+)	
**GXP_90947** [GXP_90947] (1 - 639)<br>**HS1BP3**, GXL_75700, GeneID: 64342, Homo sapiens chr. 2<br>HCLS1 binding protein 3	E2FF-NFKB	233 - 348	(+)	
**GXP_918032** [GXP_918032] (1 - 601)<br>**RBJ**, GXL_75716, GeneID: 51277, Homo sapiens chr. 2<br>Ras-associated protein Rap1	E2FF-NFKB	352 - 461	(+)	
	E2FF-NFKB	301 - 417	(+)	

**GXP_918072** [GXP_918072] (1 - 601)<br>**MPV17**, GXL_75726, GeneID: 4358, Homo sapiens chr. 2<br>MpV17 mitochondrial inner membrane protein				
**GXP_918076** [GXP_918076] (1 - 601)<br>**PPM1G**, GXL_75731, GeneID: 5496, Homo sapiens chr. 2<br>protein phosphatase 1G (formerly 2C), magnesium-dependent, gamma isoform	E2FF-NFKB	495 - 375	(-)	
**GXP_91025** [GXP_91025] (1 - 601)<br>**THUMPD2**, GXL_75754, GeneID: 80745, Homo sapiens chr. 2<br>THUMP domain containing 2	E2FF-NFKB	454 - 582	(+)	
**GXP_649459** [GXP_649459] (1 - 601)<br>**TTC32**, GXL_75783, GeneID: 130502, Homo sapiens chr. 2<br>tetratricopeptide repeat domain 32	E2FF-NFKB	221 - 343	(+)	
**GXP_910056** [GXP_910056] (1 - 679)<br>**GP2**, GXL_75848, GeneID: 2813, Homo sapiens chr. 16<br>glycoprotein 2 (zymogen granule membrane)	E2FF-NFKB	400 - 294	(-)	
**GXP_91142** [GXP_91142] (1 - 601)<br>**RNPS1**, GXL_75854, GeneID: 10921, Homo sapiens chr. 16<br>RNA binding protein S1, serine-rich domain	E2FF-NFKB	149 - 20	(-)	
**GXP_91144** [GXP_91144] (1 - 601)<br>**RNPS1**, GXL_75854, GeneID: 10921, Homo sapiens chr. 16<br>RNA binding protein S1, serine-rich domain	E2FF-NFKB	216 - 339	(+)	
	E2FF-NFKB	466 - 337	(-)	
**GXP_91148** [GXP_91148] (1 - 672)<br>**TMEM8**, GXL_75857, GeneID: 58986, Homo sapiens chr. 16<br>transmembrane protein 8 (five membrane-spanning domains)	E2FF-NFKB	318 - 205	(-)	
**GXP_909798** [GXP_909798] (1 - 601)<br>**NARFL**, GXL_75859, GeneID: 64428, Homo sapiens chr. 16<br>nuclear prelamin A recognition factor-like	E2FF-NFKB	188 - 300	(+)	
**GXP_910059** [GXP_910059] (1 - 827)<br>**UMOD**, GXL_75877, GeneID: 7369, Homo sapiens chr. 16<br>uromodulin (uromucoid, Tamm-Horsfall glycoprotein)	E2FF-NFKB	381 - 493	(+)	
**GXP_91187** [GXP_91187] (1 - 1387)<br>**ZKSCAN2**, GXL_75883, GeneID: 342357, Homo sapiens chr. 16<br>zinc finger with KRAB and SCAN domains 2	E2FF-NFKB	404 - 292	(-)	
	E2FF-NFKB	987 - 875	(-)	
**GXP_91200** [GXP_91200] (1 - 1145)<br>**CLN3**, GXL_75890, GeneID: 1201, Homo sapiens chr. 16<br>ceroid-lipofuscinosis, neuronal 3, juvenile (Batten, Spielmeyer-Vogt disease)	E2FF-NFKB	1108 - 983	(-)	
**GXP_645089** [GXP_645089] (1 - 601)<br>**GTF3C1**, GXL_75923, GeneID: 2975, Homo sapiens chr. 16<br>general transcription factor IIIC, polypeptide 1, alpha 220kDa	E2FF-NFKB	437 - 544	(+)	
	E2FF-NFKB	123 - 243	(+)	

**GXP_909926** [GXP_909926] (1 - 601)<br>**ADCY9**, GXL_75928, GeneID: 115, Homo sapiens chr. 16<br>adenylate cyclase 9				
**GXP_91278** [GXP_91278] (1 - 706)<br>**KIAA0430**, GXL_75932, GeneID: 9665, Homo sapiens chr. 16<br>KIAA0430	E2FF-NFKB	288 - 411	(+)	
**GXP_91290** [GXP_91290] (1 - 601)<br>**N-PAC**, GXL_75937, GeneID: 84656, Homo sapiens chr. 16<br>cytokine-like nuclear factor n-pac	E2FF-NFKB	224 - 99	(-)	
**GXP_91298** [GXP_91298] (1 - 601)<br>**CORO7**, GXL_75941, GeneID: 79585, Homo sapiens chr. 16<br>coronin 7	E2FF-NFKB	278 - 407	(+)	
**GXP_91381** [GXP_91381] (1 - 601)<br>**GGA2**, GXL_76004, GeneID: 23062, Homo sapiens chr. 16<br>golgi associated, gamma adaptin ear containing, ARF binding protein 2	E2FF-NFKB	341 - 232	(-)	
**GXP_91428** [GXP_91428] (1 - 672)<br>**DOC2A**, GXL_76039, GeneID: 8448, Homo sapiens chr. 16<br>double C2-like domains, alpha	E2FF-NFKB	276 - 162	(-)	
**GXP_91430** [GXP_91430] (1 - 993)<br>**KCTD13**, GXL_76040, GeneID: 253980, Homo sapiens chr. 16<br>potassium channel tetramerisation domain containing 13	E2FF-NFKB	646 - 753	(+)	
**GXP_91443** [GXP_91443] (1 - 1307)<br>**CDIPT**, GXL_76052, GeneID: 10423, Homo sapiens chr. 16<br>CDP-diacylglycerol--inositol 3-phosphatidyltransferase (phosphatidylinositol synthase)	E2FF-NFKB	1180 - 1053	(-)	
**GXP_909978** [GXP_909978] (1 - 1044)<br>**GRIN2A**, GXL_76053, GeneID: 2903, Homo sapiens chr. 16<br>glutamate receptor, ionotropic, N-methyl D-aspartate 2A	E2FF-NFKB	901 - 1010	(+)	
**GXP_91455** [GXP_91455] (1 - 699)<br>**C16orf42**, GXL_76063, GeneID: 115939, Homo sapiens chr. 16<br>chromosome 16 open reading frame 42	E2FF-NFKB	548 - 661	(+)	
**GXP_477778** [GXP_477778] (1 - 1453)<br>**CDCP2,LOC606495**, GXL_77629, GeneID: 200008,606495, Homo sapiens chr. 1<br>CUB domain containing protein 2; hypothetical protein LOC606495	E2FF-NFKB	724 - 606	(-)	
**GXP_93525** [GXP_93525] (1 - 943)<br>**SSX2IP**, GXL_77683, GeneID: 117178, Homo sapiens chr. 1<br>synovial sarcoma, X breakpoint 2 interacting protein	E2FF-NFKB	534 - 653	(+)	
**GXP_93538** [GXP_93538] (1 - 1367)<br>**DNTTIP2**, GXL_77690, GeneID: 30836, Homo sapiens chr. 1<br>deoxynucleotidyltransferase, terminal, interacting protein 2	E2FF-NFKB	1170 - 1049	(-)	
	E2FF-NFKB	71 - 186	(+)	

**GXP_93561** [GXP_93561] (1 - 877)<br>**RHOC**, GXL_77707, GeneID: 389, Homo sapiens chr. 1<br>ras homolog gene family, member C				
**GXP_93589** [GXP_93589] (1 - 601)<br>**DDAH1**, GXL_77723, GeneID: 23576, Homo sapiens chr. 1<br>dimethylarginine dimethylaminohydrolase 1	E2FF-NFKB	262 - 386	(+)	
**GXP_93595** [GXP_93595] (1 - 748)<br>**SYT6**, GXL_77727, GeneID: 148281, Homo sapiens chr. 1<br>synaptotagmin VI	E2FF-NFKB	487 - 375	(-)	
**GXP_93621** [GXP_93621] (1 - 601)<br>**C1orf183**, GXL_77746, GeneID: 55924, Homo sapiens chr. 1<br>chromosome 1 open reading frame 183	E2FF-NFKB	456 - 568	(+)	
**GXP_93628** [GXP_93628] (1 - 642)<br>**C1orf41**, GXL_77752, GeneID: 51668, Homo sapiens chr. 1<br>chromosome 1 open reading frame 41	E2FF-NFKB	423 - 537	(+)	
**GXP_914983** [GXP_914983] (1 - 1415)<br>**SYDE2**, GXL_77773, GeneID: 84144, Homo sapiens chr. 1<br>synapse defective 1, Rho GTPase, homolog 2 (C. elegans)	E2FF-NFKB	139 - 20	(-)	
**GXP_477874** [GXP_477874] (1 - 927)<br>**ANKRD13C**, GXL_77838, GeneID: 81573, Homo sapiens chr. 1<br>ankyrin repeat domain 13C	E2FF-NFKB	540 - 426	(-)	
**GXP_660265** [GXP_660265] (1 - 1070)<br>**CPA5**, GXL_78229, GeneID: 93979, Homo sapiens chr. 7<br>carboxypeptidase A5	E2FF-NFKB	1003 - 876	(-)	
**GXP_94229** [GXP_94229] (1 - 800)<br>**TMEM142B,LOC729969,LOC730276,LOC730526**, GXL_78240, GeneID: 80228,729969,730276,730526, Homo sapiens chr. 7<br>transmembrane protein 142B;similar to Protein C7orf19; similar to Protein C7orf19;similar to Protein C7orf19	E2FF-NFKB	749 - 622	(-)	
**GXP_94239** [GXP_94239] (1 - 832)<br>**LHFPL3**, GXL_78245, GeneID: 375612, Homo sapiens chr. 7<br>lipoma HMGIC fusion partner-like 3	E2FF-NFKB	293 - 175	(-)	
**GXP_94307** [GXP_94307] (1 - 601)<br>**PRKRIP1**, GXL_78288, GeneID: 79706, Homo sapiens chr. 7<br>PRKR interacting protein 1 (IL11 inducible)	E2FF-NFKB	40 - 164	(+)	
**GXP_94331** [GXP_94331] (1 - 715)<br>**ZNF277P**, GXL_78303, GeneID: 11179, Homo sapiens chr. 7<br>zinc finger protein 277 pseudogene	E2FF-NFKB	668 - 542	(-)	
**GXP_94380** [GXP_94380] (1 - 1276)<br>**FLJ25778**, GXL_78327, GeneID: 254048, Homo sapiens chr. 7<br>hypothetical protein FLJ25778	E2FF-NFKB	367 - 251	(-)	
	E2FF-NFKB	442 - 318	(-)	

**GXP_94417** [GXP_94417] (1 - 636)<br>**METTL2B**, GXL_78351, GeneID: 55798, Homo sapiens chr. 7<br>methyltransferase like 2B				
**GXP_925298** [GXP_925298] (1 - 622)<br>**SLC26A4**, GXL_78359, GeneID: 5172, Homo sapiens chr. 7<br>solute carrier family 26, member 4	E2FF-NFKB	349 - 226	(-)	
**GXP_94438** [GXP_94438] (1 - 886)<br>**CAV1**, GXL_78368, GeneID: 857, Homo sapiens chr. 7<br>caveolin 1, caveolae protein, 22kDa	E2FF-NFKB	695 - 567	(-)	
**GXP_925327** [GXP_925327] (1 - 601)<br>**CAV1**, GXL_78368, GeneID: 857, Homo sapiens chr. 7<br>caveolin 1, caveolae protein, 22kDa	E2FF-NFKB	287 - 159	(-)	
**GXP_94452** [GXP_94452] (1 - 641)<br>**ARPC1B,LOC653888**, GXL_78381, GeneID: 10095,653888, Homo sapiens chr. 7<br>actin related protein 2/3 complex, subunit 1B, 41kDa; similar to Actin-related protein 2/3 complex subunit 1B (ARP2/3 complex 41 kDa subunit) (p41-ARC)	E2FF-NFKB	81 - 206	(+)	
**GXP_94490** [GXP_94490] (1 - 924)<br>**PTPRZ1**, GXL_78417, GeneID: 5803, Homo sapiens chr. 7<br>protein tyrosine phosphatase, receptor-type, Z polypeptide 1	E2FF-NFKB	490 - 598	(+)	
**GXP_94514** [GXP_94514] (1 - 601)<br>**MOSPD3**, GXL_78440, GeneID: 64598, Homo sapiens chr. 7<br>motile sperm domain containing 3	E2FF-NFKB	547 - 431	(-)	
**GXP_659980** [GXP_659980] (1 - 1229)<br>**MOSPD3**, GXL_78440, GeneID: 64598, Homo sapiens chr. 7<br>motile sperm domain containing 3	E2FF-NFKB	936 - 820	(-)	
**GXP_94515** [GXP_94515] (1 - 715)<br>**DLD**, GXL_78441, GeneID: 1738, Homo sapiens chr. 7<br>dihydrolipoamide dehydrogenase	E2FF-NFKB	584 - 705	(+)	
**GXP_916977** [GXP_916977] (1 - 601)<br>**STX16**, GXL_81791, GeneID: 8675, Homo sapiens chr. 20<br>syntaxin 16	E2FF-NFKB	571 - 463	(-)	
**GXP_652277** [GXP_652277] (1 - 1182)<br>**OSBPL2**, GXL_81800, GeneID: 9885, Homo sapiens chr. 20<br>oxysterol binding protein-like 2	E2FF-NFKB	1093 - 984	(-)	
**GXP_917041** [GXP_917041] (1 - 601)<br>**ARFGAP1**, GXL_81801, GeneID: 55738, Homo sapiens chr. 20<br>ADP-ribosylation factor GTPase activating protein 1	E2FF-NFKB	351 - 465	(+)	
**GXP_916969** [GXP_916969] (1 - 601)<br>**RBM38**, GXL_81802, GeneID: 55544, Homo sapiens chr. 20<br>RNA binding motif protein 38	E2FF-NFKB	64 - 170	(+)	

**GXP_98094** [GXP_98094] (1 - 601)<br>**GNAS**, GXL_81818, GeneID: 2778, Homo sapiens chr. 20<br>GNAS complex locus	E2FF-NFKB	584 - 455	(-)	
**GXP_98110** [GXP_98110] (1 - 657)<br>**OGFR**, GXL_81825, GeneID: 11054, Homo sapiens chr. 20<br>opioid growth factor receptor	E2FF-NFKB	453 - 344	(-)	
**GXP_917081** [GXP_917081] (1 - 601)<br>**MYT1**, GXL_81834, GeneID: 4661, Homo sapiens chr. 20<br>myelin transcription factor 1	E2FF-NFKB	177 - 298	(+)	
**GXP_106310** [GXP_106310] (1 - 601)<br>**HSPD1**, GXL_88983, GeneID: 3329, Homo sapiens chr. 2<br>heat shock 60kDa protein 1 (chaperonin)	E2FF-NFKB	163 - 284	(+)	
**GXP_106352** [GXP_106352] (1 - 657)<br>**AAMP**, GXL_89008, GeneID: 14, Homo sapiens chr. 2<br>angio-associated, migratory cell protein	E2FF-NFKB	588 - 466	(-)	
**GXP_919420** [GXP_919420] (1 - 601)<br>**AAMP**, GXL_89008, GeneID: 14, Homo sapiens chr. 2<br>angio-associated, migratory cell protein	E2FF-NFKB<br>E2FF-NFKB	157 - 35<br>570 - 442	(-)<br>(-)	
**GXP_919412** [GXP_919412] (1 - 601)<br>**TNS1**, GXL_89022, GeneID: 7145, Homo sapiens chr. 2<br>tensin 1	E2FF-NFKB	480 - 366	(-)	
**GXP_106400** [GXP_106400] (1 - 653)<br>**ALS2CR4,LOC645747**, GXL_89036, GeneID: 65062,645747, Homo sapiens chr. 2<br>amyotrophic lateral sclerosis 2 (juvenile) chromosome region, candidate 4;hypothetical LOC645747	E2FF-NFKB	255 - 128	(-)	
**GXP_106413** [GXP_106413] (1 - 601)<br>**RPS7**, GXL_89044, GeneID: 6201, Homo sapiens chr. 2<br>ribosomal protein S7	E2FF-NFKB	558 - 441	(-)	
**GXP_649368** [GXP_649368] (1 - 622)<br>**RPS7**, GXL_89044, GeneID: 6201, Homo sapiens chr. 2<br>ribosomal protein S7	E2FF-NFKB	239 - 122	(-)	
**GXP_106419** [GXP_106419] (1 - 643)<br>**PER2**, GXL_89050, GeneID: 8864, Homo sapiens chr. 2<br>period homolog 2 (Drosophila)	E2FF-NFKB	468 - 584	(+)	
**GXP_106435** [GXP_106435] (1 - 601)<br>**HDLBP**, GXL_89056, GeneID: 3069, Homo sapiens chr. 2<br>high density lipoprotein binding protein (vigilin)	E2FF-NFKB	270 - 386	(+)	
**GXP_106442** [GXP_106442] (1 - 1123)<br>**SKIP**, GXL_89062, GeneID: 80309, Homo sapiens chr. 2<br>SPHK1 (sphingosine kinase type 1) interacting protein	E2FF-NFKB	919 - 1039	(+)	
	E2FF-NFKB	158 - 284	(+)	

**GXP_919455** [GXP_919455] (1 - 601)<br>**GLB1L**, GXL_89084, GeneID: 79411, Homo sapiens chr. 2<br>galactosidase, beta 1-like				
**GXP_106499** [GXP_106499] (1 - 766)<br>**ILKAP**, GXL_89097, GeneID: 80895, Homo sapiens chr. 2<br>integrin-linked kinase-associated serine/threonine phosphatase 2C	E2FF-NFKB	420 - 549	(+)	
**GXP_106525** [GXP_106525] (1 - 601)<br>**ZNF142**, GXL_89117, GeneID: 7701, Homo sapiens chr. 2<br>zinc finger protein 142	E2FF-NFKB	482 - 590	(+)	
**GXP_919491** [GXP_919491] (1 - 624)<br>**SERPINE2**, GXL_89120, GeneID: 5270, Homo sapiens chr. 2<br>serpin peptidase inhibitor, clade E (nexin, plasminogen activator inhibitor type 1), member 2	E2FF-NFKB	423 - 541	(+)	
**GXP_106561** [GXP_106561] (1 - 788)<br>**GTF3C3**, GXL_89141, GeneID: 9330, Homo sapiens chr. 2<br>general transcription factor IIIC, polypeptide 3, 102kDa	E2FF-NFKB	275 - 392	(+)	
**GXP_106617** [GXP_106617] (1 - 1045)<br>**RNF25**, GXL_89190, GeneID: 64320, Homo sapiens chr. 2<br>ring finger protein 25	E2FF-NFKB	894 - 773	(-)	
**GXP_106982** [GXP_106982] (1 - 634)<br>**CHM**, GXL_89491, GeneID: 1121, Homo sapiens chr. X<br>choroideremia (Rab escort protein 1)	E2FF-NFKB	533 - 405	(-)	
**GXP_106983** [GXP_106983] (1 - 601)<br>**CHM**, GXL_89491, GeneID: 1121, Homo sapiens chr. X<br>choroideremia (Rab escort protein 1)	E2FF-NFKB	274 - 146	(-)	
**GXP_107057** [GXP_107057] (1 - 1128)<br>**FAM127C**, GXL_89527, GeneID: 441518, Homo sapiens chr. X<br>family with sequence similarity 127, member C	E2FF-NFKB	498 - 384	(-)	
**GXP_928353** [GXP_928353] (1 - 601)<br>**IDH3G**, GXL_89530, GeneID: 3421, Homo sapiens chr. X<br>isocitrate dehydrogenase 3 (NAD+) gamma	E2FF-NFKB	138 - 267	(+)	
**GXP_107068** [GXP_107068] (1 - 601)<br>**F8**, GXL_89535, GeneID: 2157, Homo sapiens chr. X<br>coagulation factor VIII, procoagulant component (hemophilia A)	E2FF-NFKB	248 - 373	(+)	
**GXP_107084** [GXP_107084] (1 - 678)<br>**UPF3B**, GXL_89544, GeneID: 65109, Homo sapiens chr. X<br>UPF3 regulator of nonsense transcripts homolog B (yeast)	E2FF-NFKB	321 - 444	(+)	
	E2FF-NFKB	443 - 568	(+)	
**GXP_107137** [GXP_107137] (1 - 601)<br>**SYTL4**, GXL_89584, GeneID: 94121, Homo sapiens chr. X<br>synaptotagmin-like 4 (granuphilin-a)	E2FF-NFKB	497 - 372	(-)	
	E2FF-NFKB	125 - 5	(-)	

**GXP_107154** [GXP_107154] (1 - 601)<br>**UCHL5IP**, GXL_89593, GeneID: 55559, Homo sapiens chr. X<br>UCHL5 interacting protein				
**GXP_107183** [GXP_107183] (1 - 1222)<br>**TMSB4X**, GXL_89613, GeneID: 7114, Homo sapiens chr. X<br>thymosin, beta 4, X-linked	E2FF-NFKB	1105 - 1220	(+)	
**GXP_928386** [GXP_928386] (1 - 649)<br>**UBL4A**, GXL_89614, GeneID: 8266, Homo sapiens chr. X<br>ubiquitin-like 4A	E2FF-NFKB	155 - 273	(+)	
**GXP_107205** [GXP_107205] (1 - 878)<br>**PRPS2**, GXL_89630, GeneID: 5634, Homo sapiens chr. X<br>phosphoribosyl pyrophosphate synthetase 2	E2FF-NFKB	691 - 563	(-)	
**GXP_928389** [GXP_928389] (1 - 601)<br>**G6PD**, GXL_89652, GeneID: 2539, Homo sapiens chr. X<br>glucose-6-phosphate dehydrogenase	E2FF-NFKB	155 - 45	(-)	
**GXP_928354** [GXP_928354] (1 - 601)<br>**PDZD4**, GXL_89665, GeneID: 57595, Homo sapiens chr. X<br>PDZ domain containing 4	E2FF-NFKB	359 - 482	(+)	
**GXP_107274** [GXP_107274] (1 - 984)<br>**CUL4B**, GXL_89686, GeneID: 8450, Homo sapiens chr. X<br>cullin 4B	E2FF-NFKB	312 - 198	(-)	
**GXP_107292** [GXP_107292] (1 - 980)<br>**CHRDL1**, GXL_89699, GeneID: 91851, Homo sapiens chr. X<br>chordin-like 1	E2FF-NFKB	150 - 263	(+)	
**GXP_107339** [GXP_107339] (1 - 729)<br>**BEX2**, GXL_89745, GeneID: 84707, Homo sapiens chr. X<br>brain expressed X-linked 2	E2FF-NFKB	207 - 329	(+)	
**GXP_107340** [GXP_107340] (1 - 601)<br>**SLC10A3**, GXL_89746, GeneID: 8273, Homo sapiens chr. X<br>solute carrier family 10 (sodium/bile acid cotransporter family), member 3	E2FF-NFKB	135 - 253	(+)	
**GXP_107348** [GXP_107348] (1 - 748)<br>**GLA**, GXL_89754, GeneID: 2717, Homo sapiens chr. X<br>galactosidase, alpha	E2FF-NFKB	608 - 728	(+)	
**GXP_107357** [GXP_107357] (1 - 601)<br>**FAM58A**, GXL_89763, GeneID: 92002, Homo sapiens chr. X<br>family with sequence similarity 58, member A	E2FF-NFKB	460 - 566	(+)	
**GXP_927515** [GXP_927515] (1 - 601)<br>**CLCN4**, GXL_89770, GeneID: 1183, Homo sapiens chr. X<br>chloride channel 4	E2FF-NFKB	303 - 419	(+)	
	E2FF-NFKB	471 - 355	(-)	

**GXP_910633** [GXP_910633] (1 - 601)<br>**KIAA0174**, GXL_89810, GeneID: 9798, Homo sapiens chr. 16<br>KIAA0174				
**GXP_107460** [GXP_107460] (1 - 601)<br>**CPNE7,LOC652777**, GXL_89818, GeneID: 27132,652777, Homo sapiens chr. 16<br>copine VII;similar to Copine-7 (Copine VII)	E2FF-NFKB	456 - 330	(-)	
**GXP_107486** [GXP_107486] (1 - 620)<br>**GAN**, GXL_89829, GeneID: 8139, Homo sapiens chr. 16<br>giant axonal neuropathy (gigaxonin)	E2FF-NFKB	426 - 533	(+)	
**GXP_107514** [GXP_107514] (1 - 628)<br>**CES2**, GXL_89842, GeneID: 8824, Homo sapiens chr. 16<br>carboxylesterase 2 (intestine, liver)	E2FF-NFKB	267 - 140	(-)	
**GXP_107584** [GXP_107584] (1 - 602)<br>**IL17C**, GXL_89889, GeneID: 27189, Homo sapiens chr. 16<br>interleukin 17C	E2FF-NFKB	507 - 392	(-)	
**GXP_107678** [GXP_107678] (1 - 601)<br>**SLC9A5**, GXL_89958, GeneID: 6553, Homo sapiens chr. 16<br>solute carrier family 9 (sodium/hydrogen exchanger), member 5	E2FF-NFKB	281 - 387	(+)	
**GXP_107713** [GXP_107713] (1 - 1108)<br>**NUTF2**, GXL_89992, GeneID: 10204, Homo sapiens chr. 16<br>nuclear transport factor 2	E2FF-NFKB	930 - 816	(-)	
**GXP_904748** [GXP_904748] (1 - 601)<br>**CTBP2**, GXL_90428, GeneID: 1488, Homo sapiens chr. 10<br>C-terminal binding protein 2	E2FF-NFKB	390 - 282	(-)	
**GXP_108197** [GXP_108197] (1 - 601)<br>**MRC1**, GXL_90435, GeneID: 4360, Homo sapiens chr. 10<br>mannose receptor, C type 1	E2FF-NFKB	46 - 153	(+)	
**GXP_108206** [GXP_108206] (1 - 758)<br>**SEC61A2**, GXL_90438, GeneID: 55176, Homo sapiens chr. 10<br>Sec61 alpha 2 subunit (S. cerevisiae)	E2FF-NFKB	438 - 315	(-)	
**GXP_108213** [GXP_108213] (1 - 617)<br>**MRC1L1**, GXL_90442, GeneID: 414308, Homo sapiens chr. 10<br>mannose receptor, C type 1-like 1	E2FF-NFKB	59 - 166	(+)	
**GXP_108230** [GXP_108230] (1 - 893)<br>**MLLT10**, GXL_90450, GeneID: 8028, Homo sapiens chr. 10<br>myeloid/lymphoid or mixed-lineage leukemia (trithorax homolog, Drosophila), translocated to, 10	E2FF-NFKB	312 - 201	(-)	
**GXP_904664** [GXP_904664] (1 - 708)<br>**HSPA12A**, GXL_90451, GeneID: 259217, Homo sapiens chr. 10<br>heat shock 70kDa protein 12A	E2FF-NFKB	531 - 646	(+)	
	E2FF-NFKB	282 - 161	(-)	

**GXP_108247** [GXP_108247] (1 - 1050)<br>**THNSL1**, GXL_90455, GeneID: 79896, Homo sapiens chr. 10<br>threonine synthase-like 1 (bacterial)				
**GXP_108264** [GXP_108264] (1 - 659)<br>**DHTKD1**, GXL_90463, GeneID: 55526, Homo sapiens chr. 10<br>dehydrogenase E1 and transketolase domain containing 1	E2FF-NFKB	283 - 412	(+)	
**GXP_639287** [GXP_639287] (1 - 601)<br>**BNIP3**, GXL_90468, GeneID: 664, Homo sapiens chr. 10<br>BCL2/adenovirus E1B 19kDa interacting protein 3	E2FF-NFKB	49 - 178	(+)	
**GXP_108301** [GXP_108301] (1 - 654)<br>**GTPBP4**, GXL_90490, GeneID: 23560, Homo sapiens chr. 10<br>GTP binding protein 4	E2FF-NFKB	418 - 537	(+)	
**GXP_108339** [GXP_108339] (1 - 679)<br>**PFKP**, GXL_90508, GeneID: 5214, Homo sapiens chr. 10<br>phosphofructokinase, platelet	E2FF-NFKB	521 - 646	(+)	
**GXP_108345** [GXP_108345] (1 - 601)<br>**PFKP**, GXL_90508, GeneID: 5214, Homo sapiens chr. 10<br>phosphofructokinase, platelet	E2FF-NFKB	127 - 246	(+)	
**GXP_108356** [GXP_108356] (1 - 1248)<br>**VIM**, GXL_90515, GeneID: 7431, Homo sapiens chr. 10<br>vimentin	E2FF-NFKB	436 - 549	(+)	
**GXP_108362** [GXP_108362] (1 - 601)<br>**CAMK1D**, GXL_90517, GeneID: 57118, Homo sapiens chr. 10<br>calcium/calmodulin-dependent protein kinase ID	E2FF-NFKB	36 - 156	(+)	
	E2FF-NFKB	249 - 142	(-)	
**GXP_904802** [GXP_904802] (1 - 601)<br>**C10orf125**, GXL_90555, GeneID: 282969, Homo sapiens chr. 10<br>chromosome 10 open reading frame 125	E2FF-NFKB	259 - 383	(+)	
**GXP_904751** [GXP_904751] (1 - 602)<br>**DHX32**, GXL_90570, GeneID: 55760, Homo sapiens chr. 10<br>DEAH (Asp-Glu-Ala-His) box polypeptide 32	E2FF-NFKB	149 - 256	(+)	
**GXP_108438** [GXP_108438] (1 - 844)<br>**HSPA14**, GXL_90573, GeneID: 51182, Homo sapiens chr. 10<br>heat shock 70kDa protein 14	E2FF-NFKB	587 - 713	(+)	
**GXP_108447** [GXP_108447] (1 - 996)<br>**PLXDC2**, GXL_90581, GeneID: 84898, Homo sapiens chr. 10<br>plexin domain containing 2	E2FF-NFKB	547 - 666	(+)	
**GXP_639150** [GXP_639150] (1 - 601)<br>**GFRA1**, GXL_90623, GeneID: 2674, Homo sapiens chr. 10<br>GDNF family receptor alpha 1	E2FF-NFKB	109 - 229	(+)	
	E2FF-NFKB	474 - 594	(+)	

**GXP_639151** [GXP_639151] (1 - 601)<br>**GFRA1**, GXL_90623, GeneID: 2674, Homo sapiens chr. 10<br>GDNF family receptor alpha 1				
**GXP_108529** [GXP_108529] (1 - 669)<br>**C10orf47**, GXL_90652, GeneID: 254427, Homo sapiens chr. 10<br>chromosome 10 open reading frame 47	E2FF-NFKB	511 - 638	(+)	
**GXP_116394** [GXP_116394] (1 - 678)<br>**NSUN5**, GXL_97642, GeneID: 55695, Homo sapiens chr. 7<br>NOL1/NOP2/Sun domain family, member 5	E2FF-NFKB	221 - 348	(+)	
**GXP_116494** [GXP_116494] (1 - 601)<br>**MCM7**, GXL_97697, GeneID: 4176, Homo sapiens chr. 7<br>MCM7 minichromosome maintenance deficient 7 (S. cerevisiae)	E2FF-NFKB	242 - 362	(+)	
	E2FF-NFKB	534 - 426	(-)	
**GXP_116501** [GXP_116501] (1 - 702)<br>**CYP3A5**, GXL_97701, GeneID: 1577, Homo sapiens chr. 7<br>cytochrome P450, family 3, subfamily A, polypeptide 5	E2FF-NFKB	301 - 180	(-)	
	E2FF-NFKB	630 - 512	(-)	
**GXP_116536** [GXP_116536] (1 - 734)<br>**BCL7B**, GXL_97722, GeneID: 9275, Homo sapiens chr. 7<br>B-cell CLL/lymphoma 7B	E2FF-NFKB	518 - 411	(-)	
**GXP_116546** [GXP_116546] (1 - 818)<br>**CALN1**, GXL_97726, GeneID: 83698, Homo sapiens chr. 7<br>calneuron 1	E2FF-NFKB	48 - 159	(+)	
**GXP_116573** [GXP_116573] (1 - 814)<br>**C7orf23**, GXL_97749, GeneID: 79161, Homo sapiens chr. 7<br>chromosome 7 open reading frame 23	E2FF-NFKB	556 - 437	(-)	
**GXP_659901** [GXP_659901] (1 - 601)<br>**TMEM130**, GXL_97751, GeneID: 222865, Homo sapiens chr. 7<br>transmembrane protein 130	E2FF-NFKB	248 - 371	(+)	
**GXP_116596** [GXP_116596] (1 - 636)<br>**TMEM60**, GXL_97763, GeneID: 85025, Homo sapiens chr. 7<br>transmembrane protein 60	E2FF-NFKB	157 - 34	(-)	
**GXP_659694** [GXP_659694] (1 - 601)<br>**STYXL1**, GXL_97780, GeneID: 51657, Homo sapiens chr. 7<br>serine/threonine/tyrosine interacting-like 1	E2FF-NFKB	361 - 480	(+)	
**GXP_116651** [GXP_116651] (1 - 601)<br>**CLDN3**, GXL_97809, GeneID: 1365, Homo sapiens chr. 7<br>claudin 3	E2FF-NFKB	286 - 167	(-)	
**GXP_116713** [GXP_116713] (1 - 660)<br>**MTERF**, GXL_97863, GeneID: 7978, Homo sapiens chr. 7<br>mitochondrial transcription termination factor	E2FF-NFKB	343 - 223	(-)	
	E2FF-NFKB	426 - 542	(+)	

**GXP_117625** [GXP_117625] (1 - 693)<br>**PRR14**, GXL_98504, GeneID: 78994, Homo sapiens chr. 16<br>proline rich 14				
**GXP_910229** [GXP_910229] (1 - 1061)<br>**PRR14**, GXL_98504, GeneID: 78994, Homo sapiens chr. 16<br>proline rich 14	E2FF-NFKB	159 - 275	(+)	
**GXP_910246** [GXP_910246] (1 - 601)<br>**FBXL19**, GXL_98507, GeneID: 54620, Homo sapiens chr. 16<br>F-box and leucine-rich repeat protein 19	E2FF-NFKB	550 - 440	(-)	
**GXP_117674** [GXP_117674] (1 - 601)<br>**ATXN2L,LOC730151**, GXL_98530, GeneID: 11273,730151, Homo sapiens chr. 16<br>ataxin 2-like;hypothetical protein LOC730151	E2FF-NFKB	212 - 85	(-)	
**GXP_117706** [GXP_117706] (1 - 1295)<br>**TNRC6A**, GXL_98548, GeneID: 27327, Homo sapiens chr. 16<br>trinucleotide repeat containing 6A	E2FF-NFKB	678 - 785	(+)	
**GXP_910075** [GXP_910075] (1 - 601)<br>**LYRM1**, GXL_98561, GeneID: 57149, Homo sapiens chr. 16<br>LYR motif containing 1	E2FF-NFKB	159 - 51	(-)	
**GXP_117771** [GXP_117771] (1 - 621)<br>**MT1H,MT1P2**, GXL_98585, GeneID: 4496,645745, Homo sapiens chr. 16<br>metallothionein 1H;metallothionein 1 pseudogene 2	E2FF-NFKB<br>E2FF-NFKB	198 - 325<br>489 - 615	(+)<br>(+)	
**GXP_117796** [GXP_117796] (1 - 874)<br>**SLC6A2**, GXL_98600, GeneID: 6530, Homo sapiens chr. 16<br>solute carrier family 6 (neurotransmitter transporter, noradrenalin), member 2	E2FF-NFKB	393 - 285	(-)	
**GXP_910384** [GXP_910384] (1 - 601)<br>**SLC6A2**, GXL_98600, GeneID: 6530, Homo sapiens chr. 16<br>solute carrier family 6 (neurotransmitter transporter, noradrenalin), member 2	E2FF-NFKB	561 - 453	(-)	
**GXP_117855** [GXP_117855] (1 - 677)<br>**CYLD**, GXL_98633, GeneID: 1540, Homo sapiens chr. 16<br>cylindromatosis (turban tumor syndrome)	E2FF-NFKB	230 - 359	(+)	
**GXP_910235** [GXP_910235] (1 - 860)<br>**PHKG2,LOC650556**, GXL_98738, GeneID: 5261,650556, Homo sapiens chr. 16<br>phosphorylase kinase, gamma 2 (testis); similar to Phosphorylase b kinase gamma catalytic chain, testis/liver isoform (PHK-gamma-T) (Phosphorylase kinase gamma subunit 2) (PSK-C3)	E2FF-NFKB	476 - 599	(+)	
**GXP_120981** [GXP_120981] (1 - 601)<br>**C14orf4**, GXL_101116, GeneID: 64207, Homo sapiens chr. 14<br>chromosome 14 open reading frame 4	E2FF-NFKB	69 - 194	(+)	
**GXP_120997** [GXP_120997] (1 - 601)<br>**C14orf65**, GXL_101122, GeneID: 317762, Homo sapiens chr. 14<br>chromosome 14 open reading frame 65	E2FF-NFKB	476 - 367	(-)	

**GXP_121000** [GXP_121000] (1 - 835)<br>**TMEM179**, GXL_101124, GeneID: 388021, Homo sapiens chr. 14<br>transmembrane protein 179	E2FF-NFKB	747 - 634	(-)	
**GXP_121030** [GXP_121030] (1 - 985)<br>**TRIP11**, GXL_101140, GeneID: 9321, Homo sapiens chr. 14<br>thyroid hormone receptor interactor 11	E2FF-NFKB	456 - 332	(-)	
**GXP_121059** [GXP_121059] (1 - 647)<br>**MTAC2D1**, GXL_101155, GeneID: 123036, Homo sapiens chr. 14<br>membrane targeting (tandem) C2 domain containing 1	E2FF-NFKB	446 - 338	(-)	
**GXP_121060** [GXP_121060] (1 - 601)<br>**MTAC2D1**, GXL_101155, GeneID: 123036, Homo sapiens chr. 14<br>membrane targeting (tandem) C2 domain containing 1	E2FF-NFKB	256 - 148	(-)	
**GXP_121067** [GXP_121067] (1 - 601)<br>**PPP1R13B**, GXL_101157, GeneID: 23368, Homo sapiens chr. 14<br>protein phosphatase 1, regulatory (inhibitor) subunit 13B	E2FF-NFKB	271 - 397	(+)	
**GXP_121084** [GXP_121084] (1 - 782)<br>**ANKRD9,LOC730248,LOC732155**, GXL_101166, GeneID: 122416,730248,732155, Homo sapiens chr. 14<br>ankyrin repeat domain 9; hypothetical protein LOC730248; hypothetical protein LOC732155	E2FF-NFKB	398 - 283	(-)	
**GXP_121107** [GXP_121107] (1 - 689)<br>**DEGS2**, GXL_101178, GeneID: 123099, Homo sapiens chr. 14<br>degenerative spermatocyte homolog 2, lipid desaturase (Drosophila)	E2FF-NFKB	232 - 349	(+)	
**GXP_908649** [GXP_908649] (1 - 601)<br>**BCL11B**, GXL_101195, GeneID: 64919, Homo sapiens chr. 14<br>B-cell CLL/lymphoma 11B (zinc finger protein)	E2FF-NFKB	267 - 159	(-)	
**GXP_908481** [GXP_908481] (1 - 601)<br>**ALDH6A1**, GXL_101197, GeneID: 4329, Homo sapiens chr. 14<br>aldehyde dehydrogenase 6 family, member A1	E2FF-NFKB	534 - 407	(-)	
**GXP_908496** [GXP_908496] (1 - 601)<br>**ACYP1**, GXL_101201, GeneID: 97, Homo sapiens chr. 14<br>acylphosphatase 1, erythrocyte (common) type	E2FF-NFKB	385 - 261	(-)	
**GXP_908482** [GXP_908482] (1 - 1242)<br>**ABCD4**, GXL_101202, GeneID: 5826, Homo sapiens chr. 14<br>ATP-binding cassette, sub-family D (ALD), member 4	E2FF-NFKB	821 - 934	(+)	
	E2FF-NFKB	1030 - 920	(-)	
**GXP_121199** [GXP_121199] (1 - 601)<br>**C14orf113**, GXL_101248, GeneID: 54792, Homo sapiens chr. 14<br>chromosome 14 open reading frame 113	E2FF-NFKB	566 - 458	(-)	
**GXP_911287** [GXP_911287] (1 - 601)<br>**RHBDL3**, GXL_102467, GeneID: 162494, Homo sapiens chr. 17<br>rhomboid, veinlet-like 3 (Drosophila)	E2FF-NFKB	302 - 195	(-)	
	E2FF-NFKB	395 - 272	(-)	

**GXP_122670** [GXP_122670] (1 - 898)<br>**ZPBP2**, GXL_102470, GeneID: 124626, Homo sapiens chr. 17<br>zona pellucida binding protein 2				
**GXP_122675** [GXP_122675] (1 - 601)<br>**SUZ12**, GXL_102474, GeneID: 23512, Homo sapiens chr. 17<br>suppressor of zeste 12 homolog (Drosophila)	E2FF-NFKB	265 - 382	(+)	
**GXP_911582** [GXP_911582] (1 - 601)<br>**FMNL1**, GXL_102505, GeneID: 752, Homo sapiens chr. 17<br>formin-like 1	E2FF-NFKB	297 - 424	(+)	
**GXP_122778** [GXP_122778] (1 - 696)<br>**RASL10B**, GXL_102532, GeneID: 91608, Homo sapiens chr. 17<br>RAS-like, family 10, member B	E2FF-NFKB	510 - 628	(+)	
**GXP_122794** [GXP_122794] (1 - 1030)<br>**MAPK7**, GXL_102543, GeneID: 5598, Homo sapiens chr. 17<br>mitogen-activated protein kinase 7	E2FF-NFKB	490 - 371	(-)	
**GXP_122823** [GXP_122823] (1 - 718)<br>**DHX8**, GXL_102560, GeneID: 1659, Homo sapiens chr. 17<br>DEAH (Asp-Glu-Ala-His) box polypeptide 8	E2FF-NFKB	261 - 152	(-)	
**GXP_122833** [GXP_122833] (1 - 866)<br>**GRB7**, GXL_102569, GeneID: 2886, Homo sapiens chr. 17<br>growth factor receptor-bound protein 7	E2FF-NFKB	585 - 471	(-)	
**GXP_122842** [GXP_122842] (1 - 1156)<br>**AP2B1**, GXL_102574, GeneID: 163, Homo sapiens chr. 17<br>adaptor-related protein complex 2, beta 1 subunit	E2FF-NFKB	942 - 1066	(+)	
**GXP_911325** [GXP_911325] (1 - 601)<br>**AP2B1**, GXL_102574, GeneID: 163, Homo sapiens chr. 17<br>adaptor-related protein complex 2, beta 1 subunit	E2FF-NFKB	103 - 227	(+)	
**GXP_122880** [GXP_122880] (1 - 1004)<br>**NMT1**, GXL_102601, GeneID: 4836, Homo sapiens chr. 17<br>N-myristoyltransferase 1	E2FF-NFKB	453 - 562	(+)	
**GXP_911575** [GXP_911575] (1 - 601)<br>**NMT1**, GXL_102601, GeneID: 4836, Homo sapiens chr. 17<br>N-myristoyltransferase 1	E2FF-NFKB	244 - 357	(+)	
**GXP_122900** [GXP_122900] (1 - 687)<br>**NEK8**, GXL_102618, GeneID: 284086, Homo sapiens chr. 17<br>NIMA (never in mitosis gene a)- related kinase 8	E2FF-NFKB	158 - 48	(-)	
**GXP_646640** [GXP_646640] (1 - 601)<br>**RAMP2**, GXL_102633, GeneID: 10266, Homo sapiens chr. 17<br>receptor (G protein-coupled) activity modifying protein 2	E2FF-NFKB	449 - 578	(+)	
	E2FF-NFKB	421 - 546	(+)	

**GXP_484368** [GXP_484368] (1 - 602)<br>**RARA**, GXL_102665, GeneID: 5914, Homo sapiens chr. 17<br>retinoic acid receptor, alpha				
**GXP_484370** [GXP_484370] (1 - 982)<br>**RARA**, GXL_102665, GeneID: 5914, Homo sapiens chr. 17<br>retinoic acid receptor, alpha	E2FF-NFKB	570 - 684	(+)	
**GXP_122990** [GXP_122990] (1 - 601)<br>**SNORD42A**, GXL_102688, GeneID: 26809, Homo sapiens chr. 17<br>small nucleolar RNA, C/D box 42A	E2FF-NFKB	10 - 133	(+)	
**GXP_123013** [GXP_123013] (1 - 806)<br>**PSMD11**, GXL_102709, GeneID: 5717, Homo sapiens chr. 17<br>proteasome (prosome, macropain) 26S subunit, non-ATPase, 11	E2FF-NFKB	150 - 33	(-)	
**GXP_123021** [GXP_123021] (1 - 690)<br>**CCL2**, GXL_102717, GeneID: 6347, Homo sapiens chr. 17<br>chemokine (C-C motif) ligand 2	E2FF-NFKB	229 - 123	(-)	
**GXP_123041** [GXP_123041] (1 - 601)<br>**LHX1**, GXL_102736, GeneID: 3975, Homo sapiens chr. 17<br>LIM homeobox 1	E2FF-NFKB	182 - 63	(-)	
**GXP_123540** [GXP_123540] (1 - 672)<br>**SIL1**, GXL_103142, GeneID: 64374, Homo sapiens chr. 5<br>SIL1 homolog, endoplasmic reticulum chaperone (S. cerevisiae)	E2FF-NFKB	308 - 416	(+)	
**GXP_123555** [GXP_123555] (1 - 601)<br>**TAF7**, GXL_103153, GeneID: 6879, Homo sapiens chr. 5<br>TAF7 RNA polymerase II, TATA box binding protein (TBP)-associated factor, 55kDa	E2FF-NFKB	399 - 290	(-)	
**GXP_490656** [GXP_490656] (1 - 1041)<br>**FBN2**, GXL_103171, GeneID: 2201, Homo sapiens chr. 5<br>fibrillin 2 (congenital contractural arachnodactyly)	E2FF-NFKB	939 - 818	(-)	
**GXP_123589** [GXP_123589] (1 - 848)<br>**MCC**, GXL_103178, GeneID: 4163, Homo sapiens chr. 5<br>mutated in colorectal cancers	E2FF-NFKB	471 - 360	(-)	
**GXP_123615** [GXP_123615] (1 - 917)<br>**FLJ11292**, GXL_103194, GeneID: 55338, Homo sapiens chr. 5<br>hypothetical protein FLJ11292	E2FF-NFKB	169 - 278	(+)	
**GXP_123617** [GXP_123617] (1 - 653)<br>**FCHSD1**, GXL_103196, GeneID: 89848, Homo sapiens chr. 5<br>FCH and double SH3 domains 1	E2FF-NFKB	424 - 295	(-)	
**GXP_923035** [GXP_923035] (1 - 601)<br>**PDGFRB,LOC651091**, GXL_103221, GeneID: 5159,651091, Homo sapiens chr. 5<br>platelet-derived growth factor receptor, beta polypeptide;hypothetical LOC651091	E2FF-NFKB	103 - 214	(+)	
	E2FF-NFKB	674 - 568	(-)	

**GXP_123681** [GXP_123681] (1 - 884)<br>**HINT1**, GXL_103236, GeneID: 3094, Homo sapiens chr. 5<br>histidine triad nucleotide binding protein 1				
**GXP_123725** [GXP_123725] (1 - 1285)<br>**FBXL17**, GXL_103261, GeneID: 64839, Homo sapiens chr. 5<br>F-box and leucine-rich repeat protein 17	E2FF-NFKB	672 - 554	(-)	
**GXP_123730** [GXP_123730] (1 - 1144)<br>**CDO1**, GXL_103265, GeneID: 1036, Homo sapiens chr. 5<br>cysteine dioxygenase, type I	E2FF-NFKB	554 - 672	(+)	
**GXP_123747** [GXP_123747] (1 - 601)<br>**GLRX**, GXL_103275, GeneID: 2745, Homo sapiens chr. 5<br>glutaredoxin (thioltransferase)	E2FF-NFKB	113 - 240	(+)	
**GXP_922848** [GXP_922848] (1 - 768)<br>**IRF1**, GXL_103285, GeneID: 3659, Homo sapiens chr. 5<br>interferon regulatory factor 1	E2FF-NFKB	394 - 275	(-)	
**GXP_123782** [GXP_123782] (1 - 601)<br>**SPOCK1**, GXL_103305, GeneID: 6695, Homo sapiens chr. 5<br>sparc/osteonectin, cwcv and kazal-like domains proteoglycan (testican) 1	E2FF-NFKB	17 - 125	(+)	
**GXP_922715** [GXP_922715] (1 - 601)<br>**ARTS-1**, GXL_103322, GeneID: 51752, Homo sapiens chr. 5<br>type 1 tumor necrosis factor receptor shedding aminopeptidase regulator	E2FF-NFKB	259 - 380	(+)	
**GXP_490672** [GXP_490672] (1 - 601)<br>**P4HA2**, GXL_103335, GeneID: 8974, Homo sapiens chr. 5<br>procollagen-proline, 2-oxoglutarate 4-dioxygenase (proline 4-hydroxylase), alpha polypeptide II	E2FF-NFKB	245 - 360	(+)	
**GXP_123844** [GXP_123844] (1 - 718)<br>**PJA2**, GXL_103362, GeneID: 9867, Homo sapiens chr. 5<br>praja 2, RING-H2 motif containing	E2FF-NFKB	142 - 13	(-)	
**GXP_124155** [GXP_124155] (1 - 746)<br>**OAS2**, GXL_103581, GeneID: 4939, Homo sapiens chr. 12<br>2'-5'-oligoadenylate synthetase 2, 69/71kDa	E2FF-NFKB	224 - 339	(+)	
**GXP_907510** [GXP_907510] (1 - 649)<br>**C12orf65**, GXL_103587, GeneID: 91574, Homo sapiens chr. 12<br>chromosome 12 open reading frame 65	E2FF-NFKB	531 - 421	(-)	
**GXP_907458** [GXP_907458] (1 - 633)<br>**SETD1B**, GXL_103609, GeneID: 23067, Homo sapiens chr. 12<br>SET domain containing 1B	E2FF-NFKB	133 - 262	(+)	
**GXP_124224** [GXP_124224] (1 - 876)<br>**KIAA1033**, GXL_103617, GeneID: 23325, Homo sapiens chr. 12<br>KIAA1033	E2FF-NFKB	288 - 405	(+)	
	E2FF-NFKB	425 - 299	(-)	

**GXP_907547** [GXP_907547] (1 - 601)<br>**TMEM132C**, GXL_103669, GeneID: 92293, Homo sapiens chr. 12<br>transmembrane protein 132C				
**GXP_124345** [GXP_124345] (1 - 627)<br>**HYPE**, GXL_103684, GeneID: 11153, Homo sapiens chr. 12<br>Huntingtin interacting protein E	E2FF-NFKB	97 - 209	(+)	
**GXP_124351** [GXP_124351] (1 - 1028)<br>**C12orf38**, GXL_103689, GeneID: 79867, Homo sapiens chr. 12<br>chromosome 12 open reading frame 38	E2FF-NFKB	767 - 658	(-)	
**GXP_124353** [GXP_124353] (1 - 1011)<br>**ALDH2**, GXL_103690, GeneID: 217, Homo sapiens chr. 12<br>aldehyde dehydrogenase 2 family (mitochondrial)	E2FF-NFKB	795 - 672	(-)	
**GXP_124359** [GXP_124359] (1 - 958)<br>**SETD8**, GXL_103694, GeneID: 387893, Homo sapiens chr. 12<br>SET domain containing (lysine methyltransferase) 8	E2FF-NFKB	769 - 876	(+)	
**GXP_124392** [GXP_124392] (1 - 802)<br>**HCFC2**, GXL_103722, GeneID: 29915, Homo sapiens chr. 12<br>host cell factor C2	E2FF-NFKB	637 - 513	(-)	
**GXP_907252** [GXP_907252] (1 - 774)<br>**HCFC2**, GXL_103722, GeneID: 29915, Homo sapiens chr. 12<br>host cell factor C2	E2FF-NFKB	183 - 59	(-)	
**GXP_124395** [GXP_124395] (1 - 1049)<br>**MGC5139**, GXL_103724, GeneID: 84747, Homo sapiens chr. 12<br>hypothetical protein MGC5139	E2FF-NFKB	917 - 803	(-)	
**GXP_907301** [GXP_907301] (1 - 601)<br>**DAO**, GXL_103762, GeneID: 1610, Homo sapiens chr. 12<br>D-amino-acid oxidase	E2FF-NFKB	575 - 462	(-)	
**GXP_127714** [GXP_127714] (1 - 842)<br>**ABCB10**, GXL_106354, GeneID: 23456, Homo sapiens chr. 1<br>ATP-binding cassette, sub-family B (MDR/TAP), member 10	E2FF-NFKB	279 - 396	(+)	
**GXP_127718** [GXP_127718] (1 - 1006)<br>**HNRPU**, GXL_106356, GeneID: 3192, Homo sapiens chr. 1<br>heterogeneous nuclear ribonucleoprotein U (scaffold attachment factor A)	E2FF-NFKB	173 - 63	(-)	
**GXP_127746** [GXP_127746] (1 - 934)<br>**LIN9**, GXL_106371, GeneID: 286826, Homo sapiens chr. 1<br>lin-9 homolog (C. elegans)	E2FF-NFKB	339 - 456	(+)	
**GXP_127751** [GXP_127751] (1 - 972)<br>**SLC30A10,LOC728528,LOC730903**, GXL_106373, GeneID: 55532,728528,730903, Homo sapiens chr. 1<br>solute carrier family 30, member 10; similar to solute carrier family 30 (zinc transporter), member 10 isoform a; similar to solute carrier family 30 (zinc transporter), member 10 isoform a	E2FF-NFKB	499 - 605	(+)	

**GXP_127766** [GXP_127766] (1 - 817)<br>**VWA1,LOC727901**, GXL_106379, GeneID: 64856,727901, Homo sapiens chr. 1<br>von Willebrand factor A domain containing 1; similar to von Willebrand factor A domain-related protein isoform 1	E2FF-NFKB	258 - 132	(-)	
**GXP_127785** [GXP_127785] (1 - 674)<br>**SUSD4**, GXL_106387, GeneID: 55061, Homo sapiens chr. 1<br>sushi domain containing 4	E2FF-NFKB	6 - 134	(+)	
**GXP_127863** [GXP_127863] (1 - 651)<br>**RGS7**, GXL_106432, GeneID: 6000, Homo sapiens chr. 1<br>regulator of G-protein signalling 7	E2FF-NFKB	512 - 386	(-)	
**GXP_127870** [GXP_127870] (1 - 601)<br>**PARP1**, GXL_106436, GeneID: 142, Homo sapiens chr. 1<br>poly (ADP-ribose) polymerase family, member 1	E2FF-NFKB	413 - 536	(+)	
**GXP_127932** [GXP_127932] (1 - 601)<br>**SAMD11**, GXL_106476, GeneID: 148398, Homo sapiens chr. 1<br>sterile alpha motif domain containing 11	E2FF-NFKB	527 - 398	(-)	
**GXP_637922** [GXP_637922] (1 - 601)<br>**TP53BP2**, GXL_106560, GeneID: 7159, Homo sapiens chr. 1<br>tumor protein p53 binding protein, 2	E2FF-NFKB	202 - 80	(-)	
**GXP_137599** [GXP_137599] (1 - 916)<br>**GPR172A**, GXL_114993, GeneID: 79581, Homo sapiens chr. 8<br>G protein-coupled receptor 172A	E2FF-NFKB	662 - 771	(+)	
**GXP_926486** [GXP_926486] (1 - 949)<br>**GPR172A**, GXL_114993, GeneID: 79581, Homo sapiens chr. 8<br>G protein-coupled receptor 172A	E2FF-NFKB	126 - 235	(+)	
**GXP_137614** [GXP_137614] (1 - 1280)<br>**CYC1**, GXL_115002, GeneID: 1537, Homo sapiens chr. 8<br>cytochrome c-1	E2FF-NFKB	510 - 626	(+)	
**GXP_661506** [GXP_661506] (1 - 601)<br>**GRINA**, GXL_115013, GeneID: 2907, Homo sapiens chr. 8<br>glutamate receptor, ionotropic, N-methyl D-asparate-associated protein 1 (glutamate binding)	E2FF-NFKB	314 - 197	(-)	
**GXP_137641** [GXP_137641] (1 - 601)<br>**ZNF696**, GXL_115018, GeneID: 79943, Homo sapiens chr. 8<br>zinc finger protein 696	E2FF-NFKB	255 - 378	(+)	
**GXP_141839** [GXP_141839] (1 - 1152)<br>**SPSB4**, GXL_119089, GeneID: 92369, Homo sapiens chr. 3<br>splA/ryanodine receptor domain and SOCS box containing 4	E2FF-NFKB	269 - 390	(+)	
**GXP_141871** [GXP_141871] (1 - 1364)<br>**RNF13**, GXL_119103, GeneID: 11342, Homo sapiens chr. 3<br>ring finger protein 13	E2FF-NFKB	1325 - 1214	(-)	
	E2FF-NFKB	674 - 551	(-)	

**GXP_920541** [GXP_920541] (1 - 746)<br>**PARP14**, GXL_119106, GeneID: 54625, Homo sapiens chr. 3<br>poly (ADP-ribose) polymerase family, member 14				
**GXP_141889** [GXP_141889] (1 - 601)<br>**DTX3L**, GXL_119109, GeneID: 151636, Homo sapiens chr. 3<br>deltex 3-like (Drosophila)	E2FF-NFKB	256 - 365	(+)	
**GXP_141898** [GXP_141898] (1 - 730)<br>**SEC22A**, GXL_119116, GeneID: 26984, Homo sapiens chr. 3<br>SEC22 vesicle trafficking protein homolog A (S. cerevisiae)	E2FF-NFKB	486 - 607	(+)	
**GXP_142023** [GXP_142023] (1 - 601)<br>**GTPBP8**, GXL_119182, GeneID: 29083, Homo sapiens chr. 3<br>GTP-binding protein 8 (putative)	E2FF-NFKB	363 - 248	(-)	
**GXP_920599** [GXP_920599] (1 - 601)<br>**MCM2**, GXL_119246, GeneID: 4171, Homo sapiens chr. 3<br>MCM2 minichromosome maintenance deficient 2, mitotin (S. cerevisiae)	E2FF-NFKB	552 - 444	(-)	
**GXP_142117** [GXP_142117] (1 - 601)<br>**EPHB1**, GXL_119253, GeneID: 2047, Homo sapiens chr. 3<br>EPH receptor B1	E2FF-NFKB	423 - 317	(-)	
**GXP_142123** [GXP_142123] (1 - 646)<br>**CHST13**, GXL_119259, GeneID: 166012, Homo sapiens chr. 3<br>carbohydrate (chondroitin 4) sulfotransferase 13	E2FF-NFKB	365 - 246	(-)	
**GXP_142146** [GXP_142146] (1 - 708)<br>**UMPS**, GXL_119280, GeneID: 7372, Homo sapiens chr. 3<br>uridine monophosphate synthetase (orotate phosphoribosyl transferase and orotidine-5'-decarboxylase)	E2FF-NFKB	639 - 525	(-)	
**GXP_142488** [GXP_142488] (1 - 768)<br>**COL4A3**, GXL_119528, GeneID: 1285, Homo sapiens chr. 2<br>collagen, type IV, alpha 3 (Goodpasture antigen)	E2FF-NFKB	85 - 197	(+)	
**GXP_142524** [GXP_142524] (1 - 755)<br>**ATG16L1**, GXL_119543, GeneID: 55054, Homo sapiens chr. 2<br>ATG16 autophagy related 16-like 1 (S. cerevisiae)	E2FF-NFKB	677 - 556	(-)	
**GXP_142556** [GXP_142556] (1 - 901)<br>**BCS1L**, GXL_119560, GeneID: 617, Homo sapiens chr. 2<br>BCS1-like (yeast)	E2FF-NFKB	380 - 272	(-)	
**GXP_919436** [GXP_919436] (1 - 1190)<br>**BCS1L**, GXL_119560, GeneID: 617, Homo sapiens chr. 2<br>BCS1-like (yeast)	E2FF-NFKB	669 - 561	(-)	
**GXP_142557** [GXP_142557] (1 - 1466)<br>**NRP2**, GXL_119561, GeneID: 8828, Homo sapiens chr. 2<br>neuropilin 2	E2FF-NFKB	922 - 804	(-)	
	E2FF-NFKB	620 - 732	(+)	

**GXP_142573** [GXP_142573] (1 - 937)<br>**CTDSP1**, GXL_119568, GeneID: 58190, Homo sapiens chr. 2<br>CTD (carboxy-terminal domain, RNA polymerase II, polypeptide A) small phosphatase 1	E2FF-NFKB	833 - 719	(-)	
**GXP_142628** [GXP_142628] (1 - 691)<br>**STK36**, GXL_119596, GeneID: 27148, Homo sapiens chr. 2<br>serine/threonine kinase 36, fused homolog (Drosophila)	E2FF-NFKB	491 - 612	(+)	
**GXP_919337** [GXP_919337] (1 - 601)<br>**CCNYL1**, GXL_119597, GeneID: 151195, Homo sapiens chr. 2<br>cyclin Y-like 1	E2FF-NFKB	461 - 574	(+)	
**GXP_651613** [GXP_651613] (1 - 797)<br>**SCLY,UBE2F**, GXL_119608, GeneID: 51540,140739, Homo sapiens chr. 2<br>selenocysteine lyase; ubiquitin-conjugating enzyme E2F (putative)	E2FF-NFKB	233 - 114	(-)	
**GXP_142718** [GXP_142718] (1 - 1005)<br>**STK11IP**, GXL_119650, GeneID: 114790, Homo sapiens chr. 2<br>serine/threonine kinase 11 interacting protein	E2FF-NFKB	785 - 913	(+)	
**GXP_142719** [GXP_142719] (1 - 601)<br>**STK11IP**, GXL_119650, GeneID: 114790, Homo sapiens chr. 2<br>serine/threonine kinase 11 interacting protein	E2FF-NFKB	381 - 509	(+)	
**GXP_142742** [GXP_142742] (1 - 757)<br>**PNKD,LOC646532**, GXL_119669, GeneID: 25953,646532, Homo sapiens chr. 2<br>paroxysmal nonkinesiogenic dyskinesia; similar to ribosomal protein L19	E2FF-NFKB	192 - 314	(+)	
**GXP_919559** [GXP_919559] (1 - 696)<br>**DIS3L2,LOC730069,LOC731484**, GXL_119688, GeneID: 129563,730069,731484, Homo sapiens chr. 2<br>DIS3 mitotic control homolog (S. cerevisiae)-like 2; similar to nuclear receptor binding factor 2; similar to nuclear receptor binding factor 2	E2FF-NFKB	521 - 644	(+)	
**GXP_919467** [GXP_919467] (1 - 601)<br>**ACCN4**, GXL_119717, GeneID: 55515, Homo sapiens chr. 2<br>amiloride-sensitive cation channel 4, pituitary	E2FF-NFKB	170 - 51	(-)	
**GXP_142810** [GXP_142810] (1 - 601)<br>**INPP5D**, GXL_119724, GeneID: 3635, Homo sapiens chr. 2<br>inositol polyphosphate-5-phosphatase, 145kDa	E2FF-NFKB	4 - 115	(+)	
**GXP_637451** [GXP_637451] (1 - 607)<br>**CEP350**, GXL_120764, GeneID: 9857, Homo sapiens chr. 1<br>centrosomal protein 350kDa	E2FF-NFKB	126 - 7	(-)	
**GXP_144245** [GXP_144245] (1 - 835)<br>**ATF3**, GXL_120767, GeneID: 467, Homo sapiens chr. 1<br>activating transcription factor 3	E2FF-NFKB	188 - 67	(-)	
**GXP_915927** [GXP_915927] (1 - 601)<br>**XPR1**, GXL_120793, GeneID: 9213, Homo sapiens chr. 1<br>xenotropic and polytropic retrovirus receptor	E2FF-NFKB	152 - 25	(-)	
	E2FF-NFKB	304 - 413	(+)	

**GXP_144305** [GXP_144305] (1 - 633)<br>**SMG7**, GXL_120798, GeneID: 9887, Homo sapiens chr. 1<br>Smg-7 homolog, nonsense mediated mRNA decay factor (C. elegans)				
**GXP_915890** [GXP_915890] (1 - 601)<br>**FAM5B**, GXL_120860, GeneID: 57795, Homo sapiens chr. 1<br>family with sequence similarity 5, member B	E2FF-NFKB	236 - 121	(-)	
**GXP_144447** [GXP_144447] (1 - 601)<br>**C1orf9**, GXL_120879, GeneID: 51430, Homo sapiens chr. 1<br>chromosome 1 open reading frame 9	E2FF-NFKB	265 - 150	(-)	
**GXP_144471** [GXP_144471] (1 - 866)<br>**KCTD3**, GXL_120896, GeneID: 51133, Homo sapiens chr. 1<br>potassium channel tetramerisation domain containing 3	E2FF-NFKB	550 - 664	(+)	
**GXP_144505** [GXP_144505] (1 - 601)<br>**PROX1**, GXL_120916, GeneID: 5629, Homo sapiens chr. 1<br>prospero-related homeobox 1	E2FF-NFKB	313 - 437	(+)	
**GXP_916201** [GXP_916201] (1 - 601)<br>**PROX1**, GXL_120916, GeneID: 5629, Homo sapiens chr. 1<br>prospero-related homeobox 1	E2FF-NFKB	391 - 518	(+)	
**GXP_916202** [GXP_916202] (1 - 652)<br>**PROX1**, GXL_120916, GeneID: 5629, Homo sapiens chr. 1<br>prospero-related homeobox 1	E2FF-NFKB	249 - 126	(-)	
**GXP_144528** [GXP_144528] (1 - 694)<br>**KLHL20**, GXL_120931, GeneID: 27252, Homo sapiens chr. 1<br>kelch-like 20 (Drosophila)	E2FF-NFKB	280 - 388	(+)	
**GXP_915870** [GXP_915870] (1 - 667)<br>**KLHL20**, GXL_120931, GeneID: 27252, Homo sapiens chr. 1<br>kelch-like 20 (Drosophila)	E2FF-NFKB	20 - 128	(+)	
**GXP_915871** [GXP_915871] (1 - 601)<br>**KLHL20**, GXL_120931, GeneID: 27252, Homo sapiens chr. 1<br>kelch-like 20 (Drosophila)	E2FF-NFKB	375 - 247	(-)	
**GXP_144535** [GXP_144535] (1 - 720)<br>**CTSE**, GXL_120937, GeneID: 1510, Homo sapiens chr. 1<br>cathepsin E	E2FF-NFKB	334 - 452	(+)	
**GXP_915907** [GXP_915907] (1 - 601)<br>**SOAT1**, GXL_120944, GeneID: 6646, Homo sapiens chr. 1<br>sterol O-acyltransferase (acyl-Coenzyme A: cholesterol acyltransferase) 1	E2FF-NFKB	380 - 274	(-)	
	E2FF-NFKB	440 - 561	(+)	
**GXP_144614** [GXP_144614] (1 - 663)<br>**FAM20B**, GXL_121007, GeneID: 9917, Homo sapiens chr. 1<br>family with sequence similarity 20, member B	E2FF-NFKB	617 - 493	(-)	
	E2FF-NFKB	206 - 316	(+)	

**GXP_478795** [GXP_478795] (1 - 601)<br>**PFKFB2**, GXL_121008, GeneID: 5208, Homo sapiens chr. 1<br>6-phosphofructo-2-kinase/fructose-2,6-biphosphatase 2				
**GXP_651763** [GXP_651763] (1 - 671)<br>**C20orf27**, GXL_122526, GeneID: 54976, Homo sapiens chr. 20<br>chromosome 20 open reading frame 27	E2FF-NFKB	368 - 478	(+)	
**GXP_146653** [GXP_146653] (1 - 635)<br>**SDCBP2**, GXL_122531, GeneID: 27111, Homo sapiens chr. 20<br>syndecan binding protein (syntenin) 2	E2FF-NFKB	497 - 383	(-)	
**GXP_146672** [GXP_146672] (1 - 1289)<br>**ADAM33**, GXL_122541, GeneID: 80332, Homo sapiens chr. 20<br>ADAM metallopeptidase domain 33	E2FF-NFKB	978 - 1085	(+)	
**GXP_146705** [GXP_146705] (1 - 772)<br>**C20orf44**, GXL_122558, GeneID: 55245, Homo sapiens chr. 20<br>chromosome 20 open reading frame 44	E2FF-NFKB	535 - 651	(+)	
**GXP_146732** [GXP_146732] (1 - 1381)<br>**FAM113A**, GXL_122570, GeneID: 64773, Homo sapiens chr. 20<br>family with sequence similarity 113, member A	E2FF-NFKB	605 - 478	(-)	
**GXP_916562** [GXP_916562] (1 - 601)<br>**RASSF2**, GXL_122603, GeneID: 9770, Homo sapiens chr. 20<br>Ras association (RalGDS/AF-6) domain family 2	E2FF-NFKB	401 - 510	(+)	
**GXP_916747** [GXP_916747] (1 - 601)<br>**APBA2BP**, GXL_122632, GeneID: 63941, Homo sapiens chr. 20<br>amyloid beta (A4) precursor protein-binding, family A, member 2 binding protein	E2FF-NFKB	259 - 136	(-)	
**GXP_146863** [GXP_146863] (1 - 1131)<br>**NANP**, GXL_122645, GeneID: 140838, Homo sapiens chr. 20<br>N-acetylneuraminic acid phosphatase	E2FF-NFKB	1043 - 921	(-)	
**GXP_146970** [GXP_146970] (1 - 725)<br>**E2F1**, GXL_122737, GeneID: 1869, Homo sapiens chr. 20<br>E2F transcription factor 1	E2FF-NFKB	365 - 242	(-)	
**GXP_147538** [GXP_147538] (1 - 849)<br>**MYOD1**, GXL_123189, GeneID: 4654, Homo sapiens chr. 11<br>myogenic differentiation 1	E2FF-NFKB	316 - 438	(+)	
**GXP_147554** [GXP_147554] (1 - 906)<br>**PPFIBP2**, GXL_123200, GeneID: 8495, Homo sapiens chr. 11<br>PTPRF interacting protein, binding protein 2 (liprin beta 2)	E2FF-NFKB	334 - 460	(+)	
**GXP_905002** [GXP_905002] (1 - 870)<br>**TUB**, GXL_123209, GeneID: 7275, Homo sapiens chr. 11<br>tubby homolog (mouse)	E2FF-NFKB	346 - 472	(+)	
	E2FF-NFKB	508 - 634	(+)	

**GXP_147578** [GXP_147578] (1 - 839)<br>**SYT9**, GXL_123214, GeneID: 143425, Homo sapiens chr. 11<br>synaptotagmin IX				
**GXP_147579** [GXP_147579] (1 - 601)<br>**SYT9**, GXL_123214, GeneID: 143425, Homo sapiens chr. 11<br>synaptotagmin IX	E2FF-NFKB	310 - 196	(-)	
**GXP_905089** [GXP_905089] (1 - 634)<br>**NUCB2**, GXL_123219, GeneID: 4925, Homo sapiens chr. 11<br>nucleobindin 2	E2FF-NFKB	373 - 499	(+)	
**GXP_147628** [GXP_147628] (1 - 602)<br>**DNHD1,LOC728714,LOC731960**, GXL_123245, GeneID: 144132,728714,731960, Homo sapiens chr. 11<br>dynein heavy chain domain 1; hypothetical protein LOC728714; hypothetical protein LOC731960	E2FF-NFKB	410 - 288	(-)	
**GXP_904984** [GXP_904984] (1 - 1002)<br>**DNHD1,LOC728714,LOC731960**, GXL_123245, GeneID: 144132,728714,731960, Homo sapiens chr. 11<br>dynein heavy chain domain 1; hypothetical protein LOC728714; hypothetical protein LOC731960	E2FF-NFKB	135 - 13	(-)	
**GXP_147661** [GXP_147661] (1 - 674)<br>**PARVA**, GXL_123270, GeneID: 55742, Homo sapiens chr. 11<br>parvin, alpha	E2FF-NFKB	248 - 373	(+)	
**GXP_906261** [GXP_906261] (1 - 832)<br>**IGSF9B**, GXL_123285, GeneID: 22997, Homo sapiens chr. 11<br>immunoglobulin superfamily, member 9B	E2FF-NFKB	561 - 447	(-)	
**GXP_904858** [GXP_904858] (1 - 601)<br>**EFCAB4A**, GXL_123314, GeneID: 283229, Homo sapiens chr. 11<br>EF-hand calcium binding domain 4A	E2FF-NFKB	586 - 467	(-)	
**GXP_639672** [GXP_639672] (1 - 601)<br>**SLC6A5**, GXL_123317, GeneID: 9152, Homo sapiens chr. 11<br>solute carrier family 6 (neurotransmitter transporter, glycine), member 5	E2FF-NFKB	5 - 123	(+)	
**GXP_147722** [GXP_147722] (1 - 601)<br>**P53AIP1**, GXL_123323, GeneID: 63970, Homo sapiens chr. 11<br>p53-regulated apoptosis-inducing protein 1	E2FF-NFKB	10 - 139	(+)	
**GXP_640802** [GXP_640802] (1 - 601)<br>**FEZ1**, GXL_123336, GeneID: 9638, Homo sapiens chr. 11<br>fasciculation and elongation protein zeta 1 (zygin I)	E2FF-NFKB	357 - 239	(-)	
**GXP_147743** [GXP_147743] (1 - 827)<br>**SLC17A6**, GXL_123342, GeneID: 57084, Homo sapiens chr. 11<br>solute carrier family 17 (sodium-dependent inorganic phosphate cotransporter), member 6	E2FF-NFKB	708 - 822	(+)	
**GXP_904857** [GXP_904857] (1 - 769)<br>**PNPLA2**, GXL_123358, GeneID: 57104, Homo sapiens chr. 11<br>patatin-like phospholipase domain containing 2	E2FF-NFKB	684 - 565	(-)	
	E2FF-NFKB	589 - 463	(-)	

**GXP_147776** [GXP_147776] (1 - 616)<br>**AMPD3**, GXL_123372, GeneID: 272, Homo sapiens chr. 11<br>adenosine monophosphate deaminase (isoform E)				
**GXP_639350** [GXP_639350] (1 - 601)<br>**PTDSS2**, GXL_123376, GeneID: 81490, Homo sapiens chr. 11<br>phosphatidylserine synthase 2	E2FF-NFKB	228 - 347	(+)	
**GXP_921256** [GXP_921256] (1 - 601)<br>**PPP2R2C**, GXL_125056, GeneID: 5522, Homo sapiens chr. 4<br>protein phosphatase 2 (formerly 2A), regulatory subunit B, gamma isoform	E2FF-NFKB	227 - 119	(-)	
**GXP_921422** [GXP_921422] (1 - 601)<br>**FLJ20273**, GXL_125069, GeneID: 54502, Homo sapiens chr. 4<br>RNA-binding protein	E2FF-NFKB	118 - 7	(-)	
**GXP_921423** [GXP_921423] (1 - 758)<br>**FLJ20273**, GXL_125069, GeneID: 54502, Homo sapiens chr. 4<br>RNA-binding protein	E2FF-NFKB	588 - 477	(-)	
**GXP_149812** [GXP_149812] (1 - 601)<br>**GAK**, GXL_125078, GeneID: 2580, Homo sapiens chr. 4<br>cyclin G associated kinase	E2FF-NFKB	400 - 281	(-)	
**GXP_149813** [GXP_149813] (1 - 804)<br>**GAK**, GXL_125078, GeneID: 2580, Homo sapiens chr. 4<br>cyclin G associated kinase	E2FF-NFKB	174 - 296	(+)	
**GXP_921369** [GXP_921369] (1 - 601)<br>**CENTD1**, GXL_125117, GeneID: 116984, Homo sapiens chr. 4<br>centaurin, delta 1	E2FF-NFKB	502 - 385	(-)	
**GXP_149911** [GXP_149911] (1 - 601)<br>**HOP**, GXL_125132, GeneID: 84525, Homo sapiens chr. 4<br>homeodomain-only protein	E2FF-NFKB	443 - 567	(+)	
**GXP_149921** [GXP_149921] (1 - 1019)<br>**GRSF1**, GXL_125138, GeneID: 2926, Homo sapiens chr. 4<br>G-rich RNA sequence binding factor 1	E2FF-NFKB	92 - 206	(+)	
**GXP_149935** [GXP_149935] (1 - 610)<br>**EVC2**, GXL_125146, GeneID: 132884, Homo sapiens chr. 4<br>Ellis van Creveld syndrome 2 (limbin)	E2FF-NFKB	398 - 508	(+)	
**GXP_921318** [GXP_921318] (1 - 633)<br>**TAPT1**, GXL_125150, GeneID: 202018, Homo sapiens chr. 4<br>transmembrane anterior posterior transformation 1	E2FF-NFKB	533 - 414	(-)	
**GXP_921319** [GXP_921319] (1 - 601)<br>**TAPT1**, GXL_125150, GeneID: 202018, Homo sapiens chr. 4<br>transmembrane anterior posterior transformation 1	E2FF-NFKB	6 - 124	(+)	
	E2FF-NFKB	521 - 406	(-)	

**GXP_149971** [GXP_149971] (1 - 711)<br>**COX18**, GXL_125168, GeneID: 285521, Homo sapiens chr. 4<br>COX18 cytochrome c oxidase assembly homolog (S. cerevisiae)				
**GXP_149972** [GXP_149972] (1 - 749)<br>**LYAR**, GXL_125169, GeneID: 55646, Homo sapiens chr. 4<br>hypothetical protein FLJ20425	E2FF-NFKB	71 - 184	(+)	
**GXP_149979** [GXP_149979] (1 - 709)<br>**G3BP2**, GXL_125174, GeneID: 9908, Homo sapiens chr. 4<br>GTPase activating protein (SH3 domain) binding protein 2	E2FF-NFKB	146 - 270	(+)	
**GXP_149980** [GXP_149980] (1 - 807)<br>**G3BP2**, GXL_125174, GeneID: 9908, Homo sapiens chr. 4<br>GTPase activating protein (SH3 domain) binding protein 2	E2FF-NFKB	494 - 618	(+)	
**GXP_921292** [GXP_921292] (1 - 833)<br>**WDR1**, GXL_125176, GeneID: 9948, Homo sapiens chr. 4<br>WD repeat domain 1	E2FF-NFKB	518 - 399	(-)	
**GXP_150042** [GXP_150042] (1 - 706)<br>**CXCL10**, GXL_125222, GeneID: 3627, Homo sapiens chr. 4<br>chemokine (C-X-C motif) ligand 10	E2FF-NFKB	483 - 595	(+)	
**GXP_150055** [GXP_150055] (1 - 601)<br>**FLJ46481**, GXL_125235, GeneID: 389197, Homo sapiens chr. 4<br>FLJ46481 protein	E2FF-NFKB	80 - 207	(+)	
**GXP_150083** [GXP_150083] (1 - 601)<br>**FLJ45721**, GXL_125258, GeneID: 401123, Homo sapiens chr. 4<br>FLJ45721 protein	E2FF-NFKB	19 - 126	(+)	
**GXP_150088** [GXP_150088] (1 - 705)<br>**CXCL9**, GXL_125262, GeneID: 4283, Homo sapiens chr. 4<br>chemokine (C-X-C motif) ligand 9	E2FF-NFKB	277 - 400	(+)	
**GXP_150100** [GXP_150100] (1 - 796)<br>**CXCL3**, GXL_125273, GeneID: 2921, Homo sapiens chr. 4<br>chemokine (C-X-C motif) ligand 3	E2FF-NFKB	663 - 536	(-)	
**GXP_150115** [GXP_150115] (1 - 799)<br>**CXCL5**, GXL_125287, GeneID: 6374, Homo sapiens chr. 4<br>chemokine (C-X-C motif) ligand 5	E2FF-NFKB	636 - 509	(-)	
**GXP_921379** [GXP_921379] (1 - 601)<br>**RELL1**, GXL_125293, GeneID: 768211, Homo sapiens chr. 4<br>receptor expressed in lymphoid tissues like 1	E2FF-NFKB	224 - 106	(-)	
**GXP_153648** [GXP_153648] (1 - 1239)<br>**PLTP**, GXL_128253, GeneID: 5360, Homo sapiens chr. 20<br>phospholipid transfer protein	E2FF-NFKB	1073 - 1179	(+)	
	E2FF-NFKB	281 - 406	(+)	

**GXP_916893** [GXP_916893] (1 - 601)<br>**PLTP**, GXL_128253, GeneID: 5360, Homo sapiens chr. 20<br>phospholipid transfer protein				
**GXP_916887** [GXP_916887] (1 - 647)<br>**WFDC3**, GXL_128254, GeneID: 140686, Homo sapiens chr. 20<br>WAP four-disulfide core domain 3	E2FF-NFKB	638 - 528	(-)	
**GXP_153658** [GXP_153658] (1 - 1283)<br>**STMN3**, GXL_128257, GeneID: 50861, Homo sapiens chr. 20<br>stathmin-like 3	E2FF-NFKB	653 - 526	(-)	
**GXP_153660** [GXP_153660] (1 - 1121)<br>**NEURL2**, GXL_128258, GeneID: 140825, Homo sapiens chr. 20<br>neuralized homolog 2 (Drosophila)	E2FF-NFKB	152 - 29	(-)	
**GXP_153661** [GXP_153661] (1 - 601)<br>**ZNF217**, GXL_128259, GeneID: 7764, Homo sapiens chr. 20<br>zinc finger protein 217	E2FF-NFKB	340 - 446	(+)	
**GXP_153703** [GXP_153703] (1 - 1435)<br>**JPH2**, GXL_128285, GeneID: 57158, Homo sapiens chr. 20<br>junctophilin 2	E2FF-NFKB	875 - 985	(+)	
**GXP_153717** [GXP_153717] (1 - 601)<br>**MATN4**, GXL_128291, GeneID: 8785, Homo sapiens chr. 20<br>matrilin 4	E2FF-NFKB	350 - 224	(-)	
**GXP_153721** [GXP_153721] (1 - 712)<br>**CTCFL**, GXL_128293, GeneID: 140690, Homo sapiens chr. 20<br>CCCTC-binding factor (zinc finger protein)-like	E2FF-NFKB	571 - 700	(+)	
**GXP_153795** [GXP_153795] (1 - 1404)<br>**CDC25B**, GXL_128335, GeneID: 994, Homo sapiens chr. 20<br>cell division cycle 25 homolog B (S. pombe)	E2FF-NFKB	833 - 946	(+)	
**GXP_153798** [GXP_153798] (1 - 893)<br>**CDS2**, GXL_128337, GeneID: 8760, Homo sapiens chr. 20<br>CDP-diacylglycerol synthase (phosphatidate cytidylyltransferase) 2	E2FF-NFKB	317 - 201	(-)	
**GXP_916531** [GXP_916531] (1 - 601)<br>**GNRH2**, GXL_128341, GeneID: 2797, Homo sapiens chr. 20<br>gonadotropin-releasing hormone 2	E2FF-NFKB	73 - 194	(+)	
**GXP_153820** [GXP_153820] (1 - 643)<br>**SDC4**, GXL_128353, GeneID: 6385, Homo sapiens chr. 20<br>syndecan 4	E2FF-NFKB	532 - 409	(-)	
**GXP_153840** [GXP_153840] (1 - 894)<br>**RIMS4**, GXL_128366, GeneID: 140730, Homo sapiens chr. 20<br>regulating synaptic membrane exocytosis 4	E2FF-NFKB	173 - 288	(+)	
	E2FF-NFKB	438 - 320	(-)	

**GXP_153857** [GXP_153857] (1 - 628)<br>**SLC35C2**, GXL_128377, GeneID: 51006, Homo sapiens chr. 20<br>solute carrier family 35, member C2				
**GXP_153868** [GXP_153868] (1 - 601)<br>**PTPRA,VPS16**, GXL_128383, GeneID: 5786,64601, Homo sapiens chr. 20<br>protein tyrosine phosphatase, receptor type, A; vacuolar protein sorting 16 homolog (S. cerevisiae)	E2FF-NFKB	361 - 488	(+)	
**GXP_153883** [GXP_153883] (1 - 754)<br>**C20orf111**, GXL_128395, GeneID: 51526, Homo sapiens chr. 20<br>chromosome 20 open reading frame 111	E2FF-NFKB	592 - 699	(+)	
**GXP_916863** [GXP_916863] (1 - 1141)<br>**C20orf111**, GXL_128395, GeneID: 51526, Homo sapiens chr. 20<br>chromosome 20 open reading frame 111	E2FF-NFKB	307 - 414	(+)	
**GXP_153897** [GXP_153897] (1 - 602)<br>**WFDC12**, GXL_128409, GeneID: 128488, Homo sapiens chr. 20<br>WAP four-disulfide core domain 12	E2FF-NFKB	253 - 365	(+)	
**GXP_916517** [GXP_916517] (1 - 601)<br>**TMC2**, GXL_128419, GeneID: 117532, Homo sapiens chr. 20<br>transmembrane channel-like 2	E2FF-NFKB	280 - 158	(-)	
**GXP_153927** [GXP_153927] (1 - 774)<br>**SAMD10**, GXL_128428, GeneID: 140700, Homo sapiens chr. 20<br>sterile alpha motif domain containing 10	E2FF-NFKB	497 - 372	(-)	
**GXP_917065** [GXP_917065] (1 - 877)<br>**GM632**, GXL_128439, GeneID: 57473, Homo sapiens chr. 20<br>KIAA1196 protein	E2FF-NFKB	660 - 535	(-)	
**GXP_158111** [GXP_158111] (1 - 687)<br>**USP39**, GXL_131845, GeneID: 10713, Homo sapiens chr. 2<br>ubiquitin specific peptidase 39	E2FF-NFKB	160 - 276	(+)	
**GXP_158207** [GXP_158207] (1 - 1270)<br>**DOK1**, GXL_131894, GeneID: 1796, Homo sapiens chr. 2<br>docking protein 1, 62kDa (downstream of tyrosine kinase 1)	E2FF-NFKB	1076 - 1197	(+)	
**GXP_158311** [GXP_158311] (1 - 743)<br>**C2orf40**, GXL_131963, GeneID: 84417, Homo sapiens chr. 2<br>chromosome 2 open reading frame 40	E2FF-NFKB	604 - 498	(-)	
**GXP_158346** [GXP_158346] (1 - 602)<br>**NOTO**, GXL_131989, GeneID: 344022, Homo sapiens chr. 2<br>notochord homeobox	E2FF-NFKB	19 - 133	(+)	
**GXP_158355** [GXP_158355] (1 - 799)<br>**FLJ37440**, GXL_131995, GeneID: 129804, Homo sapiens chr. 2<br>hypothetical protein FLJ37440	E2FF-NFKB	527 - 633	(+)	
	E2FF-NFKB	286 - 402	(+)	

**GXP_158382** [GXP_158382] (1 - 804)<br>**PCBP1**, GXL_132013, GeneID: 5093, Homo sapiens chr. 2<br>poly(rC) binding protein 1				
**GXP_158434** [GXP_158434] (1 - 1022)<br>**POLE4**, GXL_132057, GeneID: 56655, Homo sapiens chr. 2<br>polymerase (DNA-directed), epsilon 4 (p12 subunit)	E2FF-NFKB	863 - 974	(+)	
**GXP_158461** [GXP_158461] (1 - 1168)<br>**HTRA2**, GXL_132082, GeneID: 27429, Homo sapiens chr. 2<br>HtrA serine peptidase 2	E2FF-NFKB	617 - 726	(+)	
**GXP_159192** [GXP_159192] (1 - 908)<br>**SNRPB2**, GXL_132624, GeneID: 6629, Homo sapiens chr. 20<br>small nuclear ribonucleoprotein polypeptide B''	E2FF-NFKB	686 - 799	(+)	
**GXP_651831** [GXP_651831] (1 - 601)<br>**SNRPB2**, GXL_132624, GeneID: 6629, Homo sapiens chr. 20<br>small nuclear ribonucleoprotein polypeptide B''	E2FF-NFKB	422 - 540	(+)	
**GXP_159234** [GXP_159234] (1 - 669)<br>**C20orf26**, GXL_132653, GeneID: 26074, Homo sapiens chr. 20<br>chromosome 20 open reading frame 26	E2FF-NFKB	143 - 264	(+)	
**GXP_159243** [GXP_159243] (1 - 824)<br>**RIN2**, GXL_132659, GeneID: 54453, Homo sapiens chr. 20<br>Ras and Rab interactor 2	E2FF-NFKB	202 - 330	(+)	
**GXP_328794** [GXP_328794] (1 - 601)<br>**NCOA3**, GXL_132680, GeneID: 8202, Homo sapiens chr. 20<br>nuclear receptor coactivator 3	E2FF-NFKB	306 - 415	(+)	
**GXP_651931** [GXP_651931] (1 - 601)<br>**ASXL1**, GXL_132694, GeneID: 171023, Homo sapiens chr. 20<br>additional sex combs like 1 (Drosophila)	E2FF-NFKB	566 - 457	(-)	
**GXP_916701** [GXP_916701] (1 - 601)<br>**HM13**, GXL_132720, GeneID: 81502, Homo sapiens chr. 20<br>histocompatibility (minor) 13	E2FF-NFKB	255 - 145	(-)	
**GXP_159352** [GXP_159352] (1 - 774)<br>**CD40**, GXL_132729, GeneID: 958, Homo sapiens chr. 20<br>CD40 molecule, TNF receptor superfamily member 5	E2FF-NFKB	129 - 235	(+)	
**GXP_159357** [GXP_159357] (1 - 764)<br>**LBP**, GXL_132734, GeneID: 3929, Homo sapiens chr. 20<br>lipopolysaccharide binding protein	E2FF-NFKB	456 - 579	(+)	
**GXP_916622** [GXP_916622] (1 - 601)<br>**PCSK2**, GXL_132737, GeneID: 5126, Homo sapiens chr. 20<br>proprotein convertase subtilisin/kexin type 2	E2FF-NFKB	449 - 332	(-)	
	E2FF-NFKB	356 - 478	(+)	

**GXP_916682** [GXP_916682] (1 - 601)<br>**ENTPD6**, GXL_132743, GeneID: 955, Homo sapiens chr. 20<br>ectonucleoside triphosphate diphosphohydrolase 6 (putative function)				
**GXP_159418** [GXP_159418] (1 - 626)<br>**SNAI1**, GXL_132778, GeneID: 6615, Homo sapiens chr. 20<br>snail homolog 1 (Drosophila)	E2FF-NFKB	409 - 295	(-)	
**GXP_396914** [GXP_396914] (1 - 601)<br>**C20orf82**, GXL_132779, GeneID: 140862, Homo sapiens chr. 20<br>chromosome 20 open reading frame 82	E2FF-NFKB	448 - 560	(+)	
**GXP_159437** [GXP_159437] (1 - 687)<br>**SLC24A3**, GXL_132794, GeneID: 57419, Homo sapiens chr. 20<br>solute carrier family 24 (sodium/potassium/calcium exchanger), member 3	E2FF-NFKB	414 - 285	(-)	
**GXP_916889** [GXP_916889] (1 - 601)<br>**DNTTIP1**, GXL_132825, GeneID: 116092, Homo sapiens chr. 20<br>deoxynucleotidyltransferase, terminal, interacting protein 1	E2FF-NFKB	335 - 457	(+)	
**GXP_160293** [GXP_160293] (1 - 657)<br>**MPHOSPH6**, GXL_133427, GeneID: 10200, Homo sapiens chr. 16<br>M-phase phosphoprotein 6	E2FF-NFKB	340 - 217	(-)	
**GXP_160334** [GXP_160334] (1 - 764)<br>**PLLP**, GXL_133449, GeneID: 51090, Homo sapiens chr. 16<br>plasma membrane proteolipid (plasmolipin)	E2FF-NFKB	269 - 150	(-)	
**GXP_160340** [GXP_160340] (1 - 601)<br>**FLJ11171**, GXL_133453, GeneID: 55783, Homo sapiens chr. 16<br>hypothetical protein FLJ11171	E2FF-NFKB	356 - 478	(+)	
**GXP_910535** [GXP_910535] (1 - 609)<br>**SLC12A4**, GXL_133455, GeneID: 6560, Homo sapiens chr. 16<br>solute carrier family 12 (potassium/chloride transporters), member 4	E2FF-NFKB	250 - 125	(-)	
**GXP_160373** [GXP_160373] (1 - 669)<br>**CGI-38**, GXL_133472, GeneID: 51673, Homo sapiens chr. 16<br>brain specific protein	E2FF-NFKB	354 - 468	(+)	
**GXP_160406** [GXP_160406] (1 - 601)<br>**LRRC29**, GXL_133488, GeneID: 26231, Homo sapiens chr. 16<br>leucine rich repeat containing 29	E2FF-NFKB	349 - 222	(-)	
**GXP_160437** [GXP_160437] (1 - 601)<br>**CD2BP2**, GXL_133506, GeneID: 10421, Homo sapiens chr. 16<br>CD2 (cytoplasmic tail) binding protein 2	E2FF-NFKB	382 - 490	(+)	
**GXP_910218** [GXP_910218] (1 - 853)<br>**CD2BP2**, GXL_133506, GeneID: 10421, Homo sapiens chr. 16<br>CD2 (cytoplasmic tail) binding protein 2	E2FF-NFKB	8 - 116	(+)	
	E2FF-NFKB	187 - 304	(+)	

**GXP_160443** [GXP_160443] (1 - 601)<br>**MAPK3**, GXL_133509, GeneID: 5595, Homo sapiens chr. 16<br>mitogen-activated protein kinase 3				
**GXP_160463** [GXP_160463] (1 - 949)<br>**FA2H**, GXL_133522, GeneID: 79152, Homo sapiens chr. 16<br>fatty acid 2-hydroxylase	E2FF-NFKB	594 - 702	(+)	
**GXP_160480** [GXP_160480] (1 - 911)<br>**PYDC1**, GXL_133531, GeneID: 260434, Homo sapiens chr. 16<br>PYD (pyrin domain) containing 1	E2FF-NFKB<br>E2FF-NFKB	644 - 773<br>657 - 543	(+)<br>(-)	
**GXP_160506** [GXP_160506] (1 - 725)<br>**ADAMTS18**, GXL_133551, GeneID: 170692, Homo sapiens chr. 16<br>ADAM metallopeptidase with thrombospondin type 1 motif, 18	E2FF-NFKB	527 - 654	(+)	
**GXP_160507** [GXP_160507] (1 - 601)<br>**ADAMTS18**, GXL_133551, GeneID: 170692, Homo sapiens chr. 16<br>ADAM metallopeptidase with thrombospondin type 1 motif, 18	E2FF-NFKB<br>E2FF-NFKB	51 - 176<br>271 - 163	(+)<br>(-)	
**GXP_160547** [GXP_160547] (1 - 876)<br>**PRSS36**, GXL_133572, GeneID: 146547, Homo sapiens chr. 16<br>protease, serine, 36	E2FF-NFKB	778 - 668	(-)	
**GXP_910258** [GXP_910258] (1 - 601)<br>**PRSS36**, GXL_133572, GeneID: 146547, Homo sapiens chr. 16<br>protease, serine, 36	E2FF-NFKB	265 - 139	(-)	
**GXP_910603** [GXP_910603] (1 - 601)<br>**ABBA-1**, GXL_133607, GeneID: 92154, Homo sapiens chr. 16<br>actin-bundling protein with BAIAP2 homology	E2FF-NFKB	34 - 152	(+)	
**GXP_160598** [GXP_160598] (1 - 952)<br>**ABCC11,LOC727881,LOC731079**, GXL_133609, GeneID: 85320,727881,731079, Homo sapiens chr. 16<br>ATP-binding cassette, sub-family C (CFTR/MRP), member 11;hypothetical protein LOC727881; hypothetical protein LOC731079	E2FF-NFKB	293 - 401	(+)	
**GXP_645213** [GXP_645213] (1 - 602)<br>**ZNF629**, GXL_133638, GeneID: 23361, Homo sapiens chr. 16<br>zinc finger protein 629	E2FF-NFKB	217 - 327	(+)	
**GXP_160633** [GXP_160633] (1 - 714)<br>**FHOD1**, GXL_133639, GeneID: 29109, Homo sapiens chr. 16<br>formin homology 2 domain containing 1	E2FF-NFKB	441 - 330	(-)	
**GXP_161335** [GXP_161335] (1 - 836)<br>**CASP7**, GXL_134154, GeneID: 840, Homo sapiens chr. 10<br>caspase 7, apoptosis-related cysteine peptidase	E2FF-NFKB<br>E2FF-NFKB	326 - 454<br>740 - 613	(+)<br>(-)	
**GXP_161369** [GXP_161369] (1 - 698)<br>**SFXN2**, GXL_134172, GeneID: 118980, Homo sapiens chr. 10<br>sideroflexin 2	E2FF-NFKB<br>E2FF-NFKB	393 - 515<br>611 - 501	(+)<br>(-)	
	E2FF-NFKB	58 - 178	(+)	

**GXP_161371** [GXP_161371] (1 - 1220)<br>**SLK**, GXL_134174, GeneID: 9748, Homo sapiens chr. 10<br>STE20-like kinase (yeast)				
**GXP_904591** [GXP_904591] (1 - 601)<br>**SLK**, GXL_134174, GeneID: 9748, Homo sapiens chr. 10<br>STE20-like kinase (yeast)	E2FF-NFKB	325 - 445	(+)	
**GXP_904804** [GXP_904804] (1 - 637)<br>**MTG1**, GXL_134176, GeneID: 92170, Homo sapiens chr. 10<br>mitochondrial GTPase 1 homolog (S. cerevisiae)	E2FF-NFKB	366 - 253	(-)	
**GXP_161407** [GXP_161407] (1 - 663)<br>**PAOX**, GXL_134193, GeneID: 196743, Homo sapiens chr. 10<br>polyamine oxidase (exo-N4-amino)	E2FF-NFKB	333 - 220	(-)	
**GXP_161417** [GXP_161417] (1 - 601)<br>**C10orf78**, GXL_134197, GeneID: 119392, Homo sapiens chr. 10<br>chromosome 10 open reading frame 78	E2FF-NFKB	429 - 557	(+)	
**GXP_904622** [GXP_904622] (1 - 601)<br>**PDCD4**, GXL_134209, GeneID: 27250, Homo sapiens chr. 10<br>programmed cell death 4 (neoplastic transformation inhibitor)	E2FF-NFKB	456 - 335	(-)	
**GXP_161445** [GXP_161445] (1 - 1161)<br>**GPR123**, GXL_134211, GeneID: 84435, Homo sapiens chr. 10<br>G protein-coupled receptor 123	E2FF-NFKB	228 - 117	(-)	
**GXP_161469** [GXP_161469] (1 - 1196)<br>**EMX2**, GXL_134224, GeneID: 2018, Homo sapiens chr. 10<br>empty spiracles homeobox 2	E2FF-NFKB	960 - 1068	(+)	
**GXP_479630** [GXP_479630] (1 - 1102)<br>**PPAPDC1A**, GXL_134226, GeneID: 196051, Homo sapiens chr. 10<br>phosphatidic acid phosphatase type 2 domain containing 1A	E2FF-NFKB	249 - 365	(+)	
**GXP_161558** [GXP_161558] (1 - 653)<br>**PPRC1**, GXL_134282, GeneID: 23082, Homo sapiens chr. 10<br>peroxisome proliferator-activated receptor gamma, coactivator-related 1	E2FF-NFKB	312 - 202	(-)	
**GXP_161575** [GXP_161575] (1 - 1478)<br>**GPR26**, GXL_134291, GeneID: 2849, Homo sapiens chr. 10<br>G protein-coupled receptor 26	E2FF-NFKB	629 - 753	(+)	
**GXP_161590** [GXP_161590] (1 - 680)<br>**INPP5F**, GXL_134304, GeneID: 22876, Homo sapiens chr. 10<br>inositol polyphosphate-5-phosphatase F	E2FF-NFKB	296 - 169	(-)	
**GXP_161599** [GXP_161599] (1 - 601)<br>**MARVELD1**, GXL_134312, GeneID: 83742, Homo sapiens chr. 10<br>MARVEL domain containing 1	E2FF-NFKB	543 - 422	(-)	
	E2FF-NFKB	443 - 549	(+)	

**GXP_904501** [GXP_904501] (1 - 601)<br>**MARVELD1**, GXL_134312, GeneID: 83742, Homo sapiens chr. 10<br>MARVEL domain containing 1				
**GXP_161635** [GXP_161635] (1 - 601)<br>**ADRB1**, GXL_134345, GeneID: 153, Homo sapiens chr. 10<br>adrenergic, beta-1-, receptor	E2FF-NFKB	420 - 535	(+)	
**GXP_167973** [GXP_167973] (1 - 793)<br>**PKP2**, GXL_139723, GeneID: 5318, Homo sapiens chr. 12<br>plakophilin 2	E2FF-NFKB	133 - 251	(+)	
**GXP_167990** [GXP_167990] (1 - 675)<br>**CPNE8**, GXL_139730, GeneID: 144402, Homo sapiens chr. 12<br>copine VIII	E2FF-NFKB	495 - 373	(-)	
**GXP_906590** [GXP_906590] (1 - 601)<br>**TM7SF3**, GXL_139749, GeneID: 51768, Homo sapiens chr. 12<br>transmembrane 7 superfamily member 3	E2FF-NFKB	447 - 555	(+)	
**GXP_906729** [GXP_906729] (1 - 601)<br>**HDAC7A**, GXL_139758, GeneID: 51564, Homo sapiens chr. 12<br>histone deacetylase 7A	E2FF-NFKB	447 - 576	(+)	
**GXP_168067** [GXP_168067] (1 - 859)<br>**PTHLH**, GXL_139768, GeneID: 5744, Homo sapiens chr. 12<br>parathyroid hormone-like hormone	E2FF-NFKB	4 - 129	(+)	
**GXP_906604** [GXP_906604] (1 - 601)<br>**PTHLH**, GXL_139768, GeneID: 5744, Homo sapiens chr. 12<br>parathyroid hormone-like hormone	E2FF-NFKB	320 - 445	(+)	
**GXP_168106** [GXP_168106] (1 - 879)<br>**PHB2**, GXL_139788, GeneID: 11331, Homo sapiens chr. 12<br>prohibitin 2	E2FF-NFKB	681 - 559	(-)	
**GXP_168122** [GXP_168122] (1 - 601)<br>**FBXL14**, GXL_139800, GeneID: 144699, Homo sapiens chr. 12<br>F-box and leucine-rich repeat protein 14	E2FF-NFKB	134 - 18	(-)	
**GXP_168146** [GXP_168146] (1 - 601)<br>**GRIN2B**, GXL_139819, GeneID: 2904, Homo sapiens chr. 12<br>glutamate receptor, ionotropic, N-methyl D-aspartate 2B	E2FF-NFKB	263 - 146	(-)	
**GXP_168186** [GXP_168186] (1 - 601)<br>**CHD4**, GXL_139849, GeneID: 1108, Homo sapiens chr. 12<br>chromodomain helicase DNA binding protein 4	E2FF-NFKB	461 - 335	(-)	
**GXP_906381** [GXP_906381] (1 - 1149)<br>**SPSB2**, GXL_139928, GeneID: 84727, Homo sapiens chr. 12<br>splA/ryanodine receptor domain and SOCS box containing 2	E2FF-NFKB	387 - 277	(-)	
	E2FF-NFKB	193 - 307	(+)	

**GXP_168338** [GXP_168338] (1 - 1021)<br>**MLF2**, GXL_139974, GeneID: 8079, Homo sapiens chr. 12<br>myeloid leukemia factor 2				
**GXP_169528** [GXP_169528] (1 - 601)<br>**BDNF**, GXL_140994, GeneID: 627, Homo sapiens chr. 11<br>brain-derived neurotrophic factor	E2FF-NFKB	141 - 33	(-)	
	E2FF-NFKB	166 - 275	(+)	
**GXP_169564** [GXP_169564] (1 - 636)<br>**CD59**, GXL_141013, GeneID: 966, Homo sapiens chr. 11<br>CD59 molecule, complement regulatory protein	E2FF-NFKB	540 - 424	(-)	
**GXP_169585** [GXP_169585] (1 - 1386)<br>**TMEM9B**, GXL_141022, GeneID: 56674, Homo sapiens chr. 11<br>TMEM9 domain family, member B	E2FF-NFKB	842 - 956	(+)	
**GXP_169604** [GXP_169604] (1 - 926)<br>**NUP98**, GXL_141036, GeneID: 4928, Homo sapiens chr. 11<br>nucleoporin 98kDa	E2FF-NFKB	224 - 333	(+)	
**GXP_169614** [GXP_169614] (1 - 612)<br>**TRIM5**, GXL_141044, GeneID: 85363, Homo sapiens chr. 11<br>tripartite motif-containing 5	E2FF-NFKB	406 - 524	(+)	
**GXP_169685** [GXP_169685] (1 - 670)<br>**UEVLD**, GXL_141090, GeneID: 55293, Homo sapiens chr. 11<br>UEV and lactate/malate dehyrogenase domains	E2FF-NFKB	340 - 469	(+)	
**GXP_169734** [GXP_169734] (1 - 696)<br>**PAX6,LOC729605,LOC732294**, GXL_141121, GeneID: 5080,729605,732294, Homo sapiens chr. 11<br>paired box gene 6 (aniridia, keratitis); similar to paired box gene 6 (aniridia, keratitis); similar to paired box gene 6 (aniridia, keratitis)	E2FF-NFKB	275 - 169	(-)	
**GXP_169748** [GXP_169748] (1 - 1249)<br>**ABTB2**, GXL_141132, GeneID: 25841, Homo sapiens chr. 11<br>ankyrin repeat and BTB (POZ) domain containing 2	E2FF-NFKB	669 - 556	(-)	
**GXP_169853** [GXP_169853] (1 - 1350)<br>**CCDC34**, GXL_141216, GeneID: 91057, Homo sapiens chr. 11<br>coiled-coil domain containing 34	E2FF-NFKB	1216 - 1087	(-)	
**GXP_905071** [GXP_905071] (1 - 944)<br>**CYP2R1**, GXL_141238, GeneID: 120227, Homo sapiens chr. 11<br>cytochrome P450, family 2, subfamily R, polypeptide 1	E2FF-NFKB	528 - 408	(-)	
**GXP_170224** [GXP_170224] (1 - 643)<br>**IGFBP6**, GXL_141532, GeneID: 3489, Homo sapiens chr. 12<br>insulin-like growth factor binding protein 6	E2FF-NFKB	298 - 426	(+)	
**GXP_170250** [GXP_170250] (1 - 1113)<br>**ERBB3**, GXL_141545, GeneID: 2065, Homo sapiens chr. 12<br>v-erb-b2 erythroblastic leukemia viral oncogene homolog 3 (avian)	E2FF-NFKB	915 - 787	(-)	
	E2FF-NFKB	204 - 323	(+)	

**GXP_170281** [GXP_170281] (1 - 601)<br>**CNOT2**, GXL_141564, GeneID: 4848, Homo sapiens chr. 12<br>CCR4-NOT transcription complex, subunit 2				
**GXP_170327** [GXP_170327] (1 - 999)<br>**TARBP2**, GXL_141592, GeneID: 6895, Homo sapiens chr. 12<br>Tar (HIV-1) RNA binding protein 2	E2FF-NFKB	563 - 452	(-)	
**GXP_170395** [GXP_170395] (1 - 608)<br>**KRT18**, GXL_141645, GeneID: 3875, Homo sapiens chr. 12<br>keratin 18	E2FF-NFKB	191 - 77	(-)	
**GXP_170420** [GXP_170420] (1 - 1223)<br>**SLC17A8**, GXL_141662, GeneID: 246213, Homo sapiens chr. 12<br>solute carrier family 17 (sodium-dependent inorganic phosphate cotransporter), member 8	E2FF-NFKB	508 - 636	(+)	
**GXP_170442** [GXP_170442] (1 - 629)<br>**SNRPF**, GXL_141676, GeneID: 6636, Homo sapiens chr. 12<br>small nuclear ribonucleoprotein polypeptide F	E2FF-NFKB	388 - 498	(+)	
**GXP_170457** [GXP_170457] (1 - 601)<br>**TENC1**, GXL_141690, GeneID: 23371, Homo sapiens chr. 12<br>tensin like C1 domain containing phosphatase (tensin 2)	E2FF-NFKB	384 - 276	(-)	
**GXP_641435** [GXP_641435] (1 - 601)<br>**TENC1**, GXL_141690, GeneID: 23371, Homo sapiens chr. 12<br>tensin like C1 domain containing phosphatase (tensin 2)	E2FF-NFKB	192 - 84	(-)	
**GXP_907101** [GXP_907101] (1 - 629)<br>**SYT1**, GXL_141692, GeneID: 6857, Homo sapiens chr. 12<br>synaptotagmin I	E2FF-NFKB	187 - 301	(+)	
**GXP_906852** [GXP_906852] (1 - 647)<br>**ESPL1**, GXL_141718, GeneID: 9700, Homo sapiens chr. 12<br>extra spindle pole bodies homolog 1 (S. cerevisiae)	E2FF-NFKB	443 - 326	(-)	
**GXP_170501** [GXP_170501] (1 - 1019)<br>**ZDHHC17**, GXL_141720, GeneID: 23390, Homo sapiens chr. 12<br>zinc finger, DHHC-type containing 17	E2FF-NFKB	127 - 241	(+)	
**GXP_907089** [GXP_907089] (1 - 601)<br>**ZDHHC17**, GXL_141720, GeneID: 23390, Homo sapiens chr. 12<br>zinc finger, DHHC-type containing 17	E2FF-NFKB	235 - 349	(+)	
**GXP_170503** [GXP_170503] (1 - 1081)<br>**LRP1**, GXL_141722, GeneID: 4035, Homo sapiens chr. 12<br>low density lipoprotein-related protein 1 (alpha-2-macroglobulin receptor)	E2FF-NFKB	799 - 908	(+)	
**GXP_170548** [GXP_170548] (1 - 722)<br>**RASSF3**, GXL_141765, GeneID: 283349, Homo sapiens chr. 12<br>Ras association (RalGDS/AF-6) domain family 3	E2FF-NFKB	137 - 29	(-)	
	E2FF-NFKB	556 - 438	(-)	

**GXP_180624** [GXP_180624] (1 - 601)<br>**CSRP1**, GXL_151179, GeneID: 1465, Homo sapiens chr. 1<br>cysteine and glycine-rich protein 1				
**GXP_637739** [GXP_637739] (1 - 601)<br>**SLC26A9**, GXL_151183, GeneID: 115019, Homo sapiens chr. 1<br>solute carrier family 26, member 9	E2FF-NFKB	254 - 128	(-)	
**GXP_180657** [GXP_180657] (1 - 1003)<br>**ETNK2**, GXL_151196, GeneID: 55224, Homo sapiens chr. 1<br>ethanolamine kinase 2	E2FF-NFKB	726 - 605	(-)	
**GXP_180658** [GXP_180658] (1 - 814)<br>**RIPK5**, GXL_151197, GeneID: 25778, Homo sapiens chr. 1<br>receptor interacting protein kinase 5	E2FF-NFKB	332 - 456	(+)	
**GXP_180668** [GXP_180668] (1 - 1134)<br>**RAB7L1**, GXL_151205, GeneID: 8934, Homo sapiens chr. 1<br>RAB7, member RAS oncogene family-like 1	E2FF-NFKB	815 - 702	(-)	
**GXP_637392** [GXP_637392] (1 - 601)<br>**RC3H1**, GXL_151226, GeneID: 149041, Homo sapiens chr. 1<br>ring finger and CCCH-type zinc finger domains 1	E2FF-NFKB	376 - 252	(-)	
**GXP_180701** [GXP_180701] (1 - 601)<br>**GPR161,LOC730012,LOC731581**, GXL_151228, GeneID: 23432,730012,731581, Homo sapiens chr. 1<br>G protein-coupled receptor 161; similar to G protein-coupled receptor 161; similar to G protein-coupled receptor 161	E2FF-NFKB	260 - 387	(+)	
**GXP_916186** [GXP_916186] (1 - 601)<br>**LPGAT1**, GXL_151241, GeneID: 9926, Homo sapiens chr. 1<br>lysophosphatidylglycerol acyltransferase 1	E2FF-NFKB	253 - 365	(+)	
**GXP_180735** [GXP_180735] (1 - 792)<br>**KCNT2**, GXL_151249, GeneID: 343450, Homo sapiens chr. 1<br>potassium channel, subfamily T, member 2	E2FF-NFKB	241 - 129	(-)	
**GXP_180738** [GXP_180738] (1 - 731)<br>**YOD1**, GXL_151250, GeneID: 55432, Homo sapiens chr. 1<br>YOD1 OTU deubiquinating enzyme 1 homolog (S. cerevisiae)	E2FF-NFKB	631 - 521	(-)	
**GXP_180749** [GXP_180749] (1 - 601)<br>**RFWD2**, GXL_151258, GeneID: 64326, Homo sapiens chr. 1<br>ring finger and WD repeat domain 2	E2FF-NFKB	463 - 353	(-)	
**GXP_180772** [GXP_180772] (1 - 693)<br>**C1orf74**, GXL_151275, GeneID: 148304, Homo sapiens chr. 1<br>chromosome 1 open reading frame 74	E2FF-NFKB	509 - 635	(+)	
**GXP_915719** [GXP_915719] (1 - 836)<br>**DEDD**, GXL_151276, GeneID: 9191, Homo sapiens chr. 1<br>death effector domain containing	E2FF-NFKB	658 - 785	(+)	
	E2FF-NFKB	326 - 442	(+)	

**GXP_180798** [GXP_180798] (1 - 992)<br>**GLUL**, GXL_151294, GeneID: 2752, Homo sapiens chr. 1<br>glutamate-ammonia ligase (glutamine synthetase)				
**GXP_180826** [GXP_180826] (1 - 656)<br>**C1orf110**, GXL_151317, GeneID: 339512, Homo sapiens chr. 1<br>chromosome 1 open reading frame 110	E2FF-NFKB	450 - 576	(+)	
**GXP_180828** [GXP_180828] (1 - 601)<br>**TMEM9**, GXL_151319, GeneID: 252839, Homo sapiens chr. 1<br>transmembrane protein 9	E2FF-NFKB	362 - 244	(-)	
	E2FF-NFKB	450 - 556	(+)	
**GXP_180829** [GXP_180829] (1 - 637)<br>**TMEM9**, GXL_151319, GeneID: 252839, Homo sapiens chr. 1<br>transmembrane protein 9	E2FF-NFKB	423 - 305	(-)	
	E2FF-NFKB	511 - 617	(+)	
**GXP_180831** [GXP_180831] (1 - 744)<br>**DDX59**, GXL_151320, GeneID: 83479, Homo sapiens chr. 1<br>DEAD (Asp-Glu-Ala-Asp) box polypeptide 59	E2FF-NFKB	444 - 323	(-)	
**GXP_637326** [GXP_637326] (1 - 601)<br>**F5**, GXL_151362, GeneID: 2153, Homo sapiens chr. 1<br>coagulation factor V (proaccelerin, labile factor)	E2FF-NFKB	484 - 367	(-)	
**GXP_182149** [GXP_182149] (1 - 634)<br>**PIGM**, GXL_152416, GeneID: 93183, Homo sapiens chr. 1<br>phosphatidylinositol glycan anchor biosynthesis, class M	E2FF-NFKB	450 - 322	(-)	
**GXP_182186** [GXP_182186] (1 - 887)<br>**FLJ16478**, GXL_152440, GeneID: 440695, Homo sapiens chr. 1<br>FLJ16478 protein	E2FF-NFKB	638 - 522	(-)	
**GXP_182193** [GXP_182193] (1 - 805)<br>**APH1A**, GXL_152444, GeneID: 51107, Homo sapiens chr. 1<br>anterior pharynx defective 1 homolog A (C. elegans)	E2FF-NFKB	637 - 757	(+)	
**GXP_182200** [GXP_182200] (1 - 1385)<br>**PYGO2**, GXL_152448, GeneID: 90780, Homo sapiens chr. 1<br>pygopus homolog 2 (Drosophila)	E2FF-NFKB	479 - 607	(+)	
**GXP_182201** [GXP_182201] (1 - 601)<br>**PYGO2**, GXL_152448, GeneID: 90780, Homo sapiens chr. 1<br>pygopus homolog 2 (Drosophila)	E2FF-NFKB	371 - 499	(+)	
**GXP_182225** [GXP_182225] (1 - 601)<br>**GBA**, GXL_152462, GeneID: 2629, Homo sapiens chr. 1<br>glucosidase, beta, acid (includes glucosylceramidase)	E2FF-NFKB	291 - 167	(-)	
**GXP_915243** [GXP_915243] (1 - 601)<br>**CSDE1**, GXL_152504, GeneID: 7812, Homo sapiens chr. 1<br>cold shock domain containing E1, RNA-binding	E2FF-NFKB	409 - 293	(-)	
	E2FF-NFKB	512 - 395	(-)	

**GXP_182305** [GXP_182305] (1 - 900)<br>**INSRR**, GXL_152513, GeneID: 3645, Homo sapiens chr. 1<br>insulin receptor-related receptor				
**GXP_182338** [GXP_182338] (1 - 631)<br>**HORMAD1**, GXL_152534, GeneID: 84072, Homo sapiens chr. 1<br>HORMA domain containing 1	E2FF-NFKB	476 - 347	(-)	
**GXP_182354** [GXP_182354] (1 - 601)<br>**SLC39A1**, GXL_152546, GeneID: 27173, Homo sapiens chr. 1<br>solute carrier family 39 (zinc transporter), member 1	E2FF-NFKB	104 - 230	(+)	
**GXP_182392** [GXP_182392] (1 - 601)<br>**DPM3**, GXL_152571, GeneID: 54344, Homo sapiens chr. 1<br>dolichyl-phosphate mannosyltransferase polypeptide 3	E2FF-NFKB	444 - 558	(+)	
**GXP_182419** [GXP_182419] (1 - 775)<br>**SEMA6C**, GXL_152591, GeneID: 10500, Homo sapiens chr. 1<br>sema domain, transmembrane domain (TM), and cytoplasmic domain, (semaphorin) 6C	E2FF-NFKB	419 - 548	(+)	
**GXP_182425** [GXP_182425] (1 - 631)<br>**PBXIP1**, GXL_152597, GeneID: 57326, Homo sapiens chr. 1<br>pre-B-cell leukemia homeobox interacting protein 1	E2FF-NFKB	378 - 250	(-)	
**GXP_182472** [GXP_182472] (1 - 715)<br>**CD5L**, GXL_152635, GeneID: 922, Homo sapiens chr. 1<br>CD5 molecule-like	E2FF-NFKB	279 - 160	(-)	
	E2FF-NFKB	412 - 290	(-)	
**GXP_186608** [GXP_186608] (1 - 936)<br>**SLC8A3**, GXL_155832, GeneID: 6547, Homo sapiens chr. 14<br>solute carrier family 8 (sodium-calcium exchanger), member 3	E2FF-NFKB	690 - 817	(+)	
**GXP_186638** [GXP_186638] (1 - 752)<br>**DPF3**, GXL_155851, GeneID: 8110, Homo sapiens chr. 14<br>D4, zinc and double PHD fingers, family 3	E2FF-NFKB	573 - 461	(-)	
**GXP_186644** [GXP_186644] (1 - 924)<br>**CFL2**, GXL_155852, GeneID: 1073, Homo sapiens chr. 14<br>cofilin 2 (muscle)	E2FF-NFKB	352 - 460	(+)	
**GXP_186678** [GXP_186678] (1 - 628)<br>**C14orf124**, GXL_155870, GeneID: 56948, Homo sapiens chr. 14<br>chromosome 14 open reading frame 124	E2FF-NFKB	507 - 616	(+)	
**GXP_642994** [GXP_642994] (1 - 601)<br>**DHRS1**, GXL_155874, GeneID: 115817, Homo sapiens chr. 14<br>dehydrogenase/reductase (SDR family) member 1	E2FF-NFKB	182 - 293	(+)	
**GXP_186693** [GXP_186693] (1 - 683)<br>**CHMP4A,MDP-1**, GXL_155882, GeneID: 29082,145553, Homo sapiens chr. 14<br>chromatin modifying protein 4A; magnesium-dependent phosphatase 1	E2FF-NFKB	466 - 343	(-)	
	E2FF-NFKB	544 - 431	(-)	

**GXP_908134** [GXP_908134] (1 - 601)<br>**MYH7**, GXL_155895, GeneID: 4625, Homo sapiens chr. 14<br>myosin, heavy chain 7, cardiac muscle, beta				
**GXP_908368** [GXP_908368] (1 - 601)<br>**SIX1**, GXL_155981, GeneID: 6495, Homo sapiens chr. 14<br>sine oculis homeobox homolog 1 (Drosophila)	E2FF-NFKB	407 - 279	(-)	
**GXP_186838** [GXP_186838] (1 - 922)<br>**NOVA1**, GXL_155984, GeneID: 4857, Homo sapiens chr. 14<br>neuro-oncological ventral antigen 1	E2FF-NFKB	719 - 840	(+)	
**GXP_186867** [GXP_186867] (1 - 1051)<br>**ERO1L**, GXL_156003, GeneID: 30001, Homo sapiens chr. 14<br>ERO1-like (S. cerevisiae)	E2FF-NFKB	366 - 491	(+)	
**GXP_908228** [GXP_908228] (1 - 894)<br>**TITF1**, GXL_156007, GeneID: 7080, Homo sapiens chr. 14<br>thyroid transcription factor 1	E2FF-NFKB	669 - 781	(+)	
**GXP_186888** [GXP_186888] (1 - 1172)<br>**TMEM30B**, GXL_156021, GeneID: 161291, Homo sapiens chr. 14<br>transmembrane protein 30B	E2FF-NFKB	386 - 512	(+)	
**GXP_186905** [GXP_186905] (1 - 606)<br>**STXBP6**, GXL_156035, GeneID: 29091, Homo sapiens chr. 14<br>syntaxin binding protein 6 (amisyn)	E2FF-NFKB	338 - 447	(+)	
**GXP_186922** [GXP_186922] (1 - 1107)<br>**NFKBIA**, GXL_156052, GeneID: 4792, Homo sapiens chr. 14<br>nuclear factor of kappa light polypeptide gene enhancer in B-cells inhibitor, alpha	E2FF-NFKB	726 - 613	(-)	
**GXP_186924** [GXP_186924] (1 - 721)<br>**PYGL**, GXL_156054, GeneID: 5836, Homo sapiens chr. 14<br>phosphorylase, glycogen, liver (Hers disease, glycogen storage disease type VI)	E2FF-NFKB	523 - 416	(-)	
**GXP_908292** [GXP_908292] (1 - 601)<br>**PYGL**, GXL_156054, GeneID: 5836, Homo sapiens chr. 14<br>phosphorylase, glycogen, liver (Hers disease, glycogen storage disease type VI)	E2FF-NFKB	497 - 391	(-)	
**GXP_186948** [GXP_186948] (1 - 692)<br>**PPARD**, GXL_156078, GeneID: 5467, Homo sapiens chr. 6<br>peroxisome proliferator-activated receptor delta	E2FF-NFKB	334 - 460	(+)	
**GXP_186968** [GXP_186968] (1 - 776)<br>**CYP21A2**, GXL_156094, GeneID: 1589, Homo sapiens chr. 6<br>cytochrome P450, family 21, subfamily A, polypeptide 2	E2FF-NFKB	71 - 178	(+)	
**GXP_186978** [GXP_186978] (1 - 722)<br>**SLC39A7**, GXL_156099, GeneID: 7922, Homo sapiens chr. 6<br>solute carrier family 39 (zinc transporter), member 7	E2FF-NFKB	125 - 241	(+)	
	E2FF-NFKB	73 - 183	(+)	

**GXP_658030** [GXP_658030] (1 - 601)<br>**HMGA1**, GXL_156107, GeneID: 3159, Homo sapiens chr. 6<br>high mobility group AT-hook 1				
**GXP_923650** [GXP_923650] (1 - 646)<br>**HMGA1**, GXL_156107, GeneID: 3159, Homo sapiens chr. 6<br>high mobility group AT-hook 1	E2FF-NFKB	335 - 218	(-)	
**GXP_187075** [GXP_187075] (1 - 863)<br>**PFDN6**, GXL_156158, GeneID: 10471, Homo sapiens chr. 6<br>prefoldin subunit 6	E2FF-NFKB	34 - 148	(+)	
**GXP_187251** [GXP_187251] (1 - 930)<br>**CD83**, GXL_156293, GeneID: 9308, Homo sapiens chr. 6<br>CD83 molecule	E2FF-NFKB	837 - 731	(-)	
**GXP_187252** [GXP_187252] (1 - 882)<br>**ITPR3**, GXL_156294, GeneID: 3710, Homo sapiens chr. 6<br>inositol 1,4,5-triphosphate receptor, type 3	E2FF-NFKB	47 - 160	(+)	
**GXP_923622** [GXP_923622] (1 - 725)<br>**HSD17B8**, GXL_156329, GeneID: 7923, Homo sapiens chr. 6<br>hydroxysteroid (17-beta) dehydrogenase 8	E2FF-NFKB	151 - 267	(+)	
**GXP_190399** [GXP_190399] (1 - 607)<br>**SLC25A30**, GXL_159013, GeneID: 253512, Homo sapiens chr. 13<br>solute carrier family 25, member 30	E2FF-NFKB	442 - 314	(-)	
**GXP_190417** [GXP_190417] (1 - 727)<br>**RP11-125A7.3**, GXL_159021, GeneID: 23078, Homo sapiens chr. 13<br>KIAA0564 protein	E2FF-NFKB	214 - 330	(+)	
**GXP_190442** [GXP_190442] (1 - 601)<br>**PCDH9**, GXL_159029, GeneID: 5101, Homo sapiens chr. 13<br>protocadherin 9	E2FF-NFKB	199 - 324	(+)	
**GXP_190443** [GXP_190443] (1 - 922)<br>**DIS3**, GXL_159030, GeneID: 22894, Homo sapiens chr. 13<br>DIS3 mitotic control homolog (S. cerevisiae)	E2FF-NFKB	632 - 521	(-)	
**GXP_907741** [GXP_907741] (1 - 601)<br>**SOHLH2**, GXL_159049, GeneID: 54937, Homo sapiens chr. 13<br>spermatogenesis and oogenesis specific basic helix-loop-helix 2	E2FF-NFKB	404 - 513	(+)	
**GXP_190507** [GXP_190507] (1 - 601)<br>**CCDC122**, GXL_159062, GeneID: 160857, Homo sapiens chr. 13<br>coiled-coil domain containing 122	E2FF-NFKB	262 - 146	(-)	
**GXP_907796** [GXP_907796] (1 - 748)<br>**LCP1**, GXL_159109, GeneID: 3936, Homo sapiens chr. 13<br>lymphocyte cytosolic protein 1 (L-plastin)	E2FF-NFKB	714 - 600	(-)	
	E2FF-NFKB	391 - 503	(+)	

**GXP_190635** [GXP_190635] (1 - 604)<br>**NEK3**, GXL_159134, GeneID: 4752, Homo sapiens chr. 13<br>NIMA (never in mitosis gene a)-related kinase 3				
**GXP_190646** [GXP_190646] (1 - 1224)<br>**UBL3**, GXL_159139, GeneID: 5412, Homo sapiens chr. 13<br>ubiquitin-like 3	E2FF-NFKB	752 - 874	(+)	
**GXP_907784** [GXP_907784] (1 - 601)<br>**TPT1**, GXL_159164, GeneID: 7178, Homo sapiens chr. 13<br>tumor protein, translationally-controlled 1	E2FF-NFKB	193 - 316	(+)	
**GXP_190754** [GXP_190754] (1 - 1097)<br>**KATNAL1**, GXL_159224, GeneID: 84056, Homo sapiens chr. 13<br>katanin p60 subunit A-like 1	E2FF-NFKB	591 - 466	(-)	
**GXP_190768** [GXP_190768] (1 - 673)<br>**PARP4**, GXL_159237, GeneID: 143, Homo sapiens chr. 13<br>poly (ADP-ribose) polymerase family, member 4	E2FF-NFKB	424 - 551	(+)	
**GXP_190781** [GXP_190781] (1 - 601)<br>**TMEM46**, GXL_159250, GeneID: 387914, Homo sapiens chr. 13<br>transmembrane protein 46	E2FF-NFKB	577 - 456	(-)	
**GXP_190804** [GXP_190804] (1 - 905)<br>**CCNU**, GXL_159271, GeneID: 10309, Homo sapiens chr. 5<br>cyclin U	E2FF-NFKB	502 - 621	(+)	
**GXP_190818** [GXP_190818] (1 - 632)<br>**CDC20B**, GXL_159279, GeneID: 166979, Homo sapiens chr. 5<br>cell division cycle 20 homolog B (S. cerevisiae)	E2FF-NFKB	277 - 390	(+)	
**GXP_190833** [GXP_190833] (1 - 601)<br>**AYTL2**, GXL_159283, GeneID: 79888, Homo sapiens chr. 5<br>acyltransferase like 2	E2FF-NFKB	160 - 273	(+)	
**GXP_190842** [GXP_190842] (1 - 740)<br>**TMEM167**, GXL_159291, GeneID: 153339, Homo sapiens chr. 5<br>transmembrane protein 167	E2FF-NFKB	586 - 696	(+)	
**GXP_190867** [GXP_190867] (1 - 601)<br>**FGF10**, GXL_159309, GeneID: 2255, Homo sapiens chr. 5<br>fibroblast growth factor 10	E2FF-NFKB	386 - 269	(-)	
**GXP_190884** [GXP_190884] (1 - 601)<br>**CTNND2**, GXL_159321, GeneID: 1501, Homo sapiens chr. 5<br>catenin (cadherin-associated protein), delta 2 (neural plakophilin-related arm-repeat protein)	E2FF-NFKB	116 - 235	(+)	
**GXP_190892** [GXP_190892] (1 - 986)<br>**CLPTM1L**, GXL_159323, GeneID: 81037, Homo sapiens chr. 5<br>CLPTM1-like	E2FF-NFKB	509 - 617	(+)	
	E2FF-NFKB	356 - 479	(+)	

**GXP_190935** [GXP_190935] (1 - 761)<br>**AMACR**, GXL_159351, GeneID: 23600, Homo sapiens chr. 5<br>alpha-methylacyl-CoA racemase				
**GXP_922630** [GXP_922630] (1 - 601)<br>**SERINC5**, GXL_159373, GeneID: 256987, Homo sapiens chr. 5<br>serine incorporator 5	E2FF-NFKB	492 - 371	(-)	
**GXP_190979** [GXP_190979] (1 - 710)<br>**ZNF622**, GXL_159384, GeneID: 90441, Homo sapiens chr. 5<br>zinc finger protein 622	E2FF-NFKB	165 - 275	(+)	
**GXP_191003** [GXP_191003] (1 - 601)<br>**RPS23**, GXL_159399, GeneID: 6228, Homo sapiens chr. 5<br>ribosomal protein S23	E2FF-NFKB	357 - 469	(+)	
	E2FF-NFKB	370 - 255	(-)	
**GXP_922458** [GXP_922458] (1 - 730)<br>**PDE4D**, GXL_159402, GeneID: 5144, Homo sapiens chr. 5<br>phosphodiesterase 4D, cAMP-specific (phosphodiesterase E3 dunce homolog, Drosophila)	E2FF-NFKB	403 - 521	(+)	
**GXP_191068** [GXP_191068] (1 - 636)<br>**FLJ32255**, GXL_159443, GeneID: 643977, Homo sapiens chr. 5<br>hypothetical protein LOC643977	E2FF-NFKB	504 - 619	(+)	
**GXP_191072** [GXP_191072] (1 - 892)<br>**ANKH**, GXL_159444, GeneID: 56172, Homo sapiens chr. 5<br>ankylosis, progressive homolog (mouse)	E2FF-NFKB	733 - 616	(-)	
**GXP_191074** [GXP_191074] (1 - 611)<br>**RICTOR**, GXL_159445, GeneID: 253260, Homo sapiens chr. 5<br>rapamycin-insensitive companion of mTOR	E2FF-NFKB	340 - 224	(-)	
**GXP_191104** [GXP_191104] (1 - 627)<br>**C5orf34**, GXL_159467, GeneID: 375444, Homo sapiens chr. 5<br>chromosome 5 open reading frame 34	E2FF-NFKB	532 - 409	(-)	
**GXP_922278** [GXP_922278] (1 - 601)<br>**CDH12**, GXL_159495, GeneID: 1010, Homo sapiens chr. 5<br>cadherin 12, type 2 (N-cadherin 2)	E2FF-NFKB	381 - 264	(-)	
**GXP_922636** [GXP_922636] (1 - 601)<br>**DP58**, GXL_159546, GeneID: 340120, Homo sapiens chr. 5<br>cytosolic phosphoprotein DP58	E2FF-NFKB	49 - 166	(+)	
**GXP_191193** [GXP_191193] (1 - 601)<br>**HTR1A**, GXL_159548, GeneID: 3350, Homo sapiens chr. 5<br>5-hydroxytryptamine (serotonin) receptor 1A	E2FF-NFKB	463 - 335	(-)	
**GXP_193309** [GXP_193309] (1 - 729)<br>**ALG8**, GXL_161224, GeneID: 79053, Homo sapiens chr. 11<br>asparagine-linked glycosylation 8 homolog (S. cerevisiae, alpha-1,3-glucosyltransferase)	E2FF-NFKB	293 - 177	(-)	
	E2FF-NFKB	1019 - 898	(-)	

**GXP_193427** [GXP_193427] (1 - 1073)<br>**ZNF259**, GXL_161298, GeneID: 8882, Homo sapiens chr. 11<br>zinc finger protein 259				
**GXP_193428** [GXP_193428] (1 - 1104)<br>**TIMM8B**, GXL_161299, GeneID: 26521, Homo sapiens chr. 11<br>translocase of inner mitochondrial membrane 8 homolog B (yeast)	E2FF-NFKB	886 - 762	(-)	
**GXP_905829** [GXP_905829] (1 - 601)<br>**GAB2**, GXL_161302, GeneID: 9846, Homo sapiens chr. 11<br>GRB2-associated binding protein 2	E2FF-NFKB	329 - 447	(+)	
**GXP_193436** [GXP_193436] (1 - 758)<br>**PHOX2A**, GXL_161305, GeneID: 401, Homo sapiens chr. 11<br>paired-like (aristaless) homeobox 2a	E2FF-NFKB	312 - 195	(-)	
**GXP_193442** [GXP_193442] (1 - 601)<br>**MAML2**, GXL_161311, GeneID: 84441, Homo sapiens chr. 11<br>mastermind-like 2 (Drosophila)	E2FF-NFKB	426 - 316	(-)	
**GXP_640710** [GXP_640710] (1 - 601)<br>**TREH**, GXL_161321, GeneID: 11181, Homo sapiens chr. 11<br>trehalase (brush-border membrane glycoprotein)	E2FF-NFKB	450 - 329	(-)	
**GXP_193462** [GXP_193462] (1 - 832)<br>**KDELC2**, GXL_161327, GeneID: 143888, Homo sapiens chr. 11<br>KDEL (Lys-Asp-Glu-Leu) containing 2	E2FF-NFKB	118 - 240	(+)	
**GXP_480687** [GXP_480687] (1 - 601)<br>**SLC37A4**, GXL_161343, GeneID: 2542, Homo sapiens chr. 11<br>solute carrier family 37 (glycerol-6-phosphate transporter), member 4	E2FF-NFKB	160 - 276	(+)	
**GXP_193483** [GXP_193483] (1 - 701)<br>**C11orf51**, GXL_161345, GeneID: 25906, Homo sapiens chr. 11<br>chromosome 11 open reading frame 51	E2FF-NFKB<br>E2FF-NFKB	432 - 557<br>692 - 564	(+)<br>(-)	
**GXP_905736** [GXP_905736] (1 - 601)<br>**C11orf51**, GXL_161345, GeneID: 25906, Homo sapiens chr. 11<br>chromosome 11 open reading frame 51	E2FF-NFKB	174 - 46	(-)	
**GXP_905753** [GXP_905753] (1 - 659)<br>**CENTD2**, GXL_161352, GeneID: 116985, Homo sapiens chr. 11<br>centaurin, delta 2	E2FF-NFKB	528 - 656	(+)	
**GXP_905797** [GXP_905797] (1 - 601)<br>**ARRB1**, GXL_161358, GeneID: 408, Homo sapiens chr. 11<br>arrestin, beta 1	E2FF-NFKB	48 - 156	(+)	
**GXP_193538** [GXP_193538] (1 - 601)<br>**DSCAML1**, GXL_161385, GeneID: 57453, Homo sapiens chr. 11<br>Down syndrome cell adhesion molecule like 1	E2FF-NFKB	129 - 255	(+)	
	E2FF-NFKB	452 - 558	(+)	

**GXP_193544** [GXP_193544] (1 - 992)<br>**GUCY1A2**, GXL_161391, GeneID: 2977, Homo sapiens chr. 11<br>guanylate cyclase 1, soluble, alpha 2				
**GXP_195961** [GXP_195961] (1 - 1123)<br>**CCT8**, GXL_163459, GeneID: 10694, Homo sapiens chr. 21<br>chaperonin containing TCP1, subunit 8 (theta)	E2FF-NFKB	672 - 793	(+)	
**GXP_917126** [GXP_917126] (1 - 601)<br>**APP**, GXL_163473, GeneID: 351, Homo sapiens chr. 21<br>amyloid beta (A4) precursor protein (peptidase nexin-II, Alzheimer disease)	E2FF-NFKB	17 - 135	(+)	
**GXP_196007** [GXP_196007] (1 - 827)<br>**CLIC6**, GXL_163482, GeneID: 54102, Homo sapiens chr. 21<br>chloride intracellular channel 6	E2FF-NFKB	605 - 495	(-)	
**GXP_917293** [GXP_917293] (1 - 735)<br>**FTCD**, GXL_163489, GeneID: 10841, Homo sapiens chr. 21<br>formiminotransferase cyclodeaminase	E2FF-NFKB	465 - 583	(+)	
**GXP_196037** [GXP_196037] (1 - 601)<br>**PIGP**, GXL_163499, GeneID: 51227, Homo sapiens chr. 21<br>phosphatidylinositol glycan anchor biosynthesis, class P	E2FF-NFKB	103 - 219	(+)	
**GXP_196062** [GXP_196062] (1 - 1107)<br>**IFNGR2**, GXL_163516, GeneID: 3460, Homo sapiens chr. 21<br>interferon gamma receptor 2 (interferon gamma transducer 1)	E2FF-NFKB	937 - 830	(-)	
**GXP_196071** [GXP_196071] (1 - 619)<br>**C21orf91**, GXL_163522, GeneID: 54149, Homo sapiens chr. 21<br>chromosome 21 open reading frame 91	E2FF-NFKB	336 - 461	(+)	
**GXP_196104** [GXP_196104] (1 - 750)<br>**SOD1**, GXL_163544, GeneID: 6647, Homo sapiens chr. 21<br>superoxide dismutase 1, soluble (amyotrophic lateral sclerosis 1 (adult))	E2FF-NFKB<br>E2FF-NFKB	514 - 392<br>720 - 593	(-)<br>(-)	
**GXP_652502** [GXP_652502] (1 - 1096)<br>**MORC3**, GXL_163567, GeneID: 23515, Homo sapiens chr. 21<br>MORC family CW-type zinc finger 3	E2FF-NFKB	476 - 361	(-)	
**GXP_196159** [GXP_196159] (1 - 601)<br>**C21orf51**, GXL_163583, GeneID: 54065, Homo sapiens chr. 21<br>chromosome 21 open reading frame 51	E2FF-NFKB	601 - 488	(-)	
**GXP_917242** [GXP_917242] (1 - 601)<br>**CBS**, GXL_163600, GeneID: 875, Homo sapiens chr. 21<br>cystathionine-beta-synthase	E2FF-NFKB	231 - 107	(-)	
**GXP_196207** [GXP_196207] (1 - 686)<br>**U2AF1**, GXL_163625, GeneID: 7307, Homo sapiens chr. 21<br>U2 small nuclear RNA auxiliary factor 1	E2FF-NFKB	271 - 396	(+)	
	E2FF-NFKB	48 - 177	(+)	

**GXP_196238** [GXP_196238] (1 - 601)<br>**ZNF295**, GXL_163653, GeneID: 49854, Homo sapiens chr. 21<br>zinc finger protein 295				
**GXP_196256** [GXP_196256] (1 - 620)<br>**TFF1**, GXL_163670, GeneID: 7031, Homo sapiens chr. 21<br>trefoil factor 1	E2FF-NFKB	533 - 406	(-)	
**GXP_196264** [GXP_196264] (1 - 735)<br>**DSCR6**, GXL_163677, GeneID: 53820, Homo sapiens chr. 21<br>Down syndrome critical region gene 6	E2FF-NFKB	279 - 155	(-)	
	E2FF-NFKB	630 - 508	(-)	
**GXP_197338** [GXP_197338] (1 - 601)<br>**D4S234E**, GXL_164647, GeneID: 27065, Homo sapiens chr. 4<br>DNA segment on chromosome 4 (unique) 234 expressed sequence	E2FF-NFKB	422 - 536	(+)	
**GXP_655287** [GXP_655287] (1 - 601)<br>**ANAPC4**, GXL_164677, GeneID: 29945, Homo sapiens chr. 4<br>anaphase promoting complex subunit 4	E2FF-NFKB	187 - 80	(-)	
**GXP_197400** [GXP_197400] (1 - 747)<br>**AGA**, GXL_164678, GeneID: 175, Homo sapiens chr. 4<br>aspartylglucosaminidase	E2FF-NFKB	91 - 213	(+)	
**GXP_197466** [GXP_197466] (1 - 715)<br>**ASB5**, GXL_164715, GeneID: 140458, Homo sapiens chr. 4<br>ankyrin repeat and SOCS box-containing 5	E2FF-NFKB	352 - 242	(-)	
**GXP_197490** [GXP_197490] (1 - 601)<br>**RBPJ**, GXL_164732, GeneID: 3516, Homo sapiens chr. 4<br>recombination signal binding protein for immunoglobulin kappa J region	E2FF-NFKB	543 - 414	(-)	
**GXP_197547** [GXP_197547] (1 - 1151)<br>**SLC34A2**, GXL_164768, GeneID: 10568, Homo sapiens chr. 4<br>solute carrier family 34 (sodium phosphate), member 2	E2FF-NFKB	823 - 696	(-)	
**GXP_197549** [GXP_197549] (1 - 601)<br>**SLC34A2**, GXL_164768, GeneID: 10568, Homo sapiens chr. 4<br>solute carrier family 34 (sodium phosphate), member 2	E2FF-NFKB	254 - 132	(-)	
**GXP_197553** [GXP_197553] (1 - 601)<br>**FAM114A1,LOC727976**, GXL_164769, GeneID: 92689,727976, Homo sapiens chr. 4<br>family with sequence similarity 114, member A1; hypothetical protein LOC727976	E2FF-NFKB	292 - 186	(-)	
**GXP_655342** [GXP_655342] (1 - 648)<br>**KLF3**, GXL_164778, GeneID: 51274, Homo sapiens chr. 4<br>Kruppel-like factor 3 (basic)	E2FF-NFKB	127 - 238	(+)	
**GXP_197579** [GXP_197579] (1 - 1038)<br>**MSX1**, GXL_164788, GeneID: 4487, Homo sapiens chr. 4<br>msh homeobox 1	E2FF-NFKB	145 - 268	(+)	
	E2FF-NFKB	134 - 15	(-)	

**GXP_655147** [GXP_655147] (1 - 601)<br>**EVC**, GXL_164809, GeneID: 2121, Homo sapiens chr. 4<br>Ellis van Creveld syndrome				
**GXP_200566** [GXP_200566] (1 - 601)<br>**MGRN1**, GXL_167432, GeneID: 23295, Homo sapiens chr. 16<br>mahogunin, ring finger 1	E2FF-NFKB	590 - 476	(-)	
**GXP_200602** [GXP_200602] (1 - 1159)<br>**UBE2I**, GXL_167448, GeneID: 7329, Homo sapiens chr. 16<br>ubiquitin-conjugating enzyme E2I (UBC9 homolog, yeast)	E2FF-NFKB	927 - 1055	(+)	
**GXP_200603** [GXP_200603] (1 - 660)<br>**UBE2I**, GXL_167448, GeneID: 7329, Homo sapiens chr. 16<br>ubiquitin-conjugating enzyme E2I (UBC9 homolog, yeast)	E2FF-NFKB	428 - 556	(+)	
**GXP_200614** [GXP_200614] (1 - 764)<br>**CYBA**, GXL_167455, GeneID: 1535, Homo sapiens chr. 16<br>cytochrome b-245, alpha polypeptide	E2FF-NFKB	612 - 487	(-)	
**GXP_483274** [GXP_483274] (1 - 601)<br>**FLYWCH1**, GXL_167465, GeneID: 84256, Homo sapiens chr. 16<br>FLYWCH-type zinc finger 1	E2FF-NFKB	511 - 388	(-)	
**GXP_200643** [GXP_200643] (1 - 850)<br>**MMP25**, GXL_167472, GeneID: 64386, Homo sapiens chr. 16<br>matrix metallopeptidase 25	E2FF-NFKB	46 - 160	(+)	
**GXP_200656** [GXP_200656] (1 - 645)<br>**CA5A**, GXL_167478, GeneID: 763, Homo sapiens chr. 16<br>carbonic anhydrase VA, mitochondrial	E2FF-NFKB	281 - 400	(+)	
**GXP_200692** [GXP_200692] (1 - 999)<br>**PAQR4**, GXL_167500, GeneID: 124222, Homo sapiens chr. 16<br>progestin and adipoQ receptor family member IV	E2FF-NFKB	926 - 816	(-)	
**GXP_200705** [GXP_200705] (1 - 666)<br>**RNF166**, GXL_167509, GeneID: 115992, Homo sapiens chr. 16<br>ring finger protein 166	E2FF-NFKB	128 - 238	(+)	
**GXP_200712** [GXP_200712] (1 - 941)<br>**SLC7A5**, GXL_167511, GeneID: 8140, Homo sapiens chr. 16<br>solute carrier family 7 (cationic amino acid transporter, y+ system), member 5	E2FF-NFKB	215 - 107	(-)	
**GXP_200744** [GXP_200744] (1 - 601)<br>**UBN1**, GXL_167524, GeneID: 29855, Homo sapiens chr. 16<br>ubinuclein 1	E2FF-NFKB	455 - 571	(+)	
**GXP_909951** [GXP_909951] (1 - 601)<br>**UBN1**, GXL_167524, GeneID: 29855, Homo sapiens chr. 16<br>ubinuclein 1	E2FF-NFKB	150 - 275	(+)	
	E2FF-NFKB	381 - 261	(-)	
	E2FF-NFKB	514 - 401	(-)	
	E2FF-NFKB	354 - 244	(-)	

**GXP_910723** [GXP_910723] (1 - 601)<br>**TAF1C**, GXL_167526, GeneID: 9013, Homo sapiens chr. 16<br>TATA box binding protein (TBP)-associated factor, RNA polymerase I, C, 110kDa				
**GXP_644747** [GXP_644747] (1 - 601)<br>**TRAF7**, GXL_167527, GeneID: 84231, Homo sapiens chr. 16<br>TNF receptor-associated factor 7	E2FF-NFKB	91 - 218	(+)	
**GXP_200757** [GXP_200757] (1 - 601)<br>**CCNF**, GXL_167532, GeneID: 899, Homo sapiens chr. 16<br>cyclin F	E2FF-NFKB	450 - 332	(-)	
**GXP_200778** [GXP_200778] (1 - 744)<br>**PCOLN3**, GXL_167550, GeneID: 5119, Homo sapiens chr. 16<br>procollagen (type III) N-endopeptidase	E2FF-NFKB	245 - 125	(-)	
**GXP_200831** [GXP_200831] (1 - 783)<br>**GNPTG**, GXL_167578, GeneID: 84572, Homo sapiens chr. 16<br>N-acetylglucosamine-1-phosphate transferase, gamma subunit	E2FF-NFKB	431 - 318	(-)	
**GXP_200836** [GXP_200836] (1 - 853)<br>**MPV17L**, GXL_167582, GeneID: 255027, Homo sapiens chr. 16<br>MPV17 mitochondrial membrane protein-like	E2FF-NFKB	786 - 677	(-)	
**GXP_200845** [GXP_200845] (1 - 601)<br>**CLDN9**, GXL_167589, GeneID: 9080, Homo sapiens chr. 16<br>claudin 9	E2FF-NFKB	132 - 20	(-)	
**GXP_200846** [GXP_200846] (1 - 601)<br>**CLDN9**, GXL_167589, GeneID: 9080, Homo sapiens chr. 16<br>claudin 9	E2FF-NFKB	383 - 271	(-)	
**GXP_200863** [GXP_200863] (1 - 601)<br>**PRSS21**, GXL_167601, GeneID: 10942, Homo sapiens chr. 16<br>protease, serine, 21 (testisin)	E2FF-NFKB	45 - 158	(+)	
**GXP_910805** [GXP_910805] (1 - 601)<br>**PRDM7**, GXL_167612, GeneID: 11105, Homo sapiens chr. 16<br>PR domain containing 7	E2FF-NFKB	17 - 128	(+)	
**GXP_483214** [GXP_483214] (1 - 601)<br>**BAIAP3**, GXL_167631, GeneID: 8938, Homo sapiens chr. 16<br>BAI1-associated protein 3	E2FF-NFKB	409 - 286	(-)	
**GXP_200917** [GXP_200917] (1 - 1013)<br>**STUB1**, GXL_167650, GeneID: 10273, Homo sapiens chr. 16<br>STIP1 homology and U-box containing protein 1	E2FF-NFKB	863 - 974	(+)	
**GXP_644678** [GXP_644678] (1 - 601)<br>**STUB1**, GXL_167650, GeneID: 10273, Homo sapiens chr. 16<br>STIP1 homology and U-box containing protein 1	E2FF-NFKB	155 - 266	(+)	
	E2FF-NFKB	221 - 108	(-)	

**GXP_909919** [GXP_909919] (1 - 601)<br>**DNASE1**, GXL_167652, GeneID: 1773, Homo sapiens chr. 16<br>deoxyribonuclease I				
**GXP_200926** [GXP_200926] (1 - 845)<br>**METRN**, GXL_167659, GeneID: 79006, Homo sapiens chr. 16<br>meteorin, glial cell differentiation regulator	E2FF-NFKB	413 - 538	(+)	
**GXP_200931** [GXP_200931] (1 - 667)<br>**HBA1**, GXL_167664, GeneID: 3039, Homo sapiens chr. 16<br>hemoglobin, alpha 1	E2FF-NFKB	303 - 425	(+)	
	E2FF-NFKB	521 - 412	(-)	
**GXP_200942** [GXP_200942] (1 - 1053)<br>**ZNF205**, GXL_167675, GeneID: 7755, Homo sapiens chr. 16<br>zinc finger protein 205	E2FF-NFKB	659 - 787	(+)	
**GXP_200946** [GXP_200946] (1 - 601)<br>**HBA2**, GXL_167679, GeneID: 3040, Homo sapiens chr. 16<br>hemoglobin, alpha 2	E2FF-NFKB	275 - 397	(+)	
	E2FF-NFKB	493 - 384	(-)	
**GXP_203167** [GXP_203167] (1 - 833)<br>**ARL6**, GXL_169655, GeneID: 84100, Homo sapiens chr. 3<br>ADP-ribosylation factor-like 6	E2FF-NFKB	618 - 506	(-)	
**GXP_203172** [GXP_203172] (1 - 743)<br>**RBM6**, GXL_169658, GeneID: 10180, Homo sapiens chr. 3<br>RNA binding motif protein 6	E2FF-NFKB	497 - 620	(+)	
**GXP_919950** [GXP_919950] (1 - 601)<br>**SLC25A38**, GXL_169659, GeneID: 54977, Homo sapiens chr. 3<br>solute carrier family 25, member 38	E2FF-NFKB	548 - 425	(-)	
**GXP_203189** [GXP_203189] (1 - 829)<br>**MYRIP**, GXL_169667, GeneID: 25924, Homo sapiens chr. 3<br>myosin VIIA and Rab interacting protein	E2FF-NFKB	248 - 361	(+)	
**GXP_203200** [GXP_203200] (1 - 1248)<br>**PH-4**, GXL_169673, GeneID: 54681, Homo sapiens chr. 3<br>hypoxia-inducible factor prolyl 4-hydroxylase	E2FF-NFKB	740 - 864	(+)	
**GXP_203205** [GXP_203205] (1 - 727)<br>**CACNA2D3**, GXL_169675, GeneID: 55799, Homo sapiens chr. 3<br>calcium channel, voltage-dependent, alpha 2/delta 3 subunit	E2FF-NFKB	209 - 315	(+)	
**GXP_203220** [GXP_203220] (1 - 622)<br>**2'-PDE**, GXL_169684, GeneID: 201626, Homo sapiens chr. 3<br>2'-phosphodiesterase	E2FF-NFKB	491 - 619	(+)	
**GXP_203245** [GXP_203245] (1 - 601)<br>**VIPR1**, GXL_169692, GeneID: 7433, Homo sapiens chr. 3<br>vasoactive intestinal peptide receptor 1	E2FF-NFKB	565 - 449	(-)	
	E2FF-NFKB	305 - 188	(-)	

**GXP_203276** [GXP_203276] (1 - 612)<br>**PRKCD**, GXL_169713, GeneID: 5580, Homo sapiens chr. 3<br>protein kinase C, delta				
**GXP_203288** [GXP_203288] (1 - 618)<br>**KCTD6**, GXL_169722, GeneID: 200845, Homo sapiens chr. 3<br>potassium channel tetramerisation domain containing 6	E2FF-NFKB	419 - 526	(+)	
**GXP_920109** [GXP_920109] (1 - 601)<br>**RNF123**, GXL_169745, GeneID: 63891, Homo sapiens chr. 3<br>ring finger protein 123	E2FF-NFKB	405 - 294	(-)	
**GXP_203355** [GXP_203355] (1 - 1348)<br>**C3orf14**, GXL_169768, GeneID: 57415, Homo sapiens chr. 3<br>chromosome 3 open reading frame 14	E2FF-NFKB	437 - 320	(-)	
**GXP_203366** [GXP_203366] (1 - 971)<br>**SPCS1**, GXL_169774, GeneID: 28972, Homo sapiens chr. 3<br>signal peptidase complex subunit 1 homolog (S. cerevisiae)	E2FF-NFKB	266 - 140	(-)	
**GXP_203381** [GXP_203381] (1 - 608)<br>**ST3GAL6**, GXL_169786, GeneID: 10402, Homo sapiens chr. 3<br>ST3 beta-galactoside alpha-2,3-sialyltransferase 6	E2FF-NFKB	465 - 357	(-)	
**GXP_203386** [GXP_203386] (1 - 774)<br>**NIT2**, GXL_169789, GeneID: 56954, Homo sapiens chr. 3<br>nitrilase family, member 2	E2FF-NFKB	386 - 265	(-)	
**GXP_203407** [GXP_203407] (1 - 1020)<br>**PPM1M**, GXL_169805, GeneID: 132160, Homo sapiens chr. 3<br>protein phosphatase 1M (PP2C domain containing)	E2FF-NFKB	320 - 201	(-)	
**GXP_203448** [GXP_203448] (1 - 773)<br>**APPL1**, GXL_169837, GeneID: 26060, Homo sapiens chr. 3<br>adaptor protein, phosphotyrosine interaction, PH domain and leucine zipper containing 1	E2FF-NFKB	499 - 391	(-)	
**GXP_919998** [GXP_919998] (1 - 601)<br>**CCBP2**, GXL_169850, GeneID: 1238, Homo sapiens chr. 3<br>chemokine binding protein 2	E2FF-NFKB	472 - 587	(+)	
**GXP_203477** [GXP_203477] (1 - 1208)<br>**APEH**, GXL_169865, GeneID: 327, Homo sapiens chr. 3<br>N-acylaminoacyl-peptide hydrolase	E2FF-NFKB	978 - 1106	(+)	
**GXP_203481** [GXP_203481] (1 - 601)<br>**DAG1**, GXL_169869, GeneID: 1605, Homo sapiens chr. 3<br>dystroglycan 1 (dystrophin-associated glycoprotein 1)	E2FF-NFKB	449 - 331	(-)	
**GXP_905184** [GXP_905184] (1 - 601)<br>**QSER1**, GXL_170746, GeneID: 79832, Homo sapiens chr. 11<br>glutamine and serine rich 1	E2FF-NFKB	446 - 335	(-)	
	E2FF-NFKB	268 - 378	(+)	

**GXP_204599** [GXP_204599] (1 - 791)<br>**MADD**, GXL_170772, GeneID: 8567, Homo sapiens chr. 11<br>MAP-kinase activating death domain				
**GXP_640064** [GXP_640064] (1 - 1237)<br>**MARK2**, GXL_170773, GeneID: 2011, Homo sapiens chr. 11<br>MAP/microtubule affinity-regulating kinase 2	E2FF-NFKB	870 - 978	(+)	
**GXP_204624** [GXP_204624] (1 - 858)<br>**INCENP**, GXL_170787, GeneID: 3619, Homo sapiens chr. 11<br>inner centromere protein antigens 135/155kDa	E2FF-NFKB	712 - 603	(-)	
**GXP_905201** [GXP_905201] (1 - 601)<br>**NAT10**, GXL_170802, GeneID: 55226, Homo sapiens chr. 11<br>N-acetyltransferase 10	E2FF-NFKB	503 - 375	(-)	
**GXP_639980** [GXP_639980] (1 - 601)<br>**PGA3,LOC649034**, GXL_170826, GeneID: 643834,649034, Homo sapiens chr. 11<br>pepsinogen 3, group I (pepsinogen A); similar to Pepsin A precursor	E2FF-NFKB	396 - 284	(-)	
**GXP_204694** [GXP_204694] (1 - 654)<br>**PRDX5**, GXL_170835, GeneID: 25824, Homo sapiens chr. 11<br>peroxiredoxin 5	E2FF-NFKB	130 - 5	(-)	
**GXP_204697** [GXP_204697] (1 - 1075)<br>**CREB3L1**, GXL_170838, GeneID: 90993, Homo sapiens chr. 11<br>cAMP responsive element binding protein 3-like 1	E2FF-NFKB	72 - 193	(+)	
**GXP_204716** [GXP_204716] (1 - 601)<br>**GPR137**, GXL_170852, GeneID: 56834, Homo sapiens chr. 11<br>G protein-coupled receptor 137	E2FF-NFKB	403 - 281	(-)	
**GXP_905537** [GXP_905537] (1 - 601)<br>**GPR137**, GXL_170852, GeneID: 56834, Homo sapiens chr. 11<br>G protein-coupled receptor 137	E2FF-NFKB	562 - 440	(-)	
**GXP_204736** [GXP_204736] (1 - 1177)<br>**RCN1**, GXL_170868, GeneID: 5954, Homo sapiens chr. 11<br>reticulocalbin 1, EF-hand calcium binding domain	E2FF-NFKB	854 - 960	(+)	
**GXP_204758** [GXP_204758] (1 - 633)<br>**SLC35C1**, GXL_170883, GeneID: 55343, Homo sapiens chr. 11<br>solute carrier family 35, member C1	E2FF-NFKB	149 - 265	(+)	
**GXP_204762** [GXP_204762] (1 - 900)<br>**KCNK4**, GXL_170884, GeneID: 50801, Homo sapiens chr. 11<br>potassium channel, subfamily K, member 4	E2FF-NFKB	561 - 675	(+)	
**GXP_204776** [GXP_204776] (1 - 1119)<br>**STX3**, GXL_170894, GeneID: 6809, Homo sapiens chr. 11<br>syntaxin 3	E2FF-NFKB	802 - 928	(+)	
	E2FF-NFKB	422 - 300	(-)	

**GXP_905224** [GXP_905224] (1 - 601)<br>**FLJ14213**, GXL_170896, GeneID: 79899, Homo sapiens chr. 11<br>hypothetical protein FLJ14213				
**GXP_204804** [GXP_204804] (1 - 601)<br>**PRDM11**, GXL_170910, GeneID: 56981, Homo sapiens chr. 11<br>PR domain containing 11	E2FF-NFKB	296 - 168	(-)	
**GXP_204810** [GXP_204810] (1 - 874)<br>**CAPN1**, GXL_170916, GeneID: 823, Homo sapiens chr. 11<br>calpain 1, (mu/l) large subunit	E2FF-NFKB	246 - 353	(+)	
**GXP_905288** [GXP_905288] (1 - 601)<br>**KIAA0652**, GXL_170918, GeneID: 9776, Homo sapiens chr. 11<br>KIAA0652	E2FF-NFKB	181 - 309	(+)	
**GXP_905263** [GXP_905263] (1 - 601)<br>**PRDM11**, GXL_170928, GeneID: 56981, Homo sapiens chr. 11<br>PR domain containing 11	E2FF-NFKB	300 - 172	(-)	
**GXP_905264** [GXP_905264] (1 - 655)<br>**PRDM11**, GXL_170928, GeneID: 56981, Homo sapiens chr. 11<br>PR domain containing 11	E2FF-NFKB	202 - 88	(-)	
**GXP_204831** [GXP_204831] (1 - 711)<br>**STIP1**, GXL_170931, GeneID: 10963, Homo sapiens chr. 11<br>stress-induced-phosphoprotein 1 (Hsp70/Hsp90-organizing protein)	E2FF-NFKB	143 - 251	(+)	
**GXP_639970** [GXP_639970] (1 - 601)<br>**TMEM109,LOC728617,LOC731022**, GXL_170987, GeneID: 79073,728617,731022, Homo sapiens chr. 11<br>transmembrane protein 109; hypothetical protein LOC728617; hypothetical protein LOC731022	E2FF-NFKB	233 - 357	(+)	
**GXP_204922** [GXP_204922] (1 - 601)<br>**C3orf63**, GXL_171014, GeneID: 23272, Homo sapiens chr. 3<br>chromosome 3 open reading frame 63	E2FF-NFKB	360 - 249	(-)	
**GXP_204954** [GXP_204954] (1 - 739)<br>**RASSF1**, GXL_171034, GeneID: 11186, Homo sapiens chr. 3<br>Ras association (RalGDS/AF-6) domain family 1	E2FF-NFKB	596 - 707	(+)	
**GXP_205058** [GXP_205058] (1 - 720)<br>**ACTR8**, GXL_171089, GeneID: 93973, Homo sapiens chr. 3<br>ARP8 actin-related protein 8 homolog (yeast)	E2FF-NFKB	344 - 470	(+)	
**GXP_205071** [GXP_205071] (1 - 699)<br>**SFMBT1**, GXL_171096, GeneID: 51460, Homo sapiens chr. 3<br>Scm-like with four mbt domains 1	E2FF-NFKB	174 - 62	(-)	
**GXP_205119** [GXP_205119] (1 - 721)<br>**CBLB**, GXL_171124, GeneID: 868, Homo sapiens chr. 3<br>Cas-Br-M (murine) ecotropic retroviral transforming sequence b	E2FF-NFKB	467 - 340	(-)	
	E2FF-NFKB	239 - 133	(-)	

**GXP_920414** [GXP_920414] (1 - 601)<br>**CBLB**, GXL_171124, GeneID: 868, Homo sapiens chr. 3<br>Cas-Br-M (murine) ecotropic retroviral transforming sequence b				
**GXP_205148** [GXP_205148] (1 - 1116)<br>**GLT8D1**, GXL_171141, GeneID: 55830, Homo sapiens chr. 3<br>glycosyltransferase 8 domain containing 1	E2FF-NFKB	958 - 1084	(+)	
**GXP_205196** [GXP_205196] (1 - 845)<br>**TWF2**, GXL_171176, GeneID: 11344, Homo sapiens chr. 3<br>twinfilin, actin-binding protein, homolog 2 (Drosophila)	E2FF-NFKB	713 - 826	(+)	
**GXP_205245** [GXP_205245] (1 - 1270)<br>**PDZRN3**, GXL_171216, GeneID: 23024, Homo sapiens chr. 3<br>PDZ domain containing RING finger 3	E2FF-NFKB	397 - 279	(-)	
**GXP_205251** [GXP_205251] (1 - 602)<br>**DNASE1L3**, GXL_171219, GeneID: 1776, Homo sapiens chr. 3<br>deoxyribonuclease I-like 3	E2FF-NFKB	579 - 450	(-)	
**GXP_920217** [GXP_920217] (1 - 615)<br>**ARHGEF3**, GXL_171243, GeneID: 50650, Homo sapiens chr. 3<br>Rho guanine nucleotide exchange factor (GEF) 3	E2FF-NFKB	556 - 434	(-)	
**GXP_206912** [GXP_206912] (1 - 1472)<br>**LY6H**, GXL_172502, GeneID: 4062, Homo sapiens chr. 8<br>lymphocyte antigen 6 complex, locus H	E2FF-NFKB	883 - 994	(+)	
**GXP_661476** [GXP_661476] (1 - 601)<br>**LY6H**, GXL_172502, GeneID: 4062, Homo sapiens chr. 8<br>lymphocyte antigen 6 complex, locus H	E2FF-NFKB	447 - 558	(+)	
**GXP_926426** [GXP_926426] (1 - 601)<br>**LY6H**, GXL_172502, GeneID: 4062, Homo sapiens chr. 8<br>lymphocyte antigen 6 complex, locus H	E2FF-NFKB	12 - 123	(+)	
**GXP_926427** [GXP_926427] (1 - 601)<br>**LY6H**, GXL_172502, GeneID: 4062, Homo sapiens chr. 8<br>lymphocyte antigen 6 complex, locus H	E2FF-NFKB	403 - 514	(+)	
**GXP_926421** [GXP_926421] (1 - 601)<br>**CYP11B1**, GXL_172514, GeneID: 1584, Homo sapiens chr. 8<br>cytochrome P450, family 11, subfamily B, polypeptide 1	E2FF-NFKB	65 - 175	(+)	
**GXP_206973** [GXP_206973] (1 - 645)<br>**LRP12**, GXL_172532, GeneID: 29967, Homo sapiens chr. 8<br>low density lipoprotein-related protein 12	E2FF-NFKB	274 - 147	(-)	
**GXP_207013** [GXP_207013] (1 - 982)<br>**TNFRSF11B**, GXL_172550, GeneID: 4982, Homo sapiens chr. 8<br>tumor necrosis factor receptor superfamily, member 11b (osteoprotegerin)	E2FF-NFKB	212 - 325	(+)	
	E2FF-NFKB	529 - 409	(-)	

**GXP_661194** [GXP_661194] (1 - 601)<br>**ZFAND1**, GXL_172582, GeneID: 79752, Homo sapiens chr. 8<br>zinc finger, AN1-type domain 1				
**GXP_207083** [GXP_207083] (1 - 794)<br>**RPESP**, GXL_172592, GeneID: 157869, Homo sapiens chr. 8<br>RPE-spondin	E2FF-NFKB	406 - 512	(+)	
**GXP_207096** [GXP_207096] (1 - 601)<br>**TSNARE1**, GXL_172600, GeneID: 203062, Homo sapiens chr. 8<br>t-SNARE domain containing 1	E2FF-NFKB	540 - 411	(-)	
**GXP_207113** [GXP_207113] (1 - 846)<br>**RRM2B**, GXL_172609, GeneID: 50484, Homo sapiens chr. 8<br>ribonucleotide reductase M2 B (TP53 inducible)	E2FF-NFKB	264 - 384	(+)	
**GXP_207152** [GXP_207152] (1 - 644)<br>**TPD52**, GXL_172636, GeneID: 7163, Homo sapiens chr. 8<br>tumor protein D52	E2FF-NFKB	121 - 227	(+)	
**GXP_207164** [GXP_207164] (1 - 1370)<br>**TMEM64**, GXL_172647, GeneID: 169200, Homo sapiens chr. 8<br>transmembrane protein 64	E2FF-NFKB	907 - 800	(-)	
**GXP_207181** [GXP_207181] (1 - 601)<br>**FAM84B**, GXL_172660, GeneID: 157638, Homo sapiens chr. 8<br>family with sequence similarity 84, member B	E2FF-NFKB	587 - 470	(-)	
**GXP_493058** [GXP_493058] (1 - 818)<br>**KLF10**, GXL_172715, GeneID: 7071, Homo sapiens chr. 8<br>Kruppel-like factor 10	E2FF-NFKB	223 - 343	(+)	
**GXP_207258** [GXP_207258] (1 - 876)<br>**SLA**, GXL_172724, GeneID: 6503, Homo sapiens chr. 8<br>Src-like-adaptor	E2FF-NFKB	715 - 835	(+)	
**GXP_207315** [GXP_207315] (1 - 673)<br>**C14orf140**, GXL_172777, GeneID: 79696, Homo sapiens chr. 14<br>chromosome 14 open reading frame 140	E2FF-NFKB	503 - 627	(+)	
**GXP_643588** [GXP_643588] (1 - 601)<br>**KIF26A**, GXL_172781, GeneID: 26153, Homo sapiens chr. 14<br>kinesin family member 26A	E2FF-NFKB	272 - 144	(-)	
**GXP_207327** [GXP_207327] (1 - 601)<br>**C14orf159**, GXL_172782, GeneID: 80017, Homo sapiens chr. 14<br>chromosome 14 open reading frame 159	E2FF-NFKB	317 - 432	(+)	
**GXP_207359** [GXP_207359] (1 - 770)<br>**LIN52**, GXL_172796, GeneID: 91750, Homo sapiens chr. 14<br>lin-52 homolog (C. elegans)	E2FF-NFKB	635 - 762	(+)	
	E2FF-NFKB	203 - 94	(-)	

**GXP_207368** [GXP_207368] (1 - 601)<br>**MEG3**, GXL_172801, GeneID: 55384, Homo sapiens chr. 14<br>maternally expressed 3				
**GXP_207372** [GXP_207372] (1 - 605)<br>**ADSSL1**, GXL_172802, GeneID: 122622, Homo sapiens chr. 14<br>adenylosuccinate synthase like 1	E2FF-NFKB	264 - 391	(+)	
**GXP_207387** [GXP_207387] (1 - 601)<br>**PAPOLA**, GXL_172809, GeneID: 10914, Homo sapiens chr. 14<br>poly(A) polymerase alpha	E2FF-NFKB	183 - 299	(+)	
**GXP_207402** [GXP_207402] (1 - 630)<br>**FCF1**, GXL_172818, GeneID: 51077, Homo sapiens chr. 14<br>FCF1 small subunit (SSU) processome component homolog (S. cerevisiae)	E2FF-NFKB	552 - 445	(-)	
**GXP_207406** [GXP_207406] (1 - 601)<br>**EIF5**, GXL_172822, GeneID: 1983, Homo sapiens chr. 14<br>eukaryotic translation initiation factor 5	E2FF-NFKB	143 - 34	(-)	
**GXP_908744** [GXP_908744] (1 - 816)<br>**EIF5**, GXL_172822, GeneID: 1983, Homo sapiens chr. 14<br>eukaryotic translation initiation factor 5	E2FF-NFKB	785 - 676	(-)	
**GXP_908745** [GXP_908745] (1 - 601)<br>**EIF5**, GXL_172822, GeneID: 1983, Homo sapiens chr. 14<br>eukaryotic translation initiation factor 5	E2FF-NFKB	238 - 129	(-)	
**GXP_207495** [GXP_207495] (1 - 601)<br>**TRAF3**, GXL_172864, GeneID: 7187, Homo sapiens chr. 14<br>TNF receptor-associated factor 3	E2FF-NFKB	137 - 259	(+)	
**GXP_207513** [GXP_207513] (1 - 601)<br>**NRXN3**, GXL_172873, GeneID: 9369, Homo sapiens chr. 14<br>neurexin 3	E2FF-NFKB	286 - 401	(+)	
**GXP_207549** [GXP_207549] (1 - 602)<br>**JDP2**, GXL_172900, GeneID: 122953, Homo sapiens chr. 14<br>jun dimerization protein 2	E2FF-NFKB	434 - 554	(+)	
**GXP_908503** [GXP_908503] (1 - 601)<br>**JDP2**, GXL_172900, GeneID: 122953, Homo sapiens chr. 14<br>jun dimerization protein 2	E2FF-NFKB	403 - 531	(+)	
**GXP_908740** [GXP_908740] (1 - 601)<br>**TNFAIP2**, GXL_172903, GeneID: 7127, Homo sapiens chr. 14<br>tumor necrosis factor, alpha-induced protein 2	E2FF-NFKB	363 - 478	(+)	
**GXP_207560** [GXP_207560] (1 - 928)<br>**C14orf166B**, GXL_172910, GeneID: 145497, Homo sapiens chr. 14<br>chromosome 14 open reading frame 166B	E2FF-NFKB	219 - 327	(+)	
	E2FF-NFKB	205 - 85	(-)	

**GXP_207588** [GXP_207588] (1 - 755)<br>**CYP46A1**, GXL_172929, GeneID: 10858, Homo sapiens chr. 14<br>cytochrome P450, family 46, subfamily A, polypeptide 1				
**GXP_207649** [GXP_207649] (1 - 625)<br>**ZNF410**, GXL_172983, GeneID: 57862, Homo sapiens chr. 14<br>zinc finger protein 410	E2FF-NFKB	472 - 348	(-)	
**GXP_208673** [GXP_208673] (1 - 624)<br>**ROBO3**, GXL_173968, GeneID: 64221, Homo sapiens chr. 11<br>roundabout, axon guidance receptor, homolog 3 (Drosophila)	E2FF-NFKB	179 - 289	(+)	
**GXP_208694** [GXP_208694] (1 - 633)<br>**MIZF**, GXL_173982, GeneID: 25988, Homo sapiens chr. 11<br>MBD2-interacting zinc finger	E2FF-NFKB	297 - 188	(-)	
**GXP_906219** [GXP_906219] (1 - 601)<br>**FOXRED1**, GXL_173990, GeneID: 55572, Homo sapiens chr. 11<br>FAD-dependent oxidoreductase domain containing 1	E2FF-NFKB	577 - 456	(-)	
**GXP_208712** [GXP_208712] (1 - 1216)<br>**BCDO2**, GXL_173993, GeneID: 83875, Homo sapiens chr. 11<br>beta-carotene dioxygenase 2	E2FF-NFKB	807 - 694	(-)	
**GXP_208718** [GXP_208718] (1 - 646)<br>**IL10RA**, GXL_173996, GeneID: 3587, Homo sapiens chr. 11<br>interleukin 10 receptor, alpha	E2FF-NFKB	176 - 56	(-)	
**GXP_906202** [GXP_906202] (1 - 601)<br>**STT3A**, GXL_174016, GeneID: 3703, Homo sapiens chr. 11<br>STT3, subunit of the oligosaccharyltransferase complex, homolog A (S. cerevisiae)	E2FF-NFKB	317 - 430	(+)	
**GXP_906207** [GXP_906207] (1 - 601)<br>**STT3A**, GXL_174016, GeneID: 3703, Homo sapiens chr. 11<br>STT3, subunit of the oligosaccharyltransferase complex, homolog A (S. cerevisiae)	E2FF-NFKB	232 - 116	(-)	
**GXP_640822** [GXP_640822] (1 - 639)<br>**FAM118B**, GXL_174038, GeneID: 79607, Homo sapiens chr. 11<br>family with sequence similarity 118, member B	E2FF-NFKB	551 - 422	(-)	
**GXP_208783** [GXP_208783] (1 - 650)<br>**SDHD**, GXL_174040, GeneID: 6392, Homo sapiens chr. 11<br>succinate dehydrogenase complex, subunit D, integral membrane protein	E2FF-NFKB	499 - 623	(+)	
**GXP_208787** [GXP_208787] (1 - 602)<br>**CHEK1**, GXL_174043, GeneID: 1111, Homo sapiens chr. 11<br>CHK1 checkpoint homolog (S. pombe)	E2FF-NFKB	349 - 233	(-)	
**GXP_208795** [GXP_208795] (1 - 788)<br>**GRAMD1B**, GXL_174048, GeneID: 57476, Homo sapiens chr. 11<br>GRAM domain containing 1B	E2FF-NFKB	203 - 74	(-)	
	E2FF-NFKB	487 - 599	(+)	

**GXP_208805** [GXP_208805] (1 - 616)<br>**TBRG1**, GXL_174055, GeneID: 84897, Homo sapiens chr. 11<br>transforming growth factor beta regulator 1				
**GXP_208811** [GXP_208811] (1 - 738)<br>**DDX25**, GXL_174058, GeneID: 29118, Homo sapiens chr. 11<br>DEAD (Asp-Glu-Ala-Asp) box polypeptide 25	E2FF-NFKB	388 - 269	(-)	
**GXP_208847** [GXP_208847] (1 - 601)<br>**FLJ34521**, GXL_174092, GeneID: 646383, Homo sapiens chr. 11<br>hypothetical protein LOC646383	E2FF-NFKB	91 - 207	(+)	
**GXP_208860** [GXP_208860] (1 - 934)<br>**FLI1**, GXL_174105, GeneID: 2313, Homo sapiens chr. 11<br>Friend leukemia virus integration 1	E2FF-NFKB	441 - 551	(+)	
**GXP_208873** [GXP_208873] (1 - 601)<br>**C11orf37**, GXL_174118, GeneID: 440072, Homo sapiens chr. 11<br>chromosome 11 open reading frame 37	E2FF-NFKB	504 - 398	(-)	
**GXP_656260** [GXP_656260] (1 - 601)<br>**GALNT7**, GXL_175059, GeneID: 51809, Homo sapiens chr. 4<br>UDP-N-acetyl-alpha-D-galactosamine:polypeptide N-acetylgalactosaminyltransferase 7 (GalNAc-T7)	E2FF-NFKB	22 - 146	(+)	
**GXP_210124** [GXP_210124] (1 - 715)<br>**SPCS3**, GXL_175166, GeneID: 60559, Homo sapiens chr. 4<br>signal peptidase complex subunit 3 homolog (S. cerevisiae)	E2FF-NFKB	414 - 299	(-)	
**GXP_912032** [GXP_912032] (1 - 601)<br>**SLC26A11,LOC652834**, GXL_175475, GeneID: 284129,652834, Homo sapiens chr. 17<br>solute carrier family 26, member 11; similar to solute carrier family 26, member 11	E2FF-NFKB	350 - 232	(-)	
**GXP_210495** [GXP_210495] (1 - 601)<br>**ASPSCR1,LOC648581**, GXL_175491, GeneID: 79058,648581, Homo sapiens chr. 17<br>alveolar soft part sarcoma chromosome region, candidate 1; similar to alveolar soft part sarcoma chromosome region, candidate 1	E2FF-NFKB	483 - 375	(-)	
**GXP_912013** [GXP_912013] (1 - 601)<br>**C1QTNF1**, GXL_175503, GeneID: 114897, Homo sapiens chr. 17<br>C1q and tumor necrosis factor related protein 1	E2FF-NFKB	330 - 457	(+)	
**GXP_210545** [GXP_210545] (1 - 601)<br>**SYNGR2**, GXL_175522, GeneID: 9144, Homo sapiens chr. 17<br>synaptogyrin 2	E2FF-NFKB	370 - 251	(-)	
**GXP_210560** [GXP_210560] (1 - 601)<br>**CBX2**, GXL_175536, GeneID: 84733, Homo sapiens chr. 17<br>chromobox homolog 2 (Pc class homolog, Drosophila)	E2FF-NFKB	147 - 275	(+)	
**GXP_484864** [GXP_484864] (1 - 601)<br>**CBX2**, GXL_175536, GeneID: 84733, Homo sapiens chr. 17<br>chromobox homolog 2 (Pc class homolog, Drosophila)	E2FF-NFKB	76 - 191	(+)	
	E2FF-NFKB	381 - 257	(-)	

**GXP_912017** [GXP_912017] (1 - 601)<br>**FLJ21865**, GXL_175545, GeneID: 64772, Homo sapiens chr. 17<br>endo-beta-N-acetylglucosaminidase				
**GXP_210962** [GXP_210962] (1 - 601)<br>**CD99**, GXL_175882, GeneID: 4267, Homo sapiens chr. Y<br>CD99 molecule	E2FF-NFKB	488 - 372	(-)	
**GXP_210980** [GXP_210980] (1 - 825)<br>**CD24,LOC728512**, GXL_175896, GeneID: 934,728512, Homo sapiens chr. Y<br>CD24 molecule;hypothetical protein LOC728512	E2FF-NFKB	475 - 367	(-)	
**GXP_210986** [GXP_210986] (1 - 601)<br>**ZFY**, GXL_175902, GeneID: 7544, Homo sapiens chr. Y<br>zinc finger protein, Y-linked	E2FF-NFKB	52 - 160	(+)	
	E2FF-NFKB	569 - 440	(-)	
**GXP_928429** [GXP_928429] (1 - 866)<br>**ZFY**, GXL_175902, GeneID: 7544, Homo sapiens chr. Y<br>zinc finger protein, Y-linked	E2FF-NFKB	446 - 554	(+)	
**GXP_211192** [GXP_211192] (1 - 660)<br>**MX1**, GXL_176084, GeneID: 4599, Homo sapiens chr. 21<br>myxovirus (influenza virus) resistance 1, interferon-inducible protein p78 (mouse)	E2FF-NFKB	427 - 313	(-)	
**GXP_917221** [GXP_917221] (1 - 601)<br>**MX1**, GXL_176084, GeneID: 4599, Homo sapiens chr. 21<br>myxovirus (influenza virus) resistance 1, interferon-inducible protein p78 (mouse)	E2FF-NFKB	312 - 198	(-)	
**GXP_211199** [GXP_211199] (1 - 601)<br>**ABCG1**, GXL_176087, GeneID: 9619, Homo sapiens chr. 21<br>ATP-binding cassette, sub-family G (WHITE), member 1	E2FF-NFKB	551 - 441	(-)	
**GXP_211201** [GXP_211201] (1 - 601)<br>**ABCG1**, GXL_176087, GeneID: 9619, Homo sapiens chr. 21<br>ATP-binding cassette, sub-family G (WHITE), member 1	E2FF-NFKB	555 - 445	(-)	
**GXP_917229** [GXP_917229] (1 - 601)<br>**ABCG1**, GXL_176087, GeneID: 9619, Homo sapiens chr. 21<br>ATP-binding cassette, sub-family G (WHITE), member 1	E2FF-NFKB	536 - 419	(-)	
**GXP_211214** [GXP_211214] (1 - 670)<br>**NDUFV3**, GXL_176095, GeneID: 4731, Homo sapiens chr. 21<br>NADH dehydrogenase (ubiquinone) flavoprotein 3, 10kDa	E2FF-NFKB	531 - 425	(-)	
**GXP_211276** [GXP_211276] (1 - 776)<br>**TMEM1**, GXL_176131, GeneID: 7109, Homo sapiens chr. 21<br>transmembrane protein 1	E2FF-NFKB	474 - 353	(-)	
**GXP_211283** [GXP_211283] (1 - 608)<br>**COL6A2**, GXL_176138, GeneID: 1292, Homo sapiens chr. 21<br>collagen, type VI, alpha 2	E2FF-NFKB	10 - 129	(+)	
	E2FF-NFKB	439 - 331	(-)	

**GXP_211294** [GXP_211294] (1 - 606)<br>**ADARB1**, GXL_176147, GeneID: 104, Homo sapiens chr. 21<br>adenosine deaminase, RNA-specific, B1 (RED1 homolog rat)				
**GXP_919673** [GXP_919673] (1 - 601)<br>**D2HGDH**, GXL_176780, GeneID: 728294, Homo sapiens chr. 2<br>D-2-hydroxyglutarate dehydrogenase	E2FF-NFKB<br>E2FF-NFKB	216 - 331<br>440 - 318	(+)<br>(-)	
**GXP_211968** [GXP_211968] (1 - 601)<br>**BOK**, GXL_176789, GeneID: 666, Homo sapiens chr. 2<br>BCL2-related ovarian killer	E2FF-NFKB	239 - 362	(+)	
**GXP_214812** [GXP_214812] (1 - 677)<br>**F10**, GXL_179557, GeneID: 2159, Homo sapiens chr. 13<br>coagulation factor X	E2FF-NFKB	193 - 318	(+)	
**GXP_920702** [GXP_920702] (1 - 629)<br>**TXNDC6**, GXL_181740, GeneID: 347736, Homo sapiens chr. 3<br>thioredoxin domain containing 6	E2FF-NFKB	363 - 490	(+)	
**GXP_217292** [GXP_217292] (1 - 601)<br>**KLF15**, GXL_181742, GeneID: 28999, Homo sapiens chr. 3<br>Kruppel-like factor 15	E2FF-NFKB	402 - 273	(-)	
**GXP_217294** [GXP_217294] (1 - 753)<br>**DBR1**, GXL_181744, GeneID: 51163, Homo sapiens chr. 3<br>debranching enzyme homolog 1 (S. cerevisiae)	E2FF-NFKB	211 - 96	(-)	
**GXP_920609** [GXP_920609] (1 - 601)<br>**MGLL**, GXL_181766, GeneID: 11343, Homo sapiens chr. 3<br>monoglyceride lipase	E2FF-NFKB	447 - 318	(-)	
**GXP_217348** [GXP_217348] (1 - 706)<br>**RPN1**, GXL_181771, GeneID: 6184, Homo sapiens chr. 3<br>ribophorin I	E2FF-NFKB	585 - 474	(-)	
**GXP_217362** [GXP_217362] (1 - 924)<br>**IFT80**, GXL_181782, GeneID: 57560, Homo sapiens chr. 3<br>intraflagellar transport 80 homolog (Chlamydomonas)	E2FF-NFKB	782 - 898	(+)	
**GXP_217367** [GXP_217367] (1 - 839)<br>**RARRES1**, GXL_181785, GeneID: 5918, Homo sapiens chr. 3<br>retinoic acid receptor responder (tazarotene induced) 1	E2FF-NFKB	517 - 397	(-)	
**GXP_217404** [GXP_217404] (1 - 758)<br>**CCDC14**, GXL_181808, GeneID: 64770, Homo sapiens chr. 3<br>coiled-coil domain containing 14	E2FF-NFKB	245 - 357	(+)	
**GXP_920680** [GXP_920680] (1 - 926)<br>**ANAPC13**, GXL_181809, GeneID: 25847, Homo sapiens chr. 3<br>anaphase promoting complex subunit 13	E2FF-NFKB	123 - 240	(+)	
	E2FF-NFKB	301 - 194	(-)	

**GXP_217476** [GXP_217476] (1 - 768)<br>**C3orf25**, GXL_181847, GeneID: 90288, Homo sapiens chr. 3<br>chromosome 3 open reading frame 25				
**GXP_217484** [GXP_217484] (1 - 1028)<br>**TNIK**, GXL_181851, GeneID: 23043, Homo sapiens chr. 3<br>TRAF2 and NCK interacting kinase	E2FF-NFKB	819 - 946	(+)	
**GXP_217550** [GXP_217550] (1 - 1214)<br>**CCNL1**, GXL_181894, GeneID: 57018, Homo sapiens chr. 3<br>cyclin L1	E2FF-NFKB	979 - 1095	(+)	
**GXP_217584** [GXP_217584] (1 - 601)<br>**GNB4**, GXL_181919, GeneID: 59345, Homo sapiens chr. 3<br>guanine nucleotide binding protein (G protein), beta polypeptide 4	E2FF-NFKB	402 - 509	(+)	
**GXP_217592** [GXP_217592] (1 - 605)<br>**DCUN1D1**, GXL_181924, GeneID: 54165, Homo sapiens chr. 3<br>DCN1, defective in cullin neddylation 1, domain containing 1 (S. cerevisiae)	E2FF-NFKB	238 - 357	(+)	
	E2FF-NFKB	431 - 325	(-)	
**GXP_218137** [GXP_218137] (1 - 843)<br>**PBEF1**, GXL_182306, GeneID: 10135, Homo sapiens chr. 7<br>pre-B-cell colony enhancing factor 1	E2FF-NFKB	169 - 277	(+)	
	E2FF-NFKB	515 - 396	(-)	
**GXP_218188** [GXP_218188] (1 - 614)<br>**DOCK4**, GXL_182340, GeneID: 9732, Homo sapiens chr. 7<br>dedicator of cytokinesis 4	E2FF-NFKB	153 - 279	(+)	
**GXP_218201** [GXP_218201] (1 - 677)<br>**KIAA0738,LOC647624**, GXL_182348, GeneID: 9747,647624, Homo sapiens chr. 7<br>KIAA0738 gene product; similar to KIAA0738 gene product	E2FF-NFKB	343 - 451	(+)	
**GXP_925442** [GXP_925442] (1 - 625)<br>**CNOT4**, GXL_182357, GeneID: 4850, Homo sapiens chr. 7<br>CCR4-NOT transcription complex, subunit 4	E2FF-NFKB	514 - 398	(-)	
**GXP_218240** [GXP_218240] (1 - 747)<br>**C7orf49**, GXL_182371, GeneID: 78996, Homo sapiens chr. 7<br>chromosome 7 open reading frame 49	E2FF-NFKB	631 - 515	(-)	
**GXP_218245** [GXP_218245] (1 - 604)<br>**TMEM176B**, GXL_182373, GeneID: 28959, Homo sapiens chr. 7<br>transmembrane protein 176B	E2FF-NFKB	384 - 267	(-)	
**GXP_218322** [GXP_218322] (1 - 601)<br>**COG5**, GXL_182420, GeneID: 10466, Homo sapiens chr. 7<br>component of oligomeric golgi complex 5	E2FF-NFKB	408 - 290	(-)	
**GXP_218344** [GXP_218344] (1 - 601)<br>**SRPK2**, GXL_182432, GeneID: 6733, Homo sapiens chr. 7<br>SFRS protein kinase 2	E2FF-NFKB	79 - 192	(+)	
	E2FF-NFKB	147 - 31	(-)	

**GXP_218365** [GXP_218365] (1 - 1167)<br>**ZNF467**, GXL_182446, GeneID: 168544, Homo sapiens chr. 7<br>zinc finger protein 467				
**GXP_660157** [GXP_660157] (1 - 601)<br>**CTTNBP2**, GXL_182447, GeneID: 83992, Homo sapiens chr. 7<br>cortactin binding protein 2	E2FF-NFKB	192 - 79	(-)	
**GXP_218415** [GXP_218415] (1 - 911)<br>**PODXL**, GXL_182484, GeneID: 5420, Homo sapiens chr. 7<br>podocalyxin-like	E2FF-NFKB	343 - 220	(-)	
**GXP_925370** [GXP_925370] (1 - 601)<br>**ZNF800**, GXL_182494, GeneID: 168850, Homo sapiens chr. 7<br>zinc finger protein 800	E2FF-NFKB	167 - 38	(-)	
**GXP_220852** [GXP_220852] (1 - 1002)<br>**HNRPL**, GXL_184286, GeneID: 3191, Homo sapiens chr. 19<br>heterogeneous nuclear ribonucleoprotein L	E2FF-NFKB	247 - 127	(-)	
**GXP_648676** [GXP_648676] (1 - 601)<br>**HNRPL**, GXL_184286, GeneID: 3191, Homo sapiens chr. 19<br>heterogeneous nuclear ribonucleoprotein L	E2FF-NFKB	198 - 313	(+)	
**GXP_220877** [GXP_220877] (1 - 601)<br>**ZNF585B,ZNF585A**, GXL_184299, GeneID: 92285,199704, Homo sapiens chr. 19<br>zinc finger protein 585B;zinc finger protein 585A	E2FF-NFKB	505 - 388	(-)	
**GXP_220895** [GXP_220895] (1 - 602)<br>**WDR87**, GXL_184305, GeneID: 83889, Homo sapiens chr. 19<br>WD repeat domain 87	E2FF-NFKB	412 - 536	(+)	
**GXP_220901** [GXP_220901] (1 - 710)<br>**PBX4**, GXL_184310, GeneID: 80714, Homo sapiens chr. 19<br>pre-B-cell leukemia homeobox 4	E2FF-NFKB	508 - 383	(-)	
**GXP_220939** [GXP_220939] (1 - 826)<br>**CALR3**, GXL_184332, GeneID: 125972, Homo sapiens chr. 19<br>calreticulin 3	E2FF-NFKB	662 - 552	(-)	
**GXP_220943** [GXP_220943] (1 - 601)<br>**ZNF780A**, GXL_184334, GeneID: 284323, Homo sapiens chr. 19<br>zinc finger protein 780A	E2FF-NFKB	253 - 366	(+)	
**GXP_220944** [GXP_220944] (1 - 601)<br>**ZNF780A**, GXL_184334, GeneID: 284323, Homo sapiens chr. 19<br>zinc finger protein 780A	E2FF-NFKB	203 - 316	(+)	
**GXP_648369** [GXP_648369] (1 - 601)<br>**CRLF1**, GXL_184383, GeneID: 9244, Homo sapiens chr. 19<br>cytokine receptor-like factor 1	E2FF-NFKB	307 - 419	(+)	
	E2FF-NFKB	128 - 253	(+)	

**GXP_221049** [GXP_221049] (1 - 601)<br>**ZNF573**, GXL_184392, GeneID: 126231, Homo sapiens chr. 19<br>zinc finger protein 573				
**GXP_221050** [GXP_221050] (1 - 687)<br>**GMIP**, GXL_184393, GeneID: 51291, Homo sapiens chr. 19<br>GEM interacting protein	E2FF-NFKB	559 - 671	(+)	
**GXP_221054** [GXP_221054] (1 - 629)<br>**ZNF599**, GXL_184397, GeneID: 148103, Homo sapiens chr. 19<br>zinc finger protein 599	E2FF-NFKB	600 - 492	(-)	
**GXP_221100** [GXP_221100] (1 - 603)<br>**PEPD**, GXL_184435, GeneID: 5184, Homo sapiens chr. 19<br>peptidase D	E2FF-NFKB	442 - 319	(-)	
**GXP_912968** [GXP_912968] (1 - 650)<br>**SLC1A6**, GXL_184446, GeneID: 6511, Homo sapiens chr. 19<br>solute carrier family 1 (high affinity aspartate/glutamate transporter), member 6	E2FF-NFKB	575 - 466	(-)	
**GXP_221123** [GXP_221123] (1 - 910)<br>**PAF1**, GXL_184455, GeneID: 54623, Homo sapiens chr. 19<br>Paf1, RNA polymerase II associated factor, homolog (S. cerevisiae)	E2FF-NFKB	820 - 705	(-)	
**GXP_221146** [GXP_221146] (1 - 1318)<br>**FKBP8**, GXL_184470, GeneID: 23770, Homo sapiens chr. 19<br>FK506 binding protein 8, 38kDa	E2FF-NFKB	519 - 638	(+)	
**GXP_912977** [GXP_912977] (1 - 617)<br>**BRD4**, GXL_184507, GeneID: 23476, Homo sapiens chr. 19<br>bromodomain containing 4	E2FF-NFKB	146 - 270	(+)	
**GXP_221623** [GXP_221623] (1 - 715)<br>**OLFML2A**, GXL_184892, GeneID: 169611, Homo sapiens chr. 9<br>olfactomedin-like 2A	E2FF-NFKB	368 - 242	(-)	
**GXP_221628** [GXP_221628] (1 - 897)<br>**NTNG2**, GXL_184894, GeneID: 84628, Homo sapiens chr. 9<br>netrin G2	E2FF-NFKB	866 - 754	(-)	
**GXP_221657** [GXP_221657] (1 - 795)<br>**PRRX2**, GXL_184906, GeneID: 51450, Homo sapiens chr. 9<br>paired related homeobox 2	E2FF-NFKB	399 - 274	(-)	
**GXP_927452** [GXP_927452] (1 - 1158)<br>**MGC59937,LOC653325**, GXL_184909, GeneID: 375791,653325, Homo sapiens chr. 9<br>Similar to RIKEN cDNA 2310002J15 gene; hypothetical LOC653325	E2FF-NFKB	1003 - 1128	(+)	
**GXP_221685** [GXP_221685] (1 - 776)<br>**FREQ**, GXL_184917, GeneID: 23413, Homo sapiens chr. 9<br>frequenin homolog (Drosophila)	E2FF-NFKB	637 - 751	(+)	
	E2FF-NFKB	601 - 484	(-)	

**GXP_221706** [GXP_221706] (1 - 601)<br>**FLJ45224**, GXL_184926, GeneID: 401562, Homo sapiens chr. 9<br>FLJ45224 protein				
**GXP_221714** [GXP_221714] (1 - 652)<br>**FPGS**, GXL_184930, GeneID: 2356, Homo sapiens chr. 9<br>folylpolyglutamate synthase	E2FF-NFKB	415 - 286	(-)	
**GXP_927193** [GXP_927193] (1 - 601)<br>**PTGS1**, GXL_184939, GeneID: 5742, Homo sapiens chr. 9<br>prostaglandin-endoperoxide synthase 1 (prostaglandin G/H synthase and cyclooxygenase)	E2FF-NFKB	122 - 2	(-)	
**GXP_221766** [GXP_221766] (1 - 824)<br>**ADAMTSL2,LOC653348**, GXL_184958, GeneID: 9719,653348, Homo sapiens chr. 9<br>ADAMTS-like 2;similar to ADAMTS-like 2	E2FF-NFKB	462 - 589	(+)	
**GXP_221775** [GXP_221775] (1 - 1114)<br>**PHYHD1**, GXL_184962, GeneID: 254295, Homo sapiens chr. 9<br>phytanoyl-CoA dioxygenase domain containing 1	E2FF-NFKB	137 - 243	(+)	
**GXP_927242** [GXP_927242] (1 - 601)<br>**LMX1B**, GXL_184970, GeneID: 4010, Homo sapiens chr. 9<br>LIM homeobox transcription factor 1, beta	E2FF-NFKB	110 - 238	(+)	
**GXP_221790** [GXP_221790] (1 - 860)<br>**TLR4**, GXL_184971, GeneID: 7099, Homo sapiens chr. 9<br>toll-like receptor 4	E2FF-NFKB	266 - 388	(+)	
**GXP_221800** [GXP_221800] (1 - 1349)<br>**LHX2**, GXL_184978, GeneID: 9355, Homo sapiens chr. 9<br>LIM homeobox 2	E2FF-NFKB	1010 - 895	(-)	
**GXP_221801** [GXP_221801] (1 - 601)<br>**LHX2**, GXL_184978, GeneID: 9355, Homo sapiens chr. 9<br>LIM homeobox 2	E2FF-NFKB	263 - 148	(-)	
**GXP_927296** [GXP_927296] (1 - 878)<br>**LRRC8A**, GXL_184997, GeneID: 56262, Homo sapiens chr. 9<br>leucine rich repeat containing 8 family, member A	E2FF-NFKB	269 - 390	(+)	
**GXP_221838** [GXP_221838] (1 - 601)<br>**ODF2**, GXL_184998, GeneID: 4957, Homo sapiens chr. 9<br>outer dense fiber of sperm tails 2	E2FF-NFKB	363 - 253	(-)	
**GXP_927278** [GXP_927278] (1 - 746)<br>**ODF2**, GXL_184998, GeneID: 4957, Homo sapiens chr. 9<br>outer dense fiber of sperm tails 2	E2FF-NFKB	212 - 102	(-)	
**GXP_221977** [GXP_221977] (1 - 1291)<br>**CDK9**, GXL_185105, GeneID: 1025, Homo sapiens chr. 9<br>cyclin-dependent kinase 9 (CDC2-related kinase)	E2FF-NFKB	530 - 403	(-)	
	E2FF-NFKB	368 - 240	(-)	

**GXP_226458** [GXP_226458] (1 - 631)<br>**ZNF615**, GXL_188965, GeneID: 284370, Homo sapiens chr. 19<br>zinc finger protein 615				
**GXP_913624** [GXP_913624] (1 - 717)<br>**PIH1D1**, GXL_188969, GeneID: 55011, Homo sapiens chr. 19<br>PIH1 domain containing 1	E2FF-NFKB	552 - 444	(-)	
**GXP_226474** [GXP_226474] (1 - 1169)<br>**NUP62,IL4I1**, GXL_188977, GeneID: 23636,259307, Homo sapiens chr. 19<br>nucleoporin 62kDa;interleukin 4 induced 1	E2FF-NFKB	172 - 47	(-)	
**GXP_226510** [GXP_226510] (1 - 625)<br>**CNFN**, GXL_188999, GeneID: 84518, Homo sapiens chr. 19<br>cornifelin	E2FF-NFKB	274 - 386	(+)	
**GXP_226536** [GXP_226536] (1 - 1112)<br>**FLJ39303,LOC729452,LOC732176**, GXL_189018, GeneID: 400706,729452,732176, Homo sapiens chr. 19<br>hypothetical gene supported by AK096622; similar to Y8A9A.2;similar to Y8A9A.2	E2FF-NFKB	261 - 373	(+)	
**GXP_913534** [GXP_913534] (1 - 601)<br>**STRN4**, GXL_189024, GeneID: 29888, Homo sapiens chr. 19<br>striatin, calmodulin binding protein 4	E2FF-NFKB	249 - 363	(+)	
**GXP_226571** [GXP_226571] (1 - 750)<br>**NAPA**, GXL_189038, GeneID: 8775, Homo sapiens chr. 19<br>N-ethylmaleimide-sensitive factor attachment protein, alpha	E2FF-NFKB	246 - 357	(+)	
**GXP_226574** [GXP_226574] (1 - 787)<br>**LENG4**, GXL_189039, GeneID: 79143, Homo sapiens chr. 19<br>leukocyte receptor cluster (LRC) member 4	E2FF-NFKB	700 - 586	(-)	
**GXP_485914** [GXP_485914] (1 - 601)<br>**KCNN4**, GXL_189043, GeneID: 3783, Homo sapiens chr. 19<br>potassium intermediate/small conductance calcium-activated channel, subfamily N, member 4	E2FF-NFKB	264 - 379	(+)	
**GXP_226634** [GXP_226634] (1 - 646)<br>**C19orf7**, GXL_189078, GeneID: 23211, Homo sapiens chr. 19<br>chromosome 19 open reading frame 7	E2FF-NFKB	138 - 30	(-)	
**GXP_913541** [GXP_913541] (1 - 601)<br>**C19orf7**, GXL_189078, GeneID: 23211, Homo sapiens chr. 19<br>chromosome 19 open reading frame 7	E2FF-NFKB	240 - 132	(-)	
**GXP_913542** [GXP_913542] (1 - 601)<br>**C19orf7**, GXL_189078, GeneID: 23211, Homo sapiens chr. 19<br>chromosome 19 open reading frame 7	E2FF-NFKB	475 - 367	(-)	
**GXP_226644** [GXP_226644] (1 - 817)<br>**JOSD2**, GXL_189084, GeneID: 126119, Homo sapiens chr. 19<br>Josephin domain containing 2	E2FF-NFKB	745 - 620	(-)	
	E2FF-NFKB	421 - 527	(+)	

**GXP_226645** [GXP_226645] (1 - 601)<br>**JOSD2**, GXL_189084, GeneID: 126119, Homo sapiens chr. 19<br>Josephin domain containing 2				
**GXP_226659** [GXP_226659] (1 - 601)<br>**SIX5**, GXL_189095, GeneID: 147912, Homo sapiens chr. 19<br>sine oculis homeobox homolog 5 (Drosophila)	E2FF-NFKB	37 - 160	(+)	
**GXP_226660** [GXP_226660] (1 - 601)<br>**SIX5**, GXL_189095, GeneID: 147912, Homo sapiens chr. 19<br>sine oculis homeobox homolog 5 (Drosophila)	E2FF-NFKB	367 - 250	(-)	
**GXP_226663** [GXP_226663] (1 - 601)<br>**LHB**, GXL_189097, GeneID: 3972, Homo sapiens chr. 19<br>luteinizing hormone beta polypeptide	E2FF-NFKB	166 - 37	(-)	
**GXP_226707** [GXP_226707] (1 - 1222)<br>**CA11**, GXL_189131, GeneID: 770, Homo sapiens chr. 19<br>carbonic anhydrase XI	E2FF-NFKB	937 - 1063	(+)	
**GXP_226725** [GXP_226725] (1 - 1316)<br>**LYPD4**, GXL_189146, GeneID: 147719, Homo sapiens chr. 19<br>LY6/PLAUR domain containing 4	E2FF-NFKB	626 - 517	(-)	
**GXP_226742** [GXP_226742] (1 - 630)<br>**ZNF228**, GXL_189163, GeneID: 7771, Homo sapiens chr. 19<br>zinc finger protein 228	E2FF-NFKB	544 - 417	(-)	
**GXP_226747** [GXP_226747] (1 - 601)<br>**KLK7**, GXL_189166, GeneID: 5650, Homo sapiens chr. 19<br>kallikrein-related peptidase 7	E2FF-NFKB	417 - 303	(-)	
**GXP_226748** [GXP_226748] (1 - 601)<br>**KLK7**, GXL_189166, GeneID: 5650, Homo sapiens chr. 19<br>kallikrein-related peptidase 7	E2FF-NFKB	496 - 382	(-)	
**GXP_226776** [GXP_226776] (1 - 986)<br>**PAFAH1B3**, GXL_189193, GeneID: 5050, Homo sapiens chr. 19<br>platelet-activating factor acetylhydrolase, isoform Ib, gamma subunit 29kDa	E2FF-NFKB	305 - 411	(+)	
**GXP_227458** [GXP_227458] (1 - 601)<br>**UACA**, GXL_189733, GeneID: 55075, Homo sapiens chr. 15<br>uveal autoantigen with coiled-coil domains and ankyrin repeats	E2FF-NFKB	558 - 431	(-)	
**GXP_227466** [GXP_227466] (1 - 807)<br>**SELS**, GXL_189738, GeneID: 55829, Homo sapiens chr. 15<br>selenoprotein S	E2FF-NFKB	528 - 413	(-)	
**GXP_227490** [GXP_227490] (1 - 756)<br>**AP3S2,C15orf38**, GXL_189753, GeneID: 10239,348110, Homo sapiens chr. 15<br>adaptor-related protein complex 3, sigma 2 subunit; chromosome 15 open reading frame 38	E2FF-NFKB	468 - 346	(-)	
	E2FF-NFKB	726 - 848	(+)	

**GXP_227509** [GXP_227509] (1 - 1069)<br>**LCTL**, GXL_189761, GeneID: 197021, Homo sapiens chr. 15<br>lactase-like				
**GXP_227510** [GXP_227510] (1 - 601)<br>**LCTL**, GXL_189761, GeneID: 197021, Homo sapiens chr. 15<br>lactase-like	E2FF-NFKB	259 - 381	(+)	
**GXP_227524** [GXP_227524] (1 - 642)<br>**SEMA7A**, GXL_189772, GeneID: 8482, Homo sapiens chr. 15<br>semaphorin 7A, GPI membrane anchor (John Milton Hagen blood group)	E2FF-NFKB	211 - 331	(+)	
**GXP_227525** [GXP_227525] (1 - 601)<br>**SEMA7A**, GXL_189772, GeneID: 8482, Homo sapiens chr. 15<br>semaphorin 7A, GPI membrane anchor (John Milton Hagen blood group)	E2FF-NFKB	76 - 194	(+)	
**GXP_227534** [GXP_227534] (1 - 601)<br>**HERC1**, GXL_189777, GeneID: 8925, Homo sapiens chr. 15<br>hect (homologous to the E6-AP (UBE3A) carboxyl terminus) domain and RCC1 (CHC1)-like domain (RLD) 1	E2FF-NFKB	349 - 231	(-)	
**GXP_227551** [GXP_227551] (1 - 1243)<br>**HCN4**, GXL_189790, GeneID: 10021, Homo sapiens chr. 15<br>hyperpolarization activated cyclic nucleotide-gated potassium channel 4	E2FF-NFKB	645 - 525	(-)	
**GXP_227564** [GXP_227564] (1 - 601)<br>**RGMA**, GXL_189797, GeneID: 56963, Homo sapiens chr. 15<br>RGM domain family, member A	E2FF-NFKB	408 - 515	(+)	
**GXP_227568** [GXP_227568] (1 - 638)<br>**ADAMTS17**, GXL_189799, GeneID: 170691, Homo sapiens chr. 15<br>ADAM metallopeptidase with thrombospondin type 1 motif, 17	E2FF-NFKB	19 - 138	(+)	
**GXP_227570** [GXP_227570] (1 - 613)<br>**C15orf40**, GXL_189800, GeneID: 123207, Homo sapiens chr. 15<br>chromosome 15 open reading frame 40	E2FF-NFKB	581 - 469	(-)	
**GXP_227602** [GXP_227602] (1 - 631)<br>**AP3B2**, GXL_189822, GeneID: 8120, Homo sapiens chr. 15<br>adaptor-related protein complex 3, beta 2 subunit	E2FF-NFKB	210 - 103	(-)	
**GXP_227618** [GXP_227618] (1 - 1061)<br>**BTBD1**, GXL_189836, GeneID: 53339, Homo sapiens chr. 15<br>BTB (POZ) domain containing 1	E2FF-NFKB	261 - 135	(-)	
**GXP_227633** [GXP_227633] (1 - 602)<br>**STRA6**, GXL_189847, GeneID: 64220, Homo sapiens chr. 15<br>stimulated by retinoic acid gene 6 homolog (mouse)	E2FF-NFKB	291 - 172	(-)	
**GXP_909423** [GXP_909423] (1 - 943)<br>**STRA6**, GXL_189847, GeneID: 64220, Homo sapiens chr. 15<br>stimulated by retinoic acid gene 6 homolog (mouse)	E2FF-NFKB	645 - 538	(-)	
	E2FF-NFKB	563 - 453	(-)	

**GXP_909702** [GXP_909702] (1 - 601)<br>**VPS33B,LOC390638**, GXL_189848, GeneID: 26276,390638, Homo sapiens chr. 15<br>vacuolar protein sorting 33 homolog B (yeast); similar to Golgin subfamily A member 2 (Cis-Golgi matrix protein GM130)				
**GXP_909479** [GXP_909479] (1 - 601)<br>**NRG4**, GXL_189877, GeneID: 145957, Homo sapiens chr. 15<br>neuregulin 4	E2FF-NFKB	461 - 336	(-)	
**GXP_227771** [GXP_227771] (1 - 716)<br>**SNRPA1**, GXL_189948, GeneID: 6627, Homo sapiens chr. 15<br>small nuclear ribonucleoprotein polypeptide A'	E2FF-NFKB	431 - 306	(-)	
**GXP_227805** [GXP_227805] (1 - 601)<br>**LINGO1**, GXL_189980, GeneID: 84894, Homo sapiens chr. 15<br>leucine rich repeat and Ig domain containing 1	E2FF-NFKB	193 - 84	(-)	
**GXP_227809** [GXP_227809] (1 - 750)<br>**RHCG**, GXL_189983, GeneID: 51458, Homo sapiens chr. 15<br>Rh family, C glycoprotein	E2FF-NFKB	403 - 287	(-)	
**GXP_227814** [GXP_227814] (1 - 604)<br>**C9orf80**, GXL_189988, GeneID: 58493, Homo sapiens chr. 9<br>chromosome 9 open reading frame 80	E2FF-NFKB	573 - 459	(-)	
**GXP_227872** [GXP_227872] (1 - 1305)<br>**ANGPTL2**, GXL_190013, GeneID: 23452, Homo sapiens chr. 9<br>angiopoietin-like 2	E2FF-NFKB	531 - 659	(+)	
**GXP_927010** [GXP_927010] (1 - 601)<br>**CDC14B**, GXL_190029, GeneID: 8555, Homo sapiens chr. 9<br>CDC14 cell division cycle 14 homolog B (S. cerevisiae)	E2FF-NFKB	595 - 471	(-)	
**GXP_227931** [GXP_227931] (1 - 926)<br>**TTLL11**, GXL_190045, GeneID: 158135, Homo sapiens chr. 9<br>tubulin tyrosine ligase-like family, member 11	E2FF-NFKB	315 - 440	(+)	
**GXP_227951** [GXP_227951] (1 - 601)<br>**GKAP1**, GXL_190057, GeneID: 80318, Homo sapiens chr. 9<br>G kinase anchoring protein 1	E2FF-NFKB	331 - 444	(+)	
**GXP_228016** [GXP_228016] (1 - 601)<br>**C9orf5**, GXL_190095, GeneID: 23731, Homo sapiens chr. 9<br>chromosome 9 open reading frame 5	E2FF-NFKB	171 - 60	(-)	
**GXP_228025** [GXP_228025] (1 - 1123)<br>**EPB41L4B**, GXL_190101, GeneID: 54566, Homo sapiens chr. 9<br>erythrocyte membrane protein band 4.1 like 4B	E2FF-NFKB	833 - 709	(-)	
**GXP_927100** [GXP_927100] (1 - 601)<br>**PTPN3**, GXL_190104, GeneID: 5774, Homo sapiens chr. 9<br>protein tyrosine phosphatase, non-receptor type 3	E2FF-NFKB	337 - 453	(+)	
	E2FF-NFKB	424 - 317	(-)	

**GXP_228039** [GXP_228039] (1 - 1057)<br>**FANCC**, GXL_190109, GeneID: 2176, Homo sapiens chr. 9<br>Fanconi anemia, complementation group C	E2FF-NFKB	454 - 567	(+)	
**GXP_228073** [GXP_228073] (1 - 601)<br>**GRIN3A**, GXL_190131, GeneID: 116443, Homo sapiens chr. 9<br>glutamate receptor, ionotropic, N-methyl-D-aspartate 3A	E2FF-NFKB	411 - 288	(-)	
**GXP_228074** [GXP_228074] (1 - 671)<br>**NINJ1**, GXL_190132, GeneID: 4814, Homo sapiens chr. 9<br>ninjurin 1	E2FF-NFKB	54 - 171	(+)	
	E2FF-NFKB	185 - 68	(-)	
**GXP_228093** [GXP_228093] (1 - 601)<br>**KLF4**, GXL_190147, GeneID: 9314, Homo sapiens chr. 9<br>Kruppel-like factor 4 (gut)	E2FF-NFKB	183 - 75	(-)	
**GXP_228143** [GXP_228143] (1 - 601)<br>**ZNF782**, GXL_190189, GeneID: 158431, Homo sapiens chr. 9<br>zinc finger protein 782	E2FF-NFKB	398 - 520	(+)	
**GXP_228150** [GXP_228150] (1 - 601)<br>**C9orf125**, GXL_190196, GeneID: 84302, Homo sapiens chr. 9<br>chromosome 9 open reading frame 125	E2FF-NFKB	179 - 69	(-)	
**GXP_228186** [GXP_228186] (1 - 903)<br>**FBP1**, GXL_190228, GeneID: 2203, Homo sapiens chr. 9<br>fructose-1,6-bisphosphatase 1	E2FF-NFKB	576 - 449	(-)	
**GXP_228620** [GXP_228620] (1 - 732)<br>**ZBTB2,LOC729584,LOC732345**, GXL_190618, GeneID: 57621,729584,732345, Homo sapiens chr. 6<br>zinc finger and BTB domain containing 2; hypothetical protein LOC729584; hypothetical protein LOC732345	E2FF-NFKB	474 - 597	(+)	
**GXP_924174** [GXP_924174] (1 - 601)<br>**C6orf170**, GXL_190623, GeneID: 221322, Homo sapiens chr. 6<br>chromosome 6 open reading frame 170	E2FF-NFKB	237 - 112	(-)	
**GXP_658741** [GXP_658741] (1 - 641)<br>**SHPRH**, GXL_190626, GeneID: 257218, Homo sapiens chr. 6<br>SNF2 histone linker PHD RING helicase	E2FF-NFKB	130 - 238	(+)	
**GXP_924431** [GXP_924431] (1 - 601)<br>**SOD2**, GXL_190635, GeneID: 6648, Homo sapiens chr. 6<br>superoxide dismutase 2, mitochondrial	E2FF-NFKB	231 - 339	(+)	
**GXP_228663** [GXP_228663] (1 - 601)<br>**DDO,LOC728464,LOC731200**, GXL_190638, GeneID: 8528,728464,731200, Homo sapiens chr. 6<br>D-aspartate oxidase;similar to D-aspartate oxidase; similar to D-aspartate oxidase	E2FF-NFKB	547 - 440	(-)	
**GXP_924116** [GXP_924116] (1 - 621)<br>**DDO,LOC728464,LOC731200**, GXL_190638, GeneID: 8528,728464,731200, Homo sapiens chr. 6<br>D-aspartate oxidase;similar to D-aspartate oxidase; similar to D-aspartate oxidase	E2FF-NFKB	336 - 442	(+)	
	E2FF-NFKB	1089 - 1213	(+)	

**GXP_228696** [GXP_228696] (1 - 1253)<br>**IRF4**, GXL_190652, GeneID: 3662, Homo sapiens chr. 6<br>interferon regulatory factor 4				
**GXP_923311** [GXP_923311] (1 - 726)<br>**IRF4**, GXL_190652, GeneID: 3662, Homo sapiens chr. 6<br>interferon regulatory factor 4	E2FF-NFKB	594 - 700	(+)	
**GXP_228704** [GXP_228704] (1 - 987)<br>**EPM2A**, GXL_190657, GeneID: 7957, Homo sapiens chr. 6<br>epilepsy, progressive myoclonus type 2A, Lafora disease (laforin)	E2FF-NFKB	566 - 445	(-)	
**GXP_228709** [GXP_228709] (1 - 601)<br>**DKFZp686I15217**, GXL_190659, GeneID: 401232, Homo sapiens chr. 6<br>hypothetical protein DKFZp686I15217	E2FF-NFKB	285 - 396	(+)	
**GXP_924373** [GXP_924373] (1 - 601)<br>**RMND1**, GXL_190677, GeneID: 55005, Homo sapiens chr. 6<br>required for meiotic nuclear division 1 homolog (S. cerevisiae)	E2FF-NFKB	586 - 460	(-)	
**GXP_228756** [GXP_228756] (1 - 601)<br>**FYN**, GXL_190691, GeneID: 2534, Homo sapiens chr. 6<br>FYN oncogene related to SRC, FGR, YES	E2FF-NFKB	190 - 76	(-)	
**GXP_924323** [GXP_924323] (1 - 601)<br>**PLAGL1,HYMAI**, GXL_190717, GeneID: 5325,57061, Homo sapiens chr. 6<br>pleiomorphic adenoma gene-like 1; hydatidiform mole associated and imprinted	E2FF-NFKB	360 - 469	(+)	
**GXP_228806** [GXP_228806] (1 - 731)<br>**MTRF1L**, GXL_190728, GeneID: 54516, Homo sapiens chr. 6<br>mitochondrial translational release factor 1-like	E2FF-NFKB	360 - 476	(+)	
	E2FF-NFKB	577 - 462	(-)	
**GXP_924352** [GXP_924352] (1 - 601)<br>**PPIL4**, GXL_190732, GeneID: 85313, Homo sapiens chr. 6<br>peptidylprolyl isomerase (cyclophilin)-like 4	E2FF-NFKB	476 - 366	(-)	
**GXP_657628** [GXP_657628] (1 - 601)<br>**RIPK1**, GXL_190757, GeneID: 8737, Homo sapiens chr. 6<br>receptor (TNFRSF)-interacting serine-threonine kinase 1	E2FF-NFKB	139 - 15	(-)	
**GXP_228909** [GXP_228909] (1 - 803)<br>**FOXF2**, GXL_190802, GeneID: 2295, Homo sapiens chr. 6<br>forkhead box F2	E2FF-NFKB	46 - 172	(+)	
**GXP_228952** [GXP_228952] (1 - 848)<br>**MAP7**, GXL_190838, GeneID: 9053, Homo sapiens chr. 6<br>microtubule-associated protein 7	E2FF-NFKB	724 - 847	(+)	
**GXP_228965** [GXP_228965] (1 - 601)<br>**RNASET2**, GXL_190848, GeneID: 8635, Homo sapiens chr. 6<br>ribonuclease T2	E2FF-NFKB	400 - 280	(-)	
	E2FF-NFKB	173 - 295	(+)	

**GXP_658578** [GXP_658578] (1 - 601)<br>**ROS1**, GXL_190872, GeneID: 6098, Homo sapiens chr. 6<br>v-ros UR2 sarcoma virus oncogene homolog 1 (avian)				
**GXP_920976** [GXP_920976] (1 - 601)<br>**MCF2L2**, GXL_193448, GeneID: 23101, Homo sapiens chr. 3<br>MCF.2 cell line derived transforming sequence-like 2	E2FF-NFKB	435 - 324	(-)	
**GXP_488516** [GXP_488516] (1 - 601)<br>**ARPC4,TTLL3**, GXL_193474, GeneID: 10093,26140, Homo sapiens chr. 3<br>actin related protein 2/3 complex, subunit 4, 20kDa; tubulin tyrosine ligase-like family, member 3	E2FF-NFKB	392 - 271	(-)	
**GXP_232075** [GXP_232075] (1 - 725)<br>**BTD**, GXL_193492, GeneID: 686, Homo sapiens chr. 3<br>biotinidase	E2FF-NFKB	139 - 255	(+)	
**GXP_654954** [GXP_654954] (1 - 601)<br>**MUC4**, GXL_193495, GeneID: 4585, Homo sapiens chr. 3<br>mucin 4, cell surface associated	E2FF-NFKB	342 - 225	(-)	
**GXP_654872** [GXP_654872] (1 - 601)<br>**CRYGS**, GXL_193497, GeneID: 1427, Homo sapiens chr. 3<br>crystallin, gamma S	E2FF-NFKB	461 - 337	(-)	
**GXP_232090** [GXP_232090] (1 - 601)<br>**HDAC11**, GXL_193501, GeneID: 79885, Homo sapiens chr. 3<br>histone deacetylase 11	E2FF-NFKB	379 - 260	(-)	
**GXP_232129** [GXP_232129] (1 - 950)<br>**VHL**, GXL_193523, GeneID: 7428, Homo sapiens chr. 3<br>von Hippel-Lindau tumor suppressor	E2FF-NFKB	600 - 722	(+)	
**GXP_232149** [GXP_232149] (1 - 823)<br>**LMCD1**, GXL_193536, GeneID: 29995, Homo sapiens chr. 3<br>LIM and cysteine-rich domains 1	E2FF-NFKB	755 - 638	(-)	
**GXP_232167** [GXP_232167] (1 - 625)<br>**UBE2E2**, GXL_193549, GeneID: 7325, Homo sapiens chr. 3<br>ubiquitin-conjugating enzyme E2E 2 (UBC4/5 homolog, yeast)	E2FF-NFKB	480 - 590	(+)	
**GXP_232182** [GXP_232182] (1 - 601)<br>**EAF1**, GXL_193561, GeneID: 85403, Homo sapiens chr. 3<br>ELL associated factor 1	E2FF-NFKB	387 - 502	(+)	
**GXP_232218** [GXP_232218] (1 - 890)<br>**SETD5**, GXL_193583, GeneID: 55209, Homo sapiens chr. 3<br>SET domain containing 5	E2FF-NFKB<br>E2FF-NFKB	22 - 147<br>383 - 493	(+)<br>(+)	
**GXP_921033** [GXP_921033] (1 - 601)<br>**TBCCD1**, GXL_193620, GeneID: 55171, Homo sapiens chr. 3<br>TBCC domain containing 1	E2FF-NFKB	464 - 340	(-)	
	E2FF-NFKB	280 - 155	(-)	

**GXP_232380** [GXP_232380] (1 - 727)<br>**AAAS**, GXL_193714, GeneID: 8086, Homo sapiens chr. 12<br>achalasia, adrenocortical insufficiency, alacrimia (Allgrove, triple-A)				
**GXP_232383** [GXP_232383] (1 - 713)<br>**BBS10**, GXL_193715, GeneID: 79738, Homo sapiens chr. 12<br>Bardet-Biedl syndrome 10	E2FF-NFKB	538 - 417	(-)	
**GXP_641439** [GXP_641439] (1 - 601)<br>**SPRYD3**, GXL_193717, GeneID: 84926, Homo sapiens chr. 12<br>SPRY domain containing 3	E2FF-NFKB	258 - 130	(-)	
**GXP_232387** [GXP_232387] (1 - 945)<br>**KRT1**, GXL_193718, GeneID: 3848, Homo sapiens chr. 12<br>keratin 1 (epidermolytic hyperkeratosis)	E2FF-NFKB	370 - 490	(+)	
**GXP_232439** [GXP_232439] (1 - 601)<br>**OSBPL8**, GXL_193750, GeneID: 114882, Homo sapiens chr. 12<br>oxysterol binding protein-like 8	E2FF-NFKB	125 - 235	(+)	
**GXP_232547** [GXP_232547] (1 - 601)<br>**NTN4**, GXL_193809, GeneID: 59277, Homo sapiens chr. 12<br>netrin 4	E2FF-NFKB	215 - 327	(+)	
**GXP_907185** [GXP_907185] (1 - 601)<br>**NTN4**, GXL_193809, GeneID: 59277, Homo sapiens chr. 12<br>netrin 4	E2FF-NFKB	166 - 284	(+)	
	E2FF-NFKB	365 - 248	(-)	
**GXP_906776** [GXP_906776] (1 - 601)<br>**FMNL3**, GXL_193820, GeneID: 91010, Homo sapiens chr. 12<br>formin-like 3	E2FF-NFKB	208 - 102	(-)	
**GXP_232651** [GXP_232651] (1 - 640)<br>**CSRP2**, GXL_193878, GeneID: 1466, Homo sapiens chr. 12<br>cysteine and glycine-rich protein 2	E2FF-NFKB	100 - 221	(+)	
**GXP_232669** [GXP_232669] (1 - 973)<br>**WNT10B**, GXL_193893, GeneID: 7480, Homo sapiens chr. 12<br>wingless-type MMTV integration site family, member 10B	E2FF-NFKB	694 - 587	(-)	
**GXP_232685** [GXP_232685] (1 - 651)<br>**MAP3K12**, GXL_193907, GeneID: 7786, Homo sapiens chr. 12<br>mitogen-activated protein kinase kinase kinase 12	E2FF-NFKB	233 - 119	(-)	
**GXP_236396** [GXP_236396] (1 - 601)<br>**KIAA1754**, GXL_197068, GeneID: 85450, Homo sapiens chr. 10<br>KIAA1754	E2FF-NFKB	199 - 314	(+)	
**GXP_236405** [GXP_236405] (1 - 602)<br>**SLC16A12**, GXL_197071, GeneID: 387700, Homo sapiens chr. 10<br>solute carrier family 16, member 12 (monocarboxylic acid transporter 12)	E2FF-NFKB	172 - 55	(-)	
	E2FF-NFKB	930 - 1044	(+)	

**GXP_236423** [GXP_236423] (1 - 1071)<br>**PANK1**, GXL_197079, GeneID: 53354, Homo sapiens chr. 10<br>pantothenate kinase 1				
**GXP_236448** [GXP_236448] (1 - 602)<br>**PPP1R3C**, GXL_197091, GeneID: 5507, Homo sapiens chr. 10<br>protein phosphatase 1, regulatory (inhibitor) subunit 3C	E2FF-NFKB	449 - 327	(-)	
**GXP_236548** [GXP_236548] (1 - 929)<br>**TMEM10**, GXL_197138, GeneID: 93377, Homo sapiens chr. 10<br>transmembrane protein 10	E2FF-NFKB	645 - 771	(+)	
**GXP_904349** [GXP_904349] (1 - 601)<br>**ANXA11**, GXL_197145, GeneID: 311, Homo sapiens chr. 10<br>annexin A11	E2FF-NFKB	591 - 465	(-)	
**GXP_236574** [GXP_236574] (1 - 601)<br>**SEC31B**, GXL_197153, GeneID: 25956, Homo sapiens chr. 10<br>SEC31 homolog B (S. cerevisiae)	E2FF-NFKB	450 - 326	(-)	
**GXP_236628** [GXP_236628] (1 - 943)<br>**ALDH18A1**, GXL_197183, GeneID: 5832, Homo sapiens chr. 10<br>aldehyde dehydrogenase 18 family, member A1	E2FF-NFKB	710 - 587	(-)	
**GXP_236633** [GXP_236633] (1 - 789)<br>**PPP3CB**, GXL_197186, GeneID: 5532, Homo sapiens chr. 10<br>protein phosphatase 3 (formerly 2B), catalytic subunit, beta isoform	E2FF-NFKB	54 - 173	(+)	
**GXP_236645** [GXP_236645] (1 - 601)<br>**SLC25A28**, GXL_197194, GeneID: 81894, Homo sapiens chr. 10<br>solute carrier family 25, member 28	E2FF-NFKB	424 - 308	(-)	
**GXP_236650** [GXP_236650] (1 - 726)<br>**ARL3**, GXL_197197, GeneID: 403, Homo sapiens chr. 10<br>ADP-ribosylation factor-like 3	E2FF-NFKB<br>E2FF-NFKB	283 - 393<br>501 - 379	(+)<br>(-)	
**GXP_236673** [GXP_236673] (1 - 650)<br>**NOC3L**, GXL_197215, GeneID: 64318, Homo sapiens chr. 10<br>nucleolar complex associated 3 homolog (S. cerevisiae)	E2FF-NFKB	488 - 369	(-)	
**GXP_260387** [GXP_260387] (1 - 601)<br>**PIK3R3**, GXL_218269, GeneID: 8503, Homo sapiens chr. 1<br>phosphoinositide-3-kinase, regulatory subunit 3 (p55, gamma)	E2FF-NFKB	532 - 419	(-)	
**GXP_914372** [GXP_914372] (1 - 601)<br>**CD164L2**, GXL_218296, GeneID: 388611, Homo sapiens chr. 1<br>CD164 sialomucin-like 2	E2FF-NFKB	481 - 368	(-)	
**GXP_260473** [GXP_260473] (1 - 601)<br>**C1orf109**, GXL_218311, GeneID: 54955, Homo sapiens chr. 1<br>chromosome 1 open reading frame 109	E2FF-NFKB	531 - 423	(-)	
	E2FF-NFKB	771 - 648	(-)	

**GXP_260478** [GXP_260478] (1 - 1005)<br>**HYI**, GXL_218314, GeneID: 81888, Homo sapiens chr. 1<br>hydroxypyruvate isomerase homolog (E. coli)				
**GXP_260482** [GXP_260482] (1 - 620)<br>**MYOM3**, GXL_218316, GeneID: 127294, Homo sapiens chr. 1<br>myomesin family, member 3	E2FF-NFKB	534 - 423	(-)	
**GXP_260486** [GXP_260486] (1 - 601)<br>**STMN1**, GXL_218317, GeneID: 3925, Homo sapiens chr. 1<br>stathmin 1/oncoprotein 18	E2FF-NFKB	218 - 327	(+)	
**GXP_260493** [GXP_260493] (1 - 914)<br>**PTP4A2**, GXL_218324, GeneID: 8073, Homo sapiens chr. 1<br>protein tyrosine phosphatase type IVA, member 2	E2FF-NFKB	156 - 265	(+)	
**GXP_260530** [GXP_260530] (1 - 702)<br>**WDR57**, GXL_218346, GeneID: 9410, Homo sapiens chr. 1<br>WD repeat domain 57 (U5 snRNP specific)	E2FF-NFKB	604 - 488	(-)	
**GXP_914541** [GXP_914541] (1 - 601)<br>**RSPO1**, GXL_218371, GeneID: 284654, Homo sapiens chr. 1<br>R-spondin homolog (Xenopus laevis)	E2FF-NFKB	483 - 371	(-)	
**GXP_914387** [GXP_914387] (1 - 1052)<br>**DNAJC8**, GXL_218373, GeneID: 22826, Homo sapiens chr. 1<br>DnaJ (Hsp40) homolog, subfamily C, member 8	E2FF-NFKB	938 - 1045	(+)	
**GXP_260597** [GXP_260597] (1 - 894)<br>**GRIK3,LOC728387,LOC732170**, GXL_218392, GeneID: 2899,728387,732170, Homo sapiens chr. 1<br>glutamate receptor, ionotropic, kainate 3; hypothetical protein LOC728387; hypothetical protein LOC732170	E2FF-NFKB	75 - 196	(+)	
**GXP_260603** [GXP_260603] (1 - 709)<br>**MOBKL2C**, GXL_218394, GeneID: 148932, Homo sapiens chr. 1<br>MOB1, Mps One Binder kinase activator-like 2C (yeast)	E2FF-NFKB	417 - 532	(+)	
**GXP_260608** [GXP_260608] (1 - 619)<br>**SCMH1,LOC732258**, GXL_218397, GeneID: 22955,732258, Homo sapiens chr. 1<br>sex comb on midleg homolog 1 (Drosophila); hypothetical protein LOC732258	E2FF-NFKB	494 - 381	(-)	
**GXP_914360** [GXP_914360] (1 - 601)<br>**SLC9A1**, GXL_218418, GeneID: 6548, Homo sapiens chr. 1<br>solute carrier family 9 (sodium/hydrogen exchanger), member 1 (antiporter, Na+/H+, amiloride sensitive)	E2FF-NFKB	324 - 443	(+)	
**GXP_914716** [GXP_914716] (1 - 918)<br>**TAL1**, GXL_218442, GeneID: 6886, Homo sapiens chr. 1<br>T-cell acute lymphocytic leukemia 1	E2FF-NFKB	404 - 529	(+)	
**GXP_260679** [GXP_260679] (1 - 637)<br>**GPATCH3**, GXL_218453, GeneID: 63906, Homo sapiens chr. 1<br>G patch domain containing 3	E2FF-NFKB	491 - 600	(+)	
	E2FF-NFKB	506 - 393	(-)	
	E2FF-NFKB	500 - 626	(+)	

**GXP_260687** [GXP_260687] (1 - 978)<br>**BMP8B**, GXL_218460, GeneID: 656, Homo sapiens chr. 1<br>bone morphogenetic protein 8b (osteogenic protein 2)				
**GXP_261735** [GXP_261735] (1 - 852)<br>**SH3KBP1**, GXL_219424, GeneID: 30011, Homo sapiens chr. X<br>SH3-domain kinase binding protein 1	E2FF-NFKB	254 - 382	(+)	
**GXP_261783** [GXP_261783] (1 - 623)<br>**BCOR**, GXL_219448, GeneID: 54880, Homo sapiens chr. X<br>BCL6 co-repressor	E2FF-NFKB	365 - 252	(-)	
**GXP_927560** [GXP_927560] (1 - 601)<br>**RBBP7**, GXL_219468, GeneID: 5931, Homo sapiens chr. X<br>retinoblastoma binding protein 7	E2FF-NFKB	491 - 374	(-)	
**GXP_261872** [GXP_261872] (1 - 637)<br>**GRIPAP1**, GXL_219497, GeneID: 56850, Homo sapiens chr. X<br>GRIP1 associated protein 1	E2FF-NFKB<br>E2FF-NFKB	221 - 101<br>481 - 604	(-)<br>(+)	
**GXP_261874** [GXP_261874] (1 - 791)<br>**FAM9B**, GXL_219499, GeneID: 171483, Homo sapiens chr. X<br>family with sequence similarity 9, member B	E2FF-NFKB	632 - 751	(+)	
**GXP_261883** [GXP_261883] (1 - 705)<br>**LMO6**, GXL_219504, GeneID: 4007, Homo sapiens chr. X<br>LIM domain only 6	E2FF-NFKB	213 - 89	(-)	
**GXP_261906** [GXP_261906] (1 - 602)<br>**FAM123B**, GXL_219520, GeneID: 139285, Homo sapiens chr. X<br>family with sequence similarity 123B	E2FF-NFKB	188 - 308	(+)	
**GXP_927620** [GXP_927620] (1 - 601)<br>**ARX**, GXL_219531, GeneID: 170302, Homo sapiens chr. X<br>aristaless related homeobox	E2FF-NFKB	295 - 188	(-)	
**GXP_261973** [GXP_261973] (1 - 601)<br>**NAP1L6**, GXL_219567, GeneID: 645996, Homo sapiens chr. X<br>nucleosome assembly protein 1-like 6	E2FF-NFKB	212 - 98	(-)	
**GXP_927810** [GXP_927810] (1 - 601)<br>**NUDT11**, GXL_219576, GeneID: 55190, Homo sapiens chr. X<br>nudix (nucleoside diphosphate linked moiety X)-type motif 11	E2FF-NFKB	212 - 100	(-)	
**GXP_262013** [GXP_262013] (1 - 1356)<br>**SLC38A5**, GXL_219600, GeneID: 92745, Homo sapiens chr. X<br>solute carrier family 38, member 5	E2FF-NFKB	894 - 773	(-)	
**GXP_262014** [GXP_262014] (1 - 601)<br>**SLC38A5**, GXL_219600, GeneID: 92745, Homo sapiens chr. X<br>solute carrier family 38, member 5	E2FF-NFKB	139 - 18	(-)	
	E2FF-NFKB	249 - 129	(-)	

**GXP_262028** [GXP_262028] (1 - 954)<br>**FAM120C**, GXL_219612, GeneID: 54954, Homo sapiens chr. X<br>family with sequence similarity 120C				
**GXP_262059** [GXP_262059] (1 - 732)<br>**DYNLT3**, GXL_219638, GeneID: 6990, Homo sapiens chr. X<br>dynein, light chain, Tctex-type 3	E2FF-NFKB	421 - 540	(+)	
**GXP_262081** [GXP_262081] (1 - 640)<br>**PDZD11**, GXL_219659, GeneID: 51248, Homo sapiens chr. X<br>PDZ domain containing 11	E2FF-NFKB	221 - 340	(+)	
**GXP_655919** [GXP_655919] (1 - 601)<br>**PITX2**, GXL_220775, GeneID: 5308, Homo sapiens chr. 4<br>paired-like homeodomain transcription factor 2	E2FF-NFKB	411 - 285	(-)	
**GXP_263496** [GXP_263496] (1 - 803)<br>**UBE2D3**, GXL_220799, GeneID: 7323, Homo sapiens chr. 4<br>ubiquitin-conjugating enzyme E2D 3 (UBC4/5 homolog, yeast)	E2FF-NFKB	583 - 470	(-)	
**GXP_263505** [GXP_263505] (1 - 1059)<br>**PDE5A**, GXL_220803, GeneID: 8654, Homo sapiens chr. 4<br>phosphodiesterase 5A, cGMP-specific	E2FF-NFKB	249 - 131	(-)	
**GXP_921670** [GXP_921670] (1 - 601)<br>**SPARCL1**, GXL_220816, GeneID: 8404, Homo sapiens chr. 4<br>SPARC-like 1 (mast9, hevin)	E2FF-NFKB	418 - 292	(-)	
**GXP_921609** [GXP_921609] (1 - 601)<br>**CCNI,LOC643280,LOC731020,LOC731024**, GXL_220833, GeneID: 10983,643280,731020,731024, Homo sapiens chr. 4<br>cyclin I;hypothetical LOC643280; hypothetical protein LOC731020; hypothetical protein LOC731024	E2FF-NFKB	266 - 141	(-)	
**GXP_263611** [GXP_263611] (1 - 601)<br>**UNC5C**, GXL_220867, GeneID: 8633, Homo sapiens chr. 4<br>unc-5 homolog C (C. elegans)	E2FF-NFKB	505 - 382	(-)	
**GXP_263770** [GXP_263770] (1 - 703)<br>**MFSD8**, GXL_220988, GeneID: 256471, Homo sapiens chr. 4<br>major facilitator superfamily domain containing 8	E2FF-NFKB	75 - 188	(+)	
	E2FF-NFKB	583 - 475	(-)	
**GXP_921813** [GXP_921813] (1 - 601)<br>**ARSJ**, GXL_220995, GeneID: 79642, Homo sapiens chr. 4<br>arylsulfatase family, member J	E2FF-NFKB	207 - 329	(+)	
**GXP_263792** [GXP_263792] (1 - 812)<br>**MAD2L1**, GXL_221009, GeneID: 4085, Homo sapiens chr. 4<br>MAD2 mitotic arrest deficient-like 1 (yeast)	E2FF-NFKB	629 - 749	(+)	
**GXP_268882** [GXP_268882] (1 - 612)<br>**SPPL2A**, GXL_225416, GeneID: 84888, Homo sapiens chr. 15<br>signal peptide peptidase-like 2A	E2FF-NFKB	575 - 468	(-)	
	E2FF-NFKB	76 - 202	(+)	

**GXP_268954** [GXP_268954] (1 - 601)<br>**DMXL2**, GXL_225445, GeneID: 23312, Homo sapiens chr. 15<br>Dmx-like 2				
**GXP_269042** [GXP_269042] (1 - 689)<br>**SRP14**, GXL_225491, GeneID: 6727, Homo sapiens chr. 15<br>signal recognition particle 14kDa (homologous Alu RNA binding protein)	E2FF-NFKB	407 - 516	(+)	
**GXP_269069** [GXP_269069] (1 - 611)<br>**ATP8B4**, GXL_225513, GeneID: 79895, Homo sapiens chr. 15<br>ATPase, Class I, type 8B, member 4	E2FF-NFKB	297 - 171	(-)	
**GXP_269103** [GXP_269103] (1 - 670)<br>**RAB27A**, GXL_225537, GeneID: 5873, Homo sapiens chr. 15<br>RAB27A, member RAS oncogene family	E2FF-NFKB	303 - 193	(-)	
**GXP_269110** [GXP_269110] (1 - 1135)<br>**LYSMD2**, GXL_225540, GeneID: 256586, Homo sapiens chr. 15<br>LysM, putative peptidoglycan-binding, domain containing 2	E2FF-NFKB<br>E2FF-NFKB	605 - 497<br>628 - 751	(-)<br>(+)	
**GXP_269182** [GXP_269182] (1 - 602)<br>**BCL2L10**, GXL_225595, GeneID: 10017, Homo sapiens chr. 15<br>BCL2-like 10 (apoptosis facilitator)	E2FF-NFKB	281 - 403	(+)	
**GXP_269193** [GXP_269193] (1 - 779)<br>**AGPAT7**, GXL_225604, GeneID: 254531, Homo sapiens chr. 15<br>1-acylglycerol-3-phosphate O-acyltransferase 7 (lysophosphatidic acid acyltransferase, eta)	E2FF-NFKB	366 - 491	(+)	
**GXP_269195** [GXP_269195] (1 - 676)<br>**TP53BP1**, GXL_225606, GeneID: 7158, Homo sapiens chr. 15<br>tumor protein p53 binding protein, 1	E2FF-NFKB	593 - 471	(-)	
**GXP_271978** [GXP_271978] (1 - 641)<br>**ANKRD26**, GXL_227909, GeneID: 22852, Homo sapiens chr. 10<br>ankyrin repeat domain 26	E2FF-NFKB	327 - 214	(-)	
**GXP_272008** [GXP_272008] (1 - 842)<br>**MKX**, GXL_227923, GeneID: 283078, Homo sapiens chr. 10<br>mohawk homeobox	E2FF-NFKB	624 - 512	(-)	
**GXP_272042** [GXP_272042] (1 - 607)<br>**TUBAL3**, GXL_227941, GeneID: 79861, Homo sapiens chr. 10<br>tubulin, alpha-like 3	E2FF-NFKB	605 - 486	(-)	
**GXP_904033** [GXP_904033] (1 - 677)<br>**HNRPF**, GXL_227945, GeneID: 3185, Homo sapiens chr. 10<br>heterogeneous nuclear ribonucleoprotein F	E2FF-NFKB	289 - 166	(-)	
**GXP_272050** [GXP_272050] (1 - 1140)<br>**C10orf63**, GXL_227946, GeneID: 219670, Homo sapiens chr. 10<br>chromosome 10 open reading frame 63	E2FF-NFKB	985 - 1106	(+)	
	E2FF-NFKB	408 - 283	(-)	

**GXP_272068** [GXP_272068] (1 - 610)<br>**ZNF33B**, GXL_227957, GeneID: 7582, Homo sapiens chr. 10<br>zinc finger protein 33B				
**GXP_904203** [GXP_904203] (1 - 601)<br>**CTNNA3**, GXL_227976, GeneID: 29119, Homo sapiens chr. 10<br>catenin (cadherin-associated protein), alpha 3	E2FF-NFKB	488 - 594	(+)	
**GXP_272096** [GXP_272096] (1 - 868)<br>**PBLD**, GXL_227978, GeneID: 64081, Homo sapiens chr. 10<br>phenazine biosynthesis-like protein domain containing	E2FF-NFKB	376 - 482	(+)	
**GXP_479060** [GXP_479060] (1 - 601)<br>**ARMETL1**, GXL_227992, GeneID: 441549, Homo sapiens chr. 10<br>arginine-rich, mutated in early stage tumors-like 1	E2FF-NFKB	238 - 112	(-)	
**GXP_903756** [GXP_903756] (1 - 774)<br>**LARP5**, GXL_228015, GeneID: 23185, Homo sapiens chr. 10<br>La ribonucleoprotein domain family, member 5	E2FF-NFKB	545 - 438	(-)	
**GXP_272267** [GXP_272267] (1 - 601)<br>**MAN1A2**, GXL_228117, GeneID: 10905, Homo sapiens chr. 1<br>mannosidase, alpha, class 1A, member 2	E2FF-NFKB	572 - 465	(-)	
**GXP_272298** [GXP_272298] (1 - 737)<br>**FAM40A**, GXL_228135, GeneID: 85369, Homo sapiens chr. 1<br>family with sequence similarity 40, member A	E2FF-NFKB	653 - 538	(-)	
**GXP_272306** [GXP_272306] (1 - 1378)<br>**HIPK1**, GXL_228140, GeneID: 204851, Homo sapiens chr. 1<br>homeodomain interacting protein kinase 1	E2FF-NFKB	445 - 571	(+)	
**GXP_272350** [GXP_272350] (1 - 1044)<br>**DDX20**, GXL_228170, GeneID: 11218, Homo sapiens chr. 1<br>DEAD (Asp-Glu-Ala-Asp) box polypeptide 20	E2FF-NFKB	745 - 633	(-)	
**GXP_914954** [GXP_914954] (1 - 601)<br>**LPHN2**, GXL_228173, GeneID: 23266, Homo sapiens chr. 1<br>latrophilin 2	E2FF-NFKB	470 - 356	(-)	
**GXP_272355** [GXP_272355] (1 - 601)<br>**HHLA3**, GXL_228174, GeneID: 11147, Homo sapiens chr. 1<br>HERV-H LTR-associating 3	E2FF-NFKB	450 - 564	(+)	
**GXP_272389** [GXP_272389] (1 - 1038)<br>**PKN2**, GXL_228192, GeneID: 5586, Homo sapiens chr. 1<br>protein kinase N2	E2FF-NFKB	616 - 490	(-)	
**GXP_915180** [GXP_915180] (1 - 601)<br>**AHCYL1**, GXL_228193, GeneID: 10768, Homo sapiens chr. 1<br>S-adenosylhomocysteine hydrolase-like 1	E2FF-NFKB	581 - 453	(-)	
	E2FF-NFKB	371 - 249	(-)	

**GXP_272395** [GXP_272395] (1 - 693)<br>**STXBP3**, GXL_228196, GeneID: 6814, Homo sapiens chr. 1<br>syntaxin binding protein 3				
**GXP_636234** [GXP_636234] (1 - 601)<br>**CDC7**, GXL_228220, GeneID: 8317, Homo sapiens chr. 1<br>cell division cycle 7 homolog (S. cerevisiae)	E2FF-NFKB<br>E2FF-NFKB	332 - 454<br>418 - 309	(+)<br>(-)	
**GXP_915028** [GXP_915028] (1 - 914)<br>**CDC7**, GXL_228220, GeneID: 8317, Homo sapiens chr. 1<br>cell division cycle 7 homolog (S. cerevisiae)	E2FF-NFKB<br>E2FF-NFKB	66 - 188<br>152 - 43	(+)<br>(-)	
**GXP_272459** [GXP_272459] (1 - 819)<br>**RTCD1**, GXL_228235, GeneID: 8634, Homo sapiens chr. 1<br>RNA terminal phosphate cyclase domain 1	E2FF-NFKB	314 - 421	(+)	
**GXP_272503** [GXP_272503] (1 - 601)<br>**HS2ST1,LOC339524**, GXL_228270, GeneID: 9653,339524, Homo sapiens chr. 1<br>heparan sulfate 2-O-sulfotransferase 1; hypothetical protein LOC339524	E2FF-NFKB	454 - 334	(-)	
**GXP_272522** [GXP_272522] (1 - 985)<br>**TXNIP**, GXL_228284, GeneID: 10628, Homo sapiens chr. 1<br>thioredoxin interacting protein	E2FF-NFKB	37 - 163	(+)	
**GXP_272550** [GXP_272550] (1 - 1276)<br>**MAGI3**, GXL_228306, GeneID: 260425, Homo sapiens chr. 1<br>membrane associated guanylate kinase, WW and PDZ domain containing 3	E2FF-NFKB	602 - 496	(-)	
**GXP_909547** [GXP_909547] (1 - 698)<br>**C15orf26**, GXL_230271, GeneID: 161502, Homo sapiens chr. 15<br>chromosome 15 open reading frame 26	E2FF-NFKB	457 - 344	(-)	
**GXP_274810** [GXP_274810] (1 - 641)<br>**LRRC49**, GXL_230272, GeneID: 54839, Homo sapiens chr. 15<br>leucine rich repeat containing 49	E2FF-NFKB	443 - 561	(+)	
**GXP_274817** [GXP_274817] (1 - 955)<br>**MORF4L1**, GXL_230277, GeneID: 10933, Homo sapiens chr. 15<br>mortality factor 4 like 1	E2FF-NFKB	794 - 679	(-)	
**GXP_909391** [GXP_909391] (1 - 601)<br>**NR2E3**, GXL_230283, GeneID: 10002, Homo sapiens chr. 15<br>nuclear receptor subfamily 2, group E, member 3	E2FF-NFKB	4 - 122	(+)	
**GXP_274833** [GXP_274833] (1 - 856)<br>**PDE8A,LOC728107**, GXL_230286, GeneID: 5151,728107, Homo sapiens chr. 15<br>phosphodiesterase 8A;hypothetical protein LOC728107	E2FF-NFKB	222 - 116	(-)	
**GXP_274844** [GXP_274844] (1 - 744)<br>**IREB2**, GXL_230289, GeneID: 3658, Homo sapiens chr. 15<br>iron-responsive element binding protein 2	E2FF-NFKB	228 - 353	(+)	
	E2FF-NFKB	494 - 619	(+)	

**GXP_274853** [GXP_274853] (1 - 748)<br>**C15orf27**, GXL_230293, GeneID: 123591, Homo sapiens chr. 15<br>chromosome 15 open reading frame 27				
**GXP_274892** [GXP_274892] (1 - 766)<br>**RPLP1,hCG_1641617**, GXL_230311, GeneID: 6176,729416, Homo sapiens chr. 15<br>ribosomal protein, large, P1;hCG1641617	E2FF-NFKB	668 - 561	(-)	
**GXP_909426** [GXP_909426] (1 - 601)<br>**CCDC33**, GXL_230322, GeneID: 80125, Homo sapiens chr. 15<br>coiled-coil domain containing 33	E2FF-NFKB	117 - 242	(+)	
**GXP_274937** [GXP_274937] (1 - 601)<br>**CLK3**, GXL_230338, GeneID: 1198, Homo sapiens chr. 15<br>CDC-like kinase 3	E2FF-NFKB	399 - 277	(-)	
**GXP_274944** [GXP_274944] (1 - 785)<br>**TTLL13**, GXL_230341, GeneID: 440307, Homo sapiens chr. 15<br>tubulin tyrosine ligase-like family, member 13	E2FF-NFKB	385 - 505	(+)	
**GXP_909677** [GXP_909677] (1 - 601)<br>**TTLL13**, GXL_230341, GeneID: 440307, Homo sapiens chr. 15<br>tubulin tyrosine ligase-like family, member 13	E2FF-NFKB	459 - 579	(+)	
**GXP_274999** [GXP_274999] (1 - 668)<br>**ZNF774**, GXL_230375, GeneID: 342132, Homo sapiens chr. 15<br>zinc finger protein 774	E2FF-NFKB	609 - 495	(-)	
**GXP_275062** [GXP_275062] (1 - 610)<br>**ADAMTSL3**, GXL_230412, GeneID: 57188, Homo sapiens chr. 15<br>ADAMTS-like 3	E2FF-NFKB	471 - 579	(+)	
**GXP_275171** [GXP_275171] (1 - 601)<br>**ARNT2**, GXL_230500, GeneID: 9915, Homo sapiens chr. 15<br>aryl-hydrocarbon receptor nuclear translocator 2	E2FF-NFKB	239 - 359	(+)	
**GXP_277485** [GXP_277485] (1 - 712)<br>**ABCF3**, GXL_232554, GeneID: 55324, Homo sapiens chr. 3<br>ATP-binding cassette, sub-family F (GCN20), member 3	E2FF-NFKB	135 - 256	(+)	
**GXP_921044** [GXP_921044] (1 - 601)<br>**ST6GAL1**, GXL_232555, GeneID: 6480, Homo sapiens chr. 3<br>ST6 beta-galactosamide alpha-2,6-sialyltranferase 1	E2FF-NFKB	175 - 294	(+)	
**GXP_921009** [GXP_921009] (1 - 601)<br>**EPHB3**, GXL_232573, GeneID: 2049, Homo sapiens chr. 3<br>EPH receptor B3	E2FF-NFKB	194 - 309	(+)	
**GXP_654603** [GXP_654603] (1 - 1331)<br>**SCHIP1**, GXL_232599, GeneID: 29970, Homo sapiens chr. 3<br>schwannomin interacting protein 1	E2FF-NFKB	242 - 113	(-)	
	E2FF-NFKB	247 - 135	(-)	

**GXP_277562** [GXP_277562] (1 - 615)<br>**TIPARP**, GXL_232601, GeneID: 25976, Homo sapiens chr. 3<br>TCDD-inducible poly(ADP-ribose) polymerase				
**GXP_277637** [GXP_277637] (1 - 601)<br>**ZNF639**, GXL_232643, GeneID: 51193, Homo sapiens chr. 3<br>zinc finger protein 639	E2FF-NFKB	547 - 418	(-)	
**GXP_277659** [GXP_277659] (1 - 727)<br>**MME**, GXL_232662, GeneID: 4311, Homo sapiens chr. 3<br>membrane metallo-endopeptidase	E2FF-NFKB	64 - 180	(+)	
**GXP_277660** [GXP_277660] (1 - 601)<br>**MME**, GXL_232662, GeneID: 4311, Homo sapiens chr. 3<br>membrane metallo-endopeptidase	E2FF-NFKB	312 - 428	(+)	
**GXP_920818** [GXP_920818] (1 - 601)<br>**MME**, GXL_232662, GeneID: 4311, Homo sapiens chr. 3<br>membrane metallo-endopeptidase	E2FF-NFKB	216 - 332	(+)	
**GXP_277668** [GXP_277668] (1 - 678)<br>**IL1RAP**, GXL_232670, GeneID: 3556, Homo sapiens chr. 3<br>interleukin 1 receptor accessory protein	E2FF-NFKB	494 - 373	(-)	
**GXP_920985** [GXP_920985] (1 - 601)<br>**HTR3E**, GXL_232674, GeneID: 285242, Homo sapiens chr. 3<br>5-hydroxytryptamine (serotonin) receptor 3, family member E	E2FF-NFKB	477 - 585	(+)	
**GXP_277693** [GXP_277693] (1 - 611)<br>**SENP5**, GXL_232691, GeneID: 205564, Homo sapiens chr. 3<br>SUMO1/sentrin specific peptidase 5	E2FF-NFKB	482 - 592	(+)	
**GXP_277747** [GXP_277747] (1 - 1155)<br>**PSMD2**, GXL_232738, GeneID: 5708, Homo sapiens chr. 3<br>proteasome (prosome, macropain) 26S subunit, non-ATPase, 2	E2FF-NFKB	1037 - 931	(-)	
**GXP_277754** [GXP_277754] (1 - 900)<br>**EIF4A2**, GXL_232745, GeneID: 1974, Homo sapiens chr. 3<br>eukaryotic translation initiation factor 4A, isoform 2	E2FF-NFKB	372 - 481	(+)	
**GXP_921038** [GXP_921038] (1 - 601)<br>**EIF4A2**, GXL_232745, GeneID: 1974, Homo sapiens chr. 3<br>eukaryotic translation initiation factor 4A, isoform 2	E2FF-NFKB	472 - 581	(+)	
**GXP_277791** [GXP_277791] (1 - 601)<br>**FNDC1**, GXL_232772, GeneID: 84624, Homo sapiens chr. 6<br>fibronectin type III domain containing 1	E2FF-NFKB	335 - 464	(+)	
**GXP_277799** [GXP_277799] (1 - 793)<br>**HSF2**, GXL_232777, GeneID: 3298, Homo sapiens chr. 6<br>heat shock transcription factor 2	E2FF-NFKB	555 - 444	(-)	
	E2FF-NFKB	131 - 238	(+)	

**GXP_277826** [GXP_277826] (1 - 686)<br>**TPD52L1**, GXL_232792, GeneID: 7164, Homo sapiens chr. 6<br>tumor protein D52-like 1				
**GXP_277827** [GXP_277827] (1 - 864)<br>**TPD52L1**, GXL_232792, GeneID: 7164, Homo sapiens chr. 6<br>tumor protein D52-like 1	E2FF-NFKB	710 - 817	(+)	
**GXP_277859** [GXP_277859] (1 - 742)<br>**C6orf72**, GXL_232808, GeneID: 116254, Homo sapiens chr. 6<br>chromosome 6 open reading frame 72	E2FF-NFKB	28 - 138	(+)	
**GXP_277902** [GXP_277902] (1 - 797)<br>**SYNJ2**, GXL_232834, GeneID: 8871, Homo sapiens chr. 6<br>synaptojanin 2	E2FF-NFKB	143 - 266	(+)	
**GXP_277904** [GXP_277904] (1 - 728)<br>**SMOC2**, GXL_232835, GeneID: 64094, Homo sapiens chr. 6<br>SPARC related modular calcium binding 2	E2FF-NFKB	4 - 124	(+)	
**GXP_491712** [GXP_491712] (1 - 630)<br>**L3MBTL3**, GXL_232863, GeneID: 84456, Homo sapiens chr. 6<br>l(3)mbt-like 3 (Drosophila)	E2FF-NFKB	228 - 99	(-)	
**GXP_277978** [GXP_277978] (1 - 676)<br>**STX11**, GXL_232884, GeneID: 8676, Homo sapiens chr. 6<br>syntaxin 11	E2FF-NFKB	357 - 244	(-)	
**GXP_278001** [GXP_278001] (1 - 821)<br>**TNFAIP3**, GXL_232901, GeneID: 7128, Homo sapiens chr. 6<br>tumor necrosis factor, alpha-induced protein 3	E2FF-NFKB	159 - 273	(+)	
	E2FF-NFKB	400 - 282	(-)	
**GXP_658867** [GXP_658867] (1 - 601)<br>**MRPL18**, GXL_232928, GeneID: 29074, Homo sapiens chr. 6<br>mitochondrial ribosomal protein L18	E2FF-NFKB	446 - 336	(-)	
**GXP_278048** [GXP_278048] (1 - 691)<br>**GJA1**, GXL_232943, GeneID: 2697, Homo sapiens chr. 6<br>gap junction protein, alpha 1, 43kDa	E2FF-NFKB	526 - 632	(+)	
**GXP_490974** [GXP_490974] (1 - 601)<br>**N4BP3**, GXL_234947, GeneID: 23138, Homo sapiens chr. 5<br>Nedd4 binding protein 3	E2FF-NFKB	358 - 487	(+)	
	E2FF-NFKB	582 - 473	(-)	
**GXP_280283** [GXP_280283] (1 - 610)<br>**UNC5A**, GXL_234958, GeneID: 90249, Homo sapiens chr. 5<br>unc-5 homolog A (C. elegans)	E2FF-NFKB	418 - 292	(-)	
**GXP_280286** [GXP_280286] (1 - 730)<br>**CANX**, GXL_234960, GeneID: 821, Homo sapiens chr. 5<br>calnexin	E2FF-NFKB	534 - 662	(+)	
	E2FF-NFKB	557 - 448	(-)	

**GXP_280294** [GXP_280294] (1 - 705)<br>**BNIP1**, GXL_234963, GeneID: 662, Homo sapiens chr. 5<br>BCL2/adenovirus E1B 19kDa interacting protein 1				
**GXP_923156** [GXP_923156] (1 - 601)<br>**TLX3**, GXL_234988, GeneID: 30012, Homo sapiens chr. 5<br>T-cell leukemia homeobox 3	E2FF-NFKB	193 - 301	(+)	
**GXP_280339** [GXP_280339] (1 - 631)<br>**UBXD8**, GXL_234991, GeneID: 23197, Homo sapiens chr. 5<br>UBX domain containing 8	E2FF-NFKB	446 - 323	(-)	
**GXP_657501** [GXP_657501] (1 - 601)<br>**FGFR4**, GXL_235009, GeneID: 2264, Homo sapiens chr. 5<br>fibroblast growth factor receptor 4	E2FF-NFKB<br>E2FF-NFKB	131 - 240<br>340 - 227	(+)<br>(-)	
**GXP_280367** [GXP_280367] (1 - 1487)<br>**PRELID1**, GXL_235016, GeneID: 27166, Homo sapiens chr. 5<br>PRELI domain containing 1	E2FF-NFKB	272 - 401	(+)	
**GXP_918991** [GXP_918991] (1 - 601)<br>**DPP4**, GXL_241067, GeneID: 1803, Homo sapiens chr. 2<br>dipeptidyl-peptidase 4 (CD26, adenosine deaminase complexing protein 2)	E2FF-NFKB	15 - 133	(+)	
**GXP_651012** [GXP_651012] (1 - 601)<br>**TTN**, GXL_241093, GeneID: 7273, Homo sapiens chr. 2<br>titin	E2FF-NFKB	361 - 468	(+)	
**GXP_286772** [GXP_286772] (1 - 709)<br>**PLA2R1**, GXL_241096, GeneID: 22925, Homo sapiens chr. 2<br>phospholipase A2 receptor 1, 180kDa	E2FF-NFKB	581 - 690	(+)	
**GXP_286803** [GXP_286803] (1 - 603)<br>**CHN1**, GXL_241113, GeneID: 1123, Homo sapiens chr. 2<br>chimerin (chimaerin) 1	E2FF-NFKB	570 - 459	(-)	
**GXP_919097** [GXP_919097] (1 - 601)<br>**WIPF1**, GXL_241114, GeneID: 7456, Homo sapiens chr. 2<br>WAS/WASL interacting protein family, member 1	E2FF-NFKB<br>E2FF-NFKB	77 - 193<br>224 - 113	(+)<br>(-)	
**GXP_286813** [GXP_286813] (1 - 1004)<br>**NCKAP1**, GXL_241119, GeneID: 10787, Homo sapiens chr. 2<br>NCK-associated protein 1	E2FF-NFKB	266 - 373	(+)	
**GXP_286900** [GXP_286900] (1 - 1278)<br>**IWS1**, GXL_241170, GeneID: 55677, Homo sapiens chr. 2<br>IWS1 homolog (S. cerevisiae)	E2FF-NFKB	1101 - 989	(-)	
**GXP_286936** [GXP_286936] (1 - 1029)<br>**SLC40A1**, GXL_241198, GeneID: 30061, Homo sapiens chr. 2<br>solute carrier family 40 (iron-regulated transporter), member 1	E2FF-NFKB	229 - 349	(+)	
	E2FF-NFKB	428 - 311	(-)	

**GXP_294563** [GXP_294563] (1 - 601)<br>**FUT8**, GXL_247702, GeneID: 2530, Homo sapiens chr. 14<br>fucosyltransferase 8 (alpha (1,6) fucosyltransferase)				
**GXP_908395** [GXP_908395] (1 - 601)<br>**SYNE2**, GXL_247711, GeneID: 23224, Homo sapiens chr. 14<br>spectrin repeat containing, nuclear envelope 2	E2FF-NFKB	261 - 386	(+)	
**GXP_294641** [GXP_294641] (1 - 1194)<br>**FLJ42220**, GXL_247747, GeneID: 400207, Homo sapiens chr. 14<br>FLJ42220 protein	E2FF-NFKB	513 - 404	(-)	
**GXP_294651** [GXP_294651] (1 - 731)<br>**KLHDC1**, GXL_247754, GeneID: 122773, Homo sapiens chr. 14<br>kelch domain containing 1	E2FF-NFKB	593 - 704	(+)	
**GXP_294691** [GXP_294691] (1 - 601)<br>**LTB4R**, GXL_247777, GeneID: 1241, Homo sapiens chr. 14<br>leukotriene B4 receptor	E2FF-NFKB	163 - 34	(-)	
**GXP_294709** [GXP_294709] (1 - 601)<br>**MGAT2**, GXL_247787, GeneID: 4247, Homo sapiens chr. 14<br>mannosyl (alpha-1,6-)-glycoprotein beta-1, 2-N-acetylglucosaminyltransferase	E2FF-NFKB	251 - 366	(+)	
**GXP_908357** [GXP_908357] (1 - 886)<br>**LRRC9**, GXL_247801, GeneID: 341883, Homo sapiens chr. 14<br>leucine rich repeat containing 9	E2FF-NFKB	712 - 594	(-)	
**GXP_294803** [GXP_294803] (1 - 601)<br>**AP4S1**, GXL_247852, GeneID: 11154, Homo sapiens chr. 14<br>adaptor-related protein complex 4, sigma 1 subunit	E2FF-NFKB	272 - 391	(+)	
**GXP_294808** [GXP_294808] (1 - 896)<br>**NFATC4**, GXL_247857, GeneID: 4776, Homo sapiens chr. 14<br>nuclear factor of activated T-cells, cytoplasmic, calcineurin-dependent 4	E2FF-NFKB	379 - 260	(-)	
**GXP_294847** [GXP_294847] (1 - 660)<br>**KIAA0323**, GXL_247889, GeneID: 23351, Homo sapiens chr. 14<br>KIAA0323	E2FF-NFKB	472 - 598	(+)	
**GXP_294886** [GXP_294886] (1 - 866)<br>**PRKCH**, GXL_247921, GeneID: 5583, Homo sapiens chr. 14<br>protein kinase C, eta	E2FF-NFKB	706 - 834	(+)	
**GXP_908373** [GXP_908373] (1 - 601)<br>**PRKCH**, GXL_247921, GeneID: 5583, Homo sapiens chr. 14<br>protein kinase C, eta	E2FF-NFKB	452 - 574	(+)	
**GXP_294891** [GXP_294891] (1 - 601)<br>**KIAA0247**, GXL_247925, GeneID: 9766, Homo sapiens chr. 14<br>KIAA0247	E2FF-NFKB	112 - 231	(+)	
	E2FF-NFKB	225 - 97	(-)	

**GXP_908364** [GXP_908364] (1 - 601)<br>**PPM1A**, GXL_247940, GeneID: 5494, Homo sapiens chr. 14<br>protein phosphatase 1A (formerly 2C), magnesium-dependent, alpha isoform				
**GXP_295893** [GXP_295893] (1 - 601)<br>**NCOA4**, GXL_248744, GeneID: 8031, Homo sapiens chr. 10<br>nuclear receptor coactivator 4	E2FF-NFKB	464 - 356	(-)	
**GXP_295898** [GXP_295898] (1 - 1280)<br>**STOX1**, GXL_248747, GeneID: 219736, Homo sapiens chr. 10<br>storkhead box 1	E2FF-NFKB<br>E2FF-NFKB	56 - 170<br>1082 - 957	(+)<br>(-)	
**GXP_638701** [GXP_638701] (1 - 601)<br>**STOX1**, GXL_248747, GeneID: 219736, Homo sapiens chr. 10<br>storkhead box 1	E2FF-NFKB	403 - 278	(-)	
**GXP_904407** [GXP_904407] (1 - 601)<br>**FAS**, GXL_248764, GeneID: 355, Homo sapiens chr. 10<br>Fas (TNF receptor superfamily, member 6)	E2FF-NFKB	448 - 567	(+)	
**GXP_904174** [GXP_904174] (1 - 601)<br>**PHYHIPL**, GXL_248775, GeneID: 84457, Homo sapiens chr. 10<br>phytanoyl-CoA 2-hydroxylase interacting protein-like	E2FF-NFKB	454 - 571	(+)	
**GXP_296002** [GXP_296002] (1 - 731)<br>**VCL**, GXL_248812, GeneID: 7414, Homo sapiens chr. 10<br>vinculin	E2FF-NFKB	411 - 532	(+)	
**GXP_296041** [GXP_296041] (1 - 830)<br>**GHITM**, GXL_248837, GeneID: 27069, Homo sapiens chr. 10<br>growth hormone inducible transmembrane protein	E2FF-NFKB	54 - 179	(+)	
**GXP_296063** [GXP_296063] (1 - 1163)<br>**ADAMTS14**, GXL_248850, GeneID: 140766, Homo sapiens chr. 10<br>ADAM metallopeptidase with thrombospondin type 1 motif, 14	E2FF-NFKB	862 - 745	(-)	
**GXP_296097** [GXP_296097] (1 - 631)<br>**BMPR1A**, GXL_248874, GeneID: 657, Homo sapiens chr. 10<br>bone morphogenetic protein receptor, type IA	E2FF-NFKB	427 - 315	(-)	
**GXP_904444** [GXP_904444] (1 - 602)<br>**PLCE1**, GXL_248876, GeneID: 51196, Homo sapiens chr. 10<br>phospholipase C, epsilon 1	E2FF-NFKB	283 - 398	(+)	
**GXP_296138** [GXP_296138] (1 - 637)<br>**ZNF33A**, GXL_248902, GeneID: 7581, Homo sapiens chr. 10<br>zinc finger protein 33A	E2FF-NFKB	568 - 443	(-)	
**GXP_479255** [GXP_479255] (1 - 601)<br>**DDX50**, GXL_248912, GeneID: 79009, Homo sapiens chr. 10<br>DEAD (Asp-Glu-Ala-Asp) box polypeptide 50	E2FF-NFKB	447 - 338	(-)	
	E2FF-NFKB	495 - 369	(-)	

**GXP_296184** [GXP_296184] (1 - 658)<br>**GPR120**, GXL_248940, GeneID: 338557, Homo sapiens chr. 10<br>G protein-coupled receptor 120				
**GXP_296220** [GXP_296220] (1 - 601)<br>**SLC18A3**, GXL_248969, GeneID: 6572, Homo sapiens chr. 10<br>solute carrier family 18 (vesicular acetylcholine), member 3	E2FF-NFKB	169 - 59	(-)	
**GXP_296221** [GXP_296221] (1 - 602)<br>**IFIT1L**, GXL_248970, GeneID: 439996, Homo sapiens chr. 10<br>interferon-induced protein with tetratricopeptide repeats 1-like	E2FF-NFKB	420 - 299	(-)	
**GXP_296248** [GXP_296248] (1 - 1268)<br>**BMS1L**, GXL_248996, GeneID: 9790, Homo sapiens chr. 10<br>BMS1-like, ribosome assembly protein (yeast)	E2FF-NFKB	280 - 170	(-)	
**GXP_296250** [GXP_296250] (1 - 611)<br>**C10orf35**, GXL_248998, GeneID: 219738, Homo sapiens chr. 10<br>chromosome 10 open reading frame 35	E2FF-NFKB	339 - 231	(-)	
**GXP_904238** [GXP_904238] (1 - 613)<br>**C10orf35**, GXL_248998, GeneID: 219738, Homo sapiens chr. 10<br>chromosome 10 open reading frame 35	E2FF-NFKB	277 - 169	(-)	
**GXP_297067** [GXP_297067] (1 - 601)<br>**CLDN5**, GXL_249621, GeneID: 7122, Homo sapiens chr. 22<br>claudin 5 (transmembrane protein deleted in velocardiofacial syndrome)	E2FF-NFKB	222 - 332	(+)	
**GXP_917404** [GXP_917404] (1 - 601)<br>**THAP7**, GXL_249622, GeneID: 80764, Homo sapiens chr. 22<br>THAP domain containing 7	E2FF-NFKB	251 - 362	(+)	
**GXP_297113** [GXP_297113] (1 - 688)<br>**PHF5A**, GXL_249647, GeneID: 84844, Homo sapiens chr. 22<br>PHD finger protein 5A	E2FF-NFKB	434 - 556	(+)	
**GXP_297135** [GXP_297135] (1 - 601)<br>**FOXRED2**, GXL_249660, GeneID: 80020, Homo sapiens chr. 22<br>FAD-dependent oxidoreductase domain containing 2	E2FF-NFKB	462 - 355	(-)	
**GXP_297212** [GXP_297212] (1 - 737)<br>**C22orf13**, GXL_249703, GeneID: 83606, Homo sapiens chr. 22<br>chromosome 22 open reading frame 13	E2FF-NFKB	438 - 312	(-)	
**GXP_297296** [GXP_297296] (1 - 812)<br>**DMC1**, GXL_249753, GeneID: 11144, Homo sapiens chr. 22<br>DMC1 dosage suppressor of mck1 homolog, meiosis-specific homologous recombination (yeast)	E2FF-NFKB	135 - 261	(+)	
**GXP_297307** [GXP_297307] (1 - 601)<br>**TPST2**, GXL_249759, GeneID: 8459, Homo sapiens chr. 22<br>tyrosylprotein sulfotransferase 2	E2FF-NFKB	257 - 369	(+)	
	E2FF-NFKB	394 - 522	(+)	

**GXP_297312** [GXP_297312] (1 - 692)<br>**LIF**, GXL_249763, GeneID: 3976, Homo sapiens chr. 22<br>leukemia inhibitory factor (cholinergic differentiation factor)				
**GXP_917675** [GXP_917675] (1 - 601)<br>**DDX17**, GXL_249785, GeneID: 10521, Homo sapiens chr. 22<br>DEAD (Asp-Glu-Ala-Asp) box polypeptide 17	E2FF-NFKB	208 - 80	(-)	
**GXP_297357** [GXP_297357] (1 - 601)<br>**L3MBTL2**, GXL_249799, GeneID: 83746, Homo sapiens chr. 22<br>l(3)mbt-like 2 (Drosophila)	E2FF-NFKB	186 - 58	(-)	
**GXP_297362** [GXP_297362] (1 - 905)<br>**EIF3S7,LOC646756**, GXL_249804, GeneID: 8664,646756, Homo sapiens chr. 22<br>eukaryotic translation initiation factor 3, subunit 7 zeta, 66/67kDa; similar to Hermansky-Pudlak syndrome 1 protein isoform b	E2FF-NFKB	518 - 403	(-)	
**GXP_917630** [GXP_917630] (1 - 779)<br>**EIF3S7,LOC646756**, GXL_249804, GeneID: 8664,646756, Homo sapiens chr. 22<br>eukaryotic translation initiation factor 3, subunit 7 zeta, 66/67kDa; similar to Hermansky-Pudlak syndrome 1 protein isoform b	E2FF-NFKB	160 - 45	(-)	
**GXP_652715** [GXP_652715] (1 - 601)<br>**GSCL**, GXL_249817, GeneID: 2928, Homo sapiens chr. 22<br>goosecoid-like	E2FF-NFKB	508 - 386	(-)	
**GXP_917688** [GXP_917688] (1 - 601)<br>**UNC84B**, GXL_249830, GeneID: 25777, Homo sapiens chr. 22<br>unc-84 homolog B (C. elegans)	E2FF-NFKB	534 - 413	(-)	
**GXP_917350** [GXP_917350] (1 - 601)<br>**SLC25A1**, GXL_249831, GeneID: 6576, Homo sapiens chr. 22<br>solute carrier family 25 (mitochondrial carrier, citrate transporter), member 1	E2FF-NFKB	445 - 552	(+)	
**GXP_917500** [GXP_917500] (1 - 641)<br>**CRYBB1**, GXL_249863, GeneID: 1414, Homo sapiens chr. 22<br>crystallin, beta B1	E2FF-NFKB	499 - 611	(+)	
**GXP_300072** [GXP_300072] (1 - 960)<br>**CRK**, GXL_252084, GeneID: 1398, Homo sapiens chr. 17<br>v-crk sarcoma virus CT10 oncogene homolog (avian)	E2FF-NFKB	945 - 831	(-)	
**GXP_300082** [GXP_300082] (1 - 601)<br>**TRPV1,CARKL**, GXL_252089, GeneID: 7442,23729, Homo sapiens chr. 17<br>transient receptor potential cation channel, subfamily V, member 1;carbohydrate kinase-like	E2FF-NFKB	276 - 149	(-)	
**GXP_300083** [GXP_300083] (1 - 601)<br>**TRPV1,CARKL**, GXL_252089, GeneID: 7442,23729, Homo sapiens chr. 17<br>transient receptor potential cation channel, subfamily V, member 1;carbohydrate kinase-like	E2FF-NFKB	504 - 389	(-)	
**GXP_300102** [GXP_300102] (1 - 661)<br>**MYBBP1A**, GXL_252099, GeneID: 10514, Homo sapiens chr. 17<br>MYB binding protein (P160) 1a	E2FF-NFKB	281 - 404	(+)	
	E2FF-NFKB	595 - 484	(-)	

**GXP_300126** [GXP_300126] (1 - 696)<br>**PMP22**, GXL_252109, GeneID: 5376, Homo sapiens chr. 17<br>peripheral myelin protein 22				
**GXP_300137** [GXP_300137] (1 - 601)<br>**ZZEF1**, GXL_252115, GeneID: 23140, Homo sapiens chr. 17<br>zinc finger, ZZ-type with EF-hand domain 1	E2FF-NFKB	307 - 416	(+)	
**GXP_645964** [GXP_645964] (1 - 601)<br>**ZZEF1**, GXL_252115, GeneID: 23140, Homo sapiens chr. 17<br>zinc finger, ZZ-type with EF-hand domain 1	E2FF-NFKB	123 - 250	(+)	
**GXP_300147** [GXP_300147] (1 - 796)<br>**SREBF1**, GXL_252119, GeneID: 6720, Homo sapiens chr. 17<br>sterol regulatory element binding transcription factor 1	E2FF-NFKB	332 - 212	(-)	
**GXP_300151** [GXP_300151] (1 - 763)<br>**SMG6**, GXL_252120, GeneID: 23293, Homo sapiens chr. 17<br>Smg-6 homolog, nonsense mediated mRNA decay factor (C. elegans)	E2FF-NFKB	211 - 86	(-)	
**GXP_483941** [GXP_483941] (1 - 601)<br>**SMG6**, GXL_252120, GeneID: 23293, Homo sapiens chr. 17<br>Smg-6 homolog, nonsense mediated mRNA decay factor (C. elegans)	E2FF-NFKB	487 - 362	(-)	
**GXP_910961** [GXP_910961] (1 - 601)<br>**GPS2,KIAA1787**, GXL_252127, GeneID: 2874,84461, Homo sapiens chr. 17<br>G protein pathway suppressor 2;KIAA1787 protein	E2FF-NFKB	184 - 298	(+)	
**GXP_646089** [GXP_646089] (1 - 686)<br>**KRBA2**, GXL_252178, GeneID: 124751, Homo sapiens chr. 17<br>KRAB-A domain containing 2	E2FF-NFKB	152 - 260	(+)	
**GXP_300284** [GXP_300284] (1 - 932)<br>**DLG4**, GXL_252202, GeneID: 1742, Homo sapiens chr. 17<br>discs, large homolog 4 (Drosophila)	E2FF-NFKB	151 - 271	(+)	
**GXP_300295** [GXP_300295] (1 - 903)<br>**C17orf76**, GXL_252210, GeneID: 388341, Homo sapiens chr. 17<br>chromosome 17 open reading frame 76	E2FF-NFKB	532 - 648	(+)	
**GXP_300320** [GXP_300320] (1 - 1088)<br>**GPR172B**, GXL_252231, GeneID: 55065, Homo sapiens chr. 17<br>G protein-coupled receptor 172B	E2FF-NFKB	595 - 472	(-)	
**GXP_300324** [GXP_300324] (1 - 1107)<br>**CLDN7**, GXL_252234, GeneID: 1366, Homo sapiens chr. 17<br>claudin 7	E2FF-NFKB	169 - 281	(+)	
**GXP_300422** [GXP_300422] (1 - 659)<br>**ATPAF2**, GXL_252322, GeneID: 91647, Homo sapiens chr. 17<br>ATP synthase mitochondrial F1 complex assembly factor 2	E2FF-NFKB	74 - 189	(+)	
	E2FF-NFKB	87 - 202	(+)	

**GXP_646182** [GXP_646182] (1 - 601)<br>**ATPAF2**, GXL_252322, GeneID: 91647, Homo sapiens chr. 17<br>ATP synthase mitochondrial F1 complex assembly factor 2				
**GXP_310465** [GXP_310465] (1 - 640)<br>**DSE**, GXL_261159, GeneID: 29940, Homo sapiens chr. 6<br>dermatan sulfate epimerase	E2FF-NFKB	474 - 347	(-)	
**GXP_341092** [GXP_341092] (1 - 601)<br>**TFAP2B**, GXL_261163, GeneID: 7021, Homo sapiens chr. 6<br>transcription factor AP-2 beta (activating enhancer binding protein 2 beta)	E2FF-NFKB	387 - 265	(-)	
**GXP_923707** [GXP_923707] (1 - 601)<br>**DNAH8**, GXL_261170, GeneID: 1769, Homo sapiens chr. 6<br>dynein, axonemal, heavy chain 8	E2FF-NFKB	483 - 367	(-)	
**GXP_923829** [GXP_923829] (1 - 601)<br>**OPN5**, GXL_261206, GeneID: 221391, Homo sapiens chr. 6<br>opsin 5	E2FF-NFKB	254 - 142	(-)	
**GXP_310571** [GXP_310571] (1 - 799)<br>**SMAP1**, GXL_261215, GeneID: 60682, Homo sapiens chr. 6<br>stromal membrane-associated protein 1	E2FF-NFKB	481 - 366	(-)	
**GXP_310598** [GXP_310598] (1 - 708)<br>**POLR1C**, GXL_261230, GeneID: 9533, Homo sapiens chr. 6<br>polymerase (RNA) I polypeptide C, 30kDa	E2FF-NFKB	259 - 146	(-)	
**GXP_923866** [GXP_923866] (1 - 601)<br>**FBXO9**, GXL_261243, GeneID: 26268, Homo sapiens chr. 6<br>F-box protein 9	E2FF-NFKB	210 - 86	(-)	
**GXP_310642** [GXP_310642] (1 - 601)<br>**C6orf49**, GXL_261251, GeneID: 29964, Homo sapiens chr. 6<br>chromosome 6 open reading frame 49	E2FF-NFKB	542 - 433	(-)	
**GXP_923741** [GXP_923741] (1 - 785)<br>**C6orf49**, GXL_261251, GeneID: 29964, Homo sapiens chr. 6<br>chromosome 6 open reading frame 49	E2FF-NFKB	726 - 617	(-)	
**GXP_310669** [GXP_310669] (1 - 769)<br>**TMEM63B**, GXL_261270, GeneID: 55362, Homo sapiens chr. 6<br>transmembrane protein 63B	E2FF-NFKB	410 - 290	(-)	
**GXP_658222** [GXP_658222] (1 - 601)<br>**HSP90AB1**, GXL_261287, GeneID: 3326, Homo sapiens chr. 6<br>heat shock protein 90kDa alpha (cytosolic), class B member 1	E2FF-NFKB	369 - 487	(+)	
**GXP_310699** [GXP_310699] (1 - 632)<br>**PPP2R5D**, GXL_261288, GeneID: 5528, Homo sapiens chr. 6<br>protein phosphatase 2, regulatory subunit B', delta isoform	E2FF-NFKB	127 - 256	(+)	
	E2FF-NFKB	383 - 266	(-)	

**GXP_310722** [GXP_310722] (1 - 842)<br>**KLHL32**, GXL_261302, GeneID: 114792, Homo sapiens chr. 6<br>kelch-like 32 (Drosophila)				
**GXP_310729** [GXP_310729] (1 - 805)<br>**KCNQ5**, GXL_261309, GeneID: 56479, Homo sapiens chr. 6<br>potassium voltage-gated channel, KQT-like subfamily, member 5	E2FF-NFKB	300 - 406	(+)	
**GXP_310751** [GXP_310751] (1 - 898)<br>**LRRC1,LOC730120,LOC732300**, GXL_261327, GeneID: 55227,730120,732300, Homo sapiens chr. 6<br>leucine rich repeat containing 1; hypothetical protein LOC730120; hypothetical protein LOC732300	E2FF-NFKB	399 - 512	(+)	
**GXP_310768** [GXP_310768] (1 - 967)<br>**C6orf203**, GXL_261339, GeneID: 51250, Homo sapiens chr. 6<br>chromosome 6 open reading frame 203	E2FF-NFKB	582 - 705	(+)	
**GXP_310784** [GXP_310784] (1 - 730)<br>**SPACA1**, GXL_261353, GeneID: 81833, Homo sapiens chr. 6<br>sperm acrosome associated 1	E2FF-NFKB	457 - 346	(-)	
**GXP_310801** [GXP_310801] (1 - 682)<br>**BCKDHB**, GXL_261369, GeneID: 594, Homo sapiens chr. 6<br>branched chain keto acid dehydrogenase E1, beta polypeptide (maple syrup urine disease)	E2FF-NFKB	430 - 309	(-)	
**GXP_310805** [GXP_310805] (1 - 601)<br>**FUT9**, GXL_261373, GeneID: 10690, Homo sapiens chr. 6<br>fucosyltransferase 9 (alpha (1,3) fucosyltransferase)	E2FF-NFKB	75 - 195	(+)	
**GXP_923915** [GXP_923915] (1 - 601)<br>**BAI3**, GXL_261410, GeneID: 577, Homo sapiens chr. 6<br>brain-specific angiogenesis inhibitor 3	E2FF-NFKB	396 - 273	(-)	
**GXP_311614** [GXP_311614] (1 - 712)<br>**IXL**, GXL_262102, GeneID: 55588, Homo sapiens chr. 19<br>intersex-like (Drosophila)	E2FF-NFKB	48 - 163	(+)	
**GXP_311643** [GXP_311643] (1 - 719)<br>**CAPNS1**, GXL_262121, GeneID: 826, Homo sapiens chr. 19<br>calpain, small subunit 1	E2FF-NFKB	699 - 584	(-)	
**GXP_311654** [GXP_311654] (1 - 1125)<br>**MRPS12**, GXL_262128, GeneID: 6183, Homo sapiens chr. 19<br>mitochondrial ribosomal protein S12	E2FF-NFKB	537 - 645	(+)	
	E2FF-NFKB	678 - 556	(-)	
**GXP_311662** [GXP_311662] (1 - 714)<br>**PLEKHG2**, GXL_262132, GeneID: 64857, Homo sapiens chr. 19<br>pleckstrin homology domain containing, family G (with RhoGef domain) member 2	E2FF-NFKB	679 - 565	(-)	
**GXP_913244** [GXP_913244] (1 - 601)<br>**APLP1**, GXL_262156, GeneID: 333, Homo sapiens chr. 19<br>amyloid beta (A4) precursor-like protein 1	E2FF-NFKB	199 - 323	(+)	
	E2FF-NFKB	421 - 535	(+)	

**GXP_311738** [GXP_311738] (1 - 613)<br>**PVR**, GXL_262173, GeneID: 5817, Homo sapiens chr. 19<br>poliovirus receptor				
**GXP_311765** [GXP_311765] (1 - 746)<br>**CLPTM1**, GXL_262189, GeneID: 1209, Homo sapiens chr. 19<br>cleft lip and palate associated transmembrane protein 1	E2FF-NFKB	589 - 480	(-)	
**GXP_311785** [GXP_311785] (1 - 601)<br>**CYP2F1**, GXL_262203, GeneID: 1572, Homo sapiens chr. 19<br>cytochrome P450, family 2, subfamily F, polypeptide 1	E2FF-NFKB	207 - 78	(-)	
**GXP_311889** [GXP_311889] (1 - 627)<br>**FXYD7**, GXL_262280, GeneID: 53822, Homo sapiens chr. 19<br>FXYD domain containing ion transport regulator 7	E2FF-NFKB	69 - 178	(+)	
**GXP_913224** [GXP_913224] (1 - 1458)<br>**GAPDHS**, GXL_262327, GeneID: 26330, Homo sapiens chr. 19<br>glyceraldehyde-3-phosphate dehydrogenase, spermatogenic	E2FF-NFKB	698 - 804	(+)	
**GXP_648806** [GXP_648806] (1 - 601)<br>**MEGF8**, GXL_262348, GeneID: 1954, Homo sapiens chr. 19<br>multiple EGF-like-domains 8	E2FF-NFKB	71 - 195	(+)	
**GXP_311988** [GXP_311988] (1 - 601)<br>**BTBD14B**, GXL_262356, GeneID: 112939, Homo sapiens chr. 19<br>BTB (POZ) domain containing 14B	E2FF-NFKB	195 - 86	(-)	
**GXP_312000** [GXP_312000] (1 - 601)<br>**SMARCA4**, GXL_262362, GeneID: 6597, Homo sapiens chr. 19<br>SWI/SNF related, matrix associated, actin dependent regulator of chromatin, subfamily a, member 4	E2FF-NFKB	55 - 173	(+)	
**GXP_912877** [GXP_912877] (1 - 601)<br>**SMARCA4**, GXL_262362, GeneID: 6597, Homo sapiens chr. 19<br>SWI/SNF related, matrix associated, actin dependent regulator of chromatin, subfamily a, member 4	E2FF-NFKB	330 - 456	(+)	
**GXP_648372** [GXP_648372] (1 - 601)<br>**CRTC1**, GXL_262369, GeneID: 23373, Homo sapiens chr. 19<br>CREB regulated transcription coactivator 1	E2FF-NFKB	310 - 191	(-)	
**GXP_648113** [GXP_648113] (1 - 601)<br>**DNM2**, GXL_262376, GeneID: 1785, Homo sapiens chr. 19<br>dynamin 2	E2FF-NFKB	452 - 570	(+)	
**GXP_312067** [GXP_312067] (1 - 601)<br>**SLC44A2**, GXL_262395, GeneID: 57153, Homo sapiens chr. 19<br>solute carrier family 44, member 2	E2FF-NFKB	396 - 287	(-)	
**GXP_912867** [GXP_912867] (1 - 601)<br>**SLC44A2**, GXL_262395, GeneID: 57153, Homo sapiens chr. 19<br>solute carrier family 44, member 2	E2FF-NFKB	314 - 205	(-)	
	E2FF-NFKB	368 - 478	(+)	

**GXP_312115** [GXP_312115] (1 - 689)<br>**C19orf44**, GXL_262423, GeneID: 84167, Homo sapiens chr. 19<br>chromosome 19 open reading frame 44				
**GXP_312122** [GXP_312122] (1 - 935)<br>**MRPL4**, GXL_262427, GeneID: 51073, Homo sapiens chr. 19<br>mitochondrial ribosomal protein L4	E2FF-NFKB	187 - 80	(-)	
**GXP_312135** [GXP_312135] (1 - 823)<br>**ZNF257**, GXL_262436, GeneID: 113835, Homo sapiens chr. 19<br>zinc finger protein 257	E2FF-NFKB	649 - 775	(+)	
**GXP_312137** [GXP_312137] (1 - 660)<br>**ZNF101**, GXL_262438, GeneID: 94039, Homo sapiens chr. 19<br>zinc finger protein 101	E2FF-NFKB	458 - 581	(+)	
**GXP_312144** [GXP_312144] (1 - 1044)<br>**RGS9BP**, GXL_262442, GeneID: 388531, Homo sapiens chr. 19<br>regulator of G protein signalling 9 binding protein	E2FF-NFKB	114 - 222	(+)	
**GXP_312149** [GXP_312149] (1 - 632)<br>**CYP4F12**, GXL_262445, GeneID: 66002, Homo sapiens chr. 19<br>cytochrome P450, family 4, subfamily F, polypeptide 12	E2FF-NFKB	490 - 596	(+)	
**GXP_312153** [GXP_312153] (1 - 620)<br>**C19orf50**, GXL_262447, GeneID: 79036, Homo sapiens chr. 19<br>chromosome 19 open reading frame 50	E2FF-NFKB	424 - 551	(+)	
**GXP_312161** [GXP_312161] (1 - 603)<br>**ARRDC2**, GXL_262453, GeneID: 27106, Homo sapiens chr. 19<br>arrestin domain containing 2	E2FF-NFKB	535 - 412	(-)	
**GXP_912900** [GXP_912900] (1 - 601)<br>**ZNF763,LOC729745**, GXL_262460, GeneID: 284390,729745, Homo sapiens chr. 19<br>zinc finger protein 763; similar to zinc finger protein 700	E2FF-NFKB	350 - 476	(+)	
**GXP_312191** [GXP_312191] (1 - 602)<br>**SLC25A42**, GXL_262469, GeneID: 284439, Homo sapiens chr. 19<br>solute carrier family 25, member 42	E2FF-NFKB	487 - 376	(-)	
**GXP_312234** [GXP_312234] (1 - 637)<br>**FLJ44968**, GXL_262498, GeneID: 374887, Homo sapiens chr. 19<br>FLJ44968 protein	E2FF-NFKB	339 - 467	(+)	
**GXP_912875** [GXP_912875] (1 - 601)<br>**CARM1**, GXL_262521, GeneID: 10498, Homo sapiens chr. 19<br>coactivator-associated arginine methyltransferase 1	E2FF-NFKB	98 - 218	(+)	
**GXP_912889** [GXP_912889] (1 - 624)<br>**CNN1**, GXL_262532, GeneID: 1264, Homo sapiens chr. 19<br>calponin 1, basic, smooth muscle	E2FF-NFKB	176 - 296	(+)	
	E2FF-NFKB	315 - 430	(+)	

**GXP_312290** [GXP_312290] (1 - 612)<br>**MORG1**, GXL_262548, GeneID: 84292, Homo sapiens chr. 19<br>mitogen-activated protein kinase organizer 1	E2FF-NFKB	437 - 553	(+)	
**GXP_477170** [GXP_477170] (1 - 876)<br>**PLCH2**, GXL_316511, GeneID: 9651, Homo sapiens chr. 1<br>phospholipase C, eta 2	E2FF-NFKB	317 - 201	(-)	
**GXP_478940** [GXP_478940] (1 - 646)<br>**C1orf31**, GXL_316566, GeneID: 388753, Homo sapiens chr. 1<br>chromosome 1 open reading frame 31	E2FF-NFKB	140 - 268	(+)	
**GXP_916367** [GXP_916367] (1 - 601)<br>**C1orf31**, GXL_316566, GeneID: 388753, Homo sapiens chr. 1<br>chromosome 1 open reading frame 31	E2FF-NFKB	355 - 483	(+)	
**GXP_479184** [GXP_479184] (1 - 848)<br>**PARG,LOC727726,LOC728407**, GXL_316576, GeneID: 8505,727726,728407, Homo sapiens chr. 10<br>poly (ADP-ribose) glycohydrolase; similar to poly (ADP-ribose) glycohydrolase; similar to Poly(ADP-ribose) glycohydrolase	E2FF-NFKB	216 - 337	(+)	
**GXP_639319** [GXP_639319] (1 - 601)<br>**SPRN**, GXL_316589, GeneID: 503542, Homo sapiens chr. 10<br>shadow of prion protein homolog (zebrafish)	E2FF-NFKB	257 - 363	(+)	
**GXP_204658** [GXP_204658] (1 - 655)<br>**GPIAP1**, GXL_316602, GeneID: 4076, Homo sapiens chr. 11<br>GPI-anchored membrane protein 1	E2FF-NFKB	446 - 318	(-)	
**GXP_906000** [GXP_906000] (1 - 601)<br>**RDX**, GXL_316615, GeneID: 5962, Homo sapiens chr. 11<br>radixin	E2FF-NFKB	231 - 116	(-)	
**GXP_481421** [GXP_481421] (1 - 604)<br>**CLLU1OS**, GXL_316632, GeneID: 574016, Homo sapiens chr. 12<br>chronic lymphocytic leukemia up-regulated 1 opposite strand	E2FF-NFKB	21 - 136	(+)	
**GXP_909097** [GXP_909097] (1 - 668)<br>**PLA2G4D**, GXL_316673, GeneID: 283748, Homo sapiens chr. 15<br>phospholipase A2, group IVD (cytosolic)	E2FF-NFKB	603 - 481	(-)	
**GXP_200818** [GXP_200818] (1 - 907)<br>**ARHGDIG**, GXL_316690, GeneID: 398, Homo sapiens chr. 16<br>Rho GDP dissociation inhibitor (GDI) gamma	E2FF-NFKB	174 - 59	(-)	
**GXP_43569** [GXP_43569] (1 - 964)<br>**ACOX1**, GXL_316751, GeneID: 51, Homo sapiens chr. 17<br>acyl-Coenzyme A oxidase 1, palmitoyl	E2FF-NFKB	488 - 598	(+)	
	E2FF-NFKB	695 - 584	(-)	
**GXP_210437** [GXP_210437] (1 - 601)<br>**RNF213**, GXL_316752, GeneID: 57674, Homo sapiens chr. 17<br>ring finger protein 213	E2FF-NFKB	185 - 70	(-)	
	E2FF-NFKB	329 - 455	(+)	

**GXP_484878** [GXP_484878] (1 - 601)<br>**RNF213**, GXL_316752, GeneID: 57674, Homo sapiens chr. 17<br>ring finger protein 213				
**GXP_485002** [GXP_485002] (1 - 606)<br>**MC2R**, GXL_316756, GeneID: 4158, Homo sapiens chr. 18<br>melanocortin 2 receptor (adrenocorticotropic hormone)	E2FF-NFKB	177 - 63	(-)	
**GXP_912251** [GXP_912251] (1 - 601)<br>**MC2R**, GXL_316756, GeneID: 4158, Homo sapiens chr. 18<br>melanocortin 2 receptor (adrenocorticotropic hormone)	E2FF-NFKB	344 - 230	(-)	
**GXP_486591** [GXP_486591] (1 - 888)<br>**RTKN**, GXL_316799, GeneID: 6242, Homo sapiens chr. 2<br>rhotekin	E2FF-NFKB	449 - 332	(-)	
**GXP_917592** [GXP_917592] (1 - 601)<br>**RFPL3S**, GXL_316856, GeneID: 10737, Homo sapiens chr. 22<br>ret finger protein-like 3 antisense	E2FF-NFKB	440 - 564	(+)	
**GXP_653201** [GXP_653201] (1 - 619)<br>**TNRC6B**, GXL_316857, GeneID: 23112, Homo sapiens chr. 22<br>trinucleotide repeat containing 6B	E2FF-NFKB	158 - 266	(+)	
**GXP_488399** [GXP_488399] (1 - 791)<br>**D15Wsu75e**, GXL_316859, GeneID: 27351, Homo sapiens chr. 22<br>DNA segment, Chr 15, Wayne State University 75, expressed	E2FF-NFKB	600 - 712	(+)	
**GXP_58231** [GXP_58231] (1 - 601)<br>**PPARA**, GXL_316862, GeneID: 5465, Homo sapiens chr. 22<br>peroxisome proliferator-activated receptor alpha	E2FF-NFKB	5 - 134	(+)	
**GXP_277606** [GXP_277606] (1 - 631)<br>**KLHL24**, GXL_316887, GeneID: 54800, Homo sapiens chr. 3<br>kelch-like 24 (Drosophila)	E2FF-NFKB	503 - 375	(-)	
**GXP_116457** [GXP_116457] (1 - 851)<br>**GTF2IRD2**, GXL_316975, GeneID: 84163, Homo sapiens chr. 7<br>GTF2I repeat domain containing 2	E2FF-NFKB	714 - 834	(+)	
**GXP_18962** [GXP_18962] (1 - 601)<br>**NTRK2**, GXL_317026, GeneID: 4915, Homo sapiens chr. 9<br>neurotrophic tyrosine kinase, receptor, type 2	E2FF-NFKB	25 - 150	(+)	
**GXP_663584** [GXP_663584] (1 - 601)<br>**ZNF711**, GXL_317068, GeneID: 7552, Homo sapiens chr. X<br>zinc finger protein 711	E2FF-NFKB	239 - 127	(-)	
**GXP_634746** [GXP_634746] (1 - 601)<br>**MIB2**, GXL_379913, GeneID: 142678, Homo sapiens chr. 1<br>mindbomb homolog 2 (Drosophila)	E2FF-NFKB	433 - 547	(+)	
	E2FF-NFKB	422 - 315	(-)	

**GXP_635684** [GXP_635684] (1 - 709)<br>**hCG_23177**, GXL_380017, GeneID: 440585, Homo sapiens chr. 1<br>hCG23177				
**GXP_180776** [GXP_180776] (1 - 601)<br>**C1orf186**, GXL_380287, GeneID: 440712, Homo sapiens chr. 1<br>chromosome 1 open reading frame 186	E2FF-NFKB	403 - 531	(+)	
**GXP_23431** [GXP_23431] (1 - 601)<br>**DUSP5P**, GXL_380326, GeneID: 574029, Homo sapiens chr. 1<br>dual specificity phosphatase 5 pseudogene	E2FF-NFKB	363 - 474	(+)	
**GXP_639173** [GXP_639173] (1 - 654)<br>**SLC18A2**, GXL_380528, GeneID: 6571, Homo sapiens chr. 10<br>solute carrier family 18 (vesicular monoamine), member 2	E2FF-NFKB	536 - 418	(-)	
**GXP_904739** [GXP_904739] (1 - 601)<br>**NKX1-2**, GXL_380543, GeneID: 390010, Homo sapiens chr. 10<br>NK1 transcription factor related, locus 2 (Drosophila)	E2FF-NFKB	384 - 255	(-)	
**GXP_904740** [GXP_904740] (1 - 601)<br>**NKX1-2**, GXL_380543, GeneID: 390010, Homo sapiens chr. 10<br>NK1 transcription factor related, locus 2 (Drosophila)	E2FF-NFKB	567 - 438	(-)	
**GXP_904741** [GXP_904741] (1 - 860)<br>**NKX1-2**, GXL_380543, GeneID: 390010, Homo sapiens chr. 10<br>NK1 transcription factor related, locus 2 (Drosophila)	E2FF-NFKB	592 - 485	(-)	
**GXP_204620** [GXP_204620] (1 - 640)<br>**DAK**, GXL_380673, GeneID: 26007, Homo sapiens chr. 11<br>dihydroxyacetone kinase 2 homolog (S. cerevisiae)	E2FF-NFKB	44 - 169	(+)	
**GXP_193422** [GXP_193422] (1 - 1136)<br>**PVRL1**, GXL_380785, GeneID: 5818, Homo sapiens chr. 11<br>poliovirus receptor-related 1 (herpesvirus entry mediator C, nectin)	E2FF-NFKB	624 - 735	(+)	
**GXP_641307** [GXP_641307] (1 - 783)<br>**COL2A1,LOC728181,LOC730690**, GXL_380870, GeneID: 1280,728181,730690, Homo sapiens chr. 12<br>collagen, type II, alpha 1 (primary osteoarthritis, spondyloepiphyseal dysplasia, congenital); hypothetical protein LOC728181; hypothetical protein LOC730690	E2FF-NFKB	589 - 705	(+)	
**GXP_907470** [GXP_907470] (1 - 625)<br>**LRRC43**, GXL_380973, GeneID: 254050, Homo sapiens chr. 12<br>leucine rich repeat containing 43	E2FF-NFKB	450 - 327	(-)	
**GXP_642284** [GXP_642284] (1 - 1205)<br>**FLJ39378,LOC728618**, GXL_380976, GeneID: 353116,728618, Homo sapiens chr. 12<br>hypothetical protein FLJ39378; hypothetical protein LOC728618	E2FF-NFKB<br>E2FF-NFKB	613 - 741<br>846 - 728	(+)<br>(-)	
**GXP_907543** [GXP_907543] (1 - 601)<br>**TMEM132B**, GXL_380980, GeneID: 114795, Homo sapiens chr. 12<br>transmembrane protein 132B	E2FF-NFKB	449 - 576	(+)	

**GXP_642521** [GXP_642521] (1 - 616)<br>**BRCA2**, GXL_381035, GeneID: 675, Homo sapiens chr. 13<br>breast cancer 2, early onset	E2FF-NFKB	521 - 393	(-)	
**GXP_642569** [GXP_642569] (1 - 601)<br>**SLC25A15**, GXL_381044, GeneID: 10166, Homo sapiens chr. 13<br>solute carrier family 25 (mitochondrial carrier, ornithine transporter) member 15	E2FF-NFKB	423 - 312	(-)	
**GXP_642760** [GXP_642760] (1 - 739)<br>**TGDS**, GXL_381106, GeneID: 23483, Homo sapiens chr. 13<br>TDP-glucose 4,6-dehydratase	E2FF-NFKB	555 - 679	(+)	
**GXP_643005** [GXP_643005] (1 - 1377)<br>**CBLN3**, GXL_381156, GeneID: 643866, Homo sapiens chr. 14<br>cerebellin 3 precursor	E2FF-NFKB	470 - 344	(-)	
**GXP_908440** [GXP_908440] (1 - 1176)<br>**SFRS5**, GXL_381196, GeneID: 6430, Homo sapiens chr. 14<br>splicing factor, arginine/serine-rich 5	E2FF-NFKB	567 - 452	(-)	
**GXP_643338** [GXP_643338] (1 - 641)<br>**KIAA0317**, GXL_381203, GeneID: 9870, Homo sapiens chr. 14<br>KIAA0317	E2FF-NFKB	422 - 529	(+)	
**GXP_643496** [GXP_643496] (1 - 781)<br>**BDKRB2**, GXL_381219, GeneID: 624, Homo sapiens chr. 14<br>bradykinin receptor B2	E2FF-NFKB	252 - 377	(+)	
**GXP_643886** [GXP_643886] (1 - 672)<br>**RYR3**, GXL_381347, GeneID: 6263, Homo sapiens chr. 15<br>ryanodine receptor 3	E2FF-NFKB	315 - 197	(-)	
**GXP_644095** [GXP_644095] (1 - 784)<br>**USP50**, GXL_381379, GeneID: 373509, Homo sapiens chr. 15<br>ubiquitin specific peptidase 50	E2FF-NFKB	292 - 182	(-)	
**GXP_909791** [GXP_909791] (1 - 601)<br>**RHOT2**, GXL_381487, GeneID: 89941, Homo sapiens chr. 16<br>ras homolog gene family, member T2	E2FF-NFKB	250 - 370	(+)	
**GXP_644691** [GXP_644691] (1 - 737)<br>**CACNA1H**, GXL_381489, GeneID: 8912, Homo sapiens chr. 16<br>calcium channel, voltage-dependent, T type, alpha 1H subunit	E2FF-NFKB	292 - 406	(+)	
**GXP_909804** [GXP_909804] (1 - 627)<br>**CACNA1H**, GXL_381489, GeneID: 8912, Homo sapiens chr. 16<br>calcium channel, voltage-dependent, T type, alpha 1H subunit	E2FF-NFKB	443 - 314	(-)	
**GXP_645901** [GXP_645901] (1 - 711)<br>**SERPINF1**, GXL_381695, GeneID: 5176, Homo sapiens chr. 17<br>serpin peptidase inhibitor, clade F (alpha-2 antiplasmin, pigment epithelium derived factor), member 1	E2FF-NFKB	79 - 188	(+)	
	E2FF-NFKB	422 - 528	(+)	

**GXP_646047** [GXP_646047] (1 - 601)<br>**AMAC1L3,LOC731108**, GXL_381710, GeneID: 643664,731108, Homo sapiens chr. 17<br>acyl-malonyl condensing enzyme 1-like 3; similar to acyl-malonyl condensing enzyme 1				
**GXP_10641** [GXP_10641] (1 - 602)<br>**SLC16A5**, GXL_381900, GeneID: 9121, Homo sapiens chr. 17<br>solute carrier family 16, member 5 (monocarboxylic acid transporter 6)	E2FF-NFKB	252 - 368	(+)	
**GXP_10642** [GXP_10642] (1 - 602)<br>**SLC16A5**, GXL_381900, GeneID: 9121, Homo sapiens chr. 17<br>solute carrier family 16, member 5 (monocarboxylic acid transporter 6)	E2FF-NFKB	485 - 601	(+)	
**GXP_43506** [GXP_43506] (1 - 691)<br>**MRPL38,FBF1**, GXL_381904, GeneID: 64978,85302, Homo sapiens chr. 17<br>mitochondrial ribosomal protein L38; Fas (TNFRSF6) binding factor 1	E2FF-NFKB	383 - 262	(-)	
**GXP_912125** [GXP_912125] (1 - 601)<br>**ENOSF1**, GXL_381934, GeneID: 55556, Homo sapiens chr. 18<br>enolase superfamily member 1	E2FF-NFKB	427 - 537	(+)	
**GXP_647576** [GXP_647576] (1 - 601)<br>**KIAA1632**, GXL_381995, GeneID: 57724, Homo sapiens chr. 18<br>KIAA1632	E2FF-NFKB	139 - 16	(-)	
**GXP_648046** [GXP_648046] (1 - 863)<br>**MYO1F**, GXL_382060, GeneID: 4542, Homo sapiens chr. 19<br>myosin IF	E2FF-NFKB	146 - 24	(-)	
**GXP_648187** [GXP_648187] (1 - 604)<br>**RPS6,LOC729389,LOC730879**, GXL_382075, GeneID: 6194,729389,730879, Homo sapiens chr. 19<br>ribosomal protein S6; similar to 40S ribosomal protein S6; similar to 40S ribosomal protein S6	E2FF-NFKB	30 - 137	(+)	
**GXP_913088** [GXP_913088] (1 - 601)<br>**MEF2B,LOC729991,LOC731041**, GXL_382096, GeneID: 4207,729991,731041, Homo sapiens chr. 19<br>MADS box transcription enhancer factor 2, polypeptide B (myocyte enhancer factor 2B); similar to K11B4.2;similar to K11B4.2	E2FF-NFKB	165 - 36	(-)	
**GXP_649067** [GXP_649067] (1 - 601)<br>**SIGLECP16**, GXL_382191, GeneID: 400709, Homo sapiens chr. 19<br>sialic acid binding Ig-like lectin, pseudogene 16	E2FF-NFKB	345 - 221	(-)	
**GXP_919611** [GXP_919611] (1 - 601)<br>**RAB17**, GXL_382567, GeneID: 64284, Homo sapiens chr. 2<br>RAB17, member RAS oncogene family	E2FF-NFKB	217 - 111	(-)	
**GXP_652065** [GXP_652065] (1 - 601)<br>**SNORA71A**, GXL_382625, GeneID: 26777, Homo sapiens chr. 20<br>small nucleolar RNA, H/ACA box 71A	E2FF-NFKB	69 - 188	(+)	
**GXP_196216** [GXP_196216] (1 - 733)<br>**PRDM15,LOC728766**, GXL_382715, GeneID: 63977,728766, Homo sapiens chr. 21<br>PR domain containing 15; similar to PR domain containing 15	E2FF-NFKB	531 - 404	(-)	
	E2FF-NFKB	222 - 335	(+)	

**GXP_653280** [GXP_653280] (1 - 631)<br>**PACSIN2**, GXL_382849, GeneID: 11252, Homo sapiens chr. 22<br>protein kinase C and casein kinase substrate in neurons 2				
**GXP_653400** [GXP_653400] (1 - 829)<br>**SHANK3**, GXL_382869, GeneID: 85358, Homo sapiens chr. 22<br>SH3 and multiple ankyrin repeat domains 3	E2FF-NFKB	125 - 15	(-)	
**GXP_919969** [GXP_919969] (1 - 601)<br>**ULK4**, GXL_382915, GeneID: 54986, Homo sapiens chr. 3<br>unc-51-like kinase 4 (C. elegans)	E2FF-NFKB	90 - 197	(+)	
**GXP_656175** [GXP_656175] (1 - 602)<br>**hCG_1814936**, GXL_383284, GeneID: 646865, Homo sapiens chr. 4<br>hCG1814936	E2FF-NFKB	488 - 368	(-)	
**GXP_922474** [GXP_922474] (1 - 601)<br>**ZSWIM6**, GXL_383413, GeneID: 57688, Homo sapiens chr. 5<br>zinc finger, SWIM-type containing 6	E2FF-NFKB	309 - 194	(-)	
**GXP_48374** [GXP_48374] (1 - 632)<br>**ICK**, GXL_383665, GeneID: 22858, Homo sapiens chr. 6<br>intestinal cell (MAK-like) kinase	E2FF-NFKB	492 - 616	(+)	
**GXP_924452** [GXP_924452] (1 - 601)<br>**QKI**, GXL_383773, GeneID: 9444, Homo sapiens chr. 6<br>quaking homolog, KH domain RNA binding (mouse)	E2FF-NFKB	375 - 484	(+)	
**GXP_40482** [GXP_40482] (1 - 601)<br>**RABGEF1**, GXL_383938, GeneID: 27342, Homo sapiens chr. 7<br>RAB guanine nucleotide exchange factor (GEF) 1	E2FF-NFKB	138 - 257	(+)	
**GXP_660502** [GXP_660502] (1 - 601)<br>**KRBA1**, GXL_384087, GeneID: 84626, Homo sapiens chr. 7<br>KRAB-A domain containing 1	E2FF-NFKB	473 - 591	(+)	
**GXP_925575** [GXP_925575] (1 - 601)<br>**KRBA1**, GXL_384087, GeneID: 84626, Homo sapiens chr. 7<br>KRAB-A domain containing 1	E2FF-NFKB	474 - 584	(+)	
**GXP_926754** [GXP_926754] (1 - 601)<br>**ZNF658B,LOC653501**, GXL_384397, GeneID: 401509,653501, Homo sapiens chr. 9<br>zinc finger protein 658B; similar to zinc finger protein 658	E2FF-NFKB	405 - 283	(-)	
**GXP_663163** [GXP_663163] (1 - 602)<br>**ZNF674**, GXL_384637, GeneID: 641339, Homo sapiens chr. X<br>zinc finger protein 674	E2FF-NFKB	273 - 161	(-)	
**GXP_905712** [GXP_905712] (1 - 601)<br>**KRTAP5-8**, GXL_479676, GeneID: 57830, Homo sapiens chr. 11<br>keratin associated protein 5-8	E2FF-NFKB	165 - 279	(+)	
	E2FF-NFKB	95 - 214	(+)	

**GXP_905755** [GXP_905755] (1 - 601)<br>**STARD10**, GXL_479683, GeneID: 10809, Homo sapiens chr. 11<br>START domain containing 10				
**GXP_907632** [GXP_907632] (1 - 623)<br>**GJA3**, GXL_479996, GeneID: 2700, Homo sapiens chr. 13<br>gap junction protein, alpha 3, 46kDa	E2FF-NFKB	127 - 245	(+)	
**GXP_207398** [GXP_207398] (1 - 601)<br>**C14orf132**, GXL_480217, GeneID: 56967, Homo sapiens chr. 14<br>chromosome 14 open reading frame 132	E2FF-NFKB	207 - 323	(+)	
**GXP_908868** [GXP_908868] (1 - 601)<br>**SNORD116-15**, GXL_480327, GeneID: 100033427, Homo sapiens chr. 15<br>small nucleolar RNA, C/D box 116-15	E2FF-NFKB	75 - 186	(+)	
**GXP_274986** [GXP_274986] (1 - 602)<br>**THSD4**, GXL_480452, GeneID: 79875, Homo sapiens chr. 15<br>thrombospondin, type I, domain containing 4	E2FF-NFKB	138 - 18	(-)	
**GXP_909651** [GXP_909651] (1 - 601)<br>**ACAN**, GXL_480509, GeneID: 176, Homo sapiens chr. 15<br>aggrecan	E2FF-NFKB	417 - 302	(-)	
**GXP_91403** [GXP_91403] (1 - 927)<br>**USP31**, GXL_480594, GeneID: 57478, Homo sapiens chr. 16<br>ubiquitin specific peptidase 31	E2FF-NFKB	188 - 81	(-)	
**GXP_910109** [GXP_910109] (1 - 601)<br>**USP31**, GXL_480594, GeneID: 57478, Homo sapiens chr. 16<br>ubiquitin specific peptidase 31	E2FF-NFKB	540 - 433	(-)	
**GXP_910210** [GXP_910210] (1 - 601)<br>**CORO1A**, GXL_480616, GeneID: 11151, Homo sapiens chr. 16<br>coronin, actin binding protein, 1A	E2FF-NFKB	380 - 263	(-)	
**GXP_910508** [GXP_910508] (1 - 1134)<br>**EXOC3L**, GXL_480692, GeneID: 283849, Homo sapiens chr. 16<br>exocyst complex component 3-like	E2FF-NFKB	517 - 403	(-)	
**GXP_911112** [GXP_911112] (1 - 631)<br>**TRE17**, GXL_480796, GeneID: 440414, Homo sapiens chr. 17<br>TRE17 protein	E2FF-NFKB	402 - 531	(+)	
**GXP_484500** [GXP_484500] (1 - 631)<br>**GJA7**, GXL_480899, GeneID: 10052, Homo sapiens chr. 17<br>gap junction protein, alpha 7, 45kDa	E2FF-NFKB	337 - 454	(+)	
**GXP_4252** [GXP_4252] (1 - 897)<br>**INSR**, GXL_481104, GeneID: 3643, Homo sapiens chr. 19<br>insulin receptor	E2FF-NFKB	152 - 259	(+)	
	E2FF-NFKB	411 - 526	(+)	

**GXP_226484** [GXP_226484] (1 - 601)<br>**VRK3**, GXL_481213, GeneID: 51231, Homo sapiens chr. 19<br>vaccinia related kinase 3				
**GXP_913766** [GXP_913766] (1 - 601)<br>**LILRB2,LOC652626**, GXL_481233, GeneID: 10288,652626, Homo sapiens chr. 19<br>leukocyte immunoglobulin-like receptor, subfamily B (with TM and ITIM domains), member 2; similar to Leukocyte immunoglobulin-like receptor subfamily B member 2 precursor (Leukocyte immunoglobulin-like receptor 2) (LIR-2) (Immunoglobulin-like transcript 4) (ILT-4) (Monocyte/macrophage immunoglobulin-like receptor 10) (MIR-10) (CD85d ant...	E2FF-NFKB	454 - 325	(-)	
**GXP_913999** [GXP_913999] (1 - 601)<br>**TNFRSF25,PLEKHG5**, GXL_481280, GeneID: 8718,57449, Homo sapiens chr. 1<br>tumor necrosis factor receptor superfamily, member 25; pleckstrin homology domain containing, family G (with RhoGef domain) member 5	E2FF-NFKB	502 - 378	(-)	
**GXP_41964** [GXP_41964] (1 - 601)<br>**CASZ1**, GXL_481288, GeneID: 54897, Homo sapiens chr. 1<br>castor zinc finger 1	E2FF-NFKB	462 - 568	(+)	
**GXP_914436** [GXP_914436] (1 - 721)<br>**COL16A1**, GXL_481360, GeneID: 1307, Homo sapiens chr. 1<br>collagen, type XVI, alpha 1	E2FF-NFKB	209 - 96	(-)	
**GXP_636730** [GXP_636730] (1 - 958)<br>**PDZK1,LOC652793,LOC728939**, GXL_481519, GeneID: 5174,652793,728939, Homo sapiens chr. 1<br>PDZ domain containing 1; similar to PDZ domain containing 1; similar to PDZ domain containing 1	E2FF-NFKB	737 - 860	(+)	
**GXP_127856** [GXP_127856] (1 - 601)<br>**NSL1**, GXL_481679, GeneID: 25936, Homo sapiens chr. 1<br>NSL1, MIND kinetochore complex component, homolog (S. cerevisiae)	E2FF-NFKB	263 - 385	(+)	
**GXP_917037** [GXP_917037] (1 - 601)<br>**HAR1B**, GXL_481834, GeneID: 768097, Homo sapiens chr. 20<br>highly accelerated region 1B (non-protein-coding RNA)	E2FF-NFKB	545 - 420	(-)	
**GXP_158439** [GXP_158439] (1 - 631)<br>**MRPL19**, GXL_482100, GeneID: 9801, Homo sapiens chr. 2<br>mitochondrial ribosomal protein L19	E2FF-NFKB	370 - 253	(-)	
**GXP_919122** [GXP_919122] (1 - 601)<br>**HNRPA3**, GXL_482280, GeneID: 220988, Homo sapiens chr. 2<br>heterogeneous nuclear ribonucleoprotein A3	E2FF-NFKB	133 - 253	(+)	
**GXP_919582** [GXP_919582] (1 - 1361)<br>**SAG**, GXL_482364, GeneID: 6295, Homo sapiens chr. 2<br>S-antigen, retina and pineal gland (arrestin)	E2FF-NFKB	177 - 56	(-)	
**GXP_919630** [GXP_919630] (1 - 1042)<br>**ASB1**, GXL_482374, GeneID: 51665, Homo sapiens chr. 2<br>ankyrin repeat and SOCS box-containing 1	E2FF-NFKB	734 - 607	(-)	
**GXP_919901** [GXP_919901] (1 - 601)<br>**SUSD5**, GXL_482431, GeneID: 26032, Homo sapiens chr. 3	E2FF-NFKB	593 - 476	(-)	

sushi domain containing 5				
**GXP_922255** [GXP_922255] (1 - 601)<br>**FAM105B**, GXL_482934, GeneID: 90268, Homo sapiens chr. 5<br>family with sequence similarity 105, member B	E2FF-NFKB	50 - 162	(+)	
**GXP_922537** [GXP_922537] (1 - 680)<br>**DKFZP686M0199,LOC730394**, GXL_482990, GeneID: 653238,730394, Homo sapiens chr. 5<br>similar to TFIIH basal transcription factor complex p44 subunit (Basic transcription factor 2 44 kDa subunit) (BTF2-p44) (General transcription factor IIH polypeptide 2); region containing general transcription factor IIH, polypeptide 2, 44kDa, similar to TFIIH basal transcription factor complex p44 subunit (Basic transcription factor 2 44 kDa subunit) (BTF2-p44) (General transcription factor IIH polypeptide 2)	E2FF-NFKB	606 - 481	(-)	
**GXP_280374** [GXP_280374] (1 - 1108)<br>**MAML1**, GXL_483150, GeneID: 9794, Homo sapiens chr. 5<br>mastermind-like 1 (Drosophila)	E2FF-NFKB	510 - 620	(+)	
**GXP_924313** [GXP_924313] (1 - 633)<br>**HIVEP2**, GXL_483369, GeneID: 3097, Homo sapiens chr. 6<br>human immunodeficiency virus type I enhancer binding protein 2	E2FF-NFKB	179 - 290	(+)	
**GXP_924653** [GXP_924653] (1 - 601)<br>**MGC87042**, GXL_483453, GeneID: 256227, Homo sapiens chr. 7<br>similar to Six transmembrane epithelial antigen of prostate	E2FF-NFKB	286 - 392	(+)	
	E2FF-NFKB	485 - 378	(-)	
**GXP_925460** [GXP_925460] (1 - 1035)<br>**KIAA1549**, GXL_483651, GeneID: 57670, Homo sapiens chr. 7<br>KIAA1549 protein	E2FF-NFKB	227 - 104	(-)	
**GXP_60208** [GXP_60208] (1 - 601)<br>**DKFZp762P2111,ZNF783,LOC791120**, GXL_483675, GeneID: 55537,155060,791120, Homo sapiens chr. 7<br>hypothetical protein DKFZp762P2111; zinc finger protein 783;hypothetical LOC791120	E2FF-NFKB	546 - 429	(-)	
**GXP_660548** [GXP_660548] (1 - 601)<br>**CENTG3**, GXL_483681, GeneID: 116988, Homo sapiens chr. 7<br>centaurin, gamma 3	E2FF-NFKB	461 - 355	(-)	
**GXP_925847** [GXP_925847] (1 - 977)<br>**TRIM35**, GXL_483751, GeneID: 23087, Homo sapiens chr. 8<br>tripartite motif-containing 35	E2FF-NFKB	672 - 565	(-)	
**GXP_51394** [GXP_51394] (1 - 768)<br>**ADAM32,TMDCII**, GXL_483765, GeneID: 203102,255926, Homo sapiens chr. 8<br>ADAM metallopeptidase domain 32;tMDC II	E2FF-NFKB	509 - 627	(+)	
**GXP_926211** [GXP_926211] (1 - 629)<br>**C8orf38**, GXL_483817, GeneID: 137682, Homo sapiens chr. 8<br>chromosome 8 open reading frame 38	E2FF-NFKB	438 - 323	(-)	
**GXP_926616** [GXP_926616] (1 - 614)<br>**ELAVL2**, GXL_483899, GeneID: 1993, Homo sapiens chr. 9<br>ELAV (embryonic lethal, abnormal vision, Drosophila)-like 2 (Hu antigen B)	E2FF-NFKB	264 - 376	(+)	

**GXP_926656** [GXP_926656] (1 - 602)<br>**ANXA2P2**, GXL_483906, GeneID: 304, Homo sapiens chr. 9<br>annexin A2 pseudogene 2	E2FF-NFKB	428 - 550	(+)	
**GXP_926713** [GXP_926713] (1 - 601)<br>**PC-3**, GXL_483914, GeneID: 692094, Homo sapiens chr. 9<br>secreted microprotein PC-3	E2FF-NFKB	410 - 293	(-)	
**GXP_927531** [GXP_927531] (1 - 601)<br>**FAM9C**, GXL_484119, GeneID: 171484, Homo sapiens chr. X<br>family with sequence similarity 9, member C	E2FF-NFKB	196 - 316	(+)	

A total of 1980 matches was found in 1901 sequences.

Sequences searched: 56193 (37306006 bp).

Evaluation of results

Model: E2FF-NFKB

Number of input genes: 1894

GO category "biological_process"

Number of genes annotated in GO: 1324
Number of significant GO groups found: 44

GO group	p-value	# genes (observed)	# genes (expected)	list of genes	GeneIDs
nervous system development	2.45211e-05	97	64.47	CACNA1A, THBS1, SH3GL2, EFNB1, PCDHAC2, PCDHAC1, PCDHA11, PCDHA10, PCDHA8, PCDHA7, PCDHA5, PCDHA4, PCDHA3, PCDHA2, PCDHA1, ChGn, BAG1, APBA1, GNAQ, LIMK1, CXCL1, ETV4, ITM2B, NRG1, TIMP2, KIF5C, EPHB2, CD9, MSI1, SEMA4C, AZU1, PLXNB2, ADORA2A, DOC2A, PTPRZ1, MYT1, SERPINE2, CHRDL1, GFRA1, BCL11B, LHX1, SPOCK1, TP53BP2, EPHB1, NRP2, SDCBP2, FEZ1, STMN3, HTRA2, PCSK2, SNAI1, EMX2, HDAC7A, BDNF, PAX6, ERBB3, SEMA6C, SIX1, TITF1, PPARD, DSCAML1, APP, SOD1, MSX1, METRN, LY6H, NRXN3, CYP46A1, ROBO3, PBX4, NTNG2, LMX1B, LHX2, NAPA, PAFAH1B3, SEMA7A, RGMA, NINJ1, EPM2A, FYN, BTD, NTN4, STMN1, ARX, UNC5C, ARNT2, TLX3, NCKAP1, LIF, PMP22, DLG4, TFAP2B, PPP2R5D, APLP1, NTRK2, SERPINF1, QKI	773, 7057, 6456, 1947, 56134, 56135, 56138, 56139, 56140, 56141, 56143, 56144, 56145, 56146, 56147, 55790, 573, 320, 2776, 3984, 2919, 2118, 9445, 3084, 7077, 3800, 2048, 928, 4440, 54910, 566, 23654, 135, 8448, 5803, 4661, 5270, 91851, 2674, 64919, 3975, 6695, 7159, 2047, 8828, 27111, 9638, 50861, 27429, 5126, 6615, 2018, 51564, 627, 5080, 2065, 10500, 6495, 7080, 5467, 57453, 351, 6647, 4487, 79006, 4062, 9369, 10858, 64221, 80714, 84628, 4010, 9355, 8775, 5050, 8482, 56963, 4814, 7957, 2534, 686, 59277, 3925, 170302, 8633, 9915, 30012, 10787, 3976, 5376, 1742, 7021, 5528, 333, 4915, 5176, 9444
cellular process	0.000106488	1236	1199.56	MKNK2, RFX2, LSM7, LONP1, FARSA, ZBTB7A, CDC37, TLE2,	2872, 5990, 51690, 9361, 2193, 51341, 11140,

				MGC19604, MAN2B1, ZNF564, ZNF709, CACNA1A, MATK, ZNF442, FBXW9, RANBP3, SHC2, LOC732442, COL5A3, TIMM13, MLLT1, POLRMT, REXO1, GCHFR, AP4E1, RAB8B, THBS1, D4ST1, MAPK6, ERBB2IP, XRCC4, THBS4, F2RL1, CACNA1G, WNT9B, EPX, CDK3, LLGL2, ICT1, PITPNC1, SOX9, KCTD2, SLC25A43, ZIC3, MTMR1, VBP1, RPL10, SLC25A14, BIRC4, DUSP9, GNA12, NDUFA4, CDCA7L, INTS1, FLJ21767, SNX8, HOXA10, TMED4, SETX, SH3GL2, RALGDS, CIZ1, AGPAT2, SMARCA2, PTGES2, JMJD2C, SH2D3C, DMRT1, CCBL1, CDC37L1, C9orf127, LCN8, OBP2B, HACL1, KIF9, IL5RA, AMT, SLC26A6, NGLY1, TSP50, TESSP5, LOC729280, LOC729752, LOC729756, ATP6V1G1, VPS13A, FOXE1, CCIN, PGM5, OSTF1, HDAC6, MID1IP1, TSPAN7, UBE1, RP2, SLC16A2, KIF4A, ARR3, EFNB1, ARF1, C1orf69, COG2, EPHX1, KIAA1804, GALNT10, FOXI1, TGFBI, ABLIM3, PCDHA9, PCDHAC2, PCDHAC1, PCDHA13, PCDHA12, PCDHA11, PCDHA10, PCDHA8, PCDHA7, PCDHA5, PCDHA4, PCDHA3, PCDHA2, PCDHA1, SGCD, C5orf14, EIF4EBP3, SFRP1, ChGn, LONRF1, ZNF395, FBXO16, NKX3-1, PSD3, STC1, LYPLA1, TRPM3, BAG1, C9orf95, RP11-138L21.1, CNTNAP3, LOC727745, KIAA1815, GBA2, TLN1, RASEF, APBA1, AQP7, LOC730908, GNAQ, BCL2L12, ZNF324, CACNG6, ZNF324B, FTL, ZIK1, RUVBL2, PRMT1, TSEN34, U2AF2, UPP1, RCP9, LIMK1, YKT6, DBNL, CCM2, RAMP3, SPATA18, SPRY1, HSPA4L, PCDH10, EXOC1, LPHN3, CXCL6, CXCL1, DCK, MAD2L2, E2F2, USP48, DDOST, TNFRSF14, RAP1GAP, DHRS3, KIF17, EVX1, ANLN, AHR, GARS, ITGB8, SNX10, ZDHHC4, JTV1, RECQL5, CHAD, ABCA8, SDK2, LYK5, SFRS1, HOXB4, SC65, HCRT, PHF12, GJC1, ACACA, HAP1, ETV4, PLCD3, SERPINH1, PACS1, SF3B2, CAPN5, NDUFV1, PRSS23, DGAT2, SIPA1, DIXDC1, ELMOD1, CCND1, AIP, MRPS18A, CYP39A1, ASCC3, TFEB, MRPL14, B3GAT2, TRFP, ATG5, CD164, PREP, GTPBP2, GSTA1, CGA, BACH2, RAP2A, AKAP11, SUGT1, ITM2B, B3GAT3, SHANK2, LRP4, CDCA5, PPP1CA, MRGPRF, SLC15A3, SLC29A2, MAP4K2, PPM1B, MTA3, ATP6V1C2, RRM2, MSH6, GRHL1, KCNF1, FLJ21839, GATA4, ZNF251, EPHX2, PLEC1, SLC25A37, NRG1, PTK2B, BMP1, FBXO25, CHMP7, HOOK3, SCARA3, FBXL6, ADAMDEC1, EIF4EBP1, RPL8, TXNDC5, DEK, TBC1D7, SERPINB9, KIF13A, NEDD9, PBX2, GMDS, SLC17A2, GNL1, RXRB, SSR1, TFAP2A, DOK3, GFPT2, RAB24, FLJ31951, SNCB, MGAT1, RASGEF1C, OSMR, SUB1, ARRB2, AATK, LOC651771, SIRT7, RNF167, USP43, PYCR1, LGALS3BP, MIS12, ST6GALNAC2, GARNL4, JMJD6, TIMP2, ALG6, SCP2, SLC5A9, CDCA8, PTPRF, AK3L1, RLF, YBX1, FOXD3, RALBP1, HMG2L1, TRIOBP, CBY1, XRCC6, FBXO7, TSPO, PARVG, ACO2, TOMM22, HMOX1, BIK, ARHGEF5, CUL1, INSIG1, UBE3C, ZBTB7B, KIRREL, TARS2, DUSP27, MTX1, EFNA3, CA14, MRPS21, ATP1B1, ATP8B2,	7089, 112812, 4125, 163050, 163051, 773, 4145, 79973, 84261, 8498, 25759, 732442, 50509, 26517, 4298, 5442, 57455, 2644, 23431, 51762, 7057, 113189, 5597, 55914, 7518, 7060, 2150, 8913, 7484, 8288, 1018, 3993, 3396, 26207, 6662, 23510, 203427, 7547, 8776, 7411, 6134, 9016, 331, 1852, 2768, 4697, 55536, 26173, 401331, 29886, 3206, 222068, 23064, 6456, 5900, 25792, 10555, 6595, 80142, 23081, 10044, 1761, 883, 55664, 51754, 138307, 29989, 26061, 64147, 3568, 275, 65010, 55768, 29122, 377047, 729280, 729752, 729756, 9550, 23230, 2304, 881, 5239, 26578, 10013, 58526, 7102, 7317, 6102, 6567, 24137, 407, 1947, 375, 200205, 22796, 2052, 84451, 55568, 2299, 7045, 22885, 9752, 56134, 56135, 56136, 56137, 56138, 56139, 56140, 56141, 56143, 56144, 56145, 56146, 56147, 6444, 79770, 8637, 6422, 55790, 91694, 55893, 157574, 4824, 23362, 6781, 10434, 80036, 573, 54981, 389722, 79937, 727745, 79956, 57704, 7094, 158158, 320, 364, 730908, 2776, 83596, 25799, 59285, 388569, 2512, 284307, 10856, 3276, 79042, 11338, 7378, 27297, 3984, 10652, 28988, 83605, 10268, 132671, 10252, 22824, 57575, 55763, 23284, 6372, 2919, 1633, 10459, 1870, 84196, 1650, 8764, 5909, 9249, 57576, 2128, 54443, 196, 2617, 3696, 29887, 55146, 7965, 9400, 1101, 10351, 54549, 92335, 6426, 3214, 10609, 3060, 57649, 125111, 31, 9001, 2118, 113026, 871, 55690, 10992, 726, 4723, 11098, 84649, 6494, 85458, 55531, 595, 9049, 55168, 51302, 10973, 7942, 64928, 135152, 9477, 9474, 8763, 5550, 54676, 2938, 1081, 60468, 5911, 11215, 10910, 9445, 26229, 22941, 4038, 113130, 5499, 219928, 51296, 3177, 5871, 5495, 57504, 245973, 6241, 2956, 29841, 3754, 60509, 2626, 90987, 2053, 5339, 51312, 3084, 2185, 649, 26260, 91782, 84376, 51435, 26233, 27299, 1978, 6132, 81567, 7913, 51256, 5272, 63971, 4739, 5089, 2762, 10246, 2794, 6257, 6745, 7020, 79930, 9945, 53917, 153830, 6620, 4245, 255426, 9180, 10923, 409, 9625, 651771, 51547, 26001, 124739, 5831, 3959, 79003, 10610, 23108, 23210, 7077, 29929, 6342, 200010, 55143, 5792, 205,

				CREB3L4, GYPC, DNAJC10, TANK, ZAK, SSB, DPP10, GAD1,	6018, 4904, 27022, 10928, 10042, 11078, 25776,
				MYO3B, CDCA7, PPIG, INPP1, PMS1, KIF5C, PER3, RCC1, SESN2,	2547, 25793, 706, 64098, 50, 56993, 3162, 638,
				RPL11, EPB41, PDPN, H6PD, ATPIF1, DNAJC16, STX12, CAMTA1,	7984, 8454, 3638, 9690, 51043, 55243, 80222,
				EPHA8, EPHB2, TSSK3, SERINC2, OPRD1, ELA2A, AQP5, CD9,	92235, 4580, 1944, 23632, 54460, 481, 57198,
				SURB7, EMG1, CAMKK2, DIABLO, SLC41A2, GALNT9, CLIP1, TBX5,	148327, 2995, 54431, 10010, 51776, 6741, 57628,
				HPD, SSH1, RAB35, GLT8D2, LHX5, EBAG9, CHD7, SULF1, SGK3,	2571, 140469, 83879, 9360, 3628, 5378, 3800,
				TRIB1, SOX17, KCNS2, CHMP4C, MRPL15, ZFP161, CYB5A, PTPN2,	8863, 1104, 83667, 6135, 2035, 10630, 9563,
				DSC2, TCF4, L3MBTL4, FBXO15, ST8SIA5, ESCO1, DUSP11,	93974, 23341, 23673, 23261, 2046, 2048, 81629,
				SEMA4C, FBXO41, SUCLG1, HMHA1, EVI5L, PTBP1, ZNF497, AZU1,	347735, 4985, 63036, 362, 928, 9412, 10436,
				SF3A2, HCN2, MADCAM1, SHD, ITGB1BP3, CHAF1A, PLXNB2,	10645, 56616, 84102, 50614, 6249, 6910, 3242,
				ADORA2A, SNRPD3, MAPK12, ITSN2, OTOF, ROCK2, GPR113,	54434, 11021, 83468, 64211, 9166, 55636, 23213,
				HS1BP3, RBJ, MPV17, PPM1G, THUMPD2, RNPS1, TMEM8, UMOD,	23678, 10221, 64321, 3788, 92421, 29088, 7541,
				ZKSCAN2, CLN3, GTF3C1, ADCY9, N-PAC, GGA2, DOC2A, KCTD13,	1528, 5771, 1824, 6925, 91133, 201456, 29906,
				CDIPT, GRIN2A, LOC606495, SSX2IP, RHOC, DDAH1, SYT6,	114799, 8446, 54910, 150726, 8802, 23526,
				C1orf41, SYDE2, CPA5, ZNF277P, FLJ25778, SLC26A4, CAV1,	115704, 5725, 162968, 566, 8175, 610, 8174,
				ARPC1B, PTPRZ1, DLD, STX16, OSBPL2, ARFGAP1, GNAS, OGFR,	56961, 27231, 10036, 23654, 135, 6634, 6300,
				MYT1, HSPD1, AAMP, TNS1, RPS7, PER2, HDLBP, ILKAP, ZNF142,	50618, 9381, 9475, 165082, 64342, 51277, 4358,
				SERPINE2, GTF3C3, RNF25, CHM, IDH3G, F8, UPF3B, SYTL4,	5496, 80745, 10921, 58986, 7369, 342357, 1201,
				TMSB4X, UBL4A, PRPS2, G6PD, CUL4B, CHRDL1, SLC10A3, GLA,	2975, 115, 84656, 23062, 8448, 253980, 10423,
				CLCN4, IL17C, SLC9A5, NUTF2, CTBP2, MRC1, SEC61A2, THNSL1,	2903, 606495, 117178, 389, 23576, 148281,
				DHTKD1, BNIP3, GTPBP4, PFKP, VIM, CAMK1D, C10orf125,	51668, 84144, 93979, 11179, 254048, 5172, 857,
				HSPA14, GFRA1, MCM7, CYP3A5, STYXL1, CLDN3, MTERF,	10095, 5803, 1738, 8675, 9885, 55738, 2778,
				FBXL19, SLC6A2, CYLD, PHKG2, LOC650556, TRIP11, PPP1R13B,	11054, 4661, 3329, 14, 7145, 6201, 8864, 3069,
				BCL11B, ALDH6A1, ACYP1, ABCD4, ZPBP2, SUZ12, FMNL1,	80895, 7701, 5270, 9330, 64320, 1121, 3421,
				RASL10B, MAPK7, DHX8, GRB7, AP2B1, NMT1, NEK8, RAMP2,	2157, 65109, 94121, 7114, 8266, 5634, 2539,
				RARA, CCL2, LHX1, SIL1, TAF7, FBN2, MCC, PDGFRB, HINT1,	8450, 91851, 8273, 2717, 1183, 27189, 6553,
				FBXL17, CDO1, GLRX, IRF1, SPOCK1, ARTS-1, P4HA2, PJA2, OAS2,	10204, 1488, 4360, 55176, 79896, 55526, 664,
				C12orf65, SETD1B, HYPE, ALDH2, SETD8, HCFC2, DAO, ABCB10,	23560, 5214, 7431, 57118, 282969, 51182, 2674,
				HNRPU, LIN9, SLC30A10, LOC728528, VWA1, RGS7, PARP1,	4176, 1577, 51657, 1365, 7978, 54620, 6530,
				TP53BP2, CYC1, ZNF696, SPSB4, PARP14, SEC22A, MCM2, EPHB1,	1540, 5261, 650556, 9321, 23368, 64919, 4329,
				CHST13, UMPS, COL4A3, ATG16L1, BCS1L, NRP2, STK36, CCNYL1,	97, 5826, 124626, 23512, 752, 91608, 5598, 1659,
				SCLY, UBE2F, ACCN4, INPP5D, ATF3, XPR1, SMG7, KCTD3,	2886, 163, 4836, 284086, 10266, 5914, 6347,
				KLHL20, CTSE, SOAT1, PFKFB2, SDCBP2, ADAM33, RASSF2,	3975, 64374, 6879, 2201, 4163, 5159, 3094,
				APBA2BP, NANP, E2F1, MYOD1, PPFIBP2, SYT9, DNHD1, PARVA,	64839, 1036, 2745, 3659, 6695, 51752, 8974,
				IGSF9B, SLC6A5, P53AIP1, FEZ1, SLC17A6, AMPD3, PTDSS2,	9867, 4939, 91574, 23067, 11153, 217, 387893,
				PPP2R2C, GAK, CENTD1, HOP, GRSF1, COX18, G3BP2, CXCL10,	29915, 1610, 23456, 3192, 286826, 55532,
				CXCL9, CXCL3, CXCL5, PLTP, STMN3, NEURL2, ZNF217, JPH2,	728528, 64856, 6000, 142, 7159, 1537, 79943,
				MATN4, CTCFL, CDC25B, CDS2, GNRH2, RIMS4, SLC35C2, PTPRA,	92369, 54625, 26984, 4171, 2047, 166012, 7372,
				VPS16, GM632, USP39, DOK1, NOTO, PCBP1, HTRA2, SNRPB2,	1285, 55054, 617, 8828, 27148, 151195, 51540,
				RIN2, NCOA3, ASXL1, CD40, LBP, PCSK2, SNAI1, SLC24A3,	140739, 55515, 3635, 467, 9213, 9887, 51133,
				MPHOSPH6, PLLP, SLC12A4, LRRC29, MAPK3, PYDC1, ADAMTS18,	27252, 1510, 6646, 5208, 27111, 80332, 9770,
				PRSS36, ABCC11, FHOD1, CASP7, SFXN2, SLK, PAOX, PDCD4,	63941, 140838, 1869, 4654, 8495, 143425,
				GPR123, EMX2, GPR26, ADRB1, PKP2, HDAC7A, PTHLH, PHB2,	144132, 55742, 22997, 9152, 63970, 9638, 57084,
				FBXL14, GRIN2B, CHD4, SPSB2, BDNF, CD59, TMEM9B, NUP98,	272, 81490, 5522, 2580, 116984, 84525, 2926,

				TRIM5, UEVLD, PAX6, ABTB2, CYP2R1, IGFBP6, ERBB3, CNOT2,	285521, 9908, 3627, 4283, 2921, 6374, 5360,
				TARBP2, SLC17A8, SNRPF, TENC1, SYT1, ESPL1, ZDHHC17, LRP1,	50861, 140825, 7764, 57158, 8785, 140690, 994,
				RASSF3, SLC26A9, RIPK5, RAB7L1, RC3H1, GPR161, LPGAT1,	8760, 2797, 140730, 51006, 5786, 64601, 57473,
				KCNT2, RFWD2, DEDD, GLUL, TMEM9, F5, PIGM, FLJ16478, APH1A,	10713, 1796, 344022, 5093, 27429, 6629, 54453,
				PYGO2, GBA, CSDE1, INSRR, SLC39A1, DPM3, SEMA6C, PBXIP1,	8202, 171023, 958, 3929, 5126, 6615, 57419,
				CD5L, SLC8A3, DPF3, CHMP4A, MYH7, SIX1, NOVA1, ERO1L, TITF1,	10200, 51090, 6560, 26231, 5595, 260434,
				STXBP6, NFKBIA, PYGL, PPARD, CYP21A2, SLC39A7, HMGA1,	170692, 146547, 85320, 29109, 840, 118980,
				PFDN6, CD83, ITPR3, HSD17B8, SLC25A30, PCDH9, DIS3, SOHLH2,	9748, 196743, 27250, 84435, 2018, 2849, 153,
				LCP1, NEK3, UBL3, TPT1, PARP4, CCNU, CDC20B, AYTL2, FGF10,	5318, 51564, 5744, 11331, 144699, 2904, 1108,
				CTNND2, SERINC5, RPS23, PDE4D, ANKH, CDH12, HTR1A, ALG8,	84727, 627, 966, 56674, 4928, 85363, 55293,
				ZNF259, TIMM8B, PHOX2A, MAML2, TREH, SLC37A4, CENTD2,	5080, 25841, 120227, 3489, 2065, 4848, 6895,
				ARRB1, DSCAML1, GUCY1A2, CCT8, APP, CLIC6, FTCD, PIGP,	246213, 6636, 23371, 6857, 9700, 23390, 4035,
				IFNGR2, SOD1, CBS, U2AF1, ZNF295, TFF1, D4S234E, ANAPC4,	283349, 115019, 25778, 8934, 149041, 23432,
				AGA, ASB5, RBPJ, SLC34A2, KLF3, MSX1, MGRN1, UBE2I, CYBA,	9926, 343450, 64326, 9191, 2752, 252839, 2153,
				MMP25, CA5A, SLC7A5, UBN1, TAF1C, TRAF7, CCNF, PCOLN3,	93183, 440695, 51107, 90780, 2629, 7812, 3645,
				CLDN9, PRSS21, PRDM7, BAIAP3, STUB1, DNASE1, METRN,	27173, 54344, 10500, 57326, 922, 6547, 8110,
				ZNF205, HBA2, ARL6, RBM6, SLC25A38, MYRIP, VIPR1, PRKCD,	29082, 4625, 6495, 4857, 30001, 7080, 29091,
				KCTD6, RNF123, SPCS1, ST3GAL6, PPM1M, APPL1, CCBP2, APEH,	4792, 5836, 5467, 1589, 7922, 3159, 10471, 9308,
				DAG1, QSER1, MADD, MARK2, INCENP, LOC649034, PRDX5,	3710, 7923, 253512, 5101, 22894, 54937, 3936,
				CREB3L1, SLC35C1, KCNK4, STX3, CAPN1, RASSF1, SFMBT1,	4752, 5412, 7178, 143, 10309, 166979, 79888,
				CBLB, GLT8D1, DNASE1L3, ARHGEF3, CYP11B1, LRP12,	2255, 1501, 256987, 6228, 5144, 56172, 1010,
				TNFRSF11B, RRM2B, TPD52, KLF10, SLA, KIF26A, ADSSL1,	3350, 79053, 8882, 26521, 401, 84441, 11181,
				PAPOLA, FCF1, EIF5, TRAF3, NRXN3, JDP2, TNFAIP2, CYP46A1,	2542, 116985, 408, 57453, 2977, 10694, 351,
				ZNF410, ROBO3, MIZF, FOXRED1, BCDO2, STT3A, SDHD, CHEK1,	54102, 10841, 51227, 3460, 6647, 875, 7307,
				TBRG1, FLI1, GALNT7, SPCS3, SLC26A11, LOC652834, C1QTNF1,	49854, 7031, 27065, 29945, 175, 140458, 3516,
				CBX2, CD99, ZFY, MX1, ABCG1, NDUFV3, TMEM1, COL6A2,	10568, 51274, 4487, 23295, 7329, 1535, 64386,
				ADARB1, D2HGDH, BOK, F10, TXNDC6, KLF15, DBR1, MGLL, RPN1,	763, 8140, 29855, 9013, 84231, 899, 5119, 9080,
				RARRES1, ANAPC13, TNIK, CCNL1, GNB4, PBEF1, CNOT4, COG5,	10942, 11105, 8938, 10273, 1773, 79006, 7755,
				SRPK2, ZNF467, HNRPL, ZNF585B, ZNF585A, PBX4, CALR3,	3040, 84100, 10180, 54977, 25924, 7433, 5580,
				ZNF780A, ZNF573, GMIP, ZNF599, PEPD, SLC1A6, FKBP8, BRD4,	200845, 63891, 28972, 10402, 132160, 26060,
				NTNG2, PRRX2, FPGS, PTGS1, LMX1B, TLR4, LHX2, CDK9, ZNF615,	1238, 327, 1605, 79832, 8567, 2011, 3619,
				NUP62, IL4I1, CNFN, STRN4, NAPA, KCNN4, SIX5, LHB, CA11,	649034, 25824, 90993, 55343, 50801, 6809, 823,
				ZNF228, KLK7, UACA, SELS, AP3S2, SEMA7A, HERC1, HCN4,	11186, 51460, 868, 55830, 1776, 50650, 1584,
				RGMA, ADAMTS17, AP3B2, VPS33B, SNRPA1, RHCG, ANGPTL2,	29967, 4982, 50484, 7163, 7071, 6503, 26153,
				CDC14B, TTLL11, GKAP1, PTPN3, FANCC, GRIN3A, NINJ1, KLF4,	122622, 10914, 51077, 1983, 7187, 9369, 122953,
				ZNF782, FBP1, ZBTB2, C6orf170, SHPRH, SOD2, DDO, IRF4,	7127, 10858, 57862, 64221, 25988, 55572, 83875,
				EPM2A, FYN, PLAGL1, MTRF1L, PPIL4, RIPK1, FOXF2, MAP7,	3703, 6392, 1111, 84897, 2313, 51809, 60559,
				RNASET2, ROS1, MCF2L2, ARPC4, TTLL3, BTD, MUC4, HDAC11,	284129, 652834, 114897, 84733, 4267, 7544,
				VHL, LMCD1, UBE2E2, EAF1, AAAS, BBS10, KRT1, OSBPL8, NTN4,	4599, 9619, 4731, 7109, 1292, 104, 728294, 666,
				FMNL3, CSRP2, WNT10B, MAP3K12, SLC16A12, PANK1, ALDH18A1,	2159, 347736, 28999, 51163, 11343, 6184, 5918,
				PPP3CB, SLC25A28, ARL3, NOC3L, PIK3R3, MYOM3, STMN1,	25847, 23043, 57018, 59345, 10135, 4850, 10466,
				PTP4A2, WDR57, RSPO1, DNAJC8, GRIK3, SCMH1, SLC9A1, TAL1,	6733, 168544, 3191, 92285, 199704, 80714,
				BMP8B, SH3KBP1, BCOR, RBBP7, ARX, PITX2, UBE2D3, PDE5A,	125972, 284323, 126231, 51291, 148103, 5184,
				CCNI, UNC5C, MFSD8, MAD2L1, DMXL2, SRP14, ATP8B4, RAB27A,	6511, 23770, 23476, 84628, 51450, 2356, 5742,

				LYSMD2, BCL2L10, AGPAT7, TP53BP1, MKX, TUBAL3, HNRPF, ZNF33B, CTNNA3, MAN1A2, HIPK1, DDX20, LPHN2, PKN2, AHCYL1, STXBP3, CDC7, RTCD1, HS2ST1, TXNIP, MAGI3, MORF4L1, NR2E3, PDE8A, IREB2, C15orf27, RPLP1, CLK3, ZNF774, ARNT2, ST6GAL1, EPHB3, TIPARP, ZNF639, MME, IL1RAP, HTR3E, SENP5, PSMD2, EIF4A2, HSF2, TPD52L1, L3MBTL3, STX11, TNFAIP3, MRPL18, GJA1, UNC5A, CANX, BNIP1, TLX3, FGFR4, DPP4, TTN, CHN1, WIPF1, NCKAP1, SLC40A1, FUT8, LTB4R, MGAT2, AP4S1, NFATC4, PRKCH, PPM1A, NCOA4, FAS, VCL, ADAMTS14, BMPR1A, PLCE1, ZNF33A, GPR120, SLC18A3, CLDN5, THAP7, PHF5A, FOXRED2, DMC1, TPST2, LIF, DDX17, L3MBTL2, EIF3S7, GSCL, UNC84B, SLC25A1, CRK, TRPV1, MYBBP1A, PMP22, ZZEF1, SREBF1, SMG6, GPS2, KRBA2, DLG4, CLDN7, ATPAF2, TFAP2B, DNAH8, OPN5, SMAP1, POLR1C, FBXO9, HSP90AB1, PPP2R5D, KCNQ5, BCKDHB, FUT9, BAI3, CAPNS1, MRPS12, PLEKHG2, APLP1, PVR, CLPTM1, CYP2F1, FXYD7, GAPDHS, BTBD14B, SMARCA4, CRTC1, DNM2, SLC44A2, MRPL4, ZNF257, ZNF101, CYP4F12, ZNF763, SLC25A42, CARM1, CNN1, MORG1, PLCH2, C1orf31, PARG, RDX, PLA2G4D, ARHGDIG, ACOX1, MC2R, RTKN, PPARA, NTRK2, ZNF711, MIB2, SLC18A2, NKX1-2, DAK, PVRL1, COL2A1, BRCA2, SLC25A15, TGDS, SFRS5, KIAA0317, BDKRB2, RYR3, USP50, RHOT2, CACNA1H, SERPINF1, SLC16A5, RPS6, MEF2B, RAB17, PRDM15, PACSIN2, SHANK3, ULK4, ICK, QKI, RABGEF1, KRBA1, ZNF674, GJA3, ACAN, USP31, CORO1A, GJA7, INSR, VRK3, LILRB2, TNFRSF25, PLEKHG5, CASZ1, COL16A1, PDZK1, NSL1, MRPL19, HNRPA3, SAG, ASB1, SUSD5, DKFZP686M0199, LOC730394, MAML1, HIVEP2, MGC87042, KIAA1549, ZNF783, CENTG3, TRIM35, ADAM32, TMDCII, ELAVL2	4010, 7099, 9355, 1025, 284370, 23636, 259307, 84518, 29888, 8775, 3783, 147912, 3972, 770, 7771, 5650, 55075, 55829, 10239, 8482, 8925, 10021, 56963, 170691, 8120, 26276, 6627, 51458, 23452, 8555, 158135, 80318, 5774, 2176, 116443, 4814, 9314, 158431, 2203, 57621, 221322, 257218, 6648, 8528, 3662, 7957, 2534, 5325, 54516, 85313, 8737, 2295, 9053, 8635, 6098, 23101, 10093, 26140, 686, 4585, 79885, 7428, 29995, 7325, 85403, 8086, 79738, 3848, 114882, 59277, 91010, 1466, 7480, 7786, 387700, 53354, 5832, 5532, 81894, 403, 64318, 8503, 127294, 3925, 8073, 9410, 284654, 22826, 2899, 22955, 6548, 6886, 656, 30011, 54880, 5931, 170302, 5308, 7323, 8654, 10983, 8633, 256471, 4085, 23312, 6727, 79895, 5873, 256586, 10017, 254531, 7158, 283078, 79861, 3185, 7582, 29119, 10905, 204851, 11218, 23266, 5586, 10768, 6814, 8317, 8634, 9653, 10628, 260425, 10933, 10002, 5151, 3658, 123591, 6176, 1198, 342132, 9915, 6480, 2049, 25976, 51193, 4311, 3556, 285242, 205564, 5708, 1974, 3298, 7164, 84456, 8676, 7128, 29074, 2697, 90249, 821, 662, 30012, 2264, 1803, 7273, 1123, 7456, 10787, 30061, 2530, 1241, 4247, 11154, 4776, 5583, 5494, 8031, 355, 7414, 140766, 657, 51196, 7581, 338557, 6572, 7122, 80764, 84844, 80020, 11144, 8459, 3976, 10521, 83746, 8664, 2928, 25777, 6576, 1398, 7442, 10514, 5376, 23140, 6720, 23293, 2874, 124751, 1742, 1366, 91647, 7021, 1769, 221391, 60682, 9533, 26268, 3326, 5528, 56479, 594, 10690, 577, 826, 6183, 64857, 333, 5817, 1209, 1572, 53822, 26330, 112939, 6597, 23373, 1785, 57153, 51073, 113835, 94039, 66002, 284390, 284439, 10498, 1264, 84292, 9651, 388753, 8505, 5962, 283748, 398, 51, 4158, 6242, 5465, 4915, 7552, 142678, 6571, 390010, 26007, 5818, 1280, 675, 10166, 23483, 6430, 9870, 624, 6263, 373509, 89941, 8912, 5176, 9121, 6194, 4207, 64284, 63977, 11252, 85358, 54986, 22858, 9444, 27342, 84626, 641339, 2700, 176, 57478, 11151, 10052, 3643, 51231, 10288, 8718, 57449, 54897, 1307, 5174, 25936, 9801, 220988, 6295, 51665, 26032, 653238, 730394, 9794, 3097, 256227,

					57670, 155060, 116988, 23087, 203102, 255926, 1993
protein modification	0.000175848	190	149.18	MKNK2, MAN2B1, MATK, FBXW9, MAPK6, CDK3, MTMR1, DUSP9, HDAC6, TSPAN7, UBE1, COG2, KIAA1804, GALNT10, FBXO16, BAG1, GNAQ, PRMT1, LIMK1, USP48, DDOST, TNFRSF14, LYK5, CCND1, SUGT1, PPP1CA, MAP4K2, PPM1B, PTK2B, FBXO25, FBXL6, FLJ31951, MGAT1, AATK, LOC651771, RNF167, USP43, ST6GALNAC2, ALG6, PTPRF, TRIOBP, FBXO7, CUL1, UBE3C, DUSP27, GYPC, ZAK, GAD1, MYO3B, EPHA8, EPHB2, TSSK3, CAMKK2, GALNT9, SSH1, SGK3, TRIB1, PTPN2, FBXO15, ST8SIA5, DUSP11, FBXO41, MAPK12, ROCK2, PPM1G, CLN3, PTPRZ1, ILKAP, RNF25, CHM, UBL4A, CUL4B, CAMK1D, STYXL1, FBXL19, CYLD, PHKG2, LOC650556, SUZ12, MAPK7, NMT1, NEK8, CCL2, PDGFRB, FBXL17, P4HA2, PJA2, PARP1, PARP14, EPHB1, STK36, UBE2F, MYOD1, GAK, NEURL2, CDC25B, PTPRA, PCSK2, LRRC29, MAPK3, SLK, FBXL14, TRIM5, UEVLD, ERBB3, ZDHHC17, RIPK5, RFWD2, PIGM, APH1A, INSRR, DPM3, ERO1L, NEK3, UBL3, PARP4, ALG8, PIGP, ANAPC4, MGRN1, UBE2I, TRAF7, STUB1, PRKCD, RNF123, SPCS1, ST3GAL6, PPM1M, MARK2, CBLB, STT3A, CHEK1, GALNT7, SPCS3, RPN1, ANAPC13, TNIK, CNOT4, SRPK2, BRD4, CDK9, HERC1, CDC14B, TTLL11, PTPN3, EPM2A, FYN, RIPK1, ROS1, TTLL3, HDAC11, VHL, UBE2E2, MAP3K12, PPP3CB, PTP4A2, UBE2D3, MAN1A2, HIPK1, PKN2, CDC7, CLK3, ST6GAL1, EPHB3, TIPARP, SENP5, TNFAIP3, FGFR4, TTN, FUT8, MGAT2, PRKCH, PPM1A, BMPR1A, TPST2, LIF, ZZEF1, OPN5, FBXO9, FUT9, CARM1, NTRK2, MIB2, KIAA0317, ULK4, ICK, RABGEF1, USP31, INSR, VRK3	2872, 4125, 4145, 84261, 5597, 1018, 8776, 1852, 10013, 7102, 7317, 22796, 84451, 55568, 157574, 573, 2776, 3276, 3984, 84196, 1650, 8764, 92335, 595, 10910, 5499, 5871, 5495, 2185, 26260, 26233, 153830, 4245, 9625, 651771, 26001, 124739, 10610, 29929, 5792, 11078, 25793, 8454, 9690, 92235, 2995, 51776, 2571, 140469, 2046, 2048, 81629, 10645, 50614, 54434, 23678, 10221, 5771, 201456, 29906, 8446, 150726, 6300, 9475, 5496, 1201, 5803, 80895, 64320, 1121, 8266, 8450, 57118, 51657, 54620, 1540, 5261, 650556, 23512, 5598, 4836, 284086, 6347, 5159, 64839, 8974, 9867, 142, 54625, 2047, 27148, 140739, 4654, 2580, 140825, 994, 5786, 5126, 26231, 5595, 9748, 144699, 85363, 55293, 2065, 23390, 25778, 64326, 93183, 51107, 3645, 54344, 30001, 4752, 5412, 143, 79053, 51227, 29945, 23295, 7329, 84231, 10273, 5580, 63891, 28972, 10402, 132160, 2011, 868, 3703, 1111, 51809, 60559, 6184, 25847, 23043, 4850, 6733, 23476, 1025, 8925, 8555, 158135, 5774, 7957, 2534, 8737, 6098, 26140, 79885, 7428, 7325, 7786, 5532, 8073, 7323, 10905, 204851, 5586, 8317, 1198, 6480, 2049, 25976, 205564, 7128, 2264, 7273, 2530, 4247, 5583, 5494, 657, 8459, 3976, 23140, 221391, 26268, 10690, 10498, 4915, 142678, 9870, 54986, 22858, 27342, 57478, 3643, 51231
RNA splicing, via transesterification reactions with bulged adenosine as nucleophile	0.000433081	16	6.37	LSM7, U2AF2, SFRS1, SF3B2, SF3A2, USP39, SNRPB2, NOVA1, U2AF1, DBR1, SRPK2, SNRPA1, DDX20, RTCD1, PHF5A, SFRS5	51690, 11338, 6426, 10992, 8175, 10713, 6629, 4857, 7307, 51163, 6733, 6627, 11218, 8634, 84844, 6430
RNA splicing, via transesterification reactions	0.000433081	16	6.37	LSM7, U2AF2, SFRS1, SF3B2, SF3A2, USP39, SNRPB2, NOVA1, U2AF1, DBR1, SRPK2, SNRPA1, DDX20, RTCD1, PHF5A, SFRS5	51690, 11338, 6426, 10992, 8175, 10713, 6629, 4857, 7307, 51163, 6733, 6627, 11218, 8634, 84844, 6430
nuclear mRNA splicing, via spliceosome	0.000433081	16	6.37	LSM7, U2AF2, SFRS1, SF3B2, SF3A2, USP39, SNRPB2, NOVA1, U2AF1, DBR1, SRPK2, SNRPA1, DDX20, RTCD1, PHF5A, SFRS5	51690, 11338, 6426, 10992, 8175, 10713, 6629, 4857, 7307, 51163, 6733, 6627, 11218, 8634, 84844, 6430
biopolymer modification	0.000629657	191	153.77	MKNK2, MAN2B1, MATK, FBXW9, MAPK6, CDK3, MTMR1, DUSP9, HDAC6, TSPAN7, UBE1, COG2, KIAA1804, GALNT10, FBXO16,	2872, 4125, 4145, 84261, 5597, 1018, 8776, 1852, 10013, 7102, 7317, 22796, 84451, 55568, 157574,

				BAG1, GNAQ, PRMT1, LIMK1, USP48, DDOST, TNFRSF14, LYK5, CCND1, SUGT1, PPP1CA, MAP4K2, PPM1B, PTK2B, FBXO25, FBXL6, FLJ31951, MGAT1, AATK, LOC651771, RNF167, USP43, ST6GALNAC2, ALG6, PTPRF, TRIOBP, FBXO7, CUL1, UBE3C, DUSP27, GYPC, ZAK, SSB, GAD1, MYO3B, EPHA8, EPHB2, TSSK3, CAMKK2, GALNT9, SSH1, SGK3, TRIB1, PTPN2, FBXO15, ST8SIA5, DUSP11, FBXO41, MAPK12, ROCK2, PPM1G, CLN3, PTPRZ1, ILKAP, RNF25, CHM, UBL4A, CUL4B, CAMK1D, STYXL1, FBXL19, CYLD, PHKG2, LOC650556, SUZ12, MAPK7, NMT1, NEK8, CCL2, PDGFRB, FBXL17, P4HA2, PJA2, PARP1, PARP14, EPHB1, STK36, UBE2F, MYOD1, GAK, NEURL2, CDC25B, PTPRA, PCSK2, LRRC29, MAPK3, SLK, FBXL14, TRIM5, UEVLD, ERBB3, ZDHHC17, RIPK5, RFWD2, PIGM, APH1A, INSRR, DPM3, ERO1L, NEK3, UBL3, PARP4, ALG8, PIGP, ANAPC4, MGRN1, UBE2I, TRAF7, STUB1, PRKCD, RNF123, SPCS1, ST3GAL6, PPM1M, MARK2, CBLB, STT3A, CHEK1, GALNT7, SPCS3, RPN1, ANAPC13, TNIK, CNOT4, SRPK2, BRD4, CDK9, HERC1, CDC14B, TTLL11, PTPN3, EPM2A, FYN, RIPK1, ROS1, TTLL3, HDAC11, VHL, UBE2E2, MAP3K12, PPP3CB, PTP4A2, UBE2D3, MAN1A2, HIPK1, PKN2, CDC7, CLK3, ST6GAL1, EPHB3, TIPARP, SENP5, TNFAIP3, FGFR4, TTN, FUT8, MGAT2, PRKCH, PPM1A, BMPR1A, TPST2, LIF, ZZEF1, OPN5, FBXO9, FUT9, CARM1, NTRK2, MIB2, KIAA0317, ULK4, ICK, RABGEF1, USP31, INSR, VRK3	573, 2776, 3276, 3984, 84196, 1650, 8764, 92335, 595, 10910, 5499, 5871, 5495, 2185, 26260, 26233, 153830, 4245, 9625, 651771, 26001, 124739, 10610, 29929, 5792, 11078, 25793, 8454, 9690, 92235, 2995, 51776, 6741, 2571, 140469, 2046, 2048, 81629, 10645, 50614, 54434, 23678, 10221, 5771, 201456, 29906, 8446, 150726, 6300, 9475, 5496, 1201, 5803, 80895, 64320, 1121, 8266, 8450, 57118, 51657, 54620, 1540, 5261, 650556, 23512, 5598, 4836, 284086, 6347, 5159, 64839, 8974, 9867, 142, 54625, 2047, 27148, 140739, 4654, 2580, 140825, 994, 5786, 5126, 26231, 5595, 9748, 144699, 85363, 55293, 2065, 23390, 25778, 64326, 93183, 51107, 3645, 54344, 30001, 4752, 5412, 143, 79053, 51227, 29945, 23295, 7329, 84231, 10273, 5580, 63891, 28972, 10402, 132160, 2011, 868, 3703, 1111, 51809, 60559, 6184, 25847, 23043, 4850, 6733, 23476, 1025, 8925, 8555, 158135, 5774, 7957, 2534, 8737, 6098, 26140, 79885, 7428, 7325, 7786, 5532, 8073, 7323, 10905, 204851, 5586, 8317, 1198, 6480, 2049, 25976, 205564, 7128, 2264, 7273, 2530, 4247, 5583, 5494, 657, 8459, 3976, 23140, 221391, 26268, 10690, 10498, 4915, 142678, 9870, 54986, 22858, 27342, 57478, 3643, 51231
protein amino acid N-linked glycosylation	0.000666693	11	3.65	TSPAN7, DDOST, MGAT1, ALG6, GYPC, ALG8, STT3A, RPN1, MAN1A2, FUT8, MGAT2	7102, 1650, 4245, 29929, 2995, 79053, 3703, 6184, 10905, 2530, 4247
system development	0.000835263	185	149.18	ZBTB7A, TLE2, CACNA1A, MATK, COL5A3, SPINT1, THBS1, SOX9, SH3GL2, DMRT1, FOXE1, OSTF1, EFNB1, FOXI1, PCDHAC2, PCDHAC1, PCDHA11, PCDHA10, PCDHA8, PCDHA7, PCDHA5, PCDHA4, PCDHA3, PCDHA2, PCDHA1, SGCD, ChGn, NKX3-1, BAG1, APBA1, GNAQ, LIMK1, CCM2, SPRY1, CXCL1, LFNG, PLXDC1, ETV4, CD164, BVES, ITM2B, MAP4K2, MSH6, GATA4, NRG1, BMP1, TFAP2A, JMJD6, TIMP2, FOXD3, CUL1, ZBTB7B, GYPC, KIF5C, PDPN, ATPIF1, EPHB2, CD9, TBX5, MSI1, SEMA4C, AZU1, ITGB1BP3, PLXNB2, ADORA2A, MAPK12, DOC2A, PTPRZ1, MOSPD3, MYT1, SERPINE2, CHM, CHRDL1, IL17C, GFRA1, BCL11B, CCL2, LHX1, SPOCK1, ARTS-1, TP53BP2, EPHB1, COL4A3, NRP2, SDCBP2, MYOD1, FEZ1, CXCL10, STMN3, NEURL2, HTRA2, CD40, PCSK2, SNAI1, MAPK3, CASP7, EMX2, PKP2, HDAC7A, PTHLH, BDNF, PAX6, ERBB3, RC3H1, CSDE1, SEMA6C, MYH7, SIX1, TITF1,	51341, 7089, 773, 4145, 50509, 6692, 7057, 6662, 6456, 1761, 2304, 26578, 1947, 2299, 56134, 56135, 56138, 56139, 56140, 56141, 56143, 56144, 56145, 56146, 56147, 6444, 55790, 4824, 573, 320, 2776, 3984, 83605, 10252, 2919, 3955, 57125, 2118, 8763, 11149, 9445, 5871, 2956, 2626, 3084, 649, 7020, 23210, 7077, 27022, 8454, 51043, 2995, 3800, 10630, 93974, 2048, 928, 6910, 4440, 54910, 566, 27231, 23654, 135, 6300, 8448, 5803, 64598, 4661, 5270, 1121, 91851, 27189, 2674, 64919, 6347, 3975, 6695, 51752, 7159, 2047, 1285, 8828, 27111, 4654, 9638, 3627, 50861, 140825, 27429, 958, 5126, 6615, 5595, 840, 2018, 5318, 51564, 5744, 627, 5080, 2065,

				NFKBIA, PPARD, FGF10, ANKH, DSCAML1, APP, SOD1, RBPJ, MSX1, EVC, CCNF, METRN, LY6H, TNFRSF11B, TPD52, KLF10, NRXN3, TNFAIP2, CYP46A1, ROBO3, FLI1, TMEM176B, PBX4, NTNG2, LMX1B, TLR4, LHX2, CNFN, NAPA, LHB, KLK7, PAFAH1B3, SEMA7A, RGMA, NINJ1, KLF4, IRF4, EPM2A, FYN, BTD, KRT1, NTN4, CSRP2, PPP3CB, STMN1, BMP8B, ARX, PITX2, UNC5C, MKX, ARNT2, GJA1, CANX, TLX3, TTN, NCKAP1, NCOA4, BMPR1A, PLCE1, DMC1, LIF, PMP22, DLG4, TFAP2B, SMAP1, PPP2R5D, APLP1, PPARA, NTRK2, COL2A1, CACNA1H, SERPINF1, MEF2B, QKI, ACAN, INSR	149041, 7812, 10500, 4625, 6495, 7080, 4792, 5467, 2255, 56172, 57453, 351, 6647, 3516, 4487, 2121, 899, 79006, 4062, 4982, 7163, 7071, 9369, 7127, 10858, 64221, 2313, 28959, 80714, 84628, 4010, 7099, 9355, 84518, 8775, 3972, 5650, 5050, 8482, 56963, 4814, 9314, 3662, 7957, 2534, 686, 3848, 59277, 1466, 5532, 3925, 656, 170302, 5308, 8633, 283078, 9915, 2697, 821, 30012, 7273, 10787, 8031, 657, 51196, 11144, 3976, 5376, 1742, 7021, 60682, 5528, 333, 5465, 4915, 1280, 8912, 5176, 4207, 9444, 176, 3643
glycoprotein metabolic process	0.000851773	27	14.24	MAN2B1, NGLY1, TSPAN7, COG2, GALNT10, DDOST, MGAT1, ST6GALNAC2, ALG6, GYPC, GALNT9, ST8SIA5, CLN3, APBA2BP, APH1A, DPM3, ALG8, AGA, ST3GAL6, STT3A, GALNT7, RPN1, MAN1A2, ST6GAL1, FUT8, MGAT2, FUT9	4125, 55768, 7102, 22796, 55568, 1650, 4245, 10610, 29929, 2995, 50614, 29906, 1201, 63941, 51107, 54344, 79053, 175, 10402, 3703, 51809, 6184, 10905, 6480, 2530, 4247, 10690
cell adhesion	0.00133923	91	66.81	COL5A3, THBS1, ERBB2IP, THBS4, SOX9, C9orf127, SLC26A6, PGM5, EFNB1, TGFBI, PCDHA9, PCDHAC2, PCDHAC1, PCDHA13, PCDHA12, PCDHA11, PCDHA10, PCDHA8, PCDHA7, PCDHA5, PCDHA4, PCDHA3, PCDHA2, PCDHA1, RP11-138L21.1, CNTNAP3, LOC727745, APBA1, PCDH10, ITGB8, SDK2, SIPA1, CD164, PTK2B, BMP1, ADAMDEC1, NEDD9, GMDS, LGALS3BP, PTPRF, PARVG, KIRREL, CD9, DSC2, L3MBTL4, AZU1, MADCAM1, TMEM8, SSX2IP, C1orf41, ARPC1B, F8, CLDN3, CCL2, FBN2, SPOCK1, VWA1, COL4A3, NRP2, PARVA, FEZ1, SNAI1, PKP2, F5, PCDH9, CTNND2, CDH12, DSCAML1, APP, CLDN9, DAG1, NRXN3, CD99, COL6A2, RGMA, NINJ1, MUC4, MYOM3, CTNNA3, TTN, VCL, CLDN5, CLDN7, APLP1, PVR, ARHGDIG, PVRL1, ACAN, COL16A1, SUSD5, ADAM32	50509, 7057, 55914, 7060, 6662, 51754, 65010, 5239, 1947, 7045, 9752, 56134, 56135, 56136, 56137, 56138, 56139, 56140, 56141, 56143, 56144, 56145, 56146, 56147, 389722, 79937, 727745, 320, 57575, 3696, 54549, 6494, 8763, 2185, 649, 27299, 4739, 2762, 3959, 5792, 64098, 55243, 928, 1824, 91133, 566, 8174, 58986, 117178, 51668, 10095, 2157, 1365, 6347, 2201, 6695, 64856, 1285, 8828, 55742, 9638, 6615, 5318, 2153, 5101, 1501, 1010, 57453, 351, 9080, 1605, 9369, 4267, 1292, 56963, 4814, 4585, 127294, 29119, 7273, 7414, 7122, 1366, 333, 5817, 398, 5818, 176, 1307, 26032, 203102
biological adhesion	0.00133923	91	66.81	COL5A3, THBS1, ERBB2IP, THBS4, SOX9, C9orf127, SLC26A6, PGM5, EFNB1, TGFBI, PCDHA9, PCDHAC2, PCDHAC1, PCDHA13, PCDHA12, PCDHA11, PCDHA10, PCDHA8, PCDHA7, PCDHA5, PCDHA4, PCDHA3, PCDHA2, PCDHA1, RP11-138L21.1, CNTNAP3, LOC727745, APBA1, PCDH10, ITGB8, SDK2, SIPA1, CD164, PTK2B, BMP1, ADAMDEC1, NEDD9, GMDS, LGALS3BP, PTPRF, PARVG, KIRREL, CD9, DSC2, L3MBTL4, AZU1, MADCAM1, TMEM8, SSX2IP, C1orf41, ARPC1B, F8, CLDN3, CCL2, FBN2, SPOCK1, VWA1, COL4A3, NRP2, PARVA, FEZ1, SNAI1, PKP2, F5, PCDH9, CTNND2, CDH12, DSCAML1, APP, CLDN9, DAG1, NRXN3, CD99, COL6A2, RGMA, NINJ1, MUC4, MYOM3, CTNNA3, TTN, VCL, CLDN5, CLDN7, APLP1, PVR, ARHGDIG, PVRL1, ACAN, COL16A1, SUSD5, ADAM32	50509, 7057, 55914, 7060, 6662, 51754, 65010, 5239, 1947, 7045, 9752, 56134, 56135, 56136, 56137, 56138, 56139, 56140, 56141, 56143, 56144, 56145, 56146, 56147, 389722, 79937, 727745, 320, 57575, 3696, 54549, 6494, 8763, 2185, 649, 27299, 4739, 2762, 3959, 5792, 64098, 55243, 928, 1824, 91133, 566, 8174, 58986, 117178, 51668, 10095, 2157, 1365, 6347, 2201, 6695, 64856, 1285, 8828, 55742, 9638, 6615, 5318, 2153, 5101, 1501, 1010, 57453, 351, 9080, 1605, 9369, 4267, 1292, 56963, 4814, 4585, 127294, 29119, 7273, 7414, 7122, 1366, 333, 5817, 398, 5818, 176, 1307, 26032, 203102
localization	0.00142282	305	262.66	CDC37, MGC19604, CACNA1A, RANBP3, COL5A3, TIMM13, AP4E1, RAB8B, THBS1, ERBB2IP, THBS4, CACNA1G, PITPNC1, KCTD2,	11140, 112812, 773, 8498, 50509, 26517, 23431, 51762, 7057, 55914, 7060, 8913, 26207, 23510,

				SLC25A43, SLC25A14, SNX8, TMED4, PTGES2, LCN8, OBP2B, KIF9, SLC26A6, ATP6V1G1, VPS13A, SLC16A2, KIF4A, EFNB1, ARF1, COG2, TRPM3, TLN1, RASEF, APBA1, AQP7, LOC730908, CACNG6, FTL, RCP9, LIMK1, YKT6, DBNL, RAMP3, EXOC1, KIF17, GARS, SNX10, ABCA8, LYK5, HAP1, ETV4, PACS1, ELMOD1, LRP4, CDCA5, SLC15A3, SLC29A2, MAP4K2, ATP6V1C2, KCNF1, SLC25A37, CHMP7, HOOK3, SCARA3, KIF13A, SLC17A2, SSR1, RAB24, SCP2, SLC5A9, RALBP1, HMG2L1, CBY1, TSPO, TOMM22, MTX1, ATP1B1, ATP8B2, KIF5C, RPL11, VPS13D, PDPN, STX12, AQP5, CD9, SLC41A2, TBX5, RAB35, KCNS2, CHMP4C, CYB5A, AZU1, HCN2, ADORA2A, ITSN2, CLN3, GTF3C1, GGA2, DOC2A, KCTD13, GRIN2A, SYT6, SLC26A4, CAV1, ARPC1B, STX16, OSBPL2, ARFGAP1, GNAS, HSPD1, AAMP, TNS1, HDLBP, SERPINE2, CHM, UPF3B, SYTL4, SLC10A3, CLCN4, SLC9A5, NUTF2, MRC1, SEC61A2, BNIP3, VIM, C10orf125, SLC6A2, ABCD4, AP2B1, RAMP2, SIL1, GLRX, SPOCK1, SETD1B, ABCB10, SLC30A10, LOC728528, CYC1, SEC22A, COL4A3, ATG16L1, NRP2, ACCN4, SMG7, KCTD3, SDCBP2, APBA2BP, SYT9, DNHD1, SLC6A5, FEZ1, SLC17A6, COX18, G3BP2, CXCL10, PLTP, RIMS4, SLC35C2, VPS16, RIN2, CD40, LBP, SLC24A3, PLLP, SLC12A4, ABCC11, SFXN2, GRIN2B, BDNF, NUP98, UEVLD, SLC17A8, SYT1, ESPL1, ZDHHC17, LRP1, SLC26A9, RAB7L1, KCNT2, TMEM9, SLC39A1, SLC8A3, CHMP4A, MYH7, SIX1, ERO1L, TITF1, STXBP6, NFKBIA, PPARD, SLC39A7, ITPR3, SLC25A30, TPT1, PARP4, FGF10, ANKH, TIMM8B, SLC37A4, CENTD2, APP, CLIC6, SLC34A2, CYBA, SLC7A5, TRAF7, PCOLN3, BAIAP3, HBA2, SLC25A38, MYRIP, KCTD6, SLC35C1, KCNK4, STX3, CBLB, LRP12, TPD52, KIF26A, NRXN3, ROBO3, SDHD, SLC26A11, LOC652834, C1QTNF1, ABCG1, TMEM1, COL6A2, KLF15, COG5, SLC1A6, LMX1B, NUP62, NAPA, KCNN4, UACA, SELS, AP3S2, HCN4, AP3B2, VPS33B, RHCG, GRIN3A, FYN, AAAS, OSBPL8, SLC16A12, SLC25A28, GRIK3, SLC9A1, SH3KBP1, ARX, UNC5C, MFSD8, SRP14, ATP8B4, RAB27A, TUBAL3, STXBP3, C15orf27, HTR3E, STX11, GJA1, CANX, BNIP1, TLX3, SLC40A1, LTB4R, AP4S1, VCL, GPR120, SLC18A3, UNC84B, SLC25A1, CRK, TRPV1, MYBBP1A, SMG6, DNAH8, KCNQ5, APLP1, FXYD7, GAPDHS, DNM2, SLC44A2, SLC25A42, RDX, PPARA, SLC18A2, COL2A1, SLC25A15, RYR3, RHOT2, CACNA1H, SLC16A5, RAB17, PACSIN2, QKI, RABGEF1, GJA3, CORO1A, GJA7, COL16A1, PDZK1, CENTG3	203427, 9016, 29886, 222068, 80142, 138307, 29989, 64147, 65010, 9550, 23230, 6567, 24137, 1947, 375, 22796, 80036, 7094, 158158, 320, 364, 730908, 59285, 2512, 27297, 3984, 10652, 28988, 10268, 55763, 57576, 2617, 29887, 10351, 92335, 9001, 2118, 55690, 55531, 4038, 113130, 51296, 3177, 5871, 245973, 3754, 51312, 91782, 84376, 51435, 63971, 10246, 6745, 53917, 6342, 200010, 10928, 10042, 25776, 706, 56993, 4580, 481, 57198, 3800, 6135, 55187, 10630, 23673, 362, 928, 84102, 6910, 11021, 3788, 92421, 1528, 566, 610, 135, 50618, 1201, 2975, 23062, 8448, 253980, 2903, 148281, 5172, 857, 10095, 8675, 9885, 55738, 2778, 3329, 14, 7145, 3069, 5270, 1121, 65109, 94121, 8273, 1183, 6553, 10204, 4360, 55176, 664, 7431, 282969, 6530, 5826, 163, 10266, 64374, 2745, 6695, 23067, 23456, 55532, 728528, 1537, 26984, 1285, 55054, 8828, 55515, 9887, 51133, 27111, 63941, 143425, 144132, 9152, 9638, 57084, 285521, 9908, 3627, 5360, 140730, 51006, 64601, 54453, 958, 3929, 57419, 51090, 6560, 85320, 118980, 2904, 627, 4928, 55293, 246213, 6857, 9700, 23390, 4035, 115019, 8934, 343450, 252839, 27173, 6547, 29082, 4625, 6495, 30001, 7080, 29091, 4792, 5467, 7922, 3710, 253512, 7178, 143, 2255, 56172, 26521, 2542, 116985, 351, 54102, 10568, 1535, 8140, 84231, 5119, 8938, 3040, 54977, 25924, 200845, 55343, 50801, 6809, 868, 29967, 7163, 26153, 9369, 64221, 6392, 284129, 652834, 114897, 9619, 7109, 1292, 28999, 10466, 6511, 4010, 23636, 8775, 3783, 55075, 55829, 10239, 10021, 8120, 26276, 51458, 116443, 2534, 8086, 114882, 387700, 81894, 2899, 6548, 30011, 170302, 8633, 256471, 6727, 79895, 5873, 79861, 6814, 123591, 285242, 8676, 2697, 821, 662, 30012, 30061, 1241, 11154, 7414, 338557, 6572, 25777, 6576, 1398, 7442, 10514, 23293, 1769, 56479, 333, 53822, 26330, 1785, 57153, 284439, 5962, 5465, 6571, 1280, 10166, 6263, 89941, 8912, 9121, 64284, 11252, 9444, 27342, 2700, 11151, 10052, 1307, 5174, 116988
	0.00159133	6	1.41	TLN1, GJC1, CD9, GJA1, VCL, GJA7	7094, 125111, 928, 2697, 7414, 10052

intercellular junction assembly					
developmental process	0.00188923	316	274.28	ZBTB7A, TLE2, CACNA1A, MATK, COL5A3, SPINT1, THBS1, ERBB2IP, THBS4, WNT9B, SOX9, ZIC3, BIRC4, GNA12, HOXA10, SETX, SH3GL2, DMRT1, FOXE1, CCIN, OSTF1, HDAC6, EFNB1, FOXI1, PCDHAC2, PCDHAC1, PCDHA11, PCDHA10, PCDHA8, PCDHA7, PCDHA5, PCDHA4, PCDHA3, PCDHA2, PCDHA1, SGCD, SFRP1, ChGn, NKX3-1, BAG1, APBA1, GNAQ, BCL2L12, RUVBL2, PRMT1, LIMK1, CCM2, SPATA18, SPRY1, CXCL1, LZIC, TNFRSF14, EVX1, LFNG, AHR, ITGB8, CHAD, HOXB4, PLXDC1, HAP1, ETV4, SIPA1, DIXDC1, CCND1, ATG5, CD164, BVES, ITM2B, LRP4, MAP4K2, MSH6, GRHL1, GATA4, NRG1, PTK2B, BMP1, TXNDC5, SERPINB9, NEDD9, PBX2, TFAP2A, JMJD6, TIMP2, FOXD3, BIK, CUL1, ZBTB7B, GYPC, ZAK, KIF5C, PDPN, ATPIF1, EPHB2, TSSK3, CD9, DIABLO, TBX5, MSI1, SSH1, EBAG9, SULF1, SGK3, SEMA4C, AZU1, MADCAM1, ITGB1BP3, PLXNB2, ADORA2A, MAPK12, CLN3, DOC2A, PTPRZ1, MOSPD3, OGFR, MYT1, HSPD1, SERPINE2, CHM, CHRDL1, IL17C, BNIP3, PLXDC2, GFRA1, PPP1R13B, BCL11B, CCL2, LHX1, FBN2, SPOCK1, ARTS-1, TP53BP2, EPHB1, COL4A3, NRP2, STK36, SDCBP2, E2F1, MYOD1, P53AIP1, FEZ1, HOP, CXCL10, STMN3, NEURL2, GNRH2, HTRA2, CD40, PCSK2, SNAI1, MAPK3, CASP7, SLK, PDCD4, EMX2, ADRB1, PKP2, HDAC7A, PTHLH, BDNF, PAX6, ABTB2, IGFBP6, ERBB3, KRT18, ESPL1, LRP1, RC3H1, DEDD, CSDE1, INSRR, SEMA6C, PBXIP1, CD5L, MYH7, SIX1, TITF1, NFKBIA, PPARD, TPT1, PARP4, FGF10, CTNND2, ANKH, CENTD2, DSCAML1, APP, SOD1, TFF1, RBPJ, KLF3, MSX1, EVC, TRAF7, CCNF, DNASE1, METRN, CCBP2, DAG1, MADD, MARK2, DNASE1L3, LY6H, TNFRSF11B, TPD52, KLF10, TRAF3, NRXN3, TNFAIP2, CYP46A1, ROBO3, FLI1, MX1, BOK, TMEM176B, SRPK2, PBX4, FKBP8, NTNG2, PRRX2, PTGS1, LMX1B, TLR4, LHX2, NUP62, CNFN, NAPA, SIX5, LHB, KLK7, PAFAH1B3, UACA, SEMA7A, RGMA, RHCG, ANGPTL2, FANCC, NINJ1, KLF4, IRF4, EPM2A, FYN, PLAGL1, RIPK1, MAP7, BTD, VHL, KRT1, NTN4, CSRP2, WNT10B, PPP3CB, NOC3L, STMN1, SCMH1, TAL1, BMP8B, SH3KBP1, RBBP7, ARX, PITX2, UNC5C, RAB27A, BCL2L10, MKX, DDX20, CDC7, TXNIP, MAGI3, MORF4L1, ARNT2, ST6GAL1, TPD52L1, TNFAIP3, GJA1, UNC5A, CANX, BNIP1, TLX3, PRELID1, TTN, NCKAP1, SLC40A1, FUT8, NCOA4, FAS, VCL, BMPR1A, PLCE1, DMC1, LIF, GSCL, PMP22, DLG4, TFAP2B, SMAP1, PPP2R5D, APLP1, CLPTM1, MEGF8, DNM2, RDX, RTKN, PPARA, NTRK2, COL2A1, RHOT2, CACNA1H, SERPINF1, MEF2B, ICK, QKI, ACAN, INSR, TNFRSF25, ASB1, TRIM35	51341, 7089, 773, 4145, 50509, 6692, 7057, 55914, 7060, 7484, 6662, 7547, 331, 2768, 3206, 23064, 6456, 1761, 2304, 881, 26578, 10013, 1947, 2299, 56134, 56135, 56138, 56139, 56140, 56141, 56143, 56144, 56145, 56146, 56147, 6444, 6422, 55790, 4824, 573, 320, 2776, 83596, 10856, 3276, 3984, 83605, 132671, 10252, 2919, 84328, 8764, 2128, 3955, 196, 3696, 1101, 3214, 57125, 9001, 2118, 6494, 85458, 595, 9474, 8763, 11149, 9445, 4038, 5871, 2956, 29841, 2626, 3084, 2185, 649, 81567, 5272, 4739, 5089, 7020, 23210, 7077, 27022, 638, 8454, 51043, 2995, 51776, 3800, 10630, 93974, 2048, 81629, 928, 56616, 6910, 4440, 54434, 9166, 23213, 23678, 54910, 566, 8174, 27231, 23654, 135, 6300, 1201, 8448, 5803, 64598, 11054, 4661, 3329, 5270, 1121, 91851, 27189, 664, 84898, 2674, 23368, 64919, 6347, 3975, 2201, 6695, 51752, 7159, 2047, 1285, 8828, 27148, 27111, 1869, 4654, 63970, 9638, 84525, 3627, 50861, 140825, 2797, 27429, 958, 5126, 6615, 5595, 840, 9748, 27250, 2018, 153, 5318, 51564, 5744, 627, 5080, 25841, 3489, 2065, 3875, 9700, 4035, 149041, 9191, 7812, 3645, 10500, 57326, 922, 4625, 6495, 7080, 4792, 5467, 7178, 143, 2255, 1501, 56172, 116985, 57453, 351, 6647, 7031, 3516, 51274, 4487, 2121, 84231, 899, 1773, 79006, 1238, 1605, 8567, 2011, 1776, 4062, 4982, 7163, 7071, 7187, 9369, 7127, 10858, 64221, 2313, 4599, 666, 28959, 6733, 80714, 23770, 84628, 51450, 5742, 4010, 7099, 9355, 23636, 84518, 8775, 147912, 3972, 5650, 5050, 55075, 8482, 56963, 51458, 23452, 2176, 4814, 9314, 3662, 7957, 2534, 5325, 8737, 9053, 686, 7428, 3848, 59277, 1466, 7480, 5532, 64318, 3925, 22955, 6886, 656, 30011, 5931, 170302, 5308, 8633, 5873, 10017, 283078, 11218, 8317, 10628, 260425, 10933, 9915, 6480, 7164, 7128, 2697, 90249, 821, 662, 30012, 27166, 7273, 10787, 30061, 2530, 8031, 355, 7414, 657, 51196, 11144, 3976, 2928, 5376, 1742, 7021, 60682, 5528, 333, 1209, 1954, 1785, 5962, 6242, 5465,

					4915, 1280, 89941, 8912, 5176, 4207, 22858, 9444, 176, 3643, 8718, 51665, 23087
establishment of localization	0.00221412	270	231.46	CDC37, MGC19604, CACNA1A, RANBP3, COL5A3, TIMM13, AP4E1, RAB8B, CACNA1G, PITPNC1, KCTD2, SLC25A43, SLC25A14, SNX8, TMED4, PTGES2, LCN8, OBP2B, KIF9, SLC26A6, ATP6V1G1, VPS13A, SLC16A2, KIF4A, ARF1, COG2, TRPM3, RASEF, APBA1, AQP7, LOC730908, CACNG6, FTL, RCP9, YKT6, DBNL, RAMP3, EXOC1, KIF17, GARS, SNX10, ABCA8, LYK5, HAP1, PACS1, ELMOD1, LRP4, CDCA5, SLC15A3, SLC29A2, MAP4K2, ATP6V1C2, KCNF1, SLC25A37, CHMP7, SCARA3, KIF13A, SLC17A2, SSR1, RAB24, SCP2, SLC5A9, RALBP1, HMG2L1, TSPO, TOMM22, MTX1, ATP1B1, ATP8B2, KIF5C, RPL11, PDPN, STX12, AQP5, SLC41A2, RAB35, KCNS2, CHMP4C, CYB5A, AZU1, HCN2, ADORA2A, ITSN2, CLN3, GTF3C1, GGA2, DOC2A, KCTD13, GRIN2A, SYT6, SLC26A4, CAV1, STX16, OSBPL2, ARFGAP1, GNAS, HSPD1, HDLBP, CHM, UPF3B, SYTL4, SLC10A3, CLCN4, SLC9A5, NUTF2, MRC1, SEC61A2, BNIP3, C10orf125, SLC6A2, ABCD4, AP2B1, RAMP2, SIL1, GLRX, SETD1B, ABCB10, SLC30A10, LOC728528, CYC1, SEC22A, COL4A3, ATG16L1, ACCN4, SMG7, KCTD3, SDCBP2, APBA2BP, SYT9, DNHD1, SLC6A5, SLC17A6, COX18, G3BP2, PLTP, RIMS4, SLC35C2, VPS16, RIN2, CD40, LBP, SLC24A3, PLLP, SLC12A4, ABCC11, SFXN2, GRIN2B, BDNF, NUP98, UEVLD, SLC17A8, SYT1, ESPL1, ZDHHC17, LRP1, SLC26A9, RAB7L1, KCNT2, TMEM9, SLC39A1, SLC8A3, CHMP4A, MYH7, ERO1L, STXBP6, NFKBIA, PPARD, SLC39A7, ITPR3, SLC25A30, TPT1, PARP4, FGF10, ANKH, TIMM8B, SLC37A4, APP, CLIC6, SLC34A2, CYBA, SLC7A5, TRAF7, PCOLN3, BAIAP3, HBA2, SLC25A38, MYRIP, KCTD6, SLC35C1, KCNK4, STX3, CBLB, LRP12, TPD52, KIF26A, SDHD, SLC26A11, LOC652834, C1QTNF1, ABCG1, TMEM1, COL6A2, KLF15, COG5, SLC1A6, NUP62, NAPA, KCNN4, UACA, SELS, AP3S2, HCN4, AP3B2, VPS33B, RHCG, GRIN3A, FYN, AAAS, OSBPL8, SLC16A12, SLC25A28, GRIK3, SLC9A1, SH3KBP1, MFSD8, SRP14, ATP8B4, RAB27A, TUBAL3, STXBP3, C15orf27, HTR3E, STX11, GJA1, CANX, BNIP1, SLC40A1, AP4S1, GPR120, SLC18A3, UNC84B, SLC25A1, TRPV1, MYBBP1A, SMG6, DNAH8, KCNQ5, APLP1, FXYD7, DNM2, SLC44A2, SLC25A42, PPARA, SLC18A2, COL2A1, SLC25A15, RYR3, RHOT2, CACNA1H, SLC16A5, RAB17, PACSIN2, QKI, RABGEF1, GJA3, CORO1A, GJA7, COL16A1, PDZK1, CENTG3	11140, 112812, 773, 8498, 50509, 26517, 23431, 51762, 8913, 26207, 23510, 203427, 9016, 29886, 222068, 80142, 138307, 29989, 64147, 65010, 9550, 23230, 6567, 24137, 375, 22796, 80036, 158158, 320, 364, 730908, 59285, 2512, 27297, 10652, 28988, 10268, 55763, 57576, 2617, 29887, 10351, 92335, 9001, 55690, 55531, 4038, 113130, 51296, 3177, 5871, 245973, 3754, 51312, 91782, 51435, 63971, 10246, 6745, 53917, 6342, 200010, 10928, 10042, 706, 56993, 4580, 481, 57198, 3800, 6135, 10630, 23673, 362, 84102, 11021, 3788, 92421, 1528, 566, 610, 135, 50618, 1201, 2975, 23062, 8448, 253980, 2903, 148281, 5172, 857, 8675, 9885, 55738, 2778, 3329, 3069, 1121, 65109, 94121, 8273, 1183, 6553, 10204, 4360, 55176, 664, 282969, 6530, 5826, 163, 10266, 64374, 2745, 23067, 23456, 55532, 728528, 1537, 26984, 1285, 55054, 55515, 9887, 51133, 27111, 63941, 143425, 144132, 9152, 57084, 285521, 9908, 5360, 140730, 51006, 64601, 54453, 958, 3929, 57419, 51090, 6560, 85320, 118980, 2904, 627, 4928, 55293, 246213, 6857, 9700, 23390, 4035, 115019, 8934, 343450, 252839, 27173, 6547, 29082, 4625, 30001, 29091, 4792, 5467, 7922, 3710, 253512, 7178, 143, 2255, 56172, 26521, 2542, 351, 54102, 10568, 1535, 8140, 84231, 5119, 8938, 3040, 54977, 25924, 200845, 55343, 50801, 6809, 868, 29967, 7163, 26153, 6392, 284129, 652834, 114897, 9619, 7109, 1292, 28999, 10466, 6511, 23636, 8775, 3783, 55075, 55829, 10239, 10021, 8120, 26276, 51458, 116443, 2534, 8086, 114882, 387700, 81894, 2899, 6548, 30011, 256471, 6727, 79895, 5873, 79861, 6814, 123591, 285242, 8676, 2697, 821, 662, 30061, 11154, 338557, 6572, 25777, 6576, 7442, 10514, 23293, 1769, 56479, 333, 53822, 1785, 57153, 284439, 5465, 6571, 1280, 10166, 6263, 89941, 8912, 9121, 64284, 11252, 9444, 27342, 2700, 11151, 10052, 1307, 5174, 116988
anatomical structure	0.00303752	213	179.36	ZBTB7A, TLE2, CACNA1A, MATK, COL5A3, SPINT1, THBS1, ERBB2IP, THBS4, SOX9, GNA12, SH3GL2, DMRT1, FOXE1, OSTF1,	51341, 7089, 773, 4145, 50509, 6692, 7057, 55914, 7060, 6662, 2768, 6456, 1761, 2304,

development				EFNB1, FOXI1, PCDHAC2, PCDHAC1, PCDHA11, PCDHA10, PCDHA8, PCDHA7, PCDHA5, PCDHA4, PCDHA3, PCDHA2, PCDHA1, SGCD, SFRP1, ChGn, NKX3-1, BAG1, APBA1, GNAQ, RUVBL2, LIMK1, CCM2, SPRY1, CXCL1, LFNG, CHAD, PLXDC1, ETV4, SIPA1, CD164, BVES, ITM2B, LRP4, MAP4K2, MSH6, GATA4, NRG1, BMP1, NEDD9, TFAP2A, JMJD6, TIMP2, FOXD3, CUL1, ZBTB7B, GYPC, KIF5C, PDPN, ATPIF1, EPHB2, CD9, TBX5, MSI1, SSH1, EBAG9, SEMA4C, AZU1, ITGB1BP3, PLXNB2, ADORA2A, MAPK12, DOC2A, PTPRZ1, MOSPD3, OGFR, MYT1, SERPINE2, CHM, CHRDL1, IL17C, GFRA1, BCL11B, CCL2, LHX1, FBN2, SPOCK1, ARTS-1, TP53BP2, EPHB1, COL4A3, NRP2, SDCBP2, MYOD1, FEZ1, CXCL10, STMN3, NEURL2, HTRA2, CD40, PCSK2, SNAI1, MAPK3, CASP7, EMX2, PKP2, HDAC7A, PTHLH, BDNF, PAX6, ABTB2, IGFBP6, ERBB3, KRT18, RC3H1, CSDE1, SEMA6C, MYH7, SIX1, TITF1, NFKBIA, PPARD, FGF10, ANKH, CENTD2, DSCAML1, APP, SOD1, RBPJ, MSX1, EVC, CCNF, METRN, DAG1, MARK2, LY6H, TNFRSF11B, TPD52, KLF10, NRXN3, TNFAIP2, CYP46A1, ROBO3, FLI1, TMEM176B, PBX4, NTNG2, LMX1B, TLR4, LHX2, CNFN, NAPA, LHB, KLK7, PAFAH1B3, SEMA7A, RGMA, RHCG, NINJ1, KLF4, IRF4, EPM2A, FYN, MAP7, BTD, VHL, KRT1, NTN4, CSRP2, PPP3CB, STMN1, SCMH1, BMP8B, ARX, PITX2, UNC5C, MKX, MORF4L1, ARNT2, GJA1, CANX, TLX3, TTN, NCKAP1, SLC40A1, NCOA4, VCL, BMPR1A, PLCE1, DMC1, LIF, GSCL, PMP22, DLG4, TFAP2B, SMAP1, PPP2R5D, APLP1, RDX, PPARA, NTRK2, COL2A1, CACNA1H, SERPINF1, MEF2B, QKI, ACAN, INSR	26578, 1947, 2299, 56134, 56135, 56138, 56139, 56140, 56141, 56143, 56144, 56145, 56146, 56147, 6444, 6422, 55790, 4824, 573, 320, 2776, 10856, 3984, 83605, 10252, 2919, 3955, 1101, 57125, 2118, 6494, 8763, 11149, 9445, 4038, 5871, 2956, 2626, 3084, 649, 4739, 7020, 23210, 7077, 27022, 8454, 51043, 2995, 3800, 10630, 93974, 2048, 928, 6910, 4440, 54434, 9166, 54910, 566, 27231, 23654, 135, 6300, 8448, 5803, 64598, 11054, 4661, 5270, 1121, 91851, 27189, 2674, 64919, 6347, 3975, 2201, 6695, 51752, 7159, 2047, 1285, 8828, 27111, 4654, 9638, 3627, 50861, 140825, 27429, 958, 5126, 6615, 5595, 840, 2018, 5318, 51564, 5744, 627, 5080, 25841, 3489, 2065, 3875, 149041, 7812, 10500, 4625, 6495, 7080, 4792, 5467, 2255, 56172, 116985, 57453, 351, 6647, 3516, 4487, 2121, 899, 79006, 1605, 2011, 4062, 4982, 7163, 7071, 9369, 7127, 10858, 64221, 2313, 28959, 80714, 84628, 4010, 7099, 9355, 84518, 8775, 3972, 5650, 5050, 8482, 56963, 51458, 4814, 9314, 3662, 7957, 2534, 9053, 686, 7428, 3848, 59277, 1466, 5532, 3925, 22955, 656, 170302, 5308, 8633, 283078, 10933, 9915, 2697, 821, 30012, 7273, 10787, 30061, 8031, 7414, 657, 51196, 11144, 3976, 2928, 5376, 1742, 7021, 60682, 5528, 333, 5962, 5465, 4915, 1280, 8912, 5176, 4207, 9444, 176, 3643
glycoprotein biosynthetic process	0.00374698	22	11.99	TSPAN7, COG2, GALNT10, DDOST, MGAT1, ST6GALNAC2, ALG6, GYPC, GALNT9, ST8SIA5, APBA2BP, DPM3, ALG8, ST3GAL6, STT3A, GALNT7, RPN1, MAN1A2, ST6GAL1, FUT8, MGAT2, FUT9	7102, 22796, 55568, 1650, 4245, 10610, 29929, 2995, 50614, 29906, 63941, 54344, 79053, 10402, 3703, 51809, 6184, 10905, 6480, 2530, 4247, 10690
multicellular organismal development	0.00392401	231	197.16	ZBTB7A, TLE2, CACNA1A, MATK, COL5A3, SPINT1, THBS1, WNT9B, SOX9, ZIC3, GNA12, HOXA10, SH3GL2, DMRT1, FOXE1, CCIN, OSTF1, HDAC6, EFNB1, FOXI1, PCDHAC2, PCDHAC1, PCDHA11, PCDHA10, PCDHA8, PCDHA7, PCDHA5, PCDHA4, PCDHA3, PCDHA2, PCDHA1, SGCD, SFRP1, ChGn, NKX3-1, BAG1, APBA1, GNAQ, PRMT1, LIMK1, CCM2, SPATA18, SPRY1, CXCL1, LZIC, EVX1, LFNG, ITGB8, HOXB4, PLXDC1, HAP1, ETV4, DIXDC1, CD164, BVES, ITM2B, LRP4, MAP4K2, MSH6, GRHL1, GATA4, NRG1, BMP1, PBX2, TFAP2A, JMJD6, TIMP2, FOXD3, CUL1, ZBTB7B, GYPC, KIF5C, PDPN, ATPIF1, EPHB2, TSSK3, CD9, TBX5, MSI1, SEMA4C, AZU1, ITGB1BP3, PLXNB2, ADORA2A, MAPK12, DOC2A, PTPRZ1, MOSPD3, MYT1, SERPINE2, CHM, CHRDL1, IL17C, PLXDC2,	51341, 7089, 773, 4145, 50509, 6692, 7057, 7484, 6662, 7547, 2768, 3206, 6456, 1761, 2304, 881, 26578, 10013, 1947, 2299, 56134, 56135, 56138, 56139, 56140, 56141, 56143, 56144, 56145, 56146, 56147, 6444, 6422, 55790, 4824, 573, 320, 2776, 3276, 3984, 83605, 132671, 10252, 2919, 84328, 2128, 3955, 3696, 3214, 57125, 9001, 2118, 85458, 8763, 11149, 9445, 4038, 5871, 2956, 29841, 2626, 3084, 649, 5089, 7020, 23210, 7077, 27022, 8454, 51043, 2995, 3800, 10630, 93974, 2048, 81629, 928, 6910, 4440, 54910, 566, 27231, 23654, 135, 6300, 8448, 5803, 64598,

				GFRA1, BCL11B, CCL2, LHX1, SPOCK1, ARTS-1, TP53BP2, EPHB1, COL4A3, NRP2, STK36, SDCBP2, MYOD1, FEZ1, HOP, CXCL10, STMN3, NEURL2, GNRH2, HTRA2, CD40, PCSK2, SNAI1, MAPK3, CASP7, EMX2, PKP2, HDAC7A, PTHLH, BDNF, PAX6, ERBB3, LRP1, RC3H1, CSDE1, INSRR, SEMA6C, PBXIP1, MYH7, SIX1, TITF1, NFKBIA, PPARD, FGF10, CTNND2, ANKH, DSCAML1, APP, SOD1, RBPJ, KLF3, MSX1, EVC, CCNF, METRN, CCBP2, MARK2, LY6H, TNFRSF11B, TPD52, KLF10, NRXN3, TNFAIP2, CYP46A1, ROBO3, FLI1, TMEM176B, PBX4, FKBP8, NTNG2, PRRX2, LMX1B, TLR4, LHX2, CNFN, NAPA, SIX5, LHB, KLK7, PAFAH1B3, SEMA7A, RGMA, ANGPTL2, NINJ1, KLF4, IRF4, EPM2A, FYN, BTD, KRT1, NTN4, CSRP2, WNT10B, PPP3CB, STMN1, SCMH1, TAL1, BMP8B, RBBP7, ARX, PITX2, UNC5C, MKX, ARNT2, ST6GAL1, GJA1, UNC5A, CANX, TLX3, PRELID1, TTN, NCKAP1, FUT8, NCOA4, BMPR1A, PLCE1, DMC1, LIF, PMP22, DLG4, TFAP2B, SMAP1, PPP2R5D, APLP1, CLPTM1, MEGF8, PPARA, NTRK2, COL2A1, CACNA1H, SERPINF1, MEF2B, ICK, QKI, ACAN, INSR, ASB1	4661, 5270, 1121, 91851, 27189, 84898, 2674, 64919, 6347, 3975, 6695, 51752, 7159, 2047, 1285, 8828, 27148, 27111, 4654, 9638, 84525, 3627, 50861, 140825, 2797, 27429, 958, 5126, 6615, 5595, 840, 2018, 5318, 51564, 5744, 627, 5080, 2065, 4035, 149041, 7812, 3645, 10500, 57326, 4625, 6495, 7080, 4792, 5467, 2255, 1501, 56172, 57453, 351, 6647, 3516, 51274, 4487, 2121, 899, 79006, 1238, 2011, 4062, 4982, 7163, 7071, 9369, 7127, 10858, 64221, 2313, 28959, 80714, 23770, 84628, 51450, 4010, 7099, 9355, 84518, 8775, 147912, 3972, 5650, 5050, 8482, 56963, 23452, 4814, 9314, 3662, 7957, 2534, 686, 3848, 59277, 1466, 7480, 5532, 3925, 22955, 6886, 656, 5931, 170302, 5308, 8633, 283078, 9915, 6480, 2697, 90249, 821, 30012, 27166, 7273, 10787, 2530, 8031, 657, 51196, 11144, 3976, 5376, 1742, 7021, 60682, 5528, 333, 1209, 1954, 5465, 4915, 1280, 8912, 5176, 4207, 22858, 9444, 176, 3643, 51665
cytoplasm organization and biogenesis	0.00398672	7	2.16	TLN1, GJC1, CD9, TNS1, GJA1, VCL, GJA7	7094, 125111, 928, 7145, 2697, 7414, 10052
protein amino acid glycosylation	0.00405573	21	11.34	TSPAN7, COG2, GALNT10, DDOST, MGAT1, ST6GALNAC2, ALG6, GYPC, GALNT9, ST8SIA5, DPM3, ALG8, ST3GAL6, STT3A, GALNT7, RPN1, MAN1A2, ST6GAL1, FUT8, MGAT2, FUT9	7102, 22796, 55568, 1650, 4245, 10610, 29929, 2995, 50614, 29906, 54344, 79053, 10402, 3703, 51809, 6184, 10905, 6480, 2530, 4247, 10690
transport	0.00437969	260	224.90	CDC37, MGC19604, CACNA1A, RANBP3, COL5A3, TIMM13, AP4E1, RAB8B, CACNA1G, PITPNC1, KCTD2, SLC25A43, SLC25A14, SNX8, TMED4, LCN8, OBP2B, KIF9, SLC26A6, ATP6V1G1, VPS13A, SLC16A2, KIF4A, ARF1, COG2, TRPM3, RASEF, APBA1, AQP7, LOC730908, CACNG6, FTL, RCP9, YKT6, DBNL, RAMP3, EXOC1, KIF17, GARS, SNX10, ABCA8, LYK5, HAP1, PACS1, ELMOD1, LRP4, SLC15A3, SLC29A2, MAP4K2, ATP6V1C2, KCNF1, SLC25A37, CHMP7, SCARA3, KIF13A, SLC17A2, SSR1, RAB24, SCP2, SLC5A9, RALBP1, HMG2L1, TSPO, TOMM22, MTX1, ATP1B1, ATP8B2, KIF5C, RPL11, PDPN, STX12, AQP5, SLC41A2, RAB35, KCNS2, CHMP4C, CYB5A, AZU1, HCN2, ADORA2A, ITSN2, CLN3, GTF3C1, GGA2, DOC2A, KCTD13, GRIN2A, SYT6, SLC26A4, CAV1, STX16, OSBPL2, ARFGAP1, GNAS, HSPD1, HDLBP, CHM, UPF3B, SYTL4, SLC10A3, CLCN4, SLC9A5, NUTF2, MRC1, SEC61A2, BNIP3, C10orf125, SLC6A2, ABCD4, AP2B1, RAMP2, SIL1, GLRX, SETD1B, ABCB10, SLC30A10, LOC728528, CYC1, SEC22A, COL4A3, ATG16L1, ACCN4, SMG7, KCTD3, SDCBP2, SYT9, DNHD1, SLC6A5, SLC17A6, G3BP2,	11140, 112812, 773, 8498, 50509, 26517, 23431, 51762, 8913, 26207, 23510, 203427, 9016, 29886, 222068, 138307, 29989, 64147, 65010, 9550, 23230, 6567, 24137, 375, 22796, 80036, 158158, 320, 364, 730908, 59285, 2512, 27297, 10652, 28988, 10268, 55763, 57576, 2617, 29887, 10351, 92335, 9001, 55690, 55531, 4038, 51296, 3177, 5871, 245973, 3754, 51312, 91782, 51435, 63971, 10246, 6745, 53917, 6342, 200010, 10928, 10042, 706, 56993, 4580, 481, 57198, 3800, 6135, 10630, 23673, 362, 84102, 11021, 3788, 92421, 1528, 566, 610, 135, 50618, 1201, 2975, 23062, 8448, 253980, 2903, 148281, 5172, 857, 8675, 9885, 55738, 2778, 3329, 3069, 1121, 65109, 94121, 8273, 1183, 6553, 10204, 4360, 55176, 664, 282969, 6530, 5826, 163, 10266, 64374, 2745, 23067, 23456, 55532, 728528, 1537, 26984, 1285,

				PLTP, RIMS4, SLC35C2, VPS16, RIN2, LBP, SLC24A3, PLLP, SLC12A4, ABCC11, SFXN2, GRIN2B, BDNF, NUP98, UEVLD, SLC17A8, SYT1, ZDHHC17, LRP1, SLC26A9, RAB7L1, KCNT2, TMEM9, SLC39A1, SLC8A3, CHMP4A, MYH7, ERO1L, STXBP6, NFKBIA, PPARD, SLC39A7, ITPR3, SLC25A30, TPT1, PARP4, FGF10, ANKH, TIMM8B, SLC37A4, APP, CLIC6, SLC34A2, CYBA, SLC7A5, TRAF7, PCOLN3, BAIAP3, HBA2, SLC25A38, MYRIP, KCTD6, SLC35C1, KCNK4, STX3, CBLB, LRP12, KIF26A, SDHD, SLC26A11, LOC652834, C1QTNF1, ABCG1, TMEM1, COL6A2, KLF15, COG5, SLC1A6, NUP62, NAPA, KCNN4, UACA, SELS, AP3S2, HCN4, AP3B2, VPS33B, RHCG, GRIN3A, FYN, AAAS, OSBPL8, SLC16A12, SLC25A28, GRIK3, SLC9A1, SH3KBP1, MFSD8, SRP14, ATP8B4, RAB27A, TUBAL3, STXBP3, C15orf27, HTR3E, STX11, GJA1, BNIP1, SLC40A1, AP4S1, SLC18A3, SLC25A1, TRPV1, MYBBP1A, SMG6, DNAH8, KCNQ5, APLP1, FXYD7, DNM2, SLC44A2, SLC25A42, PPARA, SLC18A2, COL2A1, SLC25A15, RYR3, RHOT2, CACNA1H, SLC16A5, RAB17, PACSIN2, QKI, RABGEF1, GJA3, CORO1A, GJA7, COL16A1, PDZK1, CENTG3	55054, 55515, 9887, 51133, 27111, 143425, 144132, 9152, 57084, 9908, 5360, 140730, 51006, 64601, 54453, 3929, 57419, 51090, 6560, 85320, 118980, 2904, 627, 4928, 55293, 246213, 6857, 23390, 4035, 115019, 8934, 343450, 252839, 27173, 6547, 29082, 4625, 30001, 29091, 4792, 5467, 7922, 3710, 253512, 7178, 143, 2255, 56172, 26521, 2542, 351, 54102, 10568, 1535, 8140, 84231, 5119, 8938, 3040, 54977, 25924, 200845, 55343, 50801, 6809, 868, 29967, 26153, 6392, 284129, 652834, 114897, 9619, 7109, 1292, 28999, 10466, 6511, 23636, 8775, 3783, 55075, 55829, 10239, 10021, 8120, 26276, 51458, 116443, 2534, 8086, 114882, 387700, 81894, 2899, 6548, 30011, 256471, 6727, 79895, 5873, 79861, 6814, 123591, 285242, 8676, 2697, 662, 30061, 11154, 6572, 6576, 7442, 10514, 23293, 1769, 56479, 333, 53822, 1785, 57153, 284439, 5465, 6571, 1280, 10166, 6263, 89941, 8912, 9121, 64284, 11252, 9444, 27342, 2700, 11151, 10052, 1307, 5174, 116988
intercellular junction assembly and maintenance	0.0046225	6	1.69	TLN1, GJC1, CD9, GJA1, VCL, GJA7	7094, 125111, 928, 2697, 7414, 10052
mitochondrial transport	0.00486142	11	4.59	TIMM13, SLC25A14, SLC25A37, TSPO, TOMM22, HSPD1, BNIP3, SLC25A30, TIMM8B, SLC25A1, SLC25A15	26517, 9016, 51312, 706, 56993, 3329, 664, 253512, 26521, 6576, 10166
mRNA processing	0.00522931	35	22.40	LSM7, TSEN34, U2AF2, SFRS1, SF3B2, YBX1, PTBP1, SF3A2, SNRPD3, RNPS1, DHX8, HNRPU, GRSF1, USP39, SNRPB2, SNRPF, NOVA1, APP, U2AF1, PAPOLA, ADARB1, DBR1, SRPK2, HNRPL, SNRPA1, WDR57, HNRPF, DDX20, RTCD1, PHF5A, APLP1, MORG1, SFRS5, QKI, HNRPA3	51690, 79042, 11338, 6426, 10992, 4904, 5725, 8175, 6634, 10921, 1659, 3192, 2926, 10713, 6629, 6636, 4857, 351, 7307, 10914, 104, 51163, 6733, 3191, 6627, 9410, 3185, 11218, 8634, 84844, 333, 84292, 6430, 9444, 220988
biopolymer glycosylation	0.00543135	21	11.62	TSPAN7, COG2, GALNT10, DDOST, MGAT1, ST6GALNAC2, ALG6, GYPC, GALNT9, ST8SIA5, DPM3, ALG8, ST3GAL6, STT3A, GALNT7, RPN1, MAN1A2, ST6GAL1, FUT8, MGAT2, FUT9	7102, 22796, 55568, 1650, 4245, 10610, 29929, 2995, 50614, 29906, 54344, 79053, 10402, 3703, 51809, 6184, 10905, 6480, 2530, 4247, 10690
mRNA metabolic process	0.00554499	40	26.52	LSM7, TSEN34, U2AF2, SFRS1, SF3B2, YBX1, SSB, PTBP1, SF3A2, SNRPD3, RNPS1, UPF3B, DHX8, HNRPU, SMG7, GRSF1, USP39, PCBP1, SNRPB2, SNRPF, NOVA1, APP, U2AF1, PAPOLA, ADARB1, DBR1, SRPK2, HNRPL, SNRPA1, WDR57, HNRPF, DDX20, RTCD1, PHF5A, SMG6, APLP1, MORG1, SFRS5, QKI, HNRPA3	51690, 79042, 11338, 6426, 10992, 4904, 6741, 5725, 8175, 6634, 10921, 65109, 1659, 3192, 9887, 2926, 10713, 5093, 6629, 6636, 4857, 351, 7307, 10914, 104, 51163, 6733, 3191, 6627, 9410, 3185, 11218, 8634, 84844, 23293, 333, 84292, 6430, 9444, 220988
protein metabolic process	0.00644761	348	310.64	MKNK2, LONP1, FARSA, CDC37, MAN2B1, CACNA1A, MATK, FBXW9, TIMM13, D4ST1, MAPK6, CDK3, ICT1, SLC25A43, MTMR1,	2872, 9361, 2193, 11140, 4125, 773, 4145, 84261, 26517, 113189, 5597, 1018, 3396, 203427, 8776,

				VBP1, RPL10, DUSP9, NGLY1, TSP50, TESSP5, LOC729280, LOC729752, LOC729756, HDAC6, MID1IP1, TSPAN7, UBE1, RP2, COG2, KIAA1804, GALNT10, EIF4EBP3, ChGn, LONRF1, FBXO16, BAG1, KIAA1815, TLN1, APBA1, GNAQ, RUVBL2, PRMT1, LIMK1, HSPA4L, USP48, DDOST, TNFRSF14, ANLN, GARS, JTV1, LYK5, GJC1, SERPINH1, CAPN5, PRSS23, CCND1, AIP, MRPS18A, MRPL14, PREP, SUGT1, PPP1CA, MAP4K2, PPM1B, FLJ21839, PTK2B, BMP1, FBXO25, FBXL6, ADAMDEC1, EIF4EBP1, RPL8, FLJ31951, MGAT1, AATK, LOC651771, RNF167, USP43, MIS12, ST6GALNAC2, ALG6, PTPRF, TRIOBP, FBXO7, CUL1, UBE3C, TARS2, DUSP27, MRPS21, GYPC, DNAJC10, ZAK, DPP10, GAD1, MYO3B, PPIG, RPL11, ATPIF1, DNAJC16, EPHA8, EPHB2, TSSK3, ELA2A, CD9, CAMKK2, GALNT9, SSH1, SULF1, SGK3, TRIB1, MRPL15, PTPN2, FBXO15, ST8SIA5, DUSP11, FBXO41, AZU1, CHAF1A, MAPK12, ROCK2, PPM1G, CLN3, GGA2, KCTD13, CPA5, CAV1, PTPRZ1, HSPD1, RPS7, ILKAP, SERPINE2, RNF25, CHM, UBL4A, CUL4B, CAMK1D, HSPA14, STYXL1, MTERF, FBXL19, CYLD, PHKG2, LOC650556, TRIP11, SUZ12, MAPK7, AP2B1, NMT1, NEK8, CCL2, SIL1, PDGFRB, FBXL17, IRF1, ARTS-1, P4HA2, PJA2, C12orf65, PARP1, PARP14, EPHB1, CHST13, BCS1L, STK36, UBE2F, CTSE, ADAM33, APBA2BP, MYOD1, GAK, COX18, NEURL2, CDC25B, PTPRA, USP39, HTRA2, CD40, PCSK2, LRRC29, MAPK3, PYDC1, ADAMTS18, PRSS36, CASP7, SLK, FBXL14, NUP98, TRIM5, UEVLD, ERBB3, SYT1, ESPL1, ZDHHC17, RIPK5, RFWD2, PIGM, APH1A, INSRR, DPM3, ERO1L, HMGA1, PFDN6, NEK3, UBL3, PARP4, RPS23, ALG8, TIMM8B, CCT8, APP, PIGP, ANAPC4, AGA, MGRN1, UBE2I, MMP25, TRAF7, PRSS21, STUB1, PH-4, PRKCD, RNF123, SPCS1, ST3GAL6, PPM1M, APEH, DAG1, MARK2, LOC649034, CAPN1, CBLB, EIF5, STT3A, CHEK1, GALNT7, SPCS3, C1QTNF1, TMEM1, D2HGDH, F10, RPN1, ANAPC13, TNIK, CNOT4, SRPK2, CALR3, PEPD, FKBP8, BRD4, TLR4, CDK9, KLK7, SELS, HERC1, ADAMTS17, CDC14B, TTLL11, PTPN3, FANCC, IRF4, EPM2A, FYN, MTRF1L, PPIL4, RIPK1, ROS1, ARPC4, TTLL3, HDAC11, VHL, UBE2E2, BBS10, KRT1, MAP3K12, PPP3CB, STMN1, PTP4A2, DNAJC8, UBE2D3, DMXL2, TUBAL3, MAN1A2, HIPK1, PKN2, CDC7, HS2ST1, RPLP1, CLK3, ST6GAL1, EPHB3, TIPARP, MME, IL1RAP, SENP5, EIF4A2, HSF2, TNFAIP3, MRPL18, GJA1, CANX, FGFR4, DPP4, TTN, WIPF1, FUT8, MGAT2, PRKCH, PPM1A, FAS, VCL, ADAMTS14, BMPR1A, TPST2, LIF, EIF3S7, ZZEF1, DLG4, ATPAF2, OPN5, FBXO9, HSP90AB1, KCNQ5, FUT9, MRPS12, APLP1, MRPL4, CARM1, RDX, NTRK2, MIB2, KIAA0317, USP50, RPS6, SHANK3, ULK4, ICK, QKI, RABGEF1, ACAN, USP31, GJA7, INSR, VRK3, MRPL19, ASB1, ADAM32, TMDCII	7411, 6134, 1852, 55768, 29122, 377047, 729280, 729752, 729756, 10013, 58526, 7102, 7317, 6102, 22796, 84451, 55568, 8637, 55790, 91694, 157574, 573, 79956, 7094, 320, 2776, 10856, 3276, 3984, 22824, 84196, 1650, 8764, 54443, 2617, 7965, 92335, 125111, 871, 726, 11098, 595, 9049, 55168, 64928, 5550, 10910, 5499, 5871, 5495, 60509, 2185, 649, 26260, 26233, 27299, 1978, 6132, 153830, 4245, 9625, 651771, 26001, 124739, 79003, 10610, 29929, 5792, 11078, 25793, 8454, 9690, 80222, 92235, 54460, 2995, 54431, 51776, 57628, 2571, 140469, 9360, 6135, 93974, 23341, 2046, 2048, 81629, 63036, 928, 10645, 50614, 54434, 23213, 23678, 10221, 29088, 5771, 201456, 29906, 8446, 150726, 566, 10036, 6300, 9475, 5496, 1201, 23062, 253980, 93979, 857, 5803, 3329, 6201, 80895, 5270, 64320, 1121, 8266, 8450, 57118, 51182, 51657, 7978, 54620, 1540, 5261, 650556, 9321, 23512, 5598, 163, 4836, 284086, 6347, 64374, 5159, 64839, 3659, 51752, 8974, 9867, 91574, 142, 54625, 2047, 166012, 617, 27148, 140739, 1510, 80332, 63941, 4654, 2580, 285521, 140825, 994, 5786, 10713, 27429, 958, 5126, 26231, 5595, 260434, 170692, 146547, 840, 9748, 144699, 4928, 85363, 55293, 2065, 6857, 9700, 23390, 25778, 64326, 93183, 51107, 3645, 54344, 30001, 3159, 10471, 4752, 5412, 143, 6228, 79053, 26521, 10694, 351, 51227, 29945, 175, 23295, 7329, 64386, 84231, 10942, 10273, 54681, 5580, 63891, 28972, 10402, 132160, 327, 1605, 2011, 649034, 823, 868, 1983, 3703, 1111, 51809, 60559, 114897, 7109, 728294, 2159, 6184, 25847, 23043, 4850, 6733, 125972, 5184, 23770, 23476, 7099, 1025, 5650, 55829, 8925, 170691, 8555, 158135, 5774, 2176, 3662, 7957, 2534, 54516, 85313, 8737, 6098, 10093, 26140, 79885, 7428, 7325, 79738, 3848, 7786, 5532, 3925, 8073, 22826, 7323, 23312, 79861, 10905, 204851, 5586, 8317, 9653, 6176, 1198, 6480, 2049, 25976, 4311, 3556, 205564, 1974, 3298, 7128, 29074, 2697, 821, 2264, 1803, 7273, 7456, 2530, 4247, 5583, 5494, 355, 7414, 140766, 657, 8459, 3976, 8664, 23140, 1742, 91647, 221391, 26268, 3326, 56479,

					10690, 6183, 333, 51073, 10498, 5962, 4915, 142678, 9870, 373509, 6194, 85358, 54986, 22858, 9444, 27342, 176, 57478, 10052, 3643, 51231, 9801, 51665, 203102, 255926
protein amino acid dephosphorylation	0.00655319	22	12.56	MTMR1, DUSP9, PPP1CA, PPM1B, PTPRF, DUSP27, SSH1, PTPN2, DUSP11, PPM1G, PTPRZ1, ILKAP, STYXL1, CDC25B, PTPRA, PPM1M, CDC14B, PTPN3, EPM2A, PPP3CB, PTP4A2, PPM1A	8776, 1852, 5499, 5495, 5792, 92235, 54434, 5771, 8446, 5496, 5803, 80895, 51657, 994, 5786, 132160, 8555, 5774, 7957, 5532, 8073, 5494
phosphatidylserine metabolic process	0.00709577	3	0.47	SERINC2, PTDSS2, SERINC5	347735, 81490, 256987
protein-cofactor linkage	0.00709577	3	0.47	GAD1, NMT1, ST3GAL6	2571, 4836, 10402
neuron remodeling	0.00709577	3	0.47	GNAQ, APP, NTN4	2776, 351, 59277
RNA splicing	0.00735015	31	19.68	LSM7, TSEN34, U2AF2, SFRS1, SF3B2, YBX1, PPIG, PTBP1, SF3A2, SNRPD3, RNPS1, DHX8, HNRPU, USP39, SNRPB2, SNRPF, NOVA1, U2AF1, QSER1, DBR1, SRPK2, SNRPA1, WDR57, HNRPF, DDX20, RTCD1, PHF5A, MORG1, SFRS5, QKI, HNRPA3	51690, 79042, 11338, 6426, 10992, 4904, 9360, 5725, 8175, 6634, 10921, 1659, 3192, 10713, 6629, 6636, 4857, 7307, 79832, 51163, 6733, 6627, 9410, 3185, 11218, 8634, 84844, 84292, 6430, 9444, 220988
establishment of cellular localization	0.0074358	82	62.97	CDC37, RANBP3, TIMM13, AP4E1, SLC25A14, KIF9, VPS13A, KIF4A, ARF1, COG2, APBA1, YKT6, RAMP3, KIF17, LYK5, HAP1, PACS1, CDCA5, MAP4K2, SLC25A37, KIF13A, SSR1, HMG2L1, TSPO, TOMM22, KIF5C, RPL11, STX12, RAB35, GGA2, STX16, ARFGAP1, GNAS, HSPD1, CHM, SYTL4, SEC61A2, BNIP3, AP2B1, RAMP2, SIL1, SEC22A, SMG7, SDCBP2, DNHD1, G3BP2, VPS16, NUP98, ESPL1, MYH7, NFKBIA, SLC25A30, FGF10, TIMM8B, APP, MYRIP, STX3, CBLB, KIF26A, TMEM1, COG5, NAPA, UACA, SELS, AP3S2, AP3B2, AAAS, SRP14, RAB27A, TUBAL3, STX11, BNIP1, AP4S1, UNC84B, SLC25A1, MYBBP1A, SMG6, DNAH8, SLC25A15, RHOT2, PACSIN2, CENTG3	11140, 8498, 26517, 23431, 9016, 64147, 23230, 24137, 375, 22796, 320, 10652, 10268, 57576, 92335, 9001, 55690, 113130, 5871, 51312, 63971, 6745, 10042, 706, 56993, 3800, 6135, 23673, 11021, 23062, 8675, 55738, 2778, 3329, 1121, 94121, 55176, 664, 163, 10266, 64374, 26984, 9887, 27111, 144132, 9908, 64601, 4928, 9700, 4625, 4792, 253512, 2255, 26521, 351, 25924, 6809, 868, 26153, 7109, 10466, 8775, 55075, 55829, 10239, 8120, 8086, 6727, 5873, 79861, 8676, 662, 11154, 25777, 6576, 10514, 23293, 1769, 10166, 89941, 11252, 116988
cellular localization	0.00804689	84	64.94	CDC37, RANBP3, TIMM13, AP4E1, SLC25A14, KIF9, VPS13A, KIF4A, ARF1, COG2, APBA1, YKT6, RAMP3, KIF17, LYK5, HAP1, PACS1, CDCA5, MAP4K2, SLC25A37, HOOK3, KIF13A, SSR1, HMG2L1, TSPO, TOMM22, KIF5C, RPL11, STX12, RAB35, GGA2, STX16, ARFGAP1, GNAS, HSPD1, CHM, SYTL4, SEC61A2, BNIP3, AP2B1, RAMP2, SIL1, SEC22A, SMG7, SDCBP2, DNHD1, COX18, G3BP2, VPS16, NUP98, ESPL1, MYH7, NFKBIA, SLC25A30, FGF10, TIMM8B, APP, MYRIP, STX3, CBLB, KIF26A, TMEM1, COG5, NAPA, UACA, SELS, AP3S2, AP3B2, AAAS, SRP14, RAB27A, TUBAL3, STX11, BNIP1, AP4S1, UNC84B, SLC25A1, MYBBP1A, SMG6, DNAH8, SLC25A15, RHOT2, PACSIN2, CENTG3	11140, 8498, 26517, 23431, 9016, 64147, 23230, 24137, 375, 22796, 320, 10652, 10268, 57576, 92335, 9001, 55690, 113130, 5871, 51312, 84376, 63971, 6745, 10042, 706, 56993, 3800, 6135, 23673, 11021, 23062, 8675, 55738, 2778, 3329, 1121, 94121, 55176, 664, 163, 10266, 64374, 26984, 9887, 27111, 144132, 285521, 9908, 64601, 4928, 9700, 4625, 4792, 253512, 2255, 26521, 351, 25924, 6809, 868, 26153, 7109, 10466, 8775, 55075, 55829, 10239, 8120, 8086, 6727, 5873, 79861, 8676, 662, 11154, 25777, 6576, 10514, 23293, 1769, 10166, 89941, 11252, 116988

protein deglycosylation	0.00877517	2	0.19	MAN2B1, AGA	4125, 175
positive regulation of interleukin-13 biosynthetic process	0.00877517	2	0.19	TLR4, IRF4	7099, 3662
positive regulation of interleukin-1 biosynthetic process	0.00877517	2	0.19	AZU1, TLR4	566, 7099
gap junction assembly	0.00877517	2	0.19	GJC1, GJA1	125111, 2697
heparan sulfate proteoglycan biosynthetic process, polysaccharide chain biosynthetic process	0.00877517	2	0.19	ChGn, HS2ST1	55790, 9653
regulation of interleukin-13 biosynthetic process	0.00877517	2	0.19	TLR4, IRF4	7099, 3662
regulation of interleukin-1 biosynthetic process	0.00877517	2	0.19	AZU1, TLR4	566, 7099
interleukin-1 biosynthetic process	0.00877517	2	0.19	AZU1, TLR4	566, 7099
dephosphorylation	0.00893632	24	14.43	MTMR1, DUSP9, PPP1CA, PPM1B, PTPRF, DUSP27, SSH1, PTPN2, DUSP11, PPM1G, PTPRZ1, ILKAP, STYXL1, SMG7, CDC25B, PTPRA, PPM1M, CDC14B, PTPN3, EPM2A, PPP3CB, PTP4A2, PPM1A, SMG6	8776, 1852, 5499, 5495, 5792, 92235, 54434, 5771, 8446, 5496, 5803, 80895, 51657, 9887, 994, 5786, 132160, 8555, 5774, 7957, 5532, 8073, 5494, 23293

GO category "molecular_function"

Number of genes annotated in GO: 1413
Number of significant GO groups found: 25

GO group	p-value	# genes (observed)	# genes (expected)	list of genes	GeneIDs
protein binding	2.88442e-06	619	539.02	MKNK2, LSM7, ZBTB7A, CDC37, TLE2, CACNA1A, MATK, RANBP3, SHC2, B2M, SPINT1, RAB8B, THBS1, D4ST1, MAPK6, ERBB2IP, XRCC4, DMXL1, RNF180, THBS4, F2RL1, CDK3, KCTD2, VBP1, BIRC4, GNA12, INTS1, SNX8, HOXA10, SH3GL2, SMARCA2, JMJD2C, SH2D3C, DOCK8, C9orf127, HACL1, IL5RA, NGLY1, ANP32B, CCIN, PGM5, OSTF1, HDAC6, FLJ21687, MID1IP1, UBE1, RP2, EFNB1, ARF1, COG2, KIAA1804, TGFBI, ABLIM3, PCDHA9, PCDHAC2, PCDHAC1, PCDHA13, PCDHA12, PCDHA11, PCDHA10, PCDHA8, PCDHA7, PCDHA5, PCDHA4, PCDHA3, PCDHA2, PCDHA1, SGCD, PHF15, EIF4EBP3, ANKHD1, ChGn, LONRF1, NKX3-1, RNF170, STC1, BAG1, CNTNAP3, TLN1, APBA1, PPFIA3, FTL, RUVBL2, LILRB1, PRMT1, U2AF2, PRRG2, LIMK1, DBNL, CCM2, SPRY1, PCDH10, ZNF330, CXCL6, CXCL1, LZIC, MAD2L2, E2F2, TNFRSF14, ANLN, AHR, GARS, ITGB8, SNX10, CHAD, RNF43, SDK2, LYK5, SFRS1, PHF12, GJC1, ACACA, HAP1, NUFIP2, SERPINH1, PACS1, SF3B2, SIPA1, CCND1, AIP, KHDRBS2, RP11-444E17.2, TRFP, IBTK, ATG5, CGA, BACH2, AKAP11, ITM2B, SHANK2, CDCA5, HSPC152, MAP4K2, RRM2, MSH6, KCNF1, GATA4, EPHX2, PLEC1, LOC652460, NRG1, PTK2B, BMP1, HOOK3, FBXL6, ADAMDEC1, EIF4EBP1, KBTBD11, MUTED, DEK, SERPINB9, NEDD9, PBX2, RXRB, TFAP2A, DOK3, FLJ31951, SUB1, LRRC48, RNF167, LGALS3BP, MIS12, TIMP2, C1orf94, LRRC42, SCP2, CDCA8, PTPRF, RLF, YBX1, FOXD3, SERPINB10, C18orf23, RALBP1, TRIOBP, CBY1, XRCC6, FBXO7, PARVG, BIK, ARHGEF5, CUL1, UBE3C, ZBTB7B, KIRREL, CREB3L4, GYPC, DNAJC10, TANK, ZAK, KLHL23, GAD1, MYO3B, MYO1B, KIF5C, RCC1, RPL11, EPB41, ATPIF1, DNAJC16, EPHB2, KLHDC5, CD9, SURB7, EMG1, CAMKK2, DIABLO, CLIP1, TBX5, SSH1, SGK3, TRIB1, KCNS2, ZFP161, PTPN2, DSC2, TCF4, FBXO15, SEMA4C, FBXO41, PTBP1, MADCAM1, ITGB1BP3, CHAF1A, PLXNB2, PHF21B, SNRPD3, MAPK12, ITSN2, HS1BP3, RBJ, TMEM8, CLN3, GGA2, KCTD13, SSX2IP, CAV1, MOSPD3, MYT1, HSPD1, TNS1, RPS7, PER2, RNF25, CHM, UPF3B, SYTL4, TMSB4X, PDZD4, CUL4B, GLA, KIAA0174, GAN, IL17C, NUTF2, MLLT10, BNIP3, VIM, CAMK1D, GFRA1, MCM7, BCL7B, CLDN3, FBXL19, MT1H, SLC6A2, CYLD, PHKG2, TRIP11, PPP1R13B, RHBDL3, FMNL1, GRB7, AP2B1, RARA, CCL2, LHX1, SIL1, TAF7, MCC, PDGFRB, HINT1, GLRX, ARTS-1, P4HA2, PJA2, HYPE, HCFC2, VWA1, PARP1, TP53BP2, RNF13, DTX3L, MCM2, EPHB1, COL4A3, ATG16L1, STK36, STK11IP, ATF3, SMG7, KCTD3, KLHL20, CTSE, SDCBP2,	2872, 51690, 51341, 11140, 7089, 773, 4145, 8498, 25759, 567, 6692, 51762, 7057, 113189, 5597, 55914, 7518, 1657, 285671, 7060, 2150, 1018, 23510, 7411, 331, 2768, 26173, 29886, 3206, 6456, 6595, 23081, 10044, 81704, 51754, 26061, 3568, 55768, 10541, 881, 5239, 26578, 10013, 79917, 58526, 7317, 6102, 1947, 375, 22796, 84451, 7045, 22885, 9752, 56134, 56135, 56136, 56137, 56138, 56139, 56140, 56141, 56143, 56144, 56145, 56146, 56147, 6444, 23338, 8637, 54882, 55790, 91694, 4824, 81790, 6781, 573, 79937, 7094, 320, 8541, 2512, 10856, 10859, 3276, 11338, 5639, 3984, 28988, 83605, 10252, 57575, 27309, 6372, 2919, 84328, 10459, 1870, 8764, 54443, 196, 2617, 3696, 29887, 1101, 54894, 54549, 92335, 6426, 57649, 125111, 31, 9001, 57532, 871, 55690, 10992, 6494, 595, 9049, 202559, 202500, 9477, 25998, 9474, 1081, 60468, 11215, 9445, 22941, 113130, 51504, 5871, 6241, 2956, 3754, 2626, 2053, 5339, 652460, 3084, 2185, 649, 84376, 26233, 27299, 1978, 9920, 63915, 7913, 5272, 4739, 5089, 6257, 7020, 79930, 153830, 10923, 83450, 26001, 3959, 79003, 7077, 84970, 115353, 6342, 55143, 5792, 6018, 4904, 27022, 5273, 147341, 10928, 11078, 25776, 2547, 25793, 64098, 638, 7984, 8454, 9690, 51043, 55243, 148327, 2995, 54431, 10010, 51776, 151230, 2571, 140469, 4430, 3800, 1104, 6135, 2035, 93974, 23341, 2048, 57542, 928, 9412, 10436, 10645, 56616, 6249, 6910, 54434, 23678, 10221, 3788, 7541, 5771, 1824, 6925, 201456, 54910, 150726, 5725, 8174, 27231, 10036, 23654, 112885, 6634, 6300, 50618, 64342, 51277, 58986, 1201, 23062, 253980, 117178, 857, 64598, 4661, 3329, 7145, 6201, 8864, 64320, 1121, 65109, 94121, 7114, 57595, 8450, 2717, 9798, 8139, 27189, 10204, 8028, 664, 7431, 57118, 2674, 4176, 9275, 1365, 54620, 4496, 6530, 1540, 5261, 9321, 23368, 162494, 752, 2886, 163,

				RASSF2, APBA2BP, E2F1, MYOD1, SYT9, PARVA, FEZ1, GAK, WDR1, CXCL10, CXCL9, CXCL3, CXCL5, NEURL2, JPH2, MATN4, GNRH2, SDC4, VPS16, DOK1, PCBP1, POLE4, HTRA2, SNRPB2, NCOA3, HM13, CD40, PCSK2, MPHOSPH6, MAPK3, FHOD1, CASP7, PDCD4, EMX2, ADRB1, PKP2, HDAC7A, PTHLH, PHB2, FBXL14, CHD4, MLF2, BDNF, CD59, NUP98, TRIM5, PAX6, ABTB2, IGFBP6, ERBB3, TARBP2, KRT18, SYT1, ESPL1, ZDHHC17, LRP1, RASSF3, CSRP1, RAB7L1, RC3H1, RFWD2, DEDD, APH1A, PYGO2, DPM3, PBXIP1, SLC8A3, CFL2, MYH7, SIX1, NOVA1, TITF1, NFKBIA, PPARD, HMGA1, PFDN6, ITPR3, PCDH9, LCP1, NEK3, PARP4, FGF10, CTNND2, CDH12, ZNF259, MAML2, CENTD2, ARRB1, DSCAML1, CCT8, APP, IFNGR2, U2AF1, ZNF295, TFF1, D4S234E, RBPJ, MSX1, MGRN1, UBE2I, CYBA, RNF166, TAF1C, TRAF7, PCOLN3, GNPTG, CLDN9, BAIAP3, STUB1, DNASE1, ZNF205, HBA2, ARL6, MYRIP, PRKCD, KCTD6, RNF123, APPL1, CCBP2, DAG1, MADD, MARK2, INCENP, CREB3L1, RASSF1, ACTR8, CBLB, TWF2, PDZRN3, LRP12, TNFRSF11B, TPD52, FAM84B, SLA, TRAF3, NRXN3, JDP2, C14orf166B, ROBO3, FOXRED1, IL10RA, CHEK1, CBX2, CD99, MX1, ABCG1, COL6A2, BOK, KLF15, PBEF1, CNOT4, COG5, PBX4, CALR3, CRLF1, GMIP, BRD4, LMX1B, TLR4, LRRC8A, CDK9, NUP62, STRN4, NAPA, KCNN4, SIX5, LHB, PAFAH1B3, UACA, SELS, BTBD1, NRG4, SNRPA1, LINGO1, ANGPTL2, GKAP1, EPB41L4B, PTPN3, FANCC, NINJ1, FBP1, ZBTB2, SHPRH, IRF4, EPM2A, FYN, RIPK1, FOXF2, ARPC4, MUC4, HDAC11, VHL, LMCD1, BBS10, KRT1, FMNL3, MAP3K12, ANXA11, PPP3CB, PIK3R3, MYOM3, STMN1, WDR57, DNAJC8, BMP8B, SH3KBP1, BCOR, RBBP7, PDZD11, PITX2, UBE2D3, UNC5C, MAD2L1, DMXL2, RAB27A, BCL2L10, TP53BP1, HNRPF, C10orf63, CTNNA3, HIPK1, DDX20, CDC7, TXNIP, MAGI3, LRRC49, RPLP1, CCDC33, ARNT2, TIPARP, IL1RAP, PSMD2, EIF4A2, HSF2, TPD52L1, STX11, TNFAIP3, GJA1, UNC5A, CANX, BNIP1, TLX3, TTN, CHN1, WIPF1, NCKAP1, SYNE2, LRRC9, NFATC4, PPM1A, NCOA4, FAS, VCL, BMPR1A, PLCE1, C10orf35, CLDN5, LIF, L3MBTL2, EIF3S7, UNC84B, CRK, TRPV1, MYBBP1A, ZZEF1, SMG6, GPS2, DLG4, CLDN7, ATPAF2, TFAP2B, POLR1C, HSP90AB1, PPP2R5D, KLHL32, LRRC1, BCKDHB, CAPNS1, APLP1, PVR, BTBD14B, SMARCA4, CRTC1, DNM2, CARM1, CNN1, RDX, RNF213, RTKN, PPARA, KLHL24, NTRK2, MIB2, PVRL1, LRRC43, BRCA2, SFRS5, MYO1F, RPS6, MEF2B, PACSIN2, SHANK3, RABGEF1, CORO1A, INSR, TNFRSF25, PDZK1, LOC652793, LOC728939, NSL1, SAG, MAML1, TRIM35, ADAM32	5914, 6347, 3975, 64374, 6879, 4163, 5159, 3094, 2745, 51752, 8974, 9867, 11153, 29915, 64856, 142, 7159, 11342, 151636, 4171, 2047, 1285, 55054, 27148, 114790, 467, 9887, 51133, 27252, 1510, 27111, 9770, 63941, 1869, 4654, 143425, 55742, 9638, 2580, 9948, 3627, 4283, 2921, 6374, 140825, 57158, 8785, 2797, 6385, 64601, 1796, 5093, 56655, 27429, 6629, 8202, 81502, 958, 5126, 10200, 5595, 29109, 840, 27250, 2018, 153, 5318, 51564, 5744, 11331, 144699, 1108, 8079, 627, 966, 4928, 85363, 5080, 25841, 3489, 2065, 6895, 3875, 6857, 9700, 23390, 4035, 283349, 1465, 8934, 149041, 64326, 9191, 51107, 90780, 54344, 57326, 6547, 1073, 4625, 6495, 4857, 7080, 4792, 5467, 3159, 10471, 3710, 5101, 3936, 4752, 143, 2255, 1501, 1010, 8882, 84441, 116985, 408, 57453, 10694, 351, 3460, 7307, 49854, 7031, 27065, 3516, 4487, 23295, 7329, 1535, 115992, 9013, 84231, 5119, 84572, 9080, 8938, 10273, 1773, 7755, 3040, 84100, 25924, 5580, 200845, 63891, 26060, 1238, 1605, 8567, 2011, 3619, 90993, 11186, 93973, 868, 11344, 23024, 29967, 4982, 7163, 157638, 6503, 7187, 9369, 122953, 145497, 64221, 55572, 3587, 1111, 84733, 4267, 4599, 9619, 1292, 666, 28999, 10135, 4850, 10466, 80714, 125972, 9244, 51291, 23476, 4010, 7099, 56262, 1025, 23636, 29888, 8775, 3783, 147912, 3972, 5050, 55075, 55829, 53339, 145957, 6627, 84894, 23452, 80318, 54566, 5774, 2176, 4814, 2203, 57621, 257218, 3662, 7957, 2534, 8737, 2295, 10093, 4585, 79885, 7428, 29995, 79738, 3848, 91010, 7786, 311, 5532, 8503, 127294, 3925, 9410, 22826, 656, 30011, 54880, 5931, 51248, 5308, 7323, 8633, 4085, 23312, 5873, 10017, 7158, 3185, 219670, 29119, 204851, 11218, 8317, 10628, 260425, 54839, 6176, 80125, 9915, 25976, 3556, 5708, 1974, 3298, 7164, 8676, 7128, 2697, 90249, 821, 662, 30012, 7273, 1123, 7456, 10787, 23224, 341883, 4776, 5494, 8031, 355, 7414, 657, 51196, 219738, 7122, 3976, 83746, 8664, 25777, 1398, 7442, 10514, 23140, 23293, 2874, 1742, 1366, 91647,

					7021, 9533, 3326, 5528, 114792, 55227, 594, 826, 333, 5817, 112939, 6597, 23373, 1785, 10498, 1264, 5962, 57674, 6242, 5465, 54800, 4915, 142678, 5818, 254050, 675, 6430, 4542, 6194, 4207, 11252, 85358, 27342, 11151, 3643, 8718, 5174, 652793, 728939, 25936, 6295, 9794, 23087, 203102
binding	1.91683e-05	1100	1035.29	MKNK2, RFX2, LSM7, LONP1, FARSA, ZBTB7A, CDC37, TLE2, MGC19604, MAN2B1, ZNF564, ZNF709, CACNA1A, MATK, ZNF442, RANBP3, SHC2, TIMM13, MLLT1, POLRMT, REXO1, AP4E1, B2M, SPINT1, RAB8B, THBS1, D4ST1, MAPK6, ERBB2IP, XRCC4, DMXL1, RNF180, THBS4, F2RL1, CACNA1G, EPX, CDK3, ICT1, AKAP1, SOX9, KCTD2, SLC25A43, ZIC3, VBP1, SLC25A14, BIRC4, ZFAND2A, GNA12, INTS1, SNX8, HOXA10, MYO1G, SETX, SH3GL2, CIZ1, SMARCA2, PTGES2, JMJD2C, SH2D3C, UCK1, DMRT1, DOCK8, C9orf127, LCN8, OBP2B, HACL1, KIF9, IL5RA, SLC26A6, NGLY1, ATP6V1G1, FOXE1, ANP32B, CCIN, PGM5, OSTF1, HDAC6, HNRPH2, FLJ21687, MID1IP1, UBE1, RP2, KIF4A, NUDT10, EFNB1, ARF1, COG2, KIAA1804, GALNT10, FOXI1, TGFBI, ABLIM3, PCDHA9, PCDHAC2, PCDHAC1, PCDHA13, PCDHA12, PCDHA11, PCDHA10, PCDHA8, PCDHA7, PCDHA5, PCDHA4, PCDHA3, PCDHA2, PCDHA1, RBM27, SGCD, PHF15, EIF4EBP3, ANKHD1, MASK-BP3, ChGn, LONRF1, ZNF395, NKX3-1, RNF170, STC1, TRPM3, BAG1, C9orf95, CNTNAP3, KIAA1815, TLN1, RASEF, APBA1, GNAQ, ZNF324, CACNG6, ZNF324B, PPFIA3, FTL, ZIK1, RUVBL2, LILRB1, PRMT1, U2AF2, PRRG2, LIMK1, DBNL, CCM2, SPRY1, HSPA4L, PCDH10, LPHN3, ZNF330, CXCL6, CXCL1, DCK, LZIC, MAD2L2, E2F2, TNFRSF14, DHRS3, KIF17, KIAA1706, EVX1, ANLN, AHR, GARS, ITGB8, SNX10, ZDHHC4, JTV1, RECQL5, CHAD, ABCA8, RNF43, SDK2, LYK5, SFRS1, HOXB4, PHF12, GJC1, ACACA, HAP1, ETV4, PLCD3, NUFIP2, SERPINH1, PACS1, SF3B2, NDUFV1, SIPA1, CCND1, AIP, CYP39A1, ASCC3, KHDRBS2, TFEB, RP11-444E17.2, B3GAT2, TRFP, IBTK, ATG5, GTPBP2, CGA, BACH2, RAP2A, AKAP11, SUGT1, ITM2B, NUDT8, B3GAT3, SHANK2, LRP4, CDCA5, PPP1CA, HSPC152, MAP4K2, PPM1B, MTA3, ATP6V1C2, RRM2, VSNL1, MSH6, GRHL1, KCNF1, GATA4, ZNF251, EPHX2, PLEC1, LOC652460, SLC25A37, NRG1, PTK2B, BMP1, PLEKHA2, HOOK3, FBXL6, ADAMDEC1, EIF4EBP1, RPL8, KBTBD11, MUTED, DEK, SERPINB9, KIF13A, NEDD9, PBX2, GMDS, SLC17A2, GNL1, RXRB, SSR1, TFAP2A, DOK3, GFPT2, RAB24, FLJ31951, SUB1, AATK, LOC651771, SIRT7, LRRC48, RNF167, LGALS3BP, MIS12, TIMP2, C1orf94, LRRC42, SCP2,	2872, 5990, 51690, 9361, 2193, 51341, 11140, 7089, 112812, 4125, 163050, 163051, 773, 4145, 79973, 8498, 25759, 26517, 4298, 5442, 57455, 23431, 567, 6692, 51762, 7057, 113189, 5597, 55914, 7518, 1657, 285671, 7060, 2150, 8913, 8288, 1018, 3396, 8165, 6662, 23510, 203427, 7547, 7411, 9016, 331, 90637, 2768, 26173, 29886, 3206, 64005, 23064, 6456, 25792, 6595, 80142, 23081, 10044, 83549, 1761, 81704, 51754, 138307, 29989, 26061, 64147, 3568, 65010, 55768, 9550, 2304, 10541, 881, 5239, 26578, 10013, 3188, 79917, 58526, 7317, 6102, 24137, 170685, 1947, 375, 22796, 84451, 55568, 2299, 7045, 22885, 9752, 56134, 56135, 56136, 56137, 56138, 56139, 56140, 56141, 56143, 56144, 56145, 56146, 56147, 54439, 6444, 23338, 8637, 54882, 404734, 55790, 91694, 55893, 4824, 81790, 6781, 80036, 573, 54981, 79937, 79956, 7094, 158158, 320, 2776, 25799, 59285, 388569, 8541, 2512, 284307, 10856, 10859, 3276, 11338, 5639, 3984, 28988, 83605, 10252, 22824, 57575, 23284, 27309, 6372, 2919, 1633, 84328, 10459, 1870, 8764, 9249, 57576, 80820, 2128, 54443, 196, 2617, 3696, 29887, 55146, 7965, 9400, 1101, 10351, 54894, 54549, 92335, 6426, 3214, 57649, 125111, 31, 9001, 2118, 113026, 57532, 871, 55690, 10992, 4723, 6494, 595, 9049, 51302, 10973, 202559, 7942, 202500, 135152, 9477, 25998, 9474, 54676, 1081, 60468, 5911, 11215, 10910, 9445, 254552, 26229, 22941, 4038, 113130, 5499, 51504, 5871, 5495, 57504, 245973, 6241, 7447, 2956, 29841, 3754, 2626, 90987, 2053, 5339, 652460, 51312, 3084, 2185, 649, 59339, 84376, 26233, 27299, 1978, 6132, 9920, 63915, 7913,

				CDCA8, PTPRF, AK3L1, B4GALT2, RLF, YBX1, FOXD3, SERPINB10, C18orf23, RALBP1, HMG2L1, TRIOBP, CBY1, XRCC6, FBXO7, TSPO, PARVG, ACO2, HMOX1, BIK, ARHGEF5, CUL1, UBE3C, ZBTB7B, KIRREL, TARS2, EFNA3, CA14, ATP1B1, ATP8B2, CREB3L4, GYPC, DNAJC10, TANK, ZAK, SSB, KLHL23, PHOSPHO2, GAD1, MYO3B, MYO1B, PPIG, INPP1, PMS1, KIF5C, RCC1, RPL11, EPB41, WDTC1, ZCCHC17, ATPIF1, DNAJC16, EFHD2, EPHA8, EPHB2, TSSK3, OPRD1, AEBP2, KLHDC5, CD9, SURB7, EMG1, CAMKK2, DIABLO, GALNT9, CLIP1, TBX5, HPD, MSI1, SSH1, RAB35, LHX5, CHD7, SULF1, SGK3, TRIB1, SOX17, KCNS2, ZFP161, MRCL3, NOL4, CYB5A, PTPN2, DSC2, TCF4, L3MBTL4, FBXO15, ESCO1, DUSP11, SEMA4C, FBXO41, SUCLG1, PTBP1, ZNF497, LENG9, AZU1, SF3A2, HCN2, MADCAM1, ITGB1BP3, CHAF1A, PLXNB2, PHF21B, SNRPD3, MAPK12, ITSN2, ROCK2, HS1BP3, RBJ, PPM1G, THUMPD2, TTC32, GP2, RNPS1, TMEM8, UMOD, ZKSCAN2, CLN3, GTF3C1, ADCY9, KIAA0430, N-PAC, GGA2, DOC2A, KCTD13, CDIPT, GRIN2A, SSX2IP, RHOC, DDAH1, SYT6, CPA5, ZNF277P, FLJ25778, SLC26A4, CAV1, PTPRZ1, MOSPD3, DLD, ARFGAP1, RBM38, GNAS, OGFR, MYT1, HSPD1, AAMP, TNS1, RPS7, PER2, HDLBP, GLB1L, ILKAP, ZNF142, SERPINE2, GTF3C3, RNF25, CHM, IDH3G, F8, UPF3B, SYTL4, TMSB4X, PRPS2, PDZD4, CUL4B, GLA, CLCN4, KIAA0174, GAN, IL17C, SLC9A5, NUTF2, CTBP2, MRC1, MRC1L1, MLLT10, HSPA12A, THNSL1, DHTKD1, BNIP3, GTPBP4, PFKP, VIM, CAMK1D, HSPA14, GFRA1, MCM7, CYP3A5, BCL7B, CALN1, CLDN3, MTERF, FBXL19, TNRC6A, MT1H, MT1P2, SLC6A2, CYLD, PHKG2, LOC650556, C14orf4, TRIP11, PPP1R13B, BCL11B, ALDH6A1, ABCD4, RHBDL3, SUZ12, FMNL1, RASL10B, MAPK7, DHX8, GRB7, AP2B1, NEK8, RARA, CCL2, LHX1, SIL1, TAF7, FBN2, MCC, PDGFRB, HINT1, CDO1, GLRX, IRF1, SPOCK1, ARTS-1, P4HA2, PJA2, OAS2, C12orf65, SETD1B, HYPE, HCFC2, ABCB10, HNRPU, VWA1, PARP1, TP53BP2, CYC1, ZNF696, RNF13, DTX3L, GTPBP8, MCM2, EPHB1, COL4A3, ATG16L1, BCS1L, STK36, STK11IP, DIS3L2, ACCN4, ATF3, SMG7, KCTD3, KLHL20, CTSE, PFKFB2, SDCBP2, ADAM33, RASSF2, APBA2BP, NANP, E2F1, MYOD1, SYT9, NUCB2, PARVA, IGSF9B, EFCAB4A, FEZ1, FLJ20273, GAK, CENTD1, HOP, GRSF1, LYAR, G3BP2, WDR1, CXCL10, CXCL9, CXCL3, CXCL5, PLTP, NEURL2, ZNF217, JPH2, MATN4, CTCFL, GNRH2, SDC4, VPS16, GM632, USP39, DOK1, NOTO, FLJ37440, PCBP1, POLE4, HTRA2, SNRPB2, NCOA3, ASXL1, HM13, CD40, LBP, PCSK2, ENTPD6, SNAI1, SLC24A3, MPHOSPH6, SLC12A4, MAPK3, FA2H, ADAMTS18, ABCC11, ZNF629, FHOD1, CASP7, SFXN2, SLK, MTG1, PDCD4, EMX2, PPRC1, ADRB1, PKP2,	5272, 63971, 4739, 5089, 2762, 10246, 2794, 6257, 6745, 7020, 79930, 9945, 53917, 153830, 10923, 9625, 651771, 51547, 83450, 26001, 3959, 79003, 7077, 84970, 115353, 6342, 55143, 5792, 205, 8704, 6018, 4904, 27022, 5273, 147341, 10928, 10042, 11078, 25776, 2547, 25793, 706, 64098, 50, 3162, 638, 7984, 8454, 9690, 51043, 55243, 80222, 1944, 23632, 481, 57198, 148327, 2995, 54431, 10010, 51776, 6741, 151230, 493911, 2571, 140469, 4430, 9360, 3628, 5378, 3800, 1104, 6135, 2035, 23038, 51538, 93974, 23341, 79180, 2046, 2048, 81629, 4985, 121536, 57542, 928, 9412, 10436, 10645, 56616, 50614, 6249, 6910, 3242, 4440, 54434, 11021, 64211, 55636, 23213, 23678, 10221, 64321, 3788, 7541, 10627, 8715, 1528, 5771, 1824, 6925, 91133, 201456, 114799, 8446, 54910, 150726, 8802, 5725, 162968, 94059, 566, 8175, 610, 8174, 27231, 10036, 23654, 112885, 6634, 6300, 50618, 9475, 64342, 51277, 5496, 80745, 130502, 2813, 10921, 58986, 7369, 342357, 1201, 2975, 115, 9665, 84656, 23062, 8448, 253980, 10423, 2903, 117178, 389, 23576, 148281, 93979, 11179, 254048, 5172, 857, 5803, 64598, 1738, 55738, 55544, 2778, 11054, 4661, 3329, 14, 7145, 6201, 8864, 3069, 79411, 80895, 7701, 5270, 9330, 64320, 1121, 3421, 2157, 65109, 94121, 7114, 5634, 57595, 8450, 2717, 1183, 9798, 8139, 27189, 6553, 10204, 1488, 4360, 414308, 8028, 259217, 79896, 55526, 664, 23560, 5214, 7431, 57118, 51182, 2674, 4176, 1577, 9275, 83698, 1365, 7978, 54620, 27327, 4496, 645745, 6530, 1540, 5261, 650556, 64207, 9321, 23368, 64919, 4329, 5826, 162494, 23512, 752, 91608, 5598, 1659, 2886, 163, 284086, 5914, 6347, 3975, 64374, 6879, 2201, 4163, 5159, 3094, 1036, 2745, 3659, 6695, 51752, 8974, 9867, 4939, 91574, 23067, 11153, 29915, 23456, 3192, 64856, 142, 7159, 1537, 79943, 11342, 151636, 29083, 4171, 2047, 1285, 55054, 617, 27148, 114790, 129563, 55515, 467, 9887, 51133, 27252, 1510, 5208, 27111, 80332, 9770, 63941, 140838,

				HDAC7A, PTHLH, PHB2, FBXL14, GRIN2B, CHD4, MLF2, BDNF,	1869, 4654, 143425, 4925, 55742, 22997,
				CD59, NUP98, TRIM5, PAX6, ABTB2, CYP2R1, IGFBP6, ERBB3,	283229, 9638, 54502, 2580, 116984, 84525,
				TARBP2, KRT18, SNRPF, SYT1, ESPL1, ZDHHC17, LRP1,	2926, 55646, 9908, 9948, 3627, 4283, 2921,
				RASSF3, CSRP1, RIPK5, RAB7L1, RC3H1, YOD1, RFWD2, DEDD,	6374, 5360, 140825, 7764, 57158, 8785,
				DDX59, F5, FLJ16478, APH1A, PYGO2, GBA, CSDE1, INSRR,	140690, 2797, 6385, 64601, 57473, 10713,
				SLC39A1, DPM3, PBXIP1, SLC8A3, DPF3, CFL2, CHMP4A,	1796, 344022, 129804, 5093, 56655, 27429,
				MDP-1, MYH7, SIX1, NOVA1, TITF1, NFKBIA, PYGL, PPARD,	6629, 8202, 171023, 81502, 958, 3929, 5126,
				CYP21A2, SLC39A7, HMGA1, PFDN6, ITPR3, SLC25A30,	955, 6615, 57419, 10200, 6560, 5595, 79152,
				RP11-125A7.3, PCDH9, DIS3, LCP1, NEK3, TPT1, KATNAL1,	170692, 85320, 23361, 29109, 840, 118980,
				PARP4, AYTL2, FGF10, CTNND2, ZNF622, RPS23, PDE4D,	9748, 92170, 27250, 2018, 23082, 153, 5318,
				CDH12, ZNF259, TIMM8B, PHOX2A, MAML2, TREH, CENTD2,	51564, 5744, 11331, 144699, 2904, 1108, 8079,
				ARRB1, DSCAML1, GUCY1A2, CCT8, APP, CLIC6, FTCD, IFNGR2,	627, 966, 4928, 85363, 5080, 25841, 120227,
				SOD1, MORC3, CBS, U2AF1, ZNF295, TFF1, D4S234E, RBPJ,	3489, 2065, 6895, 3875, 6636, 6857, 9700,
				SLC34A2, KLF3, MSX1, MGRN1, UBE2I, CYBA, MMP25, CA5A,	23390, 4035, 283349, 1465, 25778, 8934,
				RNF166, SLC7A5, UBN1, TAF1C, TRAF7, PCOLN3, GNPTG,	149041, 55432, 64326, 9191, 83479, 2153,
				CLDN9, PRSS21, PRDM7, BAIAP3, STUB1, DNASE1, ZNF205,	440695, 51107, 90780, 2629, 7812, 3645,
				HBA2, ARL6, RBM6, SLC25A38, MYRIP, PH-4, PRKCD, KCTD6,	27173, 54344, 57326, 6547, 8110, 1073, 29082,
				RNF123, PPM1M, APPL1, CCBP2, DAG1, MADD, MARK2,	145553, 4625, 6495, 4857, 7080, 4792, 5836,
				INCENP, NAT10, CREB3L1, RCN1, KCNK4, CAPN1, STIP1,	5467, 1589, 7922, 3159, 10471, 3710, 253512,
				RASSF1, ACTR8, CBLB, TWF2, PDZRN3, DNASE1L3, LY6H,	23078, 5101, 22894, 3936, 4752, 7178, 84056,
				CYP11B1, LRP12, TNFRSF11B, ZFAND1, RRM2B, TPD52,	143, 79888, 2255, 1501, 90441, 6228, 5144,
				FAM84B, KLF10, SLA, KIF26A, ADSSL1, PAPOLA, EIF5, TRAF3,	1010, 8882, 26521, 401, 84441, 11181, 116985,
				NRXN3, JDP2, C14orf166B, CYP46A1, ZNF410, ROBO3, MIZF,	408, 57453, 2977, 10694, 351, 54102, 10841,
				FOXRED1, BCDO2, IL10RA, SDHD, CHEK1, TBRG1, DDX25, FLI1,	3460, 6647, 23515, 875, 7307, 49854, 7031,
				GALNT7, CBX2, CD99, CD24, ZFY, MX1, ABCG1, TMEM1,	27065, 3516, 10568, 51274, 4487, 23295, 7329,
				COL6A2, ADARB1, BOK, F10, TXNDC6, KLF15, DBR1, RPN1,	1535, 64386, 763, 115992, 8140, 29855, 9013,
				C3orf25, TNIK, PBEF1, CNOT4, COG5, SRPK2, ZNF467, ZNF800,	84231, 5119, 84572, 9080, 10942, 11105, 8938,
				HNRPL, ZNF585B, ZNF585A, WDR87, PBX4, CALR3, ZNF780A,	10273, 1773, 7755, 3040, 84100, 10180, 54977,
				CRLF1, ZNF573, GMIP, ZNF599, PEPD, FKBP8, BRD4, NTNG2,	25924, 54681, 5580, 200845, 63891, 132160,
				PRRX2, FREQ, FLJ45224, FPGS, PTGS1, LMX1B, TLR4, LHX2,	26060, 1238, 1605, 8567, 2011, 3619, 55226,
				LRRC8A, CDK9, ZNF615, NUP62, STRN4, NAPA, KCNN4, C19orf7,	90993, 5954, 50801, 823, 10963, 11186, 93973,
				SIX5, LHB, CA11, LYPD4, ZNF228, PAFAH1B3, UACA, SELS,	868, 11344, 23024, 1776, 4062, 1584, 29967,
				LCTL, SEMA7A, HCN4, RGMA, ADAMTS17, AP3B2, BTBD1,	4982, 79752, 50484, 7163, 157638, 7071, 6503,
				NRG4, SNRPA1, LINGO1, ANGPTL2, GKAP1, EPB41L4B, PTPN3,	26153, 122622, 10914, 1983, 7187, 9369,
				FANCC, GRIN3A, NINJ1, KLF4, ZNF782, FBP1, ZBTB2, SHPRH,	122953, 145497, 10858, 57862, 64221, 25988,
				SOD2, IRF4, EPM2A, FYN, PLAGL1, MTRF1L, PPIL4, RIPK1,	55572, 83875, 3587, 6392, 1111, 84897, 29118,
				FOXF2, RNASET2, ROS1, ARPC4, MUC4, HDAC11, VHL, LMCD1,	2313, 51809, 84733, 4267, 934, 7544, 4599,
				BBS10, KRT1, FMNL3, CSRP2, MAP3K12, PANK1, ANXA11,	9619, 7109, 1292, 104, 666, 2159, 347736,
				PPP3CB, SLC25A28, ARL3, PIK3R3, MYOM3, STMN1, WDR57,	28999, 51163, 6184, 90288, 23043, 10135,
				RSPO1, DNAJC8, MOBKL2C, SCMH1, SLC9A1, TAL1, GPATCH3,	4850, 10466, 6733, 168544, 168850, 3191,
				BMP8B, SH3KBP1, BCOR, RBBP7, LMO6, ARX, NUDT11,	92285, 199704, 83889, 80714, 125972, 284323,
				PDZD11, PITX2, UBE2D3, PDE5A, SPARCL1, UNC5C, ARSJ,	9244, 126231, 51291, 148103, 5184, 23770,
				MAD2L1, DMXL2, SRP14, ATP8B4, RAB27A, BCL2L10, TP53BP1,	23476, 84628, 51450, 23413, 401562, 2356,
				MKX, TUBAL3, HNRPF, C10orf63, ZNF33B, CTNNA3, LARP5,	5742, 4010, 7099, 9355, 56262, 1025, 284370,

				MAN1A2, HIPK1, DDX20, LPHN2, PKN2, CDC7, RTCD1, TXNIP, MAGI3, LRRC49, NR2E3, PDE8A, IREB2, RPLP1, CCDC33, CLK3, ZNF774, ARNT2, ABCF3, EPHB3, TIPARP, ZNF639, MME, IL1RAP, HTR3E, PSMD2, EIF4A2, HSF2, TPD52L1, SYNJ2, SMOC2, STX11, TNFAIP3, GJA1, UNC5A, CANX, BNIP1, TLX3, FGFR4, TTN, PLA2R1, CHN1, WIPF1, NCKAP1, SLC40A1, SYNE2, LTB4R, LRRC9, NFATC4, PRKCH, PPM1A, NCOA4, STOX1, FAS, VCL, ADAMTS14, BMPR1A, PLCE1, ZNF33A, DDX50, GPR120, IFIT1L, BMS1L, C10orf35, CLDN5, THAP7, PHF5A, DMC1, LIF, DDX17, L3MBTL2, EIF3S7, GSCL, UNC84B, SLC25A1, CRK, TRPV1, MYBBP1A, ZZEF1, SREBF1, SMG6, GPS2, KRBA2, DLG4, CLDN7, ATPAF2, TFAP2B, DNAH8, SMAP1, POLR1C, C6orf49, HSP90AB1, PPP2R5D, KLHL32, KCNQ5, LRRC1, BCKDHB, CAPNS1, APLP1, PVR, CYP2F1, GAPDHS, MEGF8, BTBD14B, SMARCA4, CRTC1, DNM2, ZNF257, ZNF101, CYP4F12, ZNF763, LOC729745, SLC25A42, CARM1, CNN1, PLCH2, GPIAP1, RDX, PLA2G4D, ACOX1, RNF213, MC2R, RTKN, TNRC6B, PPARA, KLHL24, GTF2IRD2, NTRK2, ZNF711, MIB2, NKX1-2, DAK, PVRL1, LRRC43, BRCA2, SLC25A15, TGDS, SFRS5, BDKRB2, RYR3, RHOT2, CACNA1H, MYO1F, RPS6, MEF2B, RAB17, PRDM15, PACSIN2, SHANK3, ULK4, ZSWIM6, ICK, QKI, RABGEF1, KRBA1, ZNF658B, ZNF674, ACAN, CORO1A, INSR, VRK3, TNFRSF25, CASZ1, PDZK1, LOC652793, LOC728939, NSL1, HNRPA3, SAG, SUSD5, DKFZP686M0199, LOC730394, MAML1, HIVEP2, MGC87042, KIAA1549, ZNF783, CENTG3, TRIM35, ADAM32, ELAVL2	23636, 29888, 8775, 3783, 23211, 147912, 3972, 770, 147719, 7771, 5050, 55075, 55829, 197021, 8482, 10021, 56963, 170691, 8120, 53339, 145957, 6627, 84894, 23452, 80318, 54566, 5774, 2176, 116443, 4814, 9314, 158431, 2203, 57621, 257218, 6648, 3662, 7957, 2534, 5325, 54516, 85313, 8737, 2295, 8635, 6098, 10093, 4585, 79885, 7428, 29995, 79738, 3848, 91010, 1466, 7786, 53354, 311, 5532, 81894, 403, 8503, 127294, 3925, 9410, 284654, 22826, 148932, 22955, 6548, 6886, 63906, 656, 30011, 54880, 5931, 4007, 170302, 55190, 51248, 5308, 7323, 8654, 8404, 8633, 79642, 4085, 23312, 6727, 79895, 5873, 10017, 7158, 283078, 79861, 3185, 219670, 7582, 29119, 23185, 10905, 204851, 11218, 23266, 5586, 8317, 8634, 10628, 260425, 54839, 10002, 5151, 3658, 6176, 80125, 1198, 342132, 9915, 55324, 2049, 25976, 51193, 4311, 3556, 285242, 5708, 1974, 3298, 7164, 8871, 64094, 8676, 7128, 2697, 90249, 821, 662, 30012, 2264, 7273, 22925, 1123, 7456, 10787, 30061, 23224, 1241, 341883, 4776, 5583, 5494, 8031, 219736, 355, 7414, 140766, 657, 51196, 7581, 79009, 338557, 439996, 9790, 219738, 7122, 80764, 84844, 11144, 3976, 10521, 83746, 8664, 2928, 25777, 6576, 1398, 7442, 10514, 23140, 6720, 23293, 2874, 124751, 1742, 1366, 91647, 7021, 1769, 60682, 9533, 29964, 3326, 5528, 114792, 56479, 55227, 594, 826, 333, 5817, 1572, 26330, 1954, 112939, 6597, 23373, 1785, 113835, 94039, 66002, 284390, 729745, 284439, 10498, 1264, 9651, 4076, 5962, 283748, 51, 57674, 4158, 6242, 23112, 5465, 54800, 84163, 4915, 7552, 142678, 390010, 26007, 5818, 254050, 675, 10166, 23483, 6430, 624, 6263, 89941, 8912, 4542, 6194, 4207, 64284, 63977, 11252, 85358, 54986, 57688, 22858, 9444, 27342, 84626, 401509, 641339, 176, 11151, 3643, 51231, 8718, 54897, 5174, 652793, 728939, 25936, 220988, 6295, 26032, 653238, 730394, 9794, 3097, 256227, 57670, 155060, 116988, 23087, 203102, 1993

catalytic activity	0.00101808	523	470.06	MKNK2, LONP1, FARSA, MGC19604, MAN2B1, MATK, NDUFA11, POLRMT, REXO1, RAB8B, D4ST1, MAPK6, EPX, CDK3, MTMR1, DUSP9, GNA12, NDUFA4, SETX, SH3GL2, AGPAT2, SMARCA2, PTGES2, JMJD2C, UCK1, CCBL1, LCN8, METTL6, HACL1, AMT, NGLY1, TSP50, TESSP5, LOC729280, LOC729752, LOC729756, ATP6V1G1, PGM5, HDAC6, UBE1, RP2, NUDT10, ARF1, C1orf69, EPHX1, KIAA1804, GALNT10, C5orf14, RDHE2, ChGn, LONRF1, LYPLA1, C9orf95, KIAA1815, GBA2, GNAQ, FTL, RUVBL2, PRMT1, TSEN34, UPP1, LIMK1, YKT6, MAG1, CXCL6, DCK, USP48, DDOST, RAP1GAP, DHRS3, LFNG, C1GALT1, GARS, ZDHHC4, RECQL5, ABCA8, LYK5, ACACA, PLCD3, CAPN5, NDUFV1, PRSS23, DGAT2, CCND1, CYP39A1, ASCC3, HMGCLL1, B3GAT2, TRFP, IBTK, PREP, GTPBP2, GSTA1, RAP2A, NUDT8, B3GAT3, PPP1CA, MAP4K2, PPM1B, ATP6V1C2, IAH1, RRM2, MSH6, FLJ21839, EPHX2, PTK2B, BMP1, FBXO25, FBXL6, ADAMDEC1, TXNDC5, GMDS, RXRB, GFPT2, MGAT1, AATK, LOC651771, SIRT7, RNF167, USP43, PYCR1, ST6GALNAC2, ALG6, SCP2, PTPRF, AK3L1, B4GALT2, RALBP1, PIB5PA, XRCC6, FBXO7, ACO2, HMOX1, UBE3C, TARS2, DUSP27, EFNA3, CA14, ATP1B1, ATP8B2, ZAK, DPP10, PHOSPHO2, GAD1, MYO3B, PPIG, INPP1, H6PD, EPHA8, EPHB2, TSSK3, ELA2A, SURB7, CAMKK2, GALNT9, HPD, SSH1, RAB35, GLT8D2, CHD7, SULF1, SGK3, TRIB1, CYB5A, PTPN2, ST8SIA5, ESCO1, DUSP11, FLJ10081, SUCLG1, AZU1, ITGB1BP3, MAPK12, ROCK2, PPM1G, THUMPD2, ADCY9, N-PAC, CDIPT, LOC606495, RHOC, DDAH1, CPA5, METTL2B, PTPRZ1, DLD, GNAS, GLB1L, ILKAP, RNF25, CHM, IDH3G, F8, UCHL5IP, UBL4A, PRPS2, G6PD, GLA, CES2, CTBP2, THNSL1, DHTKD1, PFKP, CAMK1D, DHX32, NSUN5, MCM7, CYP3A5, STYXL1, FBXL19, CYLD, PHKG2, LOC650556, DEGS2, ALDH6A1, ACYP1, ABCD4, RHBDL3, SUZ12, MAPK7, DHX8, NMT1, NEK8, CCL2, PDGFRB, HINT1, CDO1, GLRX, ARTS-1, P4HA2, PJA2, OAS2, ALDH2, SETD8, DAO, ABCB10, PARP1, CYC1, PARP14, MCM2, EPHB1, CHST13, UMPS, BCS1L, NRP2, CTDSP1, STK36, SCLY, UBE2F, STK11IP, PNKD, DIS3L2, INPP5D, CTSE, SOAT1, PFKFB2, ADAM33, APBA2BP, NANP, IGSF9B, EFCAB4A, AMPD3, PTDSS2, GAK, CDC25B, CDS2, PTPRA, USP39, POLE4, HTRA2, NCOA3, HM13, PCSK2, ENTPD6, MAPK3, PYDC1, ADAMTS18, PRSS36, ABCC11, CASP7, SLK, PAOX, HDAC7A, CHD4, TRIM5, UEVLD, CYP2R1, ERBB3, ESPL1, ZDHHC17, ETNK2, RIPK5, RAB7L1, LPGAT1, RFWD2, GLUL, DDX59, F5, PIGM, GBA, INSRR, DHRS1, MDP-1, MYH7, ERO1L, PYGL, CYP21A2, HSD17B8, RP11-125A7.3, DIS3, NEK3, KATNAL1, PARP4, CCNU, AYTL2, AMACR, PDE4D, ALG8, TREH, GUCY1A2, CCT8, FTCD, PIGP, SOD1, CBS, ANAPC4, AGA, RBPJ,	2872, 9361, 2193, 112812, 4125, 4145, 126328, 5442, 57455, 51762, 113189, 5597, 8288, 1018, 8776, 1852, 2768, 4697, 23064, 6456, 10555, 6595, 80142, 23081, 83549, 883, 138307, 131965, 26061, 275, 55768, 29122, 377047, 729280, 729752, 729756, 9550, 5239, 10013, 7317, 6102, 170685, 375, 200205, 2052, 84451, 55568, 79770, 195814, 55790, 91694, 10434, 54981, 79956, 57704, 2776, 2512, 10856, 3276, 79042, 7378, 3984, 10652, 84803, 6372, 1633, 84196, 1650, 5909, 9249, 3955, 56913, 2617, 55146, 9400, 10351, 92335, 31, 113026, 726, 4723, 11098, 84649, 595, 51302, 10973, 54511, 135152, 9477, 25998, 5550, 54676, 2938, 5911, 254552, 26229, 5499, 5871, 5495, 245973, 285148, 6241, 2956, 60509, 2053, 2185, 649, 26260, 26233, 27299, 81567, 2762, 6257, 9945, 4245, 9625, 651771, 51547, 26001, 124739, 5831, 10610, 29929, 6342, 5792, 205, 8704, 10928, 27124, 2547, 25793, 50, 3162, 9690, 80222, 92235, 1944, 23632, 481, 57198, 51776, 57628, 493911, 2571, 140469, 9360, 3628, 9563, 2046, 2048, 81629, 63036, 9412, 10645, 50614, 3242, 54434, 11021, 83468, 55636, 23213, 23678, 10221, 1528, 5771, 29906, 114799, 8446, 55683, 8802, 566, 27231, 6300, 9475, 5496, 80745, 115, 84656, 10423, 606495, 389, 23576, 93979, 55798, 5803, 1738, 2778, 79411, 80895, 64320, 1121, 3421, 2157, 55559, 8266, 5634, 2539, 2717, 8824, 1488, 79896, 55526, 5214, 57118, 55760, 55695, 4176, 1577, 51657, 54620, 1540, 5261, 650556, 123099, 4329, 97, 5826, 162494, 23512, 5598, 1659, 4836, 284086, 6347, 5159, 3094, 1036, 2745, 51752, 8974, 9867, 4939, 217, 387893, 1610, 23456, 142, 1537, 54625, 4171, 2047, 166012, 7372, 617, 8828, 58190, 27148, 51540, 140739, 114790, 25953, 129563, 3635, 1510, 6646, 5208, 80332, 63941, 140838, 22997, 283229, 272, 81490, 2580, 994, 8760, 5786, 10713, 56655, 27429, 8202, 81502, 5126, 955, 5595, 260434, 170692, 146547, 85320, 840, 9748, 196743, 51564, 1108, 85363, 55293, 120227, 2065, 9700, 23390, 55224, 25778, 8934, 9926,

				MGRN1, UBE2I, CYBA, MMP25, CA5A, TRAF7, PCOLN3, PRSS21, STUB1, DNASE1, PH-4, PRKCD, RNF123, SPCS1, ST3GAL6, NIT2, PPM1M, APEH, QSER1, MARK2, NAT10, LOC649034, PRDX5, CAPN1, CBLB, GLT8D1, DNASE1L3, CYP11B1, RRM2B, ADSSL1, PAPOLA, EIF5, CYP46A1, FOXRED1, BCDO2, STT3A, SDHD, CHEK1, DDX25, GALNT7, SPCS3, FLJ21865, MX1, ABCG1, NDUFV3, TMEM1, ADARB1, D2HGDH, F10, TXNDC6, DBR1, MGLL, RPN1, TNIK, PBEF1, CNOT4, SRPK2, PEPD, FPGS, PTGS1, ADAMTSL2, LOC653348, PHYHD1, CDK9, NUP62, IL4I1, CA11, KLK7, PAFAH1B3, LCTL, HERC1, ADAMTS17, CDC14B, TTLL11, GKAP1, PTPN3, FBP1, SHPRH, SOD2, DDO, EPM2A, FYN, PPIL4, RIPK1, RNASET2, ROS1, TTLL3, BTD, HDAC11, UBE2E2, SETD5, SPRYD3, MAP3K12, PANK1, PPP1R3C, ALDH18A1, PPP3CB, PIK3R3, PTP4A2, RSPO1, MOBKL2C, SH3KBP1, NUDT11, UBE2D3, PDE5A, ARSJ, SPPL2A, ATP8B4, RAB27A, AGPAT7, TUBAL3, PBLD, MAN1A2, HIPK1, DDX20, PKN2, AHCYL1, CDC7, RTCD1, HS2ST1, MAGI3, NR2E3, PDE8A, IREB2, CLK3, ADAMTSL3, ABCF3, ST6GAL1, EPHB3, TIPARP, MME, SENP5, EIF4A2, SYNJ2, TNFAIP3, FGFR4, DPP4, TTN, FUT8, MGAT2, PRKCH, PPM1A, VCL, ADAMTS14, BMPR1A, PLCE1, DDX50, FOXRED2, DMC1, TPST2, DDX17, CARKL, MYBBP1A, SMG6, KRBA2, DSE, DNAH8, POLR1C, FBXO9, BCKDHB, FUT9, CAPNS1, CYP2F1, GAPDHS, SMARCA4, DNM2, CYP4F12, CARM1, PLCH2, C1orf31, PARG, PLA2G4D, ACOX1, RNF213, NTRK2, MIB2, DAK, BRCA2, TGDS, KIAA0317, BDKRB2, USP50, RHOT2, ENOSF1, SHANK3, ULK4, ICK, THSD4, USP31, INSR, VRK3, NSL1, MGC87042, ADAM32, TMDCII	64326, 2752, 83479, 2153, 93183, 2629, 3645, 115817, 145553, 4625, 30001, 5836, 1589, 7923, 23078, 22894, 4752, 84056, 143, 10309, 79888, 23600, 5144, 79053, 11181, 2977, 10694, 10841, 51227, 6647, 875, 29945, 175, 3516, 23295, 7329, 1535, 64386, 763, 84231, 5119, 10942, 10273, 1773, 54681, 5580, 63891, 28972, 10402, 56954, 132160, 327, 79832, 2011, 55226, 649034, 25824, 823, 868, 55830, 1776, 1584, 50484, 122622, 10914, 1983, 10858, 55572, 83875, 3703, 6392, 1111, 29118, 51809, 60559, 64772, 4599, 9619, 4731, 7109, 104, 728294, 2159, 347736, 51163, 11343, 6184, 23043, 10135, 4850, 6733, 5184, 2356, 5742, 9719, 653348, 254295, 1025, 23636, 259307, 770, 5650, 5050, 197021, 8925, 170691, 8555, 158135, 80318, 5774, 2203, 257218, 6648, 8528, 7957, 2534, 85313, 8737, 8635, 6098, 26140, 686, 79885, 7325, 55209, 84926, 7786, 53354, 5507, 5832, 5532, 8503, 8073, 284654, 148932, 30011, 55190, 7323, 8654, 79642, 84888, 79895, 5873, 254531, 79861, 64081, 10905, 204851, 11218, 5586, 10768, 8317, 8634, 9653, 260425, 10002, 5151, 3658, 1198, 57188, 55324, 6480, 2049, 25976, 4311, 205564, 1974, 8871, 7128, 2264, 1803, 7273, 2530, 4247, 5583, 5494, 7414, 140766, 657, 51196, 79009, 80020, 11144, 8459, 10521, 23729, 10514, 23293, 124751, 29940, 1769, 9533, 26268, 594, 10690, 826, 1572, 26330, 6597, 1785, 66002, 10498, 9651, 388753, 8505, 283748, 51, 57674, 4915, 142678, 26007, 675, 23483, 9870, 624, 373509, 89941, 55556, 85358, 54986, 22858, 79875, 57478, 3643, 51231, 25936, 256227, 203102, 255926
protein-tyrosine kinase activity	0.00128772	29	16.08	MATK, KIAA1804, LIMK1, PTK2B, AATK, LOC651771, EFNA3, ZAK, EPHA8, EPHB2, CAMKK2, NEK8, PDGFRB, EPHB1, NRP2, ERBB3, INSRR, NEK3, FYN, RIPK1, ROS1, SPRYD3, MAP3K12, CLK3, EPHB3, FGFR4, TTN, NTRK2, INSR	4145, 84451, 3984, 2185, 9625, 651771, 1944, 51776, 2046, 2048, 10645, 284086, 5159, 2047, 8828, 2065, 3645, 4752, 2534, 8737, 6098, 84926, 7786, 1198, 2049, 2264, 7273, 4915, 3643
iron ion binding	0.00168202	41	25.58	MGC19604, EPX, JMJD2C, FTL, NDUFV1, CYP39A1, PPP1CA, RRM2, SLC25A37, ACO2, HMOX1, HPD, CYB5A, CYP3A5, CDO1, P4HA2, CYC1, GAK, FA2H, SFXN2, CYP2R1, CYP21A2, APP,	112812, 8288, 23081, 2512, 4723, 51302, 5499, 6241, 51312, 50, 3162, 3242, 1528, 1577, 1036, 8974, 1537, 2580, 79152, 118980, 120227,

				CBS, CYBA, HBA2, PH-4, CYP11B1, RRM2B, CYP46A1, BCDO2, SDHD, PTGS1, PPP3CB, SLC25A28, RSPO1, IREB2, SLC40A1, CYP2F1, CYP4F12, MGC87042	1589, 351, 875, 1535, 3040, 54681, 1584, 50484, 10858, 83875, 6392, 5742, 5532, 81894, 284654, 3658, 30061, 1572, 66002, 256227
manganese ion binding	0.00224952	22	11.51	NUDT10, GALNT10, ACACA, B3GAT2, NUDT8, B3GAT3, PPP1CA, PPM1B, B4GALT2, GALNT9, PPM1G, CDIPT, ILKAP, PPM1M, GALNT7, PEPD, SOD2, NUDT11, PDE8A, PPM1A, BMPR1A, SMG6	170685, 55568, 31, 135152, 254552, 26229, 5499, 5495, 8704, 50614, 5496, 10423, 80895, 132160, 51809, 5184, 6648, 55190, 5151, 5494, 657, 23293
actin binding	0.00240682	39	24.48	INTS1, HDAC6, ABLIM3, TLN1, DBNL, ANLN, PLEC1, LOC652460, TRIOBP, PARVG, MYO3B, MYO1B, EPB41, SSH1, TNS1, TMSB4X, BCL7B, FMNL1, KLHL20, PARVA, WDR1, VPS16, FHOD1, CFL2, MYH7, LCP1, DNASE1, MYRIP, TWF2, ARPC4, FMNL3, WIPF1, SYNE2, VCL, CNN1, RDX, MIB2, MYO1F, CORO1A	26173, 10013, 22885, 7094, 28988, 54443, 5339, 652460, 11078, 64098, 140469, 4430, 2035, 54434, 7145, 7114, 9275, 752, 27252, 55742, 9948, 64601, 29109, 1073, 4625, 3936, 1773, 25924, 11344, 10093, 91010, 7456, 23224, 7414, 1264, 5962, 142678, 4542, 11151
protein serine/threonine phosphatase activity	0.00259259	10	3.65	MTMR1, PPP1CA, PPM1B, PPM1G, ILKAP, PPM1M, CDC14B, PPP1R3C, PPP3CB, PPM1A	8776, 5499, 5495, 5496, 80895, 132160, 8555, 5507, 5532, 5494
calcium ion binding	0.00320591	106	81.94	CACNA1A, THBS1, THBS4, CACNA1G, EPX, SLC26A6, GALNT10, PCDHA9, PCDHAC2, PCDHAC1, PCDHA13, PCDHA12, PCDHA11, PCDHA10, PCDHA8, PCDHA7, PCDHA5, PCDHA4, PCDHA3, PCDHA2, PCDHA1, TRPM3, RASEF, CACNG6, PRRG2, PCDH10, PLCD3, LRP4, VSNL1, BMP1, SSR1, HMG2L1, EFHD2, CAMKK2, GALNT9, SULF1, MRCL3, DSC2, ITSN2, UMOD, DOC2A, GRIN2A, SYT6, F8, MRC1, CAMK1D, CALN1, RHBDL3, FBN2, MCC, SPOCK1, APBA2BP, SYT9, NUCB2, EFCAB4A, FLJ37440, ENTPD6, SLC24A3, PTHLH, GRIN2B, SYT1, LRP1, F5, SLC8A3, ITPR3, PCDH9, LCP1, TPT1, AYTL2, CDH12, MMP25, TRAF7, DNASE1, PH-4, DAG1, RCN1, CAPN1, CBLB, DNASE1L3, TPD52, NRXN3, C14orf166B, GALNT7, F10, C3orf25, CALR3, FREQ, GRIN3A, ANXA11, PPP3CB, SPARCL1, ARSJ, MAN1A2, SMOC2, CANX, PLCE1, TRPV1, ZZEF1, CAPNS1, MEGF8, PLCH2, PLA2G4D, RYR3, RHOT2, CACNA1H, SAG	773, 7057, 7060, 8913, 8288, 65010, 55568, 9752, 56134, 56135, 56136, 56137, 56138, 56139, 56140, 56141, 56143, 56144, 56145, 56146, 56147, 80036, 158158, 59285, 5639, 57575, 113026, 4038, 7447, 649, 6745, 10042, 79180, 10645, 50614, 23213, 10627, 1824, 50618, 7369, 8448, 2903, 148281, 2157, 4360, 57118, 83698, 162494, 2201, 4163, 6695, 63941, 143425, 4925, 283229, 129804, 955, 57419, 5744, 2904, 6857, 4035, 2153, 6547, 3710, 5101, 3936, 7178, 79888, 1010, 64386, 84231, 1773, 54681, 1605, 5954, 823, 868, 1776, 7163, 9369, 145497, 51809, 2159, 90288, 125972, 23413, 116443, 311, 5532, 8404, 79642, 10905, 64094, 821, 51196, 7442, 23140, 826, 1954, 9651, 283748, 6263, 89941, 8912, 6295
cytoskeletal protein binding	0.00374067	52	35.72	INTS1, HDAC6, ABLIM3, TLN1, DBNL, ANLN, PLEC1, LOC652460, HOOK3, TRIOBP, PARVG, MYO3B, MYO1B, EPB41, CLIP1, SSH1, TNS1, TMSB4X, BCL7B, FMNL1, KLHL20, PARVA, FEZ1, WDR1, SDC4, VPS16, FHOD1, CFL2, MYH7, LCP1, DNASE1, MYRIP, TWF2, EPB41L4B, PTPN3, FYN, ARPC4, FMNL3, STMN1, RAB27A, TTN, WIPF1, SYNE2, VCL, UNC84B, DNM2, CNN1, RDX, MIB2, MYO1F, PACSIN2, CORO1A	26173, 10013, 22885, 7094, 28988, 54443, 5339, 652460, 84376, 11078, 64098, 140469, 4430, 2035, 6249, 54434, 7145, 7114, 9275, 752, 27252, 55742, 9638, 9948, 6385, 64601, 29109, 1073, 4625, 3936, 1773, 25924, 11344, 54566, 5774, 2534, 10093, 91010, 3925, 5873, 7273, 7456, 23224, 7414, 25777, 1785, 1264, 5962, 142678, 4542, 11252, 11151
cation binding	0.00410797	377	335.78	MKNK2, ZBTB7A, MGC19604, MAN2B1, ZNF564, ZNF709, CACNA1A, ZNF442, TIMM13, THBS1, RNF180, THBS4, CACNA1G,	2872, 51341, 112812, 4125, 163050, 163051, 773, 79973, 26517, 7057, 285671, 7060, 8913,

				EPX, ZIC3, BIRC4, ZFAND2A, CIZ1, JMJD2C, DMRT1, SLC26A6, NGLY1, HDAC6, NUDT10, GALNT10, ABLIM3, PCDHA9, PCDHAC2, PCDHAC1, PCDHA13, PCDHA12, PCDHA11, PCDHA10, PCDHA8, PCDHA7, PCDHA5, PCDHA4, PCDHA3, PCDHA2, PCDHA1, RBM27, PHF15, LONRF1, ZNF395, RNF170, TRPM3, KIAA1815, RASEF, ZNF324, CACNG6, ZNF324B, FTL, ZIK1, PRRG2, LIMK1, PCDH10, ZNF330, ZDHHC4, RNF43, PHF12, ACACA, PLCD3, NDUFV1, CYP39A1, B3GAT2, NUDT8, B3GAT3, LRP4, PPP1CA, PPM1B, MTA3, RRM2, VSNL1, KCNF1, GATA4, ZNF251, SLC25A37, BMP1, ADAMDEC1, SLC17A2, RXRB, SSR1, FLJ31951, SIRT7, RNF167, B4GALT2, RLF, C18orf23, HMG2L1, ACO2, HMOX1, ZBTB7B, CA14, ATP1B1, TANK, INPP1, ZCCHC17, EFHD2, AEBP2, CAMKK2, GALNT9, CLIP1, HPD, LHX5, SULF1, KCNS2, ZFP161, MRCL3, CYB5A, DSC2, L3MBTL4, ESCO1, FBXO41, ZNF497, LENG9, SF3A2, HCN2, PHF21B, ITSN2, ROCK2, PPM1G, UMOD, ZKSCAN2, DOC2A, CDIPT, GRIN2A, DDAH1, SYT6, CPA5, ZNF277P, PTPRZ1, ARFGAP1, MYT1, GLB1L, ILKAP, ZNF142, RNF25, F8, SYTL4, GLA, SLC9A5, MRC1, MLLT10, CAMK1D, CYP3A5, CALN1, FBXL19, MT1H, C14orf4, BCL11B, RHBDL3, SUZ12, RARA, LHX1, FBN2, MCC, HINT1, CDO1, SPOCK1, ARTS-1, P4HA2, PJA2, PARP1, CYC1, ZNF696, RNF13, DTX3L, MCM2, ACCN4, ADAM33, APBA2BP, SYT9, NUCB2, EFCAB4A, GAK, CENTD1, LYAR, ZNF217, CTCFL, GM632, USP39, FLJ37440, ASXL1, ENTPD6, SNAI1, SLC24A3, SLC12A4, FA2H, ADAMTS18, ZNF629, SFXN2, PTHLH, GRIN2B, CHD4, TRIM5, CYP2R1, SYT1, ZDHHC17, LRP1, CSRP1, RC3H1, YOD1, RFWD2, F5, PYGO2, GBA, SLC39A1, SLC8A3, DPF3, PPARD, CYP21A2, SLC39A7, ITPR3, PCDH9, LCP1, TPT1, AYTL2, ZNF622, CDH12, ZNF259, TIMM8B, CENTD2, APP, SOD1, MORC3, CBS, U2AF1, ZNF295, SLC34A2, KLF3, MGRN1, CYBA, MMP25, CA5A, RNF166, TRAF7, PCOLN3, PRDM7, DNASE1, ZNF205, HBA2, RBM6, MYRIP, PH-4, PRKCD, RNF123, PPM1M, DAG1, RCN1, KCNK4, CAPN1, RASSF1, CBLB, PDZRN3, DNASE1L3, CYP11B1, ZFAND1, RRM2B, TPD52, KLF10, TRAF3, NRXN3, C14orf166B, CYP46A1, ZNF410, MIZF, BCDO2, SDHD, GALNT7, ZFY, ADARB1, F10, KLF15, C3orf25, CNOT4, ZNF467, ZNF800, ZNF585B, ZNF585A, CALR3, ZNF780A, ZNF573, GMIP, ZNF599, PEPD, FREQ, PTGS1, LMX1B, LHX2, ZNF615, C19orf7, CA11, ZNF228, LCTL, HCN4, ADAMTS17, GRIN3A, KLF4, ZNF782, FBP1, ZBTB2, SHPRH, SOD2, PLAGL1, LMCD1, CSRP2, ANXA11, PPP3CB, SLC25A28, RSPO1, MOBKL2C, SLC9A1, LMO6, NUDT11, PDE5A, SPARCL1, ARSJ, ZNF33B, MAN1A2, NR2E3, PDE8A, IREB2, ZNF774, TIPARP, ZNF639, MME, SMOC2, TNFAIP3, CANX, CHN1, SLC40A1, PRKCH, PPM1A, ADAMTS14,	8288, 7547, 331, 90637, 25792, 23081, 1761, 65010, 55768, 10013, 170685, 55568, 22885, 9752, 56134, 56135, 56136, 56137, 56138, 56139, 56140, 56141, 56143, 56144, 56145, 56146, 56147, 54439, 23338, 91694, 55893, 81790, 80036, 79956, 158158, 25799, 59285, 388569, 2512, 284307, 5639, 3984, 57575, 27309, 55146, 54894, 57649, 31, 113026, 4723, 51302, 135152, 254552, 26229, 4038, 5499, 5495, 57504, 6241, 7447, 3754, 2626, 90987, 51312, 649, 27299, 10246, 6257, 6745, 153830, 51547, 26001, 8704, 6018, 147341, 10042, 50, 3162, 51043, 23632, 481, 10010, 3628, 51538, 79180, 121536, 10645, 50614, 6249, 3242, 64211, 23213, 3788, 7541, 10627, 1528, 1824, 91133, 114799, 150726, 162968, 94059, 8175, 610, 112885, 50618, 9475, 5496, 7369, 342357, 8448, 10423, 2903, 23576, 148281, 93979, 11179, 5803, 55738, 4661, 79411, 80895, 7701, 64320, 2157, 94121, 2717, 6553, 4360, 8028, 57118, 1577, 83698, 54620, 4496, 64207, 64919, 162494, 23512, 5914, 3975, 2201, 4163, 3094, 1036, 6695, 51752, 8974, 9867, 142, 1537, 79943, 11342, 151636, 4171, 55515, 80332, 63941, 143425, 4925, 283229, 2580, 116984, 55646, 7764, 140690, 57473, 10713, 129804, 171023, 955, 6615, 57419, 6560, 79152, 170692, 23361, 118980, 5744, 2904, 1108, 85363, 120227, 6857, 23390, 4035, 1465, 149041, 55432, 64326, 2153, 90780, 2629, 27173, 6547, 8110, 5467, 1589, 7922, 3710, 5101, 3936, 7178, 79888, 90441, 1010, 8882, 26521, 116985, 351, 6647, 23515, 875, 7307, 49854, 10568, 51274, 23295, 1535, 64386, 763, 115992, 84231, 5119, 11105, 1773, 7755, 3040, 10180, 25924, 54681, 5580, 63891, 132160, 1605, 5954, 50801, 823, 11186, 868, 23024, 1776, 1584, 79752, 50484, 7163, 7071, 7187, 9369, 145497, 10858, 57862, 25988, 83875, 6392, 51809, 7544, 104, 2159, 28999, 90288, 4850, 168544, 168850, 92285, 199704, 125972, 284323, 126231, 51291, 148103, 5184, 23413, 5742, 4010, 9355, 284370, 23211, 770, 7771, 197021, 10021, 170691, 116443, 9314, 158431,

				BMPR1A, PLCE1, ZNF33A, THAP7, L3MBTL2, TRPV1, ZZEF1, SMG6, SMAP1, C6orf49, KCNQ5, CAPNS1, APLP1, CYP2F1, MEGF8, ZNF257, ZNF101, CYP4F12, ZNF763, LOC729745, PLCH2, PLA2G4D, RNF213, PPARA, GTF2IRD2, ZNF711, MIB2, RYR3, RHOT2, CACNA1H, PRDM15, ZSWIM6, RABGEF1, ZNF658B, ZNF674, CASZ1, SAG, DKFZP686M0199, LOC730394, HIVEP2, MGC87042, CENTG3, TRIM35	2203, 57621, 257218, 6648, 5325, 29995, 1466, 311, 5532, 81894, 284654, 148932, 6548, 4007, 55190, 8654, 8404, 79642, 7582, 10905, 10002, 5151, 3658, 342132, 25976, 51193, 4311, 64094, 7128, 821, 1123, 30061, 5583, 5494, 140766, 657, 51196, 7581, 80764, 83746, 7442, 23140, 23293, 60682, 29964, 56479, 826, 333, 1572, 1954, 113835, 94039, 66002, 284390, 729745, 9651, 283748, 57674, 5465, 84163, 7552, 142678, 6263, 89941, 8912, 63977, 57688, 27342, 401509, 641339, 54897, 6295, 653238, 730394, 3097, 256227, 116988, 23087
metal ion binding	0.00456023	401	359.44	MKNK2, ZBTB7A, MGC19604, MAN2B1, ZNF564, ZNF709, CACNA1A, ZNF442, TIMM13, THBS1, RNF180, THBS4, CACNA1G, EPX, ZIC3, BIRC4, ZFAND2A, CIZ1, JMJD2C, DMRT1, HACL1, SLC26A6, NGLY1, ATP6V1G1, PGM5, HDAC6, NUDT10, GALNT10, ABLIM3, PCDHA9, PCDHAC2, PCDHAC1, PCDHA13, PCDHA12, PCDHA11, PCDHA10, PCDHA8, PCDHA7, PCDHA5, PCDHA4, PCDHA3, PCDHA2, PCDHA1, RBM27, PHF15, ChGn, LONRF1, ZNF395, RNF170, TRPM3, KIAA1815, RASEF, ZNF324, CACNG6, ZNF324B, FTL, ZIK1, PRRG2, LIMK1, PCDH10, ZNF330, ZDHHC4, RNF43, PHF12, ACACA, PLCD3, NDUFV1, CYP39A1, B3GAT2, NUDT8, B3GAT3, LRP4, PPP1CA, PPM1B, MTA3, RRM2, VSNL1, MSH6, KCNF1, GATA4, ZNF251, EPHX2, SLC25A37, BMP1, ADAMDEC1, SLC17A2, RXRB, SSR1, FLJ31951, SIRT7, RNF167, B4GALT2, RLF, C18orf23, HMG2L1, ACO2, HMOX1, ZBTB7B, CA14, ATP1B1, ATP8B2, TANK, ZAK, PHOSPHO2, INPP1, ZCCHC17, EFHD2, TSSK3, AEBP2, CAMKK2, GALNT9, CLIP1, HPD, LHX5, SULF1, KCNS2, ZFP161, MRCL3, CYB5A, DSC2, L3MBTL4, ESCO1, FBXO41, ZNF497, LENG9, SF3A2, HCN2, PHF21B, MAPK12, ITSN2, ROCK2, PPM1G, UMOD, ZKSCAN2, ADCY9, DOC2A, CDIPT, GRIN2A, DDAH1, SYT6, CPA5, ZNF277P, PTPRZ1, ARFGAP1, MYT1, ILKAP, ZNF142, RNF25, F8, SYTL4, PRPS2, SLC9A5, MRC1, MLLT10, PFKP, CAMK1D, CYP3A5, CALN1, FBXL19, MT1H, MT1P2, C14orf4, BCL11B, RHBDL3, SUZ12, NEK8, RARA, LHX1, FBN2, MCC, HINT1, CDO1, SPOCK1, ARTS-1, P4HA2, PJA2, PARP1, CYC1, ZNF696, RNF13, DTX3L, MCM2, STK36, ACCN4, ADAM33, APBA2BP, NANP, SYT9, NUCB2, EFCAB4A, GAK, CENTD1, LYAR, ZNF217, CTCFL, GM632, USP39, FLJ37440, ASXL1, ENTPD6, SNAI1, SLC24A3, SLC12A4, FA2H, ADAMTS18, ZNF629, SFXN2, PTHLH, GRIN2B, CHD4, TRIM5, CYP2R1, SYT1, ZDHHC17, LRP1, CSRP1, RC3H1, YOD1, RFWD2, F5, PYGO2, SLC39A1, SLC8A3,	2872, 51341, 112812, 4125, 163050, 163051, 773, 79973, 26517, 7057, 285671, 7060, 8913, 8288, 7547, 331, 90637, 25792, 23081, 1761, 26061, 65010, 55768, 9550, 5239, 10013, 170685, 55568, 22885, 9752, 56134, 56135, 56136, 56137, 56138, 56139, 56140, 56141, 56143, 56144, 56145, 56146, 56147, 54439, 23338, 55790, 91694, 55893, 81790, 80036, 79956, 158158, 25799, 59285, 388569, 2512, 284307, 5639, 3984, 57575, 27309, 55146, 54894, 57649, 31, 113026, 4723, 51302, 135152, 254552, 26229, 4038, 5499, 5495, 57504, 6241, 7447, 2956, 3754, 2626, 90987, 2053, 51312, 649, 27299, 10246, 6257, 6745, 153830, 51547, 26001, 8704, 6018, 147341, 10042, 50, 3162, 51043, 23632, 481, 57198, 10010, 51776, 493911, 3628, 51538, 79180, 81629, 121536, 10645, 50614, 6249, 3242, 64211, 23213, 3788, 7541, 10627, 1528, 1824, 91133, 114799, 150726, 162968, 94059, 8175, 610, 112885, 6300, 50618, 9475, 5496, 7369, 342357, 115, 8448, 10423, 2903, 23576, 148281, 93979, 11179, 5803, 55738, 4661, 80895, 7701, 64320, 2157, 94121, 5634, 6553, 4360, 8028, 5214, 57118, 1577, 83698, 54620, 4496, 645745, 64207, 64919, 162494, 23512, 284086, 5914, 3975, 2201, 4163, 3094, 1036, 6695, 51752, 8974, 9867, 142, 1537, 79943, 11342, 151636, 4171, 27148, 55515, 80332, 63941, 140838, 143425, 4925, 283229, 2580, 116984, 55646, 7764, 140690, 57473, 10713,

				DPF3, MDP-1, PPARD, CYP21A2, SLC39A7, ITPR3, PCDH9, LCP1, NEK3, TPT1, AYTL2, ZNF622, PDE4D, CDH12, ZNF259, TIMM8B, CENTD2, APP, SOD1, MORC3, CBS, U2AF1, ZNF295, SLC34A2, KLF3, MGRN1, CYBA, MMP25, CA5A, RNF166, TRAF7, PCOLN3, PRDM7, DNASE1, ZNF205, HBA2, RBM6, MYRIP, PH-4, PRKCD, RNF123, PPM1M, DAG1, MARK2, RCN1, KCNK4, CAPN1, RASSF1, CBLB, PDZRN3, DNASE1L3, CYP11B1, ZFAND1, RRM2B, TPD52, KLF10, ADSSL1, TRAF3, NRXN3, C14orf166B, CYP46A1, ZNF410, MIZF, BCDO2, SDHD, GALNT7, ZFY, ADARB1, F10, KLF15, DBR1, C3orf25, CNOT4, SRPK2, ZNF467, ZNF800, ZNF585B, ZNF585A, CALR3, ZNF780A, ZNF573, GMIP, ZNF599, PEPD, FREQ, PTGS1, LMX1B, LHX2, ZNF615, C19orf7, CA11, ZNF228, HCN4, ADAMTS17, GRIN3A, KLF4, ZNF782, FBP1, ZBTB2, SHPRH, SOD2, PLAGL1, LMCD1, CSRP2, MAP3K12, ANXA11, PPP3CB, SLC25A28, RSPO1, MOBKL2C, SLC9A1, LMO6, NUDT11, PDE5A, SPARCL1, ARSJ, ATP8B4, ZNF33B, MAN1A2, NR2E3, PDE8A, IREB2, ZNF774, TIPARP, ZNF639, MME, SMOC2, TNFAIP3, CANX, CHN1, SLC40A1, PRKCH, PPM1A, ADAMTS14, BMPR1A, PLCE1, ZNF33A, THAP7, L3MBTL2, TRPV1, ZZEF1, SMG6, SMAP1, C6orf49, KCNQ5, CAPNS1, APLP1, CYP2F1, MEGF8, ZNF257, ZNF101, CYP4F12, ZNF763, LOC729745, PLCH2, PLA2G4D, RNF213, PPARA, GTF2IRD2, ZNF711, MIB2, RYR3, RHOT2, CACNA1H, PRDM15, ZSWIM6, ICK, RABGEF1, ZNF658B, ZNF674, CASZ1, SAG, DKFZP686M0199, LOC730394, HIVEP2, MGC87042, CENTG3, TRIM35	129804, 171023, 955, 6615, 57419, 6560, 79152, 170692, 23361, 118980, 5744, 2904, 1108, 85363, 120227, 6857, 23390, 4035, 1465, 149041, 55432, 64326, 2153, 90780, 27173, 6547, 8110, 145553, 5467, 1589, 7922, 3710, 5101, 3936, 4752, 7178, 79888, 90441, 5144, 1010, 8882, 26521, 116985, 351, 6647, 23515, 875, 7307, 49854, 10568, 51274, 23295, 1535, 64386, 763, 115992, 84231, 5119, 11105, 1773, 7755, 3040, 10180, 25924, 54681, 5580, 63891, 132160, 1605, 2011, 5954, 50801, 823, 11186, 868, 23024, 1776, 1584, 79752, 50484, 7163, 7071, 122622, 7187, 9369, 145497, 10858, 57862, 25988, 83875, 6392, 51809, 7544, 104, 2159, 28999, 51163, 90288, 4850, 6733, 168544, 168850, 92285, 199704, 125972, 284323, 126231, 51291, 148103, 5184, 23413, 5742, 4010, 9355, 284370, 23211, 770, 7771, 10021, 170691, 116443, 9314, 158431, 2203, 57621, 257218, 6648, 5325, 29995, 1466, 7786, 311, 5532, 81894, 284654, 148932, 6548, 4007, 55190, 8654, 8404, 79642, 79895, 7582, 10905, 10002, 5151, 3658, 342132, 25976, 51193, 4311, 64094, 7128, 821, 1123, 30061, 5583, 5494, 140766, 657, 51196, 7581, 80764, 83746, 7442, 23140, 23293, 60682, 29964, 56479, 826, 333, 1572, 1954, 113835, 94039, 66002, 284390, 729745, 9651, 283748, 57674, 5465, 84163, 7552, 142678, 6263, 89941, 8912, 63977, 57688, 22858, 27342, 401509, 641339, 54897, 6295, 653238, 730394, 3097, 256227, 116988, 23087
ion binding	0.00531883	408	367.02	MKNK2, ZBTB7A, MGC19604, MAN2B1, ZNF564, ZNF709, CACNA1A, ZNF442, TIMM13, THBS1, RNF180, THBS4, CACNA1G, EPX, ZIC3, BIRC4, ZFAND2A, CIZ1, JMJD2C, DMRT1, HACL1, SLC26A6, NGLY1, ATP6V1G1, PGM5, HDAC6, NUDT10, GALNT10, ABLIM3, PCDHA9, PCDHAC2, PCDHAC1, PCDHA13, PCDHA12, PCDHA11, PCDHA10, PCDHA8, PCDHA7, PCDHA5, PCDHA4, PCDHA3, PCDHA2, PCDHA1, RBM27, PHF15, ChGn, LONRF1, ZNF395, RNF170, TRPM3, KIAA1815, RASEF, ZNF324, CACNG6, ZNF324B, FTL, ZIK1, PRRG2, LIMK1, PCDH10, ZNF330, ZDHHC4, RNF43, PHF12, ACACA, PLCD3, NDUFV1, CYP39A1, B3GAT2, NUDT8, B3GAT3, LRP4, PPP1CA, PPM1B, MTA3, RRM2,	2872, 51341, 112812, 4125, 163050, 163051, 773, 79973, 26517, 7057, 285671, 7060, 8913, 8288, 7547, 331, 90637, 25792, 23081, 1761, 26061, 65010, 55768, 9550, 5239, 10013, 170685, 55568, 22885, 9752, 56134, 56135, 56136, 56137, 56138, 56139, 56140, 56141, 56143, 56144, 56145, 56146, 56147, 54439, 23338, 55790, 91694, 55893, 81790, 80036, 79956, 158158, 25799, 59285, 388569, 2512, 284307, 5639, 3984, 57575, 27309, 55146, 54894, 57649, 31, 113026, 4723, 51302,

				VSNL1, MSH6, KCNF1, GATA4, ZNF251, EPHX2, SLC25A37,	135152, 254552, 26229, 4038, 5499, 5495,
				BMP1, ADAMDEC1, SLC17A2, RXRB, SSR1, FLJ31951, SIRT7,	57504, 6241, 7447, 2956, 3754, 2626, 90987,
				RNF167, B4GALT2, RLF, C18orf23, HMG2L1, ACO2, HMOX1,	2053, 51312, 649, 27299, 10246, 6257, 6745,
				ZBTB7B, CA14, ATP1B1, ATP8B2, TANK, ZAK, PHOSPHO2,	153830, 51547, 26001, 8704, 6018, 147341,
				INPP1, ZCCHC17, EFHD2, TSSK3, AEBP2, CAMKK2, GALNT9,	10042, 50, 3162, 51043, 23632, 481, 57198,
				CLIP1, HPD, LHX5, SULF1, KCNS2, ZFP161, MRCL3, CYB5A,	10010, 51776, 493911, 3628, 51538, 79180,
				DSC2, L3MBTL4, ESCO1, FBXO41, ZNF497, LENG9, SF3A2,	81629, 121536, 10645, 50614, 6249, 3242,
				HCN2, PHF21B, MAPK12, ITSN2, ROCK2, PPM1G, UMOD,	64211, 23213, 3788, 7541, 10627, 1528, 1824,
				ZKSCAN2, ADCY9, DOC2A, CDIPT, GRIN2A, DDAH1, SYT6,	91133, 114799, 150726, 162968, 94059, 8175,
				CPA5, ZNF277P, SLC26A4, PTPRZ1, ARFGAP1, MYT1, GLB1L,	610, 112885, 6300, 50618, 9475, 5496, 7369,
				ILKAP, ZNF142, RNF25, F8, SYTL4, PRPS2, GLA, CLCN4,	342357, 115, 8448, 10423, 2903, 23576,
				SLC9A5, MRC1, MLLT10, PFKP, CAMK1D, CYP3A5, CALN1,	148281, 93979, 11179, 5172, 5803, 55738,
				FBXL19, MT1H, MT1P2, C14orf4, BCL11B, RHBDL3, SUZ12, NEK8,	4661, 79411, 80895, 7701, 64320, 2157, 94121,
				RARA, LHX1, FBN2, MCC, HINT1, CDO1, SPOCK1, ARTS-1,	5634, 2717, 1183, 6553, 4360, 8028, 5214,
				P4HA2, PJA2, PARP1, CYC1, ZNF696, RNF13, DTX3L, MCM2,	57118, 1577, 83698, 54620, 4496, 645745,
				STK36, ACCN4, ADAM33, APBA2BP, NANP, SYT9, NUCB2,	64207, 64919, 162494, 23512, 284086, 5914,
				EFCAB4A, GAK, CENTD1, LYAR, ZNF217, CTCFL, GM632,	3975, 2201, 4163, 3094, 1036, 6695, 51752,
				USP39, FLJ37440, ASXL1, ENTPD6, SNAI1, SLC24A3, SLC12A4,	8974, 9867, 142, 1537, 79943, 11342, 151636,
				FA2H, ADAMTS18, ZNF629, SFXN2, PTHLH, GRIN2B, CHD4,	4171, 27148, 55515, 80332, 63941, 140838,
				TRIM5, CYP2R1, SYT1, ZDHHC17, LRP1, CSRP1, RC3H1, YOD1,	143425, 4925, 283229, 2580, 116984, 55646,
				RFWD2, F5, PYGO2, GBA, SLC39A1, SLC8A3, DPF3, MDP-1,	7764, 140690, 57473, 10713, 129804, 171023,
				PPARD, CYP21A2, SLC39A7, ITPR3, PCDH9, LCP1, NEK3, TPT1,	955, 6615, 57419, 6560, 79152, 170692, 23361,
				AYTL2, ZNF622, PDE4D, CDH12, ZNF259, TIMM8B, CENTD2,	118980, 5744, 2904, 1108, 85363, 120227,
				APP, CLIC6, SOD1, MORC3, CBS, U2AF1, ZNF295, SLC34A2,	6857, 23390, 4035, 1465, 149041, 55432,
				KLF3, MGRN1, CYBA, MMP25, CA5A, RNF166, TRAF7, PCOLN3,	64326, 2153, 90780, 2629, 27173, 6547, 8110,
				PRDM7, DNASE1, ZNF205, HBA2, RBM6, MYRIP, PH-4, PRKCD,	145553, 5467, 1589, 7922, 3710, 5101, 3936,
				RNF123, PPM1M, DAG1, MARK2, RCN1, KCNK4, CAPN1,	4752, 7178, 79888, 90441, 5144, 1010, 8882,
				RASSF1, CBLB, PDZRN3, DNASE1L3, CYP11B1, ZFAND1,	26521, 116985, 351, 54102, 6647, 23515, 875,
				RRM2B, TPD52, KLF10, ADSSL1, TRAF3, NRXN3, C14orf166B,	7307, 49854, 10568, 51274, 23295, 1535,
				CYP46A1, ZNF410, MIZF, BCDO2, SDHD, GALNT7, ZFY, ADARB1,	64386, 763, 115992, 84231, 5119, 11105, 1773,
				F10, KLF15, DBR1, C3orf25, CNOT4, SRPK2, ZNF467, ZNF800,	7755, 3040, 10180, 25924, 54681, 5580, 63891,
				ZNF585B, ZNF585A, CALR3, ZNF780A, ZNF573, GMIP, ZNF599,	132160, 1605, 2011, 5954, 50801, 823, 11186,
				PEPD, FREQ, PTGS1, LMX1B, LHX2, ZNF615, C19orf7, CA11,	868, 23024, 1776, 1584, 79752, 50484, 7163,
				ZNF228, LCTL, HCN4, ADAMTS17, GRIN3A, KLF4, ZNF782, FBP1,	7071, 122622, 7187, 9369, 145497, 10858,
				ZBTB2, SHPRH, SOD2, PLAGL1, LMCD1, CSRP2, MAP3K12,	57862, 25988, 83875, 6392, 51809, 7544, 104,
				ANXA11, PPP3CB, SLC25A28, RSPO1, MOBKL2C, SLC9A1,	2159, 28999, 51163, 90288, 4850, 6733,
				LMO6, NUDT11, PDE5A, SPARCL1, ARSJ, ATP8B4, ZNF33B,	168544, 168850, 92285, 199704, 125972,
				MAN1A2, NR2E3, PDE8A, IREB2, ZNF774, TIPARP, ZNF639,	284323, 126231, 51291, 148103, 5184, 23413,
				MME, SMOC2, TNFAIP3, CANX, CHN1, SLC40A1, PRKCH,	5742, 4010, 9355, 284370, 23211, 770, 7771,
				PPM1A, ADAMTS14, BMPR1A, PLCE1, ZNF33A, THAP7,	197021, 10021, 170691, 116443, 9314, 158431,
				L3MBTL2, TRPV1, ZZEF1, SMG6, SMAP1, C6orf49, KCNQ5,	2203, 57621, 257218, 6648, 5325, 29995, 1466,
				CAPNS1, APLP1, CYP2F1, MEGF8, ZNF257, ZNF101, CYP4F12,	7786, 311, 5532, 81894, 284654, 148932, 6548,
				ZNF763, LOC729745, PLCH2, PLA2G4D, RNF213, PPARA,	4007, 55190, 8654, 8404, 79642, 79895, 7582,
				GTF2IRD2, ZNF711, MIB2, RYR3, RHOT2, CACNA1H, PRDM15,	10905, 10002, 5151, 3658, 342132, 25976,

				ZSWIM6, ICK, RABGEF1, ZNF658B, ZNF674, CASZ1, SAG, DKFZP686M0199, LOC730394, HIVEP2, MGC87042, CENTG3, TRIM35	51193, 4311, 64094, 7128, 821, 1123, 30061, 5583, 5494, 140766, 657, 51196, 7581, 80764, 83746, 7442, 23140, 23293, 60682, 29964, 56479, 826, 333, 1572, 1954, 113835, 94039, 66002, 284390, 729745, 9651, 283748, 57674, 5465, 84163, 7552, 142678, 6263, 89941, 8912, 63977, 57688, 22858, 27342, 401509, 641339, 54897, 6295, 653238, 730394, 3097, 256227, 116988, 23087
phosphoprotein phosphatase activity	0.0054663	25	14.62	MTMR1, DUSP9, PPP1CA, PPM1B, PTPRF, DUSP27, SSH1, PTPN2, DUSP11, PPM1G, PTPRZ1, ILKAP, STYXL1, CTDSP1, CDC25B, PTPRA, MDP-1, PPM1M, CDC14B, PTPN3, EPM2A, PPP1R3C, PPP3CB, PTP4A2, PPM1A	8776, 1852, 5499, 5495, 5792, 92235, 54434, 5771, 8446, 5496, 5803, 80895, 51657, 58190, 994, 5786, 145553, 132160, 8555, 5774, 7957, 5507, 5532, 8073, 5494
protein phosphatase type 2C activity	0.00659814	3	0.46	PPM1B, PPM1G, PPM1A	5495, 5496, 5494
prolyl oligopeptidase activity	0.00659814	3	0.46	PREP, APEH, DPP4	5550, 327, 1803
transmembrane receptor protein tyrosine kinase activity	0.00786187	13	6.21	EFNA3, EPHA8, EPHB2, PDGFRB, EPHB1, NRP2, ERBB3, INSRR, ROS1, EPHB3, FGFR4, NTRK2, INSR	1944, 2046, 2048, 5159, 2047, 8828, 2065, 3645, 6098, 2049, 2264, 4915, 3643
protein-cysteine S-acyltransferase activity	0.00833836	2	0.18	YKT6, ZDHHC17	10652, 23390
protein-cysteine S-palmitoleyltransferase activity	0.00833836	2	0.18	YKT6, ZDHHC17	10652, 23390
D-amino-acid oxidase activity	0.00833836	2	0.18	DAO, DDO	1610, 8528
glucose-6-phosphate 1-dehydrogenase activity	0.00833836	2	0.18	H6PD, G6PD	9563, 2539
coreceptor, soluble ligand activity	0.00833836	2	0.18	RAMP3, RAMP2	10268, 10266
sulfate transporter activity	0.0092793	4	0.91	SLC26A6, SLC26A4, SLC26A9, LOC652834	65010, 5172, 115019, 652834
sulfate porter activity	0.0092793	4	0.91	SLC26A6, SLC26A4, SLC26A9, LOC652834	65010, 5172, 115019, 652834
phosphoric monoester hydrolase activity	0.00959072	33	21.65	MTMR1, DUSP9, PPP1CA, PPM1B, PTPRF, PIB5PA, DUSP27, PHOSPHO2, INPP1, SSH1, PTPN2, DUSP11, PPM1G, PTPRZ1, ILKAP, STYXL1, CTDSP1, INPP5D, PFKFB2, NANP, CDC25B, PTPRA, MDP-1, PPM1M, CDC14B, PTPN3, FBP1, EPM2A, PPP1R3C, PPP3CB, PTP4A2, SYNJ2, PPM1A	8776, 1852, 5499, 5495, 5792, 27124, 92235, 493911, 3628, 54434, 5771, 8446, 5496, 5803, 80895, 51657, 58190, 3635, 5208, 140838, 994, 5786, 145553, 132160, 8555, 5774, 2203, 7957, 5507, 5532, 8073, 8871, 5494

GO category "cellular_component"

Number of genes annotated in GO: 1327
Number of significant GO groups found: 14

GO group	p-value	# genes (observed)	# genes (expected)	list of genes	GeneIDs
intracellular membrane-bound organelle	2.34349e-05	653	582.25	RFX2, LSM7, LONP1, ZBTB7A, TLE2, MAN2B1, ZNF564, ZNF709, CACNA1A, ZNF442, RANBP3, TIMM13, NDUFA11, MLLT1, POLRMT, REXO1, NUT, AP4E1, ERBB2IP, XRCC4, F2RL1, AKAP1, SOX9, SLC25A43, ZIC3, RPL10, SLC25A14, DUSP9, ZFAND2A, NDUFA4, CDCA7L, INTS1, HOXA10, TMED4, SETX, CIZ1, DNAI1, AGPAT2, SMARCA2, PTGES2, JMJD2C, DMRT1, HACL1, AMT, NICN1, FOXE1, ANP32B, HDAC6, HNRPH2, MID1IP1, NHS, KIF4A, COG2, EPHX1, FOXI1, RBM27, ChGn, ZNF395, NKX3-1, LYPLA1, BAG1, GBA2, APBA1, ZNF324, ZNF324B, ZIK1, RUVBL2, PRMT1, TSEN34, U2AF2, RCP9, YKT6, RAMP3, HSPA4L, ZNF330, DCK, E2F2, USP48, DDOST, EVX1, ANLN, AHR, GARS, RECQL5, LYK5, SFRS1, HOXB4, SKAP1, SC65, HCRT, PHF12, HAP1, ETV4, NUFIP2, SERPINH1, PACS1, SF3B2, NDUFV1, PRSS23, DGAT2, SIPA1, CCND1, MRPS18A, CYP39A1, TFEB, MRPL14, TRFP, ATG5, CD164, PREP, BACH2, NUDT8, B3GAT3, CDCA5, SLC29A2, MAP4K2, MTA3, MSH6, GRHL1, GATA4, ZNF251, EPHX2, SLC25A37, FBXO25, PLEKHA2, HOOK3, TXNDC5, DEK, NEDD9, PBX2, RXRB, SSR1, TFAP2A, RAB24, SNCB, MGAT1, SUB1, ARRB2, SIRT7, MIS12, ST6GALNAC2, JMJD6, ALG6, SCP2, CDCA8, AK3L1, RLF, YBX1, FOXD3, HMG2L1, TRIOBP, CBY1, XRCC6, TSPO, ACO2, TOMM22, HMOX1, BIK, UBE3C, ZBTB7B, TARS2, MTX1, RAG1AP1, MRPS21, CREB3L4, ZAK, SSB, GAD1, CDCA7, PPIG, PMS1, KIF5C, PER3, RCC1, SESN2, RPL11, EPB41, SH3BGRL3, H6PD, ZCCHC17, ATPIF1, CAMTA1, SURB7, EMG1, DIABLO, CLIP1, TBX5, MSI1, RAB35, LHX5, CHD7, SULF1, SGK3, SOX17, MRPL15, ZFP161, NOL4, CYB5A, TCF4, L3MBTL4, ST8SIA5, ESCO1, DUSP11, FLJ10081, SUCLG1, PTBP1, ZNF497, AZU1, SF3A2, CHAF1A, SNRPD3, MPV17, PPM1G, RNPS1, ZKSCAN2, CLN3, GTF3C1, KIAA0430, CORO7, GGA2, DOC2A, KCTD13, CDIPT, SSX2IP, SYT6, ANKRD13C, ZNF277P, CAV1, DLD, STX16, GNAS, MYT1, HSPD1, RPS7, PER2, HDLBP, ZNF142, GTF3C3, IDH3G, UPF3B, SYTL4, BEX2, GLA, CES2, NUTF2, CTBP2, SEC61A2, MLLT10, BNIP3, GTPBP4, CAMK1D, MCM7, CYP3A5, MTERF, C14orf4, MTAC2D1, PPP1R13B, BCL11B, ALDH6A1, ABCD4, SUZ12, DHX8, AP2B1, RAMP2, RARA, LHX1, SIL1, TAF7, HINT1, IRF1, ARTS-1, P4HA2, PJA2, SETD1B, ALDH2, SETD8, HCFC2, DAO, ABCB10, HNRPU, LIN9, PARP1, SAMD11, CYC1, ZNF696, RNF13,	5990, 51690, 9361, 51341, 7089, 4125, 163050, 163051, 773, 79973, 8498, 26517, 126328, 4298, 5442, 57455, 256646, 23431, 55914, 7518, 2150, 8165, 6662, 203427, 7547, 6134, 9016, 1852, 90637, 4697, 55536, 26173, 3206, 222068, 23064, 25792, 27019, 10555, 6595, 80142, 23081, 1761, 26061, 275, 84276, 2304, 10541, 10013, 3188, 58526, 4810, 24137, 22796, 2052, 2299, 54439, 55790, 55893, 4824, 10434, 573, 57704, 320, 25799, 388569, 284307, 10856, 3276, 79042, 11338, 27297, 10652, 10268, 22824, 27309, 1633, 1870, 84196, 1650, 2128, 54443, 196, 2617, 9400, 92335, 6426, 3214, 8631, 10609, 3060, 57649, 9001, 2118, 57532, 871, 55690, 10992, 4723, 11098, 84649, 6494, 595, 55168, 51302, 7942, 64928, 9477, 9474, 8763, 5550, 60468, 254552, 26229, 113130, 3177, 5871, 57504, 2956, 29841, 2626, 90987, 2053, 51312, 26260, 59339, 84376, 81567, 7913, 4739, 5089, 6257, 6745, 7020, 53917, 6620, 4245, 10923, 409, 51547, 79003, 10610, 23210, 29929, 6342, 55143, 205, 6018, 4904, 27022, 10042, 11078, 25776, 2547, 706, 50, 56993, 3162, 638, 9690, 51043, 80222, 4580, 55974, 54460, 148327, 51776, 6741, 2571, 83879, 9360, 5378, 3800, 8863, 1104, 83667, 6135, 2035, 83442, 9563, 51538, 93974, 23261, 9412, 10436, 56616, 6249, 6910, 4440, 11021, 64211, 55636, 23213, 23678, 64321, 29088, 7541, 8715, 1528, 6925, 91133, 29906, 114799, 8446, 55683, 8802, 5725, 162968, 566, 8175, 10036, 6634, 4358, 5496, 10921, 342357, 1201, 2975, 9665, 79585, 23062, 8448, 253980, 10423, 117178, 148281, 81573, 11179, 857, 1738, 8675, 2778, 4661, 3329, 6201, 8864, 3069, 7701, 9330, 3421, 65109, 94121, 84707, 2717, 8824, 10204, 1488, 55176, 8028, 664, 23560, 57118, 4176, 1577, 7978,

				PARP14, SEC22A, MCM2, ATG16L1, BCS1L, CTDSP1, STK36, PNKD, CEP350, ATF3, SMG7, CTSE, SOAT1, RASSF2, APBA2BP, E2F1, MYOD1, TUB, SYT9, NUCB2, PARVA, P53AIP1, GAK, HOP, COX18, LYAR, ZNF217, JPH2, CTCFL, CDS2, VPS16, GM632, USP39, NOTO, PCBP1, POLE4, HTRA2, SNRPB2, NCOA3, ASXL1, HM13, PCSK2, SNAI1, DNTTIP1, MPHOSPH6, MAPK3, ZNF629, FHOD1, CASP7, SFXN2, PAOX, PDCD4, EMX2, PKP2, HDAC7A, PTHLH, PHB2, CHD4, MLF2, BDNF, NUP98, PAX6, CYP2R1, CNOT2, TARBP2, SNRPF, SYT1, ESPL1, LRP1, CSRP1, LPGAT1, RFWD2, DEDD, TMEM9, PIGM, FLJ16478, APH1A, PYGO2, GBA, SLC39A1, DPM3, PBXIP1, DPF3, CFL2, DHRS1, SIX1, NOVA1, ERO1L, TITF1, NFKBIA, PPARD, CYP21A2, HMGA1, ITPR3, HSD17B8, SLC25A30, DIS3, SOHLH2, NEK3, TPT1, PARP4, CCNU, AYTL2, FGF10, CTNND2, AMACR, ZNF622, ALG8, ZNF259, TIMM8B, PHOX2A, MAML2, KDELC2, SLC37A4, CENTD2, APP, CBS, U2AF1, ZNF295, D4S234E, ANAPC4, AGA, RBPJ, KLF3, MSX1, UBE2I, CYBA, CA5A, UBN1, TAF1C, CCNF, PCOLN3, GNPTG, PRDM7, ZNF205, RBM6, SLC25A38, MYRIP, PH-4, PRKCD, SPCS1, ST3GAL6, PPM1M, APPL1, QSER1, MARK2, INCENP, NAT10, PRDX5, CREB3L1, RCN1, PRDM11, STIP1, RASSF1, SFMBT1, CBLB, DNASE1L3, CYP11B1, RRM2B, TPD52, KLF10, C14orf159, PAPOLA, FCF1, JDP2, CYP46A1, ZNF410, MIZF, FOXRED1, STT3A, SDHD, CHEK1, TBRG1, FLI1, SPCS3, SYNGR2, CBX2, ZFY, ABCG1, NDUFV3, D2HGDH, KLF15, DBR1, RPN1, ANAPC13, CCNL1, CNOT4, COG5, SRPK2, ZNF467, HNRPL, ZNF585B, ZNF585A, PBX4, CALR3, ZNF780A, ZNF573, ZNF599, FKBP8, BRD4, PRRX2, FPGS, PTGS1, LMX1B, LHX2, CDK9, ZNF615, NUP62, NAPA, SIX5, ZNF228, UACA, SELS, AP3S2, LCTL, HERC1, AP3B2, SNRPA1, CDC14B, GKAP1, FANCC, KLF4, ZNF782, ZBTB2, SHPRH, SOD2, DDO, IRF4, EPM2A, PLAGL1, PPIL4, FOXF2, BTD, HDAC11, VHL, LMCD1, EAF1, SETD5, AAAS, CSRP2, TMEM10, ANXA11, ALDH18A1, SLC25A28, NOC3L, WDR57, RSPO1, SCMH1, TAL1, BCOR, RBBP7, FAM9B, ARX, PITX2, MAD2L1, DMXL2, RAB27A, BCL2L10, AGPAT7, TP53BP1, MKX, HNRPF, ZNF33B, MAN1A2, HIPK1, DDX20, AHCYL1, CDC7, RTCD1, MORF4L1, NR2E3, CLK3, ZNF774, ARNT2, ST6GAL1, TIPARP, ZNF639, SENP5, HSF2, L3MBTL3, TNFAIP3, MRPL18, CANX, BNIP1, TLX3, TTN, IWS1, SLC40A1, FUT8, MGAT2, AP4S1, NFATC4, NCOA4, STOX1, GHITM, ZNF33A, DDX50, GPR120, BMS1L, PHF5A, FOXRED2, DMC1, TPST2, DDX17, L3MBTL2, GSCL, UNC84B, SLC25A1, CRK, MYBBP1A, ZZEF1, SREBF1, SMG6, ATPAF2, DSE, TFAP2B, POLR1C, PPP2R5D, BCKDHB, FUT9, IXL, MRPS12, CYP2F1, GAPDHS, BTBD14B, SMARCA4, CRTC1, MRPL4, ZNF257, ZNF101, CYP4F12, LOC729745, SLC25A42, CARM1, MORG1, C1orf31, PARG, ARHGDIG, ACOX1, PPARA, ZNF711, MIB2, NKX1-2, BRCA2,	64207, 123036, 23368, 64919, 4329, 5826, 23512, 1659, 163, 10266, 5914, 3975, 64374, 6879, 3094, 3659, 51752, 8974, 9867, 23067, 217, 387893, 29915, 1610, 23456, 3192, 286826, 142, 148398, 1537, 79943, 11342, 54625, 26984, 4171, 55054, 617, 58190, 27148, 25953, 9857, 467, 9887, 1510, 6646, 9770, 63941, 1869, 4654, 7275, 143425, 4925, 55742, 63970, 2580, 84525, 285521, 55646, 7764, 57158, 140690, 8760, 64601, 57473, 10713, 344022, 5093, 56655, 27429, 6629, 8202, 171023, 81502, 5126, 6615, 116092, 10200, 5595, 23361, 29109, 840, 118980, 196743, 27250, 2018, 5318, 51564, 5744, 11331, 1108, 8079, 627, 4928, 5080, 120227, 4848, 6895, 6636, 6857, 9700, 4035, 1465, 9926, 64326, 9191, 252839, 93183, 440695, 51107, 90780, 2629, 27173, 54344, 57326, 8110, 1073, 115817, 6495, 4857, 30001, 7080, 4792, 5467, 1589, 3159, 3710, 7923, 253512, 22894, 54937, 4752, 7178, 143, 10309, 79888, 2255, 1501, 23600, 90441, 79053, 8882, 26521, 401, 84441, 143888, 2542, 116985, 351, 875, 7307, 49854, 27065, 29945, 175, 3516, 51274, 4487, 7329, 1535, 763, 29855, 9013, 899, 5119, 84572, 11105, 7755, 10180, 54977, 25924, 54681, 5580, 28972, 10402, 132160, 26060, 79832, 2011, 3619, 55226, 25824, 90993, 5954, 56981, 10963, 11186, 51460, 868, 1776, 1584, 50484, 7163, 7071, 80017, 10914, 51077, 122953, 10858, 57862, 25988, 55572, 3703, 6392, 1111, 84897, 2313, 60559, 9144, 84733, 7544, 9619, 4731, 728294, 28999, 51163, 6184, 25847, 57018, 4850, 10466, 6733, 168544, 3191, 92285, 199704, 80714, 125972, 284323, 126231, 148103, 23770, 23476, 51450, 2356, 5742, 4010, 9355, 1025, 284370, 23636, 8775, 147912, 7771, 55075, 55829, 10239, 197021, 8925, 8120, 6627, 8555, 80318, 2176, 9314, 158431, 57621, 257218, 6648, 8528, 3662, 7957, 5325, 85313, 2295, 686, 79885, 7428, 29995, 85403, 55209, 8086, 1466, 93377, 311, 5832, 81894, 64318, 9410, 284654, 22955, 6886, 54880, 5931, 171483, 170302, 5308, 4085, 23312, 5873, 10017, 254531, 7158, 283078, 3185, 7582, 10905, 204851, 11218, 10768, 8317, 8634, 10933, 10002, 1198, 342132, 9915, 6480, 25976, 51193,

				SLC25A15, SFRS5, RHOT2, MRPL38, RPS6, MEF2B, PRDM15, QKI, ZNF674, CORO1A, VRK3, CASZ1, NSL1, MRPL19, HNRPA3, DKFZP686M0199, LOC730394, MAML1, HIVEP2, ZNF783, CENTG3, TRIM35, FAM9C	205564, 3298, 84456, 7128, 29074, 821, 662, 30012, 7273, 55677, 30061, 2530, 4247, 11154, 4776, 8031, 219736, 27069, 7581, 79009, 338557, 9790, 84844, 80020, 11144, 8459, 10521, 83746, 2928, 25777, 6576, 1398, 10514, 23140, 6720, 23293, 91647, 29940, 7021, 9533, 5528, 594, 10690, 55588, 6183, 1572, 26330, 112939, 6597, 23373, 51073, 113835, 94039, 66002, 729745, 284439, 10498, 84292, 388753, 8505, 398, 51, 5465, 7552, 142678, 390010, 675, 10166, 6430, 89941, 64978, 6194, 4207, 63977, 9444, 641339, 11151, 51231, 54897, 25936, 9801, 220988, 653238, 730394, 9794, 3097, 155060, 116988, 23087, 171484
membrane-bound organelle	2.57398e-05	653	582.63	RFX2, LSM7, LONP1, ZBTB7A, TLE2, MAN2B1, ZNF564, ZNF709, CACNA1A, ZNF442, RANBP3, TIMM13, NDUFA11, MLLT1, POLRMT, REXO1, NUT, AP4E1, ERBB2IP, XRCC4, F2RL1, AKAP1, SOX9, SLC25A43, ZIC3, RPL10, SLC25A14, DUSP9, ZFAND2A, NDUFA4, CDCA7L, INTS1, HOXA10, TMED4, SETX, CIZ1, DNAI1, AGPAT2, SMARCA2, PTGES2, JMJD2C, DMRT1, HACL1, AMT, NICN1, FOXE1, ANP32B, HDAC6, HNRPH2, MID1IP1, NHS, KIF4A, COG2, EPHX1, FOXI1, RBM27, ChGn, ZNF395, NKX3-1, LYPLA1, BAG1, GBA2, APBA1, ZNF324, ZNF324B, ZIK1, RUVBL2, PRMT1, TSEN34, U2AF2, RCP9, YKT6, RAMP3, HSPA4L, ZNF330, DCK, E2F2, USP48, DDOST, EVX1, ANLN, AHR, GARS, RECQL5, LYK5, SFRS1, HOXB4, SKAP1, SC65, HCRT, PHF12, HAP1, ETV4, NUFIP2, SERPINH1, PACS1, SF3B2, NDUFV1, PRSS23, DGAT2, SIPA1, CCND1, MRPS18A, CYP39A1, TFEB, MRPL14, TRFP, ATG5, CD164, PREP, BACH2, NUDT8, B3GAT3, CDCA5, SLC29A2, MAP4K2, MTA3, MSH6, GRHL1, GATA4, ZNF251, EPHX2, SLC25A37, FBXO25, PLEKHA2, HOOK3, TXNDC5, DEK, NEDD9, PBX2, RXRB, SSR1, TFAP2A, RAB24, SNCB, MGAT1, SUB1, ARRB2, SIRT7, MIS12, ST6GALNAC2, JMJD6, ALG6, SCP2, CDCA8, AK3L1, RLF, YBX1, FOXD3, HMG2L1, TRIOBP, CBY1, XRCC6, TSPO, ACO2, TOMM22, HMOX1, BIK, UBE3C, ZBTB7B, TARS2, MTX1, RAG1AP1, MRPS21, CREB3L4, ZAK, SSB, GAD1, CDCA7, PPIG, PMS1, KIF5C, PER3, RCC1, SESN2, RPL11, EPB41, SH3BGRL3, H6PD, ZCCHC17, ATPIF1, CAMTA1, SURB7, EMG1, DIABLO, CLIP1, TBX5, MSI1, RAB35, LHX5, CHD7, SULF1, SGK3, SOX17, MRPL15, ZFP161, NOL4, CYB5A, TCF4, L3MBTL4, ST8SIA5, ESCO1, DUSP11, FLJ10081, SUCLG1, PTBP1, ZNF497, AZU1, SF3A2, CHAF1A, SNRPD3, MPV17, PPM1G, RNPS1, ZKSCAN2, CLN3, GTF3C1, KIAA0430, CORO7, GGA2, DOC2A, KCTD13, CDIPT, SSX2IP, SYT6, ANKRD13C, ZNF277P, CAV1, DLD, STX16, GNAS,	5990, 51690, 9361, 51341, 7089, 4125, 163050, 163051, 773, 79973, 8498, 26517, 126328, 4298, 5442, 57455, 256646, 23431, 55914, 7518, 2150, 8165, 6662, 203427, 7547, 6134, 9016, 1852, 90637, 4697, 55536, 26173, 3206, 222068, 23064, 25792, 27019, 10555, 6595, 80142, 23081, 1761, 26061, 275, 84276, 2304, 10541, 10013, 3188, 58526, 4810, 24137, 22796, 2052, 2299, 54439, 55790, 55893, 4824, 10434, 573, 57704, 320, 25799, 388569, 284307, 10856, 3276, 79042, 11338, 27297, 10652, 10268, 22824, 27309, 1633, 1870, 84196, 1650, 2128, 54443, 196, 2617, 9400, 92335, 6426, 3214, 8631, 10609, 3060, 57649, 9001, 2118, 57532, 871, 55690, 10992, 4723, 11098, 84649, 6494, 595, 55168, 51302, 7942, 64928, 9477, 9474, 8763, 5550, 60468, 254552, 26229, 113130, 3177, 5871, 57504, 2956, 29841, 2626, 90987, 2053, 51312, 26260, 59339, 84376, 81567, 7913, 4739, 5089, 6257, 6745, 7020, 53917, 6620, 4245, 10923, 409, 51547, 79003, 10610, 23210, 29929, 6342, 55143, 205, 6018, 4904, 27022, 10042, 11078, 25776, 2547, 706, 50, 56993, 3162, 638, 9690, 51043, 80222, 4580, 55974, 54460, 148327, 51776, 6741, 2571, 83879, 9360, 5378, 3800, 8863, 1104, 83667, 6135, 2035, 83442, 9563, 51538, 93974, 23261, 9412, 10436, 56616, 6249, 6910, 4440, 11021, 64211, 55636, 23213, 23678, 64321, 29088, 7541, 8715, 1528, 6925, 91133, 29906, 114799, 8446, 55683, 8802,

				MYT1, HSPD1, RPS7, PER2, HDLBP, ZNF142, GTF3C3, IDH3G, UPF3B, SYTL4, BEX2, GLA, CES2, NUTF2, CTBP2, SEC61A2, MLLT10, BNIP3, GTPBP4, CAMK1D, MCM7, CYP3A5, MTERF, C14orf4, MTAC2D1, PPP1R13B, BCL11B, ALDH6A1, ABCD4, SUZ12, DHX8, AP2B1, RAMP2, RARA, LHX1, SIL1, TAF7, HINT1, IRF1, ARTS-1, P4HA2, PJA2, SETD1B, ALDH2, SETD8, HCFC2, DAO, ABCB10, HNRPU, LIN9, PARP1, SAMD11, CYC1, ZNF696, RNF13, PARP14, SEC22A, MCM2, ATG16L1, BCS1L, CTDSP1, STK36, PNKD, CEP350, ATF3, SMG7, CTSE, SOAT1, RASSF2, APBA2BP, E2F1, MYOD1, TUB, SYT9, NUCB2, PARVA, P53AIP1, GAK, HOP, COX18, LYAR, ZNF217, JPH2, CTCFL, CDS2, VPS16, GM632, USP39, NOTO, PCBP1, POLE4, HTRA2, SNRPB2, NCOA3, ASXL1, HM13, PCSK2, SNAI1, DNTTIP1, MPHOSPH6, MAPK3, ZNF629, FHOD1, CASP7, SFXN2, PAOX, PDCD4, EMX2, PKP2, HDAC7A, PTHLH, PHB2, CHD4, MLF2, BDNF, NUP98, PAX6, CYP2R1, CNOT2, TARBP2, SNRPF, SYT1, ESPL1, LRP1, CSRP1, LPGAT1, RFWD2, DEDD, TMEM9, PIGM, FLJ16478, APH1A, PYGO2, GBA, SLC39A1, DPM3, PBXIP1, DPF3, CFL2, DHRS1, SIX1, NOVA1, ERO1L, TITF1, NFKBIA, PPARD, CYP21A2, HMGA1, ITPR3, HSD17B8, SLC25A30, DIS3, SOHLH2, NEK3, TPT1, PARP4, CCNU, AYTL2, FGF10, CTNND2, AMACR, ZNF622, ALG8, ZNF259, TIMM8B, PHOX2A, MAML2, KDELC2, SLC37A4, CENTD2, APP, CBS, U2AF1, ZNF295, D4S234E, ANAPC4, AGA, RBPJ, KLF3, MSX1, UBE2I, CYBA, CA5A, UBN1, TAF1C, CCNF, PCOLN3, GNPTG, PRDM7, ZNF205, RBM6, SLC25A38, MYRIP, PH-4, PRKCD, SPCS1, ST3GAL6, PPM1M, APPL1, QSER1, MARK2, INCENP, NAT10, PRDX5, CREB3L1, RCN1, PRDM11, STIP1, RASSF1, SFMBT1, CBLB, DNASE1L3, CYP11B1, RRM2B, TPD52, KLF10, C14orf159, PAPOLA, FCF1, JDP2, CYP46A1, ZNF410, MIZF, FOXRED1, STT3A, SDHD, CHEK1, TBRG1, FLI1, SPCS3, SYNGR2, CBX2, ZFY, ABCG1, NDUFV3, D2HGDH, KLF15, DBR1, RPN1, ANAPC13, CCNL1, CNOT4, COG5, SRPK2, ZNF467, HNRPL, ZNF585B, ZNF585A, PBX4, CALR3, ZNF780A, ZNF573, ZNF599, FKBP8, BRD4, PRRX2, FPGS, PTGS1, LMX1B, LHX2, CDK9, ZNF615, NUP62, NAPA, SIX5, ZNF228, UACA, SELS, AP3S2, LCTL, HERC1, AP3B2, SNRPA1, CDC14B, GKAP1, FANCC, KLF4, ZNF782, ZBTB2, SHPRH, SOD2, DDO, IRF4, EPM2A, PLAGL1, PPIL4, FOXF2, BTD, HDAC11, VHL, LMCD1, EAF1, SETD5, AAAS, CSRP2, TMEM10, ANXA11, ALDH18A1, SLC25A28, NOC3L, WDR57, RSPO1, SCMH1, TAL1, BCOR, RBBP7, FAM9B, ARX, PITX2, MAD2L1, DMXL2, RAB27A, BCL2L10, AGPAT7, TP53BP1, MKX, HNRPF, ZNF33B, MAN1A2, HIPK1, DDX20, AHCYL1, CDC7, RTCD1, MORF4L1, NR2E3, CLK3, ZNF774, ARNT2, ST6GAL1, TIPARP, ZNF639, SENP5, HSF2, L3MBTL3, TNFAIP3, MRPL18, CANX, BNIP1, TLX3, TTN, IWS1, SLC40A1, FUT8, MGAT2, AP4S1, NFATC4, NCOA4, STOX1, GHITM,	5725, 162968, 566, 8175, 10036, 6634, 4358, 5496, 10921, 342357, 1201, 2975, 9665, 79585, 23062, 8448, 253980, 10423, 117178, 148281, 81573, 11179, 857, 1738, 8675, 2778, 4661, 3329, 6201, 8864, 3069, 7701, 9330, 3421, 65109, 94121, 84707, 2717, 8824, 10204, 1488, 55176, 8028, 664, 23560, 57118, 4176, 1577, 7978, 64207, 123036, 23368, 64919, 4329, 5826, 23512, 1659, 163, 10266, 5914, 3975, 64374, 6879, 3094, 3659, 51752, 8974, 9867, 23067, 217, 387893, 29915, 1610, 23456, 3192, 286826, 142, 148398, 1537, 79943, 11342, 54625, 26984, 4171, 55054, 617, 58190, 27148, 25953, 9857, 467, 9887, 1510, 6646, 9770, 63941, 1869, 4654, 7275, 143425, 4925, 55742, 63970, 2580, 84525, 285521, 55646, 7764, 57158, 140690, 8760, 64601, 57473, 10713, 344022, 5093, 56655, 27429, 6629, 8202, 171023, 81502, 5126, 6615, 116092, 10200, 5595, 23361, 29109, 840, 118980, 196743, 27250, 2018, 5318, 51564, 5744, 11331, 1108, 8079, 627, 4928, 5080, 120227, 4848, 6895, 6636, 6857, 9700, 4035, 1465, 9926, 64326, 9191, 252839, 93183, 440695, 51107, 90780, 2629, 27173, 54344, 57326, 8110, 1073, 115817, 6495, 4857, 30001, 7080, 4792, 5467, 1589, 3159, 3710, 7923, 253512, 22894, 54937, 4752, 7178, 143, 10309, 79888, 2255, 1501, 23600, 90441, 79053, 8882, 26521, 401, 84441, 143888, 2542, 116985, 351, 875, 7307, 49854, 27065, 29945, 175, 3516, 51274, 4487, 7329, 1535, 763, 29855, 9013, 899, 5119, 84572, 11105, 7755, 10180, 54977, 25924, 54681, 5580, 28972, 10402, 132160, 26060, 79832, 2011, 3619, 55226, 25824, 90993, 5954, 56981, 10963, 11186, 51460, 868, 1776, 1584, 50484, 7163, 7071, 80017, 10914, 51077, 122953, 10858, 57862, 25988, 55572, 3703, 6392, 1111, 84897, 2313, 60559, 9144, 84733, 7544, 9619, 4731, 728294, 28999, 51163, 6184, 25847, 57018, 4850, 10466, 6733, 168544, 3191, 92285, 199704, 80714, 125972, 284323, 126231, 148103, 23770, 23476, 51450, 2356, 5742, 4010, 9355, 1025, 284370, 23636, 8775, 147912, 7771, 55075, 55829, 10239, 197021, 8925, 8120, 6627, 8555, 80318, 2176, 9314, 158431, 57621, 257218, 6648, 8528, 3662,

				ZNF33A, DDX50, GPR120, BMS1L, PHF5A, FOXRED2, DMC1, TPST2, DDX17, L3MBTL2, GSCL, UNC84B, SLC25A1, CRK, MYBBP1A, ZZEF1, SREBF1, SMG6, ATPAF2, DSE, TFAP2B, POLR1C, PPP2R5D, BCKDHB, FUT9, IXL, MRPS12, CYP2F1, GAPDHS, BTBD14B, SMARCA4, CRTC1, MRPL4, ZNF257, ZNF101, CYP4F12, LOC729745, SLC25A42, CARM1, MORG1, C1orf31, PARG, ARHGDIG, ACOX1, PPARA, ZNF711, MIB2, NKX1-2, BRCA2, SLC25A15, SFRS5, RHOT2, MRPL38, RPS6, MEF2B, PRDM15, QKI, ZNF674, CORO1A, VRK3, CASZ1, NSL1, MRPL19, HNRPA3, DKFZP686M0199, LOC730394, MAML1, HIVEP2, ZNF783, CENTG3, TRIM35, FAM9C	7957, 5325, 85313, 2295, 686, 79885, 7428, 29995, 85403, 55209, 8086, 1466, 93377, 311, 5832, 81894, 64318, 9410, 284654, 22955, 6886, 54880, 5931, 171483, 170302, 5308, 4085, 23312, 5873, 10017, 254531, 7158, 283078, 3185, 7582, 10905, 204851, 11218, 10768, 8317, 8634, 10933, 10002, 1198, 342132, 9915, 6480, 25976, 51193, 205564, 3298, 84456, 7128, 29074, 821, 662, 30012, 7273, 55677, 30061, 2530, 4247, 11154, 4776, 8031, 219736, 27069, 7581, 79009, 338557, 9790, 84844, 80020, 11144, 8459, 10521, 83746, 2928, 25777, 6576, 1398, 10514, 23140, 6720, 23293, 91647, 29940, 7021, 9533, 5528, 594, 10690, 55588, 6183, 1572, 26330, 112939, 6597, 23373, 51073, 113835, 94039, 66002, 729745, 284439, 10498, 84292, 388753, 8505, 398, 51, 5465, 7552, 142678, 390010, 675, 10166, 6430, 89941, 64978, 6194, 4207, 63977, 9444, 641339, 11151, 51231, 54897, 25936, 9801, 220988, 653238, 730394, 9794, 3097, 155060, 116988, 23087, 171484
intracellular	0.000500105	896	840.91	RFX2, LSM7, LONP1, FARSA, ZBTB7A, CDC37, TLE2, MAN2B1, ZNF564, ZNF709, CACNA1A, ZNF442, RANBP3, COL5A3, TIMM13, NDUFA11, MLLT1, POLRMT, REXO1, NUT, AP4E1, RAB8B, MAPK6, ERBB2IP, XRCC4, F2RL1, PITPNC1, AKAP1, SOX9, SLC25A43, ZIC3, MTMR1, VBP1, RPL10, SLC25A14, BIRC4, DUSP9, ZFAND2A, NDUFA4, CDCA7L, INTS1, FLJ21767, HOXA10, MYO1G, TMED4, SETX, SH3GL2, RALGDS, CIZ1, DNAI1, AGPAT2, SMARCA2, PTGES2, JMJD2C, SH2D3C, DMRT1, CCBL1, HACL1, KIF9, AMT, NICN1, NGLY1, VPS13A, FOXE1, ANP32B, CCIN, OSTF1, HDAC6, HNRPH2, MID1IP1, NHS, KIF4A, ARR3, ARF1, C1orf69, COG2, EPHX1, FOXI1, ABLIM3, RBM27, SGCD, EIF4EBP3, ChGn, ZNF395, NKX3-1, PSD3, LYPLA1, BAG1, GBA2, TLN1, APBA1, GNAQ, ZNF324, ZNF324B, FTL, ZIK1, RUVBL2, PRMT1, TSEN34, U2AF2, UPP1, RCP9, LIMK1, YKT6, DBNL, CCM2, RAMP3, SPRY1, HSPA4L, EXOC1, ZNF330, DCK, E2F2, USP48, DDOST, RAP1GAP, KIF17, KIAA1706, EVX1, ANLN, AHR, GARS, RECQL5, LYK5, SFRS1, HOXB4, SKAP1, SC65, HCRT, PHF12, HAP1, ETV4, NUFIP2, SERPINH1, PACS1, SF3B2, CAPN5, NDUFV1, PRSS23, DGAT2, SIPA1, DIXDC1, ELMOD1, CCND1, AIP, MRPS18A, CYP39A1, ASCC3, TFEB, MRPL14, TRFP, ATG5, CD164, PREP, GSTA1, BACH2, RAP2A, SUGT1, NUDT8, B3GAT3, CDCA5, SLC29A2, MAP4K2, MTA3, RRM2, MSH6, GRHL1, FLJ21839, GATA4, ZNF251,	5990, 51690, 9361, 2193, 51341, 11140, 7089, 4125, 163050, 163051, 773, 79973, 8498, 50509, 26517, 126328, 4298, 5442, 57455, 256646, 23431, 51762, 5597, 55914, 7518, 2150, 26207, 8165, 6662, 203427, 7547, 8776, 7411, 6134, 9016, 331, 1852, 90637, 4697, 55536, 26173, 401331, 3206, 64005, 222068, 23064, 6456, 5900, 25792, 27019, 10555, 6595, 80142, 23081, 10044, 1761, 883, 26061, 64147, 275, 84276, 55768, 23230, 2304, 10541, 881, 26578, 10013, 3188, 58526, 4810, 24137, 407, 375, 200205, 22796, 2052, 2299, 22885, 54439, 6444, 8637, 55790, 55893, 4824, 23362, 10434, 573, 57704, 7094, 320, 2776, 25799, 388569, 2512, 284307, 10856, 3276, 79042, 11338, 7378, 27297, 3984, 10652, 28988, 83605, 10268, 10252, 22824, 55763, 27309, 1633, 1870, 84196, 1650, 5909, 57576, 80820, 2128, 54443, 196, 2617, 9400, 92335, 6426, 3214, 8631, 10609, 3060, 57649, 9001, 2118, 57532, 871, 55690, 10992, 726, 4723, 11098, 84649, 6494, 85458, 55531, 595, 9049, 55168, 51302, 10973, 7942, 64928, 9477, 9474,

				EPHX2, PLEC1, SLC25A37, PTK2B, FBXO25, PLEKHA2, HOOK3,	8763, 5550, 2938, 60468, 5911, 10910, 254552,
				SCARA3, RPL8, TXNDC5, DEK, TBC1D7, SERPINB9, KIF13A,	26229, 113130, 3177, 5871, 57504, 6241, 2956,
				NEDD9, PBX2, GMDS, GNL1, RXRB, SSR1, TFAP2A, DOK3, GFPT2,	29841, 60509, 2626, 90987, 2053, 5339, 51312,
				RAB24, SNCB, MGAT1, RASGEF1C, SUB1, ARRB2, SIRT7, LRRC48,	2185, 26260, 59339, 84376, 51435, 6132, 81567,
				MIS12, ST6GALNAC2, GARNL4, JMJD6, ALG6, SCP2, CDCA8,	7913, 51256, 5272, 63971, 4739, 5089, 2762,
				AK3L1, RLF, YBX1, FOXD3, RALBP1, PIB5PA, HMG2L1, TRIOBP,	2794, 6257, 6745, 7020, 79930, 9945, 53917,
				CBY1, XRCC6, FBXO7, TSPO, PARVG, ACO2, TOMM22, HMOX1,	6620, 4245, 255426, 10923, 409, 51547, 83450,
				BIK, ARHGEF5, CUL1, UBE3C, ZBTB7B, TARS2, PSMD4, MTX1,	79003, 10610, 23108, 23210, 29929, 6342, 55143,
				RAG1AP1, MRPS21, CREB3L4, GYPC, ZAK, SSB, GAD1, MYO3B,	205, 6018, 4904, 27022, 10928, 27124, 10042,
				CDCA7, MYO1B, PPIG, PMS1, KIF5C, PER3, RCC1, SESN2, RPL11,	11078, 25776, 2547, 25793, 706, 64098, 50,
				EPB41, SH3BGRL3, H6PD, ZCCHC17, ATPIF1, CAMTA1, TSSK3,	56993, 3162, 638, 7984, 8454, 9690, 51043,
				AEBP2, SURB7, EMG1, CAMKK2, DIABLO, CLIP1, TBX5, MSI1,	80222, 5710, 4580, 55974, 54460, 148327, 2995,
				SSH1, RAB35, LHX5, CHD7, SULF1, SGK3, TRIB1, SOX17, MRPL15,	51776, 6741, 2571, 140469, 83879, 4430, 9360,
				ZFP161, MRCL3, NOL4, CYB5A, DSC2, TCF4, L3MBTL4, FBXO15,	5378, 3800, 8863, 1104, 83667, 6135, 2035,
				ST8SIA5, ESCO1, DUSP11, FLJ10081, FBXO41, SUCLG1, HMHA1,	83442, 9563, 51538, 93974, 23261, 81629,
				EVI5L, PTBP1, ZNF497, AZU1, SF3A2, ITGB1BP3, CHAF1A, SNRPD3,	121536, 9412, 10436, 10645, 56616, 6249, 6910,
				MAPK12, ITSN2, OTOF, ROCK2, MPV17, PPM1G, RNPS1, ZKSCAN2,	4440, 54434, 11021, 64211, 55636, 23213, 23678,
				CLN3, GTF3C1, KIAA0430, CORO7, GGA2, DOC2A, KCTD13, CDIPT,	10221, 64321, 29088, 7541, 10627, 8715, 1528,
				SSX2IP, RHOC, SYT6, SYDE2, ANKRD13C, ZNF277P, FLJ25778,	1824, 6925, 91133, 201456, 29906, 114799, 8446,
				CAV1, ARPC1B, DLD, STX16, GNAS, MYT1, HSPD1, TNS1, RPS7,	55683, 150726, 8802, 23526, 115704, 5725,
				PER2, HDLBP, ZNF142, GTF3C3, IDH3G, UPF3B, SYTL4, TMSB4X,	162968, 566, 8175, 27231, 10036, 6634, 6300,
				G6PD, BEX2, GLA, KIAA0174, GAN, CES2, NUTF2, CTBP2,	50618, 9381, 9475, 4358, 5496, 10921, 342357,
				SEC61A2, MLLT10, BNIP3, GTPBP4, PFKP, VIM, CAMK1D, MCM7,	1201, 2975, 9665, 79585, 23062, 8448, 253980,
				CYP3A5, MTERF, FBXL19, CYLD, C14orf4, TRIP11, MTAC2D1,	10423, 117178, 389, 148281, 84144, 81573,
				PPP1R13B, BCL11B, ALDH6A1, ABCD4, SUZ12, RASL10B, DHX8,	11179, 254048, 857, 10095, 1738, 8675, 2778,
				AP2B1, RAMP2, RARA, PSMD11, LHX1, SIL1, TAF7, HINT1, CDO1,	4661, 3329, 7145, 6201, 8864, 3069, 7701, 9330,
				GLRX, IRF1, ARTS-1, P4HA2, PJA2, SETD1B, ALDH2, SETD8,	3421, 65109, 94121, 7114, 2539, 84707, 2717,
				HCFC2, DAO, ABCB10, HNRPU, LIN9, PARP1, SAMD11, TP53BP2,	9798, 8139, 8824, 10204, 1488, 55176, 8028, 664,
				CYC1, ZNF696, RNF13, PARP14, SEC22A, GTPBP8, MCM2, COL4A3,	23560, 5214, 7431, 57118, 4176, 1577, 7978,
				ATG16L1, BCS1L, CTDSP1, STK36, SCLY, PNKD, CEP350, ATF3,	54620, 1540, 64207, 9321, 123036, 23368, 64919,
				SMG7, KLHL20, CTSE, SOAT1, SDCBP2, RASSF2, APBA2BP, E2F1,	4329, 5826, 23512, 91608, 1659, 163, 10266,
				MYOD1, PPFIBP2, TUB, SYT9, NUCB2, DNHD1, PARVA, P53AIP1,	5914, 5717, 3975, 64374, 6879, 3094, 1036, 2745,
				FEZ1, PTDSS2, PPP2R2C, GAK, CENTD1, HOP, GRSF1, COX18,	3659, 51752, 8974, 9867, 23067, 217, 387893,
				LYAR, G3BP2, WDR1, NEURL2, ZNF217, JPH2, CTCFL, CDC25B,	29915, 1610, 23456, 3192, 286826, 142, 148398,
				CDS2, VPS16, GM632, USP39, NOTO, PCBP1, POLE4, HTRA2,	7159, 1537, 79943, 11342, 54625, 26984, 29083,
				SNRPB2, NCOA3, ASXL1, HM13, PCSK2, SNAI1, DNTTIP1,	4171, 1285, 55054, 617, 58190, 27148, 51540,
				MPHOSPH6, CD2BP2, MAPK3, PRSS36, ZNF629, FHOD1, CASP7,	25953, 9857, 467, 9887, 27252, 1510, 6646,
				SFXN2, MTG1, PAOX, PDCD4, EMX2, PKP2, HDAC7A, PTHLH,	27111, 9770, 63941, 1869, 4654, 8495, 7275,
				PHB2, CHD4, MLF2, BDNF, NUP98, TRIM5, PAX6, CYP2R1, CNOT2,	143425, 4925, 144132, 55742, 63970, 9638,
				TARBP2, KRT18, SNRPF, SYT1, ESPL1, LRP1, RASSF3, CSRP1,	81490, 5522, 2580, 116984, 84525, 2926, 285521,
				RC3H1, LPGAT1, YOD1, RFWD2, DEDD, TMEM9, DDX59, PIGM,	55646, 9908, 9948, 140825, 7764, 57158, 140690,
				FLJ16478, APH1A, PYGO2, GBA, SLC39A1, DPM3, PBXIP1, DPF3,	994, 8760, 64601, 57473, 10713, 344022, 5093,
				CFL2, DHRS1, MYH7, SIX1, NOVA1, ERO1L, TITF1, NFKBIA, PPARD,	56655, 27429, 6629, 8202, 171023, 81502, 5126,
				CYP21A2, HMGA1, PFDN6, ITPR3, HSD17B8, SLC25A30, DIS3,	6615, 116092, 10200, 10421, 5595, 146547,

				SOHLH2, LCP1, NEK3, UBL3, TPT1, KATNAL1, PARP4, CCNU, AYTL2, FGF10, CTNND2, AMACR, ZNF622, RPS23, ALG8, ZNF259, TIMM8B, PHOX2A, MAML2, KDELC2, SLC37A4, C11orf51, CENTD2, ARRB1, GUCY1A2, CCT8, APP, FTCD, SOD1, CBS, U2AF1, ZNF295, D4S234E, ANAPC4, AGA, RBPJ, KLF3, MSX1, UBE2I, CYBA, CA5A, RNF166, UBN1, TAF1C, TRAF7, CCNF, PCOLN3, GNPTG, PRSS21, PRDM7, STUB1, ZNF205, HBA2, ARL6, RBM6, SLC25A38, MYRIP, PH-4, PRKCD, RNF123, SPCS1, ST3GAL6, PPM1M, APPL1, DAG1, QSER1, MARK2, INCENP, NAT10, PRDX5, CREB3L1, RCN1, KCNK4, PRDM11, CAPN1, STIP1, RASSF1, ACTR8, SFMBT1, CBLB, TWF2, DNASE1L3, ARHGEF3, CYP11B1, RRM2B, TPD52, FAM84B, KLF10, KIF26A, C14orf159, PAPOLA, FCF1, EIF5, JDP2, CYP46A1, ZNF410, MIZF, FOXRED1, BCDO2, STT3A, SDHD, CHEK1, TBRG1, DDX25, FLI1, SPCS3, C1QTNF1, SYNGR2, CBX2, FLJ21865, CD99, ZFY, MX1, ABCG1, NDUFV3, COL6A2, ADARB1, D2HGDH, KLF15, DBR1, RPN1, ANAPC13, CCNL1, CNOT4, COG5, SRPK2, ZNF467, ZNF800, HNRPL, ZNF585B, ZNF585A, PBX4, CALR3, ZNF780A, ZNF573, GMIP, ZNF599, FKBP8, BRD4, PRRX2, FPGS, PTGS1, LMX1B, LHX2, CDK9, ZNF615, NUP62, CNFN, STRN4, NAPA, SIX5, ZNF228, PAFAH1B3, UACA, SELS, AP3S2, LCTL, HERC1, AP3B2, SNRPA1, CDC14B, GKAP1, EPB41L4B, PTPN3, FANCC, KLF4, ZNF782, ZBTB2, C6orf170, SHPRH, SOD2, DDO, IRF4, EPM2A, PLAGL1, MTRF1L, PPIL4, FOXF2, MAP7, MCF2L2, ARPC4, TTLL3, BTD, HDAC11, VHL, LMCD1, EAF1, SETD5, AAAS, KRT1, CSRP2, MAP3K12, TMEM10, ANXA11, ALDH18A1, PPP3CB, SLC25A28, ARL3, NOC3L, PIK3R3, STMN1, WDR57, RSPO1, SCMH1, TAL1, GPATCH3, BCOR, RBBP7, FAM9B, ARX, NUDT11, DYNLT3, PITX2, MAD2L1, DMXL2, SRP14, RAB27A, BCL2L10, AGPAT7, TP53BP1, MKX, TUBAL3, HNRPF, ZNF33B, CTNNA3, LARP5, MAN1A2, HIPK1, DDX20, PKN2, AHCYL1, CDC7, RTCD1, TXNIP, LRRC49, MORF4L1, NR2E3, IREB2, RPLP1, CLK3, ZNF774, ARNT2, ST6GAL1, SCHIP1, TIPARP, ZNF639, SENP5, PSMD2, EIF4A2, HSF2, TPD52L1, L3MBTL3, TNFAIP3, MRPL18, N4BP3, CANX, BNIP1, TLX3, TTN, CHN1, WIPF1, IWS1, SLC40A1, FUT8, SYNE2, MGAT2, AP4S1, NFATC4, NCOA4, STOX1, FAS, PHYHIPL, VCL, GHITM, PLCE1, ZNF33A, DDX50, GPR120, BMS1L, THAP7, PHF5A, FOXRED2, DMC1, TPST2, DDX17, L3MBTL2, EIF3S7, GSCL, UNC84B, SLC25A1, CRK, MYBBP1A, ZZEF1, SREBF1, SMG6, KRBA2, DLG4, ATPAF2, DSE, TFAP2B, DNAH8, POLR1C, FBXO9, HSP90AB1, PPP2R5D, BCKDHB, FUT9, IXL, MRPS12, PLEKHG2, PVR, CYP2F1, GAPDHS, BTBD14B, SMARCA4, CRTC1, DNM2, MRPL4, ZNF257, ZNF101, CYP4F12, ZNF763, LOC729745, SLC25A42, CARM1, MORG1, C1orf31, PARG, RDX, PLA2G4D, ARHGDIG, ACOX1, RTKN, PPARA, GTF2IRD2, ZNF711, MIB2, NKX1-2, COL2A1, BRCA2, SLC25A15,	23361, 29109, 840, 118980, 92170, 196743, 27250, 2018, 5318, 51564, 5744, 11331, 1108, 8079, 627, 4928, 85363, 5080, 120227, 4848, 6895, 3875, 6636, 6857, 9700, 4035, 283349, 1465, 149041, 9926, 55432, 64326, 9191, 252839, 83479, 93183, 440695, 51107, 90780, 2629, 27173, 54344, 57326, 8110, 1073, 115817, 4625, 6495, 4857, 30001, 7080, 4792, 5467, 1589, 3159, 10471, 3710, 7923, 253512, 22894, 54937, 3936, 4752, 5412, 7178, 84056, 143, 10309, 79888, 2255, 1501, 23600, 90441, 6228, 79053, 8882, 26521, 401, 84441, 143888, 2542, 25906, 116985, 408, 2977, 10694, 351, 10841, 6647, 875, 7307, 49854, 27065, 29945, 175, 3516, 51274, 4487, 7329, 1535, 763, 115992, 29855, 9013, 84231, 899, 5119, 84572, 10942, 11105, 10273, 7755, 3040, 84100, 10180, 54977, 25924, 54681, 5580, 63891, 28972, 10402, 132160, 26060, 1605, 79832, 2011, 3619, 55226, 25824, 90993, 5954, 50801, 56981, 823, 10963, 11186, 93973, 51460, 868, 11344, 1776, 50650, 1584, 50484, 7163, 157638, 7071, 26153, 80017, 10914, 51077, 1983, 122953, 10858, 57862, 25988, 55572, 83875, 3703, 6392, 1111, 84897, 29118, 2313, 60559, 114897, 9144, 84733, 64772, 4267, 7544, 4599, 9619, 4731, 1292, 104, 728294, 28999, 51163, 6184, 25847, 57018, 4850, 10466, 6733, 168544, 168850, 3191, 92285, 199704, 80714, 125972, 284323, 126231, 51291, 148103, 23770, 23476, 51450, 2356, 5742, 4010, 9355, 1025, 284370, 23636, 84518, 29888, 8775, 147912, 7771, 5050, 55075, 55829, 10239, 197021, 8925, 8120, 6627, 8555, 80318, 54566, 5774, 2176, 9314, 158431, 57621, 221322, 257218, 6648, 8528, 3662, 7957, 5325, 54516, 85313, 2295, 9053, 23101, 10093, 26140, 686, 79885, 7428, 29995, 85403, 55209, 8086, 3848, 1466, 7786, 93377, 311, 5832, 5532, 81894, 403, 64318, 8503, 3925, 9410, 284654, 22955, 6886, 63906, 54880, 5931, 171483, 170302, 55190, 6990, 5308, 4085, 23312, 6727, 5873, 10017, 254531, 7158, 283078, 79861, 3185, 7582, 29119, 23185, 10905, 204851, 11218, 5586, 10768, 8317, 8634, 10628, 54839, 10933, 10002, 3658, 6176, 1198, 342132, 9915, 6480, 29970,

				SFRS5, KIAA0317, RYR3, RHOT2, MRPL38, MYO1F, RPS6, MEF2B, RAB17, PRDM15, PACSIN2, SHANK3, QKI, KRBA1, ZNF658B, ZNF674, KRTAP5-8, CORO1A, VRK3, TNFRSF25, PLEKHG5, CASZ1, COL16A1, NSL1, MRPL19, HNRPA3, SAG, ASB1, DKFZP686M0199, LOC730394, MAML1, HIVEP2, KIAA1549, ZNF783, CENTG3, TRIM35, FAM9C	25976, 51193, 205564, 5708, 1974, 3298, 7164, 84456, 7128, 29074, 23138, 821, 662, 30012, 7273, 1123, 7456, 55677, 30061, 2530, 23224, 4247, 11154, 4776, 8031, 219736, 355, 84457, 7414, 27069, 51196, 7581, 79009, 338557, 9790, 80764, 84844, 80020, 11144, 8459, 10521, 83746, 8664, 2928, 25777, 6576, 1398, 10514, 23140, 6720, 23293, 124751, 1742, 91647, 29940, 7021, 1769, 9533, 26268, 3326, 5528, 594, 10690, 55588, 6183, 64857, 5817, 1572, 26330, 112939, 6597, 23373, 1785, 51073, 113835, 94039, 66002, 284390, 729745, 284439, 10498, 84292, 388753, 8505, 5962, 283748, 398, 51, 6242, 5465, 84163, 7552, 142678, 390010, 1280, 675, 10166, 6430, 9870, 6263, 89941, 64978, 4542, 6194, 4207, 64284, 63977, 11252, 85358, 9444, 84626, 401509, 641339, 57830, 11151, 51231, 8718, 57449, 54897, 1307, 25936, 9801, 220988, 6295, 51665, 653238, 730394, 9794, 3097, 57670, 155060, 116988, 23087, 171484
snRNP U2	0.000824149	3	0.28	SNRPB2, SNRPA1, PHF5A	6629, 6627, 84844
intracellular part	0.00102991	828	775.05	RFX2, LSM7, LONP1, FARSA, ZBTB7A, CDC37, TLE2, MAN2B1, ZNF564, ZNF709, CACNA1A, ZNF442, RANBP3, COL5A3, TIMM13, NDUFA11, MLLT1, POLRMT, REXO1, NUT, AP4E1, MAPK6, ERBB2IP, XRCC4, F2RL1, PITPNC1, AKAP1, SOX9, SLC25A43, ZIC3, MTMR1, VBP1, RPL10, SLC25A14, BIRC4, DUSP9, ZFAND2A, NDUFA4, CDCA7L, INTS1, HOXA10, MYO1G, TMED4, SETX, SH3GL2, CIZ1, DNAI1, AGPAT2, SMARCA2, PTGES2, JMJD2C, DMRT1, CCBL1, HACL1, KIF9, AMT, NICN1, NGLY1, FOXE1, ANP32B, CCIN, HDAC6, HNRPH2, MID1IP1, NHS, KIF4A, ARR3, C1orf69, COG2, EPHX1, FOXI1, ABLIM3, RBM27, SGCD, EIF4EBP3, ChGn, ZNF395, NKX3-1, LYPLA1, BAG1, GBA2, TLN1, APBA1, GNAQ, ZNF324, ZNF324B, FTL, ZIK1, RUVBL2, PRMT1, TSEN34, U2AF2, UPP1, RCP9, LIMK1, YKT6, DBNL, CCM2, RAMP3, SPRY1, HSPA4L, EXOC1, ZNF330, DCK, E2F2, USP48, DDOST, RAP1GAP, KIF17, EVX1, ANLN, AHR, GARS, RECQL5, LYK5, SFRS1, HOXB4, SKAP1, SC65, HCRT, PHF12, HAP1, ETV4, NUFIP2, SERPINH1, PACS1, SF3B2, NDUFV1, PRSS23, DGAT2, SIPA1, ELMOD1, CCND1, AIP, MRPS18A, CYP39A1, TFEB, MRPL14, TRFP, ATG5, CD164, PREP, GSTA1, BACH2, SUGT1, NUDT8, B3GAT3, CDCA5, SLC29A2, MAP4K2, MTA3, RRM2, MSH6, GRHL1, FLJ21839, GATA4, ZNF251, EPHX2, PLEC1, SLC25A37, PTK2B, FBXO25, PLEKHA2, HOOK3, SCARA3, RPL8, TXNDC5, DEK, SERPINB9, KIF13A, NEDD9, PBX2,	5990, 51690, 9361, 2193, 51341, 11140, 7089, 4125, 163050, 163051, 773, 79973, 8498, 50509, 26517, 126328, 4298, 5442, 57455, 256646, 23431, 5597, 55914, 7518, 2150, 26207, 8165, 6662, 203427, 7547, 8776, 7411, 6134, 9016, 331, 1852, 90637, 4697, 55536, 26173, 3206, 64005, 222068, 23064, 6456, 25792, 27019, 10555, 6595, 80142, 23081, 1761, 883, 26061, 64147, 275, 84276, 55768, 2304, 10541, 881, 10013, 3188, 58526, 4810, 24137, 407, 200205, 22796, 2052, 2299, 22885, 54439, 6444, 8637, 55790, 55893, 4824, 10434, 573, 57704, 7094, 320, 2776, 25799, 388569, 2512, 284307, 10856, 3276, 79042, 11338, 7378, 27297, 3984, 10652, 28988, 83605, 10268, 10252, 22824, 55763, 27309, 1633, 1870, 84196, 1650, 5909, 57576, 2128, 54443, 196, 2617, 9400, 92335, 6426, 3214, 8631, 10609, 3060, 57649, 9001, 2118, 57532, 871, 55690, 10992, 4723, 11098, 84649, 6494, 55531, 595, 9049, 55168, 51302, 7942, 64928, 9477, 9474, 8763, 5550, 2938, 60468, 10910, 254552, 26229, 113130, 3177, 5871, 57504, 6241, 2956, 29841,

				GMDS, RXRB, SSR1, TFAP2A, DOK3, GFPT2, RAB24, SNCB, MGAT1, SUB1, ARRB2, SIRT7, LRRC48, MIS12, ST6GALNAC2, JMJD6, ALG6, SCP2, CDCA8, AK3L1, RLF, YBX1, FOXD3, PIB5PA, HMG2L1, TRIOBP, CBY1, XRCC6, FBXO7, TSPO, PARVG, ACO2, TOMM22, HMOX1, BIK, CUL1, UBE3C, ZBTB7B, TARS2, PSMD4, MTX1, RAG1AP1, MRPS21, CREB3L4, GYPC, ZAK, SSB, GAD1, MYO3B, CDCA7, MYO1B, PPIG, PMS1, KIF5C, PER3, RCC1, SESN2, RPL11, EPB41, SH3BGRL3, H6PD, ZCCHC17, ATPIF1, CAMTA1, SURB7, EMG1, DIABLO, CLIP1, TBX5, MSI1, SSH1, RAB35, LHX5, CHD7, SULF1, SGK3, TRIB1, SOX17, MRPL15, ZFP161, MRCL3, NOL4, CYB5A, DSC2, TCF4, L3MBTL4, FBXO15, ST8SIA5, ESCO1, DUSP11, FLJ10081, SUCLG1, PTBP1, ZNF497, AZU1, SF3A2, CHAF1A, SNRPD3, MAPK12, OTOF, ROCK2, MPV17, PPM1G, RNPS1, ZKSCAN2, CLN3, GTF3C1, KIAA0430, CORO7, GGA2, DOC2A, KCTD13, CDIPT, SSX2IP, SYT6, ANKRD13C, ZNF277P, FLJ25778, CAV1, ARPC1B, DLD, STX16, GNAS, MYT1, HSPD1, TNS1, RPS7, PER2, HDLBP, ZNF142, GTF3C3, IDH3G, UPF3B, SYTL4, TMSB4X, G6PD, BEX2, GLA, KIAA0174, GAN, CES2, NUTF2, CTBP2, SEC61A2, MLLT10, BNIP3, GTPBP4, PFKP, VIM, CAMK1D, MCM7, CYP3A5, MTERF, FBXL19, CYLD, C14orf4, TRIP11, MTAC2D1, PPP1R13B, BCL11B, ALDH6A1, ABCD4, SUZ12, DHX8, AP2B1, RAMP2, RARA, PSMD11, LHX1, SIL1, TAF7, HINT1, CDO1, GLRX, IRF1, ARTS-1, P4HA2, PJA2, SETD1B, ALDH2, SETD8, HCFC2, DAO, ABCB10, HNRPU, LIN9, PARP1, SAMD11, TP53BP2, CYC1, ZNF696, RNF13, PARP14, SEC22A, MCM2, COL4A3, ATG16L1, BCS1L, CTDSP1, STK36, SCLY, PNKD, CEP350, ATF3, SMG7, KLHL20, CTSE, SOAT1, SDCBP2, RASSF2, APBA2BP, E2F1, MYOD1, TUB, SYT9, NUCB2, DNHD1, PARVA, P53AIP1, FEZ1, PPP2R2C, GAK, HOP, GRSF1, COX18, LYAR, G3BP2, WDR1, NEURL2, ZNF217, JPH2, CTCFL, CDS2, VPS16, GM632, USP39, NOTO, PCBP1, POLE4, HTRA2, SNRPB2, NCOA3, ASXL1, HM13, PCSK2, SNAI1, DNTTIP1, MPHOSPH6, CD2BP2, MAPK3, PRSS36, ZNF629, FHOD1, CASP7, SFXN2, PAOX, PDCD4, EMX2, PKP2, HDAC7A, PTHLH, PHB2, CHD4, MLF2, BDNF, NUP98, PAX6, CYP2R1, CNOT2, TARBP2, KRT18, SNRPF, SYT1, ESPL1, LRP1, RASSF3, CSRP1, RC3H1, LPGAT1, RFWD2, DEDD, TMEM9, PIGM, FLJ16478, APH1A, PYGO2, GBA, SLC39A1, DPM3, PBXIP1, DPF3, CFL2, DHRS1, MYH7, SIX1, NOVA1, ERO1L, TITF1, NFKBIA, PPARD, CYP21A2, HMGA1, PFDN6, ITPR3, HSD17B8, SLC25A30, DIS3, SOHLH2, LCP1, NEK3, TPT1, KATNAL1, PARP4, CCNU, AYTL2, FGF10, CTNND2, AMACR, ZNF622, RPS23, ALG8, ZNF259, TIMM8B, PHOX2A, MAML2, KDELC2, SLC37A4, CENTD2, ARRB1, GUCY1A2, CCT8, APP, FTCD, SOD1, CBS, U2AF1, ZNF295, D4S234E, ANAPC4, AGA, RBPJ, KLF3, MSX1, UBE2I, CYBA, CA5A, UBN1, TAF1C,	60509, 2626, 90987, 2053, 5339, 51312, 2185, 26260, 59339, 84376, 51435, 6132, 81567, 7913, 5272, 63971, 4739, 5089, 2762, 6257, 6745, 7020, 79930, 9945, 53917, 6620, 4245, 10923, 409, 51547, 83450, 79003, 10610, 23210, 29929, 6342, 55143, 205, 6018, 4904, 27022, 27124, 10042, 11078, 25776, 2547, 25793, 706, 64098, 50, 56993, 3162, 638, 8454, 9690, 51043, 80222, 5710, 4580, 55974, 54460, 148327, 2995, 51776, 6741, 2571, 140469, 83879, 4430, 9360, 5378, 3800, 8863, 1104, 83667, 6135, 2035, 83442, 9563, 51538, 93974, 23261, 9412, 10436, 56616, 6249, 6910, 4440, 54434, 11021, 64211, 55636, 23213, 23678, 10221, 64321, 29088, 7541, 10627, 8715, 1528, 1824, 6925, 91133, 201456, 29906, 114799, 8446, 55683, 8802, 5725, 162968, 566, 8175, 10036, 6634, 6300, 9381, 9475, 4358, 5496, 10921, 342357, 1201, 2975, 9665, 79585, 23062, 8448, 253980, 10423, 117178, 148281, 81573, 11179, 254048, 857, 10095, 1738, 8675, 2778, 4661, 3329, 7145, 6201, 8864, 3069, 7701, 9330, 3421, 65109, 94121, 7114, 2539, 84707, 2717, 9798, 8139, 8824, 10204, 1488, 55176, 8028, 664, 23560, 5214, 7431, 57118, 4176, 1577, 7978, 54620, 1540, 64207, 9321, 123036, 23368, 64919, 4329, 5826, 23512, 1659, 163, 10266, 5914, 5717, 3975, 64374, 6879, 3094, 1036, 2745, 3659, 51752, 8974, 9867, 23067, 217, 387893, 29915, 1610, 23456, 3192, 286826, 142, 148398, 7159, 1537, 79943, 11342, 54625, 26984, 4171, 1285, 55054, 617, 58190, 27148, 51540, 25953, 9857, 467, 9887, 27252, 1510, 6646, 27111, 9770, 63941, 1869, 4654, 7275, 143425, 4925, 144132, 55742, 63970, 9638, 5522, 2580, 84525, 2926, 285521, 55646, 9908, 9948, 140825, 7764, 57158, 140690, 8760, 64601, 57473, 10713, 344022, 5093, 56655, 27429, 6629, 8202, 171023, 81502, 5126, 6615, 116092, 10200, 10421, 5595, 146547, 23361, 29109, 840, 118980, 196743, 27250, 2018, 5318, 51564, 5744, 11331, 1108, 8079, 627, 4928, 5080, 120227, 4848, 6895, 3875, 6636, 6857, 9700, 4035, 283349, 1465, 149041, 9926, 64326, 9191, 252839, 93183, 440695, 51107, 90780, 2629, 27173, 54344, 57326, 8110, 1073, 115817,

				TRAF7, CCNF, PCOLN3, GNPTG, PRSS21, PRDM7, STUB1, ZNF205, HBA2, ARL6, RBM6, SLC25A38, MYRIP, PH-4, PRKCD, RNF123, SPCS1, ST3GAL6, PPM1M, APPL1, DAG1, QSER1, MARK2, INCENP, NAT10, PRDX5, CREB3L1, RCN1, KCNK4, PRDM11, STIP1, RASSF1, ACTR8, SFMBT1, CBLB, TWF2, DNASE1L3, CYP11B1, RRM2B, TPD52, FAM84B, KLF10, KIF26A, C14orf159, PAPOLA, FCF1, EIF5, JDP2, CYP46A1, ZNF410, MIZF, FOXRED1, STT3A, SDHD, CHEK1, TBRG1, DDX25, FLI1, SPCS3, C1QTNF1, SYNGR2, CBX2, CD99, ZFY, MX1, ABCG1, NDUFV3, COL6A2, D2HGDH, KLF15, DBR1, RPN1, ANAPC13, CCNL1, CNOT4, COG5, SRPK2, ZNF467, HNRPL, ZNF585B, ZNF585A, PBX4, CALR3, ZNF780A, ZNF573, ZNF599, FKBP8, BRD4, PRRX2, FPGS, PTGS1, LMX1B, LHX2, CDK9, ZNF615, NUP62, CNFN, STRN4, NAPA, SIX5, ZNF228, PAFAH1B3, UACA, SELS, AP3S2, LCTL, HERC1, AP3B2, SNRPA1, CDC14B, GKAP1, EPB41L4B, PTPN3, FANCC, KLF4, ZNF782, ZBTB2, SHPRH, SOD2, DDO, IRF4, EPM2A, PLAGL1, MTRF1L, PPIL4, FOXF2, MAP7, ARPC4, TTLL3, BTD, HDAC11, VHL, LMCD1, EAF1, SETD5, AAAS, KRT1, CSRP2, MAP3K12, TMEM10, ANXA11, ALDH18A1, PPP3CB, SLC25A28, NOC3L, PIK3R3, STMN1, WDR57, RSPO1, SCMH1, TAL1, BCOR, RBBP7, FAM9B, ARX, DYNLT3, PITX2, MAD2L1, DMXL2, SRP14, RAB27A, BCL2L10, AGPAT7, TP53BP1, MKX, TUBAL3, HNRPF, ZNF33B, CTNNA3, LARP5, MAN1A2, HIPK1, DDX20, AHCYL1, CDC7, RTCD1, TXNIP, LRRC49, MORF4L1, NR2E3, IREB2, RPLP1, CLK3, ZNF774, ARNT2, ST6GAL1, SCHIP1, TIPARP, ZNF639, SENP5, PSMD2, EIF4A2, HSF2, TPD52L1, L3MBTL3, TNFAIP3, MRPL18, N4BP3, CANX, BNIP1, TLX3, TTN, WIPF1, IWS1, SLC40A1, FUT8, SYNE2, MGAT2, AP4S1, NFATC4, NCOA4, STOX1, FAS, PHYHIPL, VCL, GHITM, PLCE1, ZNF33A, DDX50, GPR120, BMS1L, THAP7, PHF5A, FOXRED2, DMC1, TPST2, DDX17, L3MBTL2, EIF3S7, GSCL, UNC84B, SLC25A1, CRK, MYBBP1A, ZZEF1, SREBF1, SMG6, DLG4, ATPAF2, DSE, TFAP2B, DNAH8, POLR1C, FBXO9, HSP90AB1, PPP2R5D, BCKDHB, FUT9, IXL, MRPS12, PVR, CYP2F1, GAPDHS, BTBD14B, SMARCA4, CRTC1, DNM2, MRPL4, ZNF257, ZNF101, CYP4F12, LOC729745, SLC25A42, CARM1, MORG1, C1orf31, PARG, RDX, PLA2G4D, ARHGDIG, ACOX1, PPARA, ZNF711, MIB2, NKX1-2, COL2A1, BRCA2, SLC25A15, SFRS5, RYR3, RHOT2, MRPL38, MYO1F, RPS6, MEF2B, PRDM15, PACSIN2, SHANK3, QKI, ZNF674, KRTAP5-8, CORO1A, VRK3, TNFRSF25, CASZ1, COL16A1, NSL1, MRPL19, HNRPA3, SAG, DKFZP686M0199, LOC730394, MAML1, HIVEP2, ZNF783, CENTG3, TRIM35, FAM9C	4625, 6495, 4857, 30001, 7080, 4792, 5467, 1589, 3159, 10471, 3710, 7923, 253512, 22894, 54937, 3936, 4752, 7178, 84056, 143, 10309, 79888, 2255, 1501, 23600, 90441, 6228, 79053, 8882, 26521, 401, 84441, 143888, 2542, 116985, 408, 2977, 10694, 351, 10841, 6647, 875, 7307, 49854, 27065, 29945, 175, 3516, 51274, 4487, 7329, 1535, 763, 29855, 9013, 84231, 899, 5119, 84572, 10942, 11105, 10273, 7755, 3040, 84100, 10180, 54977, 25924, 54681, 5580, 63891, 28972, 10402, 132160, 26060, 1605, 79832, 2011, 3619, 55226, 25824, 90993, 5954, 50801, 56981, 10963, 11186, 93973, 51460, 868, 11344, 1776, 1584, 50484, 7163, 157638, 7071, 26153, 80017, 10914, 51077, 1983, 122953, 10858, 57862, 25988, 55572, 3703, 6392, 1111, 84897, 29118, 2313, 60559, 114897, 9144, 84733, 4267, 7544, 4599, 9619, 4731, 1292, 728294, 28999, 51163, 6184, 25847, 57018, 4850, 10466, 6733, 168544, 3191, 92285, 199704, 80714, 125972, 284323, 126231, 148103, 23770, 23476, 51450, 2356, 5742, 4010, 9355, 1025, 284370, 23636, 84518, 29888, 8775, 147912, 7771, 5050, 55075, 55829, 10239, 197021, 8925, 8120, 6627, 8555, 80318, 54566, 5774, 2176, 9314, 158431, 57621, 257218, 6648, 8528, 3662, 7957, 5325, 54516, 85313, 2295, 9053, 10093, 26140, 686, 79885, 7428, 29995, 85403, 55209, 8086, 3848, 1466, 7786, 93377, 311, 5832, 5532, 81894, 64318, 8503, 3925, 9410, 284654, 22955, 6886, 54880, 5931, 171483, 170302, 6990, 5308, 4085, 23312, 6727, 5873, 10017, 254531, 7158, 283078, 79861, 3185, 7582, 29119, 23185, 10905, 204851, 11218, 10768, 8317, 8634, 10628, 54839, 10933, 10002, 3658, 6176, 1198, 342132, 9915, 6480, 29970, 25976, 51193, 205564, 5708, 1974, 3298, 7164, 84456, 7128, 29074, 23138, 821, 662, 30012, 7273, 7456, 55677, 30061, 2530, 23224, 4247, 11154, 4776, 8031, 219736, 355, 84457, 7414, 27069, 51196, 7581, 79009, 338557, 9790, 80764, 84844, 80020, 11144, 8459, 10521, 83746, 8664, 2928, 25777, 6576, 1398, 10514, 23140, 6720, 23293, 1742, 91647, 29940, 7021, 1769, 9533, 26268, 3326, 5528, 594, 10690, 55588, 6183, 5817, 1572, 26330, 112939, 6597, 23373,

					1785, 51073, 113835, 94039, 66002, 729745, 284439, 10498, 84292, 388753, 8505, 5962, 283748, 398, 51, 5465, 7552, 142678, 390010, 1280, 675, 10166, 6430, 6263, 89941, 64978, 4542, 6194, 4207, 63977, 11252, 85358, 9444, 641339, 57830, 11151, 51231, 8718, 54897, 1307, 25936, 9801, 220988, 6295, 653238, 730394, 9794, 3097, 155060, 116988, 23087, 171484
cytoplasm	0.00208153	438	391.80	LONP1, FARSA, CDC37, MAN2B1, CACNA1A, COL5A3, TIMM13, NDUFA11, POLRMT, AP4E1, MAPK6, ERBB2IP, F2RL1, PITPNC1, AKAP1, SLC25A43, MTMR1, VBP1, RPL10, SLC25A14, BIRC4, DUSP9, NDUFA4, TMED4, SH3GL2, AGPAT2, PTGES2, CCBL1, HACL1, AMT, NGLY1, HDAC6, KIF4A, ARR3, C1orf69, COG2, EPHX1, ABLIM3, SGCD, EIF4EBP3, ChGn, ZNF395, LYPLA1, BAG1, GBA2, TLN1, APBA1, GNAQ, RUVBL2, PRMT1, UPP1, RCP9, LIMK1, YKT6, DBNL, CCM2, RAMP3, SPRY1, HSPA4L, EXOC1, DDOST, RAP1GAP, ANLN, AHR, GARS, RECQL5, LYK5, HCRT, HAP1, NUFIP2, SERPINH1, PACS1, NDUFV1, DGAT2, CCND1, AIP, MRPS18A, CYP39A1, MRPL14, ATG5, CD164, PREP, GSTA1, NUDT8, B3GAT3, CDCA5, MAP4K2, MTA3, RRM2, FLJ21839, EPHX2, SLC25A37, PTK2B, HOOK3, SCARA3, RPL8, TXNDC5, SERPINB9, NEDD9, GMDS, SSR1, DOK3, GFPT2, RAB24, SNCB, MGAT1, ARRB2, LRRC48, ST6GALNAC2, ALG6, SCP2, AK3L1, PIB5PA, CBY1, TSPO, ACO2, TOMM22, HMOX1, BIK, TARS2, PSMD4, MTX1, MRPS21, GYPC, ZAK, GAD1, KIF5C, SESN2, RPL11, EPB41, H6PD, ZCCHC17, ATPIF1, DIABLO, CLIP1, TBX5, SSH1, RAB35, SULF1, SGK3, TRIB1, MRPL15, MRCL3, CYB5A, ST8SIA5, SUCLG1, AZU1, SNRPD3, MAPK12, OTOF, MPV17, CLN3, KIAA0430, CORO7, GGA2, DOC2A, CDIPT, SYT6, ANKRD13C, CAV1, DLD, STX16, GNAS, HSPD1, RPS7, IDH3G, UPF3B, SYTL4, TMSB4X, G6PD, GLA, KIAA0174, CES2, NUTF2, SEC61A2, BNIP3, PFKP, VIM, CYP3A5, MTERF, FBXL19, CYLD, TRIP11, PPP1R13B, ALDH6A1, ABCD4, AP2B1, RAMP2, PSMD11, SIL1, CDO1, GLRX, ARTS-1, P4HA2, PJA2, ALDH2, HCFC2, DAO, ABCB10, TP53BP2, CYC1, SEC22A, COL4A3, ATG16L1, BCS1L, STK36, SCLY, SMG7, CTSE, SOAT1, SDCBP2, APBA2BP, TUB, SYT9, NUCB2, PARVA, P53AIP1, FEZ1, GRSF1, COX18, G3BP2, NEURL2, JPH2, CDS2, VPS16, PCBP1, HTRA2, HM13, PCSK2, CD2BP2, MAPK3, PRSS36, FHOD1, CASP7, SFXN2, PAOX, PDCD4, HDAC7A, PTHLH, PHB2, BDNF, CYP2R1, SYT1, ESPL1, RC3H1, LPGAT1, DEDD, TMEM9, PIGM, APH1A, GBA, SLC39A1, DPM3, PBXIP1, DHRS1, MYH7, ERO1L, NFKBIA, CYP21A2, PFDN6, ITPR3, HSD17B8, SLC25A30, LCP1, TPT1, AYTL2, CTNND2, AMACR, RPS23, ALG8, ZNF259, TIMM8B, KDELC2, SLC37A4, CENTD2,	9361, 2193, 11140, 4125, 773, 50509, 26517, 126328, 5442, 23431, 5597, 55914, 2150, 26207, 8165, 203427, 8776, 7411, 6134, 9016, 331, 1852, 4697, 222068, 6456, 10555, 80142, 883, 26061, 275, 55768, 10013, 24137, 407, 200205, 22796, 2052, 22885, 6444, 8637, 55790, 55893, 10434, 573, 57704, 7094, 320, 2776, 10856, 3276, 7378, 27297, 3984, 10652, 28988, 83605, 10268, 10252, 22824, 55763, 1650, 5909, 54443, 196, 2617, 9400, 92335, 3060, 9001, 57532, 871, 55690, 4723, 84649, 595, 9049, 55168, 51302, 64928, 9474, 8763, 5550, 2938, 254552, 26229, 113130, 5871, 57504, 6241, 60509, 2053, 51312, 2185, 84376, 51435, 6132, 81567, 5272, 4739, 2762, 6745, 79930, 9945, 53917, 6620, 4245, 409, 83450, 10610, 29929, 6342, 205, 27124, 25776, 706, 50, 56993, 3162, 638, 80222, 5710, 4580, 54460, 2995, 51776, 2571, 3800, 83667, 6135, 2035, 9563, 51538, 93974, 56616, 6249, 6910, 54434, 11021, 23213, 23678, 10221, 29088, 10627, 1528, 29906, 8802, 566, 6634, 6300, 9381, 4358, 1201, 9665, 79585, 23062, 8448, 10423, 148281, 81573, 857, 1738, 8675, 2778, 3329, 6201, 3421, 65109, 94121, 7114, 2539, 2717, 9798, 8824, 10204, 55176, 664, 5214, 7431, 1577, 7978, 54620, 1540, 9321, 23368, 4329, 5826, 163, 10266, 5717, 64374, 1036, 2745, 51752, 8974, 9867, 217, 29915, 1610, 23456, 7159, 1537, 26984, 1285, 55054, 617, 27148, 51540, 9887, 1510, 6646, 27111, 63941, 7275, 143425, 4925, 55742, 63970, 9638, 2926, 285521, 9908, 140825, 57158, 8760, 64601, 5093, 27429, 81502, 5126, 10421, 5595, 146547, 29109, 840, 118980, 196743, 27250, 51564, 5744, 11331, 627, 120227, 6857, 9700, 149041, 9926, 9191, 252839, 93183,

				ARRB1, GUCY1A2, CCT8, APP, FTCD, SOD1, CBS, D4S234E, AGA, CYBA, CA5A, TRAF7, PCOLN3, GNPTG, PRSS21, HBA2, ARL6, SLC25A38, MYRIP, PH-4, PRKCD, RNF123, SPCS1, ST3GAL6, APPL1, DAG1, MARK2, PRDX5, RCN1, KCNK4, STIP1, CYP11B1, TPD52, FAM84B, C14orf159, PAPOLA, EIF5, CYP46A1, FOXRED1, STT3A, SDHD, DDX25, SPCS3, C1QTNF1, SYNGR2, CD99, MX1, ABCG1, NDUFV3, COL6A2, D2HGDH, RPN1, COG5, SRPK2, CALR3, FKBP8, FPGS, PTGS1, NUP62, STRN4, NAPA, PAFAH1B3, UACA, SELS, AP3S2, LCTL, HERC1, AP3B2, GKAP1, EPB41L4B, PTPN3, FANCC, SOD2, DDO, EPM2A, MTRF1L, VHL, MAP3K12, TMEM10, ANXA11, ALDH18A1, SLC25A28, PIK3R3, STMN1, DMXL2, SRP14, RAB27A, BCL2L10, AGPAT7, TP53BP1, MAN1A2, DDX20, AHCYL1, CDC7, TXNIP, IREB2, RPLP1, ST6GAL1, SCHIP1, PSMD2, EIF4A2, HSF2, TPD52L1, TNFAIP3, MRPL18, N4BP3, CANX, BNIP1, TTN, SLC40A1, FUT8, MGAT2, AP4S1, FAS, PHYHIPL, VCL, GHITM, PLCE1, GPR120, FOXRED2, TPST2, EIF3S7, SLC25A1, CRK, MYBBP1A, SREBF1, SMG6, DLG4, ATPAF2, DSE, HSP90AB1, BCKDHB, FUT9, MRPS12, PVR, CYP2F1, DNM2, MRPL4, CYP4F12, SLC25A42, CARM1, C1orf31, PARG, RDX, PLA2G4D, ARHGDIG, ACOX1, MIB2, COL2A1, BRCA2, SLC25A15, RYR3, RHOT2, MRPL38, RPS6, PACSIN2, SHANK3, CORO1A, TNFRSF25, COL16A1, MRPL19, SAG, CENTG3	51107, 2629, 27173, 54344, 57326, 115817, 4625, 30001, 4792, 1589, 10471, 3710, 7923, 253512, 3936, 7178, 79888, 1501, 23600, 6228, 79053, 8882, 26521, 143888, 2542, 116985, 408, 2977, 10694, 351, 10841, 6647, 875, 27065, 175, 1535, 763, 84231, 5119, 84572, 10942, 3040, 84100, 54977, 25924, 54681, 5580, 63891, 28972, 10402, 26060, 1605, 2011, 25824, 5954, 50801, 10963, 1584, 7163, 157638, 80017, 10914, 1983, 10858, 55572, 3703, 6392, 29118, 60559, 114897, 9144, 4267, 4599, 9619, 4731, 1292, 728294, 6184, 10466, 6733, 125972, 23770, 2356, 5742, 23636, 29888, 8775, 5050, 55075, 55829, 10239, 197021, 8925, 8120, 80318, 54566, 5774, 2176, 6648, 8528, 7957, 54516, 7428, 7786, 93377, 311, 5832, 81894, 8503, 3925, 23312, 6727, 5873, 10017, 254531, 7158, 10905, 11218, 10768, 8317, 10628, 3658, 6176, 6480, 29970, 5708, 1974, 3298, 7164, 7128, 29074, 23138, 821, 662, 7273, 30061, 2530, 4247, 11154, 355, 84457, 7414, 27069, 51196, 338557, 80020, 8459, 8664, 6576, 1398, 10514, 6720, 23293, 1742, 91647, 29940, 3326, 594, 10690, 6183, 5817, 1572, 1785, 51073, 66002, 284439, 10498, 388753, 8505, 5962, 283748, 398, 51, 142678, 1280, 675, 10166, 6263, 89941, 64978, 6194, 11252, 85358, 11151, 8718, 1307, 9801, 6295, 116988
membrane fraction	0.00226547	84	61.92	SPINT1, CACNA1G, SLC16A2, EPHX1, PCDHAC2, LYPLA1, GBA2, RAP1GAP, DGAT2, AIP, CYP39A1, CD164, ITM2B, KCNF1, SLC17A2, SNCB, XRCC6, HMOX1, DPP10, H6PD, OPRD1, CYB5A, HCN2, MADCAM1, ADORA2A, OTOF, GP2, CLN3, CORO7, DOC2A, CAV1, STX16, GNAS, CLCN4, CYP3A5, SLC6A2, OAS2, NRP2, JPH2, SLC12A4, ADRB1, CD59, CYP2R1, LRP1, SLC39A1, ERO1L, CYP21A2, SLC39A7, HSD17B8, ARRB1, APP, SLC34A2, PRSS21, SPCS1, DAG1, CYP11B1, LRP12, CYP46A1, STT3A, SPCS3, ABCG1, SLC1A6, PTGS1, STRN4, KCNN4, UACA, HCN4, ROS1, MAP3K12, GRIK3, SH3KBP1, BCL2L10, MAN1A2, MGAT2, PLCE1, SLC18A3, TPST2, APLP1, CYP2F1, CYP4F12, SLC18A2, RYR3, SLC16A5, LILRB2	6692, 8913, 6567, 2052, 56134, 10434, 57704, 5909, 84649, 9049, 51302, 8763, 9445, 3754, 10246, 6620, 2547, 3162, 57628, 9563, 4985, 1528, 610, 8174, 135, 9381, 2813, 1201, 79585, 8448, 857, 8675, 2778, 1183, 1577, 6530, 4939, 8828, 57158, 6560, 153, 966, 120227, 4035, 27173, 30001, 1589, 7922, 7923, 408, 351, 10568, 10942, 28972, 1605, 1584, 29967, 10858, 3703, 60559, 9619, 6511, 5742, 29888, 3783, 55075, 10021, 6098, 7786, 2899, 30011, 10017, 10905, 4247, 51196, 6572, 8459, 333, 1572, 66002, 6571, 6263, 9121, 10288
condensed nuclear chromosome	0.00651544	8	2.91	SC65, MIS12, RCC1, UBE2I, PCOLN3, CHEK1, TTN, DMC1	10609, 79003, 1104, 7329, 5119, 1111, 7273, 11144
	0.00666413	7	2.35	SF3A2, SNRPD3, SNRPB2, SNRPF, SNRPA1, WDR57, PHF5A	8175, 6634, 6629, 6636, 6627, 9410, 84844

small nuclear ribonucleoprotein complex					
nuclear part	0.00775723	102	80.87	RANBP3, INTS1, SMARCA2, HDAC6, HNRPH2, RUVBL2, TSEN34, U2AF2, ZNF330, E2F2, RECQL5, SFRS1, SC65, PHF12, SF3B2, TFEB, TRFP, SLC29A2, MSH6, SUB1, SIRT7, MIS12, XRCC6, PPIG, RCC1, RPL11, ZCCHC17, SURB7, EMG1, NOL4, PTBP1, SF3A2, CHAF1A, SNRPD3, GTF3C1, RPS7, GTF3C3, NUTF2, BNIP3, SUZ12, DHX8, TAF7, HNRPU, PARP1, MCM2, PNKD, E2F1, NUCB2, LYAR, SNRPB2, HDAC7A, NUP98, SNRPF, DEDD, SIX1, TITF1, HMGA1, CCNU, ZNF259, U2AF1, ANAPC4, RBPJ, UBE2I, PCOLN3, APPL1, QSER1, CHEK1, CBX2, HNRPL, PRRX2, CDK9, NUP62, UACA, SNRPA1, FOXF2, BTD, HDAC11, AAAS, ANXA11, NOC3L, WDR57, PITX2, TP53BP1, HNRPF, DDX20, RTCD1, ARNT2, BNIP1, TTN, PHF5A, DMC1, UNC84B, MYBBP1A, ZZEF1, SREBF1, SMG6, POLR1C, IXL, SMARCA4, MORG1, MRPL19, HNRPA3	8498, 26173, 6595, 10013, 3188, 10856, 79042, 11338, 27309, 1870, 9400, 6426, 10609, 57649, 10992, 7942, 9477, 3177, 2956, 10923, 51547, 79003, 2547, 9360, 1104, 6135, 51538, 9412, 10436, 8715, 5725, 8175, 10036, 6634, 2975, 6201, 9330, 10204, 664, 23512, 1659, 6879, 3192, 142, 4171, 25953, 1869, 4925, 55646, 6629, 51564, 4928, 6636, 9191, 6495, 7080, 3159, 10309, 8882, 7307, 29945, 3516, 7329, 5119, 26060, 79832, 1111, 84733, 3191, 51450, 1025, 23636, 55075, 6627, 2295, 686, 79885, 8086, 311, 64318, 9410, 5308, 7158, 3185, 11218, 8634, 9915, 662, 7273, 84844, 11144, 25777, 10514, 23140, 6720, 23293, 9533, 55588, 6597, 84292, 9801, 220988
nucleocytoplasmic shuttling complex	0.00879631	2	0.19	NUP62, MYBBP1A	23636, 10514
cell fraction	0.00882517	102	81.25	FARSA, MATK, SPINT1, CACNA1G, SLC16A2, ARR3, EFNB1, EPHX1, PCDHAC2, ChGn, LYPLA1, GBA2, RAP1GAP, GARS, DGAT2, AIP, CYP39A1, CD164, CGA, ITM2B, MAP4K2, KCNF1, EPHX2, SLC17A2, SNCB, XRCC6, HMOX1, DPP10, H6PD, OPRD1, EBAG9, CYB5A, HCN2, MADCAM1, ADORA2A, OTOF, GP2, CLN3, CORO7, DOC2A, CAV1, STX16, GNAS, CLCN4, IL17C, CYP3A5, SLC6A2, OAS2, NRP2, CXCL10, JPH2, GNRH2, SLC12A4, ADRB1, CD59, CYP2R1, LRP1, SLC39A1, ERO1L, CYP21A2, SLC39A7, HSD17B8, PDE4D, ARRB1, APP, SLC34A2, PRSS21, SPCS1, DAG1, CYP11B1, LRP12, CYP46A1, STT3A, SPCS3, ABCG1, SLC1A6, PTGS1, STRN4, KCNN4, LHB, PAFAH1B3, UACA, HCN4, ROS1, MAP3K12, GRIK3, SH3KBP1, BCL2L10, MAN1A2, MGAT2, FAS, PLCE1, SLC18A3, TPST2, APLP1, CYP2F1, CYP4F12, SLC18A2, RYR3, SLC16A5, LILRB2, SAG	2193, 4145, 6692, 8913, 6567, 407, 1947, 2052, 56134, 55790, 10434, 57704, 5909, 2617, 84649, 9049, 51302, 8763, 1081, 9445, 5871, 3754, 2053, 10246, 6620, 2547, 3162, 57628, 9563, 4985, 9166, 1528, 610, 8174, 135, 9381, 2813, 1201, 79585, 8448, 857, 8675, 2778, 1183, 27189, 1577, 6530, 4939, 8828, 3627, 57158, 2797, 6560, 153, 966, 120227, 4035, 27173, 30001, 1589, 7922, 7923, 5144, 408, 351, 10568, 10942, 28972, 1605, 1584, 29967, 10858, 3703, 60559, 9619, 6511, 5742, 29888, 3783, 3972, 5050, 55075, 10021, 6098, 7786, 2899, 30011, 10017, 10905, 4247, 355, 51196, 6572, 8459, 333, 1572, 66002, 6571, 6263, 9121, 10288, 6295
intracellular organelle	0.00952347	720	678.89	RFX2, LSM7, LONP1, ZBTB7A, TLE2, MAN2B1, ZNF564, ZNF709, CACNA1A, ZNF442, RANBP3, TIMM13, NDUFA11, MLLT1, POLRMT, REXO1, NUT, AP4E1, ERBB2IP, XRCC4, F2RL1, AKAP1, SOX9, SLC25A43, ZIC3, RPL10, SLC25A14, DUSP9, ZFAND2A, NDUFA4, CDCA7L, INTS1, HOXA10, MYO1G, TMED4, SETX, CIZ1, DNAI1, AGPAT2, SMARCA2, PTGES2, JMJD2C, DMRT1, HACL1, KIF9, AMT, NICN1, FOXE1, ANP32B, CCIN, HDAC6, HNRPH2, MID1IP1, NHS, KIF4A, COG2, EPHX1, FOXI1, RBM27, SGCD, ChGn, ZNF395, NKX3-1, LYPLA1, BAG1, GBA2, TLN1, APBA1, ZNF324, ZNF324B,	5990, 51690, 9361, 51341, 7089, 4125, 163050, 163051, 773, 79973, 8498, 26517, 126328, 4298, 5442, 57455, 256646, 23431, 55914, 7518, 2150, 8165, 6662, 203427, 7547, 6134, 9016, 1852, 90637, 4697, 55536, 26173, 3206, 64005, 222068, 23064, 25792, 27019, 10555, 6595, 80142, 23081, 1761, 26061, 64147, 275, 84276, 2304, 10541, 881, 10013, 3188, 58526, 4810, 24137, 22796, 2052, 2299, 54439, 6444, 55790, 55893, 4824,

				ZIK1, RUVBL2, PRMT1, TSEN34, U2AF2, RCP9, YKT6, DBNL, RAMP3, HSPA4L, ZNF330, DCK, E2F2, USP48, DDOST, KIF17, EVX1, ANLN, AHR, GARS, RECQL5, LYK5, SFRS1, HOXB4, SKAP1, SC65, HCRT, PHF12, HAP1, ETV4, NUFIP2, SERPINH1, PACS1, SF3B2, NDUFV1, PRSS23, DGAT2, SIPA1, ELMOD1, CCND1, MRPS18A, CYP39A1, TFEB, MRPL14, TRFP, ATG5, CD164, PREP, BACH2, SUGT1, NUDT8, B3GAT3, CDCA5, SLC29A2, MAP4K2, MTA3, MSH6, GRHL1, GATA4, ZNF251, EPHX2, PLEC1, SLC25A37, PTK2B, FBXO25, PLEKHA2, HOOK3, RPL8, TXNDC5, DEK, KIF13A, NEDD9, PBX2, RXRB, SSR1, TFAP2A, RAB24, SNCB, MGAT1, SUB1, ARRB2, SIRT7, MIS12, ST6GALNAC2, JMJD6, ALG6, SCP2, CDCA8, AK3L1, RLF, YBX1, FOXD3, HMG2L1, TRIOBP, CBY1, XRCC6, TSPO, PARVG, ACO2, TOMM22, HMOX1, BIK, UBE3C, ZBTB7B, TARS2, MTX1, RAG1AP1, MRPS21, CREB3L4, GYPC, ZAK, SSB, GAD1, MYO3B, CDCA7, MYO1B, PPIG, PMS1, KIF5C, PER3, RCC1, SESN2, RPL11, EPB41, SH3BGRL3, H6PD, ZCCHC17, ATPIF1, CAMTA1, SURB7, EMG1, DIABLO, CLIP1, TBX5, MSI1, SSH1, RAB35, LHX5, CHD7, SULF1, SGK3, SOX17, MRPL15, ZFP161, MRCL3, NOL4, CYB5A, DSC2, TCF4, L3MBTL4, ST8SIA5, ESCO1, DUSP11, FLJ10081, SUCLG1, PTBP1, ZNF497, AZU1, SF3A2, CHAF1A, SNRPD3, ROCK2, MPV17, PPM1G, RNPS1, ZKSCAN2, CLN3, GTF3C1, KIAA0430, CORO7, GGA2, DOC2A, KCTD13, CDIPT, SSX2IP, SYT6, ANKRD13C, ZNF277P, FLJ25778, CAV1, ARPC1B, DLD, STX16, GNAS, MYT1, HSPD1, TNS1, RPS7, PER2, HDLBP, ZNF142, GTF3C3, IDH3G, UPF3B, SYTL4, TMSB4X, BEX2, GLA, GAN, CES2, NUTF2, CTBP2, SEC61A2, MLLT10, BNIP3, GTPBP4, VIM, CAMK1D, MCM7, CYP3A5, MTERF, CYLD, C14orf4, TRIP11, MTAC2D1, PPP1R13B, BCL11B, ALDH6A1, ABCD4, SUZ12, DHX8, AP2B1, RAMP2, RARA, LHX1, SIL1, TAF7, HINT1, IRF1, ARTS-1, P4HA2, PJA2, SETD1B, ALDH2, SETD8, HCFC2, DAO, ABCB10, HNRPU, LIN9, PARP1, SAMD11, CYC1, ZNF696, RNF13, PARP14, SEC22A, MCM2, ATG16L1, BCS1L, CTDSP1, STK36, PNKD, CEP350, ATF3, SMG7, KLHL20, CTSE, SOAT1, RASSF2, APBA2BP, E2F1, MYOD1, TUB, SYT9, NUCB2, DNHD1, PARVA, P53AIP1, FEZ1, GAK, HOP, COX18, LYAR, WDR1, ZNF217, JPH2, CTCFL, CDS2, VPS16, GM632, USP39, NOTO, PCBP1, POLE4, HTRA2, SNRPB2, NCOA3, ASXL1, HM13, PCSK2, SNAI1, DNTTIP1, MPHOSPH6, MAPK3, ZNF629, FHOD1, CASP7, SFXN2, PAOX, PDCD4, EMX2, PKP2, HDAC7A, PTHLH, PHB2, CHD4, MLF2, BDNF, NUP98, PAX6, CYP2R1, CNOT2, TARBP2, KRT18, SNRPF, SYT1, ESPL1, LRP1, RASSF3, CSRP1, LPGAT1, RFWD2, DEDD, TMEM9, PIGM, FLJ16478, APH1A, PYGO2, GBA, SLC39A1, DPM3, PBXIP1, DPF3, CFL2, DHRS1, MYH7, SIX1, NOVA1, ERO1L, TITF1, NFKBIA, PPARD, CYP21A2, HMGA1, ITPR3, HSD17B8, SLC25A30, DIS3, SOHLH2,	10434, 573, 57704, 7094, 320, 25799, 388569, 284307, 10856, 3276, 79042, 11338, 27297, 10652, 28988, 10268, 22824, 27309, 1633, 1870, 84196, 1650, 57576, 2128, 54443, 196, 2617, 9400, 92335, 6426, 3214, 8631, 10609, 3060, 57649, 9001, 2118, 57532, 871, 55690, 10992, 4723, 11098, 84649, 6494, 55531, 595, 55168, 51302, 7942, 64928, 9477, 9474, 8763, 5550, 60468, 10910, 254552, 26229, 113130, 3177, 5871, 57504, 2956, 29841, 2626, 90987, 2053, 5339, 51312, 2185, 26260, 59339, 84376, 6132, 81567, 7913, 63971, 4739, 5089, 6257, 6745, 7020, 53917, 6620, 4245, 10923, 409, 51547, 79003, 10610, 23210, 29929, 6342, 55143, 205, 6018, 4904, 27022, 10042, 11078, 25776, 2547, 706, 64098, 50, 56993, 3162, 638, 9690, 51043, 80222, 4580, 55974, 54460, 148327, 2995, 51776, 6741, 2571, 140469, 83879, 4430, 9360, 5378, 3800, 8863, 1104, 83667, 6135, 2035, 83442, 9563, 51538, 93974, 23261, 9412, 10436, 56616, 6249, 6910, 4440, 54434, 11021, 64211, 55636, 23213, 23678, 64321, 29088, 7541, 10627, 8715, 1528, 1824, 6925, 91133, 29906, 114799, 8446, 55683, 8802, 5725, 162968, 566, 8175, 10036, 6634, 9475, 4358, 5496, 10921, 342357, 1201, 2975, 9665, 79585, 23062, 8448, 253980, 10423, 117178, 148281, 81573, 11179, 254048, 857, 10095, 1738, 8675, 2778, 4661, 3329, 7145, 6201, 8864, 3069, 7701, 9330, 3421, 65109, 94121, 7114, 84707, 2717, 8139, 8824, 10204, 1488, 55176, 8028, 664, 23560, 7431, 57118, 4176, 1577, 7978, 1540, 64207, 9321, 123036, 23368, 64919, 4329, 5826, 23512, 1659, 163, 10266, 5914, 3975, 64374, 6879, 3094, 3659, 51752, 8974, 9867, 23067, 217, 387893, 29915, 1610, 23456, 3192, 286826, 142, 148398, 1537, 79943, 11342, 54625, 26984, 4171, 55054, 617, 58190, 27148, 25953, 9857, 467, 9887, 27252, 1510, 6646, 9770, 63941, 1869, 4654, 7275, 143425, 4925, 144132, 55742, 63970, 9638, 2580, 84525, 285521, 55646, 9948, 7764, 57158, 140690, 8760, 64601, 57473, 10713, 344022, 5093, 56655, 27429, 6629, 8202, 171023, 81502, 5126, 6615, 116092, 10200, 5595, 23361, 29109, 840, 118980,

				LCP1, NEK3, TPT1, KATNAL1, PARP4, CCNU, AYTL2, FGF10, CTNND2, AMACR, ZNF622, RPS23, ALG8, ZNF259, TIMM8B, PHOX2A, MAML2, KDELC2, SLC37A4, CENTD2, APP, CBS, U2AF1, ZNF295, D4S234E, ANAPC4, AGA, RBPJ, KLF3, MSX1, UBE2I, CYBA, CA5A, UBN1, TAF1C, CCNF, PCOLN3, GNPTG, PRDM7, ZNF205, RBM6, SLC25A38, MYRIP, PH-4, PRKCD, SPCS1, ST3GAL6, PPM1M, APPL1, DAG1, QSER1, MARK2, INCENP, NAT10, PRDX5, CREB3L1, RCN1, PRDM11, STIP1, RASSF1, ACTR8, SFMBT1, CBLB, TWF2, DNASE1L3, CYP11B1, RRM2B, TPD52, KLF10, KIF26A, C14orf159, PAPOLA, FCF1, JDP2, CYP46A1, ZNF410, MIZF, FOXRED1, STT3A, SDHD, CHEK1, TBRG1, FLI1, SPCS3, SYNGR2, CBX2, ZFY, ABCG1, NDUFV3, D2HGDH, KLF15, DBR1, RPN1, ANAPC13, CCNL1, CNOT4, COG5, SRPK2, ZNF467, HNRPL, ZNF585B, ZNF585A, PBX4, CALR3, ZNF780A, ZNF573, ZNF599, FKBP8, BRD4, PRRX2, FPGS, PTGS1, LMX1B, LHX2, CDK9, ZNF615, NUP62, CNFN, NAPA, SIX5, ZNF228, UACA, SELS, AP3S2, LCTL, HERC1, AP3B2, SNRPA1, CDC14B, GKAP1, EPB41L4B, PTPN3, FANCC, KLF4, ZNF782, ZBTB2, SHPRH, SOD2, DDO, IRF4, EPM2A, PLAGL1, PPIL4, FOXF2, MAP7, ARPC4, TTLL3, BTD, HDAC11, VHL, LMCD1, EAF1, SETD5, AAAS, KRT1, CSRP2, TMEM10, ANXA11, ALDH18A1, SLC25A28, NOC3L, STMN1, WDR57, RSPO1, SCMH1, TAL1, BCOR, RBBP7, FAM9B, ARX, DYNLT3, PITX2, MAD2L1, DMXL2, RAB27A, BCL2L10, AGPAT7, TP53BP1, MKX, TUBAL3, HNRPF, ZNF33B, CTNNA3, MAN1A2, HIPK1, DDX20, AHCYL1, CDC7, RTCD1, LRRC49, MORF4L1, NR2E3, RPLP1, CLK3, ZNF774, ARNT2, ST6GAL1, TIPARP, ZNF639, SENP5, HSF2, L3MBTL3, TNFAIP3, MRPL18, CANX, BNIP1, TLX3, TTN, WIPF1, IWS1, SLC40A1, FUT8, SYNE2, MGAT2, AP4S1, NFATC4, NCOA4, STOX1, VCL, GHITM, ZNF33A, DDX50, GPR120, BMS1L, THAP7, PHF5A, FOXRED2, DMC1, TPST2, DDX17, L3MBTL2, GSCL, UNC84B, SLC25A1, CRK, MYBBP1A, ZZEF1, SREBF1, SMG6, DLG4, ATPAF2, DSE, TFAP2B, DNAH8, POLR1C, PPP2R5D, BCKDHB, FUT9, IXL, MRPS12, CYP2F1, GAPDHS, BTBD14B, SMARCA4, CRTC1, DNM2, MRPL4, ZNF257, ZNF101, CYP4F12, LOC729745, SLC25A42, CARM1, MORG1, C1orf31, PARG, RDX, ARHGDIG, ACOX1, PPARA, ZNF711, MIB2, NKX1-2, BRCA2, SLC25A15, SFRS5, RHOT2, MRPL38, MYO1F, RPS6, MEF2B, PRDM15, SHANK3, QKI, ZNF674, KRTAP5-8, CORO1A, VRK3, CASZ1, NSL1, MRPL19, HNRPA3, DKFZP686M0199, LOC730394, MAML1, HIVEP2, ZNF783, CENTG3, TRIM35, FAM9C	196743, 27250, 2018, 5318, 51564, 5744, 11331, 1108, 8079, 627, 4928, 5080, 120227, 4848, 6895, 3875, 6636, 6857, 9700, 4035, 283349, 1465, 9926, 64326, 9191, 252839, 93183, 440695, 51107, 90780, 2629, 27173, 54344, 57326, 8110, 1073, 115817, 4625, 6495, 4857, 30001, 7080, 4792, 5467, 1589, 3159, 3710, 7923, 253512, 22894, 54937, 3936, 4752, 7178, 84056, 143, 10309, 79888, 2255, 1501, 23600, 90441, 6228, 79053, 8882, 26521, 401, 84441, 143888, 2542, 116985, 351, 875, 7307, 49854, 27065, 29945, 175, 3516, 51274, 4487, 7329, 1535, 763, 29855, 9013, 899, 5119, 84572, 11105, 7755, 10180, 54977, 25924, 54681, 5580, 28972, 10402, 132160, 26060, 1605, 79832, 2011, 3619, 55226, 25824, 90993, 5954, 56981, 10963, 11186, 93973, 51460, 868, 11344, 1776, 1584, 50484, 7163, 7071, 26153, 80017, 10914, 51077, 122953, 10858, 57862, 25988, 55572, 3703, 6392, 1111, 84897, 2313, 60559, 9144, 84733, 7544, 9619, 4731, 728294, 28999, 51163, 6184, 25847, 57018, 4850, 10466, 6733, 168544, 3191, 92285, 199704, 80714, 125972, 284323, 126231, 148103, 23770, 23476, 51450, 2356, 5742, 4010, 9355, 1025, 284370, 23636, 84518, 8775, 147912, 7771, 55075, 55829, 10239, 197021, 8925, 8120, 6627, 8555, 80318, 54566, 5774, 2176, 9314, 158431, 57621, 257218, 6648, 8528, 3662, 7957, 5325, 85313, 2295, 9053, 10093, 26140, 686, 79885, 7428, 29995, 85403, 55209, 8086, 3848, 1466, 93377, 311, 5832, 81894, 64318, 3925, 9410, 284654, 22955, 6886, 54880, 5931, 171483, 170302, 6990, 5308, 4085, 23312, 5873, 10017, 254531, 7158, 283078, 79861, 3185, 7582, 29119, 10905, 204851, 11218, 10768, 8317, 8634, 54839, 10933, 10002, 6176, 1198, 342132, 9915, 6480, 25976, 51193, 205564, 3298, 84456, 7128, 29074, 821, 662, 30012, 7273, 7456, 55677, 30061, 2530, 23224, 4247, 11154, 4776, 8031, 219736, 7414, 27069, 7581, 79009, 338557, 9790, 80764, 84844, 80020, 11144, 8459, 10521, 83746, 2928, 25777, 6576, 1398, 10514, 23140, 6720, 23293, 1742, 91647, 29940, 7021, 1769, 9533, 5528, 594, 10690, 55588, 6183, 1572, 26330, 112939, 6597,

					23373, 1785, 51073, 113835, 94039, 66002, 729745, 284439, 10498, 84292, 388753, 8505, 5962, 398, 51, 5465, 7552, 142678, 390010, 675, 10166, 6430, 89941, 64978, 4542, 6194, 4207, 63977, 85358, 9444, 641339, 57830, 11151, 51231, 54897, 25936, 9801, 220988, 653238, 730394, 9794, 3097, 155060, 116988, 23087, 171484
organelle	0.00966241	720	678.98	RFX2, LSM7, LONP1, ZBTB7A, TLE2, MAN2B1, ZNF564, ZNF709, CACNA1A, ZNF442, RANBP3, TIMM13, NDUFA11, MLLT1, POLRMT, REXO1, NUT, AP4E1, ERBB2IP, XRCC4, F2RL1, AKAP1, SOX9, SLC25A43, ZIC3, RPL10, SLC25A14, DUSP9, ZFAND2A, NDUFA4, CDCA7L, INTS1, HOXA10, MYO1G, TMED4, SETX, CIZ1, DNAI1, AGPAT2, SMARCA2, PTGES2, JMJD2C, DMRT1, HACL1, KIF9, AMT, NICN1, FOXE1, ANP32B, CCIN, HDAC6, HNRPH2, MID1IP1, NHS, KIF4A, COG2, EPHX1, FOXI1, RBM27, SGCD, ChGn, ZNF395, NKX3-1, LYPLA1, BAG1, GBA2, TLN1, APBA1, ZNF324, ZNF324B, ZIK1, RUVBL2, PRMT1, TSEN34, U2AF2, RCP9, YKT6, DBNL, RAMP3, HSPA4L, ZNF330, DCK, E2F2, USP48, DDOST, KIF17, EVX1, ANLN, AHR, GARS, RECQL5, LYK5, SFRS1, HOXB4, SKAP1, SC65, HCRT, PHF12, HAP1, ETV4, NUFIP2, SERPINH1, PACS1, SF3B2, NDUFV1, PRSS23, DGAT2, SIPA1, ELMOD1, CCND1, MRPS18A, CYP39A1, TFEB, MRPL14, TRFP, ATG5, CD164, PREP, BACH2, SUGT1, NUDT8, B3GAT3, CDCA5, SLC29A2, MAP4K2, MTA3, MSH6, GRHL1, GATA4, ZNF251, EPHX2, PLEC1, SLC25A37, PTK2B, FBXO25, PLEKHA2, HOOK3, RPL8, TXNDC5, DEK, KIF13A, NEDD9, PBX2, RXRB, SSR1, TFAP2A, RAB24, SNCB, MGAT1, SUB1, ARRB2, SIRT7, MIS12, ST6GALNAC2, JMJD6, ALG6, SCP2, CDCA8, AK3L1, RLF, YBX1, FOXD3, HMG2L1, TRIOBP, CBY1, XRCC6, TSPO, PARVG, ACO2, TOMM22, HMOX1, BIK, UBE3C, ZBTB7B, TARS2, MTX1, RAG1AP1, MRPS21, CREB3L4, GYPC, ZAK, SSB, GAD1, MYO3B, CDCA7, MYO1B, PPIG, PMS1, KIF5C, PER3, RCC1, SESN2, RPL11, EPB41, SH3BGRL3, H6PD, ZCCHC17, ATPIF1, CAMTA1, SURB7, EMG1, DIABLO, CLIP1, TBX5, MSI1, SSH1, RAB35, LHX5, CHD7, SULF1, SGK3, SOX17, MRPL15, ZFP161, MRCL3, NOL4, CYB5A, DSC2, TCF4, L3MBTL4, ST8SIA5, ESCO1, DUSP11, FLJ10081, SUCLG1, PTBP1, ZNF497, AZU1, SF3A2, CHAF1A, SNRPD3, ROCK2, MPV17, PPM1G, RNPS1, ZKSCAN2, CLN3, GTF3C1, KIAA0430, CORO7, GGA2, DOC2A, KCTD13, CDIPT, SSX2IP, SYT6, ANKRD13C, ZNF277P, FLJ25778, CAV1, ARPC1B, DLD, STX16, GNAS, MYT1, HSPD1, TNS1, RPS7, PER2, HDLBP, ZNF142, GTF3C3, IDH3G, UPF3B, SYTL4, TMSB4X, BEX2, GLA, GAN, CES2, NUTF2, CTBP2, SEC61A2, MLLT10, BNIP3, GTPBP4,	5990, 51690, 9361, 51341, 7089, 4125, 163050, 163051, 773, 79973, 8498, 26517, 126328, 4298, 5442, 57455, 256646, 23431, 55914, 7518, 2150, 8165, 6662, 203427, 7547, 6134, 9016, 1852, 90637, 4697, 55536, 26173, 3206, 64005, 222068, 23064, 25792, 27019, 10555, 6595, 80142, 23081, 1761, 26061, 64147, 275, 84276, 2304, 10541, 881, 10013, 3188, 58526, 4810, 24137, 22796, 2052, 2299, 54439, 6444, 55790, 55893, 4824, 10434, 573, 57704, 7094, 320, 25799, 388569, 284307, 10856, 3276, 79042, 11338, 27297, 10652, 28988, 10268, 22824, 27309, 1633, 1870, 84196, 1650, 57576, 2128, 54443, 196, 2617, 9400, 92335, 6426, 3214, 8631, 10609, 3060, 57649, 9001, 2118, 57532, 871, 55690, 10992, 4723, 11098, 84649, 6494, 55531, 595, 55168, 51302, 7942, 64928, 9477, 9474, 8763, 5550, 60468, 10910, 254552, 26229, 113130, 3177, 5871, 57504, 2956, 29841, 2626, 90987, 2053, 5339, 51312, 2185, 26260, 59339, 84376, 6132, 81567, 7913, 63971, 4739, 5089, 6257, 6745, 7020, 53917, 6620, 4245, 10923, 409, 51547, 79003, 10610, 23210, 29929, 6342, 55143, 205, 6018, 4904, 27022, 10042, 11078, 25776, 2547, 706, 64098, 50, 56993, 3162, 638, 9690, 51043, 80222, 4580, 55974, 54460, 148327, 2995, 51776, 6741, 2571, 140469, 83879, 4430, 9360, 5378, 3800, 8863, 1104, 83667, 6135, 2035, 83442, 9563, 51538, 93974, 23261, 9412, 10436, 56616, 6249, 6910, 4440, 54434, 11021, 64211, 55636, 23213, 23678, 64321, 29088, 7541, 10627, 8715, 1528, 1824, 6925, 91133, 29906, 114799, 8446, 55683, 8802, 5725, 162968, 566, 8175, 10036, 6634, 9475, 4358, 5496, 10921, 342357, 1201, 2975, 9665, 79585, 23062, 8448, 253980, 10423,

				VIM, CAMK1D, MCM7, CYP3A5, MTERF, CYLD, C14orf4, TRIP11, MTAC2D1, PPP1R13B, BCL11B, ALDH6A1, ABCD4, SUZ12, DHX8, AP2B1, RAMP2, RARA, LHX1, SIL1, TAF7, HINT1, IRF1, ARTS-1, P4HA2, PJA2, SETD1B, ALDH2, SETD8, HCFC2, DAO, ABCB10, HNRPU, LIN9, PARP1, SAMD11, CYC1, ZNF696, RNF13, PARP14, SEC22A, MCM2, ATG16L1, BCS1L, CTDSP1, STK36, PNKD, CEP350, ATF3, SMG7, KLHL20, CTSE, SOAT1, RASSF2, APBA2BP, E2F1, MYOD1, TUB, SYT9, NUCB2, DNHD1, PARVA, P53AIP1, FEZ1, GAK, HOP, COX18, LYAR, WDR1, ZNF217, JPH2, CTCFL, CDS2, VPS16, GM632, USP39, NOTO, PCBP1, POLE4, HTRA2, SNRPB2, NCOA3, ASXL1, HM13, PCSK2, SNAI1, DNTTIP1, MPHOSPH6, MAPK3, ZNF629, FHOD1, CASP7, SFXN2, PAOX, PDCD4, EMX2, PKP2, HDAC7A, PTHLH, PHB2, CHD4, MLF2, BDNF, NUP98, PAX6, CYP2R1, CNOT2, TARBP2, KRT18, SNRPF, SYT1, ESPL1, LRP1, RASSF3, CSRP1, LPGAT1, RFWD2, DEDD, TMEM9, PIGM, FLJ16478, APH1A, PYGO2, GBA, SLC39A1, DPM3, PBXIP1, DPF3, CFL2, DHRS1, MYH7, SIX1, NOVA1, ERO1L, TITF1, NFKBIA, PPARD, CYP21A2, HMGA1, ITPR3, HSD17B8, SLC25A30, DIS3, SOHLH2, LCP1, NEK3, TPT1, KATNAL1, PARP4, CCNU, AYTL2, FGF10, CTNND2, AMACR, ZNF622, RPS23, ALG8, ZNF259, TIMM8B, PHOX2A, MAML2, KDELC2, SLC37A4, CENTD2, APP, CBS, U2AF1, ZNF295, D4S234E, ANAPC4, AGA, RBPJ, KLF3, MSX1, UBE2I, CYBA, CA5A, UBN1, TAF1C, CCNF, PCOLN3, GNPTG, PRDM7, ZNF205, RBM6, SLC25A38, MYRIP, PH-4, PRKCD, SPCS1, ST3GAL6, PPM1M, APPL1, DAG1, QSER1, MARK2, INCENP, NAT10, PRDX5, CREB3L1, RCN1, PRDM11, STIP1, RASSF1, ACTR8, SFMBT1, CBLB, TWF2, DNASE1L3, CYP11B1, RRM2B, TPD52, KLF10, KIF26A, C14orf159, PAPOLA, FCF1, JDP2, CYP46A1, ZNF410, MIZF, FOXRED1, STT3A, SDHD, CHEK1, TBRG1, FLI1, SPCS3, SYNGR2, CBX2, ZFY, ABCG1, NDUFV3, D2HGDH, KLF15, DBR1, RPN1, ANAPC13, CCNL1, CNOT4, COG5, SRPK2, ZNF467, HNRPL, ZNF585B, ZNF585A, PBX4, CALR3, ZNF780A, ZNF573, ZNF599, FKBP8, BRD4, PRRX2, FPGS, PTGS1, LMX1B, LHX2, CDK9, ZNF615, NUP62, CNFN, NAPA, SIX5, ZNF228, UACA, SELS, AP3S2, LCTL, HERC1, AP3B2, SNRPA1, CDC14B, GKAP1, EPB41L4B, PTPN3, FANCC, KLF4, ZNF782, ZBTB2, SHPRH, SOD2, DDO, IRF4, EPM2A, PLAGL1, PPIL4, FOXF2, MAP7, ARPC4, TTLL3, BTD, HDAC11, VHL, LMCD1, EAF1, SETD5, AAAS, KRT1, CSRP2, TMEM10, ANXA11, ALDH18A1, SLC25A28, NOC3L, STMN1, WDR57, RSPO1, SCMH1, TAL1, BCOR, RBBP7, FAM9B, ARX, DYNLT3, PITX2, MAD2L1, DMXL2, RAB27A, BCL2L10, AGPAT7, TP53BP1, MKX, TUBAL3, HNRPF, ZNF33B, CTNNA3, MAN1A2, HIPK1, DDX20, AHCYL1, CDC7, RTCD1, LRRC49, MORF4L1, NR2E3, RPLP1, CLK3, ZNF774, ARNT2, ST6GAL1, TIPARP, ZNF639, SENP5, HSF2, L3MBTL3, TNFAIP3, MRPL18, CANX, BNIP1, TLX3, TTN,	117178, 148281, 81573, 11179, 254048, 857, 10095, 1738, 8675, 2778, 4661, 3329, 7145, 6201, 8864, 3069, 7701, 9330, 3421, 65109, 94121, 7114, 84707, 2717, 8139, 8824, 10204, 1488, 55176, 8028, 664, 23560, 7431, 57118, 4176, 1577, 7978, 1540, 64207, 9321, 123036, 23368, 64919, 4329, 5826, 23512, 1659, 163, 10266, 5914, 3975, 64374, 6879, 3094, 3659, 51752, 8974, 9867, 23067, 217, 387893, 29915, 1610, 23456, 3192, 286826, 142, 148398, 1537, 79943, 11342, 54625, 26984, 4171, 55054, 617, 58190, 27148, 25953, 9857, 467, 9887, 27252, 1510, 6646, 9770, 63941, 1869, 4654, 7275, 143425, 4925, 144132, 55742, 63970, 9638, 2580, 84525, 285521, 55646, 9948, 7764, 57158, 140690, 8760, 64601, 57473, 10713, 344022, 5093, 56655, 27429, 6629, 8202, 171023, 81502, 5126, 6615, 116092, 10200, 5595, 23361, 29109, 840, 118980, 196743, 27250, 2018, 5318, 51564, 5744, 11331, 1108, 8079, 627, 4928, 5080, 120227, 4848, 6895, 3875, 6636, 6857, 9700, 4035, 283349, 1465, 9926, 64326, 9191, 252839, 93183, 440695, 51107, 90780, 2629, 27173, 54344, 57326, 8110, 1073, 115817, 4625, 6495, 4857, 30001, 7080, 4792, 5467, 1589, 3159, 3710, 7923, 253512, 22894, 54937, 3936, 4752, 7178, 84056, 143, 10309, 79888, 2255, 1501, 23600, 90441, 6228, 79053, 8882, 26521, 401, 84441, 143888, 2542, 116985, 351, 875, 7307, 49854, 27065, 29945, 175, 3516, 51274, 4487, 7329, 1535, 763, 29855, 9013, 899, 5119, 84572, 11105, 7755, 10180, 54977, 25924, 54681, 5580, 28972, 10402, 132160, 26060, 1605, 79832, 2011, 3619, 55226, 25824, 90993, 5954, 56981, 10963, 11186, 93973, 51460, 868, 11344, 1776, 1584, 50484, 7163, 7071, 26153, 80017, 10914, 51077, 122953, 10858, 57862, 25988, 55572, 3703, 6392, 1111, 84897, 2313, 60559, 9144, 84733, 7544, 9619, 4731, 728294, 28999, 51163, 6184, 25847, 57018, 4850, 10466, 6733, 168544, 3191, 92285, 199704, 80714, 125972, 284323, 126231, 148103, 23770, 23476, 51450, 2356, 5742, 4010, 9355, 1025, 284370, 23636, 84518, 8775, 147912, 7771, 55075, 55829, 10239, 197021, 8925, 8120, 6627,

				WIPF1, IWS1, SLC40A1, FUT8, SYNE2, MGAT2, AP4S1, NFATC4, NCOA4, STOX1, VCL, GHITM, ZNF33A, DDX50, GPR120, BMS1L, THAP7, PHF5A, FOXRED2, DMC1, TPST2, DDX17, L3MBTL2, GSCL, UNC84B, SLC25A1, CRK, MYBBP1A, ZZEF1, SREBF1, SMG6, DLG4, ATPAF2, DSE, TFAP2B, DNAH8, POLR1C, PPP2R5D, BCKDHB, FUT9, IXL, MRPS12, CYP2F1, GAPDHS, BTBD14B, SMARCA4, CRTC1, DNM2, MRPL4, ZNF257, ZNF101, CYP4F12, LOC729745, SLC25A42, CARM1, MORG1, C1orf31, PARG, RDX, ARHGDIG, ACOX1, PPARA, ZNF711, MIB2, NKX1-2, BRCA2, SLC25A15, SFRS5, RHOT2, MRPL38, MYO1F, RPS6, MEF2B, PRDM15, SHANK3, QKI, ZNF674, KRTAP5-8, CORO1A, VRK3, CASZ1, NSL1, MRPL19, HNRPA3, DKFZP686M0199, LOC730394, MAML1, HIVEP2, ZNF783, CENTG3, TRIM35, FAM9C	8555, 80318, 54566, 5774, 2176, 9314, 158431, 57621, 257218, 6648, 8528, 3662, 7957, 5325, 85313, 2295, 9053, 10093, 26140, 686, 79885, 7428, 29995, 85403, 55209, 8086, 3848, 1466, 93377, 311, 5832, 81894, 64318, 3925, 9410, 284654, 22955, 6886, 54880, 5931, 171483, 170302, 6990, 5308, 4085, 23312, 5873, 10017, 254531, 7158, 283078, 79861, 3185, 7582, 29119, 10905, 204851, 11218, 10768, 8317, 8634, 54839, 10933, 10002, 6176, 1198, 342132, 9915, 6480, 25976, 51193, 205564, 3298, 84456, 7128, 29074, 821, 662, 30012, 7273, 7456, 55677, 30061, 2530, 23224, 4247, 11154, 4776, 8031, 219736, 7414, 27069, 7581, 79009, 338557, 9790, 80764, 84844, 80020, 11144, 8459, 10521, 83746, 2928, 25777, 6576, 1398, 10514, 23140, 6720, 23293, 1742, 91647, 29940, 7021, 1769, 9533, 5528, 594, 10690, 55588, 6183, 1572, 26330, 112939, 6597, 23373, 1785, 51073, 113835, 94039, 66002, 729745, 284439, 10498, 84292, 388753, 8505, 5962, 398, 51, 5465, 7552, 142678, 390010, 675, 10166, 6430, 89941, 64978, 4542, 6194, 4207, 63977, 85358, 9444, 641339, 57830, 11151, 51231, 54897, 25936, 9801, 220988, 653238, 730394, 9794, 3097, 155060, 116988, 23087, 171484

**Extraction Options**	
**Sequence Extraction**	Selected elements with start/end ± bp<br>Complete sequence reverse complement<br>in FASTA format GenBank format
**GeneID Extraction**	
**Excel Extraction**	

**Compare results**	
**Enter GeneIDs**	

	(separated by spaces, returns, or commas)

Further Evaluation of Matches	
**Search PubMed for**	**("gene name") AND**<br>(where "gene name" is automatically extracted from the description lines)<br>**Note:** This works only for annotated genomic DNA sequences from eukaryotes!

For comments, questions, or bug reports, please contact support@genomatix.de.

© Genomatix Software GmbH 1998-2007 - All rights reserved. GEMS Launcher 4.5.0 License Agreement
