# Supplementary material for: Physiology, Pathology and Relatedness of Human Tissues from Gene Expression Meta-Analysis
Source: PLoS One. 2008 Apr 2;3(4):e1880. doi: 10.1371/journal.pone.0001880 (PMC2268968; doi:10.1371/journal.pone.0001880)
Supplement: File S1 — (7.50 MB PDF) [file pone.0001880.s004.pdf]

Index

0. Tissue selective genes....12
0.1 All tissue-selective genes (1601 genes)....12
0.2 GO: Biological Process....113
0.3 GO: Molecular Function....150
0.4 GO: Cellular Component....175
0.5 OMIM....182
1. Hippocampus....221
1.1 Hippocampus selective genes (118 genes)....221
1.2 GO: Biological Process....225
1.3 GO: Molecular Function....236
1.4 GO: Cellular Component....241
2. Bronchial Epitelia....244
2.1 BronchialEpitelia selective genes (77 genes)....244
2.2 GO: Biological Process....246
2.3 GO: Molecular Function....252
2.4 GO: Cellular Component....256
3. LimbMuscle....258
3.1 LimbMuscle selective genes (136 genes)....258
3.2 GO: Biological Process....262
3.3 GO: Molecular Function....271
3.4 GO: Cellular Component....278
4. ExtraocularMuscle....281
4.1 ExtraocularMuscle selective genes (116 genes)....281
4.2 GO: Biological Process....285
4.3 GO: Molecular Function....292
4.4 GO: Cellular Component....298
5. Kidney....301
5.1 Kidney selective genes (141 genes)....301
5.2 GO: Biological Process....305
5.3 GO: Molecular Function....316
5.4 GO: Cellular Component....324
6. SubthalamicNucleus....326
6.1 SubthalamicNucleus selective genes (24 genes)....326
6.2 GO: Biological Process....327
6.3 GO: Molecular Function....331

6.4 GO: Cellular Component....334
7. Skin....336
7.1 Skin selective genes (22 genes)....336
7.2 GO: Biological Process....337
7.3 GO: Molecular Function....340
7.4 GO: Cellular Component....341
8. GlobusPallidus....343
8.1 GlobusPallidus selective genes (16 genes)....343
8.2 GO: Biological Process....344
8.3 GO: Molecular Function....347
8.4 GO: Cellular Component....349
9. CiliaryGanglion....351
9.1 CiliaryGanglion selective genes (6 genes)....351
9.2 GO: Biological Process....352
9.3 GO: Molecular Function....354
9.4 GO: Cellular Component....355
10. AtrioVentricularNode....356
10.1 AtrioVentricularNode selective genes (2 genes)....356
10.2 GO: Biological Process....357
10.3 GO: Molecular Function....358
10.4 GO: Cellular Component....359
11. DRG....360
11.1 DRG selective genes (11 genes)....360
11.2 GO: Biological Process....361
11.3 GO: Molecular Function....365
11.4 GO: Cellular Component....366
12. Placenta....367
12.1 Placenta selective genes (115 genes)....367
12.2 GO: Biological Process....371
12.3 GO: Molecular Function....380
12.4 GO: Cellular Component....385
13. 721-BLymphoblasts....387
13.1 721-BLymphoblasts selective genes (137 genes)....387
13.2 GO: Biological Process....391
13.3 GO: Molecular Function....404
13.4 GO: Cellular Component....410
14. PB-CD8TCells....413

14.1 PB-CD8TCells selective genes (119 genes)....413
14.2 GO: Biological Process....417
14.3 GO: Molecular Function....428
14.4 GO: Cellular Component....433
15. PB-CD4TCells....435
15.1 PB-CD4TCells selective genes (92 genes)....435
15.2 GO: Biological Process....438
15.3 GO: Molecular Function....449
15.4 GO: Cellular Component....453
16. BM-CD71EarlyErythroid....455
16.1 BM-CD71EarlyErythroid selective genes (131 genes)....455
16.2 GO: Biological Process....459
16.3 GO: Molecular Function....469
16.4 GO: Cellular Component....476
17. PB-CD14Monocytes....479
17.1 PB-CD14Monocytes selective genes (163 genes)....479
17.2 GO: Biological Process....484
17.3 GO: Molecular Function....498
17.4 GO: Cellular Component....505
18. PB-CD56NKCells....507
18.1 PB-CD56NKCells selective genes (150 genes)....507
18.2 GO: Biological Process....512
18.3 GO: Molecular Function....524
18.4 GO: Cellular Component....530
19. PB-CD19BCells....533
19.1 PB-CD19BCells selective genes (108 genes)....533
19.2 GO: Biological Process....536
19.3 GO: Molecular Function....546
19.4 GO: Cellular Component....551
20. BM-CD33Myeloid....553
20.1 BM-CD33Myeloid selective genes (162 genes)....553
20.2 GO: Biological Process....558
20.3 GO: Molecular Function....574
20.4 GO: Cellular Component....581
21. BM-CD105Endothelial....583
21.1 BM-CD105Endothelial selective genes (126 genes)....583
21.2 GO: Biological Process....587

21.3 GO: Molecular Function....597
21.4 GO: Cellular Component....603
22. BM-CD34....606
22.1 BM-CD34 selective genes (105 genes)....606
22.2 GO: Biological Process....610
22.3 GO: Molecular Function....623
22.4 GO: Cellular Component....629
23. PB-BDCA4DentriticCells....631
23.1 PB-BDCA4DentriticCells selective genes (134 genes)....631
23.2 GO: Biological Process....635
23.3 GO: Molecular Function....648
23.4 GO: Cellular Component....654
25. MedullaOblongata....656
25.1 MedullaOblongata selective genes (34 genes)....656
25.2 GO: Biological Process....658
25.3 GO: Molecular Function....665
25.4 GO: Cellular Component....668
26. Pons....670
26.1 Pons selective genes (16 genes)....670
26.2 GO: Biological Process....671
26.3 GO: Molecular Function....675
26.4 GO: Cellular Component....677
27. Appendix....679
27.1 Appendix selective genes (5 genes)....679
27.2 GO: Biological Process....680
27.3 GO: Molecular Function....681
27.4 GO: Cellular Component....682
28. TrigeminalGanglion....683
28.1 TrigeminalGanglion selective genes (1 gene)....683
28.2 GO: Biological Process....684
28.3 GO: Molecular Function....685
28.4 GO: Cellular Component....686
29. TemporalLobe....687
29.1 TemporalLobe selective genes (23 genes)....687
29.2 GO: Biological Process....688
29.3 GO: Molecular Function....694
29.4 GO: Cellular Component....696

30. Tongue 698
30.1 Tongue selective genes (72 genes) 698
30.2 GO: Biological Process 700
30.3 GO: Molecular Function..706
30.4 GO: Cellular Component..709
31. UterusCorpus 711
31.1 UterusCorpus selective genes (9 genes) 711
31.2 GO: Biological Process 712
31.3 GO: Molecular Function..715
31.4 GO: Cellular Component..717
32. PsoasMuscle 718
32.1 PsoasMuscle selective genes (47 genes) 718
32.2 GO: Biological Process 720
32.3 GO: Molecular Function..723
32.4 GO: Cellular Component..726
33. FetalLung...728
33.1 FetalLung selective genes (84 genes)..728
33.2 GO: Biological Process 731
33.3 GO: Molecular Function..741
33.4 GO: Cellular Component..745
34. ParietalLobe...747
34.1 ParietalLobe selective genes (23 genes)..747
34.2 GO: Biological Process 748
34.3 GO: Molecular Function..754
34.4 GO: Cellular Component..756
35. Tonsil...758
35. Tonsil selective genes (53 genes) 758
35.2 GO: Biological Process 760
35.3 GO: Molecular Function..766
35.4 GO: Cellular Component..768
36. WholeBlood..770
36.1 WholeBlood selective genes (159 genes)..770
36.2 GO: Biological Process 775
36.3 GO: Molecular Function..788
36.4 GO: Cellular Component..795
37. HBEC 797
37.1 HBEC selective genes (72 genes)..797

37.2 GO: Biological Process 799
37.3 GO: Molecular Function....805
37.4 GO: Cellular Component....809
38. SalivaryGland 811
38.1 SalivaryGland selective genes (41 genes) 811
38.2 GO: Biological Process 813
38.3 GO: Molecular Function....817
38.4 GO: Cellular Component....819
39. AdrenalCortex 821
39.1 AdrenalCortex selective genes (21 genes) 821
39.2 GO: Biological Process 822
39.3 GO: Molecular Function....825
39.4 GO: Cellular Component....827
40. Ovary 828
40.1 Ovary selective genes (13 genes) 828
40.2 GO: Biological Process 829
40.3 GO: Molecular Function....832
40.4 GO: Cellular Component....834
41. Thyroid 835
41.1 Thyroid selective genes (69 genes) 835
41.2 GO: Biological Process 837
41.3 GO: Molecular Function....843
41.4 GO: Cellular Component....847
42. Lung....849
42.1 Lung selective genes (53 genes)....849
42.2 GO: Biological Process 851
42.3 GO: Molecular Function....858
42.4 GO: Cellular Component....862
43. FetalBrain 864
43.1 FetalBrain selective genes (62 genes) 864
43.2 GO: Biological Process 866
43.3 GO: Molecular Function....872
43.4 GO: Cellular Component....875
44. Liver 877
44.1 Liver selective genes (119 genes)....877
44.2 GO: Biological Process 881
44.3 GO: Molecular Function....894

44.4 GO: Cellular Component....901
45. LymphNode....903
45. LymphNode selective genes (35 genes)....903
45.2 GO: Biological Process....904
45.3 GO: Molecular Function....909
45.4 GO: Cellular Component....911
46. Amygdala....912
46.1 Amygdala selective genes (74 genes)....912
46.2 GO: Biological Process....914
46.3 GO: Molecular Function....922
46.4 GO: Cellular Component....927
47. Heart....929
47.1 Heart selective genes (50 genes)....929
47.2 GO: Biological Process....931
47.3 GO: Molecular Function....936
47.4 GO: Cellular Component....940
48. Uterus....942
48.1 Uterus selective genes (47 genes)....942
48.2 GO: Biological Process....944
48.3 GO: Molecular Function....949
48.4 GO: Cellular Component....952
49. Prostate....954
49.1 Prostate selective genes (48 genes)....954
49.2 GO: Biological Process....956
49.3 GO: Molecular Function....961
49.4 GO: Cellular Component....964
50. Pancreas....966
50.1 Pancreas selective genes (49 genes)....966
50.2 GO: Biological Process....968
50.3 GO: Molecular Function....974
50.4 GO: Cellular Component....977
51. PrefrontalCortex....979
51.1 PrefrontalCortex selective genes (66 genes)....979
51.2 GO: Biological Process....981
51.3 GO: Molecular Function....988
51.4 GO: Cellular Component....992
52. CingulateCortex....994

52.1 CingulateCortex selective genes (25 genes)....994
52.2 GO: Biological Process....995
52.3 GO: Molecular Function....1000
52.4 GO: Cellular Component....1003
53. Thymus....1005
53.1 Thymus selective genes (55 genes)....1005
53.2 GO: Biological Process....1007
53.3 GO: Molecular Function....1015
53.4 GO: Cellular Component....1019
54. FetalLiver....1021
54.1 FetalLiver selective genes (102 genes)....1021
54.2 GO: Biological Process....1024
54.3 GO: Molecular Function....1037
54.4 GO: Cellular Component....1043
55. Testis....1045
55.1 Testis selective genes (63 genes)....1045
55.2 GO: Biological Process....1047
55.3 GO: Molecular Function....1053
55.4 GO: Cellular Component....1056
56. Trachea....1058
56.1 Trachea selective genes (63 genes)....1058
56.2 GO: Biological Process....1060
56.3 GO: Molecular Function....1065
56.4 GO: Cellular Component....1068
57. AdrenalGland....1070
57.1 AdrenalGland selective genes (29 genes)....1070
57.2 GO: Biological Process....1071
57.3 GO: Molecular Function....1076
57.4 GO: Cellular Component....1079
58. SpinalCord....1081
58.1 SpinalCord selective genes (30 genes)....1081
58.2 GO: Biological Process....1082
58.3 GO: Molecular Function....1088
58.4 GO: Cellular Component....1091
59. Cerebellum....1093
59.1 Cerebellum selective genes (26 genes)....1093
59.2 GO: Biological Process....1094

59.3 GO: Molecular Function....1099
59.4 GO: Cellular Component....1101
60. PituitaryGland....1103
60.1 PituitaryGland selective genes (21 genes)....1103
60.2 GO: Biological Process....1104
60.3 GO: Molecular Function....1108
60.4 GO: Cellular Component....1110
61. Thalamus....1111
61.1 Thalamus selective genes (36 genes)....1111
61.2 GO: Biological Process....1113
61.3 GO: Molecular Function....1120
61.4 GO: Cellular Component....1122
62. BoneMarrow....1124
62.1 BoneMarrow selective genes (67 genes)....1124
62.2 GO: Biological Process....1126
62.3 GO: Molecular Function....1135
62.4 GO: Cellular Component....1139
63. CardiacMyocytes....1141
63.1 CardiacMyocytes selective genes (40 genes)....1141
63.2 GO: Biological Process....1143
63.3 GO: Molecular Function....1150
63.4 GO: Cellular Component....1153
64. FetalThyroid....1155
64.1 FetalThyroid selective genes (30 genes)....1155
64.2 GO: Biological Process....1156
64.3 GO: Molecular Function....1161
64.4 GO: Cellular Component....1164
65. OlfactoryBulb....1166
65.1 OlfactoryBulb selective genes (25 genes)....1166
65.2 GO: Biological Process....1167
65.3 GO: Molecular Function....1173
65.4 GO: Cellular Component....1176
66. TestisGermCell....1177
66.1 TestisGermCell selective genes (46 genes)....1177
66.2 GO: Biological Process....1179
66.3 GO: Molecular Function....1185
66.4 GO: Cellular Component....1188

67. TestisIntersitial 1189
67.1 TestisIntersitial selective genes (68 genes) 1189
67.2 GO: Biological Process 1191
67.3 GO: Molecular Function 1197
67.4 GO: Cellular Component 1200
68. TestisLeydigCell 1202
68.1 TestisLeydigCell selective genes (45 genes) 1202
68.2 GO: Biological Process 1204
68.3 GO: Molecular Function 1209
68.4 GO: Cellular Component 1212
69. Hypothalamus 1214
69.1 Hypothalamus selective genes (53 genes) 1214
69.2 GO: Biological Process 1216
69.3 GO: Molecular Function 1224
69.4 GO: Cellular Component 1228
70. OccipitalLobe 1230
70.1 OccipitalLobe selective genes (46 genes) 1230
70.2 GO: Biological Process 1232
70.3 GO: Molecular Function 1239
70.4 GO: Cellular Component 1243
71. CerebellumPeduncles 1245
71.1 CerebellumPeduncles selective genes (25 genes) 1245
71.2 GO: Biological Process 1246
71.3 GO: Molecular Function 1251
71.4 GO: Cellular Component 1253
72. SmoothMuscle 1255
72.1 SmoothMuscle selective genes (82 genes) 1255
72.2 GO: Biological Process 1258
72.3 GO: Molecular Function 1266
72.4 GO: Cellular Component 1271
73. CaudateNucleus 1273
73.1 CaudateNucleus selective genes (31 genes) 1273
73.2 GO: Biological Process 1274
73.3 GO: Molecular Function 1280
73.4 GO: Cellular Component 1283
74. WholeBrain 1285
74.1 WholeBrain selective genes (58 genes) 1285

74.2 GO: Biological Process 1287
74.3 GO: Molecular Function 1294
74.4 GO: Cellular Component 1298
75. Islet 1300
75.1 Islet selective genes (60 genes) 1300
75.2 GO: Biological Process 1302
75.3 GO: Molecular Function 1310
75.4 GO: Cellular Component 1313
76. Adipocytes 1314
76.1 Adipocytes selective genes (65 genes) 1314
76.2 GO: Biological Process 1316
76.3 GO: Molecular Function 1323
76.4 GO: Cellular Component 1327
77. TestiSeminiferousTubule 1329
77.1 TestiSeminiferousTubule selective genes (49 genes) 1329
77.2 GO: Biological Process 1331
77.3 GO: Molecular Function 1337
77.4 GO: Cellular Component 1340
78. FetalHeart 1342
78.1 FetalHeart selective genes (189 genes) 1342
78.2 GO: Biological Process 1347
78.3 GO: Molecular Function 1358
78.4 GO: Cellular Component 1366

0. Tissue selective genes

0.1 All tissue-selective genes (1601 genes)

number of tissues	tissue codes	tissue names	Symbol	Description	Chromosome	Chromosome Location	Locus Link	Cytoband	UniGene
1	78	FetalHeart	NBLA00301		4		79804	4q34.1	Hs.61435
1	78	FetalHeart	RDH14	retinol dehydrogenase 14 (all-trans/9-cis/11-cis)	2	-18599477	57665	2p24.2	Hs.288880
1	78	FetalHeart	RYR2	ryanodine receptor 2 (cardiac)	1	235272324	6262	1q42.1-q43	Hs.109514
1	78	FetalHeart	SDHB	succinate dehydrogenase complex, subunit B, iron sulfur (Ip)	1	-17217840	6390	1p36.1-p35	Hs.465924
1	78	FetalHeart	SNX7	sorting nexin 7	1	98899800, 98900005	51375	1p21.3	Hs.197015
1	78	FetalHeart	SORBS2	sorbin and SH3 domain containing 2	4	-186743592	8470	4q35.1	Hs.481342
1	78	FetalHeart	C1orf54	chromosome 1 open reading frame 54	1	148511821	79630	1q21.2	Hs.91283
1	78	FetalHeart	KIAA0774	KIAA0774	13	28496747, 28900776	23281	13q12.3	Hs.591219
1	78	FetalHeart	CASQ2	calsequestrin 2 (cardiac muscle)	1	-116044165	845	1p13.3-p11	Hs.57975
1	78	FetalHeart	REV1L	REV1-like (yeast)	2	-99383370	51455	2q11.1-q11.2	Hs.443077
1	78	FetalHeart	KCNJ8	potassium inwardly-rectifying channel, subfamily J, member 8	12	-21809156	3764	12p11.23	Hs.102308 Hs.619408
1	78	FetalHeart	ARMCX1	armadillo repeat containing, X-linked 1	X	100692169	51309	Xq21.33-q22.2	Hs.9728
1	78	FetalHeart	HIRIP5	HIRA interacting protein 5	2	-69476758	27247	2p15-p13	Hs.430439
1	78	FetalHeart	SLC27A6	solute carrier family 27 (fatty acid transporter), member 6	5	128329111, 128329108	28965	5q23.3	Hs.49765
1	78	FetalHeart	ITGB1BP2	integrin beta 1 binding protein (melusin) 2	X	70438352	26548	Xq12-q13.1	Hs.109999
1	78	FetalHeart	RHEB	Ras homolog enriched in brain	7	-150794031	6009	7q36	Hs.283521 Hs.438823 Hs.568850
1	78	FetalHeart	PPIC	peptidylprolyl isomerase C (cyclophilin C)	5	-122386978	5480	5q23.2	Hs.110364
1	78	FetalHeart	HEPH	hephaestin	X	65300833, 65299387	9843	Xq11-q12	Hs.31720
1	78	FetalHeart	DNAJC15	DnaJ (Hsp40) homolog, subfamily C, member 15	13	42495361	29103	13q14.1	Hs.438830
1	78	FetalHeart	WBP5	WW domain binding protein 5	X	102498035	51186	Xq22.1-q22.2	Hs.533287
1	78	FetalHeart	PDCD5	programmed cell death 5	19	37763943	9141	19q12-q13.1	Hs.443831
1	78	FetalHeart	PDLIM5	PDZ and LIM domain 5	4	95592060	10611	4q22	Hs.480311
1	78	FetalHeart	TNNI3K	TNNI3 interacting kinase	1	74473672	51086	1p31.1	Hs.480085
1	78	FetalHeart	CLGN	calmegin	4	-141529058	1047	4q28.3-q31.1	Hs.86368
1	78	FetalHeart	LAPTM4B	lysosomal associated protein transmembrane 4 beta	8	98856984	55353	8q22.1	Hs.492314
1	78	FetalHeart	GATA4	GATA binding protein 4	8	11599161	2626	8p23.1-p22	Hs.243987

1	78	FetalHeart	FASTKD1	FAST kinase domains 1	2	-170094513	79675	2q31	Hs.529276
1	78	FetalHeart	CORIN	corin, serine peptidase	4	-47290776	10699	4p13-p12	Hs.518618
1	78	FetalHeart	ISOC1	isochorismatase domain containing 1	5	128458340	51015	5q22.1-q33.3	Hs.483296
1	78	FetalHeart	ACYP1	acylphosphatase 1, erythrocyte (common) type	14	-74589682	97	14q24.3	Hs.18573
1	78	FetalHeart	NPPB	natriuretic peptide precursor B	1	-11840108	4879	1p36.2	Hs.219140
1	78	FetalHeart	UBE1C	ubiquitin-activating enzyme E1C (UBA3 homolog, yeast)	3	-69186571	9039	3p24.3-p13	Hs.154320
1	78	FetalHeart	NKX2-5	NK2 transcription factor related, locus 5 (Drosophila)	5	-172591743	1482	5q34	Hs.54473
1	78	FetalHeart	FGF12	fibroblast growth factor 12	3	-193342423	2257	3q28	Hs.584758
1	76	Adipocytes	COMP	cartilage oligomeric matrix protein	19	-18754582	1311	19p13.1	Hs.1584
1	76	Adipocytes	CRLF1	cytokine receptor-like factor 1	19	-18565046	9244	19p12	Hs.114948
1	76	Adipocytes	MYO1D	myosin ID	17	-27843740	4642	17q11-q12	Hs.462777
1	76	Adipocytes	FBLN2	fibulin 2	3	13565624	2199	3p25.1	Hs.198862
1	76	Adipocytes	ME1	malic enzyme 1, NADP(+)-dependent, cytosolic	6	-83977975	4199	6q12	Hs.21160
1	76	Adipocytes	SCD	stearoyl-CoA desaturase (delta-9-desaturase)	10	102096761	6319	10q23-q24	Hs.558396
1	76	Adipocytes	PLIN	perilipin	15	-88008602	5346	15q26	Hs.103253
1	76	Adipocytes	CPM	carboxypeptidase M	12	-67531224	1368	12q14.3	Hs.484551
1	76	Adipocytes	PCOLCE2	procollagen C-endopeptidase enhancer 2	3	-144019401	26577	3q21-q24	Hs.8944
1	76	Adipocytes	WISP2	WNT1 inducible signaling pathway protein 2	20	42777298	8839	20q12-q13.1	Hs.592145
1	76	Adipocytes	COL6A2	collagen, type VI, alpha 2	21	46342469	1292	21q22.3	Hs.420269
1	76	Adipocytes	PDGFRA	platelet-derived growth factor receptor, alpha polypeptide	4	54790203	5156	4q11-q13	Hs.74615
1	76	Adipocytes	CIDEC	cell death-inducing DFFA-like effector c	3	-9883398	63924	3p25.3	Hs.208388 Hs.567562
1	76	Adipocytes	FADS1	fatty acid desaturase 1	11	-61323678	3992	11q12.2-q13.1	Hs.503546
1	75	Islet	PPY	pancreatic polypeptide	17	-39373697	5539	17q21	Hs.558368
1	75	Islet	PCSK1	proprotein convertase subtilisin/kexin type 1	5	-95751874	5122	5q15-q21	Hs.78977
1	72	SmoothMuscle	P4HA2	procollagen-proline, 2-oxoglutarate 4-dioxygenase (proline 4-hydroxylase), alpha polypeptide II	5	-131556204	8974	5q31	Hs.519568
1	72	SmoothMuscle	CCL8	chemokine (C-C motif) ligand 8	17	29670178	6355	17q11.2	Hs.271387
1	72	SmoothMuscle	GREM1	gremlin 1, cysteine knot superfamily, homolog (Xenopus laevis)	15	30797496	26585	15q13-q15	Hs.40098
1	72	SmoothMuscle	CYR61	cysteine-rich, angiogenic inducer, 61	1	85819047	3491	1p31-p22	Hs.8867
1	72	SmoothMuscle	FAP	fibroblast activation protein, alpha	2	-162735447	2191	2q23	Hs.516493
1	72	SmoothMuscle	CXCL3	chemokine (C-X-C motif) ligand 3	4	-75121177	2921	4q21	Hs.89690
1	72	SmoothMuscle	SEC23A	Sec23 homolog A (S. cerevisiae)	14	-38570873	10484	14q21.1	Hs.272927
1	72	SmoothMuscle	DRAM		12	100795235	55332	12q23.2	Hs.525634
1	72	SmoothMuscle	RCN1	reticulocalbin 1, EF-hand calcium binding domain	11	32069266	5954	11p13	Hs.97887
1	72	SmoothMuscle	CDH11	cadherin 11, type 2, OB-cadherin (osteoblast)	16	-63538185	1009	16q22.1	Hs.116471

1	72	SmoothMuscle	CXCL5	chemokine (C-X-C motif) ligand 5	4	-75080223	6374	4q12-q13	Hs.89714
1	72	SmoothMuscle	CCL7	chemokine (C-C motif) ligand 7	17	29621352	6354	17q11.2-q12	Hs.251526
1	72	SmoothMuscle	KIAA1199	KIAA1199	15	78858766	57214	15q24	Hs.459088
1	72	SmoothMuscle	FLJ20701		2	-229596933	55022	2q36.3	Hs.409352
1	72	SmoothMuscle	IL1B	interleukin 1, beta	2	-113303807	3553	2q14	Hs.126256
1	69	Hypothalamus	OXT	oxytocin, prepro- (neurophysin I)	20	3000265	5020	20p13	Hs.113216
1	69	Hypothalamus	PMCH	pro-melanin-concentrating hormone	12	-101114368	5367	12q23-q24	Hs.631652
1	67	TestisIntersitial	CYLC2	cylicin, basic protein of sperm head cytoskeleton 2	9	104797483	1539	9q31.1	Hs.3232
1	67	TestisIntersitial	C20orf195	chromosome 20 open reading frame 195	20	61654816	79025	20q13.33	Hs.197755
1	67	TestisIntersitial	NUP155	nucleoporin 155kDa	5	-37327697	9631	5p13.1	Hs.547696
1	67	TestisIntersitial	ZNF165	zinc finger protein 165	6	28154550	7718	6p21.3	Hs.535177
1	67	TestisIntersitial	SALF		2	48649662	286749	2p16.3	Hs.44385
1	67	TestisIntersitial	AKAP4	A kinase (PRKA) anchor protein 4	X	-99684324	8852	Xp11.2	Hs.97633
1	67	TestisIntersitial	SPA17	sperm autoantigenic protein 17	11	124048949	53340	11q24.2	Hs.286233
1	67	TestisIntersitial	GK2	glycerol kinase 2	4	-80546531	2712	4q13	Hs.98008
1	66	TestisGermCell	SPINLW1	serine peptidase inhibitor-like, with Kunitz and WAP domains 1 (eppin)	20	-43602680	57119	20q12-q13.2	Hs.121084
1	65	OlfactoryBulb	KCNK12	potassium channel, subfamily K, member 12	2	-47601420	56660	2p22-p21	Hs.591586
1	65	OlfactoryBulb	IL17B	interleukin 17B	5	-148734024	27190	5q32-34	Hs.156979
1	64	FetalThyroid	PDE8B	phosphodiesterase 8B	5	76542461	8622	5q14.1	Hs.584830
1	62	BoneMarrow	DEFA4	defensin, alpha 4, corticostatin	8	-6780754	1669	8p23	Hs.591391
1	62	BoneMarrow	PRG3	proteoglycan 3	11	-56900818	10394	11q12	Hs.251386
1	62	BoneMarrow	MMP9	matrix metallopeptidase 9 (gelatinase B, 92kDa gelatinase, 92kDa type IV collagenase)	20	44070953	4318	20q11.2-q13.1	Hs.297413
1	62	BoneMarrow	RNASE3	ribonuclease, RNase A family, 3 (eosinophil cationic protein)	14	20429401	6037	14q24-q31	Hs.73839
1	62	BoneMarrow	CEACAM8	carcinoembryonic antigen-related cell adhesion molecule 8	19	-47776234	1088	19q13.2	Hs.41
1	62	BoneMarrow	MMP8	matrix metallopeptidase 8 (neutrophil collagenase)	11	-102088541	4317	11q22.3	Hs.161839
1	60	PituitaryGland	FSHB	follicle stimulating hormone, beta polypeptide	11	30209138	2488	11p13	Hs.36975
1	60	PituitaryGland	POMC	proopiomelanocortin (adrenocorticotropin/ beta-lipotropin/ alpha-melanocyte stimulating hormone/ beta-melanocyte stimulating hormone/ beta-endorphin)	2	-25237225	5443	2p23.3	Hs.1897
1	60	PituitaryGland	TSHB	thyroid stimulating hormone, beta	1	115373937	7252	1p13	Hs.406687
1	59	Cerebellum	CDR1	cerebellar degeneration-related protein 1, 34kDa	X	-139693090	1038	Xq27.1-q27.2	Hs.571748
1	58	SpinalCord	MAG	myelin associated glycoprotein	19	40474877	4099	19q13.1	Hs.515354
1	58	SpinalCord	FA2H	fatty acid 2-hydroxylase	16	-73304358	79152	16q23	Hs.461329
1	58	SpinalCord	MOBP	myelin-associated oligodendrocyte basic protein	3	39484073	4336	3p22.1	Hs.121333
1	58	SpinalCord	TJP2	tight junction protein 2 (zona occludens 2)	9	70978908	9414	9q13-q21	Hs.50382

1	58	SpinalCord	AGTRL1	angiotensin II receptor-like 1	11	-56757642	187	11q12	Hs.438311 Hs.632110
1	58	SpinalCord	SCRG1		4	-174545874	11341	4q31-q32	Hs.7122
1	57	AdrenalGland	DHCR24	24-dehydrocholesterol reductase	1	-55087887	1718	1p33-p31.1	Hs.498727
1	57	AdrenalGland	SLC26A2	solute carrier family 26 (sulfate transporter), member 2	5	149320492	1836	5q31-q34	Hs.302738
1	56	Trachea	DMBT1	deleted in malignant brain tumors 1	10	124310170	1755	10q25.3-q26.1	Hs.279611
1	56	Trachea	EHF	ets homologous factor	11	34599243	26298	11p12	Hs.502306
1	55	Testis	SLC25A31	solute carrier family 25 (mitochondrial carrier, adenine nucleotide translocator), member 31	4	128871023	83447	4q28.1	Hs.149030
1	55	Testis	DAZL	deleted in azoospermia-like	3	-16603306	1618	3p24.3	Hs.131179
1	55	Testis	TSSK2	testis-specific serine kinase 2	22	17498789	23617	22q11.21	Hs.505323 Hs.643453
1	55	Testis	YBX2	Y box binding protein 2	17	-7132305	51087	17p11.2-p13.1	Hs.567494
1	54	FetalLiver	C5	complement component 5	9	-122754436	727	9q33-q34	Hs.494997
1	54	FetalLiver	MBL2	mannose-binding lectin (protein C) 2, soluble (opsonic defect)	10	-54195146	4153	10q11.2-q21	Hs.499674
1	54	FetalLiver	ANGPTL3	angiopoietin-like 3	1	62835774	27329	1p31.1-p22.3	Hs.209153
1	54	FetalLiver	ACSL4	acyl-CoA synthetase long-chain family member 4	X	-108771221	2182	Xq22.3-q23	Hs.268785
1	54	FetalLiver	SLC2A2	solute carrier family 2 (facilitated glucose transporter), member 2	3	-172196830	6514	3q26.1-q26.2	Hs.167584
1	54	FetalLiver	TDO2	tryptophan 2,3-dioxygenase	4	157044296	6999	4q31-q32	Hs.183671
1	54	FetalLiver	HAL	histidine ammonia-lyase	12	-94891272	3034	12q22-q24.1	Hs.190783
1	54	FetalLiver	SLC38A4	solute carrier family 38, member 4	12	-45444810	55089	12q13	Hs.446077
1	54	FetalLiver	CYP3A7	cytochrome P450, family 3, subfamily A, polypeptide 7	7	-99140596	1551	7q21-q22.1	Hs.111944
1	53	Thymus	PRSS16	protease, serine, 16 (thymus)	6	27323486	10279	6p21	Hs.274407
1	53	Thymus	MGC2463		7	99654806	79037	7q22.1	Hs.521075
1	53	Thymus	TTC21B	tetratricopeptide repeat domain 21B	2	-166439089	79809	2q24.3	Hs.310672
1	53	Thymus	CCL25	chemokine (C-C motif) ligand 25	19	8023933	6370	19p13.2	Hs.310511
1	53	Thymus	CD1A	CD1a molecule	1	156490550	909	1q22-q23	Hs.1309
1	53	Thymus	CCR9	chemokine (C-C motif) receptor 9	3	45903054, 45903022	10803	3p21.3	Hs.225946
1	53	Thymus	ADA	adenosine deaminase	20	-42681577	100	20q12-q13.11	Hs.255479
1	53	Thymus	RAG1	recombination activating gene 1	11	36546138	5896	11p13	Hs.73958
1	53	Thymus	CTSL2	cathepsin L2	9	-98834760	1515	9q22.2	Hs.434529
1	51	PrefrontalCortex	FAM107A	family with sequence similarity 107, member A	3	-58524886	11170	3p21.1	Hs.506357
1	50	Pancreas	LGALS4	lectin, galactoside-binding, soluble, 4 (galectin 4)	19	-43984154	3960	19q13.2	Hs.5302
1	50	Pancreas	SERPINI2	serpin peptidase inhibitor, clade I (pancpin), member 2	3	-168642416	5276	3q26.1-q26.2	Hs.445555
1	50	Pancreas	CELP	carboxyl ester lipase pseudogene	9		1057	9q34.3	Hs.533258
1	50	Pancreas	KLK1	kallikrein 1, renal/pancreas/salivary	19	-56014215	3816	19q13.3	Hs.123107
1	50	Pancreas	PDIA2	protein disulfide isomerase family A, member 2	16	273118	64714	16p13.3	Hs.66581
1	50	Pancreas	ELA3B	elastase 3B, pancreatic	1	22176004	23436	1p36.12	Hs.181289

1	49	Prostate	KLK3	kallikrein 3, (prostate specific antigen)	19	56049982	354	19q13.41	Hs.171995
1	49	Prostate	ACPP	acid phosphatase, prostate	3	133518948	55	3q21-q23	Hs.433060
1	49	Prostate	SEMG1	semenogelin I	20	43269087	6406	20q12-q13.2	Hs.1968
1	49	Prostate	HOXB13	homeobox B13	17	-44157124	10481	17q21.2	Hs.66731
1	49	Prostate	FOLH1	folate hydrolase (prostate-specific membrane antigen) 1	11	-49124763	2346	11p11.2	Hs.380325 Hs.569037 Hs.638729
1	49	Prostate	KLK2	kallikrein 2, prostatic	19	56068500	3817	19q13.41	Hs.515560
1	49	Prostate	TGM4	transglutaminase 4 (prostate)	3	44891130	7047	3p22-p21.33	Hs.438265
1	49	Prostate	SEMG2	semenogelin II	20	43283423	6407	20q12-q13.1	Hs.537218
1	48	Uterus	EPS8	epidermal growth factor receptor pathway substrate 8	12	-15664342	2059	12q13	Hs.591160
1	48	Uterus	COL4A5	collagen, type IV, alpha 5 (Alport syndrome)	X	107569809	1287	Xq22	Hs.369089
1	48	Uterus	PALLD	palladin, cytoskeletal associated protein	4	169654791	23022	4q32.3	Hs.151220
1	48	Uterus	PAEP	progestagen-associated endometrial protein (placental protein 14, pregnancy-associated endometrial alpha-2-globulin, alpha uterine protein)	9	137593424	5047	9q34	Hs.532325 Hs.620596
1	48	Uterus	REV3L	REV3-like, catalytic subunit of DNA polymerase zeta (yeast)	6	-111726972	5980	6q21	Hs.232021
1	48	Uterus	ACTA2	actin, alpha 2, smooth muscle, aorta	10	-90684812	59	10q23.3	Hs.500483
1	46	Amygdala	CPE	carboxypeptidase E	4	166519543	1363	4q32.3	Hs.642731
1	45	LymphNode	CHIT1	chitinase 1 (chitotriosidase)	1	-201452417	1118	1q31-q32	Hs.201688
1	45	LymphNode	CCL21	chemokine (C-C motif) ligand 21	9	-34699003	6366	9p13	Hs.57907
1	45	LymphNode	IGLV3-19	immunoglobulin lambda variable 3-19	22		28797	22q11.2	Hs.449585
1	44	Liver	ASL	argininosuccinate lyase	7	65178210, 65178268	435	7cen-q11.2	Hs.632015
1	44	Liver	CEBPA	CCAAT/enhancer binding protein (C/EBP), alpha	19	-38482775	1050	19q13.1	Hs.643434
1	44	Liver	PROC	protein C (inactivator of coagulation factors Va and VIIIa)	2	127892486	5624	2q13-q14	Hs.224698
1	44	Liver	SDS	serine dehydratase	12	-112314635	10993	12q24.13	Hs.439023
1	44	Liver	CYP2C8	cytochrome P450, family 2, subfamily C, polypeptide 8	10	-96786519	1558	10q23.33	Hs.282871
1	44	Liver	DCXR	dicarbonyl/L-xylulose reductase	17	-77587046	51181	17q25.3	Hs.9857
1	44	Liver	CYP4F11	cytochrome P450, family 4, subfamily F, polypeptide 11	19	-15884181	57834	19p13.1	Hs.187393
1	44	Liver	F12	coagulation factor XII (Hageman factor)	5	-176761746	2161	5q33-qter	Hs.1321
1	44	Liver	HPR	haptoglobin-related protein	16	70654625	3250	16q22.1	Hs.513711
1	44	Liver	CYP2E1	cytochrome P450, family 2, subfamily E, polypeptide 1	10	135190856	1571	10q24.3-qter	Hs.12907
1	44	Liver	AGXT	alanine-glyoxylate aminotransferase (oxalosis I, hyperoxaluria I, glycolicaciduria, serine-pyruvate aminotransferase)	2	241456834	189	2q36-q37	Hs.144567
1	44	Liver	SAA4	serum amyloid A4, constitutive	11	-18209479	6291	11p15.1-p14	Hs.1955
1	44	Liver	HRG	histidine-rich glycoprotein	3	187866491	3273	3q27	Hs.1498

1	44	Liver	SERPINF2	serpin peptidase inhibitor, clade F (alpha-2 antiplasmin, pigment epithelium derived factor), member 2	17	1595035	5345	17p13	Hs.159509
1	44	Liver	PON3	paraoxonase 3	7	-94827121	5446	7q21.3	Hs.440967
1	44	Liver	APOC1	apolipoprotein C-I	19	50109760	341	19q13.2	Hs.110675
1	44	Liver	APOE	apolipoprotein E	19	50100878	348	19q13.2	Hs.110675
1	44	Liver	C8G	complement component 8, gamma polypeptide	9	138959533	733	9q34.3	Hs.1285
1	44	Liver	GNMT	glycine N-methyltransferase	6	43036477	27232	6p12	Hs.144914
1	44	Liver	ANG	angiogenin, ribonuclease, RNase A family, 5	14	20222608	283	14q11.1-q11.2	Hs.283749
1	44	Liver	SLC27A5	solute carrier family 27 (fatty acid transporter), member 5	19	-63701517	10998	19q13.43	Hs.292177
1	44	Liver	LCAT	lecithin-cholesterol acyltransferase	16	-66531287	3931	16q22.1	Hs.387239
1	44	Liver	INHBE	inhibin, beta E	12	56135362	83729	12q13.3	Hs.632713
1	44	Liver	SERPING1	serpin peptidase inhibitor, clade G (C1 inhibitor), member 1, (angioedema, hereditary)	11	57121602, 57122280	710	11q12-q13.1	Hs.384598
1	44	Liver	CYP1A2	cytochrome P450, family 1, subfamily A, polypeptide 2	15	72828236	1544	15q24	Hs.1361
1	44	Liver	FTCD	formiminotransferase cyclodeaminase	21	-92761725	10841	21q22.3	Hs.415846
1	44	Liver	HCA112		7	150128786	55365	7q36.1	Hs.438823
1	44	Liver	ALDH1L1	aldehyde dehydrogenase 1 family, member L1	3	-127305097	10840	3q21.2	Hs.434435
1	44	Liver	SERPINA4	serpin peptidase inhibitor, clade A (alpha-1 antiproteinase, antitrypsin), member 4	14	94097535	5267	14q31-q32.1	Hs.159628
1	44	Liver	PTMS	parathymosin	12	6745801	5763	12p13	Hs.504613
1	44	Liver	SLC22A1	solute carrier family 22 (organic cation transporter), member 1	6	160462852	6580	6q26	Hs.117367
1	44	Liver	ACOX2	acyl-Coenzyme A oxidase 2, branched chain	3	-58465903	8309	3p14.3	Hs.444959
1	44	Liver	PIPOX	pipecolic acid oxidase	17	24394043	51268	17q11.2	Hs.462585
1	44	Liver	FETUB	fetuin B	3	187840842	26998	3q27	Hs.81073
1	43	FetalBrain	NBEA	neurobeachin	13	34414455	26960	13q13	Hs.491172
1	43	FetalBrain	DCX	doublecortex, lissencephaly, X-linked (doublecortin)	X	-110423663	1641	Xq22.3-q23	Hs.34780
1	43	FetalBrain	STMN4	stathmin-like 4	8	-27149732	81551	8p21.2	Hs.201058
1	43	FetalBrain	MEF2C	MADS box transcription enhancer factor 2, polypeptide C (myocyte enhancer factor 2C)	5	-88051921	4208	5q14	Hs.444409
1	43	FetalBrain	PCDHAC1	protocadherin alpha subfamily C, 1	5	140286485	56135	5q31	Hs.199343
1	43	FetalBrain	PCDHA13	protocadherin alpha 13	5	140242037	56136	5q31	Hs.199343
1	43	FetalBrain	PCDHA12	protocadherin alpha 12	5	140235114	56137	5q31	Hs.199343
1	43	FetalBrain	DACT1	dapper, antagonist of beta-catenin, homolog 1 (Xenopus laevis)	14	58174611	51339	14q23.1	Hs.608548 Hs.48950 Hs.643390
1	43	FetalBrain	NETO2	neuropilin (NRP) and tolloid (TLL)-like 2	16	-45672942	81831	16q11	Hs.444046
1	43	FetalBrain	PCDHAC2	protocadherin alpha subfamily C, 2	5	140326295,	56134	5q31	Hs.199343

						140326535			
1	43	FetalBrain	PCDHA4	protocadherin alpha 4	5	140166855	56144	5q31	Hs.199343
1	43	FetalBrain	SOX11	SRY (sex determining region Y)-box 11	2	5750249	6664	2p25	Hs.432638
1	43	FetalBrain	PCDHA1	protocadherin alpha 1	5	140146059	56147	5q31	Hs.199343
1	43	FetalBrain	PCDHA11	protocadherin alpha 11	5	140228014	56138	5q31	Hs.199343
1	43	FetalBrain	FNBP1L	formin binding protein 1-like	1	93686426	54874	1p22.1	Hs.134060
1	43	FetalBrain	CEP170	centrosomal protein 170kDa	1	-241354353	9859	1q44	Hs.533635<br>Hs.644237
1	43	FetalBrain	MLLT11	myeloid/lymphoid or mixed-lineage leukemia (trithorax homolog, Drosophila), translocated to, 11	1	149298774	10962	1q21	Hs.75823
1	43	FetalBrain	PCDHA7	protocadherin alpha 7	5	140194152	56141	5q31	Hs.199343
1	43	FetalBrain	PCDHA8	protocadherin alpha 8	5	140201090	56140	5q31	Hs.199343
1	42	Lung	SFTPD	surfactant, pulmonary-associated protein D	10	-81687477	6441	10q22.2-q23.1	Hs.253495
1	42	Lung	ACP5	acid phosphatase 5, tartrate resistant	19	-11546476	54	19p13.3-p13.2	Hs.1211
1	42	Lung	CYP1A1	cytochrome P450, family 1, subfamily A, polypeptide 1	15	-72798943	1543	15q22-q24	Hs.72912
1	42	Lung	C10orf10	chromosome 10 open reading frame 10	10	-44791716	11067	10q11.21	Hs.93675
1	42	Lung	TMEM100	transmembrane protein 100	17	-51151988	55273	17q22	Hs.173233
1	42	Lung	CLDN5	claudin 5 (transmembrane protein deleted in velocardiofacial syndrome)	22	-17890549	7122	22q11.21	Hs.505337
1	41	Thyroid	FOXE1	forkhead box E1 (thyroid transcription factor 2)	9	99655357	2304	9q22	Hs.159234
1	41	Thyroid	COL9A3	collagen, type IX, alpha 3	20	60918831	1299	20q13.3	Hs.126248
1	41	Thyroid	TMEM30B	transmembrane protein 30B	14	-60814151	161291	14q23.1	Hs.146180
1	41	Thyroid	CITED2	Cbp/p300-interacting transactivator, with Glu/Asp-rich carboxy-terminal domain, 2	6	-139735091	10370	6q23.3	Hs.82071
1	41	Thyroid	CRABP1	cellular retinoic acid binding protein 1	15	76419757	1381	15q24	Hs.346950
1	41	Thyroid	SLC26A4	solute carrier family 26, member 4	7	107088315	5172	7q31	Hs.571246
1	38	SalivaryGland	CST2	cystatin SA	20	-23752403	1470	20p11.21	Hs.123114
1	38	SalivaryGland	CA6	carbonic anhydrase VI	1	8928508	765	1p36.2	Hs.100322
1	38	SalivaryGland	PROL1	proline rich, lacrimal 1	4	71298281	58503	4q13.3	Hs.631946
1	38	SalivaryGland	SMR3A	submaxillary gland androgen regulated protein 3 homolog A (mouse)	4	71261081	26952	4q13.3	Hs.631947
1	38	SalivaryGland	CST5	cystatin D	20	-23804571	1473	20p11.21	Hs.121489
1	37	HBEC	AMIGO2	adhesion molecule with Ig-like domain 2	12	-45755757	347902	12q13.11	Hs.121520
1	37	HBEC	S100A14	S100 calcium binding protein A14	1	-151853356	57402	1q21.3	Hs.288998
1	37	HBEC	CLCA2	chloride channel, calcium activated, family member 2	1	86662412	9635	1p31-p22	Hs.241551
1	37	HBEC	SART2	squamous cell carcinoma antigen recognized by T cells 2	6	116798881	29940	6q22	Hs.486292
1	37	HBEC	CDH3	cadherin 3, type 1, P-cadherin (placental)	16	67236276	1001	16q22.1	Hs.554598
1	37	HBEC	LAMA3	laminin, alpha 3	18	19707003,<br>19523559	3909	18q11.2	Hs.436367

1	37	HBEC	RAB38	RAB38, member RAS oncogene family	11	-87486078	23682	11q14	Hs.591975
1	37	HBEC	PTHLH	parathyroid hormone-like hormone	12	-56008805	5744	12p12.1-p11.2	Hs.591159
1	37	HBEC	NET1	neuroepithelial cell transforming gene 1	10	5478582	10276	10p15	Hs.25155
1	37	HBEC	GPR87	G protein-coupled receptor 87	3	-152494586	53836	3q24	Hs.591292
1	37	HBEC	UPP1	uridine phosphorylase 1	7	48095270, 48094879	7378	7p12.3	Hs.488240
1	37	HBEC	SFN	stratifin	1	27062219	2810	1p36.11	Hs.523718
1	37	HBEC	NT5E	5'-nucleotidase, ecto (CD73)	6	86216527	4907	6q14-q21	Hs.153952
1	37	HBEC	FGFBP1	fibroblast growth factor binding protein 1	4	-15546289	9982	4p16-p15	Hs.1690
1	37	HBEC	RND3	Rho family GTPase 3	2	-151032954	390	2q23.3	Hs.6838
1	36	WholeBlood	AQP9	aquaporin 9	15	56217770	366	15q22.1-22.2	Hs.104624
1	36	WholeBlood	C21orf7	chromosome 21 open reading frame 7	21	29374743	56911	21q22.3	Hs.222802
1	36	WholeBlood	LPGAT1	lysophosphatidylglycerol acyltransferase 1	1	-209984951	9926	1p36.13-q42.3	Hs.591421
1	36	WholeBlood	IRF1	interferon regulatory factor 1	5	-131846683	3659	5q31.1	Hs.436061
1	36	WholeBlood	NRBF2	nuclear receptor binding factor 2	10	64563058	29982	10q21.2	Hs.449628
1	36	WholeBlood	OSBPL8	oxysterol binding protein-like 8	12	-75269710	114882	12q14	Hs.430849
1	36	WholeBlood	ZNF217	zinc finger protein 217	20	-51617018	7764	20q13.2	Hs.155040
1	36	WholeBlood	CAPZA1	capping protein (actin filament) muscle Z-line, alpha 1	1	112963597	829	1p13.2	Hs.514934
1	36	WholeBlood	PF4V1	platelet factor 4 variant 1	4	74937876	5197	4q12-q21	Hs.72933
1	36	WholeBlood	GBP2	guanylate binding protein 2, interferon-inducible	1	-89345897	2634	1p22.2	Hs.386567
1	36	WholeBlood	IL8RB	interleukin 8 receptor, beta	2	218698990	3579	2q35	Hs.846
1	36	WholeBlood	FPRL1	formyl peptide receptor-like 1	19	56956264, 56958453	2358	19q13.3-q13.4	Hs.99855
1	36	WholeBlood	TMEM49	transmembrane protein 49	17	55139810	81671	17q23.1	Hs.444569
1	35	Tonsil	RGS13	regulator of G-protein signalling 13	1	190871904	6003	1q31.2	Hs.497220
1	32	PsoasMuscle	ACTN3	actinin, alpha 3	11	66070966	89	11q13-q14	Hs.445037
1	32	PsoasMuscle	ANKRD2	ankyrin repeat domain 2 (stretch responsive muscle)	10	99322245	26287		Hs.73708
1	31	UterusCorpus	LEFTY2	left-right determination factor 2	1	-224190926	7044	1q42.1	Hs.450763 Hs.520187
1	31	UterusCorpus	GAS1	growth arrest-specific 1	9	-88749098	2619	9q21.3-q22	Hs.65029
1	30	Tongue	LY6D	lymphocyte antigen 6 complex, locus D	8	-143863299	8581	8q24-qter	Hs.415762
1	30	Tongue	AKR1B10	aldo-keto reductase family 1, member B10 (aldose reductase)	7	133862938	57016	7q33	Hs.116724
1	30	Tongue	SCEL	sciellin	13	77007859	8796	13q22	Hs.534699
1	30	Tongue	PPL	periplakin	16	-4872508	5493	16p13.3	Hs.192233
1	30	Tongue	EMP1	epithelial membrane protein 1	12	13240923	2012	12p12.3	Hs.436298
1	30	Tongue	SLURP1	secreted LY6/PLAUR domain containing 1	8	-143819364	57152	8q24.3	Hs.103505
1	30	Tongue	PITX1	paired-like homeodomain transcription factor 1	5	-134391367	5307	5q31	Hs.84136
1	23	PB-BDCA4DentriticCells	IGLV4-60	immunoglobulin lambda variable 4-60	22		28785	22q11.2	
1	23	PB-BDCA4DentriticCells	CUTL2	cut-like 2 (Drosophila)	12	109956211	23316	12q24.11-q24.12	Hs.124953

1	23	PB-BDCA4DentriticCells	MICAL1	microtubule associated monoxygenase, calponin and LIM domain containing 1	6	-109871962	64780	6q21	Hs.33476
1	23	PB-BDCA4DentriticCells	TLR7	toll-like receptor 7	X	12795122	51284	Xp22.3	Hs.443036
1	23	PB-BDCA4DentriticCells	BTAF1	BTAF1 RNA polymerase II, B-TFIID transcription factor-associated, 170kDa (Mot1 homolog, S. cerevisiae)	10	93673715	9044	10q22-q23	Hs.500526
1	23	PB-BDCA4DentriticCells	UGCG	UDP-glucose ceramide glucosyltransferase	9	113699026	7357	9q31	Hs.304249 Hs.593014
1	23	PB-BDCA4DentriticCells	LILRA4	leukocyte immunoglobulin-like receptor, subfamily A (with TM domain), member 4	19	-59536504	23547	19q13.4	Hs.406708
1	23	PB-BDCA4DentriticCells	SIDT1	SID1 transmembrane family, member 1	3	114734182	54847	3q13.2	Hs.591291
1	23	PB-BDCA4DentriticCells	SCT	secretin	11	-616312	6343	11p15.5	Hs.632324
1	23	PB-BDCA4DentriticCells	FCHSD2	FCH and double SH3 domains 2	11	-72225438	9873	11q13.4	Hs.577053
1	23	PB-BDCA4DentriticCells	UBE2J1	ubiquitin-conjugating enzyme E2, J1 (UBC6 homolog, yeast)	6	-90093063	51465	6q15	Hs.163776
1	23	PB-BDCA4DentriticCells	GNA15	guanine nucleotide binding protein (G protein), alpha 15 (Gq class)	19	3087229	2769	19p13.3	Hs.73797
1	23	PB-BDCA4DentriticCells	IGKV4-1	immunoglobulin kappa variable 4-1	2		28908	2p12	
1	22	BM-CD34	ERG	v-ets erythroblastosis virus E26 oncogene like (avian)	21	-38675670	2078	21q22.3	Hs.473819
1	22	BM-CD34	VPREB1	pre-B lymphocyte gene 1	22	20929199	7441	22q11.2|22q11.22	Hs.247979
1	22	BM-CD34	MYC	v-myc myelocytomatosis viral oncogene homolog (avian)	8	128817497	4609	8q24.12-q24.13	Hs.202453
1	22	BM-CD34	ANKRD27	ankyrin repeat domain 27 (VPS9 domain)	19	-37779753	84079	19q13.11	Hs.59236
1	22	BM-CD34	DDX3Y	DEAD (Asp-Glu-Ala-Asp) box polypeptide 3, Y-linked	Y	13526170	8653	Yq11	Hs.99120
1	22	BM-CD34	ITGA2B	integrin, alpha 2b (platelet glycoprotein IIb of IIb/IIIa complex, antigen CD41)	17	-39805075	3674	17q21.32	Hs.411312
1	21	BM-CD105Endothelial	POLE2	polymerase (DNA directed), epsilon 2 (p59 subunit)	14	-49180030	5427	14q21-q22	Hs.162777
1	21	BM-CD105Endothelial	REXO2	REX2, RNA exonuclease 2 homolog (S. cerevisiae)	11	113815386	25996	11q23.1-q23.2	Hs.7527
1	21	BM-CD105Endothelial	CCNB2	cyclin B2	15	57184611	9133	15q22.2	Hs.194698
1	21	BM-CD105Endothelial	PRPF40A	PRP40 pre-mRNA processing factor 40 homolog A (yeast)	2		55660	2q23.3	Hs.591637 Hs.643580
1	21	BM-CD105Endothelial	ATPIF1	ATPase inhibitory factor 1	1	28435197	93974		Hs.590908
1	21	BM-CD105Endothelial	CXCL12	chemokine (C-X-C motif) ligand 12 (stromal cell-derived factor 1)	10	-132569499	6387	10q11.1	Hs.522891
1	21	BM-CD105Endothelial	SHCBP1	SHC SH2-domain binding protein 1	16	-45171970	79801	16q11.2	Hs.123253
1	21	BM-CD105Endothelial	KCNH2	potassium voltage-gated channel, subfamily H (eag-related), member 2	7	-300550428	3757	7q35-q36	Hs.438823
1	21	BM-CD105Endothelial	CCNB1	cyclin B1	5	68498668	891	5q12	Hs.23960
1	21	BM-CD105Endothelial	PAK1IP1	PAK1 interacting protein 1	6	10803173	55003	6p24.2	Hs.310231
1	21	BM-CD105Endothelial	NUP37	nucleoporin 37kDa	12	-100992104	79023	12q23.2	Hs.444276

1	21	BM-CD105Endothelial	AURKA	aurora kinase A	20	-54377851	6790	20q13.2-q13.3	Hs.250822
1	20	BM-CD33Myeloid	JUN	v-jun sarcoma virus 17 oncogene homolog (avian)	1	-59019052	3725	1p32-p31	Hs.525704
1	20	BM-CD33Myeloid	PADI4	peptidyl arginine deiminase, type IV	1	17507278	23569	1p36.13	Hs.522969
1	20	BM-CD33Myeloid	TRIB1	tribbles homolog 1 (Drosophila)	8	126511744	10221	8q24.13	Hs.444947
1	20	BM-CD33Myeloid	MCL1	myeloid cell leukemia sequence 1 (BCL2-related)	1	-148813660	4170	1q21	Hs.632486
1	20	BM-CD33Myeloid	IFI44	interferon-induced protein 44	1	78888103	10561	1p31.1	Hs.82316
1	20	BM-CD33Myeloid	AHR	aryl hydrocarbon receptor	7	17304831	196	7p15	Hs.171189
1	20	BM-CD33Myeloid	AGTPBP1	ATP/GTP binding protein 1	9	-87351274	23287	9q21.33	Hs.494321
1	19	PB-CD19BCells	P2RX5	purinergic receptor P2X, ligand-gated ion channel, 5	17	-7063600	5026	17p13.3	Hs.408615
1	19	PB-CD19BCells	PALM2-AKAP2		9	111582409	445815	9q31-q33	Hs.591908
1	19	PB-CD19BCells	EAF2	ELL associated factor 2	3	123036723	55840	3q13.33	Hs.477325
1	19	PB-CD19BCells	FCRL2	Fc receptor-like 2	1	-155982149	79368	1q21	Hs.437393
1	19	PB-CD19BCells	P2RY10	purinergic receptor P2Y, G-protein coupled, 10	X	78087484	27334	Xq21.1	Hs.296433
1	19	PB-CD19BCells	SP140	SP140 nuclear body protein	2	230798689	11262	2q37.1	Hs.632549
1	19	PB-CD19BCells	BRDG1		4	68107040	26228	4q13.2	Hs.435579
1	19	PB-CD19BCells	NUP88	nucleoporin 88kDa	17	-5230143	4927	17p13.2	Hs.584784
1	19	PB-CD19BCells	CCR6	chemokine (C-C motif) receptor 6	6	167456246	1235	6q27	Hs.46468
1	19	PB-CD19BCells	VPREB3	pre-B lymphocyte gene 3	22	-22424932	29802	22q11.23|22q11	Hs.136713
1	19	PB-CD19BCells	BTG1	B-cell translocation gene 1, anti-proliferative	12	-91061033	694	12q22	Hs.255935 Hs.585087
1	19	PB-CD19BCells	MBD4	methyl-CpG binding domain protein 4	3	-130632482	8930	3q21-q22	Hs.35947
1	19	PB-CD19BCells	OSBPL10	oxysterol binding protein-like 10	3	-31677321	114884	3p22.3	Hs.150122
1	19	PB-CD19BCells	HLA-DOB	major histocompatibility complex, class II, DO beta	6	-32888527	3112	6p21.3	Hs.1802
1	18	PB-CD56NKCells	PTPN12	protein tyrosine phosphatase, non-receptor type 12	7	77004770	5782	7q11.23	Hs.61812
1	18	PB-CD56NKCells	KLRC3	killer cell lectin-like receptor subfamily C, member 3	12	-20915633	3823	12p13	Hs.74082
1	18	PB-CD56NKCells	SPON2	spondin 2, extracellular matrix protein	4	-1150722	10417	4p16.3	Hs.302963
1	18	PB-CD56NKCells	CCDC69	coiled-coil domain containing 69	5	-150540806	26112	5q33.1	Hs.132994
1	18	PB-CD56NKCells	GNPTAB	N-acetylglucosamine-1-phosphate transferase, alpha and beta subunits	12	-100663408	79158	12q23.2	Hs.46850
1	18	PB-CD56NKCells	NCR3	natural cytotoxicity triggering receptor 3	6	-37471702	259197	6p21.3	Hs.509513
1	18	PB-CD56NKCells	LAIR2	leukocyte-associated immunoglobulin-like receptor 2	19	59705824	3904	19q13.4	Hs.43803
1	18	PB-CD56NKCells	PTGDR	prostaglandin D2 receptor (DP)	14	51804180	5729	14q22.1	Hs.306831
1	18	PB-CD56NKCells	CD97	CD97 molecule	19	14353212	976	19p13	Hs.466039
1	18	PB-CD56NKCells	APOBEC3G	apolipoprotein B mRNA editing enzyme, catalytic polypeptide-like 3G	22	37803081	60489	22q13.1-q13.2	Hs.441124
1	18	PB-CD56NKCells	EDG8	endothelial differentiation, sphingolipid G-protein-coupled receptor, 8	19	-10484622	53637	19p13.2	Hs.501561
1	18	PB-CD56NKCells	KLRD1	killer cell lectin-like receptor subfamily D, member 1	12	10351683	3824	12p13	Hs.562457

1	18	PB-CD56NKCells	PPP2R5E	protein phosphatase 2, regulatory subunit B (B56), epsilon isoform	14	-62911107	5529	14q23.1	Hs.334868
1	18	PB-CD56NKCells	IL2RB	interleukin 2 receptor, beta	22	-35851827	3560	22q13|22q13.1	Hs.474787
1	18	PB-CD56NKCells	RAB7L1	RAB7, member RAS oncogene family-like 1	1	-204004300	8934	1q32	Hs.115325
1	17	PB-CD14Monocytes	CACNA2D3	calcium channel, voltage-dependent, alpha 2/delta 3 subunit	3	54131732	55799	3p21.1	Hs.128594
1	17	PB-CD14Monocytes	LILRA3	leukocyte immunoglobulin-like receptor, subfamily A (without TM domain), member 3	19	-59491668	11026	19q13.4	Hs.113277
1	17	PB-CD14Monocytes	ITGB2	integrin, beta 2 (complement component 3 receptor 3 and 4 subunit)	21	-45130298	3689	21q22.3	Hs.375957
1	17	PB-CD14Monocytes	P2RY5	purinergic receptor P2Y, G-protein coupled, 5	13	-47883184	10161	13q14	Hs.123464
1	17	PB-CD14Monocytes	FLJ11151		16	-12664437	55313	16p13.12	Hs.460002
1	17	PB-CD14Monocytes	RCBTB2	regulator of chromosome condensation (RCC1) and BTB (POZ) domain containing protein 2	13	-47961101	1102	13q14.3	Hs.25447
1	16	BM-CD71EarlyErythroid	RHAG	Rh-associated glycoprotein	6	-49680855	6005	6p21.1-p11	Hs.120950
1	16	BM-CD71EarlyErythroid	ERMAP	erythroblast membrane-associated protein (Scianna blood group)	1	43055362, 43063835	114625	1p34.2	Hs.439437
1	16	BM-CD71EarlyErythroid	TOP1	topoisomerase (DNA) I	20	39090875	7150	20q12-q13.1	Hs.592136
1	16	BM-CD71EarlyErythroid	PIP5K1B	phosphatidylinositol-4-phosphate 5-kinase, type I, beta	9	70547029, 70510435	8395	9q13	Hs.534371
1	16	BM-CD71EarlyErythroid	CAT	catalase	11	34417053	847	11p13	Hs.502302
1	16	BM-CD71EarlyErythroid	CENPF	centromere protein F, 350/400ka (mitosin)	1	212843154	1063	1q32-q41	Hs.497741
1	16	BM-CD71EarlyErythroid	PIM1	pim-1 oncogene	6	37245963	5292	6p21.2	Hs.81170
1	16	BM-CD71EarlyErythroid	SLC7A5	solute carrier family 7 (cationic amino acid transporter, y+ system), member 5	16	-86421130	8140	16q24.3	Hs.513797
1	16	BM-CD71EarlyErythroid	HBLD2	HESB like domain containing 2	9	-88069283	81689	9q21.33	Hs.449291
1	16	BM-CD71EarlyErythroid	GATA1	GATA binding protein 1 (globin transcription factor 1)	X	48529905	2623	Xp11.23	Hs.765
1	16	BM-CD71EarlyErythroid	KLF13	Kruppel-like factor 13	15	29406374	51621	15q12	Hs.525752
1	16	BM-CD71EarlyErythroid	MARCH3	membrane-associated ring finger (C3HC4) 3	5	-126233453	115123	5q23.2	Hs.132441
1	16	BM-CD71EarlyErythroid	RPIA	ribose 5-phosphate isomerase A (ribose 5-phosphate epimerase)	2	88772290	22934	2p11.2	Hs.469264
1	16	BM-CD71EarlyErythroid	RHD	Rh blood group, D antigen	1	25471567	6007	1p36.11	Hs.643556
1	16	BM-CD71EarlyErythroid	SLC14A1	solute carrier family 14 (urea transporter), member 1 (Kidd blood group)	18	41558156	6563	18q11-q12	Hs.101307
1	16	BM-CD71EarlyErythroid	BZRPL1	benzodiazapine receptor (peripheral)-like 1	6	41118270	222642	6p21.1	Hs.357392
1	16	BM-CD71EarlyErythroid	ACSL6	acyl-CoA synthetase long-chain family member 6	5	-131317050	23305	5q31	Hs.14945
1	16	BM-CD71EarlyErythroid	NP	nucleoside phosphorylase	14	20007404	4860	14q13.1	Hs.75514
1	16	BM-CD71EarlyErythroid	CLIC2	chloride intracellular channel 2	X	-154158695	1193	Xq28	Hs.632837
1	16	BM-CD71EarlyErythroid	GCLC	glutamate-cysteine ligase, catalytic subunit	6	-53470100	2729	6p12	Hs.271264

1	16	BM-CD71EarlyErythroid	TSPAN5	tetraspanin 5	4	-99612434	10098	4q23	Hs.591706
1	16	BM-CD71EarlyErythroid	H1F0	H1 histone family, member 0	22	36531059	3005	22q13.1	Hs.226117
1	16	BM-CD71EarlyErythroid	CTSE	cathepsin E	1	204484081	1510	1q31	Hs.1355
1	16	BM-CD71EarlyErythroid	BMP2K	BMP2 inducible kinase	4	79916555	55589	4q21.21	Hs.146551
1	16	BM-CD71EarlyErythroid	LOC645719		1		645719	1p36.13	Hs.631865
1	16	BM-CD71EarlyErythroid	RIOK3	RIO kinase 3 (yeast)	18	19287253	8780	18q11.2	Hs.445511
1	16	BM-CD71EarlyErythroid	ASCC2	activating signal cointegrator 1 complex subunit 2	22	-28514602	84164	22q12.1	Hs.517438
1	16	BM-CD71EarlyErythroid	PCAF	p300/CBP-associated factor	3	20056527	8850	3p24	Hs.533055
1	16	BM-CD71EarlyErythroid	RBM38	RNA binding motif protein 38	20	55399869	55544	20q13.31	Hs.236361
1	16	BM-CD71EarlyErythroid	BCL2L1	BCL2-like 1	20	-29715923	598	20q11.21	Hs.516966
1	16	BM-CD71EarlyErythroid	TMCC2	transmembrane and coiled-coil domain family 2	1	203463713	9911	1q32.1	Hs.6360
1	16	BM-CD71EarlyErythroid	TESC	tescalcin	12	-115961112	54997	12q24.22	Hs.525709
1	16	BM-CD71EarlyErythroid	HIST1H4C	histone 1, H4c	6	26212154	8364	6p21.3	Hs.46423
1	16	BM-CD71EarlyErythroid	ALAD	aminolevulinate, delta-, dehydratase	9	-115188413	210	9q33.1	Hs.1227
1	16	BM-CD71EarlyErythroid	RSAD2	radical S-adenosyl methionine domain containing 2	2	6935246	91543	2p25.2	Hs.17518
1	16	BM-CD71EarlyErythroid	EIF2AK1	eukaryotic translation initiation factor 2-alpha kinase 1	7	-6029990	27102	7p22	Hs.520205
1	16	BM-CD71EarlyErythroid	ICAM4	intercellular adhesion molecule 4 (Landsteiner-Wiener blood group)	19	10258649	3386	19p13.2-cen	Hs.631609
1	16	BM-CD71EarlyErythroid	BNIP3L	BCL2/adenovirus E1B 19kDa interacting protein 3-like	8	26296439	665	8p21	Hs.131226
1	15	PB-CD4TCells	TCF7	transcription factor 7 (T-cell specific, HMG-box)	5	133478300, 133479214, 133479325	6932	5q31.1	Hs.573153
1	15	PB-CD4TCells	TRAT1	T cell receptor associated transmembrane adaptor 1	3	110024320	50852	3q13	Hs.138701
1	15	PB-CD4TCells	UBASH3A	ubiquitin associated and SH3 domain containing, A	21	42697087	53347	21q22.3	Hs.473912
1	14	PB-CD8TCells	GOLGA8A	golgi autoantigen, golgin subfamily a, 8A	15	-32458563	23015	15q11.2	Hs.182982
1	14	PB-CD8TCells	GPR171	G protein-coupled receptor 171	3	-152398315	29909	3q25.1	Hs.549152
1	14	PB-CD8TCells	NSUN5C	NOL1/NOP2/Sun domain family, member 5C	7	-72056768	260294	7q11.23	Hs.510927 Hs.632322
1	14	PB-CD8TCells	RNF125	ring finger protein 125	18	27852442	54941	18q12.1	Hs.272800 Hs.638727
1	14	PB-CD8TCells	EDG1	endothelial differentiation, sphingolipid G-protein-coupled receptor, 1	1	101475042	1901	1p21	Hs.154210
1	14	PB-CD8TCells	CRTAM	cytotoxic and regulatory T cell molecule	11	122214464	56253	11q22-q23	Hs.159523
1	14	PB-CD8TCells	CD8B	CD8b molecule	2	-173818172	926	2p12	Hs.405667 Hs.610012
1	13	721-BLymphoblasts	ANP32E	acidic (leucine-rich) nuclear phosphoprotein 32 family, member E	1	-148457478	81611	1q21.2	Hs.385913
1	13	721-BLymphoblasts	TP53	tumor protein p53 (Li-Fraumeni syndrome)	17	-7512464	7157	17p13.1	Hs.408312
1	13	721-BLymphoblasts	KIF14	kinesin family member 14	1	-198787929	9928	1q32.1	Hs.3104
1	13	721-BLymphoblasts	PAGE1	P antigen family, member 1 (prostate associated)	X	-49339009	8712	Xp11.23	Hs.128231

1	13	721-BLymphoblasts	CENPM	centromere protein M	22	-40664694	79019	22q13.2	Hs.208912
1	13	721-BLymphoblasts	HMMR	hyaluronan-mediated motility receptor (RHAMM)	5	162820240	3161	5q33.2-qter	Hs.72550
1	13	721-BLymphoblasts	AURKB	aurora kinase B	17	-8048774	9212	17p13.1	Hs.442658
1	13	721-BLymphoblasts	SLAMF1	signaling lymphocytic activation molecule family member 1	1	-158846513	6504	1q22-q23	Hs.523660
1	13	721-BLymphoblasts	KIF11	kinesin family member 11	10	94342970	3832	10q24.1	Hs.8878
1	13	721-BLymphoblasts	CEP55	centrosomal protein 55kDa	10	95246398	55165	10q23.33	Hs.14559
1	13	721-BLymphoblasts	MAGEA1	melanoma antigen family A, 1 (directs expression of antigen MZ2-E)	X	-152134715	4100	Xq28	Hs.72879
1	13	721-BLymphoblasts	GINS2	GINS complex subunit 2 (Psf2 homolog)	16	-84268781	51659	16q24.1	Hs.433180
1	13	721-BLymphoblasts	TMEM97	transmembrane protein 97	17	23670247	27346	17q11.2	Hs.199695
1	13	721-BLymphoblasts	NARG1	NMDA receptor regulated 1	4	140442125	80155	4q31.1	Hs.555985
1	13	721-BLymphoblasts	PSIP1	PC4 and SFRS1 interacting protein 1	9	-30914708	11168	9p22.3	Hs.493516
1	13	721-BLymphoblasts	C1QBP	complement component 1, q subcomponent binding protein	17	-5276822	708	17p13.3	Hs.555866
1	13	721-BLymphoblasts	RAB9A	RAB9A, member RAS oncogene family	X	13617261	9367	Xp22.2	Hs.495704
1	13	721-BLymphoblasts	MRPL35	mitochondrial ribosomal protein L35	2	86280090	51318	2p11.2	Hs.433439
1	13	721-BLymphoblasts	PCID1	PCI domain containing 1 (herpesvirus entry mediator)	11	32561966	10480	11p13	Hs.502244
1	13	721-BLymphoblasts	SOHLH2	spermatogenesis and oogenesis specific basic helix-loop-helix 2	13	-35640353	54937	13q13.3	Hs.124519
1	13	721-BLymphoblasts	CD58	CD58 molecule	1	-116858679	965	1p13	Hs.34341
1	13	721-BLymphoblasts	MAGEA12	melanoma antigen family A, 12	X	-151649951	4111	Xq28	Hs.169246
1	13	721-BLymphoblasts	TARS	threonyl-tRNA synthetase	5	33476654	6897	5p13.2	Hs.481860
1	13	721-BLymphoblasts	IL21R	interleukin 21 receptor	16	27346079, 27321223, 27321923	50615	16p11	Hs.210546
1	13	721-BLymphoblasts	PSMA3	proteasome (prosome, macropain) subunit, alpha type, 3	14	57781345	5684	14q23	Hs.558799
1	13	721-BLymphoblasts	IFI44L	interferon-induced protein 44-like	1	78858736	10964	1p31.1	Hs.389724
1	13	721-BLymphoblasts	MAGEA10	melanoma antigen family A, 10	X	-151053564	4109	Xq28	Hs.18048
1	13	721-BLymphoblasts	MELK	maternal embryonic leucine zipper kinase	9	36562904	9833	9p13.2	Hs.184339
1	13	721-BLymphoblasts	DFNA5	deafness, autosomal dominant 5	7	-24704499	1687	7p15	Hs.520708
1	12	Placenta	PAGE4	P antigen family, member 4 (prostate associated)	X	49480643	9506	Xp11.23	Hs.441038
1	12	Placenta	SEMA3B	sema domain, immunoglobulin domain (Ig), short basic domain, secreted, (semaphorin) 3B	3	50280043	7869	3p21.3	Hs.82222
1	12	Placenta	RASA1	RAS p21 protein activator (GTPase activating protein) 1	5	86599906, 86600521	5921	5q13.3	Hs.553501
1	12	Placenta	KISS1	KiSS-1 metastasis-suppressor	1	-202426092	3814	1q32	Hs.95008
1	12	Placenta	PSG2	pregnancy specific beta-1-glycoprotein 2	19	-48260201	5670	19q13.1-q13.2	Hs.502092
1	12	Placenta	LGALS14	lectin, galactoside-binding, soluble, 14	19	44886785	56891	19q13.2	Hs.24236
1	12	Placenta	RHOBTB3	Rho-related BTB domain containing 3	5	95092798	22836	5q15	Hs.445030

1	12	Placenta	GATA3	GATA binding protein 3	10	8136672	2625	10p15	Hs.524134
1	12	Placenta	TIMP3	TIMP metallopeptidase inhibitor 3 (Sorsby fundus dystrophy, pseudoinflammatory)	22	31526801	7078	22q12.1-q13.2|22q12.3	Hs.297324
1	12	Placenta	ADAM12	ADAM metallopeptidase domain 12 (meltrin alpha)	10	-255413317	8038	10q26.3	Hs.593858
1	12	Placenta	PGF	placental growth factor, vascular endothelial growth factor-related protein	14	-74478292	5228	14q24-q31	Hs.252820
1	12	Placenta	SVEP1	sushi, von Willebrand factor type A, EGF and pentraxin domain containing 1	9		79987	9q32	Hs.522334 Hs.606771
1	12	Placenta	PLAGL1	pleiomorphic adenoma gene-like 1	6	-144303131	5325	6q24-q25	Hs.444975
1	12	Placenta	KRT23	keratin 23 (histone deacetylase inducible)	17	-36332477	25984	17q21.2	Hs.9029
1	12	Placenta	ALPP	alkaline phosphatase, placental (Regan isozyme)	2	232951591	250	2q37	Hs.284255
1	12	Placenta	GCM1	glial cells missing homolog 1 (Drosophila)	6	-53099720	8521	6p21-p12	Hs.28346
1	12	Placenta	PSG6	pregnancy specific beta-1-glycoprotein 6	19	-96197686	5675	19q13.2	Hs.466843
1	12	Placenta	CHSY1	carbohydrate (chondroitin) synthase 1	15	-99533455	22856	15q26.3	Hs.110488
1	12	Placenta	HSD17B1	hydroxysteroid (17-beta) dehydrogenase 1	17	37957509	3292	17q11-q21	Hs.50727
1	12	Placenta	CYP19A1	cytochrome P450, family 19, subfamily A, polypeptide 1	15	-49288963	1588	15q21.1	Hs.511367
1	12	Placenta	PSG9	pregnancy specific beta-1-glycoprotein 9	19	-48449277	5678	19q13.2	Hs.502092
1	12	Placenta	INSL4	insulin-like 4 (placenta)	9	5221418	3641	9p24	Hs.418506
1	12	Placenta	P11		12	-46389794	8909	12q13.1	Hs.997
1	12	Placenta	CSHL1	chorionic somatomammotropin hormone-like 1	17	-59340696	1444	17q24.2	Hs.406754
1	12	Placenta	GH2	growth hormone 2	17	-177933915	2689	17q24.2	Hs.406754
1	12	Placenta	PSG5	pregnancy specific beta-1-glycoprotein 5	19	-48363735	5673	19q13.2	Hs.502092
1	12	Placenta	PLAC1	placenta-specific 1	X	-133527538	10761	Xq26	Hs.496811
1	12	Placenta	MAN1C1	mannosidase, alpha, class 1C, member 1	1	25816545	57134	1p35	Hs.197043
1	12	Placenta	FAM46A	family with sequence similarity 46, member A	6	-82516078	55603	6q14	Hs.10784
1	12	Placenta	PSG11	pregnancy specific beta-1-glycoprotein 11	19	-48203648	5680	19q13.2	Hs.502092
1	12	Placenta	LOC646541		14		646541	14q24.2	
1	12	Placenta	CAPN6	calpain 6	X	-110374987	827	Xq23	Hs.496593
1	12	Placenta	PSG4	pregnancy specific beta-1-glycoprotein 4	19	-48388695	5672	19q13.2	Hs.502092
1	12	Placenta	MS4A4A	membrane-spanning 4-domains, subfamily A, member 4	11	59806661, 59804714	51338	11q12	Hs.325960
1	12	Placenta	PSG1	pregnancy specific beta-1-glycoprotein 1	19	-48063197	5669	19q13.2	Hs.466843
1	12	Placenta	VGLL1	vestigial like 1 (Drosophila)	X	135441976	51442	Xq26.3	Hs.496843
1	12	Placenta	CDKN1C	cyclin-dependent kinase inhibitor 1C (p57, Kip2)	11	-2861440	1028	11p15.5	Hs.106070

1	12	Placenta	HSD3B1	hydroxy-delta-5-steroid dehydrogenase, 3 beta- and steroid delta-isomerase 1	1	119851373	3283	1p13.1	Hs.364941
1	12	Placenta	PSG3	pregnancy specific beta-1-glycoprotein 3	19	-47917634	5671	19q13.2	Hs.466843
1	7	Skin	LOR	loricrin	1	151498802	4014	1q21	Hs.251680
1	7	Skin	SCGB2A2	secretoglobin, family 2A, member 2	11	61794205	4250	11q13	Hs.46452
1	7	Skin	KRT10	keratin 10 (epidermolytic hyperkeratosis, keratosis palmaris et plantaris)	17	-36227895	3858	17q21	Hs.99936
1	7	Skin	KRT2	keratin 2 (epidermal ichthyosis bullosa of Siemens)	12	-51324609	3849	12q11-q13	Hs.707
1	5	Kidney	PEPD	peptidase D	19	-38569698	5184	19q12-q13.2	Hs.36473
1	5	Kidney	C14orf105	chromosome 14 open reading frame 105	14	-57006347	55195	14q23.1	Hs.146040
1	5	Kidney	BHMT	betaine-homocysteine methyltransferase	5	78443438	635	5q13.1-q15	Hs.80756
1	5	Kidney	HRSP12	heat-responsive protein 12	8	-99183742	10247	8q22	Hs.18426
1	5	Kidney	BBOX1	butyrobetaine (gamma), 2-oxoglutarate dioxygenase (gamma-butyrobetaine hydroxylase) 1	11	27019084	8424	11p14.2	Hs.591996
1	5	Kidney	HIBCH	3-hydroxyisobutyryl-Coenzyme A hydrolase	2	-190777604	26275	2q32.2	Hs.128598
1	5	Kidney	PCCA	propionyl Coenzyme A carboxylase, alpha polypeptide	13	99539337	5095	13q32	Hs.80741
1	5	Kidney	SLC13A1	solute carrier family 13 (sodium/sulfate symporters), member 1	7	-122540824	6561	7q31-q32	Hs.489849
1	5	Kidney	NOX4	NADPH oxidase 4	11	-88699160	50507	11q14.2-q21	Hs.371036
1	5	Kidney	UGT1A5	UDP glucuronosyltransferase 1 family, polypeptide A5	2	234286376	54579	2q37	Hs.124112
1	5	Kidney	CUBN	cubilin (intrinsic factor-cobalamin receptor)	10	-16906543	8029	10p12.31	Hs.166206
1	5	Kidney	GPX3	glutathione peroxidase 3 (plasma)	5	150380191	2878	5q23	Hs.386793 Hs.570891
1	5	Kidney	SLC22A6	solute carrier family 22 (organic anion transporter), member 6	11	-62500645	9356	11q13.1-q13.2	Hs.369252
1	5	Kidney	UGT1A10	UDP glucuronosyltransferase 1 family, polypeptide A10	2	234209861	54575	2q37	Hs.124112
1	5	Kidney	UGT1A6	UDP glucuronosyltransferase 1 family, polypeptide A6	2	234266293, 234265059	54578	2q37	Hs.124112
1	5	Kidney	ENPEP	glutamyl aminopeptidase (aminopeptidase A)	4	111616773	2028	4q25	Hs.435765
1	5	Kidney	ALDH6A1	aldehyde dehydrogenase 6 family, member A1	14	-73596625	4329	14q24.3	Hs.293970
1	5	Kidney	SULT1C1	sulfotransferase family, cytosolic, 1C, member 1	2	108271526	6819	2q11.1-q11.2	Hs.436123
1	5	Kidney	FXYD2	FXYD domain containing ion transport regulator 2	11	-117195999	486	11q23	Hs.413137
1	5	Kidney	LRP2	low density lipoprotein-related protein 2	2	-169693106	4036	2q24-q31	Hs.470538
1	5	Kidney	ECHDC3	enoyl Coenzyme A hydratase domain containing 3	10	11824388	79746	10p14	Hs.22242

1	5	Kidney	EGF	epidermal growth factor (beta-urogastrone)	4	111053498	1950	4q25	Hs.419815
1	5	Kidney	CA12	carbonic anhydrase XII	15	-61402783	771	15q22	Hs.210995
1	5	Kidney	TOM1L1	target of myb1-like 1 (chicken)	17	50333202	10040	17q23.2	Hs.153504
1	5	Kidney	PODXL	podocalyxin-like	7	-130835562	5420	7q32-q33	Hs.16426
1	5	Kidney	UGT1A1	UDP glucuronosyltransferase 1 family, polypeptide A1	2	234333657	54658	2q37	Hs.124112
1	5	Kidney	KCNJ15	potassium inwardly-rectifying channel, subfamily J, member 15	21	38550533, 38550739, 38566298	3772	21q22.2	Hs.411299
1	5	Kidney	DPEP1	dipeptidase 1 (renal)	16	88214500	1800	16q24.3	Hs.109
1	5	Kidney	GATM	glycine amidinotransferase (L-arginine:glycine amidinotransferase)	15	-43440615	2628	15q21.1	Hs.75335
1	5	Kidney	UGT1A7	UDP glucuronosyltransferase 1 family, polypeptide A7	2	234255322	54577	2q37	Hs.124112
1	5	Kidney	UMOD	uromodulin (uromucoid, Tamm-Horsfall glycoprotein)	16	-20251874	7369	16p12.3	Hs.164470
1	5	Kidney	NPHS2	nephrosis 2, idiopathic, steroid-resistant (podocin)	1	-177786298	7827	1q25-q31	Hs.412710
1	5	Kidney	CDH16	cadherin 16, KSP-cadherin	16	-65499526	1014	16q22.1	Hs.513660
1	5	Kidney	SLC7A9	solute carrier family 7 (cationic amino acid transporter, y+ system), member 9	19	-38013261	11136	19q13.1	Hs.408567
1	5	Kidney	FLRT3	fibronectin leucine rich transmembrane protein 3	20	-14252642	23767	20p11	Hs.41296
1	5	Kidney	SLC3A1	solute carrier family 3 (cystine, dibasic and neutral amino acid transporters, activator of cystine, dibasic and neutral amino acid transport), member 1	2	44356102	6519	2p16.3	Hs.112916
1	5	Kidney	CFI	complement factor I	4	-110881300	3426	4q25	Hs.312485
1	5	Kidney	UGT1A8	UDP glucuronosyltransferase 1 family, polypeptide A8	2	234191029	54576	2q37	Hs.124112
1	5	Kidney	GALNT11	UDP-N-acetyl-alpha-D-galactosamine:polypeptide N-acetylgalactosaminyltransferase 11 (GalNAc-T11)	7	151353796	63917	7q34-q36	Hs.438823
1	5	Kidney	NQO2	NAD(P)H dehydrogenase, quinone 2	6	2945229	4835	6pter-q12	Hs.533050
1	5	Kidney	EHHADH	enoyl-Coenzyme A, hydratase/3-hydroxyacyl Coenzyme A dehydrogenase	3	-186391107	1962	3q26.3-q28	Hs.429879
1	5	Kidney	XPNPEP2	X-prolyl aminopeptidase (aminopeptidase P) 2, membrane-bound	X	128700626	7512	Xq25	Hs.170499
1	5	Kidney	UGT1A9	UDP glucuronosyltransferase 1 family, polypeptide A9	2	234245282	54600	2q37	Hs.124112
1	5	Kidney	SLC34A1	solute carrier family 34 (sodium phosphate), member 1	5	176744060	6569	5q35	Hs.936
1	5	Kidney	SLC27A2	solute carrier family 27 (fatty acid transporter), member 2	15	48261713	11001	15q21.2	Hs.11729

1	5	Kidney	SLC17A3	solute carrier family 17 (sodium phosphate), member 3	6	-25953307	10786	6p21.3	Hs.327179
1	5	Kidney	FMO1	flavin containing monooxygenase 1	1	169484286	2326	1q23-q25	Hs.1424
1	5	Kidney	PTHR1	parathyroid hormone receptor 1	3	46894239	5745	3p22-p21.1	Hs.1019
1	5	Kidney	UGT1A3	UDP glucuronosyltransferase 1 family, polypeptide A3	2	234302511	54659	2q37	Hs.124112
1	5	Kidney	UGT1A4	UDP glucuronosyltransferase 1 family, polypeptide A4	2	234292176	54657	2q37	Hs.124112
1	5	Kidney	SLC22A8	solute carrier family 22 (organic anion transporter), member 8	11	-62516872	9376	11q11	Hs.266223
1	4	ExtraocularMuscle	MYH13	myosin, heavy polypeptide 13, skeletal muscle	17	-10144907	8735	17p13	Hs.278488
1	4	ExtraocularMuscle	RGS5	regulator of G-protein signalling 5	1	-161378721	8490	1q23.1	Hs.568700
1	4	ExtraocularMuscle	LRTM1	leucine-rich repeats and transmembrane domains 1	3	-54927424	57408	3p14.3	Hs.591668
1	4	ExtraocularMuscle	ITGBL1	integrin, beta-like 1 (with EGF-like repeat domains)	13	100902966	9358	13q33	Hs.643461
1	4	ExtraocularMuscle	PITX2	paired-like homeodomain transcription factor 2	4	-111758030	5308	4q25-q27	Hs.643588
1	4	ExtraocularMuscle	NDUFA5	NADH dehydrogenase (ubiquinone) 1 alpha subcomplex, 5, 13kDa	7	-122968319	4698	7q32	Hs.643550
1	4	ExtraocularMuscle	EFHA1	EF-hand domain family, member A1	13	-20964839	221154	13q12.11	Hs.412103
1	4	ExtraocularMuscle	DKFZP686A01247		4	41057560	22998	4p13	Hs.335163
1	3	LimbMuscle	PKIA	protein kinase (cAMP-dependent, catalytic) inhibitor alpha	8	79590890	5569	8q21.12	Hs.433700
1	3	LimbMuscle	CUTC	cutC copper transporter homolog (E. coli)	10	101481992	51076	10q24.2	Hs.16606
1	3	LimbMuscle	RTN2	reticulon 2	19	-50680390	6253	19q13.32	Hs.47517
1	3	LimbMuscle	LOC653816		6		653816	6p12.3	Hs.485489
1	3	LimbMuscle	DMN	desmuslin	15	97462808	23336	15q26.3	Hs.207106
1	3	LimbMuscle	CAP2	CAP, adenylate cyclase-associated protein, 2 (yeast)	6	17501714	10486	6p22.3	Hs.132902
1	3	LimbMuscle	CACNA1S	calcium channel, voltage-dependent, L type, alpha 1S subunit	1	-199275264	779	1q32	Hs.1294
1	3	LimbMuscle	LRRC2	leucine rich repeat containing 2	3	-93066791	79442	3p21.31	Hs.570630
1	3	LimbMuscle	ACHE	acetylcholinesterase (Yt blood group)	7	-100325551	43	7q22	Hs.154495
1	3	LimbMuscle	HOXC10	homeobox C10	12	52665212	3226	12q13.3	Hs.44276
1	3	LimbMuscle	USP13	ubiquitin specific peptidase 13 (isopeptidase T-3)	3	180853634	8975	3q26.2-q26.3	Hs.591319
1	3	LimbMuscle	FBP2	fructose-1,6-bisphosphatase 2	9	-96360825	8789	9q22.3	Hs.61255
1	3	LimbMuscle	CAND2	cullin-associated and neddylation-dissociated 2 (putative)	3		23066	3p25.1	Hs.343664
1	3	LimbMuscle	UBE2G1	ubiquitin-conjugating enzyme E2G 1 (UBC7 homolog, yeast)	17	-4119262	7326	1q42|17p13.2	Hs.462035
1	3	LimbMuscle	CYB5R1	cytochrome b5 reductase 1	1	-201197626	51706	1p36.13-q41	Hs.334832
1	3	LimbMuscle	FKBP3	FK506 binding protein 3, 25kDa	14	-44654858	2287	14q21.3	Hs.509226
1	3	LimbMuscle	ZFP106	zinc finger protein 106 homolog (mouse)	15	-40492313	64397	15q15.1	Hs.511143

1	3	LimbMuscle	SYNC1	syncoilin, intermediate filament 1	1	-32918564	81493	1p34.3-p33	Hs.642703
1	2	BronchialEpitelia	TEKT2	tektin 2 (testicular)	1	36322262	27285	1p35.3-p34.1	Hs.127111
1	2	BronchialEpitelia	FABP6	fatty acid binding protein 6, ileal (gastrotropin)	5	159558625, 159589014	2172	5q33.3-q34	Hs.519719
1	2	BronchialEpitelia	NTS	neurotensin	12	84792205	4922	12q21	Hs.80962
1	2	BronchialEpitelia	AGBL2	ATP/GTP binding protein-like 2	11	-47637720	79841	11p11.2	Hs.147377
1	2	BronchialEpitelia	SLC6A14	solute carrier family 6 (amino acid transporter), member 14	X	115481817	11254	Xq23-q24	Hs.522109
1	2	BronchialEpitelia	DUOX1	dual oxidase 1	15	43209483	53905	15q15.3	Hs.272813
1	2	BronchialEpitelia	DNAI1	dynein, axonemal, intermediate polypeptide 1	9	34448810	27019	9p21-p13	Hs.112667
1	2	BronchialEpitelia	MUC4	mucin 4, cell surface associated	3	-196959310	4585	3q29	Hs.369646
1	2	BronchialEpitelia	CGI-38		16	-65981214	51673	16q22.1	Hs.534458
1	2	BronchialEpitelia	TMC5	transmembrane channel-like 5	16	19375272	79838	16p12.3	Hs.115838
1	2	BronchialEpitelia	LRRC48	leucine rich repeat containing 48	17	17816923	83450	17p11.2	Hs.579264
1	2	BronchialEpitelia	GABRP	gamma-aminobutyric acid (GABA) A receptor, pi	5	170143342	2568	5q33-q34	Hs.26225
1	2	BronchialEpitelia	SPAG6	sperm associated antigen 6	10	22674404	9576	10p12.2	Hs.7444
1	2	BronchialEpitelia	EVI1	ecotropic viral integration site 1	3	-170285243	2122	3q24-q28	Hs.554762
1	2	BronchialEpitelia	PPAP2C	phosphatidic acid phosphatase type 2C	19	-232045	8612	19p13	Hs.465506
1	2	BronchialEpitelia	CETN2	centrin, EF-hand protein, 2	X	-151746528	1069	Xq28	Hs.82794
1	2	BronchialEpitelia	LZTFL1	leucine zipper transcription factor-like 1	3	-45840449	54585	3p21.3	Hs.30824
1	1	Hippocampus	LGI1	leucine-rich, glioma inactivated 1	10	95507667	9211	10q24	Hs.533670
1	1	Hippocampus	ROPN1L	ropporin 1-like	5	10495014	83853	5p15.2	Hs.381089
1	1	Hippocampus	UBQLN2	ubiquilin 2	X	56606796	29978	Xp11.23-p11.1	Hs.179309
1	1	Hippocampus	NLGN4X	neuroligin 4, X-linked	X	-5818084	57502	Xp22.32-p22.31	Hs.21107
1	1	Hippocampus	RYR3	ryanodine receptor 3	15	31390468	6263	15q14-q15	Hs.369250
1	1	Hippocampus	NFASC	neurofascin homolog (chicken)	1	203064445	23114	1q32.1	Hs.13349
1	1	Hippocampus	ST18	suppression of tumorigenicity 18 (breast carcinoma) (zinc finger protein)	8	-53185951	9705	8q11.23	Hs.147170
1	1	Hippocampus	ST6GALNAC5	ST6 (alpha-N-acetyl-neuraminyl-2,3-beta-galactosyl-1,3)-N-acetylgalactosaminide alpha-2,6-sialyltransferase 5	1	77105773	81849	1p31.1	Hs.303609
1	1	Hippocampus	ZNF312	zinc finger protein 312	3	-62330403	55079	3p14.2	Hs.241523
1	1	Hippocampus	TRIM36	tripartite motif-containing 36	5	-343564200	55521	5q22.3	Hs.519514 Hs.638953
1	1	Hippocampus	BBS10	Bardet-Biedl syndrome 10	12	-75262396	79738	12q21.2	Hs.96322
1	1	Hippocampus	LRP1B	low density lipoprotein-related protein 1B (deleted in tumors)	2	-140705465	53353	2q21.2	Hs.470117
1	1	Hippocampus	TRIM9	tripartite motif-containing 9	14	-101044267	114088	14q22.1	Hs.368928
1	1	Hippocampus	PHLPP	PH domain and leucine rich repeat protein phosphatase	18	58533713	23239	18q21.33	Hs.465337
1	1	Hippocampus	PTBP2	polypyrimidine tract binding protein 2	1	96959965	58155	1p22.1-p21.3	Hs.591430
1	1	Hippocampus	DSCAML1	Down syndrome cell adhesion molecule like 1	11	-116803698	57453		Hs.413137
1	1	Hippocampus	RUFY3	RUN and FYVE domain containing 3	4	71806575, 71806561	22902	4q13.3	Hs.7972

1	1	Hippocampus	SEH1L	SEH1-like (S. cerevisiae)	18	12937982	81929	18p11.21	Hs.301048
1	1	Hippocampus	ARNT2	aryl-hydrocarbon receptor nuclear translocator 2	15	78483746	9915	15q24	Hs.459070
2	76<br>78	Adipocytes<br>FetalHeart	LAMA2	laminin, alpha 2 (merosin, congenital muscular dystrophy)	6	129246034	3908	6q22-q23	Hs.200841
2	76<br>78	Adipocytes<br>FetalHeart	DPT	dermatopontin	1	-166931330	1805	1q12-q23	Hs.80552
2	72<br>76	SmoothMuscle<br>Adipocytes	GBE1	glucan (1,4-alpha-), branching enzyme 1 (glycogen branching enzyme, Andersen disease, glycogen storage disease type IV)	3	-81621547	2632	3p12.3	Hs.436062
2	72<br>76	SmoothMuscle<br>Adipocytes	PXDN	peroxidasin homolog (Drosophila)	2	-1614666	7837	2p25	Hs.332197
2	63<br>72	CardiacMyocytes<br>SmoothMuscle	LOX	lysyl oxidase	5	-121429918	4015	5q23.2	Hs.102267
2	63<br>72	CardiacMyocytes<br>SmoothMuscle	CXCL6	chemokine (C-X-C motif) ligand 6 (granulocyte chemotactic protein 2)	4	74921276	6372	4q21	Hs.164021
2	63<br>72	CardiacMyocytes<br>SmoothMuscle	EFEMP1	EGF-containing fibulin-like extracellular matrix protein 1	2	-55946605	2202	2p16	Hs.76224
2	61<br>69	Thalamus<br>Hypothalamus	SLC17A6	solute carrier family 17 (sodium-dependent inorganic phosphate cotransporter), member 6	11	22316242	57084	11p14.3	Hs.242821
2	60<br>69	PituitaryGland<br>Hypothalamus	DIRAS3	DIRAS family, GTP-binding RAS-like 3	1	-68284234	9077	1p31	Hs.194695
2	59<br>71	Cerebellum<br>CerebellumPeduncles	NEUROD1	neurogenic differentiation 1	2	-182249439	4760	2q32	Hs.72981
2	59<br>71	Cerebellum<br>CerebellumPeduncles	CBLN1	cerebellin 1 precursor	16	-47870201	869	16q12.1	Hs.458423
2	58<br>69	SpinalCord<br>Hypothalamus	MOG	myelin oligodendrocyte glycoprotein	6	29732787,<br>1080116	4340	6p22.1	Hs.141308
2	58<br>69	SpinalCord<br>Hypothalamus	ENPP2	ectonucleotide pyrophosphatase/phosphodiesterase 2 (autotaxin)	8	-120638499	5168	8q24.1	Hs.190977
2	55<br>73	Testis<br>CaudateNucleus	PENK	proenkephalin	8	-57516070	5179	8q23-q24	Hs.339831
2	55<br>68	Testis<br>TestisLeydigCell	SPAG11	sperm associated antigen 11	8	-7295496,<br>7742811, -<br>7306318	10407	8p23-p22	Hs.2717<br>Hs.559506
2	55<br>67	Testis<br>TestisIntersitial	YPEL1	yippee-like 1 (Drosophila)	22	-20381825	29799	22q11.2	Hs.517436
2	55<br>67	Testis<br>TestisIntersitial	CCNA1	cyclin A1	13	35904632	8900	13q12.3-q13	Hs.417050
2	55<br>67	Testis<br>TestisIntersitial	IL13RA2	interleukin 13 receptor, alpha 2	X	-114144795	3598	Xq13.1-q28	Hs.336046
2	55<br>67	Testis<br>TestisIntersitial	TBL2	transducin (beta)-like 2	7	-72621209	26608	7q11.23	Hs.520943
2	54<br>56	FetalLiver<br>Trachea	COL2A1	collagen, type II, alpha 1 (primary osteoarthritis, spondyloepiphyseal dysplasia, congenital)	12	-46653015	1280	12q13.11-q13.2	Hs.408182
2	51<br>74	PrefrontalCortex<br>WholeBrain	ZNF365	zinc finger protein 365	10	63803956,<br>63950212	22891	10q21.2	Hs.22653
2	50<br>75	Pancreas<br>Islet	CPA1	carboxypeptidase A1 (pancreatic)	7	129807590	1357	7q32	Hs.2879

2	50<br>75	Pancreas<br>Islet	PNLIPRP1	pancreatic lipase-related protein 1	10	118340479	5407	10q25.3	Hs.73923
2	50<br>75	Pancreas<br>Islet	CTRB2	chymotrypsinogen B2	16	-73795503,<br>73810397	440387	16q23.1	Hs.610926<br>Hs.632211
2	50<br>75	Pancreas<br>Islet	ELA2A		1	15655810	63036	1p36.21	Hs.631866
2	50<br>75	Pancreas<br>Islet	ELA3A	elastase 3A, pancreatic	1	22200735	10136	1p36.12	Hs.181289
2	50<br>75	Pancreas<br>Islet	TRY6		7		154754	7q34	
2	50<br>75	Pancreas<br>Islet	GP2	glycoprotein 2 (zymogen granule membrane)	16	-20229312	2813	16p12	Hs.53985
2	50<br>75	Pancreas<br>Islet	REG3A	regenerating islet-derived 3 alpha	2	-79237640	5068	2p12	Hs.567312
2	50<br>75	Pancreas<br>Islet	CTRL	chymotrypsin-like	16	-66520974	1506	16q22.1	Hs.9661
2	50<br>75	Pancreas<br>Islet	GCG	glucagon	2	-162707634	2641	2q36-q37	Hs.516494
2	50<br>75	Pancreas<br>Islet	PRSS3	protease, serine, 3 (mesotrypsin)	9	33785558	5646	9p11.2	Hs.128013
2	50<br>75	Pancreas<br>Islet	CLPS	colipase, pancreatic	6	-35870737	1208	6pter-p21.1	Hs.1340
2	50<br>75	Pancreas<br>Islet	PNLIPRP2	pancreatic lipase-related protein 2	10	118370454	5408	10q25.3	Hs.423598
2	50<br>75	Pancreas<br>Islet	PNLIP	pancreatic lipase	10	118295417	5406	10q26.1	Hs.501135
2	50<br>75	Pancreas<br>Islet	CUZD1	CUB and zona pellucida-like domains 1	10	-124581660	50624	10q26.13	Hs.114648
2	50<br>75	Pancreas<br>Islet	REG1B	regenerating islet-derived 1 beta (pancreatic stone protein, pancreatic thread protein)	2	-79165658	5968	2p12	Hs.4158
2	50<br>75	Pancreas<br>Islet	PRSS1	protease, serine, 1 (trypsin 1)	7	142136903	5644	7q32-qter|7q34	Hs.511522
2	50<br>75	Pancreas<br>Islet	REG1A	regenerating islet-derived 1 alpha (pancreatic stone protein, pancreatic thread protein)	2	79201091	5967	2p12	Hs.49407
2	50<br>75	Pancreas<br>Islet	INS	insulin	11	-2137584	3630	11p15.5	Hs.89832
2	50<br>75	Pancreas<br>Islet	PLA2G1B	phospholipase A2, group IB (pancreas)	12	-119244296	5319	12q23-q24.1	Hs.992
2	50<br>75	Pancreas<br>Islet	CPA2	carboxypeptidase A2 (pancreatic)	7	129693959	1358	7q32	Hs.490038
2	50<br>75	Pancreas<br>Islet	ELA2B		1	15675182	51032	1p36.21	Hs.631871
2	50<br>75	Pancreas<br>Islet	CTRC	chymotrypsin C (caldecrin)	1	15637524	11330	1p36.21	Hs.631869
2	50<br>75	Pancreas<br>Islet	SPINK1	serine peptidase inhibitor, Kazal type 1	5	-147184338	6690	5q32	Hs.407856
2	50<br>75	Pancreas<br>Islet	IAPP	islet amyloid polypeptide	12	21417084	3375	12p12.3-p12.1	Hs.46835
2	5<br>78	Kidney<br>FetalHeart	SFRP1	secreted frizzled-related protein 1	8	-41238635	6422	8p12-p11.1	Hs.213424
2	5<br>78	Kidney<br>FetalHeart	BDH2	3-hydroxybutyrate dehydrogenase, type 2	4	-104218231	56898	4q24	Hs.124696

2	5<br>76	Kidney<br>Adipocytes	MME	membrane metallo-endopeptidase (neutral endopeptidase, enkephalinase, CALLA, CD10)	3	156280152, 156280327, 156280748	4311	3q25.1-q25.2	Hs.307734
2	5<br>66	Kidney<br>TestisGermCell	ACE2	angiotensin I converting enzyme (peptidyl-dipeptidase A) 2	X	-15489076	59272	Xp22	Hs.178098
2	5<br>54	Kidney<br>FetalLiver	MAWBP		10	-139427265	64081	10pter-q25.3	Hs.198158
2	5<br>54	Kidney<br>FetalLiver	PDZK1	PDZ domain containing 1	1	144439082	5174	1q21	Hs.444751<br>Hs.515949
2	48<br>78	Uterus<br>FetalHeart	ASPN	asporin (LRR class 1)	9	-94258311	54829	9q22	Hs.435655
2	48<br>72	Uterus<br>SmoothMuscle	CALD1	caldesmon 1	7	134114710, 134226698	800	7q33	Hs.490203
2	48<br>7	Uterus<br>Skin	SCGB1D2	secretoglobin, family 1D, member 2	11	61766299	10647	11q13	Hs.204096
2	48<br>49	Uterus<br>Prostate	MYH11	myosin, heavy polypeptide 11, smooth muscle	16	-15704494	4629	16p13.11	Hs.460109
2	48<br>49	Uterus<br>Prostate	LOC650370		3		650370		
2	47<br>78	Heart<br>FetalHeart	MYL7	myosin, light polypeptide 7, regulatory	7	-44144989	58498	7p21-p11.2	Hs.75636
2	47<br>78	Heart<br>FetalHeart	TNNT2	troponin T type 2 (cardiac)	1	-199594764	7139	1q32	Hs.533613
2	47<br>78	Heart<br>FetalHeart	TNNI3	troponin I type 3 (cardiac)	19	-60354949	7137	19q13.4	Hs.631588
2	47<br>78	Heart<br>FetalHeart	ITGB1BP3	integrin beta 1 binding protein 3	19	3884596, 3884100	27231	19p13.3	Hs.135458
2	47<br>78	Heart<br>FetalHeart	NPPA	natriuretic peptide precursor A	1	-11828362	4878	1p36.21	Hs.75640
2	45<br>72	LymphNode<br>SmoothMuscle	C9orf26	chromosome 9 open reading frame 26 (NF-HEV)	9	6231677	90865	9p24.1	Hs.348390
2	44<br>75	Liver<br>Islet	SERPINA3	serpin peptidase inhibitor, clade A (alpha-1 antiproteinase, antitrypsin), member 3	14	94148466	12	14q32.1	Hs.534293<br>Hs.612083
2	44<br>54	Liver<br>FetalLiver	CPS1	carbamoyl-phosphate synthetase 1, mitochondrial	2	211129582	1373	2q35	Hs.149252
2	44<br>54	Liver<br>FetalLiver	SERPIND1	serpin peptidase inhibitor, clade D (heparin cofactor), member 1	22	19458382	3053	22q11.2|22q11.2 1	Hs.474270
2	44<br>54	Liver<br>FetalLiver	ITIH2	inter-alpha (globulin) inhibitor H2	10	7785241	3698	10p15	Hs.75285
2	44<br>54	Liver<br>FetalLiver	HPX	hemopexin	11	-6409017	3263	11p15.5-p15.4	Hs.426485
2	44<br>54	Liver<br>FetalLiver	LECT2	leukocyte cell-derived chemotaxin 2	5	-135310498	3950	5q31.1-q32	Hs.512580
2	44<br>54	Liver<br>FetalLiver	F7	coagulation factor VII (serum prothrombin conversion accelerator)	13	112808105	2155	13q34	Hs.36989
2	44<br>54	Liver<br>FetalLiver	CFHR2	complement factor H-related 2	1	195179556	3080	1q31-q32.1	Hs.632450
2	44<br>54	Liver<br>FetalLiver	CPB2	carboxypeptidase B2 (plasma, carboxypeptidase U)	13	-45525323	1361	13q14.11	Hs.512937
2	44<br>54	Liver<br>FetalLiver	AKR1D1	aldo-keto reductase family 1, member D1 (delta 4-3-ketosteroid-5-beta-reductase)	7	137411735	6718	7q32-q33	Hs.201667
2	44	Liver	ORM2	orosomucoid 2	9	116131889	5005	9q32	Hs.522356

	54	FetalLiver							
2	44<br>54	Liver<br>FetalLiver	F2	coagulation factor II (thrombin)	11	46697330	2147	11p11-q12	Hs.410092
2	44<br>54	Liver<br>FetalLiver	LIPC	lipase, hepatic	15	56511466	3990	15q21-q23	Hs.188630
2	44<br>54	Liver<br>FetalLiver	ADH1A	alcohol dehydrogenase 1A (class I), alpha polypeptide	4	-100416546	124	4q21-q23	Hs.4
2	44<br>54	Liver<br>FetalLiver	HAMP	hepcidin antimicrobial peptide	19	40465249	57817	19q13.1	Hs.8821
2	44<br>54	Liver<br>FetalLiver	HMGCS2	3-hydroxy-3-methylglutaryl-Coenzyme A synthase 2 (mitochondrial)	1	-120092527	3158	1p13-p12	Hs.59889
2	44<br>54	Liver<br>FetalLiver	UGT2B4	UDP glucuronosyltransferase 2 family, polypeptide B4	4	-70380472	7363	4q13	Hs.285887
2	44<br>5	Liver<br>Kidney	NAT8	N-acetyltransferase 8 (camello like)	2	-73721467	9027	2p13.1-p12	Hs.14637
2	44<br>5	Liver<br>Kidney	HPN	hepsin (transmembrane protease, serine 1)	19	40223249	3249	19q11-q13.2	Hs.182385
2	44<br>5	Liver<br>Kidney	BHMT2	betaine-homocysteine methyltransferase 2	5	78401338	23743	5q13	Hs.114172
2	44<br>5	Liver<br>Kidney	PCK1	phosphoenolpyruvate carboxykinase 1 (soluble)	20	55569542	5105	20q13.31	Hs.1872
2	44<br>5	Liver<br>Kidney	G6PC	glucose-6-phosphatase, catalytic subunit	17	38306340	2538	17q21	Hs.212293
2	44<br>5	Liver<br>Kidney	AKR7A3	aldo-keto reductase family 7, member A3 (aflatoxin aldehyde reductase)	1	-19481643	22977	1p35.1-p36.23	Hs.6980
2	44<br>5	Liver<br>Kidney	ASS	argininosuccinate synthetase	9	132315479,<br>132310092	445	9q34.1	Hs.160786
2	44<br>5	Liver<br>Kidney	UPB1	ureidopropionase, beta	22	23221250	51733	22q11.2	Hs.474388
2	44<br>5	Liver<br>Kidney	PCK2	phosphoenolpyruvate carboxykinase 2 (mitochondrial)	14	23633322	5106	14q12	Hs.75812
2	44<br>5	Liver<br>Kidney	ACY1	aminoacylase 1	3	51992602	95	3p21.1	Hs.334707
2	43<br>78	FetalBrain<br>FetalHeart	CSRP2	cysteine and glycine-rich protein 2	12	-75776626	1466	12q21.1	Hs.530904
2	43<br>78	FetalBrain<br>FetalHeart	TMSL8	thymosin-like 8	X	-101655266	11013	Xq21.33-q22.3	Hs.56145
2	43<br>73	FetalBrain<br>CaudateNucleus	HMP19		5	173405329	51617	5q35.2	Hs.559412
2	43<br>5	FetalBrain<br>Kidney	NPY1R	neuropeptide Y receptor Y1	4	-164464566	4886	4q31.3-q32	Hs.519057
2	42<br>76	Lung<br>Adipocytes	AOC3	amine oxidase, copper containing 3 (vascular adhesion protein 1)	17	38256726	8639	17q21	Hs.198241
2	42<br>47	Lung<br>Heart	C1QB	complement component 1, q subcomponent, B chain	1	22852268	713	1p36.12	Hs.8986
2	42<br>44	Lung<br>Liver	FCN3	ficolin (collagen/fibrinogen domain containing) 3 (Hakata antigen)	1	-27568189	8547	1p36.11	Hs.333383
2	41<br>64	Thyroid<br>FetalThyroid	TPO	thyroid peroxidase	2	1396241	7173	2p25	Hs.467554
2	41<br>64	Thyroid<br>FetalThyroid	TSHR	thyroid stimulating hormone receptor	14	80491621	7253	14q31	Hs.160411
2	41	Thyroid	PTH	parathyroid hormone	11	-13470176	5741	11p15.3-p15.1	Hs.37045

	64	FetalThyroid							
2	41<br>64	Thyroid<br>FetalThyroid	ID4	inhibitor of DNA binding 4, dominant negative helix-loop-helix protein	6	19945595	3400	6p22-p21	Hs.519601
2	4<br>78	ExtraocularMuscle<br>FetalHeart	NNT	nicotinamide nucleotide transhydrogenase	5	43638581, 43639063	23530	5p13.1-5cen	Hs.482043
2	4<br>5	ExtraocularMuscle<br>Kidney	GRB14	growth factor receptor-bound protein 14	2	-165057578	2888	2q22-q24	Hs.411881
2	39<br>57	AdrenalCortex<br>AdrenalGland	CYP21A2	cytochrome P450, family 21, subfamily A, polypeptide 2	6	32114060, 3413239, 3221082	1589	6p21.3	Hs.534847
2	39<br>57	AdrenalCortex<br>AdrenalGland	NOV	nephroblastoma overexpressed gene	8	120497881	4856	8q24.1	Hs.235935
2	39<br>57	AdrenalCortex<br>AdrenalGland	HSD3B2	hydroxy-delta-5-steroid dehydrogenase, 3 beta- and steroid delta-isomerase 2	1	119759295	3284	1p13.1	Hs.364941
2	39<br>57	AdrenalCortex<br>AdrenalGland	CYP17A1	cytochrome P450, family 17, subfamily A, polypeptide 1	10	-104580279	1586	10q24.3	Hs.438016
2	39<br>57	AdrenalCortex<br>AdrenalGland	ALAS1	aminolevulinate, delta-, synthase 1	3	52207183, 52207155	211	3p21.1	Hs.476308
2	39<br>57	AdrenalCortex<br>AdrenalGland	CYP11B1	cytochrome P450, family 11, subfamily B, polypeptide 1	8	-143950776	1584	8q21	Hs.184927
2	39<br>57	AdrenalCortex<br>AdrenalGland	FDXR	ferredoxin reductase	17	-70370214	2232	17q24-q25	Hs.69745
2	39<br>57	AdrenalCortex<br>AdrenalGland	KCNK3	potassium channel, subfamily K, member 3	2	26769122	3777	2p23	Hs.24040
2	39<br>57	AdrenalCortex<br>AdrenalGland	SCARB1	scavenger receptor class B, member 1	12	-123828128	949	12q24.31	Hs.298813
2	38<br>56	SalivaryGland<br>Trachea	MUC7	mucin 7, secreted	4	71372524	4589	4q13-q21	Hs.631946
2	38<br>56	SalivaryGland<br>Trachea	LPO	lactoperoxidase	17	53670846	4025	17q23.1	Hs.234742
2	38<br>56	SalivaryGland<br>Trachea	CRISP3	cysteine-rich secretory protein 3	6	-49803055	10321	6p12.3	Hs.404466
2	38<br>56	SalivaryGland<br>Trachea	PIP	prolactin-induced protein	7	142539295	5304	7q34	Hs.99949
2	38<br>56	SalivaryGland<br>Trachea	TCN1	transcobalamin I (vitamin B12 binding protein, R binder family)	11	-59376859	6947	11q11-q12	Hs.2012
2	38<br>56	SalivaryGland<br>Trachea	PRB2	proline-rich protein BstNI subfamily 2	12	-11396023	440083	12p13.2	Hs.631726
2	38<br>56	SalivaryGland<br>Trachea	ODAM	odontogenic, ameloblast asssociated	4	71096832	54959	4q13.3	Hs.143811
2	38<br>56	SalivaryGland<br>Trachea	PRB1	proline-rich protein BstNI subfamily 1	12	-11396023	5542	12p13.2	Hs.631726
2	38<br>56	SalivaryGland<br>Trachea	PRB4	proline-rich protein BstNI subfamily 4	12	-11351283	5545	12p13.2	Hs.528651
2	38<br>56	SalivaryGland<br>Trachea	PRB3	proline-rich protein BstNI subfamily 3	12	-11310124	5544	12p13.2	Hs.73031
2	38<br>41	SalivaryGland<br>Thyroid	CST4	cystatin S	20	-23614276	1472	20p11.21	Hs.123114
2	38<br>41	SalivaryGland<br>Thyroid	CST1	cystatin SN	20	-23676190	1469	20p11.21	Hs.123114
2	37<br>78	HBEC<br>FetalHeart	NT5DC2	5'-nucleotidase domain containing 2	3	-52533442	64943	3p21.1	Hs.84753

2	37<br>76	HBEC<br>Adipocytes	AKR1C2	aldo-keto reductase family 1, member C2 (dihydrodiol dehydrogenase 2, bile acid binding protein, 3-alpha hydroxysteroid dehydrogenase, type III)	10	-5021965	1646	10p15-p14	Hs.460260<br>Hs.567256
2	37<br>72	HBEC<br>SmoothMuscle	ALDH1A3	aldehyde dehydrogenase 1 family, member A3	15	99237583	220	15q26.3	Hs.459538
2	37<br>72	HBEC<br>SmoothMuscle	IL1A	interleukin 1, alpha	2	-113247963	3552	2q14	Hs.1722
2	37<br>62	HBEC<br>BoneMarrow	ANXA3	annexin A3	4	79691765	306	4q13-q22	Hs.480042
2	36<br>78	WholeBlood<br>FetalHeart	C1orf24	chromosome 1 open reading frame 24	1	-183026790	116496	1q25	Hs.518662
2	36<br>53	WholeBlood<br>Thymus	LY75	lymphocyte antigen 75	2	-160368121	4065	2q24	Hs.153563
2	36<br>5	WholeBlood<br>Kidney	TRBV9	T cell receptor beta variable 9	7		28586	7q34	
2	36<br>5	WholeBlood<br>Kidney	MGAM	maltase-glucoamylase (alpha-glucosidase)	7	141342147	8972	7q34	Hs.122785
2	33<br>78	FetalLung<br>FetalHeart	C8orf4	chromosome 8 open reading frame 4	8	40130143	56892	8p11.2	Hs.591849
2	33<br>54	FetalLung<br>FetalLiver	AFP	alpha-fetoprotein	4	74520796	174	4q11-q13	Hs.518808
2	33<br>44	FetalLung<br>Liver	APCS	amyloid P component, serum	1	157824239	325	1q21-q23	Hs.507080
2	33<br>44	FetalLung<br>Liver	CFB	complement factor B	6	32021751,<br>3360035,<br>3161513	629	6p21.3	Hs.69771
2	33<br>42	FetalLung<br>Lung	SFTPB	surfactant, pulmonary-associated protein B	2	-171477446	6439	2p12-p11.2	Hs.512690
2	33<br>42	FetalLung<br>Lung	SFTPC	surfactant, pulmonary-associated protein C	8	22075112	6440	8p21	Hs.1074
2	33<br>42	FetalLung<br>Lung	PGC	progastricsin (pepsinogen C)	6	-41812429	5225	6p21.3-p21.1	Hs.1867
2	33<br>42	FetalLung<br>Lung	LAMP3	lysosomal-associated membrane protein 3	3	-184322697	27074	3q26.3-q27	Hs.518448
2	33<br>35	FetalLung<br>Tonsil	CCL20	chemokine (C-C motif) ligand 20	2	228386813	6364	2q33-q37	Hs.75498
2	31<br>60	UterusCorpus<br>PituitaryGland	PRL	prolactin	6	-22395458	5617	6p22.2-p21.3	Hs.1905
2	30<br>53	Tongue<br>Thymus	MAL	mal, T-cell differentiation protein	2	95055205	4118	2cen-q13	Hs.80395
2	30<br>53	Tongue<br>Thymus	CALML3	calmodulin-like 3	10	5556923	810	10pter-p13	Hs.239600
2	30<br>35	Tongue<br>Tonsil	RHCG	Rh family, C glycoprotein	15	-87815645	51458	15q25	Hs.459284
2	30<br>35	Tongue<br>Tonsil	SPRR3	small proline-rich protein 3	1	151240848	6707	1q21-q22	Hs.139322
2	30<br>35	Tongue<br>Tonsil	SPRR1A	small proline-rich protein 1A	1	151223180	6698	1q21-q22	Hs.46320
2	30<br>35	Tongue<br>Tonsil	KRT4	keratin 4	12	-51486600	3851	12q12-q13	Hs.533782
2	30<br>35	Tongue<br>Tonsil	CRNN	cornulin	1	-150648342	49860	1q21	Hs.242057
2	30<br>35	Tongue<br>Tonsil	S100A7	S100 calcium binding protein A7 (psoriasin 1)	1	-151696844	6278	1q21	Hs.112408

2	30<br>35	Tongue<br>Tonsil	C1orf42	chromosome 1 open reading frame 42	1	150753601	54544	1q21	Hs.110196
2	3<br>78	LimbMuscle<br>FetalHeart	APOBEC2	apolipoprotein B mRNA editing enzyme, catalytic polypeptide-like 2	6	41128990	10930	6p21	Hs.555915
2	3<br>78	LimbMuscle<br>FetalHeart	POPDC3	popeye domain containing 3	6	-105712469	64208	6q21	Hs.458336
2	3<br>78	LimbMuscle<br>FetalHeart	TACC2	transforming, acidic coiled-coil containing protein 2	10	123913094,<br>123738678	10579	10q26	Hs.501252<br>Hs.643068
2	3<br>5	LimbMuscle<br>Kidney	SUCLG2	succinate-CoA ligase, GDP-forming, beta subunit	3	-67507835	8801	3p14.1	Hs.186512
2	3<br>48	LimbMuscle<br>Uterus	PDZRN3	PDZ domain containing RING finger 3	3	-73514341	23024	3p13	Hs.434900
2	3<br>48	LimbMuscle<br>Uterus	DOC1		3	-202084140	11259	3q12.1	Hs.104672
2	3<br>43	LimbMuscle<br>FetalBrain	MN1	meningioma (disrupted in balanced translocation) 1	22	-26474265	4330	22q11|22q12.1	Hs.268515
2	3<br>41	LimbMuscle<br>Thyroid	CA3	carbonic anhydrase III, muscle specific	8	86537709	761	8q13-q22	Hs.82129
2	3<br>4	LimbMuscle<br>ExtraocularMuscle	NRAP	nebulin-related anchoring protein	10	-115338573	4892	10q24-q26	Hs.268788
2	3<br>4	LimbMuscle<br>ExtraocularMuscle	MYF6	myogenic factor 6 (herculin)	12	79625576	4618	12q21	Hs.35937
2	3<br>4	LimbMuscle<br>ExtraocularMuscle	TMEM38B	transmembrane protein 38B	9	107496645	55151	9q31.2	Hs.411925
2	3<br>4	LimbMuscle<br>ExtraocularMuscle	MYH4	myosin, heavy polypeptide 4, skeletal muscle	17	-10287334	4622	17p13.1	Hs.272207
2	3<br>4	LimbMuscle<br>ExtraocularMuscle	SAR1B	SAR1 gene homolog B (S. cerevisiae)	5	-133970018	51128	5q31.1	Hs.432984
2	3<br>4	LimbMuscle<br>ExtraocularMuscle	FYCO1	FYVE and coiled-coil domain containing 1	3	-45934399	79443	3p21.31	Hs.200227
2	3<br>4	LimbMuscle<br>ExtraocularMuscle	TOB1	transducer of ERBB2, 1	17	-46294587	10140	17q21	Hs.531550<br>Hs.606234
2	3<br>4	LimbMuscle<br>ExtraocularMuscle	GENX-3414		4	77446832	8987	4q24-q25	Hs.109590
2	3<br>36	LimbMuscle<br>WholeBlood	UBE2D1	ubiquitin-conjugating enzyme E2D 1 (UBC4/5 homolog, yeast)	10	59764744	7321	10q11.2-q21	Hs.129683
2	3<br>32	LimbMuscle<br>PsoasMuscle	TPM3	tropomyosin 3	1	-304802035	7170	1q21.2	Hs.129512<br>Hs.146070<br>Hs.449194
2	23<br>78	PB-BDCA4DentriticCells<br>FetalHeart	DUSP5	dual specificity phosphatase 5	10	112247614	1847	10q25	Hs.2128
2	23<br>5	PB-BDCA4DentriticCells<br>Kidney	CD2AP	CD2-associated protein	6	47553898	23607	6p12	Hs.485518
2	22<br>78	BM-CD34<br>FetalHeart	PTPLA	protein tyrosine phosphatase-like (proline instead of catalytic arginine), member A	10	-17671965	9200	10p14-p13	Hs.114062
2	22<br>62	BM-CD34<br>BoneMarrow	PRTN3	proteinase 3 (serine proteinase, neutrophil, Wegener granulomatosis autoantigen)	19	791984	5657	19p13.3	Hs.928
2	22<br>62	BM-CD34<br>BoneMarrow	CTSG	cathepsin G	14	-24112564	1511	14q11.2	Hs.421724
2	22<br>56	BM-CD34<br>Trachea	CYTL1	cytokine-like 1	4	-5067216	54360	4p16-p15	Hs.13872

2	22<br>41	BM-CD34<br>Thyroid	KIT	v-kit Hardy-Zuckerman 4 feline sarcoma viral oncogene homolog	4	55218917	3815	4q11-q12	Hs.479754
2	22<br>23	BM-CD34<br>PB-BDCA4DentriticCells	CBFA2T3	core-binding factor, runt domain, alpha subunit 2, translocated to, 3	16	-87468767	863	16q24	Hs.513811
2	21<br>3	BM-CD105Endothelial<br>LimbMuscle	NAT13	N-acetyltransferase 13	3	-114920530	80218	3q13.2	Hs.269528
2	21<br>22	BM-CD105Endothelial<br>BM-CD34	NMU	neuromedin U	4	-56156161	10874	4q12	Hs.418367
2	21<br>22	BM-CD105Endothelial<br>BM-CD34	NOLA1	nucleolar protein family A, member 1 (H/ACA small nucleolar RNPs)	4	110956114	54433	4q25	Hs.69851
2	20<br>62	BM-CD33Myeloid<br>BoneMarrow	RETN	resistin	19	7639971	56729	19p13.2	Hs.283091
2	20<br>62	BM-CD33Myeloid<br>BoneMarrow	BPI	bactericidal/permeability-increasing protein	20	36365998	671	20q11.23-q12	Hs.529019
2	20<br>39	BM-CD33Myeloid<br>AdrenalCortex	NR4A2	nuclear receptor subfamily 4, group A, member 2	2	-156889196	4929	2q22-q23	Hs.563344
2	20<br>22	BM-CD33Myeloid<br>BM-CD34	EREG	epiregulin	4	75449723	2069	4q13.3	Hs.115263
2	2<br>67	BronchialEpitelia<br>TestisIntersitial	EFHC1	EF-hand domain (C-terminal) containing 1	6	52393070	114327	6p12.3	Hs.403171<br>Hs.592773
2	2<br>67	BronchialEpitelia<br>TestisIntersitial	FLJ23049		3	-168440778	79740	3q26.1	Hs.478143
2	2<br>56	BronchialEpitelia<br>Trachea	MUC5AC	mucin 5AC, oligomeric mucus/gel-forming	11		4586	11p15.5	Hs.534332<br>Hs.558950<br>Hs.592369
2	2<br>56	BronchialEpitelia<br>Trachea	MUC5B	mucin 5B, oligomeric mucus/gel-forming	11		4587	11p15	Hs.523395
2	2<br>56	BronchialEpitelia<br>Trachea	PLUNC	palate, lung and nasal epithelium carcinoma associated	20	31287462	51297	20q11.2	Hs.211092
2	2<br>56	BronchialEpitelia<br>Trachea	SERPINB3	serpin peptidase inhibitor, clade B (ovalbumin), member 3	18	-59473411	6317	18q21.3	Hs.227948
2	2<br>49	BronchialEpitelia<br>Prostate	PSCA	prostate stem cell antigen	8	143758876	8000	8q24.2	Hs.379010<br>Hs.535903
2	2<br>44	BronchialEpitelia<br>Liver	ADH1C	alcohol dehydrogenase 1C (class I), gamma polypeptide	4	-100476673	126	4q21-q23	Hs.4
2	2<br>42	BronchialEpitelia<br>Lung	MSLN	mesothelin	16	751133	10232	16p13.3	Hs.408488
2	2<br>37	BronchialEpitelia<br>HBEC	C14orf78	chromosome 14 open reading frame 78	14		113146	14q32.33	Hs.441783<br>Hs.632328
2	2<br>37	BronchialEpitelia<br>HBEC	F3	coagulation factor III (thromboplastin, tissue factor)	1	-94767460	2152	1p22-p21	Hs.62192
2	2<br>31	BronchialEpitelia<br>UterusCorpus	MMP10	matrix metallopeptidase 10 (stromelysin 2)	11	-102146443	4319	11q22.3	Hs.2258
2	19<br>23	PB-CD19BCells<br>PB-BDCA4DentriticCells	HLA-DPA1	major histocompatibility complex, class II, DP alpha 1	6	-41773262	3113	6p21.3	Hs.347270
2	19<br>23	PB-CD19BCells<br>PB-BDCA4DentriticCells	C13orf18	chromosome 13 open reading frame 18	13	-45815100	80183	13q14.12	Hs.98117
2	19<br>23	PB-CD19BCells<br>PB-BDCA4DentriticCells	OPN3	opsin 3 (encephalopsin, panopsin)	1	-239823075	23596	1q43	Hs.409081<br>Hs.534399
2	19<br>23	PB-CD19BCells<br>PB-BDCA4DentriticCells	KIAA0746		4	-25358146	23231	4p15.2	Hs.479384
2	19	PB-CD19BCells	TCF4	transcription factor 4	18	-51045966	6925	18q21.1	Hs.569908

	23	PB-BDCA4DentriticCells							
2	19<br>23	PB-CD19BCells<br>PB-BDCA4DentriticCells	P2RY14	purinergic receptor P2Y, G-protein coupled, 14	3	-152412596	9934	3q21-q25	Hs.2465
2	19<br>22	PB-CD19BCells<br>BM-CD34	CD79B	CD79b molecule, immunoglobulin-associated beta	17	-59359830	974	17q23	Hs.89575
2	19<br>20	PB-CD19BCells<br>BM-CD33Myeloid	STK17B	serine/threonine kinase 17b (apoptosis-inducing)	2	-196710102	9262	2q32.3	Hs.88297
2	18<br>37	PB-CD56NKCells<br>HBEC	AREG	amphiregulin (schwannoma-derived growth factor)	4	75529716, 75699652	374	4q13-q21	Hs.270833
2	18<br>37	PB-CD56NKCells<br>HBEC	LOC653193		4		653193	4q13.3	Hs.632601
2	17<br>5	PB-CD14Monocytes<br>Kidney	SLC7A7	solute carrier family 7 (cationic amino acid transporter, y+ system), member 7	14	-22312273	9056	14q11.2	Hs.513147
2	17<br>36	PB-CD14Monocytes<br>WholeBlood	S100A4	S100 calcium binding protein A4 (calcium protein, calvasculin, metastasin, murine placental homolog)	1	-151782721	6275	1q21	Hs.557609
2	17<br>36	PB-CD14Monocytes<br>WholeBlood	FCGR2A	Fc fragment of IgG, low affinity IIa, receptor (CD32)	1	159741843	2212	1q23	Hs.352642
2	17<br>36	PB-CD14Monocytes<br>WholeBlood	P2RY13	purinergic receptor P2Y, G-protein coupled, 13	3	-152526785	53829	3q24	Hs.546396
2	17<br>36	PB-CD14Monocytes<br>WholeBlood	DPEP2	dipeptidase 2	16	-66578800	64174	16q22.1	Hs.372633
2	17<br>36	PB-CD14Monocytes<br>WholeBlood	CKLF	chemokine-like factor	16	65143966, 65143972	51192	16q21	Hs.15159
2	17<br>36	PB-CD14Monocytes<br>WholeBlood	TLR1	toll-like receptor 1	4	-38474271	7096	4p14	Hs.575090<br>Hs.621817
2	17<br>23	PB-CD14Monocytes<br>PB-BDCA4DentriticCells	MAN2B1	mannosidase, alpha, class 2B, member 1	19	-12618321	4125	19cen-q13.1	Hs.334626<br>Hs.356769
2	17<br>20	PB-CD14Monocytes<br>BM-CD33Myeloid	MS4A6A	membrane-spanning 4-domains, subfamily A, member 6A	11	-119392204	64231	11q12.1	Hs.523702
2	17<br>20	PB-CD14Monocytes<br>BM-CD33Myeloid	VNN1	vanin 1	6	-133044424	8876	6q23-q24	Hs.12114
2	17<br>20	PB-CD14Monocytes<br>BM-CD33Myeloid	KCTD12	potassium channel tetramerisation domain containing 12	13	-76352312	115207	13q22.3	Hs.109438
2	17<br>20	PB-CD14Monocytes<br>BM-CD33Myeloid	LILRB2	leukocyte immunoglobulin-like receptor, subfamily B (with TM and ITIM domains), member 2	19	-59470131	10288	19q13.4	Hs.241797<br>Hs.631536
2	17<br>20	PB-CD14Monocytes<br>BM-CD33Myeloid	KIAA0984		12		23329	12q14.3	Hs.192492
2	17<br>20	PB-CD14Monocytes<br>BM-CD33Myeloid	SGK	serum/glucocorticoid regulated kinase	6	-134532081	6446	6q23	Hs.510078
2	17<br>20	PB-CD14Monocytes<br>BM-CD33Myeloid	STX11	syntaxin 11	6	144513361	8676	6q24.2	Hs.118958
2	17<br>20	PB-CD14Monocytes<br>BM-CD33Myeloid	IMPDH1	IMP (inosine monophosphate) dehydrogenase 1	7	-127819567	3614	7q31.3-q32	Hs.534808
2	17<br>20	PB-CD14Monocytes<br>BM-CD33Myeloid	TBXAS1	thromboxane A synthase 1 (platelet, cytochrome P450, family 5, subfamily A)	7	139175420	6916	7q34-q35	Hs.520757
2	17<br>18	PB-CD14Monocytes<br>PB-CD56NKCells	PSTPIP1	proline-serine-threonine phosphatase interacting protein 1	15	75074608	9051	15q24-q25.1	Hs.129758
2	16<br>78	BM-CD71EarlyErythroid<br>FetalHeart	EIF1AY	eukaryotic translation initiation factor 1A, Y-linked	Y	21146998	9086	Yq11.222	Hs.461178

2	16<br>54	BM-CD71EarlyErythroid<br>FetalLiver	HBZ	hemoglobin, zeta	16	142853	3050	16p13.3	Hs.585357
2	16<br>54	BM-CD71EarlyErythroid<br>FetalLiver	ARG1	arginase, liver	6	131936057	383	6q23	Hs.440934
2	16<br>5	BM-CD71EarlyErythroid<br>Kidney	PDZK1IP1	PDZK1 interacting protein 1	1	-47421847	10158	1p33	Hs.431099
2	16<br>44	BM-CD71EarlyErythroid<br>Liver	TFR2	transferrin receptor 2	7	-100055975	7036	7q22	Hs.544932
2	16<br>23	BM-CD71EarlyErythroid<br>PB-BDCA4DentriticCells	ST6GALNAC4	ST6 (alpha-N-acetyl-neuraminyl-2,3-beta-galactosyl-1,3)-N-acetylgalactosaminide alpha-2,6-sialyltransferase 4	9	-129709986	27090	9q34	Hs.3972
2	16<br>21	BM-CD71EarlyErythroid<br>BM-CD105Endothelial	UROD	uroporphyrinogen decarboxylase	1	45250416	7389	1p34	Hs.78601
2	16<br>21	BM-CD71EarlyErythroid<br>BM-CD105Endothelial	SMC2	structural maintenance of chromosomes 2	9	105896651,<br>105896361	10592	9q31.1	Hs.119023
2	16<br>21	BM-CD71EarlyErythroid<br>BM-CD105Endothelial	HBQ1	hemoglobin, theta 1	16	170334	3049	16p13.3	Hs.247921
2	16<br>21	BM-CD71EarlyErythroid<br>BM-CD105Endothelial	GYPE	glycophorin E	4	-290027384	2996	4q31.1	Hs.632594
2	16<br>21	BM-CD71EarlyErythroid<br>BM-CD105Endothelial	TTK	TTK protein kinase	6	80771077	7272	6q13-q21	Hs.169840
2	16<br>21	BM-CD71EarlyErythroid<br>BM-CD105Endothelial	TFRC	transferrin receptor (p90, CD71)	3	-197260746	7037	3q29	Hs.529618
2	16<br>21	BM-CD71EarlyErythroid<br>BM-CD105Endothelial	CCNE1	cyclin E1	19	34994740,<br>34995400	898	19q12	Hs.244723
2	16<br>21	BM-CD71EarlyErythroid<br>BM-CD105Endothelial	BLVRB	biliverdin reductase B (flavin reductase (NADPH))	19	-45645540	645	19q13.1-q13.2	Hs.515785
2	16<br>21	BM-CD71EarlyErythroid<br>BM-CD105Endothelial	LXN	latexin	3	-159866899	56925	3q25.32	Hs.478067
2	16<br>21	BM-CD71EarlyErythroid<br>BM-CD105Endothelial	GFI1B	growth factor independent 1B (potential regulator of CDKN1A, translocated in CML)	9	134843918	8328	9q34.13	Hs.553160
2	16<br>21	BM-CD71EarlyErythroid<br>BM-CD105Endothelial	TAL1	T-cell acute lymphocytic leukemia 1	1	-47454550	6886	1p32	Hs.73828
2	16<br>21	BM-CD71EarlyErythroid<br>BM-CD105Endothelial	NUDT1	nudix (nucleoside diphosphate linked moiety X)-type motif 1	7	2248382,<br>2248421,<br>2249065	4521	7p22	Hs.534331
2	16<br>21	BM-CD71EarlyErythroid<br>BM-CD105Endothelial	GCLM	glutamate-cysteine ligase, modifier subunit	1	-94125177	2730	1p22.1	Hs.315562
2	16<br>21	BM-CD71EarlyErythroid<br>BM-CD105Endothelial	FECH	ferrochelatase (protoporphyria)	18	-53366534	2235	18q21.3	Hs.465221
2	16<br>21	BM-CD71EarlyErythroid<br>BM-CD105Endothelial	SNCA	synuclein, alpha (non A4 component of amyloid precursor)	4	-181731784	6622	4q21	Hs.271771
2	16<br>21	BM-CD71EarlyErythroid<br>BM-CD105Endothelial	PPOX	protoporphyrinogen oxidase	1	159402817	5498	1q22	Hs.517373
2	16<br>21	BM-CD71EarlyErythroid<br>BM-CD105Endothelial	TRAK2	trafficking protein, kinesin binding 2	2	-201950175	66008	2q33	Hs.152774

2	16<br>21	BM-CD71EarlyErythroid<br>BM-CD105Endothelial	PRDX2	peroxiredoxin 2	19	-25541060	7001	19p13.2	Hs.631612
2	16<br>21	BM-CD71EarlyErythroid<br>BM-CD105Endothelial	KIF4A	kinesin family member 4A	X	69426686	24137	Xq13.1	Hs.438712
2	16<br>21	BM-CD71EarlyErythroid<br>BM-CD105Endothelial	AMMECR1	Alport syndrome, mental retardation, midface hypoplasia and elliptocytosis chromosomal region, gene 1	X	-109324070	9949	Xq22.3	Hs.481208
2	16<br>21	BM-CD71EarlyErythroid<br>BM-CD105Endothelial	HMBS	hydroxymethylbilane synthase	11	118460796,<br>118463906	3145	11q23.3	Hs.82609
2	16<br>18	BM-CD71EarlyErythroid<br>PB-CD56NKCells	CHST2	carbohydrate (N-acetylglucosamine-6-O) sulfotransferase 2	3	144321357	9435	3q24	Hs.8786
2	15<br>53	PB-CD4TCells<br>Thymus	TRBC1	T cell receptor beta constant 1	7		28639	7q34	Hs.511522
2	15<br>22	PB-CD4TCells<br>BM-CD34	PNN	pinin, desmosome associated protein	14	38714150	5411	14q21.1	Hs.409965
2	14<br>53	PB-CD8TCells<br>Thymus	CD8A	CD8a molecule	2	-86865239	925	2p12	Hs.85258
2	14<br>41	PB-CD8TCells<br>Thyroid	LTBP3	latent transforming growth factor beta binding protein 3	11	-65062851	4054	11q12	Hs.289019
2	14<br>19	PB-CD8TCells<br>PB-CD19BCells	TRAF5	TNF receptor-associated factor 5	1	209566801,<br>209566579,<br>209586328	7188	1q32	Hs.523930
2	14<br>19	PB-CD8TCells<br>PB-CD19BCells	KLF2	Kruppel-like factor 2 (lung)	19	16296650	10365	19p13.13-p13.11	Hs.107740
2	14<br>18	PB-CD8TCells<br>PB-CD56NKCells	PTPN4	protein tyrosine phosphatase, non-receptor type 4 (megakaryocyte)	2	120233676	5775	2q14.2	Hs.469809
2	14<br>18	PB-CD8TCells<br>PB-CD56NKCells	STK38	serine/threonine kinase 38	6	-36569649	11329	6p21	Hs.409578
2	14<br>18	PB-CD8TCells<br>PB-CD56NKCells	CTSW	cathepsin W (lymphopain)	11	65403859	1521	11q13.1	Hs.416848
2	14<br>18	PB-CD8TCells<br>PB-CD56NKCells	GZMM	granzyme M (lymphocyte met-ase 1)	19	495026	3004	19p13.3	Hs.465511
2	14<br>18	PB-CD8TCells<br>PB-CD56NKCells	ARHGEF3	Rho guanine nucleotide exchange factor (GEF) 3	3	-56736485	50650	3p21-p13	Hs.476402
2	14<br>18	PB-CD8TCells<br>PB-CD56NKCells	CD160	CD160 molecule	1	-144407154	11126	1q21.1	Hs.488237
2	14<br>18	PB-CD8TCells<br>PB-CD56NKCells	ITGAL	integrin, alpha L (antigen CD11A (p180), lymphocyte function-associated antigen 1, alpha polypeptide)	16	30391571	3683	16p11.2	Hs.174103
2	14<br>18	PB-CD8TCells<br>PB-CD56NKCells	CBLB	Cas-Br-M (murine) ecotropic retroviral transforming sequence b	3	-106859799	868	3q13.11	Hs.430589
2	14<br>18	PB-CD8TCells<br>PB-CD56NKCells	MBNL1	muscleblind-like (Drosophila)	3	153468518,<br>153499883	4154	3q25	Hs.478000<br>Hs.558914
2	14<br>15	PB-CD8TCells<br>PB-CD4TCells	PPWD1	peptidylprolyl isomerase domain and WD repeat containing 1	5	64894890	23398	5q12.3	Hs.121432
2	14<br>15	PB-CD8TCells<br>PB-CD4TCells	FLT3LG	fms-related tyrosine kinase 3 ligand	19	54669297	2323	19q13.3	Hs.428
2	14<br>15	PB-CD8TCells<br>PB-CD4TCells	RAPGEF6	Rap guanine nucleotide exchange factor (GEF) 6	5	-130789502	51735	5q31.1	Hs.483329
2	14<br>15	PB-CD8TCells<br>PB-CD4TCells	LAT	linker for activation of T cells	16	28903887,<br>28903647	27040	16p11.2	Hs.632179

2	14<br>15	PB-CD8TCells<br>PB-CD4TCells	CYLD	cylindromatosis (turban tumor syndrome)	16	49333461,<br>49333529	1540	16q12.1	Hs.578973
2	14<br>15	PB-CD8TCells<br>PB-CD4TCells	DGKA	diacylglycerol kinase, alpha 80kDa	12	54611212,<br>54612078	1606	12q13.3	Hs.524488
2	14<br>15	PB-CD8TCells<br>PB-CD4TCells	HMHA1	histocompatibility (minor) HA-1	19	1018173	23526	19p13.3	Hs.465521
2	14<br>15	PB-CD8TCells<br>PB-CD4TCells	GBP1	guanylate binding protein 1, interferon-inducible, 67kDa	1	-89290593	2633	1p22.2	Hs.62661
2	14<br>15	PB-CD8TCells<br>PB-CD4TCells	ATHL1	ATH1, acid trehalase-like 1 (yeast)	11	280693	80162	11p15.5	Hs.353181
2	14<br>15	PB-CD8TCells<br>PB-CD4TCells	TNFRSF7	tumor necrosis factor receptor superfamily, member 7	12	6424326	939	12p13	Hs.355307
2	14<br>15	PB-CD8TCells<br>PB-CD4TCells	ATM	ataxia telangiectasia mutated (includes complementation groups A, C and D)	11	107598768,<br>107663328	472	11q22-q23	Hs.435561
2	14<br>15	PB-CD8TCells<br>PB-CD4TCells	NSUN5B	NOL1/NOP2/Sun domain family, member 5B	7	74877581	155400	7q11.23	Hs.632322
2	13<br>78	721-BLymphoblasts<br>FetalHeart	C6orf79	chromosome 6 open reading frame 79	6	-13898999	63933	6p24.3-p23	Hs.214043
2	13<br>78	721-BLymphoblasts<br>FetalHeart	FHOD3	formin homology 2 domain containing 3	18	32131699	80206	18q12	Hs.436636
2	13<br>5	721-BLymphoblasts<br>Kidney	CRYZ	crystallin, zeta (quinone reductase)	1	-74943771	1429	1p31-p22	Hs.83114
2	13<br>35	721-BLymphoblasts<br>Tonsil	UBD	ubiquitin D	6	-978728	10537	6p21.3	Hs.44532
2	13<br>21	721-BLymphoblasts<br>BM-CD105Endothelial	MCM2	MCM2 minichromosome maintenance deficient 2, mitotin (S. cerevisiae)	3	128799942	4171	3q21	Hs.477481
2	13<br>21	721-BLymphoblasts<br>BM-CD105Endothelial	MAD2L1	MAD2 mitotic arrest deficient-like 1 (yeast)	4	-121200039	4085	4q27	Hs.591697
2	13<br>21	721-BLymphoblasts<br>BM-CD105Endothelial	GMNN	geminin, DNA replication inhibitor	6	24883142	51053	6p22.2	Hs.234896
2	13<br>21	721-BLymphoblasts<br>BM-CD105Endothelial	SSBP1	single-stranded DNA binding protein 1	7	141084644	6742	7q34	Hs.490394
2	13<br>19	721-BLymphoblasts<br>PB-CD19BCells	SWAP70		11	9642203	23075	11p15	Hs.153026
2	13<br>19	721-BLymphoblasts<br>PB-CD19BCells	HLA-DQA1	major histocompatibility complex, class II, DQ alpha 1	6	32713160	3117	6p21.3	Hs.387679
2	13<br>19	721-BLymphoblasts<br>PB-CD19BCells	BLK	B lymphoid tyrosine kinase	8	11388929	640	8p23-p22	Hs.146591
2	13<br>19	721-BLymphoblasts<br>PB-CD19BCells	FCER2	Fc fragment of IgE, low affinity II, receptor for (CD23)	19	-7659662	2208	19p13.3	Hs.465778
2	13<br>16	721-BLymphoblasts<br>BM-CD71EarlyErythroid	CDC2	cell division cycle 2, G1 to S and G2 to M	10	62208241,<br>62209916	983	10q21.1	Hs.334562
2	13<br>16	721-BLymphoblasts<br>BM-CD71EarlyErythroid	ASPM	asp (abnormal spindle)-like, microcephaly associated (Drosophila)	1	-195320076	259266	1q31	Hs.121028
2	12<br>78	Placenta<br>FetalHeart	TUSC3	tumor suppressor candidate 3	8	15442100	7991	8p22	Hs.591845
2	12<br>76	Placenta<br>Adipocytes	ADFP	adipose differentiation-related protein	9	-19105759	123	9p22.1	Hs.3416
2	12<br>61	Placenta<br>Thalamus	CRH	corticotropin releasing hormone	8	-67251172	1392	8q13	Hs.75294
2	12<br>60	Placenta<br>PituitaryGland	GH1	growth hormone 1	17	-59348295	2688	17q24.2	Hs.406754

2	12<br>60	Placenta<br>PituitaryGland	CSH2	chorionic somatomammotropin hormone 2	17	-59303104	1443	17q24.2	Hs.406754
2	12<br>60	Placenta<br>PituitaryGland	CGA	glycoprotein hormones, alpha polypeptide	6	-87851940	1081	6q12-q21	Hs.119689
2	12<br>55	Placenta<br>Testis	LOC645569		15		645569	15q21.2	Hs.511367
2	12<br>5	Placenta<br>Kidney	DAB2	disabled homolog 2, mitogen-responsive phosphoprotein (Drosophila)	5	-39408502	1601	5p13	Hs.481980
2	12<br>5	Placenta<br>Kidney	HSD11B2	hydroxysteroid (11-beta) dehydrogenase 2	16	66022536	3291	16q22	Hs.1376
2	12<br>43	Placenta<br>FetalBrain	DLG5	discs, large homolog 5 (Drosophila)	10	-79220556	9231	10q23	Hs.500245
2	12<br>33	Placenta<br>FetalLung	EGFL6	EGF-like-domain, multiple 6	X	13497661	25975	Xp22	Hs.12844
2	12<br>33	Placenta<br>FetalLung	GPR126	G protein-coupled receptor 126	6	142664758	57211	6q24.1	Hs.318894
2	12<br>31	Placenta<br>UterusCorpus	IGFBP3	insulin-like growth factor binding protein 3	7	-45918374	3486	7p13-p12	Hs.450230
2	12<br>13	Placenta<br>721-BLymphoblasts	EBI3	Epstein-Barr virus induced gene 3	19	4180539	10148	19p13.3	Hs.501452
2	11<br>65	DRG<br>OlfactoryBulb	MPZ	myelin protein zero (Charcot-Marie-Tooth neuropathy 1B)	1	-159541150	4359	1q22	Hs.591486
2	11<br>65	DRG<br>OlfactoryBulb	PMP22	peripheral myelin protein 22	17	-15073821	5376	17p12-p11.2	Hs.372031
2	1<br>78	Hippocampus<br>FetalHeart	BNIP3	BCL2/adenovirus E1B 19kDa interacting protein 3	10	-133631193	664	10q26.3	Hs.144873<br>Hs.562561
2	1<br>51	Hippocampus<br>PrefrontalCortex	KCNB1	potassium voltage-gated channel, Shab-related subfamily, member 1	20	-47421911	3745	20q13.2	Hs.84244
2	1<br>51	Hippocampus<br>PrefrontalCortex	KIF5C	kinesin family member 5C	2		3800	2q23.1	Hs.435557
2	1<br>5	Hippocampus<br>Kidney	SH3YL1	SH3 domain containing, Ysc84-like 1 (S. cerevisiae)	2	-208154	26751	2p25.3	Hs.515951
2	1<br>5	Hippocampus<br>Kidney	KL	klotho	13	32488570	9365	13q12	Hs.524953
2	1<br>5	Hippocampus<br>Kidney	DMXL1	Dmx-like 1	5	118435083	1657	5q22	Hs.181042
2	1<br>46	Hippocampus<br>Amygdala	SLC6A1	solute carrier family 6 (neurotransmitter transporter, GABA), member 1	3	11009455	6529	3p25-p24	Hs.443874
2	1<br>46	Hippocampus<br>Amygdala	CTNND2	catenin (cadherin-associated protein), delta 2 (neural plakophilin-related arm-repeat protein)	5	-11024951	1501	5p15.2	Hs.314543
2	1<br>46	Hippocampus<br>Amygdala	BAI3	brain-specific angiogenesis inhibitor 3	6	69404157	577	6q12	Hs.13261
2	1<br>46	Hippocampus<br>Amygdala	NPTXR	neuronal pentraxin receptor	22	-37544403	23467	22q13.1	Hs.91622
2	1<br>43	Hippocampus<br>FetalBrain	ZFHX1B	zinc finger homeobox 1b	2	-144862053	9839	2q22	Hs.34871
2	1<br>43	Hippocampus<br>FetalBrain	SCN3A	sodium channel, voltage-gated, type III, alpha	2	-165652285	6328	2q24	Hs.435274
2	1<br>4	Hippocampus<br>ExtraocularMuscle	SACS	spastic ataxia of Charlevoix-Saguenay (sacsin)	13	-22800966	26278	13q12	Hs.159492
2	1<br>36	Hippocampus<br>WholeBlood	LOC552891		9	113433486	552891	9q31.3	Hs.534196

2	1<br>36	Hippocampus<br>WholeBlood	GNG10	guanine nucleotide binding protein (G protein), gamma 10	9	113463681	2790	9q31.3	Hs.534196<br>Hs.577252
2	1<br>18	Hippocampus<br>PB-CD56NKCells	DGKZ	diacylglycerol kinase, zeta 104kDa	11	46325594,<br>46311314,<br>46323562	8525	11p11.2	Hs.502461
2	1<br>15	Hippocampus<br>PB-CD4TCells	ERBB2IP	erbb2 interacting protein	5	65258139	55914	5q12.3	Hs.591774
3	7<br>72<br>76	Skin<br>SmoothMuscle<br>Adipocytes	TMEM45A	transmembrane protein 45A	3	101694152	55076	3q12.2	Hs.126598
3	63<br>72<br>78	CardiacMyocytes<br>SmoothMuscle<br>FetalHeart	LRRC17	leucine rich repeat containing 17	7	102340701,<br>102340687	10234	7q22.1	Hs.567412
3	63<br>72<br>76	CardiacMyocytes<br>SmoothMuscle<br>Adipocytes	TPM4	tropomyosin 4	19	16048324	7171	19p13.1	Hs.631618
3	55<br>67<br>77	Testis<br>TestisIntersitial<br>TestiSeminiferousTubule	PGK2	phosphoglycerate kinase 2	6	-49861324	5232	6p12.3	Hs.367727
3	55<br>67<br>77	Testis<br>TestisIntersitial<br>TestiSeminiferousTubule	HRASLS	HRAS-like suppressor	3	194441611	57110	3q29	Hs.36761
3	55<br>67<br>77	Testis<br>TestisIntersitial<br>TestiSeminiferousTubule	TKTL1	transketolase-like 1	X	153177344	8277	Xq28	Hs.102866
3	55<br>67<br>68	Testis<br>TestisIntersitial<br>TestisLeydigCell	AKAP3	A kinase (PRKA) anchor protein 3	12	-4594937	10566	12p13.3	Hs.98397
3	50<br>60<br>75	Pancreas<br>PituitaryGland<br>Islet	CEL	carboxyl ester lipase (bile salt-stimulated lipase)	9	134927185	1056	9q34.3	Hs.533258
3	50<br>57<br>75	Pancreas<br>AdrenalGland<br>Islet	CPB1	carboxypeptidase B1 (tissue)	3	150028277	1360	3q24	Hs.477891
3	48<br>72<br>78	Uterus<br>SmoothMuscle<br>FetalHeart	TPM1	tropomyosin 1 (alpha)	15	61121890,<br>61127688	7168	15q22.1	Hs.133892
3	48<br>49<br>78	Uterus<br>Prostate<br>FetalHeart	CNN1	calponin 1, basic, smooth muscle	19	11510578	1264	19p13.2-p13.1	Hs.465929
3	48<br>49<br>78	Uterus<br>Prostate<br>FetalHeart	MEIS2	Meis1, myeloid ecotropic viral integration site 1 homolog 2 (mouse)	15	-34970524	4212	15q14	Hs.510989
3	47<br>72<br>78	Heart<br>SmoothMuscle<br>FetalHeart	FHL2	four and a half LIM domains 2	2	-105343716	2274	2q12-q14	Hs.443687
3	44<br>5<br>54	Liver<br>Kidney<br>FetalLiver	PLG	plasminogen	6	161043272	5340	6q26	Hs.143436
3	44<br>5<br>54	Liver<br>Kidney<br>FetalLiver	KNG1	kininogen 1	3	187917813	3827	3q27	Hs.77741
3	44<br>5<br>54	Liver<br>Kidney<br>FetalLiver	PAH	phenylalanine hydroxylase	12	-101756234	5053	12q22-q24.2	Hs.643451

3	44<br>5<br>54	Liver<br>Kidney<br>FetalLiver	ALDH8A1	aldehyde dehydrogenase 8 family, member A1	6	-135280221	64577	6q23.2	Hs.486520
3	44<br>5<br>54	Liver<br>Kidney<br>FetalLiver	DPYS	dihydropyrimidinase	8	-105460828	1807	8q22	Hs.443161
3	44<br>5<br>54	Liver<br>Kidney<br>FetalLiver	HPD	4-hydroxyphenylpyruvate dioxygenase	12	-120761817	3242	12q24-qter	Hs.2899
3	43<br>46<br>74	FetalBrain<br>Amygdala<br>WholeBrain	FOXG1B	forkhead box G1B	14	28306037	2290	14q12-q13	Hs.632336
3	41<br>5<br>64	Thyroid<br>Kidney<br>FetalThyroid	KCNJ16	potassium inwardly-rectifying channel, subfamily J, member 16	17	65583020, 65612589	3773	17q23.1-q24.2	Hs.463985
3	41<br>49<br>78	Thyroid<br>Prostate<br>FetalHeart	ID1	inhibitor of DNA binding 1, dominant negative helix-loop-helix protein	20	29656752	3397	20q11	Hs.504609
3	41<br>45<br>64	Thyroid<br>LymphNode<br>FetalThyroid	TG	thyroglobulin	8	133948386	7038	8q24	Hs.584811
3	41<br>42<br>44	Thyroid<br>Lung<br>Liver	HSD17B6	hydroxysteroid (17-beta) dehydrogenase 6	12	55443374	8630	12q13	Hs.524513
3	40<br>64<br>76	Ovary<br>FetalThyroid<br>Adipocytes	MGP	matrix Gla protein	12	-14926093	4256	12p13.1-p12.3	Hs.365706
3	4<br>65<br>78	ExtraocularMuscle<br>OlfactoryBulb<br>FetalHeart	ABCA8	ATP-binding cassette, sub-family A (ABC1), member 8	17	-64375025	10351	17q24	Hs.58351
3	4<br>48<br>78	ExtraocularMuscle<br>Uterus<br>FetalHeart	OGN	osteoglycin (osteoinductive factor, mimecan)	9	-94186081	4969	9q22	Hs.109439
3	4<br>48<br>55	ExtraocularMuscle<br>Uterus<br>Testis	OSR2	odd-skipped related 2 (Drosophila)	8	100025861	116039	8q22.2	Hs.253247
3	4<br>47<br>78	ExtraocularMuscle<br>Heart<br>FetalHeart	ACTC	actin, alpha, cardiac muscle	15	-32869723	70	15q11-q14	Hs.118127
3	39<br>40<br>57	AdrenalCortex<br>Ovary<br>AdrenalGland	STAR	steroidogenic acute regulator	8	-38119374	6770	8p11.2	Hs.521535
3	38<br>53<br>56	SalivaryGland<br>Thymus<br>Trachea	PRR4	proline rich 4 (lacrimal)	12	-10889748	11272	12p13	Hs.408153
3	38<br>41<br>56	SalivaryGland<br>Thyroid<br>Trachea	SMR3B	submaxillary gland androgen regulated protein 3 homolog B (mouse)	4	71283383	10879	4q13.3	Hs.631946
3	37<br>72<br>78	HBEC<br>SmoothMuscle<br>FetalHeart	ADAM9	ADAM metallopeptidase domain 9 (meltrin gamma)	8	38973661	8754	8p11.23	Hs.591852
3	37<br>63<br>72	HBEC<br>CardiacMyocytes<br>SmoothMuscle	THBS1	thrombospondin 1	15	37660571	7057	15q15	Hs.164226
3	37<br>48	HBEC<br>Uterus	SNAI2	snail homolog 2 (Drosophila)	8	-49992795	6591	8q11	Hs.360174

	76	Adipocytes							
3	37	HBEC	ZBED2	zinc finger, BED-type containing 2	3	-112794437	79413	3q13.13	Hs.136912
	41	Thyroid							
	53	Thymus							
3	36	WholeBlood	CAMP	cathelicidin antimicrobial peptide	3	48239865	820	3p21.3	Hs.51120
	62	BoneMarrow							
	66	TestisGermCell							
3	35	Tonsil	CCL19	chemokine (C-C motif) ligand 19	9	-34679568	6363	9p13	Hs.50002
	45	LymphNode							
	53	Thymus							
3	35	Tonsil	CXCL13	chemokine (C-X-C motif) ligand 13 (B-cell chemoattractant)	4	78651929	10563	4q21	Hs.100431
	38	SalivaryGland							
	45	LymphNode							
3	33	FetalLung	CCL2	chemokine (C-C motif) ligand 2	17	29606408	6347	17q11.2-q12	Hs.303649
	63	CardiacMyocytes							
	72	SmoothMuscle							
3	33	FetalLung	APOA2	apolipoprotein A-II	1	-159458706	336	1q21-q23	Hs.237658
	44	Liver							
	54	FetalLiver							
3	33	FetalLung	HP	haptoglobin	16	70646008	3240	16q22.1	Hs.134406
	44	Liver							Hs.513711
	54	FetalLiver							
3	33	FetalLung	ITIH1	inter-alpha (globulin) inhibitor H1	3	52786647	3697	3p21.2-p21.1	Hs.420257
	44	Liver							
	54	FetalLiver							
3	33	FetalLung	APOH	apolipoprotein H (beta-2-glycoprotein I)	17	-61638613	350	17q23-qter	Hs.445358
	44	Liver							
	54	FetalLiver							
3	33	FetalLung	VTN	vitronectin	17	-23718427	7448	17q11	Hs.2257
	44	Liver							
	54	FetalLiver							
3	33	FetalLung	APOB	apolipoprotein B (including Ag(x) antigen)	2	-21077806	338	2p24-p23	Hs.120759
	44	Liver							
	54	FetalLiver							
3	33	FetalLung	AGT	angiotensinogen (serpin peptidase inhibitor, clade A, member 8)	1	-228904891	183	1q42-q43	Hs.19383
	44	Liver							
	54	FetalLiver							
3	33	FetalLung	AHSG	alpha-2-HS-glycoprotein	3	187813580	197	3q27	Hs.324746
	44	Liver							
	54	FetalLiver							
3	33	FetalLung	FGB	fibrinogen beta chain	4	155703595	2244	4q28	Hs.300774
	44	Liver							
	54	FetalLiver							
3	33	FetalLung	AMBP	alpha-1-microglobulin/bikunin precursor	9	-115862230	259	9q32-q33	Hs.436911
	44	Liver							
	54	FetalLiver							
3	33	FetalLung	FGA	fibrinogen alpha chain	4	-311449607	2243	4q28	Hs.351593
	44	Liver							
	54	FetalLiver							
3	33	FetalLung	SERPINC1	serpin peptidase inhibitor, clade C (antithrombin), member 1	1	-172139565	462	1q23-q25.1	Hs.75599
	44	Liver							
	54	FetalLiver							
3	33	FetalLung	ITIH3	inter-alpha (globulin) inhibitor H3	3	52803853	3699	3p21.2-p21.1	Hs.76716
	44	Liver							
	54	FetalLiver							

3	33	FetalLung	COL21A1	collagen, type XXI, alpha 1	6	-56029347	81578	6p12.3-p11.2|6p12.3-p11.2	Hs.47629
	4	ExtraocularMuscle							
	78	FetalHeart							
3	33	FetalLung	TNFAIP6	tumor necrosis factor, alpha-induced protein 6	2	151922350	7130	2q23.3	Hs.437322
	36	WholeBlood							
	72	SmoothMuscle							
3	31	UterusCorpus	MMP2	matrix metallopeptidase 2 (gelatinase A, 72kDa gelatinase, 72kDa type IV collagenase)	16	54070588	4313	16q13-q21	Hs.513617
	72	SmoothMuscle							
	76	Adipocytes							
3	31	UterusCorpus	MYLK	myosin, light polypeptide kinase	3	-124813834	4638	3q21	Hs.556600
	48	Uterus							
	49	Prostate							
3	30	Tongue	KRT1	keratin 1 (epidermolytic hyperkeratosis)	12	-51354720	3848	12q12-q13	Hs.80828
	53	Thymus							
	7	Skin							
3	30	Tongue	KRT6B	keratin 6B	12	-51126719	3854	12q12-q13	Hs.433845
	37	HBEC							
	7	Skin							
3	30	Tongue	SPINK5	serine peptidase inhibitor, Kazal type 5	5	147423758	11005	5q32	Hs.331555
	35	Tonsil							
	7	Skin							
3	30	Tongue	KRT13	keratin 13	17	-73821520	3860	17q12-q21.2	Hs.2785
	35	Tonsil							
	56	Trachea							
3	30	Tongue	SPRR1B	small proline-rich protein 1B (cornifin)	1	151270302	6699	1q21-q22	Hs.1076
	35	Tonsil							
	37	HBEC							
3	30	Tongue	KRT16	keratin 16 (focal non-epidermolytic palmoplantar keratoderma)	17	-37019558	3868	17q12-q21	Hs.2785
	35	Tonsil							
	37	HBEC							
3	3	LimbMuscle	PDK4	pyruvate dehydrogenase kinase, isozyme 4	7	-95050748	5166	7q21.3	Hs.8364
	40	Ovary							
	75	Islet							
3	3	LimbMuscle	SLC19A2	solute carrier family 19 (thiamine transporter), member 2	1	-167699791	10560	1q23.3	Hs.30246
	4	ExtraocularMuscle							
	78	FetalHeart							
3	3	LimbMuscle	ART3	ADP-ribosyltransferase 3	4	77214886	419	4p15.1-p14|4p15.1-p14|4p15.1-p14	Hs.24976
	4	ExtraocularMuscle							
	78	FetalHeart							
3	3	LimbMuscle	AGL	amylo-1, 6-glucosidase, 4-alpha-glucanotransferase (glycogen debranching enzyme, glycogen storage disease type III)	1	100088632, 100088227, 100099353, 100089118	178	1p21	Hs.904
	4	ExtraocularMuscle							
	78	FetalHeart							
3	3	LimbMuscle	PPP1R3C	protein phosphatase 1, regulatory (inhibitor) subunit 3C	10	-93378180	5507	10q23-q24	Hs.303090
	4	ExtraocularMuscle							
	78	FetalHeart							
3	3	LimbMuscle	GBAS	glioblastoma amplified sequence	7	55999789	2631	7p12	Hs.591069
	4	ExtraocularMuscle							
	78	FetalHeart							
3	3	LimbMuscle	SGCG	sarcoglycan, gamma (35kDa dystrophin-associated glycoprotein)	13	22653090	6445	13q12	Hs.37167
	4	ExtraocularMuscle							
	78	FetalHeart							
3	3	LimbMuscle	SUCLA2	succinate-CoA ligase, ADP-forming, beta subunit	13	-47414792	8803	13q12.2-q13.3	Hs.546323
	4	ExtraocularMuscle							
	78	FetalHeart							

3	3<br>4<br>78	LimbMuscle<br>ExtraocularMuscle<br>FetalHeart	ACTN2	actinin, alpha 2	1	234916430	88	1q42-q43	Hs.498178
3	3<br>4<br>78	LimbMuscle<br>ExtraocularMuscle<br>FetalHeart	SH3BGR	SH3 domain binding glutamic acid-rich protein	21	39739666, 39745649	6450	21q22.3	Hs.473847
3	3<br>4<br>78	LimbMuscle<br>ExtraocularMuscle<br>FetalHeart	CHCHD3	coiled-coil-helix-coiled-coil-helix domain containing 3	7	-132120163	54927	7q32.3-q33	Hs.444467
3	3<br>4<br>78	LimbMuscle<br>ExtraocularMuscle<br>FetalHeart	HSPB3	heat shock 27kDa protein 3	5	53787201	8988	5q11.2	Hs.41707
3	3<br>4<br>78	LimbMuscle<br>ExtraocularMuscle<br>FetalHeart	MYOM1	myomesin 1 (skelemin) 185kDa	18	-3056805	8736	18p11.32-p11.31	Hs.464469
3	3<br>4<br>78	LimbMuscle<br>ExtraocularMuscle<br>FetalHeart	LDB3	LIM domain binding 3	10	88418407	11155	10q22.3-q23.2	Hs.49998
3	3<br>4<br>69	LimbMuscle<br>ExtraocularMuscle<br>Hypothalamus	CAPN3	calpain 3, (p94)	15	40438989, 40481879, 40484268, 40427592	825	15q15.1-q21.1	Hs.143261
3	3<br>36<br>76	LimbMuscle<br>WholeBlood<br>Adipocytes	ACSL1	acyl-CoA synthetase long-chain family member 1	4	-185913743	2180	4q34-q35	Hs.406678
3	3<br>32<br>4	LimbMuscle<br>PsoasMuscle<br>ExtraocularMuscle	PYGM	phosphorylase, glycogen, muscle (McArdle syndrome, glycogen storage disease type V)	11	-64270605	5837	11q12-q13.2	Hs.154084
3	3<br>32<br>4	LimbMuscle<br>PsoasMuscle<br>ExtraocularMuscle	MYOZ1	myozenin 1	10	-75061420	58529	10q22.1	Hs.238756
3	3<br>32<br>4	LimbMuscle<br>PsoasMuscle<br>ExtraocularMuscle	RYR1	ryanodine receptor 1 (skeletal)	19	43616179	6261	19q13.1	Hs.466664
3	3<br>32<br>4	LimbMuscle<br>PsoasMuscle<br>ExtraocularMuscle	AMPD1	adenosine monophosphate deaminase 1 (isoform M)	1	-115017244	270	1p13	Hs.89570
3	3<br>30<br>4	LimbMuscle<br>Tongue<br>ExtraocularMuscle	FHL1	four and a half LIM domains 1	X	135057345	2273	Xq26	Hs.435369
3	3<br>30<br>32	LimbMuscle<br>Tongue<br>PsoasMuscle	SLN	sarcolipin	11	-107083318	6588	11q22-q23	Hs.334629
3	27<br>48<br>49	Appendix<br>Uterus<br>Prostate	ACTG2	actin, gamma 2, smooth muscle, enteric	2	73973600	72	2p13.1	Hs.516105
3	25<br>26<br>73	MedullaOblongata<br>Pons<br>CaudateNucleus	GPR88	G protein-coupled receptor 88	1	100776280	54112	1p21.3	Hs.170053
3	22<br>36<br>62	BM-CD34<br>WholeBlood<br>BoneMarrow	CLC	Charcot-Leyden crystal protein	19	-44913735	1178	19q13.1	Hs.889
3	21<br>22<br>62	BM-CD105Endothelial<br>BM-CD34<br>BoneMarrow	IGLL1	immunoglobulin lambda-like polypeptide 1	22	-22245313	3543	22q11.23	Hs.348935
3	21	BM-CD105Endothelial	DNTT	deoxynucleotidyltransferase, terminal	10	98054074	1791	10q23-q24	Hs.534206

	22	BM-CD34							
	53	Thymus							
3	20	BM-CD33Myeloid	KCNH7	potassium voltage-gated channel, subfamily H (eag-related), member 7	2	-325924166	90134	2q24.2	Hs.470445
	39	AdrenalCortex							
	57	AdrenalGland							
3	20	BM-CD33Myeloid	IER3	immediate early response 3	6	-34936359	8870	6p21.3	Hs.591785
	37	HBEC							
	72	SmoothMuscle							
3	20	BM-CD33Myeloid	MPO	myeloperoxidase	17	-53702215	4353	17q23.1	Hs.458272
	22	BM-CD34							
	62	BoneMarrow							
3	20	BM-CD33Myeloid	ELA2	elastase 2, neutrophil	19	803290	1991	19p13.3	Hs.99863
	22	BM-CD34							
	62	BoneMarrow							
3	20	BM-CD33Myeloid	AZU1	azurocidin 1 (cationic antimicrobial protein 37)	19	778830	566	19p13.3	Hs.72885
	22	BM-CD34							
	62	BoneMarrow							
3	20	BM-CD33Myeloid	MS4A3	membrane-spanning 4-domains, subfamily A, member 3 (hematopoietic cell-specific)	11	59580676	932	11q12.1	Hs.99960
	22	BM-CD34							
	62	BoneMarrow							
3	2	BronchialEpitelia	PROS1	protein S (alpha)	3	-95074646	5627	3q11.2	Hs.64016
	5	Kidney							
	78	FetalHeart							
3	2	BronchialEpitelia	FOXA1	forkhead box A1	14	-37128941	3169	14q12-q13	Hs.163484
	49	Prostate							
	56	Trachea							
3	2	BronchialEpitelia	MSMB	microseminoprotein, beta-	10	51219558	4477	10q11.2	Hs.255462
	49	Prostate							
	56	Trachea							
3	2	BronchialEpitelia	SCGB2A1	secretoglobin, family 2A, member 1	11	61732715	4246	11q13	Hs.97644
	48	Uterus							
	75	Islet							
3	2	BronchialEpitelia	CYP4B1	cytochrome P450, family 4, subfamily B, polypeptide 1	1	47037328	1580	1p34-p12	Hs.436317
	42	Lung							
	56	Trachea							
3	2	BronchialEpitelia	FCGBP	Fc fragment of IgG binding protein	19	-45045810	8857	19q13.1	Hs.111732
	41	Thyroid							
	56	Trachea							
3	2	BronchialEpitelia	CLDN3	claudin 3	7	-72821263	1365	7q11.23	Hs.520943
	41	Thyroid							
	49	Prostate							
3	19	PB-CD19BCells	IGHV1-69	immunoglobulin heavy variable 1-69	14		28461	14q32.32-q32.33	Hs.634941
	35	Tonsil							
	45	LymphNode							
3	19	PB-CD19BCells	PLEKHF2	pleckstrin homology domain containing, family F (with FYVE domain) member 2	8	96215213	79666	8q22.1	Hs.29724
	23	PB-BDCA4DentriticCells							
	36	WholeBlood							
3	19	PB-CD19BCells	TCL1A	T-cell leukemia/lymphoma 1A	14	-95246057	8115	14q32.1	Hs.2484
	23	PB-BDCA4DentriticCells							
	35	Tonsil							
3	19	PB-CD19BCells	IRF8	interferon regulatory factor 8	16	84490274	3394	16q24.1	Hs.137427
	22	BM-CD34							
	23	PB-BDCA4DentriticCells							
3	19	PB-CD19BCells	PELI1	pellino homolog 1 (Drosophila)	2	-64173499	57162	2p13.3	Hs.7886
	20	BM-CD33Myeloid							
	36	WholeBlood							

3	18<br>23<br>36	PB-CD56NKCells<br>PB-BDCA4DentriticCells<br>WholeBlood	GZMB	granzyme B (granzyme 2, cytotoxic T-lymphocyte-associated serine esterase 1)	14	-24170003	3002	14q11.2	Hs.1051
3	17<br>20<br>44	PB-CD14Monocytes<br>BM-CD33Myeloid<br>Liver	ASGR1	asialoglycoprotein receptor 1	17	-7017476	432	17p13.2	Hs.12056
3	17<br>20<br>36	PB-CD14Monocytes<br>BM-CD33Myeloid<br>WholeBlood	USP3	ubiquitin specific peptidase 3	15	61583862	9960	15q22.3	Hs.458499
3	17<br>20<br>36	PB-CD14Monocytes<br>BM-CD33Myeloid<br>WholeBlood	CFP	complement factor properdin	X	-47368571	5199	Xp11.3-p11.23	Hs.53155
3	17<br>20<br>36	PB-CD14Monocytes<br>BM-CD33Myeloid<br>WholeBlood	SPI1	spleen focus forming virus (SFFV) proviral integration oncogene spi1	11	-47332987	6688	11p11.2	Hs.502511
3	17<br>20<br>36	PB-CD14Monocytes<br>BM-CD33Myeloid<br>WholeBlood	VNN2	vanin 2	6	-133106702	8875	6q23-q24	Hs.293130
3	17<br>20<br>36	PB-CD14Monocytes<br>BM-CD33Myeloid<br>WholeBlood	TLR2	toll-like receptor 2	4	154824890	7097	4q32	Hs.519033
3	17<br>20<br>36	PB-CD14Monocytes<br>BM-CD33Myeloid<br>WholeBlood	APOBEC3A	apolipoprotein B mRNA editing enzyme, catalytic polypeptide-like 3A	22	37683568	200315	22q13.1-q13.2	Hs.348983
3	17<br>20<br>36	PB-CD14Monocytes<br>BM-CD33Myeloid<br>WholeBlood	FRAT2	frequently rearranged in advanced T-cell lymphomas 2	10	-99082244	23401	10q24.1	Hs.140720
3	17<br>20<br>36	PB-CD14Monocytes<br>BM-CD33Myeloid<br>WholeBlood	CLEC7A	C-type lectin domain family 7, member A	12	-20331837	64581	12p13.2	Hs.143929
3	17<br>20<br>36	PB-CD14Monocytes<br>BM-CD33Myeloid<br>WholeBlood	PILRA	paired immunoglobin-like type 2 receptor alpha	7	99809003	29992	7q22.1	Hs.444407
3	17<br>20<br>36	PB-CD14Monocytes<br>BM-CD33Myeloid<br>WholeBlood	LST1	leukocyte specific transcript 1	6	31661949,<br>3000093,<br>2801557,<br>31662603,<br>3000747,<br>2802211	7940	6p21.3	Hs.436066
3	17<br>20<br>36	PB-CD14Monocytes<br>BM-CD33Myeloid<br>WholeBlood	FPR1	formyl peptide receptor 1	19	-56940838	2357	19q13.4	Hs.753
3	17<br>20<br>36	PB-CD14Monocytes<br>BM-CD33Myeloid<br>WholeBlood	DHRS8	dehydrogenase/reductase (SDR family) member 8	4	-88476788	51170	4q22.1	Hs.282984
3	17<br>20<br>36	PB-CD14Monocytes<br>BM-CD33Myeloid<br>WholeBlood	SULT1A1	sulfotransferase family, cytosolic, 1A, phenol-preferring, member 1	16	-28524418	6817	16p12.1	Hs.567342
3	17<br>20<br>36	PB-CD14Monocytes<br>BM-CD33Myeloid<br>WholeBlood	LILRA5	leukocyte immunoglobulin-like receptor, subfamily A (with TM domain), member 5	19	-238049124	353514	19q13.4	Hs.631592
3	17<br>20<br>36	PB-CD14Monocytes<br>BM-CD33Myeloid<br>WholeBlood	LILRA2	leukocyte immunoglobulin-like receptor, subfamily A (with TM domain), member 2	19	59777070	11027	19q13.4	Hs.534394

3	17<br>20<br>3	PB-CD14Monocytes<br>BM-CD33Myeloid<br>LimbMuscle	KLF10	Kruppel-like factor 10	8	-207460384	7071	8q22.2	Hs.435001
3	17<br>20<br>23	PB-CD14Monocytes<br>BM-CD33Myeloid<br>PB-BDCA4DentriticCells	CAPG	capping protein (actin filament), gelsolin-like	2	-85475381	822	2p11.2	Hs.516155
3	17<br>20<br>23	PB-CD14Monocytes<br>BM-CD33Myeloid<br>PB-BDCA4DentriticCells	KLF4	Kruppel-like factor 4 (gut)	9	-109286955	9314	9q31	Hs.376206
3	17<br>20<br>23	PB-CD14Monocytes<br>BM-CD33Myeloid<br>PB-BDCA4DentriticCells	SCAP2	src family associated phosphoprotein 2	7	-26673214	8935	7p21-p15	Hs.200770
3	17<br>20<br>22	PB-CD14Monocytes<br>BM-CD33Myeloid<br>BM-CD34	SERPINB1	serpin peptidase inhibitor, clade B (ovalbumin), member 1	6	-2778734	1992	6p25	Hs.381167
3	17<br>19<br>23	PB-CD14Monocytes<br>PB-CD19BCells<br>PB-BDCA4DentriticCells	HLA-DMA	major histocompatibility complex, class II, DM alpha	6	-41424089	3108	6p21.3	Hs.351279
3	17<br>19<br>20	PB-CD14Monocytes<br>PB-CD19BCells<br>BM-CD33Myeloid	SNX2	sorting nexin 2	5	122138648	6643	5q23	Hs.134822
3	17<br>18<br>20	PB-CD14Monocytes<br>PB-CD56NKCells<br>BM-CD33Myeloid	TCIRG1	T-cell, immune regulator 1, ATPase, H+ transporting, lysosomal V0 subunit A3	11	67563058,<br>67567022	10312	11q13.2	Hs.495985
3	17<br>18<br>20	PB-CD14Monocytes<br>PB-CD56NKCells<br>BM-CD33Myeloid	MAFB	v-maf musculoaponeurotic fibrosarcoma oncogene homolog B (avian)	20	-38747932	9935	20q11.2-q13.1	Hs.642679
3	16<br>54<br>62	BM-CD71EarlyErythroid<br>FetalLiver<br>BoneMarrow	TRIM58	tripartite motif-containing 58	1	246087123	25893	1q44	Hs.323858
3	16<br>3<br>4	BM-CD71EarlyErythroid<br>LimbMuscle<br>ExtraocularMuscle	IQWD1	IQ motif and WD repeats 1	1	166172531	55827	1q24.2	Hs.435741
3	16<br>21<br>62	BM-CD71EarlyErythroid<br>BM-CD105Endothelial<br>BoneMarrow	CA1	carbonic anhydrase I	8	-86427708	759	8q13-q22.1	Hs.23118
3	16<br>21<br>54	BM-CD71EarlyErythroid<br>BM-CD105Endothelial<br>FetalLiver	GYPA	glycophorin A (MNS blood group)	4	-145249906	2993	4q28.2-q31.1	Hs.434973
3	16<br>21<br>54	BM-CD71EarlyErythroid<br>BM-CD105Endothelial<br>FetalLiver	SPTA1	spectrin, alpha, erythrocytic 1 (elliptocytosis 2)	1	-156847119	6708	1q21	Hs.119825
3	16<br>20<br>5	BM-CD71EarlyErythroid<br>BM-CD33Myeloid<br>Kidney	MGC14376		17	-1561553	84981	17p13.3	Hs.417157
3	16<br>2<br>21	BM-CD71EarlyErythroid<br>BronchialEpitelia<br>BM-CD105Endothelial	SLC22A4	solute carrier family 22 (organic cation transporter), member 4	5	131658043	6583	5q31.1	Hs.310591<br>Hs.640104
3	14<br>18<br>36	PB-CD8TCells<br>PB-CD56NKCells<br>WholeBlood	GZMH	granzyme H (cathepsin G-like 2, protein h-CCPX)	14	-24145532	2999	14q11.2	Hs.348264
3	14<br>18<br>36	PB-CD8TCells<br>PB-CD56NKCells<br>WholeBlood	KLRF1	killer cell lectin-like receptor subfamily F, member 1	12	9871343	51348	12p12.3-13.2	Hs.183125

3	14<br>18<br>36	PB-CD8TCells<br>PB-CD56NKCells<br>WholeBlood	CST7	cystatin F (leukocystatin)	20	24877865	8530	20p11.21	Hs.143212
3	14<br>18<br>3	PB-CD8TCells<br>PB-CD56NKCells<br>LimbMuscle	SYNE2	spectrin repeat containing, nuclear envelope 2	14	63389453, 63751902, 63740825, 63750614, 63612085	23224	14q23.2	Hs.525392
3	14<br>18<br>19	PB-CD8TCells<br>PB-CD56NKCells<br>PB-CD19BCells	GPR18	G protein-coupled receptor 18	13	-98704972	2841	13q32	Hs.631765
3	14<br>17<br>18	PB-CD8TCells<br>PB-CD14Monocytes<br>PB-CD56NKCells	GPR65	G protein-coupled receptor 65	14	87541248	8477	14q31-q32.1	Hs.513440
3	14<br>17<br>18	PB-CD8TCells<br>PB-CD14Monocytes<br>PB-CD56NKCells	IL10RA	interleukin 10 receptor, alpha	11	117362318	3587	11q23	Hs.504035
3	14<br>15<br>53	PB-CD8TCells<br>PB-CD4TCells<br>Thymus	IL7R	interleukin 7 receptor	5	35892747	3575	5p13	Hs.591742
3	14<br>15<br>53	PB-CD8TCells<br>PB-CD4TCells<br>Thymus	BCL11B	B-cell CLL/lymphoma 11B (zinc finger protein)	14	-98705379	64919	14q32.2	Hs.510396
3	14<br>15<br>23	PB-CD8TCells<br>PB-CD4TCells<br>PB-BDCA4DentriticCells	C6orf111	chromosome 6 open reading frame 111	6	-99954923	25957	6q16.3	Hs.591345
3	14<br>15<br>19	PB-CD8TCells<br>PB-CD4TCells<br>PB-CD19BCells	CLEC2D	C-type lectin domain family 2, member D	12	9713575	29121	12p13	Hs.268326
3	14<br>15<br>19	PB-CD8TCells<br>PB-CD4TCells<br>PB-CD19BCells	FAIM3	Fas apoptotic inhibitory molecule 3	1	-205144353	9214	1q32.1	Hs.58831
3	14<br>15<br>18	PB-CD8TCells<br>PB-CD4TCells<br>PB-CD56NKCells	PRKCH	protein kinase C, eta	14	60858267	5583	14q22-q23	Hs.333907
3	14<br>15<br>18	PB-CD8TCells<br>PB-CD4TCells<br>PB-CD56NKCells	SEMA4D	sema domain, immunoglobulin domain (Ig), transmembrane domain (TM) and short cytoplasmic domain, (semaphorin) 4D	9	-91181971	10507	9q22-q31	Hs.494406
3	14<br>15<br>18	PB-CD8TCells<br>PB-CD4TCells<br>PB-CD56NKCells	TRAF3IP3	TRAF3 interacting protein 3	1	207996146	80342	1q32.3-q41	Hs.147434
3	14<br>15<br>18	PB-CD8TCells<br>PB-CD4TCells<br>PB-CD56NKCells	ZAP70	zeta-chain (TCR) associated protein kinase 70kDa	2	97696462, 97717300	7535	2q12	Hs.234569
3	14<br>15<br>18	PB-CD8TCells<br>PB-CD4TCells<br>PB-CD56NKCells	BTN3A3	butyrophilin, subfamily 3, member A3	6	26548741	10384	6p21.3	Hs.167741
3	14<br>15<br>18	PB-CD8TCells<br>PB-CD4TCells<br>PB-CD56NKCells	BTN3A2	butyrophilin, subfamily 3, member A2	6	26473376	11118	6p22.1	Hs.376046
3	14<br>15<br>18	PB-CD8TCells<br>PB-CD4TCells<br>PB-CD56NKCells	TXK	TXK tyrosine kinase	4	-47763517	7294	4p12	Hs.479669

3	14<br>15<br>18	PB-CD8TCells<br>PB-CD4TCells<br>PB-CD56NKCells	MYBL1	v-myb myeloblastosis viral oncogene homolog (avian)-like 1	8		4603	8q22	Hs.445898
3	14<br>15<br>18	PB-CD8TCells<br>PB-CD4TCells<br>PB-CD56NKCells	KLRK1	killer cell lectin-like receptor subfamily K, member 1	12	-10416219	22914	12p13.2-p12.3	Hs.387787
3	14<br>15<br>18	PB-CD8TCells<br>PB-CD4TCells<br>PB-CD56NKCells	DUSP2	dual specificity phosphatase 2	2	-96172643	1844	2q11	Hs.1183
3	14<br>15<br>18	PB-CD8TCells<br>PB-CD4TCells<br>PB-CD56NKCells	CD7	CD7 molecule	17	-77866034	924	17q25.2-q25.3	Hs.36972
3	14<br>15<br>18	PB-CD8TCells<br>PB-CD4TCells<br>PB-CD56NKCells	WASPIP	Wiskott-Aldrich syndrome protein interacting protein	2	-175132548	7456	2q31.1	Hs.591641
3	14<br>15<br>17	PB-CD8TCells<br>PB-CD4TCells<br>PB-CD14Monocytes	ZFP36L2	zinc finger protein 36, C3H type-like 2	2	-43304210	678	2p22.3-p21	Hs.503093<br>Hs.636758
3	13<br>66<br>77	721-BLymphoblasts<br>TestisGermCell<br>TestiSeminiferousTubule	NXF3	nuclear RNA export factor 3	X	-102217407	56000	Xq22-q23	Hs.60386
3	13<br>35<br>45	721-BLymphoblasts<br>Tonsil<br>LymphNode	CR2	complement component (3d/Epstein Barr virus) receptor 2	1	205694292	1380	1q32	Hs.445757
3	13<br>3<br>78	721-BLymphoblasts<br>LimbMuscle<br>FetalHeart	VDAC1	voltage-dependent anion channel 1	5	-133335505	7416	5q31	Hs.519320
3	13<br>23<br>65	721-BLymphoblasts<br>PB-BDCA4DentriticCells<br>OlfactoryBulb	RGS1	regulator of G-protein signalling 1	1	190811479	5996	1q31	Hs.75256
3	13<br>21<br>22	721-BLymphoblasts<br>BM-CD105Endothelial<br>BM-CD34	MTHFD1	methylenetetrahydrofolate dehydrogenase (NADP+ dependent) 1, methenyltetrahydrofolate cyclohydrolase, formyltetrahydrofolate synthetase	14	63924845	4522	14q24	Hs.632340
3	13<br>21<br>22	721-BLymphoblasts<br>BM-CD105Endothelial<br>BM-CD34	EXOSC8	exosome component 8	13	36472916	11340	13q13.1	Hs.294041
3	13<br>21<br>22	721-BLymphoblasts<br>BM-CD105Endothelial<br>BM-CD34	MCM6	MCM6 minichromosome maintenance deficient 6 (MIS5 homolog, S. pombe) (S. cerevisiae)	2	-136313673	4175	2q21	Hs.444118
3	13<br>21<br>22	721-BLymphoblasts<br>BM-CD105Endothelial<br>BM-CD34	KIAA0101	KIAA0101	15	-62444265	9768	15q22.31	Hs.81892
3	13<br>21<br>22	721-BLymphoblasts<br>BM-CD105Endothelial<br>BM-CD34	BUB1B	BUB1 budding uninhibited by benzimidazoles 1 homolog beta (yeast)	15	38240529	701	15q15	Hs.631699
3	13<br>21<br>22	721-BLymphoblasts<br>BM-CD105Endothelial<br>BM-CD34	PAICS	phosphoribosylaminoimidazole carboxylase, phosphoribosylaminoimidazole succinocarboxamide synthetase	4	56996697	10606	4pter-q21	Hs.518774
3	13<br>19<br>45	721-BLymphoblasts<br>PB-CD19BCells<br>LymphNode	BIRC3	baculoviral IAP repeat-containing 3	11	101693403	330	11q22	Hs.127799

3	13<br>19<br>35	721-BLymphoblasts<br>PB-CD19BCells<br>Tonsil	CD79A	CD79a molecule, immunoglobulin-associated alpha	19	47073029	973	19q13.2	Hs.631567
3	13<br>19<br>35	721-BLymphoblasts<br>PB-CD19BCells<br>Tonsil	CD19	CD19 molecule	16	28850760	930	16p11.2	Hs.632193
3	13<br>19<br>23	721-BLymphoblasts<br>PB-CD19BCells<br>PB-BDCA4DentriticCells	TNFRSF17	tumor necrosis factor receptor superfamily, member 17	16	11966464	608	16p13.1	Hs.2556
3	13<br>18<br>36	721-BLymphoblasts<br>PB-CD56NKCells<br>WholeBlood	CD300A	CD300a molecule	17	69974116	11314	17q25.1	Hs.9688
3	13<br>17<br>23	721-BLymphoblasts<br>PB-CD14Monocytes<br>PB-BDCA4DentriticCells	IRF7	interferon regulatory factor 7	11	-602555	3665	11p15.5	Hs.166120
3	13<br>16<br>21	721-BLymphoblasts<br>BM-CD71EarlyErythroid<br>BM-CD105Endothelial	DLG7	discs, large homolog 7 (Drosophila)	14	-54684600	9787	14q22.3	Hs.77695
3	13<br>16<br>21	721-BLymphoblasts<br>BM-CD71EarlyErythroid<br>BM-CD105Endothelial	SMC4	structural maintenance of chromosomes 4	3	161600117,<br>161600123,<br>161601040	10051	3q26.1	Hs.58992
3	13<br>16<br>21	721-BLymphoblasts<br>BM-CD71EarlyErythroid<br>BM-CD105Endothelial	RRM1	ribonucleotide reductase M1 polypeptide	11	4072586	6240	11p15.5	Hs.558393
3	13<br>16<br>21	721-BLymphoblasts<br>BM-CD71EarlyErythroid<br>BM-CD105Endothelial	TFDP1	transcription factor Dp-1	13	113287056	7027	13q34	Hs.79353
3	13<br>16<br>21	721-BLymphoblasts<br>BM-CD71EarlyErythroid<br>BM-CD105Endothelial	APOBEC3B	apolipoprotein B mRNA editing enzyme, catalytic polypeptide-like 3B	22	37708350	9582	22q13.1-q13.2	Hs.226307
3	13<br>14<br>19	721-BLymphoblasts<br>PB-CD8TCells<br>PB-CD19BCells	LRAP		5	96241023	64167	5q15	Hs.591249
3	12<br>72<br>76	Placenta<br>SmoothMuscle<br>Adipocytes	FBN1	fibrillin 1	15	-46487796	2200	15q21.1	Hs.591133
3	12<br>63<br>72	Placenta<br>CardiacMyocytes<br>SmoothMuscle	PAPPA	pregnancy-associated plasma protein A, pappalysin 1	9	117955891	5069	9q33.2	Hs.494928
3	12<br>54<br>60	Placenta<br>FetalLiver<br>PituitaryGland					645959		
3	12<br>54<br>60	Placenta<br>FetalLiver<br>PituitaryGland	PEG10	paternally expressed 10	7	94123572	23089	7q21	Hs.147492
3	12<br>5<br>72	Placenta<br>Kidney<br>SmoothMuscle	CRIM1	cysteine rich transmembrane BMP regulator 1 (chordin-like)	2	36436900	51232	2p21	Hs.332847
3	12<br>49<br>5	Placenta<br>Prostate<br>Kidney	ABP1	amiloride binding protein 1 (amine oxidase (copper-containing))	7	150180505	26	7q34-q36	Hs.438823
3	12<br>44<br>54	Placenta<br>Liver<br>FetalLiver	HSD17B2	hydroxysteroid (17-beta) dehydrogenase 2	16	80626363	3294	16q24.1-q24.2	Hs.162795

3	12	Placenta	MAFF	v-maf musculoaponeurotic fibrosarcoma oncogene homolog F (avian)	22	36927943, 36939487	23764	22q13.1	Hs.517617
	42	Lung							
	78	FetalHeart							
3	12	Placenta	ADM	adrenomedullin	11	10283217	133	11p15.4	Hs.441047
	42	Lung							
	76	Adipocytes							
3	12	Placenta	CSH1	chorionic somatomammotropin hormone 1 (placental lactogen)	17	-59326005	1442	17q24.2	Hs.406754
	42	Lung							
	60	PituitaryGland							
3	12	Placenta	FRZB	frizzled-related protein	2	-183406981	2487	2qter	Hs.128453
	4	ExtraocularMuscle							
	56	Trachea							
3	12	Placenta	CYP11A1	cytochrome P450, family 11, subfamily A, polypeptide 1	15	-72417156	1583	15q23-q24	Hs.303980
	39	AdrenalCortex							
	57	AdrenalGland							
3	12	Placenta	DKK1	dickkopf homolog 1 (Xenopus laevis)	10	53744046	22943	10q11.2	Hs.40499
	37	HBEC							
	76	Adipocytes							
3	12	Placenta	COL4A1	collagen, type IV, alpha 1	13	-109599311	1282	13q34	Hs.17441
	33	FetalLung							
	78	FetalHeart							
3	12	Placenta	RHOBTB1	Rho-related BTB domain containing 1	10	-62299205	9886	10q21.2	Hs.148670
	3	LimbMuscle							
	5	Kidney							
3	12	Placenta					653991		
	22	BM-CD34							
	62	BoneMarrow							
3	12	Placenta	PRG2	proteoglycan 2, bone marrow (natural killer cell activator, eosinophil granule major basic protein)	11	-56911410	5553	11q12	Hs.512633
	22	BM-CD34							
	62	BoneMarrow							
3	12	Placenta	TGFBR3	transforming growth factor, beta receptor III (betaglycan, 300kDa)	1	-91920574	7049	1p33-p32	Hs.482390
	18	PB-CD56NKCells							
	40	Ovary							
3	12	Placenta	IGF2BP3	insulin-like growth factor 2 mRNA binding protein 3	7	-23316353	10643	7p11	Hs.432616
	13	721-BLymphoblasts							
	72	SmoothMuscle							
3	10	AtrioVentricularNode	PLA2G2A	phospholipase A2, group IIA (platelets, synovial fluid)	1	-20174517	5320	1p35	Hs.466804
	40	Ovary							
	49	Prostate							
3	10	AtrioVentricularNode	CDH19	cadherin 19, type 2	18	-62322300	28513	18q22-q23	Hs.42771
	11	DRG							
	65	OlfactoryBulb							
3	1	Hippocampus	HOMER1	homer homolog 1 (Drosophila)	5	-78705542	9456	5q14.2	Hs.591761
	51	PrefrontalCortex							
	78	FetalHeart							
3	1	Hippocampus	INPP5F	inositol polyphosphate-5-phosphatase F	10	121475598, 121568775	22876	10q26.11	Hs.369755
	51	PrefrontalCortex							
	69	Hypothalamus							
3	1	Hippocampus	EMX2	empty spiracles homolog 2 (Drosophila)	10	119291945	2018	10q26.1	Hs.202095
	48	Uterus							
	5	Kidney							
3	1	Hippocampus	SCHIP1	schwannomin interacting protein 1	3	160474237	29970	3q25.33	Hs.134665
	46	Amygdala							
	78	FetalHeart							
3	1	Hippocampus	PCDH8	protocadherin 8	13	-52316111	5100	13q14.3-q21.1	Hs.19492
	46	Amygdala							

	74	WholeBrain							
3	1	Hippocampus	LOC653754		8		653754	8p22	Hs.592598
	46	Amygdala							
	51	PrefrontalCortex							
3	1	Hippocampus	AK5	adenylate kinase 5	1	77520874, 77520329	26289	1p31	Hs.559718
	46	Amygdala							
	51	PrefrontalCortex							
3	1	Hippocampus	RAPGEF4	Rap guanine nucleotide exchange factor (GEF) 4	2	173308852	11069	2q31-q32	Hs.470646
	46	Amygdala							
	51	PrefrontalCortex							
3	1	Hippocampus	FGF13	fibroblast growth factor 13	X	-137541401	2258	Xq26.3	Hs.6540
	43	FetalBrain							
	46	Amygdala							
3	1	Hippocampus	HPCA	hippocalcin	1	33124684	3208	1p35-p34.2	Hs.632391
	25	MedullaOblongata							
	73	CaudateNucleus							
4	66	TestisGermCell	BRDT	bromodomain, testis-specific	1	92190333, 92187719	676	1p22.1	Hs.482520
	67	TestisIntersitial							
	68	TestisLeydigCell							
	77	TestiSeminiferousTubule							
4	66	TestisGermCell	DDX4	DEAD (Asp-Glu-Ala-Asp) box polypeptide 4	5	55070532	54514	5p15.2-p13.1	Hs.223581
	67	TestisIntersitial							
	68	TestisLeydigCell							
	77	TestiSeminiferousTubule							
4	66	TestisGermCell	HSPA4L	heat shock 70kDa protein 4-like	4	128922902	22824	4q28	Hs.135554
	67	TestisIntersitial							
	68	TestisLeydigCell							
	77	TestiSeminiferousTubule							
4	55	Testis	RPL39L	ribosomal protein L39-like	3	-188321434	116832	3q27	Hs.478582
	67	TestisIntersitial							
	68	TestisLeydigCell							
	77	TestiSeminiferousTubule							
4	55	Testis	ACTL7B	actin-like 7B	9	-110656691	10880	9q31	Hs.534390
	67	TestisIntersitial							
	68	TestisLeydigCell							
	77	TestiSeminiferousTubule							
4	55	Testis	ACSBG2	acyl-CoA synthetase bubblegum family member 2	19	6086709	81616	19p13.3	Hs.465720
	67	TestisIntersitial							
	68	TestisLeydigCell							
	77	TestiSeminiferousTubule							
4	55	Testis	C19orf36	chromosome 19 open reading frame 36	19	2047867	113177	19p13.3	Hs.424049
	67	TestisIntersitial							
	68	TestisLeydigCell							
	77	TestiSeminiferousTubule							
4	55	Testis	CLPB	ClpB caseinolytic peptidase B homolog (E. coli)	11	-71681119	81570	11q13.4	Hs.523877
	66	TestisGermCell							
	67	TestisIntersitial							
	77	TestiSeminiferousTubule							
4	55	Testis	INSL3	insulin-like 3 (Leydig cell)	19	-17788321	3640	19p13.2-p12	Hs.37062
	66	TestisGermCell							
	67	TestisIntersitial							
	68	TestisLeydigCell							
4	53	Thymus	ADIPOQ	adiponectin, C1Q and collagen domain containing	3	188043156	9370	3q27	Hs.80485
	7	Skin							
	76	Adipocytes							
	9	CiliaryGanglion							

4	47	Heart	MYL9	myosin, light polypeptide 9, regulatory	20	34603310	10398	20q11.23	Hs.504687
	48	Uterus							
	49	Prostate							
	78	FetalHeart							
4	44	Liver	FABP1	fatty acid binding protein 1, liver	2	-88203624	2168	2p11	Hs.380135
	5	Kidney							
	50	Pancreas							
	54	FetalLiver							
4	41	Thyroid	CLEC3B	C-type lectin domain family 3, member B	3	45042773	7123	3p22-p21.3	Hs.476092
	42	Lung							
	47	Heart							
	78	FetalHeart							
4	4	ExtraocularMuscle	MFAP5	microfibrillar associated protein 5	12	-8689806	8076	12p13.1-p12.3	Hs.512842
	48	Uterus							
	72	SmoothMuscle							
	76	Adipocytes							
4	4	ExtraocularMuscle	CFD	complement factor D (adipsin)	19	810664	1675	19p13.3	Hs.155597
	42	Lung							
	47	Heart							
	76	Adipocytes							
4	4	ExtraocularMuscle	MATN2	matrilin 2	8	98950486	4147	8q22	Hs.189445
	41	Thyroid							
	48	Uterus							
	78	FetalHeart							
4	39	AdrenalCortex	FLJ10847		17	19377758	55244	17p11.2	Hs.232054
	44	Liver							
	5	Kidney							
	57	AdrenalGland							
4	39	AdrenalCortex	RARRES2	retinoic acid receptor responder (tazarotene induced) 2	7	-149666351	5919	7q36.1	Hs.438823
	42	Lung							
	44	Liver							
	57	AdrenalGland							
4	38	SalivaryGland	DEFB1	defensin, beta 1	8	-6715510	1672	8p23.2-p23.1	Hs.32949
	5	Kidney							
	50	Pancreas							
	75	Islet							
4	37	HBEC	MMP1	matrix metallopeptidase 1 (interstitial collagenase)	11	-102165860	4312	11q22.3	Hs.83169
	63	CardiacMyocytes							
	72	SmoothMuscle							
	75	Islet							
4	37	HBEC	CAV2	caveolin 2	7	115926679	858	7q31.1	Hs.212332 Hs.603096
	4	ExtraocularMuscle							
	41	Thyroid							
	78	FetalHeart							
4	33	FetalLung	POSTN	periostin, osteoblast specific factor	13	-37034779	10631	13q13.3	Hs.136348
	63	CardiacMyocytes							
	72	SmoothMuscle							
	78	FetalHeart							
4	33	FetalLung	PTX3	pentraxin-related gene, rapidly induced by IL-1 beta	3	158637300	5806	3q25	Hs.591286
	63	CardiacMyocytes							
	72	SmoothMuscle							
	76	Adipocytes							
4	33	FetalLung	HBG2	hemoglobin, gamma G	11	-5230996	3048	11p15.5	Hs.302145
	54	FetalLiver							
	64	FetalThyroid							
	78	FetalHeart							

4	33<br>44<br>54<br>75	FetalLung<br>Liver<br>FetalLiver<br>Islet	FGG	fibrinogen gamma chain	4	-311489914	2266	4q28	Hs.546255
4	33<br>44<br>54<br>75	FetalLung<br>Liver<br>FetalLiver<br>Islet	GC	group-specific component (vitamin D binding protein)	4	-72826276	2638	4q12-q13	Hs.418497
4	33<br>44<br>5<br>54	FetalLung<br>Liver<br>Kidney<br>FetalLiver	ALDOB	aldolase B, fructose-bisphosphate	9	-103223479	229	9q21.3-q22.2	Hs.530274
4	33<br>42<br>48<br>76	FetalLung<br>Lung<br>Uterus<br>Adipocytes	LOC284064		17		284064	17q21.31	
4	30<br>37<br>56<br>7	Tongue<br>HBEC<br>Trachea<br>Skin	SERPINB5	serpin peptidase inhibitor, clade B (ovalbumin), member 5	18	59295198	5268	18q21.3	Hs.55279
4	3<br>44<br>47<br>78	LimbMuscle<br>Liver<br>Heart<br>FetalHeart	NDUFB7	NADH dehydrogenase (ubiquinone) 1 beta subcomplex, 7, 18kDa	19	-14537891	4713	19p13.12-p13.11	Hs.532853
4	3<br>4<br>5<br>78	LimbMuscle<br>ExtraocularMuscle<br>Kidney<br>FetalHeart	ACADM	acyl-Coenzyme A dehydrogenase, C-4 to C-12 straight chain	1	75962869	34	1p31	Hs.445040
4	3<br>4<br>48<br>78	LimbMuscle<br>ExtraocularMuscle<br>Uterus<br>FetalHeart	PLN	phospholamban	6	118976164	5350	6q22.1	Hs.170839
4	3<br>4<br>47<br>78	LimbMuscle<br>ExtraocularMuscle<br>Heart<br>FetalHeart	POPDC2	popeye domain containing 2	3	-120843597	64091	3q13.33	Hs.534383
4	3<br>4<br>47<br>78	LimbMuscle<br>ExtraocularMuscle<br>Heart<br>FetalHeart	HSPB7	heat shock 27kDa protein family, member 7 (cardiovascular)	1	-16213111	27129	1p36.23-p34.3	Hs.502612
4	3<br>4<br>47<br>78	LimbMuscle<br>ExtraocularMuscle<br>Heart<br>FetalHeart	MYH6	myosin, heavy polypeptide 6, cardiac muscle, alpha (cardiomyopathy, hypertrophic 1)	14	-22921038	4624	14q12	Hs.278432
4	3<br>4<br>47<br>78	LimbMuscle<br>ExtraocularMuscle<br>Heart<br>FetalHeart	PDE4DIP	phosphodiesterase 4D interacting protein (myomegalin)	1	-430827846	9659	1q12	Hs.568247<br>Hs.584841<br>Hs.613082<br>Hs.626094
4	3<br>4<br>47<br>78	LimbMuscle<br>ExtraocularMuscle<br>Heart<br>FetalHeart	FABP3	fatty acid binding protein 3, muscle and heart (mammary-derived growth inhibitor)	1	-31610686	2170	1p33-p32	Hs.584756
4	3<br>4<br>47<br>78	LimbMuscle<br>ExtraocularMuscle<br>Heart<br>FetalHeart	LOC653513		1		653513	1q21.1	Hs.632434

4	3	LimbMuscle	MYOZ2	myozenin 2	4	120276468	51778	4q26-q27	Hs.381047
	4	ExtraocularMuscle							
	47	Heart							
	78	FetalHeart							
4	3	LimbMuscle	CPT1B	carnitine palmitoyltransferase 1B (muscle)	22	-49354156	1375	22q13.33	Hs.439777
	4	ExtraocularMuscle							
	47	Heart							
	78	FetalHeart							
4	3	LimbMuscle	COX7A1	cytochrome c oxidase subunit VIIa polypeptide 1 (muscle)	19	-41333663	1346	19q13.1	Hs.421621
	4	ExtraocularMuscle							
	47	Heart							
	78	FetalHeart							
4	3	LimbMuscle	CMKOR1	chemokine orphan receptor 1	2	237143181	57007	2q37.3	Hs.471751
	4	ExtraocularMuscle							
	41	Thyroid							
	78	FetalHeart							
4	3	LimbMuscle	MYL3	myosin, light polypeptide 3, alkali, ventricular, skeletal, slow	3	-46874370	4634	3p21.3-p21.2	Hs.517939
	32	PsoasMuscle							
	47	Heart							
	78	FetalHeart							
4	3	LimbMuscle	PGAM2	phosphoglycerate mutase 2 (muscle)	7	-44068851	5224	7p13-p12	Hs.632642
	32	PsoasMuscle							
	4	ExtraocularMuscle							
	47	Heart							
4	3	LimbMuscle	MYH1	myosin, heavy polypeptide 1, skeletal muscle, adult	17	-10336353	4619	17p13.1	Hs.499745
	32	PsoasMuscle							
	4	ExtraocularMuscle							
	41	Thyroid							
4	3	LimbMuscle	MYBPC2	myosin binding protein C, fast type	19	55628003	4606	19q13.33	Hs.85937
	30	Tongue							
	32	PsoasMuscle							
	41	Thyroid							
4	3	LimbMuscle	CASQ1	calsequestrin 1 (fast-twitch, skeletal muscle)	1	158426988	844	1q21	Hs.632476
	30	Tongue							
	32	PsoasMuscle							
	4	ExtraocularMuscle							
4	3	LimbMuscle	TNNT1	troponin T type 1 (skeletal, slow)	19	-60336008	7138	19q13.4	Hs.631558
	30	Tongue							
	32	PsoasMuscle							
	4	ExtraocularMuscle							
4	26	Pons	IL6	interleukin 6 (interferon, beta 2)	7	22733344	3569	7p21	Hs.512234
	33	FetalLung							
	63	CardiacMyocytes							
	72	SmoothMuscle							
4	25	MedullaOblongata	CHN1	chimerin (chimaerin) 1	2	-175372337	1123	2q31-q32.1	Hs.380138
	51	PrefrontalCortex							
	6	SubthalamicNucleus							
	70	OccipitalLobe							
4	25	MedullaOblongata	ARPP-21		3	35696119, 35658852, 35697432	10777	3p22.3	Hs.475902
	26	Pons							
	73	CaudateNucleus							
	74	WholeBrain							
4	20	BM-CD33Myeloid	SOD2	superoxide dismutase 2, mitochondrial	6	-320042884	6648	6q25.3	Hs.487046
	36	WholeBlood							
	72	SmoothMuscle							
	75	Islet							

4	20	BM-CD33Myeloid	CXCL2	chemokine (C-X-C motif) ligand 2	4	-75181619	2920	4q21	Hs.590921
	33	FetalLung							
	72	SmoothMuscle							
	75	Islet							
4	20	BM-CD33Myeloid	FOSB	FBJ murine osteosarcoma viral oncogene homolog B	19	50663092	2354	19q13.32	Hs.590958
	21	BM-CD105Endothelial							
	22	BM-CD34							
	23	PB-BDCA4DentriticCells							
4	2	BronchialEpitelia	TFF3	trefoil factor 3 (intestinal)	21	-42605232	7033	21q22.3	Hs.82961
	41	Thyroid							
	56	Trachea							
	64	FetalThyroid							
4	2	BronchialEpitelia	CXCL1	chemokine (C-X-C motif) ligand 1 (melanoma growth stimulating activity, alpha)	4	74953972	2919	4q21	Hs.789
	33	FetalLung							
	63	CardiacMyocytes							
	72	SmoothMuscle							
4	2	BronchialEpitelia	SCGB1A1	secretoglobin, family 1A, member 1 (uteroglobin)	11	61943098	7356	11q12.3-q13.1	Hs.523732
	33	FetalLung							
	42	Lung							
	56	Trachea							
4	2	BronchialEpitelia	ALDH3A1	aldehyde dehydrogenase 3 family, memberA1	17	-19581891	218	17p11.2	Hs.531682
	30	Tongue							
	42	Lung							
	56	Trachea							
4	2	BronchialEpitelia	PROM1	prominin 1	4	-15578954	8842	4p15.32	Hs.479220
	22	BM-CD34							
	43	FetalBrain							
	56	Trachea							
4	19	PB-CD19BCells	RHOQ	ras homolog gene family, member Q	2	46623378	23433	2p21	Hs.643481
	3	LimbMuscle							
	4	ExtraocularMuscle							
	78	FetalHeart							
4	19	PB-CD19BCells	BANK1	B-cell scaffold protein with ankyrin repeats 1	4	102930918	55024	4q24	Hs.480400
	23	PB-BDCA4DentriticCells							
	35	Tonsil							
	45	LymphNode							
4	19	PB-CD19BCells	IFT57	intraflagellar transport 57 homolog (Chlamydomonas)	3	-109362349	55081	3q13.12	Hs.412196
	2	BronchialEpitelia							
	67	TestisIntersitial							
	77	TestiSeminiferousTubule							
4	17	PB-CD14Monocytes	FBP1	fructose-1,6-bisphosphatase 1	9	-96405237	2203	9q22.3	Hs.494496
	42	Lung							
	44	Liver							
	5	Kidney							
4	17	PB-CD14Monocytes	TGFBI	transforming growth factor, beta-induced, 68kDa	5	135392596	7045	5q31	Hs.369397
	23	PB-BDCA4DentriticCells							
	63	CardiacMyocytes							
	72	SmoothMuscle							
4	17	PB-CD14Monocytes	LR8		7	-150119323	28959	7q36.1	Hs.438823
	20	BM-CD33Myeloid							
	44	Liver							
	5	Kidney							
4	17	PB-CD14Monocytes	C5AR1	complement component 5a receptor 1	19	52504970	728	19q13.3-q13.4	Hs.2161
	20	BM-CD33Myeloid							
	36	WholeBlood							
	62	BoneMarrow							

4	17<br>20<br>36<br>62	PB-CD14Monocytes<br>BM-CD33Myeloid<br>WholeBlood<br>BoneMarrow	GCA	grancalcin, EF-hand calcium binding protein	2	162908895	25801	2q24.2	Hs.377894
4	17<br>20<br>36<br>62	PB-CD14Monocytes<br>BM-CD33Myeloid<br>WholeBlood<br>BoneMarrow	FLJ22662		12	-14547863	79887	12p13.1	Hs.131933
4	17<br>20<br>36<br>54	PB-CD14Monocytes<br>BM-CD33Myeloid<br>WholeBlood<br>FetalLiver	DPYD	dihydropyrimidine dehydrogenase	1	-97315889	1806	1p22	Hs.335034
4	17<br>20<br>36<br>5	PB-CD14Monocytes<br>BM-CD33Myeloid<br>WholeBlood<br>Kidney	TNFSF10	tumor necrosis factor (ligand) superfamily, member 10	3	-173706158	8743	3q26	Hs.478275
4	17<br>20<br>36<br>37	PB-CD14Monocytes<br>BM-CD33Myeloid<br>WholeBlood<br>HBEC	DUSP6	dual specificity phosphatase 6	12	-88265969	1848	12q22-q23	Hs.298654
4	17<br>20<br>23<br>5	PB-CD14Monocytes<br>BM-CD33Myeloid<br>PB-BDCA4DentriticCells<br>Kidney	LGALS2	lectin, galactoside-binding, soluble, 2 (galectin 2)	22	-36296199	3957	22q12-q13|22q13.1	Hs.531776
4	17<br>20<br>23<br>36	PB-CD14Monocytes<br>BM-CD33Myeloid<br>PB-BDCA4DentriticCells<br>WholeBlood	CD302	CD302 molecule	2	-160333609	9936	2q24.2	Hs.130014
4	17<br>20<br>23<br>36	PB-CD14Monocytes<br>BM-CD33Myeloid<br>PB-BDCA4DentriticCells<br>WholeBlood	RAB11FIP1	RAB11 family interacting protein 1 (class I)	8	-75672971	80223	8p11.22	Hs.191179
4	17<br>20<br>23<br>36	PB-CD14Monocytes<br>BM-CD33Myeloid<br>PB-BDCA4DentriticCells<br>WholeBlood	IGSF6	immunoglobulin superfamily, member 6	16	-21560106	10261	16p12-p13	Hs.530902
4	17<br>20<br>23<br>36	PB-CD14Monocytes<br>BM-CD33Myeloid<br>PB-BDCA4DentriticCells<br>WholeBlood	CLEC4A	C-type lectin domain family 4, member A	12	8167494	50856	12p13	Hs.504657
4	17<br>20<br>23<br>36	PB-CD14Monocytes<br>BM-CD33Myeloid<br>PB-BDCA4DentriticCells<br>WholeBlood	CYBB	cytochrome b-245, beta polypeptide (chronic granulomatous disease)	X	37524263	1536	Xp21.1	Hs.292356
4	17<br>20<br>23<br>36	PB-CD14Monocytes<br>BM-CD33Myeloid<br>PB-BDCA4DentriticCells<br>WholeBlood	LY96	lymphocyte antigen 96	8	75066143	23643	8q21.11	Hs.69328
4	17<br>20<br>23<br>36	PB-CD14Monocytes<br>BM-CD33Myeloid<br>PB-BDCA4DentriticCells<br>WholeBlood	PTPRE	protein tyrosine phosphatase, receptor type, E	10	129595314, 129735823	5791	10q26	Hs.127022
4	17<br>20<br>22<br>62	PB-CD14Monocytes<br>BM-CD33Myeloid<br>BM-CD34<br>BoneMarrow	RNASE2	ribonuclease, RNase A family, 2 (liver, eosinophil-derived neurotoxin)	14	20493469	6036	14q24-q31	Hs.728

4	17	PB-CD14Monocytes	FCER1A	Fc fragment of IgE, high affinity I,	1	157526127	2205	1q23	Hs.897
	20	BM-CD33Myeloid		receptor for, alpha polypeptide					
	22	BM-CD34							
	23	PB-BDCA4DentriticCells							
4	17	PB-CD14Monocytes	TFEC	transcription factor EC	7	-115362437	22797	7q31.2	Hs.125962
	20	BM-CD33Myeloid							
	22	BM-CD34							
	23	PB-BDCA4DentriticCells							
4	17	PB-CD14Monocytes	FCGR3B	Fc fragment of IgG, low affinity IIIb,	1	-159859676	2215	1q23	Hs.372679
	18	PB-CD56NKCells		receptor (CD16b)					
	36	WholeBlood							
	62	BoneMarrow							
4	17	PB-CD14Monocytes	FCGR3A	Fc fragment of IgG, low affinity IIIa,	1	-159778174	2214	1q23	Hs.372679
	18	PB-CD56NKCells		receptor (CD16a)					
	36	WholeBlood							
	62	BoneMarrow							
4	17	PB-CD14Monocytes	MYO1F	myosin IF	19	-8491998	4542	19p13.3-p13.2	Hs.465818
	18	PB-CD56NKCells							
	20	BM-CD33Myeloid							
	36	WholeBlood							
4	17	PB-CD14Monocytes	FGR	Gardner-Rasheed feline sarcoma viral	1	-27811397	2268	1p36.2-p36.1	Hs.1422
	18	PB-CD56NKCells		(v-fgr) oncogene homolog					
	20	BM-CD33Myeloid							
	36	WholeBlood							
4	17	PB-CD14Monocytes	AOAH	acyloxyacyl hydrolase (neutrophil)	7	-36519134	313	7p14-p12	Hs.488007
	18	PB-CD56NKCells							
	20	BM-CD33Myeloid							
	36	WholeBlood							
4	17	PB-CD14Monocytes	CENTB2	centaurin, beta 2	3	-196476767	23527	3q29	Hs.478746
	18	PB-CD56NKCells							
	20	BM-CD33Myeloid							
	36	WholeBlood							
4	17	PB-CD14Monocytes	IER5	immediate early response 5	1	179324260	51278	1q25.3	Hs.15725
	18	PB-CD56NKCells							
	19	PB-CD19BCells							
	20	BM-CD33Myeloid							
4	16	BM-CD71EarlyErythroid	ERAF	erythroid associated factor	16	31446703	51327	16p11.2	Hs.274309
	21	BM-CD105Endothelial							
	54	FetalLiver							
	62	BoneMarrow							
4	16	BM-CD71EarlyErythroid	RHCE	Rh blood group, CcEe antigens	1	-25561327	6006	1p36.11	Hs.449968
	21	BM-CD105Endothelial							Hs.643556
	54	FetalLiver							
	62	BoneMarrow							
4	16	BM-CD71EarlyErythroid	EPB42	erythrocyte membrane protein band 4.2	15	-41276719	2038	15q15-q21	Hs.368642
	21	BM-CD105Endothelial							
	54	FetalLiver							
	62	BoneMarrow							
4	16	BM-CD71EarlyErythroid	TMEM14B	transmembrane protein 14B	6	10856035	81853	6p25.1-p23	Hs.273077
	21	BM-CD105Endothelial							
	3	LimbMuscle							
	78	FetalHeart							
4	16	BM-CD71EarlyErythroid	KLF1	Kruppel-like factor 1 (erythroid)	19	-12856238	10661	19p13.13-p13.12	Hs.37860
	21	BM-CD105Endothelial							
	22	BM-CD34							
	62	BoneMarrow							

4	16	BM-CD71EarlyErythroid	HBD	hemoglobin, delta	11	-5210634	3045	11p15.5	Hs.591988
	21	BM-CD105Endothelial							
	22	BM-CD34							
	62	BoneMarrow							
4	16	BM-CD71EarlyErythroid	MINPP1	multiple inositol polyphosphate histidine phosphatase, 1	10	89254632	9562	10q23	Hs.121260
	21	BM-CD105Endothelial							
	22	BM-CD34							
	54	FetalLiver							
4	16	BM-CD71EarlyErythroid	XK	X-linked Kx blood group (McLeod syndrome)	X	37430051	7504	Xp21.1	Hs.78919
	21	BM-CD105Endothelial							
	22	BM-CD34							
	54	FetalLiver							
4	14	PB-CD8TCells	TRAC	T cell receptor alpha constant	14		28755	14q11	Hs.74647
	15	PB-CD4TCells							
	36	WholeBlood							
	53	Thymus							
4	14	PB-CD8TCells	CD247	CD247 molecule	1	-165666507	919	1q22-q23	Hs.156445
	15	PB-CD4TCells							
	18	PB-CD56NKCells							
	53	Thymus							
4	14	PB-CD8TCells	ITK	IL2-inducible T-cell kinase	5	156540484	3702	5q31-q32	Hs.558348
	15	PB-CD4TCells							
	18	PB-CD56NKCells							
	53	Thymus							
4	14	PB-CD8TCells	TRDC	T cell receptor delta constant	14		28526	14q11	
	15	PB-CD4TCells							
	18	PB-CD56NKCells							
	53	Thymus							
4	14	PB-CD8TCells	GZMA	granzyme A (granzyme 1, cytotoxic T-lymphocyte-associated serine esterase 3)	5	54434229	3001	5q11-q12	Hs.90708
	15	PB-CD4TCells							
	18	PB-CD56NKCells							
	36	WholeBlood							
4	14	PB-CD8TCells	IL18RAP	interleukin 18 receptor accessory protein	2	102401685	8807	2p24.3-p24.1	Hs.158315
	15	PB-CD4TCells							
	18	PB-CD56NKCells							
	36	WholeBlood							
4	14	PB-CD8TCells	NKG7	natural killer cell group 7 sequence	19	-56566686	4818	19q13.33	Hs.10306
	15	PB-CD4TCells							
	18	PB-CD56NKCells							
	36	WholeBlood							
4	14	PB-CD8TCells	PRF1	perforin 1 (pore forming protein)	10	-72027110	5551	10q22	Hs.2200
	15	PB-CD4TCells							
	18	PB-CD56NKCells							
	36	WholeBlood							
4	14	PB-CD8TCells	KLRB1	killer cell lectin-like receptor subfamily B, member 1	12	-9639137	3820	12p13	Hs.169824
	15	PB-CD4TCells							
	18	PB-CD56NKCells							
	36	WholeBlood							
4	14	PB-CD8TCells	GNLY	granulysin	2	85774924, 85775059	10578	2p12-q11	Hs.105806
	15	PB-CD4TCells							
	18	PB-CD56NKCells							
	36	WholeBlood							
4	14	PB-CD8TCells	IL32	interleukin 32	16	3055313, 3055663, 3055639, 3055612,	9235	16p13.3	Hs.943
	15	PB-CD4TCells							
	18	PB-CD56NKCells							
	3	LimbMuscle							

						3055785			
4	14	PB-CD8TCells	ARL4C	ADP-ribosylation factor-like 4C	2	-235066426	10123	2q37.1	Hs.111554
	15	PB-CD4TCells							
	18	PB-CD56NKCells							
	23	PB-BDCA4DentriticCells							
4	14	PB-CD8TCells	RARRES3	retinoic acid receptor responder (tazarotene induced) 3	11	63060855	5920	11q23	Hs.17466
	15	PB-CD4TCells							
	18	PB-CD56NKCells							
	2	BronchialEpitelia							
4	14	PB-CD8TCells	HECA	headcase homolog (Drosophila)	6	139497941	51696	6q23-q24	Hs.197644
	15	PB-CD4TCells							
	18	PB-CD56NKCells							
	19	PB-CD19BCells							
4	14	PB-CD8TCells	PTPRCAP	protein tyrosine phosphatase, receptor type, C-associated protein	11	-66959556	5790	11q13.3	Hs.155975
	15	PB-CD4TCells							
	18	PB-CD56NKCells							
	19	PB-CD19BCells							
4	13	721-BLymphoblasts	MS4A1	membrane-spanning 4-domains, subfamily A, member 1	11	59979857	931	11q12	Hs.438040
	19	PB-CD19BCells							
	35	Tonsil							
	45	LymphNode							
4	13	721-BLymphoblasts	CCNB1IP1	cyclin B1 interacting protein 1	14	-39698736	57820	14q11.2	Hs.107003
	19	PB-CD19BCells							
	22	BM-CD34							
	78	FetalHeart							
4	13	721-BLymphoblasts	IFI16	interferon, gamma-inducible protein 16	1	157246330	3428	1q22	Hs.380250
	18	PB-CD56NKCells							
	19	PB-CD19BCells							
	23	PB-BDCA4DentriticCells							
4	13	721-BLymphoblasts	SAMSN1	SAM domain, SH3 domain and nuclear localisation signals, 1	21	-14779419	64092	21q11	Hs.473341<br>Hs.570423
	17	PB-CD14Monocytes							
	20	BM-CD33Myeloid							
	36	WholeBlood							
4	13	721-BLymphoblasts	CPOX	coproporphyrinogen oxidase	3	-99780981	1371	3q12	Hs.476982
	16	BM-CD71EarlyErythroid							
	21	BM-CD105Endothelial							
	54	FetalLiver							
4	13	721-BLymphoblasts	NUDT22	nudix (nucleoside diphosphate linked moiety X)-type motif 22	11	63750337	84304	11q13.1	Hs.172847
	16	BM-CD71EarlyErythroid							
	21	BM-CD105Endothelial							
	22	BM-CD34							
4	13	721-BLymphoblasts	DTL	denticleless homolog (Drosophila)	1	210275753	51514		Hs.632496
	16	BM-CD71EarlyErythroid							
	21	BM-CD105Endothelial							
	22	BM-CD34							
4	13	721-BLymphoblasts	VRK1	vaccinia related kinase 1	14	96333436	7443	14q32	Hs.422662
	16	BM-CD71EarlyErythroid							
	21	BM-CD105Endothelial							
	22	BM-CD34							
4	13	721-BLymphoblasts	EZH2	enhancer of zeste homolog 2 (Drosophila)	7	-148135407	2146	7q35-q36	Hs.444082
	16	BM-CD71EarlyErythroid							
	21	BM-CD105Endothelial							
	22	BM-CD34							

4	13	721-BLymphoblasts	HCAP-G		4	17421622	64151	4p15.33	Hs.446201
	16	BM-CD71EarlyErythroid							Hs.567567
	21	BM-CD105Endothelial							
	22	BM-CD34							
4	13	721-BLymphoblasts	DENND2D	DENN/MADD domain containing 2D	1	-111531323	79961	1p13.3	Hs.557850
	14	PB-CD8TCells							
	15	PB-CD4TCells							
	18	PB-CD56NKCells							
4	12	Placenta	COL1A1	collagen, type I, alpha 1	17	-45616457	1277	17q21.33	Hs.172928
	63	CardiacMyocytes							
	72	SmoothMuscle							
	76	Adipocytes							
4	12	Placenta	GPNMB	glycoprotein (transmembrane) nmb	7	23252840	10457	7p15	Hs.190495
	45	LymphNode							
	76	Adipocytes							
	78	FetalHeart							
4	12	Placenta	LIPG	lipase, endothelial	18	45342424	9388	18q21.1	Hs.465102
	41	Thyroid							
	64	FetalThyroid							
	72	SmoothMuscle							
4	12	Placenta	IGFBP1	insulin-like growth factor binding protein 1	7	45894483	3484	7p13-p12	Hs.401316
	40	Ovary							
	44	Liver							
	54	FetalLiver							
4	12	Placenta	FDX1	ferredoxin 1	11	109805803	2230	11q22	Hs.744
	39	AdrenalCortex							
	41	Thyroid							
	57	AdrenalGland							
4	12	Placenta	TFPI2	tissue factor pathway inhibitor 2	7	-93353680	7980	7q22	Hs.438231
	37	HBEC							
	63	CardiacMyocytes							
	72	SmoothMuscle							
4	12	Placenta	PHLDA2	pleckstrin homology-like domain, family A, member 2	11	-2906078	7262	11p15.5	Hs.154036
	37	HBEC							
	49	Prostate							
	72	SmoothMuscle							
4	12	Placenta	SDC1	syndecan 1	2	-20264040	6382	2p24.1	Hs.224607
	37	HBEC							
	44	Liver							
	5	Kidney							
4	12	Placenta	GPC3	glypican 3	X	-132497443	2719	Xq26.1	Hs.567276
	33	FetalLung							
	54	FetalLiver							
	78	FetalHeart							
4	12	Placenta	TCF21	transcription factor 21	6	134251969	6943	6pter-qter	Hs.78061
	33	FetalLung							
	42	Lung							
	78	FetalHeart							
4	12	Placenta	SERPINB2	serpin peptidase inhibitor, clade B (ovalbumin), member 2	18	59705921	5055	18q21.3	Hs.514913
	20	BM-CD33Myeloid							
	35	Tonsil							
	37	HBEC							
4	12	Placenta	CSF3R	colony stimulating factor 3 receptor (granulocyte)	1	-36704231	1441	1p35-p34.3	Hs.524517
	17	PB-CD14Monocytes							
	20	BM-CD33Myeloid							
	36	WholeBlood							

4	12<br>16<br>54<br>62	Placenta<br>BM-CD71EarlyErythroid<br>FetalLiver<br>BoneMarrow	BPGM	2,3-bisphosphoglycerate mutase	7	133982094,<br>133982110	669	7q31-q34	Hs.198365
4	1<br>63<br>72<br>78	Hippocampus<br>CardiacMyocytes<br>SmoothMuscle<br>FetalHeart	PLK2	polo-like kinase 2 (Drosophila)	5	-57785568	10769	5q12.1-q13.2	Hs.398157<br>Hs.642858
4	1<br>51<br>70<br>71	Hippocampus<br>PrefrontalCortex<br>OccipitalLobe<br>CerebellumPeduncles	PSD3	pleckstrin and Sec7 domain containing 3	8	-18432348	23362	8pter-p23.3	Hs.434255<br>Hs.592598
4	1<br>46<br>70<br>74	Hippocampus<br>Amygdala<br>OccipitalLobe<br>WholeBrain	GABRA5	gamma-aminobutyric acid (GABA) A receptor, alpha 5	15	24663150	2558	15q11.2-q12	Hs.24969
4	1<br>46<br>59<br>74	Hippocampus<br>Amygdala<br>Cerebellum<br>WholeBrain	NRN1	neuritin 1	6	-5943233	51299	6p25.1	Hs.103291
4	1<br>46<br>51<br>74	Hippocampus<br>Amygdala<br>PrefrontalCortex<br>WholeBrain	NAP1L2	nucleosome assembly protein 1-like 2	X	-72348879	4674	Xq13	Hs.66180
4	1<br>46<br>51<br>74	Hippocampus<br>Amygdala<br>PrefrontalCortex<br>WholeBrain	RPIP8		17	39741489	10900	17q21.31	Hs.500197
4	1<br>43<br>51<br>74	Hippocampus<br>FetalBrain<br>PrefrontalCortex<br>WholeBrain	SATB2	SATB family member 2	2	-199842468	23314	2q33	Hs.516617
4	1<br>43<br>46<br>74	Hippocampus<br>FetalBrain<br>Amygdala<br>WholeBrain	WASF1	WAS protein family, member 1	6	-110527714	8936	6q21-q22	Hs.75850
4	1<br>43<br>46<br>74	Hippocampus<br>FetalBrain<br>Amygdala<br>WholeBrain	LY6H	lymphocyte antigen 6 complex, locus H	8	-144310706	4062	8q24.3	Hs.159590
4	1<br>43<br>46<br>74	Hippocampus<br>FetalBrain<br>Amygdala<br>WholeBrain	LPPR4		1	99502487	9890	1p21.2	Hs.13245
4	1<br>33<br>46<br>74	Hippocampus<br>FetalLung<br>Amygdala<br>WholeBrain	WIF1	WNT inhibitory factor 1	12	-63730673	11197	12q14.3	Hs.284122
4	1<br>20<br>46<br>51	Hippocampus<br>BM-CD33Myeloid<br>Amygdala<br>PrefrontalCortex	KLHL2	kelch-like 2, Mayven (Drosophila)	4	166348240	11275	4q21.2	Hs.388668
4	1<br>2<br>46<br>51	Hippocampus<br>BronchialEpitelia<br>Amygdala<br>PrefrontalCortex	MKL2	MKL/myocardin-like 2	16	14072696	57496	16p13.12	Hs.592047

5	55	Testis	PHF7	PHD finger protein 7	3	52419566	51533	3p21.1	Hs.372719
	66	TestisGermCell							
	67	TestisIntersitial							
	68	TestisLeydigCell							
	77	TestiSeminiferousTubule							
5	55	Testis	PRM2	protamine 2	16	-11276993	5620	16p13.2	Hs.2324
	66	TestisGermCell							
	67	TestisIntersitial							
	68	TestisLeydigCell							
	77	TestiSeminiferousTubule							
5	55	Testis	SPATA6	spermatogenesis associated 6	1	-48536864	54558	1p33	Hs.538103
	66	TestisGermCell							
	67	TestisIntersitial							
	68	TestisLeydigCell							
	77	TestiSeminiferousTubule							
5	55	Testis	UBQLN3	ubiquilin 3	11	-5485107	50613	11p15	Hs.189184
	66	TestisGermCell							
	67	TestisIntersitial							
	68	TestisLeydigCell							
	77	TestiSeminiferousTubule							
5	55	Testis	OAZ3	ornithine decarboxylase antizyme 3	1	150005754	51686	1q21.3	Hs.591461
	66	TestisGermCell							
	67	TestisIntersitial							
	68	TestisLeydigCell							
	77	TestiSeminiferousTubule							
5	55	Testis	CCT6B	chaperonin containing TCP1, subunit 6B (zeta 2)	17	-30279051	10693	17q12	Hs.73072
	66	TestisGermCell							
	67	TestisIntersitial							
	68	TestisLeydigCell							
	77	TestiSeminiferousTubule							
5	55	Testis	GAPDHS	glyceraldehyde-3-phosphate dehydrogenase, spermatogenic	19	40716203	26330	19q13.1	Hs.248017
	66	TestisGermCell							
	67	TestisIntersitial							
	68	TestisLeydigCell							
	77	TestiSeminiferousTubule							
5	55	Testis	LOC81691		16	20725333	81691	16p12.2	Hs.177926
	66	TestisGermCell							
	67	TestisIntersitial							
	68	TestisLeydigCell							
	77	TestiSeminiferousTubule							
5	55	Testis	TCFL5	transcription factor-like 5 (basic helix-loop-helix)	20	-60942912	10732	20q13.3-qter	Hs.642593
	66	TestisGermCell							
	67	TestisIntersitial							
	68	TestisLeydigCell							
	77	TestiSeminiferousTubule							
5	55	Testis	TPTE	transmembrane phosphatase with tensin homology	21	-9928613	7179	21p11	Hs.122986
	66	TestisGermCell							
	67	TestisIntersitial							
	68	TestisLeydigCell							
	77	TestiSeminiferousTubule							
5	55	Testis	CRISP2	cysteine-rich secretory protein 2	6	-49768030	7180	6p21-qter	Hs.2042
	66	TestisGermCell							
	67	TestisIntersitial							
	68	TestisLeydigCell							
	77	TestiSeminiferousTubule							

5	55<br>66<br>67<br>68<br>77	Testis<br>TestisGermCell<br>TestisIntersitial<br>TestisLeydigCell<br>TestiSeminiferousTubule	CABYR	calcium binding tyrosine-(Y)-phosphorylation regulated (fibrousheathin 2)	18	19972952, 19973445	26256	18q11.2	Hs.511983
5	55<br>66<br>67<br>68<br>77	Testis<br>TestisGermCell<br>TestisIntersitial<br>TestisLeydigCell<br>TestiSeminiferousTubule	PRND	prion protein 2 (dublet)	20	4650555	23627	20pter-p12	Hs.406696
5	55<br>66<br>67<br>68<br>77	Testis<br>TestisGermCell<br>TestisIntersitial<br>TestisLeydigCell<br>TestiSeminiferousTubule	GSG1	germ cell associated 1	12	-26256787	83445	12p13.1	Hs.240053
5	55<br>66<br>67<br>68<br>77	Testis<br>TestisGermCell<br>TestisIntersitial<br>TestisLeydigCell<br>TestiSeminiferousTubule	LDHC	lactate dehydrogenase C	11	18390428, 18390482	3948	11p15.5-p15.3	Hs.2795
5	55<br>66<br>67<br>68<br>77	Testis<br>TestisGermCell<br>TestisIntersitial<br>TestisLeydigCell<br>TestiSeminiferousTubule	ANKRD7	ankyrin repeat domain 7	7	117652194	56311	7q31	Hs.563443
5	55<br>66<br>67<br>68<br>77	Testis<br>TestisGermCell<br>TestisIntersitial<br>TestisLeydigCell<br>TestiSeminiferousTubule	ODF2	outer dense fiber of sperm tails 2	9	130258252, 130259107	4957	9q34.11	Hs.129055
5	55<br>66<br>67<br>68<br>77	Testis<br>TestisGermCell<br>TestisIntersitial<br>TestisLeydigCell<br>TestiSeminiferousTubule	ZPBP	zona pellucida binding protein	7	-49947584	11055	7p14.3	Hs.388841
5	55<br>66<br>67<br>68<br>77	Testis<br>TestisGermCell<br>TestisIntersitial<br>TestisLeydigCell<br>TestiSeminiferousTubule	ODF1	outer dense fiber of sperm tails 1	8	103633035	4956	8q22.3	Hs.159274
5	55<br>66<br>67<br>68<br>77	Testis<br>TestisGermCell<br>TestisIntersitial<br>TestisLeydigCell<br>TestiSeminiferousTubule	TCP11	t-complex 11 (mouse)	6	-35193828	6954	6p21.3-p21.2	Hs.435371
5	55<br>66<br>67<br>68<br>77	Testis<br>TestisGermCell<br>TestisIntersitial<br>TestisLeydigCell<br>TestiSeminiferousTubule	ROPN1B	ropporin, rhophilin associated protein 1B	3	127170717	152015	3q21.2	Hs.528203
5	55<br>66<br>67<br>68<br>77	Testis<br>TestisGermCell<br>TestisIntersitial<br>TestisLeydigCell<br>TestiSeminiferousTubule	TNP1	transition protein 1 (during histone to protamine replacement)	2	-217432428	7141	2q35-q36	Hs.3017

5	55	Testis	PRM1	protamine 1	16	-11282199	5619	16p13.2	Hs.2909
	66	TestisGermCell							
	67	TestisIntersitial							
	68	TestisLeydigCell							
	77	TestiSeminiferousTubule							
5	5	Kidney	CALB1	calbindin 1, 28kDa	8	-91140013	793	8q21.3-q22.1	Hs.65425
	59	Cerebellum							
	69	Hypothalamus							
	71	CerebellumPeduncles							
	73	CaudateNucleus							
5	42	Lung	CTGF	connective tissue growth factor	6	-132311011	1490	6q23.1	Hs.591346
	48	Uterus							
	56	Trachea							
	63	CardiacMyocytes							
	72	SmoothMuscle							
5	42	Lung	NNMT	nicotinamide N-methyltransferase	11	113671744	4837	11q23.1	Hs.503911
	44	Liver							
	63	CardiacMyocytes							
	72	SmoothMuscle							
	76	Adipocytes							
5	41	Thyroid	DIO1	deiodinase, iodothyronine, type I	1	54132448	1733	1p33-p32	Hs.251415
	44	Liver							
	5	Kidney							
	54	FetalLiver							
	64	FetalThyroid							
5	4	ExtraocularMuscle	PVALB	parvalbumin	22	-35526691	5816	22q12-q13.1|22q13.1	Hs.295449
	52	CingulateCortex							
	59	Cerebellum							
	61	Thalamus							
	71	CerebellumPeduncles							
5	38	SalivaryGland	OLFM4	olfactomedin 4	13	52500972	10562	13q21.1	Hs.559736
	49	Prostate							
	50	Pancreas							
	62	BoneMarrow							
	75	Islet							
5	38	SalivaryGland	HTN3	histatin 3	4	70928764	3347	4q13	Hs.250959
	41	Thyroid							
	49	Prostate							
	56	Trachea							
	61	Thalamus							
5	38	SalivaryGland	HTN1	histatin 1	4	70950747	3346	4q13	Hs.250959
	41	Thyroid							
	49	Prostate							
	56	Trachea							
	61	Thalamus							
5	38	SalivaryGland	STATH	statherin	4	70896236	6779	4q11-q13	Hs.250959
	41	Thyroid							
	49	Prostate							
	56	Trachea							
	61	Thalamus							
5	33	FetalLung	ALB	albumin	4	74488869	213	4q11-q13	Hs.418167
	44	Liver							
	5	Kidney							
	50	Pancreas							
	54	FetalLiver							

5	33	FetalLung	ORM1	orosomucoid 1	9	116125156	5004	9q31-q32	Hs.567311
	44	Liver							
	49	Prostate							
	54	FetalLiver							
	62	BoneMarrow							
5	3	LimbMuscle	PHYH	phytanoyl-CoA 2-hydroxylase	10	-13359802	5264	10pter-p11.2	Hs.498732
	4	ExtraocularMuscle							
	5	Kidney							
	54	FetalLiver							
	78	FetalHeart							
5	3	LimbMuscle	GBA2	glucosidase, beta (bile acid) 2	9	-35726863	57704	9p13.3	Hs.443134
	4	ExtraocularMuscle							
	48	Uterus							
	72	SmoothMuscle							
	78	FetalHeart							
5	3	LimbMuscle	CRYAB	crystallin, alpha B	11	-111284559	1410	11q22.3-q23.1	Hs.408767
	4	ExtraocularMuscle							
	47	Heart							
	65	OlfactoryBulb							
	78	FetalHeart							
5	3	LimbMuscle	G0S2	G0/G1switch 2	1	207915387	50486	1q32.2-q41	Hs.432132
	33	FetalLung							
	37	HBEC							
	62	BoneMarrow							
	76	Adipocytes							
5	3	LimbMuscle	CKMT2	creatine kinase, mitochondrial 2 (sarcomeric)	5	80564937	1160	5q13.3	Hs.80691
	32	PsoasMuscle							
	4	ExtraocularMuscle							
	47	Heart							
	78	FetalHeart							
5	3	LimbMuscle	RPL3L	ribosomal protein L3-like	16	-1934581	6123	16p13.3	Hs.498569
	32	PsoasMuscle							
	4	ExtraocularMuscle							
	47	Heart							
	78	FetalHeart							
5	3	LimbMuscle	SYNPO2L	synaptopodin 2-like	10	-75075295	79933	10q22.2	Hs.533659
	32	PsoasMuscle							
	4	ExtraocularMuscle							
	47	Heart							
	78	FetalHeart							
5	3	LimbMuscle	SMPX	small muscle protein, X-linked	X	-21634012	23676	Xp22.1	Hs.86492
	30	Tongue							
	4	ExtraocularMuscle							
	47	Heart							
	78	FetalHeart							
5	3	LimbMuscle	TNNI1	troponin I type 1 (skeletal, slow)	1	-199639518	7135	1q31.3	Hs.320890
	30	Tongue							
	32	PsoasMuscle							
	4	ExtraocularMuscle							
	78	FetalHeart							
5	3	LimbMuscle	TNNT3	troponin T type 3 (skeletal, fast)	11	1900668	7140	11p15.5	Hs.73454
	30	Tongue							
	32	PsoasMuscle							
	4	ExtraocularMuscle							
	78	FetalHeart							

5	3	LimbMuscle	PDLIM3	PDZ and LIM domain 3	4	-186659904	27295	4q35	Hs.85862
	30	Tongue							
	32	PsoasMuscle							
	4	ExtraocularMuscle							
	48	Uterus							
5	3	LimbMuscle	TCAP	titin-cap (telethonin)	17	35075124	8557	17q12	Hs.514146
	30	Tongue							
	32	PsoasMuscle							
	4	ExtraocularMuscle							
	47	Heart							
5	3	LimbMuscle	MYOT	myotilin	5	137231472	9499	5q31	Hs.84665
	30	Tongue							
	32	PsoasMuscle							
	4	ExtraocularMuscle							
	41	Thyroid							
5	3	LimbMuscle	TNNI2	troponin I type 2 (skeletal, fast)	11	1817478	7136	11p15.5	Hs.523403
	30	Tongue							
	32	PsoasMuscle							
	4	ExtraocularMuscle							
	41	Thyroid							
5	3	LimbMuscle	ATP2A1	ATPase, Ca++ transporting, cardiac muscle, fast twitch 1	16	28797309	487	16p12.1	Hs.183075
	30	Tongue							
	32	PsoasMuscle							
	4	ExtraocularMuscle							
	41	Thyroid							
5	3	LimbMuscle	MYH2	myosin, heavy polypeptide 2, skeletal muscle, adult	17	-10365193	4620	17p13.1	Hs.499745
	30	Tongue							
	32	PsoasMuscle							
	4	ExtraocularMuscle							
	41	Thyroid							
5	3	LimbMuscle	TNNC2	troponin C type 2 (fast)	20	-43885262	7125	20q12-q13.11	Hs.182421
	30	Tongue							
	32	PsoasMuscle							
	4	ExtraocularMuscle							
	41	Thyroid							
5	3	LimbMuscle	KBTBD10	kelch repeat and BTB (POZ) domain containing 10	2	170074457	10324	2q31.1	Hs.50550
	30	Tongue							
	32	PsoasMuscle							
	4	ExtraocularMuscle							
	41	Thyroid							
5	3	LimbMuscle	MYBPC1	myosin binding protein C, slow type	12	100512877	4604	12q23.2	Hs.485903
	30	Tongue							
	32	PsoasMuscle							
	4	ExtraocularMuscle							
	41	Thyroid							
5	21	BM-CD105Endothelial	CETN3	centrin, EF-hand protein, 3 (CDC31 homolog, yeast)	5	-89725286	1070	5q14.3	Hs.591767
	66	TestisGermCell							
	67	TestisIntersitial							
	77	TestiSeminiferousTubule							
	78	FetalHeart							
5	21	BM-CD105Endothelial	VCAM1	vascular cell adhesion molecule 1	1	100957884	7412	1p32-p31	Hs.109225
	35	Tonsil							
	45	LymphNode							
	5	Kidney							
	54	FetalLiver							

5	20	BM-CD33Myeloid	IL8	interleukin 8	4	74825138	3576	4q13-q21	Hs.624
	23	PB-BDCA4DentriticCells							
	33	FetalLung							
	63	CardiacMyocytes							
	72	SmoothMuscle							
5	2	BronchialEpitelia	MNS1	meiosis-specific nuclear structural 1	15	-54508298	55329	15q21.3	Hs.444483
	66	TestisGermCell							
	67	TestisIntersitial							
	68	TestisLeydigCell							
	77	TestiSeminiferousTubule							
5	2	BronchialEpitelia	WFDC2	WAP four-disulfide core domain 2	20	43531807, 43541898	10406	20q12-q13.2	Hs.2719
	38	SalivaryGland							
	41	Thyroid							
	42	Lung							
	56	Trachea							
5	2	BronchialEpitelia	AGR2	anterior gradient 2 homolog (Xenopus laevis)	7	-16797960	10551	7p21.3	Hs.530009
	33	FetalLung							
	37	HBEC							
	49	Prostate							
	56	Trachea							
5	2	BronchialEpitelia	RRAD	Ras-related associated with diabetes	16	-65513089	6236	16q22	Hs.1027
	3	LimbMuscle							
	32	PsoasMuscle							
	47	Heart							
	78	FetalHeart							
5	19	PB-CD19BCells	IGHM	immunoglobulin heavy constant mu	14		3507	14q32.33	Hs.510635
	23	PB-BDCA4DentriticCells							
	35	Tonsil							
	45	LymphNode							
	47	Heart							
5	18	PB-CD56NKCells	MYOM2	myomesin (M-protein) 2, 165kDa	8	1980654	9172	8p23.3	Hs.443683
	3	LimbMuscle							
	32	PsoasMuscle							
	47	Heart							
	78	FetalHeart							
5	17	PB-CD14Monocytes	PF4	platelet factor 4 (chemokine (C-X-C motif) ligand 4)	4	-75065659	5196	4q12-q21	Hs.81564
	22	BM-CD34							
	36	WholeBlood							
	54	FetalLiver							
	62	BoneMarrow							
5	17	PB-CD14Monocytes	PTGS2	prostaglandin-endoperoxide synthase 2 (prostaglandin G/H synthase and cyclooxygenase)	1	-184907592	5743	1q25.2-q25.3	Hs.196384
	20	BM-CD33Myeloid							
	37	HBEC							
	72	SmoothMuscle							
	75	Islet							
5	17	PB-CD14Monocytes	GLIPR1	GLI pathogenesis-related 1 (glioma)	12	74160779	11010	12q21.2	Hs.205558 Hs.591034
	20	BM-CD33Myeloid							
	23	PB-BDCA4DentriticCells							
	36	WholeBlood							
	72	SmoothMuscle							
5	17	PB-CD14Monocytes	S100A12	S100 calcium binding protein A12 (calgranulin C)	1	-151612807	6283	1q21	Hs.19413
	20	BM-CD33Myeloid							
	23	PB-BDCA4DentriticCells							
	36	WholeBlood							
	62	BoneMarrow							

5	17<br>20<br>23<br>36<br>62	PB-CD14Monocytes<br>BM-CD33Myeloid<br>PB-BDCA4DentriticCells<br>WholeBlood<br>BoneMarrow	NCF2	neutrophil cytosolic factor 2 (65kDa, chronic granulomatous disease, autosomal 2)	1	-181791320	4688	1q25	Hs.587558
5	17<br>20<br>23<br>36<br>53	PB-CD14Monocytes<br>BM-CD33Myeloid<br>PB-BDCA4DentriticCells<br>WholeBlood<br>Thymus	CD1D	CD1d molecule	1	156416360	912	1q22-q23	Hs.1799<br>Hs.387539
5	17<br>2<br>20<br>36<br>5	PB-CD14Monocytes<br>BronchialEpitelia<br>BM-CD33Myeloid<br>WholeBlood<br>Kidney	FLJ20273		4	-40120497	54502	4p13-p12	Hs.518727
5	17<br>19<br>20<br>36<br>42	PB-CD14Monocytes<br>PB-CD19BCells<br>BM-CD33Myeloid<br>WholeBlood<br>Lung	ALOX5	arachidonate 5-lipoxygenase	10	45189634	240	10q11.2	Hs.89499
5	17<br>19<br>20<br>23<br>36	PB-CD14Monocytes<br>PB-CD19BCells<br>BM-CD33Myeloid<br>PB-BDCA4DentriticCells<br>WholeBlood	RNASE6	ribonuclease, RNase A family, k6	14	20319049	6039	14q11.2	Hs.23262
5	17<br>19<br>20<br>22<br>23	PB-CD14Monocytes<br>PB-CD19BCells<br>BM-CD33Myeloid<br>BM-CD34<br>PB-BDCA4DentriticCells	SYK	spleen tyrosine kinase	9	92603890	6850	9q22	Hs.371720
5	17<br>18<br>20<br>23<br>36	PB-CD14Monocytes<br>PB-CD56NKCells<br>BM-CD33Myeloid<br>PB-BDCA4DentriticCells<br>WholeBlood	C1orf38	chromosome 1 open reading frame 38	1	28071641	9473	1p35.3	Hs.10649
5	17<br>18<br>20<br>23<br>36	PB-CD14Monocytes<br>PB-CD56NKCells<br>BM-CD33Myeloid<br>PB-BDCA4DentriticCells<br>WholeBlood	TNFRSF1B	tumor necrosis factor receptor superfamily, member 1B	1	12149646	7133	1p36.3-p36.2	Hs.256278
5	17<br>18<br>20<br>23<br>36	PB-CD14Monocytes<br>PB-CD56NKCells<br>BM-CD33Myeloid<br>PB-BDCA4DentriticCells<br>WholeBlood	HCK	hemopoietic cell kinase	20	30103717	3055	20q11-q12	Hs.126521
5	17<br>18<br>20<br>23<br>36	PB-CD14Monocytes<br>PB-CD56NKCells<br>BM-CD33Myeloid<br>PB-BDCA4DentriticCells<br>WholeBlood	CASP1	caspase 1, apoptosis-related cysteine peptidase (interleukin 1, beta, convertase)	11	-104401451	834	11q23	Hs.2490
5	17<br>18<br>20<br>23<br>36	PB-CD14Monocytes<br>PB-CD56NKCells<br>BM-CD33Myeloid<br>PB-BDCA4DentriticCells<br>WholeBlood	RGS2	regulator of G-protein signalling 2, 24kDa	1	191044793	5997	1q31	Hs.78944

5	17	PB-CD14Monocytes	FGL2	fibrinogen-like 2	7	-76660625	10875	7q11.23	Hs.520989
	18	PB-CD56NKCells							
	20	BM-CD33Myeloid							
	23	PB-BDCA4DentriticCells							
	36	WholeBlood							
5	17	PB-CD14Monocytes	CTSS	cathepsin S	1	-148969177	1520	1q21	Hs.181301
	18	PB-CD56NKCells							
	20	BM-CD33Myeloid							
	23	PB-BDCA4DentriticCells							
	36	WholeBlood							
5	16	BM-CD71EarlyErythroid	CA2	carbonic anhydrase II	8	86563497	760	8q22	Hs.155097
	21	BM-CD105Endothelial							
	5	Kidney							
	62	BoneMarrow							
	69	Hypothalamus							
5	16	BM-CD71EarlyErythroid	MYL4	myosin, light polypeptide 4, alkali, atrial, embryonic	17	42641426, 42641712	4635	17q21-qter	Hs.463300
	21	BM-CD105Endothelial							
	4	ExtraocularMuscle							
	47	Heart							
	78	FetalHeart							
5	16	BM-CD71EarlyErythroid	GYPB	glycophorin B (MNS blood group)	4	-145136706	2994	4q28-q31	Hs.632594
	21	BM-CD105Endothelial							
	33	FetalLung							
	54	FetalLiver							
	62	BoneMarrow							
5	16	BM-CD71EarlyErythroid	ALAS2	aminolevulinate, delta-, synthase 2 (sideroblastic/hypochromic anemia)	X	-55052213	212	Xp11.21	Hs.522666
	21	BM-CD105Endothelial							
	33	FetalLung							
	54	FetalLiver							
	62	BoneMarrow							
5	16	BM-CD71EarlyErythroid	PRKAR2B	protein kinase, cAMP-dependent, regulatory, type II, beta	7	106472413	5577	7q22	Hs.433068
	21	BM-CD105Endothelial							
	22	BM-CD34							
	43	FetalBrain							
	76	Adipocytes							
5	16	BM-CD71EarlyErythroid	LMO2	LIM domain only 2 (rhombotin-like 1)	11	-33836698	4005	11p13	Hs.34560
	17	PB-CD14Monocytes							
	20	BM-CD33Myeloid							
	21	BM-CD105Endothelial							
	22	BM-CD34							
5	15	PB-CD4TCells	FAM117A	family with sequence similarity 117, member A	17	-45142687	81558	17q21.33	Hs.514308
	16	BM-CD71EarlyErythroid							
	18	PB-CD56NKCells							
	21	BM-CD105Endothelial							
	22	BM-CD34							
5	14	PB-CD8TCells	CX3CR1	chemokine (C-X3-C motif) receptor 1	3	-39279989	1524	3p21|3p21.3	Hs.78913
	17	PB-CD14Monocytes							
	18	PB-CD56NKCells							
	20	BM-CD33Myeloid							
	36	WholeBlood							
5	14	PB-CD8TCells	CD3D	CD3d molecule, delta (CD3-TCR complex)	11	-117714998	915	11q23	Hs.504048
	15	PB-CD4TCells							
	36	WholeBlood							
	45	LymphNode							
	53	Thymus							

5	14<br>15<br>19<br>23<br>36	PB-CD8TCells<br>PB-CD4TCells<br>PB-CD19BCells<br>PB-BDCA4DentriticCells<br>WholeBlood	LTB	lymphotoxin beta (TNF superfamily, member 3)	6	-37446700	4050	6p21.3	Hs.376208
5	14<br>15<br>18<br>36<br>45	PB-CD8TCells<br>PB-CD4TCells<br>PB-CD56NKCells<br>WholeBlood<br>LymphNode	CCL5	chemokine (C-C motif) ligand 5	17	-31222609	6352	17q11.2-q12	Hs.514821
5	14<br>15<br>18<br>20<br>36	PB-CD8TCells<br>PB-CD4TCells<br>PB-CD56NKCells<br>BM-CD33Myeloid<br>WholeBlood	LCP2	lymphocyte cytosolic protein 2 (SH2 domain containing leukocyte protein of 76kDa)	5	-169607666	3937	5q33.1-qter	Hs.304475
5	14<br>15<br>18<br>19<br>36	PB-CD8TCells<br>PB-CD4TCells<br>PB-CD56NKCells<br>PB-CD19BCells<br>WholeBlood	RASGRP1	RAS guanyl releasing protein 1 (calcium and DAG-regulated)	15	-36567597	10125	15q15	Hs.591127
5	14<br>15<br>18<br>19<br>23	PB-CD8TCells<br>PB-CD4TCells<br>PB-CD56NKCells<br>PB-CD19BCells<br>PB-BDCA4DentriticCells	TRIM22	tripartite motif-containing 22	11	5667663	10346	11p15	Hs.501778<br>Hs.621411
5	14<br>15<br>17<br>18<br>36	PB-CD8TCells<br>PB-CD4TCells<br>PB-CD14Monocytes<br>PB-CD56NKCells<br>WholeBlood	GIMAP6	GTPase, IMAP family member 6	7	-149953398	474344		Hs.438823
5	14<br>15<br>17<br>18<br>36	PB-CD8TCells<br>PB-CD4TCells<br>PB-CD14Monocytes<br>PB-CD56NKCells<br>WholeBlood	STK10	serine/threonine kinase 10	5	-171403229	6793	5q35.1	Hs.519756
5	14<br>15<br>17<br>18<br>20	PB-CD8TCells<br>PB-CD4TCells<br>PB-CD14Monocytes<br>PB-CD56NKCells<br>BM-CD33Myeloid	PTGER2	prostaglandin E receptor 2 (subtype EP2), 53kDa	14	51850862	5732	14q22	Hs.2090
5	13<br>43<br>59<br>71<br>78	721-BLymphoblasts<br>FetalBrain<br>Cerebellum<br>CerebellumPeduncles<br>FetalHeart	C5orf13	chromosome 5 open reading frame 13	5	-111092891	9315	5q22.1	Hs.36053<br>Hs.626968
5	13<br>33<br>38<br>5<br>75	721-BLymphoblasts<br>FetalLung<br>SalivaryGland<br>Kidney<br>Islet	MMP7	matrix metallopeptidase 7 (matrilysin, uterine)	11	-101896449	4316	11q21-q22	Hs.2256
5	13<br>19<br>23<br>35<br>45	721-BLymphoblasts<br>PB-CD19BCells<br>PB-BDCA4DentriticCells<br>Tonsil<br>LymphNode	POU2AF1	POU domain, class 2, associating factor 1	11	-110728189	5450	11q23.1	Hs.128180

5	13	721-BLymphoblasts	BCL2A1	BCL2-related protein A1	15	-78040289	597	15q24.3	Hs.227817
	17	PB-CD14Monocytes							
	18	PB-CD56NKCells							
	20	BM-CD33Myeloid							
	36	WholeBlood							
5	13	721-BLymphoblasts	HAT1	histone acetyltransferase 1	2	172487203	8520	2q31.2-q33.1	Hs.632532
	16	BM-CD71EarlyErythroid							
	21	BM-CD105Endothelial							
	22	BM-CD34							
	78	FetalHeart							
5	13	721-BLymphoblasts	RACGAP1	Rac GTPase activating protein 1	12	-48669214	29127	12q13.13	Hs.505469
	16	BM-CD71EarlyErythroid							
	21	BM-CD105Endothelial							
	22	BM-CD34							
	55	Testis							
5	13	721-BLymphoblasts	ZWINT	ZW10 interactor	10	-57787204	11130	10q21-q22	Hs.591363
	16	BM-CD71EarlyErythroid							
	21	BM-CD105Endothelial							
	22	BM-CD34							
	53	Thymus							
5	13	721-BLymphoblasts	TYMS	thymidylate synthetase	18	647650	7298	18p11.32	Hs.592338
	16	BM-CD71EarlyErythroid							
	21	BM-CD105Endothelial							
	22	BM-CD34							
	53	Thymus							
5	13	721-BLymphoblasts	PCNA	proliferating cell nuclear antigen	20	-5043599	5111	20pter-p12	Hs.147433
	16	BM-CD71EarlyErythroid							
	21	BM-CD105Endothelial							
	22	BM-CD34							
	53	Thymus							
5	13	721-BLymphoblasts	NUSAP1	nucleolar and spindle associated protein 1	15	39412360	51203	15q15.1	Hs.615092
	16	BM-CD71EarlyErythroid							
	21	BM-CD105Endothelial							
	22	BM-CD34							
	53	Thymus							
5	13	721-BLymphoblasts	RP6-213H19.1		X	130985316, 130984925	51765	Xq26.2	Hs.444247
	14	PB-CD8TCells							
	18	PB-CD56NKCells							
	22	BM-CD34							
	48	Uterus							
5	13	721-BLymphoblasts	CCR7	chemokine (C-C motif) receptor 7	17	-35963549	1236	17q12-q21.2	Hs.370036
	14	PB-CD8TCells							
	15	PB-CD4TCells							
	19	PB-CD19BCells							
	53	Thymus							
5	13	721-BLymphoblasts	LCK	lymphocyte-specific protein tyrosine kinase	1	32489518	3932	1p34.3	Hs.470627
	14	PB-CD8TCells							
	15	PB-CD4TCells							
	18	PB-CD56NKCells							
	53	Thymus							
5	13	721-BLymphoblasts	RUNX3	runt-related transcription factor 3	1	-25098589	864	1p36	Hs.170019
	14	PB-CD8TCells							
	15	PB-CD4TCells							
	18	PB-CD56NKCells							
	19	PB-CD19BCells							

5	12	Placenta	LOXL1	lysyl oxidase-like 1	15	72005841	4016	15q24-q25|15q22	Hs.65436
	42	Lung							
	63	CardiacMyocytes							
	72	SmoothMuscle							
	76	Adipocytes							
5	12	Placenta	TBX3	T-box 3 (ulnar mammary syndrome)	12	-113592441	6926	12q24.1	Hs.129895
	39	AdrenalCortex							
	41	Thyroid							
	49	Prostate							
	57	AdrenalGland							
5	12	Placenta	GNG11	guanine nucleotide binding protein (G protein), gamma 11	7	93388951	2791	7q21	Hs.83381
	36	WholeBlood							
	63	CardiacMyocytes							
	72	SmoothMuscle							
	78	FetalHeart							
5	12	Placenta	KRT6C	keratin 6C	12	-51167243	140446	12q12-q13	Hs.433845
	30	Tongue							
	35	Tonsil							
	37	HBEC							
	56	Trachea							
5	12	Placenta	KRT6A	keratin 6A	12	-51167243	3853	12q12-q13	Hs.433845
	30	Tongue							
	35	Tonsil							
	37	HBEC							
	56	Trachea							
5	12	Placenta	FBN2	fibrillin 2 (congenital contractural arachnodactyly)	5	-127621500	2201	5q23-q31	Hs.519294
	20	BM-CD33Myeloid							
	37	HBEC							
	76	Adipocytes							
	78	FetalHeart							
5	1	Hippocampus	DKK3	dickkopf homolog 3 (Xenopus laevis)	11	-11941120	27122	11p15.2	Hs.292156
	46	Amygdala							
	51	PrefrontalCortex							
	73	CaudateNucleus							
	74	WholeBrain							
5	1	Hippocampus	BTBD3	BTB (POZ) domain containing 3	20	11846564, 11819476	22903	20p12.2	Hs.244590
	46	Amygdala							
	51	PrefrontalCortex							
	71	CerebellumPeduncles							
	74	WholeBrain							
5	1	Hippocampus	RGS7	regulator of G-protein signalling 7	1	-239005493	6000	1q43|1q23.1 according to Sierra (Genomics 79 177 2002) [AFS]	Hs.130171
	46	Amygdala							
	51	PrefrontalCortex							
	70	OccipitalLobe							
	74	WholeBrain							
5	1	Hippocampus	GDF1	growth differentiation factor 1	19	-18840360	2657	19p12	Hs.412355
	46	Amygdala							
	51	PrefrontalCortex							
	58	SpinalCord							
	69	Hypothalamus							
5	1	Hippocampus	GABRB1	gamma-aminobutyric acid (GABA) A receptor, beta 1	4	46728335	2560	4p12	Hs.27283
	46	Amygdala							
	51	PrefrontalCortex							
	52	CingulateCortex							
	70	OccipitalLobe							

5	1	Hippocampus	FABP7	fatty acid binding protein 7, brain	6	123142344	2173	6q22-q23	Hs.26770
	43	FetalBrain							
	46	Amygdala							
	59	Cerebellum							
	71	CerebellumPeduncles							
5	1	Hippocampus	WDR47	WD repeat domain 47	1	-109314359	22911	1p13.3	Hs.485081
	43	FetalBrain							
	46	Amygdala							
	51	PrefrontalCortex							
	70	OccipitalLobe							
5	1	Hippocampus	GARNL1	GTPase activating Rap/RanGAP domain-like 1	14	-35077309	253959	14q13.2	Hs.113150 Hs.136901
	43	FetalBrain							
	46	Amygdala							
	51	PrefrontalCortex							
	69	Hypothalamus							
6	58	SpinalCord	ZIC1	Zic family member 1 (odd-paired homolog, Drosophila)	3	148609870	7545	3q24	Hs.535724 Hs.598590
	59	Cerebellum							
	61	Thalamus							
	65	OlfactoryBulb							
	69	Hypothalamus							
	71	CerebellumPeduncles							
6	42	Lung	SRPX	sushi-repeat-containing protein, X-linked	X	-37893537	8406	Xp21.1	Hs.15154
	63	CardiacMyocytes							
	64	FetalThyroid							
	72	SmoothMuscle							
	76	Adipocytes							
	78	FetalHeart							
6	42	Lung	GATA6	GATA binding protein 6	18	18003413	2627	18q11.1-q11.2	Hs.514746
	47	Heart							
	57	AdrenalGland							
	63	CardiacMyocytes							
	72	SmoothMuscle							
	78	FetalHeart							
6	33	FetalLung	MT1H	metallothionein 1H	16	55261274	4496	16q13	Hs.438462
	41	Thyroid							
	44	Liver							
	5	Kidney							
	50	Pancreas							
	54	FetalLiver							
6	33	FetalLung	PLS3	plastin 3 (T isoform)	X	114701764	5358	Xq23	Hs.496622
	37	HBEC							
	41	Thyroid							
	48	Uterus							
	5	Kidney							
	72	SmoothMuscle							
6	3	LimbMuscle	SAMD4A	sterile alpha motif domain containing 4A	14	54104386	23034	14q22.2	Hs.98259
	55	Testis							
	66	TestisGermCell							
	67	TestisIntersitial							
	68	TestisLeydigCell							
	77	TestiSeminiferousTubule							
6	3	LimbMuscle	PPP1R1A	protein phosphatase 1, regulatory (inhibitor) subunit 1A	12	-53259299	5502	12q13.2	Hs.505662
	32	PsoasMuscle							
	4	ExtraocularMuscle							
	44	Liver							
	47	Heart							

	78	FetalHeart							
6	3	LimbMuscle	CSRP3	cysteine and glycine-rich protein 3 (cardiac LIM protein)	11	-19160153	8048	11p15.1	Hs.83577
	30	Tongue							
	32	PsoasMuscle							
	4	ExtraocularMuscle							
	47	Heart							
	78	FetalHeart							
6	3	LimbMuscle	COX6A2	cytochrome c oxidase subunit VIa polypeptide 2	16	-31346555	1339	16p	Hs.250760
	30	Tongue							
	32	PsoasMuscle							
	4	ExtraocularMuscle							
	47	Heart							
	78	FetalHeart							
6	3	LimbMuscle	NEB	nebulin	2	-152050100	4703	2q22	Hs.134602 Hs.588655
	30	Tongue							
	32	PsoasMuscle							
	4	ExtraocularMuscle							
	41	Thyroid							
	64	FetalThyroid							
6	3	LimbMuscle	MYL1	myosin, light polypeptide 1, alkali, skeletal, fast	2	-210863121	4632	2q33-q34	Hs.187338
	30	Tongue							
	32	PsoasMuscle							
	4	ExtraocularMuscle							
	41	Thyroid							
	64	FetalThyroid							
6	22	BM-CD34	SPINK2	serine peptidase inhibitor, Kazal type 2 (acrosin-trypsin inhibitor)	4	-57370790	6691	4q12	Hs.98243
	55	Testis							
	66	TestisGermCell							
	67	TestisIntersitial							
	68	TestisLeydigCell							
	77	TestiSeminiferousTubule							
6	21	BM-CD105Endothelial	PRPS2	phosphoribosyl pyrophosphate synthetase 2	X	12719413	5634	Xp22.3-p22.2	Hs.104123
	22	BM-CD34							
	40	Ovary							
	66	TestisGermCell							
	67	TestisIntersitial							
	77	TestiSeminiferousTubule							
6	2	BronchialEpitelia	LCN2	lipocalin 2 (oncogene 24p3)	9	129951552	3934	9q34	Hs.204238
	33	FetalLung							
	38	SalivaryGland							
	56	Trachea							
	62	BoneMarrow							
	75	Islet							
6	2	BronchialEpitelia	KRT17	keratin 17	17	-37029220	3872	17q12-q21	Hs.2785
	30	Tongue							
	37	HBEC							
	49	Prostate							
	56	Trachea							
	7	Skin							
6	2	BronchialEpitelia	MYB	v-myb myeloblastosis viral oncogene homolog (avian)	6	135544145	4602	6q22-q23	Hs.531941
	20	BM-CD33Myeloid							
	21	BM-CD105Endothelial							
	22	BM-CD34							

	53	Thymus							
	62	BoneMarrow							
6	19	PB-CD19BCells	IGHV3-9	immunoglobulin heavy variable 3-9	14		28451	14q32.33	
	23	PB-BDCA4DentriticCells							
	35	Tonsil							
	45	LymphNode							
	53	Thymus							
	56	Trachea							
6	17	PB-CD14Monocytes	QPCT	glutaminyl-peptide cyclotransferase (glutaminyl cyclase)	2	37425256	25797	2p22.2	Hs.79033
	20	BM-CD33Myeloid							
	36	WholeBlood							
	39	AdrenalCortex							
	57	AdrenalGland							
	75	Islet							
6	17	PB-CD14Monocytes	CD14	CD14 molecule	5	-139991500	929	5q22-q32|5q31.1	Hs.163867
	20	BM-CD33Myeloid							
	23	PB-BDCA4DentriticCells							
	36	WholeBlood							
	42	Lung							
	44	Liver							
6	17	PB-CD14Monocytes	IMPA2	inositol(myo)-1(or 4)-monophosphatase 2	18	11971454	3613	18p11.2	Hs.367992
	20	BM-CD33Myeloid							
	22	BM-CD34							
	3	LimbMuscle							
	36	WholeBlood							
	5	Kidney							
6	17	PB-CD14Monocytes	CPVL	carboxypeptidase, vitellogenic-like	7	-29001773	54504	7p15-p14	Hs.233389
	20	BM-CD33Myeloid							
	21	BM-CD105Endothelial							
	23	PB-BDCA4DentriticCells							
	36	WholeBlood							
	78	FetalHeart							
6	17	PB-CD14Monocytes	FCN1	ficolin (collagen/fibrinogen domain containing) 1	9	-136941256	2219	9q34	Hs.440898
	20	BM-CD33Myeloid							
	21	BM-CD105Endothelial							
	23	PB-BDCA4DentriticCells							
	36	WholeBlood							
	62	BoneMarrow							
6	17	PB-CD14Monocytes	HHEX	homeobox, hematopoietically expressed	10	94439679	3087	10q23.33	Hs.118651
	19	PB-CD19BCells							
	20	BM-CD33Myeloid							
	22	BM-CD34							
	23	PB-BDCA4DentriticCells							
	41	Thyroid							
6	17	PB-CD14Monocytes	ITGAM	integrin, alpha M (complement component 3 receptor 3 subunit)	16	31178788	3684	16p11.2	Hs.172631
	18	PB-CD56NKCells							
	20	BM-CD33Myeloid							
	23	PB-BDCA4DentriticCells							
	36	WholeBlood							
	62	BoneMarrow							
6	16	BM-CD71EarlyErythroid	CDKN3	cyclin-dependent kinase inhibitor 3 (CDK2-associated dual specificity phosphatase)	14	53933475	1033	14q22	Hs.84113
	55	Testis							
	66	TestisGermCell							
	67	TestisIntersitial							

	68	TestisLeydigCell							
	77	TestiSeminiferousTubule							
6	16	BM-CD71EarlyErythroid	SELENBP1	selenium binding protein 1	1	-149603404	8991	1q21-q22	Hs.632460
	2	BronchialEpitelia							
	21	BM-CD105Endothelial							
	41	Thyroid							
	42	Lung							
	54	FetalLiver							
6	14	PB-CD8TCells	CD69	CD69 molecule	12	-9796352	969	12p13-p12	Hs.208854
	15	PB-CD4TCells							
	18	PB-CD56NKCells							
	19	PB-CD19BCells							
	22	BM-CD34							
	45	LymphNode							
6	14	PB-CD8TCells	RASGRP2	RAS guanyl releasing protein 2 (calcium and DAG-regulated)	11	-64250959	10235	11q13	Hs.99491
	15	PB-CD4TCells							
	18	PB-CD56NKCells							
	19	PB-CD19BCells							
	22	BM-CD34							
	23	PB-BDCA4DentriticCells							
6	14	PB-CD8TCells	BIN2	bridging integrator 2	12	-49961089	51411	12q13	Hs.14770
	15	PB-CD4TCells							
	17	PB-CD14Monocytes							
	18	PB-CD56NKCells							
	20	BM-CD33Myeloid							
	36	WholeBlood							
6	13	721-BLymphoblasts	GHR	growth hormone receptor	5	42459782	2690	5p13-p12	Hs.125180
	3	LimbMuscle							
	4	ExtraocularMuscle							
	44	Liver							
	76	Adipocytes							
	78	FetalHeart							
6	13	721-BLymphoblasts	MTHFD2	methylenetetrahydrofolate dehydrogenase (NADP+ dependent) 2, methenyltetrahydrofolate cyclohydrolase	2	74279197	10797	2p13.1	Hs.469030
	21	BM-CD105Endothelial							
	22	BM-CD34							
	37	HBEC							
	63	CardiacMyocytes							
	72	SmoothMuscle							
6	13	721-BLymphoblasts	BLNK	B-cell linker	10	-97941452	29760	10q23.2-q23.33	Hs.444049
	19	PB-CD19BCells							
	22	BM-CD34							
	23	PB-BDCA4DentriticCells							
	35	Tonsil							
	45	LymphNode							
6	13	721-BLymphoblasts	PSMC6	proteasome (prosome, macropain) 26S subunit, ATPase, 6	14	52243667	5706	14q22.1	Hs.156171 Hs.491779
	18	PB-CD56NKCells							
	19	PB-CD19BCells							
	21	BM-CD105Endothelial							
	22	BM-CD34							
	78	FetalHeart							
6	13	721-BLymphoblasts	NMI	N-myc (and STAT) interactor	2	-151835230	9111	2p24.3-q21.3	Hs.54483
	17	PB-CD14Monocytes							
	20	BM-CD33Myeloid							
	21	BM-CD105Endothelial							

	22	BM-CD34							
	36	WholeBlood							
6	13	721-BLymphoblasts	PSMB10	proteasome (prosome, macropain) subunit, beta type, 10	16	-66525907	5699	16q22.1	Hs.9661
	17	PB-CD14Monocytes							
	18	PB-CD56NKCells							
	19	PB-CD19BCells							
	20	BM-CD33Myeloid							
	23	PB-BDCA4DentriticCells							
6	13	721-BLymphoblasts	PBK	PDZ binding kinase	8	-27723058	55872	8p21.2	Hs.104741
	16	BM-CD71EarlyErythroid							
	21	BM-CD105Endothelial							
	67	TestisIntersitial							
	68	TestisLeydigCell							
	77	TestiSeminiferousTubule							
6	13	721-BLymphoblasts	TUBG1	tubulin, gamma 1	17	38015219	7283	17q21	Hs.279669
	16	BM-CD71EarlyErythroid							
	21	BM-CD105Endothelial							
	55	Testis							
	67	TestisIntersitial							
	77	TestiSeminiferousTubule							
6	13	721-BLymphoblasts	DBF4	DBF4 homolog (S. cerevisiae)	7	87343479	10926	7q21.3	Hs.485380
	16	BM-CD71EarlyErythroid							
	21	BM-CD105Endothelial							
	22	BM-CD34							
	67	TestisIntersitial							
	68	TestisLeydigCell							
6	13	721-BLymphoblasts	PRC1	protein regulator of cytokinesis 1	15	-89310276	9055	15q26.1	Hs.567385
	16	BM-CD71EarlyErythroid							
	21	BM-CD105Endothelial							
	22	BM-CD34							
	63	CardiacMyocytes							
	78	FetalHeart							
6	13	721-BLymphoblasts	TOP2A	topoisomerase (DNA) II alpha 170kDa	17	-35798323	7153	17q21-q22	Hs.156346
	16	BM-CD71EarlyErythroid							
	21	BM-CD105Endothelial							
	22	BM-CD34							
	54	FetalLiver							
	78	FetalHeart							
6	13	721-BLymphoblasts	RRM2	ribonucleotide reductase M2 polypeptide	2	10180185	6241	2p25-p24	Hs.226390
	16	BM-CD71EarlyErythroid							
	21	BM-CD105Endothelial							
	22	BM-CD34							
	53	Thymus							
	54	FetalLiver							
6	13	721-BLymphoblasts	CORO1A	coronin, actin binding protein, 1A	16	30102426	11151	16p11.2	Hs.415067
	14	PB-CD8TCells							
	15	PB-CD4TCells							
	18	PB-CD56NKCells							
	23	PB-BDCA4DentriticCells							
	36	WholeBlood							
6	12	Placenta	COL4A2	collagen, type IV, alpha 2	13	109757631	1284	13q34	Hs.508716
	33	FetalLung							
	63	CardiacMyocytes							
	72	SmoothMuscle							

	76	Adipocytes							
	78	FetalHeart							
6	12	Placenta	SERPINE1	serpin peptidase inhibitor, clade E (nexin, plasminogen activator inhibitor type 1), member 1	7	100557171	5054	7q21.3-q22	Hs.414795
	33	FetalLung							
	37	HBEC							
	63	CardiacMyocytes							
	72	SmoothMuscle							
	76	Adipocytes							
6	12	Placenta	CAV1	caveolin 1, caveolae protein, 22kDa	7	115952074	857	7q31.1	Hs.74034
	33	FetalLung							
	37	HBEC							
	48	Uterus							
	76	Adipocytes							
	78	FetalHeart							
6	12	Placenta	PERP	PERP, TP53 apoptosis effector	6	-138453618	64065	6q24	Hs.520421
	30	Tongue							
	37	HBEC							
	49	Prostate							
	56	Trachea							
	7	Skin							
6	12	Placenta	CDH5	cadherin 5, type 2, VE-cadherin (vascular epithelium)	16	64958063	1003	16q22.1	Hs.76206
	3	LimbMuscle							
	33	FetalLung							
	4	ExtraocularMuscle							
	42	Lung							
	78	FetalHeart							
6	12	Placenta	MEST	mesoderm specific transcript homolog (mouse)	7	129919173, 129913281, 129918408	4232	7q32	Hs.270978
	22	BM-CD34							
	33	FetalLung							
	63	CardiacMyocytes							
	72	SmoothMuscle							
	78	FetalHeart							
6	1	Hippocampus	TBPL1	TBP-like 1	6	134315993	9519	6q22.1-q22.3	Hs.486507
	55	Testis							
	66	TestisGermCell							
	67	TestisIntersitial							
	68	TestisLeydigCell							
	77	TestiSeminiferousTubule							
6	1	Hippocampus	DNM3	dynamin 3	1	170077260	26052	1q24.3	Hs.584880
	46	Amygdala							
	51	PrefrontalCortex							
	61	Thalamus							
	69	Hypothalamus							
	70	OccipitalLobe							
6	1	Hippocampus	TCEAL2	transcription elongation factor A (SII)-like 2	X	101267315	140597	Xq22.1-q22.3	Hs.401835
	46	Amygdala							
	51	PrefrontalCortex							
	60	PituitaryGland							
	69	Hypothalamus							
	74	WholeBrain							
6	1	Hippocampus	SNAP91	synaptosomal-associated protein, 91kDa homolog (mouse)	6	-84319331	9892	6q14.2	Hs.368046
	46	Amygdala							
	51	PrefrontalCortex							
	59	Cerebellum							
	70	OccipitalLobe							

	74	WholeBrain							
6	1	Hippocampus	AGXT2L1	alanine-glyoxylate aminotransferase 2-like 1	4	-109882652	64850	4q25	Hs.106576
	46	Amygdala							
	51	PrefrontalCortex							
	58	SpinalCord							
	69	Hypothalamus							
	73	CaudateNucleus							
6	1	Hippocampus	CHL1	cell adhesion molecule with homology to L1CAM (close homolog of L1)	3	213649	10752	3p26.1	Hs.148909
	43	FetalBrain							
	46	Amygdala							
	51	PrefrontalCortex							
	65	OlfactoryBulb							
	70	OccipitalLobe							
6	1	Hippocampus	TAC1	tachykinin, precursor 1 (substance K, substance P, neurokinin 1, neurokinin 2, neuromedin L, neurokinin alpha, neuropeptide K, neuropeptide gamma)	7	97199310	6863	7q21-q22	Hs.2563
	25	MedullaOblongata							
	46	Amygdala							
	69	Hypothalamus							
	73	CaudateNucleus							
	74	WholeBrain							
6	1	Hippocampus	TMEM16C	transmembrane protein 16C	11	26309546	63982	11p14.2	Hs.91791
	25	MedullaOblongata							
	26	Pons							
	46	Amygdala							
	51	PrefrontalCortex							
	73	CaudateNucleus							
6	1	Hippocampus	PLEKHA5	pleckstrin homology domain containing, family A member 5	12	19173994	54477	12p12	Hs.188614
	2	BronchialEpitelia							
	48	Uterus							
	5	Kidney							
	51	PrefrontalCortex							
	78	FetalHeart							
7	43	FetalBrain	SST	somatostatin	3	-188869389	6750	3q28	Hs.12409
	46	Amygdala							
	50	Pancreas							
	69	Hypothalamus							
	70	OccipitalLobe							
	74	WholeBrain							
	75	Islet							
7	36	WholeBlood	ANPEP	alanyl (membrane) aminopeptidase (aminopeptidase N, aminopeptidase M, microsomal aminopeptidase, CD13, p150)	15	-88129129	290	15q25-q26	Hs.1239
	44	Liver							
	49	Prostate							
	5	Kidney							
	50	Pancreas							
	72	SmoothMuscle							
	76	Adipocytes							
7	33	FetalLung	RBP4	retinol binding protein 4, plasma	10	-95341583	5950	10q23-q24	Hs.50223
	44	Liver							
	5	Kidney							
	54	FetalLiver							
	60	PituitaryGland							
	75	Islet							
	76	Adipocytes							

7	30	Tongue	KRT14	keratin 14 (epidermolysis bullosa simplex, Dowling-Meara, Koebner)	17	-36992058	3861	17q12-q21	Hs.2785
	35	Tonsil							
	37	HBEC							
	53	Thymus							
	56	Trachea							
	65	OlfactoryBulb							
	7	Skin							
7	3	LimbMuscle	CXCL14	chemokine (C-X-C motif) ligand 14	5	-134934274	9547	5q31	Hs.483444
	30	Tongue							
	38	SalivaryGland							
	4	ExtraocularMuscle							
	5	Kidney							
	7	Skin							
	74	WholeBrain							
7	3	LimbMuscle	ENO3	enolase 3 (beta, muscle)	17	4795158	2027	17pter-p11	Hs.224171
	30	Tongue							
	32	PsoasMuscle							
	4	ExtraocularMuscle							
	44	Liver							
	47	Heart							
	78	FetalHeart							
7	3	LimbMuscle	TNNC1	troponin C type 1 (slow)	3	-52460157	7134	3p21.3-p14.3	Hs.118845
	30	Tongue							
	32	PsoasMuscle							
	4	ExtraocularMuscle							
	41	Thyroid							
	47	Heart							
	78	FetalHeart							
7	3	LimbMuscle	MB	myoglobin	22	-34332757	4151	22q13.1	Hs.517586
	30	Tongue							
	32	PsoasMuscle							
	4	ExtraocularMuscle							
	41	Thyroid							
	47	Heart							
	78	FetalHeart							
7	29	TemporalLobe	GAP43	growth associated protein 43	3	116825141	2596	3q13.1-q13.2	Hs.134974
	43	FetalBrain							
	46	Amygdala							
	51	PrefrontalCortex							
	61	Thalamus							
	70	OccipitalLobe							
	74	WholeBrain							
7	27	Appendix	TAGLN	transgelin	11	116575249	6876	11q23.2	Hs.632099
	40	Ovary							
	48	Uterus							
	49	Prostate							
	63	CardiacMyocytes							
	72	SmoothMuscle							
	76	Adipocytes							
7	27	Appendix	ADH1B	alcohol dehydrogenase IB (class I), beta polypeptide	4	-100446551	125	4q21-q23	Hs.4
	33	FetalLung							
	4	ExtraocularMuscle							
	40	Ovary							
	44	Liver							
	48	Uterus							
	76	Adipocytes							

7	2	BronchialEpitelia	MLF1	myeloid leukemia factor 1	3	159771677	4291	3q25.1	Hs.85195
	55	Testis							
	66	TestisGermCell							
	67	TestisIntersitial							
	68	TestisLeydigCell							
	77	TestiSeminiferousTubule							
	78	FetalHeart							
7	2	BronchialEpitelia	KRT15	keratin 15	17	-36923523	3866	17q21.2	Hs.2785
	30	Tongue							
	35	Tonsil							
	49	Prostate							
	53	Thymus							
	56	Trachea							
	7	Skin							
7	2	BronchialEpitelia					650893		
	30	Tongue							
	35	Tonsil							
	37	HBEC							
	5	Kidney							
	56	Trachea							
	7	Skin							
7	19	PB-CD19BCells	IGHV3-23	immunoglobulin heavy variable 3-23	14		28442	14q32.33	
	23	PB-BDCA4DentriticCells							
	35	Tonsil							
	45	LymphNode							
	53	Thymus							
	56	Trachea							
	62	BoneMarrow							
7	19	PB-CD19BCells	IGKC	immunoglobulin kappa constant	2		3514	2p12	Hs.449621
	23	PB-BDCA4DentriticCells							
	35	Tonsil							
	38	SalivaryGland							
	45	LymphNode							
	53	Thymus							
	56	Trachea							
7	18	PB-CD56NKCells	AKR1C3	aldo-keto reductase family 1, member C3 (3-alpha hydroxysteroid dehydrogenase, type II)	10	5126567	8644	10p15-p14	Hs.78183
	21	BM-CD105Endothelial							
	22	BM-CD34							
	37	HBEC							
	5	Kidney							
	72	SmoothMuscle							
	76	Adipocytes							
7	17	PB-CD14Monocytes	EVI2A	ecotropic viral integration site 2A	17	-26668801	2123	17q11.2	Hs.591198
	18	PB-CD56NKCells							
	20	BM-CD33Myeloid							
	23	PB-BDCA4DentriticCells							
	36	WholeBlood							
	58	SpinalCord							
	69	Hypothalamus							
7	17	PB-CD14Monocytes	TYROBP	TYRO protein tyrosine kinase binding protein	19	-41087145	7305	19q13.1	Hs.515369
	18	PB-CD56NKCells							
	20	BM-CD33Myeloid							
	21	BM-CD105Endothelial							
	23	PB-BDCA4DentriticCells							
	36	WholeBlood							
	42	Lung							

7	17	PB-CD14Monocytes	LOC646082		22		646082	22q11.23	
	18	PB-CD56NKCells							
	19	PB-CD19BCells							
	2	BronchialEpitelia							
	23	PB-BDCA4DentriticCells							
	42	Lung							
	78	FetalHeart							
7	16	BM-CD71EarlyErythroid	SLC25A37	solute carrier family 25, member 37	8	23442307	51312	8p21.2	Hs.122514
	21	BM-CD105Endothelial							Hs.596025
	33	FetalLung							
	36	WholeBlood							
	49	Prostate							
	54	FetalLiver							
	62	BoneMarrow							
7	16	BM-CD71EarlyErythroid	RABGAP1L	RAB GTPase activating protein 1-like	1	173111306,	9910	1q24	Hs.585378
	17	PB-CD14Monocytes				172395256			Hs.591475
	18	PB-CD56NKCells							Hs.615081
	19	PB-CD19BCells							
	21	BM-CD105Endothelial							
	23	PB-BDCA4DentriticCells							
	78	FetalHeart							
7	14	PB-CD8TCells	ADCY7	adenylate cyclase 7	16	48879322	113	16q12-q13	Hs.513578
	15	PB-CD4TCells							
	17	PB-CD14Monocytes							
	18	PB-CD56NKCells							
	20	BM-CD33Myeloid							
	23	PB-BDCA4DentriticCells							
	36	WholeBlood							
7	14	PB-CD8TCells	ICAM3	intercellular adhesion molecule 3	19	-10305453	3385	19p13.3-p13.2	Hs.75516
	15	PB-CD4TCells							
	17	PB-CD14Monocytes							
	18	PB-CD56NKCells							
	19	PB-CD19BCells							
	20	BM-CD33Myeloid							
	36	WholeBlood							
7	14	PB-CD8TCells	ISG20	interferon stimulated exonuclease gene 20kDa	15	86983042	3669	15q26	Hs.459265
	15	PB-CD4TCells							
	16	BM-CD71EarlyErythroid							
	18	PB-CD56NKCells							
	19	PB-CD19BCells							
	23	PB-BDCA4DentriticCells							
	36	WholeBlood							
7	13	721-BLymphoblasts	PLOD2	procollagen-lysine, 2-oxoglutarate 5-dioxygenase 2	3	-147269917	5352	3q23-q24	Hs.477866
	37	HBEC							
	41	Thyroid							
	63	CardiacMyocytes							
	64	FetalThyroid							
	72	SmoothMuscle							
	78	FetalHeart							
7	13	721-BLymphoblasts	NCF4	neutrophil cytosolic factor 4, 40kDa	22	35586990	4689	22q13.1	Hs.474781
	17	PB-CD14Monocytes							
	19	PB-CD19BCells							
	20	BM-CD33Myeloid							
	23	PB-BDCA4DentriticCells							
	36	WholeBlood							
	62	BoneMarrow							

7	13	721-BLymphoblasts	LYN	v-yes-1 Yamaguchi sarcoma viral related oncogene homolog	8	56954925	4067	8q13	Hs.491767 Hs.614775
	17	PB-CD14Monocytes							
	18	PB-CD56NKCells							
	19	PB-CD19BCells							
	20	BM-CD33Myeloid							
	23	PB-BDCA4DentriticCells							
	36	WholeBlood							
7	13	721-BLymphoblasts	RBM3	RNA binding motif (RNP1, RRM) protein 3	X	48317779	5935	Xp11.2	Hs.301404
	17	PB-CD14Monocytes							
	18	PB-CD56NKCells							
	19	PB-CD19BCells							
	20	BM-CD33Myeloid							
	22	BM-CD34							
	23	PB-BDCA4DentriticCells							
7	13	721-BLymphoblasts	PMAIP1	phorbol-12-myristate-13-acetate-induced protein 1	18	55718216	5366	18q21.32	Hs.96
	17	PB-CD14Monocytes							
	18	PB-CD56NKCells							
	19	PB-CD19BCells							
	20	BM-CD33Myeloid							
	22	BM-CD34							
	23	PB-BDCA4DentriticCells							
7	13	721-BLymphoblasts	CDC20	CDC20 cell division cycle 20 homolog (S. cerevisiae)	1	43597214	991	1p34.1	Hs.524947
	16	BM-CD71EarlyErythroid							
	21	BM-CD105Endothelial							
	53	Thymus							
	55	Testis							
	63	CardiacMyocytes							
	67	TestisIntersitial							
7	13	721-BLymphoblasts	CKS2	CDC28 protein kinase regulatory subunit 2	9	91115932	1164	9q22	Hs.83758
	16	BM-CD71EarlyErythroid							
	21	BM-CD105Endothelial							
	22	BM-CD34							
	53	Thymus							
	55	Testis							
	78	FetalHeart							
7	13	721-BLymphoblasts	PSMD14	proteasome (prosome, macropain) 26S subunit, non-ATPase, 14	2	161873185	10213	2q24.2	Hs.567410
	16	BM-CD71EarlyErythroid							
	21	BM-CD105Endothelial							
	22	BM-CD34							
	23	PB-BDCA4DentriticCells							
	37	HBEC							
	78	FetalHeart							
7	13	721-BLymphoblasts	LBR	lamin B receptor	1	-223655827	3930	1q42.1	Hs.435166
	14	PB-CD8TCells							
	16	BM-CD71EarlyErythroid							
	17	PB-CD14Monocytes							
	18	PB-CD56NKCells							
	21	BM-CD105Endothelial							
	36	WholeBlood							
7	12	Placenta	GDF15	growth differentiation factor 15	19	18357984	9518	19p13.1-13.2	Hs.515258
	33	FetalLung							
	42	Lung							
	49	Prostate							
	5	Kidney							
	50	Pancreas							
	75	Islet							

7	12	Placenta	S100P	S100 calcium binding protein P	4	6746466	6286	4p16	Hs.2962
	2	BronchialEpitelia							
	20	BM-CD33Myeloid							
	36	WholeBlood							
	37	HBEC							
	56	Trachea							
	62	BoneMarrow							
7	11	DRG	LPL	lipoprotein lipase	8	19841057	4023	8p22	Hs.180878
	3	LimbMuscle							
	43	FetalBrain							
	65	OlfactoryBulb							
	76	Adipocytes							
	78	FetalHeart							
	9	CiliaryGanglion							
7	11	DRG	TSPAN8	tetraspanin 8	12	-69805143	7103	12q14.1-q21.1	Hs.170563
	2	BronchialEpitelia							
	3	LimbMuscle							
	49	Prostate							
	50	Pancreas							
	56	Trachea							
	65	OlfactoryBulb							
7	1	Hippocampus	BAALC	brain and acute leukemia, cytoplasmic	8	104222096	79870	8q22.3	Hs.533446
	51	PrefrontalCortex							
	58	SpinalCord							
	61	Thalamus							
	69	Hypothalamus							
	70	OccipitalLobe							
	74	WholeBrain							
7	1	Hippocampus	SYNGR3	synaptogyrin 3	16	1979968	9143	16p13	Hs.435277
	43	FetalBrain							
	46	Amygdala							
	51	PrefrontalCortex							
	61	Thalamus							
	69	Hypothalamus							
	74	WholeBrain							
7	1	Hippocampus	COL5A2	collagen, type V, alpha 2	2	-189604886	1290	2q14-q32	Hs.445827
	33	FetalLung							
	54	FetalLiver							
	63	CardiacMyocytes							
	72	SmoothMuscle							
	76	Adipocytes							
	78	FetalHeart							
7	1	Hippocampus	MYT1L	myelin transcription factor 1-like	2	-1771891	23040	2p25.3	Hs.434418
	25	MedullaOblongata							
	43	FetalBrain							
	46	Amygdala							
	51	PrefrontalCortex							
	70	OccipitalLobe							
	74	WholeBrain							
7	1	Hippocampus	SC4MOL	sterol-C4-methyl oxidase-like	4	166468267	6307	4q32-q34	Hs.105269
	13	721-BLymphoblasts							Hs.593050
	33	FetalLung							
	37	HBEC							
	54	FetalLiver							
	57	AdrenalGland							
	76	Adipocytes							

7	1	Hippocampus	FLJ22028		12	21481877	79912	12p12.1	Hs.46835
	13	721-BLymphoblasts							
	14	PB-CD8TCells							
	3	LimbMuscle							
	4	ExtraocularMuscle							
	5	Kidney							
	78	FetalHeart							
8	5	Kidney	SERPINA5	serpin peptidase inhibitor, clade A (alpha-1 antiproteinase, antitrypsin), member 5	14	94117563	5104	14q32.1	Hs.510334
	54	FetalLiver							
	55	Testis							
	57	AdrenalGland							
	66	TestisGermCell							
	67	TestisIntersitial							
	68	TestisLeydigCell							
	77	TestiSeminiferousTubule							
8	44	Liver	TTR	transthyretin (prealbumin, amyloidosis type I)	18	27425837	7276	18q12.1	Hs.427202
	46	Amygdala							
	54	FetalLiver							
	61	Thalamus							
	69	Hypothalamus							
	71	CerebellumPeduncles							
	74	WholeBrain							
	75	Islet							
8	38	SalivaryGland	AZGP1	alpha-2-glycoprotein 1, zinc	7	-99402287	563	7q22.1	Hs.546239
	44	Liver							
	49	Prostate							
	5	Kidney							
	50	Pancreas							
	54	FetalLiver							
	56	Trachea							
	65	OlfactoryBulb							
8	33	FetalLung	LAMB1	laminin, beta 1	7	-107351498	3912	7q22	Hs.489646
	48	Uterus							
	5	Kidney							
	63	CardiacMyocytes							
	65	OlfactoryBulb							
	72	SmoothMuscle							
	76	Adipocytes							
	78	FetalHeart							
8	33	FetalLung	C7	complement component 7	5	40945355	730	5p13	Hs.78065
	39	AdrenalCortex							
	40	Ovary							
	45	LymphNode							
	48	Uterus							
	5	Kidney							
	57	AdrenalGland							
	78	FetalHeart							
8	3	LimbMuscle	DES	desmin	2	219991342	1674	2q35	Hs.594952
	30	Tongue							
	32	PsoasMuscle							
	4	ExtraocularMuscle							
	47	Heart							
	48	Uterus							
	49	Prostate							
	78	FetalHeart							

8	3	LimbMuscle	TTN	titin	2	-358417256	7273	2q31	Hs.134602
	30	Tongue							
	32	PsoasMuscle							
	4	ExtraocularMuscle							
	41	Thyroid							
	47	Heart							
	64	FetalThyroid							
	78	FetalHeart							
8	3	LimbMuscle	MYH7	myosin, heavy polypeptide 7, cardiac muscle, beta	14	-22951789	4625	14q12	Hs.278432
	30	Tongue							
	32	PsoasMuscle							
	4	ExtraocularMuscle							
	41	Thyroid							
	47	Heart							
	49	Prostate							
	78	FetalHeart							
8	3	LimbMuscle	TPM2	tropomyosin 2 (beta)	9	-71344912	7169	9p13.2-p13.1	Hs.300772
	30	Tongue							
	32	PsoasMuscle							
	4	ExtraocularMuscle							
	41	Thyroid							
	47	Heart							
	48	Uterus							
	78	FetalHeart							
8	2	BronchialEpitelia	S100A2	S100 calcium binding protein A2	1	-151800210	6273	1q21	Hs.516484
	30	Tongue							
	35	Tonsil							
	37	HBEC							
	38	SalivaryGland							
	5	Kidney							
	56	Trachea							
	7	Skin							
8	2	BronchialEpitelia	KRT5	keratin 5 (epidermolysis bullosa simplex, Dowling-Meara/Kobner/Weber-Cockayne types)	12	-51194627	3852	12q12-q13	Hs.433845
	23	PB-BDCA4DentriticCells							
	30	Tongue							
	35	Tonsil							
	37	HBEC							
	53	Thymus							
	56	Trachea							
	7	Skin							
8	2	BronchialEpitelia	NUCB2	nucleobindin 2	11	17254887	4925	11p15.1-p14	Hs.128686
	21	BM-CD105Endothelial							
	22	BM-CD34							
	23	PB-BDCA4DentriticCells							
	38	SalivaryGland							
	50	Pancreas							
	75	Islet							
	78	FetalHeart							
8	19	PB-CD19BCells	IGKV1-39	immunoglobulin kappa variable 1-39	2		28930	2p12	
	23	PB-BDCA4DentriticCells							
	35	Tonsil							
	38	SalivaryGland							
	45	LymphNode							
	53	Thymus							
	56	Trachea							
	62	BoneMarrow							

8	17	PB-CD14Monocytes	CSPG2	chondroitin sulfate proteoglycan 2 (versican)	5	82803338	1462	5q14.3	Hs.443681
	20	BM-CD33Myeloid							
	23	PB-BDCA4DentriticCells							
	33	FetalLung							
	36	WholeBlood							
	63	CardiacMyocytes							
	72	SmoothMuscle							
	78	FetalHeart							
8	17	PB-CD14Monocytes	MNDA	myeloid cell nuclear differentiation antigen	1	157067791	4332	1q22	Hs.153837
	18	PB-CD56NKCells							
	20	BM-CD33Myeloid							
	21	BM-CD105Endothelial							
	22	BM-CD34							
	23	PB-BDCA4DentriticCells							
	36	WholeBlood							
	62	BoneMarrow							
8	17	PB-CD14Monocytes	PYCARD	PYD and CARD domain containing	16	-31120309	29108	16p12-p11.2	Hs.499094
	18	PB-CD56NKCells							
	19	PB-CD19BCells							
	20	BM-CD33Myeloid							
	22	BM-CD34							
	23	PB-BDCA4DentriticCells							
	36	WholeBlood							
	42	Lung							
8	16	BM-CD71EarlyErythroid	NFE2	nuclear factor (erythroid-derived 2), 45kDa	12	-52972164	4778	12q13	Hs.75643
	17	PB-CD14Monocytes							
	20	BM-CD33Myeloid							
	21	BM-CD105Endothelial							
	22	BM-CD34							
	36	WholeBlood							
	54	FetalLiver							
	62	BoneMarrow							
8	14	PB-CD8TCells	EVI2B	ecotropic viral integration site 2B	17	-26654918	2124	17q11.2	Hs.5509
	17	PB-CD14Monocytes							
	18	PB-CD56NKCells							
	19	PB-CD19BCells							
	20	BM-CD33Myeloid							
	23	PB-BDCA4DentriticCells							
	36	WholeBlood							
	45	LymphNode							
8	14	PB-CD8TCells	ZNF651	zinc finger protein 651	3	42675877	92999	3p22.1	Hs.409561
	15	PB-CD4TCells							
	18	PB-CD56NKCells							
	20	BM-CD33Myeloid							
	22	BM-CD34							
	23	PB-BDCA4DentriticCells							
	48	Uterus							
	78	FetalHeart							
8	14	PB-CD8TCells	CD52	CD52 molecule	1	26516997	1043	1p36	Hs.276770
	15	PB-CD4TCells							
	17	PB-CD14Monocytes							
	18	PB-CD56NKCells							
	19	PB-CD19BCells							
	20	BM-CD33Myeloid							
	36	WholeBlood							
	53	Thymus							

8	13	721-BLymphoblasts	PRG1	proteoglycan 1, secretory granule	10	70517833	5552	10q22.1	Hs.1908
	17	PB-CD14Monocytes							
	18	PB-CD56NKCells							
	20	BM-CD33Myeloid							
	22	BM-CD34							
	23	PB-BDCA4DentriticCells							
	36	WholeBlood							
	62	BoneMarrow							
8	13	721-BLymphoblasts	NBR1	neighbor of BRCA1 gene 1	17	38576771, 38576036, 38576023	4077	17q21.31	Hs.277721 Hs.373818 Hs.546264
	16	BM-CD71EarlyErythroid							
	21	BM-CD105Endothelial							
	55	Testis							
	66	TestisGermCell							
	67	TestisIntersitial							
	68	TestisLeydigCell							
	77	TestiSeminiferousTubule							
8	13	721-BLymphoblasts	DCK	deoxycytidine kinase	4	72078255	1633	4q13.3-q21.1	Hs.709
	16	BM-CD71EarlyErythroid							
	18	PB-CD56NKCells							
	19	PB-CD19BCells							
	21	BM-CD105Endothelial							
	22	BM-CD34							
	23	PB-BDCA4DentriticCells							
	53	Thymus							
8	13	721-BLymphoblasts	MCM5	MCM5 minichromosome maintenance deficient 5, cell division cycle 46 (S. cerevisiae)	22	34126127	4174	22q13.1	Hs.517582
	16	BM-CD71EarlyErythroid							
	18	PB-CD56NKCells							
	19	PB-CD19BCells							
	21	BM-CD105Endothelial							
	22	BM-CD34							
	23	PB-BDCA4DentriticCells							
	53	Thymus							
8	13	721-BLymphoblasts	HCLS1	hematopoietic cell-specific Lyn substrate 1	3	-122832936	3059	3q13	Hs.14601
	14	PB-CD8TCells							
	15	PB-CD4TCells							
	17	PB-CD14Monocytes							
	18	PB-CD56NKCells							
	20	BM-CD33Myeloid							
	23	PB-BDCA4DentriticCells							
	36	WholeBlood							
8	13	721-BLymphoblasts	CD48	CD48 molecule	1	-158915161	962	1q21.3-q22	Hs.243564
	14	PB-CD8TCells							
	15	PB-CD4TCells							
	17	PB-CD14Monocytes							
	18	PB-CD56NKCells							
	19	PB-CD19BCells							
	20	BM-CD33Myeloid							
	36	WholeBlood							
8	12	Placenta	COL1A2	collagen, type I, alpha 2	7	93861808	1278	7q22.1	Hs.489142
	33	FetalLung							
	48	Uterus							
	63	CardiacMyocytes							
	64	FetalThyroid							
	72	SmoothMuscle							
	76	Adipocytes							
	78	FetalHeart							

8	12	Placenta					492304		
	33	FetalLung							
	4	ExtraocularMuscle							
	44	Liver							
	54	FetalLiver							
	64	FetalThyroid							
	65	OlfactoryBulb							
	78	FetalHeart							
8	12	Placenta	COL15A1	collagen, type XV, alpha 1	9	100746010	1306	9q21-q22	Hs.409034
	3	LimbMuscle							
	4	ExtraocularMuscle							
	48	Uterus							
	57	AdrenalGland							
	66	TestisGermCell							
	76	Adipocytes							
	78	FetalHeart							
8	12	Placenta	CLIC3	chloride intracellular channel 3	9	-139008880	9022	9q34.3	Hs.64746
	18	PB-CD56NKCells							
	23	PB-BDCA4DentriticCells							
	30	Tongue							
	33	FetalLung							
	41	Thyroid							
	42	Lung							
	64	FetalThyroid							
8	1	Hippocampus	TUBB2A	tubulin, beta 2A	6	-3098902	7280	6p25	Hs.300701
	43	FetalBrain							
	46	Amygdala							
	59	Cerebellum							
	61	Thalamus							
	69	Hypothalamus							
	70	OccipitalLobe							
	74	WholeBrain							
8	1	Hippocampus	CHGB	chromogranin B (secretogranin 1)	20	5840167	1114	20pter-p12	Hs.516874
	39	AdrenalCortex							
	4	ExtraocularMuscle							
	57	AdrenalGland							
	59	Cerebellum							
	60	PituitaryGland							
	71	CerebellumPeduncles							
	75	Islet							
8	1	Hippocampus	MT1M	metallothionein 1M	16	55224072	4499	16q13	Hs.643532
	33	FetalLung							
	42	Lung							
	44	Liver							
	5	Kidney							
	50	Pancreas							
	54	FetalLiver							
	75	Islet							
9	3	LimbMuscle	LOC125893		19	-58144445	125893	19q13.41	Hs.446907
	30	Tongue							
	32	PsoasMuscle							
	4	ExtraocularMuscle							
	41	Thyroid							
	47	Heart							
	55	Testis							
	64	FetalThyroid							

	78	FetalHeart							
9	3	LimbMuscle	MYL2	myosin, light polypeptide 2, regulatory, cardiac, slow	12	-109833032	4633	12q23-q24.3	Hs.75535
	30	Tongue							
	32	PsoasMuscle							
	4	ExtraocularMuscle							
	41	Thyroid							
	47	Heart							
	55	Testis							
	64	FetalThyroid							
	78	FetalHeart							
9	3	LimbMuscle	CKM	creatine kinase, muscle	19	-50501511	1158	19q13.2-q13.3	Hs.334347
	30	Tongue							
	32	PsoasMuscle							
	4	ExtraocularMuscle							
	41	Thyroid							
	47	Heart							
	55	Testis							
	64	FetalThyroid							
	78	FetalHeart							
9	2	BronchialEpitelia	GSTA1	glutathione S-transferase A1	6	-52764347	2938	6p12.1	Hs.446309
	44	Liver							
	5	Kidney							
	54	FetalLiver							
	55	Testis							
	57	AdrenalGland							
	66	TestisGermCell							
	67	TestisIntersitial							
	68	TestisLeydigCell							
9	2	BronchialEpitelia	CD24	CD24 molecule	6	-19611913	934	6q21	Hs.375108
	37	HBEC							
	38	SalivaryGland							
	41	Thyroid							
	43	FetalBrain							
	5	Kidney							
	56	Trachea							
	64	FetalThyroid							
	75	Islet							
9	2	BronchialEpitelia	ALDH1A1	aldehyde dehydrogenase 1 family, member A1	9	-74705407	216	9q21.13	Hs.76392
	21	BM-CD105Endothelial							
	4	ExtraocularMuscle							
	5	Kidney							
	54	FetalLiver							
	56	Trachea							
	60	PituitaryGland							
	65	OlfactoryBulb							
	66	TestisGermCell							
9	17	PB-CD14Monocytes	PPBP	pro-platelet basic protein (chemokine (C-X-C motif) ligand 7)	4	-75071621	5473	4q12-q13	Hs.2164
	19	PB-CD19BCells							
	21	BM-CD105Endothelial							
	22	BM-CD34							
	23	PB-BDCA4DentriticCells							
	36	WholeBlood							
	54	FetalLiver							

	62	BoneMarrow							
	78	FetalHeart							
9	14	PB-CD8TCells	CXCR4	chemokine (C-X-C motif) receptor 4	2	-136588389	7852	2q21	Hs.421986
	15	PB-CD4TCells							
	17	PB-CD14Monocytes							
	18	PB-CD56NKCells							
	19	PB-CD19BCells							
	23	PB-BDCA4DentriticCells							
	36	WholeBlood							
	53	Thymus							
	62	BoneMarrow							
9	14	PB-CD8TCells	GMFG	glia maturation factor, gamma	19	-44510841	9535	19q13.2	Hs.5210
	15	PB-CD4TCells							
	17	PB-CD14Monocytes							
	18	PB-CD56NKCells							
	19	PB-CD19BCells							
	20	BM-CD33Myeloid							
	22	BM-CD34							
	23	PB-BDCA4DentriticCells							
	36	WholeBlood							
9	14	PB-CD8TCells	SELL	selectin L (lymphocyte adhesion molecule 1)	1	-167926433	6402	1q23-q25	Hs.82848
	15	PB-CD4TCells							
	17	PB-CD14Monocytes							
	18	PB-CD56NKCells							
	19	PB-CD19BCells							
	20	BM-CD33Myeloid							
	22	BM-CD34							
	23	PB-BDCA4DentriticCells							
	36	WholeBlood							
9	14	PB-CD8TCells	ITGA4	integrin, alpha 4 (antigen CD49D, alpha 4 subunit of VLA-4 receptor)	2	182029863	3676	2q31.3	Hs.440955
	15	PB-CD4TCells							
	16	BM-CD71EarlyErythroid							
	17	PB-CD14Monocytes							
	18	PB-CD56NKCells							
	20	BM-CD33Myeloid							
	21	BM-CD105Endothelial							
	22	BM-CD34							
	23	PB-BDCA4DentriticCells							
9	13	721-BLymphoblasts	IGJ	immunoglobulin J polypeptide, linker protein for immunoglobulin alpha and mu polypeptides	4	-71740547	3512	4q21	Hs.643431
	18	PB-CD56NKCells							
	19	PB-CD19BCells							
	23	PB-BDCA4DentriticCells							
	35	Tonsil							
	38	SalivaryGland							
	45	LymphNode							
	56	Trachea							
	62	BoneMarrow							
9	13	721-BLymphoblasts	HLA-DRA	major histocompatibility complex, class II, DR alpha	6	32515624	3122	6p21.3	Hs.520048
	17	PB-CD14Monocytes							
	19	PB-CD19BCells							
	20	BM-CD33Myeloid							
	22	BM-CD34							
	23	PB-BDCA4DentriticCells							

	35	Tonsil							
	36	WholeBlood							
	45	LymphNode							
9	13	721-BLymphoblasts	DPCR1	diffuse panbronchiolitis critical region 1	6	31027537, 2367969, 2166477	135656	6p21.33	Hs.631993
	17	PB-CD14Monocytes							
	19	PB-CD19BCells							
	20	BM-CD33Myeloid							
	22	BM-CD34							
	23	PB-BDCA4DentriticCells							
	35	Tonsil							
	36	WholeBlood							
	45	LymphNode							
9	13	721-BLymphoblasts	MLF1IP	MLF1 interacting protein	4	-185852231	79682	4q35.1	Hs.481307 Hs.575032
	16	BM-CD71EarlyErythroid							
	21	BM-CD105Endothelial							
	22	BM-CD34							
	55	Testis							
	66	TestisGermCell							
	67	TestisIntersitial							
	68	TestisLeydigCell							
	77	TestiSeminiferousTubule							
9	13	721-BLymphoblasts	PTPN6	protein tyrosine phosphatase, non-receptor type 6	12	6930703, 6926000	5777	12p13	Hs.63489
	14	PB-CD8TCells							
	15	PB-CD4TCells							
	17	PB-CD14Monocytes							
	18	PB-CD56NKCells							
	19	PB-CD19BCells							
	20	BM-CD33Myeloid							
	23	PB-BDCA4DentriticCells							
	36	WholeBlood							
9	13	721-BLymphoblasts	PSCDBP	pleckstrin homology, Sec7 and coiled-coil domains, binding protein	2	-157979377	9595	2q11.2	Hs.270
	14	PB-CD8TCells							
	15	PB-CD4TCells							
	17	PB-CD14Monocytes							
	18	PB-CD56NKCells							
	19	PB-CD19BCells							
	20	BM-CD33Myeloid							
	23	PB-BDCA4DentriticCells							
	36	WholeBlood							
9	13	721-BLymphoblasts	LRRFIP1	leucine rich repeat (in FLII) interacting protein 1	2	238265667	9208	2q37.3	Hs.471779
	14	PB-CD8TCells							
	15	PB-CD4TCells							
	17	PB-CD14Monocytes							
	18	PB-CD56NKCells							
	19	PB-CD19BCells							
	20	BM-CD33Myeloid							
	22	BM-CD34							
	23	PB-BDCA4DentriticCells							
9	12	Placenta	LUM	lumican	12	-90021363	4060	12q21.3-q22	Hs.406475
	33	FetalLung							
	4	ExtraocularMuscle							
	40	Ovary							
	45	LymphNode							
	48	Uterus							

	5	Kidney							
	76	Adipocytes							
	78	FetalHeart							
9	12	Placenta	KRT7	keratin 7	12	50913220	3855	12q12-q13	Hs.411501
	2	BronchialEpitelia							
	33	FetalLung							
	37	HBEC							
	41	Thyroid							
	42	Lung							
	63	CardiacMyocytes							
	64	FetalThyroid							
	72	SmoothMuscle							
9	12	Placenta	VAMP8	vesicle-associated membrane protein 8 (endobrevin)	2	85658227	8673	2p12-p11.2	Hs.534373
	17	PB-CD14Monocytes							
	18	PB-CD56NKCells							
	2	BronchialEpitelia							
	20	BM-CD33Myeloid							
	22	BM-CD34							
	23	PB-BDCA4DentriticCells							
	37	HBEC							
	5	Kidney							
9	1	Hippocampus	PLEKHB1	pleckstrin homology domain containing, family B (evectins) member 1	11	73034870	58473	11q13.5-q14.1	Hs.445489
	51	PrefrontalCortex							
	58	SpinalCord							
	61	Thalamus							
	65	OlfactoryBulb							
	69	Hypothalamus							
	70	OccipitalLobe							
	73	CaudateNucleus							
	74	WholeBrain							
9	1	Hippocampus	CAMK2B	calcium/calmodulin-dependent protein kinase (CaM kinase) II beta	7	-44225421	816	22q12|7p14.3-p14.1	Hs.351887
	25	MedullaOblongata							
	29	TemporalLobe							
	46	Amygdala							
	51	PrefrontalCortex							
	6	SubthalamicNucleus							
	70	OccipitalLobe							
	71	CerebellumPeduncles							
	74	WholeBrain							
9	1	Hippocampus	PCP4	Purkinje cell protein 4	21	40161216	5121	21q22.2	Hs.80296
	25	MedullaOblongata							
	26	Pons							
	52	CingulateCortex							
	59	Cerebellum							
	61	Thalamus							
	69	Hypothalamus							
	73	CaudateNucleus							
	74	WholeBrain							
9	1	Hippocampus	PMP2	peripheral myelin protein 2	8	-82515120	5375	8q21.3-q22.1	Hs.571512
	11	DRG							
	46	Amygdala							
	58	SpinalCord							
	61	Thalamus							
	65	OlfactoryBulb							
	69	Hypothalamus							

	70	OccipitalLobe							
	73	CaudateNucleus							
10	3	LimbMuscle	ACTA1	actin, alpha 1, skeletal muscle	1	-227633617	58	1q42.13-q42.2	Hs.1288
	30	Tongue							
	32	PsoasMuscle							
	4	ExtraocularMuscle							
	41	Thyroid							
	47	Heart							
	49	Prostate							
	55	Testis							
	64	FetalThyroid							
	78	FetalHeart							
10	2	BronchialEpitelia	TACSTD1	tumor-associated calcium signal transducer 1	2	47449970	4072	2p21	Hs.542050
	21	BM-CD105Endothelial							
	33	FetalLung							
	41	Thyroid							
	49	Prostate							
	5	Kidney							
	50	Pancreas							
	60	PituitaryGland							
	64	FetalThyroid							
	75	Islet							
10	18	PB-CD56NKCells	CTA-246H3.1		22	24043887	91353	22q11.23	Hs.567636
	19	PB-CD19BCells							
	23	PB-BDCA4DentriticCells							
	35	Tonsil							
	38	SalivaryGland							
	42	Lung							
	45	LymphNode							
	53	Thymus							
	56	Trachea							
	62	BoneMarrow							
10	17	PB-CD14Monocytes	S100A9	S100 calcium binding protein A9 (calgranulin B)	1	151596953	6280	1q21	Hs.112405
	20	BM-CD33Myeloid							
	21	BM-CD105Endothelial							
	23	PB-BDCA4DentriticCells							
	30	Tongue							
	33	FetalLung							
	35	Tonsil							
	36	WholeBlood							
	37	HBEC							
	62	BoneMarrow							
10	17	PB-CD14Monocytes	SERPINA1	serpin peptidase inhibitor, clade A (alpha-1 antiproteinase, antitrypsin), member 1	14	-93914450	5265	14q32.1	Hs.525557
	18	PB-CD56NKCells							
	20	BM-CD33Myeloid							
	33	FetalLung							
	36	WholeBlood							
	42	Lung							
	44	Liver							
	5	Kidney							
	54	FetalLiver							
	75	Islet							

10	16	BM-CD71EarlyErythroid	CD36	CD36 molecule (thrombospondin receptor)	7	80105892, 80069458	948	7q11.2	Hs.120949 Hs.633085
	17	PB-CD14Monocytes							
	20	BM-CD33Myeloid							
	21	BM-CD105Endothelial							
	23	PB-BDCA4DentriticCells							
	3	LimbMuscle							
	4	ExtraocularMuscle							
	54	FetalLiver							
	76	Adipocytes							
	78	FetalHeart							
10	14	PB-CD8TCells	ALOX5AP	arachidonate 5-lipoxygenase-activating protein	13	30207668	241	13q12	Hs.507658
	15	PB-CD4TCells							
	17	PB-CD14Monocytes							
	18	PB-CD56NKCells							
	19	PB-CD19BCells							
	20	BM-CD33Myeloid							
	23	PB-BDCA4DentriticCells							
	36	WholeBlood							
	42	Lung							
	62	BoneMarrow							
10	14	PB-CD8TCells	PTPRC	protein tyrosine phosphatase, receptor type, C	1	196874847	5788	1q31-q32	Hs.192039
	15	PB-CD4TCells							
	17	PB-CD14Monocytes							
	18	PB-CD56NKCells							
	19	PB-CD19BCells							
	20	BM-CD33Myeloid							
	23	PB-BDCA4DentriticCells							
	35	Tonsil							
	36	WholeBlood							
	45	LymphNode							
10	13	721-BLymphoblasts	TNFAIP8	tumor necrosis factor, alpha-induced protein 8	5	118719628	25816	5q23.1	Hs.271955
	14	PB-CD8TCells							
	15	PB-CD4TCells							
	17	PB-CD14Monocytes							
	18	PB-CD56NKCells							
	19	PB-CD19BCells							
	20	BM-CD33Myeloid							
	23	PB-BDCA4DentriticCells							
	36	WholeBlood							
	78	FetalHeart							
10	13	721-BLymphoblasts	GCH1	GTP cyclohydrolase 1 (dopa-responsive dystonia)	14	-54378475	2643	14q22.1-q22.2	Hs.86724
	14	PB-CD8TCells							
	15	PB-CD4TCells							
	17	PB-CD14Monocytes							
	18	PB-CD56NKCells							
	19	PB-CD19BCells							
	20	BM-CD33Myeloid							
	23	PB-BDCA4DentriticCells							
	36	WholeBlood							
	75	Islet							
10	13	721-BLymphoblasts	CD37	CD37 molecule	19	54530488	951	19q13.3	Hs.166556
	14	PB-CD8TCells							
	15	PB-CD4TCells							
	17	PB-CD14Monocytes							
	18	PB-CD56NKCells							
	19	PB-CD19BCells							

	20	BM-CD33Myeloid							
	23	PB-BDCA4DentriticCells							
	35	Tonsil							
	36	WholeBlood							
10	13	721-BLymphoblasts	DOCK2	dedicator of cytokinesis 2	5	168996870	1794	5q35.1	Hs.586174
	14	PB-CD8TCells							
	15	PB-CD4TCells							
	17	PB-CD14Monocytes							
	18	PB-CD56NKCells							
	19	PB-CD19BCells							
	20	BM-CD33Myeloid							
	22	BM-CD34							
	23	PB-BDCA4DentriticCells							
	36	WholeBlood							
10	13	721-BLymphoblasts	RNF138	ring finger protein 138	18	27925834, 27926644	51444	18q12.1	Hs.302408 Hs.501040
	14	PB-CD8TCells							
	15	PB-CD4TCells							
	17	PB-CD14Monocytes							
	18	PB-CD56NKCells							
	19	PB-CD19BCells							
	20	BM-CD33Myeloid							
	21	BM-CD105Endothelial							
	22	BM-CD34							
	23	PB-BDCA4DentriticCells							
10	12	Placenta	DLK1	delta-like 1 homolog (Drosophila)	14	100262981, 100263005	8788	14q32	Hs.533717
	33	FetalLung							
	39	AdrenalCortex							
	40	Ovary							
	54	FetalLiver							
	55	Testis							
	57	AdrenalGland							
	60	PituitaryGland							
	75	Islet							
	78	FetalHeart							
10	12	Placenta	COL3A1	collagen, type III, alpha 1 (Ehlers-Danlos syndrome type IV, autosomal dominant)	2	189547343	1281	2q31	Hs.443625
	31	UterusCorpus							
	33	FetalLung							
	48	Uterus							
	54	FetalLiver							
	63	CardiacMyocytes							
	64	FetalThyroid							
	72	SmoothMuscle							
	76	Adipocytes							
	78	FetalHeart							
10	12	Placenta	COL6A3	collagen, type VI, alpha 3	2	-237897400	1293	2q37	Hs.233240
	31	UterusCorpus							
	33	FetalLung							
	4	ExtraocularMuscle							
	42	Lung							
	48	Uterus							
	64	FetalThyroid							
	72	SmoothMuscle							
	76	Adipocytes							
	78	FetalHeart							

10	1	Hippocampus	SCG2	secretogranin II (chromogranin C)	2	-224169903	7857	2q35-q36	Hs.516726
	43	FetalBrain							
	46	Amygdala							
	52	CingulateCortex							
	60	PituitaryGland							
	61	Thalamus							
	69	Hypothalamus							
	73	CaudateNucleus							
	74	WholeBrain							
	75	Islet							
10	1	Hippocampus	CADPS	Ca2+-dependent secretion activator	3	-62359061	8618	3p14.2	Hs.127013
	43	FetalBrain							
	46	Amygdala							
	51	PrefrontalCortex							
	6	SubthalamicNucleus							
	60	PituitaryGland							
	61	Thalamus							
	69	Hypothalamus							
	70	OccipitalLobe							
	74	WholeBrain							
10	1	Hippocampus	PTPRZ1	protein tyrosine phosphatase, receptor-type, Z polypeptide 1	7	121300394	5803	7q31.3	Hs.489824
	43	FetalBrain							
	46	Amygdala							
	51	PrefrontalCortex							
	58	SpinalCord							
	61	Thalamus							
	69	Hypothalamus							
	70	OccipitalLobe							
	73	CaudateNucleus							
	74	WholeBrain							
10	1	Hippocampus	GJA1	gap junction protein, alpha 1, 43kDa (connexin 43)	6	121798486	2697	6q21-q23.2	Hs.74471
	41	Thyroid							
	46	Amygdala							
	51	PrefrontalCortex							
	57	AdrenalGland							
	58	SpinalCord							
	65	OlfactoryBulb							
	69	Hypothalamus							
	72	SmoothMuscle							
	78	FetalHeart							
10	1	Hippocampus	ATP1A2	ATPase, Na+/K+ transporting, alpha 2 (+) polypeptide	1	158352171	477	1q21-q23	Hs.34114
	3	LimbMuscle							
	4	ExtraocularMuscle							
	46	Amygdala							
	51	PrefrontalCortex							
	58	SpinalCord							
	69	Hypothalamus							
	70	OccipitalLobe							
	73	CaudateNucleus							
	74	WholeBrain							
10	1	Hippocampus	SH3GL2	SH3-domain GRB2-like 2	9	17569193	6456	9p22	Hs.75149
	29	TemporalLobe							
	46	Amygdala							
	51	PrefrontalCortex							
	52	CingulateCortex							
	59	Cerebellum							

	6	SubthalamicNucleus							
	70	OccipitalLobe							
	71	CerebellumPeduncles							
	74	WholeBrain							
10	1	Hippocampus	KIAA1598	KIAA1598	10	-118634297	57698	10q25.3	Hs.501140
	25	MedullaOblongata							
	43	FetalBrain							
	46	Amygdala							
	51	PrefrontalCortex							
	52	CingulateCortex							
	58	SpinalCord							
	61	Thalamus							
	69	Hypothalamus							
	70	OccipitalLobe							
11	25	MedullaOblongata	SYT1	synaptotagmin I	12	78135413	6857	12cen-q21	Hs.310545
	29	TemporalLobe							
	34	ParietalLobe							
	46	Amygdala							
	52	CingulateCortex							
	6	SubthalamicNucleus							
	69	Hypothalamus							
	70	OccipitalLobe							
	71	CerebellumPeduncles							
	74	WholeBrain							
	8	GlobusPallidus							
11	25	MedullaOblongata	NRGN	neurogranin (protein kinase C substrate, RC3)	11	124114951	4900	11q24	Hs.524116
	29	TemporalLobe							
	34	ParietalLobe							
	46	Amygdala							
	51	PrefrontalCortex							
	52	CingulateCortex							
	6	SubthalamicNucleus							
	70	OccipitalLobe							
	73	CaudateNucleus							
	74	WholeBrain							
	8	GlobusPallidus							
11	25	MedullaOblongata	CCK	cholecystokinin	3	-42274321	885	3p22-p21.3	Hs.458426
	26	Pons							
	29	TemporalLobe							
	34	ParietalLobe							
	46	Amygdala							
	51	PrefrontalCortex							
	52	CingulateCortex							
	6	SubthalamicNucleus							
	70	OccipitalLobe							
	74	WholeBrain							
	8	GlobusPallidus							
11	25	MedullaOblongata	VSNL1	visinin-like 1	2	17585287	7447	2p24.3	Hs.444212
	26	Pons							
	29	TemporalLobe							
	34	ParietalLobe							
	46	Amygdala							
	51	PrefrontalCortex							
	52	CingulateCortex							

	6	SubthalamicNucleus							
	70	OccipitalLobe							
	74	WholeBrain							
	8	GlobusPallidus							
11	17	PB-CD14Monocytes	S100A8	S100 calcium binding protein A8 (calgranulin A)	1	-151629132	6279	1q21	Hs.416073
	20	BM-CD33Myeloid							
	21	BM-CD105Endothelial							
	23	PB-BDCA4DentriticCells							
	30	Tongue							
	33	FetalLung							
	35	Tonsil							
	36	WholeBlood							
	37	HBEC							
	40	Ovary							
	62	BoneMarrow							
11	13	721-BLymphoblasts	COTL1	coactosin-like 1 (Dictyostelium)	16	-83156706	23406	16q24.1	Hs.289092
	14	PB-CD8TCells							
	15	PB-CD4TCells							
	17	PB-CD14Monocytes							
	18	PB-CD56NKCells							
	19	PB-CD19BCells							
	20	BM-CD33Myeloid							
	23	PB-BDCA4DentriticCells							
	36	WholeBlood							
	5	Kidney							
	72	SmoothMuscle							
11	12	Placenta	KRT19	keratin 19	17	-36933395	3880	17q21.2	Hs.2785
	2	BronchialEpitelia							
	33	FetalLung							
	35	Tonsil							
	37	HBEC							
	38	SalivaryGland							
	42	Lung							
	49	Prostate							
	50	Pancreas							
	53	Thymus							
	56	Trachea							
11	12	Placenta	DSP	desmoplakin	6	7486868	1832	6p24	Hs.519873
	2	BronchialEpitelia							
	30	Tongue							
	35	Tonsil							
	37	HBEC							
	38	SalivaryGland							
	41	Thyroid							
	49	Prostate							
	5	Kidney							
	7	Skin							
	78	FetalHeart							
11	12	Placenta	ANXA1	annexin A1	9	74956600	301	9q12-q21.2|9q12-q21.2	Hs.494173
	17	PB-CD14Monocytes							
	2	BronchialEpitelia							
	20	BM-CD33Myeloid							
	30	Tongue							
	35	Tonsil							

	36	WholeBlood							
	37	HBEC							
	63	CardiacMyocytes							
	72	SmoothMuscle							
	76	Adipocytes							
11	1	Hippocampus	TUBB2B	tubulin, beta 2B	6	-3169513	347733	6p25	Hs.300701
	43	FetalBrain							
	46	Amygdala							
	51	PrefrontalCortex							
	58	SpinalCord							
	59	Cerebellum							
	69	Hypothalamus							
	70	OccipitalLobe							
	71	CerebellumPeduncles							
	73	CaudateNucleus							
	74	WholeBrain							
11	1	Hippocampus	GPM6B	glycoprotein M6B	X	-27401000	2824	Xp22.2	Hs.495710
	25	MedullaOblongata							
	34	ParietalLobe							
	46	Amygdala							
	51	PrefrontalCortex							
	58	SpinalCord							
	65	OlfactoryBulb							
	69	Hypothalamus							
	70	OccipitalLobe							
	73	CaudateNucleus							
	74	WholeBrain							
11	1	Hippocampus	GPR37	G protein-coupled receptor 37 (endothelin receptor type B-like)	7	-124173351	2861	7q31	Hs.406094
	25	MedullaOblongata							
	34	ParietalLobe							
	46	Amygdala							
	51	PrefrontalCortex							
	58	SpinalCord							
	61	Thalamus							
	69	Hypothalamus							
	70	OccipitalLobe							
	73	CaudateNucleus							
	74	WholeBrain							
12	17	PB-CD14Monocytes	CSTA	cystatin A (stefin A)	3	123526700	1475	3q21	Hs.518198
	2	BronchialEpitelia							
	20	BM-CD33Myeloid							
	22	BM-CD34							
	23	PB-BDCA4DentriticCells							
	30	Tongue							
	35	Tonsil							
	36	WholeBlood							
	37	HBEC							
	56	Trachea							
	62	BoneMarrow							
	7	Skin							

12	13	721-BLymphoblasts	PLAC8	placenta-specific 8	4	-84230866	51316	4q21.22	Hs.546392
	14	PB-CD8TCells							
	15	PB-CD4TCells							
	17	PB-CD14Monocytes							
	18	PB-CD56NKCells							
	19	PB-CD19BCells							
	2	BronchialEpitelia							
	20	BM-CD33Myeloid							
	22	BM-CD34							
	23	PB-BDCA4DentriticCells							
	36	WholeBlood							
	62	BoneMarrow							
12	12	Placenta	KRT18	keratin 18	12	51629109,	3875	12q13	Hs.406013
	2	BronchialEpitelia				51628921			
	33	FetalLung							
	37	HBEC							
	41	Thyroid							
	42	Lung							
	44	Liver							
	49	Prostate							
	5	Kidney							
	50	Pancreas							
	63	CardiacMyocytes							
	75	Islet							
12	1	Hippocampus	EEF1A2	eukaryotic translation elongation factor	20	-61589809	1917	20q13.3	Hs.433839
	29	TemporalLobe		1 alpha 2					
	3	LimbMuscle							
	32	PsoasMuscle							
	4	ExtraocularMuscle							
	43	FetalBrain							
	46	Amygdala							
	47	Heart							
	51	PrefrontalCortex							
	6	SubthalamicNucleus							
	74	WholeBrain							
	78	FetalHeart							
12	1	Hippocampus	TF	transferrin	3	134947924	7018	3q22.1	Hs.518267
	25	MedullaOblongata							
	33	FetalLung							
	34	ParietalLobe							
	44	Liver							
	51	PrefrontalCortex							
	54	FetalLiver							
	58	SpinalCord							
	61	Thalamus							
	69	Hypothalamus							
	70	OccipitalLobe							
	73	CaudateNucleus							
12	1	Hippocampus	NEFH	neurofilament, heavy polypeptide	22	28206218	4744	22q12.2	Hs.198760
	11	DRG		200kDa					
	25	MedullaOblongata							
	28	TrigeminalGanglion							
	49	Prostate							
	51	PrefrontalCortex							
	52	CingulateCortex							
	58	SpinalCord							

	6	SubthalamicNucleus							
	61	Thalamus							
	69	Hypothalamus							
	9	CiliaryGanglion							
13	12	Placenta	FABP4	fatty acid binding protein 4, adipocyte	8	-82553489	2167	8q21	Hs.391561
	27	Appendix							
	3	LimbMuscle							
	42	Lung							
	45	LymphNode							
	53	Thymus							
	56	Trachea							
	64	FetalThyroid							
	67	TestisIntersitial							
	7	Skin							
	76	Adipocytes							
	78	FetalHeart							
	9	CiliaryGanglion							
13	1	Hippocampus	NAP1L3	nucleosome assembly protein 1-like 3	X	-92812589	4675	Xq21.3-q22	Hs.21365
	25	MedullaOblongata							
	29	TemporalLobe							
	34	ParietalLobe							
	43	FetalBrain							
	46	Amygdala							
	51	PrefrontalCortex							
	59	Cerebellum							
	6	SubthalamicNucleus							
	61	Thalamus							
	69	Hypothalamus							
	70	OccipitalLobe							
	74	WholeBrain							
14	1	Hippocampus	DCAMKL1	doublecortin and CaM kinase-like 1	13	-35243477	9201	13q13	Hs.507755
	25	MedullaOblongata							
	29	TemporalLobe							
	34	ParietalLobe							
	43	FetalBrain							
	46	Amygdala							
	51	PrefrontalCortex							
	52	CingulateCortex							
	6	SubthalamicNucleus							
	69	Hypothalamus							
	70	OccipitalLobe							
	73	CaudateNucleus							
	74	WholeBrain							
	8	GlobusPallidus							
14	1	Hippocampus	DIRAS2	DIRAS family, GTP-binding RAS-like 2	9	-92411933	54769	9q22.2	Hs.165636
	25	MedullaOblongata							
	26	Pons							
	29	TemporalLobe							
	34	ParietalLobe							
	46	Amygdala							
	51	PrefrontalCortex							
	52	CingulateCortex							
	59	Cerebellum							
	6	SubthalamicNucleus							

	70	OccipitalLobe							
	71	CerebellumPeduncles							
	74	WholeBrain							
	8	GlobusPallidus							
14	1	Hippocampus	SPP1	secreted phosphoprotein 1 (osteopontin, bone sialoprotein I, early T-lymphocyte activation 1)	4	89115825	6696	4q21-q25	Hs.313
	12	Placenta							
	25	MedullaOblongata							
	29	TemporalLobe							
	33	FetalLung							
	34	ParietalLobe							
	43	FetalBrain							
	5	Kidney							
	58	SpinalCord							
	61	Thalamus							
	65	OlfactoryBulb							
	69	Hypothalamus							
	70	OccipitalLobe							
	75	Islet							
15	17	PB-CD14Monocytes	LYZ	lysozyme (renal amyloidosis)	12	68028430	4069	12q15	Hs.524579
	18	PB-CD56NKCells							
	2	BronchialEpitelia							
	20	BM-CD33Myeloid							
	21	BM-CD105Endothelial							
	22	BM-CD34							
	23	PB-BDCA4DentriticCells							
	35	Tonsil							
	36	WholeBlood							
	38	SalivaryGland							
	42	Lung							
	45	LymphNode							
	53	Thymus							
	56	Trachea							
	62	BoneMarrow							
15	13	721-BLymphoblasts	HMGB2	high-mobility group box 2	4	-174489362	3148	4q31	Hs.434953
	14	PB-CD8TCells							
	16	BM-CD71EarlyErythroid							
	17	PB-CD14Monocytes							
	18	PB-CD56NKCells							
	19	PB-CD19BCells							
	20	BM-CD33Myeloid							
	21	BM-CD105Endothelial							
	22	BM-CD34							
	23	PB-BDCA4DentriticCells							
	36	WholeBlood							
	53	Thymus							
	54	FetalLiver							
	62	BoneMarrow							
	78	FetalHeart							

15	1	Hippocampus	DNM1	dynamin 1	9	130005483	1759	9q34	Hs.522413
	25	MedullaOblongata							
	29	TemporalLobe							
	34	ParietalLobe							
	46	Amygdala							
	51	PrefrontalCortex							
	52	CingulateCortex							
	59	Cerebellum							
	6	SubthalamicNucleus							
	61	Thalamus							
	69	Hypothalamus							
	70	OccipitalLobe							
	71	CerebellumPeduncles							
	74	WholeBrain							
	8	GlobusPallidus							
15	1	Hippocampus	GRIA2	glutamate receptor, ionotropic, AMPA 2	4	158361296	2891	4q32-q33	Hs.32763
	25	MedullaOblongata							
	26	Pons							
	29	TemporalLobe							
	34	ParietalLobe							
	43	FetalBrain							
	46	Amygdala							
	51	PrefrontalCortex							
	52	CingulateCortex							
	6	SubthalamicNucleus							
	69	Hypothalamus							
	70	OccipitalLobe							
	73	CaudateNucleus							
	74	WholeBrain							
	8	GlobusPallidus							
15	1	Hippocampus	S100B	S100 calcium binding protein, beta (neural)	21	-46842958	6285	21q22.3	Hs.422181
	11	DRG							
	25	MedullaOblongata							
	34	ParietalLobe							
	51	PrefrontalCortex							
	52	CingulateCortex							
	58	SpinalCord							
	59	Cerebellum							
	61	Thalamus							
	65	OlfactoryBulb							
	69	Hypothalamus							
	70	OccipitalLobe							
	71	CerebellumPeduncles							
	73	CaudateNucleus							
	74	WholeBrain							
16	1	Hippocampus	NEF3	neurofilament 3 (150kDa medium)	8	24827211	4741	8p21	Hs.458657 Hs.615287
	11	DRG							
	25	MedullaOblongata							
	29	TemporalLobe							
	34	ParietalLobe							
	46	Amygdala							
	51	PrefrontalCortex							
	52	CingulateCortex							
	58	SpinalCord							
	6	SubthalamicNucleus							
	61	Thalamus							

	69	Hypothalamus							
	70	OccipitalLobe							
	74	WholeBrain							
	8	GlobusPallidus							
	9	CiliaryGanglion							
17	1	Hippocampus	SCG5	secretogranin V (7B2 protein)	15	30721251	6447	15q13-q14	Hs.156540
	25	MedullaOblongata							
	29	TemporalLobe							
	34	ParietalLobe							
	43	FetalBrain							
	46	Amygdala							
	51	PrefrontalCortex							
	52	CingulateCortex							
	59	Cerebellum							
	6	SubthalamicNucleus							
	60	PituitaryGland							
	61	Thalamus							
	69	Hypothalamus							
	70	OccipitalLobe							
	73	CaudateNucleus							
	74	WholeBrain							
	75	Islet							
17	1	Hippocampus	SNAP25	synaptosomal-associated protein, 25kDa	20	10147476	6616	20p12-p11.2	Hs.167317
	25	MedullaOblongata							
	26	Pons							
	29	TemporalLobe							
	34	ParietalLobe							
	46	Amygdala							
	51	PrefrontalCortex							
	52	CingulateCortex							
	59	Cerebellum							
	6	SubthalamicNucleus							
	61	Thalamus							
	69	Hypothalamus							
	70	OccipitalLobe							
	71	CerebellumPeduncles							
	73	CaudateNucleus							
	74	WholeBrain							
	8	GlobusPallidus							
17	1	Hippocampus	MT3	metallothionein 3 (growth inhibitory factor (neurotrophic))	16	55180767	4504	16q13	Hs.73133
	25	MedullaOblongata							
	26	Pons							
	29	TemporalLobe							
	34	ParietalLobe							
	46	Amygdala							
	51	PrefrontalCortex							
	52	CingulateCortex							
	59	Cerebellum							
	6	SubthalamicNucleus							
	61	Thalamus							
	69	Hypothalamus							
	70	OccipitalLobe							

	71	CerebellumPeduncles							
	73	CaudateNucleus							
	74	WholeBrain							
	8	GlobusPallidus							
18	18	PB-CD56NKCells	IGHA1	immunoglobulin heavy constant alpha 1	14		3493	14q32.33	Hs.510635
	19	PB-CD19BCells							
	23	PB-BDCA4DentriticCells							
	27	Appendix							
	35	Tonsil							
	36	WholeBlood							
	38	SalivaryGland							
	41	Thyroid							
	42	Lung							
	45	LymphNode							
	47	Heart							
	49	Prostate							
	5	Kidney							
	50	Pancreas							
	53	Thymus							
	56	Trachea							
	62	BoneMarrow							
	75	Islet							
18	1	Hippocampus	GPM6A	glycoprotein M6A	4	-176791082	2823	4q34	Hs.75819
	25	MedullaOblongata							
	26	Pons							
	29	TemporalLobe							
	34	ParietalLobe							
	43	FetalBrain							
	46	Amygdala							
	51	PrefrontalCortex							
	52	CingulateCortex							
	59	Cerebellum							
	6	SubthalamicNucleus							
	61	Thalamus							
	69	Hypothalamus							
	70	OccipitalLobe							
	71	CerebellumPeduncles							
	73	CaudateNucleus							
	74	WholeBrain							
	8	GlobusPallidus							
18	1	Hippocampus	STMN2	stathmin-like 2	8	80685934	11075	8q21.13	Hs.521651
	11	DRG							
	25	MedullaOblongata							
	26	Pons							
	29	TemporalLobe							
	34	ParietalLobe							
	43	FetalBrain							
	46	Amygdala							
	51	PrefrontalCortex							
	52	CingulateCortex							
	6	SubthalamicNucleus							

	61	Thalamus							
	69	Hypothalamus							
	70	OccipitalLobe							
	71	CerebellumPeduncles							
	74	WholeBrain							
	8	GlobusPallidus							
	9	CiliaryGanglion							
19	1	Hippocampus	RTN1	reticulon 1	14	-59132446	6252	14q23.1	Hs.368626
	25	MedullaOblongata							
	26	Pons							
	29	TemporalLobe							
	34	ParietalLobe							
	43	FetalBrain							
	46	Amygdala							
	51	PrefrontalCortex							
	52	CingulateCortex							
	58	SpinalCord							
	59	Cerebellum							
	6	SubthalamicNucleus							
	61	Thalamus							
	69	Hypothalamus							
	70	OccipitalLobe							
	71	CerebellumPeduncles							
	73	CaudateNucleus							
	74	WholeBrain							
	8	GlobusPallidus							
20	1	Hippocampus	GFAP	glial fibrillary acidic protein	17	-40338518	2670	17q21	Hs.514227
	25	MedullaOblongata							
	26	Pons							
	29	TemporalLobe							
	34	ParietalLobe							
	43	FetalBrain							
	46	Amygdala							
	51	PrefrontalCortex							
	52	CingulateCortex							
	58	SpinalCord							
	59	Cerebellum							
	6	SubthalamicNucleus							
	61	Thalamus							
	65	OlfactoryBulb							
	69	Hypothalamus							
	70	OccipitalLobe							
	71	CerebellumPeduncles							
	73	CaudateNucleus							
	74	WholeBrain							
	8	GlobusPallidus							

20	1	Hippocampus	PLP1	proteolipid protein 1 (Pelizaeus-Merzbacher disease, spastic paraplegia 2, uncomplicated)	X	102918409	5354	Xq22	Hs.1787
	11	DRG							
	25	MedullaOblongata							
	26	Pons							
	29	TemporalLobe							
	34	ParietalLobe							
	46	Amygdala							
	51	PrefrontalCortex							
	52	CingulateCortex							
	58	SpinalCord							
	59	Cerebellum							
	6	SubthalamicNucleus							
	61	Thalamus							
	65	OlfactoryBulb							
	69	Hypothalamus							
	70	OccipitalLobe							
	71	CerebellumPeduncles							
	73	CaudateNucleus							
	74	WholeBrain							
	8	GlobusPallidus							

0.2 GO: Biological Process

GO ID	genes present in chip	genes present in tissues	p-value	GO term
GO:0006952	751	243	3.08352570982558e-40	defense response
GO:0009607	857	263	4.18727033991765e-39	response to biotic stimulus
GO:0050874	1531	389	1.04909554514902e-37	organismal physiological process
GO:0006955	684	221	2.82911301090297e-36	immune response
GO:0043207	463	161	2.67798733091433e-30	response to external biotic stimulus
GO:0009613	458	159	7.9613668614747e-30	response to pest, pathogen or parasite
GO:0009611	341	122	2.96048815311655e-24	response to wounding
GO:0050896	1578	352	8.08452809718242e-22	response to stimulus
GO:0009605	963	238	4.32988928946794e-20	response to external stimulus
GO:0006950	881	220	4.21231322403107e-19	response to stress
GO:0006954	185	69	3.37156836725436e-15	inflammatory response
GO:0006935	101	43	3.40220329124053e-12	chemotaxis
GO:0042330	101	43	3.40220329124053e-12	taxis
GO:0042221	183	62	1.17558954543299e-11	response to chemical substance
GO:0006941	29	20	2.96917703017802e-11	striated muscle contraction
GO:0009617	51	27	8.28316082999464e-11	response to bacteria
GO:0006936	132	48	1.61184434753562e-10	muscle contraction
GO:0051239	133	47	7.59976741325332e-10	regulation of organismal physiological process
GO:0006959	141	48	2.11320165848329e-09	humoral immune response
GO:0048513	872	183	8.57542802998775e-09	organ development
GO:0048609	61	27	1.34123035721390e-08	reproductive organismal physiological process
GO:0042446	25	16	1.55130559139132e-08	hormone biosynthesis
GO:0042742	34	19	1.65876775308862e-08	defense response to bacteria
GO:0050876	62	27	2.05627752329201e-08	reproductive physiological process
GO:0009628	318	82	2.6450053773323e-08	response to abiotic stimulus
GO:0046483	44	21	1.12204520368348e-07	heterocycle metabolism
GO:0006968	87	32	1.34513338371088e-07	cellular defense response
GO:0006937	22	14	1.41399032873128e-07	regulation of muscle contraction
GO:0009887	807	166	1.95061348835026e-07	organogenesis
GO:0042445	46	21	2.90665433742812e-07	hormone metabolism
GO:0016064	103	35	3.35735046030792e-07	humoral defense mechanism (sensu Vertebrata)
GO:0030595	13	10	6.43398540602484e-07	immune cell chemotaxis
GO:0007565	45	20	9.49749803408042e-07	pregnancy
GO:0000003	208	56	1.01742702390029e-06	reproduction
GO:0045087	53	22	1.14454490472344e-06	innate immune response
GO:0007398	79	28	1.92398069779668e-06	ectoderm development
GO:0019835	14	10	1.96268633099188e-06	cytolysis
GO:0050900	14	10	1.96268633099188e-06	immune cell migration
GO:0019730	78	27	4.90608238952994e-06	antimicrobial humoral response
GO:0008544	70	25	5.79210243555387e-06	epidermis development
GO:0030216	18	11	5.81325769219876e-06	keratinocyte differentiation
GO:0006778	18	11	5.81325769219876e-06	porphyrin metabolism

GO:0009056	756	150	7.07315637733239e-06	catabolism
GO:0000302	13	9	9.93922399491655e-06	response to reactive oxygen species
GO:0006779	13	9	9.93922399491655e-06	porphyrin biosynthesis
GO:0042592	157	43	1.13491298862157e-05	homeostasis
GO:0019735	77	26	1.22243005188318e-05	antimicrobial humoral response (sensu Vertebrata)
GO:0009888	158	43	1.34860945296270e-05	histogenesis
GO:0009410	26	13	1.62590375833729e-05	response to xenobiotic stimulus
GO:0006783	11	8	1.86087409066413e-05	heme biosynthesis
GO:0006700	11	8	1.86087409066413e-05	C21-steroid hormone biosynthesis
GO:0008283	473	100	1.97122108627094e-05	cell proliferation
GO:0007586	49	19	1.98657701570558e-05	digestion
GO:0006953	23	12	1.99292634536444e-05	acute-phase response
GO:0008207	14	9	2.42969917899810e-05	C21-steroid hormone metabolism
GO:0009653	1051	195	2.54289973955133e-05	morphogenesis
GO:0008015	103	31	2.58883638820865e-05	circulation
GO:0050776	77	25	3.81478145013865e-05	regulation of immune response
GO:0042743	7	6	5.14217335601547e-05	hydrogen peroxide metabolism
GO:0042744	7	6	5.14217335601547e-05	hydrogen peroxide catabolism
GO:0042168	15	9	5.304330843031e-05	heme metabolism
GO:0030154	409	87	5.45865892861057e-05	cell differentiation
GO:0044248	700	136	5.69895454923246e-05	cellular catabolism
GO:0006800	61	21	5.98683206182187e-05	oxygen and reactive oxygen species metabolism
GO:0008202	132	36	6.33582086337407e-05	steroid metabolism
GO:0006817	57	20	6.56379537958335e-05	phosphate transport
GO:0050878	94	28	7.69674952029793e-05	regulation of body fluids
GO:0006956	30	13	0.000107308934730215	complement activation
GO:0007610	269	61	0.000113444637762813	behavior
GO:0006805	23	11	0.000124054245665878	xenobiotic metabolism
GO:0030005	87	26	0.000129261729180421	di-, tri-valent inorganic cation homeostasis
GO:0006928	205	49	0.000138425775953937	cell motility
GO:0040011	205	49	0.000138425775953937	locomotion
GO:0051241	20	10	0.000159414514745244	negative regulation of organismal physiological process
GO:0006875	93	27	0.000165664159547021	metal ion homeostasis
GO:0006508	451	92	0.000168113621768069	proteolysis and peptidolysis
GO:0044257	451	92	0.000168113621768069	cellular protein catabolism
GO:0030003	98	28	0.000170799303118130	cation homeostasis
GO:0001775	98	28	0.000170799303118130	cell activation
GO:0006873	103	29	0.000174498908138131	cell ion homeostasis
GO:0008210	8	6	0.000180696410487301	estrogen metabolism
GO:0050832	8	6	0.000180696410487301	defense response to fungi
GO:0042542	8	6	0.000180696410487301	response to hydrogen peroxide
GO:0006979	44	16	0.000216497513141638	response to oxidative stress
GO:0007626	209	49	0.000227564764598215	locomotory behavior
GO:0030193	11	7	0.000231187527609989	regulation of blood coagulation
GO:0030195	11	7	0.000231187527609989	negative regulation of blood coagulation
GO:0043285	474	95	0.000255477848588278	biopolymer catabolism

GO:0007275	1543	265	0.000255966910093829	development
GO:0030163	463	93	0.000273682936812054	protein catabolism
GO:0045321	97	27	0.000353105480797321	immune cell activation
GO:0030239	4	4	0.000413181741061206	myofibril assembly
GO:0006702	4	4	0.000413181741061206	androgen biosynthesis
GO:0045445	15	8	0.000425091237246345	myoblast differentiation
GO:0018149	15	8	0.000425091237246345	peptide cross-linking
GO:0006694	60	19	0.000454037458342254	steroid biosynthesis
GO:0007155	518	101	0.000464132564005359	cell adhesion
GO:0007517	94	26	0.000501701695432891	muscle development
GO:0042692	19	9	0.000570628951777488	muscle cell differentiation
GO:0042060	85	24	0.000587572907109038	wound healing
GO:0050801	111	29	0.000686647593088444	ion homeostasis
GO:0019725	122	31	0.000760799759751676	cell homeostasis
GO:0044265	563	107	0.000827671660793537	cellular macromolecule catabolism
GO:0048468	40	14	0.000828780869822101	cell development
GO:0050818	13	7	0.000924560261183667	regulation of coagulation
GO:0050819	13	7	0.000924560261183667	negative regulation of coagulation
GO:0030500	7	5	0.0009600696769149	regulation of bone mineralization
GO:0048627	7	5	0.0009600696769149	myoblast development
GO:0048628	7	5	0.0009600696769149	myoblast maturation
GO:0050920	7	5	0.0009600696769149	regulation of chemotaxis
GO:0007596	78	22	0.000994342843392618	blood coagulation
GO:0007599	83	23	0.00100659951942438	hemostasis
GO:0050817	79	22	0.00119616125345158	coagulation
GO:0009057	575	108	0.00120003072120947	macromolecule catabolism
GO:0030574	17	8	0.00123111399957381	collagen catabolism
GO:0048469	21	9	0.00138817960107146	cell maturation
GO:0045765	25	10	0.00143109462196529	regulation of angiogenesis
GO:0015698	106	27	0.00155844033948856	inorganic anion transport
GO:0006590	5	4	0.00183066788241562	thyroid hormone generation
GO:0050926	5	4	0.00183066788241562	regulation of positive chemotaxis
GO:0050927	5	4	0.00183066788241562	positive regulation of positive chemotaxis
GO:0050918	5	4	0.00183066788241562	positive chemotaxis
GO:0050930	5	4	0.00183066788241562	induction of positive chemotaxis
GO:0030236	5	4	0.00183066788241562	anti-inflammatory response
GO:0030593	5	4	0.00183066788241562	neutrophil chemotaxis
GO:0048519	688	125	0.00184505774843891	negative regulation of biological process
GO:0006957	11	6	0.00202635987555706	complement activation, alternative pathway
GO:0009620	11	6	0.00202635987555706	response to fungi
GO:0016525	11	6	0.00202635987555706	negative regulation of angiogenesis
GO:0001817	39	13	0.00209833663124621	regulation of cytokine production
GO:0042127	265	55	0.0022326466246641	regulation of cell proliferation
GO:0006638	8	5	0.00225902512096090	neutral lipid metabolism
GO:0006639	8	5	0.00225902512096090	acylglycerol metabolism
GO:0030278	8	5	0.00225902512096090	regulation of ossification

GO:0006760	8	5	0.00225902512096090	folic acid and derivative metabolism
GO:0046850	8	5	0.00225902512096090	regulation of bone remodeling
GO:0001568	68	19	0.00241443043332215	blood vessel development
GO:0048514	68	19	0.00241443043332215	blood vessel morphogenesis
GO:0006874	68	19	0.00241443043332215	calcium ion homeostasis
GO:0006879	15	7	0.00267014800582734	iron ion homeostasis
GO:0051240	59	17	0.00281271862279692	positive regulation of organismal physiological process
GO:0001944	69	19	0.00289589412164121	vasculature development
GO:0030502	3	3	0.00290094707623766	negative regulation of bone mineralization
GO:0048246	3	3	0.00290094707623766	macrophage chemotaxis
GO:0000038	3	3	0.00290094707623766	very-long-chain fatty acid metabolism
GO:0050857	3	3	0.00290094707623766	positive regulation of antigen receptor mediated signaling pathway
GO:0050828	3	3	0.00290094707623766	regulation of liquid surface tension
GO:0050680	3	3	0.00290094707623766	negative regulation of epithelial cell proliferation
GO:0030279	3	3	0.00290094707623766	negative regulation of ossification
GO:0046851	3	3	0.00290094707623766	negative regulation of bone remodeling
GO:0042110	45	14	0.00298517735866196	T cell activation
GO:0030333	36	12	0.0030833957082105	antigen processing
GO:0001525	65	18	0.00345351567847031	angiogenesis
GO:0019882	41	13	0.00345892754167412	antigen presentation
GO:0006820	128	30	0.00352882250869354	anion transport
GO:0008285	139	32	0.00355084856791155	negative regulation of cell proliferation
GO:0015669	12	6	0.00356621616958372	gas transport
GO:0015671	12	6	0.00356621616958372	oxygen transport
GO:0019886	12	6	0.00356621616958372	antigen processing, exogenous antigen via MHC class II
GO:0046649	81	21	0.00395411724329393	lymphocyte activation
GO:0050778	56	16	0.00405628515201008	positive regulation of immune response
GO:0042440	24	9	0.0041364002087783	pigment metabolism
GO:0046916	20	8	0.00429169499759	transition metal ion homeostasis
GO:0046148	20	8	0.00429169499759	pigment biosynthesis
GO:0001816	42	13	0.00436796764104566	cytokine production
GO:0030097	87	22	0.00447435370864913	hemopoiesis
GO:0006662	9	5	0.00448728645449726	glycerol ether metabolism
GO:0008209	9	5	0.00448728645449726	androgen metabolism
GO:0042730	9	5	0.00448728645449726	fibrinolysis
GO:0046486	9	5	0.00448728645449726	glycerolipid metabolism
GO:0042035	33	11	0.00454252166730517	regulation of cytokine biosynthesis
GO:0006641	6	4	0.00486993278392384	triacylglycerol metabolism
GO:0042403	6	4	0.00486993278392384	thyroid hormone metabolism
GO:0050921	6	4	0.00486993278392384	positive regulation of chemotaxis
GO:0006703	6	4	0.00486993278392384	estrogen biosynthesis
GO:0001819	29	10	0.00511714288051114	positive regulation of cytokine production
GO:0048534	88	22	0.00518219881986377	hemopoietic or lymphoid organ development
GO:0007051	21	8	0.00607847370313987	spindle organization and biogenesis
GO:0007519	39	12	0.00640305818675338	myogenesis
GO:0006629	469	86	0.00715939213556426	lipid metabolism

GO:0016477	85	21	0.00718470729451301	cell migration
GO:0019748	26	9	0.00757795540274443	secondary metabolism
GO:0030282	10	5	0.00792739795049948	bone mineralization
GO:0009913	14	6	0.00898652102400443	epidermal cell differentiation
GO:0042089	36	11	0.0094065366874035	cytokine biosynthesis
GO:0006942	7	4	0.0100830835932691	regulation of striated muscle contraction
GO:0045884	7	4	0.0100830835932691	regulation of survival gene product activity
GO:0000050	7	4	0.0100830835932691	urea cycle
GO:0019627	7	4	0.0100830835932691	urea metabolism
GO:0050673	7	4	0.0100830835932691	epithelial cell proliferation
GO:0050678	7	4	0.0100830835932691	regulation of epithelial cell proliferation
GO:0048523	640	112	0.0103271325382146	negative regulation of cellular process
GO:0045414	4	3	0.0103642430817670	regulation of interleukin-8 biosynthesis
GO:0045416	4	3	0.0103642430817670	positive regulation of interleukin-8 biosynthesis
GO:0050764	4	3	0.0103642430817670	regulation of phagocytosis
GO:0050766	4	3	0.0103642430817670	positive regulation of phagocytosis
GO:0050854	4	3	0.0103642430817670	regulation of antigen receptor mediated signaling pathway
GO:0045073	4	3	0.0103642430817670	regulation of chemokine biosynthesis
GO:0043118	602	106	0.010417673349353	negative regulation of physiological process
GO:0016042	67	17	0.0112462140504598	lipid catabolism
GO:0006958	23	8	0.0112702973768145	complement activation, classical pathway
GO:0042108	23	8	0.0112702973768145	positive regulation of cytokine biosynthesis
GO:0048232	105	24	0.0116407839870183	male gamete generation
GO:0007283	105	24	0.0116407839870183	spermatogenesis
GO:0042107	37	11	0.0117016679545117	cytokine metabolism
GO:0007076	11	5	0.0128449296796182	mitotic chromosome condensation
GO:0019884	11	5	0.0128449296796182	antigen presentation, exogenous antigen
GO:0008217	28	9	0.0128569748416277	regulation of blood pressure
GO:0044255	383	70	0.0154106460088096	cellular lipid metabolism
GO:0006118	256	49	0.0176871699489603	electron transport
GO:0030317	8	4	0.0179071420655773	sperm motility
GO:0019953	149	31	0.0179955103905100	sexual reproduction
GO:0050777	16	6	0.0186150729362277	negative regulation of immune response
GO:0009618	16	6	0.0186150729362277	response to pathogenic bacteria
GO:0045727	25	8	0.0191568277770546	positive regulation of protein biosynthesis
GO:0042828	25	8	0.0191568277770546	response to pathogen
GO:0031424	12	5	0.0194725821867857	keratinization
GO:0030261	12	5	0.0194725821867857	chromosome condensation
GO:0048015	71	17	0.0198897763119608	phosphoinositide-mediated signaling
GO:0001867	2	2	0.0203539643344414	complement activation, lectin pathway
GO:0046653	2	2	0.0203539643344414	tetrahydrofolate metabolism
GO:0046631	2	2	0.0203539643344414	alpha-beta T cell activation
GO:0001660	2	2	0.0203539643344414	fever
GO:0006922	2	2	0.0203539643344414	cleavage of lamin
GO:0006923	2	2	0.0203539643344414	cleavage of cytoskeletal proteins
GO:0046902	2	2	0.0203539643344414	regulation of mitochondrial membrane permeability

GO:0001907	2	2	0.0203539643344414	killing of host cells
GO:0048247	2	2	0.0203539643344414	lymphocyte chemotaxis
GO:0042055	2	2	0.0203539643344414	neuron lineage restriction
GO:0019836	2	2	0.0203539643344414	hemolysis
GO:0045663	2	2	0.0203539643344414	positive regulation of myoblast differentiation
GO:0050862	2	2	0.0203539643344414	positive regulation of T cell receptor signaling pathway
GO:0050829	2	2	0.0203539643344414	defense response to Gram-negative bacteria
GO:0009186	2	2	0.0203539643344414	deoxyribonucleoside diphosphate metabolism
GO:0009263	2	2	0.0203539643344414	deoxyribonucleotide biosynthesis
GO:0051131	2	2	0.0203539643344414	chaperone-mediated protein complex assembly
GO:0044003	2	2	0.0203539643344414	modification of host morphology or physiology
GO:0044004	2	2	0.0203539643344414	disruption of host cells
GO:0018350	2	2	0.0203539643344414	protein amino acid esterification
GO:0045091	2	2	0.0203539643344414	regulation of retroviral genome replication
GO:0045059	2	2	0.0203539643344414	positive thymic T cell selection
GO:0001897	2	2	0.0203539643344414	cytolysis of host cells
GO:0009891	30	9	0.0204658261647581	positive regulation of biosynthesis
GO:0006730	30	9	0.0204658261647581	one-carbon compound metabolism
GO:0006091	446	79	0.021772428365046	generation of precursor metabolites and energy
GO:0019752	359	65	0.0230051724552028	carboxylic acid metabolism
GO:0006526	5	3	0.0231646058807941	arginine biosynthesis
GO:0006547	5	3	0.0231646058807941	histidine metabolism
GO:0015695	5	3	0.0231646058807941	organic cation transport
GO:0009075	5	3	0.0231646058807941	histidine family amino acid metabolism
GO:0042033	5	3	0.0231646058807941	chemokine biosynthesis
GO:0050755	5	3	0.0231646058807941	chemokine metabolism
GO:0008634	5	3	0.0231646058807941	negative regulation of survival gene product activity
GO:0050727	5	3	0.0231646058807941	regulation of inflammatory response
GO:0042311	5	3	0.0231646058807941	vasodilation
GO:0050852	5	3	0.0231646058807941	T cell receptor signaling pathway
GO:0042228	5	3	0.0231646058807941	interleukin-8 biosynthesis
GO:0007595	5	3	0.0231646058807941	lactation
GO:0020027	5	3	0.0231646058807941	hemoglobin metabolism
GO:0030212	5	3	0.0231646058807941	hyaluronan metabolism
GO:0001894	5	3	0.0231646058807941	tissue homeostasis
GO:0043069	129	27	0.0241858445196478	negative regulation of programmed cell death
GO:0031328	26	8	0.0243029932767481	positive regulation of cellular biosynthesis
GO:0050863	31	9	0.0252920881826220	regulation of T cell activation
GO:0051249	41	11	0.0253120862997670	regulation of lymphocyte activation
GO:0050880	17	6	0.0253738990346138	regulation of blood vessel size
GO:0006082	361	65	0.0256448892186816	organic acid metabolism
GO:0006631	124	26	0.0260507083385926	fatty acid metabolism
GO:0006006	79	18	0.0273359643041807	glucose metabolism
GO:0007276	125	26	0.0285841808598936	gametogenesis
GO:0050714	9	4	0.0286430265544413	positive regulation of protein secretion
GO:0006706	9	4	0.0286430265544413	steroid catabolism

GO:0016066	27	8	0.0303434536751575	cellular defense response (sensu Vertebrata)
GO:0030099	37	10	0.030678488258746	myeloid cell differentiation
GO:0009123	18	6	0.0335862038754002	nucleoside monophosphate metabolism
GO:0009124	18	6	0.0335862038754002	nucleoside monophosphate biosynthesis
GO:0008284	121	25	0.0337686786590884	positive regulation of cell proliferation
GO:0050865	43	11	0.0353121250664378	regulation of cell activation
GO:0008016	23	7	0.0363363190565484	regulation of heart contraction rate
GO:0043066	128	26	0.0373206639324748	negative regulation of apoptosis
GO:0006826	14	5	0.0385613143147214	iron ion transport
GO:0001666	14	5	0.0385613143147214	response to hypoxia
GO:0007067	135	27	0.040878388309922	mitosis
GO:0050851	6	3	0.0414592789776643	antigen receptor-mediated signaling pathway
GO:0031032	6	3	0.0414592789776643	actomyosin structure organization and biogenesis
GO:0045058	6	3	0.0414592789776643	T cell selection
GO:0006525	10	4	0.0424534456237359	arginine metabolism
GO:0051262	10	4	0.0424534456237359	protein tetramerization
GO:0006916	112	23	0.0430728009448884	anti-apoptosis
GO:0009968	50	12	0.0451972816839005	negative regulation of signal transduction
GO:0001501	113	23	0.0470641687971318	skeletal development
GO:0007229	45	11	0.0478037897769273	integrin-mediated signaling pathway
GO:0000087	137	27	0.0480060229037731	M phase of mitotic cell cycle
GO:0051186	143	28	0.0480188668158537	cofactor metabolism
GO:0006725	73	16	0.0496527742620921	aromatic compound metabolism
GO:0006801	15	5	0.0512432532873543	superoxide metabolism
GO:0006909	15	5	0.0512432532873543	phagocytosis
GO:0000070	20	6	0.0546899857545854	mitotic sister chromatid segregation
GO:0015889	3	2	0.0552599988508488	cobalamin transport
GO:0001659	3	2	0.0552599988508488	thermoregulation
GO:0043114	3	2	0.0552599988508488	regulation of vascular permeability
GO:0008105	3	2	0.0552599988508488	asymmetric protein localization
GO:0019731	3	2	0.0552599988508488	antibacterial humoral response
GO:0048599	3	2	0.0552599988508488	oocyte development
GO:0007320	3	2	0.0552599988508488	insemination
GO:0050856	3	2	0.0552599988508488	regulation of T cell receptor signaling pathway
GO:0045995	3	2	0.0552599988508488	regulation of embryonic development
GO:0042447	3	2	0.0552599988508488	hormone catabolism
GO:0009132	3	2	0.0552599988508488	nucleoside diphosphate metabolism
GO:0009267	3	2	0.0552599988508488	cellular response to starvation
GO:0042119	3	2	0.0552599988508488	neutrophil activation
GO:0009743	3	2	0.0552599988508488	response to carbohydrate stimulus
GO:0016127	3	2	0.0552599988508488	sterol catabolism
GO:0042771	3	2	0.0552599988508488	DNA damage response, signal transduction by p53 class mediator resulting in induction of apoptosis
GO:0042531	3	2	0.0552599988508488	positive regulation of tyrosine phosphorylation of STAT protein
GO:0042533	3	2	0.0552599988508488	tumor necrosis factor-alpha biosynthesis
GO:0042534	3	2	0.0552599988508488	regulation of tumor necrosis factor-alpha biosynthesis

GO:0042535	3	2	0.0552599988508488	positive regulation of tumor necrosis factor-alpha biosynthesis
GO:0046427	3	2	0.0552599988508488	positive regulation of JAK-STAT cascade
GO:0030157	3	2	0.0552599988508488	pancreatic juice secretion
GO:0009994	3	2	0.0552599988508488	oocyte differentiation
GO:0030146	3	2	0.0552599988508488	diuresis
GO:0030147	3	2	0.0552599988508488	natriuresis
GO:0030104	3	2	0.0552599988508488	water homeostasis
GO:0006104	3	2	0.0552599988508488	succinyl-CoA metabolism
GO:0045090	3	2	0.0552599988508488	retroviral genome replication
GO:0045061	3	2	0.0552599988508488	thymic T cell selection
GO:0006707	3	2	0.0552599988508488	cholesterol catabolism
GO:0045079	3	2	0.0552599988508488	negative regulation of chemokine biosynthesis
GO:0051247	41	10	0.0583789261336097	positive regulation of protein metabolism
GO:0000051	11	4	0.0593725975918032	urea cycle intermediate metabolism
GO:0009167	11	4	0.0593725975918032	purine ribonucleoside monophosphate metabolism
GO:0009168	11	4	0.0593725975918032	purine ribonucleoside monophosphate biosynthesis
GO:0009126	11	4	0.0593725975918032	purine nucleoside monophosphate metabolism
GO:0009127	11	4	0.0593725975918032	purine nucleoside monophosphate biosynthesis
GO:0009262	7	3	0.0649914255159607	deoxyribonucleotide metabolism
GO:0007286	7	3	0.0649914255159607	spermatid development
GO:0007516	7	3	0.0649914255159607	hemocyte development
GO:0018298	7	3	0.0649914255159607	protein-chromophore linkage
GO:0007157	16	5	0.0660742443560482	heterophilic cell adhesion
GO:0008632	53	12	0.0666600986034258	apoptotic program
GO:0009306	26	7	0.066736991988195	protein secretion
GO:0009566	26	7	0.066736991988195	fertilization
GO:0007507	26	7	0.066736991988195	heart development
GO:0007267	465	78	0.066931252252361	cell-cell signaling
GO:0019221	21	6	0.0676704098915909	cytokine and chemokine mediated signaling pathway
GO:0019883	21	6	0.0676704098915909	antigen presentation, endogenous antigen
GO:0050870	21	6	0.0676704098915909	positive regulation of T cell activation
GO:0000270	21	6	0.0676704098915909	peptidoglycan metabolism
GO:0000819	21	6	0.0676704098915909	sister chromatid segregation
GO:0051243	583	96	0.0680179836150592	negative regulation of cellular physiological process
GO:0006092	88	18	0.0699293870573447	main pathways of carbohydrate metabolism
GO:0016265	501	83	0.0760918161674429	death
GO:0050708	12	4	0.0793226052943119	regulation of protein secretion
GO:0009112	12	4	0.0793226052943119	nucleobase metabolism
GO:0051180	12	4	0.0793226052943119	vitamin transport
GO:0019885	22	6	0.0822814899073102	antigen processing, endogenous antigen via MHC class I
GO:0050670	22	6	0.0822814899073102	regulation of lymphocyte proliferation
GO:0006066	213	38	0.0824445944547838	alcohol metabolism
GO:0017148	17	5	0.0830327215733126	negative regulation of protein biosynthesis
GO:0009161	17	5	0.0830327215733126	ribonucleoside monophosphate metabolism
GO:0009156	17	5	0.0830327215733126	ribonucleoside monophosphate biosynthesis
GO:0006094	17	5	0.0830327215733126	gluconeogenesis

GO:0008219	498	82	0.0864234741702037	cell death
GO:0030155	33	8	0.0880143911635706	regulation of cell adhesion
GO:0006558	8	3	0.093241995586191	L-phenylalanine metabolism
GO:0006559	8	3	0.093241995586191	L-phenylalanine catabolism
GO:0001541	8	3	0.093241995586191	ovarian follicle development
GO:0001906	8	3	0.093241995586191	cell killing
GO:0045807	8	3	0.093241995586191	positive regulation of endocytosis
GO:0048515	8	3	0.093241995586191	spermatid differentiation
GO:0042386	8	3	0.093241995586191	hemocyte differentiation (sensu Arthropoda)
GO:0009791	8	3	0.093241995586191	post-embryonic development
GO:0051238	8	3	0.093241995586191	sequestering of metal ion
GO:0007567	8	3	0.093241995586191	parturition
GO:0007568	8	3	0.093241995586191	aging
GO:0007589	8	3	0.093241995586191	fluid secretion
GO:0042698	8	3	0.093241995586191	menstrual cycle
GO:0046112	8	3	0.093241995586191	nucleobase biosynthesis
GO:0006206	8	3	0.093241995586191	pyrimidine base metabolism
GO:0006749	8	3	0.093241995586191	glutathione metabolism
GO:0045078	8	3	0.093241995586191	positive regulation of interferon-gamma biosynthesis
GO:0046651	28	7	0.0935202921274876	lymphocyte proliferation
GO:0051187	28	7	0.0935202921274876	cofactor catabolism
GO:0051251	28	7	0.0935202921274876	positive regulation of lymphocyte activation
GO:0045410	4	2	0.100155754619931	positive regulation of interleukin-6 biosynthesis
GO:0045408	4	2	0.100155754619931	regulation of interleukin-6 biosynthesis
GO:0048168	4	2	0.100155754619931	regulation of neuronal synaptic plasticity
GO:0006940	4	2	0.100155754619931	regulation of smooth muscle contraction
GO:0035036	4	2	0.100155754619931	sperm-egg recognition
GO:0007094	4	2	0.100155754619931	mitotic spindle checkpoint
GO:0045661	4	2	0.100155754619931	regulation of myoblast differentiation
GO:0007339	4	2	0.100155754619931	binding of sperm to zona pellucida
GO:0042312	4	2	0.100155754619931	regulation of vasodilation
GO:0045946	4	2	0.100155754619931	positive regulation of translation
GO:0050672	4	2	0.100155754619931	negative regulation of lymphocyte proliferation
GO:0042226	4	2	0.100155754619931	interleukin-6 biosynthesis
GO:0051282	4	2	0.100155754619931	regulation of sequestering of calcium ion
GO:0051283	4	2	0.100155754619931	negative regulation of sequestering of calcium ion
GO:0051289	4	2	0.100155754619931	protein homotetramerization
GO:0042594	4	2	0.100155754619931	response to starvation
GO:0051209	4	2	0.100155754619931	release of sequestered calcium ion into cytoplasm
GO:0042554	4	2	0.100155754619931	superoxide release
GO:0042541	4	2	0.100155754619931	hemoglobin biosynthesis
GO:0045089	4	2	0.100155754619931	positive regulation of innate immune response
GO:0007059	34	8	0.101409741485362	chromosome segregation
GO:0031327	18	5	0.102050612995586	negative regulation of cellular biosynthesis
GO:0006090	18	5	0.102050612995586	pyruvate metabolism
GO:0030217	18	5	0.102050612995586	T cell differentiation

GO:0007093	13	4	0.102133146252022	mitotic checkpoint
GO:0051047	13	4	0.102133146252022	positive regulation of secretion
GO:0046058	13	4	0.102133146252022	cAMP metabolism
GO:0006081	13	4	0.102133146252022	aldehyde metabolism
GO:0030218	13	4	0.102133146252022	erythrocyte differentiation
GO:0043067	308	52	0.107230679669138	regulation of programmed cell death
GO:0050867	29	7	0.108851231785393	positive regulation of cell activation
GO:0043062	35	8	0.115876516928179	extracellular structure organization and biogenesis
GO:0006576	35	8	0.115876516928179	biogenic amine metabolism
GO:0030198	35	8	0.115876516928179	extracellular matrix organization and biogenesis
GO:0051046	24	6	0.116266950558511	regulation of secretion
GO:0043119	428	70	0.117762643006840	positive regulation of physiological process
GO:0006575	41	9	0.120637585508267	amino acid derivative metabolism
GO:0046849	41	9	0.120637585508267	bone remodeling
GO:0015980	132	24	0.122942626463370	energy derivation by oxidation of organic compounds
GO:0009890	19	5	0.123019181036532	negative regulation of biosynthesis
GO:0001818	9	3	0.125541480102066	negative regulation of cytokine production
GO:0046545	9	3	0.125541480102066	development of primary female sexual characteristics
GO:0046660	9	3	0.125541480102066	female sex differentiation
GO:0008272	9	3	0.125541480102066	sulfate transport
GO:0045766	9	3	0.125541480102066	positive regulation of angiogenesis
GO:0042036	9	3	0.125541480102066	negative regulation of cytokine biosynthesis
GO:0008585	9	3	0.125541480102066	female gonad development
GO:0007281	9	3	0.125541480102066	germ cell development
GO:0030178	9	3	0.125541480102066	negative regulation of Wnt receptor signaling pathway
GO:0006268	9	3	0.125541480102066	DNA unwinding
GO:0007052	14	4	0.127561879026941	mitotic spindle organization and biogenesis
GO:0042981	306	51	0.129005045803749	regulation of apoptosis
GO:0042087	25	6	0.135515738985332	cell-mediated immune response
GO:0007338	25	6	0.135515738985332	fertilization (sensu Metazoa)
GO:0016337	172	30	0.138562567902585	cell-cell adhesion
GO:0016125	66	13	0.139155578895513	sterol metabolism
GO:0008203	60	12	0.139262773525657	cholesterol metabolism
GO:0001881	1	1	0.142714791851196	receptor recycling
GO:0048003	1	1	0.142714791851196	antigen presentation, lipid antigen
GO:0048006	1	1	0.142714791851196	antigen presentation, endogenous lipid antigen
GO:0015742	1	1	0.142714791851196	alpha-ketoglutarate transport
GO:0046541	1	1	0.142714791851196	saliva secretion
GO:0006573	1	1	0.142714791851196	valine metabolism
GO:0031342	1	1	0.142714791851196	negative regulation of cell killing
GO:0001556	1	1	0.142714791851196	oocyte maturation
GO:0031335	1	1	0.142714791851196	regulation of sulfur amino acid metabolism
GO:0015855	1	1	0.142714791851196	pyrimidine transport
GO:0015886	1	1	0.142714791851196	heme transport
GO:0015888	1	1	0.142714791851196	thiamin transport
GO:0001523	1	1	0.142714791851196	retinoid metabolism

GO:0046689	1	1	0.142714791851196	response to mercury ion
GO:0006651	1	1	0.142714791851196	diacylglycerol biosynthesis
GO:0046640	1	1	0.142714791851196	regulation of alpha-beta T cell proliferation
GO:0046641	1	1	0.142714791851196	positive regulation of alpha-beta T cell proliferation
GO:0046633	1	1	0.142714791851196	alpha-beta T cell proliferation
GO:0046634	1	1	0.142714791851196	regulation of alpha-beta T cell activation
GO:0046635	1	1	0.142714791851196	positive regulation of alpha-beta T cell activation
GO:0045329	1	1	0.142714791851196	carnitine biosynthesis
GO:0015696	1	1	0.142714791851196	ammonium transport
GO:0048305	1	1	0.142714791851196	immunoglobulin secretion
GO:0045415	1	1	0.142714791851196	negative regulation of interleukin-8 biosynthesis
GO:0019371	1	1	0.142714791851196	cyclooxygenase pathway
GO:0045175	1	1	0.142714791851196	basal protein localization
GO:0045176	1	1	0.142714791851196	apical protein localization
GO:0046984	1	1	0.142714791851196	regulation of hemoglobin biosynthesis
GO:0046986	1	1	0.142714791851196	negative regulation of hemoglobin biosynthesis
GO:0048160	1	1	0.142714791851196	primary follicle stage oogenesis
GO:0048151	1	1	0.142714791851196	hyperphosphorylation
GO:0006982	1	1	0.142714791851196	response to lipid hydroperoxide
GO:0006926	1	1	0.142714791851196	programmed cell death, virus-infected cells
GO:0019430	1	1	0.142714791851196	removal of superoxide radicals
GO:0048143	1	1	0.142714791851196	astrocyte activation
GO:0043152	1	1	0.142714791851196	induction of bacterial agglutination
GO:0006931	1	1	0.142714791851196	substrate-bound cell migration, cell attachment to substrate
GO:0001952	1	1	0.142714791851196	regulation of cell-matrix adhesion
GO:0001953	1	1	0.142714791851196	negative regulation of cell-matrix adhesion
GO:0006938	1	1	0.142714791851196	sarcomere alignment
GO:0043129	1	1	0.142714791851196	surfactant homeostasis
GO:0001920	1	1	0.142714791851196	negative regulation of receptor recycling
GO:0006907	1	1	0.142714791851196	pinocytosis
GO:0001911	1	1	0.142714791851196	negative regulation of immune cell mediated cytotoxicity
GO:0001913	1	1	0.142714791851196	T cell mediated cytotoxicity
GO:0001914	1	1	0.142714791851196	regulation of T cell mediated cytotoxicity
GO:0001915	1	1	0.142714791851196	negative regulation of T cell mediated cytotoxicity
GO:0001919	1	1	0.142714791851196	regulation of receptor recycling
GO:0035041	1	1	0.142714791851196	sperm chromatin decondensation
GO:0035042	1	1	0.142714791851196	fertilization, exchange of chromosomal proteins
GO:0045214	1	1	0.142714791851196	sarcomere organization
GO:0035039	1	1	0.142714791851196	male pronucleus formation
GO:0048286	1	1	0.142714791851196	alveolus development
GO:0048245	1	1	0.142714791851196	eosinophil chemotaxis
GO:0045740	1	1	0.142714791851196	positive regulation of DNA replication
GO:0045741	1	1	0.142714791851196	positive regulation of epidermal growth factor receptor activity
GO:0018987	1	1	0.142714791851196	osmoregulation
GO:0043206	1	1	0.142714791851196	fibril organization and biogenesis
GO:0009052	1	1	0.142714791851196	pentose-phosphate shunt, non-oxidative branch

GO:0009076	1	1	0.142714791851196	histidine family amino acid biosynthesis
GO:0045869	1	1	0.142714791851196	negative regulation of retroviral genome replication
GO:0045821	1	1	0.142714791851196	positive regulation of glycolysis
GO:0045844	1	1	0.142714791851196	positive regulation of myogenesis
GO:0045837	1	1	0.142714791851196	negative regulation of membrane potential
GO:0019724	1	1	0.142714791851196	B cell mediated immunity
GO:0007039	1	1	0.142714791851196	vacuolar protein catabolism
GO:0045579	1	1	0.142714791851196	positive regulation of B cell differentiation
GO:0019860	1	1	0.142714791851196	uracil metabolism
GO:0019859	1	1	0.142714791851196	thymine metabolism
GO:0045653	1	1	0.142714791851196	negative regulation of megakaryocyte differentiation
GO:0045647	1	1	0.142714791851196	negative regulation of erythrocyte differentiation
GO:0009397	1	1	0.142714791851196	folic acid and derivative catabolism
GO:0045603	1	1	0.142714791851196	positive regulation of endothelial cell differentiation
GO:0051095	1	1	0.142714791851196	regulation of helicase activity
GO:0051097	1	1	0.142714791851196	negative regulation of helicase activity
GO:0009386	1	1	0.142714791851196	translational attenuation
GO:0050751	1	1	0.142714791851196	fractalkine biosynthesis
GO:0050752	1	1	0.142714791851196	regulation of fractalkine biosynthesis
GO:0050754	1	1	0.142714791851196	positive regulation of fractalkine biosynthesis
GO:0050756	1	1	0.142714791851196	fractalkine metabolism
GO:0050720	1	1	0.142714791851196	interleukin-1 beta biosynthesis
GO:0050722	1	1	0.142714791851196	regulation of interleukin-1 beta biosynthesis
GO:0050725	1	1	0.142714791851196	positive regulation of interleukin-1 beta biosynthesis
GO:0050728	1	1	0.142714791851196	negative regulation of inflammatory response
GO:0050746	1	1	0.142714791851196	regulation of lipoprotein metabolism
GO:0050748	1	1	0.142714791851196	negative regulation of lipoprotein metabolism
GO:0051000	1	1	0.142714791851196	positive regulation of nitric-oxide synthase activity
GO:0050891	1	1	0.142714791851196	body fluid osmoregulation
GO:0050850	1	1	0.142714791851196	positive regulation of calcium-mediated signaling
GO:0050853	1	1	0.142714791851196	B cell receptor signaling pathway
GO:0009437	1	1	0.142714791851196	carnitine metabolism
GO:0007499	1	1	0.142714791851196	ectoderm and mesoderm interaction
GO:0050848	1	1	0.142714791851196	regulation of calcium-mediated signaling
GO:0042494	1	1	0.142714791851196	detection of bacterial lipoprotein
GO:0042495	1	1	0.142714791851196	detection of triacylated bacterial lipoprotein
GO:0009162	1	1	0.142714791851196	deoxyribonucleoside monophosphate metabolism
GO:0007423	1	1	0.142714791851196	sensory organ development
GO:0009151	1	1	0.142714791851196	purine deoxyribonucleotide metabolism
GO:0009153	1	1	0.142714791851196	purine deoxyribonucleotide biosynthesis
GO:0009157	1	1	0.142714791851196	deoxyribonucleoside monophosphate biosynthesis
GO:0042471	1	1	0.142714791851196	ear morphogenesis
GO:0042472	1	1	0.142714791851196	inner ear morphogenesis
GO:0009180	1	1	0.142714791851196	purine ribonucleoside diphosphate biosynthesis
GO:0009182	1	1	0.142714791851196	purine deoxyribonucleoside diphosphate metabolism
GO:0009183	1	1	0.142714791851196	purine deoxyribonucleoside diphosphate biosynthesis

GO:0009188	1	1	0.142714791851196	ribonucleoside diphosphate biosynthesis
GO:0009189	1	1	0.142714791851196	deoxyribonucleoside diphosphate biosynthesis
GO:0045993	1	1	0.142714791851196	negative regulation of translational initiation by iron
GO:0009176	1	1	0.142714791851196	pyrimidine deoxyribonucleoside monophosphate metabolism
GO:0009177	1	1	0.142714791851196	pyrimidine deoxyribonucleoside monophosphate biosynthesis
GO:0009179	1	1	0.142714791851196	purine ribonucleoside diphosphate metabolism
GO:0009118	1	1	0.142714791851196	regulation of nucleoside metabolism
GO:0009133	1	1	0.142714791851196	nucleoside diphosphate biosynthesis
GO:0009135	1	1	0.142714791851196	purine nucleoside diphosphate metabolism
GO:0009136	1	1	0.142714791851196	purine nucleoside diphosphate biosynthesis
GO:0000303	1	1	0.142714791851196	response to superoxide
GO:0045978	1	1	0.142714791851196	negative regulation of nucleoside metabolism
GO:0045917	1	1	0.142714791851196	positive regulation of complement activation
GO:0005982	1	1	0.142714791851196	starch metabolism
GO:0005983	1	1	0.142714791851196	starch catabolism
GO:0045948	1	1	0.142714791851196	positive regulation of translational initiation
GO:0045908	1	1	0.142714791851196	negative regulation of vasodilation
GO:0009256	1	1	0.142714791851196	10-formyltetrahydrofolate metabolism
GO:0009258	1	1	0.142714791851196	10-formyltetrahydrofolate catabolism
GO:0007141	1	1	0.142714791851196	male meiosis I
GO:0007143	1	1	0.142714791851196	female meiosis
GO:0009221	1	1	0.142714791851196	pyrimidine deoxyribonucleotide biosynthesis
GO:0042117	1	1	0.142714791851196	monocyte activation
GO:0050666	1	1	0.142714791851196	regulation of homocysteine metabolism
GO:0050667	1	1	0.142714791851196	homocysteine metabolism
GO:0051353	1	1	0.142714791851196	positive regulation of oxidoreductase activity
GO:0007108	1	1	0.142714791851196	cytokinesis, initiation of separation
GO:0007290	1	1	0.142714791851196	spermatid nuclear elongation
GO:0007289	1	1	0.142714791851196	spermatid nuclear differentiation
GO:0042276	1	1	0.142714791851196	error-prone postreplication DNA repair
GO:0000114	1	1	0.142714791851196	G1-specific transcription in mitotic cell cycle
GO:0000105	1	1	0.142714791851196	histidine biosynthesis
GO:0000212	1	1	0.142714791851196	meiotic spindle organization and biogenesis
GO:0016264	1	1	0.142714791851196	gap junction assembly
GO:0046056	1	1	0.142714791851196	dADP metabolism
GO:0046073	1	1	0.142714791851196	dTMP metabolism
GO:0042700	1	1	0.142714791851196	luteinizing hormone signaling pathway
GO:0046031	1	1	0.142714791851196	ADP metabolism
GO:0051103	1	1	0.142714791851196	DNA ligation during DNA repair
GO:0050994	1	1	0.142714791851196	regulation of lipid catabolism
GO:0050995	1	1	0.142714791851196	negative regulation of lipid catabolism
GO:0000738	1	1	0.142714791851196	DNA catabolism, exonucleolytic
GO:0050922	1	1	0.142714791851196	negative regulation of chemotaxis
GO:0007525	1	1	0.142714791851196	somatic muscle development
GO:0042640	1	1	0.142714791851196	anagen
GO:0030449	1	1	0.142714791851196	regulation of complement activation

GO:0001315	1	1	0.142714791851196	age-dependent response to reactive oxygen species
GO:0006447	1	1	0.142714791851196	regulation of translational initiation by iron
GO:0044416	1	1	0.142714791851196	induction of host defense response
GO:0009935	1	1	0.142714791851196	nutrient import
GO:0046121	1	1	0.142714791851196	deoxyribonucleoside catabolism
GO:0046127	1	1	0.142714791851196	pyrimidine deoxyribonucleoside catabolism
GO:0046113	1	1	0.142714791851196	nucleobase catabolism
GO:0006172	1	1	0.142714791851196	ADP biosynthesis
GO:0006173	1	1	0.142714791851196	dADP biosynthesis
GO:0030240	1	1	0.142714791851196	muscle thin filament assembly
GO:0046104	1	1	0.142714791851196	thymidine metabolism
GO:0006280	1	1	0.142714791851196	mutagenesis
GO:0006288	1	1	0.142714791851196	base-excision repair, DNA ligation
GO:0030728	1	1	0.142714791851196	ovulation
GO:0006212	1	1	0.142714791851196	uracil catabolism
GO:0006214	1	1	0.142714791851196	thymidine catabolism
GO:0006231	1	1	0.142714791851196	dTMP biosynthesis
GO:0000920	1	1	0.142714791851196	cell separation during cytokinesis
GO:0044258	1	1	0.142714791851196	intestinal lipid catabolism
GO:0006208	1	1	0.142714791851196	pyrimidine base catabolism
GO:0000912	1	1	0.142714791851196	cytokinesis, formation of actomyosin apparatus
GO:0000915	1	1	0.142714791851196	cytokinesis, contractile ring formation
GO:0044240	1	1	0.142714791851196	organismal lipid catabolism
GO:0044243	1	1	0.142714791851196	organismal catabolism
GO:0018184	1	1	0.142714791851196	protein amino acid polyamination
GO:0044236	1	1	0.142714791851196	organismal metabolism
GO:0006781	1	1	0.142714791851196	succinyl-CoA pathway
GO:0006789	1	1	0.142714791851196	bilirubin conjugation
GO:0046730	1	1	0.142714791851196	viral induction of host immune response
GO:0046731	1	1	0.142714791851196	passive viral induction of host immune response
GO:0006710	1	1	0.142714791851196	androgen catabolism
GO:0006711	1	1	0.142714791851196	estrogen catabolism
GO:0006741	1	1	0.142714791851196	NADP biosynthesis
GO:0001780	1	1	0.142714791851196	neutrophil homeostasis
GO:0001781	1	1	0.142714791851196	programmed cell death, neutrophils
GO:0001774	1	1	0.142714791851196	microglial cell activation
GO:0006701	1	1	0.142714791851196	progesterone biosynthesis
GO:0045080	1	1	0.142714791851196	positive regulation of chemokine biosynthesis
GO:0045085	1	1	0.142714791851196	negative regulation of interleukin-2 biosynthesis
GO:0045020	1	1	0.142714791851196	error-prone DNA repair
GO:0018277	1	1	0.142714791851196	protein amino acid deamination
GO:0006855	1	1	0.142714791851196	multidrug transport
GO:0006882	1	1	0.142714791851196	zinc ion homeostasis
GO:0001890	1	1	0.142714791851196	placenta development
GO:0007204	37	8	0.147896014126051	elevation of cytoplasmic calcium ion concentration
GO:0051480	37	8	0.147896014126051	cytoplasmic calcium ion homeostasis

GO:0019098	5	2	0.151483187112688	reproductive behavior
GO:0006527	5	2	0.151483187112688	arginine catabolism
GO:0006534	5	2	0.151483187112688	cysteine metabolism
GO:0050000	5	2	0.151483187112688	chromosome localization
GO:0048477	5	2	0.151483187112688	oogenesis
GO:0001936	5	2	0.151483187112688	regulation of endothelial cell proliferation
GO:0045840	5	2	0.151483187112688	positive regulation of mitosis
GO:0019856	5	2	0.151483187112688	pyrimidine base biosynthesis
GO:0009396	5	2	0.151483187112688	folic acid and derivative biosynthesis
GO:0016358	5	2	0.151483187112688	dendrite morphogenesis
GO:0051303	5	2	0.151483187112688	establishment of chromosome localization
GO:0007569	5	2	0.151483187112688	cell aging
GO:0051208	5	2	0.151483187112688	sequestering of calcium ion
GO:0030323	5	2	0.151483187112688	respiratory tube development
GO:0007617	5	2	0.151483187112688	mating behavior
GO:0007638	5	2	0.151483187112688	mechanosensory behavior
GO:0006750	5	2	0.151483187112688	glutathione biosynthesis
GO:0006787	5	2	0.151483187112688	porphyrin catabolism
GO:0045069	5	2	0.151483187112688	regulation of viral genome replication
GO:0045088	5	2	0.151483187112688	regulation of innate immune response
GO:0045595	55	11	0.152445160486849	regulation of cell differentiation
GO:0006635	15	4	0.155314106568610	fatty acid beta-oxidation
GO:0051050	15	4	0.155314106568610	positive regulation of transport
GO:0042129	15	4	0.155314106568610	regulation of T cell proliferation
GO:0007585	15	4	0.155314106568610	respiratory gaseous exchange
GO:0009084	10	3	0.161150637735636	glutamine family amino acid biosynthesis
GO:0009074	10	3	0.161150637735636	aromatic amino acid family catabolism
GO:0045580	10	3	0.161150637735636	regulation of T cell differentiation
GO:0005978	10	3	0.161150637735636	glycogen biosynthesis
GO:0009250	10	3	0.161150637735636	glucan biosynthesis
GO:0007260	10	3	0.161150637735636	tyrosine phosphorylation of STAT protein
GO:0051242	411	66	0.161420852928281	positive regulation of cellular physiological process
GO:0001503	38	8	0.165359308485158	ossification
GO:0031214	38	8	0.165359308485158	biomineral formation
GO:0007218	56	11	0.166763420658999	neuropeptide signaling pathway
GO:0007166	952	146	0.172319934794659	cell surface receptor linked signal transduction
GO:0006690	33	7	0.182043063196173	icosanoid metabolism
GO:0007173	16	4	0.185060727306130	epidermal growth factor receptor signaling pathway
GO:0030100	16	4	0.185060727306130	regulation of endocytosis
GO:0000278	204	34	0.185839854241759	mitotic cell cycle
GO:0006260	140	24	0.193482910759977	DNA replication
GO:0019319	22	5	0.196070931733963	hexose biosynthesis
GO:0046364	22	5	0.196070931733963	monosaccharide biosynthesis
GO:0046165	22	5	0.196070931733963	alcohol biosynthesis
GO:0009615	58	11	0.197260715621651	response to virus
GO:0006921	11	3	0.199317402789573	disassembly of cell structures during apoptosis

GO:0051260	11	3	0.199317402789573	protein homooligomerization
GO:0030855	11	3	0.199317402789573	epithelial cell differentiation
GO:0045072	11	3	0.199317402789573	regulation of interferon-gamma biosynthesis
GO:0006073	28	6	0.201111703413366	glucan metabolism
GO:0006898	28	6	0.201111703413366	receptor mediated endocytosis
GO:0030098	34	7	0.202947998454531	lymphocyte differentiation
GO:0006636	6	2	0.2064951411802	fatty acid desaturation
GO:0006929	6	2	0.2064951411802	substrate-bound cell migration
GO:0001935	6	2	0.2064951411802	endothelial cell proliferation
GO:0016478	6	2	0.2064951411802	negative regulation of translation
GO:0009895	6	2	0.2064951411802	negative regulation of catabolism
GO:0012501	473	74	0.207621983752673	programmed cell death
GO:0050793	168	28	0.213414423865051	regulation of development
GO:0006521	17	4	0.216453959839305	regulation of amino acid metabolism
GO:0006692	17	4	0.216453959839305	prostanoid metabolism
GO:0006693	17	4	0.216453959839305	prostaglandin metabolism
GO:0042098	17	4	0.216453959839305	T cell proliferation
GO:0019722	17	4	0.216453959839305	calcium-mediated signaling
GO:0007611	17	4	0.216453959839305	learning and/or memory
GO:0009109	23	5	0.223174681504364	coenzyme catabolism
GO:0046456	23	5	0.223174681504364	icosanoid biosynthesis
GO:0051188	85	15	0.225117625627222	cofactor biosynthesis
GO:0006519	216	35	0.231473535030104	amino acid and derivative metabolism
GO:0006915	471	73	0.235250405672483	apoptosis
GO:0007154	2730	401	0.237379154990885	cell communication
GO:0008206	12	3	0.239315668621326	bile acid metabolism
GO:0019216	12	3	0.239315668621326	regulation of lipid metabolism
GO:0042095	12	3	0.239315668621326	interferon-gamma biosynthesis
GO:0045619	12	3	0.239315668621326	regulation of lymphocyte differentiation
GO:0042327	12	3	0.239315668621326	positive regulation of phosphorylation
GO:0007422	12	3	0.239315668621326	peripheral nervous system development
GO:0045937	12	3	0.239315668621326	positive regulation of phosphate metabolism
GO:0042102	12	3	0.239315668621326	positive regulation of T cell proliferation
GO:0006275	12	3	0.239315668621326	regulation of DNA replication
GO:0001709	12	3	0.239315668621326	cell fate determination
GO:0006807	291	46	0.246166415770257	nitrogen compound metabolism
GO:0009072	18	4	0.249140630366081	aromatic amino acid family metabolism
GO:0045597	18	4	0.249140630366081	positive regulation of cell differentiation
GO:0050671	18	4	0.249140630366081	positive regulation of lymphocyte proliferation
GO:0042088	24	5	0.251308294911168	T-helper 1 type immune response
GO:0051259	24	5	0.251308294911168	protein oligomerization
GO:0019318	113	19	0.25440006948894	hexose metabolism
GO:0048167	7	2	0.263096627445896	regulation of synaptic plasticity
GO:0045787	7	2	0.263096627445896	positive regulation of progression through cell cycle
GO:0045582	7	2	0.263096627445896	positive regulation of T cell differentiation
GO:0045621	7	2	0.263096627445896	positive regulation of lymphocyte differentiation

GO:0016045	7	2	0.263096627445896	detection of bacteria
GO:0050792	7	2	0.263096627445896	regulation of viral life cycle
GO:0050715	7	2	0.263096627445896	positive regulation of cytokine secretion
GO:0050707	7	2	0.263096627445896	regulation of cytokine secretion
GO:0009411	7	2	0.263096627445896	response to UV
GO:0050663	7	2	0.263096627445896	cytokine secretion
GO:0042509	7	2	0.263096627445896	regulation of tyrosine phosphorylation of STAT protein
GO:0046425	7	2	0.263096627445896	regulation of JAK-STAT cascade
GO:0006265	7	2	0.263096627445896	DNA topological change
GO:0000279	173	28	0.263603731540809	M phase
GO:0015791	2	1	0.265075619367950	polyol transport
GO:0043070	2	1	0.265075619367950	regulation of non-apoptotic programmed cell death
GO:0043072	2	1	0.265075619367950	negative regulation of non-apoptotic programmed cell death
GO:0030501	2	1	0.265075619367950	positive regulation of bone mineralization
GO:0006599	2	1	0.265075619367950	phosphagen metabolism
GO:0043006	2	1	0.265075619367950	calcium-dependent phospholipase A2 activation
GO:0046513	2	1	0.265075619367950	ceramide biosynthesis
GO:0006581	2	1	0.265075619367950	acetylcholine catabolism
GO:0006517	2	1	0.265075619367950	protein deglycosylation
GO:0046502	2	1	0.265075619367950	uroporphyrinogen III metabolism
GO:0006548	2	1	0.265075619367950	histidine catabolism
GO:0031341	2	1	0.265075619367950	regulation of cell killing
GO:0001550	2	1	0.265075619367950	ovarian cumulus expansion
GO:0015811	2	1	0.265075619367950	L-cystine transport
GO:0001547	2	1	0.265075619367950	antral ovarian follicle growth
GO:0015840	2	1	0.265075619367950	urea transport
GO:0001507	2	1	0.265075619367950	acetylcholine catabolism in synaptic cleft
GO:0046626	2	1	0.265075619367950	regulation of insulin receptor signaling pathway
GO:0046627	2	1	0.265075619367950	negative regulation of insulin receptor signaling pathway
GO:0015802	2	1	0.265075619367950	basic amino acid transport
GO:0006678	2	1	0.265075619367950	glucosylceramide metabolism
GO:0006679	2	1	0.265075619367950	glucosylceramide biosynthesis
GO:0045360	2	1	0.265075619367950	regulation of interleukin-1 biosynthesis
GO:0045362	2	1	0.265075619367950	positive regulation of interleukin-1 biosynthesis
GO:0006600	2	1	0.265075619367950	creatine metabolism
GO:0006601	2	1	0.265075619367950	creatine biosynthesis
GO:0045348	2	1	0.265075619367950	positive regulation of MHC class II biosynthesis
GO:0008347	2	1	0.265075619367950	glial cell migration
GO:0045123	2	1	0.265075619367950	cellular extravasation
GO:0048165	2	1	0.265075619367950	fused antrum stage oogenesis
GO:0048169	2	1	0.265075619367950	regulation of long-term neuronal synaptic plasticity
GO:0048157	2	1	0.265075619367950	oogenesis (sensu Mammalia)
GO:0006927	2	1	0.265075619367950	programmed cell death, transformed cells
GO:0048146	2	1	0.265075619367950	positive regulation of fibroblast proliferation
GO:0035081	2	1	0.265075619367950	induction of programmed cell death by hormones
GO:0001910	2	1	0.265075619367950	regulation of immune cell mediated cytotoxicity

GO:0001943	2	1	0.265075619367950	hair follicle maturation
GO:0001937	2	1	0.265075619367950	negative regulation of endothelial cell proliferation
GO:0008228	2	1	0.265075619367950	opsonization
GO:0045778	2	1	0.265075619367950	positive regulation of ossification
GO:0009077	2	1	0.265075619367950	histidine family amino acid catabolism
GO:0045885	2	1	0.265075619367950	positive regulation of survival gene product activity
GO:0045842	2	1	0.265075619367950	positive regulation of mitotic metaphase/anaphase transition
GO:0042063	2	1	0.265075619367950	gliogenesis
GO:0007079	2	1	0.265075619367950	mitotic chromosome movement towards spindle pole
GO:0019733	2	1	0.265075619367950	antibacterial humoral response (sensu Vertebrata)
GO:0000022	2	1	0.265075619367950	mitotic spindle elongation
GO:0016081	2	1	0.265075619367950	synaptic vesicle docking during exocytosis
GO:0045652	2	1	0.265075619367950	regulation of megakaryocyte differentiation
GO:0008628	2	1	0.265075619367950	induction of apoptosis by hormones
GO:0050774	2	1	0.265075619367950	negative regulation of dendrite morphogenesis
GO:0042391	2	1	0.265075619367950	regulation of membrane potential
GO:0042396	2	1	0.265075619367950	phosphagen biosynthesis
GO:0007308	2	1	0.265075619367950	oocyte construction
GO:0007309	2	1	0.265075619367950	oocyte axis determination
GO:0009185	2	1	0.265075619367950	ribonucleoside diphosphate metabolism
GO:0009120	2	1	0.265075619367950	deoxyribonucleoside metabolism
GO:0042448	2	1	0.265075619367950	progesterone metabolism
GO:0007405	2	1	0.265075619367950	neuroblast proliferation
GO:0000305	2	1	0.265075619367950	response to oxygen radicals
GO:0042167	2	1	0.265075619367950	heme catabolism
GO:0045909	2	1	0.265075619367950	positive regulation of vasodilation
GO:0050665	2	1	0.265075619367950	hydrogen peroxide biosynthesis
GO:0051382	2	1	0.265075619367950	kinetochore assembly
GO:0051383	2	1	0.265075619367950	kinetochore organization and biogenesis
GO:0051305	2	1	0.265075619367950	chromosome movement towards spindle pole
GO:0042222	2	1	0.265075619367950	interleukin-1 biosynthesis
GO:0009746	2	1	0.265075619367950	response to hexose stimulus
GO:0009749	2	1	0.265075619367950	response to glucose stimulus
GO:0007206	2	1	0.265075619367950	metabotropic glutamate receptor, phospholipase C activating pathway
GO:0007207	2	1	0.265075619367950	muscarinic acetylcholine receptor, phospholipase C activating pathway
GO:0000101	2	1	0.265075619367950	sulfur amino acid transport
GO:0051402	2	1	0.265075619367950	programmed cell death, neurons
GO:0042752	2	1	0.265075619367950	regulation of circadian rhythm
GO:0051150	2	1	0.265075619367950	regulation of smooth muscle cell differentiation
GO:0051151	2	1	0.265075619367950	negative regulation of smooth muscle cell differentiation
GO:0042732	2	1	0.265075619367950	D-xylose metabolism
GO:0016244	2	1	0.265075619367950	non-apoptotic programmed cell death
GO:0051145	2	1	0.265075619367950	smooth muscle cell differentiation
GO:0051147	2	1	0.265075619367950	regulation of muscle cell differentiation
GO:0051148	2	1	0.265075619367950	negative regulation of muscle cell differentiation
GO:0046037	2	1	0.265075619367950	GMP metabolism

GO:0009598	2	1	0.265075619367950	detection of pathogenic bacteria
GO:0051231	2	1	0.265075619367950	spindle elongation
GO:0046339	2	1	0.265075619367950	diacylglycerol metabolism
GO:0046460	2	1	0.265075619367950	neutral lipid biosynthesis
GO:0046463	2	1	0.265075619367950	acylglycerol biosynthesis
GO:0046476	2	1	0.265075619367950	glycosylceramide biosynthesis
GO:0006475	2	1	0.265075619367950	internal protein amino acid acetylation
GO:0006435	2	1	0.265075619367950	threonyl-tRNA aminoacylation
GO:0046125	2	1	0.265075619367950	pyrimidine deoxyribonucleoside metabolism
GO:0046149	2	1	0.265075619367950	pigment catabolism
GO:0006177	2	1	0.265075619367950	GMP biosynthesis
GO:0046135	2	1	0.265075619367950	pyrimidine nucleoside catabolism
GO:0006278	2	1	0.265075619367950	RNA-dependent DNA replication
GO:0006780	2	1	0.265075619367950	uroporphyrinogen III biosynthesis
GO:0001766	2	1	0.265075619367950	lipid raft polarization
GO:0001776	2	1	0.265075619367950	immune cell homeostasis
GO:0006704	2	1	0.265075619367950	glucocorticoid biosynthesis
GO:0045071	2	1	0.265075619367950	negative regulation of viral genome replication
GO:0046852	2	1	0.265075619367950	positive regulation of bone remodeling
GO:0006863	2	1	0.265075619367950	purine transport
GO:0000041	31	6	0.275777472278674	transition metal ion transport
GO:0008360	25	5	0.280256433892626	regulation of cell shape
GO:0019439	13	3	0.280470425332118	aromatic compound catabolism
GO:0006000	13	3	0.280470425332118	fructose metabolism
GO:0030168	13	3	0.280470425332118	platelet activation
GO:0005996	115	19	0.280496480154312	monosaccharide metabolism
GO:0006939	19	4	0.282773016853961	smooth muscle contraction
GO:0018108	19	4	0.282773016853961	peptidyl-tyrosine phosphorylation
GO:0008610	175	28	0.284921428991948	lipid biosynthesis
GO:0009308	269	42	0.28636918880852	amine metabolism
GO:0009582	109	18	0.288344642084432	detection of abiotic stimulus
GO:0019932	176	28	0.295811778573174	second-messenger-mediated signaling
GO:0008361	123	20	0.298994770901474	regulation of cell size
GO:0016049	123	20	0.298994770901474	cell growth
GO:0006007	51	9	0.299447222252599	glucose catabolism
GO:0009117	143	23	0.299919652462878	nucleotide metabolism
GO:0008154	45	8	0.308589192840327	actin polymerization and/or depolymerization
GO:0007498	26	5	0.309805736465996	mesoderm development
GO:0044419	26	5	0.309805736465996	interaction between organisms
GO:0051248	58	10	0.310308174750137	negative regulation of protein metabolism
GO:0044262	265	41	0.310964783839534	cellular carbohydrate metabolism
GO:0000096	20	4	0.317017376201489	sulfur amino acid metabolism
GO:0042493	20	4	0.317017376201489	response to drug
GO:0007205	20	4	0.317017376201489	protein kinase C activation
GO:0044403	20	4	0.317017376201489	symbiosis, mutualism through parasitism
GO:0044404	20	4	0.317017376201489	symbiotic interaction between host and other organism

GO:0006100	20	4	0.317017376201489	tricarboxylic acid cycle intermediate metabolism
GO:0019363	8	2	0.319714838065798	pyridine nucleotide biosynthesis
GO:0016054	8	2	0.319714838065798	organic acid catabolism
GO:0050866	8	2	0.319714838065798	negative regulation of cell activation
GO:0009166	8	2	0.319714838065798	nucleotide catabolism
GO:0009596	8	2	0.319714838065798	detection of pest, pathogen or parasite
GO:0051250	8	2	0.319714838065798	negative regulation of lymphocyte activation
GO:0009636	8	2	0.319714838065798	response to toxin
GO:0046395	8	2	0.319714838065798	carboxylic acid catabolism
GO:0042632	8	2	0.319714838065798	cholesterol homeostasis
GO:0046883	8	2	0.319714838065798	regulation of hormone secretion
GO:0015837	52	9	0.320319596936547	amine transport
GO:0000226	52	9	0.320319596936547	microtubule cytoskeleton organization and biogenesis
GO:0006869	52	9	0.320319596936547	lipid transport
GO:0002009	14	3	0.32217275614262	morphogenesis of an epithelium
GO:0009725	14	3	0.32217275614262	response to hormone stimulus
GO:0007156	105	17	0.325765084059286	homophilic cell adhesion
GO:0006919	27	5	0.339748767254867	caspase activation
GO:0009187	27	5	0.339748767254867	cyclic nucleotide metabolism
GO:0005977	27	5	0.339748767254867	glycogen metabolism
GO:0006084	27	5	0.339748767254867	acetyl-CoA metabolism
GO:0050954	86	14	0.340673598381567	sensory perception of mechanical stimulus
GO:0050982	86	14	0.340673598381567	detection of mechanical stimulus
GO:0007605	86	14	0.340673598381567	perception of sound
GO:0007588	40	7	0.342786309649543	excretion
GO:0018212	21	4	0.351560360024151	peptidyl-tyrosine modification
GO:0048522	482	72	0.353760684307136	positive regulation of cellular process
GO:0006112	34	6	0.355611519649799	energy reserve metabolism
GO:0043085	107	17	0.355626767964533	positive regulation of enzyme activity
GO:0006417	107	17	0.355626767964533	regulation of protein biosynthesis
GO:0001558	94	15	0.362294108326365	regulation of cell growth
GO:0045165	15	3	0.363887418536123	cell fate commitment
GO:0050767	15	3	0.363887418536123	regulation of neurogenesis
GO:0050730	15	3	0.363887418536123	regulation of peptidyl-tyrosine phosphorylation
GO:0007162	15	3	0.363887418536123	negative regulation of cell adhesion
GO:0030111	15	3	0.363887418536123	regulation of Wnt receptor signaling pathway
GO:0043065	155	24	0.365718314096881	positive regulation of apoptosis
GO:0007186	491	73	0.368977045038156	G-protein coupled receptor protein signaling pathway
GO:0051348	28	5	0.369887259394323	negative regulation of transferase activity
GO:0007259	28	5	0.369887259394323	JAK-STAT cascade
GO:0006469	28	5	0.369887259394323	negative regulation of protein kinase activity
GO:0006824	3	1	0.369983429626501	cobalt ion transport
GO:0048002	3	1	0.369983429626501	antigen presentation, peptide antigen
GO:0048004	3	1	0.369983429626501	antigen presentation, endogenous peptide antigen
GO:0043026	3	1	0.369983429626501	regulation of caspase activation
GO:0046520	3	1	0.369983429626501	sphingoid biosynthesis

GO:0006568	3	1	0.369983429626501	tryptophan metabolism
GO:0006586	3	1	0.369983429626501	indolalkylamine metabolism
GO:0046504	3	1	0.369983429626501	glycerol ether biosynthesis
GO:0015851	3	1	0.369983429626501	nucleobase transport
GO:0006677	3	1	0.369983429626501	glycosylceramide metabolism
GO:0045350	3	1	0.369983429626501	interferon-beta biosynthesis
GO:0045356	3	1	0.369983429626501	positive regulation of interferon-alpha biosynthesis
GO:0045357	3	1	0.369983429626501	regulation of interferon-beta biosynthesis
GO:0045359	3	1	0.369983429626501	positive regulation of interferon-beta biosynthesis
GO:0045342	3	1	0.369983429626501	MHC class II biosynthesis
GO:0045346	3	1	0.369983429626501	regulation of MHC class II biosynthesis
GO:0043303	3	1	0.369983429626501	mast cell degranulation
GO:0019321	3	1	0.369983429626501	pentose metabolism
GO:0006970	3	1	0.369983429626501	response to osmotic stress
GO:0045103	3	1	0.369983429626501	intermediate filament-based process
GO:0045104	3	1	0.369983429626501	intermediate filament cytoskeleton organization and biogenesis
GO:0043154	3	1	0.369983429626501	negative regulation of caspase activation
GO:0006930	3	1	0.369983429626501	substrate-bound cell migration, cell extension
GO:0001942	3	1	0.369983429626501	hair follicle development
GO:0001938	3	1	0.369983429626501	positive regulation of endothelial cell proliferation
GO:0008291	3	1	0.369983429626501	acetylcholine metabolism
GO:0043299	3	1	0.369983429626501	immune cell degranulation
GO:0017157	3	1	0.369983429626501	regulation of exocytosis
GO:0043249	3	1	0.369983429626501	erythrocyte maturation
GO:0015918	3	1	0.369983429626501	sterol transport
GO:0045833	3	1	0.369983429626501	negative regulation of lipid metabolism
GO:0045577	3	1	0.369983429626501	regulation of B cell differentiation
GO:0035315	3	1	0.369983429626501	hair cell differentiation
GO:0048525	3	1	0.369983429626501	negative regulation of viral life cycle
GO:0000085	3	1	0.369983429626501	G2 phase of mitotic cell cycle
GO:0008588	3	1	0.369983429626501	release of cytoplasmic sequestered NF-kappaB
GO:0008589	3	1	0.369983429626501	regulation of smoothened signaling pathway
GO:0045646	3	1	0.369983429626501	regulation of erythrocyte differentiation
GO:0045601	3	1	0.369983429626501	regulation of endothelial cell differentiation
GO:0050796	3	1	0.369983429626501	regulation of insulin secretion
GO:0050773	3	1	0.369983429626501	regulation of dendrite morphogenesis
GO:0007368	3	1	0.369983429626501	determination of left/right symmetry
GO:0050892	3	1	0.369983429626501	intestinal absorption
GO:0007340	3	1	0.369983429626501	acrosome reaction
GO:0042335	3	1	0.369983429626501	cuticle biosynthesis
GO:0042303	3	1	0.369983429626501	molting cycle
GO:0009164	3	1	0.369983429626501	nucleoside catabolism
GO:0009129	3	1	0.369983429626501	pyrimidine nucleoside monophosphate metabolism
GO:0009113	3	1	0.369983429626501	purine base biosynthesis
GO:0042430	3	1	0.369983429626501	indole and derivative metabolism
GO:0042434	3	1	0.369983429626501	indole derivative metabolism

GO:0009130	3	1	0.369983429626501	pyrimidine nucleoside monophosphate biosynthesis
GO:0009299	3	1	0.369983429626501	mRNA transcription
GO:0007176	3	1	0.369983429626501	regulation of epidermal growth factor receptor activity
GO:0042130	3	1	0.369983429626501	negative regulation of T cell proliferation
GO:0051319	3	1	0.369983429626501	G2 phase
GO:0007262	3	1	0.369983429626501	STAT protein nuclear translocation
GO:0009799	3	1	0.369983429626501	determination of symmetry
GO:0007272	3	1	0.369983429626501	ionic insulation of neurons by glial cells
GO:0009855	3	1	0.369983429626501	determination of bilateral symmetry
GO:0042723	3	1	0.369983429626501	thiamin and derivative metabolism
GO:0046040	3	1	0.369983429626501	IMP metabolism
GO:0042552	3	1	0.369983429626501	myelination
GO:0042553	3	1	0.369983429626501	cellular nerve ensheathment
GO:0007520	3	1	0.369983429626501	myoblast fusion
GO:0007500	3	1	0.369983429626501	mesodermal cell fate determination
GO:0030301	3	1	0.369983429626501	cholesterol transport
GO:0042633	3	1	0.369983429626501	hair cycle
GO:0046329	3	1	0.369983429626501	negative regulation of JNK cascade
GO:0006301	3	1	0.369983429626501	postreplication repair
GO:0006474	3	1	0.369983429626501	N-terminal protein amino acid acetylation
GO:0006449	3	1	0.369983429626501	regulation of translational termination
GO:0030299	3	1	0.369983429626501	cholesterol absorption
GO:0006188	3	1	0.369983429626501	IMP biosynthesis
GO:0006189	3	1	0.369983429626501	'de novo' IMP biosynthesis
GO:0030219	3	1	0.369983429626501	megakaryocyte differentiation
GO:0006266	3	1	0.369983429626501	DNA ligation
GO:0016998	3	1	0.369983429626501	cell wall catabolism
GO:0044274	3	1	0.369983429626501	organismal biosynthesis
GO:0044241	3	1	0.369983429626501	lipid digestion
GO:0030851	3	1	0.369983429626501	granulocyte differentiation
GO:0006772	3	1	0.369983429626501	thiamin metabolism
GO:0045017	3	1	0.369983429626501	glycerolipid biosynthesis
GO:0030573	3	1	0.369983429626501	bile acid catabolism
GO:0000122	61	10	0.370298881806699	negative regulation of transcription from RNA polymerase II promoter
GO:0051301	142	22	0.373054765039025	cell division
GO:0008366	9	2	0.37519294034115	nerve ensheathment
GO:0048598	9	2	0.37519294034115	embryonic morphogenesis
GO:0045638	9	2	0.37519294034115	negative regulation of myeloid cell differentiation
GO:0050731	9	2	0.37519294034115	positive regulation of peptidyl-tyrosine phosphorylation
GO:0007159	9	2	0.37519294034115	leukocyte adhesion
GO:0042100	9	2	0.37519294034115	B cell proliferation
GO:0000160	9	2	0.37519294034115	two-component signal transduction system (phosphorelay)
GO:0007271	9	2	0.37519294034115	synaptic transmission, cholinergic
GO:0016202	9	2	0.37519294034115	regulation of myogenesis
GO:0042551	9	2	0.37519294034115	nerve maturation
GO:0030330	9	2	0.37519294034115	DNA damage response, signal transduction by p53 class mediator

GO:0043068	156	24	0.378378854003873	positive regulation of programmed cell death
GO:0030832	35	6	0.382693378438836	regulation of actin filament length
GO:0019395	22	4	0.386113566310859	fatty acid oxidation
GO:0006099	22	4	0.386113566310859	tricarboxylic acid cycle
GO:0046356	22	4	0.386113566310859	acetyl-CoA catabolism
GO:0045859	116	18	0.389209080716989	regulation of protein kinase activity
GO:0009889	116	18	0.389209080716989	regulation of biosynthesis
GO:0006096	42	7	0.392281783352716	glycolysis
GO:0044264	42	7	0.392281783352716	cellular polysaccharide metabolism
GO:0043405	49	8	0.399802915223983	regulation of MAPK activity
GO:0006767	29	5	0.400034677490286	water-soluble vitamin metabolism
GO:0048518	565	83	0.403607725598763	positive regulation of biological process
GO:0051338	117	18	0.404070271078199	regulation of transferase activity
GO:0001932	16	3	0.405155116512276	regulation of protein amino acid phosphorylation
GO:0045637	16	3	0.405155116512276	regulation of myeloid cell differentiation
GO:0007202	16	3	0.405155116512276	phospholipase C activation
GO:0009595	16	3	0.405155116512276	detection of biotic stimulus
GO:0006473	16	3	0.405155116512276	protein amino acid acetylation
GO:0006284	16	3	0.405155116512276	base-excision repair
GO:0007200	56	9	0.405903547686034	G-protein signaling, coupled to IP3 second messenger (phospholipase C activating)
GO:0031326	111	17	0.416642113372726	regulation of cellular biosynthesis
GO:0006865	43	7	0.417091178598215	amino acid transport
GO:0008406	23	4	0.420416489428017	gonad development
GO:0045137	23	4	0.420416489428017	development of primary sexual characteristics
GO:0009116	23	4	0.420416489428017	nucleoside metabolism
GO:0001516	10	2	0.428703515992529	prostaglandin biosynthesis
GO:0001934	10	2	0.428703515992529	positive regulation of protein amino acid phosphorylation
GO:0045764	10	2	0.428703515992529	positive regulation of amino acid metabolism
GO:0008630	10	2	0.428703515992529	DNA damage response, signal transduction resulting in induction of apoptosis
GO:0046457	10	2	0.428703515992529	prostanoid biosynthesis
GO:0006739	10	2	0.428703515992529	NADP metabolism
GO:0051246	174	26	0.432264274637418	regulation of protein metabolism
GO:0006520	181	27	0.433472210959178	amino acid metabolism
GO:0051258	37	6	0.436755805315553	protein polymerization
GO:0006917	140	21	0.438695776420426	induction of apoptosis
GO:0012502	140	21	0.438695776420426	induction of programmed cell death
GO:0005976	44	7	0.441811657964146	polysaccharide metabolism
GO:0015758	17	3	0.445591078656484	glucose transport
GO:0019362	17	3	0.445591078656484	pyridine nucleotide metabolism
GO:0043284	17	3	0.445591078656484	biopolymer biosynthesis
GO:0045596	17	3	0.445591078656484	negative regulation of cell differentiation
GO:0007416	17	3	0.445591078656484	synaptogenesis
GO:0007292	17	3	0.445591078656484	female gamete generation
GO:0000271	17	3	0.445591078656484	polysaccharide biosynthesis
GO:0006270	17	3	0.445591078656484	DNA replication initiation
GO:0009892	217	32	0.449736136540579	negative regulation of metabolism

GO:0009790	65	10	0.451684701853529	embryonic development
GO:0006261	65	10	0.451684701853529	DNA-dependent DNA replication
GO:0007389	24	4	0.454238128331386	pattern specification
GO:0031324	183	27	0.457755816820726	negative regulation of cellular metabolism
GO:0000902	183	27	0.457755816820726	cellular morphogenesis
GO:0043406	31	5	0.459679816742875	positive regulation of MAPK activity
GO:0043280	31	5	0.459679816742875	positive regulation of caspase activity
GO:0043281	31	5	0.459679816742875	regulation of caspase activity
GO:0030516	4	1	0.459925987962024	regulation of axon extension
GO:0008089	4	1	0.459925987962024	anterograde axon cargo transport
GO:0006572	4	1	0.459925987962024	tyrosine catabolism
GO:0015893	4	1	0.459925987962024	drug transport
GO:0019048	4	1	0.459925987962024	virus-host interaction
GO:0006699	4	1	0.459925987962024	bile acid biosynthesis
GO:0001504	4	1	0.459925987962024	neurotransmitter uptake
GO:0045351	4	1	0.459925987962024	interferon type I biosynthesis
GO:0045354	4	1	0.459925987962024	regulation of interferon-alpha biosynthesis
GO:0045349	4	1	0.459925987962024	interferon-alpha biosynthesis
GO:0048333	4	1	0.459925987962024	mesodermal cell differentiation
GO:0045453	4	1	0.459925987962024	bone resorption
GO:0045429	4	1	0.459925987962024	positive regulation of nitric oxide biosynthesis
GO:0045197	4	1	0.459925987962024	establishment and/or maintenance of epithelial cell polarity
GO:0008156	4	1	0.459925987962024	negative regulation of DNA replication
GO:0048144	4	1	0.459925987962024	fibroblast proliferation
GO:0048145	4	1	0.459925987962024	regulation of fibroblast proliferation
GO:0035088	4	1	0.459925987962024	establishment and/or maintenance of apical/basal cell polarity
GO:0043112	4	1	0.459925987962024	receptor metabolism
GO:0015991	4	1	0.459925987962024	ATP hydrolysis coupled proton transport
GO:0008211	4	1	0.459925987962024	glucocorticoid metabolism
GO:0045785	4	1	0.459925987962024	positive regulation of cell adhesion
GO:0045749	4	1	0.459925987962024	negative regulation of S phase of mitotic cell cycle
GO:0009062	4	1	0.459925987962024	fatty acid catabolism
GO:0009086	4	1	0.459925987962024	methionine biosynthesis
GO:0045834	4	1	0.459925987962024	positive regulation of lipid metabolism
GO:0000012	4	1	0.459925987962024	single strand break repair
GO:0000018	4	1	0.459925987962024	regulation of DNA recombination
GO:0050768	4	1	0.459925987962024	negative regulation of neurogenesis
GO:0019642	4	1	0.459925987962024	anaerobic glycolysis
GO:0042346	4	1	0.459925987962024	positive regulation of NF-kappaB-nucleus import
GO:0042307	4	1	0.459925987962024	positive regulation of protein-nucleus import
GO:0042439	4	1	0.459925987962024	ethanolamine and derivative metabolism
GO:0045913	4	1	0.459925987962024	positive regulation of carbohydrate metabolism
GO:0045947	4	1	0.459925987962024	negative regulation of translational initiation
GO:0007158	4	1	0.459925987962024	neuron adhesion
GO:0007171	4	1	0.459925987962024	transmembrane receptor protein tyrosine kinase activation (dimerization)
GO:0007140	4	1	0.459925987962024	male meiosis

GO:0009219	4	1	0.459925987962024	pyrimidine deoxyribonucleotide metabolism
GO:0016486	4	1	0.459925987962024	peptide hormone processing
GO:0050679	4	1	0.459925987962024	positive regulation of epithelial cell proliferation
GO:0051310	4	1	0.459925987962024	metaphase plate congression
GO:0051341	4	1	0.459925987962024	regulation of oxidoreductase activity
GO:0009798	4	1	0.459925987962024	axis specification
GO:0007224	4	1	0.459925987962024	smoothened signaling pathway
GO:0006067	4	1	0.459925987962024	ethanol metabolism
GO:0006069	4	1	0.459925987962024	ethanol oxidation
GO:0006072	4	1	0.459925987962024	glycerol-3-phosphate metabolism
GO:0050999	4	1	0.459925987962024	regulation of nitric-oxide synthase activity
GO:0051252	4	1	0.459925987962024	regulation of RNA metabolism
GO:0051222	4	1	0.459925987962024	positive regulation of protein transport
GO:0007566	4	1	0.459925987962024	embryo implantation
GO:0030324	4	1	0.459925987962024	lung development
GO:0006349	4	1	0.459925987962024	imprinting
GO:0030199	4	1	0.459925987962024	collagen fibril organization
GO:0009953	4	1	0.459925987962024	dorsal/ventral pattern formation
GO:0042993	4	1	0.459925987962024	positive regulation of transcription factor-nucleus import
GO:0006110	4	1	0.459925987962024	regulation of glycolysis
GO:0006144	4	1	0.459925987962024	purine base metabolism
GO:0006109	4	1	0.459925987962024	regulation of carbohydrate metabolism
GO:0006213	4	1	0.459925987962024	pyrimidine nucleoside metabolism
GO:0018409	4	1	0.459925987962024	peptide or protein amino-terminal blocking
GO:0006207	4	1	0.459925987962024	'de novo' pyrimidine base biosynthesis
GO:0001710	4	1	0.459925987962024	mesodermal cell fate commitment
GO:0009310	45	7	0.466352638381692	amine catabolism
GO:0015849	59	9	0.470408345065731	organic acid transport
GO:0019320	59	9	0.470408345065731	hexose catabolism
GO:0046942	59	9	0.470408345065731	carboxylic acid transport
GO:0046365	59	9	0.470408345065731	monosaccharide catabolism
GO:0007006	11	2	0.479678207815407	mitochondrial membrane organization and biogenesis
GO:0007193	11	2	0.479678207815407	G-protein signaling, adenylate cyclase inhibiting pathway
GO:0007270	11	2	0.479678207815407	nerve-nerve synaptic transmission
GO:0000272	11	2	0.479678207815407	polysaccharide catabolism
GO:0046328	11	2	0.479678207815407	regulation of JNK cascade
GO:0006171	11	2	0.479678207815407	cAMP biosynthesis
GO:0044247	11	2	0.479678207815407	cellular polysaccharide catabolism
GO:0046879	11	2	0.479678207815407	hormone secretion
GO:0015749	18	3	0.484881168314564	monosaccharide transport
GO:0019079	18	3	0.484881168314564	viral genome replication
GO:0000080	18	3	0.484881168314564	G1 phase of mitotic cell cycle
GO:0008645	18	3	0.484881168314564	hexose transport
GO:0009416	18	3	0.484881168314564	response to light
GO:0050808	18	3	0.484881168314564	synapse organization and biogenesis
GO:0042133	18	3	0.484881168314564	neurotransmitter metabolism

GO:0030041	18	3	0.484881168314564	actin filament polymerization
GO:0000910	18	3	0.484881168314564	cytokinesis
GO:0045792	25	4	0.487377498700674	negative regulation of cell size
GO:0042325	25	4	0.487377498700674	regulation of phosphorylation
GO:0051235	25	4	0.487377498700674	maintenance of localization
GO:0030308	25	4	0.487377498700674	negative regulation of cell growth
GO:0044242	25	4	0.487377498700674	cellular lipid catabolism
GO:0009064	32	5	0.488877691716237	glutamine family amino acid metabolism
GO:0051345	32	5	0.488877691716237	positive regulation of hydrolase activity
GO:0007088	39	6	0.489892206241906	regulation of mitosis
GO:0031175	46	7	0.490629789238552	neurite morphogenesis
GO:0051049	46	7	0.490629789238552	regulation of transport
GO:0045860	53	8	0.491192835918835	positive regulation of protein kinase activity
GO:0046164	60	9	0.491637949498936	alcohol catabolism
GO:0007169	116	17	0.493377613154521	transmembrane receptor protein tyrosine kinase signaling pathway
GO:0007010	305	44	0.494458693857979	cytoskeleton organization and biogenesis
GO:0005975	355	51	0.503316361074631	carbohydrate metabolism
GO:0007050	61	9	0.512638941984131	cell cycle arrest
GO:0051347	54	8	0.513516606678071	positive regulation of transferase activity
GO:0044270	47	7	0.514565449791795	nitrogen compound catabolism
GO:0006766	40	6	0.51585207034644	vitamin metabolism
GO:0009314	33	5	0.517486047690137	response to radiation
GO:0008064	33	5	0.517486047690137	regulation of actin polymerization and/or depolymerization
GO:0019058	26	4	0.519663273652433	viral infectious cycle
GO:0006220	26	4	0.519663273652433	pyrimidine nucleotide metabolism
GO:0040008	104	15	0.525331144334865	regulation of growth
GO:0019370	12	2	0.527750715654224	leukotriene biosynthesis
GO:0009065	12	2	0.527750715654224	glutamine family amino acid catabolism
GO:0042364	12	2	0.527750715654224	water-soluble vitamin biosynthesis
GO:0035295	12	2	0.527750715654224	tube development
GO:0006887	55	8	0.535505599703166	exocytosis
GO:0030641	5	1	0.537036688174359	hydrogen ion homeostasis
GO:0019369	5	1	0.537036688174359	arachidonic acid metabolism
GO:0045446	5	1	0.537036688174359	endothelial cell differentiation
GO:0045576	5	1	0.537036688174359	mast cell activation
GO:0005513	5	1	0.537036688174359	calcium ion sensing
GO:0051053	5	1	0.537036688174359	negative regulation of DNA metabolism
GO:0008635	5	1	0.537036688174359	caspase activation via cytochrome c
GO:0050716	5	1	0.537036688174359	positive regulation of interleukin-1 secretion
GO:0050718	5	1	0.537036688174359	positive regulation of interleukin-1 beta secretion
GO:0050701	5	1	0.537036688174359	interleukin-1 secretion
GO:0050702	5	1	0.537036688174359	interleukin-1 beta secretion
GO:0050704	5	1	0.537036688174359	regulation of interleukin-1 secretion
GO:0050706	5	1	0.537036688174359	regulation of interleukin-1 beta secretion
GO:0050868	5	1	0.537036688174359	negative regulation of T cell activation
GO:0042402	5	1	0.537036688174359	biogenic amine catabolism

GO:0009251	5	1	0.537036688174359	glucan catabolism
GO:0042177	5	1	0.537036688174359	negative regulation of protein catabolism
GO:0042135	5	1	0.537036688174359	neurotransmitter catabolism
GO:0007263	5	1	0.537036688174359	nitric oxide mediated signal transduction
GO:0007217	5	1	0.537036688174359	tachykinin signaling pathway
GO:0030073	5	1	0.537036688174359	insulin secretion
GO:0030335	5	1	0.537036688174359	positive regulation of cell migration
GO:0006415	5	1	0.537036688174359	translational termination
GO:0046146	5	1	0.537036688174359	tetrahydrobiopterin metabolism
GO:0030225	5	1	0.537036688174359	macrophage differentiation
GO:0006108	5	1	0.537036688174359	malate metabolism
GO:0006729	5	1	0.537036688174359	tetrahydrobiopterin biosynthesis
GO:0046824	5	1	0.537036688174359	positive regulation of nucleocytoplasmic transport
GO:0008652	34	5	0.545395449076401	amino acid biosynthesis
GO:0000187	27	4	0.550952753025923	activation of MAPK activity
GO:0015674	99	14	0.558999287487765	di-, tri-valent inorganic cation transport
GO:0009894	20	3	0.559086870921416	regulation of catabolism
GO:0007631	20	3	0.559086870921416	feeding behavior
GO:0009893	149	21	0.561128379756662	positive regulation of metabolism
GO:0051093	49	7	0.561136577675784	negative regulation of development
GO:0009967	85	12	0.563817379712549	positive regulation of signal transduction
GO:0009063	42	6	0.56612158324128	amino acid catabolism
GO:0006732	121	17	0.568054083390748	coenzyme metabolism
GO:0016032	35	5	0.572512460849329	viral life cycle
GO:0007548	35	5	0.572512460849329	sex differentiation
GO:0015711	13	2	0.572710768440062	organic anion transport
GO:0030182	13	2	0.572710768440062	neuron differentiation
GO:0031325	136	19	0.578080578379581	positive regulation of cellular metabolism
GO:0007399	385	54	0.579100971742156	neurogenesis
GO:0019220	28	4	0.581130335297857	regulation of phosphate metabolism
GO:0051174	28	4	0.581130335297857	regulation of phosphorus metabolism
GO:0016052	79	11	0.584613134123286	carbohydrate catabolism
GO:0044275	79	11	0.584613134123286	cellular carbohydrate catabolism
GO:0006897	115	16	0.585046034567367	endocytosis
GO:0051016	21	3	0.593674622737626	barbed-end actin filament capping
GO:0051318	21	3	0.593674622737626	G1 phase
GO:0030334	21	3	0.593674622737626	regulation of cell migration
GO:0000079	36	5	0.598759105476967	regulation of cyclin dependent protein kinase activity
GO:0006814	80	11	0.602200972226225	sodium ion transport
GO:0031498	6	1	0.60314499757319	chromatin disassembly
GO:0006621	6	1	0.60314499757319	protein retention in ER
GO:0006637	6	1	0.60314499757319	acyl-CoA metabolism
GO:0010038	6	1	0.60314499757319	response to metal ion
GO:0045428	6	1	0.60314499757319	regulation of nitric oxide biosynthesis
GO:0043433	6	1	0.60314499757319	negative regulation of transcription factor activity
GO:0001909	6	1	0.60314499757319	immune cell mediated cytotoxicity

GO:0015988	6	1	0.60314499757319	energy coupled proton transport, against electrochemical gradient
GO:0007090	6	1	0.60314499757319	regulation of S phase of mitotic cell cycle
GO:0007089	6	1	0.60314499757319	traversing start control point of mitotic cell cycle
GO:0016079	6	1	0.60314499757319	synaptic vesicle exocytosis
GO:0051054	6	1	0.60314499757319	positive regulation of DNA metabolism
GO:0035113	6	1	0.60314499757319	embryonic appendage morphogenesis
GO:0009303	6	1	0.60314499757319	rRNA transcription
GO:0050871	6	1	0.60314499757319	positive regulation of B cell activation
GO:0045930	6	1	0.60314499757319	negative regulation of progression through mitotic cell cycle
GO:0042116	6	1	0.60314499757319	macrophage activation
GO:0007216	6	1	0.60314499757319	metabotropic glutamate receptor signaling pathway
GO:0042219	6	1	0.60314499757319	amino acid derivative catabolism
GO:0030072	6	1	0.60314499757319	peptide hormone secretion
GO:0009593	6	1	0.60314499757319	detection of chemical substance
GO:0006046	6	1	0.60314499757319	N-acetylglucosamine catabolism
GO:0006032	6	1	0.60314499757319	chitin catabolism
GO:0030326	6	1	0.60314499757319	embryonic limb morphogenesis
GO:0046330	6	1	0.60314499757319	positive regulation of JNK cascade
GO:0006337	6	1	0.60314499757319	nucleosome disassembly
GO:0018196	6	1	0.60314499757319	peptidyl-asparagine modification
GO:0000904	6	1	0.60314499757319	cellular morphogenesis during differentiation
GO:0018279	6	1	0.60314499757319	protein amino acid N-linked glycosylation via asparagine
GO:0048511	29	4	0.610105640547068	rhythmic process
GO:0051052	29	4	0.610105640547068	regulation of DNA metabolism
GO:0045926	29	4	0.610105640547068	negative regulation of growth
GO:0007179	29	4	0.610105640547068	transforming growth factor beta receptor signaling pathway
GO:0007265	29	4	0.610105640547068	Ras protein signal transduction
GO:0000086	44	6	0.613732415557989	G2/M transition of mitotic cell cycle
GO:0006691	14	2	0.614467103822968	leukotriene metabolism
GO:0009110	14	2	0.614467103822968	vitamin biosynthesis
GO:0030048	14	2	0.614467103822968	actin filament-based movement
GO:0007602	14	2	0.614467103822968	phototransduction
GO:0006308	14	2	0.614467103822968	DNA catabolism
GO:0030224	14	2	0.614467103822968	monocyte differentiation
GO:0006769	14	2	0.614467103822968	nicotinamide metabolism
GO:0030029	139	19	0.618305441285642	actin filament-based process
GO:0040007	154	21	0.62526885105425	growth
GO:0016044	22	3	0.6264474211208	membrane organization and biogenesis
GO:0030042	22	3	0.6264474211208	actin filament depolymerization
GO:0030834	22	3	0.6264474211208	regulation of actin filament depolymerization
GO:0030835	22	3	0.6264474211208	negative regulation of actin filament depolymerization
GO:0045055	22	3	0.6264474211208	regulated secretory pathway
GO:0009060	30	4	0.637811408588015	aerobic respiration
GO:0042113	30	4	0.637811408588015	B cell activation
GO:0030036	126	17	0.638341723338975	actin cytoskeleton organization and biogenesis
GO:0050790	214	29	0.649704624393764	regulation of enzyme activity

GO:0006839	15	2	0.653017824636329	mitochondrial transport
GO:0043407	15	2	0.653017824636329	negative regulation of MAPK activity
GO:0000188	15	2	0.653017824636329	inactivation of MAPK activity
GO:0009583	15	2	0.653017824636329	detection of light
GO:0009966	229	31	0.655158810568342	regulation of signal transduction
GO:0008277	23	3	0.657352060874718	regulation of G-protein coupled receptor protein signaling pathway
GO:0009069	23	3	0.657352060874718	serine family amino acid metabolism
GO:0007015	23	3	0.657352060874718	actin filament organization
GO:0040012	23	3	0.657352060874718	regulation of locomotion
GO:0050795	23	3	0.657352060874718	regulation of behavior
GO:0051270	23	3	0.657352060874718	regulation of cell motility
GO:0043086	46	6	0.658272693160173	negative regulation of enzyme activity
GO:0006825	7	1	0.659819725927739	copper ion transport
GO:0008088	7	1	0.659819725927739	axon cargo transport
GO:0031365	7	1	0.659819725927739	N-terminal protein amino acid modification
GO:0006555	7	1	0.659819725927739	methionine metabolism
GO:0006536	7	1	0.659819725927739	glutamate metabolism
GO:0001570	7	1	0.659819725927739	vasculogenesis
GO:0001508	7	1	0.659819725927739	regulation of action potential
GO:0048332	7	1	0.659819725927739	mesoderm morphogenesis
GO:0045768	7	1	0.659819725927739	positive regulation of anti-apoptosis
GO:0043241	7	1	0.659819725927739	protein complex disassembly
GO:0009067	7	1	0.659819725927739	aspartate family amino acid biosynthesis
GO:0007009	7	1	0.659819725927739	plasma membrane organization and biogenesis
GO:0048520	7	1	0.659819725927739	positive regulation of behavior
GO:0040017	7	1	0.659819725927739	positive regulation of locomotion
GO:0008637	7	1	0.659819725927739	apoptotic mitochondrial changes
GO:0050770	7	1	0.659819725927739	regulation of axonogenesis
GO:0035107	7	1	0.659819725927739	appendage morphogenesis
GO:0035108	7	1	0.659819725927739	limb morphogenesis
GO:0006043	7	1	0.659819725927739	glucosamine catabolism
GO:0006030	7	1	0.659819725927739	chitin metabolism
GO:0042829	7	1	0.659819725927739	defense response to pathogen
GO:0051272	7	1	0.659819725927739	positive regulation of cell motility
GO:0046348	7	1	0.659819725927739	amino sugar catabolism
GO:0045076	7	1	0.659819725927739	regulation of interleukin-2 biosynthesis
GO:0001704	7	1	0.659819725927739	formation of primary germ layer
GO:0001707	7	1	0.659819725927739	mesoderm formation
GO:0051094	31	4	0.664201274046555	positive regulation of development
GO:0016053	54	7	0.667508166256313	organic acid biosynthesis
GO:0046394	54	7	0.667508166256313	carboxylic acid biosynthesis
GO:0006470	99	13	0.671272720380204	protein amino acid dephosphorylation
GO:0006633	47	6	0.679303021457984	fatty acid biosynthesis
GO:0045934	159	21	0.684791493180698	negative regulation of nucleobase, nucleoside, nucleotide and nucleic acid metabolism
GO:0044272	24	3	0.686368336952336	sulfur compound biosynthesis
GO:0006809	16	2	0.688426782939765	nitric oxide biosynthesis

GO:0016338	16	2	0.688426782939765	calcium-independent cell-cell adhesion
GO:0046209	16	2	0.688426782939765	nitric oxide metabolism
GO:0045333	32	4	0.689247500093743	cellular respiration
GO:0007409	40	5	0.693978098278666	axonogenesis
GO:0000004	465	63	0.696764578371947	biological process unknown
GO:0006334	48	6	0.699476465721804	nucleosome assembly
GO:0006790	48	6	0.699476465721804	sulfur metabolism
GO:0007417	101	13	0.699659444529065	central nervous system development
GO:0031497	56	7	0.705280340379498	chromatin assembly
GO:0051329	79	10	0.707728000843859	interphase of mitotic cell cycle
GO:0006833	8	1	0.708406138479444	water transport
GO:0001502	8	1	0.708406138479444	cartilage condensation
GO:0006688	8	1	0.708406138479444	glycosphingolipid biosynthesis
GO:0010035	8	1	0.708406138479444	response to inorganic substance
GO:0031507	8	1	0.708406138479444	heterochromatin formation
GO:0007098	8	1	0.708406138479444	centrosome cycle
GO:0042094	8	1	0.708406138479444	interleukin-2 biosynthesis
GO:0042044	8	1	0.708406138479444	fluid transport
GO:0000097	8	1	0.708406138479444	sulfur amino acid biosynthesis
GO:0007369	8	1	0.708406138479444	gastrulation
GO:0007212	8	1	0.708406138479444	dopamine receptor signaling pathway
GO:0030071	8	1	0.708406138479444	regulation of mitotic metaphase/anaphase transition
GO:0030004	8	1	0.708406138479444	monovalent inorganic cation homeostasis
GO:0006098	8	1	0.708406138479444	pentose-phosphate shunt
GO:0051181	8	1	0.708406138479444	cofactor transport
GO:0006013	8	1	0.708406138479444	mannose metabolism
GO:0006342	8	1	0.708406138479444	chromatin silencing
GO:0006740	8	1	0.708406138479444	NADPH regeneration
GO:0006836	33	4	0.712938734908034	neurotransmitter transport
GO:0006401	33	4	0.712938734908034	RNA catabolism
GO:0006944	25	3	0.713503451259382	membrane fusion
GO:0006733	25	3	0.713503451259382	oxidoreduction coenzyme metabolism
GO:0001505	41	5	0.715185149968156	regulation of neurotransmitter levels
GO:0009309	49	6	0.718779705914272	amine biosynthesis
GO:0007160	49	6	0.718779705914272	cell-matrix adhesion
GO:0044271	49	6	0.718779705914272	nitrogen compound biosynthesis
GO:0006904	17	2	0.720804876844202	vesicle docking during exocytosis
GO:0046777	17	2	0.720804876844202	autophosphorylation
GO:0007165	2168	301	0.733877253525362	signal transduction
GO:0018193	50	6	0.737206209827265	peptidyl-amino acid modification
GO:0008643	26	3	0.738786952686375	carbohydrate transport
GO:0006811	515	69	0.739041281521629	ion transport
GO:0006461	194	25	0.741396051518243	protein complex assembly
GO:0045892	97	12	0.747043083325304	negative regulation of transcription, DNA-dependent
GO:0007167	165	21	0.748786980739088	enzyme linked receptor protein signaling pathway
GO:0006835	9	1	0.750057788246731	dicarboxylic acid transport

GO:0019059	9	1	0.750057788246731	initiation of viral infection
GO:0045767	9	1	0.750057788246731	regulation of anti-apoptosis
GO:0007091	9	1	0.750057788246731	mitotic metaphase/anaphase transition
GO:0045814	9	1	0.750057788246731	negative regulation of gene expression, epigenetic
GO:0019751	9	1	0.750057788246731	polyol metabolism
GO:0000060	9	1	0.750057788246731	protein-nucleus import, translocation
GO:0050864	9	1	0.750057788246731	regulation of B cell activation
GO:0009247	9	1	0.750057788246731	glycolipid biosynthesis
GO:0006071	9	1	0.750057788246731	glycerol metabolism
GO:0051297	9	1	0.750057788246731	centrosome organization and biogenesis
GO:0006309	9	1	0.750057788246731	DNA fragmentation during apoptosis
GO:0030101	9	1	0.750057788246731	natural killer cell activation
GO:0030260	9	1	0.750057788246731	entry into host cell
GO:0044409	9	1	0.750057788246731	entry into host
GO:0042982	9	1	0.750057788246731	amyloid precursor protein metabolism
GO:0031023	9	1	0.750057788246731	microtubule organizing center organization and biogenesis
GO:0046718	9	1	0.750057788246731	viral entry into host cell
GO:0030833	9	1	0.750057788246731	regulation of actin filament polymerization
GO:0043009	18	2	0.750295340410408	embryonic development (sensu Vertebrata)
GO:0031281	18	2	0.750295340410408	positive regulation of cyclase activity
GO:0048278	18	2	0.750295340410408	vesicle docking
GO:0045762	18	2	0.750295340410408	positive regulation of adenylate cyclase activity
GO:0007005	18	2	0.750295340410408	mitochondrion organization and biogenesis
GO:0042398	18	2	0.750295340410408	amino acid derivative biosynthesis
GO:0007190	18	2	0.750295340410408	adenylate cyclase activation
GO:0051349	18	2	0.750295340410408	positive regulation of lyase activity
GO:0016540	18	2	0.750295340410408	protein autoprocessing
GO:0006221	18	2	0.750295340410408	pyrimidine nucleotide biosynthesis
GO:0001701	18	2	0.750295340410408	embryonic development (sensu Mammalia)
GO:0030001	269	35	0.751075165777498	metal ion transport
GO:0051325	82	10	0.751401520418624	interphase
GO:0016481	151	19	0.758713288436519	negative regulation of transcription
GO:0007411	27	3	0.762266227643931	axon guidance
GO:0009792	27	3	0.762266227643931	embryonic development (sensu Metazoa)
GO:0016126	27	3	0.762266227643931	sterol biosynthesis
GO:0051336	60	7	0.771922155069724	regulation of hydrolase activity
GO:0006816	68	8	0.773364041036595	calcium ion transport
GO:0045786	145	18	0.774219970318381	negative regulation of progression through cell cycle
GO:0006664	19	2	0.77706226908064	glycolipid metabolism
GO:0007269	19	2	0.77706226908064	neurotransmitter secretion
GO:0006044	19	2	0.77706226908064	N-acetylglucosamine metabolism
GO:0006304	19	2	0.77706226908064	DNA modification
GO:0009991	19	2	0.77706226908064	response to extracellular stimulus
GO:0006570	10	1	0.785763818497198	tyrosine metabolism
GO:0006516	10	1	0.785763818497198	glycoprotein catabolism
GO:0006607	10	1	0.785763818497198	NLS-bearing substrate-nucleus import

GO:0010033	10	1	0.785763818497198	response to organic substance
GO:0019438	10	1	0.785763818497198	aromatic compound biosynthesis
GO:0009081	10	1	0.785763818497198	branched chain family amino acid metabolism
GO:0050435	10	1	0.785763818497198	beta-amyloid metabolism
GO:0007346	10	1	0.785763818497198	regulation of progression through mitotic cell cycle
GO:0042310	10	1	0.785763818497198	vasoconstriction
GO:0042345	10	1	0.785763818497198	regulation of NF-kappaB-nucleus import
GO:0042348	10	1	0.785763818497198	NF-kappaB-nucleus import
GO:0009405	10	1	0.785763818497198	pathogenesis
GO:0042176	10	1	0.785763818497198	regulation of protein catabolism
GO:0042559	10	1	0.785763818497198	pteridine and derivative biosynthesis
GO:0000082	53	6	0.787248261773173	G1/S transition of mitotic cell cycle
GO:0016051	69	8	0.787284327731802	carbohydrate biosynthesis
GO:0000075	37	4	0.794381767539581	cell cycle checkpoint
GO:0006487	37	4	0.794381767539581	protein amino acid N-linked glycosylation
GO:0046903	208	26	0.797479401776205	secretion
GO:0008629	20	2	0.801281757764998	induction of apoptosis by intracellular signals
GO:0009190	20	2	0.801281757764998	cyclic nucleotide biosynthesis
GO:0006041	20	2	0.801281757764998	glucosamine metabolism
GO:0016311	110	13	0.807948452505448	dephosphorylation
GO:0006821	38	4	0.811555849530747	chloride transport
GO:0007178	38	4	0.811555849530747	transmembrane receptor protein serine/threonine kinase signaling pathway
GO:0009165	103	12	0.816289955481928	nucleotide biosynthesis
GO:0046546	11	1	0.816372379565377	development of primary male sexual characteristics
GO:0048489	11	1	0.816372379565377	synaptic vesicle transport
GO:0008584	11	1	0.816372379565377	male gonad development
GO:0007250	11	1	0.816372379565377	activation of NF-kappaB-inducing kinase
GO:0042558	11	1	0.816372379565377	pteridine and derivative metabolism
GO:0030148	11	1	0.816372379565377	sphingolipid biosynthesis
GO:0030262	11	1	0.816372379565377	apoptotic nuclear changes
GO:0051129	30	3	0.82254046679779	negative regulation of cell organization and biogenesis
GO:0051261	30	3	0.82254046679779	protein depolymerization
GO:0030203	30	3	0.82254046679779	glycosaminoglycan metabolism
GO:0006695	21	2	0.823135140399459	cholesterol biosynthesis
GO:0007163	21	2	0.823135140399459	establishment and/or maintenance of cell polarity
GO:0007017	112	13	0.827614190935546	microtubule-based process
GO:0006752	48	5	0.834539012364431	group transfer coenzyme metabolism
GO:0000165	81	9	0.835535998535935	MAPKKK cascade
GO:0006022	31	3	0.839505380306852	aminoglycan metabolism
GO:0006367	31	3	0.839505380306852	transcription initiation from RNA polymerase II promoter
GO:0007049	601	78	0.84100355624752	cell cycle
GO:0006563	12	1	0.84261071264821	L-serine metabolism
GO:0006672	12	1	0.84261071264821	ceramide metabolism
GO:0046039	12	1	0.84261071264821	GTP metabolism
GO:0006414	12	1	0.84261071264821	translational elongation
GO:0006183	12	1	0.84261071264821	GTP biosynthesis

GO:0007189	22	2	0.842803912327325	G-protein signaling, adenylate cyclase activating pathway
GO:0042770	22	2	0.842803912327325	DNA damage response, signal transduction
GO:0006471	22	2	0.842803912327325	protein amino acid ADP-ribosylation
GO:0006446	23	2	0.86046599341805	regulation of translational initiation
GO:0007018	59	6	0.865061961563839	microtubule-based movement
GO:0030705	59	6	0.865061961563839	cytoskeleton-dependent intracellular transport
GO:0008037	13	1	0.865102374665555	cell recognition
GO:0015807	13	1	0.865102374665555	L-amino acid transport
GO:0007043	13	1	0.865102374665555	intercellular junction assembly
GO:0016458	13	1	0.865102374665555	gene silencing
GO:0007214	13	1	0.865102374665555	gamma-aminobutyric acid signaling pathway
GO:0006720	13	1	0.865102374665555	isoprenoid metabolism
GO:0006892	13	1	0.865102374665555	post-Golgi transport
GO:0006338	33	3	0.869260355353742	chromatin remodeling
GO:0044249	763	99	0.870166169075121	cellular biosynthesis
GO:0000074	394	49	0.873668561016485	regulation of progression through cell cycle
GO:0006040	24	2	0.87629305309675	amino sugar metabolism
GO:0051128	52	5	0.882041624288239	regulation of cell organization and biogenesis
GO:0007420	34	3	0.882226405257112	brain development
GO:0046519	14	1	0.884382010884216	sphingoid metabolism
GO:0006584	14	1	0.884382010884216	catecholamine metabolism
GO:0006541	14	1	0.884382010884216	glutamine metabolism
GO:0008213	14	1	0.884382010884216	protein amino acid alkylation
GO:0042401	14	1	0.884382010884216	biogenic amine biosynthesis
GO:0009220	14	1	0.884382010884216	pyrimidine ribonucleotide biosynthesis
GO:0007623	14	1	0.884382010884216	circadian rhythm
GO:0030183	14	1	0.884382010884216	B cell differentiation
GO:0006479	14	1	0.884382010884216	protein amino acid methylation
GO:0006164	61	6	0.885086074120656	purine nucleotide biosynthesis
GO:0006813	111	12	0.885297384020208	potassium ion transport
GO:0007126	44	4	0.891393974809256	meiosis
GO:0051321	44	4	0.891393974809256	meiotic cell cycle
GO:0051327	44	4	0.891393974809256	M phase of meiotic cell cycle
GO:0009058	859	111	0.893564370485957	biosynthesis
GO:0042157	35	3	0.894033897245134	lipoprotein metabolism
GO:0046661	15	1	0.900908024187636	male sex differentiation
GO:0006687	15	1	0.900908024187636	glycosphingolipid metabolism
GO:0045216	15	1	0.900908024187636	intercellular junction assembly and/or maintenance
GO:0018958	15	1	0.900908024187636	phenol metabolism
GO:0009066	15	1	0.900908024187636	aspartate family amino acid metabolism
GO:0007020	15	1	0.900908024187636	microtubule nucleation
GO:0009218	15	1	0.900908024187636	pyrimidine ribonucleotide metabolism
GO:0042990	15	1	0.900908024187636	regulation of transcription factor-nucleus import
GO:0042991	15	1	0.900908024187636	transcription factor-nucleus import
GO:0006163	63	6	0.902536858706696	purine nucleotide metabolism
GO:0006445	64	6	0.91037343721152	regulation of translation

GO:0016485	37	3	0.914506858916359	protein processing
GO:0008286	16	1	0.915073440270828	insulin receptor signaling pathway
GO:0042306	16	1	0.915073440270828	regulation of protein-nucleus import
GO:0009260	65	6	0.917657987121203	ribonucleotide biosynthesis
GO:0043087	28	2	0.924379765779992	regulation of GTPase activity
GO:0045185	17	1	0.927215225484992	maintenance of protein localization
GO:0051051	17	1	0.927215225484992	negative regulation of transport
GO:0007215	17	1	0.927215225484992	glutamate signaling pathway
GO:0051223	17	1	0.927215225484992	regulation of protein transport
GO:0009152	58	5	0.93125422226966	purine ribonucleotide biosynthesis
GO:0030521	29	2	0.933291780950598	androgen receptor signaling pathway
GO:0008624	29	2	0.933291780950598	induction of apoptosis by extracellular signals
GO:0009259	68	6	0.936495966286552	ribonucleotide metabolism
GO:0007188	59	5	0.937383135297977	G-protein signaling, coupled to cAMP nucleotide second messenger
GO:0051216	18	1	0.937622277548202	cartilage development
GO:0006986	40	3	0.938525034779553	response to unfolded protein
GO:0045944	30	2	0.94120258910437	positive regulation of transcription from RNA polymerase II promoter
GO:0016055	87	8	0.942337628062255	Wnt receptor signaling pathway
GO:0009150	60	5	0.943016981329422	purine ribonucleotide metabolism
GO:0019933	60	5	0.943016981329422	cAMP-mediated signaling
GO:0006413	41	3	0.9450300225991	translational initiation
GO:0007131	19	1	0.946542278018623	meiotic recombination
GO:0015672	223	24	0.95106854250137	monovalent inorganic cation transport
GO:0006357	240	26	0.953358371168474	regulation of transcription from RNA polymerase II promoter
GO:0006289	20	1	0.954187568558287	nucleotide-excision repair
GO:0031279	32	2	0.954427315761862	regulation of cyclase activity
GO:0045761	32	2	0.954427315761862	regulation of adenylate cyclase activity
GO:0051339	32	2	0.954427315761862	regulation of lyase activity
GO:0009108	72	6	0.955622255128136	coenzyme biosynthesis
GO:0006812	346	39	0.959292515934076	cation transport
GO:0051179	1857	242	0.960980954957732	localization
GO:0006486	92	8	0.961745203527138	protein amino acid glycosylation
GO:0006665	34	2	0.96477793043832	sphingolipid metabolism
GO:0043413	93	8	0.964834962070186	biopolymer glycosylation
GO:0051056	65	5	0.96488463948219	regulation of small GTPase mediated signal transduction
GO:0009100	111	10	0.965062760482521	glycoprotein metabolism
GO:0051090	22	1	0.966356203187129	regulation of transcription factor activity
GO:0007127	22	1	0.966356203187129	meiosis I
GO:0016197	22	1	0.966356203187129	endosome transport
GO:0007187	94	8	0.967697395951255	G-protein signaling, coupled to cyclic nucleotide second messenger
GO:0040029	35	2	0.969065447601544	regulation of gene expression, epigenetic
GO:0009581	313	34	0.970427008844296	detection of external stimulus
GO:0015992	57	4	0.971104574314074	proton transport
GO:0046822	23	1	0.971169394540269	regulation of nucleocytoplasmic transport
GO:0045893	77	6	0.972140781955072	positive regulation of transcription, DNA-dependent
GO:0050953	149	14	0.972173413786976	sensory perception of light

GO:0007601	149	14	0.972173413786976	visual perception
GO:0006352	47	3	0.972390689287807	transcription initiation
GO:0006511	105	9	0.972480711082096	ubiquitin-dependent protein catabolism
GO:0045935	105	9	0.972480711082096	positive regulation of nucleobase, nucleoside, nucleotide and nucleic acid metabolism
GO:0019941	105	9	0.972480711082096	modification-dependent protein catabolism
GO:0009101	96	8	0.972797153704622	glycoprotein biosynthesis
GO:0007243	242	25	0.973311007444682	protein kinase cascade
GO:0043123	68	5	0.973982858876453	positive regulation of I-kappaB kinase/NF-kappaB cascade
GO:0006818	58	4	0.97405645224329	hydrogen transport
GO:0007032	24	1	0.9752944528639	endosome organization and biogenesis
GO:0006457	169	16	0.977089305687028	protein folding
GO:0019935	98	8	0.977151613576268	cyclic-nucleotide-mediated signaling
GO:0030518	38	2	0.979117597473982	steroid hormone receptor signaling pathway
GO:0051234	1841	236	0.979892101037633	establishment of localization
GO:0045941	100	8	0.980857506526492	positive regulation of transcription
GO:0006333	91	7	0.981652172231456	chromatin assembly or disassembly
GO:0030522	39	2	0.981701575533009	intracellular receptor-mediated signaling pathway
GO:0019226	198	19	0.981728179218027	transmission of nerve impulse
GO:0006644	73	5	0.984437636830518	phospholipid metabolism
GO:0043122	73	5	0.984437636830518	regulation of I-kappaB kinase/NF-kappaB cascade
GO:0007268	192	18	0.984636849151854	synaptic transmission
GO:0006606	53	3	0.986485611020538	protein-nucleus import
GO:0046467	53	3	0.986485611020538	membrane lipid biosynthesis
GO:0043414	28	1	0.98668084077699	biopolymer methylation
GO:0007582	7846	1095	0.986745575049278	physiological process
GO:0007254	42	2	0.987725187808702	JNK cascade
GO:0031098	42	2	0.987725187808702	stress-activated protein kinase signaling pathway
GO:0051170	54	3	0.988028419516442	nuclear import
GO:0044260	2298	296	0.988063324360631	cellular macromolecule metabolism
GO:0006643	106	8	0.988903468317739	membrane lipid metabolism
GO:0008654	43	2	0.989264773102818	phospholipid biosynthesis
GO:0043170	2405	309	0.991392805551112	macromolecule metabolism
GO:0007249	99	7	0.99146579860432	I-kappaB kinase/NF-kappaB cascade
GO:0007600	287	28	0.991973436327307	sensory perception
GO:0035023	46	2	0.992836997157275	regulation of Rho protein signal transduction
GO:0006810	1802	225	0.993587184167608	transport
GO:0043037	122	9	0.993839531997216	translation
GO:0043038	33	1	0.993849670056164	amino acid activation
GO:0043039	33	1	0.993849670056164	tRNA aminoacylation
GO:0006418	33	1	0.993849670056164	tRNA aminoacylation for protein translation
GO:0006310	61	3	0.994951311672149	DNA recombination
GO:0006468	444	46	0.995001770986084	protein amino acid phosphorylation
GO:0015931	49	2	0.995237236408668	nucleobase, nucleoside, nucleotide and nucleic acid transport
GO:0009199	49	2	0.995237236408668	ribonucleoside triphosphate metabolism
GO:0009144	49	2	0.995237236408668	purine nucleoside triphosphate metabolism
GO:0009205	49	2	0.995237236408668	purine ribonucleoside triphosphate metabolism

GO:0016192	296	28	0.995471433018175	vesicle-mediated transport
GO:0007264	227	20	0.995583815535976	small GTPase mediated signal transduction
GO:0009141	50	2	0.995845968018464	nucleoside triphosphate metabolism
GO:0006406	36	1	0.996132283943746	mRNA-nucleus export
GO:0019538	2129	267	0.996265768613553	protein metabolism
GO:0017038	64	3	0.996537672397153	protein import
GO:0006364	64	3	0.996537672397153	rRNA processing
GO:0051028	37	1	0.996686473138556	mRNA transport
GO:0046034	37	1	0.996686473138556	ATP metabolism
GO:0006400	37	1	0.996686473138556	tRNA modification
GO:0009719	214	18	0.99693860313277	response to endogenous stimulus
GO:0044267	2109	263	0.997180878869399	cellular protein metabolism
GO:0016072	66	3	0.997313194880235	rRNA metabolism
GO:0007242	879	99	0.997488347775604	intracellular signaling cascade
GO:0045045	161	12	0.997544198136542	secretory pathway
GO:0007046	79	4	0.997655484334898	ribosome biogenesis
GO:0006366	426	42	0.997856849084905	transcription from RNA polymerase II promoter
GO:0042254	80	4	0.997920613326571	ribosome biogenesis and assembly
GO:0006405	41	1	0.998215348699677	RNA-nucleus export
GO:0050657	42	1	0.998471206282384	nucleic acid transport
GO:0050658	42	1	0.998471206282384	RNA transport
GO:0051236	42	1	0.998471206282384	establishment of RNA localization
GO:0006888	58	2	0.998624514495442	ER to Golgi transport
GO:0006403	43	1	0.998690407072374	RNA localization
GO:0007028	96	5	0.998779150584468	cytoplasm organization and biogenesis
GO:0016043	842	92	0.998872996704024	cell organization and biogenesis
GO:0009059	464	45	0.99901437702762	macromolecule biosynthesis
GO:0051168	45	1	0.999039080688029	nuclear export
GO:0050877	475	46	0.99915942217445	neurophysiological process
GO:0009142	46	1	0.99917690476649	nucleoside triphosphate biosynthesis
GO:0009145	46	1	0.99917690476649	purine nucleoside triphosphate biosynthesis
GO:0009201	46	1	0.99917690476649	ribonucleoside triphosphate biosynthesis
GO:0009206	46	1	0.99917690476649	purine ribonucleoside triphosphate biosynthesis
GO:0006793	641	66	0.999207637269645	phosphorus metabolism
GO:0006796	641	66	0.999207637269645	phosphate metabolism
GO:0006281	174	12	0.999254791547305	DNA repair
GO:0007266	63	2	0.999316703062828	Rho protein signal transduction
GO:0006913	102	5	0.999395494882833	nucleocytoplasmic transport
GO:0009451	48	1	0.999396117927846	RNA modification
GO:0051276	236	18	0.999488786470369	chromosome organization and biogenesis
GO:0006412	407	37	0.999515627077489	protein biosynthesis
GO:0006974	200	14	0.999585978147356	response to DNA damage stimulus
GO:0006323	193	13	0.999684010297582	DNA packaging
GO:0051169	96	4	0.999710472340066	nuclear transport
GO:0016310	522	49	0.999783377874438	phosphorylation
GO:0006259	489	45	0.99979873868146	DNA metabolism

GO:0048193	86	3	0.999802926476791	Golgi vesicle transport
GO:0007606	56	1	0.999825161805527	sensory perception of chemical stimulus
GO:0007001	221	15	0.999853165124092	chromosome organization and biogenesis (sensu Eukaryota)
GO:0006996	693	68	0.999894527463949	organelle organization and biogenesis
GO:0050791	2407	289	0.999926920085747	regulation of physiological process
GO:0006399	62	1	0.999931046108289	tRNA metabolism
GO:0006325	186	10	0.999973672955248	establishment and/or maintenance of chromatin architecture
GO:0050789	2689	319	0.999993355096198	regulation of biological process
GO:0006464	1087	110	0.999994148670612	protein modification
GO:0043283	2240	258	0.9999949438043	biopolymer metabolism
GO:0000398	119	3	0.99999785857608	nuclear mRNA splicing, via spliceosome
GO:0000375	119	3	0.99999785857608	RNA splicing, via transesterification reactions
GO:0000377	119	3	0.99999785857608	RNA splicing, via transesterification reactions with bulged adenosine as nucleophile
GO:0006512	255	14	0.999998301585745	ubiquitin cycle
GO:0016568	122	3	0.99999859355825	chromatin modification
GO:0043412	1160	113	0.999999674484877	biopolymer modification
GO:0006605	134	3	0.999999741480493	protein targeting
GO:0008380	137	3	0.9999998312059	RNA splicing
GO:0006886	257	12	0.999999885576145	intracellular protein transport
GO:0051244	2319	257	0.999999920957418	regulation of cellular physiological process
GO:0008104	474	32	0.999999948637683	protein localization
GO:0050794	2488	278	0.999999951493017	regulation of cellular process
GO:0006397	180	5	0.999999972378465	mRNA processing
GO:0045184	459	29	0.999999987419774	establishment of protein localization
GO:0008152	5262	653	0.999999998850327	metabolism
GO:0016071	205	5	0.99999999909682	mRNA metabolism
GO:0044237	5026	618	0.999999999147301	cellular metabolism
GO:0046907	448	25	0.999999999419499	intracellular transport
GO:0015031	439	23	0.99999999984758	protein transport
GO:0016070	391	17	0.99999999998364	RNA metabolism
GO:0006396	326	10	0.99999999999901	RNA processing
GO:0050875	7018	898	0.999999999999855	cellular physiological process
GO:0009987	8200	1091	0.999999999999996	cellular process
GO:0019222	1629	138	0.999999999999998	regulation of metabolism
GO:0031323	1566	130	0.999999999999999	regulation of cellular metabolism
GO:0044238	4820	557	0.999999999999999	primary metabolism
GO:0045449	1433	106	1	regulation of transcription
GO:0008150	9032	1289	1	biological_process
GO:0019219	1458	111	1	regulation of nucleobase, nucleoside, nucleotide and nucleic acid metabolism
GO:0006350	1499	109	1	transcription
GO:0006351	1407	104	1	transcription, DNA-dependent
GO:0006355	1359	100	1	regulation of transcription, DNA-dependent
GO:0006139	2328	184	1	nucleobase, nucleoside, nucleotide and nucleic acid metabolism

0.3 GO: Molecular Function

GO ID	genes present in chip	genes present in tissues	p-value	GO term
GO:0005179	90	40	2.69837934862105e-12	hormone activity
GO:0030246	181	62	4.54526986324209e-12	carbohydrate binding
GO:0004866	99	42	5.09510562510617e-12	endopeptidase inhibitor activity
GO:0030414	100	42	7.60509627046037e-12	protease inhibitor activity
GO:0004857	187	61	6.96899164135103e-11	enzyme inhibitor activity
GO:0008307	33	21	7.95249852274788e-11	structural constituent of muscle
GO:0005198	488	118	8.25958949250818e-10	structural molecule activity
GO:0008201	60	28	1.31105271824667e-09	heparin binding
GO:0008009	40	22	1.50194518857362e-09	chemokine activity
GO:0042379	40	22	1.50194518857362e-09	chemokine receptor binding
GO:0005102	528	124	2.08853774214639e-09	receptor binding
GO:0030247	80	33	2.34816529546826e-09	polysaccharide binding
GO:0001871	88	35	2.48092872569938e-09	pattern binding
GO:0005539	77	32	3.2843264886564e-09	glycosaminoglycan binding
GO:0001664	42	22	5.11981139732165e-09	G-protein-coupled receptor binding
GO:0004252	105	38	1.16611878144311e-08	serine-type endopeptidase activity
GO:0004867	64	27	3.78615196852025e-08	serine-type endopeptidase inhibitor activity
GO:0005200	86	32	7.80604398959678e-08	structural constituent of cytoskeleton
GO:0008236	117	39	9.98539205586558e-08	serine-type peptidase activity
GO:0003823	30	16	4.73753985333897e-07	antigen binding
GO:0019825	31	16	8.5110202501347e-07	oxygen binding
GO:0005509	646	129	1.51049750035206e-05	calcium ion binding
GO:0008233	399	86	2.55394752510016e-05	peptidase activity
GO:0005506	212	52	3.24486003128162e-05	iron ion binding
GO:0005125	181	46	3.57808564444134e-05	cytokine activity
GO:0015020	21	11	3.87473706822769e-05	glucuronosyltransferase activity
GO:0016877	12	8	4.53303484281012e-05	ligase activity, forming carbon-sulfur bonds
GO:0004181	18	10	4.54216855482631e-05	metallocarboxypeptidase activity
GO:0005529	103	30	5.71673991991461e-05	sugar binding
GO:0016491	509	103	6.55238022449466e-05	oxidoreductase activity
GO:0004871	1837	312	7.12957507467554e-05	signal transducer activity
GO:0004869	19	10	8.37715740206334e-05	cysteine protease inhibitor activity
GO:0003779	189	46	0.000110895415043167	actin binding
GO:0048503	92	27	0.000114716902896575	GPI anchor binding
GO:0004180	27	12	0.000133652076778126	carboxypeptidase activity
GO:0016209	31	13	0.00014515300860654	antioxidant activity
GO:0015645	8	6	0.000170638779282077	fatty-acid ligase activity
GO:0004467	8	6	0.000170638779282077	long-chain-fatty-acid-CoA ligase activity
GO:0008235	44	16	0.000192565293137170	metalloexopeptidase activity
GO:0004175	288	63	0.000197272485894124	endopeptidase activity
GO:0004182	14	8	0.000211054082219669	carboxypeptidase A activity
GO:0042708	6	5	0.000296239943575538	elastase activity

GO:0005201	73	22	0.000317566116340259	extracellular matrix structural constituent
GO:0004601	22	10	0.000391453124829876	peroxidase activity
GO:0016684	22	10	0.000391453124829876	oxidoreductase activity, acting on peroxide as acceptor
GO:0046906	74	22	0.000392658253649064	tetrapyrrole binding
GO:0020037	74	22	0.000392658253649064	heme binding
GO:0000146	15	8	0.000396360031698962	microfilament motor activity
GO:0008132	4	4	0.000396937741864712	pancreatic elastase activity
GO:0004806	9	6	0.000450506019035902	triacylglycerol lipase activity
GO:0008092	279	59	0.000771538523467229	cytoskeletal protein binding
GO:0008289	298	62	0.000889005766445067	lipid binding
GO:0042803	64	19	0.000976021065700912	protein homodimerization activity
GO:0046983	170	39	0.00122577022388988	protein dimerization activity
GO:0035091	132	32	0.00125388131741073	phosphoinositide binding
GO:0008238	68	19	0.00215057401686349	exopeptidase activity
GO:0019842	59	17	0.00252773746094931	vitamin binding
GO:0019834	3	3	0.00281486407576626	phospholipase A2 inhibitor activity
GO:0005344	12	6	0.00338576157777563	oxygen transporter activity
GO:0019865	12	6	0.00338576157777563	immunoglobulin binding
GO:0016705	87	22	0.00396351823637734	oxidoreductase activity, acting on paired donors, with incorporation or reduction of molecular oxygen
GO:0050381	20	8	0.00402895583540347	unspecific monooxygenase activity
GO:0005520	20	8	0.00402895583540347	insulin-like growth factor binding
GO:0008367	9	5	0.00429060546845034	bacterial binding
GO:0030234	509	93	0.00445375552553874	enzyme regulator activity
GO:0016712	25	9	0.00528977901273064	oxidoreductase activity, acting on paired donors, with incorporation or reduction of molecular oxygen, reduced flavin or flavoprotein as one donor, and incorporation of one atom of oxygen
GO:0008237	133	30	0.00551405538151843	metallopeptidase activity
GO:0016814	21	8	0.0057139982491577	hydrolase activity, acting on carbon-nitrogen (but not peptide) bonds, in cyclic amidines
GO:0005507	49	14	0.00637795156212201	copper ion binding
GO:0005543	169	36	0.00674513107059774	phospholipid binding
GO:0042802	158	34	0.00708280056874676	protein self binding
GO:0005178	26	9	0.00709414507045798	integrin binding
GO:0005246	7	4	0.00972328584445107	calcium channel regulator activity
GO:0016840	7	4	0.00972328584445107	carbon-nitrogen lyase activity
GO:0005499	4	3	0.0100686430774709	vitamin D binding
GO:0008147	4	3	0.0100686430774709	structural constituent of bone
GO:0019238	4	3	0.0100686430774709	cyclohydrolase activity
GO:0004024	4	3	0.0100686430774709	alcohol dehydrogenase activity, zinc-dependent
GO:0016841	4	3	0.0100686430774709	ammonia-lyase activity
GO:0030492	4	3	0.0100686430774709	hemoglobin binding
GO:0005496	42	12	0.0111663980449165	steroid binding
GO:0016229	15	6	0.0125786532412355	steroid dehydrogenase activity
GO:0003774	98	22	0.0168207825898841	motor activity
GO:0016805	8	4	0.0172893548034598	dipeptidase activity
GO:0016634	8	4	0.0172893548034598	oxidoreductase activity, acting on the CH-CH group of donors, oxygen as acceptor
GO:0004089	16	6	0.0177650004744852	carbonate dehydratase activity
GO:0004497	71	17	0.0181718721348831	monooxygenase activity

GO:0045012	12	5	0.0186901225253049	MHC class II receptor activity
GO:0005515	3709	559	0.0190543489621213	protein binding
GO:0016616	77	18	0.0195018684134203	oxidoreductase activity, acting on the CH-OH group of donors, NAD or NADP as acceptor
GO:0004611	2	2	0.0199487283943049	phosphoenolpyruvate carboxykinase activity
GO:0004613	2	2	0.0199487283943049	phosphoenolpyruvate carboxykinase (GTP) activity
GO:0004619	2	2	0.0199487283943049	phosphoglycerate mutase activity
GO:0008368	2	2	0.0199487283943049	Gram-negative bacterial binding
GO:0048306	2	2	0.0199487283943049	calcium-dependent protein binding
GO:0048156	2	2	0.0199487283943049	tau protein binding
GO:0004998	2	2	0.0199487283943049	transferrin receptor activity
GO:0005218	2	2	0.0199487283943049	intracellular ligand-gated calcium channel activity
GO:0005219	2	2	0.0199487283943049	ryanodine-sensitive calcium-release channel activity
GO:0047026	2	2	0.0199487283943049	3-alpha-hydroxysteroid dehydrogenase (A-specific) activity
GO:0003870	2	2	0.0199487283943049	5-aminolevulinate synthase activity
GO:0019855	2	2	0.0199487283943049	calcium channel inhibitor activity
GO:0042132	2	2	0.0199487283943049	fructose-bisphosphatase activity
GO:0051373	2	2	0.0199487283943049	FATZ binding
GO:0004082	2	2	0.0199487283943049	bisphosphoglycerate mutase activity
GO:0004083	2	2	0.0199487283943049	bisphosphoglycerate phosphatase activity
GO:0016725	2	2	0.0199487283943049	oxidoreductase activity, acting on CH2 groups
GO:0016728	2	2	0.0199487283943049	oxidoreductase activity, acting on CH2 groups, disulfide as acceptor
GO:0042805	2	2	0.0199487283943049	actinin binding
GO:0016749	2	2	0.0199487283943049	N-succinyltransferase activity
GO:0004357	2	2	0.0199487283943049	glutamate-cysteine ligase activity
GO:0004488	2	2	0.0199487283943049	methylenetetrahydrofolate dehydrogenase (NADP+) activity
GO:0004477	2	2	0.0199487283943049	methenyltetrahydrofolate cyclohydrolase activity
GO:0004281	2	2	0.0199487283943049	pancreatic elastase II activity
GO:0004232	2	2	0.0199487283943049	interstitial collagenase activity
GO:0015266	2	2	0.0199487283943049	protein channel activity
GO:0004748	2	2	0.0199487283943049	ribonucleoside-diphosphate reductase activity
GO:0046982	61	15	0.0202653591456442	protein heterodimerization activity
GO:0017022	5	3	0.0225302049065502	myosin binding
GO:0008133	5	3	0.0225302049065502	collagenase activity
GO:0019864	5	3	0.0225302049065502	IgG binding
GO:0019841	5	3	0.0225302049065502	retinol binding
GO:0016878	5	3	0.0225302049065502	acid-thiol ligase activity
GO:0004465	5	3	0.0225302049065502	lipoprotein lipase activity
GO:0030106	26	8	0.0229965048999542	MHC class I receptor activity
GO:0005504	17	6	0.024245917396985	fatty acid binding
GO:0004522	9	4	0.0276883542714674	pancreatic ribonuclease activity
GO:0005523	9	4	0.0276883542714674	tropomyosin binding
GO:0019840	9	4	0.0276883542714674	isoprenoid binding
GO:0005501	9	4	0.0276883542714674	retinoid binding
GO:0030674	69	16	0.0286215237259842	protein binding, bridging
GO:0016627	37	10	0.0288315661923007	oxidoreductase activity, acting on the CH-CH group of donors
GO:0019955	65	15	0.0347341968161742	cytokine binding

GO:0019838	49	12	0.0366973137778672	growth factor binding
GO:0016628	14	5	0.0371034205502137	oxidoreductase activity, acting on the CH-CH group of donors, NAD or NADP as acceptor
GO:0016614	83	18	0.0393295448493981	oxidoreductase activity, acting on CH-OH group of donors
GO:0008329	6	3	0.0403700841259972	pattern recognition receptor activity
GO:0004303	6	3	0.0403700841259972	estradiol 17-beta-dehydrogenase activity
GO:0016918	6	3	0.0403700841259972	retinal binding
GO:0015278	6	3	0.0403700841259972	calcium-release channel activity
GO:0008603	10	4	0.0410874847232161	cAMP-dependent protein kinase regulator activity
GO:0004033	10	4	0.0410874847232161	aldo-keto reductase activity
GO:0001614	20	6	0.0524542056254119	purinergic nucleotide receptor activity
GO:0001608	20	6	0.0524542056254119	nucleotide receptor activity, G-protein coupled
GO:0016502	20	6	0.0524542056254119	nucleotide receptor activity
GO:0045028	20	6	0.0524542056254119	purinergic nucleotide receptor activity, G-protein coupled
GO:0015643	3	2	0.0542164570313822	toxin binding
GO:0005176	3	2	0.0542164570313822	ErbB-2 class receptor binding
GO:0001948	3	2	0.0542164570313822	glycoprotein binding
GO:0008253	3	2	0.0542164570313822	5'-nucleotidase activity
GO:0003854	3	2	0.0542164570313822	3-beta-hydroxy-delta5-steroid dehydrogenase activity
GO:0047115	3	2	0.0542164570313822	trans-1,2-dihydrobenzene-1,2-diol dehydrogenase activity
GO:0016174	3	2	0.0542164570313822	NAD(P)H oxidase activity
GO:0016748	3	2	0.0542164570313822	succinyltransferase activity
GO:0030197	3	2	0.0542164570313822	extracellular matrix constituent, lubricant activity
GO:0004486	3	2	0.0542164570313822	methylenetetrahydrofolate dehydrogenase activity
GO:0004111	3	2	0.0542164570313822	creatine kinase activity
GO:0004293	3	2	0.0542164570313822	tissue kallikrein activity
GO:0004295	3	2	0.0542164570313822	trypsin activity
GO:0004237	3	2	0.0542164570313822	membrane dipeptidase activity
GO:0004769	3	2	0.0542164570313822	steroid delta-isomerase activity
GO:0004774	3	2	0.0542164570313822	succinate-CoA ligase activity
GO:0004720	3	2	0.0542164570313822	protein-lysine 6-oxidase activity
GO:0016892	11	4	0.0575298728714343	endoribonuclease activity, producing 3'-phosphomonoesters
GO:0016641	11	4	0.0575298728714343	oxidoreductase activity, acting on the CH-NH2 group of donors, oxygen as acceptor
GO:0016646	11	4	0.0575298728714343	oxidoreductase activity, acting on the CH-NH group of donors, NAD or NADP as acceptor
GO:0008200	7	3	0.0633551828371569	ion channel inhibitor activity
GO:0019203	7	3	0.0633551828371569	carbohydrate phosphatase activity
GO:0016248	7	3	0.0633551828371569	channel inhibitor activity
GO:0015101	7	3	0.0633551828371569	organic cation transporter activity
GO:0004896	48	11	0.0675910100946496	hematopoietin/interferon-class (D200-domain) cytokine receptor activity
GO:0016810	72	15	0.075853738496092	hydrolase activity, acting on carbon-nitrogen (but not peptide) bonds
GO:0008395	12	4	0.0769497480444989	steroid hydroxylase activity
GO:0017124	12	4	0.0769497480444989	SH3 domain binding
GO:0016493	12	4	0.0769497480444989	C-C chemokine receptor activity
GO:0019957	12	4	0.0769497480444989	C-C chemokine binding
GO:0001637	22	6	0.0791092166802771	G-protein chemoattractant receptor activity
GO:0004950	22	6	0.0791092166802771	chemokine receptor activity
GO:0009055	104	20	0.0904710501709608	electron carrier activity

GO:0004859	8	3	0.0909946796573752	phospholipase inhibitor activity
GO:0017017	8	3	0.0909946796573752	MAP kinase phosphatase activity
GO:0004022	8	3	0.0909946796573752	alcohol dehydrogenase activity
GO:0005497	4	2	0.0983642709852934	androgen binding
GO:0000099	4	2	0.0983642709852934	sulfur amino acid transporter activity
GO:0050664	4	2	0.0983642709852934	oxidoreductase activity, acting on NADH or NADPH, with oxygen as acceptor
GO:0003918	4	2	0.0983642709852934	DNA topoisomerase (ATP-hydrolyzing) activity
GO:0016405	4	2	0.0983642709852934	CoA-ligase activity
GO:0016713	4	2	0.0983642709852934	oxidoreductase activity, acting on paired donors, with incorporation or reduction of molecular oxygen, reduced iron-sulfur protein as one donor, and incorporation of one atom of oxygen
GO:0015184	4	2	0.0983642709852934	L-cystine transporter activity
GO:0019239	18	5	0.0986613956113253	deaminase activity
GO:0016620	18	5	0.0986613956113253	oxidoreductase activity, acting on the aldehyde or oxo group of donors, NAD or NADP as acceptor
GO:0016645	18	5	0.0986613956113253	oxidoreductase activity, acting on the CH-NH group of donors
GO:0015926	13	4	0.0991908615984268	glucosidase activity
GO:0016894	13	4	0.0991908615984268	endonuclease activity, active with either ribo- or deoxyribonucleic acids and producing 3'-phosphomonoesters
GO:0015026	13	4	0.0991908615984268	coreceptor activity
GO:0016526	13	4	0.0991908615984268	G-protein coupled receptor activity, unknown ligand
GO:0005516	112	21	0.103818919023844	calmodulin binding
GO:0019210	29	7	0.104463837588003	kinase inhibitor activity
GO:0019207	58	12	0.109007763244826	kinase regulator activity
GO:0019956	24	6	0.112049321894794	chemokine binding
GO:0019887	47	10	0.117821647402935	protein kinase regulator activity
GO:0019965	30	7	0.120515994434063	interleukin binding
GO:0051018	9	3	0.122647842350329	protein kinase A binding
GO:0016494	9	3	0.122647842350329	C-X-C chemokine receptor activity
GO:0003993	9	3	0.122647842350329	acid phosphatase activity
GO:0016638	14	4	0.124025838017712	oxidoreductase activity, acting on the CH-NH2 group of donors
GO:0048038	1	1	0.141284980609998	quinone binding
GO:0001875	1	1	0.141284980609998	lipopolysaccharide receptor activity
GO:0004850	1	1	0.141284980609998	uridine phosphorylase activity
GO:0004853	1	1	0.141284980609998	uroporphyrinogen decarboxylase activity
GO:0004833	1	1	0.141284980609998	tryptophan 2,3-dioxygenase activity
GO:0001532	1	1	0.141284980609998	interleukin-21 receptor activity
GO:0004505	1	1	0.141284980609998	phenylalanine 4-monooxygenase activity
GO:0004508	1	1	0.141284980609998	steroid 17-alpha-monooxygenase activity
GO:0004509	1	1	0.141284980609998	steroid 21-monooxygenase activity
GO:0001618	1	1	0.141284980609998	viral receptor activity
GO:0004655	1	1	0.141284980609998	porphobilinogen synthase activity
GO:0005350	1	1	0.141284980609998	pyrimidine transporter activity
GO:0004638	1	1	0.141284980609998	phosphoribosylaminoimidazole carboxylase activity
GO:0004639	1	1	0.141284980609998	phosphoribosylaminoimidazolesuccinocarboxamide synthase activity
GO:0008383	1	1	0.141284980609998	manganese superoxide dismutase activity
GO:0008386	1	1	0.141284980609998	cholesterol monooxygenase (side-chain-cleaving) activity
GO:0008310	1	1	0.141284980609998	single-stranded DNA specific 3'-5' exodeoxyribonuclease activity

GO:0008336	1	1	0.141284980609998	gamma-butyrobetaine dioxygenase activity
GO:0050031	1	1	0.141284980609998	L-pipecolate oxidase activity
GO:0050038	1	1	0.141284980609998	L-xylulose reductase activity
GO:0008453	1	1	0.141284980609998	alanine-glyoxylate transaminase activity
GO:0008413	1	1	0.141284980609998	8-oxo-7,8-dihydroguanine triphosphatase activity
GO:0005199	1	1	0.141284980609998	structural constituent of cell wall
GO:0008402	1	1	0.141284980609998	aromatase activity
GO:0048154	1	1	0.141284980609998	S100 beta binding
GO:0046904	1	1	0.141284980609998	calcium oxalate binding
GO:0008120	1	1	0.141284980609998	ceramide glucosyltransferase activity
GO:0008125	1	1	0.141284980609998	pancreatic elastase I activity
GO:0008112	1	1	0.141284980609998	nicotinamide N-methyltransferase activity
GO:0008115	1	1	0.141284980609998	sarcosine oxidase activity
GO:0004996	1	1	0.141284980609998	thyroid-stimulating hormone receptor activity
GO:0008130	1	1	0.141284980609998	neutrophil collagenase activity
GO:0004956	1	1	0.141284980609998	prostaglandin D receptor activity
GO:0005286	1	1	0.141284980609998	basic amino acid permease activity
GO:0048244	1	1	0.141284980609998	phytanoyl-CoA dioxygenase activity
GO:0004903	1	1	0.141284980609998	growth hormone receptor activity
GO:0050355	1	1	0.141284980609998	triphosphatase activity
GO:0017162	1	1	0.141284980609998	aryl hydrocarbon receptor binding
GO:0017174	1	1	0.141284980609998	glycine N-methyltransferase activity
GO:0017113	1	1	0.141284980609998	dihydropyrimidine dehydrogenase (NADP+) activity
GO:0017147	1	1	0.141284980609998	Wnt-protein binding
GO:0008750	1	1	0.141284980609998	NAD(P)+ transhydrogenase (AB-specific) activity
GO:0008753	1	1	0.141284980609998	NADPH dehydrogenase (quinone) activity
GO:0008746	1	1	0.141284980609998	NAD(P) transhydrogenase activity
GO:0050425	1	1	0.141284980609998	carboxypeptidase B activity
GO:0047017	1	1	0.141284980609998	prostaglandin-F synthase activity
GO:0008859	1	1	0.141284980609998	exoribonuclease II activity
GO:0042083	1	1	0.141284980609998	5,10-methylenetetrahydrofolate-dependent methyltransferase activity
GO:0003894	1	1	0.141284980609998	zeta DNA polymerase activity
GO:0042030	1	1	0.141284980609998	ATPase inhibitor activity
GO:0003868	1	1	0.141284980609998	4-hydroxyphenylpyruvate dioxygenase activity
GO:0003812	1	1	0.141284980609998	alternative-complement-pathway C3/C5 convertase activity
GO:0003817	1	1	0.141284980609998	complement factor D activity
GO:0003818	1	1	0.141284980609998	complement factor I activity
GO:0003844	1	1	0.141284980609998	1,4-alpha-glucan branching enzyme activity
GO:0003837	1	1	0.141284980609998	beta-ureidopropionase activity
GO:0003802	1	1	0.141284980609998	coagulation factor VIIa activity
GO:0003806	1	1	0.141284980609998	coagulation factor XIIa activity
GO:0003808	1	1	0.141284980609998	protein C (activated) activity
GO:0003809	1	1	0.141284980609998	thrombin activity
GO:0008513	1	1	0.141284980609998	organic cation porter activity
GO:0008519	1	1	0.141284980609998	ammonium transporter activity
GO:0035197	1	1	0.141284980609998	siRNA binding

GO:0008613	1	1	0.141284980609998	diuretic hormone activity
GO:0051076	1	1	0.141284980609998	Gram-positive bacterial binding
GO:0050749	1	1	0.141284980609998	apolipoprotein E receptor binding
GO:0050824	1	1	0.141284980609998	water binding
GO:0042497	1	1	0.141284980609998	triacylated lipoprotein binding
GO:0050528	1	1	0.141284980609998	acyloxyacyl hydrolase activity
GO:0047150	1	1	0.141284980609998	betaine-homocysteine S-methyltransferase activity
GO:0051393	1	1	0.141284980609998	alpha-actinin binding
GO:0051397	1	1	0.141284980609998	N-terminal basic amino acid aminopeptidase activity
GO:0051398	1	1	0.141284980609998	N-terminal lysine aminopeptidase activity
GO:0051399	1	1	0.141284980609998	N-terminal arginine aminopeptidase activity
GO:0003990	1	1	0.141284980609998	acetylcholinesterase activity
GO:0047290	1	1	0.141284980609998	(alpha-N-acetylneuraminyl-2,3-beta-galactosyl-1,3)-N-acetyl-galactosaminide 6-alpha-sialyltransferase activity
GO:0008937	1	1	0.141284980609998	ferredoxin reductase activity
GO:0003957	1	1	0.141284980609998	NAD(P)+ transhydrogenase (B-specific) activity
GO:0051370	1	1	0.141284980609998	ZASP binding
GO:0051374	1	1	0.141284980609998	FATZ 1 binding
GO:0003976	1	1	0.141284980609998	UDP-N-acetylglucosamine-lysosomal-enzyme N-acetylglucosaminephosphotransferase activity
GO:0003941	1	1	0.141284980609998	L-serine ammonia-lyase activity
GO:0003933	1	1	0.141284980609998	GTP cyclohydrolase activity
GO:0003934	1	1	0.141284980609998	GTP cyclohydrolase I activity
GO:0019977	1	1	0.141284980609998	interleukin-21 binding
GO:0000156	1	1	0.141284980609998	two-component response regulator activity
GO:0016155	1	1	0.141284980609998	formyltetrahydrofolate dehydrogenase activity
GO:0047787	1	1	0.141284980609998	cortisone beta-reductase activity
GO:0047747	1	1	0.141284980609998	cholate-CoA ligase activity
GO:0000254	1	1	0.141284980609998	C-4 methylsterol oxidase activity
GO:0004096	1	1	0.141284980609998	catalase activity
GO:0000739	1	1	0.141284980609998	DNA strand annealing activity
GO:0004051	1	1	0.141284980609998	arachidonate 5-lipoxygenase activity
GO:0004055	1	1	0.141284980609998	argininosuccinate synthase activity
GO:0004056	1	1	0.141284980609998	argininosuccinate lyase activity
GO:0004087	1	1	0.141284980609998	carbamoyl-phosphate synthase (ammonia) activity
GO:0016730	1	1	0.141284980609998	oxidoreductase activity, acting on iron-sulfur proteins as donors
GO:0016731	1	1	0.141284980609998	oxidoreductase activity, acting on iron-sulfur proteins as donors, NAD or NADP as acceptor
GO:0015067	1	1	0.141284980609998	amidinotransferase activity
GO:0015068	1	1	0.141284980609998	glycine amidinotransferase activity
GO:0030354	1	1	0.141284980609998	melanin-concentrating hormone activity
GO:0030337	1	1	0.141284980609998	DNA polymerase processivity factor activity
GO:0030303	1	1	0.141284980609998	stromelysin 2 activity
GO:0042610	1	1	0.141284980609998	CD8 receptor binding
GO:0042602	1	1	0.141284980609998	flavin reductase activity
GO:0016538	1	1	0.141284980609998	cyclin-dependent protein kinase regulator activity
GO:0030412	1	1	0.141284980609998	formimidoyltetrahydrofolate cyclodeaminase activity
GO:0004397	1	1	0.141284980609998	histidine ammonia-lyase activity

GO:0030407	1	1	0.141284980609998	formimidoyltransferase activity
GO:0030409	1	1	0.141284980609998	glutamate formimidoyltransferase activity
GO:0004324	1	1	0.141284980609998	ferredoxin-NADP+ reductase activity
GO:0004325	1	1	0.141284980609998	ferrochelatase activity
GO:0004329	1	1	0.141284980609998	formate-tetrahydrofolate ligase activity
GO:0004346	1	1	0.141284980609998	glucose-6-phosphatase activity
GO:0016603	1	1	0.141284980609998	glutaminyl-peptide cyclotransferase activity
GO:0004491	1	1	0.141284980609998	methylmalonate-semialdehyde dehydrogenase (acylating) activity
GO:0015349	1	1	0.141284980609998	thyroid hormone transporter activity
GO:0004418	1	1	0.141284980609998	hydroxymethylbilane synthase activity
GO:0004446	1	1	0.141284980609998	multiple inositol-polyphosphate phosphatase activity
GO:0004447	1	1	0.141284980609998	iodide peroxidase activity
GO:0004193	1	1	0.141284980609998	cathepsin E activity
GO:0004183	1	1	0.141284980609998	carboxypeptidase E activity
GO:0018478	1	1	0.141284980609998	malonate-semialdehyde dehydrogenase (acetylating) activity
GO:0004133	1	1	0.141284980609998	glycogen debranching enzyme activity
GO:0004134	1	1	0.141284980609998	4-alpha-glucanotransferase activity
GO:0004135	1	1	0.141284980609998	amylo-alpha-1,6-glucosidase activity
GO:0004137	1	1	0.141284980609998	deoxycytidine kinase activity
GO:0004261	1	1	0.141284980609998	cathepsin G activity
GO:0004251	1	1	0.141284980609998	X-Pro dipeptidase activity
GO:0004283	1	1	0.141284980609998	plasmin activity
GO:0004285	1	1	0.141284980609998	proprotein convertase 1 activity
GO:0016913	1	1	0.141284980609998	follicle-stimulating hormone activity
GO:0004277	1	1	0.141284980609998	granzyme A activity
GO:0004278	1	1	0.141284980609998	granzyme B activity
GO:0004228	1	1	0.141284980609998	gelatinase A activity
GO:0004229	1	1	0.141284980609998	gelatinase B activity
GO:0004218	1	1	0.141284980609998	cathepsin S activity
GO:0030881	1	1	0.141284980609998	beta-2-microglobulin binding
GO:0030882	1	1	0.141284980609998	lipid antigen binding
GO:0030884	1	1	0.141284980609998	exogenous lipid antigen binding
GO:0004230	1	1	0.141284980609998	glutamyl aminopeptidase activity
GO:0004235	1	1	0.141284980609998	matrilysin activity
GO:0015283	1	1	0.141284980609998	apoptogenic cytochrome c release channel activity
GO:0004796	1	1	0.141284980609998	thromboxane-A synthase activity
GO:0004799	1	1	0.141284980609998	thymidylate synthase activity
GO:0004760	1	1	0.141284980609998	serine-pyruvate transaminase activity
GO:0004751	1	1	0.141284980609998	ribose-5-phosphate isomerase activity
GO:0045029	1	1	0.141284980609998	UDP-activated nucleotide receptor activity
GO:0015234	1	1	0.141284980609998	thiamin transporter activity
GO:0004775	1	1	0.141284980609998	succinate-CoA ligase (ADP-forming) activity
GO:0030568	1	1	0.141284980609998	plasmin inhibitor activity
GO:0030569	1	1	0.141284980609998	chymotrypsin inhibitor activity
GO:0004729	1	1	0.141284980609998	protoporphyrinogen oxidase activity
GO:0005018	1	1	0.141284980609998	platelet-derived growth factor alpha-receptor activity

GO:0004731	1	1	0.141284980609998	purine-nucleoside phosphorylase activity
GO:0016836	43	9	0.144081788448200	hydro-lyase activity
GO:0001848	5	2	0.148920315037789	complement binding
GO:0008131	5	2	0.148920315037789	amine oxidase activity
GO:0004960	5	2	0.148920315037789	thromboxane receptor activity
GO:0008252	5	2	0.148920315037789	nucleotidase activity
GO:0008243	5	2	0.148920315037789	plasminogen activator activity
GO:0019863	5	2	0.148920315037789	IgE binding
GO:0005521	5	2	0.148920315037789	lamin binding
GO:0016742	5	2	0.148920315037789	hydroxymethyl-, formyl- and related transferase activity
GO:0030280	5	2	0.148920315037789	structural constituent of epidermis
GO:0004126	5	2	0.148920315037789	cytidine deaminase activity
GO:0004907	26	6	0.150832597249268	interleukin receptor activity
GO:0008144	15	4	0.151174941222443	drug binding
GO:0004715	15	4	0.151174941222443	non-membrane spanning protein tyrosine kinase activity
GO:0008239	10	3	0.157602281333378	dipeptidyl-peptidase activity
GO:0005542	10	3	0.157602281333378	folic acid binding
GO:0019958	10	3	0.157602281333378	C-X-C chemokine binding
GO:0016775	10	3	0.157602281333378	phosphotransferase activity, nitrogenous group as acceptor
GO:0016863	10	3	0.157602281333378	intramolecular oxidoreductase activity, transposing C=C bonds
GO:0016806	10	3	0.157602281333378	dipeptidyl-peptidase and tripeptidyl-peptidase activity
GO:0005184	21	5	0.165117544802972	neuropeptide hormone activity
GO:0016791	171	29	0.167452319426925	phosphoric monoester hydrolase activity
GO:0005319	58	11	0.188354285697607	lipid transporter activity
GO:0005149	11	3	0.195129434506606	interleukin-1 receptor binding
GO:0005154	6	2	0.203195430493685	epidermal growth factor receptor binding
GO:0008199	6	2	0.203195430493685	ferric iron binding
GO:0004982	6	2	0.203195430493685	N-formyl peptide receptor activity
GO:0008271	6	2	0.203195430493685	sulfate porter activity
GO:0050750	6	2	0.203195430493685	low-density lipoprotein receptor binding
GO:0003916	6	2	0.203195430493685	DNA topoisomerase activity
GO:0016717	6	2	0.203195430493685	oxidoreductase activity, acting on paired donors, with oxidation of a pair of donors resulting in the reduction of molecular oxygen to two molecules of water
GO:0004030	6	2	0.203195430493685	aldehyde dehydrogenase [NAD(P)+] activity
GO:0004331	6	2	0.203195430493685	fructose-2,6-bisphosphate 2-phosphatase activity
GO:0015359	6	2	0.203195430493685	amino acid permease activity
GO:0015485	6	2	0.203195430493685	cholesterol binding
GO:0015174	6	2	0.203195430493685	basic amino acid transporter activity
GO:0015116	6	2	0.203195430493685	sulfate transporter activity
GO:0046870	6	2	0.203195430493685	cadmium ion binding
GO:0016298	59	11	0.203974176754639	lipase activity
GO:0019212	17	4	0.211136209987239	phosphatase inhibitor activity
GO:0050661	17	4	0.211136209987239	NADP binding
GO:0051213	35	7	0.217149673693543	dioxygenase activity
GO:0016701	35	7	0.217149673693543	oxidoreductase activity, acting on single donors with incorporation of molecular oxygen
GO:0016702	35	7	0.217149673693543	oxidoreductase activity, acting on single donors with incorporation of molecular oxygen,

				incorporation of two atoms of oxygen
GO:0031418	12	3	0.234522663327308	L-ascorbic acid binding
GO:0016284	12	3	0.234522663327308	alanine aminopeptidase activity
GO:0004028	12	3	0.234522663327308	aldehyde dehydrogenase activity
GO:0004179	12	3	0.234522663327308	membrane alanyl aminopeptidase activity
GO:0005021	12	3	0.234522663327308	vascular endothelial growth factor receptor activity
GO:0019208	36	7	0.239181251976836	phosphatase regulator activity
GO:0048037	100	17	0.241193349661289	cofactor binding
GO:0051287	18	4	0.2432718712375	NAD binding
GO:0004725	75	13	0.255598386176414	protein tyrosine phosphatase activity
GO:0001540	7	2	0.259131529556296	beta-amyloid binding
GO:0001619	7	2	0.259131529556296	lysosphingolipid and lysophosphatidic acid receptor activity
GO:0045125	7	2	0.259131529556296	bioactive lipid receptor activity
GO:0051183	7	2	0.259131529556296	vitamin transporter activity
GO:0042834	7	2	0.259131529556296	peptidoglycan binding
GO:0016868	7	2	0.259131529556296	intramolecular transferase activity, phosphotransferases
GO:0042605	7	2	0.259131529556296	peptide antigen binding
GO:0004372	7	2	0.259131529556296	glycine hydroxymethyltransferase activity
GO:0004194	7	2	0.259131529556296	pepsin A activity
GO:0004872	1060	157	0.262581342097165	receptor activity
GO:0043014	2	1	0.262621232825693	alpha-tubulin binding
GO:0004862	2	1	0.262621232825693	cAMP-dependent protein kinase inhibitor activity
GO:0004829	2	1	0.262621232825693	threonine-tRNA ligase activity
GO:0030676	2	1	0.262621232825693	Rac guanyl-nucleotide exchange factor activity
GO:0004802	2	1	0.262621232825693	transketolase activity
GO:0001530	2	1	0.262621232825693	lipopolysaccharide binding
GO:0017075	2	1	0.262621232825693	syntaxin-1 binding
GO:0004558	2	1	0.262621232825693	alpha-glucosidase activity
GO:0004507	2	1	0.262621232825693	steroid 11-beta-monooxygenase activity
GO:0004694	2	1	0.262621232825693	eukaryotic translation initiation factor 2alpha kinase activity
GO:0004666	2	1	0.262621232825693	prostaglandin-endoperoxide synthase activity
GO:0004656	2	1	0.262621232825693	procollagen-proline 4-dioxygenase activity
GO:0004658	2	1	0.262621232825693	propionyl-CoA carboxylase activity
GO:0004687	2	1	0.262621232825693	myosin light chain kinase activity
GO:0045309	2	1	0.262621232825693	protein phosphorylated amino acid binding
GO:0008379	2	1	0.262621232825693	thioredoxin peroxidase activity
GO:0004607	2	1	0.262621232825693	phosphatidylcholine-sterol O-acyltransferase activity
GO:0008320	2	1	0.262621232825693	protein carrier activity
GO:0015665	2	1	0.262621232825693	alcohol transporter activity
GO:0008330	2	1	0.262621232825693	protein tyrosine/threonine phosphatase activity
GO:0008494	2	1	0.262621232825693	translation activator activity
GO:0008451	2	1	0.262621232825693	X-Pro aminopeptidase activity
GO:0008472	2	1	0.262621232825693	metallocarboxypeptidase D activity
GO:0008422	2	1	0.262621232825693	beta-glucosidase activity
GO:0005153	2	1	0.262621232825693	interleukin-8 receptor binding
GO:0005155	2	1	0.262621232825693	epidermal growth factor receptor activating ligand activity

GO:0008198	2	1	0.262621232825693	ferrous iron binding
GO:0048155	2	1	0.262621232825693	S100 alpha binding
GO:0008160	2	1	0.262621232825693	protein tyrosine phosphatase activator activity
GO:0005148	2	1	0.262621232825693	prolactin receptor binding
GO:0005131	2	1	0.262621232825693	growth hormone receptor binding
GO:0008184	2	1	0.262621232825693	glycogen phosphorylase activity
GO:0008177	2	1	0.262621232825693	succinate dehydrogenase (ubiquinone) activity
GO:0004991	2	1	0.262621232825693	parathyroid hormone receptor activity
GO:0008106	2	1	0.262621232825693	alcohol dehydrogenase (NADP+) activity
GO:0004920	2	1	0.262621232825693	interleukin-10 receptor activity
GO:0008297	2	1	0.262621232825693	single-stranded DNA specific exodeoxyribonuclease activity
GO:0004917	2	1	0.262621232825693	interleukin-7 receptor activity
GO:0004918	2	1	0.262621232825693	interleukin-8 receptor activity
GO:0008241	2	1	0.262621232825693	peptidyl-dipeptidase activity
GO:0031545	2	1	0.262621232825693	peptidyl-proline 4-dioxygenase activity
GO:0008898	2	1	0.262621232825693	homocysteine S-methyltransferase activity
GO:0003893	2	1	0.262621232825693	epsilon DNA polymerase activity
GO:0003858	2	1	0.262621232825693	3-hydroxybutyrate dehydrogenase activity
GO:0019862	2	1	0.262621232825693	IgA binding
GO:0019870	2	1	0.262621232825693	potassium channel inhibitor activity
GO:0019826	2	1	0.262621232825693	oxygen sensor activity
GO:0003805	2	1	0.262621232825693	coagulation factor XIa activity
GO:0008970	2	1	0.262621232825693	phospholipase A1 activity
GO:0003998	2	1	0.262621232825693	acylphosphatase activity
GO:0003960	2	1	0.262621232825693	NADPH:quinone reductase activity
GO:0008943	2	1	0.262621232825693	glyceraldehyde-3-phosphate dehydrogenase activity
GO:0008934	2	1	0.262621232825693	inositol-1(or 4)-monophosphatase activity
GO:0003955	2	1	0.262621232825693	NAD(P)H dehydrogenase (quinone) activity
GO:0003912	2	1	0.262621232825693	DNA nucleotidylexotransferase activity
GO:0019969	2	1	0.262621232825693	interleukin-10 binding
GO:0019982	2	1	0.262621232825693	interleukin-7 binding
GO:0003938	2	1	0.262621232825693	IMP dehydrogenase activity
GO:0000163	2	1	0.262621232825693	protein phosphatase type 1 activity
GO:0019911	2	1	0.262621232825693	structural constituent of myelin sheath
GO:0016151	2	1	0.262621232825693	nickel ion binding
GO:0016215	2	1	0.262621232825693	CoA desaturase activity
GO:0051117	2	1	0.262621232825693	ATPase binding
GO:0004062	2	1	0.262621232825693	aryl sulfotransferase activity
GO:0004053	2	1	0.262621232825693	arginase activity
GO:0004086	2	1	0.262621232825693	carbamoyl-phosphate synthase activity
GO:0004074	2	1	0.262621232825693	biliverdin reductase activity
GO:0016714	2	1	0.262621232825693	oxidoreductase activity, acting on paired donors, with incorporation or reduction of molecular oxygen, reduced pteridine as one donor, and incorporation of one atom of oxygen
GO:0015065	2	1	0.262621232825693	uridine nucleotide receptor activity
GO:0016842	2	1	0.262621232825693	amidine-lyase activity
GO:0016808	2	1	0.262621232825693	proprotein convertase activity

GO:0030346	2	1	0.262621232825693	protein phosphatase 2B binding
GO:0030304	2	1	0.262621232825693	trypsin inhibitor activity
GO:0042609	2	1	0.262621232825693	CD4 receptor binding
GO:0046332	2	1	0.262621232825693	SMAD binding
GO:0004365	2	1	0.262621232825693	glyceraldehyde-3-phosphate dehydrogenase (phosphorylating) activity
GO:0004370	2	1	0.262621232825693	glycerol kinase activity
GO:0016647	2	1	0.262621232825693	oxidoreductase activity, acting on the CH-NH group of donors, oxygen as acceptor
GO:0016635	2	1	0.262621232825693	oxidoreductase activity, acting on the CH-CH group of donors, quinone or related compound as acceptor
GO:0015321	2	1	0.262621232825693	sodium-dependent phosphate transporter activity
GO:0004487	2	1	0.262621232825693	methylenetetrahydrofolate dehydrogenase (NAD+) activity
GO:0004473	2	1	0.262621232825693	malate dehydrogenase (oxaloacetate-decarboxylating) (NADP+) activity
GO:0004421	2	1	0.262621232825693	hydroxymethylglutaryl-CoA synthase activity
GO:0015482	2	1	0.262621232825693	voltage-gated anion channel porin activity
GO:0004152	2	1	0.262621232825693	dihydroorotate dehydrogenase activity
GO:0004158	2	1	0.262621232825693	dihydroorotate oxidase activity
GO:0004104	2	1	0.262621232825693	cholinesterase activity
GO:0004109	2	1	0.262621232825693	coproporphyrinogen oxidase activity
GO:0015166	2	1	0.262621232825693	polyol transporter activity
GO:0015111	2	1	0.262621232825693	iodide transporter activity
GO:0004217	2	1	0.262621232825693	cathepsin L activity
GO:0004246	2	1	0.262621232825693	peptidyl-dipeptidase A activity
GO:0001758	2	1	0.262621232825693	retinal dehydrogenase activity
GO:0001784	2	1	0.262621232825693	phosphotyrosine binding
GO:0015272	2	1	0.262621232825693	ATP-activated inward rectifier potassium channel activity
GO:0004768	2	1	0.262621232825693	stearoyl-CoA 9-desaturase activity
GO:0005061	2	1	0.262621232825693	aryl hydrocarbon receptor nuclear translocator activity
GO:0015232	2	1	0.262621232825693	heme transporter activity
GO:0004771	2	1	0.262621232825693	sterol esterase activity
GO:0004776	2	1	0.262621232825693	succinate-CoA ligase (GDP-forming) activity
GO:0015204	2	1	0.262621232825693	urea transporter activity
GO:0005010	2	1	0.262621232825693	insulin-like growth factor receptor activity
GO:0046848	2	1	0.262621232825693	hydroxyapatite binding
GO:0016860	31	6	0.267852120802808	intramolecular oxidoreductase activity
GO:0016835	50	9	0.268931415067133	carbon-oxygen lyase activity
GO:0004860	25	5	0.273038025214709	protein kinase inhibitor activity
GO:0016903	25	5	0.273038025214709	oxidoreductase activity, acting on the aldehyde or oxo group of donors
GO:0015288	13	3	0.275122203845973	porin activity
GO:0005044	19	4	0.276392220711307	scavenger receptor activity
GO:0008083	144	23	0.294693374249788	growth factor activity
GO:0005275	58	10	0.299059388009888	amine transporter activity
GO:0016247	26	5	0.302134555682671	channel regulator activity
GO:0008191	8	2	0.315177146189269	metalloendopeptidase inhibitor activity
GO:0004955	8	2	0.315177146189269	prostaglandin receptor activity
GO:0042288	8	2	0.315177146189269	MHC class I protein binding
GO:0004029	8	2	0.315177146189269	aldehyde dehydrogenase (NAD) activity

GO:0016813	8	2	0.315177146189269	hydrolase activity, acting on carbon-nitrogen (but not peptide) bonds, in linear amidines
GO:0030170	8	2	0.315177146189269	pyridoxal phosphate binding
GO:0004190	8	2	0.315177146189269	aspartic-type endopeptidase activity
GO:0015114	8	2	0.315177146189269	phosphate transporter activity
GO:0005099	8	2	0.315177146189269	Ras GTPase activator activity
GO:0004726	8	2	0.315177146189269	non-membrane spanning protein tyrosine phosphatase activity
GO:0005540	14	3	0.316330303617317	hyaluronic acid binding
GO:0004091	14	3	0.316330303617317	carboxylesterase activity
GO:0016505	14	3	0.316330303617317	apoptotic protease activator activity
GO:0004759	14	3	0.316330303617317	serine esterase activity
GO:0008194	79	13	0.321038337825799	UDP-glycosyltransferase activity
GO:0005070	40	7	0.333010336736179	SH3/SH2 adaptor activity
GO:0016829	127	20	0.335733516487104	lyase activity
GO:0016789	80	13	0.338020784280441	carboxylic ester hydrolase activity
GO:0005242	15	3	0.357619144216035	inward rectifier potassium channel activity
GO:0016504	15	3	0.357619144216035	protease activator activity
GO:0008022	28	5	0.361443344208401	protein C-terminus binding
GO:0005279	28	5	0.361443344208401	amino acid-polyamine transporter activity
GO:0015203	28	5	0.361443344208401	polyamine transporter activity
GO:0015171	48	8	0.365785135315044	amino acid transporter activity
GO:0008073	3	1	0.366823620722848	ornithine decarboxylase inhibitor activity
GO:0030506	3	1	0.366823620722848	ankyrin binding
GO:0004865	3	1	0.366823620722848	type 1 serine/threonine specific protein phosphatase inhibitor activity
GO:0004873	3	1	0.366823620722848	asialoglycoprotein receptor activity
GO:0004875	3	1	0.366823620722848	complement receptor activity
GO:0004800	3	1	0.366823620722848	thyroxine 5'-deiodinase activity
GO:0001515	3	1	0.366823620722848	opioid peptide activity
GO:0004566	3	1	0.366823620722848	beta-glucuronidase activity
GO:0004528	3	1	0.366823620722848	phosphodiesterase I activity
GO:0004668	3	1	0.366823620722848	protein-arginine deiminase activity
GO:0005345	3	1	0.366823620722848	purine transporter activity
GO:0004634	3	1	0.366823620722848	phosphopyruvate hydratase activity
GO:0008308	3	1	0.366823620722848	voltage-gated ion-selective channel activity
GO:0005436	3	1	0.366823620722848	sodium:phosphate symporter activity
GO:0008475	3	1	0.366823620722848	procollagen-lysine 5-dioxygenase activity
GO:0008426	3	1	0.366823620722848	protein kinase C inhibitor activity
GO:0005185	3	1	0.366823620722848	neurohypophyseal hormone activity
GO:0008195	3	1	0.366823620722848	phosphatidate phosphatase activity
GO:0005128	3	1	0.366823620722848	erythropoietin receptor binding
GO:0005138	3	1	0.366823620722848	interleukin-6 receptor binding
GO:0004971	3	1	0.366823620722848	alpha-amino-3-hydroxy-5-methyl-4-isoxazole propionate selective glutamate receptor activity
GO:0008296	3	1	0.366823620722848	3'-5'-exodeoxyribonuclease activity
GO:0004911	3	1	0.366823620722848	interleukin-2 receptor activity
GO:0004944	3	1	0.366823620722848	C5a anaphylatoxin receptor activity
GO:0043275	3	1	0.366823620722848	glutamate carboxypeptidase II activity
GO:0017127	3	1	0.366823620722848	cholesterol transporter activity

GO:0019211	3	1	0.366823620722848	phosphatase activator activity
GO:0003796	3	1	0.366823620722848	lysozyme activity
GO:0003876	3	1	0.366823620722848	AMP deaminase activity
GO:0045502	3	1	0.366823620722848	dynein binding
GO:0016019	3	1	0.366823620722848	peptidoglycan receptor activity
GO:0050780	3	1	0.366823620722848	dopamine receptor binding
GO:0050510	3	1	0.366823620722848	N-acetylgalactosaminyl-proteoglycan 3-beta-glucuronosyltransferase activity
GO:0003997	3	1	0.366823620722848	acyl-CoA oxidase activity
GO:0016416	3	1	0.366823620722848	O-palmitoyltransferase activity
GO:0003917	3	1	0.366823620722848	DNA topoisomerase type I activity
GO:0019959	3	1	0.366823620722848	interleukin-8 binding
GO:0019976	3	1	0.366823620722848	interleukin-2 binding
GO:0047238	3	1	0.366823620722848	glucuronosyl-N-acetylgalactosaminyl-proteoglycan 4-beta-N-acetylgalactosaminyltransferase activity
GO:0004095	3	1	0.366823620722848	carnitine O-palmitoyltransferase activity
GO:0004064	3	1	0.366823620722848	arylesterase activity
GO:0016744	3	1	0.366823620722848	transferase activity, transferring aldehyde or ketonic groups
GO:0051219	3	1	0.366823620722848	phosphoprotein binding
GO:0004046	3	1	0.366823620722848	aminoacylase activity
GO:0015087	3	1	0.366823620722848	cobalt ion transporter activity
GO:0030371	3	1	0.366823620722848	translation repressor activity
GO:0016527	3	1	0.366823620722848	brain-specific angiogenesis inhibitor activity
GO:0042608	3	1	0.366823620722848	T cell receptor binding
GO:0004332	3	1	0.366823620722848	fructose-bisphosphate aldolase activity
GO:0004165	3	1	0.366823620722848	dodecenoyl-CoA delta-isomerase activity
GO:0016978	3	1	0.366823620722848	lipoate-protein ligase B activity
GO:0005087	3	1	0.366823620722848	Ran guanyl-nucleotide exchange factor activity
GO:0005017	3	1	0.366823620722848	platelet-derived growth factor receptor activity
GO:0008061	3	1	0.366823620722848	chitin binding
GO:0005391	9	2	0.37018551871468	sodium:potassium-exchanging ATPase activity
GO:0005217	9	2	0.37018551871468	intracellular ligand-gated ion channel activity
GO:0005545	9	2	0.37018551871468	phosphatidylinositol binding
GO:0008514	9	2	0.37018551871468	organic anion transporter activity
GO:0051184	9	2	0.37018551871468	cofactor transporter activity
GO:0030552	9	2	0.37018551871468	3',5'-cAMP binding
GO:0043169	2313	332	0.371965843122385	cation binding
GO:0019888	35	6	0.373209422695762	protein phosphatase regulator activity
GO:0015457	29	5	0.391277295705676	auxiliary transport protein activity
GO:0005489	152	23	0.395110444271623	electron transporter activity
GO:0005247	16	3	0.398533565266696	voltage-gated chloride channel activity
GO:0004721	118	18	0.401406188675089	phosphoprotein phosphatase activity
GO:0005554	522	76	0.405518636245487	molecular function unknown
GO:0015103	23	4	0.412504823917005	inorganic anion transporter activity
GO:0004953	10	2	0.423331328060006	icosanoid receptor activity
GO:0004954	10	2	0.423331328060006	prostanoid receptor activity
GO:0019843	10	2	0.423331328060006	rRNA binding
GO:0000175	10	2	0.423331328060006	3'-5'-exoribonuclease activity

GO:0016208	10	2	0.423331328060006	AMP binding
GO:0016755	10	2	0.423331328060006	transferase activity, transferring amino-acyl groups
GO:0004716	10	2	0.423331328060006	receptor signaling protein tyrosine kinase activity
GO:0050660	44	7	0.430905880453576	FAD binding
GO:0016706	17	3	0.438690141398008	oxidoreductase activity, acting on paired donors, with incorporation or reduction of molecular oxygen, 2-oxoglutarate as one donor, and incorporation of one atom each of oxygen into both donors
GO:0003924	121	18	0.445680494606844	GTPase activity
GO:0005478	24	4	0.446089640172913	intracellular transporter activity
GO:0008408	24	4	0.446089640172913	3'-5' exonuclease activity
GO:0016831	24	4	0.446089640172913	carboxy-lyase activity
GO:0016830	31	5	0.450449679449748	carbon-carbon lyase activity
GO:0016787	1424	203	0.454262336240417	hydrolase activity
GO:0004540	45	7	0.45526817412231	ribonuclease activity
GO:0031404	45	7	0.45526817412231	chloride ion binding
GO:0043168	45	7	0.45526817412231	anion binding
GO:0008020	4	1	0.456310070635367	G-protein coupled photoreceptor activity
GO:0043015	4	1	0.456310070635367	gamma-tubulin binding
GO:0001517	4	1	0.456310070635367	N-acetylglucosamine 6-O-sulfotransferase activity
GO:0004551	4	1	0.456310070635367	nucleotide diphosphatase activity
GO:0017016	4	1	0.456310070635367	Ras GTPase binding
GO:0031419	4	1	0.456310070635367	cobalamin binding
GO:0004618	4	1	0.456310070635367	phosphoglycerate kinase activity
GO:0004645	4	1	0.456310070635367	phosphorylase activity
GO:0005332	4	1	0.456310070635367	gamma-aminobutyric acid:sodium symporter activity
GO:0008486	4	1	0.456310070635367	diphosphoinositol-polyphosphate diphosphatase activity
GO:0008172	4	1	0.456310070635367	S-methyltransferase activity
GO:0004942	4	1	0.456310070635367	anaphylatoxin receptor activity
GO:0019136	4	1	0.456310070635367	deoxynucleoside kinase activity
GO:0031543	4	1	0.456310070635367	peptidyl-proline dioxygenase activity
GO:0019798	4	1	0.456310070635367	procollagen-proline dioxygenase activity
GO:0008517	4	1	0.456310070635367	folic acid transporter activity
GO:0008518	4	1	0.456310070635367	reduced folate carrier activity
GO:0003696	4	1	0.456310070635367	satellite DNA binding
GO:0042301	4	1	0.456310070635367	phosphate binding
GO:0050811	4	1	0.456310070635367	GABA receptor binding
GO:0003956	4	1	0.456310070635367	NAD(P)+-protein-arginine ADP-ribosyltransferase activity
GO:0030021	4	1	0.456310070635367	extracellular matrix structural constituent conferring compression resistance
GO:0016299	4	1	0.456310070635367	regulator of G-protein signaling activity
GO:0016211	4	1	0.456310070635367	ammonia ligase activity
GO:0004035	4	1	0.456310070635367	alkaline phosphatase activity
GO:0016880	4	1	0.456310070635367	acid-ammonia (or amide) ligase activity
GO:0030345	4	1	0.456310070635367	structural constituent of tooth enamel
GO:0004470	4	1	0.456310070635367	malic enzyme activity
GO:0042979	4	1	0.456310070635367	ornithine decarboxylase regulator activity
GO:0004157	4	1	0.456310070635367	dihydropyrimidinase activity
GO:0015185	4	1	0.456310070635367	L-gamma-aminobutyric acid transporter activity

GO:0016979	4	1	0.456310070635367	lipoate-protein ligase activity
GO:0004287	4	1	0.456310070635367	prolyl oligopeptidase activity
GO:0015270	4	1	0.456310070635367	dihydropyridine-sensitive calcium channel activity
GO:0015205	4	1	0.456310070635367	nucleobase transporter activity
GO:0004740	4	1	0.456310070635367	[pyruvate dehydrogenase (lipoamide)] kinase activity
GO:0004553	59	9	0.45773405707709	hydrolase activity, hydrolyzing O-glycosyl compounds
GO:0016758	115	17	0.460426359542688	transferase activity, transferring hexosyl groups
GO:0004532	11	2	0.474042859960762	exoribonuclease activity
GO:0005355	11	2	0.474042859960762	glucose transporter activity
GO:0019206	11	2	0.474042859960762	nucleoside kinase activity
GO:0050897	11	2	0.474042859960762	cobalt ion binding
GO:0042287	11	2	0.474042859960762	MHC protein binding
GO:0016896	11	2	0.474042859960762	exoribonuclease activity, producing 5'-phosphomonoesters
GO:0016866	11	2	0.474042859960762	intramolecular transferase activity
GO:0004888	694	99	0.475176263406085	transmembrane receptor activity
GO:0050662	88	13	0.477278545994645	coenzyme binding
GO:0008430	18	3	0.47777379543011	selenium binding
GO:0019900	60	9	0.478840396020675	kinase binding
GO:0005253	46	7	0.479408845279623	anion channel activity
GO:0019901	54	8	0.501391672277952	protein kinase binding
GO:0004527	40	6	0.50540679744774	exonuclease activity
GO:0016410	33	5	0.507989104657828	N-acyltransferase activity
GO:0008138	26	4	0.511218633793765	protein tyrosine/serine/threonine phosphatase activity
GO:0016769	26	4	0.511218633793765	transferase activity, transferring nitrogenous groups
GO:0005001	19	3	0.515532840689886	transmembrane receptor protein tyrosine phosphatase activity
GO:0043028	19	3	0.515532840689886	caspase regulator activity
GO:0019198	19	3	0.515532840689886	transmembrane receptor protein phosphatase activity
GO:0008656	12	2	0.521946899287453	caspase activator activity
GO:0003995	12	2	0.521946899287453	acyl-CoA dehydrogenase activity
GO:0042562	12	2	0.521946899287453	hormone binding
GO:0004198	12	2	0.521946899287453	calpain activity
GO:0004143	12	2	0.521946899287453	diacylglycerol kinase activity
GO:0015145	12	2	0.521946899287453	monosaccharide transporter activity
GO:0015149	12	2	0.521946899287453	hexose transporter activity
GO:0004518	105	15	0.524408454424638	nuclease activity
GO:0017056	5	1	0.533157509534761	structural constituent of nuclear pore
GO:0004559	5	1	0.533157509534761	alpha-mannosidase activity
GO:0008301	5	1	0.533157509534761	DNA bending activity
GO:0048487	5	1	0.533157509534761	beta-tubulin binding
GO:0005152	5	1	0.533157509534761	interleukin-1 receptor antagonist activity
GO:0046912	5	1	0.533157509534761	transferase activity, transferring acyl groups, acyl groups converted into alkyl on transfer
GO:0005104	5	1	0.533157509534761	fibroblast growth factor receptor binding
GO:0004957	5	1	0.533157509534761	prostaglandin E receptor activity
GO:0004983	5	1	0.533157509534761	neuropeptide Y receptor activity
GO:0005522	5	1	0.533157509534761	profilin binding
GO:0009374	5	1	0.533157509534761	biotin binding

GO:0047498	5	1	0.533157509534761	calcium-dependent phospholipase A2 activity
GO:0047485	5	1	0.533157509534761	protein N-terminus binding
GO:0003964	5	1	0.533157509534761	RNA-directed DNA polymerase activity
GO:0016406	5	1	0.533157509534761	carnitine O-acyltransferase activity
GO:0009881	5	1	0.533157509534761	photoreceptor activity
GO:0042826	5	1	0.533157509534761	histone deacetylase binding
GO:0016774	5	1	0.533157509534761	phosphotransferase activity, carboxyl group as acceptor
GO:0004017	5	1	0.533157509534761	adenylate kinase activity
GO:0004000	5	1	0.533157509534761	adenosine deaminase activity
GO:0030353	5	1	0.533157509534761	fibroblast growth factor receptor antagonist activity
GO:0016832	5	1	0.533157509534761	aldehyde-lyase activity
GO:0016597	5	1	0.533157509534761	amino acid binding
GO:0004339	5	1	0.533157509534761	glucan 1,4-alpha-glucosidase activity
GO:0004185	5	1	0.533157509534761	serine carboxypeptidase activity
GO:0004274	5	1	0.533157509534761	dipeptidyl-peptidase IV activity
GO:0005088	5	1	0.533157509534761	Ras guanyl-nucleotide exchange factor activity
GO:0004749	5	1	0.533157509534761	ribose phosphate diphosphokinase activity
GO:0005041	5	1	0.533157509534761	low-density lipoprotein receptor activity
GO:0004222	77	11	0.534117046701189	metalloendopeptidase activity
GO:0031402	70	10	0.537248739149437	sodium ion binding
GO:0016853	92	13	0.545647813088463	isomerase activity
GO:0003690	20	3	0.551773117378511	double-stranded DNA binding
GO:0015179	20	3	0.551773117378511	L-amino acid transporter activity
GO:0005251	13	2	0.566824116640449	delayed rectifier potassium channel activity
GO:0015923	13	2	0.566824116640449	mannosidase activity
GO:0003887	28	4	0.572611782150053	DNA-directed DNA polymerase activity
GO:0004177	28	4	0.572611782150053	aminopeptidase activity
GO:0005254	43	6	0.579801875456538	chloride channel activity
GO:0004890	21	3	0.586351709987388	GABA-A receptor activity
GO:0015002	21	3	0.586351709987388	heme-copper terminal oxidase activity
GO:0016675	21	3	0.586351709987388	oxidoreductase activity, acting on heme group of donors
GO:0016676	21	3	0.586351709987388	oxidoreductase activity, acting on heme group of donors, oxygen as acceptor
GO:0004129	21	3	0.586351709987388	cytochrome-c oxidase activity
GO:0004197	80	11	0.58797464026516	cysteine-type endopeptidase activity
GO:0008509	95	13	0.594822304726547	anion transporter activity
GO:0005031	6	1	0.599149925342977	tumor necrosis factor receptor activity
GO:0043022	6	1	0.599149925342977	ribosome binding
GO:0043027	6	1	0.599149925342977	caspase inhibitor activity
GO:0004839	6	1	0.599149925342977	ubiquitin activating enzyme activity
GO:0004568	6	1	0.599149925342977	chitinase activity
GO:0004579	6	1	0.599149925342977	dolichyl-diphosphooligosaccharide-protein glycotransferase activity
GO:0031406	6	1	0.599149925342977	carboxylic acid binding
GO:0004622	6	1	0.599149925342977	lysophospholipase activity
GO:0005484	6	1	0.599149925342977	SNAP receptor activity
GO:0003857	6	1	0.599149925342977	3-hydroxyacyl-CoA dehydrogenase activity
GO:0003810	6	1	0.599149925342977	protein-glutamine gamma-glutamyltransferase activity

GO:0016018	6	1	0.599149925342977	cyclosporin A binding
GO:0016308	6	1	0.599149925342977	1-phosphatidylinositol-4-phosphate 5-kinase activity
GO:0016421	6	1	0.599149925342977	CoA carboxylase activity
GO:0016409	6	1	0.599149925342977	palmitoyltransferase activity
GO:0016165	6	1	0.599149925342977	lipoxygenase activity
GO:0019903	6	1	0.599149925342977	protein phosphatase binding
GO:0019905	6	1	0.599149925342977	syntaxin binding
GO:0000149	6	1	0.599149925342977	SNARE binding
GO:0016778	6	1	0.599149925342977	diphosphotransferase activity
GO:0016721	6	1	0.599149925342977	oxidoreductase activity, acting on superoxide radicals as acceptor
GO:0016861	6	1	0.599149925342977	intramolecular oxidoreductase activity, interconverting aldoses and ketoses
GO:0016812	6	1	0.599149925342977	hydrolase activity, acting on carbon-nitrogen (but not peptide) bonds, in cyclic amides
GO:0016615	6	1	0.599149925342977	malate dehydrogenase activity
GO:0004300	6	1	0.599149925342977	enoyl-CoA hydratase activity
GO:0004499	6	1	0.599149925342977	dimethylaniline monooxygenase (N-oxide-forming) activity
GO:0030228	6	1	0.599149925342977	lipoprotein receptor activity
GO:0004245	6	1	0.599149925342977	neprilysin activity
GO:0015248	6	1	0.599149925342977	sterol transporter activity
GO:0004784	6	1	0.599149925342977	superoxide dismutase activity
GO:0005342	66	9	0.599792369176376	organic acid transporter activity
GO:0046943	66	9	0.599792369176376	carboxylic acid transporter activity
GO:0030693	14	2	0.608573085477638	caspase activity
GO:0008373	14	2	0.608573085477638	sialyltransferase activity
GO:0005518	14	2	0.608573085477638	collagen binding
GO:0019904	52	7	0.615689897981367	protein domain specific binding
GO:0042578	228	31	0.62164138027206	phosphoric ester hydrolase activity
GO:0016788	446	61	0.631759568032529	hydrolase activity, acting on ester bonds
GO:0043167	2556	356	0.64434193911423	ion binding
GO:0004864	15	2	0.647181384133268	protein phosphatase inhibitor activity
GO:0046915	15	2	0.647181384133268	transition metal ion transporter activity
GO:0030159	15	2	0.647181384133268	receptor signaling complex scaffold activity
GO:0016917	23	3	0.650170460440229	GABA receptor activity
GO:0016757	165	22	0.650378265964113	transferase activity, transferring glycosyl groups
GO:0005035	7	1	0.65581965797409	death receptor activity
GO:0048019	7	1	0.65581965797409	receptor antagonist activity
GO:0030547	7	1	0.65581965797409	receptor inhibitor activity
GO:0001595	7	1	0.65581965797409	angiotensin receptor activity
GO:0004571	7	1	0.65581965797409	mannosyl-oligosaccharide 1,2-alpha-mannosidase activity
GO:0004529	7	1	0.65581965797409	exodeoxyribonuclease activity
GO:0004673	7	1	0.65581965797409	protein histidine kinase activity
GO:0005375	7	1	0.65581965797409	copper ion transporter activity
GO:0004602	7	1	0.65581965797409	glutathione peroxidase activity
GO:0005487	7	1	0.65581965797409	nucleocytoplasmic transporter activity
GO:0005416	7	1	0.65581965797409	cation:amino acid symporter activity
GO:0005160	7	1	0.65581965797409	transforming growth factor beta receptor binding
GO:0043120	7	1	0.65581965797409	tumor necrosis factor binding

GO:0005283	7	1	0.65581965797409	sodium:amino acid symporter activity
GO:0004945	7	1	0.65581965797409	angiotensin type II receptor activity
GO:0003756	7	1	0.65581965797409	protein disulfide isomerase activity
GO:0005527	7	1	0.65581965797409	macrolide binding
GO:0005528	7	1	0.65581965797409	FK506 binding
GO:0000062	7	1	0.65581965797409	acyl-CoA binding
GO:0008642	7	1	0.65581965797409	ubiquitin-like activating enzyme activity
GO:0000155	7	1	0.65581965797409	two-component sensor molecule activity
GO:0016895	7	1	0.65581965797409	exodeoxyribonuclease activity, producing 5'-phosphomonoesters
GO:0016864	7	1	0.65581965797409	intramolecular oxidoreductase activity, transposing S-S bonds
GO:0016885	7	1	0.65581965797409	ligase activity, forming carbon-carbon bonds
GO:0004457	7	1	0.65581965797409	lactate dehydrogenase activity
GO:0004459	7	1	0.65581965797409	L-lactate dehydrogenase activity
GO:0030742	7	1	0.65581965797409	GTP-dependent protein binding
GO:0015108	7	1	0.65581965797409	chloride transporter activity
GO:0015250	7	1	0.65581965797409	water channel activity
GO:0015277	7	1	0.65581965797409	kainate selective glutamate receptor activity
GO:0005080	7	1	0.65581965797409	protein kinase C binding
GO:0008093	7	1	0.65581965797409	cytoskeletal adaptor activity
GO:0004521	31	4	0.655927283818901	endoribonuclease activity
GO:0031072	31	4	0.655927283818901	heat shock protein binding
GO:0030145	92	12	0.663288209737583	manganese ion binding
GO:0004623	16	2	0.682702501114207	phospholipase A2 activity
GO:0005328	16	2	0.682702501114207	neurotransmitter:sodium symporter activity
GO:0005158	16	2	0.682702501114207	insulin receptor binding
GO:0019201	16	2	0.682702501114207	nucleotide kinase activity
GO:0003705	16	2	0.682702501114207	RNA polymerase II transcription factor activity, enhancer binding
GO:0004468	16	2	0.682702501114207	lysine N-acetyltransferase activity
GO:0004402	16	2	0.682702501114207	histone acetyltransferase activity
GO:0030955	86	11	0.686259590405361	potassium ion binding
GO:0031420	146	19	0.687333002701488	alkali metal ion binding
GO:0030545	8	1	0.70448287394335	receptor regulator activity
GO:0004861	8	1	0.70448287394335	cyclin-dependent protein kinase inhibitor activity
GO:0004576	8	1	0.70448287394335	oligosaccharyl transferase activity
GO:0005372	8	1	0.70448287394335	water transporter activity
GO:0005537	8	1	0.70448287394335	mannose binding
GO:0008641	8	1	0.70448287394335	small protein activating enzyme activity
GO:0035251	8	1	0.70448287394335	UDP-glucosyltransferase activity
GO:0016307	8	1	0.70448287394335	phosphatidylinositol phosphate kinase activity
GO:0016780	8	1	0.70448287394335	phosphotransferase activity, for other substituted phosphate groups
GO:0016862	8	1	0.70448287394335	intramolecular oxidoreductase activity, interconverting keto- and enol-groups
GO:0016524	8	1	0.70448287394335	latrotoxin receptor activity
GO:0030169	8	1	0.70448287394335	low-density lipoprotein binding
GO:0016986	8	1	0.70448287394335	transcription initiation factor activity
GO:0004289	8	1	0.70448287394335	subtilase activity
GO:0003755	25	3	0.706634819352331	peptidyl-prolyl cis-trans isomerase activity

GO:0016651	57	7	0.712757089563734	oxidoreductase activity, acting on NADH or NADPH
GO:0004683	17	2	0.7152374824097	calmodulin regulated protein kinase activity
GO:0005544	17	2	0.7152374824097	calcium-dependent phospholipid binding
GO:0016796	17	2	0.7152374824097	exonuclease activity, active with either ribo- or deoxyribonucleic acids and producing 5'-phosphomonoesters
GO:0004519	65	8	0.716762728188382	endonuclease activity
GO:0015290	149	19	0.721382389879596	electrochemical potential-driven transporter activity
GO:0015291	149	19	0.721382389879596	porter activity
GO:0016746	104	13	0.725710684420636	transferase activity, transferring acyl groups
GO:0046872	2495	344	0.725712869114778	metal ion binding
GO:0005488	7228	1013	0.725720683841796	binding
GO:0019992	42	5	0.726735761120858	diacylglycerol binding
GO:0016811	34	4	0.727571348432791	hydrolase activity, acting on carbon-nitrogen (but not peptide) bonds, in linear amides
GO:0003725	26	3	0.732123887033882	double-stranded RNA binding
GO:0003704	26	3	0.732123887033882	specific RNA polymerase II transcription factor activity
GO:0016859	26	3	0.732123887033882	cis-trans isomerase activity
GO:0008034	18	2	0.74492044328215	lipoprotein binding
GO:0005326	18	2	0.74492044328215	neurotransmitter transporter activity
GO:0008374	18	2	0.74492044328215	O-acyltransferase activity
GO:0008376	18	2	0.74492044328215	acetylgalactosaminyltransferase activity
GO:0004298	18	2	0.74492044328215	threonine endopeptidase activity
GO:0030551	18	2	0.74492044328215	cyclic nucleotide binding
GO:0043021	9	1	0.746270043346934	ribonucleoprotein binding
GO:0046527	9	1	0.746270043346934	glucosyltransferase activity
GO:0004693	9	1	0.746270043346934	cyclin-dependent protein kinase activity
GO:0005388	9	1	0.746270043346934	calcium-transporting ATPase activity
GO:0005159	9	1	0.746270043346934	insulin-like growth factor receptor binding
GO:0008121	9	1	0.746270043346934	ubiquinol-cytochrome-c reductase activity
GO:0015924	9	1	0.746270043346934	mannosyl-oligosaccharide mannosidase activity
GO:0004016	9	1	0.746270043346934	adenylate cyclase activity
GO:0016500	9	1	0.746270043346934	protein-hormone receptor activity
GO:0016681	9	1	0.746270043346934	oxidoreductase activity, acting on diphenols and related substances as donors, cytochrome as acceptor
GO:0016679	9	1	0.746270043346934	oxidoreductase activity, acting on diphenols and related substances as donors
GO:0004385	9	1	0.746270043346934	guanylate kinase activity
GO:0008415	98	12	0.747009229463573	acyltransferase activity
GO:0016747	98	12	0.747009229463573	transferase activity, transferring groups other than amino-acyl groups
GO:0005386	398	52	0.754005639175994	carrier activity
GO:0004722	27	3	0.755833765107605	protein serine/threonine phosphatase activity
GO:0004713	161	20	0.766962331769457	protein-tyrosine kinase activity
GO:0050681	19	2	0.77190722005771	androgen receptor binding
GO:0008080	28	3	0.777820403062511	N-acetyltransferase activity
GO:0003697	28	3	0.777820403062511	single-stranded DNA binding
GO:0016798	77	9	0.778218891322364	hydrolase activity, acting on glycosyl bonds
GO:0005244	124	15	0.779888386978433	voltage-gated ion channel activity
GO:0048029	10	1	0.78215212282326	monosaccharide binding

GO:0001565	10	1	0.78215212282326	phorbol ester receptor activity
GO:0004697	10	1	0.78215212282326	protein kinase C activity
GO:0004653	10	1	0.78215212282326	polypeptide N-acetylgalactosaminyltransferase activity
GO:0004685	10	1	0.78215212282326	calcium- and calmodulin-dependent protein kinase activity
GO:0015932	10	1	0.78215212282326	nucleobase, nucleoside, nucleotide and nucleic acid transporter activity
GO:0003746	10	1	0.78215212282326	translation elongation factor activity
GO:0019902	10	1	0.78215212282326	phosphatase binding
GO:0015238	10	1	0.78215212282326	drug transporter activity
GO:0005057	132	16	0.782787969644636	receptor signaling protein activity
GO:0016563	62	7	0.791873177598337	transcriptional activator activity
GO:0001633	20	2	0.796366564799843	secretin-like receptor activity
GO:0005164	20	2	0.796366564799843	tumor necrosis factor receptor binding
GO:0016763	29	3	0.798150607264207	transferase activity, transferring pentosyl groups
GO:0005126	38	4	0.804905642814593	hematopoietin/interferon-class (D200-domain) cytokine receptor binding
GO:0005249	63	7	0.805532005364833	voltage-gated potassium channel activity
GO:0003727	11	1	0.812963049109509	single-stranded RNA binding
GO:0015082	30	3	0.816899114232956	di-, tri-valent inorganic cation transporter activity
GO:0004840	21	2	0.818473391622207	ubiquitin conjugating enzyme activity
GO:0016709	21	2	0.818473391622207	oxidoreductase activity, acting on paired donors, with incorporation or reduction of molecular oxygen, NAD or NADH as one donor, and incorporation of one atom of oxygen
GO:0016407	39	4	0.821179617148033	acetyltransferase activity
GO:0008234	98	11	0.835243489171968	cysteine-type peptidase activity
GO:0005267	90	10	0.835891636750897	potassium channel activity
GO:0043176	22	2	0.838403671303751	amine binding
GO:0005243	12	1	0.839419062729696	gap-junction forming channel activity
GO:0016652	12	1	0.839419062729696	oxidoreductase activity, acting on NADH or NADPH, NAD or NADP as acceptor
GO:0015175	12	1	0.839419062729696	neutral amino acid transporter activity
GO:0015285	12	1	0.839419062729696	connexon channel activity
GO:0005086	12	1	0.839419062729696	ARF guanyl-nucleotide exchange factor activity
GO:0015293	83	9	0.847338594646596	symporter activity
GO:0008146	32	3	0.849975059426172	sulfotransferase activity
GO:0003824	3409	465	0.853496856563333	catalytic activity
GO:0035258	23	2	0.856330643766944	steroid hormone receptor binding
GO:0016564	59	6	0.858367297721637	transcriptional repressor activity
GO:0005248	13	1	0.862135308237133	voltage-gated sodium channel activity
GO:0015085	13	1	0.862135308237133	calcium ion transporter activity
GO:0015036	42	4	0.86337863587859	disulfide oxidoreductase activity
GO:0001653	94	10	0.872403654003171	peptide receptor activity
GO:0008528	94	10	0.872403654003171	peptide receptor activity, G-protein coupled
GO:0008639	24	2	0.872422078351615	small protein conjugating enzyme activity
GO:0019205	34	3	0.877719461233268	nucleobase, nucleoside, nucleotide kinase activity
GO:0008094	34	3	0.877719461233268	DNA-dependent ATPase activity
GO:0004520	14	1	0.881640094603248	endodeoxyribonuclease activity
GO:0004970	14	1	0.881640094603248	ionotropic glutamate receptor activity
GO:0005234	14	1	0.881640094603248	glutamate-gated ion channel activity
GO:0042169	14	1	0.881640094603248	SH2 domain binding

GO:0003950	14	1	0.881640094603248	NAD+ ADP-ribosyltransferase activity
GO:0004364	14	1	0.881640094603248	glutathione transferase activity
GO:0004536	25	2	0.886838361742857	deoxyribonuclease activity
GO:0016655	35	3	0.889804394341873	oxidoreductase activity, acting on NADH or NADPH, quinone or similar compound as acceptor
GO:0003777	45	4	0.896744265856904	microtubule motor activity
GO:0051015	15	1	0.898387145351104	actin filament binding
GO:0004437	26	2	0.899731234635272	inositol or phosphatidylinositol phosphatase activity
GO:0016782	36	3	0.90080822563336	transferase activity, transferring sulfur-containing groups
GO:0016874	221	25	0.908707338997875	ligase activity
GO:0015267	284	33	0.909109337717187	channel or pore class transporter activity
GO:0005215	1030	132	0.909142699905086	transporter activity
GO:0008601	16	1	0.912766121633564	protein phosphatase type 2A regulator activity
GO:0015459	16	1	0.912766121633564	potassium channel regulator activity
GO:0004620	47	4	0.914794731704124	phospholipase activity
GO:0005262	47	4	0.914794731704124	calcium channel activity
GO:0019899	151	16	0.919738169310213	enzyme binding
GO:0016776	28	2	0.921506311361714	phosphotransferase activity, phosphate group as acceptor
GO:0042277	127	13	0.923785841932308	peptide binding
GO:0005212	17	1	0.925111661585055	structural constituent of eye lens
GO:0042166	17	1	0.925111661585055	acetylcholine binding
GO:0008565	67	6	0.926339799868748	protein transporter activity
GO:0015268	274	31	0.92922679424554	alpha-type channel activity
GO:0051119	29	2	0.930643770924492	sugar transporter activity
GO:0015662	40	3	0.935557016613407	ATPase activity, coupled to transmembrane movement of ions, phosphorylative mechanism
GO:0016849	18	1	0.935711145014638	phosphorus-oxygen lyase activity
GO:0008170	30	2	0.938768389259602	N-methyltransferase activity
GO:0015144	30	2	0.938768389259602	carbohydrate transporter activity
GO:0004221	41	3	0.942282699865086	ubiquitin thiolesterase activity
GO:0005343	19	1	0.9448113630678	organic acid:sodium symporter activity
GO:0001727	19	1	0.9448113630678	lipid kinase activity
GO:0001584	282	31	0.951343698108066	rhodopsin-like receptor activity
GO:0008137	32	2	0.952384297184796	NADH dehydrogenase (ubiquinone) activity
GO:0050136	32	2	0.952384297184796	NADH dehydrogenase (quinone) activity
GO:0003954	32	2	0.952384297184796	NADH dehydrogenase activity
GO:0015035	32	2	0.952384297184796	protein disulfide oxidoreductase activity
GO:0008483	20	1	0.952624247187167	transaminase activity
GO:0009975	20	1	0.952624247187167	cyclase activity
GO:0005525	226	24	0.952760360763263	GTP binding
GO:0004843	43	3	0.953822883272578	ubiquitin-specific protease activity
GO:0005216	252	27	0.956538582500043	ion channel activity
GO:0051082	127	12	0.957025854661419	unfolded protein binding
GO:0015296	21	1	0.959331789965415	anion:cation symporter activity
GO:0005085	93	8	0.961819847501029	guanyl-nucleotide exchange factor activity
GO:0015276	84	7	0.962494773754107	ligand-gated ion channel activity
GO:0005245	22	1	0.965090271806446	voltage-gated calcium channel activity
GO:0015370	35	2	0.967517930936871	solute:sodium symporter activity

GO:0005230	57	4	0.969169409000277	extracellular ligand-gated ion channel activity
GO:0004114	23	1	0.970033891262787	3',5'-cyclic-nucleotide phosphodiesterase activity
GO:0016741	87	7	0.971199462378034	transferase activity, transferring one-carbon groups
GO:0019001	235	24	0.971334023306322	guanyl nucleotide binding
GO:0015081	36	2	0.971442031248842	sodium ion transporter activity
GO:0005272	24	1	0.97427788312849	sodium channel activity
GO:0019200	24	1	0.97427788312849	carbohydrate kinase activity
GO:0004112	24	1	0.97427788312849	cyclic-nucleotide phosphodiesterase activity
GO:0016765	37	2	0.9749066812527	transferase activity, transferring alkyl or aryl (other than methyl) groups
GO:0008047	188	18	0.977012407926605	enzyme activator activity
GO:0004714	60	4	0.977623641752393	transmembrane receptor protein tyrosine kinase activity
GO:0016879	127	11	0.977801612752804	ligase activity, forming carbon-nitrogen bonds
GO:0004428	25	1	0.977921196519559	inositol or phosphatidylinositol kinase activity
GO:0015294	39	2	0.98065781954468	solute:cation symporter activity
GO:0045182	72	5	0.981307794301928	translation regulator activity
GO:0016790	51	3	0.981650165878344	thiolester hydrolase activity
GO:0005351	27	1	0.983733632025601	sugar porter activity
GO:0008168	84	6	0.984598230476163	methyltransferase activity
GO:0046873	74	5	0.984780881205358	metal ion transporter activity
GO:0015297	28	1	0.986038347605745	antiporter activity
GO:0015631	42	2	0.986959679791879	tubulin binding
GO:0051020	42	2	0.986959679791879	GTPase binding
GO:0035257	42	2	0.986959679791879	nuclear hormone receptor binding
GO:0046914	1596	198	0.987133593673393	transition metal ion binding
GO:0005261	188	17	0.987273723632714	cation channel activity
GO:0003702	197	18	0.987462951314032	RNA polymerase II transcription factor activity
GO:0008188	29	1	0.988016725344361	neuropeptide receptor activity
GO:0003684	29	1	0.988016725344361	damaged DNA binding
GO:0042923	29	1	0.988016725344361	neuropeptide binding
GO:0008066	29	1	0.988016725344361	glutamate receptor activity
GO:0051427	43	2	0.988576108890381	hormone receptor binding
GO:0000287	251	24	0.989079940286012	magnesium ion binding
GO:0005096	117	9	0.989131443185647	GTPase activator activity
GO:0008017	30	1	0.989714943830043	microtubule binding
GO:0042165	78	5	0.989984494706605	neurotransmitter binding
GO:0005089	44	2	0.989996499625455	Rho guanyl-nucleotide exchange factor activity
GO:0008757	57	3	0.991057911465256	S-adenosylmethionine-dependent methyltransferase activity
GO:0003735	100	7	0.99141335560059	structural constituent of ribosome
GO:0004930	349	35	0.99198238437946	G-protein coupled receptor activity
GO:0015077	121	9	0.992399018344269	monovalent inorganic cation transporter activity
GO:0004812	32	1	0.992423888526303	tRNA ligase activity
GO:0008452	32	1	0.992423888526303	RNA ligase activity
GO:0016875	32	1	0.992423888526303	ligase activity, forming carbon-oxygen bonds
GO:0016876	32	1	0.992423888526303	ligase activity, forming aminoacyl-tRNA and related compounds
GO:0015078	112	8	0.992936453721702	hydrogen ion transporter activity
GO:0003682	47	2	0.993299489719322	chromatin binding

GO:0005083	144	11	0.994548742950262	small GTPase regulator activity
GO:0019199	73	4	0.994770965721596	transmembrane receptor protein kinase activity
GO:0016779	95	6	0.99493010817149	nucleotidyltransferase activity
GO:0008135	62	3	0.995157329758017	translation factor activity, nucleic acid binding
GO:0031267	35	1	0.99521095065892	small GTPase binding
GO:0004879	51	2	0.99609332136068	ligand-dependent nuclear receptor activity
GO:0030594	77	4	0.99671620579451	neurotransmitter receptor activity
GO:0003713	140	10	0.99672008700241	transcription coactivator activity
GO:0015399	140	10	0.99672008700241	primary active transporter activity
GO:0016881	101	6	0.997305580859796	acid-amino acid ligase activity
GO:0005231	39	1	0.99740254776931	excitatory extracellular ligand-gated ion channel activity
GO:0030695	219	18	0.99751494206361	GTPase regulator activity
GO:0042625	68	3	0.997713622982376	ATPase activity, coupled to transmembrane movement of ions
GO:0016886	40	1	0.997771034993786	ligase activity, forming phosphoric ester bonds
GO:0004672	447	44	0.997807779633628	protein kinase activity
GO:0008134	293	26	0.99786821778122	transcription factor binding
GO:0003714	81	4	0.997952433228413	transcription corepressor activity
GO:0019829	42	1	0.998358684993568	cation-transporting ATPase activity
GO:0003743	43	1	0.998591603472215	translation initiation factor activity
GO:0008081	60	2	0.998860322963617	phosphoric diester hydrolase activity
GO:0016301	609	62	0.998905834200993	kinase activity
GO:0015075	523	51	0.99919151527293	ion transporter activity
GO:0015405	77	3	0.999276596178754	P-P-bond-hydrolysis-driven transporter activity
GO:0003707	48	1	0.999344946895882	steroid hormone receptor activity
GO:0004674	326	27	0.999624538454596	protein serine/threonine kinase activity
GO:0016773	532	50	0.999717232993049	phosphotransferase activity, alcohol group as acceptor
GO:0042626	100	4	0.999799682484023	ATPase activity, coupled to transmembrane movement of substances
GO:0003712	230	16	0.999800543533718	transcription cofactor activity
GO:0004842	87	3	0.999804337690378	ubiquitin-protein ligase activity
GO:0016820	101	4	0.999823310060036	hydrolase activity, acting on acid anhydrides, catalyzing transmembrane movement of substances
GO:0008324	421	36	0.9998646124913	cation transporter activity
GO:0016818	432	37	0.99988325082158	hydrolase activity, acting on acid anhydrides, in phosphorus-containing anhydrides
GO:0016772	708	69	0.999888268195444	transferase activity, transferring phosphorus-containing groups
GO:0016817	433	37	0.99989129838713	hydrolase activity, acting on acid anhydrides
GO:0030554	1028	108	0.999891315982568	adenyl nucleotide binding
GO:0008026	93	3	0.999911800173708	ATP-dependent helicase activity
GO:0017111	410	34	0.999918632677064	nucleoside-triphosphatase activity
GO:0016462	430	36	0.999929469932605	pyrophosphatase activity
GO:0005524	970	99	0.99994970657228	ATP binding
GO:0004386	101	3	0.99996988599809	helicase activity
GO:0017076	1248	132	0.99997244126375	purine nucleotide binding
GO:0003700	649	59	0.999977496594557	transcription factor activity
GO:0016887	255	15	0.999993121540714	ATPase activity
GO:0016740	1169	118	0.999995399355557	transferase activity
GO:0042623	229	12	0.99999649983808	ATPase activity, coupled
GO:0000166	1432	146	0.99999936059373	nucleotide binding

GO:0008270	1287	126	0.999999775029657	zinc ion binding
GO:0030528	963	84	0.999999969959804	transcription regulator activity
GO:0003723	436	21	0.999999999968262	RNA binding
GO:0003677	1384	109	0.999999999999995	DNA binding
GO:0003674	9541	1348	1	molecular_function
GO:0003676	1990	143	1	nucleic acid binding

0.4 GO: Cellular Component

GO ID	genes present in chip	genes present in tissues	p-value	GO term
GO:0005576	949	295	1.07904478710131e-46	extracellular region
GO:0005615	385	128	1.75478092266380e-22	extracellular space
GO:0030017	28	24	4.00724477338545e-17	sarcomere
GO:0043292	40	29	8.55849587227151e-17	contractile fiber
GO:0030016	32	25	5.95908389976313e-16	myofibril
GO:0005865	10	10	3.13962399446048e-09	striated muscle thin filament
GO:0005790	10	9	1.94879099241001e-07	smooth endoplasmic reticulum
GO:0005863	15	11	3.47417875327247e-07	striated muscle thick filament
GO:0005859	15	11	3.47417875327247e-07	muscle myosin
GO:0016460	15	11	3.47417875327247e-07	myosin II
GO:0045111	65	25	1.02837382090271e-06	intermediate filament cytoskeleton
GO:0005882	65	25	1.02837382090271e-06	intermediate filament
GO:0005792	108	32	2.31618838384691e-05	microsome
GO:0042598	108	32	2.31618838384691e-05	vesicular fraction
GO:0001533	12	8	4.60164193280442e-05	cornified envelope
GO:0005861	5	5	5.6528203401607e-05	troponin complex
GO:0042101	10	7	9.1027381500642e-05	T cell receptor complex
GO:0005581	30	13	9.86685558621319e-05	collagen
GO:0005856	612	119	0.00011408692925327	cytoskeleton
GO:0016459	43	16	0.000144004464000532	myosin
GO:0015629	183	44	0.000215536278842791	actin cytoskeleton
GO:0005625	183	44	0.000215536278842791	soluble fraction
GO:0001772	29	12	0.000309399124296931	immunological synapse
GO:0005583	10	6	0.00100290513813701	fibrillar collagen
GO:0005577	5	4	0.00177556060709117	fibrinogen complex
GO:0005862	3	3	0.0028331882482464	muscle thin filament tropomyosin
GO:0005584	3	3	0.0028331882482464	collagen type I
GO:0030018	3	3	0.0028331882482464	Z disc
GO:0000267	698	124	0.00322569547686345	cell fraction
GO:0005578	303	59	0.00581505462218203	extracellular matrix (sensu Metazoa)
GO:0031012	304	59	0.00625104773314239	extracellular matrix
GO:0005833	10	5	0.00766006196244783	hemoglobin complex
GO:0042627	4	3	0.0101317489405670	chylomicron
GO:0030868	2	2	0.0200365114853462	smooth endoplasmic reticulum membrane
GO:0043205	5	3	0.0226660314407808	fibril
GO:0005606	5	3	0.0226660314407808	laminin-1
GO:0005884	17	6	0.0244809941320903	actin filament
GO:0030141	18	6	0.0324371496671596	secretory granule
GO:0045121	14	5	0.0374109192057178	lipid raft
GO:0030935	6	3	0.0406039565820352	sheet-forming collagen
GO:0005587	6	3	0.0406039565820352	collagen type IV
GO:0005579	6	3	0.0406039565820352	membrane attack complex

GO:0005626	15	5	0.0497629543510898	insoluble fraction
GO:0045335	3	2	0.0544431579595457	phagocytic vesicle
GO:0005811	3	2	0.0544431579595457	lipid particle
GO:0000796	3	2	0.0544431579595457	condensin complex
GO:0043235	58	13	0.0588108754976749	receptor complex
GO:0005886	1472	228	0.0598567586526041	plasma membrane
GO:0043256	7	3	0.0637075303084579	laminin complex
GO:0030139	12	4	0.0774576730941017	endocytic vesicle
GO:0005604	44	10	0.083826205216539	basement membrane
GO:0000793	23	6	0.0955943438839286	condensed chromosome
GO:0001527	4	2	0.0987545669785243	microfibril
GO:0008305	24	6	0.112946521980637	integrin complex
GO:0005605	24	6	0.112946521980637	basal lamina
GO:0005930	9	3	0.123274541676307	axoneme
GO:0043020	1	1	0.141600092624754	NADPH oxidase complex
GO:0043033	1	1	0.141600092624754	isoamylase complex
GO:0030666	1	1	0.141600092624754	endocytic vesicle membrane
GO:0030670	1	1	0.141600092624754	phagocytic vesicle membrane
GO:0001520	1	1	0.141600092624754	outer dense fiber
GO:0031253	1	1	0.141600092624754	cell projection membrane
GO:0031232	1	1	0.141600092624754	extrinsic to external side of plasma membrane
GO:0045285	1	1	0.141600092624754	ubiquinol-cytochrome-c reductase complex
GO:0005766	1	1	0.141600092624754	primary lysosome
GO:0005782	1	1	0.141600092624754	peroxisomal matrix
GO:0031526	1	1	0.141600092624754	brush border membrane
GO:0005582	1	1	0.141600092624754	collagen type XV
GO:0005585	1	1	0.141600092624754	collagen type II
GO:0005586	1	1	0.141600092624754	collagen type III
GO:0016035	1	1	0.141600092624754	zeta DNA polymerase complex
GO:0009320	1	1	0.141600092624754	phosphoribosylaminoimidazole carboxylase complex
GO:0005971	1	1	0.141600092624754	ribonucleoside-diphosphate reductase complex
GO:0042272	1	1	0.141600092624754	nuclear RNA export factor complex
GO:0042581	1	1	0.141600092624754	specific granule
GO:0042582	1	1	0.141600092624754	azurophil granule
GO:0042575	1	1	0.141600092624754	DNA polymerase complex
GO:0046658	5	2	0.14948025733702	anchored to plasma membrane
GO:0031225	5	2	0.14948025733702	anchored to membrane
GO:0005883	5	2	0.14948025733702	neurofilament
GO:0016599	5	2	0.14948025733702	caveolar membrane
GO:0005741	26	6	0.151970197160154	mitochondrial outer membrane
GO:0005819	68	13	0.157467176951040	spindle
GO:0016363	10	3	0.158372978924085	nuclear matrix
GO:0005952	10	3	0.158372978924085	cAMP-dependent protein kinase complex
GO:0030055	11	3	0.196041653534942	cell-matrix junction
GO:0005678	6	2	0.203918407714512	chromatin assembly complex
GO:0016324	6	2	0.203918407714512	apical plasma membrane

GO:0005901	6	2	0.203918407714512	caveola
GO:0045177	7	2	0.260002758676934	apical part of cell
GO:0008290	7	2	0.260002758676934	F-actin capping protein complex
GO:0005890	7	2	0.260002758676934	sodium:potassium-exchanging ATPase complex
GO:0016528	7	2	0.260002758676934	sarcoplasm
GO:0016529	7	2	0.260002758676934	sarcoplasmic reticulum
GO:0030424	7	2	0.260002758676934	axon
GO:0031304	2	1	0.263163673764161	intrinsic to mitochondrial inner membrane
GO:0005827	2	1	0.263163673764161	polar microtubule
GO:0005589	2	1	0.263163673764161	collagen type VI
GO:0005602	2	1	0.263163673764161	complement component C1q complex
GO:0030056	2	1	0.263163673764161	hemidesmosome
GO:0000777	2	1	0.263163673764161	condensed chromosome kinetochore
GO:0000779	2	1	0.263163673764161	condensed chromosome, pericentric region
GO:0051233	2	1	0.263163673764161	spindle midzone
GO:0042599	2	1	0.263163673764161	lamellar body
GO:0000940	2	1	0.263163673764161	outer kinetochore of condensed chromosome
GO:0019898	37	7	0.263736390509531	extrinsic to membrane
GO:0005783	483	73	0.287221184032082	endoplasmic reticulum
GO:0019897	26	5	0.303809595383914	extrinsic to plasma membrane
GO:0005624	521	78	0.310858232157239	membrane fraction
GO:0030934	8	2	0.316177009236303	anchoring collagen
GO:0005925	8	2	0.316177009236303	focal adhesion
GO:0005758	14	3	0.317613791089142	mitochondrial intermembrane space
GO:0005876	14	3	0.317613791089142	spindle microtubule
GO:0000786	40	7	0.335144134081538	nucleosome
GO:0043228	977	143	0.339979605823128	non-membrane-bound organelle
GO:0043232	977	143	0.339979605823128	intracellular non-membrane-bound organelle
GO:0005764	107	17	0.343105980323829	lysosome
GO:0000323	107	17	0.343105980323829	lytic vacuole
GO:0005834	21	4	0.345895894599451	heterotrimeric G-protein complex
GO:0031226	1062	155	0.346630928075808	intrinsic to plasma membrane
GO:0005777	67	11	0.347481470132108	peroxisome
GO:0042579	67	11	0.347481470132108	microbody
GO:0009986	61	10	0.360685837117817	cell surface
GO:0005816	3	1	0.36752393166647	spindle pole body
GO:0005594	3	1	0.36752393166647	collagen type IX
GO:0005588	3	1	0.36752393166647	collagen type V
GO:0000015	3	1	0.36752393166647	phosphopyruvate hydratase complex
GO:0042588	3	1	0.36752393166647	zymogen granule
GO:0042589	3	1	0.36752393166647	zymogen granule membrane
GO:0005924	9	2	0.371291999967729	cell-substrate adherens junction
GO:0005887	1057	153	0.391945188177547	integral to plasma membrane
GO:0042995	70	11	0.404707544740074	cell projection
GO:0005773	120	18	0.434849290949486	vacuole
GO:0043005	17	3	0.440219048562187	neuron projection

GO:0000776	17	3	0.440219048562187	kinetochore
GO:0043034	4	1	0.457113719895785	costamere
GO:0031307	4	1	0.457113719895785	integral to mitochondrial outer membrane
GO:0031229	4	1	0.457113719895785	intrinsic to nuclear inner membrane
GO:0045275	4	1	0.457113719895785	respiratory chain complex III
GO:0005639	4	1	0.457113719895785	integral to nuclear inner membrane
GO:0045095	4	1	0.457113719895785	keratin filament
GO:0019867	52	8	0.45932314681788	outer membrane
GO:0005737	2530	360	0.464852344071968	cytoplasm
GO:0005802	11	2	0.475295111160574	Golgi trans face
GO:0009897	11	2	0.475295111160574	external side of plasma membrane
GO:0005912	18	3	0.479352869006049	adherens junction
GO:0016020	3514	498	0.501515283419603	membrane
GO:0030659	12	2	0.523240203989643	cytoplasmic vesicle membrane
GO:0043025	5	1	0.534022085535476	cell soma
GO:0031306	5	1	0.534022085535476	intrinsic to mitochondrial outer membrane
GO:0005814	5	1	0.534022085535476	centriole
GO:0005593	5	1	0.534022085535476	FACIT collagen
GO:0016011	5	1	0.534022085535476	dystroglycan complex
GO:0016012	5	1	0.534022085535476	sarcoglycan complex
GO:0005929	5	1	0.534022085535476	cilium
GO:0016461	5	1	0.534022085535476	unconventional myosin
GO:0005871	13	2	0.568139551633145	kinesin complex
GO:0016023	122	17	0.5687017209818	cytoplasmic membrane-bound vesicle
GO:0031410	124	17	0.597209327974946	cytoplasmic vesicle
GO:0008250	6	1	0.600042821099669	oligosaccharyl transferase complex
GO:0019861	6	1	0.600042821099669	flagellum
GO:0005663	6	1	0.600042821099669	DNA replication factor C complex
GO:0000242	6	1	0.600042821099669	pericentriolar material
GO:0030127	6	1	0.600042821099669	COPII vesicle coat
GO:0000930	6	1	0.600042821099669	gamma-tubulin complex
GO:0001725	6	1	0.600042821099669	stress fiber
GO:0030027	14	2	0.609893845057146	lamellipodium
GO:0001726	14	2	0.609893845057146	ruffle
GO:0048471	22	3	0.620805490192558	perinuclear region
GO:0000794	15	2	0.64849288625236	condensed nuclear chromosome
GO:0030667	7	1	0.656716164836792	secretory granule membrane
GO:0005637	7	1	0.656716164836792	nuclear inner membrane
GO:0005913	7	1	0.656716164836792	cell-cell adherens junction
GO:0030138	7	1	0.656716164836792	COPII-coated vesicle
GO:0031252	24	3	0.68091614339135	leading edge
GO:0005788	16	2	0.683992377241248	endoplasmic reticulum lumen
GO:0012506	16	2	0.683992377241248	vesicle membrane
GO:0042611	16	2	0.683992377241248	MHC protein complex
GO:0042612	16	2	0.683992377241248	MHC class I protein complex
GO:0005770	8	1	0.705364615636862	late endosome

GO:0005903	8	1	0.705364615636862	brush border
GO:0016605	8	1	0.705364615636862	PML body
GO:0000228	42	5	0.728698081902916	nuclear chromosome
GO:0016327	50	6	0.729983413618298	apicolateral plasma membrane
GO:0043233	18	2	0.746138315072507	organelle lumen
GO:0005839	18	2	0.746138315072507	proteasome core complex (sensu Eukaryota)
GO:0005765	9	1	0.747123692595503	lysosomal membrane
GO:0030425	9	1	0.747123692595503	dendrite
GO:0008091	9	1	0.747123692595503	spectrin
GO:0000922	51	6	0.747737206430155	spindle pole
GO:0005813	43	5	0.747979833594852	centrosome
GO:0031228	35	4	0.750518775135529	intrinsic to Golgi membrane
GO:0008021	27	3	0.757299095017623	synaptic vesicle
GO:0000775	27	3	0.757299095017623	chromosome, pericentric region
GO:0008372	523	69	0.761906024382535	cellular component unknown
GO:0019717	19	2	0.773078505076802	synaptosome
GO:0005938	28	3	0.77923258543732	cell cortex
GO:0016010	10	1	0.782968352302933	dystrophin-associated glycoprotein complex
GO:0005680	10	1	0.782968352302933	anaphase-promoting complex
GO:0005768	62	7	0.79394227645559	endosome
GO:0030173	29	3	0.799505929522276	integral to Golgi membrane
GO:0005740	119	14	0.810841664402126	mitochondrial membrane
GO:0001518	11	1	0.813735676417168	voltage-gated sodium channel complex
GO:0005746	21	2	0.819538436206916	mitochondrial electron transport chain
GO:0031224	2681	366	0.82696582903376	intrinsic to membrane
GO:0015630	191	23	0.829569188501366	microtubule cytoskeleton
GO:0031300	90	10	0.838073390514985	intrinsic to organelle membrane
GO:0005732	12	1	0.840144355728762	small nucleolar ribonucleoprotein complex
GO:0005922	12	1	0.840144355728762	connexon complex
GO:0030864	12	1	0.840144355728762	cortical actin cytoskeleton
GO:0005815	49	5	0.842632540866146	microtubule organizing center
GO:0031227	32	3	0.851146919877816	intrinsic to endoplasmic reticulum membrane
GO:0000502	41	4	0.851703139133728	proteasome complex (sensu Eukaryota)
GO:0016585	23	2	0.857280614462148	chromatin remodeling complex
GO:0005789	84	9	0.858928221052014	endoplasmic reticulum membrane
GO:0016021	2674	363	0.859454429118691	integral to membrane
GO:0005769	13	1	0.86281142273673	early endosome
GO:0005793	24	2	0.873313748195856	ER-Golgi intermediate compartment
GO:0005829	276	33	0.877523870827853	cytosol
GO:0000152	14	1	0.882266621019775	nuclear ubiquitin ligase complex
GO:0000159	14	1	0.882266621019775	protein phosphatase type 2A complex
GO:0030894	14	1	0.882266621019775	replisome
GO:0005657	15	1	0.898964744931733	replication fork
GO:0005921	15	1	0.898964744931733	gap junction
GO:0000178	15	1	0.898964744931733	exosome (RNase complex)
GO:0030176	26	2	0.900508480549817	integral to endoplasmic reticulum membrane

GO:0005923	36	3	0.901732448814897	tight junction
GO:0042175	90	9	0.90665087370664	nuclear envelope-endoplasmic reticulum network
GO:0005842	16	1	0.913296236111099	cytosolic large ribosomal subunit (sensu Eukaryota)
GO:0005665	16	1	0.913296236111099	DNA-directed RNA polymerase II, core complex
GO:0005669	16	1	0.913296236111099	transcription factor TFIID complex
GO:0016604	16	1	0.913296236111099	nuclear body
GO:0030863	16	1	0.913296236111099	cortical cytoskeleton
GO:0000785	100	10	0.916052956898603	chromatin
GO:0005905	38	3	0.920696387185662	coated pit
GO:0005941	57	5	0.921354920033089	unlocalized protein complex
GO:0031301	76	7	0.927682678244285	integral to organelle membrane
GO:0005891	18	1	0.936152633932588	voltage-gated calcium channel complex
GO:0043296	40	3	0.936257435171797	apical junction complex
GO:0005875	70	6	0.944089916858347	microtubule associated complex
GO:0005778	19	1	0.945212307757909	peroxisomal membrane
GO:0030286	19	1	0.945212307757909	dynein complex
GO:0005874	132	13	0.946192031175897	microtubule
GO:0008076	53	4	0.953859196762264	voltage-gated potassium channel complex
GO:0005635	83	7	0.959839248556773	nuclear membrane
GO:0030054	93	8	0.962565571115513	cell junction
GO:0015934	22	1	0.96538589830217	large ribosomal subunit
GO:0045211	66	5	0.966475182021535	postsynaptic membrane
GO:0000139	67	5	0.969627806531167	Golgi membrane
GO:0008287	23	1	0.970299774840353	protein serine/threonine phosphatase complex
GO:0030880	23	1	0.970299774840353	RNA polymerase complex
GO:0005694	203	20	0.974690476764795	chromosome
GO:0045202	98	8	0.975531723984052	synapse
GO:0019866	89	7	0.97642540072862	inner membrane
GO:0005759	62	4	0.982301852946535	mitochondrial matrix
GO:0005761	27	1	0.983904554171768	mitochondrial ribosome
GO:0000313	27	1	0.983904554171768	organellar ribosome
GO:0005739	490	54	0.985189072333254	mitochondrion
GO:0012505	246	24	0.985695471199418	endomembrane system
GO:0005743	85	6	0.986340935506	mitochondrial inner membrane
GO:0046930	42	2	0.987144717809138	pore complex
GO:0005643	42	2	0.987144717809138	nuclear pore
GO:0030120	42	2	0.987144717809138	vesicle coat
GO:0030136	65	4	0.98729839739513	clathrin-coated vesicle
GO:0048475	43	2	0.988743009855015	coated membrane
GO:0030117	43	2	0.988743009855015	membrane coat
GO:0030125	30	1	0.989835809523518	clathrin vesicle coat
GO:0030135	79	5	0.99118912790901	coated vesicle
GO:0030118	31	1	0.991280076393826	clathrin coat
GO:0005667	91	6	0.9925338851731	transcription factor complex
GO:0005830	33	1	0.99358247789832	cytosolic ribosome (sensu Eukaryota)
GO:0005911	82	5	0.993619786267392	intercellular junction

GO:0005730	75	4	0.995947574742883	nucleolus
GO:0005840	113	7	0.997735033846912	ribosome
GO:0005654	221	18	0.997977283385176	nucleoplasm
GO:0000151	42	1	0.998386387313485	ubiquitin ligase complex
GO:0016591	58	2	0.99853028107365	DNA-directed RNA polymerase II, holoenzyme
GO:0031090	377	33	0.999577034385698	organelle membrane
GO:0005681	57	1	0.999838931826288	spliceosome complex
GO:0005794	195	9	0.999996824069747	Golgi apparatus
GO:0043234	1247	117	0.99999998268815	protein complex
GO:0030529	254	9	0.999999997448821	ribonucleoprotein complex
GO:0043226	4515	495	1	organelle
GO:0043227	3955	388	1	membrane-bound organelle
GO:0043229	4515	495	1	intracellular organelle
GO:0043231	3955	388	1	intracellular membrane-bound organelle
GO:0005575	8637	1223	1	cellular_component
GO:0005622	5419	636	1	intracellular
GO:0005623	7802	1009	1	cell
GO:0005634	2704	234	1	nucleus

0.5 OMIM

1: #610163
IMMUNODEFICIENCY DUE TO DEFECT IN CD3-ZETA
Gene map locus 1q22-q23

2: #610102
COMPLEMENT COMPONENT 7 DEFICIENCY
Gene map locus 5p13

3: #609924
AMINOACYLASE 1 DEFICIENCY
Gene map locus 3p21.1

4: #609909
CARDIOMYOPATHY, DILATED, 1P; CMD1P
Gene map locus 6q22.1

5: #609889
ALPHA/BETA T-CELL LYMPHOPENIA WITH GAMMA/DELTA T-CELL EXPANSION, SEVERE CYTOMEGALOVIRUS INFECTION, AND AUTOIMMUNITY
Gene map locus 11p13

6: #609886
GLOMERULOCYSTIC KIDNEY DISEASE WITH HYPERURICEMIA AND ISOSTHENURIA
Gene map locus 16p12.3

7: #609830
ABDOMINAL BODY FAT DISTRIBUTION
Gene map locus 2p23.3, 20q13.1-q13.2, 3p25

8: #609812
DIABETES AND PANCREATIC EXOCRINE DYSFUNCTION
Gene map locus 9q34.3

9: #609638
EPIDERMOLYSIS BULLOSA, LETHAL ACANTHOLYTIC
Gene map locus 6p24

10: #609620
SHORT QT SYNDROME 1; SQT1
Gene map locus 7q35-q36

11: #609452
MYOPATHY, MYOFIBRILLAR, ZASP-RELATED
Gene map locus 10q22.2-q23.3

12: #609423
HUMAN IMMUNODEFICIENCY VIRUS TYPE 1, SUSCEPTIBILITY TO
HUMAN IMMUNODEFICIENCY VIRUS TYPE 1, RESISTANCE TO, INCLUDED
Gene map locus 19q13.4, 19p13.3, 17q12, 17q11.2-q12, 16p12.1-p11.2, 12q14,
10q11.1

13: #609220
BRUCK SYNDROME 2
Gene map locus 3q23-q24

14: #609200
MYOTILINOPATHY
Gene map locus 5q31

15: #609152
HYPERTHYROIDISM, NONAUTOIMMUNE
Gene map locus 14q31

16: #608957
CD8 DEFICIENCY, FAMILIAL
Gene map locus 2p12

17: #608890
WAARDENBURG SYNDROME, TYPE IID
Gene map locus 8q11

18: #608810
ALPHA-B CRYSTALLINOPATHY
ALPHA-B CRYSTALLINOPATHY WITH CATARACT, INCLUDED
Gene map locus 11q22.3-q23.1

19: #608807
MUSCULAR DYSTROPHY, LIMB-GIRDLE, TYPE 2J; LGMD2J
Gene map locus 2q24.3

20: #608758
CARDIOMYOPATHY, FAMILIAL HYPERTROPHIC, 10; CMH10
CARDIOMYOPATHY, HYPERTROPHIC, MID-LEFT VENTRICULAR CHAMBER TYPE, 2, INCLUDED
Gene map locus 12q23-q24.3

21: #608751
CARDIOMYOPATHY, FAMILIAL HYPERTROPHIC, 8; CMH8
Gene map locus 3p

22: #608716
MICROCEPHALY, PRIMARY AUTOSOMAL RECESSIVE, 5; MCPH5
Gene map locus 1q31

23: #608611
RIBOSE 5-PHOSPHATE ISOMERASE DEFICIENCY
Gene map locus 2p11.2

24: #608471
CORNEAL DYSTROPHY, LATTICE TYPE IIIA; CDL3A
Gene map locus 5q31

25: #608470
CORNEAL DYSTROPHY OF BOWMAN LAYER, TYPE I; CDB1
Gene map locus 5q31

26: %608446
MYOCARDIAL INFARCTION, SUSCEPTIBILITY TO, 1
Gene map locus 1q25, 6q25.1, 1p22.1, 1p36-p34

27: #608404
PLATELET GLYCOPROTEIN IV DEFICIENCY
Gene map locus 7q11.2

28: #608358
MYOPATHY, MYOSIN STORAGE
Gene map locus 14q12

29: #608355
PARKES WEBER SYNDROME
Gene map locus 5q13.3

30: #608354
CAPILLARY MALFORMATION-ARTERIOVENOUS MALFORMATION
Gene map locus 5q13.3

31: #608328
WEILL-MARCHESANI SYNDROME, AUTOSOMAL DOMINANT
Gene map locus 15q21.1

32: #608189
TROPICAL CALCIFIC PANCREATITIS
Gene map locus 5q32

33: #608175
AUTOIMMUNE THYROID DISEASE, SUSCEPTIBILITY TO, 3
Gene map locus 8q24.2-q24.3

34: #607941
ATRIAL SEPTAL DEFECT 2; ASD2
Gene map locus 8p23.1-p22

35: #607855
MUSCULAR DYSTROPHY, CONGENITAL MEROSIN-DEFICIENT, 1A; MDC1A
MUSCULAR DYSTROPHY, CONGENITAL, DUE TO PARTIAL LAMA2 DEFICIENCY, INCLUDED
Gene map locus 6q22-q23

36: #607832
FOCAL SEGMENTAL GLOMERULOSCLEROSIS 3; FSGS3
Gene map locus Chr.6

37: #607791
CHARCOT-MARIE-TOOTH DISEASE, DOMINANT INTERMEDIATE D
Gene map locus 1q22

38: #607748
HYPERCHOLANEMIA, FAMILIAL; FHCA
Gene map locus 1q42.1, 9q22.3, 9q12-q13

39: #607736
CHARCOT-MARIE-TOOTH DISEASE, AXONAL, TYPE 2J
Gene map locus 1q22

40: #607692
CHYLOMICRON RETENTION DISEASE WITH MARINESCO-SJOGREN SYNDROME
Gene map locus 5q31.1

41: #607689
ANDERSON DISEASE
Gene map locus 5q31.1

42: #607685
HYPEREOSINOPHILIC SYNDROME, IDIOPATHIC; HES
Gene map locus 4q12

43: #607677
CHARCOT-MARIE-TOOTH DISEASE, AXONAL, TYPE 2I
Gene map locus 1q22

44: #607655
SKIN FRAGILITY-WOOLLY HAIR SYNDROME
Gene map locus 6p24

45: #607654
KERATOSIS PALMOPLANTARIS STRIATA III
Gene map locus 12q13

46: #607602
ICHTHYOSIS, CYCLIC, WITH EPIDERMOLYTIC HYPERKERATOSIS
Gene map locus 17q21-q22, 12q13

47: #607595
BRAIN SMALL VESSEL DISEASE WITH HEMORRHAGE
Gene map locus 13q34

48: #607541
CORNEAL DYSTROPHY, AVELLINO TYPE; CDA
Gene map locus 5q31

49: #607487
CARDIOMYOPATHY, DILATED, 1N; CMD1N
Gene map locus 17q12

50: #607482
CARDIOMYOPATHY, DILATED, 1M; CMD1M
Gene map locus 11p15.1

51: #607450
ARRHYTHMOGENIC RIGHT VENTRICULAR DYSPLASIA, FAMILIAL, 8; ARVD8
Gene map locus 6p24

52: #607277
ASTHMA-RELATED TRAITS, SUSCEPTIBILITY TO, 1
Gene map locus 14q22.1

53: #607174
MENINGIOMA, FAMILIAL
MENINGIOMA CAUSED BY SOMATIC MUTATION, INCLUDED
Gene map locus 22q12.3-qter, 22q12.2

54: %606963
PULMONARY DISEASE, CHRONIC OBSTRUCTIVE, SEVERE EARLY-ONSET
Gene map locus 14q32.1, 11q22-q23, 2q

55: #606785
CRIGLER-NAJJAR SYNDROME, TYPE II
Gene map locus 2q37

56: #606764
GASTROINTESTINAL STROMAL TUMOR; GIST
Gene map locus 4q12, 4q12

57: #606664
GLYCINE N-METHYLTRANSFERASE DEFICIENCY
Gene map locus 6p12

58: #606407
HYPOTONIA-CYSTINURIA SYNDROME
Gene map locus 2p16.3

59: #606054
PROPIONIC ACIDEMIA
Gene map locus 13q32, 3q21-q22

60: #605990
URIC ACID NEPHROLITHIASIS, SUSCEPTIBILITY TO
Gene map locus 10q21.2

61: #605911
3-@HYDROXY-3-METHYLGLUTARYL-CoA SYNTHASE 2, MITOCHONDRIAL, DEFICIENCY OF
Gene map locus 1p13-p12

62: #605676
CARDIOMYOPATHY, DILATED, WITH WOOLLY HAIR AND KERATODERMA
Gene map locus 6p24

63: #605637
INCLUSION BODY MYOPATHY 3, AUTOSOMAL DOMINANT; IBM3
Gene map locus 17p13.1

64: #605543
PARKINSON DISEASE 4, AUTOSOMAL DOMINANT LEWY BODY; PARK4
Gene map locus 4q21

65: #605355
NEMALINE MYOPATHY 5; NEM5
Gene map locus 19q13.4

66: #605253
NEUROPATHY, CONGENITAL HYPOMYELINATING
Gene map locus 1q22, 10q21.1-q22.1

67: #605156
MULTICENTRIC OSTEOLYSIS, NODULOSIS, AND ARTHROPATHY; MONA
Gene map locus 16q13

68: #605027
LYMPHOMA, NON-HODGKIN, FAMILIAL
LYMPHOMA, NON-HODGKIN, CAUSED BY SOMATIC MUTATION, INCLUDED
Gene map locus 10q22, 2q33-q34

69: %604809
 PANBRONCHIOLITIS, DIFFUSE
Gene map locus 6p21.3

70: #604772
 VENTRICULAR TACHYCARDIA, CATECHOLAMINERGIC POLYMORPHIC; CPVT
Gene map locus 1q42.1-q43, 1p13.3-p11

71: #604765
 CARDIOMYOPATHY, DILATED, 1I; CMD1I
Gene map locus 2q35

72: #604416
 PYOGENIC STERILE ARTHRITIS, PYODERMA GANGRENOSUM, AND ACNE
Gene map locus 15q24-q25.1

73: #604308
 MASS SYNDROME
Gene map locus 15q21.1

74: #604271
 SHORT STATURE, IDIOPATHIC, AUTOSOMAL
Gene map locus Ypter-p11.2, Xpter-p22.32, 17q22-q24, 3q26.3

75: #604250
 HEMOCHROMATOSIS, TYPE 3; HFE3
Gene map locus 7q22

76: #604145
 CARDIOMYOPATHY, DILATED, 1G; CMD1G
Gene map locus 2q24.3

77: #604117
 VOHWINKEL SYNDROME, VARIANT FORM
Gene map locus 1q21

78: #603932
 INTERVERTEBRAL DISC DISEASE; IDD
LUMBAR DISC DISEASE, INCLUDED; LDD, INCLUDED
Gene map locus 20q13.3, 15q22, 1p33-p32.2

79: #603860
MEDULLARY CYSTIC KIDNEY DISEASE 2; MCKD2
Gene map locus 16p12.3

80: #603689
HEREDITARY MYOPATHY WITH EARLY RESPIRATORY FAILURE; HMERF
Gene map locus 2q24.3

81: #603554
OMENN SYNDROME
Gene map locus 11p13, 11p13, 10p

82: #603553
HEMOPHAGOCYTIC LYMPHOHISTIOCYTOSIS, FAMILIAL, 2; FHL2
Gene map locus 10q22

83: #603552
HEMOPHAGOCYTIC LYMPHOHISTIOCYTOSIS, FAMILIAL, 4; FHL4
Gene map locus 6q24

84: #603545
ENLARGED VESTIBULAR AQUEDUCT SYNDROME
Gene map locus 7q31

85: #603373
HYPERTHYROIDISM, FAMILIAL GESTATIONAL
Gene map locus 14q31

86: #603075
MACULAR DEGENERATION, AGE-RELATED, 1; ARMD1
Gene map locus 19q13.2, 1q32, 1q24-q25, 10q25.3-q26.2, 6p21.3, 6p21.3

87: #602481
MIGRAINE, FAMILIAL HEMIPLEGIC, 2; FHM2
MIGRAINE, FAMILIAL BASILAR, INCLUDED
Gene map locus 1q21-q23

88: #602398
DESMOSTEROLOSIS
Gene map locus 1p33-p31.1

89: #602390
HEMOCHROMATOSIS, JUVENILE; JH
HEMOCHROMATOSIS DUE TO DEFECT IN HEMOJUVELIN, INCLUDED
Gene map locus 19q13, 1q21

90: %602082
CORNEAL DYSTROPHY OF BOWMAN LAYER, TYPE II; CDB2
Gene map locus 10q24, 5q31

91: #602036
ERYTHROKERATODERMIA, PROGRESSIVE SYMMETRIC
Gene map locus 1q21

92: #601954
MUSCULAR DYSTROPHY, LIMB-GIRDLE, TYPE 2G; LGMD2G
Gene map locus 17q12

93: #601887
MALIGNANT HYPERTHERMIA, SUSCEPTIBILITY TO, 5
Gene map locus 1q32

94: #601680
ARTHROGRYPOSIS, DISTAL, TYPE 2B; DA2B
Gene map locus 17p13.1, 11p15.5, 11p15.5

95: #601634
NEURAL TUBE DEFECTS, FOLATE-SENSITIVE
SPINA BIFIDA, FOLATE-SENSITIVE, INCLUDED
Gene map locus 1q43, 14q24, 5p15.3-p15.2

96: #601626
LEUKEMIA, ACUTE MYELOID; AML
Gene map locus 21q22.3, 19q13.1, 19p13.3, 16q22, 13q12, 12p13, 11q23.3, 11q14, 10p12, 9q34.1, 9p24, 8p12, 5q35, 5q35, 4q12, 4q11-q12, 3q28, 3q25.1, 3q24

97: #601553
HYPOTRICHOSIS, CONGENITAL, WITH JUVENILE MACULAR DYSTROPHY; HJMD
Gene map locus 16q22.1

98: #601495
AGAMMAGLOBULINEMIA, NON-BRUTON TYPE, AUTOSOMAL RECESSIVE
AGAMMAGLOBULINEMIA, NON-BRUTON TYPE, AUTOSOMAL DOMINANT, INCLUDED
Gene map locus 22q11.21, 14q32.33, 9q34.13

99: #601494
CARDIOMYOPATHY, DILATED, 1D; CMD1D
Gene map locus 1q32

100: #601457
SEVERE COMBINED IMMUNODEFICIENCY, AUTOSOMAL RECESSIVE, T CELL-NEGATIVE, B CELL-NEGATIVE, NK CELL-POSITIVE
Gene map locus 11p13, 11p13

101: #601419
MYOPATHY, MYOFIBRILLAR, DESMIN-RELATED
Gene map locus 2q35

102: %601410
DIABETES MELLITUS, TRANSIENT NEONATAL, 1
Gene map locus 6q24, 6q24, 6q24

103: #601367
STROKE, ISCHEMIC
Gene map locus 13q12

104: #601003
BRODY MYOPATHY
Gene map locus 16p12

105: #601001
EPIDERMOLYSIS BULLOSA SIMPLEX, AUTOSOMAL RECESSIVE
Gene map locus 17q12-q21

106: #600996
ARRHYTHMOGENIC RIGHT VENTRICULAR DYSPLASIA, FAMILIAL, 2; ARVD2
Gene map locus 1q42.1-q43

107: #600995
NEPHROTIC SYNDROME, STEROID-RESISTANT, AUTOSOMAL RECESSIVE; SRN1
NEPHROTIC SYNDROME, IDIOPATHIC, INCLUDED
Gene map locus 1q25-q31

108: #600972
ACHONDROGENESIS, TYPE IB; ACG1B
Gene map locus 5q32-q33.1

109: #600969
EPIPHYSEAL DYSPLASIA, MULTIPLE, 3; EDM3
EPIPHYSEAL DYSPLASIA, MULTIPLE, WITH MYOPATHY, INCLUDED
Gene map locus 20q13.3

110: #600962
PALMOPLANTAR KERATODERMA, NONEPIDERMOLYTIC; NEPPK
Gene map locus 17q12-q21, 12q13

111: #600955
PROPROTEIN CONVERTASE 1 DEFICIENCY
Gene map locus 5q15-q21

112: #600807
ASTHMA, SUSCEPTIBILITY TO
ASTHMA, PROTECTION AGAINST, INCLUDED
Gene map locus 13q14.1, 11q12.3-q13.1, 10q11.2, 6p21.2-p12, 6p21.3, 6p21.3, 5q32-q34, 5q31.1-q33.1, 5q31-q34, 5q31, 2q22

113: #600802
SEVERE COMBINED IMMUNODEFICIENCY, AUTOSOMAL RECESSIVE, T CELL-NEGATIVE, B CELL-POSITIVE, NK CELL-NEGATIVE
Gene map locus 11q23, 5p13

114: #600791
DEAFNESS, NEUROSENSORY, AUTOSOMAL RECESSIVE 4; DFNB4
Gene map locus 7q31

115: #600648
NEVUS, EPIDERMAL, EPIDERMOLYTIC HYPERKERATOTIC TYPE
EPIDERMOLYTIC HYPERKERATOSIS, LINEAR FORM, INCLUDED
Gene map locus 17q21-q22

116: #600512
EPILEPSY, LATERAL TEMPORAL LOBE, AUTOSOMAL DOMINANT; ADLTE
Gene map locus 10q24

117: #600334
TIBIAL MUSCULAR DYSTROPHY, TARDIVE
Gene map locus 2q24.3

118: #600309
ATRIOVENTRICULAR SEPTAL DEFECT; AVSD
ATRIOVENTRICULAR CANAL DEFECT; AVCD
Gene map locus 6q21-q23.2, 1p31-p21

119: #600263
HELICOBACTER PYLORI INFECTION, SUSCEPTIBILITY TO
Gene map locus 7q31.3, 6q23-q24

120: #312920
SPASTIC PARAPLEGIA 2, X-LINKED; SPG2
Gene map locus Xq22

121: #312870
SIMPSON-GOLABI-BEHMEL SYNDROME, TYPE 1; SGBS1
Gene map locus Xq26

122: #312080
PELIZAEUS-MERZBACHER DISEASE; PMD
Gene map locus Xq22

123: #312060
PROPERDIN DEFICIENCY, X-LINKED
PROPERDIN DEFICIENCY, TYPE II, INCLUDED
Gene map locus Xp11.4-p11.23

124: #310200
MUSCULAR DYSTROPHY, DUCHENNE TYPE; DMD
Gene map locus Xp21.2, 12q21

125: #306400
GRANULOMATOUS DISEASE, CHRONIC, X-LINKED; CGD
CYTOCHROME b-POSITIVE GRANULOMATOUS DISEASE, CHRONIC, X-LINKED, INCLUDED
Gene map locus Xp21.1

126: #301050
ALPORT SYNDROME, X-LINKED; ATS
ALPORT SYNDROME-LIKE HEREDITARY NEPHRITIS, INCLUDED; ASLHN, INCLUDED; ASLN, INCLUDED
Gene map locus Xq22.3

127: #300497
ASPERGER SYNDROME, X-LINKED, SUSCEPTIBILITY TO, 2
Gene map locus Xp22.33

128: #300495
AUTISM, X-LINKED, SUSCEPTIBILITY TO, 2
MENTAL RETARDATION, X-LINKED, INCLUDED
Gene map locus Xp22.33

129: #300387
MENTAL RETARDATION, X-LINKED 63; MRX63
Gene map locus Xq22.3

130: #300367
DYSERYTHROPOIETIC ANEMIA WITH THROMBOCYTOPENIA
MACROTHROMBOCYTOPENIA, X-LINKED, INCLUDED
Gene map locus Xp11.23

131: #300306
OBESITY, SUSCEPTIBILITY TO, X-LINKED
Gene map locus Xq23-q24

132: %300195
ALPORT SYNDROME, MENTAL RETARDATION, MIDFACE HYPOPLASIA, AND ELLIPTOCYTOSIS CHROMOSOMAL REGION GENE 1; AMMECR1
Gene map locus Xq22.3

133: #300194
AMME COMPLEX
Gene map locus Xq22.3

134: #300067
 LISSENCEPHALY, X-LINKED
SUBCORTICAL LAMINAR HETEROTOPIA, X-LINKED, INCLUDED; SCLH, INCLUDED
Gene map locus Xq22.3-q23

135: #277950
 WINCHESTER SYNDROME
Gene map locus 16q13

136: #276710
 TYROSINEMIA, TYPE III
Gene map locus 12q24-qter

137: #275000
 GRAVES DISEASE
GRD1, INCLUDED
Gene map locus Xp11, 20q13.11, 4q12, 2q33

138: #274600
 PENDRED SYNDROME; PDS
THYROID HORMONOGENESIS, GENETIC DEFECT IN, 2B, INCLUDED
Gene map locus 7q31

139: #274500
 THYROID HORMONOGENESIS, GENETIC DEFECT IN, 2A
TOTAL IODIDE ORGANIFICATION DEFECT, INCLUDED; TIOD, INCLUDED
Gene map locus 2p25

140: #273800
 THROMBASTHENIA OF GLANZMANN AND NAEGELI
Gene map locus 17q21.32

141: %273300
 TESTICULAR TUMORS
TESTICULAR GERM CELL TUMOR, INCLUDED; TGCT, INCLUDED
Gene map locus 12q22, 4q12

142: #272430
 COLD-INDUCED SWEATING SYNDROME 1; CISS1
Gene map locus 19p12

143: #270970
SPHEROCYTOSIS, AUTOSOMAL RECESSIVE
Gene map locus 1q21

144: #270550
SPASTIC ATAXIA, CHARLEVOIX-SAGUENAY TYPE; SACS
Gene map locus 13q12

145: #269600
SEA-BLUE HISTIOCYTE DISEASE
Gene map locus 19q13.2

146: #269160
SCHIZENCEPHALY
Gene map locus 10q26.1

147: #268150
RH-NULL, REGULATOR TYPE; RHN
RH DEFICIENCY SYNDROME, INCLUDED
Gene map locus 6p21.1-p11

148: #267430
RENAL TUBULAR DYSGENESIS; RTD
Gene map locus 1q42-q43, 1q32, 17q23, 3q21-q25

149: #266600
INFLAMMATORY BOWEL DISEASE 1; IBD1
Gene map locus 19p13, 16q12, 16p, 14q11-q12, 12p13.2-q24.1, 10q23, 6p, 5q31, 3p26, 1p36

150: #266500
REFSUM DISEASE
Gene map locus 10pter-p11.2, 6q22-q24

151: #266140
PYROPOIKILOCYTOSIS, HEREDITARY; HPP
Gene map locus 1q21

152: #265120
PULMONARY ALVEOLAR PROTEINOSIS
PULMONARY ALVEOLAR PROTEINOSIS, CONGENITAL, INCLUDED
Gene map locus 22q12.2-q13.1, 8p21, 2p12-p11.2

153: #263000
PNEUMONITIS, DESQUAMATIVE INTERSTITIAL; DIP
INTERSTITIAL LUNG DISEASE, INCLUDED
Gene map locus 8p21

154: #262650
PITUITARY DWARFISM IV
Gene map locus 17q22-q24

155: #262500
PITUITARY DWARFISM II
Gene map locus 5p13-p12

156: #262400
PITUITARY DWARFISM I
ISOLATED GROWTH HORMONE DEFICIENCY, TYPE IA, INCLUDED
Gene map locus 17q22-q24

157: #261100
MEGALOBLASTIC ANEMIA 1; MGA1
Gene map locus 14q32, 10p12.1

158: #260350
PANCREATIC CARCINOMA
Gene map locus 17p13.1, 13q12.3, 12p12.1, 4q32-q34, 3p21.1

159: #259900
HYPEROXALURIA, PRIMARY, TYPE I
Gene map locus 2q36-q37

160: #259700
OSTEOPETROSIS, AUTOSOMAL RECESSIVE
Gene map locus 16p13, 11q13.4-q13.5, 6q21

161: #259420
OSTEOGENESIS IMPERFECTA, PROGRESSIVELY DEFORMING, WITH NORMAL SCLERAE
Gene map locus 17q21.31-q22, 7q22.1

162: #256500
NETHERTON SYNDROME; NETH
Gene map locus 5q32

163: #256050
NEONATAL OSSEOUS DYSPLASIA I
DE LA CHAPELLE DYSPLASIA, INCLUDED
Gene map locus 5q32-q33.1

164: #256030
NEMALINE MYOPATHY 2; NEM2
Gene map locus 2q22

165: #255310
MYOPATHY, CONGENITAL, WITH FIBER-TYPE DISPROPORTION; CFTD
MYOPATHY, CONGENITAL, WITH FIBER-TYPE DISPROPORTION 1, INCLUDED; CFTD1, INCLUDED
Gene map locus 1q42.1

166: #254770
MYOCLONIC EPILEPSY, JUVENILE, 1; EJM1
Gene map locus 6p12-p11

167: #254600
MYELOPEROXIDASE DEFICIENCY
Gene map locus 17q23.1

168: #254090
ULLRICH CONGENITAL MUSCULAR DYSTROPHY; UCMD
Gene map locus 21q22.3, 21q22.3, 2q37

169: #253700
MUSCULAR DYSTROPHY, LIMB-GIRDLE, TYPE 2C; LGMD2C
Gene map locus 13q12

170: #253600
MUSCULAR DYSTROPHY, LIMB-GIRDLE, TYPE 2A; LGMD2A
Gene map locus 15q15.1-q21.1

171: #252600
MUCOLIPIDOSIS IIIA
MUCOLIPIDOSIS IIIA, ATYPICAL, INCLUDED
Gene map locus 12q23.3

172: #252500
MUCOLIPIDOSIS II
Gene map locus 12q23.3

173: #251880
MITOCHONDRIAL DNA DEPLETION SYNDROME, HEPATOCEREBRAL FORM
Gene map locus 2p23-p21, 16q22, 13q12.2-q13, 2p13

174: #249270
THIAMINE-RESPONSIVE MEGALOBLASTIC ANEMIA SYNDROME; TRMA
Gene map locus 1q23.3

175: #248500
MANNOSIDOSIS, ALPHA B, LYSOSOMAL
ALPHA-MANNOSIDOSIS, TYPE II, INCLUDED
Gene map locus 19cen-q12

176: %248310
MALARIA, INTENSITY OF INFECTION IN
ANTIMALARIAL IMMUNE RESPONSE, INCLUDED
Gene map locus 19p13.3-p13.2, 17cen-q11.2, 7q11.2, 6p21.3, 5q31-q33, 2q14-q21

177: #248300
MAL DE MELEDA
Gene map locus 8qter

178: %246700
CHYLOMICRON RETENTION DISEASE; CMRD
Gene map locus 5q31.1

179: #246300
LEPROSY, SUSCEPTIBILITY TO
Gene map locus 4q32

180: #245900
LECITHIN:CHOLESTEROL ACYLTRANSFERASE DEFICIENCY
Gene map locus 16q22.1

181: #245660
LARYNGOONYCHOCUTANEOUS SYNDROME; LOCS
Gene map locus 18q11.2

182: #245150
KEUTEL SYNDROME
Gene map locus 12p13.1-p12.3

183: #244400
KARTAGENER SYNDROME
Gene map locus 9p21-p13, 7p21, 5p15-p14

184: #242650
PRIMARY CILIARY DYSKINESIA; PCD
CILIARY DYSKINESIA, PRIMARY, 1, INCLUDED; CILD1, INCLUDED
Gene map locus 9p21-p13

185: #241850
HYPOTHYROIDISM, ATHYROIDAL, WITH SPIKY HAIR AND CLEFT PALATE
Gene map locus 9q22

186: #241550
HYPOPLASTIC LEFT HEART SYNDROME
Gene map locus 6q21-q23.2

187: #238600
HYPERLIPOPROTEINEMIA, TYPE I
Gene map locus 8p22

188: #237900
HYPERBILIRUBINEMIA, TRANSIENT FAMILIAL NEONATAL
BREASTFEEDING JAUNDICE, INCLUDED
Gene map locus 2q37

189: #237300
CARBAMOYL PHOSPHATE SYNTHETASE I DEFICIENCY, HYPERAMMONEMIA DUE TO
Gene map locus 2q35

190: #235800
HISTIDINEMIA
Gene map locus 12q22-q23

191: #235730
MOWAT-WILSON SYNDROME
Gene map locus 2q22

192: #233910
GTP CYCLOHYDROLASE I DEFICIENCY
Gene map locus 14q22.1-q22.2

193: #233710
GRANULOMATOUS DISEASE, CHRONIC, AUTOSOMAL RECESSIVE, CYTOCHROME b-POSITIVE, TYPE II
Gene map locus 1q25

194: #232600
GLYCOGEN STORAGE DISEASE V
Gene map locus 11q13

195: #232500
GLYCOGEN STORAGE DISEASE IV
GSD IV, CLASSIC HEPATIC, INCLUDED
Gene map locus 3p12

196: #230450
GAMMA-GLUTAMYLCYSTEINE SYNTHETASE DEFICIENCY, HEMOLYTIC ANEMIA DUE TO
Gene map locus 6p12

197: #229100
FORMIMINOTRANSFERASE DEFICIENCY
Gene map locus 21q22.3

198: #229070
FOLLICLE-STIMULATING HORMONE DEFICIENCY, ISOLATED
Gene map locus 11p13

199: #227810
FANCONI-BICKEL SYNDROME; FBS
Gene map locus 3q26.1-q26.3

200: #226900
EPIPHYSEAL DYSPLASIA, MULTIPLE, 4; EDM4
Gene map locus 5q32-q33.1

201: #226700
EPIDERMOLYSIS BULLOSA LETALIS
EPILIGRIN, INCLUDED
Gene map locus 18q11.2, 1q32, 1q25-q31

202: #226650
EPIDERMOLYSIS BULLOSA, GENERALIZED ATROPHIC BENIGN; GABEB
Gene map locus 18q11.2, 1q32, 17q11-qter, 1q25-q31, 10q24.3

203: #223100
HYPOLACTASIA, ADULT TYPE
LACTASE PERSISTENCE, INCLUDED
Gene map locus 2q21, 2q21

204: #222700
LYSINURIC PROTEIN INTOLERANCE; LPI
Gene map locus 14q11.2

205: #222600
DIASTROPHIC DYSPLASIA
Gene map locus 5q32-q33.1

206: #221770
POLYCYSTIC LIPOMEMBRANOUS OSTEODYSPLASIA WITH SCLEROSING LEUKOENCEPHALOPATHY;
PLOSL
Gene map locus 19q13.1, 6p21.2

207: #220100
CYSTINURIA
CYSTINURIA, TYPE A, INCLUDED
Gene map locus 2p16.3

208: #219100
CUTIS LAXA, AUTOSOMAL RECESSIVE, TYPE I
Gene map locus 14q32.1, 11q13, 5q23.3-q31.2

209: #219050
CRYPTORCHIDISM, UNILATERAL OR BILATERAL
Gene map locus 19p13.2, 13q13.1

210: #218800
CRIGLER-NAJJAR SYNDROME
Gene map locus 2q37

211: #217090
CONJUNCTIVITIS, LIGNEOUS
Gene map locus 6q26

212: #215700
CITRULLINEMIA, CLASSIC
Gene map locus 9q34

213: #215600
CIRRHOSIS, FAMILIAL
CIRRHOSIS, FAMILIAL, WITH PULMONARY HYPERTENSION, INCLUDED
Gene map locus 12q13, 12q13

214: #215140
HYDROPS-ECTOPIC CALCIFICATION-MOTH-EATEN SKELETAL DYSPLASIA
Gene map locus 1q42.1

215: #209300
ATRANSFERRINEMIA
Gene map locus 3q21

216: #208900
ATAXIA-TELANGIECTASIA; AT
AT, COMPLEMENTATION GROUP A, INCLUDED; ATA, INCLUDED
Gene map locus 11q22.3

217: #207900
ARGININOSUCCINIC ACIDURIA
Gene map locus 7cen-q11.2

218: #207800
ARGININEMIA
Gene map locus 6q23

219: #203450
ALEXANDER DISEASE
Gene map locus 17q21, 11q13

220: #202700
NEUTROPENIA, SEVERE CONGENITAL; SCN
KOSTMANN DISEASE, INCLUDED
Gene map locus 19p13.3, 1p22

221: #202400
AFIBRINOGENEMIA, CONGENITAL
HYPOFIBRINOGENEMIA, CONGENITAL, INCLUDED
Gene map locus 4q28, 4q28

222: #202300
ADRENOCORTICAL CARCINOMA, HEREDITARY; ADCC
ADRENOCORTICAL CARCINOMA, PEDIATRIC, INCLUDED
Gene map locus 17p13.1, 11p15.5

223: #202110
ADRENAL HYPERPLASIA, CONGENITAL, DUE TO 17-ALPHA-HYDROXYLASE DEFICIENCY
17,20-@LYASE DEFICIENCY, ISOLATED, INCLUDED
Gene map locus 10q24.3

224: #201710
LIPOID CONGENITAL ADRENAL HYPERPLASIA
Gene map locus 15q23-q24, 8p11.2

225: #201450
ACYL-CoA DEHYDROGENASE, MEDIUM-CHAIN, DEFICIENCY OF
Gene map locus 1p31

226: #201400
ACTH DEFICIENCY
Gene map locus 1q23-q24, 8q13

227: #200610
ACHONDROGENESIS, TYPE II; ACG2
Gene map locus 12q13.11-q13.2

228: %194470
ZINC, ELEVATED PLASMA
HYPERZINCEMIA AND HYPERCALPROTECTINEMIA, INCLUDED
Gene map locus 4q11-q13

229: #194070
WILMS TUMOR 1; WT1
Gene map locus Xq26, 13q12.3, 11p13

230: #193900
WHITE SPONGE NEVUS OF CANNON
Gene map locus 17q21-q22, 12q13

231: #193670
WHIM SYNDROME
Gene map locus 2q21

232: #192600
CARDIOMYOPATHY, FAMILIAL HYPERTROPHIC; CMH
CARDIOMYOPATHY, FAMILIAL HYPERTROPHIC, 1, INCLUDED; CMH1, INCLUDED
Gene map locus 20q13.3, 15q14, 14q12, 14q12, 3p21.3-p14.3, 3p25

233: #190685
DOWN SYNDROME
TRISOMY 21, INCLUDED
Gene map locus Xp11.23, 21q22.3, 1q43

234: #188580
THYROTOXIC PERIODIC PARALYSIS
HASHITOXIC PERIODIC PARALYSIS, INCLUDED
Gene map locus 1q32

235: #187500
TETRALOGY OF FALLOT
Gene map locus 20p12, 8q23, 5q34

236: #186100
SYNDACTYLY, TYPE III
Gene map locus 6q21-q23.2

237: #184500
STEATOCYSTOMA MULTIPLEX
Gene map locus 17q12-q21

238: %184252
SPONDYLOMETAPHYSEAL DYSPLASIA, KOZLOWSKI TYPE
Gene map locus 12q13.11-q13.2

239: #184250
SPONDYLOEPIMETAPHYSEAL DYSPLASIA, STRUDWICK TYPE
Gene map locus 12q13.11-q13.2

240: #182940
NEURAL TUBE DEFECTS
SPINA BIFIDA, INCLUDED
Gene map locus 17q11.2-q12

241: #182920
MYOPATHY, SPHEROID BODY
Gene map locus 5q31

242: #182212
SHPRINTZEN-GOLDBERG CRANIOSYNOSTOSIS SYNDROME
Gene map locus 15q21.1

243: #181450
ULNAR-MAMMARY SYNDROME; UMS
ULNAR-MAMMARY SYNDROME OF PALLISTER, INCLUDED
Gene map locus 12q24.1

244: #180800
ROUSSY-LEVY HEREDITARY AREFLEXIC DYSTASIA
Gene map locus 17p11.2, 1q22

245: #180550
RING DERMOID OF CORNEA; RDC
Gene map locus 4q25-q26

246: #180500
RIEGER SYNDROME, TYPE 1; RIEG1
Gene map locus 4q25-q26

247: #180300
RHEUMATOID ARTHRITIS; RA
Gene map locus 21q22.3, 1q31-q32, 16p13, 1p13, 6p21.3, 5q31, 1p36

248: #180105
RETINITIS PIGMENTOSA 10; RP10
Gene map locus 7q31.3-q32

249: #179850
DOWLING-DEGOS DISEASE; DDD
Gene map locus 12q13

250: #178500
PULMONARY FIBROSIS, IDIOPATHIC
INTERSTITIAL PNEUMONITIS, USUAL, INCLUDED; UIP, INCLUDED
Gene map locus 10q22.2-q23.1, 8p21, 4q31.1

251: #177170
PSEUDOACHONDROPLASTIC DYSPLASIA
Gene map locus 19p13.1

252: #176807
PROSTATE CANCER
Gene map locus Xq27-q28, Xq11-q12, 22q12.1, 20q13, 1q42.2-q43, 19q, 17p11, 16q22.3-q23.1, 1q25, 13q12.3, 11p11.2, 10q25, 10q23.31, 10p15, 8p22, 8p22, 7q11.23, 7p11-q21, 7p22

253: #176200
PORPHYRIA VARIEGATA
Gene map locus 1q22, 6p21.3

254: #176000
PORPHYRIA, ACUTE INTERMITTENT
PORPHYRIA, ACUTE INTERMITTENT, NONERYTHROID VARIANT, INCLUDED
Gene map locus 11q23.3

255: #175780
PORENCEPHALY, FAMILIAL
Gene map locus 13q34

256: #173100
PITUITARY DWARFISM DUE TO ISOLATED GROWTH HORMONE DEFICIENCY, AUTOSOMAL DOMINANT
Gene map locus 17q22-q24

257: #172800
PIEBALD TRAIT; PBT
Gene map locus 8q11

258: #171300
PHEOCHROMOCYTOMA
Gene map locus 11q23, 10q11.2, 5p13.1-p12, 3p26-p25, 1p36.1-p35

259: #170400
HYPOKALEMIC PERIODIC PARALYSIS; HOKPP
Gene map locus 17q23.1-q25.3, 1q32, 11q13-q14

260: #169400
PELGER-HUET ANOMALY; PHA
Gene map locus 1q42.1

261: #168601
PARKINSON DISEASE, FAMILIAL, TYPE 1; PARK1
ATYPICAL PARKINSON DISEASE, INCLUDED
Gene map locus 4q21

262: #168600
PARKINSON DISEASE; PD
PARKINSONISM, INCLUDED
Gene map locus Xq21-q25, 18p11.31-p11.2, 17q21.1, 9q34, 1p, 6q27, 5q23.1-q23.3, 4p14, 2q22-q23, 2p13

263: #167800
PANCREATITIS, HEREDITARY; PCTT
PANCREATITIS, CALCIFIC, INCLUDED
Gene map locus 7q35, 5q32

264: #167210
PACHYONYCHIA CONGENITA, TYPE 2; PC2
Gene map locus 17q12-q21, 12q13

265: #167200
PACHYONYCHIA CONGENITA, TYPE 1; PC1
PACHYONYCHIA CONGENITA TARDA, TYPE 1
Gene map locus 17q12-q21, 12q13

266: #166710
OSTEOPOROSIS, INVOLUTIONAL
Gene map locus 17q21.31-q22, 12q12-q14, 11q13.4, 7q22.1, 7q21.3, 7p21, 5q31.1

267: #166220
OSTEOGENESIS IMPERFECTA, TYPE IV
Gene map locus 17q21.31-q22

268: #166210
OSTEOGENESIS IMPERFECTA CONGENITA; OIC
Gene map locus 17q21.31-q22, 7q22.1

269: #166200
OSTEOGENESIS IMPERFECTA, TYPE I
Gene map locus 17q21.31-q22, 7q22.1

270: #166000
ENCHONDROMATOSIS, MULTIPLE
MAFFUCCI SYNDROME, INCLUDED
Gene map locus 3p22-p21.1

271: #165720
OSTEOARTHRITIS
OSTEOARTHRITIS SUSCEPTIBILITY, FEMALE-SPECIFIC, INCLUDED; OASF, INCLUDED
Gene map locus 12q13.11-q13.2, 11q, 9q21.3-q22, 2q31-q33

272: #164200
OCULODENTODIGITAL DYSPLASIA; ODDD
Gene map locus 6q21-q23.2

273: #162800
CYCLIC HEMATOPOIESIS
Gene map locus 19p13.3

274: #162500
NEUROPATHY, HEREDITARY, WITH LIABILITY TO PRESSURE PALSIES; HNPP
Gene map locus 17p11.2

275: #162000
HYPERURICEMIC NEPHROPATHY, FAMILIAL JUVENILE; HNFJ
Gene map locus 16p12.3

276: #161800
NEMALINE MYOPATHY 3; NEM3
NEMALINE MYOPATHY, TYPICAL, INCLUDED
Gene map locus 1q42.1, 1q22-q23, 9p13.2-p13.1

277: #161550
NASOPHARYNGEAL CARCINOMA
Gene map locus 17p13.1, 4p15.1-q12

278: #160500
MYOPATHY, DISTAL 1; MPD1
Gene map locus 14q12

279: #160150
MYOPATHY, CENTRONUCLEAR, AUTOSOMAL DOMINANT
Gene map locus 19p13.2, 12q21

280: #159000
MUSCULAR DYSTROPHY, LIMB-GIRDLE, TYPE 1A; LGMD1A
Gene map locus 5q31

281: #158810
BETHLEM MYOPATHY
Gene map locus 21q22.3, 21q22.3, 2q37

282: #156550
KNIEST DYSPLASIA
Gene map locus 12q13.11-q13.2

283: #156400
METAPHYSEAL CHONDRODYSPLASIA, JANSEN TYPE
Gene map locus 3p22-p21.1

284: #155255
MEDULLOBLASTOMA
MEDULLOBLASTOMA, DESMOPLASTIC, INCLUDED
Gene map locus 10q25.3-q26.1, 10q24-q25, 9q22.3, 1p32

285: #154700
MARFAN SYNDROME; MFS
Gene map locus 15q21.1

286: #154020
HYPOMAGNESEMIA 2, RENAL; HOMG2
Gene map locus 11q23

287: #153550
CHROMOSOME 5q DELETION SYNDROME
MEGAKARYOCYTES, UNILOBULAR NUCLEATED, INCLUDED
Gene map locus 5q31.1

288: #152700
SYSTEMIC LUPUS ERYTHEMATOSUS; SLE
EXCESS LYMPHOCYTE LOW MOLECULAR WEIGHT DNA, INCLUDED
Gene map locus 1q41-q42, 16p12.3-q12.2, 16p13.3, 1q23, 1q23, 13q32, 12q24, 11q14, 1p13, 4p16-p15.2, 2q37.3

289: #151623
LI-FRAUMENI SYNDROME 1; LFS1
Gene map locus 17p13.1, 9p21

290: #148700
KERATOSIS PALMOPLANTARIS STRIATA I
Gene map locus 18q12.1-q12.2, 12q13

291: #148000
KAPOSI SARCOMA
Gene map locus 7p21

292: #147050
IgE RESPONSIVENESS, ATOPIC; IGER
ATOPY, SUSCEPTIBILITY TO, INCLUDED
Gene map locus 16p12.1-p11.2, 1q23-q25, 13q14.1, 11q12-q13, 6p21.2-p12, 5q33.2, 5q32

293: #146800
ICHTHYOSIS, BULLOUS TYPE
ICHTHYOSIS EXFOLIATIVA, INCLUDED
Gene map locus 12q11-q13

294: #146590
ICHTHYOSIS HYSTRIX, CURTH-MACKLIN TYPE; IHCM
Gene map locus 12q13

295: #146255
HYPOPARATHYROIDISM, SENSORINEURAL DEAFNESS, AND RENAL DISEASE
Gene map locus 10p15

296: #146200
HYPOPARATHYROIDISM, FAMILIAL ISOLATED; FIH
HYPOPARATHYROIDISM, AUTOSOMAL RECESSIVE, INCLUDED
Gene map locus 11p15.3-p15.1, 6p24.2, 3q13.3-q21

297: #145900
HYPERTROPHIC NEUROPATHY OF DEJERINE-SOTTAS
Gene map locus 19q13.1-q13.2, 17p11.2, 1q22, 10q21.1-q22.1

298: #145600
MALIGNANT HYPERTHERMIA, SUSCEPTIBILITY TO, 1; MHS1
KING SYNDROME, INCLUDED
Gene map locus 19q13.1

299: #145500
HYPERTENSION, ESSENTIAL
Gene map locus 2p25-p24, 20q13.11-q13.13, 1p36.1, 20q11-q13, 1q42-q43, 17q21-q22, 17q, 17cen-q11.2, 15q, 12p12.2-p12.1, 12p13, 7q36, 7q22.1, 5p13-q12, 4p16.3, 3q21-q25

300: #144200
 PALMOPLANTAR KERATODERMA, EPIDERMOLYTIC; EPPK
UNILATERAL PALMOPLANTAR VERRUCOUS NEVUS, INCLUDED
Gene map locus 17q12-q21, 17q12-q21

301: #144010
 HYPERCHOLESTEROLEMIA, AUTOSOMAL DOMINANT, TYPE B
Gene map locus 2p24

302: #143890
 HYPERCHOLESTEROLEMIA, AUTOSOMAL DOMINANT
Gene map locus 19p13.2, 1q21-q23, 9q22-q31, 8p21-p12, 7p15, 3p21.2-p14.1

303: #143500
 GILBERT SYNDROME
Gene map locus 2q37

304: %143200
 HYALOIDEORETINAL DEGENERATION OF WAGNER
Gene map locus 12q13.11-q13.2, 5q13-q14

305: #140350
 HAWKINSINURIA
Gene map locus 12q24-qter

306: #137800
 GLIOMA OF BRAIN, FAMILIAL
GLIOBLASTOMA MULTIFORME, INCLUDED; GLM, INCLUDED; GBM, INCLUDED
Gene map locus 17q21.1, 15q23-q26.3, 10q26, 10q25.3-q26.1, 10q24, 10p15.1, 3p25, 2p22-p21

307: #137600
 IRIDOGONIODYSGENESIS, TYPE 2; IRID2
Gene map locus 4q25-q26

308: #137215
 GASTRIC CANCER
Gene map locus 17q21.1, 16q22.1, 12p12.1, 10p15, 5q31.1, 5q21-q22, 1p34.3-p32.1, 2q33-q34, 2q14.2, 2q14

309: #136900
FUNDUS DYSTROPHY, PSEUDOINFLAMMATORY, OF SORSBY; SFD
Gene map locus 22q12.1-q13.2

310: #136120
FISH-EYE DISEASE; FED
Gene map locus 16q22.1

311: #132900
AORTIC ANEURYSM, FAMILIAL THORACIC 4
Gene map locus 16p13.13-p13.12

312: #132700
CYLINDROMATOSIS, FAMILIAL; CYLD
CYLINDROMAS, DERMAL ECCRINE, INCLUDED
Gene map locus 16q12-q13

313: #132400
EPIPHYSEAL DYSPLASIA, MULTIPLE, 1; EDM1
EPIPHYSEAL DYSPLASIA, FAIRBANK TYPE, INCLUDED
Gene map locus 19p13.1

314: #131960
EPIDERMOLYSIS BULLOSA SIMPLEX WITH MOTTLED PIGMENTATION; EBS-MP
Gene map locus 12q13

315: #131800
EPIDERMOLYSIS BULLOSA OF HANDS AND FEET
Gene map locus 17q12-q21, 17q11-qter, 12q13

316: #130650
BECKWITH-WIEDEMANN SYNDROME; BWS
BECKWITH-WIEDEMANN SYNDROME CHROMOSOME REGION, INCLUDED; BWCR, INCLUDED
Gene map locus 11p15.5, 11p15.5, 11p15.5, 5q35

317: #130600
ELLIPTOCYTOSIS, RHESUS-UNLINKED TYPE
Gene map locus 1q21

318: #130060
EHLERS-DANLOS SYNDROME, TYPE VII, AUTOSOMAL DOMINANT
EDS VIIA1, INCLUDED
Gene map locus 17q21.31-q22, 7q22.1

319: #130050
EHLERS-DANLOS SYNDROME, TYPE IV, AUTOSOMAL DOMINANT
Gene map locus 2q31

320: #130020
EHLERS-DANLOS SYNDROME, TYPE III
Gene map locus 6p21.3, 2q31

321: #130000
EHLERS-DANLOS SYNDROME, TYPE I
Gene map locus 17q21.31-q22, 9q34.2-q34.3, 2q31

322: #129600
ECTOPIA LENTIS, ISOLATED
Gene map locus 15q21.1

323: #128230
DYSTONIA, PROGRESSIVE, WITH DIURNAL VARIATION
Gene map locus 14q22.1-q22.2

324: #127750
DEMENTIA, LEWY BODY; DLB
DIFFUSE LEWY BODY DISEASE WITH GAZE PALSY, INCLUDED
Gene map locus 5q35, 4q21

325: #126600
DOYNE HONEYCOMB RETINAL DYSTROPHY; DHRD
MALATTIA LEVENTINESE, INCLUDED; MLVT, INCLUDED
Gene map locus 2p16

326: #126200
MULTIPLE SCLEROSIS, SUSCEPTIBILITY TO; MS
Gene map locus 1q31-q32, 16p13, 6q21, 6p21.3, 6p21.3

327: #125853
DIABETES MELLITUS, NONINSULIN-DEPENDENT; NIDDM
INSULIN RESISTANCE, SUSCEPTIBILITY TO, INCLUDED
Gene map locus 20q12-q13.1, 20q12-q13.1, 19q13.1-q13.2, 19p13.2, 17q25, 17cen-q21.3, 13q34, 13q12.1, 12q24.2, 11p12-p11.2, 10q25.3, 7p15-p13, 6q22-q23, 6p12, 5q34-q35.2, 2q32, 2q24.1

328: #125850
MATURITY-ONSET DIABETES OF THE YOUNG, TYPE I; MODY1
Gene map locus 20q12-q13.1, 11p15.5

329: #123450
CRI-DU-CHAT SYNDROME
Gene map locus 5p15.2

330: #122200
CORNEAL DYSTROPHY, LATTICE TYPE I; CDL1
Gene map locus 5q31

331: #121900
GROENOUW TYPE I CORNEAL DYSTROPHY; CDGG1
Gene map locus 5q31

332: #118300
CHARCOT-MARIE-TOOTH DISEASE AND DEAFNESS
Gene map locus 17p11.2

333: #118220
CHARCOT-MARIE-TOOTH DISEASE, DEMYELINATING, TYPE 1A; CMT1A
Gene map locus 17p11.2

334: #118200
CHARCOT-MARIE-TOOTH DISEASE, DEMYELINATING, TYPE 1B; CMT1B
Gene map locus 1q22

335: #117000
CENTRAL CORE DISEASE OF MUSCLE
MINICORE MYOPATHY, MODERATE, WITH HAND INVOLVEMENT, INCLUDED
Gene map locus 19q13.1

336: #116920
LEUKOCYTE ADHESION DEFICIENCY, TYPE I; LAD
Gene map locus 21q22.3

337: #115310
PARAGANGLIOMAS 4; PGL4
Gene map locus 1p36.1-p35

338: #115210
CARDIOMYOPATHY, FAMILIAL RESTRICTIVE, 1; RCM1
Gene map locus 19q13.4

339: #115196
CARDIOMYOPATHY, FAMILIAL HYPERTROPHIC, 3; CMH3
Gene map locus 15q22.1

340: #115195
CARDIOMYOPATHY, FAMILIAL HYPERTROPHIC, 2; CMH2
Gene map locus 1q32

341: #114550
HEPATOCELLULAR CARCINOMA
Gene map locus 17p13.1, 16p13.3, 8p22-p21.3, 7q31, 3p22-p21.3, 2q33-q34

342: #114500
COLORECTAL CANCER; CRC
Gene map locus 2p25, 22q13, 20q13.2-q13.3, 17q24, 17p11.2, 17p13.1, 15q15, 14q24.3, 11p11.2, 8p22-p21.3, 1p13.2, 3q26.3, 1p35

343: #114480
BREAST CANCER
BREAST CANCER, FAMILIAL MALE, INCLUDED
Gene map locus 22q12.1, 17q22-q23, 17q22, 17p13.1, 15q15.1, 13q12.3, 12p12.1, 11q22.3, 11p15.5, 8q11, 3q26.3, 2q34-q35

344: #114000
CAFFEY DISEASE
PRENATAL CORTICAL HYPEROSTOSIS, LETHAL, INCLUDED
Gene map locus 17q21.31-q22

345: #113970
BURKITT LYMPHOMA; BL
Gene map locus 8q24.12-q24.13

346: #113800
BULLOUS ERYTHRODERMA ICHTHYOSIFORMIS CONGENITA OF BROCQ
EPIDERMOLYTIC HYPERKERATOSIS, LATE-ONSET, INCLUDED
Gene map locus 17q21-q22, 12q13

347: #112100
BLOOD GROUP--Yt SYSTEM; YT
Gene map locus 7q22

348: #111750
BLOOD GROUP--SCIANNA SYSTEM; SC
Gene map locus 1p34

349: #111620
RADIN BLOOD GROUP ANTIGEN; RD
Gene map locus 1p34

350: #108900
ATRIAL SEPTAL DEFECT WITH ATRIOVENTRICULAR CONDUCTION DEFECTS
Gene map locus 5q34

351: #108300
STICKLER SYNDROME, TYPE I; STL1
Gene map locus 12q13.11-q13.2

352: #108120
ARTHROGRYPOSIS, DISTAL, TYPE 1; DA1
Gene map locus 9p13.2-p13.1

353: #106100
ANGIOEDEMA, HEREDITARY; HAE
ANGIOEDEMA, HEREDITARY, TYPE II, INCLUDED
Gene map locus 11q11-q13.1

354: #105400
 AMYOTROPHIC LATERAL SCLEROSIS 1; ALS1
AMYOTROPHIC LATERAL SCLEROSIS, AUTOSOMAL RECESSIVE, INCLUDED
Gene map locus 22q12.2, 21q22.1, 14q11, 12q12-q13, 2p13

355: #105200
 AMYLOIDOSIS, FAMILIAL VISCERAL
Gene map locus Chr.12, 4q28

356: #104310
 ALZHEIMER DISEASE 2
Gene map locus 19q13.2

357: #104300
 ALZHEIMER DISEASE; AD
ALZHEIMER DISEASE, FAMILIAL, 1, INCLUDED; AD1, INCLUDED
Gene map locus 20p, 19p13.2, 17q23.1, 17q23, 17q11.2, 12p11.23-q13.12,
12p13.3-p12.3, 10q24, 10q24, 7q36, 7q36, 7q36, 4p14

358: #104290
 ALTERNATING HEMIPLEGIA OF CHILDHOOD
Gene map locus 1q21-q23

359: #103900
 HYPERALDOSTERONISM, FAMILIAL, TYPE I
Gene map locus 8q21

360: #103780
 ALCOHOL DEPENDENCE
Gene map locus 13q14-q21, 4q22, 4p13-p12

361: #102700
 SEVERE COMBINED IMMUNODEFICIENCY, AUTOSOMAL RECESSIVE, T CELL-NEGATIVE, B
CELL-NEGATIVE, NK CELL-NEGATIVE, DUE TO ADENOSINE DEAMINASE DEFICIENCY
SCID DUE TO ADA DEFICIENCY, DELAYED ONSET, INCLUDED
Gene map locus 20q13.11

1. Hippocampus

1.1 Hippocampus selective genes (118 genes)

Symbol	Description	Chromosome	Chromosome Location	LocusLink	Cytoband	UniGene
DIRAS2	DIRAS family, GTP-binding RAS-like 2	9	-92411933	54769	9q22.2	Hs.165636
PTPRZ1	protein tyrosine phosphatase, receptor-type, Z polypeptide 1	7	121300394	5803	7q31.3	Hs.489824
GRIA2	glutamate receptor, ionotropic, AMPA 2	4	158361296	2891	4q32-q33	Hs.32763
CADPS	Ca2+-dependent secretion activator	3	-62359061	8618	3p14.2	Hs.127013
GPM6A	glycoprotein M6A	4	-176791082	2823	4q34	Hs.75819
RAPGEF4	Rap guanine nucleotide exchange factor (GEF) 4	2	173308852	11069	2q31-q32	Hs.470646
GARNL1	GTPase activating Rap/RanGAP domain-like 1	14	-35077309	253959	14q13.2	Hs.113150 Hs.136901
KIAA1598	KIAA1598	10	-118634297	57698	10q25.3	Hs.501140
PLP1	proteolipid protein 1 (Pelizaeus-Merzbacher disease, spastic paraplegia 2, uncomplicated)	X	102918409	5354	Xq22	Hs.1787
GFAP	glial fibrillary acidic protein	17	-40338518	2670	17q21	Hs.514227
MYT1L	myelin transcription factor 1-like	2	-1771891	23040	2p25.3	Hs.434418
NAP1L2	nucleosome assembly protein 1-like 2	X	-72348879	4674	Xq13	Hs.66180
KLHL2	kelch-like 2, Mayven (Drosophila)	4	166348240	11275	4q21.2	Hs.388668
AGXT2L1	alanine-glyoxylate aminotransferase 2-like 1	4	-109882652	64850	4q25	Hs.106576
GABRB1	gamma-aminobutyric acid (GABA) A receptor, beta 1	4	46728335	2560	4p12	Hs.27283
PMP2	peripheral myelin protein 2	8	-82515120	5375	8q21.3-q22.1	Hs.571512
TMEM16C	transmembrane protein 16C	11	26309546	63982	11p14.2	Hs.91791
MT3	metallothionein 3 (growth inhibitory factor (neurotrophic))	16	55180767	4504	16q13	Hs.73133
RGS7	regulator of G-protein signalling 7	1	-239005493	6000	1q43|1q23.1	Hs.130171
DCAMKL1	doublecortin and CaM kinase-like 1	13	-35243477	9201	13q13	Hs.507755
NAP1L3	nucleosome assembly protein 1-like 3	X	-92812589	4675	Xq21.3-q22	Hs.21365
PCDH8	protocadherin 8	13	-52316111	5100	13q14.3-q21.1	Hs.19492
CHL1	cell adhesion molecule with homology to L1CAM (close homolog of L1)	3	213649	10752	3p26.1	Hs.148909
TUBB2B	tubulin, beta 2B	6	-3169513	347733	6p25	Hs.300701
RTN1	reticulon 1	14	-59132446	6252	14q23.1	Hs.368626
MKL2	MKL/myocardin-like 2	16	14072696	57496	16p13.12	Hs.592047
COL5A2	collagen, type V, alpha 2	2	-189604886	1290	2q14-q32	Hs.445827
GJA1	gap junction protein, alpha 1, 43kDa (connexin 43)	6	121798486	2697	6q21-q23.2	Hs.74471
TAC1	tachykinin, precursor 1 (substance K, substance P, neurokinin 1, neurokinin 2, neuromedin L, neurokinin alpha, neuropeptide K, neuropeptide gamma)	7	97199310	6863	7q21-q22	Hs.2563
GPR37	G protein-coupled receptor 37 (endothelin receptor type B-like)	7	-124173351	2861	7q31	Hs.406094
BTBD3	BTB (POZ) domain containing 3	20	11846564, 11819476	22903	20p12.2	Hs.244590
MT1M	metallothionein 1M	16	55224072	4499	16q13	Hs.643532
WIF1	WNT inhibitory factor 1	12	-63730673	11197	12q14.3	Hs.284122

GABRA5	gamma-aminobutyric acid (GABA) A receptor, alpha 5	15	24663150	2558	15q11.2-q12	Hs.24969
BAALC	brain and acute leukemia, cytoplasmic	8	104222096	79870	8q22.3	Hs.533446
SPP1	secreted phosphoprotein 1 (osteopontin, bone sialoprotein I, early T-lymphocyte activation 1)	4	89115825	6696	4q21-q25	Hs.313
LPPR4	NA	1	99502487	9890	1p21.2	Hs.13245
SCN3A	sodium channel, voltage-gated, type III, alpha	2	-165652285	6328	2q24	Hs.435274
HPCA	Hippocalcin	1	33124684	3208	1p35-p34.2	Hs.632391
LGI1	leucine-rich, glioma inactivated 1	10	95507667	9211	10q24	Hs.533670
GPM6B	glycoprotein M6B	X	-27401000	2824	Xp22.2	Hs.495710
LY6H	lymphocyte antigen 6 complex, locus H	8	-144310706	4062	8q24.3	Hs.159590
SCG5	secretogranin V (7B2 protein)	15	30721251	6447	15q13-q14	Hs.156540
SNAP25	synaptosomal-associated protein, 25kDa	20	10147476	6616	20p12-p11.2	Hs.167317
WDR47	WD repeat domain 47	1	-109314359	22911	1p13.3	Hs.485081
PLK2	polo-like kinase 2 (Drosophila)	5	-57785568	10769	5q12.1-q13.2	Hs.398157 Hs.642858
WASF1	WAS protein family, member 1	6	-110527714	8936	6q21-q22	Hs.75850
FGF13	fibroblast growth factor 13	X	-137541401	2258	Xq26.3	Hs.6540
NEF3	neurofilament 3 (150kDa medium)	8	24827211	4741	8p21	Hs.458657 Hs.615287
ZFHX1B	zinc finger homeobox 1b	2	-144862053	9839	2q22	Hs.34871
INPP5F	inositol polyphosphate-5-phosphatase F	10	121475598, 121568775	22876	10q26.11	Hs.369755
DNM3	dynamin 3	1	170077260	26052	1q24.3	Hs.584880
GDF1	growth differentiation factor 1	19	-18840360	2657	19p12	Hs.412355
ERBB2IP	erbb2 interacting protein	5	65258139	55914	5q12.3	Hs.591774
CAMK2B	calcium/calmodulin-dependent protein kinase (CaM kinase) II beta	7	-44225421	816	22q12|7p14.3-p14.1	Hs.351887
RPIP8	NA	17	39741489	10900	17q21.31	Hs.500197
HOMER1	homer homolog 1 (Drosophila)	5	-78705542	9456	5q14.2	Hs.591761
S100B	S100 calcium binding protein, beta (neural)	21	-46842958	6285	21q22.3	Hs.422181
PTBP2	polypyrimidine tract binding protein 2	1	96959965	58155	1p22.1-p21.3	Hs.591430
FLJ22028	NA	12	21481877	79912	12p12.1	Hs.46835
STMN2	stathmin-like 2	8	80685934	11075	8q21.13	Hs.521651
SEH1L	SEH1-like (S. cerevisiae)	18	12937982	81929	18p11.21	Hs.301048
AK5	adenylate kinase 5	1	77520874, 77520329	26289	1p31	Hs.559718
NEFH	neurofilament, heavy polypeptide 200kDa	22	28206218	4744	22q12.2	Hs.198760
DNM1	dynamin 1	9	130005483	1759	9q34	Hs.522413
TCEAL2	transcription elongation factor A (SII)-like 2	X	101267315	140597	Xq22.1-q22.3	Hs.401835
TBPL1	TBP-like 1	6	134315993	9519	6q22.1-q22.3	Hs.486507
ATP1A2	ATPase, Na+/K+ transporting, alpha 2 (+) polypeptide	1	158352171	477	1q21-q23	Hs.34114
SH3GL2	SH3-domain GRB2-like 2	9	17569193	6456	9p22	Hs.75149
DKK3	dickkopf homolog 3 (Xenopus laevis)	11	-11941120	27122	11p15.2	Hs.292156
EMX2	empty spiracles homolog 2 (Drosophila)	10	119291945	2018	10q26.1	Hs.202095
PLEKHB1	pleckstrin homology domain containing, family B (evectins) member 1	11	73034870	58473	11q13.5-q14.1	Hs.445489
DGKZ	diacylglycerol kinase, zeta 104kDa	11	46325594, 46311314, 46323562	8525	11p11.2	Hs.502461
SACS	spastic ataxia of Charlevoix-Saguenay (sacsin)	13	-22800966	26278	13q12	Hs.159492
SCHIP1	schwannomin interacting protein 1	3	160474237	29970	3q25.33	Hs.134665

ST6GALNAC5	ST6 (alpha-N-acetyl-neuraminyl-2,3-beta-galactosyl-1,3)-N-acetylgalactosaminide alpha-2,6-sialyltransferase 5	1	77105773	81849	1p31.1	Hs.303609
SYNGR3	synaptogyrin 3	16	1979968	9143	16p13	Hs.435277
NRN1	neuritin 1	6	-5943233	51299	6p25.1	Hs.103291
RUFY3	RUN and FYVE domain containing 3	4	71806575, 71806561	22902	4q13.3	Hs.7972
SLC6A1	solute carrier family 6 (neurotransmitter transporter, GABA), member 1	3	11009455	6529	3p25-p24	Hs.443874
TRIM9	tripartite motif-containing 9	14	-101044267	114088	14q22.1	Hs.368928
NLGN4X	neuroligin 4, X-linked	X	-5818084	57502	Xp22.32-p22.31	Hs.21107
SCG2	secretogranin II (chromogranin C)	2	-224169903	7857	2q35-q36	Hs.516726
TUBB2A	tubulin, beta 2A	6	-3098902	7280	6p25	Hs.300701
ZNF312	zinc finger protein 312	3	-62330403	55079	3p14.2	Hs.241523
PSD3	pleckstrin and Sec7 domain containing 3	8	-18432348	23362	8pter-p23.3	Hs.434255 Hs.592598
ROPN1L	ropporin 1-like	5	10495014	83853	5p15.2	Hs.381089
KL	Klotho	13	32488570	9365	13q12	Hs.524953
SATB2	SATB family member 2	2	-199842468	23314	2q33	Hs.516617
SNAP91	synaptosomal-associated protein, 91kDa homolog (mouse)	6	-84319331	9892	6q14.2	Hs.368046
BAI3	brain-specific angiogenesis inhibitor 3	6	69404157	577	6q12	Hs.13261
KCNB1	potassium voltage-gated channel, Shab-related subfamily, member 1	20	-47421911	3745	20q13.2	Hs.84244
GNG10	guanine nucleotide binding protein (G protein), gamma 10	9	113463681	2790	9q31.3	Hs.534196 Hs.577252
LOC552891	NA	9	113433486	552891	9q31.3	Hs.534196
NPTXR	neuronal pentraxin receptor	22	-37544403	23467	22q13.1	Hs.91622
BBS10	Bardet-Biedl syndrome 10	12	-75262396	79738	12q21.2	Hs.96322
TRIM36	tripartite motif-containing 36	5	-343564200	55521	5q22.3	Hs.519514 Hs.638953
RYR3	ryanodine receptor 3	15	31390468	6263	15q14-q15	Hs.369250
LRP1B	low density lipoprotein-related protein 1B (deleted in tumors)	2	-140705465	53353	2q21.2	Hs.470117
ST18	suppression of tumorigenicity 18 (breast carcinoma) (zinc finger protein)	8	-53185951	9705	8q11.23	Hs.147170
CHGB	chromogranin B (secretogranin 1)	20	5840167	1114	20pter-p12	Hs.516874
EEF1A2	eukaryotic translation elongation factor 1 alpha 2	20	-61589809	1917	20q13.3	Hs.433839
DSCAML1	Down syndrome cell adhesion molecule like 1	11	-116803698	57453	NA	Hs.413137
UBQLN2	ubiquilin 2	X	56606796	29978	Xp11.23-p11.1	Hs.179309
TF	Transferring	3	134947924	7018	3q22.1	Hs.518267
KIF5C	kinesin family member 5C	2	NA	3800	2q23.1	Hs.435557
ARNT2	aryl-hydrocarbon receptor nuclear translocator 2	15	78483746	9915	15q24	Hs.459070
NFASC	neurofascin homolog (chicken)	1	203064445	23114	1q32.1	Hs.13349
PLEKHA5	pleckstrin homology domain containing, family A member 5	12	19173994	54477	12p12	Hs.188614
LOC653754	NA	8	NA	653754	8p22	Hs.592598
PCP4	Purkinje cell protein 4	21	40161216	5121	21q22.2	Hs.80296
SH3YL1	SH3 domain containing, Ysc84-like 1 (S. cerevisiae)	2	-208154	26751	2p25.3	Hs.515951
BNIP3	BCL2/adenovirus E1B 19kDa interacting protein 3	10	-133631193	664	10q26.3	Hs.144873 Hs.562561
DMXL1	Dmx-like 1	5	118435083	1657	5q22	Hs.181042
CTNND2	catenin (cadherin-associated protein), delta 2 (neural plakophilin-related arm-repeat protein)	5	-11024951	1501	5p15.2	Hs.314543

SC4MOL	sterol-C4-methyl oxidase-like	4	166468267	6307	4q32-q34	Hs.105269 Hs.593050
FABP7	fatty acid binding protein 7, brain	6	123142344	2173	6q22-q23	Hs.26770
PHLPP	PH domain and leucine rich repeat protein phosphatase	18	58533713	23239	18q21.33	Hs.465337

1.2 GO: Biological Process

GO ID	genes present in chip	genes present in tissues	p-value	GO term
GO:0007399	385	16	5.45E-07	neurogenesis
GO:0009887	807	21	1.53E-05	organogenesis
GO:0007417	101	7	4.63E-05	central nervous system development
GO:0048513	872	21	4.89E-05	organ development
GO:0009653	1051	23	8.77E-05	morphogenesis
GO:0001666	14	3	0.000281256	response to hypoxia
GO:0007275	1543	28	0.000326573	development
GO:0051179	1857	31	0.000616049	localization
GO:0050926	5	2	0.000879626	regulation of positive chemotaxis
GO:0050927	5	2	0.000879626	positive regulation of positive chemotaxis
GO:0050918	5	2	0.000879626	positive chemotaxis
GO:0050930	5	2	0.000879626	induction of positive chemotaxis
GO:0019725	122	6	0.001046304	cell homeostasis
GO:0051234	1841	30	0.001192847	establishment of localization
GO:0050921	6	2	0.001311274	positive regulation of chemotaxis
GO:0007267	465	12	0.00142415	cell-cell signaling
GO:0030500	7	2	0.001824431	regulation of bone mineralization
GO:0050920	7	2	0.001824431	regulation of chemotaxis
GO:0006810	1802	29	0.00183443	transport
GO:0030003	98	5	0.002362154	cation homeostasis
GO:0030278	8	2	0.002417541	regulation of ossification
GO:0046850	8	2	0.002417541	regulation of bone remodeling
GO:0046883	8	2	0.002417541	regulation of hormone secretion
GO:0006873	103	5	0.002934285	cell ion homeostasis
GO:0042592	157	6	0.003754072	homeostasis
GO:0030282	10	2	0.003837512	bone mineralization
GO:0050801	111	5	0.004049106	ion homeostasis
GO:0046879	11	2	0.004661369	hormone secretion
GO:0030182	13	2	0.006529473	neuron differentiation
GO:0030595	13	2	0.006529473	immune cell chemotaxis
GO:0050900	14	2	0.007570843	immune cell migration
GO:0007154	2730	37	0.007815183	cell communication
GO:0050767	15	2	0.008681872	regulation of neurogenesis
GO:0031175	46	3	0.009421424	neurite morphogenesis
GO:0045175	1	1	0.009521701	basal protein localization
GO:0048151	1	1	0.009521701	hyperphosphorylation
GO:0019430	1	1	0.009521701	removal of superoxide radicals
GO:0048143	1	1	0.009521701	astrocyte activation
GO:0048245	1	1	0.009521701	eosinophil chemotaxis
GO:0045844	1	1	0.009521701	positive regulation of myogenesis
GO:0045837	1	1	0.009521701	negative regulation of membrane potential
GO:0009151	1	1	0.009521701	purine deoxyribonucleotide metabolism

GO:0009153	1	1	0.009521701	purine deoxyribonucleotide biosynthesis
GO:0009180	1	1	0.009521701	purine ribonucleoside diphosphate biosynthesis
GO:0009182	1	1	0.009521701	purine deoxyribonucleoside diphosphate metabolism
GO:0009183	1	1	0.009521701	purine deoxyribonucleoside diphosphate biosynthesis
GO:0009188	1	1	0.009521701	ribonucleoside diphosphate biosynthesis
GO:0009189	1	1	0.009521701	deoxyribonucleoside diphosphate biosynthesis
GO:0009179	1	1	0.009521701	purine ribonucleoside diphosphate metabolism
GO:0009133	1	1	0.009521701	nucleoside diphosphate biosynthesis
GO:0009135	1	1	0.009521701	purine nucleoside diphosphate metabolism
GO:0009136	1	1	0.009521701	purine nucleoside diphosphate biosynthesis
GO:0045917	1	1	0.009521701	positive regulation of complement activation
GO:0016264	1	1	0.009521701	gap junction assembly
GO:0046056	1	1	0.009521701	dADP metabolism
GO:0046031	1	1	0.009521701	ADP metabolism
GO:0030449	1	1	0.009521701	regulation of complement activation
GO:0006172	1	1	0.009521701	ADP biosynthesis
GO:0006173	1	1	0.009521701	dADP biosynthesis
GO:0007268	192	6	0.009828186	synaptic transmission
GO:0006334	48	3	0.010589721	nucleosome assembly
GO:0019226	198	6	0.011328793	transmission of nerve impulse
GO:0006875	93	4	0.011769613	metal ion homeostasis
GO:0006887	55	3	0.015317531	exocytosis
GO:0031497	56	3	0.016075284	chromatin assembly
GO:0007218	56	3	0.016075284	neuropeptide signaling pathway
GO:0007186	491	10	0.017808997	G-protein coupled receptor protein signaling pathway
GO:0030501	2	1	0.018953783	positive regulation of bone mineralization
GO:0048169	2	1	0.018953783	regulation of long-term neuronal synaptic plasticity
GO:0046902	2	1	0.018953783	regulation of mitochondrial membrane permeability
GO:0001937	2	1	0.018953783	negative regulation of endothelial cell proliferation
GO:0045778	2	1	0.018953783	positive regulation of ossification
GO:0042055	2	1	0.018953783	neuron lineage restriction
GO:0016081	2	1	0.018953783	synaptic vesicle docking during exocytosis
GO:0050774	2	1	0.018953783	negative regulation of dendrite morphogenesis
GO:0042391	2	1	0.018953783	regulation of membrane potential
GO:0009185	2	1	0.018953783	ribonucleoside diphosphate metabolism
GO:0009186	2	1	0.018953783	deoxyribonucleoside diphosphate metabolism
GO:0009263	2	1	0.018953783	deoxyribonucleotide biosynthesis
GO:0007206	2	1	0.018953783	metabotropic glutamate receptor, phospholipase C activating pathway
GO:0051402	2	1	0.018953783	programmed cell death, neurons
GO:0046852	2	1	0.018953783	positive regulation of bone remodeling
GO:0051046	24	2	0.021592917	regulation of secretion
GO:0016192	296	7	0.022402501	vesicle-mediated transport
GO:0007165	2168	29	0.026143834	signal transduction
GO:0030502	3	1	0.02829708	negative regulation of bone mineralization
GO:0045103	3	1	0.02829708	intermediate filament-based process
GO:0045104	3	1	0.02829708	intermediate filament cytoskeleton organization and biogenesis

GO:0008105	3	1	0.02829708	asymmetric protein localization
GO:0050796	3	1	0.02829708	regulation of insulin secretion
GO:0050773	3	1	0.02829708	regulation of dendrite morphogenesis
GO:0007320	3	1	0.02829708	insemination
GO:0009132	3	1	0.02829708	nucleoside diphosphate metabolism
GO:0030279	3	1	0.02829708	negative regulation of ossification
GO:0046851	3	1	0.02829708	negative regulation of bone remodeling
GO:0006898	28	2	0.028858151	receptor mediated endocytosis
GO:0006461	194	5	0.037364664	protein complex assembly
GO:0001504	4	1	0.037552419	neurotransmitter uptake
GO:0045197	4	1	0.037552419	establishment and/or maintenance of epithelial cell polarity
GO:0048168	4	1	0.037552419	regulation of neuronal synaptic plasticity
GO:0035088	4	1	0.037552419	establishment and/or maintenance of apical/basal cell polarity
GO:0015991	4	1	0.037552419	ATP hydrolysis coupled proton transport
GO:0050768	4	1	0.037552419	negative regulation of neurogenesis
GO:0007158	4	1	0.037552419	neuron adhesion
GO:0016486	4	1	0.037552419	peptide hormone processing
GO:0009953	4	1	0.037552419	dorsal/ventral pattern formation
GO:0006836	33	2	0.039099672	neurotransmitter transport
GO:0030154	409	8	0.040403759	cell differentiation
GO:0030001	269	6	0.042833161	metal ion transport
GO:0007610	269	6	0.042833161	behavior
GO:0019098	5	1	0.046720617	reproductive behavior
GO:0030641	5	1	0.046720617	hydrogen ion homeostasis
GO:0001936	5	1	0.046720617	regulation of endothelial cell proliferation
GO:0008634	5	1	0.046720617	negative regulation of survival gene product activity
GO:0016358	5	1	0.046720617	dendrite morphogenesis
GO:0007217	5	1	0.046720617	tachykinin signaling pathway
GO:0030073	5	1	0.046720617	insulin secretion
GO:0007617	5	1	0.046720617	mating behavior
GO:0046903	208	5	0.0479892	secretion
GO:0051258	37	2	0.048134805	protein polymerization
GO:0030005	87	3	0.049898301	di-, tri-valent inorganic cation homeostasis
GO:0001503	38	2	0.050501789	ossification
GO:0031214	38	2	0.050501789	biomineral formation
GO:0007409	40	2	0.055358581	axonogenesis
GO:0006811	515	9	0.055550661	ion transport
GO:0006333	91	3	0.05568402	chromatin assembly or disassembly
GO:0001935	6	1	0.055802485	endothelial cell proliferation
GO:0015988	6	1	0.055802485	energy coupled proton transport, against electrochemical gradient
GO:0016079	6	1	0.055802485	synaptic vesicle exocytosis
GO:0007216	6	1	0.055802485	metabotropic glutamate receptor signaling pathway
GO:0030072	6	1	0.055802485	peptide hormone secretion
GO:0046849	41	2	0.057846445	bone remodeling
GO:0048167	7	1	0.064798827	regulation of synaptic plasticity
GO:0045884	7	1	0.064798827	regulation of survival gene product activity

GO:0009262	7	1	0.064798827	deoxyribonucleotide metabolism
GO:0009966	229	5	0.066984993	regulation of signal transduction
GO:0030004	8	1	0.07371044	monovalent inorganic cation homeostasis
GO:0007568	8	1	0.07371044	aging
GO:0009636	8	1	0.07371044	response to toxin
GO:0030317	8	1	0.07371044	sperm motility
GO:0006457	169	4	0.077319836	protein folding
GO:0009968	50	2	0.081854442	negative regulation of signal transduction
GO:0050877	475	8	0.081897206	neurophysiological process
GO:0009628	318	6	0.082142963	response to abiotic stimulus
GO:0016202	9	1	0.082538111	regulation of myogenesis
GO:0006309	9	1	0.082538111	DNA fragmentation during apoptosis
GO:0030178	9	1	0.082538111	negative regulation of Wnt receptor signaling pathway
GO:0009582	109	3	0.085388326	detection of abiotic stimulus
GO:0006813	111	3	0.089036676	potassium ion transport
GO:0006897	115	3	0.096527191	endocytosis
GO:0007200	56	2	0.099269144	G-protein signaling, coupled to IP3 second messenger (phospholipase C activating)
GO:0048489	11	1	0.099944747	synaptic vesicle transport
GO:0006921	11	1	0.099944747	disassembly of cell structures during apoptosis
GO:0007006	11	1	0.099944747	mitochondrial membrane organization and biogenesis
GO:0030262	11	1	0.099944747	apoptotic nuclear changes
GO:0006325	186	4	0.10113349	establishment and/or maintenance of chromatin architecture
GO:0007018	59	2	0.10833407	microtubule-based movement
GO:0051240	59	2	0.10833407	positive regulation of organismal physiological process
GO:0030705	59	2	0.10833407	cytoskeleton-dependent intracellular transport
GO:0042102	12	1	0.108525254	positive regulation of T cell proliferation
GO:0006414	12	1	0.108525254	translational elongation
GO:0001709	12	1	0.108525254	cell fate determination
GO:0006812	346	6	0.111440666	cation transport
GO:0006323	193	4	0.111825338	DNA packaging
GO:0006800	61	2	0.114496135	oxygen and reactive oxygen species metabolism
GO:0007166	952	13	0.115632415	cell surface receptor linked signal transduction
GO:0007043	13	1	0.117024902	intercellular junction assembly
GO:0007214	13	1	0.117024902	gamma-aminobutyric acid signaling pathway
GO:0007155	518	8	0.119099306	cell adhesion
GO:0006826	14	1	0.125444445	iron ion transport
GO:0009220	14	1	0.125444445	pyrimidine ribonucleotide biosynthesis
GO:0007602	14	1	0.125444445	phototransduction
GO:0006308	14	1	0.125444445	DNA catabolism
GO:0006928	205	4	0.131271283	cell motility
GO:0040011	205	4	0.131271283	locomotion
GO:0051239	133	3	0.133165095	regulation of organismal physiological process
GO:0006839	15	1	0.133784629	mitochondrial transport
GO:0006801	15	1	0.133784629	superoxide metabolism
GO:0045165	15	1	0.133784629	cell fate commitment
GO:0045216	15	1	0.133784629	intercellular junction assembly and/or maintenance

GO:0042129	15	1	0.133784629	regulation of T cell proliferation
GO:0009218	15	1	0.133784629	pyrimidine ribonucleotide metabolism
GO:0009583	15	1	0.133784629	detection of light
GO:0030111	15	1	0.133784629	regulation of Wnt receptor signaling pathway
GO:0006879	15	1	0.133784629	iron ion homeostasis
GO:0043123	68	2	0.136724222	positive regulation of I-kappaB kinase/NF-kappaB cascade
GO:0006874	68	2	0.136724222	calcium ion homeostasis
GO:0007626	209	4	0.138049639	locomotory behavior
GO:0045637	16	1	0.142046193	regulation of myeloid cell differentiation
GO:0007157	16	1	0.142046193	heterophilic cell adhesion
GO:0007173	16	1	0.142046193	epidermal growth factor receptor signaling pathway
GO:0048015	71	2	0.146526003	phosphoinositide-mediated signaling
GO:0006904	17	1	0.150229869	vesicle docking during exocytosis
GO:0042098	17	1	0.150229869	T cell proliferation
GO:0007416	17	1	0.150229869	synaptogenesis
GO:0007215	17	1	0.150229869	glutamate signaling pathway
GO:0007611	17	1	0.150229869	learning and/or memory
GO:0043122	73	2	0.153139978	regulation of I-kappaB kinase/NF-kappaB cascade
GO:0043009	18	1	0.158336384	embryonic development (sensu Vertebrata)
GO:0048278	18	1	0.158336384	vesicle docking
GO:0007005	18	1	0.158336384	mitochondrion organization and biogenesis
GO:0009416	18	1	0.158336384	response to light
GO:0050808	18	1	0.158336384	synapse organization and biogenesis
GO:0050671	18	1	0.158336384	positive regulation of lymphocyte proliferation
GO:0030041	18	1	0.158336384	actin filament polymerization
GO:0006221	18	1	0.158336384	pyrimidine nucleotide biosynthesis
GO:0001701	18	1	0.158336384	embryonic development (sensu Mammalia)
GO:0007001	221	4	0.159206552	chromosome organization and biogenesis (sensu Eukaryota)
GO:0015672	223	4	0.162845955	monovalent inorganic cation transport
GO:0008104	474	7	0.164460792	protein localization
GO:0007269	19	1	0.166366456	neurotransmitter secretion
GO:0050776	77	2	0.166537773	regulation of immune response
GO:0048518	565	8	0.168596128	positive regulation of biological process
GO:0046916	20	1	0.174320798	transition metal ion homeostasis
GO:0007205	20	1	0.174320798	protein kinase C activation
GO:0051241	20	1	0.174320798	negative regulation of organismal physiological process
GO:0048522	482	7	0.174458103	positive regulation of cellular process
GO:0016043	842	11	0.174922358	cell organization and biogenesis
GO:0006814	80	2	0.176717363	sodium ion transport
GO:0050870	21	1	0.182200116	positive regulation of T cell activation
GO:0007163	21	1	0.182200116	establishment and/or maintenance of cell polarity
GO:0051276	236	4	0.187219499	chromosome organization and biogenesis
GO:0016044	22	1	0.190005109	membrane organization and biogenesis
GO:0050670	22	1	0.190005109	regulation of lymphocyte proliferation
GO:0016197	22	1	0.190005109	endosome transport
GO:0045055	22	1	0.190005109	regulated secretory pathway

GO:0016477	85	2	0.193891717	cell migration
GO:0009967	85	2	0.193891717	positive regulation of signal transduction
GO:0008277	23	1	0.19773647	regulation of G-protein coupled receptor protein signaling pathway
GO:0045045	161	3	0.197805749	secretory pathway
GO:0006968	87	2	0.200822942	cellular defense response
GO:0016055	87	2	0.200822942	Wnt receptor signaling pathway
GO:0042088	24	1	0.205394885	T-helper 1 type immune response
GO:0007032	24	1	0.205394885	endosome organization and biogenesis
GO:0007389	24	1	0.205394885	pattern specification
GO:0007242	879	11	0.212219204	intracellular signaling cascade
GO:0006996	693	9	0.212411907	organelle organization and biogenesis
GO:0045792	25	1	0.212981035	negative regulation of cell size
GO:0042087	25	1	0.212981035	cell-mediated immune response
GO:0030308	25	1	0.212981035	negative regulation of cell growth
GO:0050793	168	3	0.215040152	regulation of development
GO:0009306	26	1	0.220495594	protein secretion
GO:0009410	26	1	0.220495594	response to xenobiotic stimulus
GO:0007507	26	1	0.220495594	heart development
GO:0006220	26	1	0.220495594	pyrimidine nucleotide metabolism
GO:0043119	428	6	0.2223441	positive regulation of physiological process
GO:0016066	27	1	0.227939229	cellular defense response (sensu Vertebrata)
GO:0009792	27	1	0.227939229	embryonic development (sensu Metazoa)
GO:0016126	27	1	0.227939229	sterol biosynthesis
GO:0046651	28	1	0.235312602	lymphocyte proliferation
GO:0051251	28	1	0.235312602	positive regulation of lymphocyte activation
GO:0045321	97	2	0.235852848	immune cell activation
GO:0001775	98	2	0.23938017	cell activation
GO:0050867	29	1	0.242616369	positive regulation of cell activation
GO:0045926	29	1	0.242616369	negative regulation of growth
GO:0015674	99	2	0.24291035	di-, tri-valent inorganic cation transport
GO:0007249	99	2	0.24291035	I-kappaB kinase/NF-kappaB cascade
GO:0045941	100	2	0.246443017	positive regulation of transcription
GO:0006956	30	1	0.249851179	complement activation
GO:0045944	30	1	0.249851179	positive regulation of transcription from RNA polymerase II promoter
GO:0006730	30	1	0.249851179	one-carbon compound metabolism
GO:0006935	101	2	0.249977811	chemotaxis
GO:0042330	101	2	0.249977811	taxis
GO:0042221	183	3	0.252953924	response to chemical substance
GO:0046907	448	6	0.253856796	intracellular transport
GO:0000041	31	1	0.257017675	transition metal ion transport
GO:0050863	31	1	0.257017675	regulation of T cell activation
GO:0006367	31	1	0.257017675	transcription initiation from RNA polymerase II promoter
GO:0048523	640	8	0.263567991	negative regulation of cellular process
GO:0045935	105	2	0.264131209	positive regulation of nucleobase, nucleoside, nucleotide and nucleic acid metabolism
GO:0015698	106	2	0.267671414	inorganic anion transport
GO:0042035	33	1	0.271148271	regulation of cytokine biosynthesis

GO:0009314	33	1	0.271148271	response to radiation
GO:0006338	33	1	0.271148271	chromatin remodeling
GO:0045184	459	6	0.271717293	establishment of protein localization
GO:0007420	34	1	0.278113628	brain development
GO:0006112	34	1	0.278113628	energy reserve metabolism
GO:0043062	35	1	0.285013187	extracellular structure organization and biogenesis
GO:0030198	35	1	0.285013187	extracellular matrix organization and biogenesis
GO:0006916	112	2	0.288903432	anti-apoptosis
GO:0007017	112	2	0.288903432	microtubule-based process
GO:0042089	36	1	0.291847561	cytokine biosynthesis
GO:0001501	113	2	0.292437727	skeletal development
GO:0008283	473	6	0.29489471	cell proliferation
GO:0016485	37	1	0.29861736	protein processing
GO:0042107	37	1	0.29861736	cytokine metabolism
GO:0030099	37	1	0.29861736	myeloid cell differentiation
GO:0046034	37	1	0.29861736	ATP metabolism
GO:0006821	38	1	0.305323186	chloride transport
GO:0009987	8200	80	0.310240311	cellular process
GO:0001817	39	1	0.311965637	regulation of cytokine production
GO:0007519	39	1	0.311965637	myogenesis
GO:0001505	41	1	0.325062777	regulation of neurotransmitter levels
GO:0051249	41	1	0.325062777	regulation of lymphocyte activation
GO:0008361	123	2	0.327624865	regulation of cell size
GO:0016049	123	2	0.327624865	cell growth
GO:0007010	305	4	0.330760452	cytoskeleton organization and biogenesis
GO:0006631	124	2	0.331122373	fatty acid metabolism
GO:0001816	42	1	0.331518633	cytokine production
GO:0048519	688	8	0.331574609	negative regulation of biological process
GO:0050865	43	1	0.337913449	regulation of cell activation
GO:0006820	128	2	0.34506205	anion transport
GO:0043066	128	2	0.34506205	negative regulation of apoptosis
GO:0009581	313	4	0.348189873	detection of external stimulus
GO:0043069	129	2	0.348533296	negative regulation of programmed cell death
GO:0008154	45	1	0.350522238	actin polymerization and/or depolymerization
GO:0042110	45	1	0.350522238	T cell activation
GO:0007229	45	1	0.350522238	integrin-mediated signaling pathway
GO:0051242	411	5	0.353691237	positive regulation of cellular physiological process
GO:0042445	46	1	0.356737337	hormone metabolism
GO:0006352	47	1	0.362893645	transcription initiation
GO:0007264	227	3	0.367644784	small GTPase mediated signal transduction
GO:0031325	136	2	0.372656157	positive regulation of cellular metabolism
GO:0051093	49	1	0.375032087	negative regulation of development
GO:0009199	49	1	0.375032087	ribonucleoside triphosphate metabolism
GO:0009144	49	1	0.375032087	purine nucleoside triphosphate metabolism
GO:0007160	49	1	0.375032087	cell-matrix adhesion
GO:0009205	49	1	0.375032087	purine ribonucleoside triphosphate metabolism

GO:0009141	50	1	0.381015304	nucleoside triphosphate metabolism
GO:0008285	139	2	0.382890029	negative regulation of cell proliferation
GO:0006917	140	2	0.386286104	induction of apoptosis
GO:0012502	140	2	0.386286104	induction of programmed cell death
GO:0008632	53	1	0.398627341	apoptotic program
GO:0007243	242	3	0.406532172	protein kinase cascade
GO:0015031	439	5	0.407257405	protein transport
GO:0045595	55	1	0.410092588	regulation of cell differentiation
GO:0050778	56	1	0.415743923	positive regulation of immune response
GO:0009893	149	2	0.41647798	positive regulation of metabolism
GO:0006817	57	1	0.421341742	phosphate transport
GO:0015992	57	1	0.421341742	proton transport
GO:0006818	58	1	0.426886545	hydrogen transport
GO:0009152	58	1	0.426886545	purine ribonucleotide biosynthesis
GO:0040007	154	2	0.432936734	growth
GO:0043065	155	2	0.436199621	positive regulation of apoptosis
GO:0006694	60	1	0.437819086	steroid biosynthesis
GO:0009150	60	1	0.437819086	purine ribonucleotide metabolism
GO:0043068	156	2	0.439452619	positive regulation of programmed cell death
GO:0006118	256	3	0.442215932	electron transport
GO:0048609	61	1	0.443207802	reproductive organismal physiological process
GO:0006164	61	1	0.443207802	purine nucleotide biosynthesis
GO:0009888	158	2	0.445928519	histogenesis
GO:0050876	62	1	0.44854546	reproductive physiological process
GO:0006163	63	1	0.453832538	purine nucleotide metabolism
GO:0001525	65	1	0.464256845	angiogenesis
GO:0051056	65	1	0.464256845	regulation of small GTPase mediated signal transduction
GO:0009260	65	1	0.464256845	ribonucleotide biosynthesis
GO:0009790	65	1	0.464256845	embryonic development
GO:0042127	265	3	0.464736282	regulation of cell proliferation
GO:0016125	66	1	0.469395008	sterol metabolism
GO:0006816	68	1	0.479525658	calcium ion transport
GO:0001568	68	1	0.479525658	blood vessel development
GO:0048514	68	1	0.479525658	blood vessel morphogenesis
GO:0009259	68	1	0.479525658	ribonucleotide metabolism
GO:0051243	583	6	0.48336647	negative regulation of cellular physiological process
GO:0001944	69	1	0.484519053	vasculature development
GO:0016337	172	2	0.49007573	cell-cell adhesion
GO:0019932	176	2	0.502289015	second-messenger-mediated signaling
GO:0043118	602	6	0.515159778	negative regulation of physiological process
GO:0045893	77	1	0.522798221	positive regulation of transcription, DNA-dependent
GO:0016265	501	5	0.523006171	death
GO:0000902	183	2	0.523213207	cellular morphogenesis
GO:0006954	185	2	0.529084582	inflammatory response
GO:0046649	81	1	0.540870191	lymphocyte activation
GO:0000165	81	1	0.540870191	MAPKKK cascade

GO:0006412	407	4	0.546689475	protein biosynthesis
GO:0044267	2109	20	0.550395141	cellular protein metabolism
GO:0016310	522	5	0.560191974	phosphorylation
GO:0042981	306	3	0.56180428	regulation of apoptosis
GO:0050954	86	1	0.562511445	sensory perception of mechanical stimulus
GO:0050982	86	1	0.562511445	detection of mechanical stimulus
GO:0007605	86	1	0.562511445	perception of sound
GO:0043067	308	3	0.566271689	regulation of programmed cell death
GO:0030097	87	1	0.566717125	hemopoiesis
GO:0019538	2129	20	0.569596	protein metabolism
GO:0048534	88	1	0.570882841	hemopoietic or lymphoid organ development
GO:0009605	963	9	0.575730714	response to external stimulus
GO:0006793	641	6	0.578029338	phosphorus metabolism
GO:0006796	641	6	0.578029338	phosphate metabolism
GO:0006486	92	1	0.587153513	protein amino acid glycosylation
GO:0043413	93	1	0.591124967	biopolymer glycosylation
GO:0001558	94	1	0.595058657	regulation of cell growth
GO:0007517	94	1	0.595058657	muscle development
GO:0007028	96	1	0.60281416	cytoplasm organization and biogenesis
GO:0009101	96	1	0.60281416	glycoprotein biosynthesis
GO:0006470	99	1	0.614172951	protein amino acid dephosphorylation
GO:0006468	444	4	0.616449531	protein amino acid phosphorylation
GO:0006091	446	4	0.620031635	generation of precursor metabolites and energy
GO:0009165	103	1	0.628819965	nucleotide biosynthesis
GO:0040008	104	1	0.632395	regulation of growth
GO:0009611	341	3	0.635927227	response to wounding
GO:0007156	105	1	0.635935998	homophilic cell adhesion
GO:0009613	458	4	0.641093305	response to pest, pathogen or parasite
GO:0006417	107	1	0.642917162	regulation of protein biosynthesis
GO:0043207	463	4	0.649647597	response to external biotic stimulus
GO:0009059	464	4	0.65134263	macromolecule biosynthesis
GO:0016311	110	1	0.653141546	dephosphorylation
GO:0031326	111	1	0.656484947	regulation of cellular biosynthesis
GO:0009100	111	1	0.656484947	glycoprotein metabolism
GO:0006915	471	4	0.663059057	apoptosis
GO:0012501	473	4	0.666358539	programmed cell death
GO:0007169	116	1	0.672730069	transmembrane receptor protein tyrosine kinase signaling pathway
GO:0009889	116	1	0.672730069	regulation of biosynthesis
GO:0000398	119	1	0.682110186	nuclear mRNA splicing, via spliceosome
GO:0000375	119	1	0.682110186	RNA splicing, via transesterification reactions
GO:0000377	119	1	0.682110186	RNA splicing, via transesterification reactions with bulged adenosine as nucleophile
GO:0050789	2689	24	0.686593179	regulation of biological process
GO:0008284	121	1	0.688215459	positive regulation of cell proliferation
GO:0043037	122	1	0.69122449	translation
GO:0016568	122	1	0.69122449	chromatin modification
GO:0006259	489	4	0.691977829	DNA metabolism

GO:0030036	126	1	0.702976249	actin cytoskeleton organization and biogenesis
GO:0008219	498	4	0.705777938	cell death
GO:0006886	257	2	0.707535899	intracellular protein transport
GO:0043170	2405	21	0.717498913	macromolecule metabolism
GO:0044260	2298	20	0.718621185	cellular macromolecule metabolism
GO:0050874	1531	13	0.718780316	organismal physiological process
GO:0006936	132	1	0.719779819	muscle contraction
GO:0015980	132	1	0.719779819	energy derivation by oxidation of organic compounds
GO:0008202	132	1	0.719779819	steroid metabolism
GO:0006464	1087	9	0.722179552	protein modification
GO:0008380	137	1	0.733062343	RNA splicing
GO:0030029	139	1	0.738199373	actin filament-based process
GO:0006959	141	1	0.743238672	humoral immune response
GO:0006950	881	7	0.746495962	response to stress
GO:0009117	143	1	0.748182077	nucleotide metabolism
GO:0051186	143	1	0.748182077	cofactor metabolism
GO:0050953	149	1	0.762454806	sensory perception of light
GO:0007601	149	1	0.762454806	visual perception
GO:0007600	287	2	0.763628672	sensory perception
GO:0016481	151	1	0.767032344	negative regulation of transcription
GO:0050794	2488	21	0.778223678	regulation of cellular process
GO:0045934	159	1	0.784486727	negative regulation of nucleobase, nucleoside, nucleotide and nucleic acid metabolism
GO:0043412	1160	9	0.791540418	biopolymer modification
GO:0007167	165	1	0.796723252	enzyme linked receptor protein signaling pathway
GO:0051246	174	1	0.813801644	regulation of protein metabolism
GO:0008610	175	1	0.815609395	lipid biosynthesis
GO:0006397	180	1	0.824391185	mRNA processing
GO:0031324	183	1	0.829460474	negative regulation of cellular metabolism
GO:0009058	859	6	0.839310213	biosynthesis
GO:0006355	1359	10	0.852614304	regulation of transcription, DNA-dependent
GO:0050791	2407	19	0.861482002	regulation of physiological process
GO:0019752	359	2	0.861926258	carboxylic acid metabolism
GO:0016071	205	1	0.862468186	mRNA metabolism
GO:0044249	763	5	0.862581695	cellular biosynthesis
GO:0006082	361	2	0.864036331	organic acid metabolism
GO:0000003	208	1	0.866449444	reproduction
GO:0006066	213	1	0.872833254	alcohol metabolism
GO:0009892	217	1	0.87772234	negative regulation of metabolism
GO:0006351	1407	10	0.881031017	transcription, DNA-dependent
GO:0044255	383	2	0.885393688	cellular lipid metabolism
GO:0045449	1433	10	0.89451547	regulation of transcription
GO:0006955	684	4	0.899368145	immune response
GO:0031323	1566	11	0.900669358	regulation of cellular metabolism
GO:0006357	240	1	0.902434973	regulation of transcription from RNA polymerase II promoter
GO:0050896	1578	11	0.906037518	response to stimulus
GO:0019219	1458	10	0.906299138	regulation of nucleobase, nucleoside, nucleotide and nucleic acid metabolism

GO:0006366	426	2	0.918534109	transcription from RNA polymerase II promoter
GO:0006350	1499	10	0.923290711	transcription
GO:0019222	1629	11	0.926294095	regulation of metabolism
GO:0006952	751	4	0.934953834	defense response
GO:0000004	465	2	0.940673702	biological process unknown
GO:0006629	469	2	0.942593923	lipid metabolism
GO:0050875	7018	61	0.946572954	cellular physiological process
GO:0051244	2319	16	0.95277652	regulation of cellular physiological process
GO:0006139	2328	16	0.954763651	nucleobase, nucleoside, nucleotide and nucleic acid metabolism
GO:0006396	326	1	0.958278148	RNA processing
GO:0005975	355	1	0.968729807	carbohydrate metabolism
GO:0009607	857	4	0.968798903	response to biotic stimulus
GO:0016070	391	1	0.978168558	RNA metabolism
GO:0043283	2240	14	0.97927826	biopolymer metabolism
GO:0044237	5026	38	0.987805835	cellular metabolism
GO:0044238	4820	36	0.987984216	primary metabolism
GO:0008152	5262	40	0.989654352	metabolism
GO:0007582	7846	66	0.996881378	physiological process
GO:0007049	601	1	0.997396406	cell cycle
GO:0044248	700	1	0.999062102	cellular catabolism
GO:0009056	756	1	0.999476421	catabolism
GO:0008150	9032	86	1	biological_process

1.3 GO: Molecular Function

GO ID	genes present in chip	genes present in tissues	p-value	GO term
GO:0008289	298	8	0.0089807582132362	lipid binding
GO:0015276	84	4	0.00935549926191315	ligand-gated ion channel activity
GO:0048154	1	1	0.0098522167487679	S100 beta binding
GO:0017162	1	1	0.0098522167487679	aryl hydrocarbon receptor binding
GO:0000254	1	1	0.0098522167487679	C-4 methylsterol oxidase activity
GO:0005200	86	4	0.0101447051649378	structural constituent of cytoskeleton
GO:0004890	21	2	0.017857290330387	GABA-A receptor activity
GO:0015268	274	7	0.0182867249521519	alpha-type channel activity
GO:0005230	57	3	0.0184698876531964	extracellular ligand-gated ion channel activity
GO:0048155	2	1	0.0196083898751431	S100 alpha binding
GO:0048156	2	1	0.0196083898751431	tau protein binding
GO:0005218	2	1	0.0196083898751431	intracellular ligand-gated calcium channel activity
GO:0005219	2	1	0.0196083898751431	ryanodine-sensitive calcium-release channel activity
GO:0046332	2	1	0.0196083898751431	SMAD binding
GO:0005061	2	1	0.0196083898751431	aryl hydrocarbon receptor nuclear translocator activity
GO:0042802	158	5	0.0197221499864964	protein self binding
GO:0016917	23	2	0.021241997681534	GABA receptor activity
GO:0015267	284	7	0.0217849464281203	channel or pore class transporter activity
GO:0005525	226	6	0.0239282446596095	GTP binding
GO:0042803	64	3	0.0250408201994555	protein homodimerization activity
GO:0005178	26	2	0.0267726901829596	integrin binding
GO:0019001	235	6	0.0282841596303746	guanyl nucleotide binding
GO:0004566	3	1	0.0292694456830614	beta-glucuronidase activity
GO:0005176	3	1	0.0292694456830614	ErbB-2 class receptor binding
GO:0004971	3	1	0.0292694456830614	alpha-amino-3-hydroxy-5-methyl-4-isoxazole propionate selective glutamate receptor activity
GO:0016527	3	1	0.0292694456830614	brain-specific angiogenesis inhibitor activity
GO:0003924	121	4	0.0313458759650223	GTPase activity
GO:0015075	523	10	0.0326449453342393	ion transporter activity
GO:0051082	127	4	0.036490775401127	unfolded protein binding
GO:0031072	31	2	0.0371131166114117	heat shock protein binding
GO:0005216	252	6	0.0378818780348551	ion channel activity
GO:0005332	4	1	0.0388363016387961	gamma-aminobutyric acid:sodium symporter activity
GO:0005499	4	1	0.0388363016387961	vitamin D binding
GO:0016299	4	1	0.0388363016387961	regulator of G-protein signaling activity
GO:0015185	4	1	0.0388363016387961	L-gamma-aminobutyric acid transporter activity
GO:0004017	5	1	0.0483098664543514	adenylate kinase activity
GO:0005041	5	1	0.0483098664543514	low-density lipoprotein receptor activity
GO:0004871	1837	25	0.0502112277939993	signal transducer activity
GO:0005198	488	9	0.0505830732721238	structural molecule activity
GO:0008199	6	1	0.0576910401700904	ferric iron binding
GO:0030228	6	1	0.0576910401700904	lipoprotein receptor activity
GO:0015278	6	1	0.0576910401700904	calcium-release channel activity
GO:0046870	6	1	0.0576910401700904	cadmium ion binding

GO:0005179	90	3	0.0589315273465866	hormone activity
GO:0005254	43	2	0.0668376037783828	chloride channel activity
GO:0005416	7	1	0.0669807142365835	cation:amino acid symporter activity
GO:0005283	7	1	0.0669807142365835	sodium:amino acid symporter activity
GO:0015277	7	1	0.0669807142365835	kainate selective glutamate receptor activity
GO:0005515	3709	44	0.0704866159131691	protein binding
GO:0003774	98	3	0.0722018807749619	motor activity
GO:0031404	45	2	0.0723672423033529	chloride ion binding
GO:0043168	45	2	0.0723672423033529	anion binding
GO:0005253	46	2	0.075186061703723	anion channel activity
GO:0005102	528	9	0.0752462773545085	receptor binding
GO:0030169	8	1	0.0761797715956947	low-density lipoprotein binding
GO:0005507	49	2	0.0838470993065583	copper ion binding
GO:0005391	9	1	0.0852890867609098	sodium:potassium-exchanging ATPase activity
GO:0008121	9	1	0.0852890867609098	ubiquinol-cytochrome-c reductase activity
GO:0005217	9	1	0.0852890867609098	intracellular ligand-gated ion channel activity
GO:0005545	9	1	0.0852890867609098	phosphatidylinositol binding
GO:0016681	9	1	0.0852890867609098	oxidoreductase activity, acting on diphenols and related substances as donors, cytochrome as acceptor
GO:0016679	9	1	0.0852890867609098	oxidoreductase activity, acting on diphenols and related substances as donors
GO:0030552	9	1	0.0852890867609098	3',5'-cAMP binding
GO:0046983	170	4	0.086741460432521	protein dimerization activity
GO:0004685	10	1	0.0943095258969232	calcium- and calmodulin-dependent protein kinase activity
GO:0003746	10	1	0.0943095258969232	translation elongation factor activity
GO:0008603	10	1	0.0943095258969232	cAMP-dependent protein kinase regulator activity
GO:0016208	10	1	0.0943095258969232	AMP binding
GO:0005125	181	4	0.103199911672724	cytokine activity
GO:0019206	11	1	0.103241946898464	nucleoside kinase activity
GO:0019207	58	2	0.111461504903608	kinase regulator activity
GO:0005243	12	1	0.112087199468407	gap-junction forming channel activity
GO:0004143	12	1	0.112087199468407	diacylglycerol kinase activity
GO:0015285	12	1	0.112087199468407	connexon channel activity
GO:0005086	12	1	0.112087199468407	ARF guanyl-nucleotide exchange factor activity
GO:0005261	188	4	0.114370943740248	cation channel activity
GO:0005251	13	1	0.120846125195132	delayed rectifier potassium channel activity
GO:0005248	13	1	0.120846125195132	voltage-gated sodium channel activity
GO:0015926	13	1	0.120846125195132	glucosidase activity
GO:0016526	13	1	0.120846125195132	G-protein coupled receptor activity, unknown ligand
GO:0008324	421	7	0.120876815831682	cation transporter activity
GO:0046982	61	2	0.121134334634737	protein heterodimerization activity
GO:0008373	14	1	0.129519557629186	sialyltransferase activity
GO:0004970	14	1	0.129519557629186	ionotropic glutamate receptor activity
GO:0005234	14	1	0.129519557629186	glutamate-gated ion channel activity
GO:0005057	132	3	0.141051428901925	receptor signaling protein activity
GO:0005328	16	1	0.146613237087128	neurotransmitter:sodium symporter activity
GO:0019201	16	1	0.146613237087128	nucleotide kinase activity
GO:0004089	16	1	0.146613237087128	carbonate dehydratase activity
GO:0031402	70	2	0.151268304221225	sodium ion binding

GO:0004683	17	1	0.155035111702751	calmodulin regulated protein kinase activity
GO:0017076	1248	16	0.161562248411331	purine nucleotide binding
GO:0008034	18	1	0.163374748357512	lipoprotein binding
GO:0005326	18	1	0.163374748357512	neurotransmitter transporter activity
GO:0030551	18	1	0.163374748357512	cyclic nucleotide binding
GO:0008083	144	3	0.169231821740537	growth factor activity
GO:0005083	144	3	0.169231821740537	small GTPase regulator activity
GO:0030695	219	4	0.169633555444275	GTPase regulator activity
GO:0005001	19	1	0.171632941537642	transmembrane receptor protein tyrosine phosphatase activity
GO:0005343	19	1	0.171632941537642	organic acid:sodium symporter activity
GO:0019198	19	1	0.171632941537642	transmembrane receptor protein phosphatase activity
GO:0031420	146	3	0.174081550414652	alkali metal ion binding
GO:0030594	77	2	0.175621542872258	neurotransmitter receptor activity
GO:0042165	78	2	0.179151829313982	neurotransmitter binding
GO:0001633	20	1	0.179810478136620	secretin-like receptor activity
GO:0015179	20	1	0.179810478136620	L-amino acid transporter activity
GO:0005509	646	9	0.184881923164567	calcium ion binding
GO:0005184	21	1	0.187908137526932	neuropeptide hormone activity
GO:0016709	21	1	0.187908137526932	oxidoreductase activity, acting on paired donors, with incorporation or reduction of molecular oxygen, NAD or NADH as one donor, and incorporation of one atom of oxygen
GO:0030955	86	2	0.207747772823657	potassium ion binding
GO:0005215	1030	13	0.211028681079155	transporter activity
GO:0005478	24	1	0.211729534844464	intracellular transporter activity
GO:0005272	24	1	0.211729534844464	sodium channel activity
GO:0005267	90	2	0.222228130946466	potassium channel activity
GO:0005543	169	3	0.232312472816472	phospholipid binding
GO:0005085	93	2	0.233143106628067	guanyl-nucleotide exchange factor activity
GO:0030234	509	7	0.235159138406313	enzyme regulator activity
GO:0000166	1432	17	0.238431259714496	nucleotide binding
GO:0008509	95	2	0.240439177380682	anion transporter activity
GO:0005279	28	1	0.242419111842415	amino acid-polyamine transporter activity
GO:0016776	28	1	0.242419111842415	phosphotransferase activity, phosphate group as acceptor
GO:0015203	28	1	0.242419111842415	polyamine transporter activity
GO:0019210	29	1	0.249904931613972	kinase inhibitor activity
GO:0008066	29	1	0.249904931613972	glutamate receptor activity
GO:0016209	31	1	0.264657709766415	antioxidant activity
GO:0003779	189	3	0.285452700029423	actin binding
GO:0019205	34	1	0.286250241769121	nucleobase, nucleoside, nucleotide kinase activity
GO:0015370	35	1	0.29330740778087	solute:sodium symporter activity
GO:0005488	7228	74	0.295025590022248	binding
GO:0008092	279	4	0.295522845006671	cytoskeletal protein binding
GO:0019208	36	1	0.300295531457348	phosphatase regulator activity
GO:0005231	39	1	0.32085229923056	excitatory extracellular ligand-gated ion channel activity
GO:0015294	39	1	0.32085229923056	solute:cation symporter activity
GO:0004721	118	2	0.32443620959422	phosphoprotein phosphatase activity
GO:0015662	40	1	0.327570872570101	ATPase activity, coupled to transmembrane movement of ions, phosphorylative mechanism
GO:0015077	121	2	0.335285126323835	monovalent inorganic cation transporter activity

GO:0005496	42	1	0.34081136300222	steroid binding
GO:0019992	42	1	0.34081136300222	diacylglycerol binding
GO:0005244	124	2	0.346084852328939	voltage-gated ion channel activity
GO:0016836	43	1	0.347334547745645	hydro-lyase activity
GO:0005506	212	3	0.347656385680236	iron ion binding
GO:0003777	45	1	0.360189920001351	microtubule motor activity
GO:0005262	47	1	0.372794724659359	calcium channel activity
GO:0019887	47	1	0.372794724659359	protein kinase regulator activity
GO:0035091	132	2	0.374596400186187	phosphoinositide binding
GO:0017111	410	5	0.378996571488822	nucleoside-triphosphatase activity
GO:0015171	48	1	0.379004677880554	amino acid transporter activity
GO:0016835	50	1	0.39124266297511	carbon-oxygen lyase activity
GO:0015399	140	2	0.402606930866815	primary active transporter activity
GO:0005554	522	6	0.409835628458921	molecular function unknown
GO:0016462	430	5	0.418478718285889	pyrophosphatase activity
GO:0016818	432	5	0.422412627962691	hydrolase activity, acting on acid anhydrides, in phosphorus-containing anhydrides
GO:0043169	2313	24	0.423934101735005	cation binding
GO:0016817	433	5	0.42437808266494	hydrolase activity, acting on acid anhydrides
GO:0005275	58	1	0.437853626940094	amine transporter activity
GO:0004553	59	1	0.443425888784197	hydrolase activity, hydrolyzing O-glycosyl compounds
GO:0019842	59	1	0.443425888784197	vitamin binding
GO:0016564	59	1	0.443425888784197	transcriptional repressor activity
GO:0008135	62	1	0.45981687217238	translation factor activity, nucleic acid binding
GO:0043167	2556	26	0.46333333939504	ion binding
GO:0005249	63	1	0.465173683441058	voltage-gated potassium channel activity
GO:0004713	161	2	0.473145253184821	protein-tyrosine kinase activity
GO:0005342	66	1	0.480930780863612	organic acid transporter activity
GO:0046943	66	1	0.480930780863612	carboxylic acid transporter activity
GO:0042625	68	1	0.491179440429838	ATPase activity, coupled to transmembrane movement of ions
GO:0016791	171	2	0.504974304917604	phosphoric monoester hydrolase activity
GO:0004497	71	1	0.506178188564217	monooxygenase activity
GO:0045182	72	1	0.511079904538342	translation regulator activity
GO:0005201	73	1	0.515933478196954	extracellular matrix structural constituent
GO:0004725	75	1	0.525498065255236	protein tyrosine phosphatase activity
GO:0004888	694	7	0.531225948964286	transmembrane receptor activity
GO:0016798	77	1	0.534875638404234	hydrolase activity, acting on glycosyl bonds
GO:0015405	77	1	0.534875638404234	P-P-bond-hydrolysis-driven transporter activity
GO:0004857	187	2	0.553284923996199	enzyme inhibitor activity
GO:0005386	398	4	0.555782938945936	carrier activity
GO:0008047	188	2	0.556193760571508	enzyme activator activity
GO:0016301	609	6	0.560407418071087	kinase activity
GO:0015293	83	1	0.561922110229204	symporter activity
GO:0030554	1028	10	0.566658494620549	adenyl nucleotide binding
GO:0016705	87	1	0.579082531807466	oxidoreductase activity, acting on paired donors, with incorporation or reduction of molecular oxygen
GO:0048503	92	1	0.599600442865038	GPI anchor binding
GO:0030145	92	1	0.599600442865038	manganese ion binding
GO:0016773	532	5	0.607743194841043	phosphotransferase activity, alcohol group as acceptor

GO:0008415	98	1	0.622919290663365	acyltransferase activity
GO:0016747	98	1	0.622919290663365	transferase activity, transferring groups other than amino-acyl groups
GO:0003700	649	6	0.623729029872176	transcription factor activity
GO:0005524	970	9	0.626322490363551	ATP binding
GO:0004674	326	3	0.628168903023433	protein serine/threonine kinase activity
GO:0003735	100	1	0.630389591226368	structural constituent of ribosome
GO:0042626	100	1	0.630389591226368	ATPase activity, coupled to transmembrane movement of substances
GO:0016820	101	1	0.634069644020005	hydrolase activity, acting on acid anhydrides, catalyzing transmembrane movement of substances
GO:0009055	104	1	0.644893683920201	electron carrier activity
GO:0016746	104	1	0.644893683920201	transferase activity, transferring acyl groups
GO:0004672	447	4	0.648305613216222	protein kinase activity
GO:0042578	228	2	0.661607344743941	phosphoric ester hydrolase activity
GO:0005516	112	1	0.672233490581306	calmodulin binding
GO:0015078	112	1	0.672233490581306	hydrogen ion transporter activity
GO:0046872	2495	23	0.683114275052388	metal ion binding
GO:0005096	117	1	0.688252220116342	GTPase activator activity
GO:0016772	708	6	0.707192510732501	transferase activity, transferring phosphorus-containing groups
GO:0016829	127	1	0.718001937210989	lyase activity
GO:0004872	1060	9	0.730827742216296	receptor activity
GO:0030528	963	8	0.745114297717765	transcription regulator activity
GO:0003713	140	1	0.752513143693623	transcription coactivator activity
GO:0015290	149	1	0.773923051291028	electrochemical potential-driven transporter activity
GO:0015291	149	1	0.773923051291028	porter activity
GO:0005489	152	1	0.780644133307969	electron transporter activity
GO:0008134	293	2	0.789479011802132	transcription factor binding
GO:0016757	165	1	0.807556409908069	transferase activity, transferring glycosyl groups
GO:0046914	1596	13	0.813237013032108	transition metal ion binding
GO:0008270	1287	10	0.832420571636956	zinc ion binding
GO:0003702	197	1	0.86067240158433	RNA polymerase II transcription factor activity
GO:0004930	349	2	0.863735537760016	G-protein coupled receptor activity
GO:0016491	509	3	0.88459377334242	oxidoreductase activity
GO:0042623	229	1	0.899240212157682	ATPase activity, coupled
GO:0003712	230	1	0.900257332009183	transcription cofactor activity
GO:0016740	1169	8	0.90345460379231	transferase activity
GO:0000287	251	1	0.919417745173377	magnesium ion binding
GO:0016887	255	1	0.922630544213051	ATPase activity
GO:0016788	446	2	0.938566401371644	hydrolase activity, acting on ester bonds
GO:0001584	282	1	0.94123788362873	rhodopsin-like receptor activity
GO:0003677	1384	9	0.941545777860423	DNA binding
GO:0016787	1424	8	0.978065851452689	hydrolase activity
GO:0003723	436	1	0.987952486826812	RNA binding
GO:0003676	1990	11	0.993161462567389	nucleic acid binding
GO:0003824	3409	20	0.999229216252132	catalytic activity
GO:0003674	9541	94	1	molecular_function

1.4 GO: Cellular Component

GO ID	genes present in chip	genes present in tissues	p-value	GO term
GO:0043005	17	3	0.000669404224927815	neuron projection
GO:0005883	5	2	0.00105215990373759	neurofilament
GO:0005678	6	2	0.00156754197615803	chromatin assembly complex
GO:0030424	7	2	0.00217969478042467	axon
GO:0045285	1	1	0.0104202848211198	ubiquinol-cytochrome-c reductase complex
GO:0045202	98	4	0.0189498723463095	synapse
GO:0005834	21	2	0.0198297773262040	heterotrimeric G-protein complex
GO:0030056	2	1	0.0207331813433624	hemidesmosome
GO:0016585	23	2	0.0235715593433386	chromatin remodeling complex
GO:0009986	61	3	0.0255067012936467	cell surface
GO:0005886	1472	23	0.0259928388637474	plasma membrane
GO:0019897	26	2	0.0296771927915484	extrinsic to plasma membrane
GO:0045111	65	3	0.0300558224546633	intermediate filament cytoskeleton
GO:0005882	65	3	0.0300558224546633	intermediate filament
GO:0005588	3	1	0.0309397839697780	collagen type V
GO:0045211	66	3	0.0312557094720687	postsynaptic membrane
GO:0042995	70	3	0.0363039249194101	cell projection
GO:0031307	4	1	0.0410411760757222	integral to mitochondrial outer membrane
GO:0045275	4	1	0.0410411760757222	respiratory chain complex III
GO:0031301	76	3	0.0446129249108139	integral to organelle membrane
GO:0005874	132	4	0.0486989631944924	microtubule
GO:0031306	5	1	0.0510384301187184	intrinsic to mitochondrial outer membrane
GO:0019898	37	2	0.0565189626822395	extrinsic to membrane
GO:0005905	38	2	0.0592648365480729	coated pit
GO:0031300	90	3	0.0673025320946589	intrinsic to organelle membrane
GO:0005890	7	1	0.0707247599083654	sodium:potassium-exchanging ATPase complex
GO:0005887	1057	16	0.078411874926355	integral to plasma membrane
GO:0031226	1062	16	0.0810539230600003	intrinsic to plasma membrane
GO:0030425	9	1	0.090007138748936	dendrite
GO:0005578	303	6	0.095841658159021	extracellular matrix (sensu Metazoa)
GO:0031012	304	6	0.0969888179134333	extracellular matrix
GO:0005583	10	1	0.099499414770332	fibrillar collagen
GO:0005952	10	1	0.099499414770332	cAMP-dependent protein kinase complex
GO:0016020	3514	43	0.102789244297026	membrane
GO:0005856	612	10	0.103268977093346	cytoskeleton
GO:0005615	385	7	0.106017285069485	extracellular space
GO:0001518	11	1	0.108893764216335	voltage-gated sodium channel complex
GO:0030055	11	1	0.108893764216335	cell-matrix junction
GO:0005576	949	14	0.113514884236404	extracellular region
GO:0005922	12	1	0.118191186106032	connexon complex
GO:0030139	12	1	0.118191186106032	endocytic vesicle
GO:0005871	13	1	0.127392669381448	kinesin complex

GO:0016023	122	3	0.134253699036471	cytoplasmic membrane-bound vesicle
GO:0015630	191	4	0.138105978256847	microtubule cytoskeleton
GO:0031410	124	3	0.139019799835447	cytoplasmic vesicle
GO:0016021	2674	33	0.144288462055199	integral to membrane
GO:0005921	15	1	0.145511726074167	gap junction
GO:0031224	2681	33	0.148185171779482	intrinsic to membrane
GO:0005669	16	1	0.154431227889677	transcription factor TFIID complex
GO:0030141	18	1	0.171994926699681	secretory granule
GO:0019717	19	1	0.180640994294184	synaptosome
GO:0005654	221	4	0.198596332348130	nucleoplasm
GO:0005741	26	1	0.238713776189429	mitochondrial outer membrane
GO:0030176	26	1	0.238713776189429	integral to endoplasmic reticulum membrane
GO:0008021	27	1	0.246670547777276	synaptic vesicle
GO:0030054	93	2	0.252720793874755	cell junction
GO:0030173	29	1	0.262338188586433	integral to Golgi membrane
GO:0005581	30	1	0.270050730759669	collagen
GO:0031227	32	1	0.285237377803605	intrinsic to endoplasmic reticulum membrane
GO:0031228	35	1	0.307433364239528	intrinsic to Golgi membrane
GO:0043234	1247	15	0.315079808862926	protein complex
GO:0000786	40	1	0.342921723600287	nucleosome
GO:0016327	50	1	0.408590764735905	apicolateral plasma membrane
GO:0019867	52	1	0.420923585088176	outer membrane
GO:0008076	53	1	0.426994275518236	voltage-gated potassium channel complex
GO:0005941	57	1	0.450654041016973	unlocalized protein complex
GO:0016591	58	1	0.456416411216095	DNA-directed RNA polymerase II, holoenzyme
GO:0005624	521	6	0.461496229604698	membrane fraction
GO:0012505	246	3	0.475120270655988	endomembrane system
GO:0005623	7802	82	0.490366165167899	cell
GO:0030136	65	1	0.495113079172158	clathrin-coated vesicle
GO:0000139	67	1	0.505659953335653	Golgi membrane
GO:0005875	70	1	0.521073055265696	microtubule associated complex
GO:0030135	79	1	0.564517578564893	coated vesicle
GO:0015629	183	2	0.572291946622316	actin cytoskeleton
GO:0043228	977	10	0.573964495103014	non-membrane-bound organelle
GO:0043232	977	10	0.573964495103014	intracellular non-membrane-bound organelle
GO:0005911	82	1	0.578114390075929	intercellular junction
GO:0005635	83	1	0.58255269573264	nuclear membrane
GO:0005789	84	1	0.5869448230864	endoplasmic reticulum membrane
GO:0042175	90	1	0.612354113866873	nuclear envelope-endoplasmic reticulum network
GO:0005667	91	1	0.61643602912977	transcription factor complex
GO:0008372	523	5	0.642962300952192	cellular component unknown
GO:0000785	100	1	0.651311925883997	chromatin
GO:0005840	113	1	0.696220445927496	ribosome
GO:0005740	119	1	0.714969512747504	mitochondrial membrane
GO:0000267	698	6	0.74548550300688	cell fraction
GO:0031090	377	3	0.759251028407732	organelle membrane

GO:0005625	183	1	0.855938076645688	soluble fraction
GO:0005794	195	1	0.87331241427818	Golgi apparatus
GO:0005694	203	1	0.883726798312826	chromosome
GO:0030529	254	1	0.932834808735705	ribonucleoprotein complex
GO:0005829	276	1	0.947047979111132	cytosol
GO:0005634	2704	21	0.963228768448531	nucleus
GO:0005783	483	2	0.964990625345018	endoplasmic reticulum
GO:0005737	2530	18	0.98334835888946	cytoplasm
GO:0005622	5419	46	0.991150770198312	intracellular
GO:0005739	490	1	0.994930879224058	mitochondrion
GO:0043226	4515	35	0.996174701808224	organelle
GO:0043229	4515	35	0.996174701808224	intracellular organelle
GO:0043227	3955	27	0.999271184830246	membrane-bound organelle
GO:0043231	3955	27	0.999271184830246	intracellular membrane-bound organelle
GO:0005575	8637	90	1	cellular_component

2. Bronchial Epitelia

2.1 BronchialEpitelia selective genes (77 genes)

Symbol	Description	Chromosome	Chromosome Location	LocusLink	Cytoband	UniGene
MSMB	microseminoprotein, beta-	10	51219558	4477	10q11.2	Hs.255462
KRT19	keratin 19	17	-36933395	3880	17q21.2	Hs.2785
AGR2	anterior gradient 2 homolog (Xenopus laevis)	7	-16797960	10551	7p21.3	Hs.530009
SCGB1A1	secretoglobin, family 1A, member 1 (uteroglobin)	11	61943098	7356	11q12.3-q13.1	Hs.523732
CYP4B1	Cytochrome P450, family 4, subfamily B, polypeptide 1	1	47037328	1580	1p34-p12	Hs.436317
TMC5	transmembrane channel-like 5	16	19375272	79838	16p12.3	Hs.115838
PSCA	prostate stem cell antigen	8	143758876	8000	8q24.2	Hs.379010 Hs.535903
PROM1	prominin 1	4	-15578954	8842	4p15.32	Hs.479220
GSTA1	glutathione S-transferase A1	6	-52764347	2938	6p12.1	Hs.446309
WFDC2	WAP four-disulfide core domain 2	20	43531807, 43541898	10406	20q12-q13.2	Hs.2719
KRT15	keratin 15	17	-36923523	3866	17q21.2	Hs.2785
PLUNC	palate, lung and nasal epithelium carcinoma associated	20	31287462	51297	20q11.2	Hs.211092
MUC4	mucin 4, cell surface associated	3	-196959310	4585	3q29	Hs.369646
FOXA1	forkhead box A1	14	-37128941	3169	14q12-q13	Hs.163484
NA	NA	NA	NA	650893	NA	NA
EFHC1	EF-hand domain (C-terminal) containing 1	6	52393070	114327	6p12.3	Hs.403171 Hs.592773
SERPINB3	serpin peptidase inhibitor, clade B (ovalbumin), member 3	18	-59473411	6317	18q21.3	Hs.227948
S100P	S100 calcium binding protein P	4	6746466	6286	4p16	Hs.2962
TSPAN8	tetraspanin 8	12	-69805143	7103	12q14.1-q21.1	Hs.170563
RRAD	Ras-related associated with diabetes	16	-65513089	6236	16q22	Hs.1027
LCN2	lipocalin 2 (oncogene 24p3)	9	129951552	3934	9q34	Hs.204238
MUC5AC	mucin 5AC, oligomeric mucus/gel-forming	11	NA	4586	11p15.5	Hs.534332 Hs.558950 Hs.592369
MUC5B	mucin 5B, oligomeric mucus/gel-forming	11	NA	4587	11p15	Hs.523395
SLC6A14	solute carrier family 6 (amino acid transporter), member 14	X	115481817	11254	Xq23-q24	Hs.522109
AGBL2	ATP/GTP binding protein-like 2	11	-47637720	79841	11p11.2	Hs.147377
FLJ23049	NA	3	-168440778	79740	3q26.1	Hs.478143
S100A2	S100 calcium binding protein A2	1	-151800210	6273	1q21	Hs.516484
NUCB2	nucleobindin 2	11	17254887	4925	11p15.1-p14	Hs.128686
MNS1	meiosis-specific nuclear structural 1	15	-54508298	55329	15q21.3	Hs.444483
ALDH1A1	aldehyde dehydrogenase 1 family, member A1	9	-74705407	216	9q21.13	Hs.76392
TFF3	trefoil factor 3 (intestinal)	21	-42605232	7033	21q22.3	Hs.82961
ALDH3A1	aldehyde dehydrogenase 3 family, memberA1	17	-19581891	218	17p11.2	Hs.531682
TACSTD1	tumor-associated calcium signal transducer 1	2	47449970	4072	2p21	Hs.542050
LOC646082	NA	22	NA	646082	22q11.23	NA
CGI-38	NA	16	-65981214	51673	16q22.1	Hs.534458
IFT57	intraflagellar transport 57 homolog (Chlamydomonas)	3	-109362349	55081	3q13.12	Hs.412196

KRT5	keratin 5 (epidermolysis bullosa simplex, Dowling-Meara/Kobner/Weber-Cockayne types)	12	-51194627	3852	12q12-q13	Hs.433845
SCGB2A1	secretoglobin, family 2A, member 1	11	61732715	4246	11q13	Hs.97644
KRT7	keratin 7	12	50913220	3855	12q12-q13	Hs.411501
MSLN	Mesothelin	16	751133	10232	16p13.3	Hs.408488
CSTA	cystatin A (stefin A)	3	123526700	1475	3q21	Hs.518198
CD24	CD24 molecule	6	-19611913	934	6q21	Hs.375108
FABP6	fatty acid binding protein 6, ileal (gastrotropin)	5	159558625, 159589014	2172	5q33.3-q34	Hs.519719
PLAC8	placenta-specific 8	4	-84230866	51316	4q21.22	Hs.546392
LYZ	lysozyme (renal amyloidosis)	12	68028430	4069	12q15	Hs.524579
KRT18	keratin 18	12	51629109, 51628921	3875	12q13	Hs.406013
SELENBP1	selenium binding protein 1	1	-149603404	8991	1q21-q22	Hs.632460
FCGBP	Fc fragment of IgG binding protein	19	-45045810	8857	19q13.1	Hs.111732
MYB	v-myb myeloblastosis viral oncogene homolog (avian)	6	135544145	4602	6q22-q23	Hs.531941
RARRES3	retinoic acid receptor responder (tazarotene induced) 3	11	63060855	5920	11q23	Hs.17466
PROS1	protein S (alpha)	3	-95074646	5627	3q11.2	Hs.64016
LZTFL1	leucine zipper transcription factor-like 1	3	-45840449	54585	3p21.3	Hs.30824
MLF1	myeloid leukemia factor 1	3	159771677	4291	3q25.1	Hs.85195
NTS	Neurotensin	12	84792205	4922	12q21	Hs.80962
ADH1C	alcohol dehydrogenase 1C (class I), gamma polypeptide	4	-100476673	126	4q21-q23	Hs.4
VAMP8	vesicle-associated membrane protein 8 (endobrevin)	2	85658227	8673	2p12-p11.2	Hs.534373
C14orf78	chromosome 14 open reading frame 78	14	NA	113146	14q32.33	Hs.441783 Hs.632328
SLC22A4	solute carrier family 22 (organic cation transporter), member 4	5	131658043	6583	5q31.1	Hs.310591 Hs.640104
FLJ20273	NA	4	-40120497	54502	4p13-p12	Hs.518727
CETN2	centrin, EF-hand protein, 2	X	-151746528	1069	Xq28	Hs.82794
CXCL1	chemokine (C-X-C motif) ligand 1 (melanoma growth stimulating activity, alpha)	4	74953972	2919	4q21	Hs.789
DUOX1	dual oxidase 1	15	43209483	53905	15q15.3	Hs.272813
DNAI1	dynein, axonemal, intermediate polypeptide 1	9	34448810	27019	9p21-p13	Hs.112667
GABRP	gamma-aminobutyric acid (GABA) A receptor, pi	5	170143342	2568	5q33-q34	Hs.26225
KRT17	keratin 17	17	-37029220	3872	17q12-q21	Hs.2785
MMP10	matrix metallopeptidase 10 (stromelysin 2)	11	-102146443	4319	11q22.3	Hs.2258
ANXA1	annexin A1	9	74956600	301	9q12-q21.2|9q12-q21.2	Hs.494173
PPAP2C	phosphatidic acid phosphatase type 2C	19	-232045	8612	19p13	Hs.465506
CLDN3	claudin 3	7	-72821263	1365	7q11.23	Hs.520943
LRRC48	leucine rich repeat containing 48	17	17816923	83450	17p11.2	Hs.579264
DSP	Desmoplakin	6	7486868	1832	6p24	Hs.519873
EVI1	ecotropic viral integration site 1	3	-170285243	2122	3q24-q28	Hs.554762
SPAG6	sperm associated antigen 6	10	22674404	9576	10p12.2	Hs.7444
PLEKHA5	pleckstrin homology domain containing, family A member 5	12	19173994	54477	12p12	Hs.188614
TEKT2	tektin 2 (testicular)	1	36322262	27285	1p35.3-p34.1	Hs.127111
F3	coagulation factor III (thromboplastin, tissue factor)	1	-94767460	2152	1p22-p21	Hs.62192
MKL2	MKL/myocardin-like 2	16	14072696	57496	16p13.12	Hs.592047

2.2 GO: Biological Process

GO ID	genes present in chip	genes present in tissues	p-value	GO term
GO:0018149	15	3	8.74E-05	peptide cross-linking
GO:0030216	18	3	0.000154717	keratinocyte differentiation
GO:0008544	70	4	0.000781347	epidermis development
GO:0007398	79	4	0.001230572	ectoderm development
GO:0000004	465	9	0.001577206	biological process unknown
GO:0006081	13	2	0.00262365	aldehyde metabolism
GO:0045214	1	1	0.005978742	sarcomere organization
GO:0043206	1	1	0.005978742	fibril organization and biogenesis
GO:0045844	1	1	0.005978742	positive regulation of myogenesis
GO:0030154	409	7	0.010442064	cell differentiation
GO:0050665	2	1	0.011922397	hydrogen peroxide biosynthesis
GO:0009888	158	4	0.014475409	histogenesis
GO:0042335	3	1	0.017831167	cuticle biosynthesis
GO:0016998	3	1	0.017831167	cell wall catabolism
GO:0044274	3	1	0.017831167	organismal biosynthesis
GO:0050878	94	3	0.018471218	regulation of body fluids
GO:0007519	39	2	0.022574161	myogenesis
GO:0048468	40	2	0.023672429	cell development
GO:0007566	4	1	0.023705252	embryo implantation
GO:0042554	4	1	0.023705252	superoxide release
GO:0030239	4	1	0.023705252	myofibril assembly
GO:0015695	5	1	0.029544853	organic cation transport
GO:0007586	49	2	0.034496936	digestion
GO:0009617	51	2	0.037119988	response to bacteria
GO:0048627	7	1	0.041121395	myoblast development
GO:0048628	7	1	0.041121395	myoblast maturation
GO:0007286	7	1	0.041121395	spermatid development
GO:0042743	7	1	0.041121395	hydrogen peroxide metabolism
GO:0042744	7	1	0.041121395	hydrogen peroxide catabolism
GO:0048515	8	1	0.046858729	spermatid differentiation
GO:0007589	8	1	0.046858729	fluid secretion
GO:0042542	8	1	0.046858729	response to hydrogen peroxide
GO:0006749	8	1	0.046858729	glutathione metabolism
GO:0008285	139	3	0.050093635	negative regulation of cell proliferation
GO:0016202	9	1	0.052562367	regulation of myogenesis
GO:0007275	1543	14	0.065837302	development
GO:0048513	872	9	0.071906047	organ development
GO:0000302	13	1	0.07504382	response to reactive oxygen species
GO:0007596	78	2	0.079015816	blood coagulation
GO:0019835	14	1	0.080581865	cytolysis
GO:0050817	79	2	0.080766861	coagulation
GO:0006801	15	1	0.086087363	superoxide metabolism

GO:0045445	15	1	0.086087363	myoblast differentiation
GO:0007599	83	2	0.087890522	hemostasis
GO:0042060	85	2	0.09152127	wound healing
GO:0016338	16	1	0.091560501	calcium-independent cell-cell adhesion
GO:0009618	16	1	0.091560501	response to pathogenic bacteria
GO:0006904	17	1	0.097001465	vesicle docking during exocytosis
GO:0050880	17	1	0.097001465	regulation of blood vessel size
GO:0030574	17	1	0.097001465	collagen catabolism
GO:0048278	18	1	0.102410441	vesicle docking
GO:0009887	807	8	0.105085196	organogenesis
GO:0042692	19	1	0.107787614	muscle cell differentiation
GO:0007517	94	2	0.108380995	muscle development
GO:0048469	21	1	0.118447283	cell maturation
GO:0019221	21	1	0.118447283	cytokine and chemokine mediated signaling pathway
GO:0000270	21	1	0.118447283	peptidoglycan metabolism
GO:0000003	208	3	0.127586199	reproduction
GO:0048232	105	2	0.130001303	male gamete generation
GO:0007283	105	2	0.130001303	spermatogenesis
GO:0009607	857	8	0.135501378	response to biotic stimulus
GO:0009613	458	5	0.137181398	response to pest, pathogen or parasite
GO:0042446	25	1	0.139392983	hormone biosynthesis
GO:0042828	25	1	0.139392983	response to pathogen
GO:0043207	463	5	0.141739396	response to external biotic stimulus
GO:0009611	341	4	0.145925763	response to wounding
GO:0006950	881	8	0.151595084	response to stress
GO:0006952	751	7	0.158477171	defense response
GO:0045944	30	1	0.164889234	positive regulation of transcription from RNA polymerase II promoter
GO:0009653	1051	9	0.170410864	morphogenesis
GO:0007276	125	2	0.171498722	gametogenesis
GO:0006836	33	1	0.179829567	neurotransmitter transport
GO:0042742	34	1	0.184751136	defense response to bacteria
GO:0043062	35	1	0.189643717	extracellular structure organization and biogenesis
GO:0030198	35	1	0.189643717	extracellular matrix organization and biogenesis
GO:0050874	1531	12	0.193421898	organismal physiological process
GO:0007155	518	5	0.196041664	cell adhesion
GO:0006118	256	3	0.196733796	electron transport
GO:0042127	265	3	0.210632803	regulation of cell proliferation
GO:0009605	963	8	0.213232831	response to external stimulus
GO:0007588	40	1	0.213677614	excretion
GO:0019953	149	2	0.223682051	sexual reproduction
GO:0006865	43	1	0.227760663	amino acid transport
GO:0006979	44	1	0.232399769	response to oxidative stress
GO:0007565	45	1	0.237011519	pregnancy
GO:0042445	46	1	0.241596072	hormone metabolism
GO:0006334	48	1	0.250684215	nucleosome assembly
GO:0006790	48	1	0.250684215	sulfur metabolism

GO:0007160	49	1	0.255188117	cell-matrix adhesion
GO:0015837	52	1	0.268540997	amine transport
GO:0000226	52	1	0.268540997	microtubule cytoskeleton organization and biogenesis
GO:0045087	53	1	0.272939525	innate immune response
GO:0007010	305	3	0.274661362	cytoskeleton organization and biogenesis
GO:0006887	55	1	0.281658844	exocytosis
GO:0031497	56	1	0.285979934	chromatin assembly
GO:0015849	59	1	0.298790706	organic acid transport
GO:0046942	59	1	0.298790706	carboxylic acid transport
GO:0006954	185	2	0.303677688	inflammatory response
GO:0006800	61	1	0.307205609	oxygen and reactive oxygen species metabolism
GO:0048609	61	1	0.307205609	reproductive organismal physiological process
GO:0050876	62	1	0.311375813	reproductive physiological process
GO:0006461	194	2	0.32359889	protein complex assembly
GO:0006874	68	1	0.335884567	calcium ion homeostasis
GO:0046903	208	2	0.354312512	secretion
GO:0045893	77	1	0.371050609	positive regulation of transcription, DNA-dependent
GO:0006814	80	1	0.38236137	sodium ion transport
GO:0006955	684	5	0.389245144	immune response
GO:0030097	87	1	0.407982479	hemopoiesis
GO:0030005	87	1	0.407982479	di-, tri-valent inorganic cation homeostasis
GO:0044248	700	5	0.408724989	cellular catabolism
GO:0048534	88	1	0.411556425	hemopoietic or lymphoid organ development
GO:0006333	91	1	0.422151684	chromatin assembly or disassembly
GO:0006486	92	1	0.425641653	protein amino acid glycosylation
GO:0043413	93	1	0.429110931	biopolymer glycosylation
GO:0006875	93	1	0.429110931	metal ion homeostasis
GO:0009101	96	1	0.439395827	glycoprotein biosynthesis
GO:0030003	98	1	0.446151163	cation homeostasis
GO:0045941	100	1	0.452826586	positive regulation of transcription
GO:0006935	101	1	0.456134621	chemotaxis
GO:0042330	101	1	0.456134621	taxis
GO:0008015	103	1	0.462691911	circulation
GO:0006873	103	1	0.462691911	cell ion homeostasis
GO:0051243	583	4	0.464027701	negative regulation of cellular physiological process
GO:0045935	105	1	0.469171587	positive regulation of nucleobase, nucleoside, nucleotide and nucleic acid metabolism
GO:0050896	1578	10	0.475070185	response to stimulus
GO:0009056	756	5	0.47621746	catabolism
GO:0050801	111	1	0.488153901	ion homeostasis
GO:0009100	111	1	0.488153901	glycoprotein metabolism
GO:0043118	602	4	0.489352564	negative regulation of physiological process
GO:0006916	112	1	0.491252173	anti-apoptosis
GO:0007017	112	1	0.491252173	microtubule-based process
GO:0006091	446	3	0.502778434	generation of precursor metabolites and energy
GO:0006508	451	3	0.510360032	proteolysis and peptidolysis
GO:0044257	451	3	0.510360032	cellular protein catabolism

GO:0000398	119	1	0.512430596	nuclear mRNA splicing, via spliceosome
GO:0000375	119	1	0.512430596	RNA splicing, via transesterification reactions
GO:0000377	119	1	0.512430596	RNA splicing, via transesterification reactions with bulged adenosine as nucleophile
GO:0006732	121	1	0.518320972	coenzyme metabolism
GO:0019725	122	1	0.521239911	cell homeostasis
GO:0030163	463	3	0.528319321	protein catabolism
GO:0030036	126	1	0.532743049	actin cytoskeleton organization and biogenesis
GO:0043066	128	1	0.538392451	negative regulation of apoptosis
GO:0048523	640	4	0.538571396	negative regulation of cellular process
GO:0043069	129	1	0.541191958	negative regulation of programmed cell death
GO:0008283	473	3	0.543018861	cell proliferation
GO:0043285	474	3	0.544475039	biopolymer catabolism
GO:0042981	306	2	0.550793619	regulation of apoptosis
GO:0043067	308	2	0.554367283	regulation of programmed cell death
GO:0007067	135	1	0.557642408	mitosis
GO:0031325	136	1	0.560327281	positive regulation of cellular metabolism
GO:0008380	137	1	0.562996158	RNA splicing
GO:0000087	137	1	0.562996158	M phase of mitotic cell cycle
GO:0030029	139	1	0.568286297	actin filament-based process
GO:0006959	141	1	0.573513569	humoral immune response
GO:0051301	142	1	0.576103859	cell division
GO:0008219	498	3	0.5786396	cell death
GO:0051186	143	1	0.578678706	cofactor metabolism
GO:0016265	501	3	0.582801298	death
GO:0009893	149	1	0.593808516	positive regulation of metabolism
GO:0050953	149	1	0.593808516	sensory perception of light
GO:0007601	149	1	0.593808516	visual perception
GO:0048519	688	4	0.597368531	negative regulation of biological process
GO:0006996	693	4	0.603247835	organelle organization and biogenesis
GO:0043065	155	1	0.608404714	positive regulation of apoptosis
GO:0043068	156	1	0.610786841	positive regulation of programmed cell death
GO:0042592	157	1	0.613154745	homeostasis
GO:0045045	161	1	0.622485777	secretory pathway
GO:0005975	355	2	0.632682281	carbohydrate metabolism
GO:0016337	172	1	0.647021957	cell-cell adhesion
GO:0000279	173	1	0.64917329	M phase
GO:0044265	563	3	0.66304832	cellular macromolecule catabolism
GO:0006397	180	1	0.663876449	mRNA processing
GO:0006520	181	1	0.665926908	amino acid metabolism
GO:0042221	183	1	0.669991066	response to chemical substance
GO:0006325	186	1	0.675996472	establishment and/or maintenance of chromatin architecture
GO:0009057	575	3	0.677271634	macromolecule catabolism
GO:0006323	193	1	0.689595067	DNA packaging
GO:0000278	204	1	0.709840402	mitotic cell cycle
GO:0006928	205	1	0.711615279	cell motility
GO:0040011	205	1	0.711615279	locomotion

GO:0016071	205	1	0.711615279	mRNA metabolism
GO:0051242	411	2	0.711847707	positive regulation of cellular physiological process
GO:0007626	209	1	0.718608846	locomotory behavior
GO:0006066	213	1	0.725435879	alcohol metabolism
GO:0006519	216	1	0.730449211	amino acid and derivative metabolism
GO:0043119	428	2	0.732952845	positive regulation of physiological process
GO:0007001	221	1	0.738605824	chromosome organization and biogenesis (sensu Eukaryota)
GO:0015672	223	1	0.741800201	monovalent inorganic cation transport
GO:0007264	227	1	0.748074437	small GTPase mediated signal transduction
GO:0016043	842	4	0.754281258	cell organization and biogenesis
GO:0051276	236	1	0.761649435	chromosome organization and biogenesis
GO:0006357	240	1	0.76744982	regulation of transcription from RNA polymerase II promoter
GO:0006810	1802	9	0.777142789	transport
GO:0006629	469	2	0.778622106	lipid metabolism
GO:0006915	471	2	0.780669221	apoptosis
GO:0012501	473	2	0.782700158	programmed cell death
GO:0048522	482	2	0.791641477	positive regulation of cellular process
GO:0051234	1841	9	0.799570799	establishment of localization
GO:0044262	265	1	0.800687768	cellular carbohydrate metabolism
GO:0009308	269	1	0.805554033	amine metabolism
GO:0030001	269	1	0.805554033	metal ion transport
GO:0007610	269	1	0.805554033	behavior
GO:0051179	1857	9	0.808302671	localization
GO:0006811	515	2	0.821766664	ion transport
GO:0007600	287	1	0.826047578	sensory perception
GO:0006807	291	1	0.830305263	nitrogen compound metabolism
GO:0016192	296	1	0.835483754	vesicle-mediated transport
GO:0009581	313	1	0.851956842	detection of external stimulus
GO:0009628	318	1	0.856485864	response to abiotic stimulus
GO:0048518	565	2	0.860148009	positive regulation of biological process
GO:0006396	326	1	0.863450955	RNA processing
GO:0006812	346	1	0.87944468	cation transport
GO:0007049	601	2	0.883030173	cell cycle
GO:0019752	359	1	0.888838897	carboxylic acid metabolism
GO:0006082	361	1	0.890218894	organic acid metabolism
GO:0006464	1087	4	0.903617783	protein modification
GO:0007399	385	1	0.905522895	neurogenesis
GO:0016070	391	1	0.909009101	RNA metabolism
GO:0044267	2109	9	0.911992442	cellular protein metabolism
GO:0006412	407	1	0.9177001	protein biosynthesis
GO:0019538	2129	9	0.917765315	protein metabolism
GO:0006366	426	1	0.926966785	transcription from RNA polymerase II promoter
GO:0043412	1160	4	0.929257359	biopolymer modification
GO:0007166	952	3	0.934049896	cell surface receptor linked signal transduction
GO:0043170	2405	10	0.938829152	macromolecule metabolism
GO:0009059	464	1	0.942533192	macromolecule biosynthesis

GO:0043283	2240	9	0.944450125	biopolymer metabolism
GO:0050877	475	1	0.946396217	neurophysiological process
GO:0044249	763	2	0.949536346	cellular biosynthesis
GO:0006259	489	1	0.950945919	DNA metabolism
GO:0007186	491	1	0.951564134	G-protein coupled receptor protein signaling pathway
GO:0044260	2298	9	0.955217867	cellular macromolecule metabolism
GO:0007154	2730	11	0.962504672	cell communication
GO:0009058	859	2	0.970084589	biosynthesis
GO:0007242	879	2	0.973227779	intracellular signaling cascade
GO:0051244	2319	8	0.981668545	regulation of cellular physiological process
GO:0050791	2407	8	0.987567199	regulation of physiological process
GO:0050794	2488	8	0.991421962	regulation of cellular process
GO:0006355	1359	3	0.991823422	regulation of transcription, DNA-dependent
GO:0006351	1407	3	0.99373508	transcription, DNA-dependent
GO:0045449	1433	3	0.994585411	regulation of transcription
GO:0007165	2168	6	0.994971832	signal transduction
GO:0019219	1458	3	0.995298975	regulation of nucleobase, nucleoside, nucleotide and nucleic acid metabolism
GO:0006350	1499	3	0.996279705	transcription
GO:0050789	2689	8	0.996770973	regulation of biological process
GO:0031323	1566	3	0.997476596	regulation of cellular metabolism
GO:0044237	5026	20	0.99809391	cellular metabolism
GO:0019222	1629	3	0.998259629	regulation of metabolism
GO:0008152	5262	21	0.99869463	metabolism
GO:0007582	7846	38	0.999638327	physiological process
GO:0006139	2328	4	0.999878377	nucleobase, nucleoside, nucleotide and nucleic acid metabolism
GO:0050875	7018	30	0.999921619	cellular physiological process
GO:0009987	8200	40	0.999927887	cellular process
GO:0044238	4820	15	0.999963067	primary metabolism
GO:0008150	9032	54	1	biological_process

2.3 GO: Molecular Function

GO ID	genes present in chip	genes present in tissues	p-value	GO term
GO:0005198	488	14	1.70224701096761e-06	structural molecule activity
GO:0005200	86	6	1.67912828615429e-05	structural constituent of cytoskeleton
GO:0019834	3	2	0.000120133903112198	phospholipase A2 inhibitor activity
GO:0005497	4	2	0.00023927751541215	androgen binding
GO:0004859	8	2	0.00109835269152808	phospholipase inhibitor activity
GO:0004029	8	2	0.00109835269152808	aldehyde dehydrogenase (NAD) activity
GO:0004857	187	6	0.00118732064373448	enzyme inhibitor activity
GO:0005496	42	3	0.00239078718346293	steroid binding
GO:0004028	12	2	0.00254667538424248	aldehyde dehydrogenase activity
GO:0042562	12	2	0.00254667538424248	hormone binding
GO:0008289	298	7	0.00277936154169351	lipid binding
GO:0004866	99	4	0.00362029019572850	endopeptidase inhibitor activity
GO:0030414	100	4	0.00375332114535636	protease inhibitor activity
GO:0005543	169	5	0.00439328893074359	phospholipid binding
GO:0016620	18	2	0.0057598860353529	oxidoreductase activity, acting on the aldehyde or oxo group of donors, NAD or NADP as acceptor
GO:0008513	1	1	0.00639345980505157	organic cation porter activity
GO:0030303	1	1	0.00639345980505157	stromelysin 2 activity
GO:0030674	69	3	0.00965895598005152	protein binding, bridging
GO:0035091	132	4	0.00994292587143172	phosphoinositide binding
GO:0016903	25	2	0.0109745992699244	oxidoreductase activity, acting on the aldehyde or oxo group of donors
GO:0005201	73	3	0.0112615191630274	extracellular matrix structural constituent
GO:0048306	2	1	0.0127467091710782	calcium-dependent protein binding
GO:0008106	2	1	0.0127467091710782	alcohol dehydrogenase (NADP+) activity
GO:0001758	2	1	0.0127467091710782	retinal dehydrogenase activity
GO:0030234	509	8	0.0151406271798085	enzyme regulator activity
GO:0005176	3	1	0.0190599968050614	ErbB-2 class receptor binding
GO:0008195	3	1	0.0190599968050614	phosphatidate phosphatase activity
GO:0003796	3	1	0.0190599968050614	lysozyme activity
GO:0016174	3	1	0.0190599968050614	NAD(P)H oxidase activity
GO:0030197	3	1	0.0190599968050614	extracellular matrix constituent, lubricant activity
GO:0005509	646	9	0.0207099817930560	calcium ion binding
GO:0048503	92	3	0.0208808567147127	GPI anchor binding
GO:0050664	4	1	0.0253335699016112	oxidoreductase activity, acting on NADH or NADPH, with oxygen as acceptor
GO:0004024	4	1	0.0253335699016112	alcohol dehydrogenase activity, zinc-dependent
GO:0004030	6	1	0.0377625537531709	aldehyde dehydrogenase [NAD(P)+] activity
GO:0042605	7	1	0.0439184514166272	peptide antigen binding
GO:0015101	7	1	0.0439184514166272	organic cation transporter activity
GO:0005554	522	7	0.0478427179276398	molecular function unknown
GO:0004022	8	1	0.0500356083773564	alcohol dehydrogenase activity
GO:0005099	8	1	0.0500356083773564	Ras GTPase activator activity
GO:0005545	9	1	0.0561142644026352	phosphatidylinositol binding
GO:0004033	10	1	0.0621546578008135	aldo-keto reductase activity
GO:0004867	64	2	0.0629659009481101	serine-type endopeptidase inhibitor activity

GO:0004364	14	1	0.0859383207142185	glutathione transferase activity
GO:0004182	14	1	0.0859383207142185	carboxypeptidase A activity
GO:0016616	77	2	0.086754265447086	oxidoreductase activity, acting on the CH-OH group of donors, NAD or NADP as acceptor
GO:0005328	16	1	0.0976066666514421	neurotransmitter:sodium symporter activity
GO:0016614	83	2	0.0985139606723922	oxidoreductase activity, acting on CH-OH group of donors
GO:0015293	83	2	0.0985139606723922	symporter activity
GO:0005544	17	1	0.103385773563175	calcium-dependent phospholipid binding
GO:0005504	17	1	0.103385773563175	fatty acid binding
GO:0050661	17	1	0.103385773563175	NADP binding
GO:0005125	181	3	0.109073341168233	cytokine activity
GO:0005326	18	1	0.109128472829518	neurotransmitter transporter activity
GO:0008430	18	1	0.109128472829518	selenium binding
GO:0004181	18	1	0.109128472829518	metallocarboxypeptidase activity
GO:0004869	19	1	0.114834990015006	cysteine protease inhibitor activity
GO:0050381	20	1	0.120505549310227	unspecific monooxygenase activity
GO:0004890	21	1	0.126140373540042	GABA-A receptor activity
GO:0005184	21	1	0.126140373540042	neuropeptide hormone activity
GO:0004601	22	1	0.131739684171770	peroxidase activity
GO:0016684	22	1	0.131739684171770	oxidoreductase activity, acting on peroxide as acceptor
GO:0016917	23	1	0.137303701323312	GABA receptor activity
GO:0016712	25	1	0.148326728958784	oxidoreductase activity, acting on paired donors, with incorporation or reduction of molecular oxygen, reduced flavin or flavoprotein as one donor, and incorporation of one atom of oxygen
GO:0004180	27	1	0.159211190707206	carboxypeptidase activity
GO:0008022	28	1	0.164601995559724	protein C-terminus binding
GO:0003823	30	1	0.175281814176404	antigen binding
GO:0019825	31	1	0.180571248445696	oxygen binding
GO:0016209	31	1	0.180571248445696	antioxidant activity
GO:0008307	33	1	0.191050208569587	structural constituent of muscle
GO:0015370	35	1	0.201397331568887	solute:sodium symporter activity
GO:0008237	133	2	0.208818793947454	metallopeptidase activity
GO:0016765	37	1	0.211614248801182	transferase activity, transferring alkyl or aryl (other than methyl) groups
GO:0015294	39	1	0.221702571775557	solute:cation symporter activity
GO:0016491	509	5	0.224855647401151	oxidoreductase activity
GO:0008009	40	1	0.226699008643763	chemokine activity
GO:0042379	40	1	0.226699008643763	chemokine receptor binding
GO:0001664	42	1	0.236597418975678	G-protein-coupled receptor binding
GO:0005254	43	1	0.241499783165854	chloride channel activity
GO:0008235	44	1	0.246371178536130	metalloexopeptidase activity
GO:0050660	44	1	0.246371178536130	FAD binding
GO:0015290	149	2	0.246597983715520	electrochemical potential-driven transporter activity
GO:0015291	149	2	0.246597983715520	porter activity
GO:0005102	528	5	0.247517263780794	receptor binding
GO:0031404	45	1	0.251211797479933	chloride ion binding
GO:0043168	45	1	0.251211797479933	anion binding
GO:0005253	46	1	0.256021831215583	anion channel activity
GO:0004896	48	1	0.265550902102445	hematopoietin/interferon-class (D200-domain) cytokine receptor activity
GO:0015171	48	1	0.265550902102445	amino acid transporter activity

GO:0042802	158	2	0.267995214458782	protein self binding
GO:0019904	52	1	0.28425030618961	protein domain specific binding
GO:0005230	57	1	0.306967111704077	extracellular ligand-gated ion channel activity
GO:0016651	57	1	0.306967111704077	oxidoreductase activity, acting on NADH or NADPH
GO:0005275	58	1	0.311424619737191	amine transporter activity
GO:0004553	59	1	0.315853924619193	hydrolase activity, hydrolyzing O-glycosyl compounds
GO:0016563	62	1	0.328974369886399	transcriptional activator activity
GO:0008047	188	2	0.339017232066125	enzyme activator activity
GO:0005342	66	1	0.346083977173599	organic acid transporter activity
GO:0046943	66	1	0.346083977173599	carboxylic acid transporter activity
GO:0008238	68	1	0.354477131120691	exopeptidase activity
GO:0031402	70	1	0.362764300830208	sodium ion binding
GO:0004497	71	1	0.366868553564804	monooxygenase activity
GO:0046906	74	1	0.379025936598634	tetrapyrrole binding
GO:0020037	74	1	0.379025936598634	heme binding
GO:0016798	77	1	0.390953628272066	hydrolase activity, acting on glycosyl bonds
GO:0004222	77	1	0.390953628272066	metalloendopeptidase activity
GO:0030594	77	1	0.390953628272066	neurotransmitter receptor activity
GO:0005506	212	2	0.394447795439349	iron ion binding
GO:0042165	78	1	0.394879223017988	neurotransmitter binding
GO:0015276	84	1	0.417915496794711	ligand-gated ion channel activity
GO:0016705	87	1	0.429107943908796	oxidoreductase activity, acting on paired donors, with incorporation or reduction of molecular oxygen
GO:0050662	88	1	0.432791508053239	coenzyme binding
GO:0005179	90	1	0.440088641423776	hormone activity
GO:0008509	95	1	0.457929919360026	anion transporter activity
GO:0003774	98	1	0.468365020971253	motor activity
GO:0048037	100	1	0.475211721163258	cofactor binding
GO:0005488	7228	47	0.4758027730871	binding
GO:0005516	112	1	0.514507578209415	calmodulin binding
GO:0043169	2313	15	0.5241972269129	cation binding
GO:0005096	117	1	0.530013171647839	GTPase activator activity
GO:0004721	118	1	0.533055318987555	phosphoprotein phosphatase activity
GO:0003924	121	1	0.542066022055164	GTPase activity
GO:0042277	127	1	0.559577369581274	peptide binding
GO:0043167	2556	16	0.58809224248425	ion binding
GO:0003713	140	1	0.595290161699189	transcription coactivator activity
GO:0008083	144	1	0.6056941272368	growth factor activity
GO:0005083	144	1	0.6056941272368	small GTPase regulator activity
GO:0031420	146	1	0.610797002996365	alkali metal ion binding
GO:0005515	3709	23	0.621931104654684	protein binding
GO:0046872	2495	15	0.657127317770243	metal ion binding
GO:0015075	523	3	0.657604151878497	ion transporter activity
GO:0016791	171	1	0.669355966291117	phosphoric monoester hydrolase activity
GO:0005386	398	2	0.72926014068062	carrier activity
GO:0008233	399	2	0.730553111061982	peptidase activity
GO:0008324	421	2	0.757724437943816	cation transporter activity
GO:0030695	219	1	0.75853064518388	GTPase regulator activity

GO:0005525	226	1	0.769379943720876	GTP binding
GO:0042578	228	1	0.772390681577847	phosphoric ester hydrolase activity
GO:0003712	230	1	0.775362750406303	transcription cofactor activity
GO:0019001	235	1	0.782626936218993	guanyl nucleotide binding
GO:0000287	251	1	0.80435416303715	magnesium ion binding
GO:0005216	252	1	0.805638812773935	ion channel activity
GO:0004888	694	3	0.830689582957527	transmembrane receptor activity
GO:0015268	274	1	0.83189224598629	alpha-type channel activity
GO:0015267	284	1	0.842640909149531	channel or pore class transporter activity
GO:0004175	288	1	0.84674848887546	endopeptidase activity
GO:0008134	293	1	0.851734905969901	transcription factor binding
GO:0004871	1837	9	0.856128469849158	signal transducer activity
GO:0003677	1384	6	0.894391746254303	DNA binding
GO:0046914	1596	7	0.904229961776056	transition metal ion binding
GO:0005215	1030	4	0.90757781884715	transporter activity
GO:0008270	1287	5	0.928157186466642	zinc ion binding
GO:0017111	410	1	0.931980299998613	nucleoside-triphosphatase activity
GO:0016462	430	1	0.940522496203226	pyrophosphatase activity
GO:0016818	432	1	0.941316301493606	hydrolase activity, acting on acid anhydrides, in phosphorus-containing anhydrides
GO:0016817	433	1	0.941709287069288	hydrolase activity, acting on acid anhydrides
GO:0003723	436	1	0.942872777750285	RNA binding
GO:0016788	446	1	0.946588514668397	hydrolase activity, acting on ester bonds
GO:0030528	963	3	0.953558589934665	transcription regulator activity
GO:0016787	1424	5	0.960929951624748	hydrolase activity
GO:0004872	1060	3	0.972121173069916	receptor activity
GO:0003676	1990	7	0.981544267365692	nucleic acid binding
GO:0003700	649	1	0.986583707655566	transcription factor activity
GO:0000166	1432	4	0.98707582077745	nucleotide binding
GO:0017076	1248	3	0.990188364557012	purine nucleotide binding
GO:0030554	1028	2	0.992148730052224	adenyl nucleotide binding
GO:0005524	970	1	0.99858673352959	ATP binding
GO:0003824	3409	11	0.999288159563763	catalytic activity
GO:0016740	1169	1	0.999664516921126	transferase activity
GO:0003674	9541	61	1	molecular_function

2.4 GO: Cellular Component

GO ID	genes present in chip	genes present in tissues	p-value	GO term
GO:0045111	65	7	2.46262964976085e-07	intermediate filament cytoskeleton
GO:0005882	65	7	2.46262964976085e-07	intermediate filament
GO:0005856	612	14	4.65769483450198e-05	cytoskeleton
GO:0001533	12	3	6.3767234856488e-05	cornified envelope
GO:0005930	9	2	0.00160146655346312	axoneme
GO:0043228	977	15	0.00187233042854637	non-membrane-bound organelle
GO:0043232	977	15	0.00187233042854637	intracellular non-membrane-bound organelle
GO:0015630	191	5	0.00956701640382268	microtubule cytoskeleton
GO:0005576	949	13	0.0103553467710514	extracellular region
GO:0042995	70	3	0.0119888124042774	cell projection
GO:0043034	4	1	0.0270502438419075	costamere
GO:0043025	5	1	0.0336996166686565	cell soma
GO:0043205	5	1	0.0336996166686565	fibril
GO:0005929	5	1	0.0336996166686565	cilium
GO:0005813	43	2	0.0346344011058444	centrosome
GO:0019861	6	1	0.0403043111330389	flagellum
GO:0016324	6	1	0.0403043111330389	apical plasma membrane
GO:0005815	49	2	0.0439516863020069	microtubule organizing center
GO:0045177	7	1	0.0468646222954948	apical part of cell
GO:0005913	7	1	0.0468646222954948	cell-cell adherens junction
GO:0000922	51	2	0.0472427815844031	spindle pole
GO:0005874	132	3	0.0611110325864766	microtubule
GO:0016363	10	1	0.0662821779291669	nuclear matrix
GO:0005819	68	2	0.0784728858746277	spindle
GO:0005769	13	1	0.0853107236003936	early endosome
GO:0005911	82	2	0.107789023308662	intercellular junction
GO:0005912	18	1	0.116181242085589	adherens junction
GO:0005615	385	5	0.121605593271635	extracellular space
GO:0030286	19	1	0.122231283472867	dynein complex
GO:0030054	93	2	0.132545358901847	cell junction
GO:0005793	24	1	0.151876090255623	ER-Golgi intermediate compartment
GO:0008021	27	1	0.169188194081990	synaptic vesicle
GO:0005886	1472	13	0.194707815470107	plasma membrane
GO:0030016	32	1	0.197272848812837	myofibril
GO:0005923	36	1	0.219066745579896	tight junction
GO:0043292	40	1	0.240278702449429	contractile fiber
GO:0043296	40	1	0.240278702449429	apical junction complex
GO:0000786	40	1	0.240278702449429	nucleosome
GO:0008372	523	5	0.285358473213631	cellular component unknown
GO:0016327	50	1	0.290861902181266	apicolateral plasma membrane
GO:0005681	57	1	0.324284987426481	spliceosome complex
GO:0005578	303	3	0.342557282188297	extracellular matrix (sensu Metazoa)

GO:0031012	304	3	0.344444530768327	extracellular matrix
GO:0005768	62	1	0.347205556377569	endosome
GO:0005625	183	2	0.356631707507976	soluble fraction
GO:0030136	65	1	0.360589198519722	clathrin-coated vesicle
GO:0045211	66	1	0.364990182804293	postsynaptic membrane
GO:0005875	70	1	0.382298279449845	microtubule associated complex
GO:0030135	79	1	0.419563685345533	coated vesicle
GO:0045202	98	1	0.491126725862294	synapse
GO:0000785	100	1	0.498134934288555	chromatin
GO:0005764	107	1	0.521924549162197	lysosome
GO:0000323	107	1	0.521924549162197	lytic vacuole
GO:0005792	108	1	0.525231284402459	microsome
GO:0042598	108	1	0.525231284402459	vesicular fraction
GO:0005773	120	1	0.563200418209914	vacuole
GO:0005829	276	2	0.567047451301134	cytosol
GO:0016023	122	1	0.569231512570484	cytoplasmic membrane-bound vesicle
GO:0031410	124	1	0.575180725061217	cytoplasmic vesicle
GO:0000267	698	4	0.713244857166723	cell fraction
GO:0005887	1057	6	0.744783118032595	integral to plasma membrane
GO:0031226	1062	6	0.749012409427116	intrinsic to plasma membrane
GO:0005694	203	1	0.755381298556876	chromosome
GO:0005737	2530	15	0.785593726741629	cytoplasm
GO:0030529	254	1	0.829180499205617	ribonucleoprotein complex
GO:0005624	521	2	0.878998677948446	membrane fraction
GO:0005622	5419	33	0.888053070599616	intracellular
GO:0016020	3514	19	0.93003171898139	membrane
GO:0016021	2674	13	0.952186397833857	integral to membrane
GO:0031224	2681	13	0.953475977142632	intrinsic to membrane
GO:0005783	483	1	0.966860997662594	endoplasmic reticulum
GO:0043226	4515	24	0.972712846597805	organelle
GO:0043229	4515	24	0.972712846597805	intracellular organelle
GO:0043234	1247	3	0.994089348672588	protein complex
GO:0005623	7802	47	0.996455127324384	cell
GO:0005634	2704	8	0.99955815202715	nucleus
GO:0043227	3955	12	0.999988687828016	membrane-bound organelle
GO:0043231	3955	12	0.999988687828016	intracellular membrane-bound organelle
GO:0005575	8637	59	1	cellular_component

3. LimbMuscle

3.1 LimbMuscle selective genes (136 genes)

Symbol	Description	Chromosome	Chromosome Location	LocusLink	Cytoband	UniGene
KBTBD10	kelch repeat and BTB (POZ) domain containing 10	2	170074457	10324	2q31.1	Hs.50550
MYH1	myosin, heavy polypeptide 1, skeletal muscle, adult	17	-10336353	4619	17p13.1	Hs.499745
MYOT	myotilin	5	137231472	9499	5q31	Hs.84665
TNNC1	troponin C type 1 (slow)	3	-52460157	7134	3p21.3-p14.3	Hs.118845
NEB	nebulin	2	-152050100	4703	2q22	Hs.134602 Hs.588655
CA3	carbonic anhydrase III, muscle specific	8	86537709	761	8q13-q22	Hs.82129
MYH7	myosin, heavy polypeptide 7, cardiac muscle, beta	14	-22951789	4625	14q12	Hs.278432
MYH2	myosin, heavy polypeptide 2, skeletal muscle, adult	17	-10365193	4620	17p13.1	Hs.499745
CSRP3	cysteine and glycine-rich protein 3 (cardiac LIM protein)	11	-19160153	8048	11p15.1	Hs.83577
TNNI1	troponin I type 1 (skeletal, slow)	1	-199639518	7135	1q31.3	Hs.320890
TTN	titin	2	-358417256	7273	2q31	Hs.134602
MYBPC2	myosin binding protein C, fast type	19	55628003	4606	19q13.33	Hs.85937
TPM2	tropomyosin 2 (beta)	9	-71344912	7169	9p13.2-p13.1	Hs.300772
CASQ1	calsequestrin 1 (fast-twitch, skeletal muscle)	1	158426988	844	1q21	Hs.632476
MYBPC1	myosin binding protein C, slow type	12	100512877	4604	12q23.2	Hs.485903
MYL1	myosin, light polypeptide 1, alkali, skeletal, fast	2	-210863121	4632	2q33-q34	Hs.187338
MYL2	myosin, light polypeptide 2, regulatory, cardiac, slow	12	-109833032	4633	12q23-q24.3	Hs.75535
AMPD1	adenosine monophosphate deaminase 1 (isoform M)	1	-115017244	270	1p13	Hs.89570
AGL	amylo-1, 6-glucosidase, 4-alpha-glucanotransferase (glycogen debranching enzyme, glycogen storage disease type III)	1	100088632, 100088227, 100099353, 100089118	178	1p21	Hs.904
ENO3	enolase 3 (beta, muscle)	17	4795158	2027	17pter-p11	Hs.224171
ACTA1	actin, alpha 1, skeletal muscle	1	-227633617	58	1q42.13-q42.2	Hs.1288
NRAP	nebulin-related anchoring protein	10	-115338573	4892	10q24-q26	Hs.268788
RPL3L	ribosomal protein L3-like	16	-1934581	6123	16p13.3	Hs.498569
LRRC2	leucine rich repeat containing 2	3	-93066791	79442	3p21.31	Hs.570630
CKM	creatine kinase, muscle	19	-50501511	1158	19q13.2-q13.3	Hs.334347
PLN	phospholamban	6	118976164	5350	6q22.1	Hs.170839
SMPX	small muscle protein, X-linked	X	-21634012	23676	Xp22.1	Hs.86492
TNNT1	troponin T type 1 (skeletal, slow)	19	-60336008	7138	19q13.4	Hs.631558
TNNI2	troponin I type 2 (skeletal, fast)	11	1817478	7136	11p15.5	Hs.523403
TNNT3	troponin T type 3 (skeletal, fast)	11	1900668	7140	11p15.5	Hs.73454
RYR1	ryanodine receptor 1 (skeletal)	19	43616179	6261	19q13.1	Hs.466664
LOC125893	NA	19	-58144445	125893	19q13.41	Hs.446907
ATP2A1	ATPase, Ca++ transporting, cardiac muscle, fast twitch 1	16	28797309	487	16p12.1	Hs.183075
SYNPO2L	synaptopodin 2-like	10	-75075295	79933	10q22.2	Hs.533659
MYOZ2	myozenin 2	4	120276468	51778	4q26-q27	Hs.381047
MYOM2	myomesin (M-protein) 2, 165kDa	8	1980654	9172	8p23.3	Hs.443683

RHOBTB1	Rho-related BTB domain containing 1	10	-62299205	9886	10q21.2	Hs.148670
GBA2	glucosidase, beta (bile acid) 2	9	-35726863	57704	9p13.3	Hs.443134
TNNC2	troponin C type 2 (fast)	20	-43885262	7125	20q12-q13.11	Hs.182421
CKMT2	creatine kinase, mitochondrial 2 (sarcomeric)	5	80564937	1160	5q13.3	Hs.80691
GBAS	glioblastoma amplified sequence	7	55999789	2631	7p12	Hs.591069
MB	myoglobin	22	-34332757	4151	22q13.1	Hs.517586
PDLIM3	PDZ and LIM domain 3	4	-186659904	27295	4q35	Hs.85862
COX6A2	cytochrome c oxidase subunit VIa polypeptide 2	16	-31346555	1339	16p	Hs.250760
GHR	growth hormone receptor	5	42459782	2690	5p13-p12	Hs.125180
CAPN3	calpain 3, (p94)	15	40438989, 40481879, 40484268, 40427592	825	15q15.1-q21.1	Hs.143261
LOC653513	NA	1	NA	653513	1q21.1	Hs.632434
PDE4DIP	phosphodiesterase 4D interacting protein (myomegalin)	1	-430827846	9659	1q12	Hs.568247 Hs.584841 Hs.613082 Hs.626094
FBP2	fructose-1,6-bisphosphatase 2	9	-96360825	8789	9q22.3	Hs.61255
MYOM1	myomesin 1 (skelemin) 185kDa	18	-3056805	8736	18p11.32-p11.31	Hs.464469
LDB3	LIM domain binding 3	10	88418407	11155	10q22.3-q23.2	Hs.49998
SLC19A2	solute carrier family 19 (thiamine transporter), member 2	1	-167699791	10560	1q23.3	Hs.30246
MYF6	myogenic factor 6 (herculin)	12	79625576	4618	12q21	Hs.35937
PPP1R1A	protein phosphatase 1, regulatory (inhibitor) subunit 1A	12	-53259299	5502	12q13.2	Hs.505662
DES	desmin	2	219991342	1674	2q35	Hs.594952
FYCO1	FYVE and coiled-coil domain containing 1	3	-45934399	79443	3p21.31	Hs.200227
SH3BGR	SH3 domain binding glutamic acid-rich protein	21	39739666, 39745649	6450	21q22.3	Hs.473847
TMEM38B	transmembrane protein 38B	9	107496645	55151	9q31.2	Hs.411925
PHYH	phytanoyl-CoA 2-hydroxylase	10	-13359802	5264	10pter-p11.2	Hs.498732
PGAM2	phosphoglycerate mutase 2 (muscle)	7	-44068851	5224	7p13-p12	Hs.632642
FHL1	four and a half LIM domains 1	X	135057345	2273	Xq26	Hs.435369
UBE2D1	ubiquitin-conjugating enzyme E2D 1 (UBC4/5 homolog, yeast)	10	59764744	7321	10q11.2-q21	Hs.129683
PYGM	phosphorylase, glycogen, muscle (McArdle syndrome, glycogen storage disease type V)	11	-64270605	5837	11q12-q13.2	Hs.154084
ART3	ADP-ribosyltransferase 3	4	77214886	419	4p15.1-p14|4p15.1-p14|4p15.1-p14	Hs.24976
COL15A1	collagen, type XV, alpha 1	9	100746010	1306	9q21-q22	Hs.409034
ACTN2	actinin, alpha 2	1	234916430	88	1q42-q43	Hs.498178
EEF1A2	eukaryotic translation elongation factor 1 alpha 2	20	-61589809	1917	20q13.3	Hs.433839
HSPB7	heat shock 27kDa protein family, member 7 (cardiovascular)	1	-16213111	27129	1p36.23-p34.3	Hs.502612
PDK4	pyruvate dehydrogenase kinase, isozyme 4	7	-95050748	5166	7q21.3	Hs.8364
SLN	sarcolipin	11	-107083318	6588	11q22-q23	Hs.334629
MYH6	myosin, heavy polypeptide 6, cardiac muscle, alpha (cardiomyopathy, hypertrophic 1)	14	-22921038	4624	14q12	Hs.278432
IQWD1	IQ motif and WD repeats 1	1	166172531	55827	1q24.2	Hs.435741
CD36	CD36 molecule (thrombospondin receptor)	7	80105892, 80069458	948	7q11.2	Hs.120949 Hs.633085
CAND2	cullin-associated and neddylation-dissociated 2 (putative)	3	NA	23066	3p25.1	Hs.343664
TPM3	tropomyosin 3	1	-304802035	7170	1q21.2	Hs.129512 Hs.146070 Hs.449194

SYNE2	spectrin repeat containing, nuclear envelope 2	14	63389453, 63751902, 63740825, 63750614, 63612085	23224	14q23.2	Hs.525392
TCAP	titin-cap (telethonin)	17	35075124	8557	17q12	Hs.514146
MN1	meningioma (disrupted in balanced translocation) 1	22	-26474265	4330	22q11|22q12.1	Hs.268515
FABP4	fatty acid binding protein 4, adipocyte	8	-82553489	2167	8q21	Hs.391561
SYNC1	syncoilin, intermediate filament 1	1	-32918564	81493	1p34.3-p33	Hs.642703
HSPB3	heat shock 27kDa protein 3	5	53787201	8988	5q11.2	Hs.41707
COX7A1	cytochrome c oxidase subunit VIIa polypeptide 1 (muscle)	19	-41333663	1346	19q13.1	Hs.421621
CHCHD3	coiled-coil-helix-coiled-coil-helix domain containing 3	7	-132120163	54927	7q32.3-q33	Hs.444467
VDAC1	voltage-dependent anion channel 1	5	-133335505	7416	5q31	Hs.519320
IL32	interleukin 32	16	3055313, 3055663, 3055639, 3055612, 3055785	9235	16p13.3	Hs.943
MYOZ1	myozenin 1	10	-75061420	58529	10q22.1	Hs.238756
MYL3	myosin, light polypeptide 3, alkali, ventricular, skeletal, slow	3	-46874370	4634	3p21.3-p21.2	Hs.517939
ATP1A2	ATPase, Na+/K+ transporting, alpha 2 (+) polypeptide	1	158352171	477	1q21-q23	Hs.34114
ACADM	acyl-Coenzyme A dehydrogenase, C-4 to C-12 straight chain	1	75962869	34	1p31	Hs.445040
CPT1B	carnitine palmitoyltransferase 1B (muscle)	22	-49354156	1375	22q13.33	Hs.439777
SUCLA2	succinate-CoA ligase, ADP-forming, beta subunit	13	-47414792	8803	13q12.2-q13.3	Hs.546323
KLF10	Kruppel-like factor 10	8	-207460384	7071	8q22.2	Hs.435001
PPP1R3C	protein phosphatase 1, regulatory (inhibitor) subunit 3C	10	-93378180	5507	10q23-q24	Hs.303090
SAR1B	SAR1 gene homolog B (S. cerevisiae)	5	-133970018	51128	5q31.1	Hs.432984
GENX-3414	NA	4	77446832	8987	4q24-q25	Hs.109590
FABP3	fatty acid binding protein 3, muscle and heart (mammary-derived growth inhibitor)	1	-31610686	2170	1p33-p32	Hs.584756
ACSL1	acyl-CoA synthetase long-chain family member 1	4	-185913743	2180	4q34-q35	Hs.406678
APOBEC2	apolipoprotein B mRNA editing enzyme, catalytic polypeptide-like 2	6	41128990	10930	6p21	Hs.555915
LOC653816	NA	6	NA	653816	6p12.3	Hs.485489
FLJ22028	NA	12	21481877	79912	12p12.1	Hs.46835
POPDC3	popeye domain containing 3	6	-105712469	64208	6q21	Hs.458336
CACNA1S	calcium channel, voltage-dependent, L type, alpha 1S subunit	1	-199275264	779	1q32	Hs.1294
ACHE	acetylcholinesterase (Yt blood group)	7	-100325551	43	7q22	Hs.154495
CUTC	cutC copper transporter homolog (E. coli)	10	101481992	51076	10q24.2	Hs.16606
SUCLG2	succinate-CoA ligase, GDP-forming, beta subunit	3	-67507835	8801	3p14.1	Hs.186512
DOC1	NA	3	-202084140	11259	3q12.1	Hs.104672
TOB1	transducer of ERBB2, 1	17	-46294587	10140	17q21	Hs.531550 Hs.606234
POPDC2	popeye domain containing 2	3	-120843597	64091	3q13.33	Hs.534383
SAMD4A	sterile alpha motif domain containing 4A	14	54104386	23034	14q22.2	Hs.98259
CAP2	CAP, adenylate cyclase-associated protein, 2 (yeast)	6	17501714	10486	6p22.3	Hs.132902
MYH4	myosin, heavy polypeptide 4, skeletal muscle	17	-10287334	4622	17p13.1	Hs.272207
CMKOR1	chemokine orphan receptor 1	2	237143181	57007	2q37.3	Hs.471751
IMPA2	inositol(myo)-1(or 4)-monophosphatase 2	18	11971454	3613	18p11.2	Hs.367992
G0S2	G0/G1switch 2	1	207915387	50486	1q32.2-q41	Hs.432132
RHOQ	ras homolog gene family, member Q	2	46623378	23433	2p21	Hs.643481
LPL	lipoprotein lipase	8	19841057	4023	8p22	Hs.180878
RTN2	reticulon 2	19	-50680390	6253	19q13.32	Hs.47517

NDUFB7	NADH dehydrogenase (ubiquinone) 1 beta subcomplex, 7, 18kDa	19	-14537891	4713	19p13.12-p13.11	Hs.532853
DMN	desmuslin	15	97462808	23336	15q26.3	Hs.207106
HOXC10	homeobox C10	12	52665212	3226	12q13.3	Hs.44276
PKIA	protein kinase (cAMP-dependent, catalytic) inhibitor alpha	8	79590890	5569	8q21.12	Hs.433700
PDZRN3	PDZ domain containing RING finger 3	3	-73514341	23024	3p13	Hs.434900
TACC2	transforming, acidic coiled-coil containing protein 2	10	123913094, 123738678	10579	10q26	Hs.501252 Hs.643068
FKBP3	FK506 binding protein 3, 25kDa	14	-44654858	2287	14q21.3	Hs.509226
UBE2G1	ubiquitin-conjugating enzyme E2G 1 (UBC7 homolog, yeast)	17	-4119262	7326	1q42|17p13.2	Hs.462035
SGCG	sarcoglycan, gamma (35kDa dystrophin-associated glycoprotein)	13	22653090	6445	13q12	Hs.37167
USP13	ubiquitin specific peptidase 13 (isopeptidase T-3)	3	180853634	8975	3q26.2-q26.3	Hs.591319
RRAD	Ras-related associated with diabetes	16	-65513089	6236	16q22	Hs.1027
TSPAN8	tetraspanin 8	12	-69805143	7103	12q14.1-q21.1	Hs.170563
NAT13	N-acetyltransferase 13	3	-114920530	80218	3q13.2	Hs.269528
CYB5R1	cytochrome b5 reductase 1	1	-201197626	51706	1p36.13-q41	Hs.334832
CRYAB	crystallin, alpha B	11	-111284559	1410	11q22.3-q23.1	Hs.408767
ZFP106	zinc finger protein 106 homolog (mouse)	15	-40492313	64397	15q15.1	Hs.511143
CDH5	cadherin 5, type 2, VE-cadherin (vascular epithelium)	16	64958063	1003	16q22.1	Hs.76206
TMEM14B	transmembrane protein 14B	6	10856035	81853	6p25.1-p23	Hs.273077
CXCL14	chemokine (C-X-C motif) ligand 14	5	-134934274	9547	5q31	Hs.483444

3.2 GO: Biological Process

GO ID	genes present in chip	genes present in tissues	p-value	GO term
GO:0006936	132	31	2.66E-32	muscle contraction
GO:0006941	29	16	4.51E-24	striated muscle contraction
GO:0006937	22	8	1.04E-10	regulation of muscle contraction
GO:0007517	94	11	1.56E-08	muscle development
GO:0050874	1531	40	6.85E-07	organismal physiological process
GO:0006942	7	4	7.09E-07	regulation of striated muscle contraction
GO:0051239	133	10	4.59E-06	regulation of organismal physiological process
GO:0030239	4	3	6.97E-06	myofibril assembly
GO:0048627	7	3	5.94E-05	myoblast development
GO:0048628	7	3	5.94E-05	myoblast maturation
GO:0007519	39	5	0.000101243	myogenesis
GO:0015980	132	8	0.000197561	energy derivation by oxidation of organic compounds
GO:0006091	446	15	0.000312089	generation of precursor metabolites and energy
GO:0006104	3	2	0.000437466	succinyl-CoA metabolism
GO:0045445	15	3	0.000718872	myoblast differentiation
GO:0042692	19	3	0.001477624	muscle cell differentiation
GO:0048469	21	3	0.001992527	cell maturation
GO:0005975	355	11	0.003880168	carbohydrate metabolism
GO:0005977	27	3	0.004155905	glycogen metabolism
GO:0006092	88	5	0.004298826	main pathways of carbohydrate metabolism
GO:0006073	28	3	0.004613766	glucan metabolism
GO:0044262	265	9	0.004936199	cellular carbohydrate metabolism
GO:0006112	34	3	0.007994279	energy reserve metabolism
GO:0015669	12	2	0.008958227	gas transport
GO:0015671	12	2	0.008958227	oxygen transport
GO:0006816	68	4	0.009332212	calcium ion transport
GO:0015888	1	1	0.012178919	thiamin transport
GO:0006938	1	1	0.012178919	sarcomere alignment
GO:0007525	1	1	0.012178919	somatic muscle development
GO:0030240	1	1	0.012178919	muscle thin filament assembly
GO:0006781	1	1	0.012178919	succinyl-CoA pathway
GO:0048468	40	3	0.012522708	cell development
GO:0006635	15	2	0.01391697	fatty acid beta-oxidation
GO:0044264	42	3	0.014298325	cellular polysaccharide metabolism
GO:0006006	79	4	0.015557476	glucose metabolism
GO:0005976	44	3	0.016209634	polysaccharide metabolism
GO:0006631	124	5	0.017535316	fatty acid metabolism
GO:0006100	20	2	0.024208648	tricarboxylic acid cycle intermediate metabolism
GO:0006581	2	1	0.024210845	acetylcholine catabolism
GO:0046502	2	1	0.024210845	uroporphyrinogen III metabolism
GO:0001507	2	1	0.024210845	acetylcholine catabolism in synaptic cleft
GO:0006780	2	1	0.024210845	uroporphyrinogen III biosynthesis

GO:0019395	22	2	0.028973223	fatty acid oxidation
GO:0006099	22	2	0.028973223	tricarboxylic acid cycle
GO:0046356	22	2	0.028973223	acetyl-CoA catabolism
GO:0008016	23	2	0.03148432	regulation of heart contraction rate
GO:0009109	23	2	0.03148432	coenzyme catabolism
GO:0015674	99	4	0.032452641	di-, tri-valent inorganic cation transport
GO:0008291	3	1	0.036097534	acetylcholine metabolism
GO:0008015	103	4	0.036751003	circulation
GO:0006084	27	2	0.042332156	acetyl-CoA metabolism
GO:0051187	28	2	0.04523357	cofactor catabolism
GO:0015991	4	1	0.047840725	ATP hydrolysis coupled proton transport
GO:0042439	4	1	0.047840725	ethanolamine and derivative metabolism
GO:0045946	4	1	0.047840725	positive regulation of translation
GO:0051289	4	1	0.047840725	protein homotetramerization
GO:0019318	113	4	0.048871042	hexose metabolism
GO:0009060	30	2	0.051248374	aerobic respiration
GO:0005996	115	4	0.05153056	monosaccharide metabolism
GO:0045333	32	2	0.057531788	cellular respiration
GO:0030641	5	1	0.059442134	hydrogen ion homeostasis
GO:0042402	5	1	0.059442134	biogenic amine catabolism
GO:0042135	5	1	0.059442134	neurotransmitter catabolism
GO:0009887	807	15	0.064271982	organogenesis
GO:0015988	6	1	0.070903458	energy coupled proton transport, against electrochemical gradient
GO:0042219	6	1	0.070903458	amino acid derivative catabolism
GO:0007010	305	7	0.078361035	cytoskeleton organization and biogenesis
GO:0006986	40	2	0.085035296	response to unfolded protein
GO:0030029	139	4	0.089409253	actin filament-based process
GO:0006118	256	6	0.092045182	electron transport
GO:0006096	42	2	0.092429772	glycolysis
GO:0030004	8	1	0.093412532	monovalent inorganic cation homeostasis
GO:0030317	8	1	0.093412532	sperm motility
GO:0050714	9	1	0.104463576	positive regulation of protein secretion
GO:0007271	9	1	0.104463576	synaptic transmission, cholinergic
GO:0042982	9	1	0.104463576	amyloid precursor protein metabolism
GO:0048513	872	15	0.107188886	organ development
GO:0000004	465	9	0.11301754	biological process unknown
GO:0050435	10	1	0.115381121	beta-amyloid metabolism
GO:0005978	10	1	0.115381121	glycogen biosynthesis
GO:0009250	10	1	0.115381121	glucan biosynthesis
GO:0051262	10	1	0.115381121	protein tetramerization
GO:0009167	11	1	0.126166764	purine ribonucleoside monophosphate metabolism
GO:0009168	11	1	0.126166764	purine ribonucleoside monophosphate biosynthesis
GO:0009126	11	1	0.126166764	purine nucleoside monophosphate metabolism
GO:0009127	11	1	0.126166764	purine nucleoside monophosphate biosynthesis
GO:0007270	11	1	0.126166764	nerve-nerve synaptic transmission
GO:0051260	11	1	0.126166764	protein homooligomerization

GO:0006007	51	2	0.127749769	glucose catabolism
GO:0009653	1051	17	0.135313924	morphogenesis
GO:0008206	12	1	0.136822086	bile acid metabolism
GO:0050708	12	1	0.136822086	regulation of protein secretion
GO:0051180	12	1	0.136822086	vitamin transport
GO:0006414	12	1	0.136822086	translational elongation
GO:0051047	13	1	0.147348646	positive regulation of secretion
GO:0006000	13	1	0.147348646	fructose metabolism
GO:0006779	13	1	0.147348646	porphyrin biosynthesis
GO:0019752	359	7	0.14759471	carboxylic acid metabolism
GO:0006082	361	7	0.150598095	organic acid metabolism
GO:0006457	169	4	0.150772909	protein folding
GO:0015992	57	2	0.152780829	proton transport
GO:0006818	58	2	0.157043132	hydrogen transport
GO:0030048	14	1	0.157747986	actin filament-based movement
GO:0019320	59	2	0.161328015	hexose catabolism
GO:0046365	59	2	0.161328015	monosaccharide catabolism
GO:0046164	60	2	0.165634314	alcohol catabolism
GO:0043284	17	1	0.188197834	biopolymer biosynthesis
GO:0009161	17	1	0.188197834	ribonucleoside monophosphate metabolism
GO:0009156	17	1	0.188197834	ribonucleoside monophosphate biosynthesis
GO:0007416	17	1	0.188197834	synaptogenesis
GO:0000271	17	1	0.188197834	polysaccharide biosynthesis
GO:0006094	17	1	0.188197834	gluconeogenesis
GO:0046777	17	1	0.188197834	autophosphorylation
GO:0031281	18	1	0.198103351	positive regulation of cyclase activity
GO:0045762	18	1	0.198103351	positive regulation of adenylate cyclase activity
GO:0050808	18	1	0.198103351	synapse organization and biogenesis
GO:0009123	18	1	0.198103351	nucleoside monophosphate metabolism
GO:0009124	18	1	0.198103351	nucleoside monophosphate biosynthesis
GO:0007190	18	1	0.198103351	adenylate cyclase activation
GO:0042133	18	1	0.198103351	neurotransmitter metabolism
GO:0051349	18	1	0.198103351	positive regulation of lyase activity
GO:0006090	18	1	0.198103351	pyruvate metabolism
GO:0016540	18	1	0.198103351	protein autoprocessing
GO:0006778	18	1	0.198103351	porphyrin metabolism
GO:0030036	126	3	0.198280425	actin cytoskeleton organization and biogenesis
GO:0016051	69	2	0.205174005	carbohydrate biosynthesis
GO:0007163	21	1	0.227106944	establishment and/or maintenance of cell polarity
GO:0030001	269	5	0.230205596	metal ion transport
GO:0030154	409	7	0.230598198	cell differentiation
GO:0019319	22	1	0.236541883	hexose biosynthesis
GO:0007189	22	1	0.236541883	G-protein signaling, adenylate cyclase activating pathway
GO:0046364	22	1	0.236541883	monosaccharide biosynthesis
GO:0006471	22	1	0.236541883	protein amino acid ADP-ribosylation
GO:0046165	22	1	0.236541883	alcohol biosynthesis

GO:0008285	139	3	0.239729731	negative regulation of cell proliferation
GO:0007015	23	1	0.245862681	actin filament organization
GO:0016052	79	2	0.250140416	carbohydrate catabolism
GO:0044275	79	2	0.250140416	cellular carbohydrate catabolism
GO:0051046	24	1	0.255070707	regulation of secretion
GO:0051259	24	1	0.255070707	protein oligomerization
GO:0006066	213	4	0.261064374	alcohol metabolism
GO:0009306	26	1	0.273153834	protein secretion
GO:0050954	86	2	0.281829904	sensory perception of mechanical stimulus
GO:0050982	86	2	0.281829904	detection of mechanical stimulus
GO:0007605	86	2	0.281829904	perception of sound
GO:0051348	28	1	0.290801888	negative regulation of transferase activity
GO:0006469	28	1	0.290801888	negative regulation of protein kinase activity
GO:0007155	518	8	0.295798304	cell adhesion
GO:0007264	227	4	0.299156365	small GTPase mediated signal transduction
GO:0007179	29	1	0.299466014	transforming growth factor beta receptor signaling pathway
GO:0006730	30	1	0.308025243	one-carbon compound metabolism
GO:0044255	383	6	0.323686345	cellular lipid metabolism
GO:0031279	32	1	0.324834028	regulation of cyclase activity
GO:0045761	32	1	0.324834028	regulation of adenylate cyclase activity
GO:0051339	32	1	0.324834028	regulation of lyase activity
GO:0009059	464	7	0.335913753	macromolecule biosynthesis
GO:0006629	469	7	0.345908162	lipid metabolism
GO:0043062	35	1	0.349291457	extracellular structure organization and biogenesis
GO:0006576	35	1	0.349291457	biogenic amine metabolism
GO:0030198	35	1	0.349291457	extracellular matrix organization and biogenesis
GO:0030832	35	1	0.349291457	regulation of actin filament length
GO:0016485	37	1	0.365106573	protein processing
GO:0006511	105	2	0.366806364	ubiquitin-dependent protein catabolism
GO:0019941	105	2	0.366806364	modification-dependent protein catabolism
GO:0007178	38	1	0.372870695	transmembrane receptor protein serine/threonine kinase signaling pathway
GO:0006512	255	4	0.376849232	ubiquitin cycle
GO:0009582	109	2	0.38427097	detection of abiotic stimulus
GO:0051234	1841	24	0.390457294	establishment of localization
GO:0009100	111	2	0.392927554	glycoprotein metabolism
GO:0006575	41	1	0.395602999	amino acid derivative metabolism
GO:0001505	41	1	0.395602999	regulation of neurotransmitter levels
GO:0001501	113	2	0.40153012	skeletal development
GO:0042127	265	4	0.404592389	regulation of cell proliferation
GO:0051179	1857	24	0.408503726	localization
GO:0006812	346	5	0.413883536	cation transport
GO:0006979	44	1	0.417518518	response to oxidative stress
GO:0046483	44	1	0.417518518	heterocycle metabolism
GO:0009310	45	1	0.424647241	amine catabolism
GO:0043086	46	1	0.431689502	negative regulation of enzyme activity
GO:0006732	121	2	0.435353177	coenzyme metabolism

GO:0006810	1802	23	0.438050968	transport
GO:0044270	47	1	0.438646341	nitrogen compound catabolism
GO:0006811	515	7	0.438787879	ion transport
GO:0043037	122	2	0.439510626	translation
GO:0044260	2298	29	0.448163832	cellular macromolecule metabolism
GO:0007586	49	1	0.452307847	digestion
GO:0006820	128	2	0.464102502	anion transport
GO:0043170	2405	30	0.475275344	macromolecule metabolism
GO:0008632	53	1	0.478650154	apoptotic program
GO:0006817	57	1	0.503736445	phosphate transport
GO:0009152	58	1	0.509818784	purine ribonucleotide biosynthesis
GO:0006888	58	1	0.509818784	ER to Golgi transport
GO:0007188	59	1	0.515827245	G-protein signaling, coupled to cAMP nucleotide second messenger
GO:0007582	7846	96	0.52001031	physiological process
GO:0009150	60	1	0.521762719	purine ribonucleotide metabolism
GO:0019933	60	1	0.521762719	cAMP-mediated signaling
GO:0051186	143	2	0.522733684	cofactor metabolism
GO:0006800	61	1	0.527626083	oxygen and reactive oxygen species metabolism
GO:0000122	61	1	0.527626083	negative regulation of transcription from RNA polymerase II promoter
GO:0006164	61	1	0.527626083	purine nucleotide biosynthesis
GO:0006163	63	1	0.539139944	purine nucleotide metabolism
GO:0006445	64	1	0.544792147	regulation of translation
GO:0050953	149	2	0.544972436	sensory perception of light
GO:0007601	149	2	0.544972436	visual perception
GO:0001525	65	1	0.550375651	angiogenesis
GO:0009260	65	1	0.550375651	ribonucleotide biosynthesis
GO:0006412	407	5	0.556822401	protein biosynthesis
GO:0016042	67	1	0.561339862	lipid catabolism
GO:0040007	154	2	0.562954174	growth
GO:0001568	68	1	0.566722195	blood vessel development
GO:0048514	68	1	0.566722195	blood vessel morphogenesis
GO:0009259	68	1	0.566722195	ribonucleotide metabolism
GO:0001944	69	1	0.572039081	vasculature development
GO:0009056	756	9	0.579076707	catabolism
GO:0044249	763	9	0.59049684	cellular biosynthesis
GO:0007167	165	2	0.600723302	enzyme linked receptor protein signaling pathway
GO:0007596	78	1	0.61707296	blood coagulation
GO:0050817	79	1	0.621777224	coagulation
GO:0006814	80	1	0.626424214	sodium ion transport
GO:0044248	700	8	0.626825154	cellular catabolism
GO:0007599	83	1	0.640028416	hemostasis
GO:0042060	85	1	0.648823969	wound healing
GO:0051188	85	1	0.648823969	cofactor biosynthesis
GO:0048193	86	1	0.653141546	Golgi vesicle transport
GO:0000902	183	2	0.657189493	cellular morphogenesis
GO:0006486	92	1	0.677964243	protein amino acid glycosylation

GO:0043413	93	1	0.681926652	biopolymer glycosylation
GO:0007600	287	3	0.684447783	sensory perception
GO:0050878	94	1	0.685840744	regulation of body fluids
GO:0007187	94	1	0.685840744	G-protein signaling, coupled to cyclic nucleotide second messenger
GO:0006461	194	2	0.688482644	protein complex assembly
GO:0008283	473	5	0.690021942	cell proliferation
GO:0009101	96	1	0.693526306	glycoprotein biosynthesis
GO:0045892	97	1	0.697298923	negative regulation of transcription, DNA-dependent
GO:0019935	98	1	0.701025518	cyclic-nucleotide-mediated signaling
GO:0030003	98	1	0.701025518	cation homeostasis
GO:0006935	101	1	0.711934692	chemotaxis
GO:0042330	101	1	0.711934692	taxis
GO:0007275	1543	17	0.714183097	development
GO:0009165	103	1	0.718987384	nucleotide biosynthesis
GO:0006873	103	1	0.718987384	cell ion homeostasis
GO:0046903	208	2	0.724911349	secretion
GO:0007156	105	1	0.725868937	homophilic cell adhesion
GO:0015698	106	1	0.729246826	inorganic anion transport
GO:0009058	859	9	0.730792736	biosynthesis
GO:0043085	107	1	0.732583466	positive regulation of enzyme activity
GO:0006417	107	1	0.732583466	regulation of protein biosynthesis
GO:0050790	214	2	0.739401038	regulation of enzyme activity
GO:0009581	313	3	0.739924812	detection of external stimulus
GO:0006813	111	1	0.745527429	potassium ion transport
GO:0031326	111	1	0.745527429	regulation of cellular biosynthesis
GO:0050801	111	1	0.745527429	ion homeostasis
GO:0006996	693	7	0.749538305	organelle organization and biogenesis
GO:0009628	318	3	0.749671273	response to abiotic stimulus
GO:0006897	115	1	0.757850276	endocytosis
GO:0015672	223	2	0.75992423	monovalent inorganic cation transport
GO:0045859	116	1	0.760837432	regulation of protein kinase activity
GO:0007169	116	1	0.760837432	transmembrane receptor protein tyrosine kinase signaling pathway
GO:0009889	116	1	0.760837432	regulation of biosynthesis
GO:0051338	117	1	0.76378807	regulation of transferase activity
GO:0008284	121	1	0.775234201	positive regulation of cell proliferation
GO:0019725	122	1	0.778008776	cell homeostasis
GO:0008361	123	1	0.780749409	regulation of cell size
GO:0016049	123	1	0.780749409	cell growth
GO:0009611	341	3	0.790829299	response to wounding
GO:0006357	240	2	0.794928397	regulation of transcription from RNA polymerase II promoter
GO:0008202	132	1	0.803953972	steroid metabolism
GO:0007067	135	1	0.811134418	mitosis
GO:0000087	137	1	0.815775968	M phase of mitotic cell cycle
GO:0016043	842	8	0.816641679	cell organization and biogenesis
GO:0006260	140	1	0.822527165	DNA replication
GO:0044265	563	5	0.824042038	cellular macromolecule catabolism

GO:0009117	143	1	0.829033119	nucleotide metabolism
GO:0045786	145	1	0.83323855	negative regulation of progression through cell cycle
GO:0050877	475	4	0.837611985	neurophysiological process
GO:0009057	575	5	0.837829028	macromolecule catabolism
GO:0016481	151	1	0.845249529	negative regulation of transcription
GO:0042592	157	1	0.856402717	homeostasis
GO:0045934	159	1	0.85994045	negative regulation of nucleobase, nucleoside, nucleotide and nucleic acid metabolism
GO:0045045	161	1	0.863391799	secretory pathway
GO:0016192	296	2	0.880543779	vesicle-mediated transport
GO:0016337	172	1	0.880921035	cell-cell adhesion
GO:0044267	2109	21	0.881964858	cellular protein metabolism
GO:0000279	173	1	0.882399442	M phase
GO:0051246	174	1	0.883859659	regulation of protein metabolism
GO:0019932	176	1	0.886726407	second-messenger-mediated signaling
GO:0006397	180	1	0.892251202	mRNA processing
GO:0019538	2129	21	0.892276129	protein metabolism
GO:0031324	183	1	0.89621878	negative regulation of cellular metabolism
GO:0042221	183	1	0.89621878	response to chemical substance
GO:0006954	185	1	0.898783051	inflammatory response
GO:0007268	192	1	0.907273383	synaptic transmission
GO:0019226	198	1	0.913986425	transmission of nerve impulse
GO:0006508	451	3	0.917902244	proteolysis and peptidolysis
GO:0044257	451	3	0.917902244	cellular protein catabolism
GO:0000278	204	1	0.920217568	mitotic cell cycle
GO:0006928	205	1	0.921211685	cell motility
GO:0040011	205	1	0.921211685	locomotion
GO:0016071	205	1	0.921211685	mRNA metabolism
GO:0007626	209	1	0.925066889	locomotory behavior
GO:0030163	463	3	0.926346912	protein catabolism
GO:0007267	465	3	0.927675328	cell-cell signaling
GO:0048519	688	5	0.929240358	negative regulation of biological process
GO:0051243	583	4	0.931042006	negative regulation of cellular physiological process
GO:0006519	216	1	0.931368933	amino acid and derivative metabolism
GO:0009892	217	1	0.932225265	negative regulation of metabolism
GO:0043285	474	3	0.933388245	biopolymer catabolism
GO:0043118	602	4	0.941269131	negative regulation of physiological process
GO:0007399	385	2	0.951995072	neurogenesis
GO:0000074	394	2	0.956351287	regulation of progression through cell cycle
GO:0048523	640	4	0.957731279	negative regulation of cellular process
GO:0006886	257	1	0.959032533	intracellular protein transport
GO:0006464	1087	8	0.962551655	protein modification
GO:0006950	881	6	0.963953283	response to stress
GO:0009308	269	1	0.964790901	amine metabolism
GO:0007610	269	1	0.964790901	behavior
GO:0006366	426	2	0.968994845	transcription from RNA polymerase II promoter
GO:0043119	428	2	0.969656286	positive regulation of physiological process

GO:0048518	565	3	0.971928852	positive regulation of biological process
GO:0006807	291	1	0.973342786	nitrogen compound metabolism
GO:0006468	444	2	0.974483697	protein amino acid phosphorylation
GO:0045184	459	2	0.97833528	establishment of protein localization
GO:0043412	1160	8	0.978362743	biopolymer modification
GO:0007049	601	3	0.980344475	cell cycle
GO:0009605	963	6	0.98160846	response to external stimulus
GO:0008104	474	2	0.981625841	protein localization
GO:0009607	857	5	0.982798038	response to biotic stimulus
GO:0006396	326	1	0.982902332	RNA processing
GO:0007186	491	2	0.984774806	G-protein coupled receptor protein signaling pathway
GO:0007242	879	5	0.985876029	intracellular signaling cascade
GO:0050791	2407	20	0.986168035	regulation of physiological process
GO:0006793	641	3	0.986884355	phosphorus metabolism
GO:0006796	641	3	0.986884355	phosphate metabolism
GO:0016310	522	2	0.989229837	phosphorylation
GO:0008152	5262	52	0.992484069	metabolism
GO:0016070	391	1	0.992541426	RNA metabolism
GO:0050789	2689	22	0.992658006	regulation of biological process
GO:0051242	411	1	0.994228998	positive regulation of cellular physiological process
GO:0050896	1578	10	0.995829954	response to stimulus
GO:0044237	5026	48	0.995832226	cellular metabolism
GO:0015031	439	1	0.995974248	protein transport
GO:0046907	448	1	0.996415196	intracellular transport
GO:0009613	458	1	0.996849182	response to pest, pathogen or parasite
GO:0043207	463	1	0.997046224	response to external biotic stimulus
GO:0006915	471	1	0.997336393	apoptosis
GO:0012501	473	1	0.997404406	programmed cell death
GO:0048522	482	1	0.997689779	positive regulation of cellular process
GO:0050875	7018	73	0.997884673	cellular physiological process
GO:0006259	489	1	0.997890042	DNA metabolism
GO:0007166	952	4	0.997943724	cell surface receptor linked signal transduction
GO:0008219	498	1	0.998122434	cell death
GO:0016265	501	1	0.998194114	death
GO:0006955	684	2	0.998341485	immune response
GO:0007154	2730	20	0.99866052	cell communication
GO:0006952	751	2	0.999254959	defense response
GO:0043283	2240	14	0.999467369	biopolymer metabolism
GO:0044238	4820	42	0.999536112	primary metabolism
GO:0007165	2168	12	0.999857239	signal transduction
GO:0006355	1359	4	0.999978936	regulation of transcription, DNA-dependent
GO:0009987	8200	86	0.99998381	cellular process
GO:0006351	1407	4	0.999988188	transcription, DNA-dependent
GO:0031323	1566	5	0.999990142	regulation of cellular metabolism
GO:0045449	1433	4	0.999991392	regulation of transcription
GO:0019219	1458	4	0.999993663	regulation of nucleobase, nucleoside, nucleotide and nucleic acid metabolism

GO:0019222	1629	5	0.99999537	regulation of metabolism
GO:0006350	1499	4	0.999996181	transcription
GO:0051244	2319	9	0.999999372	regulation of cellular physiological process
GO:0050794	2488	9	0.999999922	regulation of cellular process
GO:0006139	2328	7	0.999999978	nucleobase, nucleoside, nucleotide and nucleic acid metabolism
GO:0008150	9032	110	1	biological_process

3.3 GO: Molecular Function

GO ID	genes present in chip	genes present in tissues	p-value	GO term
GO:0008307	33	14	9.7194622158404e-19	structural constituent of muscle
GO:0008092	279	23	6.47619605216499e-13	cytoskeletal protein binding
GO:0003779	189	18	2.43289663510594e-11	actin binding
GO:0005198	488	24	9.1389445245372e-09	structural molecule activity
GO:0000146	15	5	8.5350441967502e-07	microfilament motor activity
GO:0003774	98	8	3.41774404712751e-05	motor activity
GO:0005523	9	3	0.000161993126060522	tropomyosin binding
GO:0051373	2	2	0.00016218210406945	FATZ binding
GO:0016877	12	3	0.000412513870866412	ligase activity, forming carbon-sulfur bonds
GO:0004111	3	2	0.000482465831552447	creatine kinase activity
GO:0004774	3	2	0.000482465831552447	succinate-CoA ligase activity
GO:0005516	112	7	0.000561917371370415	calmodulin binding
GO:0016405	4	2	0.000956847066418487	CoA-ligase activity
GO:0017022	5	2	0.00158139679746368	myosin binding
GO:0016878	5	2	0.00158139679746368	acid-thiol ligase activity
GO:0016775	10	2	0.00682426013020758	phosphotransferase activity, nitrogenous group as acceptor
GO:0005344	12	2	0.0098431334844281	oxygen transporter activity
GO:0015926	13	2	0.0115361986719228	glucosidase activity
GO:0005199	1	1	0.0127869196101035	structural constituent of cell wall
GO:0048244	1	1	0.0127869196101035	phytanoyl-CoA dioxygenase activity
GO:0004903	1	1	0.0127869196101035	growth hormone receptor activity
GO:0042030	1	1	0.0127869196101035	ATPase inhibitor activity
GO:0051393	1	1	0.0127869196101035	alpha-actinin binding
GO:0003990	1	1	0.0127869196101035	acetylcholinesterase activity
GO:0051370	1	1	0.0127869196101035	ZASP binding
GO:0051374	1	1	0.0127869196101035	FATZ 1 binding
GO:0004133	1	1	0.0127869196101035	glycogen debranching enzyme activity
GO:0004134	1	1	0.0127869196101035	4-alpha-glucanotransferase activity
GO:0004135	1	1	0.0127869196101035	amylo-alpha-1,6-glucosidase activity
GO:0015283	1	1	0.0127869196101035	apoptogenic cytochrome c release channel activity
GO:0015234	1	1	0.0127869196101035	thiamin transporter activity
GO:0004775	1	1	0.0127869196101035	succinate-CoA ligase (ADP-forming) activity
GO:0005504	17	2	0.0194562252079265	fatty acid binding
GO:0019239	18	2	0.0217074213757965	deaminase activity
GO:0004862	2	1	0.0254116571161385	cAMP-dependent protein kinase inhibitor activity
GO:0004619	2	1	0.0254116571161385	phosphoglycerate mutase activity
GO:0008422	2	1	0.0254116571161385	beta-glucosidase activity
GO:0008184	2	1	0.0254116571161385	glycogen phosphorylase activity
GO:0042132	2	1	0.0254116571161385	fructose-bisphosphatase activity
GO:0008934	2	1	0.0254116571161385	inositol-1(or 4)-monophosphatase activity
GO:0000163	2	1	0.0254116571161385	protein phosphatase type 1 activity
GO:0004082	2	1	0.0254116571161385	bisphosphoglycerate mutase activity

GO:0004083	2	1	0.0254116571161385	bisphosphoglycerate phosphatase activity
GO:0042805	2	1	0.0254116571161385	actinin binding
GO:0030346	2	1	0.0254116571161385	protein phosphatase 2B binding
GO:0015482	2	1	0.0254116571161385	voltage-gated anion channel porin activity
GO:0004104	2	1	0.0254116571161385	cholinesterase activity
GO:0015266	2	1	0.0254116571161385	protein channel activity
GO:0004776	2	1	0.0254116571161385	succinate-CoA ligase (GDP-forming) activity
GO:0016814	21	2	0.0290636256508610	hydrolase activity, acting on carbon-nitrogen (but not peptide) bonds, in cyclic amidines
GO:0015002	21	2	0.0290636256508610	heme-copper terminal oxidase activity
GO:0016675	21	2	0.0290636256508610	oxidoreductase activity, acting on heme group of donors
GO:0016676	21	2	0.0290636256508610	oxidoreductase activity, acting on heme group of donors, oxygen as acceptor
GO:0004129	21	2	0.0290636256508610	cytochrome-c oxidase activity
GO:0015399	140	5	0.0335388275078125	primary active transporter activity
GO:0004865	3	1	0.0378762527584315	type 1 serine/threonine specific protein phosphatase inhibitor activity
GO:0004634	3	1	0.0378762527584315	phosphopyruvate hydratase activity
GO:0008308	3	1	0.0378762527584315	voltage-gated ion-selective channel activity
GO:0003876	3	1	0.0378762527584315	AMP deaminase activity
GO:0016416	3	1	0.0378762527584315	O-palmitoyltransferase activity
GO:0004095	3	1	0.0378762527584315	carnitine O-palmitoyltransferase activity
GO:0030371	3	1	0.0378762527584315	translation repressor activity
GO:0019842	59	3	0.0395064644816211	vitamin binding
GO:0004645	4	1	0.0501827213224353	phosphorylase activity
GO:0008517	4	1	0.0501827213224353	folic acid transporter activity
GO:0008518	4	1	0.0501827213224353	reduced folate carrier activity
GO:0003956	4	1	0.0501827213224353	NAD(P)+-protein-arginine ADP-ribosyltransferase activity
GO:0015270	4	1	0.0501827213224353	dihydropyridine-sensitive calcium channel activity
GO:0004740	4	1	0.0501827213224353	[pyruvate dehydrogenase (lipoamide)] kinase activity
GO:0016406	5	1	0.0623330524536778	carnitine O-acyltransferase activity
GO:0004465	5	1	0.0623330524536778	lipoprotein lipase activity
GO:0004126	5	1	0.0623330524536778	cytidine deaminase activity
GO:0015077	121	4	0.0692551210383427	monovalent inorganic cation transporter activity
GO:0016409	6	1	0.0743292109688465	palmitoyltransferase activity
GO:0019903	6	1	0.0743292109688465	protein phosphatase binding
GO:0004331	6	1	0.0743292109688465	fructose-2,6-bisphosphate 2-phosphatase activity
GO:0015278	6	1	0.0743292109688465	calcium-release channel activity
GO:0001540	7	1	0.0861731371630567	beta-amyloid binding
GO:0005246	7	1	0.0861731371630567	calcium channel regulator activity
GO:0019203	7	1	0.0861731371630567	carbohydrate phosphatase activity
GO:0005527	7	1	0.0861731371630567	macrolide binding
GO:0005528	7	1	0.0861731371630567	FK506 binding
GO:0051183	7	1	0.0861731371630567	vitamin transporter activity
GO:0016868	7	1	0.0861731371630567	intramolecular transferase activity, phosphotransferases
GO:0015662	40	2	0.092406091597048	ATPase activity, coupled to transmembrane movement of ions, phosphorylative mechanism
GO:0005070	40	2	0.092406091597048	SH3/SH2 adaptor activity
GO:0015645	8	1	0.0978667471133522	fatty-acid ligase activity
GO:0030170	8	1	0.0978667471133522	pyridoxal phosphate binding

GO:0004467	8	1	0.0978667471133522	long-chain-fatty-acid-CoA ligase activity
GO:0000287	251	6	0.102415173983092	magnesium ion binding
GO:0016836	43	2	0.104413953776989	hydro-lyase activity
GO:0005391	9	1	0.109411932978471	sodium:potassium-exchanging ATPase activity
GO:0005388	9	1	0.109411932978471	calcium-transporting ATPase activity
GO:0051184	9	1	0.109411932978471	cofactor transporter activity
GO:0003746	10	1	0.120810563294944	translation elongation factor activity
GO:0005542	10	1	0.120810563294944	folic acid binding
GO:0019902	10	1	0.120810563294944	phosphatase binding
GO:0005262	47	2	0.121033082166547	calcium channel activity
GO:0005509	646	12	0.122544909372385	calcium ion binding
GO:0004896	48	2	0.125285032000654	hematopoietin/interferon-class (D200-domain) cytokine receptor activity
GO:0005554	522	10	0.130675375053690	molecular function unknown
GO:0016866	11	1	0.132064483269555	intramolecular transferase activity
GO:0016835	50	2	0.133894071981500	carbon-oxygen lyase activity
GO:0031418	12	1	0.143175515068203	L-ascorbic acid binding
GO:0003995	12	1	0.143175515068203	acyl-CoA dehydrogenase activity
GO:0004198	12	1	0.143175515068203	calpain activity
GO:0042802	158	4	0.143888055244471	protein self binding
GO:0016874	221	5	0.152977212983957	ligase activity
GO:0015085	13	1	0.154145458101226	calcium ion transporter activity
GO:0016526	13	1	0.154145458101226	G-protein coupled receptor activity, unknown ligand
GO:0015288	13	1	0.154145458101226	porin activity
GO:0005525	226	5	0.163223759261995	GTP binding
GO:0003950	14	1	0.164976089305220	NAD+ ADP-ribosyltransferase activity
GO:0004091	14	1	0.164976089305220	carboxylesterase activity
GO:0004759	14	1	0.164976089305220	serine esterase activity
GO:0005319	58	2	0.169510665703076	lipid transporter activity
GO:0015078	112	3	0.172570731026886	hydrogen ion transporter activity
GO:0004553	59	2	0.174072216623127	hydrolase activity, hydrolyzing O-glycosyl compounds
GO:0016791	171	4	0.17550396795754	phosphoric monoester hydrolase activity
GO:0004864	15	1	0.175669163421391	protein phosphatase inhibitor activity
GO:0008144	15	1	0.175669163421391	drug binding
GO:0019001	235	5	0.182340570421887	guanyl nucleotide binding
GO:0004089	16	1	0.186226413270498	carbonate dehydratase activity
GO:0005212	17	1	0.196649550024408	structural constituent of eye lens
GO:0019212	17	1	0.196649550024408	phosphatase inhibitor activity
GO:0042166	17	1	0.196649550024408	acetylcholine binding
GO:0042803	64	2	0.197154413303071	protein homodimerization activity
GO:0008374	18	1	0.206940263474326	O-acyltransferase activity
GO:0042625	68	2	0.215879538620471	ATPase activity, coupled to transmembrane movement of ions
GO:0030674	69	2	0.220587727400958	protein binding, bridging
GO:0045182	72	2	0.234760665089374	translation regulator activity
GO:0016810	72	2	0.234760665089374	hydrolase activity, acting on carbon-nitrogen (but not peptide) bonds
GO:0004840	21	1	0.237034446742951	ubiquitin conjugating enzyme activity
GO:0046873	74	2	0.244240060065274	metal ion transporter activity

GO:0004601	22	1	0.246811946480069	peroxidase activity
GO:0043176	22	1	0.246811946480069	amine binding
GO:0005245	22	1	0.246811946480069	voltage-gated calcium channel activity
GO:0016684	22	1	0.246811946480069	oxidoreductase activity, acting on peroxide as acceptor
GO:0005386	398	7	0.247102620583334	carrier activity
GO:0016798	77	2	0.258487239303344	hydrolase activity, acting on glycosyl bonds
GO:0015405	77	2	0.258487239303344	P-P-bond-hydrolysis-driven transporter activity
GO:0008639	24	1	0.265995655216853	small protein conjugating enzyme activity
GO:0016789	80	2	0.272747859582237	carboxylic ester hydrolase activity
GO:0004197	80	2	0.272747859582237	cysteine-type endopeptidase activity
GO:0004860	25	1	0.275404978539701	protein kinase inhibitor activity
GO:0003755	25	1	0.275404978539701	peptidyl-prolyl cis-trans isomerase activity
GO:0005515	3709	51	0.281371692376581	protein binding
GO:0016247	26	1	0.284694658302013	channel regulator activity
GO:0016859	26	1	0.284694658302013	cis-trans isomerase activity
GO:0004437	26	1	0.284694658302013	inositol or phosphatidylinositol phosphatase activity
GO:0008324	421	7	0.29284251796392	cation transporter activity
GO:0004722	27	1	0.293866203408388	protein serine/threonine phosphatase activity
GO:0005200	86	2	0.301225864697346	structural constituent of cytoskeleton
GO:0008022	28	1	0.302921103890223	protein C-terminus binding
GO:0004842	87	2	0.305958290773212	ubiquitin-protein ligase activity
GO:0005489	152	3	0.307805125679979	electron transporter activity
GO:0019210	29	1	0.311860831139817	kinase inhibitor activity
GO:0016763	29	1	0.311860831139817	transferase activity, transferring pentosyl groups
GO:0015457	29	1	0.311860831139817	auxiliary transport protein activity
GO:0015082	30	1	0.320686838141598	di-, tri-valent inorganic cation transporter activity
GO:0016491	509	8	0.325924851913527	oxidoreductase activity
GO:0017076	1248	18	0.328800946682658	purine nucleotide binding
GO:0019825	31	1	0.329400559700501	oxygen binding
GO:0016209	31	1	0.329400559700501	antioxidant activity
GO:0048503	92	2	0.329522117670834	GPI anchor binding
GO:0016853	92	2	0.329522117670834	isomerase activity
GO:0042578	228	4	0.33342264416365	phosphoric ester hydrolase activity
GO:0005215	1030	15	0.336574135372999	transporter activity
GO:0008137	32	1	0.33800341266754	NADH dehydrogenase (ubiquinone) activity
GO:0050136	32	1	0.33800341266754	NADH dehydrogenase (quinone) activity
GO:0003954	32	1	0.33800341266754	NADH dehydrogenase activity
GO:0016788	446	7	0.344563353846654	hydrolase activity, acting on ester bonds
GO:0015075	523	8	0.353117510419356	ion transporter activity
GO:0016757	165	3	0.353437891780164	transferase activity, transferring glycosyl groups
GO:0008234	98	2	0.357507069718443	cysteine-type peptidase activity
GO:0019888	35	1	0.36316066387835	protein phosphatase regulator activity
GO:0016655	35	1	0.36316066387835	oxidoreductase activity, acting on NADH or NADPH, quinone or similar compound as acceptor
GO:0048037	100	2	0.366746736728848	cofactor binding
GO:0003735	100	2	0.366746736728848	structural constituent of ribosome
GO:0042626	100	2	0.366746736728848	ATPase activity, coupled to transmembrane movement of substances

GO:0046983	170	3	0.370931267270900	protein dimerization activity
GO:0019208	36	1	0.371333859650161	phosphatase regulator activity
GO:0015081	36	1	0.371333859650161	sodium ion transporter activity
GO:0016881	101	2	0.371347844150645	acid-amino acid ligase activity
GO:0016820	101	2	0.371347844150645	hydrolase activity, acting on acid anhydrides, catalyzing transmembrane movement of substances
GO:0016627	37	1	0.379403009478955	oxidoreductase activity, acting on the CH-CH group of donors
GO:0009055	104	2	0.385071133672517	electron carrier activity
GO:0008009	40	1	0.402999238371691	chemokine activity
GO:0042379	40	1	0.402999238371691	chemokine receptor binding
GO:0004221	41	1	0.410665178053688	ubiquitin thiolesterase activity
GO:0001664	42	1	0.418233477872367	G-protein-coupled receptor binding
GO:0019829	42	1	0.418233477872367	cation-transporting ATPase activity
GO:0004843	43	1	0.425705371303209	ubiquitin-specific protease activity
GO:0004857	187	3	0.429619857872178	enzyme inhibitor activity
GO:0050660	44	1	0.433082076367539	FAD binding
GO:0016758	115	2	0.434218265799139	transferase activity, transferring hexosyl groups
GO:0005253	46	1	0.447554717360917	anion channel activity
GO:0019887	47	1	0.454653013778185	protein kinase regulator activity
GO:0003924	121	2	0.460142111081258	GTPase activity
GO:0005507	49	1	0.468579349143399	copper ion binding
GO:0000166	1432	19	0.4691849824557	nucleotide binding
GO:0016790	51	1	0.482152893602383	thiolester hydrolase activity
GO:0051082	127	2	0.485372709272747	unfolded protein binding
GO:0016879	127	2	0.485372709272747	ligase activity, forming carbon-nitrogen bonds
GO:0016829	127	2	0.485372709272747	lyase activity
GO:0035091	132	2	0.505839004742294	phosphoinositide binding
GO:0016651	57	1	0.520844333466844	oxidoreductase activity, acting on NADH or NADPH
GO:0019207	58	1	0.527008082024103	kinase regulator activity
GO:0008289	298	4	0.532329800147347	lipid binding
GO:0016298	59	1	0.533093183151705	lipase activity
GO:0008201	60	1	0.539100632176752	heparin binding
GO:0008135	62	1	0.55088649291924	translation factor activity, nucleic acid binding
GO:0016563	62	1	0.55088649291924	transcriptional activator activity
GO:0030554	1028	13	0.560668861136185	adenyl nucleotide binding
GO:0042623	229	3	0.564472930679477	ATPase activity, coupled
GO:0008565	67	1	0.579059539261692	protein transporter activity
GO:0005488	7228	92	0.58450787790724	binding
GO:0043167	2556	32	0.590411186748258	ion binding
GO:0005524	970	12	0.592996495515997	ATP binding
GO:0031402	70	1	0.595114493295774	sodium ion binding
GO:0017111	410	5	0.606866389337228	nucleoside-triphosphatase activity
GO:0046872	2495	31	0.608709565021427	metal ion binding
GO:0046906	74	1	0.615580037083381	tetrapyrrole binding
GO:0020037	74	1	0.615580037083381	heme binding
GO:0005216	252	3	0.629731347582223	ion channel activity
GO:0005539	77	1	0.630252776688432	glycosaminoglycan binding

GO:0042165	78	1	0.635019171578965	neurotransmitter binding
GO:0016887	255	3	0.637735884696426	ATPase activity
GO:0005543	169	2	0.640247329309945	phospholipid binding
GO:0030247	80	1	0.644369895732884	polysaccharide binding
GO:0016462	430	5	0.649113539281051	pyrophosphatase activity
GO:0016818	432	5	0.653180801380777	hydrolase activity, acting on acid anhydrides, in phosphorus-containing anhydrides
GO:0016817	433	5	0.655203423905927	hydrolase activity, acting on acid anhydrides
GO:0016787	1424	17	0.660061976760636	hydrolase activity
GO:0015276	84	1	0.662364386072592	ligand-gated ion channel activity
GO:0043169	2313	28	0.66519909778927	cation binding
GO:0030955	86	1	0.671019984923082	potassium ion binding
GO:0005125	181	2	0.677257811403306	cytokine activity
GO:0030246	181	2	0.677257811403306	carbohydrate binding
GO:0001871	88	1	0.679455462028864	pattern binding
GO:0050662	88	1	0.679455462028864	coenzyme binding
GO:0015268	274	3	0.685637823475177	alpha-type channel activity
GO:0005261	188	2	0.697395597709967	cation channel activity
GO:0008509	95	1	0.707325098058198	anion transporter activity
GO:0015267	284	3	0.708907048971442	channel or pore class transporter activity
GO:0008415	98	1	0.718520580125732	acyltransferase activity
GO:0016747	98	1	0.718520580125732	transferase activity, transferring groups other than amino-acyl groups
GO:0016746	104	1	0.739652912256552	transferase activity, transferring acyl groups
GO:0005506	212	2	0.758720660366333	iron ion binding
GO:0003824	3409	40	0.780572236650871	catalytic activity
GO:0004721	118	1	0.783038596869753	phosphoprotein phosphatase activity
GO:0005244	124	1	0.799360642355027	voltage-gated ion channel activity
GO:0031420	146	1	0.84944811161193	alkali metal ion binding
GO:0015290	149	1	0.85523792204024	electrochemical potential-driven transporter activity
GO:0015291	149	1	0.85523792204024	porter activity
GO:0019899	151	1	0.85897454800768	enzyme binding
GO:0004713	161	1	0.876270170887635	protein-tyrosine kinase activity
GO:0004175	288	2	0.887541304221147	endopeptidase activity
GO:0030234	509	4	0.896720592278973	enzyme regulator activity
GO:0003702	197	1	0.922837689914937	RNA polymerase II transcription factor activity
GO:0008270	1287	11	0.950496405257202	zinc ion binding
GO:0016301	609	4	0.95702034738254	kinase activity
GO:0046914	1596	14	0.959646740880575	transition metal ion binding
GO:0008233	399	2	0.966294961287815	peptidase activity
GO:0001584	282	1	0.974878752788088	rhodopsin-like receptor activity
GO:0003723	436	2	0.977837331723599	RNA binding
GO:0016772	708	4	0.983224186958133	transferase activity, transferring phosphorus-containing groups
GO:0004674	326	1	0.986004557097129	protein serine/threonine kinase activity
GO:0004872	1060	7	0.986300460205734	receptor activity
GO:0016740	1169	8	0.986830264494812	transferase activity
GO:0004930	349	1	0.989703277922346	G-protein coupled receptor activity
GO:0003700	649	3	0.991190976988958	transcription factor activity

GO:0005102	528	2	0.992415898210478	receptor binding
GO:0004871	1837	14	0.9926070695059	signal transducer activity
GO:0016773	532	2	0.992767666238487	phosphotransferase activity, alcohol group as acceptor
GO:0004888	694	3	0.994625201771713	transmembrane receptor activity
GO:0004672	447	1	0.997239490165018	protein kinase activity
GO:0030528	963	4	0.998849479952987	transcription regulator activity
GO:0003677	1384	3	0.999998952135683	DNA binding
GO:0003676	1990	7	0.999999404217703	nucleic acid binding
GO:0003674	9541	122	1	molecular_function

3.4 GO: Cellular Component

GO ID	genes present in chip	genes present in tissues	p-value	GO term
GO:0043292	40	20	2.31113749307084e-29	contractile fiber
GO:0030017	28	18	2.74877589653669e-29	sarcomere
GO:0030016	32	18	9.52738946019296e-28	myofibril
GO:0005863	15	9	1.11541706153878e-14	striated muscle thick filament
GO:0015629	183	19	1.50941507766353e-13	actin cytoskeleton
GO:0005865	10	7	2.44751858300273e-12	striated muscle thin filament
GO:0005859	15	7	1.25241349425033e-10	muscle myosin
GO:0016460	15	7	1.25241349425033e-10	myosin II
GO:0005790	10	6	3.93944777036048e-10	smooth endoplasmic reticulum
GO:0016459	43	9	9.65016816385729e-10	myosin
GO:0005856	612	26	2.86846289297111e-09	cytoskeleton
GO:0005737	2530	55	3.75658408627299e-08	cytoplasm
GO:0043228	977	28	2.76974585992351e-06	non-membrane-bound organelle
GO:0043232	977	28	2.76974585992351e-06	intracellular non-membrane-bound organelle
GO:0005861	5	3	1.43688885972852e-05	troponin complex
GO:0005862	3	2	0.000387295404181372	muscle thin filament tropomyosin
GO:0030018	3	2	0.000387295404181372	Z disc
GO:0005622	5419	78	0.000424851334582374	intracellular
GO:0016528	7	2	0.00263093428285029	sarcoplasm
GO:0016529	7	2	0.00263093428285029	sarcoplasmic reticulum
GO:0005739	490	13	0.00376870260217309	mitochondrion
GO:0043033	1	1	0.0114623133032311	isoamylase complex
GO:0005582	1	1	0.0114623133032311	collagen type XV
GO:0005884	17	2	0.0158143954082177	actin filament
GO:0043226	4515	62	0.0237430347859428	organelle
GO:0043229	4515	62	0.0237430347859428	intracellular organelle
GO:0005605	24	2	0.0304740614313555	basal lamina
GO:0000015	3	1	0.0339981833544761	phosphopyruvate hydratase complex
GO:0042627	4	1	0.0450746461582652	chylomicron
GO:0016011	5	1	0.0560253712863007	dystroglycan complex
GO:0016012	5	1	0.0560253712863007	sarcoglycan complex
GO:0001725	6	1	0.0668517716851252	stress fiber
GO:0005890	7	1	0.0775552445855047	sodium:potassium-exchanging ATPase complex
GO:0005604	44	2	0.090249568172612	basement membrane
GO:0016010	10	1	0.108941838486819	dystrophin-associated glycoprotein complex
GO:0019867	52	2	0.119412075736571	outer membrane
GO:0005623	7802	93	0.144796805787329	cell
GO:0005740	119	3	0.155918794818959	mitochondrial membrane
GO:0005759	62	2	0.158548432044198	mitochondrial matrix
GO:0000794	15	1	0.158920254621448	condensed nuclear chromosome
GO:0045111	65	2	0.170719935008577	intermediate filament cytoskeleton
GO:0005882	65	2	0.170719935008577	intermediate filament

GO:0043234	1247	18	0.176630422356060	protein complex
GO:0005777	67	2	0.178920046597676	peroxisome
GO:0042579	67	2	0.178920046597676	microbody
GO:0005891	18	1	0.187564618317713	voltage-gated calcium channel complex
GO:0005783	483	8	0.188565562586559	endoplasmic reticulum
GO:0005746	21	1	0.215242971458456	mitochondrial electron transport chain
GO:0000793	23	1	0.233174507866101	condensed chromosome
GO:0005741	26	1	0.259313996943175	mitochondrial outer membrane
GO:0030176	26	1	0.259313996943175	integral to endoplasmic reticulum membrane
GO:0005581	30	1	0.292799237706909	collagen
GO:0031227	32	1	0.308975429879132	intrinsic to endoplasmic reticulum membrane
GO:0005840	113	2	0.372757288815651	ribosome
GO:0000228	42	1	0.384522652508744	nuclear chromosome
GO:0005624	521	7	0.388897041369577	membrane fraction
GO:0000267	698	9	0.40740241604702	cell fraction
GO:0031301	76	1	0.5852235695986	integral to organelle membrane
GO:0005789	84	1	0.622092393505318	endoplasmic reticulum membrane
GO:0005625	183	2	0.624407623390197	soluble fraction
GO:0005743	85	1	0.626466630970882	mitochondrial inner membrane
GO:0031090	377	4	0.633280564455011	organelle membrane
GO:0019866	89	1	0.643467977762587	inner membrane
GO:0031300	90	1	0.647597209185318	intrinsic to organelle membrane
GO:0042175	90	1	0.647597209185318	nuclear envelope-endoplasmic reticulum network
GO:0005615	385	4	0.649808659014682	extracellular space
GO:0045202	98	1	0.67897099976714	synapse
GO:0005578	303	3	0.680637933041804	extracellular matrix (sensu Metazoa)
GO:0031012	304	3	0.682812351732141	extracellular matrix
GO:0005764	107	1	0.710970832084662	lysosome
GO:0000323	107	1	0.710970832084662	lytic vacuole
GO:0005792	108	1	0.714325332392237	microsome
GO:0042598	108	1	0.714325332392237	vesicular fraction
GO:0005773	120	1	0.751694836804078	vacuole
GO:0030529	254	2	0.793449286764048	ribonucleoprotein complex
GO:0008372	523	4	0.858104645332447	cellular component unknown
GO:0005887	1057	9	0.870410507384696	integral to plasma membrane
GO:0016021	2674	26	0.870845304187723	integral to membrane
GO:0031226	1062	9	0.873945562604496	intrinsic to plasma membrane
GO:0031224	2681	26	0.87444945442903	intrinsic to membrane
GO:0005694	203	1	0.906355899799248	chromosome
GO:0005576	949	7	0.929166792211925	extracellular region
GO:0005886	1472	12	0.930995713017136	plasma membrane
GO:0012505	246	1	0.943711123785542	endomembrane system
GO:0016020	3514	33	0.946619045209983	membrane
GO:0005829	276	1	0.96059781473644	cytosol
GO:0043227	3955	35	0.986660470227637	membrane-bound organelle
GO:0043231	3955	35	0.986660470227637	intracellular membrane-bound organelle

GO:0005634	2704	12	0.999998070308328	nucleus
GO:0005575	8637	99	1	cellular_component

4. ExtraocularMuscle

4.1 ExtraocularMuscle selective genes (116 genes)

Symbol	Description	Chromosome	Chromosome Location	LocusLink	Cytoband	UniGene
KBTBD10	kelch repeat and BTB (POZ) domain containing 10	2	170074457	10324	2q31.1	Hs.50550
MYH1	myosin, heavy polypeptide 1, skeletal muscle, adult	17	-10336353	4619	17p13.1	Hs.499745
MYOT	myotilin	5	137231472	9499	5q31	Hs.84665
MYH2	myosin, heavy polypeptide 2, skeletal muscle, adult	17	-10365193	4620	17p13.1	Hs.499745
NEB	nebulin	2	-152050100	4703	2q22	Hs.134602 Hs.588655
TTN	titin	2	-358417256	7273	2q31	Hs.134602
MYH13	myosin, heavy polypeptide 13, skeletal muscle	17	-10144907	8735	17p13	Hs.278488
MYL1	myosin, light polypeptide 1, alkali, skeletal, fast	2	-210863121	4632	2q33-q34	Hs.187338
CASQ1	calsequestrin 1 (fast-twitch, skeletal muscle)	1	158426988	844	1q21	Hs.632476
ACTA1	actin, alpha 1, skeletal muscle	1	-227633617	58	1q42.13-q42.2	Hs.1288
CKM	creatine kinase, muscle	19	-50501511	1158	19q13.2-q13.3	Hs.334347
ENO3	enolase 3 (beta, muscle)	17	4795158	2027	17pter-p11	Hs.224171
TNNC1	troponin C type 1 (slow)	3	-52460157	7134	3p21.3-p14.3	Hs.118845
LOC125893	NA	19	-58144445	125893	19q13.41	Hs.446907
TPM2	tropomyosin 2 (beta)	9	-71344912	7169	9p13.2-p13.1	Hs.300772
TNNI2	troponin I type 2 (skeletal, fast)	11	1817478	7136	11p15.5	Hs.523403
ATP2A1	ATPase, Ca++ transporting, cardiac muscle, fast twitch 1	16	28797309	487	16p12.1	Hs.183075
MYL2	myosin, light polypeptide 2, regulatory, cardiac, slow	12	-109833032	4633	12q23-q24.3	Hs.75535
AGL	amylo-1, 6-glucosidase, 4-alpha-glucanotransferase (glycogen debranching enzyme, glycogen storage disease type III)	1	100088632, 100088227, 100099353, 100089118	178	1p21	Hs.904
CKMT2	creatine kinase, mitochondrial 2 (sarcomeric)	5	80564937	1160	5q13.3	Hs.80691
COL15A1	collagen, type XV, alpha 1	9	100746010	1306	9q21-q22	Hs.409034
SMPX	small muscle protein, X-linked	X	-21634012	23676	Xp22.1	Hs.86492
MYH4	myosin, heavy polypeptide 4, skeletal muscle	17	-10287334	4622	17p13.1	Hs.272207
MYBPC1	myosin binding protein C, slow type	12	100512877	4604	12q23.2	Hs.485903
MYH7	myosin, heavy polypeptide 7, cardiac muscle, beta	14	-22951789	4625	14q12	Hs.278432
LUM	lumican	12	-90021363	4060	12q21.3-q22	Hs.406475
ADH1B	alcohol dehydrogenase IB (class I), beta polypeptide	4	-100446551	125	4q21-q23	Hs.4
OGN	osteoglycin (osteoinductive factor, mimecan)	9	-94186081	4969	9q22	Hs.109439
MYH6	myosin, heavy polypeptide 6, cardiac muscle, alpha (cardiomyopathy, hypertrophic 1)	14	-22921038	4624	14q12	Hs.278432
CHGB	chromogranin B (secretogranin 1)	20	5840167	1114	20pter-p12	Hs.516874
RYR1	ryanodine receptor 1 (skeletal)	19	43616179	6261	19q13.1	Hs.466664
MB	myoglobin	22	-34332757	4151	22q13.1	Hs.517586

TNNT1	troponin T type 1 (skeletal, slow)	19	-60336008	7138	19q13.4	Hs.631558
SLC19A2	solute carrier family 19 (thiamine transporter), member 2	1	-167699791	10560	1q23.3	Hs.30246
TNNT3	troponin T type 3 (skeletal, fast)	11	1900668	7140	11p15.5	Hs.73454
RPL3L	ribosomal protein L3-like	16	-1934581	6123	16p13.3	Hs.498569
CSRP3	cysteine and glycine-rich protein 3 (cardiac LIM protein)	11	-19160153	8048	11p15.1	Hs.83577
COX6A2	cytochrome c oxidase subunit VIa polypeptide 2	16	-31346555	1339	16p	Hs.250760
MYF6	myogenic factor 6 (herculin)	12	79625576	4618	12q21	Hs.35937
TNNC2	troponin C type 2 (fast)	20	-43885262	7125	20q12-q13.11	Hs.182421
ABCA8	ATP-binding cassette, sub-family A (ABC1), member 8	17	-64375025	10351	17q24	Hs.58351
GBAS	glioblastoma amplified sequence	7	55999789	2631	7p12	Hs.591069
GRB14	growth factor receptor-bound protein 14	2	-165057578	2888	2q22-q24	Hs.411881
SUCLA2	succinate-CoA ligase, ADP-forming, beta subunit	13	-47414792	8803	13q12.2-q13.3	Hs.546323
NRAP	nebulin-related anchoring protein	10	-115338573	4892	10q24-q26	Hs.268788
ACADM	acyl-Coenzyme A dehydrogenase, C-4 to C-12 straight chain	1	75962869	34	1p31	Hs.445040
TNNI1	troponin I type 1 (skeletal, slow)	1	-199639518	7135	1q31.3	Hs.320890
SH3BGR	SH3 domain binding glutamic acid-rich protein	21	39739666, 39745649	6450	21q22.3	Hs.473847
GBA2	glucosidase, beta (bile acid) 2	9	-35726863	57704	9p13.3	Hs.443134
PPP1R3C	protein phosphatase 1, regulatory (inhibitor) subunit 3C	10	-93378180	5507	10q23-q24	Hs.303090
PYGM	phosphorylase, glycogen, muscle (McArdle syndrome, glycogen storage disease type V)	11	-64270605	5837	11q12-q13.2	Hs.154084
MATN2	matrilin 2	8	98950486	4147	8q22	Hs.189445
TMEM38B	transmembrane protein 38B	9	107496645	55151	9q31.2	Hs.411925
PHYH	phytanoyl-CoA 2-hydroxylase	10	-13359802	5264	10pter-p11.2	Hs.498732
LRTM1	leucine-rich repeats and transmembrane domains 1	3	-54927424	57408	3p14.3	Hs.591668
COX7A1	cytochrome c oxidase subunit VIIa polypeptide 1 (muscle)	19	-41333663	1346	19q13.1	Hs.421621
SACS	spastic ataxia of Charlevoix-Saguenay (sacsin)	13	-22800966	26278	13q12	Hs.159492
MFAP5	microfibrillar associated protein 5	12	-8689806	8076	12p13.1-p12.3	Hs.512842
DES	desmin	2	219991342	1674	2q35	Hs.594952
CD36	CD36 molecule (thrombospondin receptor)	7	80105892, 80069458	948	7q11.2	Hs.120949 Hs.633085
PLN	phospholamban	6	118976164	5350	6q22.1	Hs.170839
PDLIM3	PDZ and LIM domain 3	4	-186659904	27295	4q35	Hs.85862
LOC653513	NA	1	NA	653513	1q21.1	Hs.632434
PDE4DIP	phosphodiesterase 4D interacting protein (myomegalin)	1	-430827846	9659	1q12	Hs.568247 Hs.584841 Hs.613082 Hs.626094
ATP1A2	ATPase, Na+/K+ transporting, alpha 2 (+) polypeptide	1	158352171	477	1q21-q23	Hs.34114
EEF1A2	eukaryotic translation elongation factor 1 alpha 2	20	-61589809	1917	20q13.3	Hs.433839
FHL1	four and a half LIM domains 1	X	135057345	2273	Xq26	Hs.435369
AMPD1	adenosine monophosphate deaminase 1 (isoform M)	1	-115017244	270	1p13	Hs.89570
LDB3	LIM domain binding 3	10	88418407	11155	10q22.3-q23.2	Hs.49998

ART3	ADP-ribosyltransferase 3	4	77214886	419	4p15.1-p14|4p15.1-p14|4p15.1-p14	Hs.24976
MYOM1	myomesin 1 (skelemin) 185kDa	18	-3056805	8736	18p11.32-p11.31	Hs.464469
HSPB3	heat shock 27kDa protein 3	5	53787201	8988	5q11.2	Hs.41707
FYCO1	FYVE and coiled-coil domain containing 1	3	-45934399	79443	3p21.31	Hs.200227
PVALB	parvalbumin	22	-35526691	5816	22q12-q13.1|22q13.1	Hs.295449
CFD	complement factor D (adipsin)	19	810664	1675	19p13.3	Hs.155597
CMKOR1	chemokine orphan receptor 1	2	237143181	57007	2q37.3	Hs.471751
COL21A1	collagen, type XXI, alpha 1	6	-56029347	81578	6p12.3-p11.2|6p12.3-p11.2	Hs.47629
CDH5	cadherin 5, type 2, VE-cadherin (vascular epithelium)	16	64958063	1003	16q22.1	Hs.76206
NDUFA5	NADH dehydrogenase (ubiquinone) 1 alpha subcomplex, 5, 13kDa	7	-122968319	4698	7q32	Hs.643550
FLJ22028	NA	12	21481877	79912	12p12.1	Hs.46835
DKFZP686A01247	NA	4	41057560	22998	4p13	Hs.335163
TCAP	titin-cap (telethonin)	17	35075124	8557	17q12	Hs.514146
SAR1B	SAR1 gene homolog B (S. cerevisiae)	5	-133970018	51128	5q31.1	Hs.432984
OSR2	odd-skipped related 2 (Drosophila)	8	100025861	116039	8q22.2	Hs.253247
GHR	growth hormone receptor	5	42459782	2690	5p13-p12	Hs.125180
HSPB7	heat shock 27kDa protein family, member 7 (cardiovascular)	1	-16213111	27129	1p36.23-p34.3	Hs.502612
SYNPO2L	synaptopodin 2-like	10	-75075295	79933	10q22.2	Hs.533659
FABP3	fatty acid binding protein 3, muscle and heart (mammary-derived growth inhibitor)	1	-31610686	2170	1p33-p32	Hs.584756
ALDH1A1	aldehyde dehydrogenase 1 family, member A1	9	-74705407	216	9q21.13	Hs.76392
MYOZ2	myozenin 2	4	120276468	51778	4q26-q27	Hs.381047
CXCL14	chemokine (C-X-C motif) ligand 14	5	-134934274	9547	5q31	Hs.483444
NNT	nicotinamide nucleotide transhydrogenase	5	43638581, 43639063	23530	5p13.1-5cen	Hs.482043
CAPN3	calpain 3, (p94)	15	40438989, 40481879, 40484268, 40427592	825	15q15.1-q21.1	Hs.143261
PGAM2	phosphoglycerate mutase 2 (muscle)	7	-44068851	5224	7p13-p12	Hs.632642
ACTN2	actinin, alpha 2	1	234916430	88	1q42-q43	Hs.498178
GENX-3414	NA	4	77446832	8987	4q24-q25	Hs.109590
CRYAB	crystallin, alpha B	11	-111284559	1410	11q22.3-q23.1	Hs.408767
CPT1B	carnitine palmitoyltransferase 1B (muscle)	22	-49354156	1375	22q13.33	Hs.439777
FRZB	frizzled-related protein	2	-183406981	2487	2qter	Hs.128453
CHCHD3	coiled-coil-helix-coiled-coil-helix domain containing 3	7	-132120163	54927	7q32.3-q33	Hs.444467
COL6A3	collagen, type VI, alpha 3	2	-237897400	1293	2q37	Hs.233240
NA	NA	NA	NA	492304	NA	NA
ACTC	actin, alpha, cardiac muscle	15	-32869723	70	15q11-q14	Hs.118127
RHOQ	ras homolog gene family, member Q	2	46623378	23433	2p21	Hs.643481
MYL4	myosin, light polypeptide 4, alkali, atrial, embryonic	17	42641426, 42641712	4635	17q21-qter	Hs.463300
CAV2	caveolin 2	7	115926679	858	7q31.1	Hs.212332 Hs.603096
ITGBL1	integrin, beta-like 1 (with EGF-like repeat domains)	13	100902966	9358	13q33	Hs.643461
POPDC2	popeye domain containing 2	3	-120843597	64091	3q13.33	Hs.534383

PPP1R1A	protein phosphatase 1, regulatory (inhibitor) subunit 1A	12	-53259299	5502	12q13.2	Hs.505662
RGS5	regulator of G-protein signalling 5	1	-161378721	8490	1q23.1	Hs.568700
TOB1	Transducer of ERBB2, 1	17	-46294587	10140	17q21	Hs.531550 Hs.606234
SGCG	sarcoglycan, gamma (35kDa dystrophin-associated glycoprotein)	13	22653090	6445	13q12	Hs.37167
EFHA1	EF-hand domain family, member A1	13	-20964839	221154	13q12.11	Hs.412103
PITX2	paired-like homeodomain transcription factor 2	4	-111758030	5308	4q25-q27	Hs.643588
MYOZ1	myozenin 1	10	-75061420	58529	10q22.1	Hs.238756
IQWD1	IQ motif and WD repeats 1	1	166172531	55827	1q24.2	Hs.435741

4.2 GO: Biological Process

GO ID	genes present in chip	genes present in tissues	p-value	GO term
GO:0006936	132	28	3.07E-30	muscle contraction
GO:0006941	29	16	2.09E-25	striated muscle contraction
GO:0006937	22	7	1.36E-09	regulation of muscle contraction
GO:0007517	94	11	2.30E-09	muscle development
GO:0006942	7	4	3.45E-07	regulation of striated muscle contraction
GO:0050874	1531	35	9.96E-07	organismal physiological process
GO:0030239	4	3	4.06E-06	myofibril assembly
GO:0051239	133	9	7.68E-06	regulation of organismal physiological process
GO:0048627	7	3	3.48E-05	myoblast development
GO:0048628	7	3	3.48E-05	myoblast maturation
GO:0007519	39	5	4.30E-05	myogenesis
GO:0015980	132	7	0.000379153	energy derivation by oxidation of organic compounds
GO:0045445	15	3	0.000425885	myoblast differentiation
GO:0006091	446	13	0.000547677	generation of precursor metabolites and energy
GO:0042692	19	3	0.000880616	muscle cell differentiation
GO:0048469	21	3	0.001191005	cell maturation
GO:0005977	27	3	0.002506218	glycogen metabolism
GO:0006073	28	3	0.002786421	glucan metabolism
GO:0006112	34	3	0.004870617	energy reserve metabolism
GO:0015669	12	2	0.006338687	gas transport
GO:0015671	12	2	0.006338687	oxygen transport
GO:0009887	807	16	0.007011358	organogenesis
GO:0048468	40	3	0.007696443	cell development
GO:0044264	42	3	0.008813195	cellular polysaccharide metabolism
GO:0006635	15	2	0.009886251	fatty acid beta-oxidation
GO:0005975	355	9	0.009940337	carbohydrate metabolism
GO:0005976	44	3	0.010020163	polysaccharide metabolism
GO:0015888	1	1	0.010186005	thiamin transport
GO:0006938	1	1	0.010186005	sarcomere alignment
GO:0007525	1	1	0.010186005	somatic muscle development
GO:0030240	1	1	0.010186005	muscle thin filament assembly
GO:0006781	1	1	0.010186005	succinyl-CoA pathway
GO:0006092	88	4	0.012267697	main pathways of carbohydrate metabolism
GO:0030029	139	5	0.013567419	actin filament-based process
GO:0048513	872	16	0.014213543	organ development
GO:0044262	265	7	0.018224415	cellular carbohydrate metabolism
GO:0006817	57	3	0.020141415	phosphate transport
GO:0015992	57	3	0.020141415	proton transport
GO:0046502	2	1	0.020269372	uroporphyrinogen III metabolism
GO:0006780	2	1	0.020269372	uroporphyrinogen III biosynthesis
GO:0019395	22	2	0.020769965	fatty acid oxidation
GO:0006099	22	2	0.020769965	tricarboxylic acid cycle

GO:0046356	22	2	0.020769965	acetyl-CoA catabolism
GO:0006818	58	3	0.021086707	hydrogen transport
GO:0008016	23	2	0.022599225	regulation of heart contraction rate
GO:0009109	23	2	0.022599225	coenzyme catabolism
GO:0007368	3	1	0.030251124	determination of left/right symmetry
GO:0009799	3	1	0.030251124	determination of symmetry
GO:0009855	3	1	0.030251124	determination of bilateral symmetry
GO:0006104	3	1	0.030251124	succinyl-CoA metabolism
GO:0006084	27	2	0.030542167	acetyl-CoA metabolism
GO:0006816	68	3	0.031861291	calcium ion transport
GO:0051187	28	2	0.032677224	cofactor catabolism
GO:0007010	305	7	0.035629199	cytoskeleton organization and biogenesis
GO:0009060	30	2	0.037116818	aerobic respiration
GO:0030036	126	4	0.039434602	actin cytoskeleton organization and biogenesis
GO:0015991	4	1	0.040132273	ATP hydrolysis coupled proton transport
GO:0006067	4	1	0.040132273	ethanol metabolism
GO:0006069	4	1	0.040132273	ethanol oxidation
GO:0051289	4	1	0.040132273	protein homotetramerization
GO:0030199	4	1	0.040132273	collagen fibril organization
GO:0045333	32	2	0.041773463	cellular respiration
GO:0006118	256	6	0.046171256	electron transport
GO:0030641	5	1	0.049913823	hydrogen ion homeostasis
GO:0030154	409	8	0.056309567	cell differentiation
GO:0015988	6	1	0.059596766	energy coupled proton transport, against electrochemical gradient
GO:0031032	6	1	0.059596766	actomyosin structure organization and biogenesis
GO:0006986	40	2	0.062367054	response to unfolded protein
GO:0009653	1051	16	0.064255769	morphogenesis
GO:0006096	42	2	0.067959085	glycolysis
GO:0030004	8	1	0.078670756	monovalent inorganic cation homeostasis
GO:0030317	8	1	0.078670756	sperm motility
GO:0015674	99	3	0.079886777	di-, tri-valent inorganic cation transport
GO:0008015	103	3	0.087556494	circulation
GO:0030178	9	1	0.088063741	negative regulation of Wnt receptor signaling pathway
GO:0009968	50	2	0.09183527	negative regulation of signal transduction
GO:0015698	106	3	0.093506377	inorganic anion transport
GO:0006457	169	4	0.093570364	protein folding
GO:0006007	51	2	0.094972502	glucose catabolism
GO:0005978	10	1	0.097361993	glycogen biosynthesis
GO:0009250	10	1	0.097361993	glucan biosynthesis
GO:0051262	10	1	0.097361993	protein tetramerization
GO:0000004	465	8	0.100646783	biological process unknown
GO:0006957	11	1	0.10656646	complement activation, alternative pathway
GO:0009167	11	1	0.10656646	purine ribonucleoside monophosphate metabolism
GO:0009168	11	1	0.10656646	purine ribonucleoside monophosphate biosynthesis
GO:0009126	11	1	0.10656646	purine nucleoside monophosphate metabolism
GO:0009127	11	1	0.10656646	purine nucleoside monophosphate biosynthesis

GO:0051260	11	1	0.10656646	protein homooligomerization
GO:0008206	12	1	0.115678076	bile acid metabolism
GO:0051180	12	1	0.115678076	vitamin transport
GO:0006414	12	1	0.115678076	translational elongation
GO:0019320	59	2	0.121098246	hexose catabolism
GO:0046365	59	2	0.121098246	monosaccharide catabolism
GO:0046164	60	2	0.124479238	alcohol catabolism
GO:0006081	13	1	0.124697767	aldehyde metabolism
GO:0006779	13	1	0.124697767	porphyrin biosynthesis
GO:0006631	124	3	0.132439	fatty acid metabolism
GO:0030048	14	1	0.133626452	actin filament-based movement
GO:0006820	128	3	0.141765371	anion transport
GO:0030111	15	1	0.142465037	regulation of Wnt receptor signaling pathway
GO:0006811	515	8	0.153234772	ion transport
GO:0007155	518	8	0.156757168	cell adhesion
GO:0043284	17	1	0.159875501	biopolymer biosynthesis
GO:0009161	17	1	0.159875501	ribonucleoside monophosphate metabolism
GO:0009156	17	1	0.159875501	ribonucleoside monophosphate biosynthesis
GO:0000271	17	1	0.159875501	polysaccharide biosynthesis
GO:0046777	17	1	0.159875501	autophosphorylation
GO:0009123	18	1	0.168449151	nucleoside monophosphate metabolism
GO:0009124	18	1	0.168449151	nucleoside monophosphate biosynthesis
GO:0016540	18	1	0.168449151	protein autoprocessing
GO:0006778	18	1	0.168449151	porphyrin metabolism
GO:0006100	20	1	0.185337652	tricarboxylic acid cycle intermediate metabolism
GO:0016052	79	2	0.192202077	carbohydrate catabolism
GO:0006006	79	2	0.192202077	glucose metabolism
GO:0044275	79	2	0.192202077	cellular carbohydrate catabolism
GO:0050953	149	3	0.193936657	sensory perception of light
GO:0007601	149	3	0.193936657	visual perception
GO:0006471	22	1	0.201886806	protein amino acid ADP-ribosylation
GO:0006810	1802	22	0.20238776	transport
GO:0008277	23	1	0.210036242	regulation of G-protein coupled receptor protein signaling pathway
GO:0007015	23	1	0.210036242	actin filament organization
GO:0007389	24	1	0.21810336	pattern specification
GO:0051259	24	1	0.21810336	protein oligomerization
GO:0050954	86	2	0.218272497	sensory perception of mechanical stimulus
GO:0050982	86	2	0.218272497	detection of mechanical stimulus
GO:0007605	86	2	0.218272497	perception of sound
GO:0051234	1841	22	0.233617569	establishment of localization
GO:0051179	1857	22	0.247058768	localization
GO:0006956	30	1	0.264822892	complement activation
GO:0006812	346	5	0.276848452	cation transport
GO:0030001	269	4	0.293522261	metal ion transport
GO:0043062	35	1	0.30163821	extracellular structure organization and biogenesis
GO:0030198	35	1	0.30163821	extracellular matrix organization and biogenesis

GO:0030832	35	1	0.30163821	regulation of actin filament length
GO:0009582	109	2	0.305155328	detection of abiotic stimulus
GO:0016485	37	1	0.315848352	protein processing
GO:0001501	113	2	0.32020266	skeletal development
GO:0019318	113	2	0.32020266	hexose metabolism
GO:0005996	115	2	0.327699322	monosaccharide metabolism
GO:0007600	287	4	0.335181005	sensory perception
GO:0006732	121	2	0.350053871	coenzyme metabolism
GO:0006979	44	1	0.36336819	response to oxidative stress
GO:0046483	44	1	0.36336819	heterocycle metabolism
GO:0006066	213	3	0.369866527	alcohol metabolism
GO:0007229	45	1	0.36988467	integrin-mediated signaling pathway
GO:0007160	49	1	0.395297326	cell-matrix adhesion
GO:0009581	313	4	0.395758013	detection of external stimulus
GO:0015672	223	3	0.397620025	monovalent inorganic cation transport
GO:0008285	139	2	0.415445406	negative regulation of cell proliferation
GO:0045087	53	1	0.41969578	innate immune response
GO:0051186	143	2	0.429552931	cofactor metabolism
GO:0009152	58	1	0.448828487	purine ribonucleotide biosynthesis
GO:0006888	58	1	0.448828487	ER to Golgi transport
GO:0009150	60	1	0.460072225	purine ribonucleotide metabolism
GO:0006800	61	1	0.465608712	oxygen and reactive oxygen species metabolism
GO:0006164	61	1	0.465608712	purine nucleotide biosynthesis
GO:0040007	154	2	0.467410328	growth
GO:0006163	63	1	0.476513765	purine nucleotide metabolism
GO:0001525	65	1	0.487198654	angiogenesis
GO:0009260	65	1	0.487198654	ribonucleotide biosynthesis
GO:0019752	359	4	0.500269771	carboxylic acid metabolism
GO:0001568	68	1	0.502822775	blood vessel development
GO:0048514	68	1	0.502822775	blood vessel morphogenesis
GO:0009259	68	1	0.502822775	ribonucleotide metabolism
GO:0006082	361	4	0.504660848	organic acid metabolism
GO:0001944	69	1	0.507925442	vasculature development
GO:0016051	69	1	0.507925442	carbohydrate biosynthesis
GO:0007275	1543	16	0.512056417	development
GO:0006629	469	5	0.524079179	lipid metabolism
GO:0007596	78	1	0.551579504	blood coagulation
GO:0044255	383	4	0.551813726	cellular lipid metabolism
GO:0050817	79	1	0.556186907	coagulation
GO:0006814	80	1	0.560747479	sodium ion transport
GO:0006996	693	7	0.566077951	organelle organization and biogenesis
GO:0007599	83	1	0.574152898	hemostasis
GO:0042060	85	1	0.582864199	wound healing
GO:0051188	85	1	0.582864199	cofactor biosynthesis
GO:0048193	86	1	0.587153513	Golgi vesicle transport
GO:0016055	87	1	0.591399196	Wnt receptor signaling pathway

GO:0006461	194	2	0.591738401	protein complex assembly
GO:0050878	94	1	0.619933622	regulation of body fluids
GO:0009628	318	3	0.634064329	response to abiotic stimulus
GO:0016043	842	8	0.635184964	cell organization and biogenesis
GO:0030003	98	1	0.635344509	cation homeostasis
GO:0006935	101	1	0.646495516	chemotaxis
GO:0042330	101	1	0.646495516	taxis
GO:0009165	103	1	0.653741447	nucleotide biosynthesis
GO:0006873	103	1	0.653741447	cell ion homeostasis
GO:0007156	105	1	0.660840436	homophilic cell adhesion
GO:0007264	227	2	0.677299546	small GTPase mediated signal transduction
GO:0006813	111	1	0.681284944	potassium ion transport
GO:0050801	111	1	0.681284944	ion homeostasis
GO:0009966	229	2	0.681992195	regulation of signal transduction
GO:0006897	115	1	0.694232413	endocytosis
GO:0007169	116	1	0.697387131	transmembrane receptor protein tyrosine kinase signaling pathway
GO:0009059	464	4	0.702895496	macromolecule biosynthesis
GO:0043037	122	1	0.715650539	translation
GO:0019725	122	1	0.715650539	cell homeostasis
GO:0008361	123	1	0.718586583	regulation of cell size
GO:0016049	123	1	0.718586583	cell growth
GO:0050877	475	4	0.720356383	neurophysiological process
GO:0044248	700	6	0.727508643	cellular catabolism
GO:0008202	132	1	0.743697163	steroid metabolism
GO:0007067	135	1	0.751564427	mitosis
GO:0044260	2298	21	0.754674093	cellular macromolecule metabolism
GO:0000087	137	1	0.756676076	M phase of mitotic cell cycle
GO:0042127	265	2	0.757352518	regulation of cell proliferation
GO:0043170	2405	22	0.758430862	macromolecule metabolism
GO:0006959	141	1	0.766589308	humoral immune response
GO:0009117	143	1	0.771395039	nucleotide metabolism
GO:0007582	7846	78	0.778568139	physiological process
GO:0006412	407	3	0.790648951	protein biosynthesis
GO:0009056	756	6	0.79315668	catabolism
GO:0044249	763	6	0.80046215	cellular biosynthesis
GO:0042592	157	1	0.802401926	homeostasis
GO:0016192	296	2	0.809531097	vesicle-mediated transport
GO:0045045	161	1	0.810470108	secretory pathway
GO:0007167	165	1	0.818212292	enzyme linked receptor protein signaling pathway
GO:0016337	172	1	0.831015589	cell-cell adhesion
GO:0000279	173	1	0.83277028	M phase
GO:0044265	563	4	0.833755637	cellular macromolecule catabolism
GO:0009057	575	4	0.845834383	macromolecule catabolism
GO:0042221	183	1	0.849355478	response to chemical substance
GO:0000902	183	1	0.849355478	cellular morphogenesis
GO:0006954	185	1	0.852471768	inflammatory response

GO:0009611	341	2	0.867687538	response to wounding
GO:0009605	963	7	0.872922922	response to external stimulus
GO:0000278	204	1	0.879071367	mitotic cell cycle
GO:0006928	205	1	0.880331611	cell motility
GO:0040011	205	1	0.880331611	locomotion
GO:0009058	859	6	0.881912188	biosynthesis
GO:0046903	208	1	0.884034918	secretion
GO:0007626	209	1	0.885243983	locomotory behavior
GO:0006950	881	6	0.896105021	response to stress
GO:0048523	640	4	0.899252223	negative regulation of cellular process
GO:0006357	240	1	0.917127068	regulation of transcription from RNA polymerase II promoter
GO:0048519	688	4	0.927642899	negative regulation of biological process
GO:0006886	257	1	0.930708763	intracellular protein transport
GO:0007610	269	1	0.938945766	behavior
GO:0009607	857	5	0.944877566	response to biotic stimulus
GO:0006508	451	2	0.948403682	proteolysis and peptidolysis
GO:0044257	451	2	0.948403682	cellular protein catabolism
GO:0009613	458	2	0.951505134	response to pest, pathogen or parasite
GO:0043207	463	2	0.9536119	response to external biotic stimulus
GO:0030163	463	2	0.9536119	protein catabolism
GO:0008283	473	2	0.95756887	cell proliferation
GO:0043285	474	2	0.957946514	biopolymer catabolism
GO:0007186	491	2	0.963899337	G-protein coupled receptor protein signaling pathway
GO:0050896	1578	10	0.971332041	response to stimulus
GO:0007166	952	5	0.971723527	cell surface receptor linked signal transduction
GO:0044267	2109	14	0.980137452	cellular protein metabolism
GO:0007399	385	1	0.98219768	neurogenesis
GO:0019538	2129	14	0.982335398	protein metabolism
GO:0051243	583	2	0.984503707	negative regulation of cellular physiological process
GO:0043118	602	2	0.98703649	negative regulation of physiological process
GO:0006366	426	1	0.988529851	transcription from RNA polymerase II promoter
GO:0015031	439	1	0.990026526	protein transport
GO:0006468	444	1	0.990549234	protein amino acid phosphorylation
GO:0046907	448	1	0.990947811	intracellular transport
GO:0045184	459	1	0.991960177	establishment of protein localization
GO:0007267	465	1	0.992464299	cell-cell signaling
GO:0008104	474	1	0.993162422	protein localization
GO:0006955	684	2	0.994078655	immune response
GO:0007242	879	3	0.995315914	intracellular signaling cascade
GO:0016310	522	1	0.995935894	phosphorylation
GO:0007154	2730	17	0.996443867	cell communication
GO:0006952	751	2	0.996924574	defense response
GO:0007049	601	1	0.998284837	cell cycle
GO:0050791	2407	13	0.998764448	regulation of physiological process
GO:0006793	641	1	0.99889527	phosphorus metabolism
GO:0006796	641	1	0.99889527	phosphate metabolism

GO:0050789	2689	15	0.999088059	regulation of biological process
GO:0008152	5262	39	0.999269274	metabolism
GO:0006464	1087	3	0.999337593	protein modification
GO:0050875	7018	58	0.999564123	cellular physiological process
GO:0007165	2168	10	0.999590359	signal transduction
GO:0043412	1160	3	0.999676499	biopolymer modification
GO:0044237	5026	35	0.999778423	cellular metabolism
GO:0043283	2240	8	0.999981543	biopolymer metabolism
GO:0044238	4820	30	0.999982572	primary metabolism
GO:0009987	8200	70	0.999993397	cellular process
GO:0006355	1359	2	0.999995085	regulation of transcription, DNA-dependent
GO:0006351	1407	2	0.999997141	transcription, DNA-dependent
GO:0045449	1433	2	0.999997873	regulation of transcription
GO:0019219	1458	2	0.999998401	regulation of nucleobase, nucleoside, nucleotide and nucleic acid metabolism
GO:0006350	1499	2	0.999999002	transcription
GO:0031323	1566	2	0.999999541	regulation of cellular metabolism
GO:0019222	1629	2	0.999999781	regulation of metabolism
GO:0050794	2488	6	0.999999946	regulation of cellular process
GO:0051244	2319	4	0.999999993	regulation of cellular physiological process
GO:0006139	2328	3	0.999999999	nucleobase, nucleoside, nucleotide and nucleic acid metabolism
GO:0008150	9032	92	1	biological_process

4.3 GO: Molecular Function

GO ID	genes present in chip	genes present in tissues	p-value	GO term
GO:0008307	33	11	2.65786970935151e-14	structural constituent of muscle
GO:0008092	279	22	2.16624504781092e-13	cytoskeletal protein binding
GO:0003779	189	17	1.93566584965997e-11	actin binding
GO:0005198	488	25	6.09954840795369e-11	structural molecule activity
GO:0000146	15	6	7.10522180261536e-09	microfilament motor activity
GO:0003774	98	9	1.16923073058558e-06	motor activity
GO:0005523	9	3	0.000103689349746858	tropomyosin binding
GO:0051373	2	2	0.000119972129551443	FATZ binding
GO:0005516	112	7	0.000224789853111346	calmodulin binding
GO:0004111	3	2	0.000357325523921380	creatine kinase activity
GO:0017022	5	2	0.00117401986292016	myosin binding
GO:0015399	140	6	0.0043964254465095	primary active transporter activity
GO:0016775	10	2	0.00509642344620811	phosphotransferase activity, nitrogenous group as acceptor
GO:0005344	12	2	0.0073682849134001	oxygen transporter activity
GO:0015926	13	2	0.00864581217945093	glucosidase activity
GO:0005070	40	3	0.00952506286454099	SH3/SH2 adaptor activity
GO:0005515	3709	53	0.00996481109259095	protein binding
GO:0015077	121	5	0.0106509168142534	monovalent inorganic cation transporter activity
GO:0005509	646	14	0.0109919258361562	calcium ion binding
GO:0005199	1	1	0.0110051357300072	structural constituent of cell wall
GO:0048244	1	1	0.0110051357300072	phytanoyl-CoA dioxygenase activity
GO:0004903	1	1	0.0110051357300072	growth hormone receptor activity
GO:0017147	1	1	0.0110051357300072	Wnt-protein binding
GO:0008750	1	1	0.0110051357300072	NAD(P)+ transhydrogenase (AB-specific) activity
GO:0008746	1	1	0.0110051357300072	NAD(P) transhydrogenase activity
GO:0042030	1	1	0.0110051357300072	ATPase inhibitor activity
GO:0003817	1	1	0.0110051357300072	complement factor D activity
GO:0051393	1	1	0.0110051357300072	alpha-actinin binding
GO:0003957	1	1	0.0110051357300072	NAD(P)+ transhydrogenase (B-specific) activity
GO:0051370	1	1	0.0110051357300072	ZASP binding
GO:0051374	1	1	0.0110051357300072	FATZ 1 binding
GO:0004133	1	1	0.0110051357300072	glycogen debranching enzyme activity
GO:0004134	1	1	0.0110051357300072	4-alpha-glucanotransferase activity
GO:0004135	1	1	0.0110051357300072	amylo-alpha-1,6-glucosidase activity
GO:0015234	1	1	0.0110051357300072	thiamin transporter activity
GO:0004775	1	1	0.0110051357300072	succinate-CoA ligase (ADP-forming) activity
GO:0004619	2	1	0.0218902993304635	phosphoglycerate mutase activity
GO:0008422	2	1	0.0218902993304635	beta-glucosidase activity
GO:0008184	2	1	0.0218902993304635	glycogen phosphorylase activity
GO:0000163	2	1	0.0218902993304635	protein phosphatase type 1 activity
GO:0004082	2	1	0.0218902993304635	bisphosphoglycerate mutase activity
GO:0004083	2	1	0.0218902993304635	bisphosphoglycerate phosphatase activity

GO:0042805	2	1	0.0218902993304635	actinin binding
GO:0030346	2	1	0.0218902993304635	protein phosphatase 2B binding
GO:0001758	2	1	0.0218902993304635	retinal dehydrogenase activity
GO:0015002	21	2	0.0219855003103568	heme-copper terminal oxidase activity
GO:0016675	21	2	0.0219855003103568	oxidoreductase activity, acting on heme group of donors
GO:0016676	21	2	0.0219855003103568	oxidoreductase activity, acting on heme group of donors, oxygen as acceptor
GO:0004129	21	2	0.0219855003103568	cytochrome-c oxidase activity
GO:0019842	59	3	0.0269864611290097	vitamin binding
GO:0005386	398	9	0.0309633794775685	carrier activity
GO:0004865	3	1	0.0326567862337344	type 1 serine/threonine specific protein phosphatase inhibitor activity
GO:0004634	3	1	0.0326567862337344	phosphopyruvate hydratase activity
GO:0003876	3	1	0.0326567862337344	AMP deaminase activity
GO:0016416	3	1	0.0326567862337344	O-palmitoyltransferase activity
GO:0004095	3	1	0.0326567862337344	carnitine O-palmitoyltransferase activity
GO:0004774	3	1	0.0326567862337344	succinate-CoA ligase activity
GO:0015078	112	4	0.0348355013880332	hydrogen ion transporter activity
GO:0030674	69	3	0.0402247441450568	protein binding, bridging
GO:0004645	4	1	0.0433058780187476	phosphorylase activity
GO:0005497	4	1	0.0433058780187476	androgen binding
GO:0008517	4	1	0.0433058780187476	folic acid transporter activity
GO:0008518	4	1	0.0433058780187476	reduced folate carrier activity
GO:0003956	4	1	0.0433058780187476	NAD(P)+-protein-arginine ADP-ribosyltransferase activity
GO:0016405	4	1	0.0433058780187476	CoA-ligase activity
GO:0004024	4	1	0.0433058780187476	alcohol dehydrogenase activity, zinc-dependent
GO:0005488	7228	87	0.051763862604277	binding
GO:0016491	509	10	0.0531116800443724	oxidoreductase activity
GO:0016406	5	1	0.0538388425577045	carnitine O-acyltransferase activity
GO:0016878	5	1	0.0538388425577045	acid-thiol ligase activity
GO:0016409	6	1	0.0642569341612533	palmitoyltransferase activity
GO:0019903	6	1	0.0642569341612533	protein phosphatase binding
GO:0015278	6	1	0.0642569341612533	calcium-release channel activity
GO:0005200	86	3	0.0688663211391639	structural constituent of cytoskeleton
GO:0015662	40	2	0.0714203877615202	ATPase activity, coupled to transmembrane movement of ions, phosphorylative mechanism
GO:0005246	7	1	0.0745613937221409	calcium channel regulator activity
GO:0051183	7	1	0.0745613937221409	vitamin transporter activity
GO:0016868	7	1	0.0745613937221409	intramolecular transferase activity, phosphotransferases
GO:0004022	8	1	0.0847534488573598	alcohol dehydrogenase activity
GO:0004029	8	1	0.0847534488573598	aldehyde dehydrogenase (NAD) activity
GO:0030170	8	1	0.0847534488573598	pyridoxal phosphate binding
GO:0005099	8	1	0.0847534488573598	Ras GTPase activator activity
GO:0005489	152	4	0.086508910829835	electron transporter activity
GO:0005391	9	1	0.0948343140487974	sodium:potassium-exchanging ATPase activity
GO:0005388	9	1	0.0948343140487974	calcium-transporting ATPase activity
GO:0051184	9	1	0.0948343140487974	cofactor transporter activity
GO:0042802	158	4	0.0963649251197731	protein self binding
GO:0004896	48	2	0.0976866611179018	hematopoietin/interferon-class (D200-domain) cytokine receptor activity

GO:0048037	100	3	0.09771133145257	cofactor binding
GO:0042626	100	3	0.09771133145257	ATPase activity, coupled to transmembrane movement of substances
GO:0016820	101	3	0.0999349718671335	hydrolase activity, acting on acid anhydrides, catalyzing transmembrane movement of substances
GO:0003746	10	1	0.104805190782419	translation elongation factor activity
GO:0005542	10	1	0.104805190782419	folic acid binding
GO:0019902	10	1	0.104805190782419	phosphatase binding
GO:0009055	104	3	0.106727296750720	electron carrier activity
GO:0016866	11	1	0.114667267685980	intramolecular transferase activity
GO:0031418	12	1	0.124421720665306	L-ascorbic acid binding
GO:0003995	12	1	0.124421720665306	acyl-CoA dehydrogenase activity
GO:0004028	12	1	0.124421720665306	aldehyde dehydrogenase activity
GO:0042562	12	1	0.124421720665306	hormone binding
GO:0016877	12	1	0.124421720665306	ligase activity, forming carbon-sulfur bonds
GO:0016652	12	1	0.124421720665306	oxidoreductase activity, acting on NADH or NADPH, NAD or NADP as acceptor
GO:0004198	12	1	0.124421720665306	calpain activity
GO:0016651	57	2	0.129881948906281	oxidoreductase activity, acting on NADH or NADPH
GO:0015085	13	1	0.134069713039127	calcium ion transporter activity
GO:0016526	13	1	0.134069713039127	G-protein coupled receptor activity, unknown ligand
GO:0004553	59	2	0.137339374761783	hydrolase activity, hydrolyzing O-glycosyl compounds
GO:0017076	1248	18	0.137519706901512	purine nucleotide binding
GO:0005518	14	1	0.143612395672512	collagen binding
GO:0003950	14	1	0.143612395672512	NAD+ ADP-ribosyltransferase activity
GO:0004864	15	1	0.153050907108891	protein phosphatase inhibitor activity
GO:0030554	1028	15	0.156163558017692	adenyl nucleotide binding
GO:0042803	64	2	0.156371953172078	protein homodimerization activity
GO:0005215	1030	15	0.157911383228135	transporter activity
GO:0051082	127	3	0.164123322652476	unfolded protein binding
GO:0005212	17	1	0.171619909738645	structural constituent of eye lens
GO:0019212	17	1	0.171619909738645	phosphatase inhibitor activity
GO:0005504	17	1	0.171619909738645	fatty acid binding
GO:0050661	17	1	0.171619909738645	NADP binding
GO:0042625	68	2	0.171938372383494	ATPase activity, coupled to transmembrane movement of ions
GO:0005524	970	14	0.177282814594013	ATP binding
GO:0008374	18	1	0.180752617579619	O-acyltransferase activity
GO:0019239	18	1	0.180752617579619	deaminase activity
GO:0051287	18	1	0.180752617579619	NAD binding
GO:0016620	18	1	0.180752617579619	oxidoreductase activity, acting on the aldehyde or oxo group of donors, NAD or NADP as acceptor
GO:0008324	421	7	0.181144534231459	cation transporter activity
GO:0005201	73	2	0.191733730193939	extracellular matrix structural constituent
GO:0046873	74	2	0.195729979364590	metal ion transporter activity
GO:0016814	21	1	0.207556619131083	hydrolase activity, acting on carbon-nitrogen (but not peptide) bonds, in cyclic amidines
GO:0016798	77	2	0.207779913998547	hydrolase activity, acting on glycosyl bonds
GO:0015405	77	2	0.207779913998547	P-P-bond-hydrolysis-driven transporter activity
GO:0004601	22	1	0.216296803478902	peroxidase activity
GO:0016684	22	1	0.216296803478902	oxidoreductase activity, acting on peroxide as acceptor
GO:0000166	1432	19	0.221599629212739	nucleotide binding

GO:0016903	25	1	0.241948532694415	oxidoreductase activity, acting on the aldehyde or oxo group of donors
GO:0042623	229	4	0.244866960113966	ATPase activity, coupled
GO:0016247	26	1	0.250312908909956	channel regulator activity
GO:0050662	88	2	0.252493235617857	coenzyme binding
GO:0004722	27	1	0.258585861570435	protein serine/threonine phosphatase activity
GO:0008022	28	1	0.266768380441057	protein C-terminus binding
GO:0016757	165	3	0.272880909749711	transferase activity, transferring glycosyl groups
GO:0016763	29	1	0.2748614446746	transferase activity, transferring pentosyl groups
GO:0015457	29	1	0.2748614446746	auxiliary transport protein activity
GO:0015082	30	1	0.282866022924092	di-, tri-valent inorganic cation transporter activity
GO:0046983	170	3	0.287876441393416	protein dimerization activity
GO:0019825	31	1	0.290783073454317	oxygen binding
GO:0016209	31	1	0.290783073454317	antioxidant activity
GO:0031072	31	1	0.290783073454317	heat shock protein binding
GO:0008137	32	1	0.29861354425214	NADH dehydrogenase (ubiquinone) activity
GO:0050136	32	1	0.29861354425214	NADH dehydrogenase (quinone) activity
GO:0003954	32	1	0.29861354425214	NADH dehydrogenase activity
GO:0000287	251	4	0.298631465840222	magnesium ion binding
GO:0003735	100	2	0.301530643626308	structural constituent of ribosome
GO:0016887	255	4	0.308586203200424	ATPase activity
GO:0019888	35	1	0.321594806235546	protein phosphatase regulator activity
GO:0016655	35	1	0.321594806235546	oxidoreductase activity, acting on NADH or NADPH, quinone or similar compound as acceptor
GO:0019208	36	1	0.329088236210853	phosphatase regulator activity
GO:0015081	36	1	0.329088236210853	sodium ion transporter activity
GO:0016627	37	1	0.336499676000212	oxidoreductase activity, acting on the CH-CH group of donors
GO:0004857	187	3	0.339210290160367	enzyme inhibitor activity
GO:0015075	523	7	0.35308672828051	ion transporter activity
GO:0008009	40	1	0.358250892387659	chemokine activity
GO:0042379	40	1	0.358250892387659	chemokine receptor binding
GO:0016758	115	2	0.362014493278215	transferase activity, transferring hexosyl groups
GO:0005096	117	2	0.369950009874722	GTPase activator activity
GO:0001664	42	1	0.372357790491809	G-protein-coupled receptor binding
GO:0005496	42	1	0.372357790491809	steroid binding
GO:0019829	42	1	0.372357790491809	cation-transporting ATPase activity
GO:0016836	43	1	0.379295618894626	hydro-lyase activity
GO:0050660	44	1	0.386157480340832	FAD binding
GO:0005262	47	1	0.406295457803961	calcium channel activity
GO:0016835	50	1	0.425778867192563	carbon-oxygen lyase activity
GO:0005525	226	3	0.45505629692424	GTP binding
GO:0017111	410	5	0.472821964562536	nucleoside-triphosphatase activity
GO:0005319	58	1	0.474692691894047	lipid transporter activity
GO:0019001	235	3	0.480735238045244	guanyl nucleotide binding
GO:0043169	2313	26	0.488169648916217	cation binding
GO:0008135	62	1	0.497578429408503	translation factor activity, nucleic acid binding
GO:0004867	64	1	0.508648127576082	serine-type endopeptidase inhibitor activity
GO:0016462	430	5	0.515159228234868	pyrophosphatase activity

GO:0005554	522	6	0.516645561970296	molecular function unknown
GO:0016818	432	5	0.519321279376084	hydrolase activity, acting on acid anhydrides, in phosphorus-containing anhydrides
GO:0016817	433	5	0.521396914563245	hydrolase activity, acting on acid anhydrides
GO:0031402	70	1	0.54042826874047	sodium ion binding
GO:0043167	2556	28	0.54883641936801	ion binding
GO:0045182	72	1	0.550562375430525	translation regulator activity
GO:0016810	72	1	0.550562375430525	hydrolase activity, acting on carbon-nitrogen (but not peptide) bonds
GO:0046906	74	1	0.560475094658884	tetrapyrrole binding
GO:0020037	74	1	0.560475094658884	heme binding
GO:0016791	171	2	0.564748368114972	phosphoric monoester hydrolase activity
GO:0016616	77	1	0.574939523933872	oxidoreductase activity, acting on the CH-OH group of donors, NAD or NADP as acceptor
GO:0046872	2495	27	0.578083149306388	metal ion binding
GO:0004197	80	1	0.588932325156036	cysteine-type endopeptidase activity
GO:0016614	83	1	0.602468733181547	oxidoreductase activity, acting on CH-OH group of donors
GO:0015276	84	1	0.606882011148975	ligand-gated ion channel activity
GO:0030955	86	1	0.61556349536436	potassium ion binding
GO:0008047	188	2	0.616650661573539	enzyme activator activity
GO:0005179	90	1	0.632360780878306	hormone activity
GO:0048503	92	1	0.640484726331006	GPI anchor binding
GO:0016853	92	1	0.640484726331006	isomerase activity
GO:0008415	98	1	0.663804821996964	acyltransferase activity
GO:0008234	98	1	0.663804821996964	cysteine-type peptidase activity
GO:0016747	98	1	0.663804821996964	transferase activity, transferring groups other than amino-acyl groups
GO:0030234	509	5	0.66608522650504	enzyme regulator activity
GO:0004866	99	1	0.667543093064455	endopeptidase inhibitor activity
GO:0030414	100	1	0.671240188513325	protease inhibitor activity
GO:0005506	212	2	0.68174655384131	iron ion binding
GO:0016746	104	1	0.685625726031439	transferase activity, transferring acyl groups
GO:0004252	105	1	0.689123585389996	serine-type endopeptidase activity
GO:0030695	219	2	0.6989708614665	GTPase regulator activity
GO:0042578	228	2	0.719987571822512	phosphoric ester hydrolase activity
GO:0008236	117	1	0.728208011665466	serine-type peptidase activity
GO:0004721	118	1	0.731236254319873	phosphoprotein phosphatase activity
GO:0003924	121	1	0.740121906692457	GTPase activity
GO:0016829	127	1	0.757029463830213	lyase activity
GO:0035091	132	1	0.770283289549733	phosphoinositide binding
GO:0008083	144	1	0.799241945343975	growth factor activity
GO:0005083	144	1	0.799241945343975	small GTPase regulator activity
GO:0031420	146	1	0.803703568322934	alkali metal ion binding
GO:0015290	149	1	0.810212490862287	electrochemical potential-driven transporter activity
GO:0015291	149	1	0.810212490862287	porter activity
GO:0019899	151	1	0.814432538676264	enzyme binding
GO:0004175	288	2	0.830754214251723	endopeptidase activity
GO:0004713	161	1	0.834178273668737	protein-tyrosine kinase activity
GO:0008289	298	2	0.844860446794327	lipid binding
GO:0005543	169	1	0.848464150717042	phospholipid binding

GO:0005125	181	1	0.86763800555935	cytokine activity
GO:0030246	181	1	0.86763800555935	carbohydrate binding
GO:0016787	1424	12	0.877368553067538	hydrolase activity
GO:0005261	188	1	0.877691606088298	cation channel activity
GO:0016874	221	1	0.915787896809466	ligase activity
GO:0005102	528	3	0.935398852181064	receptor binding
GO:0008233	399	2	0.938060614673986	peptidase activity
GO:0005216	252	1	0.940763509931882	ion channel activity
GO:0003824	3409	30	0.951652808352084	catalytic activity
GO:0015268	274	1	0.953884203220335	alpha-type channel activity
GO:0004871	1837	14	0.957899656092327	signal transducer activity
GO:0001584	282	1	0.957903725230915	rhodopsin-like receptor activity
GO:0015267	284	1	0.958853132757218	channel or pore class transporter activity
GO:0016788	446	2	0.96040549265087	hydrolase activity, acting on ester bonds
GO:0008134	293	1	0.96286964378044	transcription factor binding
GO:0016301	609	3	0.967716980090058	kinase activity
GO:0004674	326	1	0.974543813442787	protein serine/threonine kinase activity
GO:0008270	1287	8	0.979118975310769	zinc ion binding
GO:0016740	1169	7	0.979149181961602	transferase activity
GO:0004930	349	1	0.98044825381897	G-protein coupled receptor activity
GO:0004872	1060	6	0.980977470053423	receptor activity
GO:0004888	694	3	0.984972105954175	transmembrane receptor activity
GO:0016772	708	3	0.986792926937472	transferase activity, transferring phosphorus-containing groups
GO:0046914	1596	10	0.987840416340255	transition metal ion binding
GO:0003723	436	1	0.992837827196606	RNA binding
GO:0004672	447	1	0.993696201575862	protein kinase activity
GO:0003700	649	2	0.994853308489825	transcription factor activity
GO:0016773	532	1	0.997661728475477	phosphotransferase activity, alcohol group as acceptor
GO:0030528	963	2	0.99982949561147	transcription regulator activity
GO:0003677	1384	2	0.999998767959172	DNA binding
GO:0003676	1990	5	0.99999947712543	nucleic acid binding
GO:0003674	9541	105	1	molecular_function

4.4 GO: Cellular Component

GO ID	genes present in chip	genes present in tissues	p-value	GO term
GO:0043292	40	18	1.05893028109436e-26	contractile fiber
GO:0030017	28	16	4.83600059374419e-26	sarcomere
GO:0030016	32	16	9.27009785548102e-25	myofibril
GO:0015629	183	19	7.7425378834059e-15	actin cytoskeleton
GO:0005863	15	8	3.82587220651185e-13	striated muscle thick filament
GO:0005859	15	8	3.82587220651185e-13	muscle myosin
GO:0016460	15	8	3.82587220651185e-13	myosin II
GO:0005865	10	6	1.54625077035568e-10	striated muscle thin filament
GO:0016459	43	9	2.41306584655027e-10	myosin
GO:0005856	612	24	2.40689408254103e-09	cytoskeleton
GO:0005790	10	5	1.98761562467349e-08	smooth endoplasmic reticulum
GO:0005737	2530	49	4.23681190201095e-08	cytoplasm
GO:0043228	977	26	1.23260145672958e-06	non-membrane-bound organelle
GO:0043232	977	26	1.23260145672958e-06	intracellular non-membrane-bound organelle
GO:0005861	5	3	9.07048501191093e-06	troponin complex
GO:0030018	3	2	0.000285332989417021	Z disc
GO:0005884	17	3	0.000566231597207604	actin filament
GO:0005581	30	3	0.00308299153739349	collagen
GO:0005622	5419	65	0.00483145859271794	intracellular
GO:0005739	490	11	0.00827594540622566	mitochondrion
GO:0043033	1	1	0.00984138010883473	isoamylase complex
GO:0005582	1	1	0.00984138010883473	collagen type XV
GO:0005746	21	2	0.0178050678989502	mitochondrial electron transport chain
GO:0005589	2	1	0.0195870358164245	collagen type VI
GO:0005862	3	1	0.0292378872299283	muscle thin filament tropomyosin
GO:0000015	3	1	0.0292378872299283	phosphopyruvate hydratase complex
GO:0005578	303	7	0.0292668582435527	extracellular matrix (sensu Metazoa)
GO:0005740	119	4	0.0295298587415707	mitochondrial membrane
GO:0031012	304	7	0.0297242062113077	extracellular matrix
GO:0043234	1247	19	0.0318967324965796	protein complex
GO:0001527	4	1	0.0387948457179357	microfibril
GO:0043205	5	1	0.0482588139924601	fibril
GO:0016011	5	1	0.0482588139924601	dystroglycan complex
GO:0016012	5	1	0.0482588139924601	sarcoglycan complex
GO:0016599	5	1	0.0482588139924601	caveolar membrane
GO:0005743	85	3	0.0510683261991899	mitochondrial inner membrane
GO:0019866	89	3	0.05712057594594	inner membrane
GO:0005901	6	1	0.0576306861901718	caveola
GO:0001725	6	1	0.0576306861901718	stress fiber
GO:0005890	7	1	0.0669113479528687	sodium:potassium-exchanging ATPase complex
GO:0016528	7	1	0.0669113479528687	sarcoplasm
GO:0016529	7	1	0.0669113479528687	sarcoplasmic reticulum

GO:0030934	8	1	0.0761016765072143	anchoring collagen
GO:0043226	4515	51	0.0924161202349238	organelle
GO:0043229	4515	51	0.0924161202349238	intracellular organelle
GO:0005583	10	1	0.0942148012950489	fibrillar collagen
GO:0016010	10	1	0.0942148012950489	dystrophin-associated glycoprotein complex
GO:0005759	62	2	0.124134435293235	mitochondrial matrix
GO:0045121	14	1	0.129394734596985	lipid raft
GO:0000794	15	1	0.137976602573242	condensed nuclear chromosome
GO:0048471	22	1	0.195753171719564	perinuclear region
GO:0000793	23	1	0.203688282619603	condensed chromosome
GO:0008305	24	1	0.211546013752332	integrin complex
GO:0005605	24	1	0.211546013752332	basal lamina
GO:0005840	113	2	0.305834046891861	ribosome
GO:0005576	949	11	0.329461396133485	extracellular region
GO:0005783	483	6	0.339280531986877	endoplasmic reticulum
GO:0000228	42	1	0.340569918478935	nuclear chromosome
GO:0005604	44	1	0.353549005309115	basement membrane
GO:0019867	52	1	0.402988334513665	outer membrane
GO:0043235	58	1	0.437597835922528	receptor complex
GO:0045111	65	1	0.47547588315747	intermediate filament cytoskeleton
GO:0005882	65	1	0.47547588315747	intermediate filament
GO:0005777	67	1	0.48582727716428	peroxisome
GO:0042579	67	1	0.48582727716428	microbody
GO:0031090	377	4	0.511980030040587	organelle membrane
GO:0005615	385	4	0.528714136116993	extracellular space
GO:0005624	521	5	0.588504163551878	membrane fraction
GO:0005667	91	1	0.595355859866609	transcription factor complex
GO:0005792	108	1	0.658647218847905	microsome
GO:0042598	108	1	0.658647218847905	vesicular fraction
GO:0000267	698	6	0.693958504923696	cell fraction
GO:0005623	7802	76	0.695168982735077	cell
GO:0030529	254	2	0.718734325178845	ribonucleoprotein complex
GO:0005887	1057	9	0.728097021040705	integral to plasma membrane
GO:0031226	1062	9	0.733376949778519	intrinsic to plasma membrane
GO:0008372	523	4	0.765228947866322	cellular component unknown
GO:0005886	1472	12	0.804609125291696	plasma membrane
GO:0005625	183	1	0.839479339260074	soluble fraction
GO:0005694	203	1	0.868883261137926	chromosome
GO:0005654	221	1	0.890757060817327	nucleoplasm
GO:0005829	276	1	0.937607048236368	cytosol
GO:0016021	2674	20	0.948990086640126	integral to membrane
GO:0031224	2681	20	0.950628327057412	intrinsic to membrane
GO:0016020	3514	26	0.979500017156196	membrane
GO:0043227	3955	26	0.998576925860449	membrane-bound organelle
GO:0043231	3955	26	0.998576925860449	intracellular membrane-bound organelle
GO:0005634	2704	9	0.99999857047387	nucleus

GO:0005575	8637	85	1	cellular_component

5. Kidney

5.1 Kidney selective genes (141 genes)

Symbol	Description	Chromosome	Chromosome Location	LocusLink	Cytoband	UniGene
SLC3A1	solute carrier family 3 (cystine, dibasic and neutral amino acid transporters, activator of cystine, dibasic and neutral amino acid transport), member 1	2	44356102	6519	2p16.3	Hs.112916
BBOX1	butyrobetaine (gamma), 2-oxoglutarate dioxygenase (gamma-butyrobetaine hydroxylase) 1	11	27019084	8424	11p14.2	Hs.591996
PCK1	phosphoenolpyruvate carboxykinase 1 (soluble)	20	55569542	5105	20q13.31	Hs.1872
PDZK1	PDZ domain containing 1	1	144439082	5174	1q21	Hs.444751 Hs.515949
ALDOB	aldolase B, fructose-bisphosphate	9	-103223479	229	9q21.3-q22.2	Hs.530274
PAH	phenylalanine hydroxylase	12	-101756234	5053	12q22-q24.2	Hs.643451
NAT8	N-acetyltransferase 8 (camello like)	2	-73721467	9027	2p13.1-p12	Hs.14637
KL	klotho	13	32488570	9365	13q12	Hs.524953
UMOD	uromodulin (uromucoid, Tamm-Horsfall glycoprotein)	16	-20251874	7369	16p12.3	Hs.164470
NOX4	NADPH oxidase 4	11	-88699160	50507	11q14.2-q21	Hs.371036
KCNJ16	potassium inwardly-rectifying channel, subfamily J, member 16	17	65583020, 65612589	3773	17q23.1-q24.2	Hs.463985
CALB1	calbindin 1, 28kDa	8	-91140013	793	8q21.3-q22.1	Hs.65425
ALDH8A1	aldehyde dehydrogenase 8 family, member A1	6	-135280221	64577	6q23.2	Hs.486520
MAWBP	NA	10	-139427265	64081	10pter-q25.3	Hs.198158
DEFB1	defensin, beta 1	8	-6715510	1672	8p23.2-p23.1	Hs.32949
GSTA1	glutathione S-transferase A1	6	-52764347	2938	6p12.1	Hs.446309
TRBV9	T cell receptor beta variable 9	7	NA	28586	7q34	NA
CXCL14	chemokine (C-X-C motif) ligand 14	5	-134934274	9547	5q31	Hs.483444
DPYS	dihydropyrimidinase	8	-105460828	1807	8q22	Hs.443161
EHHADH	enoyl-Coenzyme A, hydratase/3-hydroxyacyl Coenzyme A dehydrogenase	3	-186391107	1962	3q26.3-q28	Hs.429879
PDZK1IP1	PDZK1 interacting protein 1	1	-47421847	10158	1p33	Hs.431099
PCCA	propionyl Coenzyme A carboxylase, alpha polypeptide	13	99539337	5095	13q32	Hs.80741
TACSTD1	tumor-associated calcium signal transducer 1	2	47449970	4072	2p21	Hs.542050
FABP1	fatty acid binding protein 1, liver	2	-88203624	2168	2p11	Hs.380135
C14orf105	chromosome 14 open reading frame 105	14	-57006347	55195	14q23.1	Hs.146040
ANPEP	alanyl (membrane) aminopeptidase (aminopeptidase N, aminopeptidase M, microsomal aminopeptidase, CD13, p150)	15	-88129129	290	15q25-q26	Hs.1239
ABP1	amiloride binding protein 1 (amine oxidase (copper-containing))	7	150180505	26	7q34-q36	Hs.438823
HPD	4-hydroxyphenylpyruvate dioxygenase	12	-120761817	3242	12q24-qter	Hs.2899
SLC13A1	solute carrier family 13 (sodium/sulfate symporters), member 1	7	-122540824	6561	7q31-q32	Hs.489849

DIO1	deiodinase, iodothyronine, type I	1	54132448	1733	1p33-p32	Hs.251415
ENPEP	glutamyl aminopeptidase (aminopeptidase A)	4	111616773	2028	4q25	Hs.435765
ALB	albumin	4	74488869	213	4q11-q13	Hs.418167
CUBN	cubilin (intrinsic factor-cobalamin receptor)	10	-16906543	8029	10p12.31	Hs.166206
BHMT2	betaine-homocysteine methyltransferase 2	5	78401338	23743	5q13	Hs.114172
SPP1	secreted phosphoprotein 1 (osteopontin, bone sialoprotein I, early T-lymphocyte activation 1)	4	89115825	6696	4q21-q25	Hs.313
FXYD2	FXYD domain containing ion transport regulator 2	11	-117195999	486	11q23	Hs.413137
FLJ20273	NA	4	-40120497	54502	4p13-p12	Hs.518727
AZGP1	alpha-2-glycoprotein 1, zinc	7	-99402287	563	7q22.1	Hs.546239
HSD11B2	hydroxysteroid (11-beta) dehydrogenase 2	16	66022536	3291	16q22	Hs.1376
ACY1	aminoacylase 1	3	51992602	95	3p21.1	Hs.334707
UGT1A10	UDP glucuronosyltransferase 1 family, polypeptide A10	2	234209861	54575	2q37	Hs.124112
UGT1A7	UDP glucuronosyltransferase 1 family, polypeptide A7	2	234255322	54577	2q37	Hs.124112
UGT1A6	UDP glucuronosyltransferase 1 family, polypeptide A6	2	234266293, 234265059	54578	2q37	Hs.124112
UGT1A5	UDP glucuronosyltransferase 1 family, polypeptide A5	2	234286376	54579	2q37	Hs.124112
UGT1A9	UDP glucuronosyltransferase 1 family, polypeptide A9	2	234245282	54600	2q37	Hs.124112
UGT1A4	UDP glucuronosyltransferase 1 family, polypeptide A4	2	234292176	54657	2q37	Hs.124112
UGT1A3	UDP glucuronosyltransferase 1 family, polypeptide A3	2	234302511	54659	2q37	Hs.124112
FMO1	flavin containing monooxygenase 1	1	169484286	2326	1q23-q25	Hs.1424
UGT1A8	UDP glucuronosyltransferase 1 family, polypeptide A8	2	234191029	54576	2q37	Hs.124112
NA	NA	NA	NA	650893	NA	NA
AKR1C3	aldo-keto reductase family 1, member C3 (3-alpha hydroxysteroid dehydrogenase, type II)	10	5126567	8644	10p15-p14	Hs.78183
KNG1	kininogen 1	3	187917813	3827	3q27	Hs.77741
S100A2	S100 calcium binding protein A2	1	-151800210	6273	1q21	Hs.516484
LRP2	low density lipoprotein-related protein 2	2	-169693106	4036	2q24-q31	Hs.470538
TOM1L1	target of myb1-like 1 (chicken)	17	50333202	10040	17q23.2	Hs.153504
UGT1A1	UDP glucuronosyltransferase 1 family, polypeptide A1	2	234333657	54658	2q37	Hs.124112
EMX2	empty spiracles homolog 2 (Drosophila)	10	119291945	2018	10q26.1	Hs.202095
MMP7	matrix metallopeptidase 7 (matrilysin, uterine)	11	-101896449	4316	11q21-q22	Hs.2256
FLJ10847	NA	17	19377758	55244	17p11.2	Hs.232054
BDH2	3-hydroxybutyrate dehydrogenase, type 2	4	-104218231	56898	4q24	Hs.124696
RBP4	retinol binding protein 4, plasma	10	-95341583	5950	10q23-q24	Hs.50223
SLC27A2	solute carrier family 27 (fatty acid transporter), member 2	15	48261713	11001	15q21.2	Hs.11729
SH3YL1	SH3 domain containing, Ysc84-like 1 (S. cerevisiae)	2	-208154	26751	2p25.3	Hs.515951
EGF	epidermal growth factor (beta-urogastrone)	4	111053498	1950	4q25	Hs.419815
PHYH	phytanoyl-CoA 2-hydroxylase	10	-13359802	5264	10pter-p11.2	Hs.498732
LR8	NA	7	-150119323	28959	7q36.1	Hs.438823
CDH16	cadherin 16, KSP-cadherin	16	-65499526	1014	16q22.1	Hs.513660
SERPINA1	serpin peptidase inhibitor, clade A (alpha-1 antiproteinase, antitrypsin), member 1	14	-93914450	5265	14q32.1	Hs.525557
DPEP1	dipeptidase 1 (renal)	16	88214500	1800	16q24.3	Hs.109
PTHR1	parathyroid hormone receptor 1	3	46894239	5745	3p22-p21.1	Hs.1019
IMPA2	inositol(myo)-1(or 4)-monophosphatase 2	18	11971454	3613	18p11.2	Hs.367992

GDF15	growth differentiation factor 15	19	18357984	9518	19p13.1-13.2	Hs.515258
LGALS2	lectin, galactoside-binding, soluble, 2 (galectin 2)	22	-36296199	3957	22q12-q13|22q13.1	Hs.531776
ACE2	angiotensin I converting enzyme (peptidyl-dipeptidase A) 2	X	-15489076	59272	Xp22	Hs.178098
ALDH1A1	aldehyde dehydrogenase 1 family, member A1	9	-74705407	216	9q21.13	Hs.76392
VCAM1	vascular cell adhesion molecule 1	1	100957884	7412	1p32-p31	Hs.109225
FLJ22028	NA	12	21481877	79912	12p12.1	Hs.46835
SLC22A6	solute carrier family 22 (organic anion transporter), member 6	11	-62500645	9356	11q13.1-q13.2	Hs.369252
PLG	plasminogen	6	161043272	5340	6q26	Hs.143436
ASS	argininosuccinate synthetase	9	132315479, 132310092	445	9q34.1	Hs.160786
CFI	complement factor I	4	-110881300	3426	4q25	Hs.312485
SULT1C1	sulfotransferase family, cytosolic, 1C, member 1	2	108271526	6819	2q11.1-q11.2	Hs.436123
FBP1	fructose-1,6-bisphosphatase 1	9	-96405237	2203	9q22.3	Hs.494496
CA2	carbonic anhydrase II	8	86563497	760	8q22	Hs.155097
NQO2	NAD(P)H dehydrogenase, quinone 2	6	2945229	4835	6pter-q12	Hs.533050
RHOBTB1	Rho-related BTB domain containing 1	10	-62299205	9886	10q21.2	Hs.148670
G6PC	glucose-6-phosphatase, catalytic subunit	17	38306340	2538	17q21	Hs.212293
SLC17A3	solute carrier family 17 (sodium phosphate), member 3	6	-25953307	10786	6p21.3	Hs.327179
COTL1	coactosin-like 1 (Dictyostelium)	16	-83156706	23406	16q24.1	Hs.289092
GALNT11	UDP-N-acetyl-alpha-D-galactosamine:polypeptide N-acetylgalactosaminyltransferase 11 (GalNAc-T11)	7	151353796	63917	7q34-q36	Hs.438823
BHMT	betaine-homocysteine methyltransferase	5	78443438	635	5q13.1-q15	Hs.80756
MT1H	metallothionein 1H	16	55261274	4496	16q13	Hs.438462
CRYZ	crystallin, zeta (quinone reductase)	1	-74943771	1429	1p31-p22	Hs.83114
KCNJ15	potassium inwardly-rectifying channel, subfamily J, member 15	21	38550533, 38550739, 38566298	3772	21q22.2	Hs.411299
GATM	glycine amidinotransferase (L-arginine:glycine amidinotransferase)	15	-43440615	2628	15q21.1	Hs.75335
CD2AP	CD2-associated protein	6	47553898	23607	6p12	Hs.485518
PODXL	podocalyxin-like	7	-130835562	5420	7q32-q33	Hs.16426
ECHDC3	enoyl Coenzyme A hydratase domain containing 3	10	11824388	79746	10p14	Hs.22242
CRIM1	cysteine rich transmembrane BMP regulator 1 (chordin-like)	2	36436900	51232	2p21	Hs.332847
PCK2	phosphoenolpyruvate carboxykinase 2 (mitochondrial)	14	23633322	5106	14q12	Hs.75812
CD24	CD24 molecule	6	-19611913	934	6q21	Hs.375108
HRSP12	heat-responsive protein 12	8	-99183742	10247	8q22	Hs.18426
ACADM	acyl-Coenzyme A dehydrogenase, C-4 to C-12 straight chain	1	75962869	34	1p31	Hs.445040
MT1M	metallothionein 1M	16	55224072	4499	16q13	Hs.643532
SLC7A7	solute carrier family 7 (cationic amino acid transporter, y+ system), member 7	14	-22312273	9056	14q11.2	Hs.513147
XPNPEP2	X-prolyl aminopeptidase (aminopeptidase P) 2, membrane-bound	X	128700626	7512	Xq25	Hs.170499
FLRT3	fibronectin leucine rich transmembrane protein 3	20	-14252642	23767	20p11	Hs.41296
MME	membrane metallo-endopeptidase (neutral endopeptidase, enkephalinase, CALLA, CD10)	3	156280152, 156280327, 156280748	4311	3q25.1-q25.2	Hs.307734
HIBCH	3-hydroxyisobutyryl-Coenzyme A hydrolase	2	-190777604	26275	2q32.2	Hs.128598
C7	complement component 7	5	40945355	730	5p13	Hs.78065

GRB14	growth factor receptor-bound protein 14	2	-165057578	2888	2q22-q24	Hs.411881
SLC34A1	solute carrier family 34 (sodium phosphate), member 1	5	176744060	6569	5q35	Hs.936
GPX3	glutathione peroxidase 3 (plasma)	5	150380191	2878	5q23	Hs.386793 Hs.570891
SLC7A9	solute carrier family 7 (cationic amino acid transporter, y+ system), member 9	19	-38013261	11136	19q13.1	Hs.408567
SFRP1	secreted frizzled-related protein 1	8	-41238635	6422	8p12-p11.1	Hs.213424
HPN	hepsin (transmembrane protease, serine 1)	19	40223249	3249	19q11-q13.2	Hs.182385
SUCLG2	succinate-CoA ligase, GDP-forming, beta subunit	3	-67507835	8801	3p14.1	Hs.186512
IGHA1	immunoglobulin heavy constant alpha 1	14	NA	3493	14q32.33	Hs.510635
NPHS2	nephrosis 2, idiopathic, steroid-resistant (podocin)	1	-177786298	7827	1q25-q31	Hs.412710
PROS1	protein S (alpha)	3	-95074646	5627	3q11.2	Hs.64016
SERPINA5	serpin peptidase inhibitor, clade A (alpha-1 antiproteinase, antitrypsin), member 5	14	94117563	5104	14q32.1	Hs.510334
DSP	desmoplakin	6	7486868	1832	6p24	Hs.519873
SDC1	syndecan 1	2	-20264040	6382	2p24.1	Hs.224607
TNFSF10	tumor necrosis factor (ligand) superfamily, member 10	3	-173706158	8743	3q26	Hs.478275
DAB2	disabled homolog 2, mitogen-responsive phosphoprotein (Drosophila)	5	-39408502	1601	5p13	Hs.481980
ALDH6A1	aldehyde dehydrogenase 6 family, member A1	14	-73596625	4329	14q24.3	Hs.293970
KRT18	keratin 18	12	51629109, 51628921	3875	12q13	Hs.406013
DMXL1	Dmx-like 1	5	118435083	1657	5q22	Hs.181042
PEPD	peptidase D	19	-38569698	5184	19q12-q13.2	Hs.36473
PLEKHA5	pleckstrin homology domain containing, family A member 5	12	19173994	54477	12p12	Hs.188614
LUM	lumican	12	-90021363	4060	12q21.3-q22	Hs.406475
VAMP8	vesicle-associated membrane protein 8 (endobrevin)	2	85658227	8673	2p12-p11.2	Hs.534373
CA12	carbonic anhydrase XII	15	-61402783	771	15q22	Hs.210995
UPB1	ureidopropionase, beta	22	23221250	51733	22q11.2	Hs.474388
LAMB1	laminin, beta 1	7	-107351498	3912	7q22	Hs.489646
NPY1R	neuropeptide Y receptor Y1	4	-164464566	4886	4q31.3-q32	Hs.519057
AKR7A3	aldo-keto reductase family 7, member A3 (aflatoxin aldehyde reductase)	1	-19481643	22977	1p35.1-p36.23	Hs.6980
PLS3	plastin 3 (T isoform)	X	114701764	5358	Xq23	Hs.496622
MGAM	maltase-glucoamylase (alpha-glucosidase)	7	141342147	8972	7q34	Hs.122785
SLC22A8	solute carrier family 22 (organic anion transporter), member 8	11	-62516872	9376	11q11	Hs.266223
MGC14376	NA	17	-1561553	84981	17p13.3	Hs.417157

5.2 GO: Biological Process

GO ID	genes present in chip	genes present in tissues	p-value	GO term
GO:0019752	359	22	1.22E-09	carboxylic acid metabolism
GO:0006082	361	22	1.35E-09	organic acid metabolism
GO:0006519	216	13	4.87E-06	amino acid and derivative metabolism
GO:0006807	291	15	5.90E-06	nitrogen compound metabolism
GO:0009308	269	14	1.11E-05	amine metabolism
GO:0006520	181	10	0.000131245	amino acid metabolism
GO:0006814	80	6	0.000626076	sodium ion transport
GO:0044248	700	20	0.000758184	cellular catabolism
GO:0009056	756	21	0.000795277	catabolism
GO:0006631	124	7	0.001219389	fatty acid metabolism
GO:0006094	17	3	0.001325148	gluconeogenesis
GO:0006090	18	3	0.001574978	pyruvate metabolism
GO:0006091	446	14	0.002126377	generation of precursor metabolites and energy
GO:0042221	183	8	0.002821114	response to chemical substance
GO:0019319	22	3	0.00286052	hexose biosynthesis
GO:0046364	22	3	0.00286052	monosaccharide biosynthesis
GO:0046165	22	3	0.00286052	alcohol biosynthesis
GO:0006805	23	3	0.003258247	xenobiotic metabolism
GO:0009605	963	23	0.003291302	response to external stimulus
GO:0030500	7	2	0.00346201	regulation of bone mineralization
GO:0043285	474	14	0.00370132	biopolymer catabolism
GO:0006558	8	2	0.004576342	L-phenylalanine metabolism
GO:0006559	8	2	0.004576342	L-phenylalanine catabolism
GO:0007568	8	2	0.004576342	aging
GO:0030278	8	2	0.004576342	regulation of ossification
GO:0046850	8	2	0.004576342	regulation of bone remodeling
GO:0009410	26	3	0.004648121	response to xenobiotic stimulus
GO:0006092	88	5	0.005992656	main pathways of carbohydrate metabolism
GO:0006508	451	13	0.006284511	proteolysis and peptidolysis
GO:0044257	451	13	0.006284511	cellular protein catabolism
GO:0044265	563	15	0.006916127	cellular macromolecule catabolism
GO:0005975	355	11	0.007020998	carbohydrate metabolism
GO:0009074	10	2	0.007229153	aromatic amino acid family catabolism
GO:0030282	10	2	0.007229153	bone mineralization
GO:0015849	59	4	0.007465661	organic acid transport
GO:0046942	59	4	0.007465661	carboxylic acid transport
GO:0030163	463	13	0.007777293	protein catabolism
GO:0015980	132	6	0.007853749	energy derivation by oxidation of organic compounds
GO:0050878	94	5	0.00789066	regulation of body fluids
GO:0009057	575	15	0.008339416	macromolecule catabolism
GO:0006629	469	13	0.008622615	lipid metabolism
GO:0030193	11	2	0.00875993	regulation of blood coagulation

GO:0030195	11	2	0.00875993	negative regulation of blood coagulation
GO:0015672	223	8	0.009145493	monovalent inorganic cation transport
GO:0015711	13	2	0.012211535	organic anion transport
GO:0019439	13	2	0.012211535	aromatic compound catabolism
GO:0050818	13	2	0.012211535	regulation of coagulation
GO:0050819	13	2	0.012211535	negative regulation of coagulation
GO:0006081	13	2	0.012211535	aldehyde metabolism
GO:0006000	13	2	0.012211535	fructose metabolism
GO:0016051	69	4	0.012829377	carbohydrate biosynthesis
GO:0015742	1	1	0.013175376	alpha-ketoglutarate transport
GO:0006573	1	1	0.013175376	valine metabolism
GO:0031335	1	1	0.013175376	regulation of sulfur amino acid metabolism
GO:0045329	1	1	0.013175376	carnitine biosynthesis
GO:0006982	1	1	0.013175376	response to lipid hydroperoxide
GO:0018987	1	1	0.013175376	osmoregulation
GO:0019859	1	1	0.013175376	thymine metabolism
GO:0050891	1	1	0.013175376	body fluid osmoregulation
GO:0009437	1	1	0.013175376	carnitine metabolism
GO:0005982	1	1	0.013175376	starch metabolism
GO:0005983	1	1	0.013175376	starch catabolism
GO:0050666	1	1	0.013175376	regulation of homocysteine metabolism
GO:0050667	1	1	0.013175376	homocysteine metabolism
GO:0006789	1	1	0.013175376	bilirubin conjugation
GO:0006855	1	1	0.013175376	multidrug transport
GO:0019835	14	2	0.014125106	cytolysis
GO:0007588	40	3	0.015460068	excretion
GO:0006635	15	2	0.016159139	fatty acid beta-oxidation
GO:0006575	41	3	0.016523384	amino acid derivative metabolism
GO:0008152	5262	81	0.017294072	metabolism
GO:0006865	43	3	0.018770451	amino acid transport
GO:0006006	79	4	0.020181015	glucose metabolism
GO:0030574	17	2	0.020574853	collagen catabolism
GO:0042445	46	3	0.02244346	hormone metabolism
GO:0009072	18	2	0.022949838	aromatic amino acid family metabolism
GO:0042398	18	2	0.022949838	amino acid derivative biosynthesis
GO:0050671	18	2	0.022949838	positive regulation of lymphocyte proliferation
GO:0044262	265	8	0.023598967	cellular carbohydrate metabolism
GO:0009628	318	9	0.024321577	response to abiotic stimulus
GO:0030001	269	8	0.02552525	metal ion transport
GO:0043070	2	1	0.026178602	regulation of non-apoptotic programmed cell death
GO:0043072	2	1	0.026178602	negative regulation of non-apoptotic programmed cell death
GO:0030501	2	1	0.026178602	positive regulation of bone mineralization
GO:0006599	2	1	0.026178602	phosphagen metabolism
GO:0015811	2	1	0.026178602	L-cystine transport
GO:0015802	2	1	0.026178602	basic amino acid transport
GO:0006600	2	1	0.026178602	creatine metabolism

GO:0006601	2	1	0.026178602	creatine biosynthesis
GO:0001907	2	1	0.026178602	killing of host cells
GO:0045778	2	1	0.026178602	positive regulation of ossification
GO:0019836	2	1	0.026178602	hemolysis
GO:0042396	2	1	0.026178602	phosphagen biosynthesis
GO:0000101	2	1	0.026178602	sulfur amino acid transport
GO:0016244	2	1	0.026178602	non-apoptotic programmed cell death
GO:0044003	2	1	0.026178602	modification of host morphology or physiology
GO:0044004	2	1	0.026178602	disruption of host cells
GO:0006704	2	1	0.026178602	glucocorticoid biosynthesis
GO:0046852	2	1	0.026178602	positive regulation of bone remodeling
GO:0001897	2	1	0.026178602	cytolysis of host cells
GO:0009309	49	3	0.026480101	amine biosynthesis
GO:0044271	49	3	0.026480101	nitrogen compound biosynthesis
GO:0042493	20	2	0.028017818	response to drug
GO:0044403	20	2	0.028017818	symbiosis, mutualism through parasitism
GO:0044404	20	2	0.028017818	symbiotic interaction between host and other organism
GO:0044255	383	10	0.02943849	cellular lipid metabolism
GO:0019883	21	2	0.030704511	antigen presentation, endogenous antigen
GO:0015837	52	3	0.030879471	amine transport
GO:0045087	53	3	0.032426128	innate immune response
GO:0019395	22	2	0.03348891	fatty acid oxidation
GO:0019885	22	2	0.03348891	antigen processing, endogenous antigen via MHC class I
GO:0050670	22	2	0.03348891	regulation of lymphocyte proliferation
GO:0006958	23	2	0.036368021	complement activation, classical pathway
GO:0030502	3	1	0.039011907	negative regulation of bone mineralization
GO:0015889	3	1	0.039011907	cobalamin transport
GO:0006930	3	1	0.039011907	substrate-bound cell migration, cell extension
GO:0000038	3	1	0.039011907	very-long-chain fatty acid metabolism
GO:0009267	3	1	0.039011907	cellular response to starvation
GO:0030146	3	1	0.039011907	diuresis
GO:0030147	3	1	0.039011907	natriuresis
GO:0006449	3	1	0.039011907	regulation of translational termination
GO:0030104	3	1	0.039011907	water homeostasis
GO:0030279	3	1	0.039011907	negative regulation of ossification
GO:0006104	3	1	0.039011907	succinyl-CoA metabolism
GO:0046851	3	1	0.039011907	negative regulation of bone remodeling
GO:0042446	25	2	0.042398703	hormone biosynthesis
GO:0051240	59	3	0.042537705	positive regulation of organismal physiological process
GO:0044419	26	2	0.045544584	interaction between organisms
GO:0006572	4	1	0.051677494	tyrosine catabolism
GO:0015893	4	1	0.051677494	drug transport
GO:0008211	4	1	0.051677494	glucocorticoid metabolism
GO:0050679	4	1	0.051677494	positive regulation of epithelial cell proliferation
GO:0051289	4	1	0.051677494	protein homotetramerization
GO:0042594	4	1	0.051677494	response to starvation

GO:0042554	4	1	0.051677494	superoxide release
GO:0030199	4	1	0.051677494	collagen fibril organization
GO:0046651	28	2	0.052083641	lymphocyte proliferation
GO:0051187	28	2	0.052083641	cofactor catabolism
GO:0006073	28	2	0.052083641	glucan metabolism
GO:0051251	28	2	0.052083641	positive regulation of lymphocyte activation
GO:0006898	28	2	0.052083641	receptor mediated endocytosis
GO:0006118	256	7	0.052091412	electron transport
GO:0050896	1578	28	0.055382607	response to stimulus
GO:0050867	29	2	0.055471469	positive regulation of cell activation
GO:0006956	30	2	0.058934692	complement activation
GO:0006730	30	2	0.058934692	one-carbon compound metabolism
GO:0042127	265	7	0.060514959	regulation of cell proliferation
GO:0019318	113	4	0.061819638	hexose metabolism
GO:0006590	5	1	0.064177536	thyroid hormone generation
GO:0006526	5	1	0.064177536	arginine biosynthesis
GO:0042311	5	1	0.064177536	vasodilation
GO:0009251	5	1	0.064177536	glucan catabolism
GO:0050926	5	1	0.064177536	regulation of positive chemotaxis
GO:0050927	5	1	0.064177536	positive regulation of positive chemotaxis
GO:0050918	5	1	0.064177536	positive chemotaxis
GO:0050930	5	1	0.064177536	induction of positive chemotaxis
GO:0007569	5	1	0.064177536	cell aging
GO:0006415	5	1	0.064177536	translational termination
GO:0006787	5	1	0.064177536	porphyrin catabolism
GO:0001894	5	1	0.064177536	tissue homeostasis
GO:0005996	115	4	0.065089385	monosaccharide metabolism
GO:0006955	684	14	0.065277776	immune response
GO:0006725	73	3	0.071437572	aromatic compound metabolism
GO:0008652	34	2	0.073491575	amino acid biosynthesis
GO:0006929	6	1	0.076514179	substrate-bound cell migration
GO:0042403	6	1	0.076514179	thyroid hormone metabolism
GO:0050921	6	1	0.076514179	positive regulation of chemotaxis
GO:0006576	35	2	0.077294757	biogenic amine metabolism
GO:0009613	458	10	0.079540208	response to pest, pathogen or parasite
GO:0030333	36	2	0.081158885	antigen processing
GO:0007596	78	3	0.083431528	blood coagulation
GO:0043207	463	10	0.084092549	response to external biotic stimulus
GO:0050817	79	3	0.085928259	coagulation
GO:0006812	346	8	0.086058087	cation transport
GO:0006820	128	4	0.088479264	anion transport
GO:0000050	7	1	0.08868954	urea cycle
GO:0019627	7	1	0.08868954	urea metabolism
GO:0050673	7	1	0.08868954	epithelial cell proliferation
GO:0050678	7	1	0.08868954	regulation of epithelial cell proliferation
GO:0042743	7	1	0.08868954	hydrogen peroxide metabolism

GO:0042744	7	1	0.08868954	hydrogen peroxide catabolism
GO:0050920	7	1	0.08868954	regulation of chemotaxis
GO:0018298	7	1	0.08868954	protein-chromophore linkage
GO:0001503	38	2	0.089061089	ossification
GO:0031214	38	2	0.089061089	biomineral formation
GO:0007599	83	3	0.096227434	hemostasis
GO:0050874	1531	26	0.097679705	organismal physiological process
GO:0001906	8	1	0.100705711	cell killing
GO:0008210	8	1	0.100705711	estrogen metabolism
GO:0007589	8	1	0.100705711	fluid secretion
GO:0042542	8	1	0.100705711	response to hydrogen peroxide
GO:0009636	8	1	0.100705711	response to toxin
GO:0006206	8	1	0.100705711	pyrimidine base metabolism
GO:0006749	8	1	0.100705711	glutathione metabolism
GO:0019882	41	2	0.101317161	antigen presentation
GO:0051249	41	2	0.101317161	regulation of lymphocyte activation
GO:0046849	41	2	0.101317161	bone remodeling
GO:0042060	85	3	0.101558195	wound healing
GO:0016477	85	3	0.101558195	cell migration
GO:0009063	42	2	0.105501679	amino acid catabolism
GO:0044264	42	2	0.105501679	cellular polysaccharide metabolism
GO:0050865	43	2	0.109732471	regulation of cell activation
GO:0008285	139	4	0.111018718	negative regulation of cell proliferation
GO:0006835	9	1	0.112564756	dicarboxylic acid transport
GO:0019059	9	1	0.112564756	initiation of viral infection
GO:0008272	9	1	0.112564756	sulfate transport
GO:0042730	9	1	0.112564756	fibrinolysis
GO:0030260	9	1	0.112564756	entry into host cell
GO:0044409	9	1	0.112564756	entry into host
GO:0046718	9	1	0.112564756	viral entry into host cell
GO:0005976	44	2	0.114007617	polysaccharide metabolism
GO:0046483	44	2	0.114007617	heterocycle metabolism
GO:0006952	751	14	0.116918396	defense response
GO:0009310	45	2	0.118325239	amine catabolism
GO:0006570	10	1	0.124268712	tyrosine metabolism
GO:0006525	10	1	0.124268712	arginine metabolism
GO:0009081	10	1	0.124268712	branched chain family amino acid metabolism
GO:0009084	10	1	0.124268712	glutamine family amino acid biosynthesis
GO:0005978	10	1	0.124268712	glycogen biosynthesis
GO:0009250	10	1	0.124268712	glucan biosynthesis
GO:0051262	10	1	0.124268712	protein tetramerization
GO:0044270	47	2	0.127080608	nitrogen compound catabolism
GO:0006790	48	2	0.131514801	sulfur metabolism
GO:0050953	149	4	0.133506429	sensory perception of light
GO:0007601	149	4	0.133506429	visual perception
GO:0006957	11	1	0.13581959	complement activation, alternative pathway

GO:0000051	11	1	0.13581959	urea cycle intermediate metabolism
GO:0007193	11	1	0.13581959	G-protein signaling, adenylate cyclase inhibiting pathway
GO:0000272	11	1	0.13581959	polysaccharide catabolism
GO:0051260	11	1	0.13581959	protein homooligomerization
GO:0016525	11	1	0.13581959	negative regulation of angiogenesis
GO:0044247	11	1	0.13581959	cellular polysaccharide catabolism
GO:0009617	51	2	0.14502291	response to bacteria
GO:0009112	12	1	0.147219376	nucleobase metabolism
GO:0042102	12	1	0.147219376	positive regulation of T cell proliferation
GO:0051180	12	1	0.147219376	vitamin transport
GO:0006066	213	5	0.149804961	alcohol metabolism
GO:0009888	158	4	0.155210592	histogenesis
GO:0009607	857	15	0.155741203	response to biotic stimulus
GO:0015807	13	1	0.15847003	L-amino acid transport
GO:0000302	13	1	0.15847003	response to reactive oxygen species
GO:0030595	13	1	0.15847003	immune cell chemotaxis
GO:0009611	341	7	0.162961449	response to wounding
GO:0050778	56	2	0.168125141	positive regulation of immune response
GO:0008213	14	1	0.169573485	protein amino acid alkylation
GO:0042401	14	1	0.169573485	biogenic amine biosynthesis
GO:0050900	14	1	0.169573485	immune cell migration
GO:0006479	14	1	0.169573485	protein amino acid methylation
GO:0008283	473	9	0.170914453	cell proliferation
GO:0006813	111	3	0.180048866	potassium ion transport
GO:0006801	15	1	0.180531653	superoxide metabolism
GO:0007162	15	1	0.180531653	negative regulation of cell adhesion
GO:0042129	15	1	0.180531653	regulation of T cell proliferation
GO:0018149	15	1	0.180531653	peptide cross-linking
GO:0001501	113	3	0.186661333	skeletal development
GO:0045637	16	1	0.191346417	regulation of myeloid cell differentiation
GO:0007173	16	1	0.191346417	epidermal growth factor receptor signaling pathway
GO:0009618	16	1	0.191346417	response to pathogenic bacteria
GO:0006800	61	2	0.191802531	oxygen and reactive oxygen species metabolism
GO:0007582	7846	107	0.198887861	physiological process
GO:0006521	17	1	0.20201964	regulation of amino acid metabolism
GO:0006692	17	1	0.20201964	prostanoid metabolism
GO:0006693	17	1	0.20201964	prostaglandin metabolism
GO:0006904	17	1	0.20201964	vesicle docking during exocytosis
GO:0043284	17	1	0.20201964	biopolymer biosynthesis
GO:0042098	17	1	0.20201964	T cell proliferation
GO:0050880	17	1	0.20201964	regulation of blood vessel size
GO:0000271	17	1	0.20201964	polysaccharide biosynthesis
GO:0001525	65	2	0.211045642	angiogenesis
GO:0048278	18	1	0.212553158	vesicle docking
GO:0030216	18	1	0.212553158	keratinocyte differentiation
GO:0006778	18	1	0.212553158	porphyrin metabolism

GO:0008284	121	3	0.213708275	positive regulation of cell proliferation
GO:0006939	19	1	0.222948784	smooth muscle contraction
GO:0009991	19	1	0.222948784	response to extracellular stimulus
GO:0001568	68	2	0.225602652	blood vessel development
GO:0048514	68	2	0.225602652	blood vessel morphogenesis
GO:0006954	185	4	0.227069343	inflammatory response
GO:0001944	69	2	0.23047275	vasculature development
GO:0000096	20	1	0.233208309	sulfur amino acid metabolism
GO:0051241	20	1	0.233208309	negative regulation of organismal physiological process
GO:0007631	20	1	0.233208309	feeding behavior
GO:0006100	20	1	0.233208309	tricarboxylic acid cycle intermediate metabolism
GO:0050870	21	1	0.243333499	positive regulation of T cell activation
GO:0000270	21	1	0.243333499	peptidoglycan metabolism
GO:0008202	132	3	0.252133652	steroid metabolism
GO:0006461	194	4	0.252729242	protein complex assembly
GO:0006099	22	1	0.253326098	tricarboxylic acid cycle
GO:0046356	22	1	0.253326098	acetyl-CoA catabolism
GO:0051239	133	3	0.255680768	regulation of organismal physiological process
GO:0006953	23	1	0.263187829	acute-phase response
GO:0009109	23	1	0.263187829	coenzyme catabolism
GO:0006950	881	14	0.269238015	response to stress
GO:0007267	465	8	0.269418687	cell-cell signaling
GO:0050776	77	2	0.269606773	regulation of immune response
GO:0042088	24	1	0.272920391	T-helper 1 type immune response
GO:0051259	24	1	0.272920391	protein oligomerization
GO:0016052	79	2	0.279404247	carbohydrate catabolism
GO:0044275	79	2	0.279404247	cellular carbohydrate catabolism
GO:0045765	25	1	0.282525461	regulation of angiogenesis
GO:0042087	25	1	0.282525461	cell-mediated immune response
GO:0042828	25	1	0.282525461	response to pathogen
GO:0006959	141	3	0.28428448	humoral immune response
GO:0046649	81	2	0.289193607	lymphocyte activation
GO:0051186	143	3	0.291484394	cofactor metabolism
GO:0019058	26	1	0.292004696	viral infectious cycle
GO:0006220	26	1	0.292004696	pyrimidine nucleotide metabolism
GO:0016066	27	1	0.30135973	cellular defense response (sensu Vertebrata)
GO:0005977	27	1	0.30135973	glycogen metabolism
GO:0000187	27	1	0.30135973	activation of MAPK activity
GO:0006084	27	1	0.30135973	acetyl-CoA metabolism
GO:0044249	763	12	0.303471218	cellular biosynthesis
GO:0043414	28	1	0.310592178	biopolymer methylation
GO:0006968	87	2	0.318452198	cellular defense response
GO:0007179	29	1	0.319703632	transforming growth factor beta receptor signaling pathway
GO:0006767	29	1	0.319703632	water-soluble vitamin metabolism
GO:0009060	30	1	0.328695665	aerobic respiration
GO:0043119	428	7	0.334052698	positive regulation of physiological process

GO:0043065	155	3	0.334873944	positive regulation of apoptosis
GO:0006810	1802	26	0.335595773	transport
GO:0043406	31	1	0.337569828	positive regulation of MAPK activity
GO:0050863	31	1	0.337569828	regulation of T cell activation
GO:0043068	156	3	0.33849352	positive regulation of programmed cell death
GO:0016265	501	8	0.339842484	death
GO:0009058	859	13	0.341888727	biosynthesis
GO:0045333	32	1	0.346327653	cellular respiration
GO:0009064	32	1	0.346327653	glutamine family amino acid metabolism
GO:0007187	94	2	0.352225544	G-protein signaling, coupled to cyclic nucleotide second messenger
GO:0006690	33	1	0.354970654	icosanoid metabolism
GO:0030155	33	1	0.354970654	regulation of cell adhesion
GO:0042742	34	1	0.363500324	defense response to bacteria
GO:0006112	34	1	0.363500324	energy reserve metabolism
GO:0045321	97	2	0.366532875	immune cell activation
GO:0006811	515	8	0.368061713	ion transport
GO:0019935	98	2	0.371275868	cyclic-nucleotide-mediated signaling
GO:0001775	98	2	0.371275868	cell activation
GO:0043062	35	1	0.371918134	extracellular structure organization and biogenesis
GO:0016032	35	1	0.371918134	viral life cycle
GO:0030198	35	1	0.371918134	extracellular matrix organization and biogenesis
GO:0042981	306	5	0.377431847	regulation of apoptosis
GO:0051234	1841	26	0.380161629	establishment of localization
GO:0043067	308	5	0.38270377	regulation of programmed cell death
GO:0006935	101	2	0.385419882	chemotaxis
GO:0042330	101	2	0.385419882	taxis
GO:0030099	37	1	0.388423981	myeloid cell differentiation
GO:0016064	103	2	0.394774024	humoral defense mechanism (sensu Vertebrata)
GO:0007178	38	1	0.39651487	transmembrane receptor protein serine/threonine kinase signaling pathway
GO:0051179	1857	26	0.398813744	localization
GO:0015698	106	2	0.408684252	inorganic anion transport
GO:0006766	40	1	0.412379575	vitamin metabolism
GO:0006096	42	1	0.427830634	glycolysis
GO:0006979	44	1	0.442878744	response to oxidative stress
GO:0006897	115	2	0.449454348	endocytosis
GO:0042110	45	1	0.45025496	T cell activation
GO:0051242	411	6	0.458563414	positive regulation of cellular physiological process
GO:0007610	269	4	0.47537336	behavior
GO:0006732	121	2	0.475753587	coenzyme metabolism
GO:0043405	49	1	0.47880385	regulation of MAPK activity
GO:0007160	49	1	0.47880385	cell-matrix adhesion
GO:0007586	49	1	0.47880385	digestion
GO:0044237	5026	67	0.480591685	cellular metabolism
GO:0008219	498	7	0.486142844	cell death
GO:0006007	51	1	0.49252197	glucose catabolism
GO:0043066	128	2	0.505467577	negative regulation of apoptosis

GO:0045860	53	1	0.505881978	positive regulation of protein kinase activity
GO:0043170	2405	32	0.509201576	macromolecule metabolism
GO:0043069	129	2	0.509623923	negative regulation of programmed cell death
GO:0006928	205	3	0.509695055	cell motility
GO:0040011	205	3	0.509695055	locomotion
GO:0051347	54	1	0.512430596	positive regulation of transferase activity
GO:0045595	55	1	0.518893144	regulation of cell differentiation
GO:0006887	55	1	0.518893144	exocytosis
GO:0007626	209	3	0.522742107	locomotory behavior
GO:0007218	56	1	0.525270744	neuropeptide signaling pathway
GO:0007600	287	4	0.526320337	sensory perception
GO:0007155	518	7	0.528401296	cell adhesion
GO:0006817	57	1	0.531564504	phosphate transport
GO:0019320	59	1	0.543904856	hexose catabolism
GO:0007188	59	1	0.543904856	G-protein signaling, coupled to cAMP nucleotide second messenger
GO:0046365	59	1	0.543904856	monosaccharide catabolism
GO:0006694	60	1	0.549953594	steroid biosynthesis
GO:0008203	60	1	0.549953594	cholesterol metabolism
GO:0019933	60	1	0.549953594	cAMP-mediated signaling
GO:0046164	60	1	0.549953594	alcohol catabolism
GO:0006917	140	2	0.553828427	induction of apoptosis
GO:0012502	140	2	0.553828427	induction of programmed cell death
GO:0044260	2298	30	0.558811086	cellular macromolecule metabolism
GO:0006445	64	1	0.573363321	regulation of translation
GO:0009059	464	6	0.578132531	macromolecule biosynthesis
GO:0016125	66	1	0.58461125	sterol metabolism
GO:0016042	67	1	0.59012444	lipid catabolism
GO:0009581	313	4	0.595497178	detection of external stimulus
GO:0043123	68	1	0.595565064	positive regulation of I-kappaB kinase/NF-kappaB cascade
GO:0008544	70	1	0.606232389	epidermis development
GO:0048522	482	6	0.615773506	positive regulation of cellular process
GO:0042592	157	2	0.616512789	homeostasis
GO:0043122	73	1	0.621712413	regulation of I-kappaB kinase/NF-kappaB cascade
GO:0048518	565	7	0.621941381	positive regulation of biological process
GO:0007167	165	2	0.643616321	enzyme linked receptor protein signaling pathway
GO:0007398	79	1	0.650884872	ectoderm development
GO:0050793	168	2	0.653387763	regulation of development
GO:0000165	81	1	0.660104327	MAPKKK cascade
GO:0016337	172	2	0.666086604	cell-cell adhesion
GO:0009967	85	1	0.677825083	positive regulation of signal transduction
GO:0019932	176	2	0.678412028	second-messenger-mediated signaling
GO:0050954	86	1	0.682110186	sensory perception of mechanical stimulus
GO:0050982	86	1	0.682110186	detection of mechanical stimulus
GO:0007605	86	1	0.682110186	perception of sound
GO:0016055	87	1	0.686338767	Wnt receptor signaling pathway
GO:0030097	87	1	0.686338767	hemopoiesis

GO:0043118	602	7	0.688076993	negative regulation of physiological process
GO:0048534	88	1	0.690511566	hemopoietic or lymphoid organ development
GO:0000902	183	2	0.699096328	cellular morphogenesis
GO:0006486	92	1	0.706659392	protein amino acid glycosylation
GO:0043413	93	1	0.710564038	biopolymer glycosylation
GO:0001558	94	1	0.71441714	regulation of cell growth
GO:0009101	96	1	0.721971403	glycoprotein biosynthesis
GO:0007249	99	1	0.732932807	I-kappaB kinase/NF-kappaB cascade
GO:0000004	465	5	0.740516859	biological process unknown
GO:0008015	103	1	0.746884086	circulation
GO:0009887	807	9	0.747496035	organogenesis
GO:0048523	640	7	0.747982253	negative regulation of cellular process
GO:0040008	104	1	0.750257453	regulation of growth
GO:0006915	471	5	0.750683805	apoptosis
GO:0007156	105	1	0.753586235	homophilic cell adhesion
GO:0016192	296	3	0.753645322	vesicle-mediated transport
GO:0012501	473	5	0.754007568	programmed cell death
GO:0043085	107	1	0.760112372	positive regulation of enzyme activity
GO:0006417	107	1	0.760112372	regulation of protein biosynthesis
GO:0046903	208	2	0.764191642	secretion
GO:0009582	109	1	0.766467083	detection of abiotic stimulus
GO:0031326	111	1	0.772654832	regulation of cellular biosynthesis
GO:0009100	111	1	0.772654832	glycoprotein metabolism
GO:0006916	112	1	0.77568746	anti-apoptosis
GO:0045859	116	1	0.787422023	regulation of protein kinase activity
GO:0007169	116	1	0.787422023	transmembrane receptor protein tyrosine kinase signaling pathway
GO:0009889	116	1	0.787422023	regulation of biosynthesis
GO:0051243	583	6	0.788759831	negative regulation of cellular physiological process
GO:0051338	117	1	0.790259257	regulation of transferase activity
GO:0030154	409	4	0.793799806	cell differentiation
GO:0043037	122	1	0.803891881	translation
GO:0008361	123	1	0.806511058	regulation of cell size
GO:0016049	123	1	0.806511058	cell growth
GO:0048519	688	7	0.811584871	negative regulation of biological process
GO:0048513	872	9	0.8238568	organ development
GO:0006936	132	1	0.828576001	muscle contraction
GO:0009653	1051	11	0.832009555	morphogenesis
GO:0007243	242	2	0.833054286	protein kinase cascade
GO:0006260	140	1	0.846083613	DNA replication
GO:0009117	143	1	0.852181464	nucleotide metabolism
GO:0040007	154	1	0.872559941	growth
GO:0044267	2109	23	0.877115517	cellular protein metabolism
GO:0050877	475	4	0.878841402	neurophysiological process
GO:0045045	161	1	0.884050815	secretory pathway
GO:0019538	2129	23	0.8881615	protein metabolism
GO:0051246	174	1	0.90273262	regulation of protein metabolism

GO:0008610	175	1	0.904039328	lipid biosynthesis
GO:0050875	7018	86	0.93580321	cellular physiological process
GO:0050790	214	1	0.94344286	regulation of enzyme activity
GO:0007001	221	1	0.948575753	chromosome organization and biogenesis (sensu Eukaryota)
GO:0007264	227	1	0.952605831	small GTPase mediated signal transduction
GO:0009966	229	1	0.953878315	regulation of signal transduction
GO:0051276	236	1	0.958070976	chromosome organization and biogenesis
GO:0007399	385	2	0.965502425	neurogenesis
GO:0044238	4820	54	0.967766351	primary metabolism
GO:0006886	257	1	0.968512061	intracellular protein transport
GO:0006412	407	2	0.973293802	protein biosynthesis
GO:0007275	1543	13	0.977705059	development
GO:0007242	879	6	0.979292338	intracellular signaling cascade
GO:0015031	439	2	0.981689298	protein transport
GO:0045184	459	2	0.985577851	establishment of protein localization
GO:0043283	2240	20	0.986688923	biopolymer metabolism
GO:0008104	474	2	0.987958335	protein localization
GO:0007186	491	2	0.990198017	G-protein coupled receptor protein signaling pathway
GO:0006793	641	3	0.992273037	phosphorus metabolism
GO:0006796	641	3	0.992273037	phosphate metabolism
GO:0007154	2730	24	0.995231707	cell communication
GO:0007166	952	5	0.9964953	cell surface receptor linked signal transduction
GO:0046907	448	1	0.997745715	intracellular transport
GO:0006259	489	1	0.998729861	DNA metabolism
GO:0016043	842	3	0.999275074	cell organization and biogenesis
GO:0006464	1087	4	0.999812637	protein modification
GO:0009987	8200	96	0.999821223	cellular process
GO:0043412	1160	4	0.999923621	biopolymer modification
GO:0006996	693	1	0.999929861	organelle organization and biogenesis
GO:0050791	2407	15	0.999944342	regulation of physiological process
GO:0051244	2319	14	0.999950409	regulation of cellular physiological process
GO:0007165	2168	11	0.999991478	signal transduction
GO:0050794	2488	14	0.999992207	regulation of cellular process
GO:0050789	2689	15	0.999997541	regulation of biological process
GO:0031323	1566	3	0.999999958	regulation of cellular metabolism
GO:0019222	1629	3	0.999999983	regulation of metabolism
GO:0006355	1359	1	0.999999997	regulation of transcription, DNA-dependent
GO:0006351	1407	1	0.999999998	transcription, DNA-dependent
GO:0045449	1433	1	0.999999999	regulation of transcription
GO:0019219	1458	1	0.999999999	regulation of nucleobase, nucleoside, nucleotide and nucleic acid metabolism
GO:0006350	1499	1	1	transcription
GO:0006139	2328	4	1	nucleobase, nucleoside, nucleotide and nucleic acid metabolism
GO:0008150	9032	119	1	biological_process

5.3 GO: Molecular Function

GO ID	genes present in chip	genes present in tissues	p-value	GO term
GO:0015020	21	9	2.18940938254860e-12	glucuronosyltransferase activity
GO:0008194	79	10	6.96308306529472e-08	UDP-glycosyltransferase activity
GO:0003824	3409	72	4.39788725193781e-07	catalytic activity
GO:0016491	509	22	6.20497959016695e-07	oxidoreductase activity
GO:0016758	115	10	2.42241272302784e-06	transferase activity, transferring hexosyl groups
GO:0019842	59	7	1.08517985847142e-05	vitamin binding
GO:0016757	165	10	5.84682720987156e-05	transferase activity, transferring glycosyl groups
GO:0008237	133	9	5.91731246127088e-05	metallopeptidase activity
GO:0043169	2313	49	0.000134983233745371	cation binding
GO:0004611	2	2	0.000170290110627048	phosphoenolpyruvate carboxykinase activity
GO:0004613	2	2	0.000170290110627048	phosphoenolpyruvate carboxykinase (GTP) activity
GO:0005386	398	15	0.000210664982185836	carrier activity
GO:0008238	68	6	0.000252700061902821	exopeptidase activity
GO:0008235	44	5	0.000256984157839002	metalloexopeptidase activity
GO:0031402	70	6	0.000296332301458892	sodium ion binding
GO:0031420	146	8	0.000646058573925745	alkali metal ion binding
GO:0015290	149	8	0.000738781599350205	electrochemical potential-driven transporter activity
GO:0015291	149	8	0.000738781599350205	porter activity
GO:0043167	2556	50	0.000842159371094821	ion binding
GO:0005499	4	2	0.00100425856670640	vitamin D binding
GO:0000099	4	2	0.00100425856670640	sulfur amino acid transporter activity
GO:0015184	4	2	0.00100425856670640	L-cystine transporter activity
GO:0005504	17	3	0.00130529552568307	fatty acid binding
GO:0046872	2495	48	0.00168607713634124	metal ion binding
GO:0048037	100	6	0.00195894467352969	cofactor binding
GO:0008233	399	13	0.00214315858372015	peptidase activity
GO:0015174	6	2	0.00246777523268838	basic amino acid transporter activity
GO:0046870	6	2	0.00246777523268838	cadmium ion binding
GO:0019203	7	2	0.0034253059922147	carbohydrate phosphatase activity
GO:0005507	49	4	0.00377372659425182	copper ion binding
GO:0016805	8	2	0.00452801303796876	dipeptidase activity
GO:0015114	8	2	0.00452801303796876	phosphate transporter activity
GO:0015293	83	5	0.00458033606181277	symporter activity
GO:0004177	28	3	0.0056624078396188	aminopeptidase activity
GO:0008514	9	2	0.00577198939684793	organic anion transporter activity
GO:0050662	88	5	0.00586896699698189	coenzyme binding
GO:0016829	127	6	0.00638694322245922	lyase activity
GO:0004033	10	2	0.00715340413203267	aldo-keto reductase activity
GO:0016830	31	3	0.00755125230037827	carbon-carbon lyase activity
GO:0004866	99	5	0.00956615134936968	endopeptidase inhibitor activity
GO:0030414	100	5	0.00996653064809811	protease inhibitor activity
GO:0016284	12	2	0.0103135974112175	alanine aminopeptidase activity
GO:0016877	12	2	0.0103135974112175	ligase activity, forming carbon-sulfur bonds

GO:0004179	12	2	0.0103135974112175	membrane alanyl aminopeptidase activity
GO:0015926	13	2	0.0120850827029998	glucosidase activity
GO:0004505	1	1	0.0131013520595317	phenylalanine 4-monooxygenase activity
GO:0001618	1	1	0.0131013520595317	viral receptor activity
GO:0008336	1	1	0.0131013520595317	gamma-butyrobetaine dioxygenase activity
GO:0005286	1	1	0.0131013520595317	basic amino acid permease activity
GO:0048244	1	1	0.0131013520595317	phytanoyl-CoA dioxygenase activity
GO:0008753	1	1	0.0131013520595317	NADPH dehydrogenase (quinone) activity
GO:0047017	1	1	0.0131013520595317	prostaglandin-F synthase activity
GO:0003868	1	1	0.0131013520595317	4-hydroxyphenylpyruvate dioxygenase activity
GO:0003818	1	1	0.0131013520595317	complement factor I activity
GO:0003837	1	1	0.0131013520595317	beta-ureidopropionase activity
GO:0050824	1	1	0.0131013520595317	water binding
GO:0047150	1	1	0.0131013520595317	betaine-homocysteine S-methyltransferase activity
GO:0004055	1	1	0.0131013520595317	argininosuccinate synthase activity
GO:0015067	1	1	0.0131013520595317	amidinotransferase activity
GO:0015068	1	1	0.0131013520595317	glycine amidinotransferase activity
GO:0004346	1	1	0.0131013520595317	glucose-6-phosphatase activity
GO:0004491	1	1	0.0131013520595317	methylmalonate-semialdehyde dehydrogenase (acylating) activity
GO:0018478	1	1	0.0131013520595317	malonate-semialdehyde dehydrogenase (acetylating) activity
GO:0004251	1	1	0.0131013520595317	X-Pro dipeptidase activity
GO:0004283	1	1	0.0131013520595317	plasmin activity
GO:0004230	1	1	0.0131013520595317	glutamyl aminopeptidase activity
GO:0004235	1	1	0.0131013520595317	matrilysin activity
GO:0008144	15	2	0.0159931316437104	drug binding
GO:0005242	15	2	0.0159931316437104	inward rectifier potassium channel activity
GO:0008289	298	9	0.0162517356668781	lipid binding
GO:0005496	42	3	0.0173812057569039	steroid binding
GO:0004089	16	2	0.0181228242808177	carbonate dehydratase activity
GO:0016616	77	4	0.0182282386105212	oxidoreductase activity, acting on the CH-OH group of donors, NAD or NADP as acceptor
GO:0016836	43	3	0.0185096982178034	hydro-lyase activity
GO:0050660	44	3	0.0196780367344513	FAD binding
GO:0046914	1596	30	0.0226724557005881	transition metal ion binding
GO:0008430	18	2	0.0227168755328142	selenium binding
GO:0016620	18	2	0.0227168755328142	oxidoreductase activity, acting on the aldehyde or oxo group of donors, NAD or NADP as acceptor
GO:0016614	83	4	0.0233240502579721	oxidoreductase activity, acting on CH-OH group of donors
GO:0015171	48	3	0.0247507981554045	amino acid transporter activity
GO:0005044	19	2	0.0251747640079115	scavenger receptor activity
GO:0004558	2	1	0.0260324140084378	alpha-glucosidase activity
GO:0004658	2	1	0.0260324140084378	propionyl-CoA carboxylase activity
GO:0008320	2	1	0.0260324140084378	protein carrier activity
GO:0008451	2	1	0.0260324140084378	X-Pro aminopeptidase activity
GO:0008472	2	1	0.0260324140084378	metallocarboxypeptidase D activity
GO:0005155	2	1	0.0260324140084378	epidermal growth factor receptor activating ligand activity
GO:0004991	2	1	0.0260324140084378	parathyroid hormone receptor activity
GO:0008241	2	1	0.0260324140084378	peptidyl-dipeptidase activity
GO:0008898	2	1	0.0260324140084378	homocysteine S-methyltransferase activity

GO:0047026	2	1	0.0260324140084378	3-alpha-hydroxysteroid dehydrogenase (A-specific) activity
GO:0003858	2	1	0.0260324140084378	3-hydroxybutyrate dehydrogenase activity
GO:0019826	2	1	0.0260324140084378	oxygen sensor activity
GO:0003960	2	1	0.0260324140084378	NADPH:quinone reductase activity
GO:0042132	2	1	0.0260324140084378	fructose-bisphosphatase activity
GO:0008934	2	1	0.0260324140084378	inositol-1(or 4)-monophosphatase activity
GO:0003955	2	1	0.0260324140084378	NAD(P)H dehydrogenase (quinone) activity
GO:0016714	2	1	0.0260324140084378	oxidoreductase activity, acting on paired donors, with incorporation or reduction of molecular oxygen, reduced pteridine as one donor, and incorporation of one atom of oxygen
GO:0015321	2	1	0.0260324140084378	sodium-dependent phosphate transporter activity
GO:0004246	2	1	0.0260324140084378	peptidyl-dipeptidase A activity
GO:0001758	2	1	0.0260324140084378	retinal dehydrogenase activity
GO:0004776	2	1	0.0260324140084378	succinate-CoA ligase (GDP-forming) activity
GO:0005010	2	1	0.0260324140084378	insulin-like growth factor receptor activity
GO:0005215	1030	21	0.0261492405538567	transporter activity
GO:0016835	50	3	0.0275268217164625	carbon-oxygen lyase activity
GO:0015179	20	2	0.0277356883105285	L-amino acid transporter activity
GO:0035091	132	5	0.0295076294548979	phosphoinositide binding
GO:0048503	92	4	0.0324499102581874	GPI anchor binding
GO:0030145	92	4	0.0324499102581874	manganese ion binding
GO:0008509	95	4	0.0358961277688744	anion transporter activity
GO:0015103	23	2	0.0360062269446729	inorganic anion transporter activity
GO:0016651	57	3	0.0384907968250927	oxidoreductase activity, acting on NADH or NADPH
GO:0004800	3	1	0.0387953816412023	thyroxine 5'-deiodinase activity
GO:0004566	3	1	0.0387953816412023	beta-glucuronidase activity
GO:0015643	3	1	0.0387953816412023	toxin binding
GO:0005436	3	1	0.0387953816412023	sodium:phosphate symporter activity
GO:0047115	3	1	0.0387953816412023	trans-1,2-dihydrobenzene-1,2-diol dehydrogenase activity
GO:0016174	3	1	0.0387953816412023	NAD(P)H oxidase activity
GO:0004046	3	1	0.0387953816412023	aminoacylase activity
GO:0004332	3	1	0.0387953816412023	fructose-bisphosphate aldolase activity
GO:0004165	3	1	0.0387953816412023	dodecenoyl-CoA delta-isomerase activity
GO:0004237	3	1	0.0387953816412023	membrane dipeptidase activity
GO:0004774	3	1	0.0387953816412023	succinate-CoA ligase activity
GO:0016831	24	2	0.0389491499221218	carboxy-lyase activity
GO:0005275	58	3	0.0402136464124171	amine transporter activity
GO:0016903	25	2	0.0419803373295698	oxidoreductase activity, acting on the aldehyde or oxo group of donors
GO:0030106	26	2	0.0450970118030617	MHC class I receptor activity
GO:0009055	104	4	0.0474620471593845	electron carrier activity
GO:0004180	27	2	0.0482964525725034	carboxypeptidase activity
GO:0004867	64	3	0.0513523228951182	serine-type endopeptidase inhibitor activity
GO:0031419	4	1	0.0513924226660344	cobalamin binding
GO:0005497	4	1	0.0513924226660344	androgen binding
GO:0008172	4	1	0.0513924226660344	S-methyltransferase activity
GO:0050664	4	1	0.0513924226660344	oxidoreductase activity, acting on NADH or NADPH, with oxygen as acceptor
GO:0016405	4	1	0.0513924226660344	CoA-ligase activity
GO:0004157	4	1	0.0513924226660344	dihydropyrimidinase activity

GO:0005279	28	2	0.051575994475033	amino acid-polyamine transporter activity
GO:0015203	28	2	0.051575994475033	polyamine transporter activity
GO:0005342	66	3	0.0553642095195453	organic acid transporter activity
GO:0046943	66	3	0.0553642095195453	carboxylic acid transporter activity
GO:0003823	30	2	0.0583649932547405	antigen binding
GO:0030674	69	3	0.0616540447665018	protein binding, bridging
GO:0019825	31	2	0.0618693891826275	oxygen binding
GO:0016209	31	2	0.0618693891826275	antioxidant activity
GO:0008131	5	1	0.0638256770612056	amine oxidase activity
GO:0004983	5	1	0.0638256770612056	neuropeptide Y receptor activity
GO:0019841	5	1	0.0638256770612056	retinol binding
GO:0009374	5	1	0.0638256770612056	biotin binding
GO:0016878	5	1	0.0638256770612056	acid-thiol ligase activity
GO:0016832	5	1	0.0638256770612056	aldehyde-lyase activity
GO:0016597	5	1	0.0638256770612056	amino acid binding
GO:0004339	5	1	0.0638256770612056	glucan 1,4-alpha-glucosidase activity
GO:0016810	72	3	0.0682621469381167	hydrolase activity, acting on carbon-nitrogen (but not peptide) bonds
GO:0005543	169	5	0.070995577488675	phospholipid binding
GO:0016811	34	2	0.072792885672106	hydrolase activity, acting on carbon-nitrogen (but not peptide) bonds, in linear amides
GO:0005154	6	1	0.0760972574269098	epidermal growth factor receptor binding
GO:0003857	6	1	0.0760972574269098	3-hydroxyacyl-CoA dehydrogenase activity
GO:0016421	6	1	0.0760972574269098	CoA carboxylase activity
GO:0016812	6	1	0.0760972574269098	hydrolase activity, acting on carbon-nitrogen (but not peptide) bonds, in cyclic amides
GO:0004331	6	1	0.0760972574269098	fructose-2,6-bisphosphate 2-phosphatase activity
GO:0004300	6	1	0.0760972574269098	enoyl-CoA hydratase activity
GO:0004303	6	1	0.0760972574269098	estradiol 17-beta-dehydrogenase activity
GO:0015359	6	1	0.0760972574269098	amino acid permease activity
GO:0004499	6	1	0.0760972574269098	dimethylaniline monooxygenase (N-oxide-forming) activity
GO:0016918	6	1	0.0760972574269098	retinal binding
GO:0004245	6	1	0.0760972574269098	neprilysin activity
GO:0051213	35	2	0.0765629819569894	dioxygenase activity
GO:0016701	35	2	0.0765629819569894	oxidoreductase activity, acting on single donors with incorporation of molecular oxygen
GO:0016702	35	2	0.0765629819569894	oxidoreductase activity, acting on single donors with incorporation of molecular oxygen, incorporation of two atoms of oxygen
GO:0005509	646	13	0.079999119839246	calcium ion binding
GO:0016627	37	2	0.0842829731734362	oxidoreductase activity, acting on the CH-CH group of donors
GO:0004602	7	1	0.0882092493326913	glutathione peroxidase activity
GO:0000062	7	1	0.0882092493326913	acyl-CoA binding
GO:0016885	7	1	0.0882092493326913	ligase activity, forming carbon-carbon bonds
GO:0004857	187	5	0.098904310209027	enzyme inhibitor activity
GO:0015645	8	1	0.100163711660510	fatty-acid ligase activity
GO:0004029	8	1	0.100163711660510	aldehyde dehydrogenase (NAD) activity
GO:0030170	8	1	0.100163711660510	pyridoxal phosphate binding
GO:0004467	8	1	0.100163711660510	long-chain-fatty-acid-CoA ligase activity
GO:0005099	8	1	0.100163711660510	Ras GTPase activator activity
GO:0030955	86	3	0.103003643332278	potassium ion binding
GO:0005200	86	3	0.103003643332278	structural constituent of cytoskeleton

GO:0016705	87	3	0.105712446111175	oxidoreductase activity, acting on paired donors, with incorporation or reduction of molecular oxygen
GO:0005391	9	1	0.111962676943467	sodium:potassium-exchanging ATPase activity
GO:0005545	9	1	0.111962676943467	phosphatidylinositol binding
GO:0019840	9	1	0.111962676943467	isoprenoid binding
GO:0005501	9	1	0.111962676943467	retinoid binding
GO:0004653	10	1	0.123608151700294	polypeptide N-acetylgalactosaminyltransferase activity
GO:0008239	10	1	0.123608151700294	dipeptidyl-peptidase activity
GO:0016863	10	1	0.123608151700294	intramolecular oxidoreductase activity, transposing C=C bonds
GO:0016806	10	1	0.123608151700294	dipeptidyl-peptidase and tripeptidyl-peptidase activity
GO:0015238	10	1	0.123608151700294	drug transporter activity
GO:0050897	11	1	0.135102116765602	cobalt ion binding
GO:0016641	11	1	0.135102116765602	oxidoreductase activity, acting on the CH-NH2 group of donors, oxygen as acceptor
GO:0005489	152	4	0.138644524496028	electron transporter activity
GO:0005506	212	5	0.145428594133102	iron ion binding
GO:0031418	12	1	0.146446527616002	L-ascorbic acid binding
GO:0017124	12	1	0.146446527616002	SH3 domain binding
GO:0003995	12	1	0.146446527616002	acyl-CoA dehydrogenase activity
GO:0004028	12	1	0.146446527616002	aldehyde dehydrogenase activity
GO:0042562	12	1	0.146446527616002	hormone binding
GO:0004252	105	3	0.158804721722514	serine-type endopeptidase activity
GO:0016787	1424	23	0.165059190575052	hydrolase activity
GO:0005518	14	1	0.168694383716381	collagen binding
GO:0004364	14	1	0.168694383716381	glutathione transferase activity
GO:0016628	14	1	0.168694383716381	oxidoreductase activity, acting on the CH-CH group of donors, NAD or NADP as acceptor
GO:0016638	14	1	0.168694383716381	oxidoreductase activity, acting on the CH-NH2 group of donors
GO:0004175	288	6	0.176492783235155	endopeptidase activity
GO:0016229	15	1	0.179601616007286	steroid dehydrogenase activity
GO:0004553	59	2	0.180718273511284	hydrolase activity, hydrolyzing O-glycosyl compounds
GO:0008201	60	2	0.185440460890017	heparin binding
GO:0008236	117	3	0.197896083608255	serine-type peptidase activity
GO:0005249	63	2	0.199708424084428	voltage-gated potassium channel activity
GO:0050661	17	1	0.200991975497855	NADP binding
GO:0016706	17	1	0.200991975497855	oxidoreductase activity, acting on paired donors, with incorporation or reduction of molecular oxygen, 2-oxoglutarate as one donor, and incorporation of one atom each of oxygen into both donors
GO:0008376	18	1	0.211478746084034	acetylgalactosaminyltransferase activity
GO:0051287	18	1	0.211478746084034	NAD binding
GO:0004181	18	1	0.211478746084034	metallocarboxypeptidase activity
GO:0005125	181	4	0.213149807360981	cytokine activity
GO:0030246	181	4	0.213149807360981	carbohydrate binding
GO:0004869	19	1	0.221828967310485	cysteine protease inhibitor activity
GO:0001633	20	1	0.232044403047326	secretin-like receptor activity
GO:0005164	20	1	0.232044403047326	tumor necrosis factor receptor binding
GO:0005520	20	1	0.232044403047326	insulin-like growth factor binding
GO:0004497	71	2	0.238291757230225	monooxygenase activity
GO:0016709	21	1	0.242126794562826	oxidoreductase activity, acting on paired donors, with incorporation or reduction of molecular oxygen, NAD or NADH as one donor, and incorporation of one atom of oxygen
GO:0015296	21	1	0.242126794562826	anion:cation symporter activity

GO:0005201	73	2	0.248012164459349	extracellular matrix structural constituent
GO:0004601	22	1	0.252077860810688	peroxidase activity
GO:0043176	22	1	0.252077860810688	amine binding
GO:0016684	22	1	0.252077860810688	oxidoreductase activity, acting on peroxide as acceptor
GO:0005539	77	2	0.267487652175948	glycosaminoglycan binding
GO:0016798	77	2	0.267487652175948	hydrolase activity, acting on glycosyl bonds
GO:0004222	77	2	0.267487652175948	metalloendopeptidase activity
GO:0030247	80	2	0.282096937648932	polysaccharide binding
GO:0005178	26	1	0.290602486974333	integrin binding
GO:0016769	26	1	0.290602486974333	transferase activity, transferring nitrogenous groups
GO:0004437	26	1	0.290602486974333	inositol or phosphatidylinositol phosphatase activity
GO:0008083	144	3	0.292187884168686	growth factor activity
GO:0008080	28	1	0.309119948182643	N-acetyltransferase activity
GO:0008022	28	1	0.309119948182643	protein C-terminus binding
GO:0015297	28	1	0.309119948182643	antiporter activity
GO:0016741	87	2	0.316064778520745	transferase activity, transferring one-carbon groups
GO:0008188	29	1	0.318198052511158	neuropeptide receptor activity
GO:0042923	29	1	0.318198052511158	neuropeptide binding
GO:0001871	88	2	0.320893047627248	pattern binding
GO:0016874	221	4	0.328866840065387	ligase activity
GO:0005267	90	2	0.330524824245656	potassium channel activity
GO:0016860	31	1	0.336000760641591	intramolecular oxidoreductase activity
GO:0008146	32	1	0.344728405743568	sulfotransferase activity
GO:0016410	33	1	0.353342239930344	N-acyltransferase activity
GO:0016655	35	1	0.37023434591266	oxidoreductase activity, acting on NADH or NADPH, quinone or similar compound as acceptor
GO:0016782	36	1	0.378515505891718	transferase activity, transferring sulfur-containing groups
GO:0016765	37	1	0.386688631800559	transferase activity, transferring alkyl or aryl (other than methyl) groups
GO:0016791	171	3	0.38904957512848	phosphoric monoester hydrolase activity
GO:0005529	103	2	0.392036882955928	sugar binding
GO:0004518	105	2	0.401290431207959	nuclease activity
GO:0016407	39	1	0.402716356130665	acetyltransferase activity
GO:0008009	40	1	0.410573697267654	chemokine activity
GO:0015662	40	1	0.410573697267654	ATPase activity, coupled to transmembrane movement of ions, phosphorylative mechanism
GO:0042379	40	1	0.410573697267654	chemokine receptor binding
GO:0005070	40	1	0.410573697267654	SH3/SH2 adaptor activity
GO:0008270	1287	18	0.421278446445812	zinc ion binding
GO:0001664	42	1	0.42598206271724	G-protein-coupled receptor binding
GO:0015036	42	1	0.42598206271724	disulfide oxidoreductase activity
GO:0004540	45	1	0.448348480278756	ribonuclease activity
GO:0031404	45	1	0.448348480278756	chloride ion binding
GO:0043168	45	1	0.448348480278756	anion binding
GO:0016740	1169	16	0.466361914288746	transferase activity
GO:0019838	49	1	0.476830899812878	growth factor binding
GO:0005244	124	2	0.485576004791398	voltage-gated ion channel activity
GO:0019904	52	1	0.4972308453571	protein domain specific binding
GO:0008757	57	1	0.529491681730612	S-adenosylmethionine-dependent methyltransferase activity
GO:0015075	523	7	0.53249466367747	ion transporter activity

GO:0005319	58	1	0.535693025022859	lipid transporter activity
GO:0005102	528	7	0.54275763021034	receptor binding
GO:0004714	60	1	0.547853496621456	transmembrane receptor protein tyrosine kinase activity
GO:0042803	64	1	0.571234562432702	protein homodimerization activity
GO:0005525	226	3	0.572030078085314	GTP binding
GO:0004519	65	1	0.576889904808549	endonuclease activity
GO:0042578	228	3	0.578050364048264	phosphoric ester hydrolase activity
GO:0008565	67	1	0.587979538071856	protein transporter activity
GO:0042625	68	1	0.593415737960078	ATPase activity, coupled to transmembrane movement of ions
GO:0019001	235	3	0.59870516076632	guanyl nucleotide binding
GO:0042802	158	2	0.616775457413814	protein self binding
GO:0019199	73	1	0.61954765632726	transmembrane receptor protein kinase activity
GO:0046906	74	1	0.624570527362229	tetrapyrrole binding
GO:0020037	74	1	0.624570527362229	heme binding
GO:0015405	77	1	0.639247807609984	P-P-bond-hydrolysis-driven transporter activity
GO:0030594	77	1	0.639247807609984	neurotransmitter receptor activity
GO:0042165	78	1	0.64401260305047	neurotransmitter binding
GO:0005216	252	3	0.64608794356172	ion channel activity
GO:0004197	80	1	0.653355696686563	cysteine-type endopeptidase activity
GO:0046983	170	2	0.656596162190197	protein dimerization activity
GO:0030234	509	6	0.662907183437712	enzyme regulator activity
GO:0008168	84	1	0.671318257169329	methyltransferase activity
GO:0005179	90	1	0.696544930540858	hormone activity
GO:0016788	446	5	0.701324248199652	hydrolase activity, acting on ester bonds
GO:0015268	274	3	0.70140486474732	alpha-type channel activity
GO:0016853	92	1	0.704519328681539	isomerase activity
GO:0005261	188	2	0.7100960636888	cation channel activity
GO:0001653	94	1	0.71228580284809	peptide receptor activity
GO:0008528	94	1	0.71228580284809	peptide receptor activity, G-protein coupled
GO:0003779	189	2	0.712855751714817	actin binding
GO:0008092	279	3	0.713029522861306	cytoskeletal protein binding
GO:0015267	284	3	0.724306974732633	channel or pore class transporter activity
GO:0008415	98	1	0.727216356452191	acyltransferase activity
GO:0008234	98	1	0.727216356452191	cysteine-type peptidase activity
GO:0016747	98	1	0.727216356452191	transferase activity, transferring groups other than amino-acyl groups
GO:0042626	100	1	0.734390782893778	ATPase activity, coupled to transmembrane movement of substances
GO:0016820	101	1	0.737907481563228	hydrolase activity, acting on acid anhydrides, catalyzing transmembrane movement of substances
GO:0016746	104	1	0.748182814070932	transferase activity, transferring acyl groups
GO:0005198	488	5	0.773284871812715	structural molecule activity
GO:0005096	117	1	0.788283698904465	GTPase activator activity
GO:0008324	421	4	0.8082677199867	cation transporter activity
GO:0005488	7228	91	0.812120894481257	binding
GO:0004888	694	7	0.813522751915446	transmembrane receptor activity
GO:0042277	127	1	0.814759431439006	peptide binding
GO:0016879	127	1	0.814759431439006	ligase activity, forming carbon-nitrogen bonds
GO:0005057	132	1	0.8267378337893	receptor signaling protein activity
GO:0004872	1060	11	0.833962913421498	receptor activity

GO:0015399	140	1	0.844324727148947	primary active transporter activity
GO:0004871	1837	20	0.85218691617287	signal transducer activity
GO:0005083	144	1	0.852442037906373	small GTPase regulator activity
GO:0019899	151	1	0.865649610170872	enzyme binding
GO:0004713	161	1	0.882507770264477	protein-tyrosine kinase activity
GO:0008047	188	1	0.918252209715663	enzyme activator activity
GO:0030695	219	1	0.946168175180585	GTPase regulator activity
GO:0004930	349	2	0.946575243494846	G-protein coupled receptor activity
GO:0017076	1248	11	0.947408427683645	purine nucleotide binding
GO:0000166	1432	13	0.948573970389859	nucleotide binding
GO:0042623	229	1	0.952969297323054	ATPase activity, coupled
GO:0005515	3709	40	0.954889095861855	protein binding
GO:0000287	251	1	0.96507703292987	magnesium ion binding
GO:0030554	1028	8	0.966432274904872	adenyl nucleotide binding
GO:0016887	255	1	0.966919327550853	ATPase activity
GO:0005554	522	3	0.970577835994722	molecular function unknown
GO:0001584	282	1	0.977068449966207	rhodopsin-like receptor activity
GO:0008134	293	1	0.980254677349736	transcription factor binding
GO:0017111	410	1	0.996022836360086	nucleoside-triphosphatase activity
GO:0005524	970	5	0.996870002911265	ATP binding
GO:0016462	430	1	0.996981995741396	pyrophosphatase activity
GO:0016818	432	1	0.997064244248773	hydrolase activity, acting on acid anhydrides, in phosphorus-containing anhydrides
GO:0016817	433	1	0.997104530720274	hydrolase activity, acting on acid anhydrides
GO:0003723	436	1	0.997222128808607	RNA binding
GO:0004672	447	1	0.997614190245065	protein kinase activity
GO:0016773	532	1	0.999268328578073	phosphotransferase activity, alcohol group as acceptor
GO:0016301	609	1	0.99975164025522	kinase activity
GO:0003700	649	1	0.999858839693028	transcription factor activity
GO:0016772	708	1	0.999938939915034	transferase activity, transferring phosphorus-containing groups
GO:0030528	963	1	0.999998472267914	transcription regulator activity
GO:0003677	1384	2	0.99999993911291	DNA binding
GO:0003676	1990	3	0.999999999904793	nucleic acid binding
GO:0003674	9541	125	1	molecular_function

5.4 GO: Cellular Component

GO ID	genes present in chip	genes present in tissues	p-value	GO term
GO:0005792	108	13	6.72462099801944e-10	microsome
GO:0042598	108	13	6.72462099801944e-10	vesicular fraction
GO:0005624	521	20	5.67299633966529e-06	membrane fraction
GO:0000267	698	22	4.11378934346869e-05	cell fraction
GO:0005576	949	25	0.000212895090165077	extracellular region
GO:0016021	2674	51	0.000267539699821672	integral to membrane
GO:0031224	2681	51	0.000287112016409404	intrinsic to membrane
GO:0005783	483	16	0.000305875298663655	endoplasmic reticulum
GO:0016020	3514	61	0.000616610930724047	membrane
GO:0005615	385	11	0.0085157199928292	extracellular space
GO:0005777	67	4	0.00969208895350015	peroxisome
GO:0042579	67	4	0.00969208895350015	microbody
GO:0005737	2530	43	0.0118825186070402	cytoplasm
GO:0005886	1472	28	0.0124628230010878	plasma membrane
GO:0031253	1	1	0.012504341785342	cell projection membrane
GO:0031232	1	1	0.012504341785342	extrinsic to external side of plasma membrane
GO:0005782	1	1	0.012504341785342	peroxisomal matrix
GO:0031526	1	1	0.012504341785342	brush border membrane
GO:0005887	1057	20	0.0370942696998163	integral to plasma membrane
GO:0031226	1062	20	0.0387168945383262	intrinsic to plasma membrane
GO:0005606	5	1	0.0609913246602207	laminin-1
GO:0005579	6	1	0.0727398113303663	membrane attack complex
GO:0019898	37	2	0.0777609883537831	extrinsic to membrane
GO:0043256	7	1	0.0843426499789965	laminin complex
GO:0005890	7	1	0.0843426499789965	sodium:potassium-exchanging ATPase complex
GO:0005913	7	1	0.0843426499789965	cell-cell adherens junction
GO:0005903	8	1	0.0958016295621095	brush border
GO:0005583	10	1	0.118295058775685	fibrillar collagen
GO:0009897	11	1	0.129332978522089	external side of plasma membrane
GO:0001533	12	1	0.140233979950284	cornified envelope
GO:0005769	13	1	0.150999745766559	early endosome
GO:0005758	14	1	0.161631938189705	mitochondrial intermembrane space
GO:0001726	14	1	0.161631938189705	ruffle
GO:0005578	303	6	0.178405689785334	extracellular matrix (sensu Metazoa)
GO:0031012	304	6	0.180265809505871	extracellular matrix
GO:0005768	62	2	0.181525433725662	endosome
GO:0042611	16	1	0.18250215077392	MHC protein complex
GO:0042612	16	1	0.18250215077392	MHC class I protein complex
GO:0045111	65	2	0.195101970774035	intermediate filament cytoskeleton
GO:0005882	65	2	0.195101970774035	intermediate filament
GO:0005912	18	1	0.202857515019451	adherens junction
GO:0042995	70	2	0.217994585448584	cell projection

GO:0031252	24	1	0.260958851312465	leading edge
GO:0005793	24	1	0.260958851312465	ER-Golgi intermediate compartment
GO:0005605	24	1	0.260958851312465	basal lamina
GO:0019897	26	1	0.279377660879176	extrinsic to plasma membrane
GO:0008021	27	1	0.288415776385512	synaptic vesicle
GO:0001772	29	1	0.306156429263957	immunological synapse
GO:0005581	30	1	0.314861715699771	collagen
GO:0008372	523	8	0.330376997411995	cellular component unknown
GO:0031227	32	1	0.33194895592419	intrinsic to endoplasmic reticulum membrane
GO:0005905	38	1	0.380717964247245	coated pit
GO:0005764	107	2	0.388027632505884	lysosome
GO:0000323	107	2	0.388027632505884	lytic vacuole
GO:0005739	490	7	0.414738870804633	mitochondrion
GO:0005604	44	1	0.425957306373758	basement membrane
GO:0005773	120	2	0.444552400877563	vacuole
GO:0009986	61	1	0.537111719838518	cell surface
GO:0005759	62	1	0.542941003217416	mitochondrial matrix
GO:0030136	65	1	0.559996007917482	clathrin-coated vesicle
GO:0030135	79	1	0.631611030343774	coated vesicle
GO:0005911	82	1	0.645384305790609	intercellular junction
GO:0005789	84	1	0.654281783636014	endoplasmic reticulum membrane
GO:0015629	183	2	0.671503670965361	actin cytoskeleton
GO:0005625	183	2	0.671503670965361	soluble fraction
GO:0005829	276	3	0.676189566277897	cytosol
GO:0031300	90	1	0.679668527370419	intrinsic to organelle membrane
GO:0042175	90	1	0.679668527370419	nuclear envelope-endoplasmic reticulum network
GO:0030054	93	1	0.691660255770154	cell junction
GO:0016023	122	1	0.786916744654435	cytoplasmic membrane-bound vesicle
GO:0031410	124	1	0.792288061924968	cytoplasmic vesicle
GO:0005856	612	5	0.88912328403807	cytoskeleton
GO:0012505	246	1	0.956739005160625	endomembrane system
GO:0031090	377	1	0.992178759215207	organelle membrane
GO:0005623	7802	90	0.992857816313744	cell
GO:0043228	977	5	0.995613223392708	non-membrane-bound organelle
GO:0043232	977	5	0.995613223392708	intracellular non-membrane-bound organelle
GO:0043227	3955	33	0.999594481296357	membrane-bound organelle
GO:0043231	3955	33	0.999594481296357	intracellular membrane-bound organelle
GO:0005622	5419	50	0.999835670844765	intracellular
GO:0043226	4515	38	0.999888934502748	organelle
GO:0043229	4515	38	0.999888934502748	intracellular organelle
GO:0043234	1247	4	0.999947526471218	protein complex
GO:0005634	2704	5	0.999999999999518	nucleus
GO:0005575	8637	108	1	cellular_component

6. SubthalamicNucleus

6.1 SubthalamicNucleus selective genes (24 genes)

Symbol	Description	Chromosome	Chromosome Location	LocusLink	Cytoband	UniGene
GRIA2	glutamate receptor, ionotropic, AMPA 2	4	158361296	2891	4q32-q33	Hs.32763
SYT1	synaptotagmin I	12	78135413	6857	12cen-q21	Hs.310545
PLP1	proteolipid protein 1 (Pelizaeus-Merzbacher disease, spastic paraplegia 2, uncomplicated)	X	102918409	5354	Xq22	Hs.1787
GPM6A	glycoprotein M6A	4	-176791082	2823	4q34	Hs.75819
SNAP25	synaptosomal-associated protein, 25kDa	20	10147476	6616	20p12-p11.2	Hs.167317
DIRAS2	DIRAS family, GTP-binding RAS-like 2	9	-92411933	54769	9q22.2	Hs.165636
RTN1	reticulon 1	14	-59132446	6252	14q23.1	Hs.368626
STMN2	stathmin-like 2	8	80685934	11075	8q21.13	Hs.521651
CCK	cholecystokinin	3	-42274321	885	3p22-p21.3	Hs.458426
VSNL1	visinin-like 1	2	17585287	7447	2p24.3	Hs.444212
GFAP	glial fibrillary acidic protein	17	-40338518	2670	17q21	Hs.514227
DNM1	dynamin 1	9	130005483	1759	9q34	Hs.522413
NEF3	neurofilament 3 (150kDa medium)	8	24827211	4741	8p21	Hs.458657 Hs.615287
CADPS	Ca2+dependent secretion activator	3	-62359061	8618	3p14.2	Hs.127013
DCAMKL1	doublecortin and CaM kinase-like 1	13	-35243477	9201	13q13	Hs.507755
NEFH	neurofilament, heavy polypeptide 200kDa	22	28206218	4744	22q12.2	Hs.198760
MT3	metallothionein 3 (growth inhibitory factor (neurotrophic))	16	55180767	4504	16q13	Hs.73133
SCG5	secretogranin V (7B2 protein)	15	30721251	6447	15q13-q14	Hs.156540
NRGN	neurogranin (protein kinase C substrate, RC3)	11	124114951	4900	11q24	Hs.524116
SH3GL2	SH3-domain GRB2-like 2	9	17569193	6456	9p22	Hs.75149
NAP1L3	nucleosome assembly protein 1-like 3	X	-92812589	4675	Xq21.3-q22	Hs.21365
CHN1	chimerin (chimaerin) 1	2	-175372337	1123	2q31-q32.1	Hs.380138
CAMK2B	calcium/calmodulin-dependent protein kinase (CaM kinase) II beta	7	-44225421	816	22q12|7p14.3-p14.1	Hs.351887
EEF1A2	eukaryotic translation elongation factor 1 alpha 2	20	-61589809	1917	20q13.3	Hs.433839

6.2 GO: Biological Process

GO ID	genes present in chip	genes present in tissues	p-value	GO term
GO:0046883	8	2	0.000116518	regulation of hormone secretion
GO:0007154	2730	14	0.000116668	cell communication
GO:0006887	55	3	0.000193248	exocytosis
GO:0046879	11	2	0.000228014	hormone secretion
GO:0016192	296	5	0.000291063	vesicle-mediated transport
GO:0030182	13	2	0.000322555	neuron differentiation
GO:0007268	192	4	0.000597215	synaptic transmission
GO:0019226	198	4	0.000670679	transmission of nerve impulse
GO:0007269	19	2	0.000701836	neurotransmitter secretion
GO:0046903	208	4	0.000807107	secretion
GO:0045055	22	2	0.000944534	regulated secretory pathway
GO:0007399	385	5	0.000971242	neurogenesis
GO:0051046	24	2	0.001125708	regulation of secretion
GO:0007165	2168	11	0.001586025	signal transduction
GO:0019430	1	1	0.002103632	removal of superoxide radicals
GO:0007267	465	5	0.002255612	cell-cell signaling
GO:0001505	41	2	0.003274005	regulation of neurotransmitter levels
GO:0016081	2	1	0.00420307	synaptic vesicle docking during exocytosis
GO:0050774	2	1	0.00420307	negative regulation of dendrite morphogenesis
GO:0045045	161	3	0.00436738	secretory pathway
GO:0006334	48	2	0.00446446	nucleosome assembly
GO:0031497	56	2	0.006034394	chromatin assembly
GO:0017157	3	1	0.006298324	regulation of exocytosis
GO:0050796	3	1	0.006298324	regulation of insulin secretion
GO:0050773	3	1	0.006298324	regulation of dendrite morphogenesis
GO:0001504	4	1	0.008389401	neurotransmitter uptake
GO:0050768	4	1	0.008389401	negative regulation of neurogenesis
GO:0016486	4	1	0.008389401	peptide hormone processing
GO:0005513	5	1	0.010476308	calcium ion sensing
GO:0016358	5	1	0.010476308	dendrite morphogenesis
GO:0030073	5	1	0.010476308	insulin secretion
GO:0016079	6	1	0.012559055	synaptic vesicle exocytosis
GO:0030072	6	1	0.012559055	peptide hormone secretion
GO:0009593	6	1	0.012559055	detection of chemical substance
GO:0006333	91	2	0.015359677	chromatin assembly or disassembly
GO:0050877	475	4	0.015583269	neurophysiological process
GO:0007417	101	2	0.018707048	central nervous system development
GO:0009887	807	5	0.022685659	organogenesis
GO:0006810	1802	8	0.02283198	transport
GO:0048489	11	1	0.02291064	synaptic vesicle transport
GO:0051260	11	1	0.02291064	protein homooligomerization
GO:0006414	12	1	0.024968582	translational elongation

GO:0051234	1841	8	0.025797627	establishment of localization
GO:0051179	1857	8	0.027093242	localization
GO:0001666	14	1	0.029072154	response to hypoxia
GO:0048513	872	5	0.030639155	organ development
GO:0006801	15	1	0.031117799	superoxide metabolism
GO:0050767	15	1	0.031117799	regulation of neurogenesis
GO:0007275	1543	7	0.031624325	development
GO:0006904	17	1	0.035196847	vesicle docking during exocytosis
GO:0048278	18	1	0.037230264	vesicle docking
GO:0016197	22	1	0.045323406	endosome transport
GO:0007032	24	1	0.049345767	endosome organization and biogenesis
GO:0051259	24	1	0.049345767	protein oligomerization
GO:0045792	25	1	0.051350921	negative regulation of cell size
GO:0030308	25	1	0.051350921	negative regulation of cell growth
GO:0030154	409	3	0.052112085	cell differentiation
GO:0006898	28	1	0.057342371	receptor mediated endocytosis
GO:0006325	186	2	0.057350145	establishment and/or maintenance of chromatin architecture
GO:0045926	29	1	0.059331542	negative regulation of growth
GO:0006323	193	2	0.06122789	DNA packaging
GO:0009653	1051	5	0.0612556	morphogenesis
GO:0006461	194	2	0.061789258	protein complex assembly
GO:0006836	33	1	0.067248531	neurotransmitter transport
GO:0016485	37	1	0.075102366	protein processing
GO:0007001	221	2	0.07760574	chromosome organization and biogenesis (sensu Eukaryota)
GO:0051276	236	2	0.086902664	chromosome organization and biogenesis
GO:0031175	46	1	0.092545052	neurite morphogenesis
GO:0051049	46	1	0.092545052	regulation of transport
GO:0042445	46	1	0.092545052	hormone metabolism
GO:0016043	842	4	0.094154903	cell organization and biogenesis
GO:0051093	49	1	0.098289697	negative regulation of development
GO:0007242	879	4	0.106385942	intracellular signaling cascade
GO:0007218	56	1	0.111560178	neuropeptide signaling pathway
GO:0006800	61	1	0.120925583	oxygen and reactive oxygen species metabolism
GO:0044267	2109	7	0.132707195	cellular protein metabolism
GO:0019538	2129	7	0.137971629	protein metabolism
GO:0009987	8200	19	0.159123044	cellular process
GO:0006996	693	3	0.174843664	organelle organization and biogenesis
GO:0006875	93	1	0.178685487	metal ion homeostasis
GO:0001558	94	1	0.180431205	regulation of cell growth
GO:0044260	2298	7	0.186945706	cellular macromolecule metabolism
GO:0030003	98	1	0.187378989	cation homeostasis
GO:0006873	103	1	0.195985265	cell ion homeostasis
GO:0040008	104	1	0.197696127	regulation of growth
GO:0009582	109	1	0.206198824	detection of abiotic stimulus
GO:0006813	111	1	0.209575941	potassium ion transport
GO:0050801	111	1	0.209575941	ion homeostasis

GO:0006897	115	1	0.216289385	endocytosis
GO:0043170	2405	7	0.22183938	macromolecule metabolism
GO:0043037	122	1	0.227908115	translation
GO:0019725	122	1	0.227908115	cell homeostasis
GO:0008361	123	1	0.229554551	regulation of cell size
GO:0016049	123	1	0.229554551	cell growth
GO:0015031	439	2	0.235284895	protein transport
GO:0006468	444	2	0.239236461	protein amino acid phosphorylation
GO:0046907	448	2	0.24240163	intracellular transport
GO:0045184	459	2	0.251121542	establishment of protein localization
GO:0008104	474	2	0.263041698	protein localization
GO:0006259	489	2	0.27498402	DNA metabolism
GO:0040007	154	1	0.278975205	growth
GO:0042592	157	1	0.283595066	homeostasis
GO:0050793	168	1	0.300295774	regulation of development
GO:0016310	522	2	0.301275892	phosphorylation
GO:0006457	169	1	0.301795592	protein folding
GO:0000902	183	1	0.322475847	cellular morphogenesis
GO:0015672	223	1	0.37841217	monovalent inorganic cation transport
GO:0007264	227	1	0.383758526	small GTPase mediated signal transduction
GO:0006793	641	2	0.3947845	phosphorus metabolism
GO:0006796	641	2	0.3947845	phosphate metabolism
GO:0006464	1087	3	0.405180599	protein modification
GO:0050874	1531	4	0.406263554	organismal physiological process
GO:0006118	256	1	0.421236886	electron transport
GO:0006886	257	1	0.422489906	intracellular protein transport
GO:0030001	269	1	0.437327075	metal ion transport
GO:0043412	1160	3	0.448608997	biopolymer modification
GO:0009581	313	1	0.488698998	detection of external stimulus
GO:0009628	318	1	0.494247043	response to abiotic stimulus
GO:0006812	346	1	0.524277122	cation transport
GO:0043283	2240	5	0.526594778	biopolymer metabolism
GO:0006412	407	1	0.583955506	protein biosynthesis
GO:0006091	446	1	0.618315574	generation of precursor metabolites and energy
GO:0009059	464	1	0.633250828	macromolecule biosynthesis
GO:0008283	473	1	0.64050917	cell proliferation
GO:0007186	491	1	0.654619576	G-protein coupled receptor protein signaling pathway
GO:0006811	515	1	0.672618779	ion transport
GO:0051243	583	1	0.718914736	negative regulation of cellular physiological process
GO:0043118	602	1	0.730696955	negative regulation of physiological process
GO:0048523	640	1	0.752872301	negative regulation of cellular process
GO:0048519	688	1	0.778416189	negative regulation of biological process
GO:0050791	2407	4	0.78705718	regulation of physiological process
GO:0044249	763	1	0.813382479	cellular biosynthesis
GO:0009058	859	1	0.850552588	biosynthesis
GO:0006950	881	1	0.858021584	response to stress

GO:0050789	2689	4	0.862503449	regulation of biological process
GO:0050896	1578	2	0.869532715	response to stimulus
GO:0007166	952	1	0.879787112	cell surface receptor linked signal transduction
GO:0009605	963	1	0.882862158	response to external stimulus
GO:0044237	5026	8	0.921870476	cellular metabolism
GO:0008152	5262	8	0.950789987	metabolism
GO:0044238	4820	7	0.953439358	primary metabolism
GO:0050875	7018	12	0.957541152	cellular physiological process
GO:0051244	2319	2	0.973182796	regulation of cellular physiological process
GO:0006139	2328	2	0.973739888	nucleobase, nucleoside, nucleotide and nucleic acid metabolism
GO:0050794	2488	2	0.98204788	regulation of cellular process
GO:0007582	7846	12	0.998294515	physiological process
GO:0008150	9032	19	1	biological_process

6.3 GO: Molecular Function

GO ID	genes present in chip	genes present in tissues	p-value	GO term
GO:0005525	226	4	0.00160294636681704	GTP binding
GO:0019001	235	4	0.00185041603713840	guanyl nucleotide binding
GO:0008289	298	4	0.00437191517822256	lipid binding
GO:0017075	2	1	0.00460660018101289	syntaxin-1 binding
GO:0004971	3	1	0.00690229729769354	alpha-amino-3-hydroxy-5-methyl-4-isoxazole propionate selective glutamate receptor activity
GO:0050750	6	1	0.0137591041397397	low-density lipoprotein receptor binding
GO:0019905	6	1	0.0137591041397397	syntaxin binding
GO:0000149	6	1	0.0137591041397397	SNARE binding
GO:0015277	7	1	0.0160346468464960	kainate selective glutamate receptor activity
GO:0005200	86	2	0.0165032657391628	structural constituent of cytoskeleton
GO:0005545	9	1	0.0205707061496851	phosphatidylinositol binding
GO:0004685	10	1	0.0228312437561382	calcium- and calmodulin-dependent protein kinase activity
GO:0003746	10	1	0.0228312437561382	translation elongation factor activity
GO:0005198	488	4	0.0237356305014009	structural molecule activity
GO:0005516	112	2	0.0270740885844006	calmodulin binding
GO:0004970	14	1	0.0318236913553124	ionotropic glutamate receptor activity
GO:0005234	14	1	0.0318236913553124	glutamate-gated ion channel activity
GO:0004683	17	1	0.0385161305980619	calmodulin regulated protein kinase activity
GO:0017076	1248	6	0.0580565073852536	purine nucleotide binding
GO:0008066	29	1	0.0648469362726827	glutamate receptor activity
GO:0008047	188	2	0.0689531184233889	enzyme activator activity
GO:0016209	31	1	0.0691679317974676	antioxidant activity
GO:0005231	39	1	0.0862620914546401	excitatory extracellular ligand-gated ion channel activity
GO:0005070	40	1	0.088377670699851	SH3/SH2 adaptor activity
GO:0019992	42	1	0.0925948108804562	diacylglycerol binding
GO:0000166	1432	6	0.0999251660224832	nucleotide binding
GO:0005507	49	1	0.107208638787293	copper ion binding
GO:0005230	57	1	0.123635055036467	extracellular ligand-gated ion channel activity
GO:0008135	62	1	0.133754634271794	translation factor activity, nucleic acid binding
GO:0030674	69	1	0.147734820049606	protein binding, bridging
GO:0045182	72	1	0.153660165060625	translation regulator activity
GO:0004674	326	2	0.172253687068931	protein serine/threonine kinase activity
GO:0015276	84	1	0.176970668741934	ligand-gated ion channel activity
GO:0005509	646	3	0.183681460863248	calcium ion binding
GO:0005179	90	1	0.188394841149613	hormone activity
GO:0005267	90	1	0.188394841149613	potassium channel activity
GO:0005515	3709	11	0.195836546784488	protein binding
GO:0008415	98	1	0.203391794064723	acyltransferase activity
GO:0003774	98	1	0.203391794064723	motor activity
GO:0016747	98	1	0.203391794064723	transferase activity, transferring groups other than amino-acyl groups
GO:0009055	104	1	0.214465526758407	electron carrier activity
GO:0016746	104	1	0.214465526758407	transferase activity, transferring acyl groups

GO:0004871	1837	6	0.237671752776767	signal transducer activity
GO:0005096	117	1	0.237956690287924	GTPase activator activity
GO:0003924	121	1	0.245048790789769	GTPase activity
GO:0051082	127	1	0.255568926349378	unfolded protein binding
GO:0035091	132	1	0.264228669186852	phosphoinositide binding
GO:0005057	132	1	0.264228669186852	receptor signaling protein activity
GO:0004672	447	2	0.275735387331699	protein kinase activity
GO:0005489	152	1	0.29791569248802	electron transporter activity
GO:0042802	158	1	0.307731257146428	protein self binding
GO:0004713	161	1	0.312589782250961	protein-tyrosine kinase activity
GO:0005543	169	1	0.325387236461171	phospholipid binding
GO:0030234	509	2	0.329561293552797	enzyme regulator activity
GO:0005554	522	2	0.340771979544913	molecular function unknown
GO:0005102	528	2	0.345931750535417	receptor binding
GO:0016773	532	2	0.349366044050551	phosphotransferase activity, alcohol group as acceptor
GO:0004857	187	1	0.353355172982802	enzyme inhibitor activity
GO:0005261	188	1	0.354876039584724	cation channel activity
GO:0005506	212	1	0.390369021761627	iron ion binding
GO:0030695	219	1	0.400364879666476	GTPase regulator activity
GO:0016301	609	2	0.414345301556372	kinase activity
GO:0005215	1030	3	0.429361739990393	transporter activity
GO:0005216	252	1	0.445417981524445	ion channel activity
GO:0015268	274	1	0.473638024791078	alpha-type channel activity
GO:0015267	284	1	0.486007229192619	channel or pore class transporter activity
GO:0016772	708	2	0.493507598948575	transferase activity, transferring phosphorus-containing groups
GO:0016740	1169	3	0.516615718357898	transferase activity
GO:0005488	7228	17	0.550090552215568	binding
GO:0005386	398	1	0.608774003229106	carrier activity
GO:0017111	410	1	0.619928793780857	nucleoside-triphosphatase activity
GO:0008324	421	1	0.629886779409912	cation transporter activity
GO:0016462	430	1	0.63784849192876	pyrophosphatase activity
GO:0016818	432	1	0.639595424250573	hydrolase activity, acting on acid anhydrides, in phosphorus-containing anhydrides
GO:0016817	433	1	0.640465871134588	hydrolase activity, acting on acid anhydrides
GO:0043169	2313	5	0.646745135948969	cation binding
GO:0005524	970	2	0.670394004023415	ATP binding
GO:0030554	1028	2	0.702598900314263	adenyl nucleotide binding
GO:0015075	523	1	0.711099283239611	ion transporter activity
GO:0046872	2495	5	0.719517688811628	metal ion binding
GO:0043167	2556	5	0.741715644348004	ion binding
GO:0004888	694	1	0.810494958310548	transmembrane receptor activity
GO:0008270	1287	2	0.817524757258568	zinc ion binding
GO:0046914	1596	2	0.903667722514226	transition metal ion binding
GO:0004872	1060	1	0.925277158030273	receptor activity
GO:0003676	1990	2	0.960584788372975	nucleic acid binding
GO:0003677	1384	1	0.968317642142974	DNA binding
GO:0016787	1424	1	0.971569230092228	hydrolase activity

GO:0003824	3409	4	0.979251333831172	catalytic activity
GO:0003674	9541	22	1	molecular_function

6.4 GO: Cellular Component

GO ID	genes present in chip	genes present in tissues	p-value	GO term
GO:0005883	5	2	5.0733676347485e-05	neurofilament
GO:0030424	7	2	0.000106244898611547	axon
GO:0045111	65	3	0.000423270144840332	intermediate filament cytoskeleton
GO:0005882	65	3	0.000423270144840332	intermediate filament
GO:0043005	17	2	0.000678567685351072	neuron projection
GO:0045202	98	3	0.00140374123539613	synapse
GO:0008021	27	2	0.00172715856936337	synaptic vesicle
GO:0016023	122	3	0.00263000335165573	cytoplasmic membrane-bound vesicle
GO:0031410	124	3	0.00275444655940254	cytoplasmic vesicle
GO:0030136	65	2	0.00971018608973851	clathrin-coated vesicle
GO:0042995	70	2	0.0111964244661128	cell projection
GO:0005678	6	1	0.0138175065386726	chromatin assembly complex
GO:0030135	79	2	0.0141076252186705	coated vesicle
GO:0005578	303	3	0.0313180435461735	extracellular matrix (sensu Metazoa)
GO:0031012	304	3	0.0315836931037519	extracellular matrix
GO:0030141	18	1	0.0409102713001872	secretory granule
GO:0019717	19	1	0.0431357956735476	synaptosome
GO:0005856	612	4	0.048734503998626	cytoskeleton
GO:0016585	23	1	0.0519889296935718	chromatin remodeling complex
GO:0030176	26	1	0.0585776544125273	integral to endoplasmic reticulum membrane
GO:0005625	183	2	0.0661005015617147	soluble fraction
GO:0031227	32	1	0.071624823740659	intrinsic to endoplasmic reticulum membrane
GO:0005905	38	1	0.084500094166173	coated pit
GO:0000786	40	1	0.0887540230535118	nucleosome
GO:0005623	7802	20	0.130567900755194	cell
GO:0009986	61	1	0.132301198851853	cell surface
GO:0045211	66	1	0.14237421272526	postsynaptic membrane
GO:0031301	76	1	0.162187940563367	integral to organelle membrane
GO:0005576	949	4	0.170245806384392	extracellular region
GO:0005789	84	1	0.177725016406144	endoplasmic reticulum membrane
GO:0043228	977	4	0.183389785108259	non-membrane-bound organelle
GO:0043232	977	4	0.183389785108259	intracellular non-membrane-bound organelle
GO:0031300	90	1	0.189197772028406	intrinsic to organelle membrane
GO:0042175	90	1	0.189197772028406	nuclear envelope-endoplasmic reticulum network
GO:0000785	100	1	0.207981808978392	chromatin
GO:0000267	698	3	0.216244607462632	cell fraction
GO:0016020	3514	10	0.264904854086803	membrane
GO:0005874	132	1	0.265351722870038	microtubule
GO:0005624	521	2	0.342017093482708	membrane fraction
GO:0015630	191	1	0.360932445583668	microtubule cytoskeleton
GO:0005694	203	1	0.378868951883008	chromosome
GO:0005654	221	1	0.404879536950394	nucleoplasm

GO:0012505	246	1	0.439293601385115	endomembrane system
GO:0005886	1472	4	0.451735044084065	plasma membrane
GO:0005737	2530	6	0.556340918895366	cytoplasm
GO:0031090	377	1	0.590828070585219	organelle membrane
GO:0016021	2674	6	0.619978030912506	integral to membrane
GO:0031224	2681	6	0.622972665132605	intrinsic to membrane
GO:0043226	4515	10	0.666147394945611	organelle
GO:0043229	4515	10	0.666147394945611	intracellular organelle
GO:0005783	483	1	0.684071390122655	endoplasmic reticulum
GO:0005622	5419	12	0.691133369402011	intracellular
GO:0005887	1057	2	0.721958817050856	integral to plasma membrane
GO:0031226	1062	2	0.724518153308085	intrinsic to plasma membrane
GO:0043234	1247	2	0.806874505265619	protein complex
GO:0043227	3955	7	0.884759585580149	membrane-bound organelle
GO:0043231	3955	7	0.884759585580149	intracellular membrane-bound organelle
GO:0005634	2704	3	0.972993606049484	nucleus
GO:0005575	8637	20	1	cellular_component

7. Skin

7.1 Skin selective genes (22 genes)

Symbol	Description	Chromosome	Chromosome Location	LocusLink	Cytoband	UniGene
SCGB2A2	secretoglobin, family 2A, member 2	11	61794205	4250	11q13	Hs.46452
KRT1	keratin 1 (epidermolytic hyperkeratosis)	12	-51354720	3848	12q12-q13	Hs.80828
KRT14	keratin 14 (epidermolysis bullosa simplex, Dowling-Meara, Koebner)	17	-36992058	3861	17q12-q21	Hs.2785
SCGB1D2	secretoglobin, family 1D, member 2	11	61766299	10647	11q13	Hs.204096
KRT15	keratin 15	17	-36923523	3866	17q21.2	Hs.2785
DSP	desmoplakin	6	7486868	1832	6p24	Hs.519873
KRT5	keratin 5 (epidermolysis bullosa simplex, Dowling-Meara/Kobner/Weber-Cockayne types)	12	-51194627	3852	12q12-q13	Hs.433845
LOR	loricrin	1	151498802	4014	1q21	Hs.251680
TMEM45A	transmembrane protein 45A	3	101694152	55076	3q12.2	Hs.126598
FABP4	fatty acid binding protein 4, adipocyte	8	-82553489	2167	8q21	Hs.391561
SERPINB5	serpin peptidase inhibitor, clade B (ovalbumin), member 5	18	59295198	5268	18q21.3	Hs.55279
KRT10	keratin 10 (epidermolytic hyperkeratosis, keratosis palmaris et plantaris)	17	-36227895	3858	17q21	Hs.99936
CXCL14	chemokine (C-X-C motif) ligand 14	5	-134934274	9547	5q31	Hs.483444
PERP	PERP, TP53 apoptosis effector	6	-138453618	64065	6q24	Hs.520421
KRT17	keratin 17	17	-37029220	3872	17q12-q21	Hs.2785
KRT6B	keratin 6B	12	-51126719	3854	12q12-q13	Hs.433845
SPINK5	serine peptidase inhibitor, Kazal type 5	5	147423758	11005	5q32	Hs.331555
CSTA	cystatin A (stefin A)	3	123526700	1475	3q21	Hs.518198
KRT2	keratin 2 (epidermal ichthyosis bullosa of Siemens)	12	-51324609	3849	12q11-q13	Hs.707
S100A2	S100 calcium binding protein A2	1	-151800210	6273	1q21	Hs.516484
ADIPOQ	adiponectin, C1Q and collagen domain containing	3	188043156	9370	3q27	Hs.80485
NA	NA	NA	NA	650893	NA	NA

7.2 GO: Biological Process

GO ID	genes present in chip	genes present in tissues	p-value	GO term
GO:0007398	79	11	3.37E-19	ectoderm development
GO:0008544	70	10	1.67E-17	epidermis development
GO:0009888	158	11	9.48E-16	histogenesis
GO:0009887	807	11	4.80E-08	organogenesis
GO:0048513	872	11	1.08E-07	organ development
GO:0009653	1051	11	7.38E-07	morphogenesis
GO:0018149	15	3	2.98E-06	peptide cross-linking
GO:0007275	1543	12	3.99E-06	development
GO:0030216	18	3	5.32E-06	keratinocyte differentiation
GO:0009913	14	2	0.000336578	epidermal cell differentiation
GO:0045765	25	2	0.00109528	regulation of angiogenesis
GO:0042640	1	1	0.001992914	anagen
GO:0001867	2	1	0.003982077	complement activation, lectin pathway
GO:0001943	2	1	0.003982077	hair follicle maturation
GO:0001942	3	1	0.005967494	hair follicle development
GO:0035315	3	1	0.005967494	hair cell differentiation
GO:0042303	3	1	0.005967494	molting cycle
GO:0042633	3	1	0.005967494	hair cycle
GO:0001525	65	2	0.007244242	angiogenesis
GO:0030154	409	4	0.007645124	cell differentiation
GO:0001568	68	2	0.007905876	blood vessel development
GO:0048514	68	2	0.007905876	blood vessel morphogenesis
GO:0001944	69	2	0.008132298	vasculature development
GO:0030236	5	1	0.009927122	anti-inflammatory response
GO:0000004	465	4	0.011924005	biological process unknown
GO:0042730	9	1	0.017801731	fibrinolysis
GO:0045580	10	1	0.01976112	regulation of T cell differentiation
GO:0016525	11	1	0.021716817	negative regulation of angiogenesis
GO:0030193	11	1	0.021716817	regulation of blood coagulation
GO:0030195	11	1	0.021716817	negative regulation of blood coagulation
GO:0030855	11	1	0.021716817	epithelial cell differentiation
GO:0031424	12	1	0.023668829	keratinization
GO:0045619	12	1	0.023668829	regulation of lymphocyte differentiation
GO:0050818	13	1	0.025617162	regulation of coagulation
GO:0050819	13	1	0.025617162	negative regulation of coagulation
GO:0002009	14	1	0.027561822	morphogenesis of an epithelium
GO:0050777	16	1	0.031440151	negative regulation of immune response
GO:0006959	141	2	0.031441671	humoral immune response
GO:0030217	18	1	0.035303869	T cell differentiation
GO:0051241	20	1	0.039153027	negative regulation of organismal physiological process
GO:0006956	30	1	0.05818222	complement activation
GO:0009613	458	3	0.059973287	response to pest, pathogen or parasite

GO:0050863	31	1	0.060065437	regulation of T cell activation
GO:0043207	463	3	0.061579306	response to external biotic stimulus
GO:0030098	34	1	0.065693772	lymphocyte differentiation
GO:0043062	35	1	0.0675628	extracellular structure organization and biogenesis
GO:0030198	35	1	0.0675628	extracellular matrix organization and biogenesis
GO:0051249	41	1	0.078703024	regulation of lymphocyte activation
GO:0050865	43	1	0.082388412	regulation of cell activation
GO:0006979	44	1	0.084225881	response to oxidative stress
GO:0042110	45	1	0.086059875	T cell activation
GO:0045595	55	1	0.104210019	regulation of cell differentiation
GO:0006817	57	1	0.107798958	phosphate transport
GO:0006800	61	1	0.114936134	oxygen and reactive oxygen species metabolism
GO:0019735	77	1	0.142949204	antimicrobial humoral response (sensu Vertebrata)
GO:0050776	77	1	0.142949204	regulation of immune response
GO:0019730	78	1	0.14467192	antimicrobial humoral response
GO:0007596	78	1	0.14467192	blood coagulation
GO:0050817	79	1	0.146391364	coagulation
GO:0009611	341	2	0.146408602	response to wounding
GO:0046649	81	1	0.149820464	lymphocyte activation
GO:0006955	684	3	0.15125503	immune response
GO:0007599	83	1	0.153236551	hemostasis
GO:0042060	85	1	0.156639672	wound healing
GO:0030097	87	1	0.160029872	hemopoiesis
GO:0048534	88	1	0.161720142	hemopoietic or lymphoid organ development
GO:0050878	94	1	0.171794494	regulation of body fluids
GO:0045321	97	1	0.176788684	immune cell activation
GO:0001775	98	1	0.178447084	cell activation
GO:0006935	101	1	0.183403373	chemotaxis
GO:0042330	101	1	0.183403373	taxis
GO:0006952	751	3	0.184366347	defense response
GO:0016064	103	1	0.186691862	humoral defense mechanism (sensu Vertebrata)
GO:0015698	106	1	0.191601153	inorganic anion transport
GO:0006820	128	1	0.226756805	anion transport
GO:0051239	133	1	0.234542811	regulation of organismal physiological process
GO:0009607	857	3	0.240559862	response to biotic stimulus
GO:0006950	881	3	0.25377413	response to stress
GO:0050793	168	1	0.287007247	regulation of development
GO:0009605	963	3	0.29983984	response to external stimulus
GO:0042221	183	1	0.308435884	response to chemical substance
GO:0006954	185	1	0.311246641	inflammatory response
GO:0006928	205	1	0.338767064	cell motility
GO:0040011	205	1	0.338767064	locomotion
GO:0043118	602	2	0.339737187	negative regulation of physiological process
GO:0007626	209	1	0.344145039	locomotory behavior
GO:0006464	1087	3	0.370868087	protein modification
GO:0048519	688	2	0.403333467	negative regulation of biological process

GO:0043412	1160	3	0.412633118	biopolymer modification
GO:0007610	269	1	0.420019011	behavior
GO:0009628	318	1	0.475752397	response to abiotic stimulus
GO:0006091	446	1	0.598447314	generation of precursor metabolites and energy
GO:0050874	1531	3	0.609991997	organismal physiological process
GO:0007267	465	1	0.61416001	cell-cell signaling
GO:0006915	471	1	0.619000034	apoptosis
GO:0012501	473	1	0.620600592	programmed cell death
GO:0050896	1578	3	0.632202964	response to stimulus
GO:0008219	498	1	0.640079243	cell death
GO:0016265	501	1	0.642352154	death
GO:0006811	515	1	0.652780995	ion transport
GO:0007155	518	1	0.654978062	cell adhesion
GO:0044267	2109	3	0.82777009	cellular protein metabolism
GO:0019538	2129	3	0.83317608	protein metabolism
GO:0043283	2240	3	0.860828314	biopolymer metabolism
GO:0044260	2298	3	0.873761421	cellular macromolecule metabolism
GO:0043170	2405	3	0.89508408	macromolecule metabolism
GO:0006810	1802	2	0.90030838	transport
GO:0051234	1841	2	0.907555039	establishment of localization
GO:0051179	1857	2	0.910393424	localization
GO:0050791	2407	2	0.971628356	regulation of physiological process
GO:0050789	2689	2	0.985169671	regulation of biological process
GO:0007154	2730	2	0.986550414	cell communication
GO:0007165	2168	1	0.992888633	signal transduction
GO:0051244	2319	1	0.995237405	regulation of cellular physiological process
GO:0044237	5026	5	0.995858672	cellular metabolism
GO:0050794	2488	1	0.996992074	regulation of cellular process
GO:0050875	7018	9	0.997816122	cellular physiological process
GO:0008152	5262	5	0.997924741	metabolism
GO:0044238	4820	3	0.999761399	primary metabolism
GO:0007582	7846	10	0.999820264	physiological process
GO:0009987	8200	10	0.999989646	cellular process
GO:0008150	9032	18	1	biological_process

7.3 GO: Molecular Function

GO ID	genes present in chip	genes present in tissues	p-value	GO term
GO:0005200	86	9	2.19561064892188e-14	structural constituent of cytoskeleton
GO:0005198	488	12	1.02002993415826e-11	structural molecule activity
GO:0030280	5	2	3.7439932888596e-05	structural constituent of epidermis
GO:0030674	69	3	0.000322870103409482	protein binding, bridging
GO:0004866	99	3	0.000930739960623984	endopeptidase inhibitor activity
GO:0030414	100	3	0.000958318791743724	protease inhibitor activity
GO:0004857	187	3	0.00569680254157351	enzyme inhibitor activity
GO:0004867	64	2	0.00703775496861771	serine-type endopeptidase inhibitor activity
GO:0005504	17	1	0.0333474183587451	fatty acid binding
GO:0004869	19	1	0.0372006399733341	cysteine protease inhibitor activity
GO:0005515	3709	11	0.0728535538354576	protein binding
GO:0008009	40	1	0.0767906164190206	chemokine activity
GO:0042379	40	1	0.0767906164190206	chemokine receptor binding
GO:0030234	509	3	0.0774792641217441	enzyme regulator activity
GO:0001664	42	1	0.0804795671650752	G-protein-coupled receptor binding
GO:0005496	42	1	0.0804795671650752	steroid binding
GO:0005179	90	1	0.164936964378574	hormone activity
GO:0005529	103	1	0.186513389194452	sugar binding
GO:0005554	522	2	0.279089017216152	molecular function unknown
GO:0005102	528	2	0.283614830660854	receptor binding
GO:0005125	181	1	0.305285457833643	cytokine activity
GO:0030246	181	1	0.305285457833643	carbohydrate binding
GO:0008289	298	1	0.453096371293894	lipid binding
GO:0005488	7228	14	0.696164322378263	binding
GO:0005509	646	1	0.736413584547134	calcium ion binding
GO:0004871	1837	3	0.738040147475825	signal transducer activity
GO:0004872	1060	1	0.89352785760845	receptor activity
GO:0043169	2313	1	0.994911776940366	cation binding
GO:0046872	2495	1	0.996867683108676	metal ion binding
GO:0043167	2556	1	0.99734524073219	ion binding
GO:0003674	9541	19	1	molecular_function

7.4 GO: Cellular Component

GO ID	genes present in chip	genes present in tissues	p-value	GO term
GO:0045111	65	9	6.8583185532415e-15	intermediate filament cytoskeleton
GO:0005882	65	9	6.8583185532415e-15	intermediate filament
GO:0005856	612	12	1.07346355106558e-09	cytoskeleton
GO:0043228	977	12	2.18080253289123e-07	non-membrane-bound organelle
GO:0043232	977	12	2.18080253289123e-07	intracellular non-membrane-bound organelle
GO:0001533	12	3	2.30549816450911e-06	cornified envelope
GO:0042599	2	1	0.00462614312229792	lamellar body
GO:0005913	7	1	0.0161027168120159	cell-cell adherens junction
GO:0008372	523	4	0.0296647380199276	cellular component unknown
GO:0005626	15	1	0.0342041558631354	insoluble fraction
GO:0005912	18	1	0.0409102713001872	adherens junction
GO:0030141	18	1	0.0409102713001872	secretory granule
GO:0048471	22	1	0.0497829702513223	perinuclear region
GO:0005938	28	1	0.0629459443213919	cell cortex
GO:0005576	949	4	0.170245806384392	extracellular region
GO:0005911	82	1	0.173866613649375	intercellular junction
GO:0005789	84	1	0.177725016406144	endoplasmic reticulum membrane
GO:0043226	4515	13	0.180072887520944	organelle
GO:0043229	4515	13	0.180072887520944	intracellular organelle
GO:0005622	5419	15	0.184318911307112	intracellular
GO:0042175	90	1	0.189197772028406	nuclear envelope-endoplasmic reticulum network
GO:0030054	93	1	0.1948769637985	cell junction
GO:0005615	385	2	0.223361327541223	extracellular space
GO:0016023	122	1	0.247861944558041	cytoplasmic membrane-bound vesicle
GO:0031410	124	1	0.251391240214359	cytoplasmic vesicle
GO:0005625	183	1	0.348702582114350	soluble fraction
GO:0012505	246	1	0.439293601385115	endomembrane system
GO:0005829	276	1	0.478096487937146	cytosol
GO:0000267	698	2	0.488861623525991	cell fraction
GO:0031090	377	1	0.590828070585219	organelle membrane
GO:0005783	483	1	0.684071390122655	endoplasmic reticulum
GO:0016020	3514	5	0.955304284861446	membrane
GO:0005737	2530	3	0.959292053733415	cytoplasm
GO:0005623	7802	16	0.962109429137035	cell
GO:0005886	1472	1	0.97628083033978	plasma membrane
GO:0016021	2674	2	0.994007214307388	integral to membrane
GO:0031224	2681	2	0.994126557439681	intrinsic to membrane
GO:0043227	3955	1	0.999995287165732	membrane-bound organelle
GO:0043231	3955	1	0.999995287165732	intracellular membrane-bound organelle

GO:0005575	8637	20	1 cellular_component

8. GlobusPallidus

8.1 GlobusPallidus selective genes (16 genes)

Symbol	Description	Chromosome	Chromosome Location	LocusLink	Cytoband	UniGene
PLP1	proteolipid protein 1 (Pelizaeus-Merzbacher disease, spastic paraplegia 2, uncomplicated)	X	102918409	5354	Xq22	Hs.1787
GPM6A	glycoprotein M6A	4	-176791082	2823	4q34	Hs.75819
SNAP25	synaptosomal-associated protein, 25kDa	20	10147476	6616	20p12-p11.2	Hs.167317
RTN1	reticulon 1	14	-59132446	6252	14q23.1	Hs.368626
GRIA2	glutamate receptor, ionotropic, AMPA 2	4	158361296	2891	4q32-q33	Hs.32763
SYT1	synaptotagmin I	12	78135413	6857	12cen-q21	Hs.310545
STMN2	stathmin-like 2	8	80685934	11075	8q21.13	Hs.521651
GFAP	glial fibrillary acidic protein	17	-40338518	2670	17q21	Hs.514227
VSNL1	visinin-like 1	2	17585287	7447	2p24.3	Hs.444212
CCK	cholecystokinin	3	-42274321	885	3p22-p21.3	Hs.458426
DIRAS2	DIRAS family, GTP-binding RAS-like 2	9	-92411933	54769	9q22.2	Hs.165636
DNM1	dynamin 1	9	130005483	1759	9q34	Hs.522413
DCAMKL1	doublecortin and CaM kinase-like 1	13	-35243477	9201	13q13	Hs.507755
NEF3	neurofilament 3 (150kDa medium)	8	24827211	4741	8p21	Hs.458657 Hs.615287
MT3	metallothionein 3 (growth inhibitory factor (neurotrophic))	16	55180767	4504	16q13	Hs.73133
NRGN	neurogranin (protein kinase C substrate, RC3)	11	124114951	4900	11q24	Hs.524116

8.2 GO: Biological Process

GO ID	genes present in chip	genes present in tissues	p-value	GO term
GO:0007154	2730	10	5.02E-05	cell communication
GO:0007268	192	4	5.81E-05	synaptic transmission
GO:0019226	198	4	6.55E-05	transmission of nerve impulse
GO:0030182	13	2	0.000104422	neuron differentiation
GO:0007269	19	2	0.000228014	neurotransmitter secretion
GO:0045055	22	2	0.000307406	regulated secretory pathway
GO:0016192	296	4	0.000310847	vesicle-mediated transport
GO:0001505	41	2	0.001077529	regulation of neurotransmitter levels
GO:0019430	1	1	0.001217892	removal of superoxide radicals
GO:0007267	465	4	0.001712888	cell-cell signaling
GO:0050877	475	4	0.001853667	neurophysiological process
GO:0006887	55	2	0.001933305	Exocytosis
GO:0016081	2	1	0.002434435	synaptic vesicle docking during exocytosis
GO:0050774	2	1	0.002434435	negative regulation of dendrite morphogenesis
GO:0017157	3	1	0.003649631	regulation of exocytosis
GO:0050796	3	1	0.003649631	regulation of insulin secretion
GO:0050773	3	1	0.003649631	regulation of dendrite morphogenesis
GO:0001504	4	1	0.004863482	neurotransmitter uptake
GO:0050768	4	1	0.004863482	negative regulation of neurogenesis
GO:0007165	2168	7	0.005912027	signal transduction
GO:0005513	5	1	0.006075987	calcium ion sensing
GO:0016358	5	1	0.006075987	dendrite morphogenesis
GO:0030073	5	1	0.006075987	insulin secretion
GO:0016079	6	1	0.00728715	synaptic vesicle exocytosis
GO:0030072	6	1	0.00728715	peptide hormone secretion
GO:0009593	6	1	0.00728715	detection of chemical substance
GO:0046883	8	1	0.009705451	regulation of hormone secretion
GO:0007399	385	3	0.009819374	neurogenesis
GO:0006810	1802	6	0.011465365	transport
GO:0030154	409	3	0.011588507	cell differentiation
GO:0051234	1841	6	0.012757146	establishment of localization
GO:0051179	1857	6	0.013317505	localization
GO:0048489	11	1	0.013322861	synaptic vesicle transport
GO:0051260	11	1	0.013322861	protein homooligomerization
GO:0046879	11	1	0.013322861	hormone secretion
GO:0045045	161	2	0.015627777	secretory pathway
GO:0001666	14	1	0.016928256	response to hypoxia
GO:0006801	15	1	0.018127389	superoxide metabolism
GO:0050767	15	1	0.018127389	regulation of neurogenesis
GO:0006904	17	1	0.020521669	vesicle docking during exocytosis
GO:0048278	18	1	0.021716817	vesicle docking
GO:0046903	208	2	0.025317734	secretion

GO:0016197	22	1	0.026484165	endosome transport
GO:0007275	1543	5	0.027002678	development
GO:0007032	24	1	0.02885991	endosome organization and biogenesis
GO:0051046	24	1	0.02885991	regulation of secretion
GO:0051259	24	1	0.02885991	protein oligomerization
GO:0045792	25	1	0.030045804	negative regulation of cell size
GO:0030308	25	1	0.030045804	negative regulation of cell growth
GO:0006898	28	1	0.033595594	receptor mediated endocytosis
GO:0045926	29	1	0.034776231	negative regulation of growth
GO:0006836	33	1	0.039485675	neurotransmitter transport
GO:0031175	46	1	0.054647471	neurite morphogenesis
GO:0051049	46	1	0.054647471	regulation of transport
GO:0051093	49	1	0.058115302	negative regulation of development
GO:0009887	807	3	0.06812996	organogenesis
GO:0006800	61	1	0.071871286	oxygen and reactive oxygen species metabolism
GO:0048513	872	3	0.082226311	organ development
GO:0007242	879	3	0.083820613	intracellular signaling cascade
GO:0050874	1531	4	0.100354622	organismal physiological process
GO:0006875	93	1	0.107665763	metal ion homeostasis
GO:0001558	94	1	0.108763836	regulation of cell growth
GO:0030003	98	1	0.113143856	cation homeostasis
GO:0007417	101	1	0.116416019	central nervous system development
GO:0006873	103	1	0.118591359	cell ion homeostasis
GO:0040008	104	1	0.119677202	regulation of growth
GO:0009582	109	1	0.1250882	detection of abiotic stimulus
GO:0006813	111	1	0.12724412	potassium ion transport
GO:0050801	111	1	0.12724412	ion homeostasis
GO:0009653	1051	3	0.127341056	morphogenesis
GO:0006897	115	1	0.131541477	endocytosis
GO:0019725	122	1	0.139015592	cell homeostasis
GO:0008361	123	1	0.140078536	regulation of cell size
GO:0016049	123	1	0.140078536	cell growth
GO:0040007	154	1	0.172443947	growth
GO:0042592	157	1	0.175516553	homeostasis
GO:0050793	168	1	0.186694255	regulation of development
GO:0000902	183	1	0.201714587	cellular morphogenesis
GO:0015672	223	1	0.240544222	monovalent inorganic cation transport
GO:0007264	227	1	0.244331166	small GTPase mediated signal transduction
GO:0006118	256	1	0.271277235	electron transport
GO:0016043	842	2	0.273776047	cell organization and biogenesis
GO:0030001	269	1	0.283070478	metal ion transport
GO:0009581	313	1	0.321710299	detection of external stimulus
GO:0009628	318	1	0.325979187	response to abiotic stimulus
GO:0009987	8200	11	0.345191909	cellular process
GO:0006812	346	1	0.349436898	cation transport
GO:0006468	444	1	0.425814343	protein amino acid phosphorylation

GO:0006091	446	1	0.427284386	generation of precursor metabolites and energy
GO:0046907	448	1	0.428751007	intracellular transport
GO:0008283	473	1	0.446797919	cell proliferation
GO:0006811	515	1	0.475953456	ion transport
GO:0016310	522	1	0.480674565	phosphorylation
GO:0051243	583	1	0.520208486	negative regulation of cellular physiological process
GO:0043118	602	1	0.531951245	negative regulation of physiological process
GO:0048523	640	1	0.554656457	negative regulation of cellular process
GO:0006793	641	1	0.555240201	phosphorus metabolism
GO:0006796	641	1	0.555240201	phosphate metabolism
GO:0048519	688	1	0.58190421	negative regulation of biological process
GO:0006996	693	1	0.584653515	organelle organization and biogenesis
GO:0050791	2407	3	0.594793398	regulation of physiological process
GO:0050896	1578	2	0.59748687	response to stimulus
GO:0006950	881	1	0.676845261	response to stress
GO:0050789	2689	3	0.681510807	regulation of biological process
GO:0009605	963	1	0.710880244	response to external stimulus
GO:0006464	1087	1	0.756192212	protein modification
GO:0044267	2109	2	0.766718066	cellular protein metabolism
GO:0019538	2129	2	0.771869513	protein metabolism
GO:0043412	1160	1	0.779746564	biopolymer modification
GO:0044260	2298	2	0.81205658	cellular macromolecule metabolism
GO:0051244	2319	2	0.816644492	regulation of cellular physiological process
GO:0043170	2405	2	0.834536523	macromolecule metabolism
GO:0050794	2488	2	0.850482245	regulation of cellular process
GO:0043283	2240	1	0.956601147	biopolymer metabolism
GO:0050875	7018	6	0.980162209	cellular physiological process
GO:0044237	5026	3	0.986798027	cellular metabolism
GO:0008152	5262	3	0.991757133	metabolism
GO:0044238	4820	2	0.99693221	primary metabolism
GO:0007582	7846	6	0.99870278	physiological process
GO:0008150	9032	11	1	biological_process

8.3 GO: Molecular Function

GO ID	genes present in chip	genes present in tissues	p-value	GO term
GO:0017075	2	1	0.00314201733795061	syntaxin-1 binding
GO:0004971	3	1	0.0047095684166728	alpha-amino-3-hydroxy-5-methyl-4-isoxazole propionate selective glutamate receptor activity
GO:0005200	86	2	0.0078136676301911	structural constituent of cytoskeleton
GO:0050750	6	1	0.00939842896355658	low-density lipoprotein receptor binding
GO:0019905	6	1	0.00939842896355658	syntaxin binding
GO:0000149	6	1	0.00939842896355658	SNARE binding
GO:0015277	7	1	0.0109567953574253	kainate selective glutamate receptor activity
GO:0005545	9	1	0.0140666662155583	phosphatidylinositol binding
GO:0004970	14	1	0.0218014607908067	ionotropic glutamate receptor activity
GO:0005234	14	1	0.0218014607908067	glutamate-gated ion channel activity
GO:0005198	488	3	0.038221655377793	structural molecule activity
GO:0008066	29	1	0.0446673953795858	glutamate receptor activity
GO:0016209	31	1	0.047678211683621	antioxidant activity
GO:0005525	226	2	0.0478955999372431	GTP binding
GO:0019001	235	2	0.0513769987804119	guanyl nucleotide binding
GO:0005231	39	1	0.0596331373611864	excitatory extracellular ligand-gated ion channel activity
GO:0005507	49	1	0.074379834461411	copper ion binding
GO:0005230	57	1	0.0860214837504307	extracellular ligand-gated ion channel activity
GO:0015276	84	1	0.124310984890419	ligand-gated ion channel activity
GO:0005179	90	1	0.132613917586207	hormone activity
GO:0005267	90	1	0.132613917586207	potassium channel activity
GO:0003774	98	1	0.143570229691105	motor activity
GO:0004871	1837	5	0.145366220552367	signal transducer activity
GO:0009055	104	1	0.151702548332266	electron carrier activity
GO:0005516	112	1	0.162433557755499	calmodulin binding
GO:0003924	121	1	0.174354441193993	GTPase activity
GO:0035091	132	1	0.188709351541957	phosphoinositide binding
GO:0005057	132	1	0.188709351541957	receptor signaling protein activity
GO:0005102	528	2	0.200106286859181	receptor binding
GO:0005489	152	1	0.214214169102267	electron transporter activity
GO:0005215	1030	3	0.215384018904198	transporter activity
GO:0005543	169	1	0.235302822087327	phospholipid binding
GO:0005261	188	1	0.258246832974479	cation channel activity
GO:0005509	646	2	0.270027004341341	calcium ion binding
GO:0005506	212	1	0.286310620010377	iron ion binding
GO:0017076	1248	3	0.311818826265322	purine nucleotide binding
GO:0005216	252	1	0.330892018276936	ion channel activity
GO:0015268	274	1	0.354289949611008	alpha-type channel activity
GO:0005515	3709	7	0.355582919209048	protein binding
GO:0015267	284	1	0.364670917964515	channel or pore class transporter activity
GO:0008289	298	1	0.37894279289445	lipid binding
GO:0000166	1432	3	0.396187143959770	nucleotide binding

GO:0004674	326	1	0.40659336593463	protein serine/threonine kinase activity
GO:0005386	398	1	0.472510170903929	carrier activity
GO:0017111	410	1	0.482807910438765	nucleoside-triphosphatase activity
GO:0008324	421	1	0.492082382395531	cation transporter activity
GO:0016462	430	1	0.499554881661969	pyrophosphatase activity
GO:0016818	432	1	0.501201442686404	hydrolase activity, acting on acid anhydrides, in phosphorus-containing anhydrides
GO:0016817	433	1	0.502022825753668	hydrolase activity, acting on acid anhydrides
GO:0004672	447	1	0.513390437079473	protein kinase activity
GO:0005554	522	1	0.57027516630986	molecular function unknown
GO:0015075	523	1	0.57098986555649	ion transporter activity
GO:0016773	532	1	0.577372421197488	phosphotransferase activity, alcohol group as acceptor
GO:0016301	609	1	0.628468138531248	kinase activity
GO:0004888	694	1	0.678147673929576	transmembrane receptor activity
GO:0016772	708	1	0.685709306556799	transferase activity, transferring phosphorus-containing groups
GO:0043169	2313	3	0.743036132395058	cation binding
GO:0046872	2495	3	0.793790524176482	metal ion binding
GO:0005524	970	1	0.80000344938118	ATP binding
GO:0043167	2556	3	0.809064422405979	ion binding
GO:0030554	1028	1	0.819385218377772	adenyl nucleotide binding
GO:0004872	1060	1	0.829313370222208	receptor activity
GO:0016740	1169	1	0.859439994464422	transferase activity
GO:0005488	7228	10	0.867970636283457	binding
GO:0008270	1287	1	0.886417223805791	zinc ion binding
GO:0016787	1424	1	0.91165431472959	hydrolase activity
GO:0046914	1596	1	0.935947083563505	transition metal ion binding
GO:0003824	3409	2	0.987735439060794	catalytic activity
GO:0003674	9541	15	1	molecular_function

8.4 GO: Cellular Component

GO ID	genes present in chip	genes present in tissues	p-value	GO term
GO:0008021	27	2	0.000836840940732341	synaptic vesicle
GO:0045111	65	2	0.00478765282906668	intermediate filament cytoskeleton
GO:0005882	65	2	0.00478765282906668	intermediate filament
GO:0030136	65	2	0.00478765282906668	clathrin-coated vesicle
GO:0030135	79	2	0.0070004753190222	coated vesicle
GO:0005883	5	1	0.00808029952179512	neurofilament
GO:0045202	98	2	0.0106114804843621	synapse
GO:0030424	7	1	0.0112954101822078	axon
GO:0016023	122	2	0.0161174217348237	cytoplasmic membrane-bound vesicle
GO:0031410	124	2	0.0166216915047857	cytoplasmic vesicle
GO:0043005	17	1	0.0272263152734905	neuron projection
GO:0019717	19	1	0.0303837540145840	synaptosome
GO:0005625	183	2	0.0343747947431459	soluble fraction
GO:0030176	26	1	0.0413600041605174	integral to endoplasmic reticulum membrane
GO:0031227	32	1	0.0506762699833315	intrinsic to endoplasmic reticulum membrane
GO:0005905	38	1	0.0599084068802395	coated pit
GO:0016020	3514	9	0.0647118211923708	membrane
GO:0005856	612	3	0.071723622133866	cytoskeleton
GO:0005578	303	2	0.084582161345426	extracellular matrix (sensu Metazoa)
GO:0031012	304	2	0.0850643161866862	extracellular matrix
GO:0009986	61	1	0.0945314765141463	cell surface
GO:0000267	698	3	0.0979680994683056	cell fraction
GO:0045211	66	1	0.101899816575938	postsynaptic membrane
GO:0042995	70	1	0.107754358640974	cell projection
GO:0031301	76	1	0.116469755423438	integral to organelle membrane
GO:0005789	84	1	0.127967345708868	endoplasmic reticulum membrane
GO:0031300	90	1	0.136499193008033	intrinsic to organelle membrane
GO:0042175	90	1	0.136499193008033	nuclear envelope-endoplasmic reticulum network
GO:0005576	949	3	0.193297163150096	extracellular region
GO:0005874	132	1	0.194086398942311	microtubule
GO:0005886	1472	4	0.204969445077003	plasma membrane
GO:0043228	977	3	0.205292565340337	non-membrane-bound organelle
GO:0043232	977	3	0.205292565340337	intracellular non-membrane-bound organelle
GO:0005624	521	2	0.205298778790423	membrane fraction
GO:0005623	7802	14	0.240609137397464	cell
GO:0016021	2674	6	0.244152924121058	integral to membrane
GO:0031224	2681	6	0.246302068190529	intrinsic to membrane
GO:0015630	191	1	0.268977711793389	microtubule cytoskeleton
GO:0012505	246	1	0.332921519374537	endomembrane system
GO:0031090	377	1	0.464905212695089	organelle membrane
GO:0005887	1057	2	0.525436436444417	integral to plasma membrane
GO:0031226	1062	2	0.528125598023989	intrinsic to plasma membrane

GO:0005783	483	1	0.553483316383632	endoplasmic reticulum
GO:0005737	2530	3	0.825189448586886	cytoplasm
GO:0043226	4515	6	0.83479354460606	organelle
GO:0043229	4515	6	0.83479354460606	intracellular organelle
GO:0005622	5419	7	0.895221168686871	intracellular
GO:0043227	3955	3	0.985348718613163	membrane-bound organelle
GO:0043231	3955	3	0.985348718613163	intracellular membrane-bound organelle
GO:0005575	8637	14	1	cellular_component

9. CiliaryGanglion

9.1 CiliaryGanglion selective genes (6 genes)

Symbol	Description	Chromosome	Chromosome Location	LocusLink	Cytoband	UniGene
NEFH	neurofilament, heavy polypeptide 200kDa	22	28206218	4744	22q12.2	Hs.198760
ADIPOQ	adiponectin, C1Q and collagen domain containing	3	188043156	9370	3q27	Hs.80485
FABP4	fatty acid binding protein 4, adipocyte	8	-82553489	2167	8q21	Hs.391561
NEF3	neurofilament 3 (150kDa medium)	8	24827211	4741	8p21	Hs.458657 Hs.615287
STMN2	stathmin-like 2	8	80685934	11075	8q21.13	Hs.521651
LPL	lipoprotein lipase	8	19841057	4023	8p22	Hs.180878

9.2 GO: Biological Process

GO ID	genes present in chip	genes present in tissues	p-value	GO term
GO:0030182	13	1	0.007177532	neuron differentiation
GO:0006334	48	1	0.026297014	nucleosome assembly
GO:0031497	56	1	0.030625538	chromatin assembly
GO:0006817	57	1	0.031165519	phosphate transport
GO:0016042	67	1	0.03655211	lipid catabolism
GO:0006333	91	1	0.049382217	chromatin assembly or disassembly
GO:0008015	103	1	0.055745808	circulation
GO:0015698	106	1	0.057331368	inorganic anion transport
GO:0006631	124	1	0.066800061	fatty acid metabolism
GO:0006820	128	1	0.068893845	anion transport
GO:0006325	186	1	0.098833753	establishment and/or maintenance of chromatin architecture
GO:0006323	193	1	0.102394464	DNA packaging
GO:0006461	194	1	0.102902217	protein complex assembly
GO:0007001	221	1	0.116524934	chromosome organization and biogenesis (sensu Eukaryota)
GO:0051276	236	1	0.124021285	chromosome organization and biogenesis
GO:0019752	359	1	0.18359214	carboxylic acid metabolism
GO:0006082	361	1	0.184533244	organic acid metabolism
GO:0044255	383	1	0.194828228	cellular lipid metabolism
GO:0007399	385	1	0.195758955	neurogenesis
GO:0007275	1543	2	0.20429482	development
GO:0030154	409	1	0.206860697	cell differentiation
GO:0006091	446	1	0.223735384	generation of precursor metabolites and energy
GO:0006629	469	1	0.234079435	lipid metabolism
GO:0006259	489	1	0.242984356	DNA metabolism
GO:0006811	515	1	0.254436726	ion transport
GO:0006810	1802	2	0.261699711	transport
GO:0051234	1841	2	0.270562726	establishment of localization
GO:0051179	1857	2	0.274211733	localization
GO:0006996	693	1	0.329173437	organelle organization and biogenesis
GO:0009056	756	1	0.354139349	catabolism
GO:0009887	807	1	0.373800532	organogenesis
GO:0016043	842	1	0.387014202	cell organization and biogenesis
GO:0048513	872	1	0.398161804	organ development
GO:0007242	879	1	0.400739423	intracellular signaling cascade
GO:0009653	1051	1	0.461353842	morphogenesis
GO:0044237	5026	3	0.604999686	cellular metabolism
GO:0050874	1531	1	0.605017363	organismal physiological process
GO:0009987	8200	5	0.616738148	cellular process
GO:0008152	5262	3	0.652104245	metabolism
GO:0050875	7018	4	0.689656659	cellular physiological process
GO:0044267	2109	1	0.735512302	cellular protein metabolism
GO:0019538	2129	1	0.739311804	protein metabolism

GO:0007165	2168	1	0.746595223	signal transduction
GO:0043283	2240	1	0.759613486	biopolymer metabolism
GO:0044260	2298	1	0.769706456	cellular macromolecule metabolism
GO:0006139	2328	1	0.774792243	nucleobase, nucleoside, nucleotide and nucleic acid metabolism
GO:0043170	2405	1	0.787435547	macromolecule metabolism
GO:0007154	2730	1	0.834703382	cell communication
GO:0044238	4820	2	0.851814712	primary metabolism
GO:0007582	7846	4	0.868599032	physiological process
GO:0008150	9032	5	1	biological_process

9.3 GO: Molecular Function

GO ID	genes present in chip	genes present in tissues	p-value	GO term
GO:0004465	5	1	0.00314102993853504	lipoprotein lipase activity
GO:0005504	17	1	0.0106459724075498	fatty acid binding
GO:0008289	298	2	0.0134198993831583	lipid binding
GO:0005198	488	2	0.0341401285290386	structural molecule activity
GO:0005319	58	1	0.0359335918175531	lipid transporter activity
GO:0016298	59	1	0.0365435674000788	lipase activity
GO:0008201	60	1	0.0371532213333837	heparin binding
GO:0005539	77	1	0.0474682571983021	glycosaminoglycan binding
GO:0016789	80	1	0.0492789623312914	carboxylic ester hydrolase activity
GO:0030247	80	1	0.0492789623312914	polysaccharide binding
GO:0005200	86	1	0.0528917684062971	structural constituent of cytoskeleton
GO:0001871	88	1	0.054093491659439	pattern binding
GO:0005179	90	1	0.055293943987555	hormone activity
GO:0048503	92	1	0.056493126466161	GPI anchor binding
GO:0035091	132	1	0.0802117579012409	phosphoinositide binding
GO:0005543	169	1	0.101707057837018	phospholipid binding
GO:0030246	181	1	0.108587932919473	carbohydrate binding
GO:0016788	446	1	0.249726944531551	hydrolase activity, acting on ester bonds
GO:0005554	522	1	0.286576496485745	molecular function unknown
GO:0005102	528	1	0.289420233840501	receptor binding
GO:0005215	1030	1	0.496223964699483	transporter activity
GO:0005488	7228	5	0.551965990732236	binding
GO:0003677	1384	1	0.609605668818139	DNA binding
GO:0016787	1424	1	0.620955615187589	hydrolase activity
GO:0004871	1837	1	0.722941555262064	signal transducer activity
GO:0005515	3709	2	0.74888375019207	protein binding
GO:0003676	1990	1	0.754368858128866	nucleic acid binding
GO:0003824	3409	1	0.929584262429503	catalytic activity
GO:0003674	9541	6	1	molecular_function

9.4 GO: Cellular Component

GO ID	genes present in chip	genes present in tissues	p-value	GO term
GO:0005883	5	2	4.01830837505638e-06	neurofilament
GO:0030424	7	2	8.43323557834762e-06	axon
GO:0043005	17	2	5.44467339126728e-05	neuron projection
GO:0045111	65	2	0.000820437685408937	intermediate filament cytoskeleton
GO:0005882	65	2	0.000820437685408937	intermediate filament
GO:0042995	70	2	0.000951100920177924	cell projection
GO:0042627	4	1	0.00277633014395307	chylomicron
GO:0005625	183	2	0.00633218776135905	soluble fraction
GO:0000786	40	1	0.0274755424305253	nucleosome
GO:0005856	612	2	0.062114931245143	cytoskeleton
GO:0000785	100	1	0.0675075329137371	chromatin
GO:0000267	698	2	0.0786321910426457	cell fraction
GO:0005694	203	1	0.133026345132828	chromosome
GO:0005576	949	2	0.134160332057068	extracellular region
GO:0043228	977	2	0.140919275440818	non-membrane-bound organelle
GO:0043232	977	2	0.140919275440818	intracellular non-membrane-bound organelle
GO:0005615	385	1	0.239424041639618	extracellular space
GO:0005624	521	1	0.311623123781834	membrane fraction
GO:0005623	7802	6	0.543222062227152	cell
GO:0005737	2530	2	0.564470466664422	cytoplasm
GO:0005622	5419	4	0.601038187002145	intracellular
GO:0043234	1247	1	0.607751057292341	protein complex
GO:0016020	3514	2	0.777317993143544	membrane
GO:0005634	2704	1	0.895015631229013	nucleus
GO:0043226	4515	2	0.910604663985241	organelle
GO:0043229	4515	2	0.910604663985241	intracellular organelle
GO:0043227	3955	1	0.974661936274433	membrane-bound organelle
GO:0043231	3955	1	0.974661936274433	intracellular membrane-bound organelle
GO:0005575	8637	6	1	cellular_component

10. AtrioVentricularNode

10.1 AtrioVentricularNode selective genes (2 genes)

Symbol	Description	Chromosome	Chromosome Location	LocusLink	Cytoband	UniGene
PLA2G2A	phospholipase A2, group IIA (platelets, synovial fluid)	1	-20174517	5320	1p35	Hs.466804
CDH19	cadherin 19, type 2	18	-62322300	28513	18q22-q23	Hs.42771

10.2 GO: Biological Process

GO ID	genes present in chip	genes present in tissues	p-value	GO term
GO:0016042	67	1	0.014781926	lipid catabolism
GO:0006644	73	1	0.016100311	phospholipid metabolism
GO:0007156	105	1	0.023116788	homophilic cell adhesion
GO:0006643	106	1	0.023335649	membrane lipid metabolism
GO:0016337	172	1	0.03772622	cell-cell adhesion
GO:0044255	383	1	0.083015897	cellular lipid metabolism
GO:0006629	469	1	0.101162059	lipid metabolism
GO:0007155	518	1	0.111420053	cell adhesion
GO:0009056	756	1	0.160407185	catabolism
GO:0007154	2730	1	0.513180342	cell communication
GO:0044238	4820	1	0.782552794	primary metabolism
GO:0044237	5026	1	0.803304831	cellular metabolism
GO:0009987	8200	2	0.824242431	cellular process
GO:0008152	5262	1	0.825800174	metabolism
GO:0050875	7018	1	0.950296901	cellular physiological process
GO:0007582	7846	1	0.98277008	physiological process
GO:0008150	9032	2	1	biological_process

10.3 GO: Molecular Function

GO ID	genes present in chip	genes present in tissues	p-value	GO term
GO:0047498	5	1	0.00104788843558823	calcium-dependent phospholipase A2 activity
GO:0004623	16	1	0.00335130937714045	phospholipase A2 activity
GO:0005509	646	2	0.00457772776741754	calcium ion binding
GO:0004620	47	1	0.00982846402495063	phospholipase activity
GO:0016298	59	1	0.0123300806823553	lipase activity
GO:0016789	80	1	0.0167002962169009	carboxylic ester hydrolase activity
GO:0043169	2313	2	0.0587518020538965	cation binding
GO:0046872	2495	2	0.0683635691664596	metal ion binding
GO:0043167	2556	2	0.0717479477844377	ion binding
GO:0016788	446	1	0.0913107658286848	hydrolase activity, acting on ester bonds
GO:0016787	1424	1	0.276238772663141	hydrolase activity
GO:0005488	7228	2	0.573896965034716	binding
GO:0003824	3409	1	0.586960875242828	catalytic activity
GO:0005515	3709	1	0.626390177051177	protein binding
GO:0003674	9541	2	1	molecular_function

10.4 GO: Cellular Component

GO ID	genes present in chip	genes present in tissues	p-value	GO term
GO:0016020	3514	2	0.165502421988233	membrane
GO:0005576	949	1	0.207690793345068	extracellular region
GO:0016021	2674	1	0.52337016068239	integral to membrane
GO:0031224	2681	1	0.524488634617708	intrinsic to membrane
GO:0005623	7802	2	0.815982172845234	cell
GO:0005575	8637	2	1	cellular_component

11. DRG

11.1 DRG selective genes (11 genes)

Symbol	Description	Chromosome	Chromosome Location	LocusLink	Cytoband	UniGene
NEFH	neurofilament, heavy polypeptide 200kDa	22	28206218	4744	22q12.2	Hs.198760
CDH19	cadherin 19, type 2	18	-62322300	28513	18q22-q23	Hs.42771
MPZ	myelin protein zero (Charcot-Marie-Tooth neuropathy 1B)	1	-159541150	4359	1q22	Hs.591486
NEF3	neurofilament 3 (150kDa medium)	8	24827211	4741	8p21	Hs.458657 Hs.615287
S100B	S100 calcium binding protein, beta (neural)	21	-46842958	6285	21q22.3	Hs.422181
PMP22	peripheral myelin protein 22	17	-15073821	5376	17p12-p11.2	Hs.372031
PMP2	peripheral myelin protein 2	8	-82515120	5375	8q21.3-q22.1	Hs.571512
LPL	lipoprotein lipase	8	19841057	4023	8p22	Hs.180878
STMN2	stathmin-like 2	8	80685934	11075	8q21.13	Hs.521651
TSPAN8	tetraspanin 8	12	-69805143	7103	12q14.1-q21.1	Hs.170563
PLP1	proteolipid protein 1 (Pelizaeus-Merzbacher disease, spastic paraplegia 2, uncomplicated)	X	102918409	5354	Xq22	Hs.1787

11.2 GO: Biological Process

GO ID	genes present in chip	genes present in tissues	p-value	GO term
GO:0007638	5	2	8.81E-06	mechanosensory behavior
GO:0007422	12	2	5.80E-05	peripheral nervous system development
GO:0007399	385	4	0.000345309	neurogenesis
GO:0048151	1	1	0.000996457	hyperphosphorylation
GO:0048143	1	1	0.000996457	astrocyte activation
GO:0045917	1	1	0.000996457	positive regulation of complement activation
GO:0030449	1	1	0.000996457	regulation of complement activation
GO:0007610	269	3	0.001920699	behavior
GO:0048169	2	1	0.001992031	regulation of long-term neuronal synaptic plasticity
GO:0048168	4	1	0.003980535	regulation of neuronal synaptic plasticity
GO:0007156	105	2	0.004569418	homophilic cell adhesion
GO:0009887	807	4	0.005527717	organogenesis
GO:0048167	7	1	0.006956687	regulation of synaptic plasticity
GO:0048513	872	4	0.007312201	organ development
GO:0008366	9	1	0.008936394	nerve ensheathment
GO:0042551	9	1	0.008936394	nerve maturation
GO:0007275	1543	5	0.009930514	development
GO:0016337	172	2	0.011887383	cell-cell adhesion
GO:0030182	13	1	0.012885286	neuron differentiation
GO:0009653	1051	4	0.014187708	morphogenesis
GO:0007268	192	2	0.014668738	synaptic transmission
GO:0050767	15	1	0.014854483	regulation of neurogenesis
GO:0019226	198	2	0.015553863	transmission of nerve impulse
GO:0007416	17	1	0.016820187	synaptogenesis
GO:0007611	17	1	0.016820187	learning and/or memory
GO:0050808	18	1	0.017801731	synapse organization and biogenesis
GO:0048469	21	1	0.020741138	cell maturation
GO:0006956	30	1	0.029512503	complement activation
GO:0042035	33	1	0.032420732	regulation of cytokine biosynthesis
GO:0006112	34	1	0.033388419	energy reserve metabolism
GO:0043062	35	1	0.034355245	extracellular structure organization and biogenesis
GO:0030198	35	1	0.034355245	extracellular matrix organization and biogenesis
GO:0042089	36	1	0.035321212	cytokine biosynthesis
GO:0042107	37	1	0.03628632	cytokine metabolism
GO:0001817	39	1	0.038213961	regulation of cytokine production
GO:0048468	40	1	0.039176495	cell development
GO:0007409	40	1	0.039176495	axonogenesis
GO:0001816	42	1	0.041098996	cytokine production
GO:0031175	46	1	0.044933744	neurite morphogenesis

GO:0006334	48	1	0.046846	nucleosome assembly
GO:0050874	1531	4	0.050643485	organismal physiological process
GO:0031497	56	1	0.054461039	chromatin assembly
GO:0006412	407	2	0.059113774	protein biosynthesis
GO:0030154	409	2	0.059634841	cell differentiation
GO:0016042	67	1	0.064843407	lipid catabolism
GO:0006874	68	1	0.065782215	calcium ion homeostasis
GO:0050776	77	1	0.074193858	regulation of immune response
GO:0009059	464	2	0.074609063	macromolecule biosynthesis
GO:0007267	465	2	0.074892374	cell-cell signaling
GO:0008283	473	2	0.07717242	cell proliferation
GO:0050877	475	2	0.077746168	neurophysiological process
GO:0050954	86	1	0.082538111	sensory perception of mechanical stimulus
GO:0050982	86	1	0.082538111	detection of mechanical stimulus
GO:0007605	86	1	0.082538111	perception of sound
GO:0030005	87	1	0.083461111	di-, tri-valent inorganic cation homeostasis
GO:0006333	91	1	0.087144862	chromatin assembly or disassembly
GO:0006486	92	1	0.088063741	protein amino acid glycosylation
GO:0043413	93	1	0.088981797	biopolymer glycosylation
GO:0006875	93	1	0.088981797	metal ion homeostasis
GO:0007155	518	2	0.090429207	cell adhesion
GO:0009101	96	1	0.09173104	glycoprotein biosynthesis
GO:0045321	97	1	0.092645814	immune cell activation
GO:0030003	98	1	0.093559769	cation homeostasis
GO:0001775	98	1	0.093559769	cell activation
GO:0007417	101	1	0.096296726	central nervous system development
GO:0008015	103	1	0.098117281	circulation
GO:0006873	103	1	0.098117281	cell ion homeostasis
GO:0006417	107	1	0.101748612	regulation of protein biosynthesis
GO:0009582	109	1	0.1035594	detection of abiotic stimulus
GO:0031326	111	1	0.105366942	regulation of cellular biosynthesis
GO:0050801	111	1	0.105366942	ion homeostasis
GO:0009100	111	1	0.105366942	glycoprotein metabolism
GO:0009889	116	1	0.109871631	regulation of biosynthesis
GO:0019725	122	1	0.115250636	cell homeostasis
GO:0006631	124	1	0.117037206	fatty acid metabolism
GO:0015980	132	1	0.124151461	energy derivation by oxidation of organic compounds
GO:0051239	133	1	0.125037151	regulation of organismal physiological process
GO:0008285	139	1	0.130334589	negative regulation of cell proliferation
GO:0006917	140	1	0.131214719	induction of apoptosis
GO:0012502	140	1	0.131214719	induction of programmed cell death
GO:0006959	141	1	0.132094056	humoral immune response
GO:0043065	155	1	0.144321984	positive regulation of apoptosis

GO:0043068	156	1	0.145189518	positive regulation of programmed cell death
GO:0042592	157	1	0.146056271	homeostasis
GO:0050793	168	1	0.155539117	regulation of development
GO:0051246	174	1	0.160672024	regulation of protein metabolism
GO:0006325	186	1	0.170854716	establishment and/or maintenance of chromatin architecture
GO:0044249	763	2	0.172799801	cellular biosynthesis
GO:0006323	193	1	0.176743774	DNA packaging
GO:0006461	194	1	0.177582026	protein complex assembly
GO:0007001	221	1	0.199929965	chromosome organization and biogenesis (sensu Eukaryota)
GO:0009058	859	2	0.208359981	biosynthesis
GO:0051276	236	1	0.212110791	chromosome organization and biogenesis
GO:0042127	265	1	0.235193902	regulation of cell proliferation
GO:0009605	963	2	0.248082054	response to external stimulus
GO:0007600	287	1	0.252302174	sensory perception
GO:0042981	306	1	0.266802837	regulation of apoptosis
GO:0043067	308	1	0.268314583	regulation of programmed cell death
GO:0009581	313	1	0.272081834	detection of external stimulus
GO:0007154	2730	4	0.275448926	cell communication
GO:0009628	318	1	0.275831834	response to abiotic stimulus
GO:0019752	359	1	0.30593993	carboxylic acid metabolism
GO:0006082	361	1	0.307379723	organic acid metabolism
GO:0044255	383	1	0.323043114	cellular lipid metabolism
GO:0051242	411	1	0.342522542	positive regulation of cellular physiological process
GO:0044267	2109	3	0.353150041	cellular protein metabolism
GO:0043119	428	1	0.354104749	positive regulation of physiological process
GO:0019538	2129	3	0.359327684	protein metabolism
GO:0006091	446	1	0.366170248	generation of precursor metabolites and energy
GO:0009613	458	1	0.374102198	response to pest, pathogen or parasite
GO:0043207	463	1	0.377381051	response to external biotic stimulus
GO:0006629	469	1	0.381295513	lipid metabolism
GO:0006915	471	1	0.382595464	apoptosis
GO:0012501	473	1	0.383892986	programmed cell death
GO:0048522	482	1	0.389701891	positive regulation of cellular process
GO:0006259	489	1	0.394186217	DNA metabolism
GO:0008219	498	1	0.399908732	cell death
GO:0016265	501	1	0.40180553	death
GO:0044260	2298	3	0.411740633	cellular macromolecule metabolism
GO:0016310	522	1	0.414934578	phosphorylation
GO:0009987	8200	9	0.418883795	cellular process
GO:0048518	565	1	0.441021515	positive regulation of biological process
GO:0043170	2405	3	0.444881351	macromolecule metabolism
GO:0051243	583	1	0.451630983	negative regulation of cellular physiological process
GO:0043118	602	1	0.462635322	negative regulation of physiological process

GO:0048523	640	1	0.48405689	negative regulation of cellular process
GO:0050896	1578	2	0.484086166	response to stimulus
GO:0006793	641	1	0.484610213	phosphorus metabolism
GO:0006796	641	1	0.484610213	phosphate metabolism
GO:0006955	684	1	0.507909843	immune response
GO:0048519	688	1	0.510028889	negative regulation of biological process
GO:0006996	693	1	0.512666287	organelle organization and biogenesis
GO:0006952	751	1	0.5423509	defense response
GO:0009056	756	1	0.544833021	catabolism
GO:0016043	842	1	0.585693569	cell organization and biogenesis
GO:0009607	857	1	0.592476267	response to biotic stimulus
GO:0007242	879	1	0.602245712	intracellular signaling cascade
GO:0006950	881	1	0.603123434	response to stress
GO:0006464	1087	1	0.684824273	protein modification
GO:0043283	2240	2	0.694979761	biopolymer metabolism
GO:0043412	1160	1	0.70996183	biopolymer modification
GO:0051244	2319	2	0.715731635	regulation of cellular physiological process
GO:0050791	2407	2	0.73769506	regulation of physiological process
GO:0050794	2488	2	0.756845673	regulation of cellular process
GO:0050789	2689	2	0.800046884	regulation of biological process
GO:0044238	4820	4	0.807846296	primary metabolism
GO:0031323	1566	1	0.819958271	regulation of cellular metabolism
GO:0019222	1629	1	0.833185724	regulation of metabolism
GO:0044237	5026	4	0.844119543	cellular metabolism
GO:0006810	1802	1	0.865178991	transport
GO:0051234	1841	1	0.871588252	establishment of localization
GO:0051179	1857	1	0.87413834	localization
GO:0008152	5262	4	0.880080105	metabolism
GO:0050875	7018	6	0.881071679	cellular physiological process
GO:0007582	7846	7	0.896737553	physiological process
GO:0007165	2168	1	0.9155513	signal transduction
GO:0006139	2328	1	0.931711254	nucleobase, nucleoside, nucleotide and nucleic acid metabolism
GO:0008150	9032	9	1	biological_process

11.3 GO: Molecular Function

GO ID	genes present in chip	genes present in tissues	p-value	GO term
GO:0048154	1	1	0.00104810816476208	S100 beta binding
GO:0005198	488	4	0.00110894713653292	structural molecule activity
GO:0048155	2	1	0.00209522754823754	S100 alpha binding
GO:0048156	2	1	0.00209522754823754	tau protein binding
GO:0004465	5	1	0.00523066130043148	lipoprotein lipase activity
GO:0019210	29	1	0.0299967072587638	kinase inhibitor activity
GO:0008289	298	2	0.0370748434213998	lipid binding
GO:0005319	58	1	0.0591811490756199	lipid transporter activity
GO:0019207	58	1	0.0591811490756199	kinase regulator activity
GO:0016298	59	1	0.0601732600646784	lipase activity
GO:0008201	60	1	0.0611644293748828	heparin binding
GO:0042803	64	1	0.0651197057099888	protein homodimerization activity
GO:0005539	77	1	0.0778709984636039	glycosaminoglycan binding
GO:0016789	80	1	0.080791282393479	carboxylic ester hydrolase activity
GO:0030247	80	1	0.080791282393479	polysaccharide binding
GO:0005200	86	1	0.0866068932357916	structural constituent of cytoskeleton
GO:0001871	88	1	0.0885380584218165	pattern binding
GO:0048503	92	1	0.0923893685375536	GPI anchor binding
GO:0005554	522	2	0.100489193629751	molecular function unknown
GO:0035091	132	1	0.130104726510041	phosphoinositide binding
GO:0005509	646	2	0.143631691357039	calcium ion binding
GO:0042802	158	1	0.153857148620890	protein self binding
GO:0005543	169	1	0.163729302941210	phospholipid binding
GO:0046983	170	1	0.164621610556509	protein dimerization activity
GO:0030246	181	1	0.174380594281133	carbohydrate binding
GO:0004857	187	1	0.179660317311939	enzyme inhibitor activity
GO:0008092	279	1	0.256900337530535	cytoskeletal protein binding
GO:0016788	446	1	0.380576667212693	hydrolase activity, acting on ester bonds
GO:0030234	509	1	0.422190315001835	enzyme regulator activity
GO:0005215	1030	1	0.68112616737154	transporter activity
GO:0043169	2313	2	0.738631143635427	cation binding
GO:0008270	1287	1	0.765368632739372	zinc ion binding
GO:0046872	2495	2	0.78104871413507	metal ion binding
GO:0003677	1384	1	0.791542333890742	DNA binding
GO:0043167	2556	2	0.7940637975194	ion binding
GO:0016787	1424	1	0.801547354940713	hydrolase activity
GO:0005515	3709	3	0.814010451149342	protein binding
GO:0046914	1596	1	0.839827378878336	transition metal ion binding
GO:0004871	1837	1	0.882311237343687	signal transducer activity
GO:0003676	1990	1	0.903713474580475	nucleic acid binding
GO:0005488	7228	6	0.930510022212393	binding
GO:0003824	3409	1	0.98800658703172	catalytic activity
GO:0003674	9541	10	1	molecular_function

11.4 GO: Cellular Component

GO ID	genes present in chip	genes present in tissues	p-value	GO term
GO:0005883	5	2	1.47167364742646e-05	neurofilament
GO:0030424	7	2	3.08622128663789e-05	axon
GO:0043005	17	2	0.000198485119699253	neuron projection
GO:0045111	65	2	0.00293604636888435	intermediate filament cytoskeleton
GO:0005882	65	2	0.00293604636888435	intermediate filament
GO:0042995	70	2	0.00339709315191160	cell projection
GO:0042627	4	1	0.00508551914094613	chylomicron
GO:0000786	40	1	0.0498083700298646	nucleosome
GO:0016020	3514	7	0.107853812906940	membrane
GO:0000785	100	1	0.120302093239105	chromatin
GO:0005764	107	1	0.128208745955325	lysosome
GO:0000323	107	1	0.128208745955325	lytic vacuole
GO:0005624	521	2	0.139347239607834	membrane fraction
GO:0005887	1057	3	0.142686340706914	integral to plasma membrane
GO:0005773	120	1	0.142721352148169	vacuole
GO:0031226	1062	3	0.144200263570660	intrinsic to plasma membrane
GO:0005856	612	2	0.180606705718653	cytoskeleton
GO:0005625	183	1	0.209989890650529	soluble fraction
GO:0000267	698	2	0.22144488422931	cell fraction
GO:0005694	203	1	0.230318889657675	chromosome
GO:0016021	2674	5	0.231784608441508	integral to membrane
GO:0031224	2681	5	0.233651205622557	intrinsic to membrane
GO:0005886	1472	3	0.285254705509256	plasma membrane
GO:0005623	7802	11	0.326570578574414	cell
GO:0005576	949	2	0.344682887294264	extracellular region
GO:0043228	977	2	0.358411038834111	non-membrane-bound organelle
GO:0043232	977	2	0.358411038834111	intracellular non-membrane-bound organelle
GO:0005615	385	1	0.39461708371303	extracellular space
GO:0008372	523	1	0.49717771940599	cellular component unknown
GO:0005737	2530	3	0.66893080704091	cytoplasm
GO:0043234	1247	1	0.820266252195815	protein complex
GO:0005634	2704	2	0.903508538103346	nucleus
GO:0043226	4515	4	0.913580310959973	organelle
GO:0043229	4515	4	0.913580310959973	intracellular organelle
GO:0005622	5419	5	0.930653725622934	intracellular
GO:0043227	3955	3	0.941273284461302	membrane-bound organelle
GO:0043231	3955	3	0.941273284461302	intracellular membrane-bound organelle
GO:0005575	8637	11	1	cellular_component

12. Placenta

12.1 Placenta selective genes (115 genes)

Symbol	Description	Chromosome	Chromosome Location	LocusLink	Cytoband	UniGene
HSD3B1	hydroxy-delta-5-steroid dehydrogenase, 3 beta- and steroid delta-isomerase 1	1	119851373	3283	1p13.1	Hs.364941
IGFBP1	insulin-like growth factor binding protein 1	7	45894483	3484	7p13-p12	Hs.401316
PSG3	pregnancy specific beta-1-glycoprotein 3	19	-47917634	5671	19q13.2	Hs.466843
GH2	growth hormone 2	17	-177933915	2689	17q24.2	Hs.406754
CSH1	chorionic somatomammotropin hormone 1 (placental lactogen)	17	-59326005	1442	17q24.2	Hs.406754
TFPI2	tissue factor pathway inhibitor 2	7	-93353680	7980	7q22	Hs.438231
HSD17B1	hydroxysteroid (17-beta) dehydrogenase 1	17	37957509	3292	17q11-q21	Hs.50727
ABP1	amiloride binding protein 1 (amine oxidase (copper-containing))	7	150180505	26	7q34-q36	Hs.438823
PAPPA	pregnancy-associated plasma protein A, pappalysin 1	9	117955891	5069	9q33.2	Hs.494928
GH1	growth hormone 1	17	-59348295	2688	17q24.2	Hs.406754
PRG2	proteoglycan 2, bone marrow (natural killer cell activator, eosinophil granule major basic protein)	11	-56911410	5553	11q12	Hs.512633
GDF15	growth differentiation factor 15	19	18357984	9518	19p13.1-13.2	Hs.515258
CSH2	chorionic somatomammotropin hormone 2	17	-59303104	1443	17q24.2	Hs.406754
PHLDA2	pleckstrin homology-like domain, family A, member 2	11	-2906078	7262	11p15.5	Hs.154036
CYP19A1	cytochrome P450, family 19, subfamily A, polypeptide 1	15	-49288963	1588	15q21.1	Hs.511367
CAPN6	calpain 6	X	-110374987	827	Xq23	Hs.496593
PSG6	pregnancy specific beta-1-glycoprotein 6	19	-96197686	5675	19q13.2	Hs.466843
SERPINB2	serpin peptidase inhibitor, clade B (ovalbumin), member 2	18	59705921	5055	18q21.3	Hs.514913
DLK1	delta-like 1 homolog (Drosophila)	14	100262981, 100263005	8788	14q32	Hs.533717
EGFL6	EGF-like-domain, multiple 6	X	13497661	25975	Xp22	Hs.12844
ADAM12	ADAM metallopeptidase domain 12 (meltrin alpha)	10	-255413317	8038	10q26.3	Hs.593858
ALPP	alkaline phosphatase, placental (Regan isozyme)	2	232951591	250	2q37	Hs.284255
KISS1	KiSS-1 metastasis-suppressor	1	-202426092	3814	1q32	Hs.95008
CRH	corticotropin releasing hormone	8	-67251172	1392	8q13	Hs.75294
CGA	glycoprotein hormones, alpha polypeptide	6	-87851940	1081	6q12-q21	Hs.119689
CSHL1	chorionic somatomammotropin hormone-like 1	17	-59340696	1444	17q24.2	Hs.406754
EBI3	Epstein-Barr virus induced gene 3	19	4180539	10148	19p13.3	Hs.501452
KRT19	keratin 19	17	-36933395	3880	17q21.2	Hs.2785
PSG1	pregnancy specific beta-1-glycoprotein 1	19	-48063197	5669	19q13.2	Hs.466843
NA	NA	NA	NA	653991	NA	NA
MAFF	v-maf musculoaponeurotic fibrosarcoma oncogene homolog F (avian)	22	36927943, 36939487	23764	22q13.1	Hs.517617
CYP11A1	cytochrome P450, family 11, subfamily A, polypeptide 1	15	-72417156	1583	15q23-q24	Hs.303980
GPC3	glypican 3	X	-132497443	2719	Xq26.1	Hs.567276

PSG2	pregnancy specific beta-1-glycoprotein 2	19	-48260201	5670	19q13.1-q13.2	Hs.502092
S100P	S100 calcium binding protein P	4	6746466	6286	4p16	Hs.2962
CLIC3	chloride intracellular channel 3	9	-139008880	9022	9q34.3	Hs.64746
LOC645569	NA	15	NA	645569	15q21.2	Hs.511367
CRIM1	cysteine rich transmembrane BMP regulator 1 (chordin-like)	2	36436900	51232	2p21	Hs.332847
FBN2	fibrillin 2 (congenital contractural arachnodactyly)	5	-127621500	2201	5q23-q31	Hs.519294
COL15A1	collagen, type XV, alpha 1	9	100746010	1306	9q21-q22	Hs.409034
KRT7	keratin 7	12	50913220	3855	12q12-q13	Hs.411501
SVEP1	sushi, von Willebrand factor type A, EGF and pentraxin domain containing 1	9	NA	79987	9q32	Hs.522334 Hs.606771
PSG11	pregnancy specific beta-1-glycoprotein 11	19	-48203648	5680	19q13.2	Hs.502092
PLAC1	placenta-specific 1	X	-133527538	10761	Xq26	Hs.496811
MEST	mesoderm specific transcript homolog (mouse)	7	129919173, 129913281, 129918408	4232	7q32	Hs.270978
NA	NA	NA	NA	492304	NA	NA
BPGM	2,3-bisphosphoglycerate mutase	7	133982094, 133982110	669	7q31-q34	Hs.198365
CDH5	cadherin 5, type 2, VE-cadherin (vascular epithelium)	16	64958063	1003	16q22.1	Hs.76206
HSD11B2	hydroxysteroid (11-beta) dehydrogenase 2	16	66022536	3291	16q22	Hs.1376
HSD17B2	hydroxysteroid (17-beta) dehydrogenase 2	16	80626363	3294	16q24.1-q24.2	Hs.162795
SERPINE1	serpin peptidase inhibitor, clade E (nexin, plasminogen activator inhibitor type 1), member 1	7	100557171	5054	7q21.3-q22	Hs.414795
LOC646541	NA	14	NA	646541	14q24.2	NA
COL3A1	collagen, type III, alpha 1 (Ehlers-Danlos syndrome type IV, autosomal dominant)	2	189547343	1281	2q31	Hs.443625
IGF2BP3	insulin-like growth factor 2 mRNA binding protein 3	7	-23316353	10643	7p11	Hs.432616
PGF	placental growth factor, vascular endothelial growth factor-related protein	14	-74478292	5228	14q24-q31	Hs.252820
DLG5	discs, large homolog 5 (Drosophila)	10	-79220556	9231	10q23	Hs.500245
GCM1	glial cells missing homolog 1 (Drosophila)	6	-53099720	8521	6p21-p12	Hs.28346
SDC1	syndecan 1	2	-20264040	6382	2p24.1	Hs.224607
PSG9	pregnancy specific beta-1-glycoprotein 9	19	-48449277	5678	19q13.2	Hs.502092
LGALS14	lectin, galactoside-binding, soluble, 14	19	44886785	56891	19q13.2	Hs.24236
ADM	adrenomedullin	11	10283217	133	11p15.4	Hs.441047
PAGE4	P antigen family, member 4 (prostate associated)	X	49480643	9506	Xp11.23	Hs.441038
CSF3R	colony stimulating factor 3 receptor (granulocyte)	1	-36704231	1441	1p35-p34.3	Hs.524517
KRT18	keratin 18	12	51629109, 51628921	3875	12q13	Hs.406013
FDX1	ferredoxin 1	11	109805803	2230	11q22	Hs.744
MS4A4A	membrane-spanning 4-domains, subfamily A, member 4	11	59806661, 59804714	51338	11q12	Hs.325960
KRT6C	keratin 6C	12	-51167243	140446	12q12-q13	Hs.433845
KRT6A	keratin 6A	12	-51167243	3853	12q12-q13	Hs.433845
LUM	lumican	12	-90021363	4060	12q21.3-q22	Hs.406475
MAN1C1	mannosidase, alpha, class 1C, member 1	1	25816545	57134	1p35	Hs.197043
GPR126	G protein-coupled receptor 126	6	142664758	57211	6q24.1	Hs.318894
PSG4	pregnancy specific beta-1-glycoprotein 4	19	-48388695	5672	19q13.2	Hs.502092
TCF21	transcription factor 21	6	134251969	6943	6pter-qter	Hs.78061
P11	NA	12	-46389794	8909	12q13.1	Hs.997

TBX3	T-box 3 (ulnar mammary syndrome)	12	-113592441	6926	12q24.1	Hs.129895
COL4A2	collagen, type IV, alpha 2	13	109757631	1284	13q34	Hs.508716
RHOBTB1	Rho-related BTB domain containing 1	10	-62299205	9886	10q21.2	Hs.148670
SPP1	secreted phosphoprotein 1 (osteopontin, bone sialoprotein I, early T-lymphocyte activation 1)	4	89115825	6696	4q21-q25	Hs.313
DKK1	dickkopf homolog 1 (Xenopus laevis)	10	53744046	22943	10q11.2	Hs.40499
FABP4	fatty acid binding protein 4, adipocyte	8	-82553489	2167	8q21	Hs.391561
TGFBR3	transforming growth factor, beta receptor III (betaglycan, 300kDa)	1	-91920574	7049	1p33-p32	Hs.482390
TUSC3	tumor suppressor candidate 3	8	15442100	7991	8p22	Hs.591845
IGFBP3	insulin-like growth factor binding protein 3	7	-45918374	3486	7p13-p12	Hs.450230
SEMA3B	sema domain, immunoglobulin domain (Ig), short basic domain, secreted, (semaphorin) 3B	3	50280043	7869	3p21.3	Hs.82222
CHSY1	carbohydrate (chondroitin) synthase 1	15	-99533455	22856	15q26.3	Hs.110488
ANXA1	annexin A1	9	74956600	301	9q12-q21.2|9q12-q21.2	Hs.494173
VGLL1	vestigial like 1 (Drosophila)	X	135441976	51442	Xq26.3	Hs.496843
DSP	desmoplakin	6	7486868	1832	6p24	Hs.519873
GNG11	guanine nucleotide binding protein (G protein), gamma 11	7	93388951	2791	7q21	Hs.83381
CDKN1C	cyclin-dependent kinase inhibitor 1C (p57, Kip2)	11	-2861440	1028	11p15.5	Hs.106070
CAV1	caveolin 1, caveolae protein, 22kDa	7	115952074	857	7q31.1	Hs.74034
FRZB	frizzled-related protein	2	-183406981	2487	2qter	Hs.128453
DAB2	disabled homolog 2, mitogen-responsive phosphoprotein (Drosophila)	5	-39408502	1601	5p13	Hs.481980
TIMP3	TIMP metallopeptidase inhibitor 3 (Sorsby fundus dystrophy, pseudoinflammatory)	22	31526801	7078	22q12.1-q13.2|22q12.3	Hs.297324
FAM46A	family with sequence similarity 46, member A	6	-82516078	55603	6q14	Hs.10784
PSG5	pregnancy specific beta-1-glycoprotein 5	19	-48363735	5673	19q13.2	Hs.502092
COL4A1	collagen, type IV, alpha 1	13	-109599311	1282	13q34	Hs.17441
GPNMB	glycoprotein (transmembrane) nmb	7	23252840	10457	7p15	Hs.190495
LIPG	lipase, endothelial	18	45342424	9388	18q21.1	Hs.465102
RASA1	RAS p21 protein activator (GTPase activating protein) 1	5	86599906, 86600521	5921	5q13.3	Hs.553501
PEG10	paternally expressed 10	7	94123572	23089	7q21	Hs.147492
NA	NA	NA	NA	645959	NA	NA
GATA3	GATA binding protein 3	10	8136672	2625	10p15	Hs.524134
KRT23	keratin 23 (histone deacetylase inducible)	17	-36332477	25984	17q21.2	Hs.9029
ADFP	adipose differentiation-related protein	9	-19105759	123	9p22.1	Hs.3416
COL6A3	collagen, type VI, alpha 3	2	-237897400	1293	2q37	Hs.233240
RHOBTB3	Rho-related BTB domain containing 3	5	95092798	22836	5q15	Hs.445030
COL1A2	collagen, type I, alpha 2	7	93861808	1278	7q22.1	Hs.489142
FBN1	fibrillin 1	15	-46487796	2200	15q21.1	Hs.591133
PERP	PERP, TP53 apoptosis effector	6	-138453618	64065	6q24	Hs.520421
COL1A1	collagen, type I, alpha 1	17	-45616457	1277	17q21.33	Hs.172928
INSL4	insulin-like 4 (placenta)	9	5221418	3641	9p24	Hs.418506
PLAGL1	pleiomorphic adenoma gene-like 1	6	-144303131	5325	6q24-q25	Hs.444975
VAMP8	vesicle-associated membrane protein 8 (endobrevin)	2	85658227	8673	2p12-p11.2	Hs.534373

LOXL1	lysyl oxidase-like 1	15	72005841	4016	15q24-q25|15q22	Hs.65436

12.2 GO: Biological Process

GO ID	genes present in chip	genes present in tissues	p-value	GO term
GO:0007565	45	14	1.12E-17	pregnancy
GO:0048609	61	15	3.81E-17	reproductive organismal physiological process
GO:0050876	62	15	4.98E-17	reproductive physiological process
GO:0000003	208	15	4.15E-09	reproduction
GO:0042446	25	6	1.85E-07	hormone biosynthesis
GO:0006817	57	7	2.11E-06	phosphate transport
GO:0006694	60	7	3.00E-06	steroid biosynthesis
GO:0009653	1051	27	8.02E-06	morphogenesis
GO:0042445	46	6	8.19E-06	hormone metabolism
GO:0015698	106	8	1.56E-05	inorganic anion transport
GO:0006703	6	3	2.27E-05	estrogen biosynthesis
GO:0007275	1543	33	2.92E-05	development
GO:0006820	128	8	6.10E-05	anion transport
GO:0008210	8	3	6.27E-05	estrogen metabolism
GO:0008202	132	8	7.59E-05	steroid metabolism
GO:0006700	11	3	0.000180508	C21-steroid hormone biosynthesis
GO:0009887	807	20	0.000259158	organogenesis
GO:0008207	14	3	0.000389089	C21-steroid hormone metabolism
GO:0045445	15	3	0.000482622	myoblast differentiation
GO:0006702	4	2	0.000661576	androgen biosynthesis
GO:0048513	872	20	0.000709649	organ development
GO:0050874	1531	29	0.000900404	organismal physiological process
GO:0042692	19	3	0.000996616	muscle cell differentiation
GO:0001501	113	6	0.001247823	skeletal development
GO:0048627	7	2	0.002267755	myoblast development
GO:0048628	7	2	0.002267755	myoblast maturation
GO:0008610	175	7	0.002546266	lipid biosynthesis
GO:0007567	8	2	0.003002773	parturition
GO:0008209	9	2	0.003834044	androgen metabolism
GO:0030178	9	2	0.003834044	negative regulation of Wnt receptor signaling pathway
GO:0030154	409	11	0.003951889	cell differentiation
GO:0009888	158	6	0.006623988	histogenesis
GO:0042102	12	2	0.006884671	positive regulation of T cell proliferation
GO:0007519	39	3	0.008063249	myogenesis
GO:0046640	1	1	0.010628875	regulation of alpha-beta T cell proliferation
GO:0046641	1	1	0.010628875	positive regulation of alpha-beta T cell proliferation
GO:0046633	1	1	0.010628875	alpha-beta T cell proliferation
GO:0046634	1	1	0.010628875	regulation of alpha-beta T cell activation
GO:0046635	1	1	0.010628875	positive regulation of alpha-beta T cell activation
GO:0001952	1	1	0.010628875	regulation of cell-matrix adhesion
GO:0001953	1	1	0.010628875	negative regulation of cell-matrix adhesion
GO:0045214	1	1	0.010628875	sarcomere organization
GO:0006701	1	1	0.010628875	progesterone biosynthesis

GO:0018277	1	1	0.010628875	protein amino acid deamination
GO:0042129	15	2	0.010728406	regulation of T cell proliferation
GO:0030111	15	2	0.010728406	regulation of Wnt receptor signaling pathway
GO:0018149	15	2	0.010728406	peptide cross-linking
GO:0042098	17	2	0.01370571	T cell proliferation
GO:0050671	18	2	0.015313205	positive regulation of lymphocyte proliferation
GO:0030216	18	2	0.015313205	keratinocyte differentiation
GO:0009968	50	3	0.015904149	negative regulation of signal transduction
GO:0007517	94	4	0.017657721	muscle development
GO:0048469	21	2	0.02058946	cell maturation
GO:0050870	21	2	0.02058946	positive regulation of T cell activation
GO:0046631	2	1	0.021145942	alpha-beta T cell activation
GO:0045663	2	1	0.021145942	positive regulation of myoblast differentiation
GO:0042448	2	1	0.021145942	progesterone metabolism
GO:0006704	2	1	0.021145942	glucocorticoid biosynthesis
GO:0050670	22	2	0.022493633	regulation of lymphocyte proliferation
GO:0007267	465	10	0.025473551	cell-cell signaling
GO:0042088	24	2	0.026509899	T-helper 1 type immune response
GO:0006629	469	10	0.02682724	lipid metabolism
GO:0009611	341	8	0.028315273	response to wounding
GO:0042087	25	2	0.028618685	cell-mediated immune response
GO:0007498	26	2	0.030792412	mesoderm development
GO:0006916	112	4	0.03116668	anti-apoptosis
GO:0030502	3	1	0.031552364	negative regulation of bone mineralization
GO:0015918	3	1	0.031552364	sterol transport
GO:0007520	3	1	0.031552364	myoblast fusion
GO:0030301	3	1	0.031552364	cholesterol transport
GO:0030279	3	1	0.031552364	negative regulation of ossification
GO:0046851	3	1	0.031552364	negative regulation of bone remodeling
GO:0016066	27	2	0.033029493	cellular defense response (sensu Vertebrata)
GO:0046651	28	2	0.035328364	lymphocyte proliferation
GO:0051251	28	2	0.035328364	positive regulation of lymphocyte activation
GO:0001568	68	3	0.03549485	blood vessel development
GO:0048514	68	3	0.03549485	blood vessel morphogenesis
GO:0001944	69	3	0.036830281	vasculature development
GO:0050867	29	2	0.037687491	positive regulation of cell activation
GO:0007179	29	2	0.037687491	transforming growth factor beta receptor signaling pathway
GO:0008211	4	1	0.041849293	glucocorticoid metabolism
GO:0045661	4	1	0.041849293	regulation of myoblast differentiation
GO:0051252	4	1	0.041849293	regulation of RNA metabolism
GO:0030199	4	1	0.041849293	collagen fibril organization
GO:0030239	4	1	0.041849293	myofibril assembly
GO:0050863	31	2	0.042580487	regulation of T cell activation
GO:0042981	306	7	0.043978252	regulation of apoptosis
GO:0043067	308	7	0.045280435	regulation of programmed cell death
GO:0043066	128	4	0.047173014	negative regulation of apoptosis
GO:0043069	129	4	0.048300389	negative regulation of programmed cell death

GO:0007596	78	3	0.049987223	blood coagulation
GO:0044255	383	8	0.050553835	cellular lipid metabolism
GO:0007398	79	3	0.05157322	ectoderm development
GO:0050817	79	3	0.05157322	coagulation
GO:0050926	5	1	0.052037869	regulation of positive chemotaxis
GO:0050927	5	1	0.052037869	positive regulation of positive chemotaxis
GO:0050918	5	1	0.052037869	positive chemotaxis
GO:0050930	5	1	0.052037869	induction of positive chemotaxis
GO:0043062	35	2	0.053024669	extracellular structure organization and biogenesis
GO:0030198	35	2	0.053024669	extracellular matrix organization and biogenesis
GO:0007599	83	3	0.0581585	hemostasis
GO:0006487	37	2	0.058553404	protein amino acid N-linked glycosylation
GO:0048519	688	12	0.060014359	negative regulation of biological process
GO:0007178	38	2	0.061389702	transmembrane receptor protein serine/threonine kinase signaling pathway
GO:0042060	85	3	0.061593555	wound healing
GO:0016477	85	3	0.061593555	cell migration
GO:0050921	6	1	0.062119221	positive regulation of chemotaxis
GO:0018196	6	1	0.062119221	peptidyl-asparagine modification
GO:0018279	6	1	0.062119221	protein amino acid N-linked glycosylation via asparagine
GO:0042127	265	6	0.062677138	regulation of cell proliferation
GO:0050954	86	3	0.063346064	sensory perception of mechanical stimulus
GO:0050982	86	3	0.063346064	detection of mechanical stimulus
GO:0007605	86	3	0.063346064	perception of sound
GO:0048468	40	2	0.067199657	cell development
GO:0051249	41	2	0.070170777	regulation of lymphocyte activation
GO:0030500	7	1	0.072094465	regulation of bone mineralization
GO:0001570	7	1	0.072094465	vasculogenesis
GO:0045768	7	1	0.072094465	positive regulation of anti-apoptosis
GO:0050920	7	1	0.072094465	regulation of chemotaxis
GO:0050865	43	2	0.076239115	regulation of cell activation
GO:0048523	640	11	0.076385493	negative regulation of cellular process
GO:0001558	94	3	0.078180365	regulation of cell growth
GO:0050878	94	3	0.078180365	regulation of body fluids
GO:0042632	8	1	0.081964707	cholesterol homeostasis
GO:0030278	8	1	0.081964707	regulation of ossification
GO:0045078	8	1	0.081964707	positive regulation of interferon-gamma biosynthesis
GO:0046850	8	1	0.081964707	regulation of bone remodeling
GO:0042110	45	2	0.082467585	T cell activation
GO:0045767	9	1	0.09173104	regulation of anti-apoptosis
GO:0042730	9	1	0.09173104	fibrinolysis
GO:0030833	9	1	0.09173104	regulation of actin filament polymerization
GO:0007160	49	2	0.095367955	cell-matrix adhesion
GO:0007167	165	4	0.098548291	enzyme linked receptor protein signaling pathway
GO:0040008	104	3	0.09863978	regulation of growth
GO:0007155	518	9	0.098691077	cell adhesion
GO:0030282	10	1	0.101394547	bone mineralization
GO:0050793	168	4	0.103542312	regulation of development

GO:0009582	109	3	0.109603699	detection of abiotic stimulus
GO:0030193	11	1	0.110956298	regulation of blood coagulation
GO:0030195	11	1	0.110956298	negative regulation of blood coagulation
GO:0006171	11	1	0.110956298	cAMP biosynthesis
GO:0045072	11	1	0.110956298	regulation of interferon-gamma biosynthesis
GO:0045595	55	2	0.115696559	regulation of cell differentiation
GO:0050778	56	2	0.119185576	positive regulation of immune response
GO:0042095	12	1	0.120417355	interferon-gamma biosynthesis
GO:0006915	471	8	0.127231519	apoptosis
GO:0008283	473	8	0.129454602	cell proliferation
GO:0012501	473	8	0.129454602	programmed cell death
GO:0050818	13	1	0.129778767	regulation of coagulation
GO:0050819	13	1	0.129778767	negative regulation of coagulation
GO:0046058	13	1	0.129778767	cAMP metabolism
GO:0030595	13	1	0.129778767	immune cell chemotaxis
GO:0051240	59	2	0.129806301	positive regulation of organismal physiological process
GO:0000902	183	4	0.130187106	cellular morphogenesis
GO:0007050	61	2	0.13700569	cell cycle arrest
GO:0008284	121	3	0.137695142	positive regulation of cell proliferation
GO:0050900	14	1	0.139041572	immune cell migration
GO:0008361	123	3	0.142599319	regulation of cell size
GO:0016049	123	3	0.142599319	cell growth
GO:0007162	15	1	0.148206798	negative regulation of cell adhesion
GO:0007585	15	1	0.148206798	respiratory gaseous exchange
GO:0001525	65	2	0.15165832	angiogenesis
GO:0045637	16	1	0.157275463	regulation of myeloid cell differentiation
GO:0007610	269	5	0.157866245	behavior
GO:0008219	498	8	0.158928608	cell death
GO:0016265	501	8	0.162668321	death
GO:0006904	17	1	0.166248572	vesicle docking during exocytosis
GO:0007611	17	1	0.166248572	learning and/or memory
GO:0008544	70	2	0.170382271	epidermis development
GO:0006928	205	4	0.173771996	cell motility
GO:0040011	205	4	0.173771996	locomotion
GO:0048278	18	1	0.175127123	vesicle docking
GO:0045597	18	1	0.175127123	positive regulation of cell differentiation
GO:0000080	18	1	0.175127123	G1 phase of mitotic cell cycle
GO:0030041	18	1	0.175127123	actin filament polymerization
GO:0000910	18	1	0.175127123	cytokinesis
GO:0006811	515	8	0.180663542	ion transport
GO:0007626	209	4	0.182195038	locomotory behavior
GO:0008285	139	3	0.183762068	negative regulation of cell proliferation
GO:0043118	602	9	0.188936582	negative regulation of physiological process
GO:0007600	287	5	0.189730722	sensory perception
GO:0009190	20	1	0.192604484	cyclic nucleotide biosynthesis
GO:0051241	20	1	0.192604484	negative regulation of organismal physiological process
GO:0050776	77	2	0.197188146	regulation of immune response

GO:0051318	21	1	0.201205235	G1 phase
GO:0046649	81	2	0.21273518	lymphocyte activation
GO:0042108	23	1	0.218135658	positive regulation of cytokine biosynthesis
GO:0009605	963	13	0.220081827	response to external stimulus
GO:0040007	154	3	0.224843636	growth
GO:0043065	155	3	0.227647908	positive regulation of apoptosis
GO:0043068	156	3	0.230459074	positive regulation of programmed cell death
GO:0008360	25	1	0.234710902	regulation of cell shape
GO:0045727	25	1	0.234710902	positive regulation of protein biosynthesis
GO:0006968	87	2	0.236263044	cellular defense response
GO:0016055	87	2	0.236263044	Wnt receptor signaling pathway
GO:0009581	313	5	0.239393467	detection of external stimulus
GO:0031328	26	1	0.242867641	positive regulation of cellular biosynthesis
GO:0007507	26	1	0.242867641	heart development
GO:0007411	27	1	0.250938339	axon guidance
GO:0009187	27	1	0.250938339	cyclic nucleotide metabolism
GO:0006486	92	2	0.255983032	protein amino acid glycosylation
GO:0043413	93	2	0.259933235	biopolymer glycosylation
GO:0001819	29	1	0.266825194	positive regulation of cytokine production
GO:0008624	29	1	0.266825194	induction of apoptosis by extracellular signals
GO:0051242	411	6	0.271625208	positive regulation of cellular physiological process
GO:0009101	96	2	0.271787686	glycoprotein biosynthesis
GO:0045944	30	1	0.274643119	positive regulation of transcription from RNA polymerase II promoter
GO:0009891	30	1	0.274643119	positive regulation of biosynthesis
GO:0006952	751	10	0.274759803	defense response
GO:0045321	97	2	0.275738943	immune cell activation
GO:0051243	583	8	0.278910929	negative regulation of cellular physiological process
GO:0001775	98	2	0.279689358	cell activation
GO:0051094	31	1	0.282378541	positive regulation of development
GO:0007154	2730	32	0.286200784	cell communication
GO:0006118	256	4	0.289261015	electron transport
GO:0044249	763	10	0.291281647	cellular biosynthesis
GO:0042035	33	1	0.297605308	regulation of cytokine biosynthesis
GO:0030155	33	1	0.297605308	regulation of cell adhesion
GO:0008064	33	1	0.297605308	regulation of actin polymerization and/or depolymerization
GO:0008015	103	2	0.299414245	circulation
GO:0043119	428	6	0.303080864	positive regulation of physiological process
GO:0009058	859	11	0.303451404	biosynthesis
GO:0042742	34	1	0.30509835	defense response to bacteria
GO:0030832	35	1	0.312512282	regulation of actin filament length
GO:0006954	185	3	0.314027814	inflammatory response
GO:0042089	36	1	0.31984793	cytokine biosynthesis
GO:0000079	36	1	0.31984793	regulation of cyclin dependent protein kinase activity
GO:0042107	37	1	0.327106111	cytokine metabolism
GO:0030099	37	1	0.327106111	myeloid cell differentiation
GO:0051258	37	1	0.327106111	protein polymerization
GO:0009100	111	2	0.330786763	glycoprotein metabolism

GO:0006821	38	1	0.334287635	chloride transport
GO:0001503	38	1	0.334287635	ossification
GO:0031214	38	1	0.334287635	biomineral formation
GO:0001817	39	1	0.341393304	regulation of cytokine production
GO:0007409	40	1	0.348423911	axonogenesis
GO:0007588	40	1	0.348423911	excretion
GO:0006766	40	1	0.348423911	vitamin metabolism
GO:0007169	116	2	0.350210158	transmembrane receptor protein tyrosine kinase signaling pathway
GO:0051247	41	1	0.355380239	positive regulation of protein metabolism
GO:0046849	41	1	0.355380239	bone remodeling
GO:0007165	2168	25	0.356593749	signal transduction
GO:0001816	42	1	0.362263066	cytokine production
GO:0006096	42	1	0.362263066	glycolysis
GO:0000086	44	1	0.375811282	G2/M transition of mitotic cell cycle
GO:0008154	45	1	0.382478184	actin polymerization and/or depolymerization
GO:0007229	45	1	0.382478184	integrin-mediated signaling pathway
GO:0031175	46	1	0.389074612	neurite morphogenesis
GO:0050877	475	6	0.392773156	neurophysiological process
GO:0048522	482	6	0.406254318	positive regulation of cellular process
GO:0007010	305	4	0.407933646	cytoskeleton organization and biogenesis
GO:0050896	1578	18	0.411336768	response to stimulus
GO:0051239	133	2	0.414659468	regulation of organismal physiological process
GO:0018193	50	1	0.414770206	peptidyl-amino acid modification
GO:0006007	51	1	0.421025167	glucose catabolism
GO:0009617	51	1	0.421025167	response to bacteria
GO:0031325	136	2	0.425720069	positive regulation of cellular metabolism
GO:0051128	52	1	0.427213963	regulation of cell organization and biogenesis
GO:0006869	52	1	0.427213963	lipid transport
GO:0009607	857	10	0.427577605	response to biotic stimulus
GO:0000082	53	1	0.433337289	G1/S transition of mitotic cell cycle
GO:0009628	318	4	0.439156115	response to abiotic stimulus
GO:0006917	140	2	0.440300256	induction of apoptosis
GO:0012502	140	2	0.440300256	induction of programmed cell death
GO:0009966	229	3	0.441170293	regulation of signal transduction
GO:0006887	55	1	0.445390258	exocytosis
GO:0007218	56	1	0.451321253	neuropeptide signaling pathway
GO:0045786	145	2	0.458242784	negative regulation of progression through cell cycle
GO:0019320	59	1	0.468740224	hexose catabolism
GO:0046365	59	1	0.468740224	monosaccharide catabolism
GO:0009893	149	2	0.472360012	positive regulation of metabolism
GO:0050953	149	2	0.472360012	sensory perception of light
GO:0007601	149	2	0.472360012	visual perception
GO:0008203	60	1	0.474424047	cholesterol metabolism
GO:0046164	60	1	0.474424047	alcohol catabolism
GO:0009790	65	1	0.501952926	embryonic development
GO:0016125	66	1	0.507284979	sterol metabolism
GO:0016042	67	1	0.512560536	lipid catabolism

GO:0006091	446	5	0.51700006	generation of precursor metabolites and energy
GO:0006508	451	5	0.52703304	proteolysis and peptidolysis
GO:0044257	451	5	0.52703304	cellular protein catabolism
GO:0016337	172	2	0.549114135	cell-cell adhesion
GO:0030163	463	5	0.550746785	protein catabolism
GO:0000004	465	5	0.554645908	biological process unknown
GO:0051246	174	2	0.555415061	regulation of protein metabolism
GO:0044265	563	6	0.557694157	cellular macromolecule catabolism
GO:0048518	565	6	0.561239298	positive regulation of biological process
GO:0045893	77	1	0.562336142	positive regulation of transcription, DNA-dependent
GO:0007166	952	10	0.565302728	cell surface receptor linked signal transduction
GO:0016052	79	1	0.57167011	carbohydrate catabolism
GO:0051329	79	1	0.57167011	interphase of mitotic cell cycle
GO:0006006	79	1	0.57167011	glucose metabolism
GO:0044275	79	1	0.57167011	cellular carbohydrate catabolism
GO:0043285	474	5	0.571992097	biopolymer catabolism
GO:0009057	575	6	0.578770367	macromolecule catabolism
GO:0051325	82	1	0.585302913	interphase
GO:0030097	87	1	0.607076383	hemopoiesis
GO:0000074	394	4	0.60842117	regulation of progression through cell cycle
GO:0048534	88	1	0.611293338	hemopoietic or lymphoid organ development
GO:0006092	88	1	0.611293338	main pathways of carbohydrate metabolism
GO:0006412	407	4	0.634202761	protein biosynthesis
GO:0045941	100	1	0.658534318	positive regulation of transcription
GO:0006935	101	1	0.662204347	chemotaxis
GO:0042330	101	1	0.662204347	taxis
GO:0006066	213	2	0.665869247	alcohol metabolism
GO:0009165	103	1	0.669427702	nucleotide biosynthesis
GO:0006366	426	4	0.669913878	transcription from RNA polymerase II promoter
GO:0045935	105	1	0.676498169	positive regulation of nucleobase, nucleoside, nucleotide and nucleic acid metabolism
GO:0007156	105	1	0.676498169	homophilic cell adhesion
GO:0006417	107	1	0.683418951	regulation of protein biosynthesis
GO:0031326	111	1	0.696823939	regulation of cellular biosynthesis
GO:0009056	756	7	0.703041827	catabolism
GO:0019318	113	1	0.703314223	hexose metabolism
GO:0005996	115	1	0.709666983	monosaccharide metabolism
GO:0045859	116	1	0.712792694	regulation of protein kinase activity
GO:0009889	116	1	0.712792694	regulation of biosynthesis
GO:0051338	117	1	0.715885102	regulation of transferase activity
GO:0009613	458	4	0.724595762	response to pest, pathogen or parasite
GO:0006357	240	2	0.728776075	regulation of transcription from RNA polymerase II promoter
GO:0006950	881	8	0.73139171	response to stress
GO:0043207	463	4	0.732514889	response to external biotic stimulus
GO:0009059	464	4	0.734078512	macromolecule biosynthesis
GO:0030036	126	1	0.742274814	actin cytoskeleton organization and biogenesis
GO:0015980	132	1	0.758504972	energy derivation by oxidation of organic compounds
GO:0044248	700	6	0.764352979	cellular catabolism

GO:0030029	139	1	0.776165408	actin filament-based process
GO:0006959	141	1	0.780972185	humoral immune response
GO:0051301	142	1	0.783337124	cell division
GO:0009117	143	1	0.78567679	nucleotide metabolism
GO:0016043	842	7	0.803301857	cell organization and biogenesis
GO:0042592	157	1	0.815923244	homeostasis
GO:0045045	161	1	0.82376081	secretory pathway
GO:0042221	183	1	0.861318486	response to chemical substance
GO:0006955	684	5	0.861912785	immune response
GO:0050794	2488	22	0.873184464	regulation of cellular process
GO:0007268	192	1	0.874291749	synaptic transmission
GO:0006461	194	1	0.877007391	protein complex assembly
GO:0019226	198	1	0.882265703	transmission of nerve impulse
GO:0050791	2407	21	0.88260848	regulation of physiological process
GO:0006810	1802	15	0.886491976	transport
GO:0051244	2319	20	0.888691882	regulation of cellular physiological process
GO:0000278	204	1	0.889738788	mitotic cell cycle
GO:0007049	601	4	0.889847129	cell cycle
GO:0046903	208	1	0.894458047	secretion
GO:0005975	355	2	0.896332846	carbohydrate metabolism
GO:0050790	214	1	0.901164621	regulation of enzyme activity
GO:0051234	1841	15	0.905037383	establishment of localization
GO:0051179	1857	15	0.911914592	localization
GO:0007264	227	1	0.914282805	small GTPase mediated signal transduction
GO:0050789	2689	23	0.916154673	regulation of biological process
GO:0007399	385	2	0.920557719	neurogenesis
GO:0016070	391	2	0.92472303	RNA metabolism
GO:0006996	693	4	0.943413455	organelle organization and biogenesis
GO:0044262	265	1	0.943536995	cellular carbohydrate metabolism
GO:0016192	296	1	0.95988822	vesicle-mediated transport
GO:0007186	491	2	0.970134747	G-protein coupled receptor protein signaling pathway
GO:0006396	326	1	0.971221734	RNA processing
GO:0044267	2109	14	0.988155854	cellular protein metabolism
GO:0019538	2129	14	0.989581591	protein metabolism
GO:0031323	1566	9	0.990826646	regulation of cellular metabolism
GO:0019219	1458	8	0.991764953	regulation of nucleobase, nucleoside, nucleotide and nucleic acid metabolism
GO:0044260	2298	15	0.992814496	cellular macromolecule metabolism
GO:0006464	1087	5	0.992882771	protein modification
GO:0006355	1359	7	0.993303996	regulation of transcription, DNA-dependent
GO:0019222	1629	9	0.994213923	regulation of metabolism
GO:0007582	7846	75	0.994266545	physiological process
GO:0006351	1407	7	0.995411489	transcription, DNA-dependent
GO:0043412	1160	5	0.996167542	biopolymer modification
GO:0045449	1433	7	0.996275457	regulation of transcription
GO:0043170	2405	15	0.99653061	macromolecule metabolism
GO:0007242	879	3	0.996652409	intracellular signaling cascade
GO:0006350	1499	7	0.997832712	transcription

GO:0043283	2240	12	0.999130143	biopolymer metabolism
GO:0006139	2328	10	0.99995394	nucleobase, nucleoside, nucleotide and nucleic acid metabolism
GO:0050875	7018	58	0.999960744	cellular physiological process
GO:0044238	4820	31	0.999991024	primary metabolism
GO:0009987	8200	73	0.999995823	cellular process
GO:0008152	5262	33	0.999999359	metabolism
GO:0044237	5026	30	0.999999631	cellular metabolism
GO:0008150	9032	96	1	biological_process

12.3 GO: Molecular Function

GO ID	genes present in chip	genes present in tissues	p-value	GO term
GO:0005198	488	21	1.32637712231484e-08	structural molecule activity
GO:0005201	73	9	4.52174663261707e-08	extracellular matrix structural constituent
GO:0005179	90	9	2.85431070040729e-07	hormone activity
GO:0005102	528	17	2.00569921536269e-05	receptor binding
GO:0016229	15	3	0.0004241872584432	steroid dehydrogenase activity
GO:0004867	64	5	0.000464672720559031	serine-type endopeptidase inhibitor activity
GO:0004866	99	6	0.000494694415125668	endopeptidase inhibitor activity
GO:0030414	100	6	0.000522004100624951	protease inhibitor activity
GO:0008147	4	2	0.00060571453706097	structural constituent of bone
GO:0004857	187	8	0.000611931482493618	enzyme inhibitor activity
GO:0008243	5	2	0.00100283174167855	plasminogen activator activity
GO:0005520	20	3	0.00102432082127741	insulin-like growth factor binding
GO:0005539	77	5	0.00108534549774523	glycosaminoglycan binding
GO:0030247	80	5	0.00128934631419206	polysaccharide binding
GO:0004303	6	2	0.00149428302219377	estradiol 17-beta-dehydrogenase activity
GO:0001871	88	5	0.0019713419431439	pattern binding
GO:0030246	181	7	0.00240707455930320	carbohydrate binding
GO:0008201	60	4	0.00316769948797595	heparin binding
GO:0016641	11	2	0.00530035768547147	oxidoreductase activity, acting on the CH-NH2 group of donors, oxygen as acceptor
GO:0016638	14	2	0.00859747935677708	oxidoreductase activity, acting on the CH-NH2 group of donors
GO:0008386	1	1	0.0101666491981973	cholesterol monooxygenase (side-chain-cleaving) activity
GO:0008402	1	1	0.0101666491981973	aromatase activity
GO:0017147	1	1	0.0101666491981973	Wnt-protein binding
GO:0005200	86	4	0.0112950247010611	structural constituent of cytoskeleton
GO:0019838	49	3	0.0133882785935585	growth factor binding
GO:0008083	144	5	0.0155337451118911	growth factor activity
GO:0004619	2	1	0.0202309924925138	phosphoglycerate mutase activity
GO:0048306	2	1	0.0202309924925138	calcium-dependent protein binding
GO:0008160	2	1	0.0202309924925138	protein tyrosine phosphatase activator activity
GO:0005131	2	1	0.0202309924925138	growth hormone receptor binding
GO:0019870	2	1	0.0202309924925138	potassium channel inhibitor activity
GO:0008970	2	1	0.0202309924925138	phospholipase A1 activity
GO:0004082	2	1	0.0202309924925138	bisphosphoglycerate mutase activity
GO:0004083	2	1	0.0202309924925138	bisphosphoglycerate phosphatase activity
GO:0005010	2	1	0.0202309924925138	insulin-like growth factor receptor activity
GO:0019211	3	1	0.0301940487592318	phosphatase activator activity
GO:0003854	3	1	0.0301940487592318	3-beta-hydroxy-delta5-steroid dehydrogenase activity
GO:0019834	3	1	0.0301940487592318	phospholipase A2 inhibitor activity
GO:0050510	3	1	0.0301940487592318	N-acetylgalactosaminyl-proteoglycan 3-beta-glucuronosyltransferase activity
GO:0047238	3	1	0.0301940487592318	glucuronosyl-N-acetylgalactosaminyl-proteoglycan 4-beta-N-acetylgalactosaminyltransferase activity
GO:0004769	3	1	0.0301940487592318	steroid delta-isomerase activity
GO:0004720	3	1	0.0301940487592318	protein-lysine 6-oxidase activity

GO:0030234	509	10	0.0335900832820842	enzyme regulator activity
GO:0019825	31	2	0.0392971910965106	oxygen binding
GO:0016713	4	1	0.040056826833289	oxidoreductase activity, acting on paired donors, with incorporation or reduction of molecular oxygen, reduced iron-sulfur protein as one donor, and incorporation of one atom of oxygen
GO:0004035	4	1	0.040056826833289	alkaline phosphatase activity
GO:0004871	1837	26	0.0426576912098424	signal transducer activity
GO:0016616	77	3	0.0434237309258604	oxidoreductase activity, acting on the CH-OH group of donors, NAD or NADP as acceptor
GO:0008131	5	1	0.0498203256061914	amine oxidase activity
GO:0004465	5	1	0.0498203256061914	lipoprotein lipase activity
GO:0016614	83	3	0.0522488408568638	oxidoreductase activity, acting on CH-OH group of donors
GO:0005515	3709	46	0.0524783423713979	protein binding
GO:0004579	6	1	0.0594855341229903	dolichyl-diphosphooligosaccharide-protein glycotransferase activity
GO:0015485	6	1	0.0594855341229903	cholesterol binding
GO:0004571	7	1	0.0690534316783197	mannosyl-oligosaccharide 1,2-alpha-mannosidase activity
GO:0008200	7	1	0.0690534316783197	ion channel inhibitor activity
GO:0016248	7	1	0.0690534316783197	channel inhibitor activity
GO:0016868	7	1	0.0690534316783197	intramolecular transferase activity, phosphotransferases
GO:0004175	288	6	0.0727677420975418	endopeptidase activity
GO:0016491	509	9	0.0734940420539434	oxidoreductase activity
GO:0004861	8	1	0.0785249879115075	cyclin-dependent protein kinase inhibitor activity
GO:0004859	8	1	0.0785249879115075	phospholipase inhibitor activity
GO:0004576	8	1	0.0785249879115075	oligosaccharyl transferase activity
GO:0008191	8	1	0.0785249879115075	metalloendopeptidase inhibitor activity
GO:0005099	8	1	0.0785249879115075	Ras GTPase activator activity
GO:0004896	48	2	0.0854011596341177	hematopoietin/interferon-class (D200-domain) cytokine receptor activity
GO:0005529	103	3	0.0872311938543314	sugar binding
GO:0004806	9	1	0.0879011629007638	triacylglycerol lipase activity
GO:0005159	9	1	0.0879011629007638	insulin-like growth factor receptor binding
GO:0015924	9	1	0.0879011629007638	mannosyl-oligosaccharide mannosidase activity
GO:0005507	49	2	0.0884655375507372	copper ion binding
GO:0009055	104	3	0.0891890850411353	electron carrier activity
GO:0004252	105	3	0.0911654817080558	serine-type endopeptidase activity
GO:0016863	10	1	0.097182907256474	intramolecular oxidoreductase activity, transposing C=C bonds
GO:0016866	11	1	0.106371162213574	intramolecular transferase activity
GO:0008233	399	7	0.111084533309568	peptidase activity
GO:0005125	181	4	0.112410068771834	cytokine activity
GO:0008395	12	1	0.115466859723048	steroid hydroxylase activity
GO:0004198	12	1	0.115466859723048	calpain activity
GO:0008236	117	3	0.116251545387088	serine-type peptidase activity
GO:0015923	13	1	0.124470922542532	mannosidase activity
GO:0005518	14	1	0.133384264326052	collagen binding
GO:0008144	15	1	0.142207789712886	drug binding
GO:0030159	15	1	0.142207789712886	receptor signaling complex scaffold activity
GO:0005247	16	1	0.150942394415578	voltage-gated chloride channel activity
GO:0015459	16	1	0.150942394415578	potassium channel regulator activity
GO:0030674	69	2	0.155396598574912	protein binding, bridging
GO:0005544	17	1	0.159588965307094	calcium-dependent phospholipid binding

GO:0005504	17	1	0.159588965307094	fatty acid binding
GO:0004497	71	2	0.162542889669034	monooxygenase activity
GO:0008376	18	1	0.168148380507137	acetylgalactosaminyltransferase activity
GO:0046906	74	2	0.173371105677401	tetrapyrrole binding
GO:0020037	74	2	0.173371105677401	heme binding
GO:0050381	20	1	0.185009213057388	unspecific monooxygenase activity
GO:0005184	21	1	0.193312343645922	neuropeptide hormone activity
GO:0015020	21	1	0.193312343645922	glucuronosyltransferase activity
GO:0004197	80	2	0.195356108973054	cysteine-type endopeptidase activity
GO:0005489	152	3	0.201055559925075	electron transporter activity
GO:0016705	87	2	0.221416443890428	oxidoreductase activity, acting on paired donors, with incorporation or reduction of molecular oxygen
GO:0004860	25	1	0.225695890530617	protein kinase inhibitor activity
GO:0016712	25	1	0.225695890530617	oxidoreductase activity, acting on paired donors, with incorporation or reduction of molecular oxygen, reduced flavin or flavoprotein as one donor, and incorporation of one atom of oxygen
GO:0005178	26	1	0.233588649948285	integrin binding
GO:0016247	26	1	0.233588649948285	channel regulator activity
GO:0048503	92	2	0.24020603642135	GPI anchor binding
GO:0016853	92	2	0.24020603642135	isomerase activity
GO:0005543	169	3	0.246489254422409	phospholipid binding
GO:0019210	29	1	0.256792304913042	kinase inhibitor activity
GO:0015457	29	1	0.256792304913042	auxiliary transport protein activity
GO:0008234	98	2	0.262851769189908	cysteine-type peptidase activity
GO:0019965	30	1	0.264371273208189	interleukin binding
GO:0016860	31	1	0.271873742611911	intramolecular oxidoreductase activity
GO:0015035	32	1	0.279300477308719	protein disulfide oxidoreductase activity
GO:0008307	33	1	0.286652233928875	structural constituent of muscle
GO:0019888	35	1	0.301133802131656	protein phosphatase regulator activity
GO:0019208	36	1	0.308265089865006	phosphatase regulator activity
GO:0005126	38	1	0.322312308391959	hematopoietin/interferon-class (D200-domain) cytokine receptor binding
GO:0016758	115	2	0.326861682237963	transferase activity, transferring hexosyl groups
GO:0005496	42	1	0.349565228582238	steroid binding
GO:0051020	42	1	0.349565228582238	GTPase binding
GO:0015036	42	1	0.349565228582238	disulfide oxidoreductase activity
GO:0005254	43	1	0.356207209088346	chloride channel activity
GO:0008289	298	4	0.359497171848726	lipid binding
GO:0005506	212	3	0.365930781967082	iron ion binding
GO:0031404	45	1	0.36929044236101	chloride ion binding
GO:0043168	45	1	0.36929044236101	anion binding
GO:0005253	46	1	0.375733031566042	anion channel activity
GO:0004620	47	1	0.382110482428401	phospholipase activity
GO:0019887	47	1	0.382110482428401	protein kinase regulator activity
GO:0035091	132	2	0.389407375050882	phosphoinositide binding
GO:0008237	133	2	0.393013981595251	metallopeptidase activity
GO:0005554	522	6	0.439451630734131	molecular function unknown
GO:0019207	58	1	0.448145190355923	kinase regulator activity
GO:0004553	59	1	0.453790019685827	hydrolase activity, hydrolyzing O-glycosyl compounds
GO:0016298	59	1	0.453790019685827	lipase activity

GO:0004714	60	1	0.459377698244198	transmembrane receptor protein tyrosine kinase activity
GO:0019955	65	1	0.486478674747763	cytokine binding
GO:0005509	646	7	0.487625768838158	calcium ion binding
GO:0016757	165	2	0.502653660257945	transferase activity, transferring glycosyl groups
GO:0016791	171	2	0.52179252846664	phosphoric monoester hydrolase activity
GO:0019199	73	1	0.527069770291476	transmembrane receptor protein kinase activity
GO:0016798	77	1	0.546157691126439	hydrolase activity, acting on glycosyl bonds
GO:0004222	77	1	0.546157691126439	metalloendopeptidase activity
GO:0008194	79	1	0.555413693611857	UDP-glycosyltransferase activity
GO:0016789	80	1	0.559971384556651	carboxylic ester hydrolase activity
GO:0008047	188	2	0.573326806603799	enzyme activator activity
GO:0003702	197	2	0.598957750353357	RNA polymerase II transcription factor activity
GO:0008509	95	1	0.623039036839719	anion transporter activity
GO:0042578	228	2	0.67843282452757	phosphoric ester hydrolase activity
GO:0005096	117	1	0.699693436177018	GTPase activator activity
GO:0003924	121	1	0.711869823238156	GTPase activity
GO:0005244	124	1	0.720680246143717	voltage-gated ion channel activity
GO:0000287	251	2	0.728842076505934	magnesium ion binding
GO:0005057	132	1	0.742892385217202	receptor signaling protein activity
GO:0003713	140	1	0.763354930807116	transcription coactivator activity
GO:0005083	144	1	0.772973180258024	small GTPase regulator activity
GO:0005386	398	3	0.776301832420277	carrier activity
GO:0019899	151	1	0.788883056424372	enzyme binding
GO:0016787	1424	12	0.800669095511205	hydrolase activity
GO:0008134	293	2	0.803831346633201	transcription factor binding
GO:0004713	161	1	0.809714892672836	protein-tyrosine kinase activity
GO:0046983	170	1	0.826716591943888	protein dimerization activity
GO:0016788	446	3	0.838286006601627	hydrolase activity, acting on ester bonds
GO:0004888	694	5	0.843542956800592	transmembrane receptor activity
GO:0043167	2556	22	0.849782994344287	ion binding
GO:0004872	1060	8	0.858381516381035	receptor activity
GO:0046872	2495	21	0.872041441176866	metal ion binding
GO:0005488	7228	69	0.881063531518675	binding
GO:0043169	2313	19	0.885837359007013	cation binding
GO:0030695	219	1	0.896065081522288	GTPase regulator activity
GO:0005525	226	1	0.90340556171741	GTP binding
GO:0003700	649	4	0.903972139278342	transcription factor activity
GO:0046914	1596	12	0.90610536974033	transition metal ion binding
GO:0003712	230	1	0.907367250439253	transcription cofactor activity
GO:0019001	235	1	0.912093892557606	guanyl nucleotide binding
GO:0005216	252	1	0.926448363790932	ion channel activity
GO:0030528	963	6	0.935755952965675	transcription regulator activity
GO:0015268	274	1	0.941631120903036	alpha-type channel activity
GO:0008092	279	1	0.944623276304897	cytoskeletal protein binding
GO:0015267	284	1	0.947463545979151	channel or pore class transporter activity
GO:0004930	349	1	0.973572189338271	G-protein coupled receptor activity
GO:0008270	1287	7	0.98242761391684	zinc ion binding

GO:0005215	1030	5	0.983680621938555	transporter activity
GO:0017111	410	1	0.986193330953633	nucleoside-triphosphatase activity
GO:0016462	430	1	0.98885123879762	pyrophosphatase activity
GO:0016818	432	1	0.98908737793775	hydrolase activity, acting on acid anhydrides, in phosphorus-containing anhydrides
GO:0016817	433	1	0.989203584364365	hydrolase activity, acting on acid anhydrides
GO:0003723	436	1	0.989544905597023	RNA binding
GO:0004672	447	1	0.990707503880938	protein kinase activity
GO:0015075	523	1	0.995900722312103	ion transporter activity
GO:0003824	3409	23	0.99615839912104	catalytic activity
GO:0016773	532	1	0.996281067880631	phosphotransferase activity, alcohol group as acceptor
GO:0003677	1384	6	0.996912296511734	DNA binding
GO:0016301	609	1	0.998389852327374	kinase activity
GO:0016772	708	1	0.999457021149386	transferase activity, transferring phosphorus-containing groups
GO:0016740	1169	3	0.999687062655387	transferase activity
GO:0003676	1990	8	0.99979284180879	nucleic acid binding
GO:0000166	1432	2	0.999997629752343	nucleotide binding
GO:0017076	1248	1	0.99999884575846	purine nucleotide binding
GO:0003674	9541	97	1	molecular_function

12.4 GO: Cellular Component

GO ID	genes present in chip	genes present in tissues	p-value	GO term
GO:0005576	949	44	6.76627882075025e-18	extracellular region
GO:0005581	30	8	9.0227529679953e-10	collagen
GO:0005583	10	4	2.98143147514759e-06	fibrillar collagen
GO:0005615	385	16	5.13529691328615e-06	extracellular space
GO:0005578	303	14	6.41011302384595e-06	extracellular matrix (sensu Metazoa)
GO:0031012	304	14	6.65814370219026e-06	extracellular matrix
GO:0045111	65	7	7.41079202907778e-06	intermediate filament cytoskeleton
GO:0005882	65	7	7.41079202907778e-06	intermediate filament
GO:0005584	3	2	0.000371784774864311	collagen type I
GO:0030935	6	2	0.00181838792550241	sheet-forming collagen
GO:0005587	6	2	0.00181838792550241	collagen type IV
GO:0001533	12	2	0.0076570625777457	cornified envelope
GO:0005582	1	1	0.0112307514183171	collagen type XV
GO:0005586	1	1	0.0112307514183171	collagen type III
GO:0005604	44	3	0.0130389605116375	basement membrane
GO:0005625	183	6	0.0168025847748627	soluble fraction
GO:0005589	2	1	0.0223366589116492	collagen type VI
GO:0030868	2	1	0.0223366589116492	smooth endoplasmic reticulum membrane
GO:0005811	3	1	0.0333190959800422	lipid particle
GO:0043034	4	1	0.0441794211699815	costamere
GO:0001527	4	1	0.0441794211699815	microfibril
GO:0000267	698	13	0.0477602174113873	cell fraction
GO:0043205	5	1	0.0549189782354867	fibril
GO:0016599	5	1	0.0549189782354867	caveolar membrane
GO:0008250	6	1	0.0655390962974844	oligosaccharyl transferase complex
GO:0005901	6	1	0.0655390962974844	caveola
GO:0005789	84	3	0.0682491502669016	endoplasmic reticulum membrane
GO:0005913	7	1	0.0760410900014756	cell-cell adherens junction
GO:0042175	90	3	0.0802762416473516	nuclear envelope-endoplasmic reticulum network
GO:0030934	8	1	0.0864262596735322	anchoring collagen
GO:0005790	10	1	0.106851257553323	smooth endoplasmic reticulum
GO:0005792	108	3	0.121244685801323	microsome
GO:0042598	108	3	0.121244685801323	vesicular fraction
GO:0005769	13	1	0.136644275915185	early endosome
GO:0012505	246	5	0.142707071350424	endomembrane system
GO:0045121	14	1	0.146355025594711	lipid raft
GO:0005758	14	1	0.146355025594711	mitochondrial intermembrane space
GO:0000139	67	2	0.173366955473958	Golgi membrane
GO:0005912	18	1	0.184128772066116	adherens junction
GO:0005834	21	1	0.211369000462279	heterotrimeric G-protein complex
GO:0005856	612	9	0.248200141411280	cytoskeleton
GO:0019897	26	1	0.254783018473052	extrinsic to plasma membrane
GO:0008021	27	1	0.263177635498731	synaptic vesicle

GO:0030173	29	1	0.279687106854223	integral to Golgi membrane
GO:0005783	483	7	0.298897636488242	endoplasmic reticulum
GO:0030016	32	1	0.303767231537933	myofibril
GO:0031228	35	1	0.327050379110691	intrinsic to Golgi membrane
GO:0019898	37	1	0.342142642256644	extrinsic to membrane
GO:0043292	40	1	0.364155101387454	contractile fiber
GO:0005624	521	7	0.368810767893413	membrane fraction
GO:0005737	2530	30	0.398286374005901	cytoplasm
GO:0031090	377	5	0.417982801252305	organelle membrane
GO:0005768	62	1	0.504775894356543	endosome
GO:0030136	65	1	0.521394276220291	clathrin-coated vesicle
GO:0005777	67	1	0.532165336317793	peroxisome
GO:0042579	67	1	0.532165336317793	microbody
GO:0031301	76	1	0.577740957682202	integral to organelle membrane
GO:0030135	79	1	0.591933740744826	coated vesicle
GO:0005911	82	1	0.605654236186852	intercellular junction
GO:0005743	85	1	0.618918001833102	mitochondrial inner membrane
GO:0019866	89	1	0.635918496399499	inner membrane
GO:0031300	90	1	0.64004997813198	intrinsic to organelle membrane
GO:0005667	91	1	0.644135055015237	transcription factor complex
GO:0005794	195	2	0.647945412408752	Golgi apparatus
GO:0030054	93	1	0.652168051962497	cell junction
GO:0008372	523	5	0.707309000453775	cellular component unknown
GO:0016020	3514	37	0.729728596074407	membrane
GO:0005740	119	1	0.741624100323898	mitochondrial membrane
GO:0016023	122	1	0.750351860117441	cytoplasmic membrane-bound vesicle
GO:0031410	124	1	0.756007607854623	cytoplasmic vesicle
GO:0043228	977	9	0.782963341794299	non-membrane-bound organelle
GO:0043232	977	9	0.782963341794299	intracellular non-membrane-bound organelle
GO:0005739	490	3	0.919158227542789	mitochondrion
GO:0005654	221	1	0.920226675443786	nucleoplasm
GO:0005886	1472	11	0.955424059332426	plasma membrane
GO:0016021	2674	20	0.991883229888544	integral to membrane
GO:0031224	2681	20	0.99224693078652	intrinsic to membrane
GO:0005622	5419	48	0.997271165738597	intracellular
GO:0005887	1057	4	0.998468709733055	integral to plasma membrane
GO:0031226	1062	4	0.998542676771393	intrinsic to plasma membrane
GO:0043226	4515	32	0.999961485442072	organelle
GO:0043229	4515	32	0.999961485442072	intracellular organelle
GO:0043234	1247	3	0.99996218128743	protein complex
GO:0005623	7802	75	0.999963487681153	cell
GO:0043227	3955	23	0.999998202368107	membrane-bound organelle
GO:0043231	3955	23	0.999998202368107	intracellular membrane-bound organelle
GO:0005634	2704	11	0.999999132797953	nucleus
GO:0005575	8637	97	1	cellular_component

13. 721-BLymphoblasts

13.1 721-BLymphoblasts selective genes (137 genes)

Symbol	Description	Chromosome	Chromosome Location	LocusLink	Cytoband	UniGene
BIRC3	baculoviral IAP repeat-containing 3	11	101693403	330	11q22	Hs.127799
PMAIP1	phorbol-12-myristate-13-acetate-induced protein 1	18	55718216	5366	18q21.32	Hs.96
CCR7	chemokine (C-C motif) receptor 7	17	-35963549	1236	17q12-q21.2	Hs.370036
RRM2	ribonucleotide reductase M2 polypeptide	2	10180185	6241	2p25-p24	Hs.226390
MCM5	MCM5 minichromosome maintenance deficient 5, cell division cycle 46 (S. cerevisiae)	22	34126127	4174	22q13.1	Hs.517582
TOP2A	topoisomerase (DNA) II alpha 170kDa	17	-35798323	7153	17q21-q22	Hs.156346
CDC20	CDC20 cell division cycle 20 homolog (S. cerevisiae)	1	43597214	991	1p34.1	Hs.524947
DLG7	discs, large homolog 7 (Drosophila)	14	-54684600	9787	14q22.3	Hs.77695
DTL	denticleless homolog (Drosophila)	1	210275753	51514	NA	Hs.632496
GHR	growth hormone receptor	5	42459782	2690	5p13-p12	Hs.125180
BUB1B	BUB1 budding uninhibited by benzimidazoles 1 homolog beta (yeast)	15	38240529	701	15q15	Hs.631699
BLNK	B-cell linker	10	-97941452	29760	10q23.2-q23.33	Hs.444049
POU2AF1	POU domain, class 2, associating factor 1	11	-110728189	5450	11q23.1	Hs.128180
MS4A1	membrane-spanning 4-domains, subfamily A, member 1	11	59979857	931	11q12	Hs.438040
BCL2A1	BCL2-related protein A1	15	-78040289	597	15q24.3	Hs.227817
ZWINT	ZW10 interactor	10	-57787204	11130	10q21-q22	Hs.591363
IGF2BP3	insulin-like growth factor 2 mRNA binding protein 3	7	-23316353	10643	7p11	Hs.432616
APOBEC3B	apolipoprotein B mRNA editing enzyme, catalytic polypeptide-like 3B	22	37708350	9582	22q13.1-q13.2	Hs.226307
MTHFD2	methylenetetrahydrofolate dehydrogenase (NADP+ dependent) 2, methenyltetrahydrofolate cyclohydrolase	2	74279197	10797	2p13.1	Hs.469030
NUSAP1	nucleolar and spindle associated protein 1	15	39412360	51203	15q15.1	Hs.615092
RGS1	regulator of G-protein signalling 1	1	190811479	5996	1q31	Hs.75256
IGJ	immunoglobulin J polypeptide, linker protein for immunoglobulin alpha and mu polypeptides	4	-71740547	3512	4q21	Hs.643431
MLF1IP	MLF1 interacting protein	4	-185852231	79682	4q35.1	Hs.481307 Hs.575032
PAGE1	P antigen family, member 1 (prostate associated)	X	-49339009	8712	Xp11.23	Hs.128231
NXF3	nuclear RNA export factor 3	X	-102217407	56000	Xq22-q23	Hs.60386
EBI3	Epstein-Barr virus induced gene 3	19	4180539	10148	19p13.3	Hs.501452
DOCK2	dedicator of cytokinesis 2	5	168996870	1794	5q35.1	Hs.586174
HMGB2	high-mobility group box 2	4	-174489362	3148	4q31	Hs.434953
PLAC8	placenta-specific 8	4	-84230866	51316	4q21.22	Hs.546392
PSMD14	proteasome (prosome, macropain) 26S subunit, non-ATPase, 14	2	161873185	10213	2q24.2	Hs.567410
SOHLH2	spermatogenesis and oogenesis specific basic helix-loop-helix 2	13	-35640353	54937	13q13.3	Hs.124519

MAGEA1	melanoma antigen family A, 1 (directs expression of antigen MZ2-E)	X	-152134715	4100	Xq28	Hs.72879
PRC1	protein regulator of cytokinesis 1	15	-89310276	9055	15q26.1	Hs.567385
NCF4	neutrophil cytosolic factor 4, 40kDa	22	35586990	4689	22q13.1	Hs.474781
PCNA	proliferating cell nuclear antigen	20	-5043599	5111	20pter-p12	Hs.147433
HAT1	histone acetyltransferase 1	2	172487203	8520	2q31.2-q33.1	Hs.632532
SAMSN1	SAM domain, SH3 domain and nuclear localisation signals, 1	21	-14779419	64092	21q11	Hs.473341 Hs.570423
TARS	threonyl-tRNA synthetase	5	33476654	6897	5p13.2	Hs.481860
MMP7	matrix metallopeptidase 7 (matrilysin, uterine)	11	-101896449	4316	11q21-q22	Hs.2256
PAICS	phosphoribosylaminoimidazole carboxylase, phosphoribosylaminoimidazole succinocarboxamide synthetase	4	56996697	10606	4pter-q21	Hs.518774
NBR1	neighbor of BRCA1 gene 1	17	38576771, 38576036, 38576023	4077	17q21.31	Hs.277721 Hs.373818 Hs.546264
CEP55	centrosomal protein 55kDa	10	95246398	55165	10q23.33	Hs.14559
HCAP-G	NA	4	17421622	64151	4p15.33	Hs.446201 Hs.567567
COTL1	coactosin-like 1 (Dictyostelium)	16	-83156706	23406	16q24.1	Hs.289092
TYMS	thymidylate synthetase	18	647650	7298	18p11.32	Hs.592338
CKS2	CDC28 protein kinase regulatory subunit 2	9	91115932	1164	9q22	Hs.83758
EZH2	enhancer of zeste homolog 2 (Drosophila)	7	-148135407	2146	7q35-q36	Hs.444082
PBK	PDZ binding kinase	8	-27723058	55872	8p21.2	Hs.104741
SSBP1	single-stranded DNA binding protein 1	7	141084644	6742	7q34	Hs.490394
AURKB	aurora kinase B	17	-8048774	9212	17p13.1	Hs.442658
SWAP70	NA	11	9642203	23075	11p15	Hs.153026
FHOD3	formin homology 2 domain containing 3	18	32131699	80206	18q12	Hs.436636
RP6-213H19.1	NA	X	130985316, 130984925	51765	Xq26.2	Hs.444247
SC4MOL	sterol-C4-methyl oxidase-like	4	166468267	6307	4q32-q34	Hs.105269 Hs.593050
RACGAP1	Rac GTPase activating protein 1	12	-48669214	29127	12q13.13	Hs.505469
FLJ22028	NA	12	21481877	79912	12p12.1	Hs.46835
GINS2	GINS complex subunit 2 (Psf2 homolog)	16	-84268781	51659	16q24.1	Hs.433180
ASPM	asp (abnormal spindle)-like, microcephaly associated (Drosophila)	1	-195320076	259266	1q31	Hs.121028
PSCDBP	pleckstrin homology, Sec7 and coiled-coil domains, binding protein	2	-157979377	9595	2q11.2	Hs.270
DPCR1	diffuse panbronchiolitis critical region 1	6	31027537, 2367969, 2166477	135656	6p21.33	Hs.631993
HLA-DRA	major histocompatibility complex, class II, DR alpha	6	32515624	3122	6p21.3	Hs.520048
RUNX3	runt-related transcription factor 3	1	-25098589	864	1p36	Hs.170019
PSMA3	proteasome (prosome, macropain) subunit, alpha type, 3	14	57781345	5684	14q23	Hs.558799
TNFAIP8	tumor necrosis factor, alpha-induced protein 8	5	118719628	25816	5q23.1	Hs.271955
DBF4	DBF4 homolog (S. cerevisiae)	7	87343479	10926	7q21.3	Hs.485380
SLAMF1	signaling lymphocytic activation molecule family member 1	1	-158846513	6504	1q22-q23	Hs.523660
PTPN6	protein tyrosine phosphatase, non-receptor type 6	12	6930703, 6926000	5777	12p13	Hs.63489
DENND2D	DENN/MADD domain containing 2D	1	-111531323	79961	1p13.3	Hs.557850
VRK1	vaccinia related kinase 1	14	96333436	7443	14q32	Hs.422662
TNFRSF17	tumor necrosis factor receptor superfamily, member 17	16	11966464	608	16p13.1	Hs.2556

PRG1	proteoglycan 1, secretory granule	10	70517833	5552	10q22.1	Hs.1908
LBR	lamin B receptor	1	-223655827	3930	1q42.1	Hs.435166
NMI	N-myc (and STAT) interactor	2	-151835230	9111	2p24.3-q21.3	Hs.54483
RRM1	ribonucleotide reductase M1 polypeptide	11	4072586	6240	11p15.5	Hs.558393
TP53	tumor protein p53 (Li-Fraumeni syndrome)	17	-7512464	7157	17p13.1	Hs.408312
CD48	CD48 molecule	1	-158915161	962	1q21.3-q22	Hs.243564
PLOD2	procollagen-lysine, 2-oxoglutarate 5-dioxygenase 2	3	-147269917	5352	3q23-q24	Hs.477866
MTHFD1	methylenetetrahydrofolate dehydrogenase (NADP+ dependent) 1, methenyltetrahydrofolate cyclohydrolase, formyltetrahydrofolate synthetase	14	63924845	4522	14q24	Hs.632340
MAD2L1	MAD2 mitotic arrest deficient-like 1 (yeast)	4	-121200039	4085	4q27	Hs.591697
CENPM	centromere protein M	22	-40664694	79019	22q13.2	Hs.208912
UBD	ubiquitin D	6	-978728	10537	6p21.3	Hs.44532
SMC4	structural maintenance of chromosomes 4	3	161600117, 161600123, 161601040	10051	3q26.1	Hs.58992
VDAC1	voltage-dependent anion channel 1	5	-133335505	7416	5q31	Hs.519320
RAB9A	RAB9A, member RAS oncogene family	X	13617261	9367	Xp22.2	Hs.495704
CORO1A	coronin, actin binding protein, 1A	16	30102426	11151	16p11.2	Hs.415067
CD19	CD19 molecule	16	28850760	930	16p11.2	Hs.632193
TUBG1	tubulin, gamma 1	17	38015219	7283	17q21	Hs.279669
KIF11	kinesin family member 11	10	94342970	3832	10q24.1	Hs.8878
CD300A	CD300a molecule	17	69974116	11314	17q25.1	Hs.9688
IFI44L	interferon-induced protein 44-like	1	78858736	10964	1p31.1	Hs.389724
CR2	complement component (3d/Epstein Barr virus) receptor 2	1	205694292	1380	1q32	Hs.445757
LCK	lymphocyte-specific protein tyrosine kinase	1	32489518	3932	1p34.3	Hs.470627
RBM3	RNA binding motif (RNP1, RRM) protein 3	X	48317779	5935	Xp11.2	Hs.301404
MCM6	MCM6 minichromosome maintenance deficient 6 (MIS5 homolog, S. pombe) (S. cerevisiae)	2	-136313673	4175	2q21	Hs.444118
CD37	CD37 molecule	19	54530488	951	19q13.3	Hs.166556
MELK	maternal embryonic leucine zipper kinase	9	36562904	9833	9p13.2	Hs.184339
MAGEA10	melanoma antigen family A, 10	X	-151053564	4109	Xq28	Hs.18048
PSMB10	proteasome (prosome, macropain) subunit, beta type, 10	16	-66525907	5699	16q22.1	Hs.9661
CRYZ	crystallin, zeta (quinone reductase)	1	-74943771	1429	1p31-p22	Hs.83114
ANP32E	acidic (leucine-rich) nuclear phosphoprotein 32 family, member E	1	-148457478	81611	1q21.2	Hs.385913
GCH1	GTP cyclohydrolase 1 (dopa-responsive dystonia)	14	-54378475	2643	14q22.1-q22.2	Hs.86724
IRF7	interferon regulatory factor 7	11	-602555	3665	11p15.5	Hs.166120
HMMR	hyaluronan-mediated motility receptor (RHAMM)	5	162820240	3161	5q33.2-qter	Hs.72550
NUDT22	nudix (nucleoside diphosphate linked moiety X)-type motif 22	11	63750337	84304	11q13.1	Hs.172847
PCID1	PCI domain containing 1 (herpesvirus entry mediator)	11	32561966	10480	11p13	Hs.502244
C6orf79	chromosome 6 open reading frame 79	6	-13898999	63933	6p24.3-p23	Hs.214043
FCER2	Fc fragment of IgE, low affinity II, receptor for (CD23)	19	-7659662	2208	19p13.3	Hs.465778
PSIP1	PC4 and SFRS1 interacting protein 1	9	-30914708	11168	9p22.3	Hs.493516
NARG1	NMDA receptor regulated 1	4	140442125	80155	4q31.1	Hs.555985

GMNN	geminin, DNA replication inhibitor	6	24883142	51053	6p22.2	Hs.234896
TFDP1	transcription factor Dp-1	13	113287056	7027	13q34	Hs.79353
MCM2	MCM2 minichromosome maintenance deficient 2, mitotin (S. cerevisiae)	3	128799942	4171	3q21	Hs.477481
LRAP	NA	5	96241023	64167	5q15	Hs.591249
HLA-DQA1	major histocompatibility complex, class II, DQ alpha 1	6	32713160	3117	6p21.3	Hs.387679
MAGEA12	melanoma antigen family A, 12	X	-151649951	4111	Xq28	Hs.169246
IFI16	interferon, gamma-inducible protein 16	1	157246330	3428	1q22	Hs.380250
CCNB1IP1	cyclin B1 interacting protein 1	14	-39698736	57820	14q11.2	Hs.107003
TMEM97	transmembrane protein 97	17	23670247	27346	17q11.2	Hs.199695
KIF14	kinesin family member 14	1	-198787929	9928	1q32.1	Hs.3104
BLK	B lymphoid tyrosine kinase	8	11388929	640	8p23-p22	Hs.146591
MRPL35	mitochondrial ribosomal protein L35	2	86280090	51318	2p11.2	Hs.433439
EXOSC8	exosome component 8	13	36472916	11340	13q13.1	Hs.294041
HCLS1	hematopoietic cell-specific Lyn substrate 1	3	-122832936	3059	3q13	Hs.14601
C1QBP	complement component 1, q subcomponent binding protein	17	-5276822	708	17p13.3	Hs.555866
LRRFIP1	leucine rich repeat (in FLII) interacting protein 1	2	238265667	9208	2q37.3	Hs.471779
C5orf13	chromosome 5 open reading frame 13	5	-111092891	9315	5q22.1	Hs.36053 Hs.626968
CD79A	CD79a molecule, immunoglobulin-associated alpha	19	47073029	973	19q13.2	Hs.631567
DFNA5	deafness, autosomal dominant 5	7	-24704499	1687	7p15	Hs.520708
CPOX	coproporphyrinogen oxidase	3	-99780981	1371	3q12	Hs.476982
PSMC6	proteasome (prosome, macropain) 26S subunit, ATPase, 6	14	52243667	5706	14q22.1	Hs.156171 Hs.491779
CDC2	cell division cycle 2, G1 to S and G2 to M	10	62208241, 62209916	983	10q21.1	Hs.334562
CD58	CD58 molecule	1	-116858679	965	1p13	Hs.34341
DCK	deoxycytidine kinase	4	72078255	1633	4q13.3-q21.1	Hs.709
IL21R	interleukin 21 receptor	16	27346079, 27321223, 27321923	50615	16p11	Hs.210546
RNF138	ring finger protein 138	18	27925834, 27926644	51444	18q12.1	Hs.302408 Hs.501040
LYN	v-yes-1 Yamaguchi sarcoma viral related oncogene homolog	8	56954925	4067	8q13	Hs.491767 Hs.614775
KIAA0101	KIAA0101	15	-62444265	9768	15q22.31	Hs.81892

13.2 GO: Biological Process

GO ID	genes present in chip	genes present in tissues	p-value	GO term
GO:0007049	601	28	3.69E-10	cell cycle
GO:0007067	135	14	7.63E-10	mitosis
GO:0000087	137	14	9.29E-10	M phase of mitotic cell cycle
GO:0006260	140	13	1.25E-08	DNA replication
GO:0000279	173	14	1.96E-08	M phase
GO:0000278	204	15	2.16E-08	mitotic cell cycle
GO:0006952	751	28	5.25E-08	defense response
GO:0006955	684	26	1.21E-07	immune response
GO:0007051	21	6	1.33E-07	spindle organization and biogenesis
GO:0051301	142	12	1.35E-07	cell division
GO:0009607	857	28	8.23E-07	response to biotic stimulus
GO:0006261	65	7	1.28E-05	DNA-dependent DNA replication
GO:0048015	71	7	2.30E-05	phosphoinositide-mediated signaling
GO:0050896	1578	37	2.86E-05	response to stimulus
GO:0000226	52	6	3.67E-05	microtubule cytoskeleton organization and biogenesis
GO:0006959	141	9	5.20E-05	humoral immune response
GO:0043283	2240	45	0.000135689	biopolymer metabolism
GO:0006259	489	16	0.00026443	DNA metabolism
GO:0009613	458	15	0.000414066	response to pest, pathogen or parasite
GO:0007017	112	7	0.000415748	microtubule-based process
GO:0042771	3	2	0.000437466	DNA damage response, signal transduction by p53 class mediator resulting in induction of apoptosis
GO:0042531	3	2	0.000437466	positive regulation of tyrosine phosphorylation of STAT protein
GO:0046427	3	2	0.000437466	positive regulation of JAK-STAT cascade
GO:0046649	81	6	0.000440147	lymphocyte activation
GO:0043207	463	15	0.000464356	response to external biotic stimulus
GO:0007052	14	3	0.000580225	mitotic spindle organization and biogenesis
GO:0007582	7846	106	0.000698552	physiological process
GO:0000074	394	13	0.000961463	regulation of progression through cell cycle
GO:0006270	17	3	0.001055468	DNA replication initiation
GO:0045321	97	6	0.001145371	immune cell activation
GO:0009719	214	9	0.001164863	response to endogenous stimulus
GO:0001775	98	6	0.001208166	cell activation
GO:0019932	176	8	0.001340992	second-messenger-mediated signaling
GO:0009396	5	2	0.00143509	folic acid and derivative biosynthesis
GO:0016064	103	6	0.001562661	humoral defense mechanism (sensu Vertebrata)
GO:0000070	20	3	0.001723059	mitotic sister chromatid segregation
GO:0008283	473	14	0.001732726	cell proliferation
GO:0046483	44	4	0.001943532	heterocycle metabolism
GO:0000819	21	3	0.001992527	sister chromatid segregation
GO:0019735	77	5	0.002409027	antimicrobial humoral response (sensu Vertebrata)
GO:0019730	78	5	0.002549455	antimicrobial humoral response
GO:0009262	7	2	0.002965975	deoxyribonucleotide metabolism
GO:0042509	7	2	0.002965975	regulation of tyrosine phosphorylation of STAT protein

GO:0046425	7	2	0.002965975	regulation of JAK-STAT cascade
GO:0006760	8	2	0.003923247	folic acid and derivative metabolism
GO:0006950	881	20	0.004440062	response to stress
GO:0007259	28	3	0.004613766	JAK-STAT cascade
GO:0050731	9	2	0.00500418	positive regulation of peptidyl-tyrosine phosphorylation
GO:0030330	9	2	0.00500418	DNA damage response, signal transduction by p53 class mediator
GO:0006268	9	2	0.00500418	DNA unwinding
GO:0001934	10	2	0.006205673	positive regulation of protein amino acid phosphorylation
GO:0045764	10	2	0.006205673	positive regulation of amino acid metabolism
GO:0008630	10	2	0.006205673	DNA damage response, signal transduction resulting in induction of apoptosis
GO:0007260	10	2	0.006205673	tyrosine phosphorylation of STAT protein
GO:0007076	11	2	0.007524685	mitotic chromosome condensation
GO:0019884	11	2	0.007524685	antigen presentation, exogenous antigen
GO:0007059	34	3	0.007994279	chromosome segregation
GO:0050874	1531	29	0.008137842	organismal physiological process
GO:0019886	12	2	0.008958227	antigen processing, exogenous antigen via MHC class II
GO:0042327	12	2	0.008958227	positive regulation of phosphorylation
GO:0045937	12	2	0.008958227	positive regulation of phosphate metabolism
GO:0030261	12	2	0.008958227	chromosome condensation
GO:0006275	12	2	0.008958227	regulation of DNA replication
GO:0006511	105	5	0.009008358	ubiquitin-dependent protein catabolism
GO:0019941	105	5	0.009008358	modification-dependent protein catabolism
GO:0030333	36	3	0.009372637	antigen processing
GO:0030099	37	3	0.010110573	myeloid cell differentiation
GO:0007093	13	2	0.010503367	mitotic checkpoint
GO:0030218	13	2	0.010503367	erythrocyte differentiation
GO:0006974	200	7	0.010967625	response to DNA damage stimulus
GO:0007088	39	3	0.011685365	regulation of mitosis
GO:0006725	73	4	0.011910183	aromatic compound metabolism
GO:0007010	305	9	0.011928604	cytoskeleton organization and biogenesis
GO:0009725	14	2	0.012157224	response to hormone stimulus
GO:0046640	1	1	0.012178919	regulation of alpha-beta T cell proliferation
GO:0046641	1	1	0.012178919	positive regulation of alpha-beta T cell proliferation
GO:0046633	1	1	0.012178919	alpha-beta T cell proliferation
GO:0046634	1	1	0.012178919	regulation of alpha-beta T cell activation
GO:0046635	1	1	0.012178919	positive regulation of alpha-beta T cell activation
GO:0009076	1	1	0.012178919	histidine family amino acid biosynthesis
GO:0051095	1	1	0.012178919	regulation of helicase activity
GO:0051097	1	1	0.012178919	negative regulation of helicase activity
GO:0009162	1	1	0.012178919	deoxyribonucleoside monophosphate metabolism
GO:0009157	1	1	0.012178919	deoxyribonucleoside monophosphate biosynthesis
GO:0009176	1	1	0.012178919	pyrimidine deoxyribonucleoside monophosphate metabolism
GO:0009177	1	1	0.012178919	pyrimidine deoxyribonucleoside monophosphate biosynthesis
GO:0009221	1	1	0.012178919	pyrimidine deoxyribonucleotide biosynthesis
GO:0007108	1	1	0.012178919	cytokinesis, initiation of separation
GO:0000105	1	1	0.012178919	histidine biosynthesis
GO:0000212	1	1	0.012178919	meiotic spindle organization and biogenesis

GO:0046073	1	1	0.012178919	dTMP metabolism
GO:0051103	1	1	0.012178919	DNA ligation during DNA repair
GO:0044416	1	1	0.012178919	induction of host defense response
GO:0006288	1	1	0.012178919	base-excision repair, DNA ligation
GO:0006231	1	1	0.012178919	dTMP biosynthesis
GO:0000920	1	1	0.012178919	cell separation during cytokinesis
GO:0000912	1	1	0.012178919	cytokinesis, formation of actomyosin apparatus
GO:0000915	1	1	0.012178919	cytokinesis, contractile ring formation
GO:0046730	1	1	0.012178919	viral induction of host immune response
GO:0046731	1	1	0.012178919	passive viral induction of host immune response
GO:0006882	1	1	0.012178919	zinc ion homeostasis
GO:0051247	41	3	0.013393633	positive regulation of protein metabolism
GO:0050730	15	2	0.01391697	regulation of peptidyl-tyrosine phosphorylation
GO:0001932	16	2	0.01577983	regulation of protein amino acid phosphorylation
GO:0006473	16	2	0.01577983	protein amino acid acetylation
GO:0006284	16	2	0.01577983	base-excision repair
GO:0006521	17	2	0.017743078	regulation of amino acid metabolism
GO:0007242	879	18	0.019021006	intracellular signaling cascade
GO:0006468	444	11	0.019071083	protein amino acid phosphorylation
GO:0009123	18	2	0.01980404	nucleoside monophosphate metabolism
GO:0009124	18	2	0.01980404	nucleoside monophosphate biosynthesis
GO:0000910	18	2	0.01980404	cytokinesis
GO:0006996	693	15	0.020446688	organelle organization and biogenesis
GO:0030097	87	4	0.02141535	hemopoiesis
GO:0018108	19	2	0.021960089	peptidyl-tyrosine phosphorylation
GO:0048534	88	4	0.022230423	hemopoietic or lymphoid organ development
GO:0018193	50	3	0.022766466	peptidyl-amino acid modification
GO:0008629	20	2	0.024208648	induction of apoptosis by intracellular signals
GO:0046653	2	1	0.024210845	tetrahydrofolate metabolism
GO:0046631	2	1	0.024210845	alpha-beta T cell activation
GO:0046902	2	1	0.024210845	regulation of mitochondrial membrane permeability
GO:0048247	2	1	0.024210845	lymphocyte chemotaxis
GO:0045842	2	1	0.024210845	positive regulation of mitotic metaphase/anaphase transition
GO:0007079	2	1	0.024210845	mitotic chromosome movement towards spindle pole
GO:0000022	2	1	0.024210845	mitotic spindle elongation
GO:0050862	2	1	0.024210845	positive regulation of T cell receptor signaling pathway
GO:0009186	2	1	0.024210845	deoxyribonucleoside diphosphate metabolism
GO:0007405	2	1	0.024210845	neuroblast proliferation
GO:0009263	2	1	0.024210845	deoxyribonucleotide biosynthesis
GO:0051305	2	1	0.024210845	chromosome movement towards spindle pole
GO:0051231	2	1	0.024210845	spindle elongation
GO:0006475	2	1	0.024210845	internal protein amino acid acetylation
GO:0006435	2	1	0.024210845	threonyl-tRNA aminoacylation
GO:0008632	53	3	0.026510058	apoptotic program
GO:0050870	21	2	0.026547187	positive regulation of T cell activation
GO:0018212	21	2	0.026547187	peptidyl-tyrosine modification
GO:0051242	411	10	0.02787387	positive regulation of cellular physiological process

GO:0042770	22	2	0.028973223	DNA damage response, signal transduction
GO:0009117	143	5	0.030217779	nucleotide metabolism
GO:0042981	306	8	0.032924628	regulation of apoptosis
GO:0043067	308	8	0.034028625	regulation of programmed cell death
GO:0007018	59	3	0.034923947	microtubule-based movement
GO:0030705	59	3	0.034923947	cytoskeleton-dependent intracellular transport
GO:0043119	428	10	0.035411147	positive regulation of physiological process
GO:0050856	3	1	0.036097534	regulation of T cell receptor signaling pathway
GO:0050857	3	1	0.036097534	positive regulation of antigen receptor mediated signaling pathway
GO:0009129	3	1	0.036097534	pyrimidine nucleoside monophosphate metabolism
GO:0009113	3	1	0.036097534	purine base biosynthesis
GO:0009130	3	1	0.036097534	pyrimidine nucleoside monophosphate biosynthesis
GO:0009132	3	1	0.036097534	nucleoside diphosphate metabolism
GO:0050680	3	1	0.036097534	negative regulation of epithelial cell proliferation
GO:0046040	3	1	0.036097534	IMP metabolism
GO:0006474	3	1	0.036097534	N-terminal protein amino acid acetylation
GO:0006188	3	1	0.036097534	IMP biosynthesis
GO:0006189	3	1	0.036097534	'de novo' IMP biosynthesis
GO:0006266	3	1	0.036097534	DNA ligation
GO:0042325	25	2	0.036752176	regulation of phosphorylation
GO:0006220	26	2	0.039504285	pyrimidine nucleotide metabolism
GO:0006919	27	2	0.042332156	caspase activation
GO:0009605	963	18	0.042336466	response to external stimulus
GO:0019220	28	2	0.04523357	regulation of phosphate metabolism
GO:0051174	28	2	0.04523357	regulation of phosphorus metabolism
GO:0051251	28	2	0.04523357	positive regulation of lymphocyte activation
GO:0019048	4	1	0.047840725	virus-host interaction
GO:0008156	4	1	0.047840725	negative regulation of DNA replication
GO:0009086	4	1	0.047840725	methionine biosynthesis
GO:0050854	4	1	0.047840725	regulation of antigen receptor mediated signaling pathway
GO:0009219	4	1	0.047840725	pyrimidine deoxyribonucleotide metabolism
GO:0051282	4	1	0.047840725	regulation of sequestering of calcium ion
GO:0051283	4	1	0.047840725	negative regulation of sequestering of calcium ion
GO:0051209	4	1	0.047840725	release of sequestered calcium ion into cytoplasm
GO:0042541	4	1	0.047840725	hemoglobin biosynthesis
GO:0006144	4	1	0.047840725	purine base metabolism
GO:0018409	4	1	0.047840725	peptide or protein amino-terminal blocking
GO:0016043	842	16	0.048186682	cell organization and biogenesis
GO:0051052	29	2	0.048206354	regulation of DNA metabolism
GO:0050867	29	2	0.048206354	positive regulation of cell activation
GO:0042113	30	2	0.051248374	B cell activation
GO:0006730	30	2	0.051248374	one-carbon compound metabolism
GO:0016310	522	11	0.052551471	phosphorylation
GO:0043280	31	2	0.054357536	positive regulation of caspase activity
GO:0043281	31	2	0.054357536	regulation of caspase activity
GO:0050863	31	2	0.054357536	regulation of T cell activation
GO:0051345	32	2	0.057531788	positive regulation of hydrolase activity

GO:0006547	5	1	0.059442134	histidine metabolism
GO:0050000	5	1	0.059442134	chromosome localization
GO:0009075	5	1	0.059442134	histidine family amino acid metabolism
GO:0045840	5	1	0.059442134	positive regulation of mitosis
GO:0051053	5	1	0.059442134	negative regulation of DNA metabolism
GO:0008635	5	1	0.059442134	caspase activation via cytochrome c
GO:0050852	5	1	0.059442134	T cell receptor signaling pathway
GO:0051303	5	1	0.059442134	establishment of chromosome localization
GO:0007569	5	1	0.059442134	cell aging
GO:0051208	5	1	0.059442134	sequestering of calcium ion
GO:0046146	5	1	0.059442134	tetrahydrobiopterin metabolism
GO:0020027	5	1	0.059442134	hemoglobin metabolism
GO:0006729	5	1	0.059442134	tetrahydrobiopterin biosynthesis
GO:0008284	121	4	0.059976558	positive regulation of cell proliferation
GO:0006915	471	10	0.060601154	apoptosis
GO:0006281	174	5	0.061034154	DNA repair
GO:0012501	473	10	0.062000954	programmed cell death
GO:0006464	1087	19	0.065508197	protein modification
GO:0043412	1160	20	0.066559657	biopolymer modification
GO:0048522	482	10	0.068561401	positive regulation of cellular process
GO:0006520	181	5	0.069801282	amino acid metabolism
GO:0007089	6	1	0.070903458	traversing start control point of mitotic cell cycle
GO:0050851	6	1	0.070903458	antigen receptor-mediated signaling pathway
GO:0031032	6	1	0.070903458	actomyosin structure organization and biogenesis
GO:0000075	37	2	0.074310237	cell cycle checkpoint
GO:0007204	37	2	0.074310237	elevation of cytoplasmic calcium ion concentration
GO:0051480	37	2	0.074310237	cytoplasmic calcium ion homeostasis
GO:0008219	498	10	0.081293444	cell death
GO:0031365	7	1	0.082226371	N-terminal protein amino acid modification
GO:0006555	7	1	0.082226371	methionine metabolism
GO:0045787	7	1	0.082226371	positive regulation of progression through cell cycle
GO:0009067	7	1	0.082226371	aspartate family amino acid biosynthesis
GO:0050673	7	1	0.082226371	epithelial cell proliferation
GO:0050678	7	1	0.082226371	regulation of epithelial cell proliferation
GO:0006265	7	1	0.082226371	DNA topological change
GO:0016265	501	10	0.083834446	death
GO:0031325	136	4	0.084089893	positive regulation of cellular metabolism
GO:0051188	85	3	0.084881545	cofactor biosynthesis
GO:0009967	85	3	0.084881545	positive regulation of signal transduction
GO:0019882	41	2	0.088709227	antigen presentation
GO:0051249	41	2	0.088709227	regulation of lymphocyte activation
GO:0006793	641	12	0.089300102	phosphorus metabolism
GO:0006796	641	12	0.089300102	phosphate metabolism
GO:0006968	87	3	0.089553895	cellular defense response
GO:0006917	140	4	0.0912179	induction of apoptosis
GO:0012502	140	4	0.0912179	induction of programmed cell death
GO:0006558	8	1	0.093412532	L-phenylalanine metabolism

GO:0006559	8	1	0.093412532	L-phenylalanine catabolism
GO:0000097	8	1	0.093412532	sulfur amino acid biosynthesis
GO:0030071	8	1	0.093412532	regulation of mitotic metaphase/anaphase transition
GO:0051238	8	1	0.093412532	sequestering of metal ion
GO:0007568	8	1	0.093412532	aging
GO:0046112	8	1	0.093412532	nucleobase biosynthesis
GO:0045078	8	1	0.093412532	positive regulation of interferon-gamma biosynthesis
GO:0044267	2109	32	0.0957389	cellular protein metabolism
GO:0050865	43	2	0.096195276	regulation of cell activation
GO:0042110	45	2	0.103854702	T cell activation
GO:0007091	9	1	0.104463576	mitotic metaphase/anaphase transition
GO:0030101	9	1	0.104463576	natural killer cell activation
GO:0019538	2129	32	0.105725019	protein metabolism
GO:0009893	149	4	0.108271804	positive regulation of metabolism
GO:0019438	10	1	0.115381121	aromatic compound biosynthesis
GO:0009074	10	1	0.115381121	aromatic amino acid family catabolism
GO:0051262	10	1	0.115381121	protein tetramerization
GO:0042559	10	1	0.115381121	pteridine and derivative biosynthesis
GO:0006752	48	2	0.115641509	group transfer coenzyme metabolism
GO:0043065	155	4	0.120385878	positive regulation of apoptosis
GO:0009611	341	7	0.12191735	response to wounding
GO:0043068	156	4	0.122460219	positive regulation of programmed cell death
GO:0006519	216	5	0.123319549	amino acid and derivative metabolism
GO:0007006	11	1	0.126166764	mitochondrial membrane organization and biogenesis
GO:0009167	11	1	0.126166764	purine ribonucleoside monophosphate metabolism
GO:0009168	11	1	0.126166764	purine ribonucleoside monophosphate biosynthesis
GO:0009126	11	1	0.126166764	purine nucleoside monophosphate metabolism
GO:0009127	11	1	0.126166764	purine nucleoside monophosphate biosynthesis
GO:0007193	11	1	0.126166764	G-protein signaling, adenylate cyclase inhibiting pathway
GO:0042558	11	1	0.126166764	pteridine and derivative metabolism
GO:0006783	11	1	0.126166764	heme biosynthesis
GO:0045072	11	1	0.126166764	regulation of interferon-gamma biosynthesis
GO:0006139	2328	34	0.130262137	nucleobase, nucleoside, nucleotide and nucleic acid metabolism
GO:0009165	103	3	0.130512129	nucleotide biosynthesis
GO:0042095	12	1	0.136822086	interferon-gamma biosynthesis
GO:0009112	12	1	0.136822086	nucleobase metabolism
GO:0042102	12	1	0.136822086	positive regulation of T cell proliferation
GO:0019439	13	1	0.147348646	aromatic compound catabolism
GO:0006779	13	1	0.147348646	porphyrin biosynthesis
GO:0030595	13	1	0.147348646	immune cell chemotaxis
GO:0019752	359	7	0.14759471	carboxylic acid metabolism
GO:0050778	56	2	0.1485423	positive regulation of immune response
GO:0048518	565	10	0.14955164	positive regulation of biological process
GO:0006082	361	7	0.150598095	organic acid metabolism
GO:0006916	112	3	0.15597696	anti-apoptosis
GO:0009615	58	2	0.157043132	response to virus
GO:0050900	14	1	0.157747986	immune cell migration

GO:0030224	14	1	0.157747986	monocyte differentiation
GO:0051240	59	2	0.161328015	positive regulation of organismal physiological process
GO:0051336	60	2	0.165634314	regulation of hydrolase activity
GO:0006839	15	1	0.16802163	mitochondrial transport
GO:0009066	15	1	0.16802163	aspartate family amino acid metabolism
GO:0007020	15	1	0.16802163	microtubule nucleation
GO:0042168	15	1	0.16802163	heme metabolism
GO:0042129	15	1	0.16802163	regulation of T cell proliferation
GO:0006164	61	2	0.169960885	purine nucleotide biosynthesis
GO:0050875	7018	90	0.177271117	cellular physiological process
GO:0006809	16	1	0.178171083	nitric oxide biosynthesis
GO:0046209	16	1	0.178171083	nitric oxide metabolism
GO:0006163	63	2	0.178670397	purine nucleotide metabolism
GO:0006508	451	8	0.183593723	proteolysis and peptidolysis
GO:0044257	451	8	0.183593723	cellular protein catabolism
GO:0042098	17	1	0.188197834	T cell proliferation
GO:0009161	17	1	0.188197834	ribonucleoside monophosphate metabolism
GO:0009156	17	1	0.188197834	ribonucleoside monophosphate biosynthesis
GO:0030574	17	1	0.188197834	collagen catabolism
GO:0006954	185	4	0.18867163	inflammatory response
GO:0009072	18	1	0.198103351	aromatic amino acid family metabolism
GO:0007005	18	1	0.198103351	mitochondrion organization and biogenesis
GO:0000080	18	1	0.198103351	G1 phase of mitotic cell cycle
GO:0042133	18	1	0.198103351	neurotransmitter metabolism
GO:0050671	18	1	0.198103351	positive regulation of lymphocyte proliferation
GO:0030217	18	1	0.198103351	T cell differentiation
GO:0006221	18	1	0.198103351	pyrimidine nucleotide biosynthesis
GO:0006778	18	1	0.198103351	porphyrin metabolism
GO:0006874	68	2	0.200723553	calcium ion homeostasis
GO:0030163	463	8	0.202183069	protein catabolism
GO:0044237	5026	66	0.204164943	cellular metabolism
GO:0043066	128	3	0.204542192	negative regulation of apoptosis
GO:0043069	129	3	0.207690498	negative regulation of programmed cell death
GO:0044249	763	12	0.217135421	cellular biosynthesis
GO:0044260	2298	32	0.21747358	cellular macromolecule metabolism
GO:0046916	20	1	0.217556479	transition metal ion homeostasis
GO:0000096	20	1	0.217556479	sulfur amino acid metabolism
GO:0042493	20	1	0.217556479	response to drug
GO:0044403	20	1	0.217556479	symbiosis, mutualism through parasitism
GO:0044404	20	1	0.217556479	symbiotic interaction between host and other organism
GO:0046148	20	1	0.217556479	pigment biosynthesis
GO:0006289	20	1	0.217556479	nucleotide-excision repair
GO:0009108	72	2	0.218587909	coenzyme biosynthesis
GO:0043285	474	8	0.219876389	biopolymer catabolism
GO:0042127	265	5	0.221300134	regulation of cell proliferation
GO:0051318	21	1	0.227106944	G1 phase
GO:0000270	21	1	0.227106944	peptidoglycan metabolism

GO:0009308	269	5	0.230205596	amine metabolism
GO:0051244	2319	32	0.234647986	regulation of cellular physiological process
GO:0019885	22	1	0.236541883	antigen processing, endogenous antigen via MHC class I
GO:0016044	22	1	0.236541883	membrane organization and biogenesis
GO:0050670	22	1	0.236541883	regulation of lymphocyte proliferation
GO:0050776	77	2	0.241100216	regulation of immune response
GO:0050794	2488	34	0.243524438	regulation of cellular process
GO:0009058	859	13	0.24487663	biosynthesis
GO:0006958	23	1	0.245862681	complement activation, classical pathway
GO:0042108	23	1	0.245862681	positive regulation of cytokine biosynthesis
GO:0051329	79	2	0.250140416	interphase of mitotic cell cycle
GO:0051186	143	3	0.252781364	cofactor metabolism
GO:0042088	24	1	0.255070707	T-helper 1 type immune response
GO:0042440	24	1	0.255070707	pigment metabolism
GO:0051259	24	1	0.255070707	protein oligomerization
GO:0044272	24	1	0.255070707	sulfur compound biosynthesis
GO:0045786	145	3	0.259347277	negative regulation of progression through cell cycle
GO:0006366	426	7	0.261977591	transcription from RNA polymerase II promoter
GO:0051325	82	2	0.26371938	interphase
GO:0045792	25	1	0.264167313	negative regulation of cell size
GO:0045727	25	1	0.264167313	positive regulation of protein biosynthesis
GO:0042087	25	1	0.264167313	cell-mediated immune response
GO:0051235	25	1	0.264167313	maintenance of localization
GO:0030308	25	1	0.264167313	negative regulation of cell growth
GO:0031328	26	1	0.273153834	positive regulation of cellular biosynthesis
GO:0019748	26	1	0.273153834	secondary metabolism
GO:0044419	26	1	0.273153834	interaction between organisms
GO:0006807	291	5	0.280871875	nitrogen compound metabolism
GO:0016066	27	1	0.282031591	cellular defense response (sensu Vertebrata)
GO:0016126	27	1	0.282031591	sterol biosynthesis
GO:0030005	87	2	0.286354046	di-, tri-valent inorganic cation homeostasis
GO:0040007	154	3	0.289147809	growth
GO:0046651	28	1	0.290801888	lymphocyte proliferation
GO:0008217	28	1	0.290801888	regulation of blood pressure
GO:0001819	29	1	0.299466014	positive regulation of cytokine production
GO:0045926	29	1	0.299466014	negative regulation of growth
GO:0007265	29	1	0.299466014	Ras protein signal transduction
GO:0009966	229	4	0.304661079	regulation of signal transduction
GO:0045934	159	3	0.305827814	negative regulation of nucleobase, nucleoside, nucleotide and nucleic acid metabolism
GO:0006956	30	1	0.308025243	complement activation
GO:0009891	30	1	0.308025243	positive regulation of biosynthesis
GO:0043170	2405	32	0.311346304	macromolecule metabolism
GO:0050791	2407	32	0.313236971	regulation of physiological process
GO:0006875	93	2	0.313422649	metal ion homeostasis
GO:0006367	31	1	0.316480833	transcription initiation from RNA polymerase II promoter
GO:0001558	94	2	0.317916097	regulation of cell growth
GO:0051276	236	4	0.324012345	chromosome organization and biogenesis

GO:0043038	33	1	0.333086057	amino acid activation
GO:0043039	33	1	0.333086057	tRNA aminoacylation
GO:0042035	33	1	0.333086057	regulation of cytokine biosynthesis
GO:0030155	33	1	0.333086057	regulation of cell adhesion
GO:0006418	33	1	0.333086057	tRNA aminoacylation for protein translation
GO:0006357	240	4	0.335114474	regulation of transcription from RNA polymerase II promoter
GO:0030003	98	2	0.335819294	cation homeostasis
GO:0007243	242	4	0.34067363	protein kinase cascade
GO:0008652	34	1	0.341238133	amino acid biosynthesis
GO:0030098	34	1	0.341238133	lymphocyte differentiation
GO:0006935	101	2	0.34915918	chemotaxis
GO:0042330	101	2	0.34915918	taxis
GO:0016032	35	1	0.349291457	viral life cycle
GO:0051246	174	3	0.356002272	regulation of protein metabolism
GO:0042089	36	1	0.357247213	cytokine biosynthesis
GO:0000079	36	1	0.357247213	regulation of cyclin dependent protein kinase activity
GO:0006406	36	1	0.357247213	mRNA-nucleus export
GO:0006873	103	2	0.358004059	cell ion homeostasis
GO:0040008	104	2	0.362410731	regulation of growth
GO:0051028	37	1	0.365106573	mRNA transport
GO:0042107	37	1	0.365106573	cytokine metabolism
GO:0051258	37	1	0.365106573	protein polymerization
GO:0006487	37	1	0.365106573	protein amino acid N-linked glycosylation
GO:0006400	37	1	0.365106573	tRNA modification
GO:0044238	4820	61	0.365577668	primary metabolism
GO:0043085	107	2	0.375562981	positive regulation of enzyme activity
GO:0006412	407	6	0.376155744	protein biosynthesis
GO:0048523	640	9	0.377412562	negative regulation of cellular process
GO:0044265	563	8	0.379004001	cellular macromolecule catabolism
GO:0006118	256	4	0.379629657	electron transport
GO:0001817	39	1	0.380540722	regulation of cytokine production
GO:0030154	409	6	0.38055502	cell differentiation
GO:0031324	183	3	0.385946901	negative regulation of cellular metabolism
GO:0042221	183	3	0.385946901	response to chemical substance
GO:0050801	111	2	0.392927554	ion homeostasis
GO:0008152	5262	66	0.393531047	metabolism
GO:0001505	41	1	0.395602999	regulation of neurotransmitter levels
GO:0006405	41	1	0.395602999	RNA-nucleus export
GO:0009057	575	8	0.401616527	macromolecule catabolism
GO:0001816	42	1	0.402997467	cytokine production
GO:0009063	42	1	0.402997467	amino acid catabolism
GO:0050657	42	1	0.402997467	nucleic acid transport
GO:0050658	42	1	0.402997467	RNA transport
GO:0051236	42	1	0.402997467	establishment of RNA localization
GO:0006403	43	1	0.410302282	RNA localization
GO:0051243	583	8	0.416713485	negative regulation of cellular physiological process
GO:0000086	44	1	0.417518518	G2/M transition of mitotic cell cycle

GO:0007126	44	1	0.417518518	meiosis
GO:0051321	44	1	0.417518518	meiotic cell cycle
GO:0051327	44	1	0.417518518	M phase of meiotic cell cycle
GO:0006323	193	3	0.418844282	DNA packaging
GO:0009310	45	1	0.424647241	amine catabolism
GO:0051168	45	1	0.424647241	nuclear export
GO:0043086	46	1	0.431689502	negative regulation of enzyme activity
GO:0050789	2689	34	0.432143985	regulation of biological process
GO:0006732	121	2	0.435353177	coenzyme metabolism
GO:0006352	47	1	0.438646341	transcription initiation
GO:0044270	47	1	0.438646341	nitrogen compound catabolism
GO:0019725	122	2	0.439510626	cell homeostasis
GO:0008361	123	2	0.443651625	regulation of cell size
GO:0016049	123	2	0.443651625	cell growth
GO:0009451	48	1	0.445518785	RNA modification
GO:0006334	48	1	0.445518785	nucleosome assembly
GO:0006790	48	1	0.445518785	sulfur metabolism
GO:0015931	49	1	0.452307847	nucleobase, nucleoside, nucleotide and nucleic acid transport
GO:0051093	49	1	0.452307847	negative regulation of development
GO:0009309	49	1	0.452307847	amine biosynthesis
GO:0044271	49	1	0.452307847	nitrogen compound biosynthesis
GO:0043118	602	8	0.452497955	negative regulation of physiological process
GO:0030036	126	2	0.455973778	actin cytoskeleton organization and biogenesis
GO:0006928	205	3	0.457550429	cell motility
GO:0040011	205	3	0.457550429	locomotion
GO:0009968	50	1	0.45901453	negative regulation of signal transduction
GO:0048519	688	9	0.462531377	negative regulation of biological process
GO:0007626	209	3	0.470221472	locomotory behavior
GO:0000082	53	1	0.478650154	G1/S transition of mitotic cell cycle
GO:0045087	53	1	0.478650154	innate immune response
GO:0044248	700	9	0.483651191	cellular catabolism
GO:0051239	133	2	0.484112586	regulation of organismal physiological process
GO:0050790	214	3	0.485876011	regulation of enzyme activity
GO:0009892	217	3	0.495164533	negative regulation of metabolism
GO:0031497	56	1	0.497579329	chromatin assembly
GO:0009059	464	6	0.500307062	macromolecule biosynthesis
GO:0007001	221	3	0.507421293	chromosome organization and biogenesis (sensu Eukaryota)
GO:0030029	139	2	0.507514751	actin filament-based process
GO:0009152	58	1	0.509818784	purine ribonucleotide biosynthesis
GO:0007188	59	1	0.515827245	G-protein signaling, coupled to cAMP nucleotide second messenger
GO:0016070	391	5	0.520711722	RNA metabolism
GO:0006694	60	1	0.521762719	steroid biosynthesis
GO:0009150	60	1	0.521762719	purine ribonucleotide metabolism
GO:0019933	60	1	0.521762719	cAMP-mediated signaling
GO:0007050	61	1	0.527626083	cell cycle arrest
GO:0000122	61	1	0.527626083	negative regulation of transcription from RNA polymerase II promoter
GO:0006399	62	1	0.533418205	tRNA metabolism

GO:0006364	64	1	0.544792147	rRNA processing
GO:0009628	318	4	0.545519532	response to abiotic stimulus
GO:0031323	1566	19	0.547056028	regulation of cellular metabolism
GO:0001525	65	1	0.550375651	angiogenesis
GO:0009260	65	1	0.550375651	ribonucleotide biosynthesis
GO:0009790	65	1	0.550375651	embryonic development
GO:0016481	151	2	0.552225665	negative regulation of transcription
GO:0016072	66	1	0.555891284	rRNA metabolism
GO:0016125	66	1	0.555891284	sterol metabolism
GO:0006396	326	4	0.565375081	RNA processing
GO:0001568	68	1	0.566722195	blood vessel development
GO:0048514	68	1	0.566722195	blood vessel morphogenesis
GO:0009259	68	1	0.566722195	ribonucleotide metabolism
GO:0001944	69	1	0.572039081	vasculature development
GO:0042592	157	2	0.573500016	homeostasis
GO:0009056	756	9	0.579076707	catabolism
GO:0006512	255	3	0.604922009	ubiquitin cycle
GO:0050793	168	2	0.610593861	regulation of development
GO:0045893	77	1	0.612310725	positive regulation of transcription, DNA-dependent
GO:0019219	1458	17	0.618448274	regulation of nucleobase, nucleoside, nucleotide and nucleic acid metabolism
GO:0007046	79	1	0.621777224	ribosome biogenesis
GO:0019222	1629	19	0.621934198	regulation of metabolism
GO:0042254	80	1	0.626424214	ribosome biogenesis and assembly
GO:0007610	269	3	0.641238744	behavior
GO:0046907	448	5	0.642853859	intracellular transport
GO:0016477	85	1	0.648823969	cell migration
GO:0050954	86	1	0.653141546	sensory perception of mechanical stimulus
GO:0050982	86	1	0.653141546	detection of mechanical stimulus
GO:0007605	86	1	0.653141546	perception of sound
GO:0000902	183	2	0.657189493	cellular morphogenesis
GO:0006351	1407	16	0.658319308	transcription, DNA-dependent
GO:0006325	186	2	0.665962329	establishment and/or maintenance of chromatin architecture
GO:0006333	91	1	0.673952927	chromatin assembly or disassembly
GO:0000004	465	5	0.675391258	biological process unknown
GO:0006486	92	1	0.677964243	protein amino acid glycosylation
GO:0043413	93	1	0.681926652	biopolymer glycosylation
GO:0007187	94	1	0.685840744	G-protein signaling, coupled to cyclic nucleotide second messenger
GO:0006461	194	2	0.688482644	protein complex assembly
GO:0007028	96	1	0.693526306	cytoplasm organization and biogenesis
GO:0009101	96	1	0.693526306	glycoprotein biosynthesis
GO:0051169	96	1	0.693526306	nuclear transport
GO:0045892	97	1	0.697298923	negative regulation of transcription, DNA-dependent
GO:0019935	98	1	0.701025518	cyclic-nucleotide-mediated signaling
GO:0006355	1359	15	0.701380092	regulation of transcription, DNA-dependent
GO:0006470	99	1	0.704706645	protein amino acid dephosphorylation
GO:0045941	100	1	0.708342856	positive regulation of transcription
GO:0006913	102	1	0.715482692	nucleocytoplasmic transport

GO:0008015	103	1	0.718987384	circulation
GO:0048232	105	1	0.725868937	male gamete generation
GO:0045935	105	1	0.725868937	positive regulation of nucleobase, nucleoside, nucleotide and nucleic acid metabolism
GO:0007283	105	1	0.725868937	spermatogenesis
GO:0006417	107	1	0.732583466	regulation of protein biosynthesis
GO:0009582	109	1	0.73913499	detection of abiotic stimulus
GO:0007165	2168	24	0.739218169	signal transduction
GO:0016311	110	1	0.742350853	dephosphorylation
GO:0031326	111	1	0.745527429	regulation of cellular biosynthesis
GO:0009100	111	1	0.745527429	glycoprotein metabolism
GO:0001501	113	1	0.751764612	skeletal development
GO:0006350	1499	16	0.756925096	transcription
GO:0006897	115	1	0.757850276	endocytosis
GO:0045859	116	1	0.760837432	regulation of protein kinase activity
GO:0009889	116	1	0.760837432	regulation of biosynthesis
GO:0051338	117	1	0.76378807	regulation of transferase activity
GO:0007264	227	2	0.768593065	small GTPase mediated signal transduction
GO:0045449	1433	15	0.777395503	regulation of transcription
GO:0043037	122	1	0.778008776	translation
GO:0006631	124	1	0.78345651	fatty acid metabolism
GO:0007276	125	1	0.786130487	gametogenesis
GO:0009987	8200	98	0.789008299	cellular process
GO:0006820	128	1	0.793957661	anion transport
GO:0006091	446	4	0.799329637	generation of precursor metabolites and energy
GO:0008202	132	1	0.803953972	steroid metabolism
GO:0008285	139	1	0.82030446	negative regulation of cell proliferation
GO:0007154	2730	29	0.839381675	cell communication
GO:0019953	149	1	0.841344307	sexual reproduction
GO:0050953	149	1	0.841344307	sensory perception of light
GO:0007601	149	1	0.841344307	visual perception
GO:0007600	287	2	0.869447052	sensory perception
GO:0016337	172	1	0.880921035	cell-cell adhesion
GO:0008610	175	1	0.885301908	lipid biosynthesis
GO:0006397	180	1	0.892251202	mRNA processing
GO:0009581	313	2	0.899182185	detection of external stimulus
GO:0007268	192	1	0.907273383	synaptic transmission
GO:0019226	198	1	0.913986425	transmission of nerve impulse
GO:0048513	872	7	0.916711701	organ development
GO:0016071	205	1	0.921211685	mRNA metabolism
GO:0000003	208	1	0.924120981	reproduction
GO:0006066	213	1	0.928735085	alcohol metabolism
GO:0050877	475	3	0.933996992	neurophysiological process
GO:0007186	491	3	0.943071482	G-protein coupled receptor protein signaling pathway
GO:0007166	952	7	0.952869896	cell surface receptor linked signal transduction
GO:0007155	518	3	0.955829918	cell adhesion
GO:0044262	265	1	0.962966646	cellular carbohydrate metabolism
GO:0016192	296	1	0.974978784	vesicle-mediated transport

GO:0051179	1857	15	0.977435001	localization
GO:0007267	465	2	0.979714227	cell-cell signaling
GO:0051234	1841	14	0.987034203	establishment of localization
GO:0007275	1543	11	0.987359468	development
GO:0005975	355	1	0.988182334	carbohydrate metabolism
GO:0009653	1051	6	0.991445266	morphogenesis
GO:0006810	1802	13	0.991663686	transport
GO:0044255	383	1	0.991736862	cellular lipid metabolism
GO:0007399	385	1	0.991945722	neurogenesis
GO:0015031	439	1	0.995974248	protein transport
GO:0045184	459	1	0.996889606	establishment of protein localization
GO:0006629	469	1	0.997266614	lipid metabolism
GO:0008104	474	1	0.997437765	protein localization
GO:0009887	807	3	0.997749699	organogenesis
GO:0006811	515	1	0.998494357	ion transport
GO:0008150	9032	110	1	biological_process

13.3 GO: Molecular Function

GO ID	genes present in chip	genes present in tissues	p-value	GO term
GO:0019238	4	3	5.59664365815989e-06	cyclohydrolase activity
GO:0005524	970	25	6.17290294510361e-05	ATP binding
GO:0016725	2	2	0.000126959517316527	oxidoreductase activity, acting on CH2 groups
GO:0016728	2	2	0.000126959517316527	oxidoreductase activity, acting on CH2 groups, disulfide as acceptor
GO:0004488	2	2	0.000126959517316527	methylenetetrahydrofolate dehydrogenase (NADP+) activity
GO:0004477	2	2	0.000126959517316527	methenyltetrahydrofolate cyclohydrolase activity
GO:0004748	2	2	0.000126959517316527	ribonucleoside-diphosphate reductase activity
GO:0030554	1028	25	0.000157867163001813	adenyl nucleotide binding
GO:0004486	3	2	0.000378056933575421	methylenetetrahydrofolate dehydrogenase activity
GO:0000166	1432	30	0.000433360835292632	nucleotide binding
GO:0017076	1248	27	0.000554765641047983	purine nucleotide binding
GO:0005515	3709	58	0.00118183437930361	protein binding
GO:0003690	20	3	0.0013976470612838	double-stranded DNA binding
GO:0016814	21	3	0.00161723553138566	hydrolase activity, acting on carbon-nitrogen (but not peptide) bonds, in cyclic amidines
GO:0003697	28	3	0.00376113071318732	single-stranded DNA binding
GO:0005488	7228	93	0.00569812658186156	binding
GO:0016646	11	2	0.00653227334248909	oxidoreductase activity, acting on the CH-NH group of donors, NAD or NADP as acceptor
GO:0008094	34	3	0.00654114117820727	DNA-dependent ATPase activity
GO:0045012	12	2	0.00778110749341026	MHC class II receptor activity
GO:0001532	1	1	0.0113195681794357	interleukin-21 receptor activity
GO:0004638	1	1	0.0113195681794357	phosphoribosylaminoimidazole carboxylase activity
GO:0004639	1	1	0.0113195681794357	phosphoribosylaminoimidazolesuccinocarboxamide synthase activity
GO:0004903	1	1	0.0113195681794357	growth hormone receptor activity
GO:0042083	1	1	0.0113195681794357	5,10-methylenetetrahydrofolate-dependent methyltransferase activity
GO:0051397	1	1	0.0113195681794357	N-terminal basic amino acid aminopeptidase activity
GO:0051398	1	1	0.0113195681794357	N-terminal lysine aminopeptidase activity
GO:0051399	1	1	0.0113195681794357	N-terminal arginine aminopeptidase activity
GO:0003933	1	1	0.0113195681794357	GTP cyclohydrolase activity
GO:0003934	1	1	0.0113195681794357	GTP cyclohydrolase I activity
GO:0019977	1	1	0.0113195681794357	interleukin-21 binding
GO:0000254	1	1	0.0113195681794357	C-4 methylsterol oxidase activity
GO:0000739	1	1	0.0113195681794357	DNA strand annealing activity
GO:0030337	1	1	0.0113195681794357	DNA polymerase processivity factor activity
GO:0042610	1	1	0.0113195681794357	CD8 receptor binding
GO:0016538	1	1	0.0113195681794357	cyclin-dependent protein kinase regulator activity
GO:0004329	1	1	0.0113195681794357	formate-tetrahydrofolate ligase activity
GO:0004137	1	1	0.0113195681794357	deoxycytidine kinase activity
GO:0004235	1	1	0.0113195681794357	matrilysin activity
GO:0015283	1	1	0.0113195681794357	apoptogenic cytochrome c release channel activity
GO:0004799	1	1	0.0113195681794357	thymidylate synthase activity
GO:0004896	48	3	0.0168708713064317	hematopoietin/interferon-class (D200-domain) cytokine receptor activity
GO:0016645	18	2	0.0172593020841907	oxidoreductase activity, acting on the CH-NH group of donors

GO:0004298	18	2	0.0172593020841907	threonine endopeptidase activity
GO:0043014	2	1	0.0225121768415560	alpha-tubulin binding
GO:0004829	2	1	0.0225121768415560	threonine-tRNA ligase activity
GO:0030676	2	1	0.0225121768415560	Rac guanyl-nucleotide exchange factor activity
GO:0045309	2	1	0.0225121768415560	protein phosphorylated amino acid binding
GO:0003960	2	1	0.0225121768415560	NADPH:quinone reductase activity
GO:0051117	2	1	0.0225121768415560	ATPase binding
GO:0042609	2	1	0.0225121768415560	CD4 receptor binding
GO:0004487	2	1	0.0225121768415560	methylenetetrahydrofolate dehydrogenase (NAD+) activity
GO:0015482	2	1	0.0225121768415560	voltage-gated anion channel porin activity
GO:0004109	2	1	0.0225121768415560	coproporphyrinogen oxidase activity
GO:0015266	2	1	0.0225121768415560	protein channel activity
GO:0001784	2	1	0.0225121768415560	phosphotyrosine binding
GO:0004672	447	10	0.0297251704201798	protein kinase activity
GO:0042802	158	5	0.0333884481924966	protein self binding
GO:0004875	3	1	0.0335792367955461	complement receptor activity
GO:0008308	3	1	0.0335792367955461	voltage-gated ion-selective channel activity
GO:0008475	3	1	0.0335792367955461	procollagen-lysine 5-dioxygenase activity
GO:0005128	3	1	0.0335792367955461	erythropoietin receptor binding
GO:0001948	3	1	0.0335792367955461	glycoprotein binding
GO:0051219	3	1	0.0335792367955461	phosphoprotein binding
GO:0042608	3	1	0.0335792367955461	T cell receptor binding
GO:0019955	65	3	0.0371455247240196	cytokine binding
GO:0016773	532	11	0.0379416905231758	phosphotransferase activity, alcohol group as acceptor
GO:0043015	4	1	0.0445221433195637	gamma-tubulin binding
GO:0019136	4	1	0.0445221433195637	deoxynucleoside kinase activity
GO:0042301	4	1	0.0445221433195637	phosphate binding
GO:0003918	4	1	0.0445221433195637	DNA topoisomerase (ATP-hydrolyzing) activity
GO:0003823	30	2	0.0449571616444154	antigen binding
GO:0019965	30	2	0.0449571616444154	interleukin binding
GO:0016810	72	3	0.0479511132349158	hydrolase activity, acting on carbon-nitrogen (but not peptide) bonds
GO:0001848	5	1	0.0553422763300998	complement binding
GO:0008301	5	1	0.0553422763300998	DNA bending activity
GO:0048487	5	1	0.0553422763300998	beta-tubulin binding
GO:0019863	5	1	0.0553422763300998	IgE binding
GO:0005521	5	1	0.0553422763300998	lamin binding
GO:0047485	5	1	0.0553422763300998	protein N-terminus binding
GO:0005057	132	4	0.0625329643620612	receptor signaling protein activity
GO:0043022	6	1	0.0660410005495154	ribosome binding
GO:0003916	6	1	0.0660410005495154	DNA topoisomerase activity
GO:0016407	39	2	0.071760631481759	acetyltransferase activity
GO:0004674	326	7	0.0762785853028374	protein serine/threonine kinase activity
GO:0016301	609	11	0.0830151436987704	kinase activity
GO:0016634	8	1	0.0870796065263337	oxidoreductase activity, acting on the CH-CH group of donors, oxygen as acceptor
GO:0003777	45	2	0.0918350754603114	microtubule motor activity
GO:0005506	212	5	0.0924216089388378	iron ion binding
GO:0043021	9	1	0.0974221432403951	ribonucleoprotein binding

GO:0004693	9	1	0.0974221432403951	cyclin-dependent protein kinase activity
GO:0016772	708	12	0.103319413793277	transferase activity, transferring phosphorus-containing groups
GO:0000175	10	1	0.107648581399233	3'-5'-exoribonuclease activity
GO:0004716	10	1	0.107648581399233	receptor signaling protein tyrosine kinase activity
GO:0004713	161	4	0.109590332335398	protein-tyrosine kinase activity
GO:0004532	11	1	0.117760212204907	exoribonuclease activity
GO:0019206	11	1	0.117760212204907	nucleoside kinase activity
GO:0016896	11	1	0.117760212204907	exoribonuclease activity, producing 5'-phosphomonoesters
GO:0016491	509	9	0.122060432206819	oxidoreductase activity
GO:0046983	170	4	0.126716461829768	protein dimerization activity
GO:0031418	12	1	0.127758312633225	L-ascorbic acid binding
GO:0019865	12	1	0.127758312633225	immunoglobulin binding
GO:0016493	12	1	0.127758312633225	C-C chemokine receptor activity
GO:0019957	12	1	0.127758312633225	C-C chemokine binding
GO:0016284	12	1	0.127758312633225	alanine aminopeptidase activity
GO:0004179	12	1	0.127758312633225	membrane alanyl aminopeptidase activity
GO:0015288	13	1	0.137644145589004	porin activity
GO:0016564	59	2	0.143688938389730	transcriptional repressor activity
GO:0005540	14	1	0.147418960059658	hyaluronic acid binding
GO:0042169	14	1	0.147418960059658	SH2 domain binding
GO:0004721	118	3	0.149301480277151	phosphoprotein phosphatase activity
GO:0046982	61	2	0.151531052926042	protein heterodimerization activity
GO:0003824	3409	44	0.160559617616406	catalytic activity
GO:0016887	255	5	0.162525934502266	ATPase activity
GO:0004468	16	1	0.166640460818160	lysine N-acetyltransferase activity
GO:0004402	16	1	0.166640460818160	histone acetyltransferase activity
GO:0019212	17	1	0.176089576852978	phosphatase inhibitor activity
GO:0016796	17	1	0.176089576852978	exonuclease activity, active with either ribo- or deoxyribonucleic acids and producing 5'-phosphomonoesters
GO:0016706	17	1	0.176089576852978	oxidoreductase activity, acting on paired donors, with incorporation or reduction of molecular oxygen, 2-oxoglutarate as one donor, and incorporation of one atom each of oxygen into both donors
GO:0017111	410	7	0.182296919978181	nucleoside-triphosphatase activity
GO:0035091	132	3	0.188054315991874	phosphoinositide binding
GO:0016709	21	1	0.212836205496359	oxidoreductase activity, acting on paired donors, with incorporation or reduction of molecular oxygen, NAD or NADH as one donor, and incorporation of one atom of oxygen
GO:0016462	430	7	0.213954034131890	pyrophosphatase activity
GO:0016818	432	7	0.217229487735202	hydrolase activity, acting on acid anhydrides, in phosphorus-containing anhydrides
GO:0016817	433	7	0.218874223390347	hydrolase activity, acting on acid anhydrides
GO:0001637	22	1	0.221766214929804	G-protein chemoattractant receptor activity
GO:0004950	22	1	0.221766214929804	chemokine receptor activity
GO:0008408	24	1	0.239326213658087	3'-5' exonuclease activity
GO:0019956	24	1	0.239326213658087	chemokine binding
GO:0016831	24	1	0.239326213658087	carboxy-lyase activity
GO:0016874	221	4	0.240859219777393	ligase activity
GO:0019899	151	3	0.244130785595273	enzyme binding
GO:0016787	1424	19	0.253146756293419	hydrolase activity
GO:0005178	26	1	0.256493576694867	integrin binding
GO:0004907	26	1	0.256493576694867	interleukin receptor activity
GO:0003725	26	1	0.256493576694867	double-stranded RNA binding

GO:0003704	26	1	0.256493576694867	specific RNA polymerase II transcription factor activity
GO:0016705	87	2	0.258566921828201	oxidoreductase activity, acting on paired donors, with incorporation or reduction of molecular oxygen
GO:0004722	27	1	0.264932745766540	protein serine/threonine phosphatase activity
GO:0008080	28	1	0.273277003014513	N-acetyltransferase activity
GO:0008022	28	1	0.273277003014513	protein C-terminus binding
GO:0003887	28	1	0.273277003014513	DNA-directed DNA polymerase activity
GO:0004177	28	1	0.273277003014513	aminopeptidase activity
GO:0048503	92	2	0.279601513664188	GPI anchor binding
GO:0016830	31	1	0.297750821262411	carbon-carbon lyase activity
GO:0005543	169	3	0.299408043554605	phospholipid binding
GO:0008415	98	2	0.304789482062152	acyltransferase activity
GO:0003774	98	2	0.304789482062152	motor activity
GO:0016747	98	2	0.304789482062152	transferase activity, transferring groups other than amino-acyl groups
GO:0016791	171	3	0.305614289224138	phosphoric monoester hydrolase activity
GO:0004812	32	1	0.305725890800124	tRNA ligase activity
GO:0008452	32	1	0.305725890800124	RNA ligase activity
GO:0016875	32	1	0.305725890800124	ligase activity, forming carbon-oxygen bonds
GO:0016876	32	1	0.305725890800124	ligase activity, forming aminoacyl-tRNA and related compounds
GO:0004872	1060	14	0.311095418029701	receptor activity
GO:0016410	33	1	0.313611220886735	N-acyltransferase activity
GO:0019205	34	1	0.3214078119831	nucleobase, nucleoside, nucleotide kinase activity
GO:0019888	35	1	0.329116653500489	protein phosphatase regulator activity
GO:0051213	35	1	0.329116653500489	dioxygenase activity
GO:0016701	35	1	0.329116653500489	oxidoreductase activity, acting on single donors with incorporation of molecular oxygen
GO:0016702	35	1	0.329116653500489	oxidoreductase activity, acting on single donors with incorporation of molecular oxygen, incorporation of two atoms of oxygen
GO:0016655	35	1	0.329116653500489	oxidoreductase activity, acting on NADH or NADPH, quinone or similar compound as acceptor
GO:0009055	104	2	0.329822928887083	electron carrier activity
GO:0016746	104	2	0.329822928887083	transferase activity, transferring acyl groups
GO:0004518	105	2	0.333973722385132	nuclease activity
GO:0019208	36	1	0.336738723921481	phosphatase regulator activity
GO:0016740	1169	15	0.342721599756512	transferase activity
GO:0016627	37	1	0.344274990919533	oxidoreductase activity, acting on the CH-CH group of donors
GO:0005126	38	1	0.351726411477265	hematopoietin/interferon-class (D200-domain) cytokine receptor binding
GO:0005516	112	2	0.362808683860994	calmodulin binding
GO:0004527	40	1	0.366378488448803	exonuclease activity
GO:0016886	40	1	0.366378488448803	ligase activity, forming phosphoric ester bonds
GO:0015631	42	1	0.380702401294716	tubulin binding
GO:0019992	42	1	0.380702401294716	diacylglycerol binding
GO:0005096	117	2	0.383125472414699	GTPase activator activity
GO:0004888	694	9	0.386308609666315	transmembrane receptor activity
GO:0008235	44	1	0.394705432777498	metalloexopeptidase activity
GO:0003677	1384	17	0.397776906939289	DNA binding
GO:0003924	121	2	0.399182542456705	GTPase activity
GO:0003676	1990	24	0.40002702199392	nucleic acid binding
GO:0004540	45	1	0.401588849989253	ribonuclease activity
GO:0005253	46	1	0.40839470552855	anion channel activity
GO:0019887	47	1	0.415123865276092	protein kinase regulator activity

GO:0016879	127	2	0.422902461585519	ligase activity, forming carbon-nitrogen bonds
GO:0005507	49	1	0.428355513142099	copper ion binding
GO:0008289	298	4	0.437506858122222	lipid binding
GO:0008237	133	2	0.446141305030678	metallopeptidase activity
GO:0019904	52	1	0.447648868463848	protein domain specific binding
GO:0030695	219	3	0.452891939744165	GTPase regulator activity
GO:0019901	54	1	0.460151250494458	protein kinase binding
GO:0003713	140	2	0.472592135407884	transcription coactivator activity
GO:0016651	57	1	0.478380973989081	oxidoreductase activity, acting on NADH or NADPH
GO:0042578	228	3	0.479335317834938	phosphoric ester hydrolase activity
GO:0042623	229	3	0.482238093375656	ATPase activity, coupled
GO:0019207	58	1	0.484320962897682	kinase regulator activity
GO:0003712	230	3	0.485133424613441	transcription cofactor activity
GO:0019842	59	1	0.490193928837474	vitamin binding
GO:0019900	60	1	0.496000621063329	kinase binding
GO:0005489	152	2	0.516144991855832	electron transporter activity
GO:0042803	64	1	0.518579349405364	protein homodimerization activity
GO:0008565	67	1	0.534853020251582	protein transporter activity
GO:0008238	68	1	0.540155519070753	exopeptidase activity
GO:0005554	522	6	0.544559268147157	molecular function unknown
GO:0030674	69	1	0.545398124785981	protein binding, bridging
GO:0003723	436	5	0.552904500317366	RNA binding
GO:0004497	71	1	0.555706330499596	monooxygenase activity
GO:0045182	72	1	0.560773248800129	translation regulator activity
GO:0016788	446	5	0.573366799395096	hydrolase activity, acting on ester bonds
GO:0004725	75	1	0.575633022645745	protein tyrosine phosphatase activity
GO:0005539	77	1	0.585261709454137	glycosaminoglycan binding
GO:0004222	77	1	0.585261709454137	metalloendopeptidase activity
GO:0030247	80	1	0.5993003266462	polysaccharide binding
GO:0005125	181	2	0.6112672959605	cytokine activity
GO:0030246	181	2	0.6112672959605	carbohydrate binding
GO:0008092	279	3	0.616322555163802	cytoskeletal protein binding
GO:0008168	84	1	0.6172886213338	methyltransferase activity
GO:0005200	86	1	0.625980379122107	structural constituent of cytoskeleton
GO:0016741	87	1	0.630252628625524	transferase activity, transferring one-carbon groups
GO:0008047	188	2	0.63200940555428	enzyme activator activity
GO:0001871	88	1	0.634476524977168	pattern binding
GO:0003779	189	2	0.6349020408522	actin binding
GO:0004175	288	3	0.637873263507983	endopeptidase activity
GO:0008134	293	3	0.649481524364501	transcription factor binding
GO:0016853	92	1	0.650899332952114	isomerase activity
GO:0005085	93	1	0.654889477098708	guanyl-nucleotide exchange factor activity
GO:0003702	197	2	0.657412605907495	RNA polymerase II transcription factor activity
GO:0001653	94	1	0.658834432271585	peptide receptor activity
GO:0008528	94	1	0.658834432271585	peptide receptor activity, G-protein coupled
GO:0030528	963	10	0.661070102680105	transcription regulator activity
GO:0008509	95	1	0.66273470551332	anion transporter activity

GO:0016779	95	1	0.66273470551332	nucleotidyltransferase activity
GO:0008233	399	4	0.667355235579796	peptidase activity
GO:0003735	100	1	0.681583198168083	structural constituent of ribosome
GO:0016881	101	1	0.685225716396856	acid-amino acid ligase activity
GO:0030234	509	5	0.690195169656115	enzyme regulator activity
GO:0005529	103	1	0.692387355453569	sugar binding
GO:0004871	1837	19	0.707356613392511	signal transducer activity
GO:0005102	528	5	0.72091106151577	receptor binding
GO:0005525	226	2	0.729878739133897	GTP binding
GO:0019001	235	2	0.749592282587606	guanyl nucleotide binding
GO:0003700	649	6	0.752347204222704	transcription factor activity
GO:0042277	127	1	0.76670951700371	peptide binding
GO:0016829	127	1	0.76670951700371	lyase activity
GO:0000287	251	2	0.781621371963107	magnesium ion binding
GO:0005083	144	1	0.80829446804191	small GTPase regulator activity
GO:0046914	1596	15	0.821513608165459	transition metal ion binding
GO:0008270	1287	11	0.878430698165536	zinc ion binding
GO:0004857	187	1	0.883513231118603	enzyme inhibitor activity
GO:0005198	488	3	0.919789027734773	structural molecule activity
GO:0005386	398	2	0.94375322685437	carrier activity
GO:0005216	252	1	0.945384318575325	ion channel activity
GO:0015268	274	1	0.957786374562424	alpha-type channel activity
GO:0001584	282	1	0.961566641342358	rhodopsin-like receptor activity
GO:0015267	284	1	0.962458058587125	channel or pore class transporter activity
GO:0004930	349	1	0.982538641289823	G-protein coupled receptor activity
GO:0046872	2495	18	0.99317175241451	metal ion binding
GO:0043169	2313	16	0.994301873418138	cation binding
GO:0043167	2556	18	0.995478550776995	ion binding
GO:0005509	646	2	0.995597894036566	calcium ion binding
GO:0015075	523	1	0.99781026219055	ion transporter activity
GO:0005215	1030	3	0.999589119427327	transporter activity
GO:0003674	9541	108	1	molecular_function

13.4 GO: Cellular Component

GO ID	genes present in chip	genes present in tissues	p-value	GO term
GO:0005819	68	6	0.000126968955679188	spindle
GO:0000502	41	4	0.00123345080721412	proteasome complex (sensu Eukaryota)
GO:0043228	977	22	0.00152330362150827	non-membrane-bound organelle
GO:0043232	977	22	0.00152330362150827	intracellular non-membrane-bound organelle
GO:0015630	191	8	0.00161923053978985	microtubule cytoskeleton
GO:0000793	23	3	0.00225414884033331	condensed chromosome
GO:0005694	203	8	0.00237478475564344	chromosome
GO:0005634	2704	45	0.00264400280139432	nucleus
GO:0005622	5419	76	0.00320767435464821	intracellular
GO:0043226	4515	66	0.00361250766968629	organelle
GO:0043229	4515	66	0.00361250766968629	intracellular organelle
GO:0043227	3955	59	0.00520497932189121	membrane-bound organelle
GO:0043231	3955	59	0.00520497932189121	intracellular membrane-bound organelle
GO:0005876	14	2	0.0110308947168173	spindle microtubule
GO:0043020	1	1	0.0115780942456878	NADPH oxidase complex
GO:0009320	1	1	0.0115780942456878	phosphoribosylaminoimidazole carboxylase complex
GO:0005971	1	1	0.0115780942456878	ribonucleoside-diphosphate reductase complex
GO:0042272	1	1	0.0115780942456878	nuclear RNA export factor complex
GO:0000776	17	2	0.0161185623054063	kinetochore
GO:0005839	18	2	0.0179979926257672	proteasome core complex (sensu Eukaryota)
GO:0005874	132	5	0.0183232480939951	microtubule
GO:0005827	2	1	0.023023461380405	polar microtubule
GO:0051233	2	1	0.023023461380405	spindle midzone
GO:0005856	612	13	0.0237496064416422	cytoskeleton
GO:0005816	3	1	0.0343376077454274	spindle pole body
GO:0000796	3	1	0.0343376077454274	condensin complex
GO:0005737	2530	38	0.0371004421281152	cytoplasm
GO:0000775	27	2	0.038608306278255	chromosome, pericentric region
GO:0005654	221	6	0.0428471888505910	nucleoplasm
GO:0031229	4	1	0.0455220227588	intrinsic to nuclear inner membrane
GO:0005639	4	1	0.0455220227588	integral to nuclear inner membrane
GO:0005663	6	1	0.0675075329137369	DNA replication factor C complex
GO:0000242	6	1	0.0675075329137369	pericentriolar material
GO:0000930	6	1	0.0675075329137369	gamma-tubulin complex
GO:0005637	7	1	0.0783115239586488	nuclear inner membrane
GO:0000228	42	2	0.0847856168014771	nuclear chromosome
GO:0005813	43	2	0.0882729586945227	centrosome
GO:0005770	8	1	0.088991575824713	late endosome
GO:0005765	9	1	0.099549096095604	lysosomal membrane
GO:0005680	10	1	0.109985476530287	anaphase-promoting complex
GO:0016363	10	1	0.109985476530287	nuclear matrix
GO:0005815	49	2	0.110036792242655	microtubule organizing center
GO:0005739	490	9	0.113401369834087	mitochondrion

GO:0000922	51	2	0.117578883366946	spindle pole
GO:0043234	1247	19	0.124266244380732	protein complex
GO:0005764	107	3	0.126977765110974	lysosome
GO:0000323	107	3	0.126977765110974	lytic vacuole
GO:0005623	7802	94	0.137879745615063	cell
GO:0005871	13	1	0.140581462720459	kinesin complex
GO:0045121	14	1	0.150546891028431	lipid raft
GO:0000152	14	1	0.150546891028431	nuclear ubiquitin ligase complex
GO:0030894	14	1	0.150546891028431	replisome
GO:0005657	15	1	0.160397907020216	replication fork
GO:0005626	15	1	0.160397907020216	insoluble fraction
GO:0000178	15	1	0.160397907020216	exosome (RNase complex)
GO:0000794	15	1	0.160397907020216	condensed nuclear chromosome
GO:0005759	62	2	0.161073254667455	mitochondrial matrix
GO:0005773	120	3	0.162020705806072	vacuole
GO:0005788	16	1	0.170135811137355	endoplasmic reticulum lumen
GO:0005665	16	1	0.170135811137355	DNA-directed RNA polymerase II, core complex
GO:0043233	18	1	0.189277412516622	organelle lumen
GO:0005875	70	2	0.194271952454607	microtubule associated complex
GO:0005829	276	5	0.215357532485580	cytosol
GO:0005730	75	2	0.215445142741889	nucleolus
GO:0048471	22	1	0.226258887944068	perinuclear region
GO:0030880	23	1	0.235240212518136	RNA polymerase complex
GO:0005741	26	1	0.261569541108023	mitochondrial outer membrane
GO:0005761	27	1	0.270144973216861	mitochondrial ribosome
GO:0000313	27	1	0.270144973216861	organellar ribosome
GO:0005938	28	1	0.278621802796224	cell cortex
GO:0005667	91	2	0.284143016670935	transcription factor complex
GO:0000785	100	2	0.322713848072346	chromatin
GO:0005783	483	7	0.326137357496268	endoplasmic reticulum
GO:0005886	1472	19	0.339491217701105	plasma membrane
GO:0000151	42	1	0.38755029933407	ubiquitin ligase complex
GO:0008372	523	7	0.40280941013366	cellular component unknown
GO:0019867	52	1	0.455223127114319	outer membrane
GO:0005941	57	1	0.486227997511385	unlocalized protein complex
GO:0016591	58	1	0.492216016188409	DNA-directed RNA polymerase II, holoenzyme
GO:0009986	61	1	0.509768617608892	cell surface
GO:0005768	62	1	0.515484935034161	endosome
GO:0031301	76	1	0.588915653073712	integral to organelle membrane
GO:0005635	83	1	0.621384204034257	nuclear membrane
GO:0019866	89	1	0.647184462885465	inner membrane
GO:0031300	90	1	0.651311925883997	intrinsic to organelle membrane
GO:0005887	1057	11	0.693350190174942	integral to plasma membrane
GO:0031226	1062	11	0.699452012013053	intrinsic to plasma membrane
GO:0005840	113	1	0.734094813187525	ribosome
GO:0005740	119	1	0.75227648394204	mitochondrial membrane
GO:0016023	122	1	0.760900165680698	cytoplasmic membrane-bound vesicle

GO:0031410	124	1	0.766483482627346	cytoplasmic vesicle
GO:0015629	183	1	0.883988152176635	actin cytoskeleton
GO:0005625	183	1	0.883988152176635	soluble fraction
GO:0005794	195	1	0.899434631145783	Golgi apparatus
GO:0031090	377	2	0.936909694703344	organelle membrane
GO:0005615	385	2	0.941724305315426	extracellular space
GO:0012505	246	1	0.94533293961463	endomembrane system
GO:0030529	254	1	0.95033461757304	ribonucleoprotein complex
GO:0016020	3514	32	0.971371676566142	membrane
GO:0005578	303	1	0.972461711518172	extracellular matrix (sensu Metazoa)
GO:0031012	304	1	0.972792144545311	extracellular matrix
GO:0016021	2674	20	0.995130787843296	integral to membrane
GO:0031224	2681	20	0.995364634851284	intrinsic to membrane
GO:0000267	698	2	0.997939383503633	cell fraction
GO:0005576	949	3	0.99925485472845	extracellular region
GO:0005575	8637	100	1	cellular_component

14. PB-CD8TCells

14.1 PB-CD8TCells selective genes (119 genes)

Symbol	Description	Chromosome	Chromosome Location	LocusLink	Cytoband	UniGene
CCL5	chemokine (C-C motif) ligand 5	17	-31222609	6352	17q11.2-q12	Hs.514821
CLEC2D	C-type lectin domain family 2, member D	12	9713575	29121	12p13	Hs.268326
RASGRP1	RAS guanyl releasing protein 1 (calcium and DAG-regulated)	15	-36567597	10125	15q15	Hs.591127
ITK	IL2-inducible T-cell kinase	5	156540484	3702	5q31-q32	Hs.558348
CD69	CD69 molecule	12	-9796352	969	12p13-p12	Hs.208854
NKG7	natural killer cell group 7 sequence	19	-56566686	4818	19q13.33	Hs.10306
PTPRC	protein tyrosine phosphatase, receptor type, C	1	196874847	5788	1q31-q32	Hs.192039
GZMA	granzyme A (granzyme 1, cytotoxic T-lymphocyte-associated serine esterase 3)	5	54434229	3001	5q11-q12	Hs.90708
CD3D	CD3d molecule, delta (CD3-TCR complex)	11	-117714998	915	11q23	Hs.504048
MYBL1	v-myb myeloblastosis viral oncogene homolog (avian)-like 1	8	NA	4603	8q22	Hs.445898
ZNF651	zinc finger protein 651	3	42675877	92999	3p22.1	Hs.409561
PRF1	perforin 1 (pore forming protein)	10	-72027110	5551	10q22	Hs.2200
GIMAP6	GTPase, IMAP family member 6	7	-149953398	474344	NA	Hs.438823
CCR7	chemokine (C-C motif) receptor 7	17	-35963549	1236	17q12-q21.2	Hs.370036
BCL11B	B-cell CLL/lymphoma 11B (zinc finger protein)	14	-98705379	64919	14q32.2	Hs.510396
RASGRP2	RAS guanyl releasing protein 2 (calcium and DAG-regulated)	11	-64250959	10235	11q13	Hs.99491
LCK	lymphocyte-specific protein tyrosine kinase	1	32489518	3932	1p34.3	Hs.470627
KLRB1	killer cell lectin-like receptor subfamily B, member 1	12	-9639137	3820	12p13	Hs.169824
TRAF3IP3	TRAF3 interacting protein 3	1	207996146	80342	1q32.3-q41	Hs.147434
IL7R	interleukin 7 receptor	5	35892747	3575	5p13	Hs.591742
PSCDBP	pleckstrin homology, Sec7 and coiled-coil domains, binding protein	2	-157979377	9595	2q11.2	Hs.270
BTN3A3	butyrophilin, subfamily 3, member A3	6	26548741	10384	6p21.3	Hs.167741
GNLY	granulysin	2	85774924, 85775059	10578	2p12-q11	Hs.105806
TRAC	T cell receptor alpha constant	14	NA	28755	14q11	Hs.74647
PPWD1	peptidylprolyl isomerase domain and WD repeat containing 1	5	64894890	23398	5q12.3	Hs.121432
TXK	TXK tyrosine kinase	4	-47763517	7294	4p12	Hs.479669
ATHL1	ATH1, acid trehalase-like 1 (yeast)	11	280693	80162	11p15.5	Hs.353181
CXCR4	chemokine (C-X-C motif) receptor 4	2	-136588389	7852	2q21	Hs.421986
LTB	lymphotoxin beta (TNF superfamily, member 3)	6	-37446700	4050	6p21.3	Hs.376208
CD247	CD247 molecule	1	-165666507	919	1q22-q23	Hs.156445
LCP2	lymphocyte cytosolic protein 2 (SH2 domain containing leukocyte protein of 76kDa)	5	-169607666	3937	5q33.1-qter	Hs.304475

C6orf111	chromosome 6 open reading frame 111	6	-99954923	25957	6q16.3	Hs.591345
BTN3A2	butyrophilin, subfamily 3, member A2	6	26473376	11118	6p22.1	Hs.376046
PTPN6	protein tyrosine phosphatase, non-receptor type 6	12	6930703, 6926000	5777	12p13	Hs.63489
DENND2D	DENN/MADD domain containing 2D	1	-111531323	79961	1p13.3	Hs.557850
TNFRSF7	tumor necrosis factor receptor superfamily, member 7	12	6424326	939	12p13	Hs.355307
ITGA4	integrin, alpha 4 (antigen CD49D, alpha 4 subunit of VLA-4 receptor)	2	182029863	3676	2q31.3	Hs.440955
TNFAIP8	tumor necrosis factor, alpha-induced protein 8	5	118719628	25816	5q23.1	Hs.271955
ATM	ataxia telangiectasia mutated (includes complementation groups A, C and D)	11	107598768, 107663328	472	11q22-q23	Hs.435561
DOCK2	dedicator of cytokinesis 2	5	168996870	1794	5q35.1	Hs.586174
ADCY7	adenylate cyclase 7	16	48879322	113	16q12-q13	Hs.513578
IL18RAP	interleukin 18 receptor accessory protein	2	102401685	8807	2p24.3-p24.1	Hs.158315
TRDC	T cell receptor delta constant	14	NA	28526	14q11	NA
DGKA	diacylglycerol kinase, alpha 80kDa	12	54611212, 54612078	1606	12q13.3	Hs.524488
SELL	selectin L (lymphocyte adhesion molecule 1)	1	-167926433	6402	1q23-q25	Hs.82848
RUNX3	runt-related transcription factor 3	1	-25098589	864	1p36	Hs.170019
NSUN5B	NOL1/NOP2/Sun domain family, member 5B	7	74877581	155400	7q11.23	Hs.632322
GMFG	glia maturation factor, gamma	19	-44510841	9535	19q13.2	Hs.5210
GCH1	GTP cyclohydrolase 1 (dopa-responsive dystonia)	14	-54378475	2643	14q22.1-q22.2	Hs.86724
ZAP70	zeta-chain (TCR) associated protein kinase 70kDa	2	97696462, 97717300	7535	2q12	Hs.234569
PLAC8	placenta-specific 8	4	-84230866	51316	4q21.22	Hs.546392
GPR65	G protein-coupled receptor 65	14	87541248	8477	14q31-q32.1	Hs.513440
ISG20	interferon stimulated exonuclease gene 20kDa	15	86983042	3669	15q26	Hs.459265
CX3CR1	chemokine (C-X3-C motif) receptor 1	3	-39279989	1524	3p21|3p21.3	Hs.78913
FLJ22028	NA	12	21481877	79912	12p12.1	Hs.46835
RAPGEF6	Rap guanine nucleotide exchange factor (GEF) 6	5	-130789502	51735	5q31.1	Hs.483329
CD7	CD7 molecule	17	-77866034	924	17q25.2-q25.3	Hs.36972
RARRES3	retinoic acid receptor responder (tazarotene induced) 3	11	63060855	5920	11q23	Hs.17466
IL32	interleukin 32	16	3055313, 3055663, 3055639, 3055612, 3055785	9235	16p13.3	Hs.943
CST7	cystatin F (leukocystatin)	20	24877865	8530	20p11.21	Hs.143212
ICAM3	intercellular adhesion molecule 3	19	-10305453	3385	19p13.3-p13.2	Hs.75516
GPR18	G protein-coupled receptor 18	13	-98704972	2841	13q32	Hs.631765
STK10	serine/threonine kinase 10	5	-171403229	6793	5q35.1	Hs.519756
PTGER2	prostaglandin E receptor 2 (subtype EP2), 53kDa	14	51850862	5732	14q22	Hs.2090
HECA	headcase homolog (Drosophila)	6	139497941	51696	6q23-q24	Hs.197644
EVI2B	ecotropic viral integration site 2B	17	-26654918	2124	17q11.2	Hs.5509
DUSP2	dual specificity phosphatase 2	2	-96172643	1844	2q11	Hs.1183
CD52	CD52 molecule	1	26516997	1043	1p36	Hs.276770
ITGAL	integrin, alpha L (antigen CD11A (p180), lymphocyte function-associated antigen 1, alpha polypeptide)	16	30391571	3683	16p11.2	Hs.174103
FLT3LG	fms-related tyrosine kinase 3 ligand	19	54669297	2323	19q13.3	Hs.428
CD8A	CD8a molecule	2	-86865239	925	2p12	Hs.85258
PTPRCAP	protein tyrosine phosphatase, receptor type, C-associated protein	11	-66959556	5790	11q13.3	Hs.155975
LRAP	NA	5	96241023	64167	5q15	Hs.591249

BIN2	bridging integrator 2	12	-49961089	51411	12q13	Hs.14770
GZMH	granzyme H (cathepsin G-like 2, protein h-CCPX)	14	-24145532	2999	14q11.2	Hs.348264
RP6-213H19.1	NA	X	130985316, 130984925	51765	Xq26.2	Hs.444247
CD48	CD48 molecule	1	-158915161	962	1q21.3-q22	Hs.243564
WASPIP	Wiskott-Aldrich syndrome protein interacting protein	2	-175132548	7456	2q31.1	Hs.591641
TRIM22	tripartite motif-containing 22	11	5667663	10346	11p15	Hs.501778 Hs.621411
LRRFIP1	leucine rich repeat (in FLII) interacting protein 1	2	238265667	9208	2q37.3	Hs.471779
RNF138	ring finger protein 138	18	27925834, 27926644	51444	18q12.1	Hs.302408 Hs.501040
PRKCH	protein kinase C, eta	14	60858267	5583	14q22-q23	Hs.333907
ARL4C	ADP-ribosylation factor-like 4C	2	-235066426	10123	2q37.1	Hs.111554
KLRK1	killer cell lectin-like receptor subfamily K, member 1	12	-10416219	22914	12p13.2-p12.3	Hs.387787
CRTAM	cytotoxic and regulatory T cell molecule	11	122214464	56253	11q22-q23	Hs.159523
CBLB	Cas-Br-M (murine) ecotropic retroviral transforming sequence b	3	-106859799	868	3q13.11	Hs.430589
CD8B	CD8b molecule	2	-173818172	926	2p12	Hs.405667 Hs.610012
FAIM3	Fas apoptotic inhibitory molecule 3	1	-205144353	9214	1q32.1	Hs.58831
SEMA4D	sema domain, immunoglobulin domain (Ig), transmembrane domain (TM) and short cytoplasmic domain, (semaphorin) 4D	9	-91181971	10507	9q22-q31	Hs.494406
CYLD	cylindromatosis (turban tumor syndrome)	16	49333461, 49333529	1540	16q12.1	Hs.578973
LAT	linker for activation of T cells	16	28903887, 28903647	27040	16p11.2	Hs.632179
GBP1	guanylate binding protein 1, interferon-inducible, 67kDa	1	-89290593	2633	1p22.2	Hs.62661
ZFP36L2	zinc finger protein 36, C3H type-like 2	2	-43304210	678	2p22.3-p21	Hs.503093 Hs.636758
HMHA1	histocompatibility (minor) HA-1	19	1018173	23526	19p13.3	Hs.465521
SYNE2	spectrin repeat containing, nuclear envelope 2	14	63389453, 63751902, 63740825, 63750614, 63612085	23224	14q23.2	Hs.525392
CD37	CD37 molecule	19	54530488	951	19q13.3	Hs.166556
CTSW	cathepsin W (lymphopain)	11	65403859	1521	11q13.1	Hs.416848
KLRF1	killer cell lectin-like receptor subfamily F, member 1	12	9871343	51348	12p12.3-13.2	Hs.183125
COTL1	coactosin-like 1 (Dictyostelium)	16	-83156706	23406	16q24.1	Hs.289092
NSUN5C	NOL1/NOP2/Sun domain family, member 5C	7	-72056768	260294	7q11.23	Hs.510927 Hs.632322
ARHGEF3	Rho guanine nucleotide exchange factor (GEF) 3	3	-56736485	50650	3p21-p13	Hs.476402
CORO1A	coronin, actin binding protein, 1A	16	30102426	11151	16p11.2	Hs.415067
MBNL1	muscleblind-like (Drosophila)	3	153468518, 153499883	4154	3q25	Hs.478000 Hs.558914
STK38	serine/threonine kinase 38	6	-36569649	11329	6p21	Hs.409578
RNF125	ring finger protein 125	18	27852442	54941	18q12.1	Hs.272800 Hs.638727
IL10RA	interleukin 10 receptor, alpha	11	117362318	3587	11q23	Hs.504035
HCLS1	hematopoietic cell-specific Lyn substrate 1	3	-122832936	3059	3q13	Hs.14601
EDG1	endothelial differentiation, sphingolipid G-protein-coupled receptor, 1	1	101475042	1901	1p21	Hs.154210
ALOX5AP	arachidonate 5-lipoxygenase-activating protein	13	30207668	241	13q12	Hs.507658
GOLGA8A	golgi autoantigen, golgin subfamily a, 8A	15	-32458563	23015	15q11.2	Hs.182982
CD160	CD160 molecule	1	-144407154	11126	1q21.1	Hs.488237
PTPN4	protein tyrosine phosphatase, non-receptor type 4 (megakaryocyte)	2	120233676	5775	2q14.2	Hs.469809

HMGB2	high-mobility group box 2	4	-174489362	3148	4q31	Hs.434953
GZMM	granzyme M (lymphocyte met-ase 1)	19	495026	3004	19p13.3	Hs.465511
TRAF5	TNF receptor-associated factor 5	1	209566801, 209566579, 209586328	7188	1q32	Hs.523930
LTBP3	latent transforming growth factor beta binding protein 3	11	-65062851	4054	11q12	Hs.289019
LBR	lamin B receptor	1	-223655827	3930	1q42.1	Hs.435166
KLF2	Kruppel-like factor 2 (lung)	19	16296650	10365	19p13.13-p13.11	Hs.107740
GPR171	G protein-coupled receptor 171	3	-152398315	29909	3q25.1	Hs.549152

14.2 GO: Biological Process

GO ID	genes present in chip	genes present in tissues	p-value	GO term
GO:0006952	751	40	1.10E-19	defense response
GO:0009607	857	41	1.65E-18	response to biotic stimulus
GO:0006955	684	37	2.75E-18	immune response
GO:0050896	1578	44	2.60E-11	response to stimulus
GO:0042110	45	9	5.73E-10	T cell activation
GO:0007165	2168	48	6.91E-09	signal transduction
GO:0007166	952	30	9.88E-09	cell surface receptor linked signal transduction
GO:0006968	87	10	1.71E-08	cellular defense response
GO:0007154	2730	54	2.06E-08	cell communication
GO:0009613	458	20	2.84E-08	response to pest, pathogen or parasite
GO:0043207	463	20	3.40E-08	response to external biotic stimulus
GO:0050874	1531	38	3.75E-08	organismal physiological process
GO:0046649	81	9	1.25E-07	lymphocyte activation
GO:0045321	97	9	5.97E-07	immune cell activation
GO:0001775	98	9	6.52E-07	cell activation
GO:0009611	341	15	1.78E-06	response to wounding
GO:0019835	14	4	9.75E-06	cytolysis
GO:0050863	31	5	1.43E-05	regulation of T cell activation
GO:0030098	34	5	2.28E-05	lymphocyte differentiation
GO:0030217	18	4	2.89E-05	T cell differentiation
GO:0050870	21	4	5.51E-05	positive regulation of T cell activation
GO:0051249	41	5	5.81E-05	regulation of lymphocyte activation
GO:0007242	879	22	6.19E-05	intracellular signaling cascade
GO:0006950	881	22	6.41E-05	response to stress
GO:0050865	43	5	7.34E-05	regulation of cell activation
GO:0045059	2	2	0.000104894	positive thymic T cell selection
GO:0008219	498	15	0.000155351	cell death
GO:0016265	501	15	0.000165984	death
GO:0051251	28	4	0.000178499	positive regulation of lymphocyte activation
GO:0050867	29	4	0.000205436	positive regulation of cell activation
GO:0007265	29	4	0.000205436	Ras protein signal transduction
GO:0030097	87	6	0.00026175	hemopoiesis
GO:0048534	88	6	0.000278599	hemopoietic or lymphoid organ development
GO:0006915	471	14	0.000302144	apoptosis
GO:0050857	3	2	0.000312568	positive regulation of antigen receptor mediated signaling pathway
GO:0045061	3	2	0.000312568	thymic T cell selection
GO:0012501	473	14	0.000315519	programmed cell death
GO:0050854	4	2	0.00062094	regulation of antigen receptor mediated signaling pathway
GO:0051282	4	2	0.00062094	regulation of sequestering of calcium ion
GO:0051283	4	2	0.00062094	negative regulation of sequestering of calcium ion
GO:0051209	4	2	0.00062094	release of sequestered calcium ion into cytoplasm
GO:0009605	963	21	0.000635546	response to external stimulus
GO:0050852	5	2	0.001027957	T cell receptor signaling pathway

GO:0051208	5	2	0.001027957	sequestering of calcium ion
GO:0050776	77	5	0.001144338	regulation of immune response
GO:0050851	6	2	0.0015316	antigen receptor-mediated signaling pathway
GO:0045058	6	2	0.0015316	T cell selection
GO:0009967	85	5	0.001781184	positive regulation of signal transduction
GO:0045582	7	2	0.002129878	positive regulation of T cell differentiation
GO:0045621	7	2	0.002129878	positive regulation of lymphocyte differentiation
GO:0009966	229	8	0.002414574	regulation of signal transduction
GO:0050778	56	4	0.002570719	positive regulation of immune response
GO:0001906	8	2	0.002820832	cell killing
GO:0051238	8	2	0.002820832	sequestering of metal ion
GO:0051240	59	4	0.003111736	positive regulation of organismal physiological process
GO:0042100	9	2	0.00360253	B cell proliferation
GO:0016064	103	5	0.004113175	humoral defense mechanism (sensu Vertebrata)
GO:0042981	306	9	0.004144295	regulation of apoptosis
GO:0043067	308	9	0.004324784	regulation of programmed cell death
GO:0045580	10	2	0.004473071	regulation of T cell differentiation
GO:0006874	68	4	0.005190744	calcium ion homeostasis
GO:0006793	641	14	0.005663193	phosphorus metabolism
GO:0006796	641	14	0.005663193	phosphate metabolism
GO:0007204	37	3	0.006375311	elevation of cytoplasmic calcium ion concentration
GO:0051480	37	3	0.006375311	cytoplasmic calcium ion homeostasis
GO:0045619	12	2	0.006473221	regulation of lymphocyte differentiation
GO:0019735	77	4	0.008042562	antimicrobial humoral response (sensu Vertebrata)
GO:0019730	78	4	0.008411733	antimicrobial humoral response
GO:0031342	1	1	0.010296723	negative regulation of cell killing
GO:0048305	1	1	0.010296723	immunoglobulin secretion
GO:0006926	1	1	0.010296723	programmed cell death, virus-infected cells
GO:0001911	1	1	0.010296723	negative regulation of immune cell mediated cytotoxicity
GO:0001913	1	1	0.010296723	T cell mediated cytotoxicity
GO:0001914	1	1	0.010296723	regulation of T cell mediated cytotoxicity
GO:0001915	1	1	0.010296723	negative regulation of T cell mediated cytotoxicity
GO:0019724	1	1	0.010296723	B cell mediated immunity
GO:0045579	1	1	0.010296723	positive regulation of B cell differentiation
GO:0050853	1	1	0.010296723	B cell receptor signaling pathway
GO:0051103	1	1	0.010296723	DNA ligation during DNA repair
GO:0000738	1	1	0.010296723	DNA catabolism, exonucleolytic
GO:0006288	1	1	0.010296723	base-excision repair, DNA ligation
GO:0006882	1	1	0.010296723	zinc ion homeostasis
GO:0007229	45	3	0.010975294	integrin-mediated signaling pathway
GO:0042221	183	6	0.011341511	response to chemical substance
GO:0007186	491	11	0.01178189	G-protein coupled receptor protein signaling pathway
GO:0051239	133	5	0.011878335	regulation of organismal physiological process
GO:0006954	185	6	0.011921288	inflammatory response
GO:0030005	87	4	0.012241672	di-, tri-valent inorganic cation homeostasis
GO:0045597	18	2	0.014416913	positive regulation of cell differentiation
GO:0006959	141	5	0.014989776	humoral immune response

GO:0006875	93	4	0.015329419	metal ion homeostasis
GO:0006468	444	10	0.015625428	protein amino acid phosphorylation
GO:0030003	98	4	0.018247263	cation homeostasis
GO:0006470	99	4	0.018869454	protein amino acid dephosphorylation
GO:0006935	101	4	0.020153058	chemotaxis
GO:0042330	101	4	0.020153058	taxis
GO:0031341	2	1	0.020488551	regulation of cell killing
GO:0006922	2	1	0.020488551	cleavage of lamin
GO:0006923	2	1	0.020488551	cleavage of cytoskeletal proteins
GO:0006927	2	1	0.020488551	programmed cell death, transformed cells
GO:0001910	2	1	0.020488551	regulation of immune cell mediated cytotoxicity
GO:0001907	2	1	0.020488551	killing of host cells
GO:0048247	2	1	0.020488551	lymphocyte chemotaxis
GO:0019836	2	1	0.020488551	hemolysis
GO:0050862	2	1	0.020488551	positive regulation of T cell receptor signaling pathway
GO:0044003	2	1	0.020488551	modification of host morphology or physiology
GO:0044004	2	1	0.020488551	disruption of host cells
GO:0001897	2	1	0.020488551	cytolysis of host cells
GO:0006873	103	4	0.021489415	cell ion homeostasis
GO:0009615	58	3	0.021692147	response to virus
GO:0016311	110	4	0.02658906	dephosphorylation
GO:0051235	25	2	0.026984358	maintenance of localization
GO:0006464	1087	18	0.027102864	protein modification
GO:0050801	111	4	0.027371916	ion homeostasis
GO:0006916	112	4	0.02816849	anti-apoptosis
GO:0044419	26	2	0.029040174	interaction between organisms
GO:0051056	65	3	0.029175776	regulation of small GTPase mediated signal transduction
GO:0007264	227	6	0.02939345	small GTPase mediated signal transduction
GO:0043303	3	1	0.030576543	mast cell degranulation
GO:0043299	3	1	0.030576543	immune cell degranulation
GO:0045577	3	1	0.030576543	regulation of B cell differentiation
GO:0008588	3	1	0.030576543	release of cytoplasmic sequestered NF-kappaB
GO:0050856	3	1	0.030576543	regulation of T cell receptor signaling pathway
GO:0050680	3	1	0.030576543	negative regulation of epithelial cell proliferation
GO:0042531	3	1	0.030576543	positive regulation of tyrosine phosphorylation of STAT protein
GO:0046427	3	1	0.030576543	positive regulation of JAK-STAT cascade
GO:0006266	3	1	0.030576543	DNA ligation
GO:0016066	27	2	0.031156618	cellular defense response (sensu Vertebrata)
GO:0007169	116	4	0.03149271	transmembrane receptor protein tyrosine kinase signaling pathway
GO:0046651	28	2	0.033332254	lymphocyte proliferation
GO:0051348	28	2	0.033332254	negative regulation of transferase activity
GO:0006469	28	2	0.033332254	negative regulation of protein kinase activity
GO:0019725	122	4	0.036895988	cell homeostasis
GO:0042113	30	2	0.037855462	B cell activation
GO:0007243	242	6	0.038392981	protein kinase cascade
GO:0007155	518	10	0.039818945	cell adhesion
GO:0051094	31	2	0.040200271	positive regulation of development

GO:0000018	4	1	0.040561744	regulation of DNA recombination
GO:0042346	4	1	0.040561744	positive regulation of NF-kappaB-nucleus import
GO:0042307	4	1	0.040561744	positive regulation of protein-nucleus import
GO:0051222	4	1	0.040561744	positive regulation of protein transport
GO:0042993	4	1	0.040561744	positive regulation of transcription factor-nucleus import
GO:0016310	522	10	0.041614779	phosphorylation
GO:0043066	128	4	0.042803135	negative regulation of apoptosis
GO:0043069	129	4	0.04383677	negative regulation of programmed cell death
GO:0043412	1160	18	0.047371545	biopolymer modification
GO:0045576	5	1	0.050445191	mast cell activation
GO:0030323	5	1	0.050445191	respiratory tube development
GO:0046146	5	1	0.050445191	tetrahydrobiopterin metabolism
GO:0006729	5	1	0.050445191	tetrahydrobiopterin biosynthesis
GO:0046824	5	1	0.050445191	positive regulation of nucleocytoplasmic transport
GO:0008283	473	9	0.053978612	cell proliferation
GO:0006917	140	4	0.05613061	induction of apoptosis
GO:0012502	140	4	0.05613061	induction of programmed cell death
GO:0030154	409	8	0.059298233	cell differentiation
GO:0006928	205	5	0.060056396	cell motility
GO:0040011	205	5	0.060056396	locomotion
GO:0001909	6	1	0.060227909	immune cell mediated cytotoxicity
GO:0035113	6	1	0.060227909	embryonic appendage morphogenesis
GO:0050871	6	1	0.060227909	positive regulation of B cell activation
GO:0030326	6	1	0.060227909	embryonic limb morphogenesis
GO:0046330	6	1	0.060227909	positive regulation of JNK cascade
GO:0007626	209	5	0.064167847	locomotory behavior
GO:0051247	41	2	0.066387685	positive regulation of protein metabolism
GO:0050790	214	5	0.069526227	regulation of enzyme activity
GO:0035107	7	1	0.069910914	appendage morphogenesis
GO:0035108	7	1	0.069910914	limb morphogenesis
GO:0050673	7	1	0.069910914	epithelial cell proliferation
GO:0050678	7	1	0.069910914	regulation of epithelial cell proliferation
GO:0042509	7	1	0.069910914	regulation of tyrosine phosphorylation of STAT protein
GO:0046425	7	1	0.069910914	regulation of JAK-STAT cascade
GO:0043065	155	4	0.075583999	positive regulation of apoptosis
GO:0043068	156	4	0.07698857	positive regulation of programmed cell death
GO:0042592	157	4	0.078406322	homeostasis
GO:0006558	8	1	0.079495212	L-phenylalanine metabolism
GO:0006559	8	1	0.079495212	L-phenylalanine catabolism
GO:0050832	8	1	0.079495212	defense response to fungi
GO:0045078	8	1	0.079495212	positive regulation of interferon-gamma biosynthesis
GO:0044267	2109	28	0.079782634	cellular protein metabolism
GO:0043086	46	2	0.081105908	negative regulation of enzyme activity
GO:0019538	2129	28	0.08782166	protein metabolism
GO:0048598	9	1	0.088981797	embryonic morphogenesis
GO:0050731	9	1	0.088981797	positive regulation of peptidyl-tyrosine phosphorylation
GO:0050864	9	1	0.088981797	regulation of B cell activation

GO:0006268	9	1	0.088981797	DNA unwinding
GO:0007167	165	4	0.090215733	enzyme linked receptor protein signaling pathway
GO:0048519	688	11	0.094962724	negative regulation of biological process
GO:0043085	107	3	0.097899706	positive regulation of enzyme activity
GO:0006607	10	1	0.098371656	NLS-bearing substrate-nucleus import
GO:0019438	10	1	0.098371656	aromatic compound biosynthesis
GO:0001934	10	1	0.098371656	positive regulation of protein amino acid phosphorylation
GO:0045764	10	1	0.098371656	positive regulation of amino acid metabolism
GO:0009074	10	1	0.098371656	aromatic amino acid family catabolism
GO:0042345	10	1	0.098371656	regulation of NF-kappaB-nucleus import
GO:0042348	10	1	0.098371656	NF-kappaB-nucleus import
GO:0009405	10	1	0.098371656	pathogenesis
GO:0007260	10	1	0.098371656	tyrosine phosphorylation of STAT protein
GO:0042559	10	1	0.098371656	pteridine and derivative biosynthesis
GO:0006606	53	2	0.10318981	protein-nucleus import
GO:0008632	53	2	0.10318981	apoptotic program
GO:0051170	54	2	0.106468021	nuclear import
GO:0006921	11	1	0.107665763	disassembly of cell structures during apoptosis
GO:0042558	11	1	0.107665763	pteridine and derivative metabolism
GO:0009620	11	1	0.107665763	response to fungi
GO:0046328	11	1	0.107665763	regulation of JNK cascade
GO:0006171	11	1	0.107665763	cAMP biosynthesis
GO:0045072	11	1	0.107665763	regulation of interferon-gamma biosynthesis
GO:0019932	176	4	0.107763921	second-messenger-mediated signaling
GO:0009628	318	6	0.109286552	response to abiotic stimulus
GO:0045595	55	2	0.10977423	regulation of cell differentiation
GO:0006887	55	2	0.10977423	exocytosis
GO:0019370	12	1	0.116865085	leukotriene biosynthesis
GO:0042095	12	1	0.116865085	interferon-gamma biosynthesis
GO:0035295	12	1	0.116865085	tube development
GO:0042327	12	1	0.116865085	positive regulation of phosphorylation
GO:0045937	12	1	0.116865085	positive regulation of phosphate metabolism
GO:0042102	12	1	0.116865085	positive regulation of T cell proliferation
GO:0048522	482	8	0.122626478	positive regulation of cellular process
GO:0019439	13	1	0.125970578	aromatic compound catabolism
GO:0046058	13	1	0.125970578	cAMP metabolism
GO:0030218	13	1	0.125970578	erythrocyte differentiation
GO:0030595	13	1	0.125970578	immune cell chemotaxis
GO:0048518	565	9	0.126498337	positive regulation of biological process
GO:0051336	60	2	0.126694002	regulation of hydrolase activity
GO:0008284	121	3	0.128581657	positive regulation of cell proliferation
GO:0006310	61	2	0.130149661	DNA recombination
GO:0051242	411	7	0.130306965	positive regulation of cellular physiological process
GO:0043170	2405	30	0.132762883	macromolecule metabolism
GO:0006691	14	1	0.134983189	leukotriene metabolism
GO:0009725	14	1	0.134983189	response to hormone stimulus
GO:0050900	14	1	0.134983189	immune cell migration

GO:0006308	14	1	0.134983189	DNA catabolism
GO:0030183	14	1	0.134983189	B cell differentiation
GO:0042127	265	5	0.137481049	regulation of cell proliferation
GO:0017038	64	2	0.140645172	protein import
GO:0043283	2240	28	0.142643881	biopolymer metabolism
GO:0007610	269	5	0.143760582	behavior
GO:0045445	15	1	0.143903854	myoblast differentiation
GO:0043407	15	1	0.143903854	negative regulation of MAPK activity
GO:0051050	15	1	0.143903854	positive regulation of transport
GO:0050730	15	1	0.143903854	regulation of peptidyl-tyrosine phosphorylation
GO:0042129	15	1	0.143903854	regulation of T cell proliferation
GO:0000188	15	1	0.143903854	inactivation of MAPK activity
GO:0042990	15	1	0.143903854	regulation of transcription factor-nucleus import
GO:0042991	15	1	0.143903854	transcription factor-nucleus import
GO:0051243	583	9	0.144981033	negative regulation of cellular physiological process
GO:0043119	428	7	0.151304238	positive regulation of physiological process
GO:0006809	16	1	0.152733503	nitric oxide biosynthesis
GO:0001932	16	1	0.152733503	regulation of protein amino acid phosphorylation
GO:0042306	16	1	0.152733503	regulation of protein-nucleus import
GO:0006284	16	1	0.152733503	base-excision repair
GO:0046209	16	1	0.152733503	nitric oxide metabolism
GO:0006521	17	1	0.161473053	regulation of amino acid metabolism
GO:0042098	17	1	0.161473053	T cell proliferation
GO:0019722	17	1	0.161473053	calcium-mediated signaling
GO:0051223	17	1	0.161473053	regulation of protein transport
GO:0048015	71	2	0.165785542	phosphoinositide-mediated signaling
GO:0043118	602	9	0.165918345	negative regulation of physiological process
GO:0043009	18	1	0.170123414	embryonic development (sensu Vertebrata)
GO:0031281	18	1	0.170123414	positive regulation of cyclase activity
GO:0045762	18	1	0.170123414	positive regulation of adenylate cyclase activity
GO:0009072	18	1	0.170123414	aromatic amino acid family metabolism
GO:0007190	18	1	0.170123414	adenylate cyclase activation
GO:0042133	18	1	0.170123414	neurotransmitter metabolism
GO:0050671	18	1	0.170123414	positive regulation of lymphocyte proliferation
GO:0051349	18	1	0.170123414	positive regulation of lyase activity
GO:0001701	18	1	0.170123414	embryonic development (sensu Mammalia)
GO:0044260	2298	28	0.178308296	cellular macromolecule metabolism
GO:0007131	19	1	0.178685487	meiotic recombination
GO:0042692	19	1	0.178685487	muscle cell differentiation
GO:0018108	19	1	0.178685487	peptidyl-tyrosine phosphorylation
GO:0046916	20	1	0.187160162	transition metal ion homeostasis
GO:0042493	20	1	0.187160162	response to drug
GO:0009190	20	1	0.187160162	cyclic nucleotide biosynthesis
GO:0007205	20	1	0.187160162	protein kinase C activation
GO:0044403	20	1	0.187160162	symbiosis, mutualism through parasitism
GO:0044404	20	1	0.187160162	symbiotic interaction between host and other organism
GO:0045786	145	3	0.187680623	negative regulation of progression through cell cycle

GO:0018212	21	1	0.195548323	peptidyl-tyrosine modification
GO:0000165	81	2	0.202882512	MAPKKK cascade
GO:0019885	22	1	0.203850843	antigen processing, endogenous antigen via MHC class I
GO:0007189	22	1	0.203850843	G-protein signaling, adenylate cyclase activating pathway
GO:0007127	22	1	0.203850843	meiosis I
GO:0050670	22	1	0.203850843	regulation of lymphocyte proliferation
GO:0045055	22	1	0.203850843	regulated secretory pathway
GO:0048523	640	9	0.211833804	negative regulation of cellular process
GO:0006805	23	1	0.212068587	xenobiotic metabolism
GO:0042108	23	1	0.212068587	positive regulation of cytokine biosynthesis
GO:0046456	23	1	0.212068587	icosanoid biosynthesis
GO:0046822	23	1	0.212068587	regulation of nucleocytoplasmic transport
GO:0042088	24	1	0.220202411	T-helper 1 type immune response
GO:0045727	25	1	0.228253163	positive regulation of protein biosynthesis
GO:0042087	25	1	0.228253163	cell-mediated immune response
GO:0042325	25	1	0.228253163	regulation of phosphorylation
GO:0009987	8200	87	0.234810662	cellular process
GO:0031328	26	1	0.236221683	positive regulation of cellular biosynthesis
GO:0009306	26	1	0.236221683	protein secretion
GO:0009410	26	1	0.236221683	response to xenobiotic stimulus
GO:0006919	27	1	0.244108801	caspase activation
GO:0009187	27	1	0.244108801	cyclic nucleotide metabolism
GO:0009792	27	1	0.244108801	embryonic development (sensu Metazoa)
GO:0043087	28	1	0.251915339	regulation of GTPase activity
GO:0008217	28	1	0.251915339	regulation of blood pressure
GO:0019220	28	1	0.251915339	regulation of phosphate metabolism
GO:0007259	28	1	0.251915339	JAK-STAT cascade
GO:0051174	28	1	0.251915339	regulation of phosphorus metabolism
GO:0001819	29	1	0.259642113	positive regulation of cytokine production
GO:0051052	29	1	0.259642113	regulation of DNA metabolism
GO:0051169	96	2	0.259933235	nuclear transport
GO:0009891	30	1	0.267289929	positive regulation of biosynthesis
GO:0007249	99	2	0.271417252	I-kappaB kinase/NF-kappaB cascade
GO:0043280	31	1	0.274859584	positive regulation of caspase activity
GO:0043281	31	1	0.274859584	regulation of caspase activity
GO:0031279	32	1	0.282351869	regulation of cyclase activity
GO:0045761	32	1	0.282351869	regulation of adenylate cyclase activity
GO:0051345	32	1	0.282351869	positive regulation of hydrolase activity
GO:0051339	32	1	0.282351869	regulation of lyase activity
GO:0006913	102	2	0.282898327	nucleocytoplasmic transport
GO:0006690	33	1	0.289767567	icosanoid metabolism
GO:0042035	33	1	0.289767567	regulation of cytokine biosynthesis
GO:0030155	33	1	0.289767567	regulation of cell adhesion
GO:0006401	33	1	0.289767567	RNA catabolism
GO:0042742	34	1	0.297107451	defense response to bacteria
GO:0042089	36	1	0.311562838	cytokine biosynthesis
GO:0030333	36	1	0.311562838	antigen processing

GO:0042107	37	1	0.318679852	cytokine metabolism
GO:0030099	37	1	0.318679852	myeloid cell differentiation
GO:0006487	37	1	0.318679852	protein amino acid N-linked glycosylation
GO:0006508	451	6	0.319799321	proteolysis and peptidolysis
GO:0044257	451	6	0.319799321	cellular protein catabolism
GO:0006461	194	3	0.322610155	protein complex assembly
GO:0050794	2488	28	0.325430784	regulation of cellular process
GO:0001817	39	1	0.332696239	regulation of cytokine production
GO:0007519	39	1	0.332696239	myogenesis
GO:0050789	2689	30	0.335312911	regulation of biological process
GO:0045859	116	2	0.33614725	regulation of protein kinase activity
GO:0051338	117	2	0.33991523	regulation of transferase activity
GO:0030163	463	6	0.341908449	protein catabolism
GO:0001505	41	1	0.346427314	regulation of neurotransmitter levels
GO:0019882	41	1	0.346427314	antigen presentation
GO:0001816	42	1	0.353187659	cytokine production
GO:0009063	42	1	0.353187659	amino acid catabolism
GO:0007254	42	1	0.353187659	JNK cascade
GO:0031098	42	1	0.353187659	stress-activated protein kinase signaling pathway
GO:0044265	563	7	0.359789267	cellular macromolecule catabolism
GO:0046903	208	3	0.362222254	secretion
GO:0043285	474	6	0.362330092	biopolymer catabolism
GO:0006979	44	1	0.366501501	response to oxidative stress
GO:0007126	44	1	0.366501501	meiosis
GO:0051321	44	1	0.366501501	meiotic cell cycle
GO:0051327	44	1	0.366501501	M phase of meiotic cell cycle
GO:0008154	45	1	0.373056392	actin polymerization and/or depolymerization
GO:0009310	45	1	0.373056392	amine catabolism
GO:0030036	126	2	0.373512145	actin cytoskeleton organization and biogenesis
GO:0009719	214	3	0.379121533	response to endogenous stimulus
GO:0035023	46	1	0.37954418	regulation of Rho protein signal transduction
GO:0051049	46	1	0.37954418	regulation of transport
GO:0009057	575	7	0.380339281	macromolecule catabolism
GO:0000074	394	5	0.38251053	regulation of progression through cell cycle
GO:0006633	47	1	0.385965546	fatty acid biosynthesis
GO:0044270	47	1	0.385965546	nitrogen compound catabolism
GO:0006334	48	1	0.392321161	nucleosome assembly
GO:0043405	49	1	0.398611692	regulation of MAPK activity
GO:0006605	134	2	0.402804351	protein targeting
GO:0018193	50	1	0.404837799	peptidyl-amino acid modification
GO:0031325	136	2	0.410030145	positive regulation of cellular metabolism
GO:0009617	51	1	0.411000133	response to bacteria
GO:0008285	139	2	0.420790023	negative regulation of cell proliferation
GO:0030029	139	2	0.420790023	actin filament-based process
GO:0045860	53	1	0.423136063	positive regulation of protein kinase activity
GO:0016053	54	1	0.429110931	organic acid biosynthesis
GO:0051347	54	1	0.429110931	positive regulation of transferase activity

GO:0046394	54	1	0.429110931	carboxylic acid biosynthesis
GO:0044248	700	8	0.433498507	cellular catabolism
GO:0031497	56	1	0.440877611	chromatin assembly
GO:0007200	56	1	0.440877611	G-protein signaling, coupled to IP3 second messenger (phospholipase C activating)
GO:0009893	149	2	0.455922914	positive regulation of metabolism
GO:0007188	59	1	0.458079203	G-protein signaling, coupled to cAMP nucleotide second messenger
GO:0019933	60	1	0.463695902	cAMP-mediated signaling
GO:0006800	61	1	0.469255006	oxygen and reactive oxygen species metabolism
GO:0007266	63	1	0.480202762	Rho protein signal transduction
GO:0006364	64	1	0.485592565	rRNA processing
GO:0009790	65	1	0.490927075	embryonic development
GO:0006261	65	1	0.490927075	DNA-dependent DNA replication
GO:0006512	255	3	0.491012736	ubiquitin cycle
GO:0016072	66	1	0.496206855	rRNA metabolism
GO:0045045	161	2	0.496452573	secretory pathway
GO:0006816	68	1	0.506604436	calcium ion transport
GO:0043123	68	1	0.506604436	positive regulation of I-kappaB kinase/NF-kappaB cascade
GO:0050793	168	2	0.519210106	regulation of development
GO:0009056	756	8	0.52201065	catabolism
GO:0051244	2319	24	0.528375259	regulation of cellular physiological process
GO:0043122	73	1	0.531678802	regulation of I-kappaB kinase/NF-kappaB cascade
GO:0006725	73	1	0.531678802	aromatic compound metabolism
GO:0000279	173	2	0.535049003	M phase
GO:0051246	174	2	0.538174421	regulation of protein metabolism
GO:0006281	174	2	0.538174421	DNA repair
GO:0048513	872	9	0.548783719	organ development
GO:0006520	181	2	0.559651829	amino acid metabolism
GO:0007046	79	1	0.560108745	ribosome biogenesis
GO:0042254	80	1	0.564678151	ribosome biogenesis and assembly
GO:0016477	85	1	0.586830255	cell migration
GO:0007049	601	6	0.59139098	cell cycle
GO:0006333	91	1	0.611944947	chromatin assembly or disassembly
GO:0050791	2407	24	0.612555479	regulation of physiological process
GO:0006974	200	2	0.614358509	response to DNA damage stimulus
GO:0006486	92	1	0.61598131	protein amino acid glycosylation
GO:0043413	93	1	0.619976136	biopolymer glycosylation
GO:0007187	94	1	0.623929846	G-protein signaling, coupled to cyclic nucleotide second messenger
GO:0007517	94	1	0.623929846	muscle development
GO:0007028	96	1	0.631715594	cytoplasm organization and biogenesis
GO:0009101	96	1	0.631715594	glycoprotein biosynthesis
GO:0019935	98	1	0.639341851	cyclic-nucleotide-mediated signaling
GO:0015674	99	1	0.643096184	di-, tri-valent inorganic cation transport
GO:0006519	216	2	0.656335701	amino acid and derivative metabolism
GO:0008015	103	1	0.657730779	circulation
GO:0009165	103	1	0.657730779	nucleotide biosynthesis
GO:0006511	105	1	0.664823854	ubiquitin-dependent protein catabolism
GO:0048232	105	1	0.664823854	male gamete generation

GO:0007283	105	1	0.664823854	spermatogenesis
GO:0019941	105	1	0.664823854	modification-dependent protein catabolism
GO:0007001	221	2	0.668694543	chromosome organization and biogenesis (sensu Eukaryota)
GO:0006417	107	1	0.671771483	regulation of protein biosynthesis
GO:0031326	111	1	0.685242144	regulation of cellular biosynthesis
GO:0009100	111	1	0.685242144	glycoprotein metabolism
GO:0009889	116	1	0.701313652	regulation of biosynthesis
GO:0051276	236	2	0.703651669	chromosome organization and biogenesis
GO:0006357	240	2	0.712447613	regulation of transcription from RNA polymerase II promoter
GO:0000004	465	4	0.712533956	biological process unknown
GO:0019752	359	3	0.721273724	carboxylic acid metabolism
GO:0006082	361	3	0.724799128	organic acid metabolism
GO:0006631	124	1	0.725355602	fatty acid metabolism
GO:0007276	125	1	0.728222905	gametogenesis
GO:0006886	257	2	0.747451266	intracellular protein transport
GO:0006259	489	4	0.749128958	DNA metabolism
GO:0007067	135	1	0.755315971	mitosis
GO:0000087	137	1	0.760404865	M phase of mitotic cell cycle
GO:0006260	140	1	0.767842519	DNA replication
GO:0009308	269	2	0.769933441	amine metabolism
GO:0009117	143	1	0.775051695	nucleotide metabolism
GO:0019953	149	1	0.788812184	sexual reproduction
GO:0016481	151	1	0.793211317	negative regulation of transcription
GO:0006412	407	3	0.796700031	protein biosynthesis
GO:0006807	291	2	0.806700374	nitrogen compound metabolism
GO:0044249	763	6	0.808743419	cellular biosynthesis
GO:0045934	159	1	0.809919443	negative regulation of nucleobase, nucleoside, nucleotide and nucleic acid metabolism
GO:0016192	296	2	0.814301686	vesicle-mediated transport
GO:0006366	426	3	0.82154182	transcription from RNA polymerase II promoter
GO:0007010	305	2	0.827321886	cytoskeleton organization and biogenesis
GO:0006457	169	1	0.828937548	protein folding
GO:0016337	172	1	0.834266743	cell-cell adhesion
GO:0008610	175	1	0.839431645	lipid biosynthesis
GO:0006996	693	5	0.851009213	organelle organization and biogenesis
GO:0031324	183	1	0.852439141	negative regulation of cellular metabolism
GO:0006325	186	1	0.857043376	establishment and/or maintenance of chromatin architecture
GO:0009059	464	3	0.863630617	macromolecule biosynthesis
GO:0007267	465	3	0.864611764	cell-cell signaling
GO:0007268	192	1	0.865829692	synaptic transmission
GO:0006323	193	1	0.867241212	DNA packaging
GO:0019226	198	1	0.87408144	transmission of nerve impulse
GO:0000278	204	1	0.881830812	mitotic cell cycle
GO:0000003	208	1	0.886732987	reproduction
GO:0009058	859	6	0.887998064	biosynthesis
GO:0009892	217	1	0.897039425	negative regulation of metabolism
GO:0007399	385	2	0.911665443	neurogenesis
GO:0016070	391	2	0.916127282	RNA metabolism

GO:0006118	256	1	0.931979877	electron transport
GO:0030001	269	1	0.940782856	metal ion transport
GO:0016043	842	5	0.942564539	cell organization and biogenesis
GO:0007582	7846	76	0.943250283	physiological process
GO:0015031	439	2	0.944969629	protein transport
GO:0046907	448	2	0.94921459	intracellular transport
GO:0045184	459	2	0.953984157	establishment of protein localization
GO:0044238	4820	42	0.955170951	primary metabolism
GO:0044237	5026	44	0.95787639	cellular metabolism
GO:0008104	474	2	0.959809727	protein localization
GO:0006396	326	1	0.96782833	RNA processing
GO:0007275	1543	10	0.96788484	development
GO:0031323	1566	10	0.972070819	regulation of cellular metabolism
GO:0019219	1458	9	0.974026473	regulation of nucleobase, nucleoside, nucleotide and nucleic acid metabolism
GO:0006812	346	1	0.974053021	cation transport
GO:0005975	355	1	0.976449931	carbohydrate metabolism
GO:0006355	1359	8	0.977658628	regulation of transcription, DNA-dependent
GO:0008152	5262	45	0.979032178	metabolism
GO:0019222	1629	10	0.981169039	regulation of metabolism
GO:0044255	383	1	0.982591959	cellular lipid metabolism
GO:0006351	1407	8	0.983834022	transcription, DNA-dependent
GO:0045449	1433	8	0.986488286	regulation of transcription
GO:0006091	446	1	0.991212079	generation of precursor metabolites and energy
GO:0006350	1499	8	0.991537053	transcription
GO:0009887	807	3	0.991723867	organogenesis
GO:0006629	469	1	0.993161493	lipid metabolism
GO:0050877	475	1	0.993595316	neurophysiological process
GO:0050875	7018	62	0.995113347	cellular physiological process
GO:0006811	515	1	0.995867372	ion transport
GO:0006139	2328	13	0.998137597	nucleobase, nucleoside, nucleotide and nucleic acid metabolism
GO:0051179	1857	9	0.998503538	localization
GO:0009653	1051	3	0.999155349	morphogenesis
GO:0006810	1802	7	0.999760546	transport
GO:0051234	1841	7	0.999831805	establishment of localization
GO:0008150	9032	93	1	biological_process

14.3 GO: Molecular Function

GO ID	genes present in chip	genes present in tissues	p-value	GO term
GO:0004871	1837	39	7.19960616445021e-07	signal transducer activity
GO:0004872	1060	28	7.88655536123293e-07	receptor activity
GO:0004888	694	19	4.32716231257553e-05	transmembrane receptor activity
GO:0015026	13	3	0.000254437395133822	coreceptor activity
GO:0004715	15	3	0.000398976148188805	non-membrane spanning protein tyrosine kinase activity
GO:0019955	65	5	0.000453689912189385	cytokine binding
GO:0001637	22	3	0.00128386623428512	G-protein chemoattractant receptor activity
GO:0004950	22	3	0.00128386623428512	chemokine receptor activity
GO:0019956	24	3	0.00166323611380062	chemokine binding
GO:0042288	8	2	0.00264184296649638	MHC class I protein binding
GO:0016494	9	2	0.00337467662009692	C-X-C chemokine receptor activity
GO:0019958	10	2	0.00419107076228925	C-X-C chemokine binding
GO:0042287	11	2	0.00508932417093371	MHC protein binding
GO:0005102	528	12	0.00597443476559782	receptor binding
GO:0016493	12	2	0.00606776059896261	C-C chemokine receptor activity
GO:0019957	12	2	0.00606776059896261	C-C chemokine binding
GO:0004721	118	5	0.0063599894315453	phosphoprotein phosphatase activity
GO:0004725	75	4	0.00653947335674394	protein tyrosine phosphatase activity
GO:0001584	282	8	0.0069483917211166	rhodopsin-like receptor activity
GO:0008310	1	1	0.00995702756524364	single-stranded DNA specific 3'-5' exodeoxyribonuclease activity
GO:0008859	1	1	0.00995702756524364	exoribonuclease II activity
GO:0051397	1	1	0.00995702756524364	N-terminal basic amino acid aminopeptidase activity
GO:0051398	1	1	0.00995702756524364	N-terminal lysine aminopeptidase activity
GO:0051399	1	1	0.00995702756524364	N-terminal arginine aminopeptidase activity
GO:0003933	1	1	0.00995702756524364	GTP cyclohydrolase activity
GO:0003934	1	1	0.00995702756524364	GTP cyclohydrolase I activity
GO:0042610	1	1	0.00995702756524364	CD8 receptor binding
GO:0004277	1	1	0.00995702756524364	granzyme A activity
GO:0019838	49	3	0.0126587281007947	growth factor binding
GO:0005085	93	4	0.0137243180667149	guanyl-nucleotide exchange factor activity
GO:0001614	20	2	0.0165886827409235	purinergic nucleotide receptor activity
GO:0001608	20	2	0.0165886827409235	nucleotide receptor activity, G-protein coupled
GO:0016502	20	2	0.0165886827409235	nucleotide receptor activity
GO:0045028	20	2	0.0165886827409235	purinergic nucleotide receptor activity, G-protein coupled
GO:0005529	103	4	0.0192860698018490	sugar binding
GO:0030676	2	1	0.0198159460538503	Rac guanyl-nucleotide exchange factor activity
GO:0008330	2	1	0.0198159460538503	protein tyrosine/threonine phosphatase activity
GO:0004920	2	1	0.0198159460538503	interleukin-10 receptor activity
GO:0008297	2	1	0.0198159460538503	single-stranded DNA specific exodeoxyribonuclease activity
GO:0004917	2	1	0.0198159460538503	interleukin-7 receptor activity
GO:0019969	2	1	0.0198159460538503	interleukin-10 binding
GO:0019982	2	1	0.0198159460538503	interleukin-7 binding
GO:0051117	2	1	0.0198159460538503	ATPase binding

GO:0042609	2	1	0.0198159460538503	CD4 receptor binding
GO:0004713	161	5	0.0220867095128753	protein-tyrosine kinase activity
GO:0005515	3709	47	0.0224088494429629	protein binding
GO:0004930	349	8	0.0227408182390976	G-protein coupled receptor activity
GO:0004907	26	2	0.0273031557980672	interleukin receptor activity
GO:0003725	26	2	0.0273031557980672	double-stranded RNA binding
GO:0030106	26	2	0.0273031557980672	MHC class I receptor activity
GO:0016791	171	5	0.0277504124607587	phosphoric monoester hydrolase activity
GO:0001948	3	1	0.0295777119753189	glycoprotein binding
GO:0008296	3	1	0.0295777119753189	3'-5'-exodeoxyribonuclease activity
GO:0042608	3	1	0.0295777119753189	T cell receptor binding
GO:0004672	447	9	0.0335367758594187	protein kinase activity
GO:0019965	30	2	0.0356213113315051	interleukin binding
GO:0016773	532	10	0.0383995325341376	phosphotransferase activity, alcohol group as acceptor
GO:0016301	609	11	0.0390313473381069	kinase activity
GO:0017016	4	1	0.0392432726130145	Ras GTPase binding
GO:0019238	4	1	0.0392432726130145	cyclohydrolase activity
GO:0003779	189	5	0.0401122385724205	actin binding
GO:0008301	5	1	0.0488135661122031	DNA bending activity
GO:0004960	5	1	0.0488135661122031	thromboxane receptor activity
GO:0004957	5	1	0.0488135661122031	prostaglandin E receptor activity
GO:0005521	5	1	0.0488135661122031	lamin binding
GO:0005522	5	1	0.0488135661122031	profilin binding
GO:0005088	5	1	0.0488135661122031	Ras guanyl-nucleotide exchange factor activity
GO:0005083	144	4	0.0552996850273094	small GTPase regulator activity
GO:0043027	6	1	0.058289521567252	caspase inhibitor activity
GO:0008092	279	6	0.0594412887441995	cytoskeletal protein binding
GO:0019899	151	4	0.0636331794839788	enzyme binding
GO:0048503	92	3	0.0637252958458673	GPI anchor binding
GO:0019992	42	2	0.0653295445553872	diacylglycerol binding
GO:0001653	94	3	0.0670787389234063	peptide receptor activity
GO:0008528	94	3	0.0670787389234063	peptide receptor activity, G-protein coupled
GO:0004529	7	1	0.0676720591080073	exodeoxyribonuclease activity
GO:0001619	7	1	0.0676720591080073	lysosphingolipid and lysophosphatidic acid receptor activity
GO:0045125	7	1	0.0676720591080073	bioactive lipid receptor activity
GO:0016895	7	1	0.0676720591080073	exodeoxyribonuclease activity, producing 5'-phosphomonoesters
GO:0030742	7	1	0.0676720591080073	GTP-dependent protein binding
GO:0042802	158	4	0.0725916361031013	protein self binding
GO:0008289	298	6	0.0764563253484184	lipid binding
GO:0042578	228	5	0.0769149318787328	phosphoric ester hydrolase activity
GO:0017017	8	1	0.0769620899853665	MAP kinase phosphatase activity
GO:0004955	8	1	0.0769620899853665	prostaglandin receptor activity
GO:0004726	8	1	0.0769620899853665	non-membrane spanning protein tyrosine phosphatase activity
GO:0004896	48	2	0.0824098971667887	hematopoietin/interferon-class (D200-domain) cytokine receptor activity
GO:0004016	9	1	0.0861605166560251	adenylate cyclase activity
GO:0004252	105	3	0.086908385814344	serine-type endopeptidase activity
GO:0016772	708	11	0.0927369295343758	transferase activity, transferring phosphorus-containing groups

GO:0001565	10	1	0.0952682328664408	phorbol ester receptor activity
GO:0004697	10	1	0.0952682328664408	protein kinase C activity
GO:0004953	10	1	0.0952682328664408	icosanoid receptor activity
GO:0004954	10	1	0.0952682328664408	prostanoid receptor activity
GO:0000175	10	1	0.0952682328664408	3'-5'-exoribonuclease activity
GO:0019901	54	2	0.100682804769391	protein kinase binding
GO:0004532	11	1	0.104286123735992	exoribonuclease activity
GO:0016896	11	1	0.104286123735992	exoribonuclease activity, producing 5'-phosphomonoesters
GO:0005125	181	4	0.106229963456222	cytokine activity
GO:0030246	181	4	0.106229963456222	carbohydrate binding
GO:0008236	117	3	0.111009924737972	serine-type peptidase activity
GO:0016284	12	1	0.113215065839358	alanine aminopeptidase activity
GO:0004179	12	1	0.113215065839358	membrane alanyl aminopeptidase activity
GO:0004143	12	1	0.113215065839358	diacylglycerol kinase activity
GO:0019900	60	2	0.119954540886498	kinase binding
GO:0016563	62	2	0.126569569432953	transcriptional activator activity
GO:0042169	14	1	0.130809567811592	SH2 domain binding
GO:0042277	127	3	0.132830681239829	peptide binding
GO:0042803	64	2	0.133269849917002	protein homodimerization activity
GO:0030234	509	8	0.133774293866801	enzyme regulator activity
GO:0035091	132	3	0.144265237123260	phosphoinositide binding
GO:0016788	446	7	0.155681843737422	hydrolase activity, acting on ester bonds
GO:0016796	17	1	0.156555637065411	exonuclease activity, active with either ribo- or deoxyribonucleic acids and producing 5'-phosphomonoesters
GO:0004175	288	5	0.159257891519168	endopeptidase activity
GO:0016849	18	1	0.164968826321898	phosphorus-oxygen lyase activity
GO:0005001	19	1	0.173298970341578	transmembrane receptor protein tyrosine phosphatase activity
GO:0043028	19	1	0.173298970341578	caspase regulator activity
GO:0004869	19	1	0.173298970341578	cysteine protease inhibitor activity
GO:0019198	19	1	0.173298970341578	transmembrane receptor protein phosphatase activity
GO:0030695	219	4	0.174136392185534	GTPase regulator activity
GO:0005164	20	1	0.181546880215298	tumor necrosis factor receptor binding
GO:0003690	20	1	0.181546880215298	double-stranded DNA binding
GO:0009975	20	1	0.181546880215298	cyclase activity
GO:0004197	80	2	0.189275256647971	cysteine-type endopeptidase activity
GO:0016814	21	1	0.18971335919645	hydrolase activity, acting on carbon-nitrogen (but not peptide) bonds, in cyclic amidines
GO:0008233	399	6	0.206334830706543	peptidase activity
GO:0008408	24	1	0.213732127325011	3'-5' exonuclease activity
GO:0004860	25	1	0.221580761128113	protein kinase inhibitor activity
GO:0004536	25	1	0.221580761128113	deoxyribonuclease activity
GO:0003755	25	1	0.221580761128113	peptidyl-prolyl cis-trans isomerase activity
GO:0004674	326	5	0.224502385059725	protein serine/threonine kinase activity
GO:0005178	26	1	0.229351865341315	integrin binding
GO:0008138	26	1	0.229351865341315	protein tyrosine/serine/threonine phosphatase activity
GO:0016859	26	1	0.229351865341315	cis-trans isomerase activity
GO:0005543	169	3	0.23702166803394	phospholipid binding
GO:0004722	27	1	0.237046197742007	protein serine/threonine phosphatase activity
GO:0046983	170	3	0.239674254348967	protein dimerization activity

GO:0000287	251	4	0.240232386752028	magnesium ion binding
GO:0008022	28	1	0.244664508779899	protein C-terminus binding
GO:0003697	28	1	0.244664508779899	single-stranded DNA binding
GO:0004177	28	1	0.244664508779899	aminopeptidase activity
GO:0019210	29	1	0.252207541647124	kinase inhibitor activity
GO:0008234	98	2	0.255218698474409	cysteine-type peptidase activity
GO:0003823	30	1	0.259676032347663	antigen binding
GO:0005488	7228	75	0.275713133140293	binding
GO:0004857	187	3	0.285426245975025	enzyme inhibitor activity
GO:0031267	35	1	0.295925618930195	small GTPase binding
GO:0008009	40	1	0.330418037288763	chemokine activity
GO:0004527	40	1	0.330418037288763	exonuclease activity
GO:0042379	40	1	0.330418037288763	chemokine receptor binding
GO:0005070	40	1	0.330418037288763	SH3/SH2 adaptor activity
GO:0004221	41	1	0.337113152166941	ubiquitin thiolesterase activity
GO:0003924	121	2	0.339924342669419	GTPase activity
GO:0001664	42	1	0.343742020645272	G-protein-coupled receptor binding
GO:0051020	42	1	0.343742020645272	GTPase binding
GO:0004843	43	1	0.350305291309415	ubiquitin-specific protease activity
GO:0008235	44	1	0.356803606462669	metalloexopeptidase activity
GO:0005089	44	1	0.356803606462669	Rho guanyl-nucleotide exchange factor activity
GO:0004540	45	1	0.363237602186166	ribonuclease activity
GO:0019887	47	1	0.375915148914786	protein kinase regulator activity
GO:0005525	226	3	0.391836369116881	GTP binding
GO:0016790	51	1	0.400525719960746	thiolester hydrolase activity
GO:0019904	52	1	0.406526779666091	protein domain specific binding
GO:0019001	235	3	0.41604721069831	guanyl nucleotide binding
GO:0019207	58	1	0.441304933136503	kinase regulator activity
GO:0016564	59	1	0.446901899429029	transcriptional repressor activity
GO:0016787	1424	15	0.449889665707481	hydrolase activity
GO:0046982	61	1	0.457929919360025	protein heterodimerization activity
GO:0005509	646	7	0.465455386436431	calcium ion binding
GO:0008238	68	1	0.494841957424732	exopeptidase activity
GO:0030674	69	1	0.499907935894557	protein binding, bridging
GO:0016810	72	1	0.514806192976284	hydrolase activity, acting on carbon-nitrogen (but not peptide) bonds
GO:0008270	1287	13	0.523411172567608	zinc ion binding
GO:0003714	81	1	0.55691606983796	transcription corepressor activity
GO:0008047	188	2	0.561958214757339	enzyme activator activity
GO:0017076	1248	12	0.598075060387389	purine nucleotide binding
GO:0016853	92	1	0.603499750503063	isomerase activity
GO:0030145	92	1	0.603499750503063	manganese ion binding
GO:0016740	1169	11	0.626823603096292	transferase activity
GO:0004866	99	1	0.630585214391804	endopeptidase inhibitor activity
GO:0003700	649	6	0.634067376456247	transcription factor activity
GO:0003735	100	1	0.634302054535076	structural constituent of ribosome
GO:0030414	100	1	0.634302054535076	protease inhibitor activity
GO:0005524	970	9	0.639167061996925	ATP binding

GO:0016874	221	2	0.650429192885914	ligase activity
GO:0004518	105	1	0.652338388965647	nuclease activity
GO:0030554	1028	9	0.70802416273646	adenyl nucleotide binding
GO:0016829	127	1	0.721792955944365	lyase activity
GO:0005198	488	4	0.723223168396159	structural molecule activity
GO:0005057	132	1	0.735552779717323	receptor signaling protein activity
GO:0008237	133	1	0.738222828173785	metallopeptidase activity
GO:0046914	1596	14	0.739943305329243	transition metal ion binding
GO:0043167	2556	23	0.750846907421166	ion binding
GO:0030528	963	8	0.755670635132754	transcription regulator activity
GO:0008083	144	1	0.765889393856023	growth factor activity
GO:0003824	3409	31	0.769166600362188	catalytic activity
GO:0005554	522	4	0.77099746726993	molecular function unknown
GO:0046872	2495	22	0.781438598705451	metal ion binding
GO:0000166	1432	12	0.783543352693703	nucleotide binding
GO:0043169	2313	20	0.800607989526855	cation binding
GO:0003723	436	3	0.81585280621215	RNA binding
GO:0003702	197	1	0.86357782519901	RNA polymerase II transcription factor activity
GO:0003677	1384	10	0.899420061508661	DNA binding
GO:0003712	230	1	0.90268570224713	transcription cofactor activity
GO:0017111	410	2	0.91988132528515	nucleoside-triphosphatase activity
GO:0016462	430	2	0.93232191603639	pyrophosphatase activity
GO:0016818	432	2	0.933461374112816	hydrolase activity, acting on acid anhydrides, in phosphorus-containing anhydrides
GO:0016817	433	2	0.93402435138936	hydrolase activity, acting on acid anhydrides
GO:0008134	293	1	0.949109094370024	transcription factor binding
GO:0003676	1990	14	0.950443984231876	nucleic acid binding
GO:0016491	509	1	0.994672529130895	oxidoreductase activity
GO:0003674	9541	95	1	molecular_function

14.4 GO: Cellular Component

GO ID	genes present in chip	genes present in tissues	p-value	GO term
GO:0001772	29	7	1.07438274039115e-08	immunological synapse
GO:0042101	10	5	2.23680931546075e-08	T cell receptor complex
GO:0043235	58	7	1.63476595909372e-06	receptor complex
GO:0005886	1472	33	2.45386039179105e-06	plasma membrane
GO:0016020	3514	54	4.27313476603821e-05	membrane
GO:0031224	2681	44	0.000105438574467713	intrinsic to membrane
GO:0016021	2674	43	0.000228874662206993	integral to membrane
GO:0031226	1062	23	0.000243722162792185	intrinsic to plasma membrane
GO:0005887	1057	22	0.000603003612319505	integral to plasma membrane
GO:0045121	14	2	0.00843745155919236	lipid raft
GO:0008305	24	2	0.0239794882557132	integrin complex
GO:0031229	4	1	0.0396938509017705	intrinsic to nuclear inner membrane
GO:0005639	4	1	0.0396938509017705	integral to nuclear inner membrane
GO:0046658	5	1	0.0493714409598663	anchored to plasma membrane
GO:0031225	5	1	0.0493714409598663	anchored to membrane
GO:0000242	6	1	0.0589526138788299	pericentriolar material
GO:0005637	7	1	0.0684383191960055	nuclear inner membrane
GO:0005925	8	1	0.0778294972064283	focal adhesion
GO:0016605	8	1	0.0778294972064283	PML body
GO:0005856	612	10	0.0868789038592771	cytoskeleton
GO:0005765	9	1	0.0871270790517213	lysosomal membrane
GO:0005924	9	1	0.0871270790517213	cell-substrate adherens junction
GO:0030055	11	1	0.105445133573855	cell-matrix junction
GO:0005623	7802	82	0.141816154476498	cell
GO:0005788	16	1	0.149665793322323	endoplasmic reticulum lumen
GO:0005665	16	1	0.149665793322323	DNA-directed RNA polymerase II, core complex
GO:0016604	16	1	0.149665793322323	nuclear body
GO:0043233	18	1	0.166742707561272	organelle lumen
GO:0005912	18	1	0.166742707561272	adherens junction
GO:0048471	22	1	0.199885936918181	perinuclear region
GO:0000793	23	1	0.207966020898230	condensed chromosome
GO:0030880	23	1	0.207966020898230	RNA polymerase complex
GO:0005938	28	1	0.247172204241257	cell cortex
GO:0000785	100	2	0.266924913823082	chromatin
GO:0043228	977	12	0.276427859565357	non-membrane-bound organelle
GO:0043232	977	12	0.276427859565357	intracellular non-membrane-bound organelle
GO:0000786	40	1	0.333611506392896	nucleosome
GO:0005615	385	5	0.346606255299249	extracellular space
GO:0000228	42	1	0.347031486632263	nuclear chromosome
GO:0005813	43	1	0.353640941043314	centrosome
GO:0005815	49	1	0.39193138917124	microtubule organizing center
GO:0016327	50	1	0.398091376262775	apicolateral plasma membrane
GO:0000922	51	1	0.404189670226341	spindle pole

GO:0008372	523	6	0.432597363515250	cellular component unknown
GO:0016591	58	1	0.445200877818468	DNA-directed RNA polymerase II, holoenzyme
GO:0005819	68	1	0.498990932180677	spindle
GO:0031301	76	1	0.538282830739757	integral to organelle membrane
GO:0015629	183	2	0.55380002375841	actin cytoskeleton
GO:0005635	83	1	0.570154069161616	nuclear membrane
GO:0019866	89	1	0.595734319322395	inner membrane
GO:0031300	90	1	0.599848862399016	intrinsic to organelle membrane
GO:0005694	203	2	0.610657640923009	chromosome
GO:0030054	93	1	0.61194573124714	cell junction
GO:0005654	221	2	0.657044439726862	nucleoplasm
GO:0005764	107	1	0.663785760633854	lysosome
GO:0000323	107	1	0.663785760633854	lytic vacuole
GO:0005840	113	1	0.683848840857039	ribosome
GO:0005773	120	1	0.705763872341586	vacuole
GO:0005624	521	4	0.778317389235619	membrane fraction
GO:0043234	1247	10	0.825172375774557	protein complex
GO:0000267	698	5	0.842509544637884	cell fraction
GO:0005625	183	1	0.846276034369895	soluble fraction
GO:0015630	191	1	0.858490101967855	microtubule cytoskeleton
GO:0005794	195	1	0.864232256914018	Golgi apparatus
GO:0012505	246	1	0.920077828866888	endomembrane system
GO:0030529	254	1	0.926473983671257	ribonucleoprotein complex
GO:0005576	949	6	0.927230513878296	extracellular region
GO:0005829	276	1	0.941569524835177	cytosol
GO:0005634	2704	20	0.966886615086268	nucleus
GO:0005737	2530	18	0.973920262784753	cytoplasm
GO:0031090	377	1	0.979814224868648	organelle membrane
GO:0005783	483	1	0.993476844315979	endoplasmic reticulum
GO:0005622	5419	43	0.996010918276137	intracellular
GO:0043226	4515	31	0.999413849471805	organelle
GO:0043229	4515	31	0.999413849471805	intracellular organelle
GO:0043227	3955	23	0.999939185729987	membrane-bound organelle
GO:0043231	3955	23	0.999939185729987	intracellular membrane-bound organelle
GO:0005575	8637	87	1	cellular_component

15. PB-CD4TCells

15.1 PB-CD4TCells selective genes (92 genes)

Symbol	Description	Chromosome	Chromosome Location	LocusLink	Cytoband	UniGene
ITK	IL2-inducible T-cell kinase	5	156540484	3702	5q31-q32	Hs.558348
CD69	CD69 molecule	12	-9796352	969	12p13-p12	Hs.208854
RASGRP1	RAS guanyl releasing protein 1 (calcium and DAG-regulated)	15	-36567597	10125	15q15	Hs.591127
CD3D	CD3d molecule, delta (CD3-TCR complex)	11	-117714998	915	11q23	Hs.504048
PTPRC	protein tyrosine phosphatase, receptor type, C	1	196874847	5788	1q31-q32	Hs.192039
CLEC2D	C-type lectin domain family 2, member D	12	9713575	29121	12p13	Hs.268326
CCL5	chemokine (C-C motif) ligand 5	17	-31222609	6352	17q11.2-q12	Hs.514821
CCR7	chemokine (C-C motif) receptor 7	17	-35963549	1236	17q12-q21.2	Hs.370036
BCL11B	B-cell CLL/lymphoma 11B (zinc finger protein)	14	-98705379	64919	14q32.2	Hs.510396
ZNF651	zinc finger protein 651	3	42675877	92999	3p22.1	Hs.409561
RASGRP2	RAS guanyl releasing protein 2 (calcium and DAG-regulated)	11	-64250959	10235	11q13	Hs.99491
GIMAP6	GTPase, IMAP family member 6	7	-149953398	474344		Hs.438823
GZMA	granzyme A (granzyme 1, cytotoxic T-lymphocyte-associated serine esterase 3)	5	54434229	3001	5q11-q12	Hs.90708
IL7R	interleukin 7 receptor	5	35892747	3575	5p13	Hs.591742
LCK	lymphocyte-specific protein tyrosine kinase	1	32489518	3932	1p34.3	Hs.470627
PRF1	perforin 1 (pore forming protein)	10	-72027110	5551	10q22	Hs.2200
KLRB1	killer cell lectin-like receptor subfamily B, member 1	12	-9639137	3820	12p13	Hs.169824
TRAC	T cell receptor alpha constant	14		28755	14q11	Hs.74647
PSCDBP	pleckstrin homology, Sec7 and coiled-coil domains, binding protein	2	-157979377	9595	2q11.2	Hs.270
LTB	lymphotoxin beta (TNF superfamily, member 3)	6	-37446700	4050	6p21.3	Hs.376208
GNLY	granulysin	2	85774924, 85775059	10578	2p12-q11	Hs.105806
CD247	CD247 molecule	1	-165666507	919	1q22-q23	Hs.156445
TRAF3IP3	TRAF3 interacting protein 3	1	207996146	80342	1q32.3-q41	Hs.147434
PPWD1	peptidylprolyl isomerase domain and WD repeat containing 1	5	64894890	23398	5q12.3	Hs.121432
TXK	TXK tyrosine kinase	4	-47763517	7294	4p12	Hs.479669
DGKA	diacylglycerol kinase, alpha 80kDa	12	54611212, 54612078	1606	12q13.3	Hs.524488
BTN3A3	butyrophilin, subfamily 3, member A3	6	26548741	10384	6p21.3	Hs.167741
CXCR4	chemokine (C-X-C motif) receptor 4	2	-136588389	7852	2q21	Hs.421986
NKG7	natural killer cell group 7 sequence	19	-56566686	4818	19q13.33	Hs.10306
ATHL1	ATH1, acid trehalase-like 1 (yeast)	11	280693	80162	11p15.5	Hs.353181
DENND2D	DENN/MADD domain containing 2D	1	-111531323	79961	1p13.3	Hs.557850
SELL	selectin L (lymphocyte adhesion molecule 1)	1	-167926433	6402	1q23-q25	Hs.82848
TNFRSF7	tumor necrosis factor receptor superfamily, member 7	12	6424326	939	12p13	Hs.355307

DOCK2	dedicator of cytokinesis 2	5	168996870	1794	5q35.1	Hs.586174
GMFG	glia maturation factor, gamma	19	-44510841	9535	19q13.2	Hs.5210
PTPN6	protein tyrosine phosphatase, non-receptor type 6	12	6930703, 6926000	5777	12p13	Hs.63489
TNFAIP8	tumor necrosis factor, alpha-induced protein 8	5	118719628	25816	5q23.1	Hs.271955
MYBL1	v-myb myeloblastosis viral oncogene homolog (avian)-like 1	8		4603	8q22	Hs.445898
C6orf111	chromosome 6 open reading frame 111	6	-99954923	25957	6q16.3	Hs.591345
BTN3A2	butyrophilin, subfamily 3, member A2	6	26473376	11118	6p22.1	Hs.376046
ITGA4	integrin, alpha 4 (antigen CD49D, alpha 4 subunit of VLA-4 receptor)	2	182029863	3676	2q31.3	Hs.440955
IL18RAP	interleukin 18 receptor accessory protein	2	102401685	8807	2p24.3-p24.1	Hs.158315
LCP2	lymphocyte cytosolic protein 2 (SH2 domain containing leukocyte protein of 76kDa)	5	-169607666	3937	5q33.1-qter	Hs.304475
FLT3LG	fms-related tyrosine kinase 3 ligand	19	54669297	2323	19q13.3	Hs.428
ISG20	interferon stimulated exonuclease gene 20kDa	15	86983042	3669	15q26	Hs.459265
ZAP70	zeta-chain (TCR) associated protein kinase 70kDa	2	97696462, 97717300	7535	2q12	Hs.234569
NSUN5B	NOL1/NOP2/Sun domain family, member 5B	7	74877581	155400	7q11.23	Hs.632322
PLAC8	placenta-specific 8	4	-84230866	51316	4q21.22	Hs.546392
RUNX3	runt-related transcription factor 3	1	-25098589	864	1p36	Hs.170019
ICAM3	intercellular adhesion molecule 3	19	-10305453	3385	19p13.3-p13.2	Hs.75516
CD52	CD52 molecule	1	26516997	1043	1p36	Hs.276770
IL32	interleukin 32	16	3055313, 3055663, 3055639, 3055612, 3055785	9235	16p13.3	Hs.943
CYLD	cylindromatosis (turban tumor syndrome)	16	49333461, 49333529	1540	16q12.1	Hs.578973
CD7	CD7 molecule	17	-77866034	924	17q25.2-q25.3	Hs.36972
ATM	ataxia telangiectasia mutated (includes complementation groups A, C and D)	11	107598768, 107663328	472	11q22-q23	Hs.435561
HECA	headcase homolog (Drosophila)	6	139497941	51696	6q23-q24	Hs.197644
FAIM3	Fas apoptotic inhibitory molecule 3	1	-205144353	9214	1q32.1	Hs.58831
ADCY7	adenylate cyclase 7	16	48879322	113	16q12-q13	Hs.513578
RAPGEF6	Rap guanine nucleotide exchange factor (GEF) 6	5	-130789502	51735	5q31.1	Hs.483329
SEMA4D	sema domain, immunoglobulin domain (Ig), transmembrane domain (TM) and short cytoplasmic domain, (semaphorin) 4D	9	-91181971	10507	9q22-q31	Hs.494406
RARRES3	retinoic acid receptor responder (tazarotene induced) 3	11	63060855	5920	11q23	Hs.17466
CD37	CD37 molecule	19	54530488	951	19q13.3	Hs.166556
GCH1	GTP cyclohydrolase 1 (dopa-responsive dystonia)	14	-54378475	2643	14q22.1-q22.2	Hs.86724
LRRFIP1	leucine rich repeat (in FLII) interacting protein 1	2	238265667	9208	2q37.3	Hs.471779
BIN2	bridging integrator 2	12	-49961089	51411	12q13	Hs.14770
STK10	serine/threonine kinase 10	5	-171403229	6793	5q35.1	Hs.519756
PTPRCAP	protein tyrosine phosphatase, receptor type, C-associated protein	11	-66959556	5790	11q13.3	Hs.155975
CD48	CD48 molecule	1	-158915161	962	1q21.3-q22	Hs.243564
TRIM22	tripartite motif-containing 22	11	5667663	10346	11p15	Hs.501778 Hs.621411
RNF138	ring finger protein 138	18	27925834, 27926644	51444	18q12.1	Hs.302408 Hs.501040
CORO1A	coronin, actin binding protein, 1A	16	30102426	11151	16p11.2	Hs.415067
LAT	linker for activation of T cells	16	28903887, 28903647	27040	16p11.2	Hs.632179

PRKCH	protein kinase C, eta	14	60858267	5583	14q22-q23	Hs.333907
ARL4C	ADP-ribosylation factor-like 4C	2	-235066426	10123	2q37.1	Hs.111554
ZFP36L2	zinc finger protein 36, C3H type-like 2	2	-43304210	678	2p22.3-p21	Hs.503093 Hs.636758
TRDC	T cell receptor delta constant	14		28526	14q11	
TRAT1	T cell receptor associated transmembrane adaptor 1	3	110024320	50852	3q13	Hs.138701
PTGER2	prostaglandin E receptor 2 (subtype EP2), 53kDa	14	51850862	5732	14q22	Hs.2090
WASPIP	Wiskott-Aldrich syndrome protein interacting protein	2	-175132548	7456	2q31.1	Hs.591641
HMHA1	histocompatibility (minor) HA-1	19	1018173	23526	19p13.3	Hs.465521
HCLS1	hematopoietic cell-specific Lyn substrate 1	3	-122832936	3059	3q13	Hs.14601
ERBB2IP	erbb2 interacting protein	5	65258139	55914	5q12.3	Hs.591774
COTL1	coactosin-like 1 (Dictyostelium)	16	-83156706	23406	16q24.1	Hs.289092
DUSP2	dual specificity phosphatase 2	2	-96172643	1844	2q11	Hs.1183
UBASH3A	ubiquitin associated and SH3 domain containing, A	21	42697087	53347	21q22.3	Hs.473912
TCF7	transcription factor 7 (T-cell specific, HMG-box)	5	133478300, 133479214, 133479325	6932	5q31.1	Hs.573153
KLRK1	killer cell lectin-like receptor subfamily K, member 1	12	-10416219	22914	12p13.2-p12.3	Hs.387787
TRBC1	T cell receptor beta constant 1	7		28639	7q34	Hs.511522
ALOX5AP	arachidonate 5-lipoxygenase-activating protein	13	30207668	241	13q12	Hs.507658
GBP1	guanylate binding protein 1, interferon-inducible, 67kDa	1	-89290593	2633	1p22.2	Hs.62661
FAM117A	family with sequence similarity 117, member A	17	-45142687	81558	17q21.33	Hs.514308
PNN	pinin, desmosome associated protein	14	38714150	5411	14q21.1	Hs.409965

15.2 GO: Biological Process

GO ID	genes present in chip	genes present in tissues	p-value	GO term
GO:0006952	751	31	1.50E-15	defense response
GO:0009607	857	32	7.54E-15	response to biotic stimulus
GO:0006955	684	28	7.56E-14	immune response
GO:0050896	1578	34	5.06E-09	response to stimulus
GO:0042110	45	7	5.42E-08	T cell activation
GO:0050857	3	3	4.86E-07	positive regulation of antigen receptor mediated signaling pathway
GO:0007154	2730	42	6.43E-07	cell communication
GO:0007165	2168	36	1.43E-06	signal transduction
GO:0050854	4	3	1.93E-06	regulation of antigen receptor mediated signaling pathway
GO:0050874	1531	29	2.17E-06	organismal physiological process
GO:0009613	458	15	2.40E-06	response to pest, pathogen or parasite
GO:0007166	952	22	2.58E-06	cell surface receptor linked signal transduction
GO:0043207	463	15	2.74E-06	response to external biotic stimulus
GO:0046649	81	7	3.31E-06	lymphocyte activation
GO:0050863	31	5	4.04E-06	regulation of T cell activation
GO:0050852	5	3	4.80E-06	T cell receptor signaling pathway
GO:0006968	87	7	5.35E-06	cellular defense response
GO:0030098	34	5	6.50E-06	lymphocyte differentiation
GO:0050851	6	3	9.55E-06	antigen receptor-mediated signaling pathway
GO:0030217	18	4	1.04E-05	T cell differentiation
GO:0045321	97	7	1.10E-05	immune cell activation
GO:0001775	98	7	1.18E-05	cell activation
GO:0051249	41	5	1.68E-05	regulation of lymphocyte activation
GO:0050870	21	4	2.01E-05	positive regulation of T cell activation
GO:0050865	43	5	2.13E-05	regulation of cell activation
GO:0050862	2	2	6.27E-05	positive regulation of T cell receptor signaling pathway
GO:0045059	2	2	6.27E-05	positive thymic T cell selection
GO:0030097	87	6	6.30E-05	hemopoiesis
GO:0051251	28	4	6.58E-05	positive regulation of lymphocyte activation
GO:0048534	88	6	6.72E-05	hemopoietic or lymphoid organ development
GO:0009611	341	11	7.24E-05	response to wounding
GO:0050867	29	4	7.58E-05	positive regulation of cell activation
GO:0007265	29	4	7.58E-05	Ras protein signal transduction
GO:0007242	879	18	0.000133592	intracellular signaling cascade
GO:0050856	3	2	0.000187043	regulation of T cell receptor signaling pathway
GO:0045061	3	2	0.000187043	thymic T cell selection
GO:0050776	77	5	0.000353967	regulation of immune response
GO:0051282	4	2	0.000372154	regulation of sequestering of calcium ion
GO:0051283	4	2	0.000372154	negative regulation of sequestering of calcium ion
GO:0051209	4	2	0.000372154	release of sequestered calcium ion into cytoplasm
GO:0009967	85	5	0.000559392	positive regulation of signal transduction
GO:0051208	5	2	0.000617055	sequestering of calcium ion
GO:0045058	6	2	0.000920808	T cell selection

GO:0050778	56	4	0.000997226	positive regulation of immune response
GO:0051240	59	4	0.001213706	positive regulation of organismal physiological process
GO:0006950	881	16	0.001280236	response to stress
GO:0045582	7	2	0.001282484	positive regulation of T cell differentiation
GO:0045621	7	2	0.001282484	positive regulation of lymphocyte differentiation
GO:0001906	8	2	0.001701167	cell killing
GO:0051238	8	2	0.001701167	sequestering of metal ion
GO:0006874	68	4	0.002057928	calcium ion homeostasis
GO:0042100	9	2	0.00217595	B cell proliferation
GO:0009966	229	7	0.002232646	regulation of signal transduction
GO:0045580	10	2	0.002705937	regulation of T cell differentiation
GO:0048519	688	13	0.002770868	negative regulation of biological process
GO:0007204	37	3	0.003107794	elevation of cytoplasmic calcium ion concentration
GO:0051480	37	3	0.003107794	cytoplasmic calcium ion homeostasis
GO:0009605	963	16	0.003214286	response to external stimulus
GO:0045619	12	2	0.00392799	regulation of lymphocyte differentiation
GO:0006915	471	10	0.003946322	apoptosis
GO:0012501	473	10	0.004066758	programmed cell death
GO:0051239	133	5	0.004081915	regulation of organismal physiological process
GO:0030005	87	4	0.005022009	di-, tri-valent inorganic cation homeostasis
GO:0019835	14	2	0.005360366	cytolysis
GO:0007229	45	3	0.005423431	integrin-mediated signaling pathway
GO:0008219	498	10	0.005834041	cell death
GO:0016265	501	10	0.006081392	death
GO:0051243	583	11	0.006087062	negative regulation of cellular physiological process
GO:0006875	93	4	0.006356372	metal ion homeostasis
GO:0030003	98	4	0.007633774	cation homeostasis
GO:0043118	602	11	0.00769796	negative regulation of physiological process
GO:0019722	17	2	0.007888448	calcium-mediated signaling
GO:0001881	1	1	0.007971656	receptor recycling
GO:0031342	1	1	0.007971656	negative regulation of cell killing
GO:0048305	1	1	0.007971656	immunoglobulin secretion
GO:0045175	1	1	0.007971656	basal protein localization
GO:0006926	1	1	0.007971656	programmed cell death, virus-infected cells
GO:0001920	1	1	0.007971656	negative regulation of receptor recycling
GO:0001911	1	1	0.007971656	negative regulation of immune cell mediated cytotoxicity
GO:0001913	1	1	0.007971656	T cell mediated cytotoxicity
GO:0001914	1	1	0.007971656	regulation of T cell mediated cytotoxicity
GO:0001915	1	1	0.007971656	negative regulation of T cell mediated cytotoxicity
GO:0001919	1	1	0.007971656	regulation of receptor recycling
GO:0019724	1	1	0.007971656	B cell mediated immunity
GO:0045579	1	1	0.007971656	positive regulation of B cell differentiation
GO:0050850	1	1	0.007971656	positive regulation of calcium-mediated signaling
GO:0050853	1	1	0.007971656	B cell receptor signaling pathway
GO:0050848	1	1	0.007971656	regulation of calcium-mediated signaling
GO:0000738	1	1	0.007971656	DNA catabolism, exonucleolytic
GO:0006882	1	1	0.007971656	zinc ion homeostasis

GO:0045597	18	2	0.008829035	positive regulation of cell differentiation
GO:0016064	103	4	0.009070073	humoral defense mechanism (sensu Vertebrata)
GO:0006873	103	4	0.009070073	cell ion homeostasis
GO:0042981	306	7	0.010718501	regulation of apoptosis
GO:0009615	58	3	0.010956335	response to virus
GO:0043067	308	7	0.011083838	regulation of programmed cell death
GO:0050801	111	4	0.011716954	ion homeostasis
GO:0048523	640	11	0.011913416	negative regulation of cellular process
GO:0006793	641	11	0.01204443	phosphorus metabolism
GO:0006796	641	11	0.01204443	phosphate metabolism
GO:0006916	112	4	0.012079141	anti-apoptosis
GO:0042221	183	5	0.015151017	response to chemical substance
GO:0006954	185	5	0.01581336	inflammatory response
GO:0031341	2	1	0.015880641	regulation of cell killing
GO:0006922	2	1	0.015880641	cleavage of lamin
GO:0006923	2	1	0.015880641	cleavage of cytoskeletal proteins
GO:0006927	2	1	0.015880641	programmed cell death, transformed cells
GO:0001910	2	1	0.015880641	regulation of immune cell mediated cytotoxicity
GO:0001907	2	1	0.015880641	killing of host cells
GO:0048247	2	1	0.015880641	lymphocyte chemotaxis
GO:0019836	2	1	0.015880641	hemolysis
GO:0044003	2	1	0.015880641	modification of host morphology or physiology
GO:0044004	2	1	0.015880641	disruption of host cells
GO:0001897	2	1	0.015880641	cytolysis of host cells
GO:0019725	122	4	0.01610091	cell homeostasis
GO:0051235	25	2	0.016701824	maintenance of localization
GO:0044419	26	2	0.018001375	interaction between organisms
GO:0043066	128	4	0.018874653	negative regulation of apoptosis
GO:0043069	129	4	0.019364009	negative regulation of programmed cell death
GO:0046651	28	2	0.020724184	lymphocyte proliferation
GO:0051348	28	2	0.020724184	negative regulation of transferase activity
GO:0006469	28	2	0.020724184	negative regulation of protein kinase activity
GO:0019735	77	3	0.023338529	antimicrobial humoral response (sensu Vertebrata)
GO:0042113	30	2	0.02360715	B cell activation
GO:0043303	3	1	0.02372744	mast cell degranulation
GO:0045103	3	1	0.02372744	intermediate filament-based process
GO:0045104	3	1	0.02372744	intermediate filament cytoskeleton organization and biogenesis
GO:0008105	3	1	0.02372744	asymmetric protein localization
GO:0043299	3	1	0.02372744	immune cell degranulation
GO:0045577	3	1	0.02372744	regulation of B cell differentiation
GO:0008588	3	1	0.02372744	release of cytoplasmic sequestered NF-kappaB
GO:0050680	3	1	0.02372744	negative regulation of epithelial cell proliferation
GO:0042531	3	1	0.02372744	positive regulation of tyrosine phosphorylation of STAT protein
GO:0046427	3	1	0.02372744	positive regulation of JAK-STAT cascade
GO:0019730	78	3	0.024136246	antimicrobial humoral response
GO:0006468	444	8	0.024356715	protein amino acid phosphorylation
GO:0051094	31	2	0.025106928	positive regulation of development

GO:0006917	140	4	0.025269971	induction of apoptosis
GO:0012502	140	4	0.025269971	induction of programmed cell death
GO:0006959	141	4	0.025855139	humoral immune response
GO:0050790	214	5	0.027673128	regulation of enzyme activity
GO:0045786	145	4	0.028277497	negative regulation of progression through cell cycle
GO:0045197	4	1	0.031512535	establishment and/or maintenance of epithelial cell polarity
GO:0035088	4	1	0.031512535	establishment and/or maintenance of apical/basal cell polarity
GO:0043112	4	1	0.031512535	receptor metabolism
GO:0000018	4	1	0.031512535	regulation of DNA recombination
GO:0042346	4	1	0.031512535	positive regulation of NF-kappaB-nucleus import
GO:0042307	4	1	0.031512535	positive regulation of protein-nucleus import
GO:0051222	4	1	0.031512535	positive regulation of protein transport
GO:0042993	4	1	0.031512535	positive regulation of transcription factor-nucleus import
GO:0008283	473	8	0.033795616	cell proliferation
GO:0007264	227	5	0.034447505	small GTPase mediated signal transduction
GO:0048518	565	9	0.034690142	positive regulation of biological process
GO:0043065	155	4	0.034911295	positive regulation of apoptosis
GO:0043068	156	4	0.035620461	positive regulation of programmed cell death
GO:0042592	157	4	0.036337993	homeostasis
GO:0048522	482	8	0.037175104	positive regulation of cellular process
GO:0045576	5	1	0.039236405	mast cell activation
GO:0030323	5	1	0.039236405	respiratory tube development
GO:0046146	5	1	0.039236405	tetrahydrobiopterin metabolism
GO:0006729	5	1	0.039236405	tetrahydrobiopterin biosynthesis
GO:0046824	5	1	0.039236405	positive regulation of nucleocytoplasmic transport
GO:0051247	41	2	0.042081522	positive regulation of protein metabolism
GO:0030154	409	7	0.043678354	cell differentiation
GO:0006470	99	3	0.044251365	protein amino acid dephosphorylation
GO:0051242	411	7	0.044647832	positive regulation of cellular physiological process
GO:0006935	101	3	0.04649601	chemotaxis
GO:0042330	101	3	0.04649601	taxis
GO:0001909	6	1	0.046899524	immune cell mediated cytotoxicity
GO:0050871	6	1	0.046899524	positive regulation of B cell activation
GO:0046330	6	1	0.046899524	positive regulation of JNK cascade
GO:0019932	176	4	0.051565376	second-messenger-mediated signaling
GO:0043086	46	2	0.051788949	negative regulation of enzyme activity
GO:0051049	46	2	0.051788949	regulation of transport
GO:0007155	518	8	0.052977274	cell adhesion
GO:0043119	428	7	0.053456292	positive regulation of physiological process
GO:0043085	107	3	0.053561913	positive regulation of enzyme activity
GO:0050673	7	1	0.054502364	epithelial cell proliferation
GO:0050678	7	1	0.054502364	regulation of epithelial cell proliferation
GO:0042509	7	1	0.054502364	regulation of tyrosine phosphorylation of STAT protein
GO:0046425	7	1	0.054502364	regulation of JAK-STAT cascade
GO:0016310	522	8	0.05496673	phosphorylation
GO:0016311	110	3	0.057278465	dephosphorylation
GO:0042127	265	5	0.059795513	regulation of cell proliferation

GO:0050789	2689	28	0.060747132	regulation of biological process
GO:0006558	8	1	0.062045393	L-phenylalanine metabolism
GO:0006559	8	1	0.062045393	L-phenylalanine catabolism
GO:0050832	8	1	0.062045393	defense response to fungi
GO:0045078	8	1	0.062045393	positive regulation of interferon-gamma biosynthesis
GO:0007169	116	3	0.065069353	transmembrane receptor protein tyrosine kinase signaling pathway
GO:0008632	53	2	0.066562581	apoptotic program
GO:0050794	2488	26	0.069358247	regulation of cellular process
GO:0050731	9	1	0.069529073	positive regulation of peptidyl-tyrosine phosphorylation
GO:0050864	9	1	0.069529073	regulation of B cell activation
GO:0045595	55	2	0.071013974	regulation of cell differentiation
GO:0006887	55	2	0.071013974	exocytosis
GO:0008284	121	3	0.071916355	positive regulation of cell proliferation
GO:0019438	10	1	0.076953866	aromatic compound biosynthesis
GO:0001934	10	1	0.076953866	positive regulation of protein amino acid phosphorylation
GO:0045764	10	1	0.076953866	positive regulation of amino acid metabolism
GO:0009074	10	1	0.076953866	aromatic amino acid family catabolism
GO:0042345	10	1	0.076953866	regulation of NF-kappaB-nucleus import
GO:0042348	10	1	0.076953866	NF-kappaB-nucleus import
GO:0009405	10	1	0.076953866	pathogenesis
GO:0007260	10	1	0.076953866	tyrosine phosphorylation of STAT protein
GO:0042559	10	1	0.076953866	pteridine and derivative biosynthesis
GO:0006928	205	4	0.080576975	cell motility
GO:0040011	205	4	0.080576975	locomotion
GO:0051336	60	2	0.082547878	regulation of hydrolase activity
GO:0006921	11	1	0.084320228	disassembly of cell structures during apoptosis
GO:0042558	11	1	0.084320228	pteridine and derivative metabolism
GO:0009620	11	1	0.084320228	response to fungi
GO:0046328	11	1	0.084320228	regulation of JNK cascade
GO:0006171	11	1	0.084320228	cAMP biosynthesis
GO:0045072	11	1	0.084320228	regulation of interferon-gamma biosynthesis
GO:0006310	61	2	0.084920191	DNA recombination
GO:0007626	209	4	0.085107828	locomotory behavior
GO:0006464	1087	13	0.086572849	protein modification
GO:0019370	12	1	0.091628614	leukotriene biosynthesis
GO:0042095	12	1	0.091628614	interferon-gamma biosynthesis
GO:0035295	12	1	0.091628614	tube development
GO:0042327	12	1	0.091628614	positive regulation of phosphorylation
GO:0045937	12	1	0.091628614	positive regulation of phosphate metabolism
GO:0042102	12	1	0.091628614	positive regulation of T cell proliferation
GO:0000074	394	6	0.093351477	regulation of progression through cell cycle
GO:0051056	65	2	0.094611963	regulation of small GTPase mediated signal transduction
GO:0019439	13	1	0.098879472	aromatic compound catabolism
GO:0046058	13	1	0.098879472	cAMP metabolism
GO:0030218	13	1	0.098879472	erythrocyte differentiation
GO:0030595	13	1	0.098879472	immune cell chemotaxis
GO:0007049	601	8	0.104287352	cell cycle

GO:0006691	14	1	0.106073249	leukotriene metabolism
GO:0009725	14	1	0.106073249	response to hormone stimulus
GO:0050900	14	1	0.106073249	immune cell migration
GO:0006308	14	1	0.106073249	DNA catabolism
GO:0030183	14	1	0.106073249	B cell differentiation
GO:0009628	318	5	0.109117075	response to abiotic stimulus
GO:0043407	15	1	0.113210389	negative regulation of MAPK activity
GO:0051050	15	1	0.113210389	positive regulation of transport
GO:0050730	15	1	0.113210389	regulation of peptidyl-tyrosine phosphorylation
GO:0042129	15	1	0.113210389	regulation of T cell proliferation
GO:0000188	15	1	0.113210389	inactivation of MAPK activity
GO:0042990	15	1	0.113210389	regulation of transcription factor-nucleus import
GO:0042991	15	1	0.113210389	transcription factor-nucleus import
GO:0006809	16	1	0.120291331	nitric oxide biosynthesis
GO:0001932	16	1	0.120291331	regulation of protein amino acid phosphorylation
GO:0042306	16	1	0.120291331	regulation of protein-nucleus import
GO:0007173	16	1	0.120291331	epidermal growth factor receptor signaling pathway
GO:0046209	16	1	0.120291331	nitric oxide metabolism
GO:0043412	1160	13	0.126883899	biopolymer modification
GO:0007243	242	4	0.127003603	protein kinase cascade
GO:0006521	17	1	0.127316511	regulation of amino acid metabolism
GO:0042098	17	1	0.127316511	T cell proliferation
GO:0051051	17	1	0.127316511	negative regulation of transport
GO:0051223	17	1	0.127316511	regulation of protein transport
GO:0031281	18	1	0.134286362	positive regulation of cyclase activity
GO:0045762	18	1	0.134286362	positive regulation of adenylate cyclase activity
GO:0009072	18	1	0.134286362	aromatic amino acid family metabolism
GO:0007190	18	1	0.134286362	adenylate cyclase activation
GO:0042133	18	1	0.134286362	neurotransmitter metabolism
GO:0050671	18	1	0.134286362	positive regulation of lymphocyte proliferation
GO:0051349	18	1	0.134286362	positive regulation of lyase activity
GO:0000165	81	2	0.136127058	MAPKKK cascade
GO:0051244	2319	23	0.139094696	regulation of cellular physiological process
GO:0007131	19	1	0.141201315	meiotic recombination
GO:0018108	19	1	0.141201315	peptidyl-tyrosine phosphorylation
GO:0007167	165	3	0.144293244	enzyme linked receptor protein signaling pathway
GO:0046916	20	1	0.148061795	transition metal ion homeostasis
GO:0042493	20	1	0.148061795	response to drug
GO:0009190	20	1	0.148061795	cyclic nucleotide biosynthesis
GO:0007205	20	1	0.148061795	protein kinase C activation
GO:0044403	20	1	0.148061795	symbiosis, mutualism through parasitism
GO:0044404	20	1	0.148061795	symbiotic interaction between host and other organism
GO:0007163	21	1	0.154868225	establishment and/or maintenance of cell polarity
GO:0018212	21	1	0.154868225	peptidyl-tyrosine modification
GO:0007189	22	1	0.161621026	G-protein signaling, adenylate cyclase activating pathway
GO:0007127	22	1	0.161621026	meiosis I
GO:0050670	22	1	0.161621026	regulation of lymphocyte proliferation

GO:0045055	22	1	0.161621026	regulated secretory pathway
GO:0007610	269	4	0.166610395	behavior
GO:0006805	23	1	0.168320614	xenobiotic metabolism
GO:0042108	23	1	0.168320614	positive regulation of cytokine biosynthesis
GO:0046456	23	1	0.168320614	icosanoid biosynthesis
GO:0046822	23	1	0.168320614	regulation of nucleocytoplasmic transport
GO:0042088	24	1	0.174967402	T-helper 1 type immune response
GO:0045727	25	1	0.181561801	positive regulation of protein biosynthesis
GO:0042087	25	1	0.181561801	cell-mediated immune response
GO:0042325	25	1	0.181561801	regulation of phosphorylation
GO:0050791	2407	23	0.186455545	regulation of physiological process
GO:0031328	26	1	0.188104218	positive regulation of cellular biosynthesis
GO:0009306	26	1	0.188104218	protein secretion
GO:0009410	26	1	0.188104218	response to xenobiotic stimulus
GO:0006919	27	1	0.194595057	caspase activation
GO:0016066	27	1	0.194595057	cellular defense response (sensu Vertebrata)
GO:0009187	27	1	0.194595057	cyclic nucleotide metabolism
GO:0007186	491	6	0.196143348	G-protein coupled receptor protein signaling pathway
GO:0043087	28	1	0.201034719	regulation of GTPase activity
GO:0019220	28	1	0.201034719	regulation of phosphate metabolism
GO:0007259	28	1	0.201034719	JAK-STAT cascade
GO:0051174	28	1	0.201034719	regulation of phosphorus metabolism
GO:0001819	29	1	0.207423602	positive regulation of cytokine production
GO:0051052	29	1	0.207423602	regulation of DNA metabolism
GO:0009891	30	1	0.2137621	positive regulation of biosynthesis
GO:0043280	31	1	0.220050606	positive regulation of caspase activity
GO:0043281	31	1	0.220050606	regulation of caspase activity
GO:0031279	32	1	0.226289508	regulation of cyclase activity
GO:0045761	32	1	0.226289508	regulation of adenylate cyclase activity
GO:0051345	32	1	0.226289508	positive regulation of hydrolase activity
GO:0051339	32	1	0.226289508	regulation of lyase activity
GO:0046903	208	3	0.230308963	secretion
GO:0006690	33	1	0.232479192	icosanoid metabolism
GO:0042035	33	1	0.232479192	regulation of cytokine biosynthesis
GO:0030155	33	1	0.232479192	regulation of cell adhesion
GO:0006401	33	1	0.232479192	RNA catabolism
GO:0045859	116	2	0.236254383	regulation of protein kinase activity
GO:0042742	34	1	0.238620041	defense response to bacteria
GO:0051338	117	2	0.23920814	regulation of transferase activity
GO:0042089	36	1	0.250756749	cytokine biosynthesis
GO:0042107	37	1	0.256753361	cytokine metabolism
GO:0030099	37	1	0.256753361	myeloid cell differentiation
GO:0006487	37	1	0.256753361	protein amino acid N-linked glycosylation
GO:0030036	126	2	0.265859742	actin cytoskeleton organization and biogenesis
GO:0001817	39	1	0.268604953	regulation of cytokine production
GO:0001505	41	1	0.280270146	regulation of neurotransmitter levels
GO:0001816	42	1	0.286033749	cytokine production

GO:0009063	42	1	0.286033749	amino acid catabolism
GO:0007254	42	1	0.286033749	JNK cascade
GO:0031098	42	1	0.286033749	stress-activated protein kinase signaling pathway
GO:0031325	136	2	0.295490444	positive regulation of cellular metabolism
GO:0006979	44	1	0.297424751	response to oxidative stress
GO:0007126	44	1	0.297424751	meiosis
GO:0051321	44	1	0.297424751	meiotic cell cycle
GO:0051327	44	1	0.297424751	M phase of meiotic cell cycle
GO:0008154	45	1	0.303052857	actin polymerization and/or depolymerization
GO:0009310	45	1	0.303052857	amine catabolism
GO:0008285	139	2	0.30435743	negative regulation of cell proliferation
GO:0030029	139	2	0.30435743	actin filament-based process
GO:0044267	2109	19	0.311820335	cellular protein metabolism
GO:0006633	47	1	0.314176024	fatty acid biosynthesis
GO:0044270	47	1	0.314176024	nitrogen compound catabolism
GO:0043405	49	1	0.325124095	regulation of MAPK activity
GO:0019538	2129	19	0.327980312	protein metabolism
GO:0018193	50	1	0.330533319	peptidyl-amino acid modification
GO:0009893	149	2	0.333760615	positive regulation of metabolism
GO:0009617	51	1	0.335899786	response to bacteria
GO:0006606	53	1	0.346505774	protein-nucleus import
GO:0045860	53	1	0.346505774	positive regulation of protein kinase activity
GO:0016053	54	1	0.351745955	organic acid biosynthesis
GO:0051347	54	1	0.351745955	positive regulation of transferase activity
GO:0051170	54	1	0.351745955	nuclear import
GO:0046394	54	1	0.351745955	carboxylic acid biosynthesis
GO:0043170	2405	21	0.354511886	macromolecule metabolism
GO:0007200	56	1	0.362102318	G-protein signaling, coupled to IP3 second messenger (phospholipase C activating)
GO:0045045	161	2	0.36858127	secretory pathway
GO:0007188	59	1	0.377331678	G-protein signaling, coupled to cAMP nucleotide second messenger
GO:0019933	60	1	0.382328013	cAMP-mediated signaling
GO:0006800	61	1	0.38728481	oxygen and reactive oxygen species metabolism
GO:0050793	168	2	0.388582818	regulation of development
GO:0048513	872	8	0.393428978	organ development
GO:0017038	64	1	0.401921043	protein import
GO:0006364	64	1	0.401921043	rRNA processing
GO:0000279	173	2	0.402704898	M phase
GO:0051246	174	2	0.405511655	regulation of protein metabolism
GO:0016072	66	1	0.411486433	rRNA metabolism
GO:0006816	68	1	0.420900929	calcium ion transport
GO:0006520	181	2	0.424984136	amino acid metabolism
GO:0048015	71	1	0.434744874	phosphoinositide-mediated signaling
GO:0007010	305	3	0.440801221	cytoskeleton organization and biogenesis
GO:0006725	73	1	0.443792328	aromatic compound metabolism
GO:0006461	194	2	0.460269955	protein complex assembly
GO:0007046	79	1	0.47008655	ribosome biogenesis
GO:0044260	2298	19	0.471643823	cellular macromolecule metabolism

GO:0042254	80	1	0.474348112	ribosome biogenesis and assembly
GO:0016477	85	1	0.495154191	cell migration
GO:0009987	8200	66	0.500676027	cellular process
GO:0016055	87	1	0.50324733	Wnt receptor signaling pathway
GO:0000004	465	4	0.51172776	biological process unknown
GO:0009719	214	2	0.512074922	response to endogenous stimulus
GO:0006519	216	2	0.517078639	amino acid and derivative metabolism
GO:0009892	217	2	0.519568051	negative regulation of metabolism
GO:0006486	92	1	0.522924678	protein amino acid glycosylation
GO:0043413	93	1	0.526766896	biopolymer glycosylation
GO:0043283	2240	18	0.529848678	biopolymer metabolism
GO:0007187	94	1	0.530578595	G-protein signaling, coupled to cyclic nucleotide second messenger
GO:0007028	96	1	0.538111396	cytoplasm organization and biogenesis
GO:0009101	96	1	0.538111396	glycoprotein biosynthesis
GO:0051169	96	1	0.538111396	nuclear transport
GO:0019935	98	1	0.54552497	cyclic-nucleotide-mediated signaling
GO:0015674	99	1	0.54918763	di-, tri-valent inorganic cation transport
GO:0007249	99	1	0.54918763	I-kappaB kinase/NF-kappaB cascade
GO:0019752	359	3	0.550034318	carboxylic acid metabolism
GO:0006082	361	3	0.553848121	organic acid metabolism
GO:0006913	102	1	0.560001854	nucleocytoplasmic transport
GO:0009165	103	1	0.563549431	nucleotide biosynthesis
GO:0006511	105	1	0.57056018	ubiquitin-dependent protein catabolism
GO:0048232	105	1	0.57056018	male gamete generation
GO:0007283	105	1	0.57056018	spermatogenesis
GO:0019941	105	1	0.57056018	modification-dependent protein catabolism
GO:0006357	240	2	0.57447919	regulation of transcription from RNA polymerase II promoter
GO:0044249	763	6	0.575725632	cellular biosynthesis
GO:0006417	107	1	0.577459854	regulation of protein biosynthesis
GO:0031326	111	1	0.590932892	regulation of cellular biosynthesis
GO:0009100	111	1	0.590932892	glycoprotein metabolism
GO:0009889	116	1	0.607179742	regulation of biosynthesis
GO:0008361	123	1	0.628861281	regulation of cell size
GO:0016049	123	1	0.628861281	cell growth
GO:0006631	124	1	0.631860719	fatty acid metabolism
GO:0007276	125	1	0.63483625	gametogenesis
GO:0006412	407	3	0.636196302	protein biosynthesis
GO:0009308	269	2	0.637143927	amine metabolism
GO:0006996	693	5	0.656642183	organelle organization and biogenesis
GO:0006605	134	1	0.660570755	protein targeting
GO:0007067	135	1	0.663317316	mitosis
GO:0006366	426	3	0.667053754	transcription from RNA polymerase II promoter
GO:0000087	137	1	0.668744858	M phase of mitotic cell cycle
GO:0006807	291	2	0.679786698	nitrogen compound metabolism
GO:0009117	143	1	0.684515076	nucleotide metabolism
GO:0016192	296	2	0.688900742	vesicle-mediated transport
GO:0009058	859	6	0.692728594	biosynthesis

GO:0019953	149	1	0.69954445	sexual reproduction
GO:0016481	151	1	0.704395589	negative regulation of transcription
GO:0040007	154	1	0.711527824	growth
GO:0045934	159	1	0.723039629	negative regulation of nucleobase, nucleoside, nucleotide and nucleic acid metabolism
GO:0009059	464	3	0.723078011	macromolecule biosynthesis
GO:0007267	465	3	0.724450416	cell-cell signaling
GO:0006457	169	1	0.744721056	protein folding
GO:0016337	172	1	0.750892741	cell-cell adhesion
GO:0006281	174	1	0.754925213	DNA repair
GO:0006259	489	3	0.755850714	DNA metabolism
GO:0008610	175	1	0.756917241	lipid biosynthesis
GO:0031324	183	1	0.772289075	negative regulation of cellular metabolism
GO:0000902	183	1	0.772289075	cellular morphogenesis
GO:0019222	1629	11	0.773738001	regulation of metabolism
GO:0007268	192	1	0.78843857	synaptic transmission
GO:0019226	198	1	0.798571881	transmission of nerve impulse
GO:0006974	200	1	0.801842096	response to DNA damage stimulus
GO:0000278	204	1	0.808226251	mitotic cell cycle
GO:0016043	842	5	0.813699085	cell organization and biogenesis
GO:0000003	208	1	0.814407491	reproduction
GO:0044248	700	4	0.8195767	cellular catabolism
GO:0031323	1566	10	0.823547297	regulation of cellular metabolism
GO:0016070	391	2	0.825208816	RNA metabolism
GO:0007001	221	1	0.833170689	chromosome organization and biogenesis (sensu Eukaryota)
GO:0044265	563	3	0.835139746	cellular macromolecule catabolism
GO:0019219	1458	9	0.842877018	regulation of nucleobase, nucleoside, nucleotide and nucleic acid metabolism
GO:0009057	575	3	0.845709041	macromolecule catabolism
GO:0051276	236	1	0.85250485	chromosome organization and biogenesis
GO:0007582	7846	60	0.85646506	physiological process
GO:0009056	756	4	0.863624839	catabolism
GO:0006355	1359	8	0.867604453	regulation of transcription, DNA-dependent
GO:0006512	255	1	0.873850292	ubiquitin cycle
GO:0006886	257	1	0.875911597	intracellular protein transport
GO:0044238	4820	34	0.87845138	primary metabolism
GO:0006508	451	2	0.881298848	proteolysis and peptidolysis
GO:0044257	451	2	0.881298848	cellular protein catabolism
GO:0007275	1543	9	0.887590157	development
GO:0030001	269	1	0.887599965	metal ion transport
GO:0030163	463	2	0.890336228	protein catabolism
GO:0006351	1407	8	0.891680056	transcription, DNA-dependent
GO:0008104	474	2	0.898064515	protein localization
GO:0043285	474	2	0.898064515	biopolymer catabolism
GO:0045449	1433	8	0.903168477	regulation of transcription
GO:0044237	5026	35	0.907146484	cellular metabolism
GO:0006350	1499	8	0.927910633	transcription
GO:0006396	326	1	0.92987485	RNA processing
GO:0008152	5262	36	0.938273605	metabolism

GO:0006812	346	1	0.940616981	cation transport
GO:0005975	355	1	0.944904947	carbohydrate metabolism
GO:0006139	2328	13	0.953914033	nucleobase, nucleoside, nucleotide and nucleic acid metabolism
GO:0044255	383	1	0.956385618	cellular lipid metabolism
GO:0007399	385	1	0.957108787	neurogenesis
GO:0050875	7018	50	0.962691204	cellular physiological process
GO:0015031	439	1	0.972730918	protein transport
GO:0051179	1857	9	0.973488325	localization
GO:0046907	448	1	0.974720591	intracellular transport
GO:0045184	459	1	0.976958841	establishment of protein localization
GO:0006629	469	1	0.978823366	lipid metabolism
GO:0050877	475	1	0.979869811	neurophysiological process
GO:0006811	515	1	0.985654243	ion transport
GO:0009887	807	2	0.99064413	organogenesis
GO:0009653	1051	3	0.992742043	morphogenesis
GO:0006810	1802	6	0.998023651	transport
GO:0051234	1841	6	0.998485127	establishment of localization
GO:0008150	9032	72	1	biological_process

15.3 GO: Molecular Function

GO ID	genes present in chip	genes present in tissues	p-value	GO term
GO:0004871	1837	27	9.72695779351921e-05	signal transducer activity
GO:0004715	15	3	0.000154782465409647	non-membrane spanning protein tyrosine kinase activity
GO:0004872	1060	17	0.00114777588073991	receptor activity
GO:0016493	12	2	0.00324655656074809	C-C chemokine receptor activity
GO:0019957	12	2	0.00324655656074809	C-C chemokine binding
GO:0005102	528	10	0.00440734984568513	receptor binding
GO:0008310	1	1	0.00723194633686175	single-stranded DNA specific 3'-5' exodeoxyribonuclease activity
GO:0008859	1	1	0.00723194633686175	exoribonuclease II activity
GO:0003933	1	1	0.00723194633686175	GTP cyclohydrolase activity
GO:0003934	1	1	0.00723194633686175	GTP cyclohydrolase I activity
GO:0042610	1	1	0.00723194633686175	CD8 receptor binding
GO:0004277	1	1	0.00723194633686175	granzyme A activity
GO:0005125	181	5	0.0098157401008993	cytokine activity
GO:0004721	118	4	0.0103651276581568	phosphoprotein phosphatase activity
GO:0004888	694	11	0.0105148503768692	transmembrane receptor activity
GO:0001637	22	2	0.0108457561235989	G-protein chemoattractant receptor activity
GO:0004950	22	2	0.0108457561235989	chemokine receptor activity
GO:0019955	65	3	0.0114853132164413	cytokine binding
GO:0019956	24	2	0.0128387722994728	chemokine binding
GO:0030676	2	1	0.0144123442092685	Rac guanyl-nucleotide exchange factor activity
GO:0008330	2	1	0.0144123442092685	protein tyrosine/threonine phosphatase activity
GO:0008297	2	1	0.0144123442092685	single-stranded DNA specific exodeoxyribonuclease activity
GO:0004917	2	1	0.0144123442092685	interleukin-7 receptor activity
GO:0019982	2	1	0.0144123442092685	interleukin-7 binding
GO:0051117	2	1	0.0144123442092685	ATPase binding
GO:0042609	2	1	0.0144123442092685	CD4 receptor binding
GO:0005178	26	2	0.0149785105269502	integrin binding
GO:0004725	75	3	0.0168714261549980	protein tyrosine phosphatase activity
GO:0005176	3	1	0.0215415556831711	ErbB-2 class receptor binding
GO:0001948	3	1	0.0215415556831711	glycoprotein binding
GO:0008296	3	1	0.0215415556831711	3'-5'-exodeoxyribonuclease activity
GO:0042608	3	1	0.0215415556831711	T cell receptor binding
GO:0019899	151	4	0.0235613149476492	enzyme binding
GO:0017016	4	1	0.0286199403191389	Ras GTPase binding
GO:0019238	4	1	0.0286199403191389	cyclohydrolase activity
GO:0004713	161	4	0.0289434452737689	protein-tyrosine kinase activity
GO:0005085	93	3	0.0295876427771032	guanyl-nucleotide exchange factor activity
GO:0016301	609	9	0.0304972078219276	kinase activity
GO:0016791	171	4	0.0350030573773421	phosphoric monoester hydrolase activity
GO:0004960	5	1	0.0356478551894311	thromboxane receptor activity
GO:0004957	5	1	0.0356478551894311	prostaglandin E receptor activity
GO:0005522	5	1	0.0356478551894311	profilin binding
GO:0005088	5	1	0.0356478551894311	Ras guanyl-nucleotide exchange factor activity

GO:0019992	42	2	0.0368562611424668	diacylglycerol binding
GO:0016773	532	8	0.0372829785517668	phosphotransferase activity, alcohol group as acceptor
GO:0005529	103	3	0.0383335596183218	sugar binding
GO:0004672	447	7	0.0419142915770134	protein kinase activity
GO:0043027	6	1	0.0426256548949608	caspase inhibitor activity
GO:0003779	189	4	0.0476441865888174	actin binding
GO:0004529	7	1	0.0495536915821386	exodeoxyribonuclease activity
GO:0016895	7	1	0.0495536915821386	exodeoxyribonuclease activity, producing 5'-phosphomonoesters
GO:0030742	7	1	0.0495536915821386	GTP-dependent protein binding
GO:0005515	3709	34	0.0501892782174286	protein binding
GO:0017017	8	1	0.056432314959613	MAP kinase phosphatase activity
GO:0004955	8	1	0.056432314959613	prostaglandin receptor activity
GO:0019901	54	2	0.0579794976114152	protein kinase binding
GO:0016494	9	1	0.0632618723148821	C-X-C chemokine receptor activity
GO:0004016	9	1	0.0632618723148821	adenylate cyclase activity
GO:0008289	298	5	0.0639816665826667	lipid binding
GO:0005488	7228	58	0.0657023964197181	binding
GO:0016772	708	9	0.0677595904955308	transferase activity, transferring phosphorus-containing groups
GO:0019900	60	2	0.0697801097453845	kinase binding
GO:0001565	10	1	0.0700427085308158	phorbol ester receptor activity
GO:0004697	10	1	0.0700427085308158	protein kinase C activity
GO:0004953	10	1	0.0700427085308158	icosanoid receptor activity
GO:0004954	10	1	0.0700427085308158	prostanoid receptor activity
GO:0019958	10	1	0.0700427085308158	C-X-C chemokine binding
GO:0000175	10	1	0.0700427085308158	3'-5'-exoribonuclease activity
GO:0004532	11	1	0.0767751661020434	exoribonuclease activity
GO:0016896	11	1	0.0767751661020434	exoribonuclease activity, producing 5'-phosphomonoesters
GO:0042803	64	2	0.0780465673507144	protein homodimerization activity
GO:0042578	228	4	0.0826221407147852	phosphoric ester hydrolase activity
GO:0004143	12	1	0.0834595851512534	diacylglycerol kinase activity
GO:0005083	144	3	0.0859935158295405	small GTPase regulator activity
GO:0042169	14	1	0.0966856564115948	SH2 domain binding
GO:0016788	446	6	0.102619274915753	hydrolase activity, acting on ester bonds
GO:0042802	158	3	0.106156473641075	protein self binding
GO:0016796	17	1	0.116172842854326	exonuclease activity, active with either ribo- or deoxyribonucleic acids and producing 5'-phosphomonoesters
GO:0016849	18	1	0.122576042543853	phosphorus-oxygen lyase activity
GO:0046983	170	3	0.124777365030451	protein dimerization activity
GO:0005001	19	1	0.128933519501164	transmembrane receptor protein tyrosine phosphatase activity
GO:0043028	19	1	0.128933519501164	caspase regulator activity
GO:0019198	19	1	0.128933519501164	transmembrane receptor protein phosphatase activity
GO:0005164	20	1	0.135245595446808	tumor necrosis factor receptor binding
GO:0009975	20	1	0.135245595446808	cyclase activity
GO:0016814	21	1	0.141512589871151	hydrolase activity, acting on carbon-nitrogen (but not peptide) bonds, in cyclic amidines
GO:0008092	279	4	0.142729995130414	cytoskeletal protein binding
GO:0030246	181	3	0.142813905033316	carbohydrate binding
GO:0048503	92	2	0.142859560070658	GPI anchor binding
GO:0001653	94	2	0.147847759149095	peptide receptor activity

GO:0008528	94	2	0.147847759149095	peptide receptor activity, G-protein coupled
GO:0008408	24	1	0.160046245786377	3'-5' exonuclease activity
GO:0030234	509	6	0.161279716537765	enzyme regulator activity
GO:0004860	25	1	0.166136064956361	protein kinase inhibitor activity
GO:0004536	25	1	0.166136064956361	deoxyribonuclease activity
GO:0003755	25	1	0.166136064956361	peptidyl-prolyl cis-trans isomerase activity
GO:0008138	26	1	0.172182367133538	protein tyrosine/serine/threonine phosphatase activity
GO:0004907	26	1	0.172182367133538	interleukin receptor activity
GO:0003725	26	1	0.172182367133538	double-stranded RNA binding
GO:0016859	26	1	0.172182367133538	cis-trans isomerase activity
GO:0004722	27	1	0.178185458743394	protein serine/threonine phosphatase activity
GO:0008022	28	1	0.184145644085701	protein C-terminus binding
GO:0019210	29	1	0.190063225349035	kinase inhibitor activity
GO:0003823	30	1	0.195938502625202	antigen binding
GO:0019965	30	1	0.195938502625202	interleukin binding
GO:0030695	219	3	0.210782067903111	GTPase regulator activity
GO:0003924	121	2	0.217924899320190	GTPase activity
GO:0005525	226	3	0.224036679651365	GTP binding
GO:0031267	35	1	0.224690687435699	small GTPase binding
GO:0042277	127	2	0.233941972817195	peptide binding
GO:0019001	235	3	0.24131908047196	guanyl nucleotide binding
GO:0035091	132	2	0.247347366049311	phosphoinositide binding
GO:0008009	40	1	0.252429105503306	chemokine activity
GO:0004527	40	1	0.252429105503306	exonuclease activity
GO:0042379	40	1	0.252429105503306	chemokine receptor binding
GO:0005070	40	1	0.252429105503306	SH3/SH2 adaptor activity
GO:0004221	41	1	0.257858259457656	ubiquitin thiolesterase activity
GO:0001664	42	1	0.263248552099489	G-protein-coupled receptor binding
GO:0051020	42	1	0.263248552099489	GTPase binding
GO:0004843	43	1	0.268600257532181	ubiquitin-specific protease activity
GO:0004540	45	1	0.279188993673173	ribonuclease activity
GO:0019887	47	1	0.289626622847691	protein kinase regulator activity
GO:0004896	48	1	0.294789437575256	hematopoietin/interferon-class (D200-domain) cytokine receptor activity
GO:0019838	49	1	0.299915270168463	growth factor binding
GO:0016790	51	1	0.310057031301299	thiolester hydrolase activity
GO:0019904	52	1	0.315073476489941	protein domain specific binding
GO:0001584	282	3	0.334139017169821	rhodopsin-like receptor activity
GO:0019207	58	1	0.344426045924811	kinase regulator activity
GO:0005543	169	2	0.346359903314195	phospholipid binding
GO:0016564	59	1	0.349196118985149	transcriptional repressor activity
GO:0046982	61	1	0.358633881358381	protein heterodimerization activity
GO:0016563	62	1	0.363302052475077	transcriptional activator activity
GO:0004857	187	2	0.393259118450189	enzyme inhibitor activity
GO:0030674	69	1	0.395054719817484	protein binding, bridging
GO:0008047	188	2	0.395824002710505	enzyme activator activity
GO:0016810	72	1	0.408180428315016	hydrolase activity, acting on carbon-nitrogen (but not peptide) bonds
GO:0017076	1248	10	0.415921261168068	purine nucleotide binding

GO:0003702	197	2	0.418686337597245	RNA polymerase II transcription factor activity
GO:0004674	326	3	0.421119035276185	protein serine/threonine kinase activity
GO:0004197	80	1	0.441825762184733	cysteine-type endopeptidase activity
GO:0003714	81	1	0.445896581591694	transcription corepressor activity
GO:0004930	349	3	0.465263915555403	G-protein coupled receptor activity
GO:0005200	86	1	0.465815876761795	structural constituent of cytoskeleton
GO:0016740	1169	9	0.474795930386236	transferase activity
GO:0016853	92	1	0.488789213110995	isomerase activity
GO:0030145	92	1	0.488789213110995	manganese ion binding
GO:0008234	98	1	0.510788258399409	cysteine-type peptidase activity
GO:0003735	100	1	0.517911854399282	structural constituent of ribosome
GO:0005554	522	4	0.526446768349296	molecular function unknown
GO:0004518	105	1	0.535276669318857	nuclease activity
GO:0004252	105	1	0.535276669318857	serine-type endopeptidase activity
GO:0030528	963	7	0.553192261545344	transcription regulator activity
GO:0005524	970	7	0.561185147868178	ATP binding
GO:0008236	117	1	0.574476903474578	serine-type peptidase activity
GO:0016787	1424	10	0.592212331802122	hydrolase activity
GO:0000166	1432	10	0.59976657726306	nucleotide binding
GO:0016829	127	1	0.604639774584433	lyase activity
GO:0005057	132	1	0.618920903369647	receptor signaling protein activity
GO:0004175	288	2	0.621252658063233	endopeptidase activity
GO:0030554	1028	7	0.624943409430472	adenyl nucleotide binding
GO:0008083	144	1	0.651154202415715	growth factor activity
GO:0003677	1384	9	0.686876927257061	DNA binding
GO:0005198	488	3	0.692843767645311	structural molecule activity
GO:0005509	646	4	0.696132639199102	calcium ion binding
GO:0003700	649	4	0.69966465326244	transcription factor activity
GO:0008233	399	2	0.790625791322695	peptidase activity
GO:0003676	1990	12	0.803038709702537	nucleic acid binding
GO:0017111	410	2	0.803201491725748	nucleoside-triphosphatase activity
GO:0003712	230	1	0.815448735835685	transcription cofactor activity
GO:0016462	430	2	0.824386465077722	pyrophosphatase activity
GO:0016818	432	2	0.826390449950551	hydrolase activity, acting on acid anhydrides, in phosphorus-containing anhydrides
GO:0016817	433	2	0.827384858422018	hydrolase activity, acting on acid anhydrides
GO:0003723	436	2	0.830337980796996	RNA binding
GO:0008270	1287	7	0.840071726785509	zinc ion binding
GO:0000287	251	1	0.842163960225211	magnesium ion binding
GO:0003824	3409	21	0.852855654473187	catalytic activity
GO:0008134	293	1	0.884675649368444	transcription factor binding
GO:0046914	1596	8	0.910106516844717	transition metal ion binding
GO:0043169	2313	12	0.934297603580282	cation binding
GO:0043167	2556	13	0.953146410898004	ion binding
GO:0046872	2495	12	0.968601674872137	metal ion binding
GO:0003674	9541	69	1	molecular_function

15.4 GO: Cellular Component

GO ID	genes present in chip	genes present in tissues	p-value	GO term
GO:0001772	29	6	8.56424864917154e-08	immunological synapse
GO:0042101	10	4	7.55630098483906e-07	T cell receptor complex
GO:0043235	58	5	9.27466932203223e-05	receptor complex
GO:0005886	1472	25	9.65407596925735e-05	plasma membrane
GO:0016020	3514	42	0.000539833907660502	membrane
GO:0030055	11	2	0.00330232778104823	cell-matrix junction
GO:0045121	14	2	0.00537997613514034	lipid raft
GO:0016021	2674	31	0.00989301411179665	integral to membrane
GO:0031224	2681	31	0.0102997773052845	intrinsic to membrane
GO:0030056	2	1	0.0159148654525160	hemidesmosome
GO:0005887	1057	14	0.0376565667985367	integral to plasma membrane
GO:0031226	1062	14	0.0389812741037748	intrinsic to plasma membrane
GO:0000242	6	1	0.0469994469699492	pericentriolar material
GO:0016327	50	2	0.0602805908317612	apicolateral plasma membrane
GO:0005925	8	1	0.0621768243750751	focal adhesion
GO:0016605	8	1	0.0621768243750751	PML body
GO:0005765	9	1	0.0696759319330901	lysosomal membrane
GO:0005924	9	1	0.0696759319330901	cell-substrate adherens junction
GO:0005856	612	8	0.113367950910168	cytoskeleton
GO:0005665	16	1	0.120540265552164	DNA-directed RNA polymerase II, core complex
GO:0016604	16	1	0.120540265552164	nuclear body
GO:0005912	18	1	0.134562622558188	adherens junction
GO:0030880	23	1	0.168661774144394	RNA polymerase complex
GO:0030054	93	2	0.169987864084237	cell junction
GO:0008305	24	1	0.175320972842331	integrin complex
GO:0005615	385	5	0.193239752738432	extracellular space
GO:0005938	28	1	0.201436095648731	cell cortex
GO:0008372	523	6	0.238416429932551	cellular component unknown
GO:0000786	40	1	0.274988817117742	nucleosome
GO:0005813	43	1	0.292308020291621	centrosome
GO:0005815	49	1	0.325732284573746	microtubule organizing center
GO:0005623	7802	64	0.331891855236769	cell
GO:0000922	51	1	0.336524147625275	spindle pole
GO:0016591	58	1	0.372972118092989	DNA-directed RNA polymerase II, holoenzyme
GO:0043228	977	9	0.378241074138621	non-membrane-bound organelle
GO:0043232	977	9	0.378241074138621	intracellular non-membrane-bound organelle
GO:0005819	68	1	0.421641275496436	spindle
GO:0015629	183	2	0.43164549435499	actin cytoskeleton
GO:0005654	221	2	0.530739844743581	nucleoplasm
GO:0000785	100	1	0.553692920133894	chromatin
GO:0005764	107	1	0.578348118784957	lysosome
GO:0000323	107	1	0.578348118784957	lytic vacuole
GO:0005840	113	1	0.598409163221455	ribosome

GO:0005624	521	4	0.605198228098118	membrane fraction
GO:0005773	120	1	0.62062714319265	vacuole
GO:0000267	698	5	0.665350119388222	cell fraction
GO:0005625	183	1	0.773177514665252	soluble fraction
GO:0005576	949	6	0.784334094115916	extracellular region
GO:0015630	191	1	0.787577337116735	microtubule cytoskeleton
GO:0043234	1247	8	0.798309685497252	protein complex
GO:0005694	203	1	0.80750381364301	chromosome
GO:0005634	2704	18	0.858153230770694	nucleus
GO:0030529	254	1	0.873547280971163	ribonucleoprotein complex
GO:0005829	276	1	0.894595083218254	cytosol
GO:0005737	2530	13	0.983145873866062	cytoplasm
GO:0005622	5419	35	0.9849642867187	intracellular
GO:0043226	4515	25	0.997526818476703	organelle
GO:0043229	4515	25	0.997526818476703	intracellular organelle
GO:0043227	3955	19	0.999417961475184	membrane-bound organelle
GO:0043231	3955	19	0.999417961475184	intracellular membrane-bound organelle
GO:0005575	8637	69	1	cellular_component

16. BM-CD71EarlyErythroid

16.1 BM-CD71EarlyErythroid selective genes (131 genes)

Symbol	Description	Chromosome	Chromosome Location	LocusLink	Cytoband	UniGene
TRIM58	tripartite motif-containing 58	1	246087123	25893	1q44	Hs.323858
XK	X-linked Kx blood group (McLeod syndrome)	X	37430051	7504	Xp21.1	Hs.78919
GYPA	glycophorin A (MNS blood group)	4	-145249906	2993	4q28.2-q31.1	Hs.434973
NFE2	nuclear factor (erythroid-derived 2), 45kDa	12	-52972164	4778	12q13	Hs.75643
ERAF	erythroid associated factor	16	31446703	51327	16p11.2	Hs.274309
HBD	hemoglobin, delta	11	-5210634	3045	11p15.5	Hs.591988
GYPB	glycophorin B (MNS blood group)	4	-145136706	2994	4q28-q31	Hs.632594
ALAS2	aminolevulinate, delta-, synthase 2 (sideroblastic/hypochromic anemia)	X	-55052213	212	Xp11.21	Hs.522666
MINPP1	multiple inositol polyphosphate histidine phosphatase, 1	10	89254632	9562	10q23	Hs.121260
EPB42	erythrocyte membrane protein band 4.2	15	-41276719	2038	15q15-q21	Hs.368642
GYPE	glycophorin E	4	-290027384	2996	4q31.1	Hs.632594
CA2	carbonic anhydrase II	8	86563497	760	8q22	Hs.155097
SLC25A37	solute carrier family 25, member 37	8	23442307	51312	8p21.2	Hs.122514 Hs.596025
ACSL6	acyl-CoA synthetase long-chain family member 6	5	-131317050	23305	5q31	Hs.14945
KLF1	Kruppel-like factor 1 (erythroid)	19	-12856238	10661	19p13.13-p13.12	Hs.37860
SPTA1	spectrin, alpha, erythrocytic 1 (elliptocytosis 2)	1	-156847119	6708	1q21	Hs.119825
RHCE	Rh blood group, CcEe antigens	1	-25561327	6006	1p36.11	Hs.449968 Hs.643556
BPGM	2,3-bisphosphoglycerate mutase	7	133982094, 133982110	669	7q31-q34	Hs.198365
DLG7	discs, large homolog 7 (Drosophila)	14	-54684600	9787	14q22.3	Hs.77695
TOP2A	topoisomerase (DNA) II alpha 170kDa	17	-35798323	7153	17q21-q22	Hs.156346
NUSAP1	nucleolar and spindle associated protein 1	15	39412360	51203	15q15.1	Hs.615092
TSPAN5	tetraspanin 5	4	-99612434	10098	4q23	Hs.591706
CD36	CD36 molecule (thrombospondin receptor)	7	80105892, 80069458	948	7q11.2	Hs.120949 Hs.633085
MLF1IP	MLF1 interacting protein	4	-185852231	79682	4q35.1	Hs.481307 Hs.575032
ERMAP	erythroblast membrane-associated protein (Scianna blood group)	1	43055362, 43063835	114625	1p34.2	Hs.439437
SELENBP1	selenium binding protein 1	1	-149603404	8991	1q21-q22	Hs.632460
CPOX	coproporphyrinogen oxidase	3	-99780981	1371	3q12	Hs.476982
GFI1B	growth factor independent 1B (potential regulator of CDKN1A, translocated in CML)	9	134843918	8328	9q34.13	Hs.553160
BZRPL1	benzodiazapine receptor (peripheral)-like 1	6	41118270	222642	6p21.1	Hs.357392
PRC1	protein regulator of cytokinesis 1	15	-89310276	9055	15q26.1	Hs.567385
TRAK2	trafficking protein, kinesin binding 2	2	-201950175	66008	2q33	Hs.152774
HMGB2	high-mobility group box 2	4	-174489362	3148	4q31	Hs.434953
MYL4	myosin, light polypeptide 4, alkali, atrial, embryonic	17	42641426, 42641712	4635	17q21-qter	Hs.463300
NBR1	neighbor of BRCA1 gene 1	17	38576771, 38576036, 38576023	4077	17q21.31	Hs.277721 Hs.373818 Hs.546264
TFDP1	transcription factor Dp-1	13	113287056	7027	13q34	Hs.79353

DCK	deoxycytidine kinase	4	72078255	1633	4q13.3-q21.1	Hs.709
RHAG	Rh-associated glycoprotein	6	-49680855	6005	6p21.1-p11	Hs.120950
SNCA	synuclein, alpha (non A4 component of amyloid precursor)	4	-181731784	6622	4q21	Hs.271771
TMCC2	transmembrane and coiled-coil domain family 2	1	203463713	9911	1q32.1	Hs.6360
SLC22A4	solute carrier family 22 (organic cation transporter), member 4	5	131658043	6583	5q31.1	Hs.310591 Hs.640104
TUBG1	tubulin, gamma 1	17	38015219	7283	17q21	Hs.279669
DTL	denticleless homolog (Drosophila)	1	210275753	51514	NA	Hs.632496
CLIC2	chloride intracellular channel 2	X	-154158695	1193	Xq28	Hs.632837
AMMECR1	Alport syndrome, mental retardation, midface hypoplasia and elliptocytosis chromosomal region, gene 1	X	-109324070	9949	Xq22.3	Hs.481208
TAL1	T-cell acute lymphocytic leukemia 1	1	-47454550	6886	1p32	Hs.73828
FECH	ferrochelatase (protoporphyria)	18	-53366534	2235	18q21.3	Hs.465221
CA1	carbonic anhydrase I	8	-86427708	759	8q13-q22.1	Hs.23118
ZWINT	ZW10 interactor	10	-57787204	11130	10q21-q22	Hs.591363
PPOX	protoporphyrinogen oxidase	1	159402817	5498	1q22	Hs.517373
PCAF	p300/CBP-associated factor	3	20056527	8850	3p24	Hs.533055
GCLM	glutamate-cysteine ligase, modifier subunit	1	-94125177	2730	1p22.1	Hs.315562
MCM5	MCM5 minichromosome maintenance deficient 5, cell division cycle 46 (S. cerevisiae)	22	34126127	4174	22q13.1	Hs.517582
ISG20	interferon stimulated exonuclease gene 20kDa	15	86983042	3669	15q26	Hs.459265
HAT1	histone acetyltransferase 1	2	172487203	8520	2q31.2-q33.1	Hs.632532
CDC20	CDC20 cell division cycle 20 homolog (S. cerevisiae)	1	43597214	991	1p34.1	Hs.524947
FAM117A	family with sequence similarity 117, member A	17	-45142687	81558	17q21.33	Hs.514308
HBQ1	hemoglobin, theta 1	16	170334	3049	16p13.3	Hs.247921
PCNA	proliferating cell nuclear antigen	20	-5043599	5111	20pter-p12	Hs.147433
PDZK1IP1	PDZK1 interacting protein 1	1	-47421847	10158	1p33	Hs.431099
LBR	lamin B receptor	1	-223655827	3930	1q42.1	Hs.435166
RRM2	ribonucleotide reductase M2 polypeptide	2	10180185	6241	2p25-p24	Hs.226390
VRK1	vaccinia related kinase 1	14	96333436	7443	14q32	Hs.422662
PRKAR2B	protein kinase, cAMP-dependent, regulatory, type II, beta	7	106472413	5577	7q22	Hs.433068
CKS2	CDC28 protein kinase regulatory subunit 2	9	91115932	1164	9q22	Hs.83758
EIF1AY	eukaryotic translation initiation factor 1A, Y-linked	Y	21146998	9086	Yq11.222	Hs.461178
HMBS	hydroxymethylbilane synthase	11	118460796, 118463906	3145	11q23.3	Hs.82609
CHST2	carbohydrate (N-acetylglucosamine-6-O) sulfotransferase 2	3	144321357	9435	3q24	Hs.8786
RHD	Rh blood group, D antigen	1	25471567	6007	1p36.11	Hs.643556
EZH2	enhancer of zeste homolog 2 (Drosophila)	7	-148135407	2146	7q35-q36	Hs.444082
TMEM14B	transmembrane protein 14B	6	10856035	81853	6p25.1-p23	Hs.273077
ICAM4	intercellular adhesion molecule 4 (Landsteiner-Wiener blood group)	19	10258649	3386	19p13.2-cen	Hs.631609

ST6GALNAC4	ST6 (alpha-N-acetyl-neuraminyl-2,3-beta-galactosyl-1,3)-N-acetylgalactosaminide alpha-2,6-sialyltransferase 4	9	-129709986	27090	9q34	Hs.3972
GCLC	glutamate-cysteine ligase, catalytic subunit	6	-53470100	2729	6p12	Hs.271264
BMP2K	BMP2 inducible kinase	4	79916555	55589	4q21.21	Hs.146551
NP	nucleoside phosphorylase	14	20007404	4860	14q13.1	Hs.75514
RPIA	ribose 5-phosphate isomerase A (ribose 5-phosphate epimerase)	2	88772290	22934	2p11.2	Hs.469264
CDKN3	cyclin-dependent kinase inhibitor 3 (CDK2-associated dual specificity phosphatase)	14	53933475	1033	14q22	Hs.84113
ARG1	arginase, liver	6	131936057	383	6q23	Hs.440934
APOBEC3B	apolipoprotein B mRNA editing enzyme, catalytic polypeptide-like 3B	22	37708350	9582	22q13.1-q13.2	Hs.226307
HCAP-G	NA	4	17421622	64151	4p15.33	Hs.446201 Hs.567567
DBF4	DBF4 homolog (S. cerevisiae)	7	87343479	10926	7q21.3	Hs.485380
RSAD2	radical S-adenosyl methionine domain containing 2	2	6935246	91543	2p25.2	Hs.17518
RACGAP1	Rac GTPase activating protein 1	12	-48669214	29127	12q13.13	Hs.505469
BLVRB	biliverdin reductase B (flavin reductase (NADPH))	19	-45645540	645	19q13.1-q13.2	Hs.515785
SMC4	structural maintenance of chromosomes 4	3	161600117, 161600123, 161601040	10051	3q26.1	Hs.58992
TESC	tescalcin	12	-115961112	54997	12q24.22	Hs.525709
KLF13	Kruppel-like factor 13	15	29406374	51621	15q12	Hs.525752
PIM1	pim-1 oncogene	6	37245963	5292	6p21.2	Hs.81170
NUDT1	nudix (nucleoside diphosphate linked moiety X)-type motif 1	7	2248382, 2248421, 2249065	4521	7p22	Hs.534331
CDC2	cell division cycle 2, G1 to S and G2 to M	10	62208241, 62209916	983	10q21.1	Hs.334562
LMO2	LIM domain only 2 (rhombotin-like 1)	11	-33836698	4005	11p13	Hs.34560
UROD	uroporphyrinogen decarboxylase	1	45250416	7389	1p34	Hs.78601
PSMD14	proteasome (prosome, macropain) 26S subunit, non-ATPase, 14	2	161873185	10213	2q24.2	Hs.567410
TOP1	topoisomerase (DNA) I	20	39090875	7150	20q12-q13.1	Hs.592136
SLC14A1	solute carrier family 14 (urea transporter), member 1 (Kidd blood group)	18	41558156	6563	18q11-q12	Hs.101307
LXN	latexin	3	-159866899	56925	3q25.32	Hs.478067
CAT	catalase	11	34417053	847	11p13	Hs.502302
CENPF	centromere protein F, 350/400ka (mitosin)	1	212843154	1063	1q32-q41	Hs.497741
HBLD2	HESB like domain containing 2	9	-88069283	81689	9q21.33	Hs.449291
NUDT22	nudix (nucleoside diphosphate linked moiety X)-type motif 22	11	63750337	84304	11q13.1	Hs.172847
SMC2	structural maintenance of chromosomes 2	9	105896651, 105896361	10592	9q31.1	Hs.119023
PRDX2	peroxiredoxin 2	19	-25541060	7001	19p13.2	Hs.631612
RRM1	ribonucleotide reductase M1 polypeptide	11	4072586	6240	11p15.5	Hs.558393
ASPM	asp (abnormal spindle)-like, microcephaly associated (Drosophila)	1	-195320076	259266	1q31	Hs.121028
RIOK3	RIO kinase 3 (yeast)	18	19287253	8780	18q11.2	Hs.445511
IQWD1	IQ motif and WD repeats 1	1	166172531	55827	1q24.2	Hs.435741
PBK	PDZ binding kinase	8	-27723058	55872	8p21.2	Hs.104741

LOC645719	NA	1	NA	645719	1p36.13	Hs.631865
MARCH3	membrane-associated ring finger (C3HC4) 3	5	-126233453	115123	5q23.2	Hs.132441
CTSE	cathepsin E	1	204484081	1510	1q31	Hs.1355
GATA1	GATA binding protein 1 (globin transcription factor 1)	X	48529905	2623	Xp11.23	Hs.765
TYMS	thymidylate synthetase	18	647650	7298	18p11.32	Hs.592338
KIF4A	kinesin family member 4A	X	69426686	24137	Xq13.1	Hs.438712
SLC7A5	solute carrier family 7 (cationic amino acid transporter, y+ system), member 5	16	-86421130	8140	16q24.3	Hs.513797
MGC14376	NA	17	-1561553	84981	17p13.3	Hs.417157
RBM38	RNA binding motif protein 38	20	55399869	55544	20q13.31	Hs.236361
EIF2AK1	eukaryotic translation initiation factor 2-alpha kinase 1	7	-6029990	27102	7p22	Hs.520205
HIST1H4C	histone 1, H4c	6	26212154	8364	6p21.3	Hs.46423
TFRC	transferrin receptor (p90, CD71)	3	-197260746	7037	3q29	Hs.529618
HBZ	hemoglobin, zeta	16	142853	3050	16p13.3	Hs.585357
TTK	TTK protein kinase	6	80771077	7272	6q13-q21	Hs.169840
BNIP3L	BCL2/adenovirus E1B 19kDa interacting protein 3-like	8	26296439	665	8p21	Hs.131226
TFR2	transferrin receptor 2	7	-100055975	7036	7q22	Hs.544932
ASCC2	activating signal cointegrator 1 complex subunit 2	22	-28514602	84164	22q12.1	Hs.517438
ALAD	aminolevulinate, delta-, dehydratase	9	-115188413	210	9q33.1	Hs.1227
CCNE1	cyclin E1	19	34994740, 34995400	898	19q12	Hs.244723
H1F0	H1 histone family, member 0	22	36531059	3005	22q13.1	Hs.226117
ITGA4	integrin, alpha 4 (antigen CD49D, alpha 4 subunit of VLA-4 receptor)	2	182029863	3676	2q31.3	Hs.440955
PIP5K1B	phosphatidylinositol-4-phosphate 5-kinase, type I, beta	9	70547029, 70510435	8395	9q13	Hs.534371
BCL2L1	BCL2-like 1	20	-29715923	598	20q11.21	Hs.516966
RABGAP1L	RAB GTPase activating protein 1-like	1	173111306, 172395256	9910	1q24	Hs.585378 Hs.591475 Hs.615081

16.2 GO: Biological Process

GO ID	genes present in chip	genes present in tissues	p-value	GO term
GO:0006783	11	7	6.05E-12	heme biosynthesis
GO:0006779	13	7	3.09E-11	porphyrin biosynthesis
GO:0042168	15	7	1.14E-10	heme metabolism
GO:0006778	18	7	5.47E-10	porphyrin metabolism
GO:0000278	204	16	8.75E-10	mitotic cell cycle
GO:0046148	20	7	1.31E-09	pigment biosynthesis
GO:0042440	24	7	5.63E-09	pigment metabolism
GO:0019748	26	7	1.05E-08	secondary metabolism
GO:0007067	135	12	3.27E-08	mitosis
GO:0000087	137	12	3.86E-08	M phase of mitotic cell cycle
GO:0007049	601	23	1.59E-07	cell cycle
GO:0051188	85	9	4.23E-07	cofactor biosynthesis
GO:0000279	173	12	5.04E-07	M phase
GO:0051301	142	11	5.16E-07	cell division
GO:0046483	44	7	5.18E-07	heterocycle metabolism
GO:0051186	143	11	5.54E-07	cofactor metabolism
GO:0007059	34	6	1.86E-06	chromosome segregation
GO:0007051	21	5	2.93E-06	spindle organization and biogenesis
GO:0006260	140	9	2.70E-05	DNA replication
GO:0006259	489	17	2.96E-05	DNA metabolism
GO:0000070	20	4	6.46E-05	mitotic sister chromatid segregation
GO:0000819	21	4	7.92E-05	sister chromatid segregation
GO:0050875	7018	94	8.69E-05	cellular physiological process
GO:0006268	9	3	0.000111815	DNA unwinding
GO:0048015	71	6	0.000141548	phosphoinositide-mediated signaling
GO:0007076	11	3	0.000216051	mitotic chromosome condensation
GO:0051329	79	6	0.000255452	interphase of mitotic cell cycle
GO:0000226	52	5	0.000283969	microtubule cytoskeleton organization and biogenesis
GO:0015669	12	3	0.000285707	gas transport
GO:0015671	12	3	0.000285707	oxygen transport
GO:0030261	12	3	0.000285707	chromosome condensation
GO:0051325	82	6	0.000313153	interphase
GO:0006826	14	3	0.000465008	iron ion transport
GO:0007052	14	3	0.000465008	mitotic spindle organization and biogenesis
GO:0007094	4	2	0.000746654	mitotic spindle checkpoint
GO:0006261	65	5	0.000803795	DNA-dependent DNA replication
GO:0006534	5	2	0.001235251	cysteine metabolism
GO:0015695	5	2	0.001235251	organic cation transport
GO:0050000	5	2	0.001235251	chromosome localization
GO:0008634	5	2	0.001235251	negative regulation of survival gene product activity
GO:0051303	5	2	0.001235251	establishment of chromosome localization
GO:0020027	5	2	0.001235251	hemoglobin metabolism
GO:0006750	5	2	0.001235251	glutathione biosynthesis

GO:0044237	5026	72	0.001331204	cellular metabolism
GO:0051276	236	9	0.00135142	chromosome organization and biogenesis
GO:0000074	394	12	0.001553489	regulation of progression through cell cycle
GO:0007017	112	6	0.001630207	microtubule-based process
GO:0006996	693	17	0.001812122	organelle organization and biogenesis
GO:0008283	473	13	0.002469296	cell proliferation
GO:0045884	7	2	0.00255598	regulation of survival gene product activity
GO:0009262	7	2	0.00255598	deoxyribonucleotide metabolism
GO:0006265	7	2	0.00255598	DNA topological change
GO:0006749	8	2	0.00338292	glutathione metabolism
GO:0000041	31	3	0.004996632	transition metal ion transport
GO:0016043	842	18	0.00583308	cell organization and biogenesis
GO:0008152	5262	72	0.006622841	metabolism
GO:0007093	13	2	0.009083381	mitotic checkpoint
GO:0007088	39	3	0.009519095	regulation of mitosis
GO:0015696	1	1	0.01129318	ammonium transport
GO:0046984	1	1	0.01129318	regulation of hemoglobin biosynthesis
GO:0046986	1	1	0.01129318	negative regulation of hemoglobin biosynthesis
GO:0009052	1	1	0.01129318	pentose-phosphate shunt, non-oxidative branch
GO:0009162	1	1	0.01129318	deoxyribonucleoside monophosphate metabolism
GO:0009157	1	1	0.01129318	deoxyribonucleoside monophosphate biosynthesis
GO:0045993	1	1	0.01129318	negative regulation of translational initiation by iron
GO:0009176	1	1	0.01129318	pyrimidine deoxyribonucleoside monophosphate metabolism
GO:0009177	1	1	0.01129318	pyrimidine deoxyribonucleoside monophosphate biosynthesis
GO:0009221	1	1	0.01129318	pyrimidine deoxyribonucleotide biosynthesis
GO:0007108	1	1	0.01129318	cytokinesis, initiation of separation
GO:0000114	1	1	0.01129318	G1-specific transcription in mitotic cell cycle
GO:0000212	1	1	0.01129318	meiotic spindle organization and biogenesis
GO:0046073	1	1	0.01129318	dTMP metabolism
GO:0051103	1	1	0.01129318	DNA ligation during DNA repair
GO:0000738	1	1	0.01129318	DNA catabolism, exonucleolytic
GO:0006447	1	1	0.01129318	regulation of translational initiation by iron
GO:0006288	1	1	0.01129318	base-excision repair, DNA ligation
GO:0006231	1	1	0.01129318	dTMP biosynthesis
GO:0000920	1	1	0.01129318	cell separation during cytokinesis
GO:0000912	1	1	0.01129318	cytokinesis, formation of actomyosin apparatus
GO:0000915	1	1	0.01129318	cytokinesis, contractile ring formation
GO:0006879	15	2	0.012049511	iron ion homeostasis
GO:0006979	44	3	0.013247039	response to oxidative stress
GO:0006473	16	2	0.013670335	protein amino acid acetylation
GO:0019932	176	6	0.0145086	second-messenger-mediated signaling
GO:0043066	128	5	0.014754356	negative regulation of apoptosis
GO:0043069	129	5	0.015215248	negative regulation of programmed cell death
GO:0006334	48	3	0.016749459	nucleosome assembly
GO:0006790	48	3	0.016749459	sulfur metabolism
GO:0000080	18	2	0.017176448	G1 phase of mitotic cell cycle
GO:0000910	18	2	0.017176448	cytokinesis

GO:0015837	52	3	0.020722055	amine transport
GO:0046916	20	2	0.021020876	transition metal ion homeostasis
GO:0000096	20	2	0.021020876	sulfur amino acid metabolism
GO:0007010	305	8	0.021777772	cytoskeleton organization and biogenesis
GO:0000082	53	3	0.021789123	G1/S transition of mitotic cell cycle
GO:0006323	193	6	0.021839597	DNA packaging
GO:0015840	2	1	0.02246006	urea transport
GO:0045842	2	1	0.02246006	positive regulation of mitotic metaphase/anaphase transition
GO:0007079	2	1	0.02246006	mitotic chromosome movement towards spindle pole
GO:0000022	2	1	0.02246006	mitotic spindle elongation
GO:0009186	2	1	0.02246006	deoxyribonucleoside diphosphate metabolism
GO:0007405	2	1	0.02246006	neuroblast proliferation
GO:0009263	2	1	0.02246006	deoxyribonucleotide biosynthesis
GO:0051382	2	1	0.02246006	kinetochore assembly
GO:0051383	2	1	0.02246006	kinetochore organization and biogenesis
GO:0051305	2	1	0.02246006	chromosome movement towards spindle pole
GO:0051231	2	1	0.02246006	spindle elongation
GO:0006475	2	1	0.02246006	internal protein amino acid acetylation
GO:0051318	21	2	0.023064731	G1 phase
GO:0031497	56	3	0.025168042	chromatin assembly
GO:0009069	23	2	0.027385576	serine family amino acid metabolism
GO:0044272	24	2	0.029658614	sulfur compound biosynthesis
GO:0006800	61	3	0.031390671	oxygen and reactive oxygen species metabolism
GO:0008360	25	2	0.032004148	regulation of cell shape
GO:0007582	7846	95	0.033477953	physiological process
GO:0043249	3	1	0.033502039	erythrocyte maturation
GO:0000085	3	1	0.033502039	G2 phase of mitotic cell cycle
GO:0009129	3	1	0.033502039	pyrimidine nucleoside monophosphate metabolism
GO:0009130	3	1	0.033502039	pyrimidine nucleoside monophosphate biosynthesis
GO:0009132	3	1	0.033502039	nucleoside diphosphate metabolism
GO:0051319	3	1	0.033502039	G2 phase
GO:0006266	3	1	0.033502039	DNA ligation
GO:0006220	26	2	0.034420287	pyrimidine nucleotide metabolism
GO:0006916	112	4	0.037708572	anti-apoptosis
GO:0007001	221	6	0.03876308	chromosome organization and biogenesis (sensu Eukaryota)
GO:0008089	4	1	0.044420501	anterograde axon cargo transport
GO:0045947	4	1	0.044420501	negative regulation of translational initiation
GO:0009219	4	1	0.044420501	pyrimidine deoxyribonucleotide metabolism
GO:0051310	4	1	0.044420501	metaphase plate congression
GO:0042541	4	1	0.044420501	hemoglobin biosynthesis
GO:0006730	30	2	0.044754167	one-carbon compound metabolism
GO:0006281	174	5	0.046835765	DNA repair
GO:0006732	121	4	0.047821091	coenzyme metabolism
GO:0009064	32	2	0.050297884	glutamine family amino acid metabolism
GO:0043283	2240	33	0.051256737	biopolymer metabolism
GO:0006527	5	1	0.055216814	arginine catabolism
GO:0045840	5	1	0.055216814	positive regulation of mitosis

GO:0006325	186	5	0.059150318	establishment and/or maintenance of chromatin architecture
GO:0000079	36	2	0.062071319	regulation of cyclin dependent protein kinase activity
GO:0051243	583	11	0.063921991	negative regulation of cellular physiological process
GO:0000075	37	2	0.065148215	cell cycle checkpoint
GO:0006637	6	1	0.06589233	acyl-CoA metabolism
GO:0007089	6	1	0.06589233	traversing start control point of mitotic cell cycle
GO:0016478	6	1	0.06589233	negative regulation of translation
GO:0031032	6	1	0.06589233	actomyosin structure organization and biogenesis
GO:0000904	6	1	0.06589233	cellular morphogenesis during differentiation
GO:0006974	200	5	0.075651486	response to DNA damage stimulus
GO:0008088	7	1	0.076448388	axon cargo transport
GO:0006536	7	1	0.076448388	glutamate metabolism
GO:0045787	7	1	0.076448388	positive regulation of progression through cell cycle
GO:0000050	7	1	0.076448388	urea cycle
GO:0008637	7	1	0.076448388	apoptotic mitochondrial changes
GO:0019627	7	1	0.076448388	urea metabolism
GO:0042743	7	1	0.076448388	hydrogen peroxide metabolism
GO:0042744	7	1	0.076448388	hydrogen peroxide catabolism
GO:0043118	602	11	0.076530165	negative regulation of physiological process
GO:0006413	41	2	0.077944028	translational initiation
GO:0009117	143	4	0.078230715	nucleotide metabolism
GO:0009987	8200	97	0.08205514	cellular process
GO:0006333	91	3	0.083473875	chromatin assembly or disassembly
GO:0006865	43	2	0.084614482	amino acid transport
GO:0030071	8	1	0.086886312	regulation of mitotic metaphase/anaphase transition
GO:0006098	8	1	0.086886312	pentose-phosphate shunt
GO:0007589	8	1	0.086886312	fluid secretion
GO:0042542	8	1	0.086886312	response to hydrogen peroxide
GO:0006740	8	1	0.086886312	NADPH regeneration
GO:0044249	763	13	0.087475838	cellular biosynthesis
GO:0000086	44	2	0.088012932	G2/M transition of mitotic cell cycle
GO:0050878	94	3	0.089953188	regulation of body fluids
GO:0009719	214	5	0.094410571	response to endogenous stimulus
GO:0007091	9	1	0.097207411	mitotic metaphase/anaphase transition
GO:0016202	9	1	0.097207411	regulation of myogenesis
GO:0015674	99	3	0.101203315	di-, tri-valent inorganic cation transport
GO:0048523	640	11	0.106245222	negative regulation of cellular process
GO:0006525	10	1	0.10741298	arginine metabolism
GO:0006739	10	1	0.10741298	NADP metabolism
GO:0008015	103	3	0.110589375	circulation
GO:0006873	103	3	0.110589375	cell ion homeostasis
GO:0006007	51	2	0.112859295	glucose catabolism
GO:0000051	11	1	0.117504298	urea cycle intermediate metabolism
GO:0009065	12	1	0.127482633	glutamine family amino acid catabolism
GO:0019886	12	1	0.127482633	antigen processing, exogenous antigen via MHC class II
GO:0006275	12	1	0.127482633	regulation of DNA replication
GO:0006468	444	8	0.128336806	protein amino acid phosphorylation

GO:0050801	111	3	0.130312107	ion homeostasis
GO:0006357	240	5	0.134898271	regulation of transcription from RNA polymerase II promoter
GO:0000302	13	1	0.137349238	response to reactive oxygen species
GO:0030218	13	1	0.137349238	erythrocyte differentiation
GO:0051248	58	2	0.139257384	negative regulation of protein metabolism
GO:0015849	59	2	0.143132619	organic acid transport
GO:0019320	59	2	0.143132619	hexose catabolism
GO:0046942	59	2	0.143132619	carboxylic acid transport
GO:0007018	59	2	0.143132619	microtubule-based movement
GO:0046365	59	2	0.143132619	monosaccharide catabolism
GO:0030705	59	2	0.143132619	cytoskeleton-dependent intracellular transport
GO:0046164	60	2	0.147030735	alcohol catabolism
GO:0006308	14	1	0.14710535	DNA catabolism
GO:0006769	14	1	0.14710535	nicotinamide metabolism
GO:0006520	181	4	0.148088058	amino acid metabolism
GO:0007050	61	2	0.150950737	cell cycle arrest
GO:0048519	688	11	0.152326715	negative regulation of biological process
GO:0007020	15	1	0.156752196	microtubule nucleation
GO:0007585	15	1	0.156752196	respiratory gaseous exchange
GO:0018149	15	1	0.156752196	peptide cross-linking
GO:0019725	122	3	0.15926343	cell homeostasis
GO:0006284	16	1	0.166290987	base-excision repair
GO:0009058	859	13	0.169326439	biosynthesis
GO:0019362	17	1	0.175722921	pyridine nucleotide metabolism
GO:0017148	17	1	0.175722921	negative regulation of protein biosynthesis
GO:0006270	17	1	0.175722921	DNA replication initiation
GO:0046777	17	1	0.175722921	autophosphorylation
GO:0006461	194	4	0.176184301	protein complex assembly
GO:0031327	18	1	0.185049184	negative regulation of cellular biosynthesis
GO:0009416	18	1	0.185049184	response to light
GO:0009123	18	1	0.185049184	nucleoside monophosphate metabolism
GO:0009124	18	1	0.185049184	nucleoside monophosphate biosynthesis
GO:0016540	18	1	0.185049184	protein autoprocessing
GO:0006221	18	1	0.185049184	pyrimidine nucleotide biosynthesis
GO:0009308	269	5	0.187664708	amine metabolism
GO:0006664	19	1	0.194270948	glycolipid metabolism
GO:0009890	19	1	0.194270948	negative regulation of biosynthesis
GO:0006044	19	1	0.194270948	N-acetylglucosamine metabolism
GO:0006304	19	1	0.194270948	DNA modification
GO:0009108	72	2	0.195248903	coenzyme biosynthesis
GO:0042493	20	1	0.203389373	response to drug
GO:0006041	20	1	0.203389373	glucosamine metabolism
GO:0006366	426	7	0.205623023	transcription from RNA polymerase II promoter
GO:0048469	21	1	0.212405605	cell maturation
GO:0051016	21	1	0.212405605	barbed-end actin filament capping
GO:0019752	359	6	0.219414919	carboxylic acid metabolism
GO:0030042	22	1	0.221320779	actin filament depolymerization

GO:0030834	22	1	0.221320779	regulation of actin filament depolymerization
GO:0030835	22	1	0.221320779	negative regulation of actin filament depolymerization
GO:0006082	361	6	0.223056099	organic acid metabolism
GO:0016052	79	2	0.224226417	carbohydrate catabolism
GO:0006006	79	2	0.224226417	glucose metabolism
GO:0044275	79	2	0.224226417	cellular carbohydrate catabolism
GO:0006519	216	4	0.227373352	amino acid and derivative metabolism
GO:0009892	217	4	0.229789447	negative regulation of metabolism
GO:0007015	23	1	0.230136015	actin filament organization
GO:0006446	23	1	0.230136015	regulation of translational initiation
GO:0006807	291	5	0.231978034	nitrogen compound metabolism
GO:0016310	522	8	0.236239493	phosphorylation
GO:0006091	446	7	0.238677246	generation of precursor metabolites and energy
GO:0006040	24	1	0.238852424	amino sugar metabolism
GO:0007599	83	2	0.240938868	hemostasis
GO:0042087	25	1	0.247471102	cell-mediated immune response
GO:0006733	25	1	0.247471102	oxidoreduction coenzyme metabolism
GO:0030097	87	2	0.257710809	hemopoiesis
GO:0030005	87	2	0.257710809	di-, tri-valent inorganic cation homeostasis
GO:0042592	157	3	0.261249325	homeostasis
GO:0048534	88	2	0.261908581	hemopoietic or lymphoid organ development
GO:0006092	88	2	0.261908581	main pathways of carbohydrate metabolism
GO:0042981	306	5	0.26379716	regulation of apoptosis
GO:0016066	27	1	0.264419595	cellular defense response (sensu Vertebrata)
GO:0044248	700	10	0.26526086	cellular catabolism
GO:0043067	308	5	0.268118754	regulation of programmed cell death
GO:0044238	4820	58	0.270769796	primary metabolism
GO:0044260	2298	29	0.2761032	cellular macromolecule metabolism
GO:0030521	29	1	0.280990032	androgen receptor signaling pathway
GO:0006941	29	1	0.280990032	striated muscle contraction
GO:0051052	29	1	0.280990032	regulation of DNA metabolism
GO:0006767	29	1	0.280990032	water-soluble vitamin metabolism
GO:0006875	93	2	0.282898327	metal ion homeostasis
GO:0051129	30	1	0.289136096	negative regulation of cell organization and biogenesis
GO:0051261	30	1	0.289136096	protein depolymerization
GO:0006950	881	12	0.290343637	response to stress
GO:0043170	2405	30	0.294850169	macromolecule metabolism
GO:0006793	641	9	0.297996009	phosphorus metabolism
GO:0006796	641	9	0.297996009	phosphate metabolism
GO:0006139	2328	29	0.302867492	nucleobase, nucleoside, nucleotide and nucleic acid metabolism
GO:0044265	563	8	0.302929064	cellular macromolecule catabolism
GO:0030003	98	2	0.303842584	cation homeostasis
GO:0006836	33	1	0.313029959	neurotransmitter transport
GO:0009314	33	1	0.313029959	response to radiation
GO:0006338	33	1	0.313029959	chromatin remodeling
GO:0006401	33	1	0.313029959	RNA catabolism
GO:0008064	33	1	0.313029959	regulation of actin polymerization and/or depolymerization

GO:0009057	575	8	0.323282234	macromolecule catabolism
GO:0043412	1160	15	0.327736452	biopolymer modification
GO:0006118	256	4	0.327827688	electron transport
GO:0030832	35	1	0.328515609	regulation of actin filament length
GO:0051234	1841	23	0.329301968	establishment of localization
GO:0006511	105	2	0.332978024	ubiquitin-dependent protein catabolism
GO:0019941	105	2	0.332978024	modification-dependent protein catabolism
GO:0030333	36	1	0.336128303	antigen processing
GO:0044267	2109	26	0.339786402	cellular protein metabolism
GO:0031324	183	3	0.341603422	negative regulation of cellular metabolism
GO:0006464	1087	14	0.342007005	protein modification
GO:0016485	37	1	0.343655528	protein processing
GO:0030099	37	1	0.343655528	myeloid cell differentiation
GO:0051258	37	1	0.343655528	protein polymerization
GO:0051179	1857	23	0.345806478	localization
GO:0009056	756	10	0.348722494	catabolism
GO:0006821	38	1	0.351098233	chloride transport
GO:0030518	38	1	0.351098233	steroid hormone receptor signaling pathway
GO:0006812	346	5	0.352554543	cation transport
GO:0030522	39	1	0.358457359	intracellular receptor-mediated signaling pathway
GO:0007519	39	1	0.358457359	myogenesis
GO:0019538	2129	26	0.35964826	protein metabolism
GO:0030001	269	4	0.361371404	metal ion transport
GO:0048468	40	1	0.365733835	cell development
GO:0007588	40	1	0.365733835	excretion
GO:0006766	40	1	0.365733835	vitamin metabolism
GO:0019318	113	2	0.365849479	hexose metabolism
GO:0005996	115	2	0.373976562	monosaccharide metabolism
GO:0051244	2319	28	0.376332586	regulation of cellular physiological process
GO:0045859	116	2	0.37802478	regulation of protein kinase activity
GO:0009063	42	1	0.380042504	amino acid catabolism
GO:0006096	42	1	0.380042504	glycolysis
GO:0051338	117	2	0.382062402	regulation of transferase activity
GO:0007126	44	1	0.394031471	meiosis
GO:0051321	44	1	0.394031471	meiotic cell cycle
GO:0051327	44	1	0.394031471	M phase of meiotic cell cycle
GO:0008154	45	1	0.400908284	actin polymerization and/or depolymerization
GO:0009310	45	1	0.400908284	amine catabolism
GO:0007229	45	1	0.400908284	integrin-mediated signaling pathway
GO:0043037	122	2	0.402082131	translation
GO:0006631	124	2	0.410006724	fatty acid metabolism
GO:0044270	47	1	0.414430914	nitrogen compound catabolism
GO:0030036	126	2	0.41788089	actin cytoskeleton organization and biogenesis
GO:0007586	49	1	0.427651239	digestion
GO:0015980	132	2	0.441183952	energy derivation by oxidation of organic compounds
GO:0051128	52	1	0.446929503	regulation of cell organization and biogenesis
GO:0008632	53	1	0.453211596	apoptotic program

GO:0050791	2407	28	0.464726642	regulation of physiological process
GO:0008285	139	2	0.467723139	negative regulation of cell proliferation
GO:0030029	139	2	0.467723139	actin filament-based process
GO:0006810	1802	21	0.475370521	transport
GO:0009615	58	1	0.483577711	response to virus
GO:0045786	145	2	0.489877336	negative regulation of progression through cell cycle
GO:0000122	61	1	0.500989467	negative regulation of transcription from RNA polymerase II promoter
GO:0016481	151	2	0.511455199	negative regulation of transcription
GO:0006445	64	1	0.517819729	regulation of translation
GO:0009790	65	1	0.523303938	embryonic development
GO:0006811	515	6	0.529030259	ion transport
GO:0045934	159	2	0.539294028	negative regulation of nucleobase, nucleoside, nucleotide and nucleic acid metabolism
GO:0050794	2488	28	0.546535717	regulation of cellular process
GO:0042127	265	3	0.579740652	regulation of cell proliferation
GO:0044262	265	3	0.579740652	cellular carbohydrate metabolism
GO:0006508	451	5	0.581587902	proteolysis and peptidolysis
GO:0044257	451	5	0.581587902	cellular protein catabolism
GO:0045893	77	1	0.584487977	positive regulation of transcription, DNA-dependent
GO:0051246	174	2	0.588520429	regulation of protein metabolism
GO:0007596	78	1	0.589220777	blood coagulation
GO:0050817	79	1	0.593900192	coagulation
GO:0006814	80	1	0.598526818	sodium ion transport
GO:0030163	463	5	0.605244374	protein catabolism
GO:0000902	183	2	0.616160582	cellular morphogenesis
GO:0006915	471	5	0.620598015	apoptosis
GO:0042060	85	1	0.620888522	wound healing
GO:0012501	473	5	0.62438248	programmed cell death
GO:0043285	474	5	0.6262665	biopolymer catabolism
GO:0006968	87	1	0.629483824	cellular defense response
GO:0006486	92	1	0.650137036	protein amino acid glycosylation
GO:0043413	93	1	0.654128761	biopolymer glycosylation
GO:0007517	94	1	0.658075384	muscle development
GO:0009101	96	1	0.665835338	glycoprotein biosynthesis
GO:0045892	97	1	0.669649662	negative regulation of transcription, DNA-dependent
GO:0008219	498	5	0.669791536	cell death
GO:0007242	879	9	0.671454082	intracellular signaling cascade
GO:0016265	501	5	0.674998054	death
GO:0006470	99	1	0.677149442	protein amino acid dephosphorylation
GO:0019222	1629	17	0.680835041	regulation of metabolism
GO:0045941	100	1	0.680835858	positive regulation of transcription
GO:0007417	101	1	0.68448059	central nervous system development
GO:0051242	411	4	0.68864034	positive regulation of cellular physiological process
GO:0009165	103	1	0.691646861	nucleotide biosynthesis
GO:0019219	1458	15	0.694896933	regulation of nucleobase, nucleoside, nucleotide and nucleic acid metabolism
GO:0006066	213	2	0.698095757	alcohol metabolism
GO:0048232	105	1	0.698651926	male gamete generation
GO:0045935	105	1	0.698651926	positive regulation of nucleobase, nucleoside, nucleotide and nucleic acid metabolism

GO:0007283	105	1	0.698651926	spermatogenesis
GO:0050790	214	2	0.700563213	regulation of enzyme activity
GO:0006643	106	1	0.702095133	membrane lipid metabolism
GO:0015698	106	1	0.702095133	inorganic anion transport
GO:0006417	107	1	0.705499379	regulation of protein biosynthesis
GO:0031323	1566	16	0.710670602	regulation of cellular metabolism
GO:0016311	110	1	0.715482692	dephosphorylation
GO:0031326	111	1	0.718735412	regulation of cellular biosynthesis
GO:0009100	111	1	0.718735412	glycoprotein metabolism
GO:0043119	428	4	0.719023112	positive regulation of physiological process
GO:0050789	2689	28	0.730814195	regulation of biological process
GO:0006897	115	1	0.731382136	endocytosis
GO:0009889	116	1	0.734454809	regulation of biosynthesis
GO:0009611	341	3	0.746479408	response to wounding
GO:0008284	121	1	0.74930381	positive regulation of cell proliferation
GO:0016568	122	1	0.752173411	chromatin modification
GO:0007276	125	1	0.760588497	gametogenesis
GO:0045449	1433	14	0.763262635	regulation of transcription
GO:0006820	128	1	0.768720552	anion transport
GO:0005975	355	3	0.770724765	carbohydrate metabolism
GO:0006936	132	1	0.779139271	muscle contraction
GO:0006355	1359	13	0.782661835	regulation of transcription, DNA-dependent
GO:0031325	136	1	0.789093041	positive regulation of cellular metabolism
GO:0006917	140	1	0.798602415	induction of apoptosis
GO:0012502	140	1	0.798602415	induction of programmed cell death
GO:0048522	482	4	0.801130283	positive regulation of cellular process
GO:0044255	383	3	0.813603121	cellular lipid metabolism
GO:0019953	149	1	0.818474086	sexual reproduction
GO:0009893	149	1	0.818474086	positive regulation of metabolism
GO:0006350	1499	14	0.819535363	transcription
GO:0006351	1407	13	0.823128053	transcription, DNA-dependent
GO:0043065	155	1	0.83063019	positive regulation of apoptosis
GO:0043068	156	1	0.832576311	positive regulation of programmed cell death
GO:0006412	407	3	0.844835309	protein biosynthesis
GO:0006457	169	1	0.855948803	protein folding
GO:0016337	172	1	0.860865782	cell-cell adhesion
GO:0050896	1578	14	0.873533173	response to stimulus
GO:0042221	183	1	0.877514145	response to chemical substance
GO:0009628	318	2	0.879421494	response to abiotic stimulus
GO:0006954	185	1	0.880321751	inflammatory response
GO:0048518	565	4	0.888903466	positive regulation of biological process
GO:0009613	458	3	0.896616654	response to pest, pathogen or parasite
GO:0043207	463	3	0.900761161	response to external biotic stimulus
GO:0009059	464	3	0.901572119	macromolecule biosynthesis
GO:0000004	465	3	0.902377177	biological process unknown
GO:0006629	469	3	0.905539095	lipid metabolism
GO:0046903	208	1	0.9083701	secretion

GO:0000003	208	1	0.9083701	reproduction
GO:0015672	223	1	0.923045246	monovalent inorganic cation transport
GO:0009605	963	7	0.928701494	response to external stimulus
GO:0009607	857	6	0.930766834	response to biotic stimulus
GO:0007399	385	2	0.935877053	neurogenesis
GO:0007155	518	3	0.93746852	cell adhesion
GO:0048513	872	6	0.93759546	organ development
GO:0006512	255	1	0.947022653	ubiquitin cycle
GO:0007275	1543	12	0.947305993	development
GO:0030154	409	2	0.949199962	cell differentiation
GO:0046907	448	2	0.965434379	intracellular transport
GO:0016192	296	1	0.967230453	vesicle-mediated transport
GO:0009653	1051	6	0.983654108	morphogenesis
GO:0009887	807	4	0.984504887	organogenesis
GO:0016070	391	1	0.989327313	RNA metabolism
GO:0050874	1531	9	0.993895183	organismal physiological process
GO:0007267	465	1	0.995584036	cell-cell signaling
GO:0006955	684	2	0.997069939	immune response
GO:0006952	751	2	0.998595913	defense response
GO:0007165	2168	12	0.999431901	signal transduction
GO:0007154	2730	15	0.999921006	cell communication
GO:0007166	952	1	0.999989121	cell surface receptor linked signal transduction
GO:0008150	9032	102	1	biological_process

16.3 GO: Molecular Function

GO ID	genes present in chip	genes present in tissues	p-value	GO term
GO:0004998	2	2	0.000134144661339113	transferrin receptor activity
GO:0016725	2	2	0.000134144661339113	oxidoreductase activity, acting on CH2 groups
GO:0016728	2	2	0.000134144661339113	oxidoreductase activity, acting on CH2 groups, disulfide as acceptor
GO:0004357	2	2	0.000134144661339113	glutamate-cysteine ligase activity
GO:0004748	2	2	0.000134144661339113	ribonucleoside-diphosphate reductase activity
GO:0005344	12	3	0.000312356129918380	oxygen transporter activity
GO:0003918	4	2	0.000792657311549805	DNA topoisomerase (ATP-hydrolyzing) activity
GO:0005506	212	9	0.00079311003120434	iron ion binding
GO:0005521	5	2	0.0013110493674021	lamin binding
GO:0003916	6	2	0.00195163267152852	DNA topoisomerase activity
GO:0015101	7	2	0.00271154519063022	organic cation transporter activity
GO:0003824	3409	54	0.00334611881616247	catalytic activity
GO:0016634	8	2	0.00358797390973613	oxidoreductase activity, acting on the CH-CH group of donors, oxygen as acceptor
GO:0019825	31	3	0.00543523045486913	oxygen binding
GO:0046982	61	4	0.00544186177757883	protein heterodimerization activity
GO:0016410	33	3	0.00648726939368043	N-acyltransferase activity
GO:0016627	37	3	0.00893368628818444	oxidoreductase activity, acting on the CH-CH group of donors
GO:0042802	158	6	0.0101953962081435	protein self binding
GO:0046906	74	4	0.0106968234459397	tetrapyrrole binding
GO:0020037	74	4	0.0106968234459397	heme binding
GO:0004853	1	1	0.0116340006288645	uroporphyrinogen decarboxylase activity
GO:0004655	1	1	0.0116340006288645	porphobilinogen synthase activity
GO:0008310	1	1	0.0116340006288645	single-stranded DNA specific 3'-5' exodeoxyribonuclease activity
GO:0008413	1	1	0.0116340006288645	8-oxo-7,8-dihydroguanine triphosphatase activity
GO:0050355	1	1	0.0116340006288645	triphosphatase activity
GO:0008859	1	1	0.0116340006288645	exoribonuclease II activity
GO:0042083	1	1	0.0116340006288645	5,10-methylenetetrahydrofolate-dependent methyltransferase activity
GO:0008513	1	1	0.0116340006288645	organic cation porter activity
GO:0008519	1	1	0.0116340006288645	ammonium transporter activity
GO:0047290	1	1	0.0116340006288645	(alpha-N-acetylneuraminyl-2,3-beta-galactosyl-1,3)-N-acetyl-galactosaminide 6-alpha-sialyltransferase activity
GO:0004096	1	1	0.0116340006288645	catalase activity
GO:0030337	1	1	0.0116340006288645	DNA polymerase processivity factor activity
GO:0042602	1	1	0.0116340006288645	flavin reductase activity
GO:0016538	1	1	0.0116340006288645	cyclin-dependent protein kinase regulator activity
GO:0004325	1	1	0.0116340006288645	ferrochelatase activity
GO:0004418	1	1	0.0116340006288645	hydroxymethylbilane synthase activity
GO:0004446	1	1	0.0116340006288645	multiple inositol-polyphosphate phosphatase activity
GO:0004193	1	1	0.0116340006288645	cathepsin E activity
GO:0004137	1	1	0.0116340006288645	deoxycytidine kinase activity
GO:0004799	1	1	0.0116340006288645	thymidylate synthase activity
GO:0004751	1	1	0.0116340006288645	ribose-5-phosphate isomerase activity
GO:0004729	1	1	0.0116340006288645	protoporphyrinogen oxidase activity
GO:0004731	1	1	0.0116340006288645	purine-nucleoside phosphorylase activity

GO:0016836	43	3	0.0134943674799455	hydro-lyase activity
GO:0046983	170	6	0.0142507650939473	protein dimerization activity
GO:0004089	16	2	0.0144720271942443	carbonate dehydratase activity
GO:0004468	16	2	0.0144720271942443	lysine N-acetyltransferase activity
GO:0004402	16	2	0.0144720271942443	histone acetyltransferase activity
GO:0016829	127	5	0.0161313626077341	lyase activity
GO:0016835	50	3	0.0202165686264249	carbon-oxygen lyase activity
GO:0016853	92	4	0.0221769298792090	isomerase activity
GO:0043014	2	1	0.0231338565963904	alpha-tubulin binding
GO:0004694	2	1	0.0231338565963904	eukaryotic translation initiation factor 2alpha kinase activity
GO:0004619	2	1	0.0231338565963904	phosphoglycerate mutase activity
GO:0008379	2	1	0.0231338565963904	thioredoxin peroxidase activity
GO:0008198	2	1	0.0231338565963904	ferrous iron binding
GO:0008297	2	1	0.0231338565963904	single-stranded DNA specific exodeoxyribonuclease activity
GO:0003870	2	1	0.0231338565963904	5-aminolevulinate synthase activity
GO:0004053	2	1	0.0231338565963904	arginase activity
GO:0004082	2	1	0.0231338565963904	bisphosphoglycerate mutase activity
GO:0004083	2	1	0.0231338565963904	bisphosphoglycerate phosphatase activity
GO:0004074	2	1	0.0231338565963904	biliverdin reductase activity
GO:0016749	2	1	0.0231338565963904	N-succinyltransferase activity
GO:0004109	2	1	0.0231338565963904	coproporphyrinogen oxidase activity
GO:0015204	2	1	0.0231338565963904	urea transporter activity
GO:0004601	22	2	0.0266274282695180	peroxidase activity
GO:0016684	22	2	0.0266274282695180	oxidoreductase activity, acting on peroxide as acceptor
GO:0016740	1169	21	0.0272693467293350	transferase activity
GO:0030554	1028	19	0.0274614513422526	adenyl nucleotide binding
GO:0005275	58	3	0.0297811243640132	amine transporter activity
GO:0030506	3	1	0.0345011007433451	ankyrin binding
GO:0008296	3	1	0.0345011007433451	3'-5'-exodeoxyribonuclease activity
GO:0045502	3	1	0.0345011007433451	dynein binding
GO:0003917	3	1	0.0345011007433451	DNA topoisomerase type I activity
GO:0016748	3	1	0.0345011007433451	succinyltransferase activity
GO:0004674	326	8	0.036039047188259	protein serine/threonine kinase activity
GO:0042803	64	3	0.0382659203172545	protein homodimerization activity
GO:0008080	28	2	0.0416569964248792	N-acetyltransferase activity
GO:0043015	4	1	0.0457372485539437	gamma-tubulin binding
GO:0001517	4	1	0.0457372485539437	N-acetylglucosamine 6-O-sulfotransferase activity
GO:0019136	4	1	0.0457372485539437	deoxynucleoside kinase activity
GO:0050811	4	1	0.0457372485539437	GABA receptor binding
GO:0030492	4	1	0.0457372485539437	hemoglobin binding
GO:0016209	31	2	0.0501106595303912	antioxidant activity
GO:0016301	609	12	0.0502133852771681	kinase activity
GO:0005524	970	17	0.0556941772014725	ATP binding
GO:0008301	5	1	0.0568437983505793	DNA bending activity
GO:0048487	5	1	0.0568437983505793	beta-tubulin binding
GO:0042826	5	1	0.0568437983505793	histone deacetylase binding
GO:0016772	708	13	0.0669646054938018	transferase activity, transferring phosphorus-containing groups

GO:0016308	6	1	0.0678222314863889	1-phosphatidylinositol-4-phosphate 5-kinase activity
GO:0016861	6	1	0.0678222314863889	intramolecular oxidoreductase activity, interconverting aldoses and ketoses
GO:0015359	6	1	0.0678222314863889	amino acid permease activity
GO:0016407	39	2	0.0752514594626067	acetyltransferase activity
GO:0005200	86	3	0.0784759387627624	structural constituent of cytoskeleton
GO:0004529	7	1	0.0786740125356817	exodeoxyribonuclease activity
GO:0016895	7	1	0.0786740125356817	exodeoxyribonuclease activity, producing 5'-phosphomonoesters
GO:0016868	7	1	0.0786740125356817	intramolecular transferase activity, phosphotransferases
GO:0004194	7	1	0.0786740125356817	pepsin A activity
GO:0003713	140	4	0.0802498884068395	transcription coactivator activity
GO:0017076	1248	20	0.0833147351955007	purine nucleotide binding
GO:0015645	8	1	0.089400589482246	fatty-acid ligase activity
GO:0008191	8	1	0.089400589482246	metalloendopeptidase inhibitor activity
GO:0016307	8	1	0.089400589482246	phosphatidylinositol phosphate kinase activity
GO:0016813	8	1	0.089400589482246	hydrolase activity, acting on carbon-nitrogen (but not peptide) bonds, in linear amidines
GO:0004467	8	1	0.089400589482246	long-chain-fatty-acid-CoA ligase activity
GO:0004190	8	1	0.089400589482246	aspartic-type endopeptidase activity
GO:0016773	532	10	0.0902442976990754	phosphotransferase activity, alcohol group as acceptor
GO:0030145	92	3	0.0917116879349712	manganese ion binding
GO:0004693	9	1	0.100003393905562	cyclin-dependent protein kinase activity
GO:0003993	9	1	0.100003393905562	acid phosphatase activity
GO:0030552	9	1	0.100003393905562	3',5'-cAMP binding
GO:0019887	47	2	0.103500695039645	protein kinase regulator activity
GO:0008415	98	3	0.105807385345067	acyltransferase activity
GO:0016747	98	3	0.105807385345067	transferase activity, transferring groups other than amino-acyl groups
GO:0046914	1596	24	0.105947579051698	transition metal ion binding
GO:0015171	48	2	0.107212042802197	amino acid transporter activity
GO:0008603	10	1	0.110483841164950	cAMP-dependent protein kinase regulator activity
GO:0000175	10	1	0.110483841164950	3'-5'-exoribonuclease activity
GO:0016208	10	1	0.110483841164950	AMP binding
GO:0016746	104	3	0.12069619958959	transferase activity, transferring acyl groups
GO:0004532	11	1	0.120843330581662	exoribonuclease activity
GO:0019206	11	1	0.120843330581662	nucleoside kinase activity
GO:0016896	11	1	0.120843330581662	exoribonuclease activity, producing 5'-phosphomonoesters
GO:0016866	11	1	0.120843330581662	intramolecular transferase activity
GO:0016646	11	1	0.120843330581662	oxidoreductase activity, acting on the CH-NH group of donors, NAD or NADP as acceptor
GO:0008134	293	6	0.126190550680442	transcription factor binding
GO:0003712	230	5	0.129958835579090	transcription cofactor activity
GO:0016877	12	1	0.131083245618959	ligase activity, forming carbon-sulfur bonds
GO:0015175	12	1	0.131083245618959	neutral amino acid transporter activity
GO:0016491	509	9	0.137741897126141	oxidoreductase activity
GO:0016791	171	4	0.138234282640754	phosphoric monoester hydrolase activity
GO:0019207	58	2	0.146067249631244	kinase regulator activity
GO:0004672	447	8	0.148987980622538	protein kinase activity
GO:0008373	14	1	0.151209808184784	sialyltransferase activity
GO:0016628	14	1	0.151209808184784	oxidoreductase activity, acting on the CH-CH group of donors, NAD or NADP as acceptor
GO:0000166	1432	21	0.152345738028975	nucleotide binding

GO:0051015	15	1	0.161099144942576	actin filament binding
GO:0005247	16	1	0.170874286125798	voltage-gated chloride channel activity
GO:0005342	66	2	0.178902814996792	organic acid transporter activity
GO:0046943	66	2	0.178902814996792	carboxylic acid transporter activity
GO:0019212	17	1	0.180536538539450	phosphatase inhibitor activity
GO:0016796	17	1	0.180536538539450	exonuclease activity, active with either ribo- or deoxyribonucleic acids and producing 5'-phosphomonoesters
GO:0008430	18	1	0.190087194169660	selenium binding
GO:0016645	18	1	0.190087194169660	oxidoreductase activity, acting on the CH-NH group of donors
GO:0030551	18	1	0.190087194169660	cyclic nucleotide binding
GO:0050681	19	1	0.199527530350188	androgen receptor binding
GO:0001727	19	1	0.199527530350188	lipid kinase activity
GO:0003690	20	1	0.208858809927077	double-stranded DNA binding
GO:0015002	21	1	0.218082281421467	heme-copper terminal oxidase activity
GO:0016675	21	1	0.218082281421467	oxidoreductase activity, acting on heme group of donors
GO:0016676	21	1	0.218082281421467	oxidoreductase activity, acting on heme group of donors, oxygen as acceptor
GO:0004129	21	1	0.218082281421467	cytochrome-c oxidase activity
GO:0035258	23	1	0.236210723587061	steroid hormone receptor binding
GO:0005478	24	1	0.245118121116147	intracellular transporter activity
GO:0008408	24	1	0.245118121116147	3'-5' exonuclease activity
GO:0016831	24	1	0.245118121116147	carboxy-lyase activity
GO:0004536	25	1	0.253922564591623	deoxyribonuclease activity
GO:0004428	25	1	0.253922564591623	inositol or phosphatidylinositol kinase activity
GO:0005178	26	1	0.26262523328964	integrin binding
GO:0008138	26	1	0.26262523328964	protein tyrosine/serine/threonine phosphatase activity
GO:0003704	26	1	0.26262523328964	specific RNA polymerase II transcription factor activity
GO:0016769	26	1	0.26262523328964	transferase activity, transferring nitrogenous groups
GO:0004437	26	1	0.26262523328964	inositol or phosphatidylinositol phosphatase activity
GO:0050662	88	2	0.273058520578788	coenzyme binding
GO:0042578	228	4	0.274052370728079	phosphoric ester hydrolase activity
GO:0008022	28	1	0.279729896681571	protein C-terminus binding
GO:0005279	28	1	0.279729896681571	amino acid-polyamine transporter activity
GO:0003887	28	1	0.279729896681571	DNA-directed DNA polymerase activity
GO:0003697	28	1	0.279729896681571	single-stranded DNA binding
GO:0015203	28	1	0.279729896681571	polyamine transporter activity
GO:0016763	29	1	0.288134183601402	transferase activity, transferring pentosyl groups
GO:0016860	31	1	0.304652301190380	intramolecular oxidoreductase activity
GO:0016830	31	1	0.304652301190380	carbon-carbon lyase activity
GO:0008146	32	1	0.312768346886265	sulfotransferase activity
GO:0003774	98	2	0.316172766758597	motor activity
GO:0008307	33	1	0.320790506261134	structural constituent of muscle
GO:0048037	100	2	0.324741243967179	cofactor binding
GO:0019205	34	1	0.328719855630614	nucleobase, nucleoside, nucleotide kinase activity
GO:0008094	34	1	0.328719855630614	DNA-dependent ATPase activity
GO:0016881	101	2	0.329015674534144	acid-amino acid ligase activity
GO:0019888	35	1	0.336557459083333	protein phosphatase regulator activity
GO:0019208	36	1	0.344304368618548	phosphatase regulator activity
GO:0016782	36	1	0.344304368618548	transferase activity, transferring sulfur-containing groups

GO:0016765	37	1	0.351961624282234	transferase activity, transferring alkyl or aryl (other than methyl) groups
GO:0043169	2313	29	0.355517801699916	cation binding
GO:0004527	40	1	0.374405692020287	exonuclease activity
GO:0015631	42	1	0.388938676238334	tubulin binding
GO:0035257	42	1	0.388938676238334	nuclear hormone receptor binding
GO:0019992	42	1	0.388938676238334	diacylglycerol binding
GO:0005254	43	1	0.396079197023421	chloride channel activity
GO:0003743	43	1	0.396079197023421	translation initiation factor activity
GO:0051427	43	1	0.396079197023421	hormone receptor binding
GO:0004721	118	2	0.40028872359345	phosphoprotein phosphatase activity
GO:0050660	44	1	0.403137020684234	FAD binding
GO:0005554	522	7	0.405721012855833	molecular function unknown
GO:0004540	45	1	0.41011309636119	ribonuclease activity
GO:0031404	45	1	0.41011309636119	chloride ion binding
GO:0043168	45	1	0.41011309636119	anion binding
GO:0003777	45	1	0.41011309636119	microtubule motor activity
GO:0003924	121	2	0.412523985822978	GTPase activity
GO:0005253	46	1	0.417008362399934	anion channel activity
GO:0005215	1030	13	0.421764276080607	transporter activity
GO:0003682	47	1	0.423823746472983	chromatin binding
GO:0043167	2556	31	0.428432110847954	ion binding
GO:0016879	127	2	0.436622506641182	ligase activity, forming carbon-nitrogen bonds
GO:0005507	49	1	0.437218526766838	copper ion binding
GO:0046872	2495	30	0.452450530428672	metal ion binding
GO:0016874	221	3	0.476805269353395	ligase activity
GO:0008135	62	1	0.517068192248394	translation factor activity, nucleic acid binding
GO:0015290	149	2	0.520141580976657	electrochemical potential-driven transporter activity
GO:0015291	149	2	0.520141580976657	porter activity
GO:0017111	410	5	0.522148840289173	nucleoside-triphosphatase activity
GO:0003677	1384	16	0.552512116494805	DNA binding
GO:0031402	70	1	0.56051356737214	sodium ion binding
GO:0016462	430	5	0.565036461210529	pyrophosphatase activity
GO:0016818	432	5	0.569221694705436	hydrolase activity, acting on acid anhydrides, in phosphorus-containing anhydrides
GO:0045182	72	1	0.570755288723367	translation regulator activity
GO:0016810	72	1	0.570755288723367	hydrolase activity, acting on carbon-nitrogen (but not peptide) bonds
GO:0016817	433	5	0.571306805647763	hydrolase activity, acting on acid anhydrides
GO:0016887	255	3	0.573532929805728	ATPase activity
GO:0016757	165	2	0.575556467958535	transferase activity, transferring glycosyl groups
GO:0004725	75	1	0.585675997483984	protein tyrosine phosphatase activity
GO:0016788	446	5	0.597941336616998	hydrolase activity, acting on ester bonds
GO:0015293	83	1	0.622998414604203	symporter activity
GO:0008168	84	1	0.627422941563277	methyltransferase activity
GO:0008092	279	3	0.6348639851193	cytoskeletal protein binding
GO:0016741	87	1	0.64039010274459	transferase activity, transferring one-carbon groups
GO:0003676	1990	22	0.643805703796787	nucleic acid binding
GO:0004857	187	2	0.643990355740018	enzyme inhibitor activity
GO:0003702	197	2	0.672130348520059	RNA polymerase II transcription factor activity

GO:0008509	95	1	0.672823310699875	anion transporter activity
GO:0016779	95	1	0.672823310699875	nucleotidyltransferase activity
GO:0005386	398	4	0.686345308927823	carrier activity
GO:0004866	99	1	0.687935334811713	endopeptidase inhibitor activity
GO:0030414	100	1	0.69160396199196	protease inhibitor activity
GO:0030528	963	10	0.694825749585437	transcription regulator activity
GO:0009055	104	1	0.705855935002794	electron carrier activity
GO:0004518	105	1	0.709315720020775	nuclease activity
GO:0030234	509	5	0.71309528330722	enzyme regulator activity
GO:0005488	7228	82	0.7221895039623	binding
GO:0005516	112	1	0.732430631590697	calmodulin binding
GO:0015078	112	1	0.732430631590697	hydrogen ion transporter activity
GO:0005096	117	1	0.747816738706201	GTPase activator activity
GO:0015077	121	1	0.759491592630576	monovalent inorganic cation transporter activity
GO:0005244	124	1	0.767894733113326	voltage-gated ion channel activity
GO:0051082	127	1	0.776006828844475	unfolded protein binding
GO:0016787	1424	14	0.791555518005581	hydrolase activity
GO:0000287	251	2	0.794366252865283	magnesium ion binding
GO:0015399	140	1	0.808033009473207	primary active transporter activity
GO:0005515	3709	39	0.818463076606118	protein binding
GO:0031420	146	1	0.82124086621944	alkali metal ion binding
GO:0005198	488	4	0.826496436024334	structural molecule activity
GO:0019899	151	1	0.831556425207532	enzyme binding
GO:0015268	274	2	0.833019020363921	alpha-type channel activity
GO:0005489	152	1	0.83354761123543	electron transporter activity
GO:0008270	1287	12	0.833971479468006	zinc ion binding
GO:0015267	284	2	0.847703476219215	channel or pore class transporter activity
GO:0008233	399	3	0.849159002182903	peptidase activity
GO:0004713	161	1	0.850450364114757	protein-tyrosine kinase activity
GO:0015075	523	4	0.865040523056969	ion transporter activity
GO:0008324	421	3	0.873753508685785	cation transporter activity
GO:0003700	649	5	0.881768048004615	transcription factor activity
GO:0003723	436	3	0.888445751173076	RNA binding
GO:0008047	188	1	0.891606920138235	enzyme activator activity
GO:0003779	189	1	0.892893312938904	actin binding
GO:0030695	219	1	0.925181742656359	GTPase regulator activity
GO:0005525	226	1	0.931201403451022	GTP binding
GO:0042623	229	1	0.933631936768746	ATPase activity, coupled
GO:0019001	235	1	0.938240598083772	guanyl nucleotide binding
GO:0005509	646	4	0.948302217986576	calcium ion binding
GO:0005216	252	1	0.949645994422795	ion channel activity
GO:0005102	528	3	0.949678956378386	receptor binding
GO:0004175	288	1	0.967361955145786	endopeptidase activity
GO:0004872	1060	7	0.97011872252111	receptor activity
GO:0008289	298	1	0.97107422388068	lipid binding
GO:0004888	694	2	0.997864797505692	transmembrane receptor activity
GO:0004871	1837	10	0.999105731145734	signal transducer activity

GO:0003674	9541	111	1 molecular_function

16.4 GO: Cellular Component

GO ID	genes present in chip	genes present in tissues	p-value	GO term
GO:0005833	10	4	2.98143147514759e-06	hemoglobin complex
GO:0000793	23	5	4.61734343347116e-06	condensed chromosome
GO:0005694	203	10	8.74667451189899e-05	chromosome
GO:0005819	68	6	0.000107214662710656	spindle
GO:0000796	3	2	0.000371784774864311	condensin complex
GO:0005876	14	3	0.000456932848671222	spindle microtubule
GO:0015630	191	7	0.0055707958550314	microtubule cytoskeleton
GO:0005622	5419	73	0.00590205719199843	intracellular
GO:0005971	1	1	0.0112307514183171	ribonucleoside-diphosphate reductase complex
GO:0000228	42	3	0.0114855414879150	nuclear chromosome
GO:0005623	7802	94	0.0124318616281360	cell
GO:0031300	90	4	0.0183186888458729	intrinsic to organelle membrane
GO:0031304	2	1	0.0223366589116492	intrinsic to mitochondrial inner membrane
GO:0005827	2	1	0.0223366589116492	polar microtubule
GO:0000777	2	1	0.0223366589116492	condensed chromosome kinetochore
GO:0000779	2	1	0.0223366589116492	condensed chromosome, pericentric region
GO:0000940	2	1	0.0223366589116492	outer kinetochore of condensed chromosome
GO:0005737	2530	38	0.0227848199401972	cytoplasm
GO:0043228	977	18	0.0229402931274595	non-membrane-bound organelle
GO:0043232	977	18	0.0229402931274595	intracellular non-membrane-bound organelle
GO:0005634	2704	40	0.0240007467799042	nucleus
GO:0000785	100	4	0.0258431142481928	chromatin
GO:0043227	3955	54	0.0315606419341496	membrane-bound organelle
GO:0043231	3955	54	0.0315606419341496	intracellular membrane-bound organelle
GO:0005816	3	1	0.0333190959800422	spindle pole body
GO:0005741	26	2	0.0340622636391169	mitochondrial outer membrane
GO:0043226	4515	60	0.035579422984278	organelle
GO:0043229	4515	60	0.035579422984278	intracellular organelle
GO:0031229	4	1	0.0441794211699815	intrinsic to nuclear inner membrane
GO:0005639	4	1	0.0441794211699815	integral to nuclear inner membrane
GO:0005740	119	4	0.04463866027836	mitochondrial membrane
GO:0031228	35	2	0.0584559795199066	intrinsic to Golgi membrane
GO:0031090	377	8	0.0609085987178874	organelle membrane
GO:0005874	132	4	0.0609598756727963	microtubule
GO:0005663	6	1	0.0655390962974844	DNA replication factor C complex
GO:0000930	6	1	0.0655390962974844	gamma-tubulin complex
GO:0005635	83	3	0.066331376309483	nuclear membrane
GO:0000786	40	2	0.0739447488857851	nucleosome
GO:0005637	7	1	0.0760410900014756	nuclear inner membrane
GO:0016605	8	1	0.0864262596735322	PML body
GO:0008091	9	1	0.0966958914746286	spectrin
GO:0005739	490	9	0.0984694280628076	mitochondrion
GO:0016363	10	1	0.106851257553323	nuclear matrix

GO:0005952	10	1	0.106851257553323	cAMP-dependent protein kinase complex
GO:0000922	51	2	0.111809601368078	spindle pole
GO:0019867	52	2	0.115465768298730	outer membrane
GO:0005802	11	1	0.116893616196806	Golgi trans face
GO:0030864	12	1	0.126824211980355	cortical actin cytoskeleton
GO:0012505	246	5	0.142707071350424	endomembrane system
GO:0030027	14	1	0.146355025594711	lamellipodium
GO:0030894	14	1	0.146355025594711	replisome
GO:0001726	14	1	0.146355025594711	ruffle
GO:0005856	612	10	0.147921495519414	cytoskeleton
GO:0005768	62	2	0.153522471625118	endosome
GO:0005859	15	1	0.155957665339268	muscle myosin
GO:0005657	15	1	0.155957665339268	replication fork
GO:0016460	15	1	0.155957665339268	myosin II
GO:0000794	15	1	0.155957665339268	condensed nuclear chromosome
GO:0016604	16	1	0.165453386339279	nuclear body
GO:0030863	16	1	0.165453386339279	cortical cytoskeleton
GO:0005777	67	2	0.173366955473958	peroxisome
GO:0000139	67	2	0.173366955473958	Golgi membrane
GO:0042579	67	2	0.173366955473958	microbody
GO:0000776	17	1	0.174843366796892	kinetochore
GO:0005778	19	1	0.193310754791442	peroxisomal membrane
GO:0005829	276	5	0.198262020890321	cytosol
GO:0031301	76	2	0.210003963002130	integral to organelle membrane
GO:0048471	22	1	0.220247506376295	perinuclear region
GO:0043234	1247	17	0.229324912983797	protein complex
GO:0008305	24	1	0.237708800309977	integrin complex
GO:0031252	24	1	0.237708800309977	leading edge
GO:0000775	27	1	0.263177635498731	chromosome, pericentric region
GO:0019866	89	2	0.264040169307317	inner membrane
GO:0005938	28	1	0.271478654007049	cell cortex
GO:0030173	29	1	0.279687106854223	integral to Golgi membrane
GO:0043292	40	1	0.364155101387454	contractile fiber
GO:0000502	41	1	0.3713293430026	proteasome complex (sensu Eukaryota)
GO:0005813	43	1	0.385438346343835	centrosome
GO:0016459	43	1	0.385438346343835	myosin
GO:0005886	1472	18	0.385929925822582	plasma membrane
GO:0005815	49	1	0.425912071757619	microtubule organizing center
GO:0031224	2681	31	0.460303303455612	intrinsic to membrane
GO:0005941	57	1	0.475800417768003	unlocalized protein complex
GO:0043235	58	1	0.481726683441256	receptor complex
GO:0016021	2674	30	0.541245305628035	integral to membrane
GO:0005875	70	1	0.547873658986789	microtubule associated complex
GO:0042995	70	1	0.547873658986789	cell projection
GO:0015629	183	2	0.613262787446667	actin cytoskeleton
GO:0005743	85	1	0.618918001833102	mitochondrial inner membrane
GO:0005667	91	1	0.644135055015237	transcription factor complex

GO:0005794	195	2	0.647945412408752	Golgi apparatus
GO:0005887	1057	11	0.653435694298226	integral to plasma membrane
GO:0031226	1062	11	0.659785567529436	intrinsic to plasma membrane
GO:0005624	521	5	0.704086491239794	membrane fraction
GO:0005792	108	1	0.706959985050526	microsome
GO:0042598	108	1	0.706959985050526	vesicular fraction
GO:0005654	221	2	0.714685718891915	nucleoplasm
GO:0016020	3514	37	0.729728596074407	membrane
GO:0016023	122	1	0.750351860117441	cytoplasmic membrane-bound vesicle
GO:0031410	124	1	0.756007607854623	cytoplasmic vesicle
GO:0005783	483	4	0.799306591138258	endoplasmic reticulum
GO:0000267	698	6	0.806655380846998	cell fraction
GO:0005625	183	1	0.87619787726375	soluble fraction
GO:0008372	523	3	0.938863236230636	cellular component unknown
GO:0005576	949	2	0.999846657138945	extracellular region
GO:0005575	8637	97	1	cellular_component

17. PB-CD14Monocytes

17.1 PB-CD14Monocytes selective genes (163 genes)

Symbol	Description	Chromosome	Chromosome Location	LocusLink	Cytoband	UniGene
MNDA	myeloid cell nuclear differentiation antigen	1	157067791	4332	1q22	Hs.153837
S100A9	S100 calcium binding protein A9 (calgranulin B)	1	151596953	6280	1q21	Hs.112405
FCN1	ficolin (collagen/fibrinogen domain containing) 1	9	-136941256	2219	9q34	Hs.440898
CSTA	cystatin A (stefin A)	3	123526700	1475	3q21	Hs.518198
CD1D	CD1d molecule	1	156416360	912	1q22-q23	Hs.1799 Hs.387539
LYZ	lysozyme (renal amyloidosis)	12	68028430	4069	12q15	Hs.524579
FGL2	fibrinogen-like 2	7	-76660625	10875	7q11.23	Hs.520989
IGSF6	immunoglobulin superfamily, member 6	16	-21560106	10261	16p12-p13	Hs.530902
HCK	hemopoietic cell kinase	20	30103717	3055	20q11-q12	Hs.126521
EVI2B	ecotropic viral integration site 2B	17	-26654918	2124	17q11.2	Hs.5509
TYROBP	TYRO protein tyrosine kinase binding protein	19	-41087145	7305	19q13.1	Hs.515369
APOBEC3A	apolipoprotein B mRNA editing enzyme, catalytic polypeptide-like 3A	22	37683568	200315	22q13.1-q13.2	Hs.348983
CSPG2	chondroitin sulfate proteoglycan 2 (versican)	5	82803338	1462	5q14.3	Hs.443681
S100A8	S100 calcium binding protein A8 (calgranulin A)	1	-151629132	6279	1q21	Hs.416073
PYCARD	PYD and CARD domain containing	16	-31120309	29108	16p12-p11.2	Hs.499094
ITGAM	integrin, alpha M (complement component 3 receptor 3 subunit)	16	31178788	3684	16p11.2	Hs.172631
S100A12	S100 calcium binding protein A12 (calgranulin C)	1	-151612807	6283	1q21	Hs.19413
COTL1	coactosin-like 1 (Dictyostelium)	16	-83156706	23406	16q24.1	Hs.289092
NCF2	neutrophil cytosolic factor 2 (65kDa, chronic granulomatous disease, autosomal 2)	1	-181791320	4688	1q25	Hs.587558
CTSS	cathepsin S	1	-148969177	1520	1q21	Hs.181301
TNFRSF1B	tumor necrosis factor receptor superfamily, member 1B	1	12149646	7133	1p36.3-p36.2	Hs.256278
PPBP	pro-platelet basic protein (chemokine (C-X-C motif) ligand 7)	4	-75071621	5473	4q12-q13	Hs.2164
C5AR1	complement component 5a receptor 1	19	52504970	728	19q13.3-q13.4	Hs.2161
PTPN6	protein tyrosine phosphatase, non-receptor type 6	12	6930703, 6926000	5777	12p13	Hs.63489
VNN2	vanin 2	6	-133106702	8875	6q23-q24	Hs.293130
NFE2	nuclear factor (erythroid-derived 2), 45kDa	12	-52972164	4778	12q13	Hs.75643
GCH1	GTP cyclohydrolase 1 (dopa-responsive dystonia)	14	-54378475	2643	14q22.1-q22.2	Hs.86724
CD302	CD302 molecule	2	-160333609	9936	2q24.2	Hs.130014
CD36	CD36 molecule (thrombospondin receptor)	7	80105892, 80069458	948	7q11.2	Hs.120949 Hs.633085

LY96	lymphocyte antigen 96	8	75066143	23643	8q21.11	Hs.69328
CD14	CD14 molecule	5	-139991500	929	5q22-q32|5q31.1	Hs.163867
DOCK2	dedicator of cytokinesis 2	5	168996870	1794	5q35.1	Hs.586174
CASP1	caspase 1, apoptosis-related cysteine peptidase (interleukin 1, beta, convertase)	11	-104401451	834	11q23	Hs.2490
PTPRC	protein tyrosine phosphatase, receptor type, C	1	196874847	5788	1q31-q32	Hs.192039
SERPINA1	serpin peptidase inhibitor, clade A (alpha-1 antiproteinase, antitrypsin), member 1	14	-93914450	5265	14q32.1	Hs.525557
QPCT	glutaminyl-peptide cyclotransferase (glutaminyl cyclase)	2	37425256	25797	2p22.2	Hs.79033
GCA	grancalcin, EF-hand calcium binding protein	2	162908895	25801	2q24.2	Hs.377894
RNASE6	ribonuclease, RNase A family, k6	14	20319049	6039	14q11.2	Hs.23262
CFP	complement factor properdin	X	-47368571	5199	Xp11.3-p11.23	Hs.53155
CPVL	carboxypeptidase, vitellogenic-like	7	-29001773	54504	7p15-p14	Hs.233389
BCL2A1	BCL2-related protein A1	15	-78040289	597	15q24.3	Hs.227817
FCER1A	Fc fragment of IgE, high affinity I, receptor for, alpha polypeptide	1	157526127	2205	1q23	Hs.897
EVI2A	ecotropic viral integration site 2A	17	-26668801	2123	17q11.2	Hs.591198
FCGR3B	Fc fragment of IgG, low affinity IIIb, receptor (CD16b)	1	-159859676	2215	1q23	Hs.372679
AOAH	acyloxyacyl hydrolase (neutrophil)	7	-36519134	313	7p14-p12	Hs.488007
DPYD	dihydropyrimidine dehydrogenase	1	-97315889	1806	1p22	Hs.335034
FLJ22662	NA	12	-14547863	79887	12p13.1	Hs.131933
FCGR3A	Fc fragment of IgG, low affinity IIIa, receptor (CD16a)	1	-159778174	2214	1q23	Hs.372679
FGR	Gardner-Rasheed feline sarcoma viral (v-fgr) oncogene homolog	1	-27811397	2268	1p36.2-p36.1	Hs.1422
LRRFIP1	leucine rich repeat (in FLII) interacting protein 1	2	238265667	9208	2q37.3	Hs.471779
CSF3R	colony stimulating factor 3 receptor (granulocyte)	1	-36704231	1441	1p35-p34.3	Hs.524517
SPI1	spleen focus forming virus (SFFV) proviral integration oncogene spi1	11	-47332987	6688	11p11.2	Hs.502511
CX3CR1	chemokine (C-X3-C motif) receptor 1	3	-39279989	1524	3p21|3p21.3	Hs.78913
GMFG	glia maturation factor, gamma	19	-44510841	9535	19q13.2	Hs.5210
ALOX5	arachidonate 5-lipoxygenase	10	45189634	240	10q11.2	Hs.89499
TFEC	transcription factor EC	7	-115362437	22797	7q31.2	Hs.125962
PILRA	paired immunoglobin-like type 2 receptor alpha	7	99809003	29992	7q22.1	Hs.444407
NCF4	neutrophil cytosolic factor 4, 40kDa	22	35586990	4689	22q13.1	Hs.474781
ASGR1	asialoglycoprotein receptor 1	17	-7017476	432	17p13.2	Hs.12056
SULT1A1	sulfotransferase family, cytosolic, 1A, phenol-preferring, member 1	16	-28524418	6817	16p12.1	Hs.567342
GPR65	G protein-coupled receptor 65	14	87541248	8477	14q31-q32.1	Hs.513440
ADCY7	adenylate cyclase 7	16	48879322	113	16q12-q13	Hs.513578
SELL	selectin L (lymphocyte adhesion molecule 1)	1	-167926433	6402	1q23-q25	Hs.82848
CYBB	cytochrome b-245, beta polypeptide (chronic granulomatous disease)	X	37524263	1536	Xp21.1	Hs.292356

GLIPR1	GLI pathogenesis-related 1 (glioma)	12	74160779	11010	12q21.2	Hs.205558 Hs.591034
CLEC4A	C-type lectin domain family 4, member A	12	8167494	50856	12p13	Hs.504657
PLAC8	placenta-specific 8	4	-84230866	51316	4q21.22	Hs.546392
DUSP6	dual specificity phosphatase 6	12	-88265969	1848	12q22-q23	Hs.298654
GIMAP6	GTPase, IMAP family member 6	7	-149953398	474344	NA	Hs.438823
LYN	v-yes-1 Yamaguchi sarcoma viral related oncogene homolog	8	56954925	4067	8q13	Hs.491767 Hs.614775
MAFB	v-maf musculoaponeurotic fibrosarcoma oncogene homolog B (avian)	20	-38747932	9935	20q11.2-q13.1	Hs.642679
VNN1	vanin 1	6	-133044424	8876	6q23-q24	Hs.12114
CLEC7A	C-type lectin domain family 7, member A	12	-20331837	64581	12p13.2	Hs.143929
LILRA2	leukocyte immunoglobulin-like receptor, subfamily A (with TM domain), member 2	19	59777070	11027	19q13.4	Hs.534394
C1orf38	chromosome 1 open reading frame 38	1	28071641	9473	1p35.3	Hs.10649
TBXAS1	thromboxane A synthase 1 (platelet, cytochrome P450, family 5, subfamily A)	7	139175420	6916	7q34-q35	Hs.520757
LR8	NA	7	-150119323	28959	7q36.1	Hs.438823
LMO2	LIM domain only 2 (rhombotin-like 1)	11	-33836698	4005	11p13	Hs.34560
FLJ20273	NA	4	-40120497	54502	4p13-p12	Hs.518727
CD37	CD37 molecule	19	54530488	951	19q13.3	Hs.166556
RGS2	regulator of G-protein signalling 2, 24kDa	1	191044793	5997	1q31	Hs.78944
VAMP8	vesicle-associated membrane protein 8 (endobrevin)	2	85658227	8673	2p12-p11.2	Hs.534373
LOC646082	NA	22	NA	646082	22q11.23	NA
TNFSF10	tumor necrosis factor (ligand) superfamily, member 10	3	-173706158	8743	3q26	Hs.478275
USP3	ubiquitin specific peptidase 3	15	61583862	9960	15q22.3	Hs.458499
IRF7	interferon regulatory factor 7	11	-602555	3665	11p15.5	Hs.166120
HHEX	homeobox, hematopoietically expressed	10	94439679	3087	10q23.33	Hs.118651
FCGR2A	Fc fragment of IgG, low affinity IIa, receptor (CD32)	1	159741843	2212	1q23	Hs.352642
FPR1	formyl peptide receptor 1	19	-56940838	2357	19q13.4	Hs.753
CACNA2D3	calcium channel, voltage-dependent, alpha 2/delta 3 subunit	3	54131732	55799	3p21.1	Hs.128594
RNASE2	ribonuclease, RNase A family, 2 (liver, eosinophil-derived neurotoxin)	14	20493469	6036	14q24-q31	Hs.728
NMI	N-myc (and STAT) interactor	2	-151835230	9111	2p24.3-q21.3	Hs.54483
TLR2	toll-like receptor 2	4	154824890	7097	4q32	Hs.519033
SAMSN1	SAM domain, SH3 domain and nuclear localisation signals, 1	21	-14779419	64092	21q11	Hs.473341 Hs.570423
ICAM3	intercellular adhesion molecule 3	19	-10305453	3385	19p13.3-p13.2	Hs.75516
PSCDBP	pleckstrin homology, Sec7 and coiled-coil domains, binding protein	2	-157979377	9595	2q11.2	Hs.270
RAB11FIP1	RAB11 family interacting protein 1 (class I)	8	-75672971	80223	8p11.22	Hs.191179
PTGS2	prostaglandin-endoperoxide synthase 2 (prostaglandin G/H synthase and	1	-184907592	5743	1q25.2-q25.3	Hs.196384

	cyclooxygenase)					
ANXA1	annexin A1	9	74956600	301	9q12-q21.2|9q12-q21.2	Hs.494173
PRG1	proteoglycan 1, secretory granule	10	70517833	5552	10q22.1	Hs.1908
LILRA3	leukocyte immunoglobulin-like receptor, subfamily A (without TM domain), member 3	19	-59491668	11026	19q13.4	Hs.113277
LGALS2	lectin, galactoside-binding, soluble, 2 (galectin 2)	22	-36296199	3957	22q12-q13|22q13.1	Hs.531776
CENTB2	centaurin, beta 2	3	-196476767	23527	3q29	Hs.478746
STX11	syntaxin 11	6	144513361	8676	6q24.2	Hs.118958
PF4	platelet factor 4 (chemokine (C-X-C motif) ligand 4)	4	-75065659	5196	4q12-q21	Hs.81564
IMPDH1	IMP (inosine monophosphate) dehydrogenase 1	7	-127819567	3614	7q31.3-q32	Hs.534808
DPCR1	diffuse panbronchiolitis critical region 1	6	31027537, 2367969, 2166477	135656	6p21.33	Hs.631993
HLA-DRA	major histocompatibility complex, class II, DR alpha	6	32515624	3122	6p21.3	Hs.520048
KLF4	Kruppel-like factor 4 (gut)	9	-109286955	9314	9q31	Hs.376206
CD48	CD48 molecule	1	-158915161	962	1q21.3-q22	Hs.243564
PSMB10	proteasome (prosome, macropain) subunit, beta type, 10	16	-66525907	5699	16q22.1	Hs.9661
P2RY5	purinergic receptor P2Y, G-protein coupled, 5	13	-47883184	10161	13q14	Hs.123464
FRAT2	frequently rearranged in advanced T-cell lymphomas 2	10	-99082244	23401	10q24.1	Hs.140720
LILRB2	leukocyte immunoglobulin-like receptor, subfamily B (with TM and ITIM domains), member 2	19	-59470131	10288	19q13.4	Hs.241797 Hs.631536
HMGB2	high-mobility group box 2	4	-174489362	3148	4q31	Hs.434953
LST1	leukocyte specific transcript 1	6	31661949, 3000093, 2801557, 31662603, 3000747, 2802211	7940	6p21.3	Hs.436066
MYO1F	myosin IF	19	-8491998	4542	19p13.3-p13.2	Hs.465818
PSTPIP1	proline-serine-threonine phosphatase interacting protein 1	15	75074608	9051	15q24-q25.1	Hs.129758
TLR1	toll-like receptor 1	4	-38474271	7096	4p14	Hs.575090 Hs.621817
TNFAIP8	tumor necrosis factor, alpha-induced protein 8	5	118719628	25816	5q23.1	Hs.271955
CAPG	capping protein (actin filament), gelsolin-like	2	-85475381	822	2p11.2	Hs.516155
KIAA0984	NA	12	NA	23329	12q14.3	Hs.192492
ALOX5AP	arachidonate 5-lipoxygenase-activating protein	13	30207668	241	13q12	Hs.507658
PTGER2	prostaglandin E receptor 2 (subtype EP2), 53kDa	14	51850862	5732	14q22	Hs.2090
PTPRE	protein tyrosine phosphatase, receptor type, E	10	129595314, 129735823	5791	10q26	Hs.127022
SLC7A7	solute carrier family 7 (cationic amino acid transporter, y+ system), member 7	14	-22312273	9056	14q11.2	Hs.513147
FLJ11151	NA	16	-12664437	55313	16p13.12	Hs.460002
LILRA5	leukocyte immunoglobulin-like receptor, subfamily A (with TM domain), member 5	19	-238049124	353514	19q13.4	Hs.631592
HCLS1	hematopoietic cell-specific Lyn substrate 1	3	-122832936	3059	3q13	Hs.14601
IL10RA	interleukin 10 receptor, alpha	11	117362318	3587	11q23	Hs.504035

PMAIP1	phorbol-12-myristate-13-acetate-induced protein 1	18	55718216	5366	18q21.32	Hs.96
CD52	CD52 molecule	1	26516997	1043	1p36	Hs.276770
BIN2	bridging integrator 2	12	-49961089	51411	12q13	Hs.14770
P2RY13	purinergic receptor P2Y, G-protein coupled, 13	3	-152526785	53829	3q24	Hs.546396
SCAP2	src family associated phosphoprotein 2	7	-26673214	8935	7p21-p15	Hs.200770
SYK	spleen tyrosine kinase	9	92603890	6850	9q22	Hs.371720
MS4A6A	membrane-spanning 4-domains, subfamily A, member 6A	11	-119392204	64231	11q12.1	Hs.523702
RNF138	ring finger protein 138	18	27925834, 27926644	51444	18q12.1	Hs.302408 Hs.501040
FBP1	fructose-1,6-bisphosphatase 1	9	-96405237	2203	9q22.3	Hs.494496
KCTD12	potassium channel tetramerisation domain containing 12	13	-76352312	115207	13q22.3	Hs.109438
SGK	serum/glucocorticoid regulated kinase	6	-134532081	6446	6q23	Hs.510078
HLA-DMA	major histocompatibility complex, class II, DM alpha	6	-41424089	3108	6p21.3	Hs.351279
RCBTB2	regulator of chromosome condensation (RCC1) and BTB (POZ) domain containing protein 2	13	-47961101	1102	13q14.3	Hs.25447
IER5	immediate early response 5	1	179324260	51278	1q25.3	Hs.15725
SERPINB1	serpin peptidase inhibitor, clade B (ovalbumin), member 1	6	-2778734	1992	6p25	Hs.381167
RBM3	RNA binding motif (RNP1, RRM) protein 3	X	48317779	5935	Xp11.2	Hs.301404
TCIRG1	T-cell, immune regulator 1, ATPase, H+ transporting, lysosomal V0 subunit A3	11	67563058, 67567022	10312	11q13.2	Hs.495985
DPEP2	dipeptidase 2	16	-66578800	64174	16q22.1	Hs.372633
ITGB2	integrin, beta 2 (complement component 3 receptor 3 and 4 subunit)	21	-45130298	3689	21q22.3	Hs.375957
TGFBI	transforming growth factor, beta-induced, 68kDa	5	135392596	7045	5q31	Hs.369397
DHRS8	dehydrogenase/reductase (SDR family) member 8	4	-88476788	51170	4q22.1	Hs.282984
CXCR4	chemokine (C-X-C motif) receptor 4	2	-136588389	7852	2q21	Hs.421986
SNX2	sorting nexin 2	5	122138648	6643	5q23	Hs.134822
STK10	serine/threonine kinase 10	5	-171403229	6793	5q35.1	Hs.519756
S100A4	S100 calcium binding protein A4 (calcium protein, calvasculin, metastasin, murine placental homolog)	1	-151782721	6275	1q21	Hs.557609
ITGA4	integrin, alpha 4 (antigen CD49D, alpha 4 subunit of VLA-4 receptor)	2	182029863	3676	2q31.3	Hs.440955
CKLF	chemokine-like factor	16	65143966, 65143972	51192	16q21	Hs.15159
LBR	lamin B receptor	1	-223655827	3930	1q42.1	Hs.435166
ZFP36L2	zinc finger protein 36, C3H type-like 2	2	-43304210	678	2p22.3-p21	Hs.503093 Hs.636758
MAN2B1	mannosidase, alpha, class 2B, member 1	19	-12618321	4125	19cen-q13.1	Hs.334626 Hs.356769
IMPA2	inositol(myo)-1(or 4)-monophosphatase 2	18	11971454	3613	18p11.2	Hs.367992
RABGAP1L	RAB GTPase activating protein 1-like	1	173111306, 172395256	9910	1q24	Hs.585378 Hs.591475 Hs.615081
KLF10	Kruppel-like factor 10	8	-207460384	7071	8q22.2	Hs.435001

17.2 GO: Biological Process

GO ID	genes present in chip	genes present in tissues	p-value	GO term
GO:0006952	751	55	3.22E-26	defense response
GO:0009607	857	58	4.79E-26	response to biotic stimulus
GO:0006955	684	51	1.89E-24	immune response
GO:0043207	463	40	3.63E-21	response to external biotic stimulus
GO:0009613	458	39	2.17E-20	response to pest, pathogen or parasite
GO:0050896	1578	67	4.98E-19	response to stimulus
GO:0009611	341	30	4.95E-16	response to wounding
GO:0006954	185	21	1.40E-13	inflammatory response
GO:0006950	881	43	1.64E-13	response to stress
GO:0009605	963	45	1.72E-13	response to external stimulus
GO:0050874	1531	57	4.42E-13	organismal physiological process
GO:0030595	13	5	6.71E-07	immune cell chemotaxis
GO:0050900	14	5	1.03E-06	immune cell migration
GO:0006935	101	10	1.71E-06	chemotaxis
GO:0042330	101	10	1.71E-06	taxis
GO:0009617	51	7	7.44E-06	response to bacteria
GO:0030593	5	3	2.85E-05	neutrophil chemotaxis
GO:0006968	87	8	3.34E-05	cellular defense response
GO:0042221	183	11	6.27E-05	response to chemical substance
GO:0042742	34	5	0.000113829	defense response to bacteria
GO:0006928	205	11	0.000172824	cell motility
GO:0040011	205	11	0.000172824	locomotion
GO:0007626	209	11	0.000204694	locomotory behavior
GO:0048247	2	2	0.000205595	lymphocyte chemotaxis
GO:0046456	23	4	0.000293469	icosanoid biosynthesis
GO:0042531	3	2	0.000610957	positive regulation of tyrosine phosphorylation of STAT protein
GO:0046427	3	2	0.000610957	positive regulation of JAK-STAT cascade
GO:0007165	2168	48	0.000619075	signal transduction
GO:0030218	13	3	0.000749849	erythrocyte differentiation
GO:0019735	77	6	0.000814076	antimicrobial humoral response (sensu Vertebrata)
GO:0050776	77	6	0.000814076	regulation of immune response
GO:0019730	78	6	0.00087166	antimicrobial humoral response
GO:0007166	952	26	0.000929798	cell surface receptor linked signal transduction
GO:0006959	141	8	0.000947394	humoral immune response
GO:0050730	15	3	0.00116807	regulation of peptidyl-tyrosine phosphorylation
GO:0007154	2730	56	0.001207052	cell communication
GO:0006690	33	4	0.001213391	icosanoid metabolism
GO:0001932	16	3	0.001422574	regulation of protein amino acid phosphorylation
GO:0009595	16	3	0.001422574	detection of biotic stimulus
GO:0030097	87	6	0.00154266	hemopoiesis
GO:0048534	88	6	0.001636326	hemopoietic or lymphoid organ development
GO:0007610	269	11	0.001684601	behavior
GO:0006521	17	3	0.001709338	regulation of amino acid metabolism

GO:0030099	37	4	0.001873433	myeloid cell differentiation
GO:0030216	18	3	0.00202976	keratinocyte differentiation
GO:0018108	19	3	0.002385155	peptidyl-tyrosine phosphorylation
GO:0018212	21	3	0.003205754	peptidyl-tyrosine modification
GO:0016064	103	6	0.003625015	humoral defense mechanism (sensu Vertebrata)
GO:0007229	45	4	0.003868446	integrin-mediated signaling pathway
GO:0016045	7	2	0.004117757	detection of bacteria
GO:0042509	7	2	0.004117757	regulation of tyrosine phosphorylation of STAT protein
GO:0046425	7	2	0.004117757	regulation of JAK-STAT cascade
GO:0043085	107	6	0.004373627	positive regulation of enzyme activity
GO:0006633	47	4	0.004529885	fatty acid biosynthesis
GO:0008219	498	15	0.005169643	cell death
GO:0042325	25	3	0.00531636	regulation of phosphorylation
GO:0009596	8	2	0.005438749	detection of pest, pathogen or parasite
GO:0016265	501	15	0.005460494	death
GO:0009628	318	11	0.006053753	response to abiotic stimulus
GO:0045638	9	2	0.006927039	negative regulation of myeloid cell differentiation
GO:0050731	9	2	0.006927039	positive regulation of peptidyl-tyrosine phosphorylation
GO:0007159	9	2	0.006927039	leukocyte adhesion
GO:0045860	53	4	0.006961064	positive regulation of protein kinase activity
GO:0045087	53	4	0.006961064	innate immune response
GO:0019220	28	3	0.007338693	regulation of phosphate metabolism
GO:0007259	28	3	0.007338693	JAK-STAT cascade
GO:0051174	28	3	0.007338693	regulation of phosphorus metabolism
GO:0016053	54	4	0.007435702	organic acid biosynthesis
GO:0051347	54	4	0.007435702	positive regulation of transferase activity
GO:0046394	54	4	0.007435702	carboxylic acid biosynthesis
GO:0016477	85	5	0.007487707	cell migration
GO:0009967	85	5	0.007487707	positive regulation of signal transduction
GO:0006915	471	14	0.007684553	apoptosis
GO:0012501	473	14	0.007964386	programmed cell death
GO:0007243	242	9	0.008118119	protein kinase cascade
GO:0050778	56	4	0.008447943	positive regulation of immune response
GO:0001516	10	2	0.008577613	prostaglandin biosynthesis
GO:0001934	10	2	0.008577613	positive regulation of protein amino acid phosphorylation
GO:0045764	10	2	0.008577613	positive regulation of amino acid metabolism
GO:0007260	10	2	0.008577613	tyrosine phosphorylation of STAT protein
GO:0046457	10	2	0.008577613	prostanoid biosynthesis
GO:0051240	59	4	0.010129027	positive regulation of organismal physiological process
GO:0019884	11	2	0.010385566	antigen presentation, exogenous antigen
GO:0050790	214	8	0.011990718	regulation of enzyme activity
GO:0051239	133	6	0.012277657	regulation of organismal physiological process
GO:0019370	12	2	0.012346094	leukotriene biosynthesis
GO:0019886	12	2	0.012346094	antigen processing, exogenous antigen via MHC class II
GO:0042327	12	2	0.012346094	positive regulation of phosphorylation
GO:0045937	12	2	0.012346094	positive regulation of phosphate metabolism
GO:0019752	359	11	0.014285534	carboxylic acid metabolism

GO:0048003	1	1	0.014393268	antigen presentation, lipid antigen
GO:0048006	1	1	0.014393268	antigen presentation, endogenous lipid antigen
GO:0031342	1	1	0.014393268	negative regulation of cell killing
GO:0019371	1	1	0.014393268	cyclooxygenase pathway
GO:0001911	1	1	0.014393268	negative regulation of immune cell mediated cytotoxicity
GO:0001913	1	1	0.014393268	T cell mediated cytotoxicity
GO:0001914	1	1	0.014393268	regulation of T cell mediated cytotoxicity
GO:0001915	1	1	0.014393268	negative regulation of T cell mediated cytotoxicity
GO:0019860	1	1	0.014393268	uracil metabolism
GO:0045653	1	1	0.014393268	negative regulation of megakaryocyte differentiation
GO:0045647	1	1	0.014393268	negative regulation of erythrocyte differentiation
GO:0050853	1	1	0.014393268	B cell receptor signaling pathway
GO:0042494	1	1	0.014393268	detection of bacterial lipoprotein
GO:0042495	1	1	0.014393268	detection of triacylated bacterial lipoprotein
GO:0007423	1	1	0.014393268	sensory organ development
GO:0051103	1	1	0.014393268	DNA ligation during DNA repair
GO:0044416	1	1	0.014393268	induction of host defense response
GO:0046121	1	1	0.014393268	deoxyribonucleoside catabolism
GO:0046127	1	1	0.014393268	pyrimidine deoxyribonucleoside catabolism
GO:0046113	1	1	0.014393268	nucleobase catabolism
GO:0046104	1	1	0.014393268	thymidine metabolism
GO:0006288	1	1	0.014393268	base-excision repair, DNA ligation
GO:0006212	1	1	0.014393268	uracil catabolism
GO:0006214	1	1	0.014393268	thymidine catabolism
GO:0006208	1	1	0.014393268	pyrimidine base catabolism
GO:0046730	1	1	0.014393268	viral induction of host immune response
GO:0046731	1	1	0.014393268	passive viral induction of host immune response
GO:0006082	361	11	0.014836441	organic acid metabolism
GO:0006691	14	2	0.016706195	leukotriene metabolism
GO:0009725	14	2	0.016706195	response to hormone stimulus
GO:0007155	518	14	0.016708734	cell adhesion
GO:0008283	473	13	0.018420554	cell proliferation
GO:0006909	15	2	0.019096676	phagocytosis
GO:0018149	15	2	0.019096676	peptide cross-linking
GO:0048519	688	17	0.019502059	negative regulation of biological process
GO:0019882	41	3	0.020862858	antigen presentation
GO:0051247	41	3	0.020862858	positive regulation of protein metabolism
GO:0045637	16	2	0.021621547	regulation of myeloid cell differentiation
GO:0007157	16	2	0.021621547	heterophilic cell adhesion
GO:0006692	17	2	0.024276509	prostanoid metabolism
GO:0006693	17	2	0.024276509	prostaglandin metabolism
GO:0045596	17	2	0.024276509	negative regulation of cell differentiation
GO:0045859	116	5	0.02580126	regulation of protein kinase activity
GO:0051338	117	5	0.026652235	regulation of transferase activity
GO:0006517	2	1	0.028580941	protein deglycosylation
GO:0031341	2	1	0.028580941	regulation of cell killing
GO:0001910	2	1	0.028580941	regulation of immune cell mediated cytotoxicity

GO:0008228	2	1	0.028580941	opsonization
GO:0019733	2	1	0.028580941	antibacterial humoral response (sensu Vertebrata)
GO:0045652	2	1	0.028580941	regulation of megakaryocyte differentiation
GO:0009120	2	1	0.028580941	deoxyribonucleoside metabolism
GO:0046037	2	1	0.028580941	GMP metabolism
GO:0009598	2	1	0.028580941	detection of pathogenic bacteria
GO:0046125	2	1	0.028580941	pyrimidine deoxyribonucleoside metabolism
GO:0006177	2	1	0.028580941	GMP biosynthesis
GO:0046135	2	1	0.028580941	pyrimidine nucleoside catabolism
GO:0007599	83	4	0.031476305	hemostasis
GO:0006631	124	5	0.033112099	fatty acid metabolism
GO:0043405	49	3	0.033202639	regulation of MAPK activity
GO:0018193	50	3	0.034960771	peptidyl-amino acid modification
GO:0019221	21	2	0.036114484	cytokine and chemokine mediated signaling pathway
GO:0050670	22	2	0.03935861	regulation of lymphocyte proliferation
GO:0048523	640	15	0.041309717	negative regulation of cellular process
GO:0048002	3	1	0.042565933	antigen presentation, peptide antigen
GO:0048004	3	1	0.042565933	antigen presentation, endogenous peptide antigen
GO:0048246	3	1	0.042565933	macrophage chemotaxis
GO:0019731	3	1	0.042565933	antibacterial humoral response
GO:0045646	3	1	0.042565933	regulation of erythrocyte differentiation
GO:0050857	3	1	0.042565933	positive regulation of antigen receptor mediated signaling pathway
GO:0009164	3	1	0.042565933	nucleoside catabolism
GO:0042533	3	1	0.042565933	tumor necrosis factor-alpha biosynthesis
GO:0042534	3	1	0.042565933	regulation of tumor necrosis factor-alpha biosynthesis
GO:0042535	3	1	0.042565933	positive regulation of tumor necrosis factor-alpha biosynthesis
GO:0007500	3	1	0.042565933	mesodermal cell fate determination
GO:0030219	3	1	0.042565933	megakaryocyte differentiation
GO:0006266	3	1	0.042565933	DNA ligation
GO:0016998	3	1	0.042565933	cell wall catabolism
GO:0050878	94	4	0.046376402	regulation of body fluids
GO:0008360	25	2	0.049713562	regulation of cell shape
GO:0006944	25	2	0.049713562	membrane fusion
GO:0045321	97	4	0.051016915	immune cell activation
GO:0045892	97	4	0.051016915	negative regulation of transcription, DNA-dependent
GO:0001775	98	4	0.052618484	cell activation
GO:0007498	26	2	0.053360751	mesoderm development
GO:0044419	26	2	0.053360751	interaction between organisms
GO:0006470	99	4	0.054247327	protein amino acid dephosphorylation
GO:0019048	4	1	0.056351118	virus-host interaction
GO:0048333	4	1	0.056351118	mesodermal cell differentiation
GO:0045410	4	1	0.056351118	positive regulation of interleukin-6 biosynthesis
GO:0045408	4	1	0.056351118	regulation of interleukin-6 biosynthesis
GO:0050854	4	1	0.056351118	regulation of antigen receptor mediated signaling pathway
GO:0007171	4	1	0.056351118	transmembrane receptor protein tyrosine kinase activation (dimerization)
GO:0050672	4	1	0.056351118	negative regulation of lymphocyte proliferation
GO:0042226	4	1	0.056351118	interleukin-6 biosynthesis

GO:0051282	4	1	0.056351118	regulation of sequestering of calcium ion
GO:0051283	4	1	0.056351118	negative regulation of sequestering of calcium ion
GO:0051209	4	1	0.056351118	release of sequestered calcium ion into cytoplasm
GO:0042541	4	1	0.056351118	hemoglobin biosynthesis
GO:0006213	4	1	0.056351118	pyrimidine nucleoside metabolism
GO:0006207	4	1	0.056351118	'de novo' pyrimidine base biosynthesis
GO:0001710	4	1	0.056351118	mesodermal cell fate commitment
GO:0045089	4	1	0.056351118	positive regulation of innate immune response
GO:0000187	27	2	0.057099918	activation of MAPK activity
GO:0000122	61	3	0.057362205	negative regulation of transcription from RNA polymerase II promoter
GO:0006807	291	8	0.058663471	nitrogen compound metabolism
GO:0046651	28	2	0.060927704	lymphocyte proliferation
GO:0051348	28	2	0.060927704	negative regulation of transferase activity
GO:0006469	28	2	0.060927704	negative regulation of protein kinase activity
GO:0006461	194	6	0.060985596	protein complex assembly
GO:0048518	565	13	0.062614038	positive regulation of biological process
GO:0001819	29	2	0.06484083	positive regulation of cytokine production
GO:0016481	151	5	0.066618931	negative regulation of transcription
GO:0019369	5	1	0.069939327	arachidonic acid metabolism
GO:0019856	5	1	0.069939327	pyrimidine base biosynthesis
GO:0050727	5	1	0.069939327	regulation of inflammatory response
GO:0050716	5	1	0.069939327	positive regulation of interleukin-1 secretion
GO:0050718	5	1	0.069939327	positive regulation of interleukin-1 beta secretion
GO:0050701	5	1	0.069939327	interleukin-1 secretion
GO:0050702	5	1	0.069939327	interleukin-1 beta secretion
GO:0050704	5	1	0.069939327	regulation of interleukin-1 secretion
GO:0050706	5	1	0.069939327	regulation of interleukin-1 beta secretion
GO:0050852	5	1	0.069939327	T cell receptor signaling pathway
GO:0016358	5	1	0.069939327	dendrite morphogenesis
GO:0007263	5	1	0.069939327	nitric oxide mediated signal transduction
GO:0051208	5	1	0.069939327	sequestering of calcium ion
GO:0046146	5	1	0.069939327	tetrahydrobiopterin metabolism
GO:0020027	5	1	0.069939327	hemoglobin metabolism
GO:0006729	5	1	0.069939327	tetrahydrobiopterin biosynthesis
GO:0045088	5	1	0.069939327	regulation of innate immune response
GO:0030154	409	10	0.070237013	cell differentiation
GO:0043406	31	2	0.072910346	positive regulation of MAPK activity
GO:0016311	110	4	0.073940374	dephosphorylation
GO:0042981	306	8	0.073971803	regulation of apoptosis
GO:0043067	308	8	0.076180953	regulation of programmed cell death
GO:0006793	641	14	0.076653378	phosphorus metabolism
GO:0006796	641	14	0.076653378	phosphate metabolism
GO:0007242	879	18	0.07905256	intracellular signaling cascade
GO:0045934	159	5	0.079206782	negative regulation of nucleobase, nucleoside, nucleotide and nucleic acid metabolism
GO:0030155	33	2	0.081283688	regulation of cell adhesion
GO:0006401	33	2	0.081283688	RNA catabolism
GO:0001909	6	1	0.083333355	immune cell mediated cytotoxicity

GO:0050851	6	1	0.083333355	antigen receptor-mediated signaling pathway
GO:0042116	6	1	0.083333355	macrophage activation
GO:0045058	6	1	0.083333355	T cell selection
GO:0006897	115	4	0.083941404	endocytosis
GO:0030098	34	2	0.08557686	lymphocyte differentiation
GO:0042127	265	7	0.08765229	regulation of cell proliferation
GO:0006725	73	3	0.087771304	aromatic compound metabolism
GO:0006519	216	6	0.091327067	amino acid and derivative metabolism
GO:0009892	217	6	0.092872203	negative regulation of metabolism
GO:0030333	36	2	0.094361937	antigen processing
GO:0048332	7	1	0.096535954	mesoderm morphogenesis
GO:0050715	7	1	0.096535954	positive regulation of cytokine secretion
GO:0050707	7	1	0.096535954	regulation of cytokine secretion
GO:0050663	7	1	0.096535954	cytokine secretion
GO:0001704	7	1	0.096535954	formation of primary germ layer
GO:0001707	7	1	0.096535954	mesoderm formation
GO:0008284	121	4	0.096771286	positive regulation of cell proliferation
GO:0007204	37	2	0.098848334	elevation of cytoplasmic calcium ion concentration
GO:0051480	37	2	0.098848334	cytoplasmic calcium ion homeostasis
GO:0007596	78	3	0.102085472	blood coagulation
GO:0050817	79	3	0.105054179	coagulation
GO:0051246	174	5	0.105987739	regulation of protein metabolism
GO:0008610	175	5	0.107915547	lipid biosynthesis
GO:0001817	39	2	0.107995552	regulation of cytokine production
GO:0006558	8	1	0.109549841	L-phenylalanine metabolism
GO:0006559	8	1	0.109549841	L-phenylalanine catabolism
GO:0001906	8	1	0.109549841	cell killing
GO:0007369	8	1	0.109549841	gastrulation
GO:0050866	8	1	0.109549841	negative regulation of cell activation
GO:0050832	8	1	0.109549841	defense response to fungi
GO:0006013	8	1	0.109549841	mannose metabolism
GO:0051250	8	1	0.109549841	negative regulation of lymphocyte activation
GO:0051238	8	1	0.109549841	sequestering of metal ion
GO:0046112	8	1	0.109549841	nucleobase biosynthesis
GO:0006206	8	1	0.109549841	pyrimidine base metabolism
GO:0046649	81	3	0.1110927	lymphocyte activation
GO:0000165	81	3	0.1110927	MAPKKK cascade
GO:0009966	229	6	0.112507493	regulation of signal transduction
GO:0051249	41	2	0.117358341	regulation of lymphocyte activation
GO:0006520	181	5	0.119840744	amino acid metabolism
GO:0001816	42	2	0.122114427	cytokine production
GO:0050714	9	1	0.122377691	positive regulation of protein secretion
GO:0042100	9	1	0.122377691	B cell proliferation
GO:0006268	9	1	0.122377691	DNA unwinding
GO:0042060	85	3	0.123557162	wound healing
GO:0031324	183	5	0.123949526	negative regulation of cellular metabolism
GO:0050865	43	2	0.126917147	regulation of cell activation

GO:0044248	700	14	0.130589797	cellular catabolism
GO:0006357	240	6	0.132222459	regulation of transcription from RNA polymerase II promoter
GO:0006516	10	1	0.135022144	glycoprotein catabolism
GO:0019438	10	1	0.135022144	aromatic compound biosynthesis
GO:0009074	10	1	0.135022144	aromatic amino acid family catabolism
GO:0042559	10	1	0.135022144	pteridine and derivative biosynthesis
GO:0043086	46	2	0.141582382	negative regulation of enzyme activity
GO:0006917	140	4	0.142869717	induction of apoptosis
GO:0012502	140	4	0.142869717	induction of programmed cell death
GO:0006957	11	1	0.147485802	complement activation, alternative pathway
GO:0009167	11	1	0.147485802	purine ribonucleoside monophosphate metabolism
GO:0009168	11	1	0.147485802	purine ribonucleoside monophosphate biosynthesis
GO:0009126	11	1	0.147485802	purine nucleoside monophosphate metabolism
GO:0009127	11	1	0.147485802	purine nucleoside monophosphate biosynthesis
GO:0007250	11	1	0.147485802	activation of NF-kappaB-inducing kinase
GO:0042558	11	1	0.147485802	pteridine and derivative metabolism
GO:0009620	11	1	0.147485802	response to fungi
GO:0016525	11	1	0.147485802	negative regulation of angiogenesis
GO:0006171	11	1	0.147485802	cAMP biosynthesis
GO:0051093	49	2	0.156587798	negative regulation of development
GO:0050708	12	1	0.15977123	regulation of protein secretion
GO:0007422	12	1	0.15977123	peripheral nervous system development
GO:0009112	12	1	0.15977123	nucleobase metabolism
GO:0042102	12	1	0.15977123	positive regulation of T cell proliferation
GO:0046039	12	1	0.15977123	GTP metabolism
GO:0006183	12	1	0.15977123	GTP biosynthesis
GO:0001709	12	1	0.15977123	cell fate determination
GO:0043119	428	9	0.163345538	positive regulation of physiological process
GO:0007186	491	10	0.169213158	G-protein coupled receptor protein signaling pathway
GO:0007249	99	3	0.1707317	I-kappaB kinase/NF-kappaB cascade
GO:0008037	13	1	0.171880957	cell recognition
GO:0019439	13	1	0.171880957	aromatic compound catabolism
GO:0051047	13	1	0.171880957	positive regulation of secretion
GO:0046058	13	1	0.171880957	cAMP metabolism
GO:0006000	13	1	0.171880957	fructose metabolism
GO:0030168	13	1	0.171880957	platelet activation
GO:0006584	14	1	0.183817477	catecholamine metabolism
GO:0019835	14	1	0.183817477	cytolysis
GO:0043065	155	4	0.184289094	positive regulation of apoptosis
GO:0044265	563	11	0.186728004	cellular macromolecule catabolism
GO:0043068	156	4	0.187182352	positive regulation of programmed cell death
GO:0045595	55	2	0.187403524	regulation of cell differentiation
GO:0006468	444	9	0.189424801	protein amino acid phosphorylation
GO:0009308	269	6	0.19128027	amine metabolism
GO:0006801	15	1	0.195583249	superoxide metabolism
GO:0045165	15	1	0.195583249	cell fate commitment
GO:0043407	15	1	0.195583249	negative regulation of MAPK activity

GO:0018958	15	1	0.195583249	phenol metabolism
GO:0007162	15	1	0.195583249	negative regulation of cell adhesion
GO:0042129	15	1	0.195583249	regulation of T cell proliferation
GO:0000188	15	1	0.195583249	inactivation of MAPK activity
GO:0009056	756	14	0.197794197	catabolism
GO:0006508	451	9	0.201386685	proteolysis and peptidolysis
GO:0044257	451	9	0.201386685	cellular protein catabolism
GO:0009615	58	2	0.20311576	response to virus
GO:0009057	575	11	0.204930358	macromolecule catabolism
GO:0006809	16	1	0.207180696	nitric oxide biosynthesis
GO:0050777	16	1	0.207180696	negative regulation of immune response
GO:0007202	16	1	0.207180696	phospholipase C activation
GO:0009618	16	1	0.207180696	response to pathogenic bacteria
GO:0006284	16	1	0.207180696	base-excision repair
GO:0046209	16	1	0.207180696	nitric oxide metabolism
GO:0007188	59	2	0.208387133	G-protein signaling, coupled to cAMP nucleotide second messenger
GO:0051336	60	2	0.2136729	regulation of hydrolase activity
GO:0019933	60	2	0.2136729	cAMP-mediated signaling
GO:0006464	1087	19	0.215122195	protein modification
GO:0051243	583	11	0.21748653	negative regulation of cellular physiological process
GO:0006916	112	3	0.218362206	anti-apoptosis
GO:0015758	17	1	0.218612208	glucose transport
GO:0006904	17	1	0.218612208	vesicle docking during exocytosis
GO:0042098	17	1	0.218612208	T cell proliferation
GO:0009161	17	1	0.218612208	ribonucleoside monophosphate metabolism
GO:0009156	17	1	0.218612208	ribonucleoside monophosphate biosynthesis
GO:0006094	17	1	0.218612208	gluconeogenesis
GO:0030163	463	9	0.222612806	protein catabolism
GO:0015749	18	1	0.22988014	monosaccharide transport
GO:0031281	18	1	0.22988014	positive regulation of cyclase activity
GO:0048278	18	1	0.22988014	vesicle docking
GO:0045762	18	1	0.22988014	positive regulation of adenylate cyclase activity
GO:0009072	18	1	0.22988014	aromatic amino acid family metabolism
GO:0008645	18	1	0.22988014	hexose transport
GO:0009123	18	1	0.22988014	nucleoside monophosphate metabolism
GO:0009124	18	1	0.22988014	nucleoside monophosphate biosynthesis
GO:0007190	18	1	0.22988014	adenylate cyclase activation
GO:0042133	18	1	0.22988014	neurotransmitter metabolism
GO:0050671	18	1	0.22988014	positive regulation of lymphocyte proliferation
GO:0051349	18	1	0.22988014	positive regulation of lyase activity
GO:0006090	18	1	0.22988014	pyruvate metabolism
GO:0030217	18	1	0.22988014	T cell differentiation
GO:0006629	469	9	0.233544107	lipid metabolism
GO:0051242	411	8	0.239866878	positive regulation of cellular physiological process
GO:0043285	474	9	0.242804346	biopolymer catabolism
GO:0019932	176	4	0.247775287	second-messenger-mediated signaling
GO:0043118	602	11	0.248544158	negative regulation of physiological process

GO:0009190	20	1	0.251934527	cyclic nucleotide biosynthesis
GO:0051241	20	1	0.251934527	negative regulation of organismal physiological process
GO:0044403	20	1	0.251934527	symbiosis, mutualism through parasitism
GO:0044404	20	1	0.251934527	symbiotic interaction between host and other organism
GO:0043123	68	2	0.256306217	positive regulation of I-kappaB kinase/NF-kappaB cascade
GO:0006874	68	2	0.256306217	calcium ion homeostasis
GO:0048522	482	9	0.25788758	positive regulation of cellular process
GO:0019883	21	1	0.262725529	antigen presentation, endogenous antigen
GO:0051016	21	1	0.262725529	barbed-end actin filament capping
GO:0050870	21	1	0.262725529	positive regulation of T cell activation
GO:0048015	71	2	0.272366708	phosphoinositide-mediated signaling
GO:0019319	22	1	0.273362049	hexose biosynthesis
GO:0007189	22	1	0.273362049	G-protein signaling, adenylate cyclase activating pathway
GO:0030042	22	1	0.273362049	actin filament depolymerization
GO:0046364	22	1	0.273362049	monosaccharide biosynthesis
GO:0046165	22	1	0.273362049	alcohol biosynthesis
GO:0030834	22	1	0.273362049	regulation of actin filament depolymerization
GO:0030835	22	1	0.273362049	negative regulation of actin filament depolymerization
GO:0043066	128	3	0.280089147	negative regulation of apoptosis
GO:0043122	73	2	0.283070169	regulation of I-kappaB kinase/NF-kappaB cascade
GO:0006805	23	1	0.283846282	xenobiotic metabolism
GO:0006953	23	1	0.283846282	acute-phase response
GO:0008277	23	1	0.283846282	regulation of G-protein coupled receptor protein signaling pathway
GO:0009116	23	1	0.283846282	nucleoside metabolism
GO:0042108	23	1	0.283846282	positive regulation of cytokine biosynthesis
GO:0043069	129	3	0.284014731	negative regulation of programmed cell death
GO:0051046	24	1	0.29418039	regulation of secretion
GO:0009581	313	6	0.295744137	detection of external stimulus
GO:0045765	25	1	0.304366508	regulation of angiogenesis
GO:0045727	25	1	0.304366508	positive regulation of protein biosynthesis
GO:0042828	25	1	0.304366508	response to pathogen
GO:0051235	25	1	0.304366508	maintenance of localization
GO:0043412	1160	19	0.308314127	biopolymer modification
GO:0006118	256	5	0.308437489	electron transport
GO:0031325	136	3	0.311605854	positive regulation of cellular metabolism
GO:0044255	383	7	0.313622227	cellular lipid metabolism
GO:0031328	26	1	0.314406738	positive regulation of cellular biosynthesis
GO:0008643	26	1	0.314406738	carbohydrate transport
GO:0009306	26	1	0.314406738	protein secretion
GO:0009410	26	1	0.314406738	response to xenobiotic stimulus
GO:0008285	139	3	0.323465517	negative regulation of cell proliferation
GO:0006919	27	1	0.324303154	caspase activation
GO:0009187	27	1	0.324303154	cyclic nucleotide metabolism
GO:0009792	27	1	0.324303154	embryonic development (sensu Metazoa)
GO:0043087	28	1	0.334057801	regulation of GTPase activity
GO:0008217	28	1	0.334057801	regulation of blood pressure
GO:0051251	28	1	0.334057801	positive regulation of lymphocyte activation

GO:0006898	28	1	0.334057801	receptor mediated endocytosis
GO:0016310	522	9	0.337232517	phosphorylation
GO:0050867	29	1	0.343672692	positive regulation of cell activation
GO:0007179	29	1	0.343672692	transforming growth factor beta receptor signaling pathway
GO:0006956	30	1	0.353149817	complement activation
GO:0042113	30	1	0.353149817	B cell activation
GO:0009891	30	1	0.353149817	positive regulation of biosynthesis
GO:0051129	30	1	0.353149817	negative regulation of cell organization and biogenesis
GO:0051261	30	1	0.353149817	protein depolymerization
GO:0030005	87	2	0.35722278	di-, tri-valent inorganic cation homeostasis
GO:0043280	31	1	0.362491132	positive regulation of caspase activity
GO:0043281	31	1	0.362491132	regulation of caspase activity
GO:0050863	31	1	0.362491132	regulation of T cell activation
GO:0006367	31	1	0.362491132	transcription initiation from RNA polymerase II promoter
GO:0009893	149	3	0.362964312	positive regulation of metabolism
GO:0009719	214	4	0.371016169	response to endogenous stimulus
GO:0031279	32	1	0.371698571	regulation of cyclase activity
GO:0045761	32	1	0.371698571	regulation of adenylate cyclase activity
GO:0051345	32	1	0.371698571	positive regulation of hydrolase activity
GO:0051339	32	1	0.371698571	regulation of lyase activity
GO:0042035	33	1	0.380774036	regulation of cytokine biosynthesis
GO:0008064	33	1	0.380774036	regulation of actin polymerization and/or depolymerization
GO:0006875	93	2	0.38823299	metal ion homeostasis
GO:0007187	94	2	0.393340002	G-protein signaling, coupled to cyclic nucleotide second messenger
GO:0006576	35	1	0.398536528	biogenic amine metabolism
GO:0016032	35	1	0.398536528	viral life cycle
GO:0030832	35	1	0.398536528	regulation of actin filament length
GO:0042089	36	1	0.407227231	cytokine biosynthesis
GO:0019935	98	2	0.413572936	cyclic-nucleotide-mediated signaling
GO:0030003	98	2	0.413572936	cation homeostasis
GO:0006366	426	7	0.415397929	transcription from RNA polymerase II promoter
GO:0042107	37	1	0.415793311	cytokine metabolism
GO:0006487	37	1	0.415793311	protein amino acid N-linked glycosylation
GO:0044249	763	12	0.418323049	cellular biosynthesis
GO:0016192	296	5	0.423053341	vesicle-mediated transport
GO:0007178	38	1	0.424236543	transmembrane receptor protein serine/threonine kinase signaling pathway
GO:0007167	165	3	0.425304725	enzyme linked receptor protein signaling pathway
GO:0008015	103	2	0.438390112	circulation
GO:0009165	103	2	0.438390112	nucleotide biosynthesis
GO:0006873	103	2	0.438390112	cell ion homeostasis
GO:0006511	105	2	0.44815885	ubiquitin-dependent protein catabolism
GO:0019941	105	2	0.44815885	modification-dependent protein catabolism
GO:0006575	41	1	0.448846506	amino acid derivative metabolism
GO:0001505	41	1	0.448846506	regulation of neurotransmitter levels
GO:0016337	172	3	0.451961885	cell-cell adhesion
GO:0007582	7846	114	0.452959858	physiological process
GO:0009063	42	1	0.456815581	amino acid catabolism

GO:0006865	43	1	0.464670306	amino acid transport
GO:0009058	859	13	0.46782851	biosynthesis
GO:0046483	44	1	0.472412309	heterocycle metabolism
GO:0050801	111	2	0.476886555	ion homeostasis
GO:0009100	111	2	0.476886555	glycoprotein metabolism
GO:0008154	45	1	0.480043194	actin polymerization and/or depolymerization
GO:0009310	45	1	0.480043194	amine catabolism
GO:0042110	45	1	0.480043194	T cell activation
GO:0044267	2109	31	0.480757026	cellular protein metabolism
GO:0019318	113	2	0.486261729	hexose metabolism
GO:0031175	46	1	0.487564546	neurite morphogenesis
GO:0009142	46	1	0.487564546	nucleoside triphosphate biosynthesis
GO:0009145	46	1	0.487564546	purine nucleoside triphosphate biosynthesis
GO:0009201	46	1	0.487564546	ribonucleoside triphosphate biosynthesis
GO:0009206	46	1	0.487564546	purine ribonucleoside triphosphate biosynthesis
GO:0006352	47	1	0.494977923	transcription initiation
GO:0044270	47	1	0.494977923	nitrogen compound catabolism
GO:0005996	115	2	0.495533118	monosaccharide metabolism
GO:0007169	116	2	0.500129337	transmembrane receptor protein tyrosine kinase signaling pathway
GO:0006334	48	1	0.502284864	nucleosome assembly
GO:0043170	2405	35	0.503168942	macromolecule metabolism
GO:0019538	2129	31	0.504751822	protein metabolism
GO:0009199	49	1	0.509486887	ribonucleoside triphosphate metabolism
GO:0009144	49	1	0.509486887	purine nucleoside triphosphate metabolism
GO:0007160	49	1	0.509486887	cell-matrix adhesion
GO:0009205	49	1	0.509486887	purine ribonucleoside triphosphate metabolism
GO:0009141	50	1	0.516585485	nucleoside triphosphate metabolism
GO:0009968	50	1	0.516585485	negative regulation of signal transduction
GO:0019725	122	2	0.527141921	cell homeostasis
GO:0015837	52	1	0.530478282	amine transport
GO:0051128	52	1	0.530478282	regulation of cell organization and biogenesis
GO:0008632	53	1	0.537275367	apoptotic program
GO:0030036	126	2	0.54460094	actin cytoskeleton organization and biogenesis
GO:0006887	55	1	0.550577971	exocytosis
GO:0031497	56	1	0.557086255	chromatin assembly
GO:0007200	56	1	0.557086255	G-protein signaling, coupled to IP3 second messenger (phospholipase C activating)
GO:0007606	56	1	0.557086255	sensory perception of chemical stimulus
GO:0006817	57	1	0.563501004	phosphate transport
GO:0015992	57	1	0.563501004	proton transport
GO:0006818	58	1	0.569823552	hydrogen transport
GO:0009152	58	1	0.569823552	purine ribonucleotide biosynthesis
GO:0051248	58	1	0.569823552	negative regulation of protein metabolism
GO:0008202	132	2	0.569946749	steroid metabolism
GO:0015849	59	1	0.576055214	organic acid transport
GO:0046942	59	1	0.576055214	carboxylic acid transport
GO:0046903	208	3	0.579619273	secretion
GO:0006694	60	1	0.582197287	steroid biosynthesis

GO:0009150	60	1	0.582197287	purine ribonucleotide metabolism
GO:0050789	2689	38	0.58719091	regulation of biological process
GO:0006800	61	1	0.588251049	oxygen and reactive oxygen species metabolism
GO:0006164	61	1	0.588251049	purine nucleotide biosynthesis
GO:0006066	213	3	0.595844743	alcohol metabolism
GO:0030029	139	2	0.598221181	actin filament-based process
GO:0006163	63	1	0.600098662	purine nucleotide metabolism
GO:0001525	65	1	0.611607921	angiogenesis
GO:0009260	65	1	0.611607921	ribonucleotide biosynthesis
GO:0009790	65	1	0.611607921	embryonic development
GO:0006261	65	1	0.611607921	DNA-dependent DNA replication
GO:0009117	143	2	0.613747819	nucleotide metabolism
GO:0044260	2298	32	0.619744796	cellular macromolecule metabolism
GO:0006091	446	6	0.625977114	generation of precursor metabolites and energy
GO:0015672	223	3	0.627069995	monovalent inorganic cation transport
GO:0001568	68	1	0.628258299	blood vessel development
GO:0048514	68	1	0.628258299	blood vessel morphogenesis
GO:0009259	68	1	0.628258299	ribonucleotide metabolism
GO:0001944	69	1	0.633649466	vasculature development
GO:0016051	69	1	0.633649466	carbohydrate biosynthesis
GO:0050953	149	2	0.63617955	sensory perception of light
GO:0007601	149	2	0.63617955	visual perception
GO:0007275	1543	21	0.647865305	development
GO:0042592	157	2	0.664499775	homeostasis
GO:0007267	465	6	0.666273315	cell-cell signaling
GO:0009888	158	2	0.667913505	histogenesis
GO:0045045	161	2	0.677988184	secretory pathway
GO:0006006	79	1	0.683471328	glucose metabolism
GO:0006814	80	1	0.68806741	sodium ion transport
GO:0050793	168	2	0.700536542	regulation of development
GO:0016055	87	1	0.718437644	Wnt receptor signaling pathway
GO:0048513	872	11	0.721835573	organ development
GO:0006092	88	1	0.722529663	main pathways of carbohydrate metabolism
GO:0006333	91	1	0.734454969	chromatin assembly or disassembly
GO:0006486	92	1	0.73831593	protein amino acid glycosylation
GO:0043413	93	1	0.742121179	biopolymer glycosylation
GO:0000902	183	2	0.744485406	cellular morphogenesis
GO:0009101	96	1	0.753210577	glycoprotein biosynthesis
GO:0043283	2240	29	0.775824878	biopolymer metabolism
GO:0015698	106	1	0.786867616	inorganic anion transport
GO:0007600	287	3	0.78724914	sensory perception
GO:0006974	200	2	0.787555339	response to DNA damage stimulus
GO:0006417	107	1	0.789971717	regulation of protein biosynthesis
GO:0050794	2488	32	0.801889759	regulation of cellular process
GO:0006813	111	1	0.801945853	potassium ion transport
GO:0031326	111	1	0.801945853	regulation of cellular biosynthesis
GO:0000004	465	5	0.806440485	biological process unknown

GO:0001501	113	1	0.807676346	skeletal development
GO:0009889	116	1	0.815964993	regulation of biosynthesis
GO:0006820	128	1	0.845721111	anion transport
GO:0050791	2407	30	0.848312721	regulation of physiological process
GO:0015980	132	1	0.854537154	energy derivation by oxidation of organic compounds
GO:0006260	140	1	0.870697027	DNA replication
GO:0006811	515	5	0.870775265	ion transport
GO:0045786	145	1	0.879878641	negative regulation of progression through cell cycle
GO:0006812	346	3	0.880371389	cation transport
GO:0051244	2319	28	0.884234698	regulation of cellular physiological process
GO:0006886	257	2	0.889004826	intracellular protein transport
GO:0005975	355	3	0.890873579	carbohydrate metabolism
GO:0044262	265	2	0.898991801	cellular carbohydrate metabolism
GO:0045184	459	4	0.902929332	establishment of protein localization
GO:0030001	269	2	0.903667916	metal ion transport
GO:0009059	464	4	0.907421135	macromolecule biosynthesis
GO:0009987	8200	114	0.912046918	cellular process
GO:0008104	474	4	0.915860993	protein localization
GO:0051179	1857	21	0.916590602	localization
GO:0050877	475	4	0.916666474	neurophysiological process
GO:0006281	174	1	0.92170573	DNA repair
GO:0016070	391	3	0.925145245	RNA metabolism
GO:0000074	394	3	0.927506729	regulation of progression through cell cycle
GO:0006325	186	1	0.934441278	establishment and/or maintenance of chromatin architecture
GO:0006412	407	3	0.936976496	protein biosynthesis
GO:0007010	305	2	0.937570689	cytoskeleton organization and biogenesis
GO:0007268	192	1	0.940015201	synaptic transmission
GO:0006323	193	1	0.94089733	DNA packaging
GO:0051234	1841	20	0.941621205	establishment of localization
GO:0019226	198	1	0.945118554	transmission of nerve impulse
GO:0009887	807	7	0.952258184	organogenesis
GO:0006810	1802	19	0.954280575	transport
GO:0015031	439	3	0.955662115	protein transport
GO:0044237	5026	63	0.959550385	cellular metabolism
GO:0007001	221	1	0.960992001	chromosome organization and biogenesis (sensu Eukaryota)
GO:0008152	5262	66	0.966041603	metabolism
GO:0051276	236	1	0.968793547	chromosome organization and biogenesis
GO:0019222	1629	16	0.970894637	regulation of metabolism
GO:0044238	4820	59	0.972820714	primary metabolism
GO:0031323	1566	15	0.974606814	regulation of cellular metabolism
GO:0006512	255	1	0.976489959	ubiquitin cycle
GO:0007049	601	4	0.977068098	cell cycle
GO:0007399	385	2	0.977102246	neurogenesis
GO:0050875	7018	91	0.984915241	cellular physiological process
GO:0006351	1407	12	0.987975725	transcription, DNA-dependent
GO:0009653	1051	8	0.988087616	morphogenesis
GO:0006350	1499	13	0.988551686	transcription

GO:0046907	448	2	0.989919545	intracellular transport
GO:0045449	1433	12	0.990385171	regulation of transcription
GO:0006355	1359	11	0.991497827	regulation of transcription, DNA-dependent
GO:0006396	326	1	0.991885358	RNA processing
GO:0019219	1458	12	0.992277136	regulation of nucleobase, nucleoside, nucleotide and nucleic acid metabolism
GO:0016043	842	5	0.994974928	cell organization and biogenesis
GO:0006996	693	3	0.997938847	organelle organization and biogenesis
GO:0006259	489	1	0.99931745	DNA metabolism
GO:0006139	2328	19	0.999322877	nucleobase, nucleoside, nucleotide and nucleic acid metabolism
GO:0008150	9032	130	1	biological_process

17.3 GO: Molecular Function

GO ID	genes present in chip	genes present in tissues	p-value	GO term
GO:0004871	1837	54	5.31090422058036e-08	signal transducer activity
GO:0004872	1060	38	8.14096242015097e-08	receptor activity
GO:0019865	12	4	1.95048073839061e-05	immunoglobulin binding
GO:0019864	5	3	2.96233079580852e-05	IgG binding
GO:0035091	132	9	0.000127027538879623	phosphoinositide binding
GO:0005543	169	10	0.000173794467640364	phospholipid binding
GO:0048503	92	7	0.000374143212026926	GPI anchor binding
GO:0005529	103	7	0.00074113975360668	sugar binding
GO:0003823	30	4	0.000880905756950534	antigen binding
GO:0004888	694	20	0.0024738754105351	transmembrane receptor activity
GO:0008329	6	2	0.00304199323274668	pattern recognition receptor activity
GO:0008289	298	11	0.0041304347498414	lipid binding
GO:0042834	7	2	0.00421819877271117	peptidoglycan binding
GO:0030246	181	8	0.00490475596826529	carbohydrate binding
GO:0005178	26	3	0.00615630587661155	integrin binding
GO:0004522	9	2	0.00709424453032308	pancreatic ribonuclease activity
GO:0016494	9	2	0.00709424453032308	C-X-C chemokine receptor activity
GO:0019958	10	2	0.00878357542584136	C-X-C chemokine binding
GO:0016892	11	2	0.0106336289858617	endoribonuclease activity, producing 3'-phosphomonoesters
GO:0008656	12	2	0.0126394325953264	caspase activator activity
GO:0045012	12	2	0.0126394325953264	MHC class II receptor activity
GO:0001875	1	1	0.0145687034901995	lipopolysaccharide receptor activity
GO:0005286	1	1	0.0145687034901995	basic amino acid permease activity
GO:0017113	1	1	0.0145687034901995	dihydropyrimidine dehydrogenase (NADP+) activity
GO:0051076	1	1	0.0145687034901995	Gram-positive bacterial binding
GO:0042497	1	1	0.0145687034901995	triacylated lipoprotein binding
GO:0050528	1	1	0.0145687034901995	acyloxyacyl hydrolase activity
GO:0003933	1	1	0.0145687034901995	GTP cyclohydrolase activity
GO:0003934	1	1	0.0145687034901995	GTP cyclohydrolase I activity
GO:0004051	1	1	0.0145687034901995	arachidonate 5-lipoxygenase activity
GO:0016603	1	1	0.0145687034901995	glutaminyl-peptide cyclotransferase activity
GO:0004218	1	1	0.0145687034901995	cathepsin S activity
GO:0030881	1	1	0.0145687034901995	beta-2-microglobulin binding
GO:0030882	1	1	0.0145687034901995	lipid antigen binding
GO:0030884	1	1	0.0145687034901995	exogenous lipid antigen binding
GO:0004796	1	1	0.0145687034901995	thromboxane-A synthase activity
GO:0019955	65	4	0.0147147391970402	cytokine binding
GO:0016894	13	2	0.0147961226038278	endonuclease activity, active with either ribo- or deoxyribonucleic acids and producing 3'-phosphomonoesters
GO:0015026	13	2	0.0147961226038278	coreceptor activity
GO:0030693	14	2	0.0170989422433803	caspase activity
GO:0016505	14	2	0.0170989422433803	apoptotic protease activator activity
GO:0016504	15	2	0.0195432395830512	protease activator activity

GO:0008009	40	3	0.0201847960454094	chemokine activity
GO:0042379	40	3	0.0201847960454094	chemokine receptor binding
GO:0016810	72	4	0.0206924019139182	hydrolase activity, acting on carbon-nitrogen (but not peptide) bonds
GO:0001664	42	3	0.0229671877255315	G-protein-coupled receptor binding
GO:0004725	75	4	0.0236501707283619	protein tyrosine phosphatase activity
GO:0030676	2	1	0.0289266647286551	Rac guanyl-nucleotide exchange factor activity
GO:0001530	2	1	0.0289266647286551	lipopolysaccharide binding
GO:0004666	2	1	0.0289266647286551	prostaglandin-endoperoxide synthase activity
GO:0045309	2	1	0.0289266647286551	protein phosphorylated amino acid binding
GO:0004920	2	1	0.0289266647286551	interleukin-10 receptor activity
GO:0042132	2	1	0.0289266647286551	fructose-bisphosphatase activity
GO:0008934	2	1	0.0289266647286551	inositol-1(or 4)-monophosphatase activity
GO:0019969	2	1	0.0289266647286551	interleukin-10 binding
GO:0003938	2	1	0.0289266647286551	IMP dehydrogenase activity
GO:0004062	2	1	0.0289266647286551	aryl sulfotransferase activity
GO:0004152	2	1	0.0289266647286551	dihydroorotate dehydrogenase activity
GO:0004158	2	1	0.0289266647286551	dihydroorotate oxidase activity
GO:0001784	2	1	0.0289266647286551	phosphotyrosine binding
GO:0004197	80	4	0.0291245371812415	cysteine-type endopeptidase activity
GO:0005001	19	2	0.0306457251025986	transmembrane receptor protein tyrosine phosphatase activity
GO:0043028	19	2	0.0306457251025986	caspase regulator activity
GO:0019198	19	2	0.0306457251025986	transmembrane receptor protein phosphatase activity
GO:0016814	21	2	0.0369322577694199	hydrolase activity, acting on carbon-nitrogen (but not peptide) bonds, in cyclic amidines
GO:0016791	171	6	0.0387456746181906	phosphoric monoester hydrolase activity
GO:0001871	88	4	0.0393295099238820	pattern binding
GO:0001637	22	2	0.0402450381922454	G-protein chemoattractant receptor activity
GO:0004950	22	2	0.0402450381922454	chemokine receptor activity
GO:0004873	3	1	0.043076910415071	asialoglycoprotein receptor activity
GO:0005128	3	1	0.043076910415071	erythropoietin receptor binding
GO:0001948	3	1	0.043076910415071	glycoprotein binding
GO:0004944	3	1	0.043076910415071	C5a anaphylatoxin receptor activity
GO:0003796	3	1	0.043076910415071	lysozyme activity
GO:0019834	3	1	0.043076910415071	phospholipase A2 inhibitor activity
GO:0016019	3	1	0.043076910415071	peptidoglycan receptor activity
GO:0051219	3	1	0.043076910415071	phosphoprotein binding
GO:0042608	3	1	0.043076910415071	T cell receptor binding
GO:0004237	3	1	0.043076910415071	membrane dipeptidase activity
GO:0005087	3	1	0.043076910415071	Ran guanyl-nucleotide exchange factor activity
GO:0019956	24	2	0.0471902122520441	chemokine binding
GO:0005488	7228	114	0.0477630489489399	binding
GO:0001653	94	4	0.0481645874824345	peptide receptor activity
GO:0008528	94	4	0.0481645874824345	peptide receptor activity, G-protein coupled
GO:0005515	3709	64	0.0494830721979368	protein binding
GO:0008234	98	4	0.0546165879323198	cysteine-type peptidase activity
GO:0008047	188	6	0.0566786789317429	enzyme activator activity
GO:0004942	4	1	0.0570224240921834	anaphylatoxin receptor activity
GO:0019238	4	1	0.0570224240921834	cyclohydrolase activity

GO:0030234	509	12	0.0674482138492229	enzyme regulator activity
GO:0004559	5	1	0.070766146756668	alpha-mannosidase activity
GO:0008301	5	1	0.070766146756668	DNA bending activity
GO:0004960	5	1	0.070766146756668	thromboxane receptor activity
GO:0004957	5	1	0.070766146756668	prostaglandin E receptor activity
GO:0019863	5	1	0.070766146756668	IgE binding
GO:0005521	5	1	0.070766146756668	lamin binding
GO:0004465	5	1	0.070766146756668	lipoprotein lipase activity
GO:0004185	5	1	0.070766146756668	serine carboxypeptidase activity
GO:0004521	31	2	0.0744735230657452	endoribonuclease activity
GO:0030674	69	3	0.0792356783776181	protein binding, bridging
GO:0005031	6	1	0.0843109774614524	tumor necrosis factor receptor activity
GO:0005484	6	1	0.0843109774614524	SNAP receptor activity
GO:0004982	6	1	0.0843109774614524	N-formyl peptide receptor activity
GO:0016165	6	1	0.0843109774614524	lipoxygenase activity
GO:0004331	6	1	0.0843109774614524	fructose-2,6-bisphosphate 2-phosphatase activity
GO:0015359	6	1	0.0843109774614524	amino acid permease activity
GO:0015174	6	1	0.0843109774614524	basic amino acid transporter activity
GO:0016811	34	2	0.0873813157720836	hydrolase activity, acting on carbon-nitrogen (but not peptide) bonds, in linear amides
GO:0051213	35	2	0.0918230603974325	dioxygenase activity
GO:0016701	35	2	0.0918230603974325	oxidoreductase activity, acting on single donors with incorporation of molecular oxygen
GO:0016702	35	2	0.0918230603974325	oxidoreductase activity, acting on single donors with incorporation of molecular oxygen, incorporation of two atoms of oxygen
GO:0004721	118	4	0.0934146604841363	phosphoprotein phosphatase activity
GO:0005035	7	1	0.0976597739095751	death receptor activity
GO:0043120	7	1	0.0976597739095751	tumor necrosis factor binding
GO:0019203	7	1	0.0976597739095751	carbohydrate phosphatase activity
GO:0046983	170	5	0.102429870625803	protein dimerization activity
GO:0004859	8	1	0.110815353039696	phospholipase inhibitor activity
GO:0017017	8	1	0.110815353039696	MAP kinase phosphatase activity
GO:0004955	8	1	0.110815353039696	prostaglandin receptor activity
GO:0016805	8	1	0.110815353039696	dipeptidase activity
GO:0016634	8	1	0.110815353039696	oxidoreductase activity, acting on the CH-CH group of donors, oxygen as acceptor
GO:0042277	127	4	0.114206978075854	peptide binding
GO:0016788	446	10	0.115655934240449	hydrolase activity, acting on ester bonds
GO:0042578	228	6	0.115744661087269	phosphoric ester hydrolase activity
GO:0001584	282	7	0.117593313036041	rhodopsin-like receptor activity
GO:0008367	9	1	0.123780491603368	bacterial binding
GO:0004016	9	1	0.123780491603368	adenylate cyclase activity
GO:0005125	181	5	0.124493251719697	cytokine activity
GO:0005057	132	4	0.126569280356716	receptor signaling protein activity
GO:0004953	10	1	0.136557926734205	icosanoid receptor activity
GO:0004954	10	1	0.136557926734205	prostanoid receptor activity
GO:0008239	10	1	0.136557926734205	dipeptidyl-peptidase activity
GO:0016755	10	1	0.136557926734205	transferase activity, transferring amino-acyl groups
GO:0016806	10	1	0.136557926734205	dipeptidyl-peptidase and tripeptidyl-peptidase activity
GO:0004716	10	1	0.136557926734205	receptor signaling protein tyrosine kinase activity
GO:0004857	187	5	0.137387220542663	enzyme inhibitor activity

GO:0004540	45	2	0.139361448635940	ribonuclease activity
GO:0005102	528	11	0.147145104922723	receptor binding
GO:0005355	11	1	0.149150356509039	glucose transporter activity
GO:0004896	48	2	0.154503811346328	hematopoietin/interferon-class (D200-domain) cytokine receptor activity
GO:0016493	12	1	0.161560440501194	C-C chemokine receptor activity
GO:0019957	12	1	0.161560440501194	C-C chemokine binding
GO:0015145	12	1	0.161560440501194	monosaccharide transporter activity
GO:0015149	12	1	0.161560440501194	hexose transporter activity
GO:0015923	13	1	0.173790800325975	mannosidase activity
GO:0004866	99	3	0.175058936645858	endopeptidase inhibitor activity
GO:0030414	100	3	0.178679007688071	protease inhibitor activity
GO:0019901	54	2	0.185633475777288	protein kinase binding
GO:0005540	14	1	0.185844020178483	hyaluronic acid binding
GO:0016628	14	1	0.185844020178483	oxidoreductase activity, acting on the CH-CH group of donors, NAD or NADP as acceptor
GO:0005506	212	5	0.196848041268029	iron ion binding
GO:0000146	15	1	0.197722647363870	microfilament motor activity
GO:0004715	15	1	0.197722647363870	non-membrane spanning protein tyrosine kinase activity
GO:0004713	161	4	0.207793063699888	protein-tyrosine kinase activity
GO:0004553	59	2	0.212194396875997	hydrolase activity, hydrolyzing O-glycosyl compounds
GO:0016564	59	2	0.212194396875997	transcriptional repressor activity
GO:0019900	60	2	0.217553740951356	kinase binding
GO:0005544	17	1	0.220966131633578	calcium-dependent phospholipid binding
GO:0008233	399	8	0.226043548413686	peptidase activity
GO:0005509	646	12	0.231332156715944	calcium ion binding
GO:0008034	18	1	0.232335903546947	lipoprotein binding
GO:0016849	18	1	0.232335903546947	phosphorus-oxygen lyase activity
GO:0004298	18	1	0.232335903546947	threonine endopeptidase activity
GO:0004867	64	2	0.239097365506856	serine-type endopeptidase inhibitor activity
GO:0004869	19	1	0.243540913460522	cysteine protease inhibitor activity
GO:0004519	65	2	0.244502746374726	endonuclease activity
GO:0004930	349	7	0.246637299474665	G-protein coupled receptor activity
GO:0001614	20	1	0.254583531926075	purinergic nucleotide receptor activity
GO:0001608	20	1	0.254583531926075	nucleotide receptor activity, G-protein coupled
GO:0005164	20	1	0.254583531926075	tumor necrosis factor receptor binding
GO:0003690	20	1	0.254583531926075	double-stranded DNA binding
GO:0016502	20	1	0.254583531926075	nucleotide receptor activity
GO:0009975	20	1	0.254583531926075	cyclase activity
GO:0045028	20	1	0.254583531926075	purinergic nucleotide receptor activity, G-protein coupled
GO:0008565	67	2	0.255327490605592	protein transporter activity
GO:0008238	68	2	0.260744319053091	exopeptidase activity
GO:0004601	22	1	0.276190907893034	peroxidase activity
GO:0016684	22	1	0.276190907893034	oxidoreductase activity, acting on peroxide as acceptor
GO:0003779	189	4	0.296732055733071	actin binding
GO:0005478	24	1	0.297176327228621	intracellular transporter activity
GO:0004860	25	1	0.307441378244195	protein kinase inhibitor activity
GO:0016712	25	1	0.307441378244195	oxidoreductase activity, acting on paired donors, with incorporation or reduction of molecular oxygen, reduced flavin or flavoprotein as one donor, and incorporation of one atom of oxygen

GO:0005539	77	2	0.309421340722877	glycosaminoglycan binding
GO:0016798	77	2	0.309421340722877	hydrolase activity, acting on glycosyl bonds
GO:0008138	26	1	0.317557566603176	protein tyrosine/serine/threonine phosphatase activity
GO:0004907	26	1	0.317557566603176	interleukin receptor activity
GO:0003725	26	1	0.317557566603176	double-stranded RNA binding
GO:0003704	26	1	0.317557566603176	specific RNA polymerase II transcription factor activity
GO:0004437	26	1	0.317557566603176	inositol or phosphatidylinositol phosphatase activity
GO:0030247	80	2	0.325536399705378	polysaccharide binding
GO:0004180	27	1	0.327527035677496	carboxypeptidase activity
GO:0004722	27	1	0.327527035677496	protein serine/threonine phosphatase activity
GO:0016787	1424	23	0.328953090184168	hydrolase activity
GO:0005279	28	1	0.337351898200182	amino acid-polyamine transporter activity
GO:0003697	28	1	0.337351898200182	single-stranded DNA binding
GO:0015203	28	1	0.337351898200182	polyamine transporter activity
GO:0003700	649	11	0.346095313019619	transcription factor activity
GO:0019210	29	1	0.347034236700147	kinase inhibitor activity
GO:0051119	29	1	0.347034236700147	sugar transporter activity
GO:0019965	30	1	0.356576103930875	interleukin binding
GO:0015144	30	1	0.356576103930875	carbohydrate transporter activity
GO:0016705	87	2	0.3627255795418	oxidoreductase activity, acting on paired donors, with incorporation or reduction of molecular oxygen
GO:0016209	31	1	0.365979523293046	antioxidant activity
GO:0016860	31	1	0.365979523293046	intramolecular oxidoreductase activity
GO:0008146	32	1	0.375246489251223	sulfotransferase activity
GO:0019899	151	3	0.378038739994334	enzyme binding
GO:0005085	93	2	0.393990572065077	guanyl-nucleotide exchange factor activity
GO:0030695	219	4	0.396135863229551	GTPase regulator activity
GO:0042802	158	3	0.405676422425238	protein self binding
GO:0004175	288	5	0.410164842023644	endopeptidase activity
GO:0016782	36	1	0.410988718414884	transferase activity, transferring sulfur-containing groups
GO:0016627	37	1	0.419602349991983	oxidoreductase activity, acting on the CH-CH group of donors
GO:0005126	38	1	0.428090909898456	hematopoietin/interferon-class (D200-domain) cytokine receptor binding
GO:0005070	40	1	0.444700001275198	SH3/SH2 adaptor activity
GO:0009055	104	2	0.449409800127487	electron carrier activity
GO:0004221	41	1	0.452824061881739	ubiquitin thiolesterase activity
GO:0004518	105	2	0.454309468801786	nuclease activity
GO:0015036	42	1	0.460830109818417	disulfide oxidoreductase activity
GO:0004843	43	1	0.468719847131317	ubiquitin-specific protease activity
GO:0008235	44	1	0.476494951495262	metalloexopeptidase activity
GO:0050660	44	1	0.476494951495262	FAD binding
GO:0005516	112	2	0.487898197021564	calmodulin binding
GO:0019887	47	1	0.499148885313661	protein kinase regulator activity
GO:0015171	48	1	0.506481759227859	amino acid transporter activity
GO:0005096	117	2	0.511095724632205	GTPase activator activity
GO:0019838	49	1	0.513708034953903	growth factor binding
GO:0016790	51	1	0.527846924009849	thiolester hydrolase activity
GO:0005244	124	2	0.54240530092883	voltage-gated ion channel activity
GO:0005275	58	1	0.57419432690653	amine transporter activity

GO:0019207	58	1	0.57419432690653	kinase regulator activity
GO:0016298	59	1	0.580435705010505	lipase activity
GO:0008092	279	4	0.583582952121388	cytoskeletal protein binding
GO:0008201	60	1	0.586586246774219	heparin binding
GO:0046982	61	1	0.592647264778479	protein heterodimerization activity
GO:0016563	62	1	0.598620052773815	transcriptional activator activity
GO:0005249	63	1	0.604505885948669	voltage-gated potassium channel activity
GO:0003713	140	2	0.608659835312946	transcription coactivator activity
GO:0042803	64	1	0.610306021193777	protein homodimerization activity
GO:0016491	509	7	0.61703254223393	oxidoreductase activity
GO:0005342	66	1	0.621654135529411	organic acid transporter activity
GO:0046943	66	1	0.621654135529411	carboxylic acid transporter activity
GO:0008134	293	4	0.622447872349634	transcription factor binding
GO:0008083	144	2	0.624051943553318	growth factor activity
GO:0005083	144	2	0.624051943553318	small GTPase regulator activity
GO:0004672	447	6	0.639599068226653	protein kinase activity
GO:0004497	71	1	0.648609280915833	monooxygenase activity
GO:0005489	152	2	0.653435579451813	electron transporter activity
GO:0003712	230	3	0.655854106584421	transcription cofactor activity
GO:0030528	963	13	0.656595476115062	transcription regulator activity
GO:0046906	74	1	0.663857931543602	tetrapyrrole binding
GO:0020037	74	1	0.663857931543602	heme binding
GO:0016616	77	1	0.678449419520772	oxidoreductase activity, acting on the CH-OH group of donors, NAD or NADP as acceptor
GO:0016789	80	1	0.692411869806449	carboxylic ester hydrolase activity
GO:0003714	81	1	0.696930921713954	transcription corepressor activity
GO:0016614	83	1	0.705772212091447	oxidoreductase activity, acting on CH-OH group of donors
GO:0000287	251	3	0.713065944170741	magnesium ion binding
GO:0030955	86	1	0.718556231237691	potassium ion binding
GO:0050662	88	1	0.726770966642333	coenzyme binding
GO:0005267	90	1	0.734747606551216	potassium channel activity
GO:0016853	92	1	0.742493003347705	isomerase activity
GO:0008415	98	1	0.764407354875953	acyltransferase activity
GO:0003774	98	1	0.764407354875953	motor activity
GO:0016747	98	1	0.764407354875953	transferase activity, transferring groups other than amino-acyl groups
GO:0048037	100	1	0.771292469192258	cofactor binding
GO:0016746	104	1	0.784469026789539	transferase activity, transferring acyl groups
GO:0003702	197	2	0.78595941273436	RNA polymerase II transcription factor activity
GO:0016301	609	7	0.792290119296631	kinase activity
GO:0016773	532	6	0.794720958812298	phosphotransferase activity, alcohol group as acceptor
GO:0043169	2313	30	0.797250932157651	cation binding
GO:0015078	112	1	0.80860300690001	hydrogen ion transporter activity
GO:0008236	117	1	0.822303608710112	serine-type peptidase activity
GO:0046872	2495	32	0.826715751636184	metal ion binding
GO:0015077	121	1	0.832559315685891	monovalent inorganic cation transporter activity
GO:0016829	127	1	0.84685111184919	lyase activity
GO:0008237	133	1	0.859931067281147	metallopeptidase activity
GO:0046914	1596	19	0.862974438009566	transition metal ion binding

GO:0043167	2556	32	0.866689754864855	ion binding
GO:0031420	146	1	0.884588195995479	alkali metal ion binding
GO:0003723	436	4	0.885103113146597	RNA binding
GO:0005216	252	2	0.886258090481503	ion channel activity
GO:0015290	149	1	0.889635898709244	electrochemical potential-driven transporter activity
GO:0015291	149	1	0.889635898709244	porter activity
GO:0016772	708	7	0.899207078646857	transferase activity, transferring phosphorus-containing groups
GO:0008270	1287	14	0.909799585543837	zinc ion binding
GO:0015268	274	2	0.91257965025563	alpha-type channel activity
GO:0015267	284	2	0.922565825822247	channel or pore class transporter activity
GO:0005386	398	3	0.933823182056203	carrier activity
GO:0005261	188	1	0.938373665753725	cation channel activity
GO:0005554	522	4	0.950568600921113	molecular function unknown
GO:0004674	326	2	0.953943919907844	protein serine/threonine kinase activity
GO:0005525	226	1	0.965152714110057	GTP binding
GO:0019001	235	1	0.969564641026454	guanyl nucleotide binding
GO:0005215	1030	9	0.970754480894989	transporter activity
GO:0003677	1384	13	0.974132706720426	DNA binding
GO:0003824	3409	39	0.978322860608727	catalytic activity
GO:0015075	523	3	0.984207773894675	ion transporter activity
GO:0008324	421	2	0.986469517275391	cation transporter activity
GO:0030554	1028	8	0.986581476074162	adenyl nucleotide binding
GO:0005524	970	7	0.990430518156904	ATP binding
GO:0016740	1169	9	0.99162901723977	transferase activity
GO:0003676	1990	18	0.994433622009241	nucleic acid binding
GO:0005198	488	2	0.99448203290403	structural molecule activity
GO:0017076	1248	9	0.99612140099092	purine nucleotide binding
GO:0000166	1432	11	0.996164844149184	nucleotide binding
GO:0003674	9541	139	1	molecular_function

17.4 GO: Cellular Component

GO ID	genes present in chip	genes present in tissues	p-value	GO term
GO:0005886	1472	45	7.54567606212292e-08	plasma membrane
GO:0005887	1057	35	6.03631991359341e-07	integral to plasma membrane
GO:0031226	1062	35	6.76096690699003e-07	intrinsic to plasma membrane
GO:0016020	3514	71	4.40050722745411e-05	membrane
GO:0016021	2674	54	0.00099727166668358	integral to membrane
GO:0031224	2681	54	0.00106685740442000	intrinsic to membrane
GO:0008305	24	3	0.00437921712373607	integrin complex
GO:0043235	58	4	0.00868641912482182	receptor complex
GO:0001533	12	2	0.0117204896463696	cornified envelope
GO:0043020	1	1	0.0140094940372818	NADPH oxidase complex
GO:0045335	3	1	0.041447165545656	phagocytic vesicle
GO:0005615	385	10	0.0433295880808765	extracellular space
GO:0031229	4	1	0.0548806717964067	intrinsic to nuclear inner membrane
GO:0005639	4	1	0.0548806717964067	integral to nuclear inner membrane
GO:0005577	5	1	0.0681274502873868	fibrinogen complex
GO:0016461	5	1	0.0681274502873868	unconventional myosin
GO:0008290	7	1	0.094071085646825	F-actin capping protein complex
GO:0005637	7	1	0.094071085646825	nuclear inner membrane
GO:0005925	8	1	0.106772985836481	focal adhesion
GO:0005625	183	5	0.113929534714001	soluble fraction
GO:0005924	9	1	0.119298245856620	cell-substrate adherens junction
GO:0042101	10	1	0.131649301982183	T cell receptor complex
GO:0030055	11	1	0.143828557164768	cell-matrix junction
GO:0030139	12	1	0.155838381484621	endocytic vesicle
GO:0005769	13	1	0.167681112596547	early endosome
GO:0005764	107	3	0.189195963605687	lysosome
GO:0000323	107	3	0.189195963605687	lytic vacuole
GO:0005626	15	1	0.190874486322129	insoluble fraction
GO:0005665	16	1	0.202229646047834	DNA-directed RNA polymerase II, core complex
GO:0005768	62	2	0.215506096268454	endosome
GO:0005839	18	1	0.224467973108408	proteasome core complex (sensu Eukaryota)
GO:0005912	18	1	0.224467973108408	adherens junction
GO:0005773	120	3	0.236584509202628	vacuole
GO:0005576	949	16	0.252341794409916	extracellular region
GO:0048471	22	1	0.267116716836047	perinuclear region
GO:0000793	23	1	0.277410260337247	condensed chromosome
GO:0030880	23	1	0.277410260337247	RNA polymerase complex
GO:0008021	27	1	0.317170137209718	synaptic vesicle
GO:0005938	28	1	0.326766236326748	cell cortex
GO:0001772	29	1	0.33622857636676	immunological synapse
GO:0000267	698	11	0.387742578866975	cell fraction
GO:0019898	37	1	0.407328627917553	extrinsic to membrane
GO:0000502	41	1	0.439991832692264	proteasome complex (sensu Eukaryota)

GO:0000228	42	1	0.447874683814209	nuclear chromosome
GO:0016459	43	1	0.455647477677906	myosin
GO:0016327	50	1	0.507100841949698	apicolateral plasma membrane
GO:0016023	122	2	0.512683233634654	cytoplasmic membrane-bound vesicle
GO:0031410	124	2	0.521413327511116	cytoplasmic vesicle
GO:0008076	53	1	0.527647399093381	voltage-gated potassium channel complex
GO:0005829	276	4	0.544096544295602	cytosol
GO:0016591	58	1	0.560020710088394	DNA-directed RNA polymerase II, holoenzyme
GO:0009986	61	1	0.57837815890194	cell surface
GO:0030136	65	1	0.601678169414534	clathrin-coated vesicle
GO:0005856	612	8	0.63272069701201	cytoskeleton
GO:0031301	76	1	0.659372653128457	integral to organelle membrane
GO:0030135	79	1	0.673614258187198	coated vesicle
GO:0005635	83	1	0.691688390683387	nuclear membrane
GO:0019866	89	1	0.716954618596882	inner membrane
GO:0031300	90	1	0.720961227294797	intrinsic to organelle membrane
GO:0015629	183	2	0.73099543977199	actin cytoskeleton
GO:0030054	93	1	0.732646658720536	cell junction
GO:0000785	100	1	0.758059150539072	chromatin
GO:0005623	7802	107	0.81038540456137	cell
GO:0005624	521	5	0.862503534119508	membrane fraction
GO:0008372	523	5	0.864716309476174	cellular component unknown
GO:0005737	2530	29	0.920997875081823	cytoplasm
GO:0005578	303	2	0.929623202074849	extracellular matrix (sensu Metazoa)
GO:0031012	304	2	0.930451070720896	extracellular matrix
GO:0005794	195	1	0.938132679621866	Golgi apparatus
GO:0043228	977	9	0.940332595715385	non-membrane-bound organelle
GO:0043232	977	9	0.940332595715385	intracellular non-membrane-bound organelle
GO:0005694	203	1	0.944883486093855	chromosome
GO:0005654	221	1	0.957518268044818	nucleoplasm
GO:0012505	246	1	0.970437406112102	endomembrane system
GO:0043234	1247	10	0.986331542159616	protein complex
GO:0031090	377	1	0.995656392945882	organelle membrane
GO:0005783	483	1	0.999100276572009	endoplasmic reticulum
GO:0005634	2704	20	0.999937761619727	nucleus
GO:0005622	5419	50	0.999999519909935	intracellular
GO:0043227	3955	27	0.99999998093822	membrane-bound organelle
GO:0043231	3955	27	0.99999998093822	intracellular membrane-bound organelle
GO:0043226	4515	33	0.99999999461746	organelle
GO:0043229	4515	33	0.99999999461746	intracellular organelle
GO:0005575	8637	121	1	cellular_component

18. PB-CD56NKCells

18.1 PB-CD56NKCells selective genes (150 genes)

Symbol	Description	Chromosome	Chromosome Location	LocusLink	Cytoband	UniGene
KLRF1	killer cell lectin-like receptor subfamily F, member 1	12	9871343	51348	12p12.3-13.2	Hs.183125
MYOM2	myomesin (M-protein) 2, 165kDa	8	1980654	9172	8p23.3	Hs.443683
NKG7	natural killer cell group 7 sequence	19	-56566686	4818	19q13.33	Hs.10306
FCGR3B	Fc fragment of IgG, low affinity IIIb, receptor (CD16b)	1	-159859676	2215	1q23	Hs.372679
GZMB	granzyme B (granzyme 2, cytotoxic T-lymphocyte-associated serine esterase 1)	14	-24170003	3002	14q11.2	Hs.1051
GNLY	granulysin	2	85774924, 85775059	10578	2p12-q11	Hs.105806
PRF1	perforin 1 (pore forming protein)	10	-72027110	5551	10q22	Hs.2200
FCGR3A	Fc fragment of IgG, low affinity IIIa, receptor (CD16a)	1	-159778174	2214	1q23	Hs.372679
GZMA	granzyme A (granzyme 1, cytotoxic T-lymphocyte-associated serine esterase 3)	5	54434229	3001	5q11-q12	Hs.90708
CLIC3	chloride intracellular channel 3	9	-139008880	9022	9q34.3	Hs.64746
CD69	CD69 molecule	12	-9796352	969	12p13-p12	Hs.208854
CD247	CD247 molecule	1	-165666507	919	1q22-q23	Hs.156445
TRDC	T cell receptor delta constant	14	NA	28526	14q11	NA
CD160	CD160 molecule	1	-144407154	11126	1q21.1	Hs.488237
ITGAM	integrin, alpha M (complement component 3 receptor 3 subunit)	16	31178788	3684	16p11.2	Hs.172631
CCL5	chemokine (C-C motif) ligand 5	17	-31222609	6352	17q11.2-q12	Hs.514821
MYBL1	v-myb myeloblastosis viral oncogene homolog (avian)-like 1	8	NA	4603	8q22	Hs.445898
KLRB1	killer cell lectin-like receptor subfamily B, member 1	12	-9639137	3820	12p13	Hs.169824
PTGDR	prostaglandin D2 receptor (DP)	14	51804180	5729	14q22.1	Hs.306831
PTPRC	protein tyrosine phosphatase, receptor type, C	1	196874847	5788	1q31-q32	Hs.192039
IL18RAP	interleukin 18 receptor accessory protein	2	102401685	8807	2p24.3-p24.1	Hs.158315
KLRC3	killer cell lectin-like receptor subfamily C, member 3	12	-20915633	3823	12p13	Hs.74082
GIMAP6	GTPase, IMAP family member 6	7	-149953398	474344	NA	Hs.438823
GPR65	G protein-coupled receptor 65	14	87541248	8477	14q31-q32.1	Hs.513440
RASGRP1	RAS guanyl releasing protein 1 (calcium and DAG-regulated)	15	-36567597	10125	15q15	Hs.591127
TYROBP	TYRO protein tyrosine kinase binding protein	19	-41087145	7305	19q13.1	Hs.515369
IGHA1	immunoglobulin heavy constant alpha 1	14	NA	3493	14q32.33	Hs.510635
RUNX3	runt-related transcription factor 3	1	-25098589	864	1p36	Hs.170019
CST7	cystatin F (leukocystatin)	20	24877865	8530	20p11.21	Hs.143212
LAIR2	leukocyte-associated immunoglobulin-like receptor 2	19	59705824	3904	19q13.4	Hs.43803
CX3CR1	chemokine (C-X3-C motif) receptor 1	3	-39279989	1524	3p21|3p21.3	Hs.78913
GCH1	GTP cyclohydrolase 1 (dopa-responsive dystonia)	14	-54378475	2643	14q22.1-q22.2	Hs.86724
RP6-	NA	X	130985316, 130984925	51765	Xq26.2	Hs.444247

213H19.1						
PSCDBP	pleckstrin homology, Sec7 and coiled-coil domains, binding protein	2	-157979377	9595	2q11.2	Hs.270
PTGER2	prostaglandin E receptor 2 (subtype EP2), 53kDa	14	51850862	5732	14q22	Hs.2090
TXK	TXK tyrosine kinase	4	-47763517	7294	4p12	Hs.479669
PLAC8	placenta-specific 8	4	-84230866	51316	4q21.22	Hs.546392
PMAIP1	phorbol-12-myristate-13-acetate-induced protein 1	18	55718216	5366	18q21.32	Hs.96
ADCY7	adenylate cyclase 7	16	48879322	113	16q12-q13	Hs.513578
GZMH	granzyme H (cathepsin G-like 2, protein h-CCPX)	14	-24145532	2999	14q11.2	Hs.348264
RABGAP1L	RAB GTPase activating protein 1-like	1	173111306, 172395256	9910	1q24	Hs.585378 Hs.591475 Hs.615081
PTPN6	protein tyrosine phosphatase, non-receptor type 6	12	6930703, 6926000	5777	12p13	Hs.63489
TRAF3IP3	TRAF3 interacting protein 3	1	207996146	80342	1q32.3-q41	Hs.147434
IGJ	immunoglobulin J polypeptide, linker protein for immunoglobulin alpha and mu polypeptides	4	-71740547	3512	4q21	Hs.643431
CD7	CD7 molecule	17	-77866034	924	17q25.2-q25.3	Hs.36972
EVI2B	ecotropic viral integration site 2B	17	-26654918	2124	17q11.2	Hs.5509
TNFRSF1B	tumor necrosis factor receptor superfamily, member 1B	1	12149646	7133	1p36.3-p36.2	Hs.256278
ITK	IL2-inducible T-cell kinase	5	156540484	3702	5q31-q32	Hs.558348
AKR1C3	aldo-keto reductase family 1, member C3 (3-alpha hydroxysteroid dehydrogenase, type II)	10	5126567	8644	10p15-p14	Hs.78183
ZAP70	zeta-chain (TCR) associated protein kinase 70kDa	2	97696462, 97717300	7535	2q12	Hs.234569
DOCK2	dedicator of cytokinesis 2	5	168996870	1794	5q35.1	Hs.586174
IL2RB	interleukin 2 receptor, beta	22	-35851827	3560	22q13|22q13.1	Hs.474787
KLRD1	killer cell lectin-like receptor subfamily D, member 1	12	10351683	3824	12p13	Hs.562457
LCK	lymphocyte-specific protein tyrosine kinase	1	32489518	3932	1p34.3	Hs.470627
BTN3A3	butyrophilin, subfamily 3, member A3	6	26548741	10384	6p21.3	Hs.167741
CHST2	carbohydrate (N-acetylglucosamine-6-O) sulfotransferase 2	3	144321357	9435	3q24	Hs.8786
BIN2	bridging integrator 2	12	-49961089	51411	12q13	Hs.14770
BTN3A2	butyrophilin, subfamily 3, member A2	6	26473376	11118	6p22.1	Hs.376046
FGR	Gardner-Rasheed feline sarcoma viral (v-fgr) oncogene homolog	1	-27811397	2268	1p36.2-p36.1	Hs.1422
GMFG	glia maturation factor, gamma	19	-44510841	9535	19q13.2	Hs.5210
RARRES3	retinoic acid receptor responder (tazarotene induced) 3	11	63060855	5920	11q23	Hs.17466
PPP2R5E	protein phosphatase 2, regulatory subunit B (B56), epsilon isoform	14	-62911107	5529	14q23.1	Hs.334868
CTA-246H3.1	NA	22	24043887	91353	22q11.23	Hs.567636
LYZ	lysozyme (renal amyloidosis)	12	68028430	4069	12q15	Hs.524579
PYCARD	PYD and CARD domain containing	16	-31120309	29108	16p12-p11.2	Hs.499094
TGFBR3	transforming growth factor, beta receptor III (betaglycan, 300kDa)	1	-91920574	7049	1p33-p32	Hs.482390
DENND2D	DENN/MADD domain containing 2D	1	-111531323	79961	1p13.3	Hs.557850
COTL1	coactosin-like 1 (Dictyostelium)	16	-83156706	23406	16q24.1	Hs.289092
STK10	serine/threonine kinase 10	5	-171403229	6793	5q35.1	Hs.519756

FGL2	fibrinogen-like 2	7	-76660625	10875	7q11.23	Hs.520989
ITGA4	integrin, alpha 4 (antigen CD49D, alpha 4 subunit of VLA-4 receptor)	2	182029863	3676	2q31.3	Hs.440955
TNFAIP8	tumor necrosis factor, alpha-induced protein 8	5	118719628	25816	5q23.1	Hs.271955
PSMB10	proteasome (prosome, macropain) subunit, beta type, 10	16	-66525907	5699	16q22.1	Hs.9661
ARHGEF3	Rho guanine nucleotide exchange factor (GEF) 3	3	-56736485	50650	3p21-p13	Hs.476402
CD300A	CD300a molecule	17	69974116	11314	17q25.1	Hs.9688
CXCR4	chemokine (C-X-C motif) receptor 4	2	-136588389	7852	2q21	Hs.421986
LYN	v-yes-1 Yamaguchi sarcoma viral related oncogene homolog	8	56954925	4067	8q13	Hs.491767 Hs.614775
GNPTAB	N-acetylglucosamine-1-phosphate transferase, alpha and beta subunits	12	-100663408	79158	12q23.2	Hs.46850
PRKCH	protein kinase C, eta	14	60858267	5583	14q22-q23	Hs.333907
AOAH	acyloxyacyl hydrolase (neutrophil)	7	-36519134	313	7p14-p12	Hs.488007
PTPN4	protein tyrosine phosphatase, non-receptor type 4 (megakaryocyte)	2	120233676	5775	2q14.2	Hs.469809
HCK	hemopoietic cell kinase	20	30103717	3055	20q11-q12	Hs.126521
PTPRCAP	protein tyrosine phosphatase, receptor type, C-associated protein	11	-66959556	5790	11q13.3	Hs.155975
GZMM	granzyme M (lymphocyte met-ase 1)	19	495026	3004	19p13.3	Hs.465511
ALOX5AP	arachidonate 5-lipoxygenase-activating protein	13	30207668	241	13q12	Hs.507658
LCP2	lymphocyte cytosolic protein 2 (SH2 domain containing leukocyte protein of 76kDa)	5	-169607666	3937	5q33.1-qter	Hs.304475
SELL	selectin L (lymphocyte adhesion molecule 1)	1	-167926433	6402	1q23-q25	Hs.82848
GPR18	G protein-coupled receptor 18	13	-98704972	2841	13q32	Hs.631765
WASPIP	Wiskott-Aldrich syndrome protein interacting protein	2	-175132548	7456	2q31.1	Hs.591641
ARL4C	ADP-ribosylation factor-like 4C	2	-235066426	10123	2q37.1	Hs.111554
RAB7L1	RAB7, member RAS oncogene family-like 1	1	-204004300	8934	1q32	Hs.115325
CTSW	cathepsin W (lymphopain)	11	65403859	1521	11q13.1	Hs.416848
VAMP8	vesicle-associated membrane protein 8 (endobrevin)	2	85658227	8673	2p12-p11.2	Hs.534373
PRG1	proteoglycan 1, secretory granule	10	70517833	5552	10q22.1	Hs.1908
ISG20	interferon stimulated exonuclease gene 20kDa	15	86983042	3669	15q26	Hs.459265
RASGRP2	RAS guanyl releasing protein 2 (calcium and DAG-regulated)	11	-64250959	10235	11q13	Hs.99491
ITGAL	integrin, alpha L (antigen CD11A (p180), lymphocyte function-associated antigen 1, alpha polypeptide)	16	30391571	3683	16p11.2	Hs.174103
ICAM3	intercellular adhesion molecule 3	19	-10305453	3385	19p13.3-p13.2	Hs.75516
SPON2	spondin 2, extracellular matrix protein	4	-1150722	10417	4p16.3	Hs.302963
DUSP2	dual specificity phosphatase 2	2	-96172643	1844	2q11	Hs.1183
APOBEC3G	apolipoprotein B mRNA editing enzyme, catalytic polypeptide-like 3G	22	37803081	60489	22q13.1-q13.2	Hs.441124
CD48	CD48 molecule	1	-158915161	962	1q21.3-q22	Hs.243564
BCL2A1	BCL2-related protein A1	15	-78040289	597	15q24.3	Hs.227817
CD52	CD52 molecule	1	26516997	1043	1p36	Hs.276770
RNF138	ring finger protein 138	18	27925834, 27926644	51444	18q12.1	Hs.302408 Hs.501040

CASP1	caspase 1, apoptosis-related cysteine peptidase (interleukin 1, beta, convertase)	11	-104401451	834	11q23	Hs.2490
PTPN12	protein tyrosine phosphatase, non-receptor type 12	7	77004770	5782	7q11.23	Hs.61812
DCK	deoxycytidine kinase	4	72078255	1633	4q13.3-q21.1	Hs.709
EVI2A	ecotropic viral integration site 2A	17	-26668801	2123	17q11.2	Hs.591198
MYO1F	myosin IF	19	-8491998	4542	19p13.3-p13.2	Hs.465818
MNDA	myeloid cell nuclear differentiation antigen	1	157067791	4332	1q22	Hs.153837
CTSS	cathepsin S	1	-148969177	1520	1q21	Hs.181301
PSMC6	proteasome (prosome, macropain) 26S subunit, ATPase, 6	14	52243667	5706	14q22.1	Hs.156171 Hs.491779
LOC646082	NA	22	NA	646082	22q11.23	NA
RBM3	RNA binding motif (RNP1, RRM) protein 3	X	48317779	5935	Xp11.2	Hs.301404
IFI16	interferon, gamma-inducible protein 16	1	157246330	3428	1q22	Hs.380250
CD37	CD37 molecule	19	54530488	951	19q13.3	Hs.166556
CBLB	Cas-Br-M (murine) ecotropic retroviral transforming sequence b	3	-106859799	868	3q13.11	Hs.430589
LRRFIP1	leucine rich repeat (in FLII) interacting protein 1	2	238265667	9208	2q37.3	Hs.471779
ZNF651	zinc finger protein 651	3	42675877	92999	3p22.1	Hs.409561
CD97	CD97 molecule	19	14353212	976	19p13	Hs.466039
MAFB	v-maf musculoaponeurotic fibrosarcoma oncogene homolog B (avian)	20	-38747932	9935	20q11.2-q13.1	Hs.642679
EDG8	endothelial differentiation, sphingolipid G-protein-coupled receptor, 8	19	-10484622	53637	19p13.2	Hs.501561
HMGB2	high-mobility group box 2	4	-174489362	3148	4q31	Hs.434953
SEMA4D	sema domain, immunoglobulin domain (Ig), transmembrane domain (TM) and short cytoplasmic domain, (semaphorin) 4D	9	-91181971	10507	9q22-q31	Hs.494406
CCDC69	coiled-coil domain containing 69	5	-150540806	26112	5q33.1	Hs.132994
STK38	serine/threonine kinase 38	6	-36569649	11329	6p21	Hs.409578
KLRK1	killer cell lectin-like receptor subfamily K, member 1	12	-10416219	22914	12p13.2-p12.3	Hs.387787
FAM117A	family with sequence similarity 117, member A	17	-45142687	81558	17q21.33	Hs.514308
C1orf38	chromosome 1 open reading frame 38	1	28071641	9473	1p35.3	Hs.10649
IER5	immediate early response 5	1	179324260	51278	1q25.3	Hs.15725
CENTB2	centaurin, beta 2	3	-196476767	23527	3q29	Hs.478746
HECA	headcase homolog (Drosophila)	6	139497941	51696	6q23-q24	Hs.197644
TRIM22	tripartite motif-containing 22	11	5667663	10346	11p15	Hs.501778 Hs.621411
SERPINA1	serpin peptidase inhibitor, clade A (alpha-1 antiproteinase, antitrypsin), member 1	14	-93914450	5265	14q32.1	Hs.525557
IL32	interleukin 32	16	3055313, 3055663, 3055639, 3055612, 3055785	9235	16p13.3	Hs.943
TCIRG1	T-cell, immune regulator 1, ATPase, H+ transporting, lysosomal V0 subunit A3	11	67563058, 67567022	10312	11q13.2	Hs.495985
IL10RA	interleukin 10 receptor, alpha	11	117362318	3587	11q23	Hs.504035
DGKZ	diacylglycerol kinase, zeta 104kDa	11	46325594, 46311314, 46323562	8525	11p11.2	Hs.502461
HCLS1	hematopoietic cell-specific Lyn substrate 1	3	-122832936	3059	3q13	Hs.14601
MBNL1	muscleblind-like (Drosophila)	3	153468518, 153499883	4154	3q25	Hs.478000 Hs.558914

SYNE2	spectrin repeat containing, nuclear envelope 2	14	63389453, 63751902, 63740825, 63750614, 63612085	23224	14q23.2	Hs.525392
RGS2	regulator of G-protein signalling 2, 24kDa	1	191044793	5997	1q31	Hs.78944
MCM5	MCM5 minichromosome maintenance deficient 5, cell division cycle 46 (S. cerevisiae)	22	34126127	4174	22q13.1	Hs.517582
PSTPIP1	proline-serine-threonine phosphatase interacting protein 1	15	75074608	9051	15q24-q25.1	Hs.129758
AREG	amphiregulin (schwannoma-derived growth factor)	4	75529716, 75699652	374	4q13-q21	Hs.270833
LOC653193	NA	4	NA	653193	4q13.3	Hs.632601
CORO1A	coronin, actin binding protein, 1A	16	30102426	11151	16p11.2	Hs.415067
LBR	lamin B receptor	1	-223655827	3930	1q42.1	Hs.435166
NCR3	natural cytotoxicity triggering receptor 3	6	-37471702	259197	6p21.3	Hs.509513

18.2 GO: Biological Process

GO ID	genes present in chip	genes present in tissues	p-value	GO term
GO:0009607	857	53	2.01E-23	response to biotic stimulus
GO:0006952	751	50	2.30E-23	defense response
GO:0006955	684	46	1.76E-21	immune response
GO:0009613	458	31	2.55E-14	response to pest, pathogen or parasite
GO:0043207	463	31	3.43E-14	response to external biotic stimulus
GO:0050896	1578	56	1.60E-13	response to stimulus
GO:0050874	1531	48	2.00E-09	organismal physiological process
GO:0019835	14	6	1.40E-08	cytolysis
GO:0006968	87	11	1.87E-08	cellular defense response
GO:0006950	881	32	9.85E-08	response to stress
GO:0009611	341	19	1.15E-07	response to wounding
GO:0009605	963	32	7.53E-07	response to external stimulus
GO:0007154	2730	61	2.33E-06	cell communication
GO:0007165	2168	52	2.96E-06	signal transduction
GO:0007166	952	30	5.52E-06	cell surface receptor linked signal transduction
GO:0008219	498	20	8.88E-06	cell death
GO:0016265	501	20	9.71E-06	death
GO:0006915	471	18	5.08E-05	apoptosis
GO:0012501	473	18	5.37E-05	programmed cell death
GO:0009615	58	6	0.000116996	response to virus
GO:0030097	87	7	0.000156734	hemopoiesis
GO:0048534	88	7	0.000168411	hemopoietic or lymphoid organ development
GO:0006922	2	2	0.000178011	cleavage of lamin
GO:0006923	2	2	0.000178011	cleavage of cytoskeletal proteins
GO:0050857	3	2	0.000529341	positive regulation of antigen receptor mediated signaling pathway
GO:0042531	3	2	0.000529341	positive regulation of tyrosine phosphorylation of STAT protein
GO:0046427	3	2	0.000529341	positive regulation of JAK-STAT cascade
GO:0030218	13	3	0.000608142	erythrocyte differentiation
GO:0006954	185	9	0.000821874	inflammatory response
GO:0050854	4	2	0.001049391	regulation of antigen receptor mediated signaling pathway
GO:0051282	4	2	0.001049391	regulation of sequestering of calcium ion
GO:0051283	4	2	0.001049391	negative regulation of sequestering of calcium ion
GO:0051209	4	2	0.001049391	release of sequestered calcium ion into cytoplasm
GO:0007242	879	23	0.001255428	intracellular signaling cascade
GO:0030099	37	4	0.001438407	myeloid cell differentiation
GO:0030217	18	3	0.001652304	T cell differentiation
GO:0050852	5	2	0.001733651	T cell receptor signaling pathway
GO:0051208	5	2	0.001733651	sequestering of calcium ion
GO:0006793	641	18	0.002100405	phosphorus metabolism
GO:0006796	641	18	0.002100405	phosphate metabolism
GO:0050851	6	2	0.0025777	antigen receptor-mediated signaling pathway
GO:0050870	21	3	0.002615402	positive regulation of T cell activation
GO:0042981	306	11	0.002615703	regulation of apoptosis

GO:0043067	308	11	0.002750909	regulation of programmed cell death
GO:0042110	45	4	0.002988754	T cell activation
GO:0042509	7	2	0.003577205	regulation of tyrosine phosphorylation of STAT protein
GO:0046425	7	2	0.003577205	regulation of JAK-STAT cascade
GO:0001906	8	2	0.004727919	cell killing
GO:0051238	8	2	0.004727919	sequestering of metal ion
GO:0008632	53	4	0.005411499	apoptotic program
GO:0009967	85	5	0.005552465	positive regulation of signal transduction
GO:0051251	28	3	0.006018081	positive regulation of lymphocyte activation
GO:0050731	9	2	0.006025679	positive regulation of peptidyl-tyrosine phosphorylation
GO:0006468	444	13	0.006355212	protein amino acid phosphorylation
GO:0050778	56	4	0.006582565	positive regulation of immune response
GO:0050867	29	3	0.006647656	positive regulation of cell activation
GO:0001934	10	2	0.007466403	positive regulation of protein amino acid phosphorylation
GO:0045764	10	2	0.007466403	positive regulation of amino acid metabolism
GO:0007260	10	2	0.007466403	tyrosine phosphorylation of STAT protein
GO:0051240	59	4	0.007910629	positive regulation of organismal physiological process
GO:0050863	31	3	0.008020644	regulation of T cell activation
GO:0006921	11	2	0.009046093	disassembly of cell structures during apoptosis
GO:0007155	518	14	0.009235484	cell adhesion
GO:0030098	34	3	0.01037195	lymphocyte differentiation
GO:0006470	99	5	0.010447388	protein amino acid dephosphorylation
GO:0042327	12	2	0.01076083	positive regulation of phosphorylation
GO:0045937	12	2	0.01076083	positive regulation of phosphate metabolism
GO:0009966	229	8	0.011690805	regulation of signal transduction
GO:0031342	1	1	0.013396811	negative regulation of cell killing
GO:0006926	1	1	0.013396811	programmed cell death, virus-infected cells
GO:0001911	1	1	0.013396811	negative regulation of immune cell mediated cytotoxicity
GO:0001913	1	1	0.013396811	T cell mediated cytotoxicity
GO:0001914	1	1	0.013396811	regulation of T cell mediated cytotoxicity
GO:0001915	1	1	0.013396811	negative regulation of T cell mediated cytotoxicity
GO:0045869	1	1	0.013396811	negative regulation of retroviral genome replication
GO:0045647	1	1	0.013396811	negative regulation of erythrocyte differentiation
GO:0050853	1	1	0.013396811	B cell receptor signaling pathway
GO:0007423	1	1	0.013396811	sensory organ development
GO:0051103	1	1	0.013396811	DNA ligation during DNA repair
GO:0000738	1	1	0.013396811	DNA catabolism, exonucleolytic
GO:0006288	1	1	0.013396811	base-excision repair, DNA ligation
GO:0006882	1	1	0.013396811	zinc ion homeostasis
GO:0009725	14	2	0.01458016	response to hormone stimulus
GO:0016311	110	5	0.015929204	dephosphorylation
GO:0050730	15	2	0.016677302	regulation of peptidyl-tyrosine phosphorylation
GO:0051249	41	3	0.017270116	regulation of lymphocyte activation
GO:0001932	16	2	0.018894586	regulation of protein amino acid phosphorylation
GO:0050776	77	4	0.019587709	regulation of immune response
GO:0050865	43	3	0.019612487	regulation of cell activation
GO:0006928	205	7	0.020082495	cell motility

GO:0040011	205	7	0.020082495	locomotion
GO:0006521	17	2	0.021228472	regulation of amino acid metabolism
GO:0007626	209	7	0.022060822	locomotory behavior
GO:0007229	45	3	0.022121776	integrin-mediated signaling pathway
GO:0016310	522	13	0.022237847	phosphorylation
GO:0046649	81	4	0.023126292	lymphocyte activation
GO:0018108	19	2	0.026232251	peptidyl-tyrosine phosphorylation
GO:0031341	2	1	0.026615612	regulation of cell killing
GO:0006927	2	1	0.026615612	programmed cell death, transformed cells
GO:0001910	2	1	0.026615612	regulation of immune cell mediated cytotoxicity
GO:0001907	2	1	0.026615612	killing of host cells
GO:0048247	2	1	0.026615612	lymphocyte chemotaxis
GO:0045885	2	1	0.026615612	positive regulation of survival gene product activity
GO:0019836	2	1	0.026615612	hemolysis
GO:0050862	2	1	0.026615612	positive regulation of T cell receptor signaling pathway
GO:0044003	2	1	0.026615612	modification of host morphology or physiology
GO:0044004	2	1	0.026615612	disruption of host cells
GO:0045091	2	1	0.026615612	regulation of retroviral genome replication
GO:0045059	2	1	0.026615612	positive thymic T cell selection
GO:0045071	2	1	0.026615612	negative regulation of viral genome replication
GO:0001897	2	1	0.026615612	cytolysis of host cells
GO:0007186	491	12	0.031347985	G-protein coupled receptor protein signaling pathway
GO:0019221	21	2	0.031661737	cytokine and chemokine mediated signaling pathway
GO:0019883	21	2	0.031661737	antigen presentation, endogenous antigen
GO:0018212	21	2	0.031661737	peptidyl-tyrosine modification
GO:0019885	22	2	0.034528011	antigen processing, endogenous antigen via MHC class I
GO:0048525	3	1	0.039658747	negative regulation of viral life cycle
GO:0045646	3	1	0.039658747	regulation of erythrocyte differentiation
GO:0050856	3	1	0.039658747	regulation of T cell receptor signaling pathway
GO:0050680	3	1	0.039658747	negative regulation of epithelial cell proliferation
GO:0042771	3	1	0.039658747	DNA damage response, signal transduction by p53 class mediator resulting in induction of apoptosis
GO:0006266	3	1	0.039658747	DNA ligation
GO:0016998	3	1	0.039658747	cell wall catabolism
GO:0045090	3	1	0.039658747	retroviral genome replication
GO:0045061	3	1	0.039658747	thymic T cell selection
GO:0006917	140	5	0.03969768	induction of apoptosis
GO:0012502	140	5	0.03969768	induction of programmed cell death
GO:0045321	97	4	0.040993858	immune cell activation
GO:0001775	98	4	0.042311701	cell activation
GO:0042325	25	2	0.04369563	regulation of phosphorylation
GO:0051235	25	2	0.04369563	maintenance of localization
GO:0051336	60	3	0.046226237	regulation of hydrolase activity
GO:0044419	26	2	0.046931104	interaction between organisms
GO:0030154	409	10	0.047402093	cell differentiation
GO:0006919	27	2	0.050251535	caspase activation
GO:0042541	4	1	0.052528532	hemoglobin biosynthesis
GO:0006464	1087	21	0.052630401	protein modification

GO:0019220	28	2	0.053654109	regulation of phosphate metabolism
GO:0051348	28	2	0.053654109	negative regulation of transferase activity
GO:0007259	28	2	0.053654109	JAK-STAT cascade
GO:0051174	28	2	0.053654109	regulation of phosphorus metabolism
GO:0006469	28	2	0.053654109	negative regulation of protein kinase activity
GO:0043085	107	4	0.055241627	positive regulation of enzyme activity
GO:0043065	155	5	0.056949249	positive regulation of apoptosis
GO:0007265	29	2	0.057136068	Ras protein signal transduction
GO:0043068	156	5	0.058231518	positive regulation of programmed cell death
GO:0006874	68	3	0.062756558	calcium ion homeostasis
GO:0006916	112	4	0.063249867	anti-apoptosis
GO:0043280	31	2	0.064327411	positive regulation of caspase activity
GO:0043281	31	2	0.064327411	regulation of caspase activity
GO:0050716	5	1	0.065227253	positive regulation of interleukin-1 secretion
GO:0050718	5	1	0.065227253	positive regulation of interleukin-1 beta secretion
GO:0050701	5	1	0.065227253	interleukin-1 secretion
GO:0050702	5	1	0.065227253	interleukin-1 beta secretion
GO:0050704	5	1	0.065227253	regulation of interleukin-1 secretion
GO:0050706	5	1	0.065227253	regulation of interleukin-1 beta secretion
GO:0030323	5	1	0.065227253	respiratory tube development
GO:0046146	5	1	0.065227253	tetrahydrobiopterin metabolism
GO:0020027	5	1	0.065227253	hemoglobin metabolism
GO:0006729	5	1	0.065227253	tetrahydrobiopterin biosynthesis
GO:0045069	5	1	0.065227253	regulation of viral genome replication
GO:0050790	214	6	0.067436035	regulation of enzyme activity
GO:0051345	32	2	0.068031567	positive regulation of hydrolase activity
GO:0007610	269	7	0.06925338	behavior
GO:0006690	33	2	0.071804656	icosanoid metabolism
GO:0042742	34	2	0.0756442	defense response to bacteria
GO:0001909	6	1	0.077757163	immune cell mediated cytotoxicity
GO:0035113	6	1	0.077757163	embryonic appendage morphogenesis
GO:0030326	6	1	0.077757163	embryonic limb morphogenesis
GO:0045058	6	1	0.077757163	T cell selection
GO:0008284	121	4	0.079114216	positive regulation of cell proliferation
GO:0006508	451	10	0.080128513	proteolysis and peptidolysis
GO:0044257	451	10	0.080128513	cellular protein catabolism
GO:0030333	36	2	0.083513014	antigen processing
GO:0019735	77	3	0.084155073	antimicrobial humoral response (sensu Vertebrata)
GO:0043283	2240	37	0.086539007	biopolymer metabolism
GO:0019730	78	3	0.086703736	antimicrobial humoral response
GO:0007204	37	2	0.087537593	elevation of cytoplasmic calcium ion concentration
GO:0051480	37	2	0.087537593	cytoplasmic calcium ion homeostasis
GO:0045884	7	1	0.090120489	regulation of survival gene product activity
GO:0045582	7	1	0.090120489	positive regulation of T cell differentiation
GO:0045621	7	1	0.090120489	positive regulation of lymphocyte differentiation
GO:0050792	7	1	0.090120489	regulation of viral life cycle
GO:0050715	7	1	0.090120489	positive regulation of cytokine secretion

GO:0035107	7	1	0.090120489	appendage morphogenesis
GO:0035108	7	1	0.090120489	limb morphogenesis
GO:0050707	7	1	0.090120489	regulation of cytokine secretion
GO:0050663	7	1	0.090120489	cytokine secretion
GO:0050673	7	1	0.090120489	epithelial cell proliferation
GO:0050678	7	1	0.090120489	regulation of epithelial cell proliferation
GO:0018298	7	1	0.090120489	protein-chromophore linkage
GO:0044267	2109	35	0.090439497	cellular protein metabolism
GO:0043412	1160	21	0.091041666	biopolymer modification
GO:0030163	463	10	0.091564842	protein catabolism
GO:0043066	128	4	0.092697717	negative regulation of apoptosis
GO:0043069	129	4	0.094724011	negative regulation of programmed cell death
GO:0043170	2405	39	0.09832364	macromolecule metabolism
GO:0042221	183	5	0.099020818	response to chemical substance
GO:0051242	411	9	0.099758924	positive regulation of cellular physiological process
GO:0019538	2129	35	0.100507774	protein metabolism
GO:0008283	473	10	0.101814189	cell proliferation
GO:0006558	8	1	0.102319428	L-phenylalanine metabolism
GO:0006559	8	1	0.102319428	L-phenylalanine catabolism
GO:0050832	8	1	0.102319428	defense response to fungi
GO:0043285	474	10	0.102875002	biopolymer catabolism
GO:0051239	133	4	0.103037342	regulation of organismal physiological process
GO:0019882	41	2	0.104184659	antigen presentation
GO:0051247	41	2	0.104184659	positive regulation of protein metabolism
GO:0007243	242	6	0.106057501	protein kinase cascade
GO:0030005	87	3	0.111052773	di-, tri-valent inorganic cation homeostasis
GO:0048522	482	10	0.111595224	positive regulation of cellular process
GO:0048598	9	1	0.114356147	embryonic morphogenesis
GO:0045638	9	1	0.114356147	negative regulation of myeloid cell differentiation
GO:0050714	9	1	0.114356147	positive regulation of protein secretion
GO:0042100	9	1	0.114356147	B cell proliferation
GO:0030330	9	1	0.114356147	DNA damage response, signal transduction by p53 class mediator
GO:0006268	9	1	0.114356147	DNA unwinding
GO:0043119	428	9	0.119883755	positive regulation of physiological process
GO:0006959	141	4	0.120626695	humoral immune response
GO:0043086	46	2	0.126070485	negative regulation of enzyme activity
GO:0006607	10	1	0.126232785	NLS-bearing substrate-nucleus import
GO:0019438	10	1	0.126232785	aromatic compound biosynthesis
GO:0009074	10	1	0.126232785	aromatic amino acid family catabolism
GO:0045580	10	1	0.126232785	regulation of T cell differentiation
GO:0008630	10	1	0.126232785	DNA damage response, signal transduction resulting in induction of apoptosis
GO:0009405	10	1	0.126232785	pathogenesis
GO:0042559	10	1	0.126232785	pteridine and derivative biosynthesis
GO:0006875	93	3	0.128581657	metal ion homeostasis
GO:0048519	688	13	0.130402638	negative regulation of biological process
GO:0044265	563	11	0.132998765	cellular macromolecule catabolism
GO:0048518	565	11	0.135330308	positive regulation of biological process

GO:0042558	11	1	0.137951454	pteridine and derivative metabolism
GO:0009620	11	1	0.137951454	response to fungi
GO:0006171	11	1	0.137951454	cAMP biosynthesis
GO:0044248	700	13	0.143175728	cellular catabolism
GO:0030003	98	3	0.143890921	cation homeostasis
GO:0018193	50	2	0.144289016	peptidyl-amino acid modification
GO:0042127	265	6	0.144795696	regulation of cell proliferation
GO:0009057	575	11	0.147321926	macromolecule catabolism
GO:0009617	51	2	0.148927273	response to bacteria
GO:0019370	12	1	0.149514238	leukotriene biosynthesis
GO:0045619	12	1	0.149514238	regulation of lymphocyte differentiation
GO:0050708	12	1	0.149514238	regulation of protein secretion
GO:0035295	12	1	0.149514238	tube development
GO:0007422	12	1	0.149514238	peripheral nervous system development
GO:0042102	12	1	0.149514238	positive regulation of T cell proliferation
GO:0006935	101	3	0.153354759	chemotaxis
GO:0042330	101	3	0.153354759	taxis
GO:0016064	103	3	0.159771596	humoral defense mechanism (sensu Vertebrata)
GO:0006873	103	3	0.159771596	cell ion homeostasis
GO:0044260	2298	36	0.16075937	cellular macromolecule metabolism
GO:0019439	13	1	0.160923193	aromatic compound catabolism
GO:0051047	13	1	0.160923193	positive regulation of secretion
GO:0046058	13	1	0.160923193	cAMP metabolism
GO:0030595	13	1	0.160923193	immune cell chemotaxis
GO:0045595	55	2	0.167765083	regulation of cell differentiation
GO:0006887	55	2	0.167765083	exocytosis
GO:0006691	14	1	0.17218035	leukotriene metabolism
GO:0050900	14	1	0.17218035	immune cell migration
GO:0006308	14	1	0.17218035	DNA catabolism
GO:0030224	14	1	0.17218035	monocyte differentiation
GO:0007167	165	4	0.180175693	enzyme linked receptor protein signaling pathway
GO:0045445	15	1	0.18328771	myoblast differentiation
GO:0043407	15	1	0.18328771	negative regulation of MAPK activity
GO:0042129	15	1	0.18328771	regulation of T cell proliferation
GO:0000188	15	1	0.18328771	inactivation of MAPK activity
GO:0050801	111	3	0.186213757	ion homeostasis
GO:0007264	227	5	0.188442965	small GTPase mediated signal transduction
GO:0006809	16	1	0.194247252	nitric oxide biosynthesis
GO:0045637	16	1	0.194247252	regulation of myeloid cell differentiation
GO:0006284	16	1	0.194247252	base-excision repair
GO:0046209	16	1	0.194247252	nitric oxide metabolism
GO:0007169	116	3	0.203290101	transmembrane receptor protein tyrosine kinase signaling pathway
GO:0006692	17	1	0.205060926	prostanoid metabolism
GO:0006693	17	1	0.205060926	prostaglandin metabolism
GO:0006904	17	1	0.205060926	vesicle docking during exocytosis
GO:0042098	17	1	0.205060926	T cell proliferation
GO:0045596	17	1	0.205060926	negative regulation of cell differentiation

GO:0006270	17	1	0.205060926	DNA replication initiation
GO:0009056	756	13	0.211271362	catabolism
GO:0043009	18	1	0.215730657	embryonic development (sensu Vertebrata)
GO:0019079	18	1	0.215730657	viral genome replication
GO:0031281	18	1	0.215730657	positive regulation of cyclase activity
GO:0048278	18	1	0.215730657	vesicle docking
GO:0045762	18	1	0.215730657	positive regulation of adenylate cyclase activity
GO:0009072	18	1	0.215730657	aromatic amino acid family metabolism
GO:0045597	18	1	0.215730657	positive regulation of cell differentiation
GO:0007190	18	1	0.215730657	adenylate cyclase activation
GO:0042133	18	1	0.215730657	neurotransmitter metabolism
GO:0050671	18	1	0.215730657	positive regulation of lymphocyte proliferation
GO:0051349	18	1	0.215730657	positive regulation of lyase activity
GO:0001701	18	1	0.215730657	embryonic development (sensu Mammalia)
GO:0051056	65	2	0.216333572	regulation of small GTPase mediated signal transduction
GO:0006261	65	2	0.216333572	DNA-dependent DNA replication
GO:0019725	122	3	0.224240313	cell homeostasis
GO:0006044	19	1	0.226258346	N-acetylglucosamine metabolism
GO:0042692	19	1	0.226258346	muscle cell differentiation
GO:0046916	20	1	0.236645869	transition metal ion homeostasis
GO:0008629	20	1	0.236645869	induction of apoptosis by intracellular signals
GO:0042493	20	1	0.236645869	response to drug
GO:0009190	20	1	0.236645869	cyclic nucleotide biosynthesis
GO:0007205	20	1	0.236645869	protein kinase C activation
GO:0006041	20	1	0.236645869	glucosamine metabolism
GO:0044403	20	1	0.236645869	symbiosis, mutualism through parasitism
GO:0044404	20	1	0.236645869	symbiotic interaction between host and other organism
GO:0048015	71	2	0.246066017	phosphoinositide-mediated signaling
GO:0007189	22	1	0.257007793	G-protein signaling, adenylate cyclase activating pathway
GO:0050670	22	1	0.257007793	regulation of lymphocyte proliferation
GO:0042770	22	1	0.257007793	DNA damage response, signal transduction
GO:0006461	194	4	0.262183878	protein complex assembly
GO:0006805	23	1	0.266985823	xenobiotic metabolism
GO:0006953	23	1	0.266985823	acute-phase response
GO:0008277	23	1	0.266985823	regulation of G-protein coupled receptor protein signaling pathway
GO:0046456	23	1	0.266985823	icosanoid biosynthesis
GO:0051046	24	1	0.276830947	regulation of secretion
GO:0006040	24	1	0.276830947	amino sugar metabolism
GO:0019058	26	1	0.296129471	viral infectious cycle
GO:0009306	26	1	0.296129471	protein secretion
GO:0009410	26	1	0.296129471	response to xenobiotic stimulus
GO:0007498	26	1	0.296129471	mesoderm development
GO:0006220	26	1	0.296129471	pyrimidine nucleotide metabolism
GO:0007411	27	1	0.305586314	axon guidance
GO:0009187	27	1	0.305586314	cyclic nucleotide metabolism
GO:0009792	27	1	0.305586314	embryonic development (sensu Metazoa)
GO:0043087	28	1	0.314917137	regulation of GTPase activity

GO:0046651	28	1	0.314917137	lymphocyte proliferation
GO:0016477	85	2	0.315677055	cell migration
GO:0009719	214	4	0.322369257	response to endogenous stimulus
GO:0001819	29	1	0.324123604	positive regulation of cytokine production
GO:0006941	29	1	0.324123604	striated muscle contraction
GO:0007179	29	1	0.324123604	transforming growth factor beta receptor signaling pathway
GO:0042113	30	1	0.333207358	B cell activation
GO:0051094	31	1	0.342170023	positive regulation of development
GO:0031279	32	1	0.351013199	regulation of cyclase activity
GO:0045761	32	1	0.351013199	regulation of adenylate cyclase activity
GO:0051339	32	1	0.351013199	regulation of lyase activity
GO:0042592	157	3	0.351653043	homeostasis
GO:0048523	640	10	0.354054923	negative regulation of cellular process
GO:0030155	33	1	0.359738466	regulation of cell adhesion
GO:0006401	33	1	0.359738466	RNA catabolism
GO:0016032	35	1	0.376841489	viral life cycle
GO:0006487	37	1	0.393491323	protein amino acid N-linked glycosylation
GO:0006821	38	1	0.401650028	chloride transport
GO:0007178	38	1	0.401650028	transmembrane receptor protein serine/threonine kinase signaling pathway
GO:0001817	39	1	0.409699877	regulation of cytokine production
GO:0007519	39	1	0.409699877	myogenesis
GO:0006511	105	2	0.412207588	ubiquitin-dependent protein catabolism
GO:0019941	105	2	0.412207588	modification-dependent protein catabolism
GO:0007409	40	1	0.417642312	axonogenesis
GO:0019932	176	3	0.420766458	second-messenger-mediated signaling
GO:0009628	318	5	0.423046156	response to abiotic stimulus
GO:0001505	41	1	0.425478753	regulation of neurotransmitter levels
GO:0001816	42	1	0.433210604	cytokine production
GO:0009063	42	1	0.433210604	amino acid catabolism
GO:0006520	181	3	0.438596382	amino acid metabolism
GO:0006979	44	1	0.448366054	response to oxidative stress
GO:0008154	45	1	0.455792368	actin polymerization and/or depolymerization
GO:0009310	45	1	0.455792368	amine catabolism
GO:0045859	116	2	0.462476158	regulation of protein kinase activity
GO:0031175	46	1	0.46311952	neurite morphogenesis
GO:0035023	46	1	0.46311952	regulation of Rho protein signal transduction
GO:0051338	117	2	0.46692358	regulation of transferase activity
GO:0006633	47	1	0.470348826	fatty acid biosynthesis
GO:0044270	47	1	0.470348826	nitrogen compound catabolism
GO:0006334	48	1	0.47748158	nucleosome assembly
GO:0006790	48	1	0.47748158	sulfur metabolism
GO:0043405	49	1	0.48451906	regulation of MAPK activity
GO:0051093	49	1	0.48451906	negative regulation of development
GO:0009308	269	4	0.488350795	amine metabolism
GO:0009968	50	1	0.491462531	negative regulation of signal transduction
GO:0006631	124	2	0.497440603	fatty acid metabolism
GO:0030036	126	2	0.505956288	actin cytoskeleton organization and biogenesis

GO:0048513	872	12	0.506138532	organ development
GO:0006606	53	1	0.511741251	protein-nucleus import
GO:0045860	53	1	0.511741251	positive regulation of protein kinase activity
GO:0045087	53	1	0.511741251	innate immune response
GO:0016053	54	1	0.518320972	organic acid biosynthesis
GO:0051347	54	1	0.518320972	positive regulation of transferase activity
GO:0051170	54	1	0.518320972	nuclear import
GO:0046394	54	1	0.518320972	carboxylic acid biosynthesis
GO:0051243	583	8	0.524876122	negative regulation of cellular physiological process
GO:0019752	359	5	0.52941201	carboxylic acid metabolism
GO:0046903	208	3	0.530772084	secretion
GO:0031497	56	1	0.531217744	chromatin assembly
GO:0007218	56	1	0.531217744	neuropeptide signaling pathway
GO:0007200	56	1	0.531217744	G-protein signaling, coupled to IP3 second messenger (phospholipase C activating)
GO:0006082	361	5	0.534402044	organic acid metabolism
GO:0015992	57	1	0.537537113	proton transport
GO:0006818	58	1	0.543771989	hydrogen transport
GO:0031325	136	2	0.547120956	positive regulation of cellular metabolism
GO:0007188	59	1	0.549923492	G-protein signaling, coupled to cAMP nucleotide second messenger
GO:0006807	291	4	0.550586126	nitrogen compound metabolism
GO:0019933	60	1	0.555992728	cAMP-mediated signaling
GO:0006519	216	3	0.556466889	amino acid and derivative metabolism
GO:0008285	139	2	0.5590003	negative regulation of cell proliferation
GO:0030029	139	2	0.5590003	actin filament-based process
GO:0006800	61	1	0.561980789	oxygen and reactive oxygen species metabolism
GO:0043118	602	8	0.562131991	negative regulation of physiological process
GO:0006260	140	2	0.562911114	DNA replication
GO:0007266	63	1	0.573717676	Rho protein signal transduction
GO:0009117	143	2	0.574495895	nucleotide metabolism
GO:0017038	64	1	0.579468614	protein import
GO:0006364	64	1	0.579468614	rRNA processing
GO:0045786	145	2	0.582095617	negative regulation of progression through cell cycle
GO:0009790	65	1	0.585142599	embryonic development
GO:0016072	66	1	0.590740652	rRNA metabolism
GO:0009893	149	2	0.596997731	positive regulation of metabolism
GO:0006816	68	1	0.601712982	calcium ion transport
GO:0043123	68	1	0.601712982	positive regulation of I-kappaB kinase/NF-kappaB cascade
GO:0000074	394	5	0.613036611	regulation of progression through cell cycle
GO:0043122	73	1	0.627883948	regulation of I-kappaB kinase/NF-kappaB cascade
GO:0006725	73	1	0.627883948	aromatic compound metabolism
GO:0045045	161	2	0.639323729	secretory pathway
GO:0007046	79	1	0.657046524	ribosome biogenesis
GO:0042254	80	1	0.661681548	ribosome biogenesis and assembly
GO:0050793	168	2	0.662376659	regulation of development
GO:0000165	81	1	0.66625444	MAPKKK cascade
GO:0051246	174	2	0.681191764	regulation of protein metabolism
GO:0007049	601	7	0.703065307	cell cycle

GO:0006333	91	1	0.708742565	chromatin assembly or disassembly
GO:0006486	92	1	0.712684199	protein amino acid glycosylation
GO:0043413	93	1	0.716572925	biopolymer glycosylation
GO:0007187	94	1	0.720409448	G-protein signaling, coupled to cyclic nucleotide second messenger
GO:0007517	94	1	0.720409448	muscle development
GO:0007028	96	1	0.727928654	cytoplasm organization and biogenesis
GO:0009101	96	1	0.727928654	glycoprotein biosynthesis
GO:0051169	96	1	0.727928654	nuclear transport
GO:0019935	98	1	0.735247267	cyclic-nucleotide-mediated signaling
GO:0015674	99	1	0.738833016	di-, tri-valent inorganic cation transport
GO:0007249	99	1	0.738833016	I-kappaB kinase/NF-kappaB cascade
GO:0006913	102	1	0.74930381	nucleocytoplasmic transport
GO:0009165	103	1	0.752700701	nucleotide biosynthesis
GO:0006974	200	2	0.753083353	response to DNA damage stimulus
GO:0015698	106	1	0.762619889	inorganic anion transport
GO:0007399	385	4	0.76449224	neurogenesis
GO:0016070	391	4	0.775007683	RNA metabolism
GO:0009100	111	1	0.778282471	glycoprotein metabolism
GO:0050789	2689	32	0.816781102	regulation of biological process
GO:0006820	128	1	0.824254531	anion transport
GO:0006936	132	1	0.833616203	muscle contraction
GO:0006357	240	2	0.83650743	regulation of transcription from RNA polymerase II promoter
GO:0006605	134	1	0.838109844	protein targeting
GO:0007067	135	1	0.840311317	mitosis
GO:0000087	137	1	0.844625582	M phase of mitotic cell cycle
GO:0005975	355	3	0.860323891	carbohydrate metabolism
GO:0016481	151	1	0.871754638	negative regulation of transcription
GO:0000004	465	4	0.876510204	biological process unknown
GO:0007267	465	4	0.876510204	cell-cell signaling
GO:0009888	158	1	0.883500751	histogenesis
GO:0045934	159	1	0.885089257	negative regulation of nucleobase, nucleoside, nucleotide and nucleic acid metabolism
GO:0050794	2488	28	0.885335172	regulation of cellular process
GO:0016337	172	1	0.903886021	cell-cell adhesion
GO:0000279	173	1	0.905198639	M phase
GO:0006281	174	1	0.906493476	DNA repair
GO:0008610	175	1	0.907770772	lipid biosynthesis
GO:0016192	296	2	0.910992577	vesicle-mediated transport
GO:0031324	183	1	0.917385288	negative regulation of cellular metabolism
GO:0007010	305	2	0.919504996	cytoskeleton organization and biogenesis
GO:0006325	186	1	0.920728518	establishment and/or maintenance of chromatin architecture
GO:0007268	192	1	0.927017663	synaptic transmission
GO:0006323	193	1	0.92801663	DNA packaging
GO:0006366	426	3	0.929657949	transcription from RNA polymerase II promoter
GO:0019226	198	1	0.932811645	transmission of nerve impulse
GO:0006396	326	2	0.936503307	RNA processing
GO:0000278	204	1	0.938149149	mitotic cell cycle
GO:0007275	1543	15	0.938256037	development

GO:0051244	2319	24	0.946836422	regulation of cellular physiological process
GO:0009892	217	1	0.948313972	negative regulation of metabolism
GO:0009987	8200	105	0.948724829	cellular process
GO:0006812	346	2	0.949506161	cation transport
GO:0045184	459	3	0.949689007	establishment of protein localization
GO:0007001	221	1	0.95109443	chromosome organization and biogenesis (sensu Eukaryota)
GO:0044238	4820	56	0.951837385	primary metabolism
GO:0015672	223	1	0.952428506	monovalent inorganic cation transport
GO:0006629	469	3	0.954630305	lipid metabolism
GO:0008104	474	3	0.956928939	protein localization
GO:0007582	7846	99	0.958464622	physiological process
GO:0051276	236	1	0.96026211	chromosome organization and biogenesis
GO:0006259	489	3	0.963192901	DNA metabolism
GO:0009887	807	6	0.965286447	organogenesis
GO:0044255	383	2	0.967200272	cellular lipid metabolism
GO:0050791	2407	24	0.968077054	regulation of physiological process
GO:0006512	255	1	0.969465707	ubiquitin cycle
GO:0006118	256	1	0.969886654	electron transport
GO:0006886	257	1	0.970301844	intracellular protein transport
GO:0006811	515	3	0.972082765	ion transport
GO:0044262	265	1	0.973425309	cellular carbohydrate metabolism
GO:0030001	269	1	0.974862565	metal ion transport
GO:0006412	407	2	0.975324591	protein biosynthesis
GO:0044249	763	5	0.979532101	cellular biosynthesis
GO:0015031	439	2	0.983205958	protein transport
GO:0044237	5026	56	0.985099674	cellular metabolism
GO:0006996	693	4	0.986007619	organelle organization and biogenesis
GO:0009059	464	2	0.987614761	macromolecule biosynthesis
GO:0006355	1359	10	0.991394128	regulation of transcription, DNA-dependent
GO:0008152	5262	58	0.991708196	metabolism
GO:0009058	859	5	0.992099782	biosynthesis
GO:0006351	1407	10	0.994344963	transcription, DNA-dependent
GO:0045449	1433	10	0.995520023	regulation of transcription
GO:0019219	1458	10	0.996431683	regulation of nucleobase, nucleoside, nucleotide and nucleic acid metabolism
GO:0009653	1051	6	0.996616718	morphogenesis
GO:0031323	1566	11	0.996746302	regulation of cellular metabolism
GO:0016043	842	4	0.99722779	cell organization and biogenesis
GO:0006350	1499	10	0.997561068	transcription
GO:0006091	446	1	0.9979081	generation of precursor metabolites and energy
GO:0046907	448	1	0.997966649	intracellular transport
GO:0019222	1629	11	0.998177971	regulation of metabolism
GO:0050877	475	1	0.998614862	neurophysiological process
GO:0051179	1857	12	0.999475035	localization
GO:0006139	2328	16	0.999772742	nucleobase, nucleoside, nucleotide and nucleic acid metabolism
GO:0050875	7018	77	0.999867301	cellular physiological process
GO:0051234	1841	10	0.999926793	establishment of localization
GO:0006810	1802	9	0.999966207	transport

GO:0008150	9032	121	1 biological_process

18.3 GO: Molecular Function

GO ID	genes present in chip	genes present in tissues	p-value	GO term
GO:0004872	1060	37	1.20597977498215e-08	receptor activity
GO:0004871	1837	49	1.57616115977391e-07	signal transducer activity
GO:0004888	694	23	2.89535272004166e-05	transmembrane receptor activity
GO:0030106	26	4	0.000335753083958531	MHC class I receptor activity
GO:0001948	3	2	0.000506478742908059	glycoprotein binding
GO:0004715	15	3	0.000890274637692793	non-membrane spanning protein tyrosine kinase activity
GO:0004713	161	8	0.00122271008359935	protein-tyrosine kinase activity
GO:0019955	65	5	0.00157037684472674	cytokine binding
GO:0004960	5	2	0.00165940508535134	thromboxane receptor activity
GO:0019864	5	2	0.00165940508535134	IgG binding
GO:0005529	103	6	0.00227623717577966	sugar binding
GO:0004725	75	5	0.00295787940028114	protein tyrosine phosphatase activity
GO:0004721	118	6	0.00447655246344005	phosphoprotein phosphatase activity
GO:0004955	8	2	0.00452801303796876	prostaglandin receptor activity
GO:0004726	8	2	0.00452801303796876	non-membrane spanning protein tyrosine phosphatase activity
GO:0016494	9	2	0.00577198939684793	C-X-C chemokine receptor activity
GO:0004953	10	2	0.00715340413203267	icosanoid receptor activity
GO:0004954	10	2	0.00715340413203267	prostanoid receptor activity
GO:0019958	10	2	0.00715340413203267	C-X-C chemokine binding
GO:0030246	181	7	0.00961560455769858	carbohydrate binding
GO:0042803	64	4	0.00973966554388911	protein homodimerization activity
GO:0019865	12	2	0.0103135974112175	immunoglobulin binding
GO:0008656	12	2	0.0103135974112175	caspase activator activity
GO:0008310	1	1	0.0131013520595317	single-stranded DNA specific 3'-5' exodeoxyribonuclease activity
GO:0004956	1	1	0.0131013520595317	prostaglandin D receptor activity
GO:0047017	1	1	0.0131013520595317	prostaglandin-F synthase activity
GO:0008859	1	1	0.0131013520595317	exoribonuclease II activity
GO:0050528	1	1	0.0131013520595317	acyloxyacyl hydrolase activity
GO:0003976	1	1	0.0131013520595317	UDP-N-acetylglucosamine-lysosomal-enzyme N-acetylglucosaminephosphotransferase activity
GO:0003933	1	1	0.0131013520595317	GTP cyclohydrolase activity
GO:0003934	1	1	0.0131013520595317	GTP cyclohydrolase I activity
GO:0042610	1	1	0.0131013520595317	CD8 receptor binding
GO:0004137	1	1	0.0131013520595317	deoxycytidine kinase activity
GO:0004277	1	1	0.0131013520595317	granzyme A activity
GO:0004278	1	1	0.0131013520595317	granzyme B activity
GO:0004218	1	1	0.0131013520595317	cathepsin S activity
GO:0004175	288	9	0.0132642482120359	endopeptidase activity
GO:0030693	14	2	0.0139794173762414	caspase activity
GO:0016505	14	2	0.0139794173762414	apoptotic protease activator activity
GO:0016504	15	2	0.0159931316437104	protease activator activity
GO:0042802	158	6	0.0175149603002396	protein self binding
GO:0004197	80	4	0.0206793092832661	cysteine-type endopeptidase activity
GO:0016773	532	13	0.0218382758376541	phosphotransferase activity, alcohol group as acceptor

GO:0005515	3709	60	0.0228248927190183	protein binding
GO:0016791	171	6	0.0247561697541245	phosphoric monoester hydrolase activity
GO:0043028	19	2	0.0251747640079115	caspase regulator activity
GO:0030676	2	1	0.0260324140084378	Rac guanyl-nucleotide exchange factor activity
GO:0008330	2	1	0.0260324140084378	protein tyrosine/threonine phosphatase activity
GO:0004920	2	1	0.0260324140084378	interleukin-10 receptor activity
GO:0008297	2	1	0.0260324140084378	single-stranded DNA specific exodeoxyribonuclease activity
GO:0047026	2	1	0.0260324140084378	3-alpha-hydroxysteroid dehydrogenase (A-specific) activity
GO:0019969	2	1	0.0260324140084378	interleukin-10 binding
GO:0051117	2	1	0.0260324140084378	ATPase binding
GO:0042609	2	1	0.0260324140084378	CD4 receptor binding
GO:0003690	20	2	0.0277356883105285	double-stranded DNA binding
GO:0016301	609	14	0.0279765995318530	kinase activity
GO:0016814	21	2	0.0303965727373075	hydrolase activity, acting on carbon-nitrogen (but not peptide) bonds, in cyclic amidines
GO:0004672	447	11	0.0323501668046565	protein kinase activity
GO:0048503	92	4	0.0324499102581874	GPI anchor binding
GO:0001637	22	2	0.0331544033577239	G-protein chemoattractant receptor activity
GO:0004950	22	2	0.0331544033577239	chemokine receptor activity
GO:0030234	509	12	0.034480017872851	enzyme regulator activity
GO:0008047	188	6	0.0369276385979698	enzyme activator activity
GO:0003779	189	6	0.0377445318723556	actin binding
GO:0005128	3	1	0.0387953816412023	erythropoietin receptor binding
GO:0008296	3	1	0.0387953816412023	3'-5'-exodeoxyribonuclease activity
GO:0004911	3	1	0.0387953816412023	interleukin-2 receptor activity
GO:0003796	3	1	0.0387953816412023	lysozyme activity
GO:0047115	3	1	0.0387953816412023	trans-1,2-dihydrobenzene-1,2-diol dehydrogenase activity
GO:0019976	3	1	0.0387953816412023	interleukin-2 binding
GO:0042608	3	1	0.0387953816412023	T cell receptor binding
GO:0019956	24	2	0.0389491499221218	chemokine binding
GO:0008234	98	4	0.0395466361282665	cysteine-type peptidase activity
GO:0016772	708	15	0.0433181636733625	transferase activity, transferring phosphorus-containing groups
GO:0004907	26	2	0.0450970118030617	interleukin receptor activity
GO:0003725	26	2	0.0450970118030617	double-stranded RNA binding
GO:0004252	105	4	0.0488608575227027	serine-type endopeptidase activity
GO:0001517	4	1	0.0513924226660344	N-acetylglucosamine 6-O-sulfotransferase activity
GO:0019136	4	1	0.0513924226660344	deoxynucleoside kinase activity
GO:0019238	4	1	0.0513924226660344	cyclohydrolase activity
GO:0003823	30	2	0.0583649932547405	antigen binding
GO:0019965	30	2	0.0583649932547405	interleukin binding
GO:0008301	5	1	0.0638256770612056	DNA bending activity
GO:0004957	5	1	0.0638256770612056	prostaglandin E receptor activity
GO:0005521	5	1	0.0638256770612056	lamin binding
GO:0005522	5	1	0.0638256770612056	profilin binding
GO:0004465	5	1	0.0638256770612056	lipoprotein lipase activity
GO:0004126	5	1	0.0638256770612056	cytidine deaminase activity
GO:0005088	5	1	0.0638256770612056	Ras guanyl-nucleotide exchange factor activity
GO:0008236	117	4	0.0674014596422667	serine-type peptidase activity

GO:0046983	170	5	0.072415505493972	protein dimerization activity
GO:0008092	279	7	0.073623066583633	cytoskeletal protein binding
GO:0005031	6	1	0.0760972574269098	tumor necrosis factor receptor activity
GO:0004303	6	1	0.0760972574269098	estradiol 17-beta-dehydrogenase activity
GO:0001584	282	7	0.0769609599114925	rhodopsin-like receptor activity
GO:0008233	399	9	0.0782131315560971	peptidase activity
GO:0042578	228	6	0.0788582632364286	phosphoric ester hydrolase activity
GO:0004930	349	8	0.087411378104155	G-protein coupled receptor activity
GO:0005035	7	1	0.0882092493326913	death receptor activity
GO:0004529	7	1	0.0882092493326913	exodeoxyribonuclease activity
GO:0001619	7	1	0.0882092493326913	lysosphingolipid and lysophosphatidic acid receptor activity
GO:0045125	7	1	0.0882092493326913	bioactive lipid receptor activity
GO:0043120	7	1	0.0882092493326913	tumor necrosis factor binding
GO:0016895	7	1	0.0882092493326913	exodeoxyribonuclease activity, producing 5'-phosphomonoesters
GO:0035091	132	4	0.0949801692386123	phosphoinositide binding
GO:0008289	298	7	0.0962454759811143	lipid binding
GO:0017017	8	1	0.100163711660510	MAP kinase phosphatase activity
GO:0016780	8	1	0.100163711660510	phosphotransferase activity, for other substituted phosphate groups
GO:0019992	42	2	0.104532017838896	diacylglycerol binding
GO:0004016	9	1	0.111962676943467	adenylate cyclase activity
GO:0016787	1424	24	0.112677103668741	hydrolase activity
GO:0005085	93	3	0.122536398164822	guanyl-nucleotide exchange factor activity
GO:0001565	10	1	0.123608151700294	phorbol ester receptor activity
GO:0004697	10	1	0.123608151700294	protein kinase C activity
GO:0000175	10	1	0.123608151700294	3'-5'-exoribonuclease activity
GO:0004033	10	1	0.123608151700294	aldo-keto reductase activity
GO:0004716	10	1	0.123608151700294	receptor signaling protein tyrosine kinase activity
GO:0004896	48	2	0.130336874682458	hematopoietin/interferon-class (D200-domain) cytokine receptor activity
GO:0005524	970	17	0.130818169013632	ATP binding
GO:0019838	49	2	0.134771683475761	growth factor binding
GO:0004532	11	1	0.135102116765602	exoribonuclease activity
GO:0019206	11	1	0.135102116765602	nucleoside kinase activity
GO:0016896	11	1	0.135102116765602	exoribonuclease activity, producing 5'-phosphomonoesters
GO:0017124	12	1	0.146446527616002	SH3 domain binding
GO:0016493	12	1	0.146446527616002	C-C chemokine receptor activity
GO:0019957	12	1	0.146446527616002	C-C chemokine binding
GO:0004143	12	1	0.146446527616002	diacylglycerol kinase activity
GO:0019904	52	2	0.14827200871227	protein domain specific binding
GO:0005102	528	10	0.153898204442623	receptor binding
GO:0019901	54	2	0.157419672717270	protein kinase binding
GO:0015026	13	1	0.157643314692085	coreceptor activity
GO:0030695	219	5	0.159908478287536	GTPase regulator activity
GO:0042169	14	1	0.168694383716381	SH2 domain binding
GO:0016628	14	1	0.168694383716381	oxidoreductase activity, acting on the CH-CH group of donors, NAD or NADP as acceptor
GO:0000146	15	1	0.179601616007286	microfilament motor activity
GO:0016229	15	1	0.179601616007286	steroid dehydrogenase activity
GO:0016564	59	2	0.180718273511284	transcriptional repressor activity

GO:0005543	169	4	0.180975455717139	phospholipid binding
GO:0019900	60	2	0.185440460890017	kinase binding
GO:0030554	1028	17	0.186788867380324	adenyl nucleotide binding
GO:0005247	16	1	0.19036686878905	voltage-gated chloride channel activity
GO:0008601	16	1	0.19036686878905	protein phosphatase type 2A regulator activity
GO:0017076	1248	20	0.197397061174459	purine nucleotide binding
GO:0016796	17	1	0.200991975497855	exonuclease activity, active with either ribo- or deoxyribonucleic acids and producing 5'-phosphomonoesters
GO:0019239	18	1	0.211478746084034	deaminase activity
GO:0016849	18	1	0.211478746084034	phosphorus-oxygen lyase activity
GO:0004298	18	1	0.211478746084034	threonine endopeptidase activity
GO:0005125	181	4	0.213149807360981	cytokine activity
GO:0005001	19	1	0.221828967310485	transmembrane receptor protein tyrosine phosphatase activity
GO:0004869	19	1	0.221828967310485	cysteine protease inhibitor activity
GO:0019198	19	1	0.221828967310485	transmembrane receptor protein phosphatase activity
GO:0016788	446	8	0.229875719393641	hydrolase activity, acting on ester bonds
GO:0001614	20	1	0.232044403047326	purinergic nucleotide receptor activity
GO:0001608	20	1	0.232044403047326	nucleotide receptor activity, G-protein coupled
GO:0016502	20	1	0.232044403047326	nucleotide receptor activity
GO:0009975	20	1	0.232044403047326	cyclase activity
GO:0045028	20	1	0.232044403047326	purinergic nucleotide receptor activity, G-protein coupled
GO:0000287	251	5	0.232659582293187	magnesium ion binding
GO:0016810	72	2	0.243149746095714	hydrolase activity, acting on carbon-nitrogen (but not peptide) bonds
GO:0005057	132	3	0.249538295941151	receptor signaling protein activity
GO:0008408	24	1	0.271592783443757	3'-5' exonuclease activity
GO:0004860	25	1	0.281159968698515	protein kinase inhibitor activity
GO:0004536	25	1	0.281159968698515	deoxyribonuclease activity
GO:0005178	26	1	0.290602486974333	integrin binding
GO:0008138	26	1	0.290602486974333	protein tyrosine/serine/threonine phosphatase activity
GO:0005083	144	3	0.292187884168686	small GTPase regulator activity
GO:0004722	27	1	0.299921949835943	protein serine/threonine phosphatase activity
GO:0008022	28	1	0.309119948182643	protein C-terminus binding
GO:0003697	28	1	0.309119948182643	single-stranded DNA binding
GO:0019899	151	3	0.317326980703568	enzyme binding
GO:0019210	29	1	0.318198052511158	kinase inhibitor activity
GO:0000166	1432	21	0.322313711687526	nucleotide binding
GO:0008146	32	1	0.344728405743568	sulfotransferase activity
GO:0001653	94	2	0.349672715157976	peptide receptor activity
GO:0008528	94	2	0.349672715157976	peptide receptor activity, G-protein coupled
GO:0008307	33	1	0.353342239930344	structural constituent of muscle
GO:0019205	34	1	0.361843735513927	nucleobase, nucleoside, nucleotide kinase activity
GO:0008094	34	1	0.361843735513927	DNA-dependent ATPase activity
GO:0019888	35	1	0.37023434591266	protein phosphatase regulator activity
GO:0004866	99	2	0.373345803295286	endopeptidase inhibitor activity
GO:0030414	100	2	0.378040539915591	protease inhibitor activity
GO:0019208	36	1	0.378515505891718	phosphatase regulator activity
GO:0016782	36	1	0.378515505891718	transferase activity, transferring sulfur-containing groups
GO:0016627	37	1	0.386688631800559	oxidoreductase activity, acting on the CH-CH group of donors

GO:0005126	38	1	0.394755121807390	hematopoietin/interferon-class (D200-domain) cytokine receptor binding
GO:0008009	40	1	0.410573697267654	chemokine activity
GO:0004527	40	1	0.410573697267654	exonuclease activity
GO:0042379	40	1	0.410573697267654	chemokine receptor binding
GO:0001664	42	1	0.42598206271724	G-protein-coupled receptor binding
GO:0005516	112	2	0.43314381736182	calmodulin binding
GO:0005254	43	1	0.433535725435458	chloride channel activity
GO:0005089	44	1	0.440990772215892	Rho guanyl-nucleotide exchange factor activity
GO:0004857	187	3	0.445179757284355	enzyme inhibitor activity
GO:0004540	45	1	0.448348480278756	ribonuclease activity
GO:0031404	45	1	0.448348480278756	chloride ion binding
GO:0043168	45	1	0.448348480278756	anion binding
GO:0005096	117	2	0.455342741536488	GTPase activator activity
GO:0005253	46	1	0.455610110435154	anion channel activity
GO:0019887	47	1	0.462776907296197	protein kinase regulator activity
GO:0016740	1169	16	0.466361914288746	transferase activity
GO:0003924	121	2	0.472743685412574	GTPase activity
GO:0042277	127	2	0.498215402816209	peptide binding
GO:0019207	58	1	0.535693025022859	kinase regulator activity
GO:0004553	59	1	0.541813279359265	hydrolase activity, hydrolyzing O-glycosyl compounds
GO:0016298	59	1	0.541813279359265	lipase activity
GO:0016563	62	1	0.559697959945225	transcriptional activator activity
GO:0008083	144	2	0.566014418184029	growth factor activity
GO:0004867	64	1	0.571234562432702	serine-type endopeptidase inhibitor activity
GO:0005525	226	3	0.572030078085314	GTP binding
GO:0019001	235	3	0.59870516076632	guanyl nucleotide binding
GO:0030674	69	1	0.598780778892728	protein binding, bridging
GO:0005488	7228	94	0.605537542778217	binding
GO:0005509	646	8	0.617618655838149	calcium ion binding
GO:0004674	326	4	0.62313289334156	protein serine/threonine kinase activity
GO:0005539	77	1	0.639247807609984	glycosaminoglycan binding
GO:0016798	77	1	0.639247807609984	hydrolase activity, acting on glycosyl bonds
GO:0016616	77	1	0.639247807609984	oxidoreductase activity, acting on the CH-OH group of donors, NAD or NADP as acceptor
GO:0016789	80	1	0.653355696686563	carboxylic ester hydrolase activity
GO:0030247	80	1	0.653355696686563	polysaccharide binding
GO:0003824	3409	43	0.654962205498503	catalytic activity
GO:0003714	81	1	0.657935607680558	transcription corepressor activity
GO:0016614	83	1	0.666916112322674	oxidoreductase activity, acting on CH-OH group of donors
GO:0003723	436	5	0.682096810658876	RNA binding
GO:0001871	88	1	0.688357087968645	pattern binding
GO:0030145	92	1	0.704519328681539	manganese ion binding
GO:0008509	95	1	0.71609275475282	anion transporter activity
GO:0003774	98	1	0.727216356452191	motor activity
GO:0008134	293	3	0.74374146306601	transcription factor binding
GO:0004518	105	1	0.751518317752307	nuclease activity
GO:0015078	112	1	0.773671024267399	hydrogen ion transporter activity
GO:0017111	410	4	0.791399516248833	nucleoside-triphosphatase activity

GO:0015077	121	1	0.799296724114299	monovalent inorganic cation transporter activity
GO:0005244	124	1	0.80718177761975	voltage-gated ion channel activity
GO:0016829	127	1	0.814759431439006	lyase activity
GO:0016462	430	4	0.82123963090852	pyrophosphatase activity
GO:0016818	432	4	0.824023347285159	hydrolase activity, acting on acid anhydrides, in phosphorus-containing anhydrides
GO:0016817	433	4	0.825401904651776	hydrolase activity, acting on acid anhydrides
GO:0016887	255	2	0.851830216623485	ATPase activity
GO:0005198	488	4	0.888590245436237	structural molecule activity
GO:0003702	197	1	0.927579214900232	RNA polymerase II transcription factor activity
GO:0003700	649	5	0.933810300349659	transcription factor activity
GO:0030528	963	8	0.94486722747587	transcription regulator activity
GO:0016874	221	1	0.94760224926697	ligase activity
GO:0042623	229	1	0.952969297323054	ATPase activity, coupled
GO:0003712	230	1	0.95360061581904	transcription cofactor activity
GO:0005216	252	1	0.96554693291736	ion channel activity
GO:0005554	522	3	0.970577835994722	molecular function unknown
GO:0015268	274	1	0.974435639585696	alpha-type channel activity
GO:0015267	284	1	0.977683472633049	channel or pore class transporter activity
GO:0008270	1287	10	0.979943965147206	zinc ion binding
GO:0043167	2556	23	0.98953492722729	ion binding
GO:0003677	1384	10	0.990827051992036	DNA binding
GO:0015075	523	2	0.993071992071268	ion transporter activity
GO:0003676	1990	16	0.993109673684514	nucleic acid binding
GO:0043169	2313	19	0.995272002161825	cation binding
GO:0046872	2495	21	0.995372168431018	metal ion binding
GO:0046914	1596	11	0.99650782694905	transition metal ion binding
GO:0008324	421	1	0.996582671549643	cation transporter activity
GO:0016491	509	1	0.998991457820187	oxidoreductase activity
GO:0005215	1030	2	0.99999069332367	transporter activity
GO:0003674	9541	125	1	molecular_function

18.4 GO: Cellular Component

GO ID	genes present in chip	genes present in tissues	p-value	GO term
GO:0016020	3514	66	0.000141424411676253	membrane
GO:0031224	2681	53	0.000357402862632838	intrinsic to membrane
GO:0005886	1472	34	0.000486414694711181	plasma membrane
GO:0001772	29	4	0.000529996228693737	immunological synapse
GO:0016021	2674	52	0.000661691819835844	integral to membrane
GO:0043235	58	5	0.000962869089078454	receptor complex
GO:0008305	24	3	0.00370350700865086	integrin complex
GO:0031226	1062	24	0.00538172570914386	intrinsic to plasma membrane
GO:0042101	10	2	0.00725193476584834	T cell receptor complex
GO:0005887	1057	23	0.0101439982906152	integral to plasma membrane
GO:0031229	4	1	0.0517688045257108	intrinsic to nuclear inner membrane
GO:0005639	4	1	0.0517688045257108	integral to nuclear inner membrane
GO:0046658	5	1	0.0642903331118414	anchored to plasma membrane
GO:0031225	5	1	0.0642903331118414	anchored to membrane
GO:0005577	5	1	0.0642903331118414	fibrinogen complex
GO:0016461	5	1	0.0642903331118414	unconventional myosin
GO:0000242	6	1	0.0766479445605501	pericentriolar material
GO:0005637	7	1	0.0888437659393133	nuclear inner membrane
GO:0005925	8	1	0.100879896957032	focal adhesion
GO:0016605	8	1	0.100879896957032	PML body
GO:0000502	41	2	0.101606738208315	proteasome complex (sensu Eukaryota)
GO:0005765	9	1	0.112758410312797	lysosomal membrane
GO:0005924	9	1	0.112758410312797	cell-substrate adherens junction
GO:0005802	11	1	0.136050741847518	Golgi trans face
GO:0030055	11	1	0.136050741847518	cell-matrix junction
GO:0009897	11	1	0.136050741847518	external side of plasma membrane
GO:0005769	13	1	0.158736814916981	early endosome
GO:0005764	107	3	0.167606505093090	lysosome
GO:0000323	107	3	0.167606505093090	lytic vacuole
GO:0045121	14	1	0.169857408968403	lipid raft
GO:0000159	14	1	0.169857408968403	protein phosphatase type 2A complex
GO:0005863	15	1	0.180832273328557	striated muscle thick filament
GO:0005665	16	1	0.191663301029849	DNA-directed RNA polymerase II, core complex
GO:0016604	16	1	0.191663301029849	nuclear body
GO:0005773	120	3	0.210982523700631	vacuole
GO:0005839	18	1	0.21290129702748	proteasome core complex (sensu Eukaryota)
GO:0005912	18	1	0.21290129702748	adherens junction
GO:0005615	385	7	0.245705652321338	extracellular space
GO:0048471	22	1	0.253731874978317	perinuclear region
GO:0008287	23	1	0.263607042274019	protein serine/threonine phosphatase complex
GO:0000793	23	1	0.263607042274019	condensed chromosome
GO:0030880	23	1	0.263607042274019	RNA polymerase complex
GO:0005856	612	10	0.287840655489557	cytoskeleton

GO:0008021	27	1	0.301829006392805	synaptic vesicle
GO:0005938	28	1	0.31107308226635	cell cortex
GO:0030017	28	1	0.31107308226635	sarcomere
GO:0005623	7802	105	0.325582336891818	cell
GO:0031300	90	2	0.333780504008518	intrinsic to organelle membrane
GO:0030016	32	1	0.346851712637151	myofibril
GO:0031228	35	1	0.372471206381351	intrinsic to Golgi membrane
GO:0043292	40	1	0.412975080062088	contractile fiber
GO:0000228	42	1	0.428441168680401	nuclear chromosome
GO:0005813	43	1	0.436022053703138	centrosome
GO:0016459	43	1	0.436022053703138	myosin
GO:0015629	183	3	0.436161148652474	actin cytoskeleton
GO:0005815	49	1	0.479458997123729	microtubule organizing center
GO:0016327	50	1	0.486368833445096	apicolateral plasma membrane
GO:0000922	51	1	0.493187740279526	spindle pole
GO:0005941	57	1	0.532257022687259	unlocalized protein complex
GO:0016591	58	1	0.538471789518687	DNA-directed RNA polymerase II, holoenzyme
GO:0009986	61	1	0.556629197198617	cell surface
GO:0005654	221	3	0.5620751468166	nucleoplasm
GO:0005768	62	1	0.562522885575407	endosome
GO:0030136	65	1	0.579741999115223	clathrin-coated vesicle
GO:0000139	67	1	0.590846430621328	Golgi membrane
GO:0005819	68	1	0.596289080202328	spindle
GO:0005730	75	1	0.632429691675156	nucleolus
GO:0031301	76	1	0.63732376025131	integral to organelle membrane
GO:0043228	977	12	0.649288113925944	non-membrane-bound organelle
GO:0043232	977	12	0.649288113925944	intracellular non-membrane-bound organelle
GO:0030135	79	1	0.65162174349144	coated vesicle
GO:0005635	83	1	0.66982004846731	nuclear membrane
GO:0019866	89	1	0.695365130430342	inner membrane
GO:0005829	276	3	0.711291071058232	cytosol
GO:0030054	93	1	0.711296649600429	cell junction
GO:0005794	195	2	0.73307143324926	Golgi apparatus
GO:0000785	100	1	0.737214301535865	chromatin
GO:0016023	122	1	0.804567929903032	cytoplasmic membrane-bound vesicle
GO:0031410	124	1	0.8097661466923	cytoplasmic vesicle
GO:0005576	949	10	0.817763260496773	extracellular region
GO:0005624	521	5	0.82580156268465	membrane fraction
GO:0012505	246	2	0.840758807248194	endomembrane system
GO:0005737	2530	28	0.890447556462788	cytoplasm
GO:0000267	698	6	0.907665393965088	cell fraction
GO:0005625	183	1	0.914365359275235	soluble fraction
GO:0008372	523	4	0.920970262612919	cellular component unknown
GO:0015630	191	1	0.923182483239137	microtubule cytoskeleton
GO:0005694	203	1	0.93474827488592	chromosome
GO:0031090	377	2	0.96258271583237	organelle membrane
GO:0005634	2704	27	0.971643569519047	nucleus

GO:0043234	1247	10	0.975373269650876	protein complex
GO:0005578	303	1	0.983402428605548	extracellular matrix (sensu Metazoa)
GO:0031012	304	1	0.983629465219295	extracellular matrix
GO:0005783	483	2	0.98937648172656	endoplasmic reticulum
GO:0005622	5419	56	0.998950797437111	intracellular
GO:0043226	4515	40	0.999930812259414	organelle
GO:0043229	4515	40	0.999930812259414	intracellular organelle
GO:0043227	3955	32	0.999968446028916	membrane-bound organelle
GO:0043231	3955	32	0.999968446028916	intracellular membrane-bound organelle
GO:0005575	8637	114	1	cellular_component

19. PB-CD19BCells

19.1 PB-CD19BCells selective genes (108 genes)

Symbol	Description	Chromosome	Chromosome Location	LocusLink	Cytoband	UniGene
BANK1	B-cell scaffold protein with ankyrin repeats 1	4	102930918	55024	4q24	Hs.480400
BIRC3	baculoviral IAP repeat-containing 3	11	101693403	330	11q22	Hs.127799
IGJ	immunoglobulin J polypeptide, linker protein for immunoglobulin alpha and mu polypeptides	4	-71740547	3512	4q21	Hs.643431
IGHA1	immunoglobulin heavy constant alpha 1	14	NA	3493	14q32.33	Hs.510635
IGKV1-39	immunoglobulin kappa variable 1-39	2	NA	28930	2p12	NA
CD69	CD69 molecule	12	-9796352	969	12p13-p12	Hs.208854
P2RY10	purinergic receptor P2Y, G-protein coupled, 10	X	78087484	27334	Xq21.1	Hs.296433
EVI2B	ecotropic viral integration site 2B	17	-26654918	2124	17q11.2	Hs.5509
POU2AF1	POU domain, class 2, associating factor 1	11	-110728189	5450	11q23.1	Hs.128180
C13orf18	chromosome 13 open reading frame 18	13	-45815100	80183	13q14.12	Hs.98117
MS4A1	membrane-spanning 4-domains, subfamily A, member 1	11	59979857	931	11q12	Hs.438040
PLEKHF2	pleckstrin homology domain containing, family F (with FYVE domain) member 2	8	96215213	79666	8q22.1	Hs.29724
CD79B	CD79b molecule, immunoglobulin-associated beta	17	-59359830	974	17q23	Hs.89575
CTA-246H3.1	NA	22	24043887	91353	22q11.23	Hs.567636
CD37	CD37 molecule	19	54530488	951	19q13.3	Hs.166556
PMAIP1	phorbol-12-myristate-13-acetate-induced protein 1	18	55718216	5366	18q21.32	Hs.96
PPBP	pro-platelet basic protein (chemokine (C-X-C motif) ligand 7)	4	-75071621	5473	4q12-q13	Hs.2164
TCL1A	T-cell leukemia/lymphoma 1A	14	-95246057	8115	14q32.1	Hs.2484
BLNK	B-cell linker	10	-97941452	29760	10q23.2-q23.33	Hs.444049
GPR18	G protein-coupled receptor 18	13	-98704972	2841	13q32	Hs.631765
LTB	lymphotoxin beta (TNF superfamily, member 3)	6	-37446700	4050	6p21.3	Hs.376208
PTPN6	protein tyrosine phosphatase, non-receptor type 6	12	6930703, 6926000	5777	12p13	Hs.63489
ISG20	interferon stimulated exonuclease gene 20kDa	15	86983042	3669	15q26	Hs.459265
CCR6	chemokine (C-C motif) receptor 6	6	167456246	1235	6q27	Hs.46468
HHEX	homeobox, hematopoietically expressed	10	94439679	3087	10q23.33	Hs.118651
BRDG1	NA	4	68107040	26228	4q13.2	Hs.435579
PLAC8	placenta-specific 8	4	-84230866	51316	4q21.22	Hs.546392
PTPRC	protein tyrosine phosphatase, receptor type, C	1	196874847	5788	1q31-q32	Hs.192039
CD19	CD19 molecule	16	28850760	930	16p11.2	Hs.632193
IGHM	immunoglobulin heavy constant mu	14	NA	3507	14q32.33	Hs.510635
OSBPL10	oxysterol binding protein-like 10	3	-31677321	114884	3p22.3	Hs.150122
CCR7	chemokine (C-C motif) receptor 7	17	-35963549	1236	17q12-q21.2	Hs.370036
SWAP70	NA	11	9642203	23075	11p15	Hs.153026

RASGRP2	RAS guanyl releasing protein 2 (calcium and DAG-regulated)	11	-64250959	10235	11q13	Hs.99491
CXCR4	chemokine (C-X-C motif) receptor 4	2	-136588389	7852	2q21	Hs.421986
P2RY14	purinergic receptor P2Y, G-protein coupled, 14	3	-152412596	9934	3q21-q25	Hs.2465
DCK	deoxycytidine kinase	4	72078255	1633	4q13.3-q21.1	Hs.709
COTL1	coactosin-like 1 (Dictyostelium)	16	-83156706	23406	16q24.1	Hs.289092
BLK	B lymphoid tyrosine kinase	8	11388929	640	8p23-p22	Hs.146591
DOCK2	dedicator of cytokinesis 2	5	168996870	1794	5q35.1	Hs.586174
LYN	v-yes-1 Yamaguchi sarcoma viral related oncogene homolog	8	56954925	4067	8q13	Hs.491767 Hs.614775
CD79A	CD79a molecule, immunoglobulin-associated alpha	19	47073029	973	19q13.2	Hs.631567
SELL	selectin L (lymphocyte adhesion molecule 1)	1	-167926433	6402	1q23-q25	Hs.82848
TNFAIP8	tumor necrosis factor, alpha-induced protein 8	5	118719628	25816	5q23.1	Hs.271955
IFT57	intraflagellar transport 57 homolog (Chlamydomonas)	3	-109362349	55081	3q13.12	Hs.412196
SNX2	sorting nexin 2	5	122138648	6643	5q23	Hs.134822
CD52	CD52 molecule	1	26516997	1043	1p36	Hs.276770
PSCDBP	pleckstrin homology, Sec7 and coiled-coil domains, binding protein	2	-157979377	9595	2q11.2	Hs.270
CLEC2D	C-type lectin domain family 2, member D	12	9713575	29121	12p13	Hs.268326
FAIM3	Fas apoptotic inhibitory molecule 3	1	-205144353	9214	1q32.1	Hs.58831
LOC646082	NA	22	NA	646082	22q11.23	NA
DPCR1	diffuse panbronchiolitis critical region 1	6	31027537, 2367969, 2166477	135656	6p21.33	Hs.631993
HLA-DRA	major histocompatibility complex, class II, DR alpha	6	32515624	3122	6p21.3	Hs.520048
RNASE6	ribonuclease, RNase A family, k6	14	20319049	6039	14q11.2	Hs.23262
RASGRP1	RAS guanyl releasing protein 1 (calcium and DAG-regulated)	15	-36567597	10125	15q15	Hs.591127
ALOX5	arachidonate 5-lipoxygenase	10	45189634	240	10q11.2	Hs.89499
GMFG	glia maturation factor, gamma	19	-44510841	9535	19q13.2	Hs.5210
IGHV3-9	immunoglobulin heavy variable 3-9	14	NA	28451	14q32.33	NA
MBD4	methyl-CpG binding domain protein 4	3	-130632482	8930	3q21-q22	Hs.35947
P2RX5	purinergic receptor P2X, ligand-gated ion channel, 5	17	-7063600	5026	17p13.3	Hs.408615
GCH1	GTP cyclohydrolase 1 (dopa-responsive dystonia)	14	-54378475	2643	14q22.1-q22.2	Hs.86724
MCM5	MCM5 minichromosome maintenance deficient 5, cell division cycle 46 (S. cerevisiae)	22	34126127	4174	22q13.1	Hs.517582
RUNX3	runt-related transcription factor 3	1	-25098589	864	1p36	Hs.170019
HLA-DMA	major histocompatibility complex, class II, DM alpha	6	-41424089	3108	6p21.3	Hs.351279
ALOX5AP	arachidonate 5-lipoxygenase-activating protein	13	30207668	241	13q12	Hs.507658
FCER2	Fc fragment of IgE, low affinity II, receptor for (CD23)	19	-7659662	2208	19p13.3	Hs.465778
IGHV3-23	immunoglobulin heavy variable 3-23	14	NA	28442	14q32.33	NA
OPN3	opsin 3 (encephalopsin, panopsin)	1	-239823075	23596	1q43	Hs.409081 Hs.534399
TNFRSF17	tumor necrosis factor receptor superfamily, member 17	16	11966464	608	16p13.1	Hs.2556
CD48	CD48 molecule	1	-158915161	962	1q21.3-q22	Hs.243564
PTPRCAP	protein tyrosine phosphatase, receptor type, C-associated protein	11	-66959556	5790	11q13.3	Hs.155975
ICAM3	intercellular adhesion molecule 3	19	-10305453	3385	19p13.3-p13.2	Hs.75516

RBM3	RNA binding motif (RNP1, RRM) protein 3	X	48317779	5935	Xp11.2	Hs.301404
RABGAP1L	RAB GTPase activating protein 1-like	1	173111306, 172395256	9910	1q24	Hs.585378 Hs.591475 Hs.615081
IRF8	interferon regulatory factor 8	16	84490274	3394	16q24.1	Hs.137427
IER5	immediate early response 5	1	179324260	51278	1q25.3	Hs.15725
RNF138	ring finger protein 138	18	27925834, 27926644	51444	18q12.1	Hs.302408 Hs.501040
PELI1	pellino homolog 1 (Drosophila)	2	-64173499	57162	2p13.3	Hs.7886
NCF4	neutrophil cytosolic factor 4, 40kDa	22	35586990	4689	22q13.1	Hs.474781
SYK	spleen tyrosine kinase	9	92603890	6850	9q22	Hs.371720
EAF2	ELL associated factor 2	3	123036723	55840	3q13.33	Hs.477325
HMGB2	high-mobility group box 2	4	-174489362	3148	4q31	Hs.434953
LRAP	NA	5	96241023	64167	5q15	Hs.591249
NUP88	nucleoporin 88kDa	17	-5230143	4927	17p13.2	Hs.584784
PSMC6	proteasome (prosome, macropain) 26S subunit, ATPase, 6	14	52243667	5706	14q22.1	Hs.156171 Hs.491779
PYCARD	PYD and CARD domain containing	16	-31120309	29108	16p12-p11.2	Hs.499094
HLA-DOB	major histocompatibility complex, class II, DO beta	6	-32888527	3112	6p21.3	Hs.1802
LRRFIP1	leucine rich repeat (in FLII) interacting protein 1	2	238265667	9208	2q37.3	Hs.471779
IFI16	interferon, gamma-inducible protein 16	1	157246330	3428	1q22	Hs.380250
BTG1	B-cell translocation gene 1, anti-proliferative	12	-91061033	694	12q22	Hs.255935 Hs.585087
HLA-DPA1	major histocompatibility complex, class II, DP alpha 1	6	-41773262	3113	6p21.3	Hs.347270
KIAA0746	NA	4	-25358146	23231	4p15.2	Hs.479384
FCRL2	Fc receptor-like 2	1	-155982149	79368	1q21	Hs.437393
VPREB3	pre-B lymphocyte gene 3	22	-22424932	29802	22q11.23|22q11	Hs.136713
RHOQ	ras homolog gene family, member Q	2	46623378	23433	2p21	Hs.643481
CCNB1IP1	cyclin B1 interacting protein 1	14	-39698736	57820	14q11.2	Hs.107003
PSMB10	proteasome (prosome, macropain) subunit, beta type, 10	16	-66525907	5699	16q22.1	Hs.9661
HECA	headcase homolog (Drosophila)	6	139497941	51696	6q23-q24	Hs.197644
IGKC	immunoglobulin kappa constant	2	NA	3514	2p12	Hs.449621
STK17B	serine/threonine kinase 17b (apoptosis-inducing)	2	-196710102	9262	2q32.3	Hs.88297
IGHV1-69	immunoglobulin heavy variable 1-69	14	NA	28461	14q32.32-q32.33	Hs.634941
TRAF5	TNF receptor-associated factor 5	1	209566801, 209566579, 209586328	7188	1q32	Hs.523930
SP140	SP140 nuclear body protein	2	230798689	11262	2q37.1	Hs.632549
PALM2-AKAP2	NA	9	111582409	445815	9q31-q33	Hs.591908
TRIM22	tripartite motif-containing 22	11	5667663	10346	11p15	Hs.501778 Hs.621411
KLF2	Kruppel-like factor 2 (lung)	19	16296650	10365	19p13.13-p13.11	Hs.107740
HLA-DQA1	major histocompatibility complex, class II, DQ alpha 1	6	32713160	3117	6p21.3	Hs.387679
TCF4	transcription factor 4	18	-51045966	6925	18q21.1	Hs.569908

19.2 GO: Biological Process

GO ID	genes present in chip	genes present in tissues	p-value	GO term
GO:0006952	751	41	5.36E-23	defense response
GO:0009607	857	42	8.16E-22	response to biotic stimulus
GO:0006955	684	35	2.45E-18	immune response
GO:0050896	1578	46	4.92E-15	response to stimulus
GO:0030333	36	10	5.15E-13	antigen processing
GO:0019882	41	9	8.27E-11	antigen presentation
GO:0050874	1531	37	3.30E-09	organismal physiological process
GO:0019884	11	5	2.57E-08	antigen presentation, exogenous antigen
GO:0019886	12	5	4.37E-08	antigen processing, exogenous antigen via MHC class II
GO:0009613	458	18	1.23E-07	response to pest, pathogen or parasite
GO:0043207	463	18	1.45E-07	response to external biotic stimulus
GO:0019885	22	5	1.35E-06	antigen processing, endogenous antigen via MHC class I
GO:0019883	21	4	3.52E-05	antigen presentation, endogenous antigen
GO:0006950	881	20	0.000103683	response to stress
GO:0006959	141	7	0.000300627	humoral immune response
GO:0042981	306	10	0.000467295	regulation of apoptosis
GO:0043067	308	10	0.000491865	regulation of programmed cell death
GO:0009605	963	19	0.000949846	response to external stimulus
GO:0006915	471	12	0.001159017	apoptosis
GO:0012501	473	12	0.001202174	programmed cell death
GO:0018298	7	2	0.001700531	protein-chromophore linkage
GO:0008219	498	12	0.001864721	cell death
GO:0016265	501	12	0.001961362	death
GO:0006935	101	5	0.00230636	chemotaxis
GO:0042330	101	5	0.00230636	taxis
GO:0042113	30	3	0.002540302	B cell activation
GO:0007165	2168	31	0.004381425	signal transduction
GO:0007204	37	3	0.004642336	elevation of cytoplasmic calcium ion concentration
GO:0051480	37	3	0.004642336	cytoplasmic calcium ion homeostasis
GO:0019370	12	2	0.005187356	leukotriene biosynthesis
GO:0019735	77	4	0.005395343	antimicrobial humoral response (sensu Vertebrata)
GO:0019730	78	4	0.005647819	antimicrobial humoral response
GO:0030595	13	2	0.006094099	immune cell chemotaxis
GO:0006691	14	2	0.00706758	leukotriene metabolism
GO:0050900	14	2	0.00706758	immune cell migration
GO:0031342	1	1	0.009189548	negative regulation of cell killing
GO:0001911	1	1	0.009189548	negative regulation of immune cell mediated cytotoxicity
GO:0001913	1	1	0.009189548	T cell mediated cytotoxicity
GO:0001914	1	1	0.009189548	regulation of T cell mediated cytotoxicity
GO:0001915	1	1	0.009189548	negative regulation of T cell mediated cytotoxicity
GO:0045603	1	1	0.009189548	positive regulation of endothelial cell differentiation
GO:0050853	1	1	0.009189548	B cell receptor signaling pathway
GO:0051103	1	1	0.009189548	DNA ligation during DNA repair

GO:0000738	1	1	0.009189548	DNA catabolism, exonucleolytic
GO:0006288	1	1	0.009189548	base-excision repair, DNA ligation
GO:0006917	140	5	0.009199724	induction of apoptosis
GO:0012502	140	5	0.009199724	induction of programmed cell death
GO:0006284	16	2	0.009209689	base-excision repair
GO:0009611	341	8	0.012790183	response to wounding
GO:0007154	2730	35	0.013566756	cell communication
GO:0043065	155	5	0.013858998	positive regulation of apoptosis
GO:0043068	156	5	0.014217028	positive regulation of programmed cell death
GO:0016064	103	4	0.014736631	humoral defense mechanism (sensu Vertebrata)
GO:0009615	58	3	0.016064117	response to virus
GO:0031341	2	1	0.018295657	regulation of cell killing
GO:0001910	2	1	0.018295657	regulation of immune cell mediated cytotoxicity
GO:0048247	2	1	0.018295657	lymphocyte chemotaxis
GO:0045663	2	1	0.018295657	positive regulation of myoblast differentiation
GO:0042752	2	1	0.018295657	regulation of circadian rhythm
GO:0046456	23	2	0.018627772	icosanoid biosynthesis
GO:0006874	68	3	0.024445483	calcium ion homeostasis
GO:0007166	952	15	0.025528048	cell surface receptor linked signal transduction
GO:0042221	183	5	0.02635921	response to chemical substance
GO:0045601	3	1	0.027319075	regulation of endothelial cell differentiation
GO:0050857	3	1	0.027319075	positive regulation of antigen receptor mediated signaling pathway
GO:0050680	3	1	0.027319075	negative regulation of epithelial cell proliferation
GO:0042771	3	1	0.027319075	DNA damage response, signal transduction by p53 class mediator resulting in induction of apoptosis
GO:0042531	3	1	0.027319075	positive regulation of tyrosine phosphorylation of STAT protein
GO:0046427	3	1	0.027319075	positive regulation of JAK-STAT cascade
GO:0006266	3	1	0.027319075	DNA ligation
GO:0006954	185	5	0.027459222	inflammatory response
GO:0008283	473	9	0.028989805	cell proliferation
GO:0045661	4	1	0.036260543	regulation of myoblast differentiation
GO:0050854	4	1	0.036260543	regulation of antigen receptor mediated signaling pathway
GO:0051282	4	1	0.036260543	regulation of sequestering of calcium ion
GO:0051283	4	1	0.036260543	negative regulation of sequestering of calcium ion
GO:0051209	4	1	0.036260543	release of sequestered calcium ion into cytoplasm
GO:0042541	4	1	0.036260543	hemoglobin biosynthesis
GO:0006690	33	2	0.036650571	icosanoid metabolism
GO:0006401	33	2	0.036650571	RNA catabolism
GO:0046649	81	3	0.038253316	lymphocyte activation
GO:0006928	205	5	0.040084696	cell motility
GO:0040011	205	5	0.040084696	locomotion
GO:0007626	209	5	0.042972738	locomotory behavior
GO:0016477	85	3	0.043154106	cell migration
GO:0009967	85	3	0.043154106	positive regulation of signal transduction
GO:0045446	5	1	0.045120797	endothelial cell differentiation
GO:0050716	5	1	0.045120797	positive regulation of interleukin-1 secretion
GO:0050718	5	1	0.045120797	positive regulation of interleukin-1 beta secretion
GO:0050701	5	1	0.045120797	interleukin-1 secretion

GO:0050702	5	1	0.045120797	interleukin-1 beta secretion
GO:0050704	5	1	0.045120797	regulation of interleukin-1 secretion
GO:0050706	5	1	0.045120797	regulation of interleukin-1 beta secretion
GO:0050852	5	1	0.045120797	T cell receptor signaling pathway
GO:0051208	5	1	0.045120797	sequestering of calcium ion
GO:0030323	5	1	0.045120797	respiratory tube development
GO:0046146	5	1	0.045120797	tetrahydrobiopterin metabolism
GO:0020027	5	1	0.045120797	hemoglobin metabolism
GO:0006729	5	1	0.045120797	tetrahydrobiopterin biosynthesis
GO:0030593	5	1	0.045120797	neutrophil chemotaxis
GO:0030099	37	2	0.045158027	myeloid cell differentiation
GO:0006968	87	3	0.045717167	cellular defense response
GO:0030097	87	3	0.045717167	hemopoiesis
GO:0030005	87	3	0.045717167	di-, tri-valent inorganic cation homeostasis
GO:0048534	88	3	0.047026575	hemopoietic or lymphoid organ development
GO:0006875	93	3	0.053848722	metal ion homeostasis
GO:0001909	6	1	0.053900566	immune cell mediated cytotoxicity
GO:0050851	6	1	0.053900566	antigen receptor-mediated signaling pathway
GO:0007242	879	13	0.056857067	intracellular signaling cascade
GO:0009966	229	5	0.059270707	regulation of signal transduction
GO:0045321	97	3	0.059630535	immune cell activation
GO:0030003	98	3	0.061120102	cation homeostasis
GO:0001775	98	3	0.061120102	cell activation
GO:0050715	7	1	0.062600572	positive regulation of cytokine secretion
GO:0050707	7	1	0.062600572	regulation of cytokine secretion
GO:0050663	7	1	0.062600572	cytokine secretion
GO:0050673	7	1	0.062600572	epithelial cell proliferation
GO:0050678	7	1	0.062600572	regulation of epithelial cell proliferation
GO:0007286	7	1	0.062600572	spermatid development
GO:0042509	7	1	0.062600572	regulation of tyrosine phosphorylation of STAT protein
GO:0046425	7	1	0.062600572	regulation of JAK-STAT cascade
GO:0006873	103	3	0.068826779	cell ion homeostasis
GO:0006633	47	2	0.069143464	fatty acid biosynthesis
GO:0006558	8	1	0.07122153	L-phenylalanine metabolism
GO:0006559	8	1	0.07122153	L-phenylalanine catabolism
GO:0001906	8	1	0.07122153	cell killing
GO:0048515	8	1	0.07122153	spermatid differentiation
GO:0009596	8	1	0.07122153	detection of pest, pathogen or parasite
GO:0051238	8	1	0.07122153	sequestering of metal ion
GO:0009628	318	6	0.071806679	response to abiotic stimulus
GO:0048522	482	8	0.074371657	positive regulation of cellular process
GO:0043085	107	3	0.075295268	positive regulation of enzyme activity
GO:0045766	9	1	0.079764152	positive regulation of angiogenesis
GO:0050714	9	1	0.079764152	positive regulation of protein secretion
GO:0050731	9	1	0.079764152	positive regulation of peptidyl-tyrosine phosphorylation
GO:0007159	9	1	0.079764152	leukocyte adhesion
GO:0042100	9	1	0.079764152	B cell proliferation

GO:0030330	9	1	0.079764152	DNA damage response, signal transduction by p53 class mediator
GO:0006268	9	1	0.079764152	DNA unwinding
GO:0007186	491	8	0.080856961	G-protein coupled receptor protein signaling pathway
GO:0050801	111	3	0.082024146	ion homeostasis
GO:0006916	112	3	0.083746025	anti-apoptosis
GO:0016053	54	2	0.087890522	organic acid biosynthesis
GO:0046394	54	2	0.087890522	carboxylic acid biosynthesis
GO:0019438	10	1	0.088229138	aromatic compound biosynthesis
GO:0001934	10	1	0.088229138	positive regulation of protein amino acid phosphorylation
GO:0045764	10	1	0.088229138	positive regulation of amino acid metabolism
GO:0009074	10	1	0.088229138	aromatic amino acid family catabolism
GO:0008630	10	1	0.088229138	DNA damage response, signal transduction resulting in induction of apoptosis
GO:0007260	10	1	0.088229138	tyrosine phosphorylation of STAT protein
GO:0042559	10	1	0.088229138	pteridine and derivative biosynthesis
GO:0050778	56	2	0.093498584	positive regulation of immune response
GO:0042558	11	1	0.096617188	pteridine and derivative metabolism
GO:0007610	269	5	0.101117553	behavior
GO:0019725	122	3	0.101797953	cell homeostasis
GO:0051240	59	2	0.102098996	positive regulation of organismal physiological process
GO:0050708	12	1	0.104928991	regulation of protein secretion
GO:0035295	12	1	0.104928991	tube development
GO:0042327	12	1	0.104928991	positive regulation of phosphorylation
GO:0045937	12	1	0.104928991	positive regulation of phosphate metabolism
GO:0042102	12	1	0.104928991	positive regulation of T cell proliferation
GO:0019439	13	1	0.113165232	aromatic compound catabolism
GO:0051047	13	1	0.113165232	positive regulation of secretion
GO:0030218	13	1	0.113165232	erythrocyte differentiation
GO:0043066	128	3	0.113315673	negative regulation of apoptosis
GO:0043069	129	3	0.115282322	negative regulation of programmed cell death
GO:0006261	65	2	0.1199115	DNA-dependent DNA replication
GO:0009725	14	1	0.121326589	response to hormone stimulus
GO:0007623	14	1	0.121326589	circadian rhythm
GO:0007602	14	1	0.121326589	phototransduction
GO:0006308	14	1	0.121326589	DNA catabolism
GO:0030224	14	1	0.121326589	monocyte differentiation
GO:0045445	15	1	0.129413736	myoblast differentiation
GO:0050730	15	1	0.129413736	regulation of peptidyl-tyrosine phosphorylation
GO:0042129	15	1	0.129413736	regulation of T cell proliferation
GO:0009583	15	1	0.129413736	detection of light
GO:0009719	214	4	0.133807317	response to endogenous stimulus
GO:0006809	16	1	0.13742734	nitric oxide biosynthesis
GO:0001932	16	1	0.13742734	regulation of protein amino acid phosphorylation
GO:0007157	16	1	0.13742734	heterophilic cell adhesion
GO:0009595	16	1	0.13742734	detection of biotic stimulus
GO:0046209	16	1	0.13742734	nitric oxide metabolism
GO:0015758	17	1	0.14536806	glucose transport
GO:0006521	17	1	0.14536806	regulation of amino acid metabolism

GO:0042098	17	1	0.14536806	T cell proliferation
GO:0006270	17	1	0.14536806	DNA replication initiation
GO:0048518	565	8	0.146564484	positive regulation of biological process
GO:0015749	18	1	0.153236551	monosaccharide transport
GO:0009072	18	1	0.153236551	aromatic amino acid family metabolism
GO:0045597	18	1	0.153236551	positive regulation of cell differentiation
GO:0008645	18	1	0.153236551	hexose transport
GO:0009416	18	1	0.153236551	response to light
GO:0042133	18	1	0.153236551	neurotransmitter metabolism
GO:0050671	18	1	0.153236551	positive regulation of lymphocyte proliferation
GO:0030217	18	1	0.153236551	T cell differentiation
GO:0050776	77	2	0.157518994	regulation of immune response
GO:0042692	19	1	0.161033463	muscle cell differentiation
GO:0018108	19	1	0.161033463	peptidyl-tyrosine phosphorylation
GO:0051243	583	8	0.165721927	negative regulation of cellular physiological process
GO:0008629	20	1	0.168759439	induction of apoptosis by intracellular signals
GO:0042592	157	3	0.175054496	homeostasis
GO:0051242	411	6	0.17593268	positive regulation of cellular physiological process
GO:0050870	21	1	0.176415117	positive regulation of T cell activation
GO:0018212	21	1	0.176415117	peptidyl-tyrosine modification
GO:0006357	240	4	0.178839101	regulation of transcription from RNA polymerase II promoter
GO:0048519	688	9	0.179115223	negative regulation of biological process
GO:0007243	242	4	0.182501093	protein kinase cascade
GO:0050794	2488	27	0.183718051	regulation of cellular process
GO:0050670	22	1	0.184001128	regulation of lymphocyte proliferation
GO:0042770	22	1	0.184001128	DNA damage response, signal transduction
GO:0043118	602	8	0.187159769	negative regulation of physiological process
GO:0006366	426	6	0.196799132	transcription from RNA polymerase II promoter
GO:0051046	24	1	0.19896665	regulation of secretion
GO:0043119	428	6	0.199651645	positive regulation of physiological process
GO:0006944	25	1	0.206347397	membrane fusion
GO:0045792	25	1	0.206347397	negative regulation of cell size
GO:0045765	25	1	0.206347397	regulation of angiogenesis
GO:0042325	25	1	0.206347397	regulation of phosphorylation
GO:0051235	25	1	0.206347397	maintenance of localization
GO:0030308	25	1	0.206347397	negative regulation of cell growth
GO:0008643	26	1	0.213660949	carbohydrate transport
GO:0009306	26	1	0.213660949	protein secretion
GO:0044419	26	1	0.213660949	interaction between organisms
GO:0006220	26	1	0.213660949	pyrimidine nucleotide metabolism
GO:0006919	27	1	0.220907911	caspase activation
GO:0042127	265	4	0.226281558	regulation of cell proliferation
GO:0046651	28	1	0.228088882	lymphocyte proliferation
GO:0008217	28	1	0.228088882	regulation of blood pressure
GO:0019220	28	1	0.228088882	regulation of phosphate metabolism
GO:0051348	28	1	0.228088882	negative regulation of transferase activity
GO:0007259	28	1	0.228088882	JAK-STAT cascade

GO:0051174	28	1	0.228088882	regulation of phosphorus metabolism
GO:0051251	28	1	0.228088882	positive regulation of lymphocyte activation
GO:0006469	28	1	0.228088882	negative regulation of protein kinase activity
GO:0006470	99	2	0.230730868	protein amino acid dephosphorylation
GO:0048523	640	8	0.23338847	negative regulation of cellular process
GO:0001819	29	1	0.235204456	positive regulation of cytokine production
GO:0048511	29	1	0.235204456	rhythmic process
GO:0050867	29	1	0.235204456	positive regulation of cell activation
GO:0045926	29	1	0.235204456	negative regulation of growth
GO:0007265	29	1	0.235204456	Ras protein signal transduction
GO:0050789	2689	28	0.247887702	regulation of biological process
GO:0043280	31	1	0.249241758	positive regulation of caspase activity
GO:0043281	31	1	0.249241758	regulation of caspase activity
GO:0051094	31	1	0.249241758	positive regulation of development
GO:0050863	31	1	0.249241758	regulation of T cell activation
GO:0006511	105	2	0.251169604	ubiquitin-dependent protein catabolism
GO:0019941	105	2	0.251169604	modification-dependent protein catabolism
GO:0000004	465	6	0.255011024	biological process unknown
GO:0051345	32	1	0.256164648	positive regulation of hydrolase activity
GO:0009314	33	1	0.263024463	response to radiation
GO:0030155	33	1	0.263024463	regulation of cell adhesion
GO:0016311	110	2	0.26824781	dephosphorylation
GO:0030098	34	1	0.269821771	lymphocyte differentiation
GO:0042742	34	1	0.269821771	defense response to bacteria
GO:0050791	2407	25	0.27231165	regulation of physiological process
GO:0006974	200	3	0.278814187	response to DNA damage stimulus
GO:0051244	2319	24	0.285585475	regulation of cellular physiological process
GO:0006487	37	1	0.28984425	protein amino acid N-linked glycosylation
GO:0000074	394	5	0.295462798	regulation of progression through cell cycle
GO:0001817	39	1	0.302890216	regulation of cytokine production
GO:0007519	39	1	0.302890216	myogenesis
GO:0008284	121	2	0.305776141	positive regulation of cell proliferation
GO:0048468	40	1	0.309324121	cell development
GO:0050790	214	3	0.314077538	regulation of enzyme activity
GO:0001505	41	1	0.315699354	regulation of neurotransmitter levels
GO:0051247	41	1	0.315699354	positive regulation of protein metabolism
GO:0051249	41	1	0.315699354	regulation of lymphocyte activation
GO:0006631	124	2	0.315965626	fatty acid metabolism
GO:0001816	42	1	0.322016444	cytokine production
GO:0009063	42	1	0.322016444	amino acid catabolism
GO:0050865	43	1	0.328275914	regulation of cell activation
GO:0009310	45	1	0.340624067	amine catabolism
GO:0042110	45	1	0.340624067	T cell activation
GO:0007229	45	1	0.340624067	integrin-mediated signaling pathway
GO:0051239	133	2	0.346321277	regulation of organismal physiological process
GO:0043086	46	1	0.346713775	negative regulation of enzyme activity
GO:0044270	47	1	0.352747912	nitrogen compound catabolism

GO:0006334	48	1	0.35872698	nucleosome assembly
GO:0051093	49	1	0.364651474	negative regulation of development
GO:0008285	139	2	0.366326848	negative regulation of cell proliferation
GO:0006260	140	2	0.3696397	DNA replication
GO:0018193	50	1	0.370521888	peptidyl-amino acid modification
GO:0006793	641	7	0.375242374	phosphorus metabolism
GO:0006796	641	7	0.375242374	phosphate metabolism
GO:0009617	51	1	0.376338709	response to bacteria
GO:0006869	52	1	0.38210242	lipid transport
GO:0045786	145	2	0.386102413	negative regulation of progression through cell cycle
GO:0006468	444	5	0.387205736	protein amino acid phosphorylation
GO:0045860	53	1	0.3878135	positive regulation of protein kinase activity
GO:0008632	53	1	0.3878135	apoptotic program
GO:0051347	54	1	0.393472424	positive regulation of transferase activity
GO:0045595	55	1	0.399079663	regulation of cell differentiation
GO:0031497	56	1	0.404635682	chromatin assembly
GO:0016481	151	2	0.405614731	negative regulation of transcription
GO:0006464	1087	11	0.415244681	protein modification
GO:0044265	563	6	0.415518113	cellular macromolecule catabolism
GO:0019752	359	4	0.421046136	carboxylic acid metabolism
GO:0006082	361	4	0.425210449	organic acid metabolism
GO:0051336	60	1	0.426356752	regulation of hydrolase activity
GO:0045934	159	2	0.431171949	negative regulation of nucleobase, nucleoside, nucleotide and nucleic acid metabolism
GO:0000122	61	1	0.431663528	negative regulation of transcription from RNA polymerase II promoter
GO:0009057	575	6	0.435516028	macromolecule catabolism
GO:0045449	1433	14	0.446892773	regulation of transcription
GO:0001525	65	1	0.452409921	angiogenesis
GO:0051056	65	1	0.452409921	regulation of small GTPase mediated signal transduction
GO:0044248	700	7	0.46585457	cellular catabolism
GO:0001568	68	1	0.46747702	blood vessel development
GO:0043123	68	1	0.46747702	positive regulation of I-kappaB kinase/NF-kappaB cascade
GO:0048514	68	1	0.46747702	blood vessel morphogenesis
GO:0016337	172	2	0.47144851	cell-cell adhesion
GO:0001944	69	1	0.472407788	vasculature development
GO:0019219	1458	14	0.474522771	regulation of nucleobase, nucleoside, nucleotide and nucleic acid metabolism
GO:0031323	1566	15	0.474831836	regulation of cellular metabolism
GO:0006281	174	2	0.47749666	DNA repair
GO:0008610	175	2	0.480505297	lipid biosynthesis
GO:0048015	71	1	0.482134402	phosphoinositide-mediated signaling
GO:0006355	1359	13	0.484379729	regulation of transcription, DNA-dependent
GO:0043122	73	1	0.491683818	regulation of I-kappaB kinase/NF-kappaB cascade
GO:0006725	73	1	0.491683818	aromatic compound metabolism
GO:0006520	181	2	0.498336897	amino acid metabolism
GO:0043283	2240	21	0.50014686	biopolymer metabolism
GO:0031324	183	2	0.504195444	negative regulation of cellular metabolism
GO:0043412	1160	11	0.504823093	biopolymer modification
GO:0006350	1499	14	0.519487664	transcription

GO:0030154	409	4	0.52231963	cell differentiation
GO:0016310	522	5	0.528154022	phosphorylation
GO:0006461	194	2	0.535632249	protein complex assembly
GO:0006351	1407	13	0.53837411	transcription, DNA-dependent
GO:0019222	1629	15	0.541707447	regulation of metabolism
GO:0009056	756	7	0.549278596	catabolism
GO:0006333	91	1	0.570162685	chromatin assembly or disassembly
GO:0006486	92	1	0.574152898	protein amino acid glycosylation
GO:0043413	93	1	0.578106512	biopolymer glycosylation
GO:0001558	94	1	0.582023859	regulation of cell growth
GO:0007517	94	1	0.582023859	muscle development
GO:0009101	96	1	0.58975106	glycoprotein biosynthesis
GO:0007582	7846	72	0.592563362	physiological process
GO:0045892	97	1	0.593561564	negative regulation of transcription, DNA-dependent
GO:0006519	216	2	0.594378713	amino acid and derivative metabolism
GO:0009892	217	2	0.596915606	negative regulation of metabolism
GO:0006508	451	4	0.600735753	proteolysis and peptidolysis
GO:0044257	451	4	0.600735753	cellular protein catabolism
GO:0007249	99	1	0.601077977	I-kappaB kinase/NF-kappaB cascade
GO:0045184	459	4	0.614802508	establishment of protein localization
GO:0008015	103	1	0.615701205	circulation
GO:0040008	104	1	0.619273475	regulation of growth
GO:0007264	227	2	0.621642398	small GTPase mediated signal transduction
GO:0030163	463	4	0.62172362	protein catabolism
GO:0048232	105	1	0.622812935	male gamete generation
GO:0007283	105	1	0.622812935	spermatogenesis
GO:0009582	109	1	0.636648592	detection of abiotic stimulus
GO:0008104	474	4	0.64036232	protein localization
GO:0043285	474	4	0.64036232	biopolymer catabolism
GO:0009100	111	1	0.643377177	glycoprotein metabolism
GO:0007049	601	5	0.655256882	cell cycle
GO:0006897	115	1	0.65646719	endocytosis
GO:0045859	116	1	0.659664815	regulation of protein kinase activity
GO:0051338	117	1	0.662833032	regulation of transferase activity
GO:0006259	489	4	0.664825703	DNA metabolism
GO:0008361	123	1	0.681239553	regulation of cell size
GO:0016049	123	1	0.681239553	cell growth
GO:0006118	256	2	0.686807592	electron transport
GO:0007276	125	1	0.687151629	gametogenesis
GO:0006886	257	2	0.688884253	intracellular protein transport
GO:0030036	126	1	0.690066911	actin cytoskeleton organization and biogenesis
GO:0044255	383	3	0.690068417	cellular lipid metabolism
GO:0016070	391	3	0.703577399	RNA metabolism
GO:0008202	132	1	0.707003393	steroid metabolism
GO:0007155	518	4	0.708921828	cell adhesion
GO:0009308	269	2	0.712943747	amine metabolism
GO:0031325	136	1	0.717783	positive regulation of cellular metabolism

GO:0030029	139	1	0.725609684	actin filament-based process
GO:0009117	143	1	0.735712585	nucleotide metabolism
GO:0019953	149	1	0.750181716	sexual reproduction
GO:0009893	149	1	0.750181716	positive regulation of metabolism
GO:0050953	149	1	0.750181716	sensory perception of light
GO:0007601	149	1	0.750181716	visual perception
GO:0006807	291	2	0.753060582	nitrogen compound metabolism
GO:0040007	154	1	0.761639296	growth
GO:0006139	2328	19	0.764131842	nucleobase, nucleoside, nucleotide and nucleic acid metabolism
GO:0015031	439	3	0.775467263	protein transport
GO:0009581	313	2	0.788324127	detection of external stimulus
GO:0050793	168	1	0.791030718	regulation of development
GO:0051246	174	1	0.802502829	regulation of protein metabolism
GO:0019932	176	1	0.806186817	second-messenger-mediated signaling
GO:0006629	469	3	0.812819434	lipid metabolism
GO:0043170	2405	19	0.814524963	macromolecule metabolism
GO:0000902	183	1	0.818554076	cellular morphogenesis
GO:0006325	186	1	0.823612569	establishment and/or maintenance of chromatin architecture
GO:0048513	872	6	0.825011211	organ development
GO:0007268	192	1	0.833315231	synaptic transmission
GO:0006323	193	1	0.834880258	DNA packaging
GO:0044249	763	5	0.841205328	cellular biosynthesis
GO:0019226	198	1	0.842490252	transmission of nerve impulse
GO:0044267	2109	16	0.844427301	cellular protein metabolism
GO:0019538	2129	16	0.855337035	protein metabolism
GO:0046903	208	1	0.856685765	secretion
GO:0000003	208	1	0.856685765	reproduction
GO:0007001	221	1	0.873265192	chromosome organization and biogenesis (sensu Eukaryota)
GO:0051276	236	1	0.890051404	chromosome organization and biogenesis
GO:0006412	407	2	0.894039033	protein biosynthesis
GO:0044238	4820	39	0.899709974	primary metabolism
GO:0009058	859	5	0.906548605	biosynthesis
GO:0006512	255	1	0.908192847	ubiquitin cycle
GO:0006091	446	2	0.921544728	generation of precursor metabolites and energy
GO:0046907	448	2	0.922758948	intracellular transport
GO:0044260	2298	16	0.926116636	cellular macromolecule metabolism
GO:0009059	464	2	0.931862207	macromolecule biosynthesis
GO:0007600	287	1	0.932299036	sensory perception
GO:0007267	465	2	0.932396573	cell-cell signaling
GO:0050877	475	2	0.937529281	neurophysiological process
GO:0016192	296	1	0.937869765	vesicle-mediated transport
GO:0007010	305	1	0.94298718	cytoskeleton organization and biogenesis
GO:0006396	326	1	0.953364413	RNA processing
GO:0044237	5026	39	0.955583617	cellular metabolism
GO:0007275	1543	9	0.958733221	development
GO:0005975	355	1	0.964691924	carbohydrate metabolism
GO:0008152	5262	40	0.975495067	metabolism

GO:0009987	8200	70	0.981391158	cellular process
GO:0009887	807	3	0.982642265	organogenesis
GO:0051179	1857	10	0.985268803	localization
GO:0016043	842	3	0.986790925	cell organization and biogenesis
GO:0006996	693	2	0.989768158	organelle organization and biogenesis
GO:0006811	515	1	0.992522286	ion transport
GO:0051234	1841	9	0.99306245	establishment of localization
GO:0006810	1802	8	0.996544888	transport
GO:0050875	7018	54	0.997240932	cellular physiological process
GO:0009653	1051	3	0.997613932	morphogenesis
GO:0008150	9032	83	1	biological_process

19.3 GO: Molecular Function

GO ID	genes present in chip	genes present in tissues	p-value	GO term
GO:0045012	12	5	3.53918932878829e-08	MHC class II receptor activity
GO:0004888	694	20	1.66094084664387e-06	transmembrane receptor activity
GO:0004872	1060	25	2.50617729507908e-06	receptor activity
GO:0004871	1837	34	5.32786396093488e-06	signal transducer activity
GO:0030106	26	4	7.21100219804638e-05	MHC class I receptor activity
GO:0003823	30	4	0.000128689307707648	antigen binding
GO:0016493	12	3	0.000136775585236505	C-C chemokine receptor activity
GO:0019957	12	3	0.000136775585236505	C-C chemokine binding
GO:0001614	20	3	0.000673577930209099	purinergic nucleotide receptor activity
GO:0001608	20	3	0.000673577930209099	nucleotide receptor activity, G-protein coupled
GO:0016502	20	3	0.000673577930209099	nucleotide receptor activity
GO:0045028	20	3	0.000673577930209099	purinergic nucleotide receptor activity, G-protein coupled
GO:0001637	22	3	0.000898437479714637	G-protein chemoattractant receptor activity
GO:0004950	22	3	0.000898437479714637	chemokine receptor activity
GO:0019956	24	3	0.00116591200070877	chemokine binding
GO:0005178	26	3	0.00147883845728484	integrin binding
GO:0008310	1	1	0.00880410858400538	single-stranded DNA specific 3'-5' exodeoxyribonuclease activity
GO:0008859	1	1	0.00880410858400538	exoribonuclease II activity
GO:0051397	1	1	0.00880410858400538	N-terminal basic amino acid aminopeptidase activity
GO:0051398	1	1	0.00880410858400538	N-terminal lysine aminopeptidase activity
GO:0051399	1	1	0.00880410858400538	N-terminal arginine aminopeptidase activity
GO:0003933	1	1	0.00880410858400538	GTP cyclohydrolase activity
GO:0003934	1	1	0.00880410858400538	GTP cyclohydrolase I activity
GO:0004051	1	1	0.00880410858400538	arachidonate 5-lipoxygenase activity
GO:0004137	1	1	0.00880410858400538	deoxycytidine kinase activity
GO:0045029	1	1	0.00880410858400538	UDP-activated nucleotide receptor activity
GO:0001584	282	7	0.0119243504594151	rhodopsin-like receptor activity
GO:0003690	20	2	0.0131307068644151	double-stranded DNA binding
GO:0030676	2	1	0.0175316195776053	Rac guanyl-nucleotide exchange factor activity
GO:0008297	2	1	0.0175316195776053	single-stranded DNA specific exodeoxyribonuclease activity
GO:0015065	2	1	0.0175316195776053	uridine nucleotide receptor activity
GO:0019955	65	3	0.0194379666602033	cytokine binding
GO:0004536	25	2	0.0201529604910486	deoxyribonuclease activity
GO:0005128	3	1	0.0261831914358173	erythropoietin receptor binding
GO:0008296	3	1	0.0261831914358173	3'-5'-exodeoxyribonuclease activity
GO:0042608	3	1	0.0261831914358173	T cell receptor binding
GO:0035091	132	4	0.0288795466957665	phosphoinositide binding
GO:0005057	132	4	0.0288795466957665	receptor signaling protein activity
GO:0004930	349	7	0.0337115744621136	G-protein coupled receptor activity
GO:0008020	4	1	0.0347594770218304	G-protein coupled photoreceptor activity
GO:0019136	4	1	0.0347594770218304	deoxynucleoside kinase activity
GO:0019238	4	1	0.0347594770218304	cyclohydrolase activity
GO:0003696	4	1	0.0347594770218304	satellite DNA binding

GO:0008301	5	1	0.0432611236539125	DNA bending activity
GO:0019863	5	1	0.0432611236539125	IgE binding
GO:0009881	5	1	0.0432611236539125	photoreceptor activity
GO:0005088	5	1	0.0432611236539125	Ras guanyl-nucleotide exchange factor activity
GO:0001653	94	3	0.0498232158279747	peptide receptor activity
GO:0008528	94	3	0.0498232158279747	peptide receptor activity, G-protein coupled
GO:0016165	6	1	0.0516887731519271	lipoxygenase activity
GO:0004540	45	2	0.0594298884629756	ribonuclease activity
GO:0001595	7	1	0.0600430618834673	angiotensin receptor activity
GO:0004529	7	1	0.0600430618834673	exodeoxyribonuclease activity
GO:0004945	7	1	0.0600430618834673	angiotensin type II receptor activity
GO:0016895	7	1	0.0600430618834673	exodeoxyribonuclease activity, producing 5'-phosphomonoesters
GO:0005543	169	4	0.0616240706269197	phospholipid binding
GO:0005529	103	3	0.0621903175035043	sugar binding
GO:0004518	105	3	0.0651160474501676	nuclease activity
GO:0004522	9	1	0.0765340755302583	pancreatic ribonuclease activity
GO:0016494	9	1	0.0765340755302583	C-X-C chemokine receptor activity
GO:0019958	10	1	0.0846720463291949	C-X-C chemokine binding
GO:0000175	10	1	0.0846720463291949	3'-5'-exoribonuclease activity
GO:0004716	10	1	0.0846720463291949	receptor signaling protein tyrosine kinase activity
GO:0005102	528	8	0.0924009934687648	receptor binding
GO:0004532	11	1	0.0927391482186445	exoribonuclease activity
GO:0005355	11	1	0.0927391482186445	glucose transporter activity
GO:0019206	11	1	0.0927391482186445	nucleoside kinase activity
GO:0016892	11	1	0.0927391482186445	endoribonuclease activity, producing 3'-phosphomonoesters
GO:0016896	11	1	0.0927391482186445	exoribonuclease activity, producing 5'-phosphomonoesters
GO:0016564	59	2	0.0950240986782745	transcriptional repressor activity
GO:0005515	3709	39	0.0951997598261348	protein binding
GO:0019900	60	2	0.0977590100482778	kinase binding
GO:0019865	12	1	0.100735990983559	immunoglobulin binding
GO:0008656	12	1	0.100735990983559	caspase activator activity
GO:0016284	12	1	0.100735990983559	alanine aminopeptidase activity
GO:0004179	12	1	0.100735990983559	membrane alanyl aminopeptidase activity
GO:0015145	12	1	0.100735990983559	monosaccharide transporter activity
GO:0015149	12	1	0.100735990983559	hexose transporter activity
GO:0042277	127	3	0.101235603976115	peptide binding
GO:0016894	13	1	0.108663179225492	endonuclease activity, active with either ribo- or deoxyribonucleic acids and producing 3'-phosphomonoesters
GO:0004519	65	2	0.111756330229272	endonuclease activity
GO:0030693	14	1	0.116521312406124	caspase activity
GO:0004520	14	1	0.116521312406124	endodeoxyribonuclease activity
GO:0016505	14	1	0.116521312406124	apoptotic protease activator activity
GO:0008289	298	5	0.121892533212395	lipid binding
GO:0004864	15	1	0.124310984890419	protein phosphatase inhibitor activity
GO:0016504	15	1	0.124310984890419	protease activator activity
GO:0004715	15	1	0.124310984890419	non-membrane spanning protein tyrosine kinase activity
GO:0003705	16	1	0.132032785989433	RNA polymerase II transcription factor activity, enhancer binding
GO:0030528	963	12	0.136950513144887	transcription regulator activity

GO:0019212	17	1	0.139687300002755	phosphatase inhibitor activity
GO:0016796	17	1	0.139687300002755	exonuclease activity, active with either ribo- or deoxyribonucleic acids and producing 5'-phosphomonoesters
GO:0004725	75	2	0.141141636695576	protein tyrosine phosphatase activity
GO:0004298	18	1	0.147275106260605	threonine endopeptidase activity
GO:0019899	151	3	0.147739050458959	enzyme binding
GO:0005001	19	1	0.154796779165583	transmembrane receptor protein tyrosine phosphatase activity
GO:0043028	19	1	0.154796779165583	caspase regulator activity
GO:0019198	19	1	0.154796779165583	transmembrane receptor protein phosphatase activity
GO:0005164	20	1	0.162252888234066	tumor necrosis factor receptor binding
GO:0004713	161	3	0.168867564406752	protein-tyrosine kinase activity
GO:0016814	21	1	0.169643998137263	hydrolase activity, acting on carbon-nitrogen (but not peptide) bonds, in cyclic amidines
GO:0008408	24	1	0.191432907742418	3'-5' exonuclease activity
GO:0048503	92	2	0.194140142230526	GPI anchor binding
GO:0005085	93	2	0.197340327928805	guanyl-nucleotide exchange factor activity
GO:0004860	25	1	0.198569572211225	protein kinase inhibitor activity
GO:0003725	26	1	0.205643989606586	double-stranded RNA binding
GO:0005125	181	3	0.213464092155743	cytokine activity
GO:0030246	181	3	0.213464092155743	carbohydrate binding
GO:0003697	28	1	0.219608224383866	single-stranded DNA binding
GO:0004177	28	1	0.219608224383866	aminopeptidase activity
GO:0019210	29	1	0.226499100989747	kinase inhibitor activity
GO:0051119	29	1	0.226499100989747	sugar transporter activity
GO:0008047	188	3	0.229652091020780	enzyme activator activity
GO:0015144	30	1	0.233329849046607	carbohydrate transporter activity
GO:0004521	31	1	0.240100986958508	endoribonuclease activity
GO:0003702	197	3	0.250794470714772	RNA polymerase II transcription factor activity
GO:0019205	34	1	0.260061857856194	nucleobase, nucleoside, nucleotide kinase activity
GO:0008094	34	1	0.260061857856194	DNA-dependent ATPase activity
GO:0019888	35	1	0.26659965147564	protein phosphatase regulator activity
GO:0051213	35	1	0.26659965147564	dioxygenase activity
GO:0016701	35	1	0.26659965147564	oxidoreductase activity, acting on single donors with incorporation of molecular oxygen
GO:0016702	35	1	0.26659965147564	oxidoreductase activity, acting on single donors with incorporation of molecular oxygen, incorporation of two atoms of oxygen
GO:0019208	36	1	0.273080361477328	phosphatase regulator activity
GO:0004721	118	2	0.278668367896251	phosphoprotein phosphatase activity
GO:0005126	38	1	0.285872496280554	hematopoietin/interferon-class (D200-domain) cytokine receptor binding
GO:0030234	509	6	0.290929632611828	enzyme regulator activity
GO:0008009	40	1	0.298442151812586	chemokine activity
GO:0004527	40	1	0.298442151812586	exonuclease activity
GO:0042379	40	1	0.298442151812586	chemokine receptor binding
GO:0001664	42	1	0.310793151589779	G-protein-coupled receptor binding
GO:0008235	44	1	0.322929254205478	metalloexopeptidase activity
GO:0005488	7228	66	0.322999764448043	binding
GO:0003712	230	3	0.330144053073164	transcription cofactor activity
GO:0019887	47	1	0.340738541046598	protein kinase regulator activity
GO:0003700	649	7	0.345879237938720	transcription factor activity
GO:0016788	446	5	0.356340120116202	hydrolase activity, acting on ester bonds
GO:0008083	144	2	0.362849169907721	growth factor activity

GO:0005083	144	2	0.362849169907721	small GTPase regulator activity
GO:0019901	54	1	0.380513451727763	protein kinase binding
GO:0019207	58	1	0.402167434884436	kinase regulator activity
GO:0016563	62	1	0.423073207582605	transcriptional activator activity
GO:0042803	64	1	0.433253534072767	protein homodimerization activity
GO:0046983	170	2	0.443280547466432	protein dimerization activity
GO:0016791	171	2	0.446268227115479	phosphoric monoester hydrolase activity
GO:0008565	67	1	0.448192097024887	protein transporter activity
GO:0008238	68	1	0.453084630680145	exopeptidase activity
GO:0030674	69	1	0.457934297208475	protein binding, bridging
GO:0016810	72	1	0.472229789237213	hydrolase activity, acting on carbon-nitrogen (but not peptide) bonds
GO:0008134	293	3	0.479338816145113	transcription factor binding
GO:0005524	970	9	0.486617692069301	ATP binding
GO:0004857	187	2	0.492835443583339	enzyme inhibitor activity
GO:0003779	189	2	0.498485490594122	actin binding
GO:0016773	532	5	0.506557278618967	phosphotransferase activity, alcohol group as acceptor
GO:0004197	80	1	0.508555299434732	cysteine-type endopeptidase activity
GO:0003714	81	1	0.512918617778193	transcription corepressor activity
GO:0030145	92	1	0.558460077929747	manganese ion binding
GO:0004672	447	4	0.559077622810442	protein kinase activity
GO:0030554	1028	9	0.559487334876532	adenyl nucleotide binding
GO:0003677	1384	12	0.570527033676805	DNA binding
GO:0030695	219	2	0.57834080199006	GTPase regulator activity
GO:0008234	98	1	0.581500072883053	cysteine-type peptidase activity
GO:0003676	1990	17	0.59899631351842	nucleic acid binding
GO:0042578	228	2	0.600448426375385	phosphoric ester hydrolase activity
GO:0016787	1424	12	0.612632431325905	hydrolase activity
GO:0016301	609	5	0.628768054424521	kinase activity
GO:0003924	121	1	0.659326533505122	GTPase activity
GO:0016887	255	2	0.661579423479865	ATPase activity
GO:0017076	1248	10	0.674394110737229	purine nucleotide binding
GO:0005554	522	4	0.682073973653974	molecular function unknown
GO:0008233	399	3	0.688677912816092	peptidase activity
GO:0008237	133	1	0.694064275845033	metallopeptidase activity
GO:0017111	410	3	0.706430098455908	nucleoside-triphosphatase activity
GO:0008092	279	2	0.709512385370648	cytoskeletal protein binding
GO:0003713	140	1	0.712686423737588	transcription coactivator activity
GO:0004175	288	2	0.725992543691395	endopeptidase activity
GO:0000166	1432	11	0.734144808766007	nucleotide binding
GO:0016462	430	3	0.736745523942292	pyrophosphatase activity
GO:0016818	432	3	0.739639520771416	hydrolase activity, acting on acid anhydrides, in phosphorus-containing anhydrides
GO:0016817	433	3	0.741077239159976	hydrolase activity, acting on acid anhydrides
GO:0003723	436	3	0.745353391055365	RNA binding
GO:0042802	158	1	0.75558429137153	protein self binding
GO:0016772	708	5	0.756768847225977	transferase activity, transferring phosphorus-containing groups
GO:0008270	1287	9	0.816572348022645	zinc ion binding
GO:0005506	212	1	0.849812701173067	iron ion binding

GO:0046914	1596	11	0.852563812844715	transition metal ion binding
GO:0016874	221	1	0.861559113519767	ligase activity
GO:0005525	226	1	0.867687708312193	GTP binding
GO:0042623	229	1	0.871235367167809	ATPase activity, coupled
GO:0019001	235	1	0.878051086726056	guanyl nucleotide binding
GO:0005216	252	1	0.89548581484216	ion channel activity
GO:0015268	274	1	0.9144376332544	alpha-type channel activity
GO:0015267	284	1	0.92188801075858	channel or pore class transporter activity
GO:0005509	646	3	0.93026824285026	calcium ion binding
GO:0004674	326	1	0.946780090267032	protein serine/threonine kinase activity
GO:0043169	2313	14	0.964948199256024	cation binding
GO:0016740	1169	5	0.982061215806072	transferase activity
GO:0005215	1030	4	0.984610308487266	transporter activity
GO:0043167	2556	14	0.989969444451308	ion binding
GO:0016491	509	1	0.990205857483425	oxidoreductase activity
GO:0015075	523	1	0.991407610563426	ion transporter activity
GO:0003824	3409	20	0.993307078036732	catalytic activity
GO:0046872	2495	13	0.993412568788766	metal ion binding
GO:0003674	9541	84	1	molecular_function

19.4 GO: Cellular Component

GO ID	genes present in chip	genes present in tissues	p-value	GO term
GO:0005887	1057	20	0.000729697457271519	integral to plasma membrane
GO:0031226	1062	20	0.000775178531224386	intrinsic to plasma membrane
GO:0005886	1472	23	0.00368808657394421	plasma membrane
GO:0016020	3514	43	0.00495529038021216	membrane
GO:0016021	2674	35	0.00508305009841172	integral to membrane
GO:0031224	2681	35	0.00532285082746664	intrinsic to membrane
GO:0043020	1	1	0.00891513256917953	NADPH oxidase complex
GO:0000502	41	2	0.0514411750780423	proteasome complex (sensu Eukaryota)
GO:0005925	8	1	0.0691620339595194	focal adhesion
GO:0016605	8	1	0.0691620339595194	PML body
GO:0005924	9	1	0.0774682714592425	cell-substrate adherens junction
GO:0042101	10	1	0.085701343213722	T cell receptor complex
GO:0030055	11	1	0.0938618852993304	cell-matrix junction
GO:0005623	7802	73	0.122268078090057	cell
GO:0005788	16	1	0.133598533972750	endoplasmic reticulum lumen
GO:0016604	16	1	0.133598533972750	nuclear body
GO:0043233	18	1	0.149007145232832	organelle lumen
GO:0005839	18	1	0.149007145232832	proteasome core complex (sensu Eukaryota)
GO:0005912	18	1	0.149007145232832	adherens junction
GO:0005635	83	2	0.168926651839043	nuclear membrane
GO:0048471	22	1	0.179017437078156	perinuclear region
GO:0000793	23	1	0.186355296317271	condensed chromosome
GO:0005938	28	1	0.222084682966213	cell cortex
GO:0001772	29	1	0.229042457319983	immunological synapse
GO:0005764	107	2	0.247084685192837	lysosome
GO:0000323	107	2	0.247084685192837	lytic vacuole
GO:0005773	120	2	0.290169614818648	vacuole
GO:0046930	42	1	0.314093695267501	pore complex
GO:0005643	42	1	0.314093695267501	nuclear pore
GO:0000228	42	1	0.314093695267501	nuclear chromosome
GO:0005654	221	3	0.315079934016169	nucleoplasm
GO:0008372	523	6	0.32283490118453	cellular component unknown
GO:0016327	50	1	0.361757036205724	apicolateral plasma membrane
GO:0043235	58	1	0.40614831599877	receptor complex
GO:0005783	483	5	0.432620721182756	endoplasmic reticulum
GO:0005829	276	3	0.448467899888336	cytosol
GO:0005730	75	1	0.490609411386718	nucleolus
GO:0005624	521	5	0.499381240296457	membrane fraction
GO:0005634	2704	24	0.553555199049215	nucleus
GO:0030054	93	1	0.567127885098234	cell junction
GO:0000785	100	1	0.593717479343147	chromatin
GO:0005792	108	1	0.622135737787241	microsome
GO:0042598	108	1	0.622135737787241	vesicular fraction

GO:0012505	246	2	0.649257718016282	endomembrane system
GO:0000267	698	5	0.756449721113393	cell fraction
GO:0005856	612	4	0.804872336392305	cytoskeleton
GO:0005694	203	1	0.84111621875444	chromosome
GO:0031090	377	2	0.855957088061246	organelle membrane
GO:0005615	385	2	0.864043140414005	extracellular space
GO:0043228	977	6	0.881577891275841	non-membrane-bound organelle
GO:0043232	977	6	0.881577891275841	intracellular non-membrane-bound organelle
GO:0005576	949	5	0.935881934019305	extracellular region
GO:0043227	3955	29	0.940618312307679	membrane-bound organelle
GO:0043231	3955	29	0.940618312307679	intracellular membrane-bound organelle
GO:0005737	2530	16	0.965646131772643	cytoplasm
GO:0005622	5419	41	0.966450646083536	intracellular
GO:0043226	4515	32	0.977627100317784	organelle
GO:0043229	4515	32	0.977627100317784	intracellular organelle
GO:0043234	1247	5	0.990791693696654	protein complex
GO:0005575	8637	77	1	cellular_component

20. BM-CD33Myeloid

20.1 BM-CD33Myeloid selective genes (162 genes)

Symbol	Description	Chromosome	Chromosome Location	LocusLink	Cytoband	UniGene
MNDA	myeloid cell nuclear differentiation antigen	1	157067791	4332	1q22	Hs.153837
S100A9	S100 calcium binding protein A9 (calgranulin B)	1	151596953	6280	1q21	Hs.112405
FCN1	ficolin (collagen/fibrinogen domain containing) 1	9	-136941256	2219	9q34	Hs.440898
LYZ	lysozyme (renal amyloidosis)	12	68028430	4069	12q15	Hs.524579
ITGAM	integrin, alpha M (complement component 3 receptor 3 subunit)	16	31178788	3684	16p11.2	Hs.172631
CSTA	cystatin A (stefin A)	3	123526700	1475	3q21	Hs.518198
FGL2	fibrinogen-like 2	7	-76660625	10875	7q11.23	Hs.520989
EVI2B	ecotropic viral integration site 2B	17	-26654918	2124	17q11.2	Hs.5509
CSPG2	chondroitin sulfate proteoglycan 2 (versican)	5	82803338	1462	5q14.3	Hs.443681
S100A12	S100 calcium binding protein A12 (calgranulin C)	1	-151612807	6283	1q21	Hs.19413
TYROBP	TYRO protein tyrosine kinase binding protein	19	-41087145	7305	19q13.1	Hs.515369
S100A8	S100 calcium binding protein A8 (calgranulin A)	1	-151629132	6279	1q21	Hs.416073
FCER1A	Fc fragment of IgE, high affinity I, receptor for, alpha polypeptide	1	157526127	2205	1q23	Hs.897
IL8	interleukin 8	4	74825138	3576	4q13-q21	Hs.624
TFEC	transcription factor EC	7	-115362437	22797	7q31.2	Hs.125962
CD1D	CD1d molecule	1	156416360	912	1q22-q23	Hs.1799 Hs.387539
HCK	hemopoietic cell kinase	20	30103717	3055	20q11-q12	Hs.126521
CTSS	cathepsin S	1	-148969177	1520	1q21	Hs.181301
C5AR1	complement component 5a receptor 1	19	52504970	728	19q13.3-q13.4	Hs.2161
NCF2	neutrophil cytosolic factor 2 (65kDa, chronic granulomatous disease, autosomal 2)	1	-181791320	4688	1q25	Hs.587558
MPO	myeloperoxidase	17	-53702215	4353	17q23.1	Hs.458272
CD36	CD36 molecule (thrombospondin receptor)	7	80105892, 80069458	948	7q11.2	Hs.120949 Hs.633085
IGSF6	immunoglobulin superfamily, member 6	16	-21560106	10261	16p12-p13	Hs.530902
CD302	CD302 molecule	2	-160333609	9936	2q24.2	Hs.130014
RNASE2	ribonuclease, RNase A family, 2 (liver, eosinophil-derived neurotoxin)	14	20493469	6036	14q24-q31	Hs.728
PYCARD	PYD and CARD domain containing	16	-31120309	29108	16p12-p11.2	Hs.499094
APOBEC3A	apolipoprotein B mRNA editing enzyme, catalytic polypeptide-like 3A	22	37683568	200315	22q13.1-q13.2	Hs.348983
GCA	grancalcin, EF-hand calcium binding protein	2	162908895	25801	2q24.2	Hs.377894
CD14	CD14 molecule	5	-139991500	929	5q22-q32|5q31.1	Hs.163867
KIAA0984	NA	12	NA	23329	12q14.3	Hs.192492
COTL1	coactosin-like 1 (Dictyostelium)	16	-83156706	23406	16q24.1	Hs.289092
LY96	lymphocyte antigen 96	8	75066143	23643	8q21.11	Hs.69328

PTPRC	protein tyrosine phosphatase, receptor type, C	1	196874847	5788	1q31-q32	Hs.192039
DOCK2	dedicator of cytokinesis 2	5	168996870	1794	5q35.1	Hs.586174
QPCT	glutaminyl-peptide cyclotransferase (glutaminyl cyclase)	2	37425256	25797	2p22.2	Hs.79033
VNN2	vanin 2	6	-133106702	8875	6q23-q24	Hs.293130
DUSP6	dual specificity phosphatase 6	12	-88265969	1848	12q22-q23	Hs.298654
FOSB	FBJ murine osteosarcoma viral oncogene homolog B	19	50663092	2354	19q13.32	Hs.590958
KLF4	Kruppel-like factor 4 (gut)	9	-109286955	9314	9q31	Hs.376206
RETN	resistin	19	7639971	56729	19p13.2	Hs.283091
CASP1	caspase 1, apoptosis-related cysteine peptidase (interleukin 1, beta, convertase)	11	-104401451	834	11q23	Hs.2490
DPYD	dihydropyrimidine dehydrogenase	1	-97315889	1806	1p22	Hs.335034
CXCL2	chemokine (C-X-C motif) ligand 2	4	-75181619	2920	4q21	Hs.590921
FLJ22662	NA	12	-14547863	79887	12p13.1	Hs.131933
TNFRSF1B	tumor necrosis factor receptor superfamily, member 1B	1	12149646	7133	1p36.3-p36.2	Hs.256278
GCH1	GTP cyclohydrolase 1 (dopa-responsive dystonia)	14	-54378475	2643	14q22.1-q22.2	Hs.86724
RNASE6	ribonuclease, RNase A family, k6	14	20319049	6039	14q11.2	Hs.23262
CPVL	carboxypeptidase, vitellogenic-like	7	-29001773	54504	7p15-p14	Hs.233389
NFE2	nuclear factor (erythroid-derived 2), 45kDa	12	-52972164	4778	12q13	Hs.75643
CLEC4A	C-type lectin domain family 4, member A	12	8167494	50856	12p13	Hs.504657
LGALS2	lectin, galactoside-binding, soluble, 2 (galectin 2)	22	-36296199	3957	22q12-q13|22q13.1	Hs.531776
RAB11FIP1	RAB11 family interacting protein 1 (class I)	8	-75672971	80223	8p11.22	Hs.191179
PTPN6	protein tyrosine phosphatase, non-receptor type 6	12	6930703, 6926000	5777	12p13	Hs.63489
HMGB2	high-mobility group box 2	4	-174489362	3148	4q31	Hs.434953
NCF4	neutrophil cytosolic factor 4, 40kDa	22	35586990	4689	22q13.1	Hs.474781
PSCDBP	pleckstrin homology, Sec7 and coiled-coil domains, binding protein	2	-157979377	9595	2q11.2	Hs.270
FGR	Gardner-Rasheed feline sarcoma viral (v-fgr) oncogene homolog	1	-27811397	2268	1p36.2-p36.1	Hs.1422
SERPINA1	serpin peptidase inhibitor, clade A (alpha-1 antiproteinase, antitrypsin), member 1	14	-93914450	5265	14q32.1	Hs.525557
ADCY7	adenylate cyclase 7	16	48879322	113	16q12-q13	Hs.513578
LRRFIP1	leucine rich repeat (in FLII) interacting protein 1	2	238265667	9208	2q37.3	Hs.471779
KCNH7	potassium voltage-gated channel, subfamily H (eag-related), member 7	2	-325924166	90134	2q24.2	Hs.470445
MAFB	v-maf musculoaponeurotic fibrosarcoma oncogene homolog B (avian)	20	-38747932	9935	20q11.2-q13.1	Hs.642679
SELL	selectin L (lymphocyte adhesion molecule 1)	1	-167926433	6402	1q23-q25	Hs.82848
CSF3R	colony stimulating factor 3 receptor (granulocyte)	1	-36704231	1441	1p35-p34.3	Hs.524517
EVI2A	ecotropic viral integration site 2A	17	-26668801	2123	17q11.2	Hs.591198
TLR2	toll-like receptor 2	4	154824890	7097	4q32	Hs.519033
GMFG	glia maturation factor, gamma	19	-44510841	9535	19q13.2	Hs.5210
CLEC7A	C-type lectin domain family 7, member A	12	-20331837	64581	12p13.2	Hs.143929
PMAIP1	phorbol-12-myristate-13-acetate-induced protein 1	18	55718216	5366	18q21.32	Hs.96
AZU1	azurocidin 1 (cationic antimicrobial protein 37)	19	778830	566	19p13.3	Hs.72885
MGC14376	NA	17	-1561553	84981	17p13.3	Hs.417157

CFP	complement factor properdin	X	-47368571	5199	Xp11.3-p11.23	Hs.53155
USP3	ubiquitin specific peptidase 3	15	61583862	9960	15q22.3	Hs.458499
PLAC8	placenta-specific 8	4	-84230866	51316	4q21.22	Hs.546392
CYBB	cytochrome b-245, beta polypeptide (chronic granulomatous disease)	X	37524263	1536	Xp21.1	Hs.292356
ALOX5	arachidonate 5-lipoxygenase	10	45189634	240	10q11.2	Hs.89499
AOAH	acyloxyacyl hydrolase (neutrophil)	7	-36519134	313	7p14-p12	Hs.488007
SULT1A1	sulfotransferase family, cytosolic, 1A, phenol-preferring, member 1	16	-28524418	6817	16p12.1	Hs.567342
GLIPR1	GLI pathogenesis-related 1 (glioma)	12	74160779	11010	12q21.2	Hs.205558 Hs.591034
ELA2	elastase 2, neutrophil	19	803290	1991	19p13.3	Hs.99863
PTGS2	prostaglandin-endoperoxide synthase 2 (prostaglandin G/H synthase and cyclooxygenase)	1	-184907592	5743	1q25.2-q25.3	Hs.196384
CX3CR1	chemokine (C-X3-C motif) receptor 1	3	-39279989	1524	3p21|3p21.3	Hs.78913
RNF138	ring finger protein 138	18	27925834, 27926644	51444	18q12.1	Hs.302408 Hs.501040
C1orf38	chromosome 1 open reading frame 38	1	28071641	9473	1p35.3	Hs.10649
SCAP2	src family associated phosphoprotein 2	7	-26673214	8935	7p21-p15	Hs.200770
SGK	serum/glucocorticoid regulated kinase	6	-134532081	6446	6q23	Hs.510078
PTGER2	prostaglandin E receptor 2 (subtype EP2), 53kDa	14	51850862	5732	14q22	Hs.2090
PRG1	proteoglycan 1, secretory granule	10	70517833	5552	10q22.1	Hs.1908
PTPRE	protein tyrosine phosphatase, receptor type, E	10	129595314, 129735823	5791	10q26	Hs.127022
TRIB1	tribbles homolog 1 (Drosophila)	8	126511744	10221	8q24.13	Hs.444947
IMPDH1	IMP (inosine monophosphate) dehydrogenase 1	7	-127819567	3614	7q31.3-q32	Hs.534808
PELI1	pellino homolog 1 (Drosophila)	2	-64173499	57162	2p13.3	Hs.7886
LYN	v-yes-1 Yamaguchi sarcoma viral related oncogene homolog	8	56954925	4067	8q13	Hs.491767 Hs.614775
CENTB2	centaurin, beta 2	3	-196476767	23527	3q29	Hs.478746
ANXA1	annexin A1	9	74956600	301	9q12-q21.2|9q12-q21.2	Hs.494173
LMO2	LIM domain only 2 (rhombotin-like 1)	11	-33836698	4005	11p13	Hs.34560
VAMP8	vesicle-associated membrane protein 8 (endobrevin)	2	85658227	8673	2p12-p11.2	Hs.534373
ALOX5AP	arachidonate 5-lipoxygenase-activating protein	13	30207668	241	13q12	Hs.507658
SAMSN1	SAM domain, SH3 domain and nuclear localisation signals, 1	21	-14779419	64092	21q11	Hs.473341 Hs.570423
NR4A2	nuclear receptor subfamily 4, group A, member 2	2	-156889196	4929	2q22-q23	Hs.563344
FLJ20273	NA	4	-40120497	54502	4p13-p12	Hs.518727
STK17B	serine/threonine kinase 17b (apoptosis-inducing)	2	-196710102	9262	2q32.3	Hs.88297
RGS2	regulator of G-protein signalling 2, 24kDa	1	191044793	5997	1q31	Hs.78944
STX11	syntaxin 11	6	144513361	8676	6q24.2	Hs.118958
DPCR1	diffuse panbronchiolitis critical region 1	6	31027537, 2367969, 2166477	135656	6p21.33	Hs.631993
HLA-DRA	major histocompatibility complex, class II, DR alpha	6	32515624	3122	6p21.3	Hs.520048
BCL2A1	BCL2-related protein A1	15	-78040289	597	15q24.3	Hs.227817
CD37	CD37 molecule	19	54530488	951	19q13.3	Hs.166556
PILRA	paired immunoglobin-like type 2 receptor alpha	7	99809003	29992	7q22.1	Hs.444407
SYK	spleen tyrosine kinase	9	92603890	6850	9q22	Hs.371720
FPR1	formyl peptide receptor 1	19	-56940838	2357	19q13.4	Hs.753
AHR	aryl hydrocarbon receptor	7	17304831	196	7p15	Hs.171189

ZNF651	zinc finger protein 651	3	42675877	92999	3p22.1	Hs.409561
KCTD12	potassium channel tetramerisation domain containing 12	13	-76352312	115207	13q22.3	Hs.109438
S100P	S100 calcium binding protein P	4	6746466	6286	4p16	Hs.2962
ICAM3	intercellular adhesion molecule 3	19	-10305453	3385	19p13.3-p13.2	Hs.75516
LILRA2	leukocyte immunoglobulin-like receptor, subfamily A (with TM domain), member 2	19	59777070	11027	19q13.4	Hs.534394
BIN2	bridging integrator 2	12	-49961089	51411	12q13	Hs.14770
SERPINB1	serpin peptidase inhibitor, clade B (ovalbumin), member 1	6	-2778734	1992	6p25	Hs.381167
AGTPBP1	ATP/GTP binding protein 1	9	-87351274	23287	9q21.33	Hs.494321
IER5	immediate early response 5	1	179324260	51278	1q25.3	Hs.15725
MCL1	myeloid cell leukemia sequence 1 (BCL2-related)	1	-148813660	4170	1q21	Hs.632486
RBM3	RNA binding motif (RNP1, RRM) protein 3	X	48317779	5935	Xp11.2	Hs.301404
CD52	CD52 molecule	1	26516997	1043	1p36	Hs.276770
NMI	N-myc (and STAT) interactor	2	-151835230	9111	2p24.3-q21.3	Hs.54483
KLF10	Kruppel-like factor 10	8	-207460384	7071	8q22.2	Hs.435001
MYO1F	myosin IF	19	-8491998	4542	19p13.3-p13.2	Hs.465818
CAPG	capping protein (actin filament), gelsolin-like	2	-85475381	822	2p11.2	Hs.516155
JUN	v-jun sarcoma virus 17 oncogene homolog (avian)	1	-59019052	3725	1p32-p31	Hs.525704
LST1	leukocyte specific transcript 1	6	31661949, 3000093, 2801557, 31662603, 3000747, 2802211	7940	6p21.3	Hs.436066
ITGA4	integrin, alpha 4 (antigen CD49D, alpha 4 subunit of VLA-4 receptor)	2	182029863	3676	2q31.3	Hs.440955
CD48	CD48 molecule	1	-158915161	962	1q21.3-q22	Hs.243564
MS4A6A	membrane-spanning 4-domains, subfamily A, member 6A	11	-119392204	64231	11q12.1	Hs.523702
HCLS1	hematopoietic cell-specific Lyn substrate 1	3	-122832936	3059	3q13	Hs.14601
SNX2	sorting nexin 2	5	122138648	6643	5q23	Hs.134822
KLHL2	kelch-like 2, Mayven (Drosophila)	4	166348240	11275	4q21.2	Hs.388668
SERPINB2	serpin peptidase inhibitor, clade B (ovalbumin), member 2	18	59705921	5055	18q21.3	Hs.514913
FBN2	fibrillin 2 (congenital contractural arachnodactyly)	5	-127621500	2201	5q23-q31	Hs.519294
EREG	epiregulin	4	75449723	2069	4q13.3	Hs.115263
MYB	v-myb myeloblastosis viral oncogene homolog (avian)	6	135544145	4602	6q22-q23	Hs.531941
TNFSF10	tumor necrosis factor (ligand) superfamily, member 10	3	-173706158	8743	3q26	Hs.478275
IMPA2	inositol(myo)-1(or 4)-monophosphatase 2	18	11971454	3613	18p11.2	Hs.367992
PADI4	peptidyl arginine deiminase, type IV	1	17507278	23569	1p36.13	Hs.522969
BPI	bactericidal/permeability-increasing protein	20	36365998	671	20q11.23-q12	Hs.529019
LILRB2	leukocyte immunoglobulin-like receptor, subfamily B (with TM and ITIM domains), member 2	19	-59470131	10288	19q13.4	Hs.241797 Hs.631536
LR8	NA	7	-150119323	28959	7q36.1	Hs.438823
DHRS8	dehydrogenase/reductase (SDR family) member 8	4	-88476788	51170	4q22.1	Hs.282984
PSMB10	proteasome (prosome, macropain) subunit, beta type, 10	16	-66525907	5699	16q22.1	Hs.9661
TNFAIP8	tumor necrosis factor, alpha-induced protein 8	5	118719628	25816	5q23.1	Hs.271955
IER3	immediate early response 3	6	-34936359	8870	6p21.3	Hs.591785

IFI44	interferon-induced protein 44	1	78888103	10561	1p31.1	Hs.82316
LCP2	lymphocyte cytosolic protein 2 (SH2 domain containing leukocyte protein of 76kDa)	5	-169607666	3937	5q33.1-qter	Hs.304475
HHEX	homeobox, hematopoietically expressed	10	94439679	3087	10q23.33	Hs.118651
SPI1	spleen focus forming virus (SFFV) proviral integration oncogene spi1	11	-47332987	6688	11p11.2	Hs.502511
VNN1	vanin 1	6	-133044424	8876	6q23-q24	Hs.12114
FRAT2	frequently rearranged in advanced T-cell lymphomas 2	10	-99082244	23401	10q24.1	Hs.140720
TCIRG1	T-cell, immune regulator 1, ATPase, H+ transporting, lysosomal V0 subunit A3	11	67563058, 67567022	10312	11q13.2	Hs.495985
TBXAS1	thromboxane A synthase 1 (platelet, cytochrome P450, family 5, subfamily A)	7	139175420	6916	7q34-q35	Hs.520757
SOD2	superoxide dismutase 2, mitochondrial	6	-320042884	6648	6q25.3	Hs.487046
MS4A3	membrane-spanning 4-domains, subfamily A, member 3 (hematopoietic cell-specific)	11	59580676	932	11q12.1	Hs.99960
LILRA5	leukocyte immunoglobulin-like receptor, subfamily A (with TM domain), member 5	19	-238049124	353514	19q13.4	Hs.631592
ASGR1	asialoglycoprotein receptor 1	17	-7017476	432	17p13.2	Hs.12056

20.2 GO: Biological Process

GO ID	genes present in chip	genes present in tissues	p-value	GO term
GO:0006952	751	53	1.94E-23	defense response
GO:0009607	857	56	2.94E-23	response to biotic stimulus
GO:0006955	684	48	6.51E-21	immune response
GO:0043207	463	40	1.72E-20	response to external biotic stimulus
GO:0009613	458	39	9.75E-20	response to pest, pathogen or parasite
GO:0050896	1578	65	1.48E-16	response to stimulus
GO:0009611	341	31	1.83E-16	response to wounding
GO:0006950	881	46	6.45E-15	response to stress
GO:0009605	963	46	1.78E-13	response to external stimulus
GO:0006954	185	20	2.95E-12	inflammatory response
GO:0050874	1531	53	4.54E-10	organismal physiological process
GO:0006935	101	10	2.41E-06	chemotaxis
GO:0042330	101	10	2.41E-06	taxis
GO:0006968	87	9	5.45E-06	cellular defense response
GO:0042742	34	6	9.51E-06	defense response to bacteria
GO:0009617	51	7	9.54E-06	response to bacteria
GO:0042221	183	12	1.76E-05	response to chemical substance
GO:0050776	77	8	1.79E-05	regulation of immune response
GO:0030595	13	4	3.07E-05	immune cell chemotaxis
GO:0050900	14	4	4.25E-05	immune cell migration
GO:0008219	498	20	4.63E-05	cell death
GO:0016265	501	20	5.03E-05	death
GO:0006915	471	19	6.88E-05	apoptosis
GO:0012501	473	19	7.28E-05	programmed cell death
GO:0050920	7	3	0.0001094	regulation of chemotaxis
GO:0030216	18	4	0.000124146	keratinocyte differentiation
GO:0050778	56	6	0.000175407	positive regulation of immune response
GO:0042981	306	14	0.000184908	regulation of apoptosis
GO:0043067	308	14	0.000197873	regulation of programmed cell death
GO:0048519	688	23	0.000198883	negative regulation of biological process
GO:0051240	59	6	0.000234661	positive regulation of organismal physiological process
GO:0006916	112	8	0.000261006	anti-apoptosis
GO:0051247	41	5	0.000336915	positive regulation of protein metabolism
GO:0046456	23	4	0.000339009	icosanoid biosynthesis
GO:0048523	640	21	0.000499632	negative regulation of cellular process
GO:0043066	128	8	0.000642817	negative regulation of apoptosis
GO:0042327	12	3	0.000650916	positive regulation of phosphorylation
GO:0045937	12	3	0.000650916	positive regulation of phosphate metabolism
GO:0042531	3	2	0.000658802	positive regulation of tyrosine phosphorylation of STAT protein
GO:0046427	3	2	0.000658802	positive regulation of JAK-STAT cascade
GO:0043069	129	8	0.000676831	negative regulation of programmed cell death
GO:0043405	49	5	0.000779179	regulation of MAPK activity
GO:0051239	133	8	0.000827721	regulation of organismal physiological process

GO:0030218	13	3	0.000836968	erythrocyte differentiation
GO:0001819	29	4	0.000848303	positive regulation of cytokine production
GO:0007165	2168	49	0.000851457	signal transduction
GO:0006928	205	10	0.000975254	cell motility
GO:0040011	205	10	0.000975254	locomotion
GO:0019735	77	6	0.000991603	antimicrobial humoral response (sensu Vertebrata)
GO:0051243	583	19	0.001027718	negative regulation of cellular physiological process
GO:0019730	78	6	0.001061252	antimicrobial humoral response
GO:0043085	107	7	0.001074782	positive regulation of enzyme activity
GO:0045860	53	5	0.001117988	positive regulation of protein kinase activity
GO:0007626	209	10	0.001129905	locomotory behavior
GO:0006959	141	8	0.001210487	humoral immune response
GO:0051347	54	5	0.001217479	positive regulation of transferase activity
GO:0006909	15	3	0.001302701	phagocytosis
GO:0045089	4	2	0.00130468	positive regulation of innate immune response
GO:0045073	4	2	0.00130468	regulation of chemokine biosynthesis
GO:0050790	214	10	0.001350903	regulation of enzyme activity
GO:0006690	33	4	0.001395551	icosanoid metabolism
GO:0043118	602	19	0.001498459	negative regulation of physiological process
GO:0007166	952	26	0.001651558	cell surface receptor linked signal transduction
GO:0009967	85	6	0.001659714	positive regulation of signal transduction
GO:0045859	116	7	0.001720197	regulation of protein kinase activity
GO:0051338	117	7	0.001807285	regulation of transferase activity
GO:0042033	5	2	0.002153165	chemokine biosynthesis
GO:0050755	5	2	0.002153165	chemokine metabolism
GO:0050727	5	2	0.002153165	regulation of inflammatory response
GO:0050926	5	2	0.002153165	regulation of positive chemotaxis
GO:0050927	5	2	0.002153165	positive regulation of positive chemotaxis
GO:0050918	5	2	0.002153165	positive chemotaxis
GO:0050930	5	2	0.002153165	induction of positive chemotaxis
GO:0045088	5	2	0.002153165	regulation of innate immune response
GO:0030593	5	2	0.002153165	neutrophil chemotaxis
GO:0007610	269	11	0.002276596	behavior
GO:0001817	39	4	0.002617764	regulation of cytokine production
GO:0009628	318	12	0.002824166	response to abiotic stimulus
GO:0050921	6	2	0.003198145	positive regulation of chemotaxis
GO:0007154	2730	56	0.003365077	cell communication
GO:0001816	42	4	0.003441566	cytokine production
GO:0016064	103	6	0.004362995	humoral defense mechanism (sensu Vertebrata)
GO:0042509	7	2	0.004433643	regulation of tyrosine phosphorylation of STAT protein
GO:0046425	7	2	0.004433643	regulation of JAK-STAT cascade
GO:0042108	23	3	0.004646697	positive regulation of cytokine biosynthesis
GO:0006633	47	4	0.005178445	fatty acid biosynthesis
GO:0045727	25	3	0.005904863	positive regulation of protein biosynthesis
GO:0042325	25	3	0.005904863	regulation of phosphorylation
GO:0031328	26	3	0.006602956	positive regulation of cellular biosynthesis
GO:0050731	9	2	0.007452948	positive regulation of peptidyl-tyrosine phosphorylation

GO:0045087	53	4	0.007937277	innate immune response
GO:0019220	28	3	0.008141141	regulation of phosphate metabolism
GO:0007259	28	3	0.008141141	JAK-STAT cascade
GO:0051174	28	3	0.008141141	regulation of phosphorus metabolism
GO:0016053	54	4	0.008474861	organic acid biosynthesis
GO:0046394	54	4	0.008474861	carboxylic acid biosynthesis
GO:0001516	10	2	0.009225455	prostaglandin biosynthesis
GO:0001934	10	2	0.009225455	positive regulation of protein amino acid phosphorylation
GO:0045764	10	2	0.009225455	positive regulation of amino acid metabolism
GO:0007260	10	2	0.009225455	tyrosine phosphorylation of STAT protein
GO:0046457	10	2	0.009225455	prostanoid biosynthesis
GO:0030097	87	5	0.009621665	hemopoiesis
GO:0009891	30	3	0.009873184	positive regulation of biosynthesis
GO:0048518	565	16	0.009965239	positive regulation of biological process
GO:0048534	88	5	0.010081823	hemopoietic or lymphoid organ development
GO:0007243	242	9	0.010277776	protein kinase cascade
GO:0043406	31	3	0.010813366	positive regulation of MAPK activity
GO:0042035	33	3	0.012844459	regulation of cytokine biosynthesis
GO:0030155	33	3	0.012844459	regulation of cell adhesion
GO:0019370	12	2	0.013268865	leukotriene biosynthesis
GO:0001709	12	2	0.013268865	cell fate determination
GO:0045321	97	5	0.014919629	immune cell activation
GO:0048003	1	1	0.014946856	antigen presentation, lipid antigen
GO:0048006	1	1	0.014946856	antigen presentation, endogenous lipid antigen
GO:0031342	1	1	0.014946856	negative regulation of cell killing
GO:0001556	1	1	0.014946856	oocyte maturation
GO:0045415	1	1	0.014946856	negative regulation of interleukin-8 biosynthesis
GO:0019371	1	1	0.014946856	cyclooxygenase pathway
GO:0048160	1	1	0.014946856	primary follicle stage oogenesis
GO:0001911	1	1	0.014946856	negative regulation of immune cell mediated cytotoxicity
GO:0001913	1	1	0.014946856	T cell mediated cytotoxicity
GO:0001914	1	1	0.014946856	regulation of T cell mediated cytotoxicity
GO:0001915	1	1	0.014946856	negative regulation of T cell mediated cytotoxicity
GO:0045740	1	1	0.014946856	positive regulation of DNA replication
GO:0045741	1	1	0.014946856	positive regulation of epidermal growth factor receptor activity
GO:0019860	1	1	0.014946856	uracil metabolism
GO:0045647	1	1	0.014946856	negative regulation of erythrocyte differentiation
GO:0050751	1	1	0.014946856	fractalkine biosynthesis
GO:0050752	1	1	0.014946856	regulation of fractalkine biosynthesis
GO:0050754	1	1	0.014946856	positive regulation of fractalkine biosynthesis
GO:0050756	1	1	0.014946856	fractalkine metabolism
GO:0050720	1	1	0.014946856	interleukin-1 beta biosynthesis
GO:0050722	1	1	0.014946856	regulation of interleukin-1 beta biosynthesis
GO:0050725	1	1	0.014946856	positive regulation of interleukin-1 beta biosynthesis
GO:0050728	1	1	0.014946856	negative regulation of inflammatory response
GO:0050853	1	1	0.014946856	B cell receptor signaling pathway
GO:0007423	1	1	0.014946856	sensory organ development

GO:0000303	1	1	0.014946856	response to superoxide
GO:0007143	1	1	0.014946856	female meiosis
GO:0042117	1	1	0.014946856	monocyte activation
GO:0042700	1	1	0.014946856	luteinizing hormone signaling pathway
GO:0051103	1	1	0.014946856	DNA ligation during DNA repair
GO:0050922	1	1	0.014946856	negative regulation of chemotaxis
GO:0001315	1	1	0.014946856	age-dependent response to reactive oxygen species
GO:0046121	1	1	0.014946856	deoxyribonucleoside catabolism
GO:0046127	1	1	0.014946856	pyrimidine deoxyribonucleoside catabolism
GO:0046113	1	1	0.014946856	nucleobase catabolism
GO:0046104	1	1	0.014946856	thymidine metabolism
GO:0006288	1	1	0.014946856	base-excision repair, DNA ligation
GO:0030728	1	1	0.014946856	ovulation
GO:0006212	1	1	0.014946856	uracil catabolism
GO:0006214	1	1	0.014946856	thymidine catabolism
GO:0006208	1	1	0.014946856	pyrimidine base catabolism
GO:0001774	1	1	0.014946856	microglial cell activation
GO:0045080	1	1	0.014946856	positive regulation of chemokine biosynthesis
GO:0000302	13	2	0.01552921	response to reactive oxygen species
GO:0001775	98	5	0.015538308	cell activation
GO:0009892	217	8	0.015976214	negative regulation of metabolism
GO:0042089	36	3	0.016273494	cytokine biosynthesis
GO:0042107	37	3	0.017519603	cytokine metabolism
GO:0030099	37	3	0.017519603	myeloid cell differentiation
GO:0042127	265	9	0.017718908	regulation of cell proliferation
GO:0006691	14	2	0.017941807	leukotriene metabolism
GO:0009725	14	2	0.017941807	response to hormone stimulus
GO:0031324	183	7	0.019738591	negative regulation of cellular metabolism
GO:0007242	879	21	0.020442262	intracellular signaling cascade
GO:0006801	15	2	0.020501669	superoxide metabolism
GO:0045165	15	2	0.020501669	cell fate commitment
GO:0050730	15	2	0.020501669	regulation of peptidyl-tyrosine phosphorylation
GO:0018149	15	2	0.020501669	peptide cross-linking
GO:0001932	16	2	0.023203924	regulation of protein amino acid phosphorylation
GO:0050777	16	2	0.023203924	negative regulation of immune response
GO:0016481	151	6	0.025522502	negative regulation of transcription
GO:0006521	17	2	0.02604381	regulation of amino acid metabolism
GO:0006692	17	2	0.02604381	prostanoid metabolism
GO:0006693	17	2	0.02604381	prostaglandin metabolism
GO:0045596	17	2	0.02604381	negative regulation of cell differentiation
GO:0043119	428	12	0.026279795	positive regulation of physiological process
GO:0006897	115	5	0.028783288	endocytosis
GO:0007229	45	3	0.029361384	integrin-mediated signaling pathway
GO:0031341	2	1	0.029671933	regulation of cell killing
GO:0001550	2	1	0.029671933	ovarian cumulus expansion
GO:0001547	2	1	0.029671933	antral ovarian follicle growth
GO:0045360	2	1	0.029671933	regulation of interleukin-1 biosynthesis

GO:0045362	2	1	0.029671933	positive regulation of interleukin-1 biosynthesis
GO:0045348	2	1	0.029671933	positive regulation of MHC class II biosynthesis
GO:0008347	2	1	0.029671933	glial cell migration
GO:0045123	2	1	0.029671933	cellular extravasation
GO:0048165	2	1	0.029671933	fused antrum stage oogenesis
GO:0048157	2	1	0.029671933	oogenesis (sensu Mammalia)
GO:0048146	2	1	0.029671933	positive regulation of fibroblast proliferation
GO:0001910	2	1	0.029671933	regulation of immune cell mediated cytotoxicity
GO:0048247	2	1	0.029671933	lymphocyte chemotaxis
GO:0008228	2	1	0.029671933	opsonization
GO:0042063	2	1	0.029671933	gliogenesis
GO:0019733	2	1	0.029671933	antibacterial humoral response (sensu Vertebrata)
GO:0050829	2	1	0.029671933	defense response to Gram-negative bacteria
GO:0009120	2	1	0.029671933	deoxyribonucleoside metabolism
GO:0000305	2	1	0.029671933	response to oxygen radicals
GO:0042222	2	1	0.029671933	interleukin-1 biosynthesis
GO:0051150	2	1	0.029671933	regulation of smooth muscle cell differentiation
GO:0051151	2	1	0.029671933	negative regulation of smooth muscle cell differentiation
GO:0051145	2	1	0.029671933	smooth muscle cell differentiation
GO:0051147	2	1	0.029671933	regulation of muscle cell differentiation
GO:0051148	2	1	0.029671933	negative regulation of muscle cell differentiation
GO:0046037	2	1	0.029671933	GMP metabolism
GO:0046125	2	1	0.029671933	pyrimidine deoxyribonucleoside metabolism
GO:0006177	2	1	0.029671933	GMP biosynthesis
GO:0046135	2	1	0.029671933	pyrimidine nucleoside catabolism
GO:0045091	2	1	0.029671933	regulation of retroviral genome replication
GO:0045934	159	6	0.03178754	negative regulation of nucleobase, nucleoside, nucleotide and nucleic acid metabolism
GO:0018108	19	2	0.032117964	peptidyl-tyrosine phosphorylation
GO:0008284	121	5	0.034765853	positive regulation of cell proliferation
GO:0051241	20	2	0.035343244	negative regulation of organismal physiological process
GO:0006631	124	5	0.038025807	fatty acid metabolism
GO:0016477	85	4	0.03818277	cell migration
GO:0019221	21	2	0.038688171	cytokine and chemokine mediated signaling pathway
GO:0018212	21	2	0.038688171	peptidyl-tyrosine modification
GO:0050670	22	2	0.042148505	regulation of lymphocyte proliferation
GO:0030154	409	11	0.042275674	cell differentiation
GO:0051242	411	11	0.043535218	positive regulation of cellular physiological process
GO:0048002	3	1	0.044178499	antigen presentation, peptide antigen
GO:0048004	3	1	0.044178499	antigen presentation, endogenous peptide antigen
GO:0045342	3	1	0.044178499	MHC class II biosynthesis
GO:0045346	3	1	0.044178499	regulation of MHC class II biosynthesis
GO:0043114	3	1	0.044178499	regulation of vascular permeability
GO:0048246	3	1	0.044178499	macrophage chemotaxis
GO:0019731	3	1	0.044178499	antibacterial humoral response
GO:0048599	3	1	0.044178499	oocyte development
GO:0045646	3	1	0.044178499	regulation of erythrocyte differentiation
GO:0050857	3	1	0.044178499	positive regulation of antigen receptor mediated signaling pathway

GO:0009164	3	1	0.044178499	nucleoside catabolism
GO:0009299	3	1	0.044178499	mRNA transcription
GO:0007176	3	1	0.044178499	regulation of epidermal growth factor receptor activity
GO:0042119	3	1	0.044178499	neutrophil activation
GO:0050680	3	1	0.044178499	negative regulation of epithelial cell proliferation
GO:0042533	3	1	0.044178499	tumor necrosis factor-alpha biosynthesis
GO:0042534	3	1	0.044178499	regulation of tumor necrosis factor-alpha biosynthesis
GO:0042535	3	1	0.044178499	positive regulation of tumor necrosis factor-alpha biosynthesis
GO:0007500	3	1	0.044178499	mesodermal cell fate determination
GO:0009994	3	1	0.044178499	oocyte differentiation
GO:0006266	3	1	0.044178499	DNA ligation
GO:0016998	3	1	0.044178499	cell wall catabolism
GO:0045090	3	1	0.044178499	retroviral genome replication
GO:0045079	3	1	0.044178499	negative regulation of chemokine biosynthesis
GO:0007155	518	13	0.045699737	cell adhesion
GO:0051246	174	6	0.046069117	regulation of protein metabolism
GO:0019932	176	6	0.048232136	second-messenger-mediated signaling
GO:0031325	136	5	0.052896708	positive regulation of cellular metabolism
GO:0006944	25	2	0.053181021	membrane fusion
GO:0006793	641	15	0.055087379	phosphorus metabolism
GO:0006796	641	15	0.055087379	phosphate metabolism
GO:0009966	229	7	0.055701302	regulation of signal transduction
GO:0048522	482	12	0.056514427	positive regulation of cellular process
GO:0009410	26	2	0.057062526	response to xenobiotic stimulus
GO:0007498	26	2	0.057062526	mesoderm development
GO:0008285	139	5	0.05707686	negative regulation of cell proliferation
GO:0045892	97	4	0.057117325	negative regulation of transcription, DNA-dependent
GO:0044248	700	16	0.057540406	cellular catabolism
GO:0048333	4	1	0.058469772	mesodermal cell differentiation
GO:0045410	4	1	0.058469772	positive regulation of interleukin-6 biosynthesis
GO:0045414	4	1	0.058469772	regulation of interleukin-8 biosynthesis
GO:0045416	4	1	0.058469772	positive regulation of interleukin-8 biosynthesis
GO:0045408	4	1	0.058469772	regulation of interleukin-6 biosynthesis
GO:0048144	4	1	0.058469772	fibroblast proliferation
GO:0048145	4	1	0.058469772	regulation of fibroblast proliferation
GO:0045785	4	1	0.058469772	positive regulation of cell adhesion
GO:0050764	4	1	0.058469772	regulation of phagocytosis
GO:0050766	4	1	0.058469772	positive regulation of phagocytosis
GO:0050854	4	1	0.058469772	regulation of antigen receptor mediated signaling pathway
GO:0007171	4	1	0.058469772	transmembrane receptor protein tyrosine kinase activation (dimerization)
GO:0050672	4	1	0.058469772	negative regulation of lymphocyte proliferation
GO:0042226	4	1	0.058469772	interleukin-6 biosynthesis
GO:0051282	4	1	0.058469772	regulation of sequestering of calcium ion
GO:0051283	4	1	0.058469772	negative regulation of sequestering of calcium ion
GO:0051209	4	1	0.058469772	release of sequestered calcium ion into cytoplasm
GO:0042541	4	1	0.058469772	hemoglobin biosynthesis
GO:0006213	4	1	0.058469772	pyrimidine nucleoside metabolism

GO:0006207	4	1	0.058469772	'de novo' pyrimidine base biosynthesis
GO:0001710	4	1	0.058469772	mesodermal cell fate commitment
GO:0006917	140	5	0.058511478	induction of apoptosis
GO:0012502	140	5	0.058511478	induction of programmed cell death
GO:0006470	99	4	0.060685075	protein amino acid dephosphorylation
GO:0000187	27	2	0.061039677	activation of MAPK activity
GO:0006800	61	3	0.062812456	oxygen and reactive oxygen species metabolism
GO:0000122	61	3	0.062812456	negative regulation of transcription from RNA polymerase II promoter
GO:0046651	28	2	0.065108795	lymphocyte proliferation
GO:0051348	28	2	0.065108795	negative regulation of transferase activity
GO:0006469	28	2	0.065108795	negative regulation of protein kinase activity
GO:0006357	240	7	0.068085409	regulation of transcription from RNA polymerase II promoter
GO:0009893	149	5	0.072347549	positive regulation of metabolism
GO:0019369	5	1	0.072548923	arachidonic acid metabolism
GO:0048477	5	1	0.072548923	oogenesis
GO:0045840	5	1	0.072548923	positive regulation of mitosis
GO:0019856	5	1	0.072548923	pyrimidine base biosynthesis
GO:0050716	5	1	0.072548923	positive regulation of interleukin-1 secretion
GO:0050718	5	1	0.072548923	positive regulation of interleukin-1 beta secretion
GO:0050701	5	1	0.072548923	interleukin-1 secretion
GO:0050702	5	1	0.072548923	interleukin-1 beta secretion
GO:0050704	5	1	0.072548923	regulation of interleukin-1 secretion
GO:0050706	5	1	0.072548923	regulation of interleukin-1 beta secretion
GO:0050852	5	1	0.072548923	T cell receptor signaling pathway
GO:0016358	5	1	0.072548923	dendrite morphogenesis
GO:0007263	5	1	0.072548923	nitric oxide mediated signal transduction
GO:0042228	5	1	0.072548923	interleukin-8 biosynthesis
GO:0051208	5	1	0.072548923	sequestering of calcium ion
GO:0046146	5	1	0.072548923	tetrahydrobiopterin metabolism
GO:0020027	5	1	0.072548923	hemoglobin metabolism
GO:0030236	5	1	0.072548923	anti-inflammatory response
GO:0006729	5	1	0.072548923	tetrahydrobiopterin biosynthesis
GO:0045069	5	1	0.072548923	regulation of viral genome replication
GO:0006874	68	3	0.0812443	calcium ion homeostasis
GO:0016311	110	4	0.082352666	dephosphorylation
GO:0043065	155	5	0.082487185	positive regulation of apoptosis
GO:0043068	156	5	0.084247311	positive regulation of programmed cell death
GO:0050789	2689	48	0.084417291	regulation of biological process
GO:0001909	6	1	0.086419079	immune cell mediated cytotoxicity
GO:0051054	6	1	0.086419079	positive regulation of DNA metabolism
GO:0050851	6	1	0.086419079	antigen receptor-mediated signaling pathway
GO:0016478	6	1	0.086419079	negative regulation of translation
GO:0045058	6	1	0.086419079	T cell selection
GO:0006401	33	2	0.086711158	RNA catabolism
GO:0048015	71	3	0.08978273	phosphoinositide-mediated signaling
GO:0030098	34	2	0.091259595	lymphocyte differentiation
GO:0007169	116	4	0.095577858	transmembrane receptor protein tyrosine kinase signaling pathway

GO:0006725	73	3	0.09567567	aromatic compound metabolism
GO:0008283	473	11	0.096164114	cell proliferation
GO:0009056	756	16	0.098244511	catabolism
GO:0048332	7	1	0.100083318	mesoderm morphogenesis
GO:0045787	7	1	0.100083318	positive regulation of progression through cell cycle
GO:0016045	7	1	0.100083318	detection of bacteria
GO:0050792	7	1	0.100083318	regulation of viral life cycle
GO:0050715	7	1	0.100083318	positive regulation of cytokine secretion
GO:0050707	7	1	0.100083318	regulation of cytokine secretion
GO:0009411	7	1	0.100083318	response to UV
GO:0050663	7	1	0.100083318	cytokine secretion
GO:0050673	7	1	0.100083318	epithelial cell proliferation
GO:0050678	7	1	0.100083318	regulation of epithelial cell proliferation
GO:0042743	7	1	0.100083318	hydrogen peroxide metabolism
GO:0042744	7	1	0.100083318	hydrogen peroxide catabolism
GO:0001704	7	1	0.100083318	formation of primary germ layer
GO:0001707	7	1	0.100083318	mesoderm formation
GO:0007167	165	5	0.100971778	enzyme linked receptor protein signaling pathway
GO:0007204	37	2	0.105304246	elevation of cytoplasmic calcium ion concentration
GO:0051480	37	2	0.105304246	cytoplasmic calcium ion homeostasis
GO:0019725	122	4	0.109739705	cell homeostasis
GO:0006558	8	1	0.113544676	L-phenylalanine metabolism
GO:0006559	8	1	0.113544676	L-phenylalanine catabolism
GO:0001541	8	1	0.113544676	ovarian follicle development
GO:0001906	8	1	0.113544676	cell killing
GO:0045807	8	1	0.113544676	positive regulation of endocytosis
GO:0007369	8	1	0.113544676	gastrulation
GO:0050866	8	1	0.113544676	negative regulation of cell activation
GO:0050832	8	1	0.113544676	defense response to fungi
GO:0009791	8	1	0.113544676	post-embryonic development
GO:0051250	8	1	0.113544676	negative regulation of lymphocyte activation
GO:0051238	8	1	0.113544676	sequestering of metal ion
GO:0042542	8	1	0.113544676	response to hydrogen peroxide
GO:0042698	8	1	0.113544676	menstrual cycle
GO:0046112	8	1	0.113544676	nucleobase biosynthesis
GO:0006206	8	1	0.113544676	pyrimidine base metabolism
GO:0046649	81	3	0.120738668	lymphocyte activation
GO:0000165	81	3	0.120738668	MAPKKK cascade
GO:0008610	175	5	0.12135438	lipid biosynthesis
GO:0019882	41	2	0.124853933	antigen presentation
GO:0051249	41	2	0.124853933	regulation of lymphocyte activation
GO:0001818	9	1	0.126806142	negative regulation of cytokine production
GO:0046545	9	1	0.126806142	development of primary female sexual characteristics
GO:0046660	9	1	0.126806142	female sex differentiation
GO:0045766	9	1	0.126806142	positive regulation of angiogenesis
GO:0042036	9	1	0.126806142	negative regulation of cytokine biosynthesis
GO:0008585	9	1	0.126806142	female gonad development

GO:0045638	9	1	0.126806142	negative regulation of myeloid cell differentiation
GO:0050714	9	1	0.126806142	positive regulation of protein secretion
GO:0007159	9	1	0.126806142	leukocyte adhesion
GO:0042100	9	1	0.126806142	B cell proliferation
GO:0007281	9	1	0.126806142	germ cell development
GO:0006268	9	1	0.126806142	DNA unwinding
GO:0007599	83	3	0.127350135	hemostasis
GO:0042060	85	3	0.134088858	wound healing
GO:0050865	43	2	0.134932635	regulation of cell activation
GO:0044265	563	12	0.135546302	cellular macromolecule catabolism
GO:0006508	451	10	0.137153083	proteolysis and peptidolysis
GO:0044257	451	10	0.137153083	cellular protein catabolism
GO:0019438	10	1	0.139870663	aromatic compound biosynthesis
GO:0009074	10	1	0.139870663	aromatic amino acid family catabolism
GO:0042559	10	1	0.139870663	pteridine and derivative biosynthesis
GO:0006979	44	2	0.140038997	response to oxidative stress
GO:0030005	87	3	0.140949266	di-, tri-valent inorganic cation homeostasis
GO:0043086	46	2	0.15037367	negative regulation of enzyme activity
GO:0009057	575	12	0.150850581	macromolecule catabolism
GO:0006957	11	1	0.152741142	complement activation, alternative pathway
GO:0019884	11	1	0.152741142	antigen presentation, exogenous antigen
GO:0009167	11	1	0.152741142	purine ribonucleoside monophosphate metabolism
GO:0009168	11	1	0.152741142	purine ribonucleoside monophosphate biosynthesis
GO:0009126	11	1	0.152741142	purine nucleoside monophosphate metabolism
GO:0009127	11	1	0.152741142	purine nucleoside monophosphate biosynthesis
GO:0042558	11	1	0.152741142	pteridine and derivative metabolism
GO:0009620	11	1	0.152741142	response to fungi
GO:0006171	11	1	0.152741142	cAMP biosynthesis
GO:0030163	463	10	0.15444502	protein catabolism
GO:0006875	93	3	0.162204806	metal ion homeostasis
GO:0006461	194	5	0.164839742	protein complex assembly
GO:0019886	12	1	0.165420439	antigen processing, exogenous antigen via MHC class II
GO:0050708	12	1	0.165420439	regulation of protein secretion
GO:0007422	12	1	0.165420439	peripheral nervous system development
GO:0009112	12	1	0.165420439	nucleobase metabolism
GO:0042102	12	1	0.165420439	positive regulation of T cell proliferation
GO:0046039	12	1	0.165420439	GTP metabolism
GO:0006183	12	1	0.165420439	GTP biosynthesis
GO:0006275	12	1	0.165420439	regulation of DNA replication
GO:0050878	94	3	0.165838427	regulation of body fluids
GO:0051093	49	2	0.16614615	negative regulation of development
GO:0019752	359	8	0.16878422	carboxylic acid metabolism
GO:0043285	474	10	0.171223948	biopolymer catabolism
GO:0018193	50	2	0.171467004	peptidyl-amino acid modification
GO:0006082	361	8	0.172376743	organic acid metabolism
GO:0008037	13	1	0.177911375	cell recognition
GO:0019439	13	1	0.177911375	aromatic compound catabolism

GO:0051047	13	1	0.177911375	positive regulation of secretion
GO:0046058	13	1	0.177911375	cAMP metabolism
GO:0030003	98	3	0.180607669	cation homeostasis
GO:0050791	2407	41	0.186635019	regulation of physiological process
GO:0006366	426	9	0.186841532	transcription from RNA polymerase II promoter
GO:0008632	53	2	0.187590734	apoptotic program
GO:0006584	14	1	0.190216727	catecholamine metabolism
GO:0019835	14	1	0.190216727	cytolysis
GO:0045595	55	2	0.198454745	regulation of cell differentiation
GO:0007186	491	10	0.198795096	G-protein coupled receptor protein signaling pathway
GO:0008015	103	3	0.199546379	circulation
GO:0006873	103	3	0.199546379	cell ion homeostasis
GO:0043407	15	1	0.202339231	negative regulation of MAPK activity
GO:0018958	15	1	0.202339231	phenol metabolism
GO:0051050	15	1	0.202339231	positive regulation of transport
GO:0042129	15	1	0.202339231	regulation of T cell proliferation
GO:0000188	15	1	0.202339231	inactivation of MAPK activity
GO:0007200	56	2	0.203915641	G-protein signaling, coupled to IP3 second messenger (phospholipase C activating)
GO:0042592	157	4	0.207958183	homeostasis
GO:0006809	16	1	0.214281584	nitric oxide biosynthesis
GO:0045637	16	1	0.214281584	regulation of myeloid cell differentiation
GO:0007157	16	1	0.214281584	heterophilic cell adhesion
GO:0007173	16	1	0.214281584	epidermal growth factor receptor signaling pathway
GO:0007202	16	1	0.214281584	phospholipase C activation
GO:0009595	16	1	0.214281584	detection of biotic stimulus
GO:0030100	16	1	0.214281584	regulation of endocytosis
GO:0006284	16	1	0.214281584	base-excision repair
GO:0046209	16	1	0.214281584	nitric oxide metabolism
GO:0051248	58	2	0.214886431	negative regulation of protein metabolism
GO:0009615	58	2	0.214886431	response to virus
GO:0006417	107	3	0.215026941	regulation of protein biosynthesis
GO:0006468	444	9	0.219277393	protein amino acid phosphorylation
GO:0007188	59	2	0.220392953	G-protein signaling, coupled to cAMP nucleotide second messenger
GO:0051244	2319	39	0.221121086	regulation of cellular physiological process
GO:0051336	60	2	0.225911388	regulation of hydrolase activity
GO:0019933	60	2	0.225911388	cAMP-mediated signaling
GO:0006904	17	1	0.226046445	vesicle docking during exocytosis
GO:0017148	17	1	0.226046445	negative regulation of protein biosynthesis
GO:0042098	17	1	0.226046445	T cell proliferation
GO:0019722	17	1	0.226046445	calcium-mediated signaling
GO:0009161	17	1	0.226046445	ribonucleoside monophosphate metabolism
GO:0009156	17	1	0.226046445	ribonucleoside monophosphate biosynthesis
GO:0007292	17	1	0.226046445	female gamete generation
GO:0031326	111	3	0.230755386	regulation of cellular biosynthesis
GO:0050801	111	3	0.230755386	ion homeostasis
GO:0019079	18	1	0.237636432	viral genome replication
GO:0031327	18	1	0.237636432	negative regulation of cellular biosynthesis

GO:0031281	18	1	0.237636432	positive regulation of cyclase activity
GO:0048278	18	1	0.237636432	vesicle docking
GO:0045762	18	1	0.237636432	positive regulation of adenylate cyclase activity
GO:0009072	18	1	0.237636432	aromatic amino acid family metabolism
GO:0009123	18	1	0.237636432	nucleoside monophosphate metabolism
GO:0009124	18	1	0.237636432	nucleoside monophosphate biosynthesis
GO:0007190	18	1	0.237636432	adenylate cyclase activation
GO:0042133	18	1	0.237636432	neurotransmitter metabolism
GO:0050671	18	1	0.237636432	positive regulation of lymphocyte proliferation
GO:0051349	18	1	0.237636432	positive regulation of lyase activity
GO:0030217	18	1	0.237636432	T cell differentiation
GO:0009890	19	1	0.249054125	negative regulation of biosynthesis
GO:0042692	19	1	0.249054125	muscle cell differentiation
GO:0009889	116	3	0.250703303	regulation of biosynthesis
GO:0016310	522	10	0.253593693	phosphorylation
GO:0001525	65	2	0.253628755	angiogenesis
GO:0006261	65	2	0.253628755	DNA-dependent DNA replication
GO:0050794	2488	41	0.257516853	regulation of cellular process
GO:0009190	20	1	0.260302066	cyclic nucleotide biosynthesis
GO:0007205	20	1	0.260302066	protein kinase C activation
GO:0006464	1087	19	0.267221154	protein modification
GO:0006629	469	9	0.267650184	lipid metabolism
GO:0006807	291	6	0.268910153	nitrogen compound metabolism
GO:0001568	68	2	0.270308042	blood vessel development
GO:0043123	68	2	0.270308042	positive regulation of I-kappaB kinase/NF-kappaB cascade
GO:0048514	68	2	0.270308042	blood vessel morphogenesis
GO:0048469	21	1	0.271382761	cell maturation
GO:0019883	21	1	0.271382761	antigen presentation, endogenous antigen
GO:0051016	21	1	0.271382761	barbed-end actin filament capping
GO:0050870	21	1	0.271382761	positive regulation of T cell activation
GO:0001944	69	2	0.275868	vasculature development
GO:0016192	296	6	0.281743373	vesicle-mediated transport
GO:0007189	22	1	0.282298677	G-protein signaling, adenylate cyclase activating pathway
GO:0030042	22	1	0.282298677	actin filament depolymerization
GO:0030834	22	1	0.282298677	regulation of actin filament depolymerization
GO:0030835	22	1	0.282298677	negative regulation of actin filament depolymerization
GO:0006805	23	1	0.293052249	xenobiotic metabolism
GO:0008406	23	1	0.293052249	gonad development
GO:0045137	23	1	0.293052249	development of primary sexual characteristics
GO:0006953	23	1	0.293052249	acute-phase response
GO:0008277	23	1	0.293052249	regulation of G-protein coupled receptor protein signaling pathway
GO:0009116	23	1	0.293052249	nucleoside metabolism
GO:0043122	73	2	0.298076726	regulation of I-kappaB kinase/NF-kappaB cascade
GO:0051046	24	1	0.303645872	regulation of secretion
GO:0008360	25	1	0.314081907	regulation of cell shape
GO:0045765	25	1	0.314081907	regulation of angiogenesis
GO:0051235	25	1	0.314081907	maintenance of localization

GO:0019058	26	1	0.324362682	viral infectious cycle
GO:0009306	26	1	0.324362682	protein secretion
GO:0044419	26	1	0.324362682	interaction between organisms
GO:0007596	78	2	0.325685864	blood coagulation
GO:0050817	79	2	0.331177445	coagulation
GO:0006919	27	1	0.33449049	caspase activation
GO:0016066	27	1	0.33449049	cellular defense response (sensu Vertebrata)
GO:0009187	27	1	0.33449049	cyclic nucleotide metabolism
GO:0009792	27	1	0.33449049	embryonic development (sensu Metazoa)
GO:0006118	256	5	0.336481851	electron transport
GO:0043087	28	1	0.34446759	regulation of GTPase activity
GO:0008217	28	1	0.34446759	regulation of blood pressure
GO:0051251	28	1	0.34446759	positive regulation of lymphocyte activation
GO:0006898	28	1	0.34446759	receptor mediated endocytosis
GO:0044255	383	7	0.348181469	cellular lipid metabolism
GO:0044249	763	13	0.352558477	cellular biosynthesis
GO:0048511	29	1	0.354296208	rhythmic process
GO:0051052	29	1	0.354296208	regulation of DNA metabolism
GO:0050867	29	1	0.354296208	positive regulation of cell activation
GO:0007179	29	1	0.354296208	transforming growth factor beta receptor signaling pathway
GO:0007275	1543	25	0.362140983	development
GO:0006956	30	1	0.363978537	complement activation
GO:0042113	30	1	0.363978537	B cell activation
GO:0051129	30	1	0.363978537	negative regulation of cell organization and biogenesis
GO:0051261	30	1	0.363978537	protein depolymerization
GO:0043412	1160	19	0.370990669	biopolymer modification
GO:0043280	31	1	0.37351674	positive regulation of caspase activity
GO:0043281	31	1	0.37351674	regulation of caspase activity
GO:0050863	31	1	0.37351674	regulation of T cell activation
GO:0031279	32	1	0.382912944	regulation of cyclase activity
GO:0045761	32	1	0.382912944	regulation of adenylate cyclase activity
GO:0051345	32	1	0.382912944	positive regulation of hydrolase activity
GO:0051339	32	1	0.382912944	regulation of lyase activity
GO:0009314	33	1	0.39216925	response to radiation
GO:0008064	33	1	0.39216925	regulation of actin polymerization and/or depolymerization
GO:0006519	216	4	0.404775804	amino acid and derivative metabolism
GO:0009058	859	14	0.40807785	biosynthesis
GO:0006576	35	1	0.410270405	biogenic amine metabolism
GO:0016032	35	1	0.410270405	viral life cycle
GO:0007548	35	1	0.410270405	sex differentiation
GO:0030832	35	1	0.410270405	regulation of actin filament length
GO:0007187	94	2	0.411652475	G-protein signaling, coupled to cyclic nucleotide second messenger
GO:0030333	36	1	0.419119299	antigen processing
GO:0006487	37	1	0.427836383	protein amino acid N-linked glycosylation
GO:0015672	223	4	0.428402031	monovalent inorganic cation transport
GO:0019935	98	2	0.432343678	cyclic-nucleotide-mediated signaling
GO:0007178	38	1	0.436423608	transmembrane receptor protein serine/threonine kinase signaling pathway

GO:0007249	99	2	0.437456841	I-kappaB kinase/NF-kappaB cascade
GO:0007088	39	1	0.444882894	regulation of mitosis
GO:0048468	40	1	0.453216132	cell development
GO:0009165	103	2	0.457658229	nucleotide biosynthesis
GO:0006575	41	1	0.461425187	amino acid derivative metabolism
GO:0001505	41	1	0.461425187	regulation of neurotransmitter levels
GO:0006511	105	2	0.467602905	ubiquitin-dependent protein catabolism
GO:0019941	105	2	0.467602905	modification-dependent protein catabolism
GO:0009063	42	1	0.469511896	amino acid catabolism
GO:0007126	44	1	0.485325489	meiosis
GO:0051321	44	1	0.485325489	meiotic cell cycle
GO:0051327	44	1	0.485325489	M phase of meiotic cell cycle
GO:0046483	44	1	0.485325489	heterocycle metabolism
GO:0007582	7846	118	0.489053362	physiological process
GO:0008154	45	1	0.493055914	actin polymerization and/or depolymerization
GO:0009310	45	1	0.493055914	amine catabolism
GO:0042110	45	1	0.493055914	T cell activation
GO:0044267	2109	32	0.494792246	cellular protein metabolism
GO:0006813	111	2	0.496781818	potassium ion transport
GO:0031175	46	1	0.500671075	neurite morphogenesis
GO:0051049	46	1	0.500671075	regulation of transport
GO:0009142	46	1	0.500671075	nucleoside triphosphate biosynthesis
GO:0009145	46	1	0.500671075	purine nucleoside triphosphate biosynthesis
GO:0009201	46	1	0.500671075	ribonucleoside triphosphate biosynthesis
GO:0009206	46	1	0.500671075	purine ribonucleoside triphosphate biosynthesis
GO:0044270	47	1	0.508172678	nitrogen compound catabolism
GO:0006520	181	3	0.510715906	amino acid metabolism
GO:0006334	48	1	0.515562404	nucleosome assembly
GO:0019538	2129	32	0.519232917	protein metabolism
GO:0009199	49	1	0.522841909	ribonucleoside triphosphate metabolism
GO:0009144	49	1	0.522841909	purine nucleoside triphosphate metabolism
GO:0009205	49	1	0.522841909	purine ribonucleoside triphosphate metabolism
GO:0009141	50	1	0.530012825	nucleoside triphosphate metabolism
GO:0009968	50	1	0.530012825	negative regulation of signal transduction
GO:0051128	52	1	0.5440353	regulation of cell organization and biogenesis
GO:0048513	872	13	0.547205061	organ development
GO:0007267	465	7	0.547678944	cell-cell signaling
GO:0006887	55	1	0.564294032	exocytosis
GO:0030036	126	2	0.565154004	actin cytoskeleton organization and biogenesis
GO:0031497	56	1	0.570846366	chromatin assembly
GO:0007606	56	1	0.570846366	sensory perception of chemical stimulus
GO:0043283	2240	33	0.571818383	biopolymer metabolism
GO:0009308	269	4	0.575040252	amine metabolism
GO:0006817	57	1	0.577300883	phosphate transport
GO:0015992	57	1	0.577300883	proton transport
GO:0006818	58	1	0.583659031	hydrogen transport
GO:0009152	58	1	0.583659031	purine ribonucleotide biosynthesis

GO:0008202	132	2	0.590592036	steroid metabolism
GO:0006694	60	1	0.596091915	steroid biosynthesis
GO:0009150	60	1	0.596091915	purine ribonucleotide metabolism
GO:0007050	61	1	0.602169444	cell cycle arrest
GO:0048609	61	1	0.602169444	reproductive organismal physiological process
GO:0006164	61	1	0.602169444	purine nucleotide biosynthesis
GO:0043170	2405	35	0.606422625	macromolecule metabolism
GO:0050876	62	1	0.608156193	reproductive physiological process
GO:0006163	63	1	0.614053508	purine nucleotide metabolism
GO:0030029	139	2	0.618864518	actin filament-based process
GO:0006445	64	1	0.619862715	regulation of translation
GO:0006260	140	2	0.622779889	DNA replication
GO:0009719	214	3	0.62468037	response to endogenous stimulus
GO:0009260	65	1	0.62558512	ribonucleotide biosynthesis
GO:0009790	65	1	0.62558512	embryonic development
GO:0009117	143	2	0.634341266	nucleotide metabolism
GO:0045786	145	2	0.641895368	negative regulation of progression through cell cycle
GO:0009259	68	1	0.642244309	ribonucleotide metabolism
GO:0007049	601	8	0.683916444	cell cycle
GO:0009888	158	2	0.688045129	histogenesis
GO:0006814	80	1	0.701837073	sodium ion transport
GO:0000074	394	5	0.707629671	regulation of progression through cell cycle
GO:0044260	2298	32	0.710874652	cellular macromolecule metabolism
GO:0050793	168	2	0.720158208	regulation of development
GO:0016055	87	1	0.731932967	Wnt receptor signaling pathway
GO:0016337	172	2	0.732209852	cell-cell adhesion
GO:0006412	407	5	0.734220955	protein biosynthesis
GO:0019222	1629	22	0.735115003	regulation of metabolism
GO:0031323	1566	21	0.742797872	regulation of cellular metabolism
GO:0006886	257	3	0.744095752	intracellular protein transport
GO:0006333	91	1	0.747755796	chromatin assembly or disassembly
GO:0006486	92	1	0.751564427	protein amino acid glycosylation
GO:0043413	93	1	0.755315971	biopolymer glycosylation
GO:0006812	346	4	0.765899174	cation transport
GO:0009101	96	1	0.766236512	glycoprotein biosynthesis
GO:0030001	269	3	0.7715197	metal ion transport
GO:0006811	515	6	0.789592541	ion transport
GO:0045935	105	1	0.796178698	positive regulation of nucleobase, nucleoside, nucleotide and nucleic acid metabolism
GO:0006351	1407	18	0.798609084	transcription, DNA-dependent
GO:0015698	106	1	0.799261019	inorganic anion transport
GO:0006091	446	5	0.803452495	generation of precursor metabolites and energy
GO:0009100	111	1	0.813992169	glycoprotein metabolism
GO:0006350	1499	19	0.817622961	transcription
GO:0001501	113	1	0.819579534	skeletal development
GO:0046903	208	2	0.822118919	secretion
GO:0045449	1433	18	0.823076926	regulation of transcription
GO:0045184	459	5	0.823136137	establishment of protein localization

GO:0009059	464	5	0.830276758	macromolecule biosynthesis
GO:0000004	465	5	0.831676809	biological process unknown
GO:0000398	119	1	0.835361444	nuclear mRNA splicing, via spliceosome
GO:0000375	119	1	0.835361444	RNA splicing, via transesterification reactions
GO:0000377	119	1	0.835361444	RNA splicing, via transesterification reactions with bulged adenosine as nucleophile
GO:0016070	391	4	0.842394891	RNA metabolism
GO:0043037	122	1	0.842730571	translation
GO:0008104	474	5	0.843863649	protein localization
GO:0019219	1458	18	0.844552518	regulation of nucleobase, nucleoside, nucleotide and nucleic acid metabolism
GO:0007276	125	1	0.849772194	gametogenesis
GO:0009581	313	3	0.852300524	detection of external stimulus
GO:0006820	128	1	0.856500765	anion transport
GO:0009887	807	9	0.862932329	organogenesis
GO:0007067	135	1	0.87105983	mitosis
GO:0008380	137	1	0.874943346	RNA splicing
GO:0000087	137	1	0.874943346	M phase of mitotic cell cycle
GO:0044237	5026	69	0.875997449	cellular metabolism
GO:0006355	1359	16	0.880963455	regulation of transcription, DNA-dependent
GO:0009987	8200	119	0.885786377	cellular process
GO:0008152	5262	72	0.895289202	metabolism
GO:0019953	149	1	0.895922957	sexual reproduction
GO:0050953	149	1	0.895922957	sensory perception of light
GO:0007601	149	1	0.895922957	visual perception
GO:0015031	439	4	0.899875428	protein transport
GO:0045045	161	1	0.913404674	secretory pathway
GO:0009653	1051	11	0.926512469	morphogenesis
GO:0000279	173	1	0.927968073	M phase
GO:0006281	174	1	0.929065749	DNA repair
GO:0044238	4820	64	0.931089243	primary metabolism
GO:0007399	385	3	0.93177921	neurogenesis
GO:0007600	287	2	0.931978425	sensory perception
GO:0006397	180	1	0.935311694	mRNA processing
GO:0000902	183	1	0.938226754	cellular morphogenesis
GO:0006325	186	1	0.941011382	establishment and/or maintenance of chromatin architecture
GO:0051179	1857	21	0.944246981	localization
GO:0007010	305	2	0.945875809	cytoskeleton organization and biogenesis
GO:0007268	192	1	0.946212263	synaptic transmission
GO:0006323	193	1	0.947033682	DNA packaging
GO:0019226	198	1	0.950957692	transmission of nerve impulse
GO:0006974	200	1	0.952445238	response to DNA damage stimulus
GO:0000278	204	1	0.955287282	mitotic cell cycle
GO:0016071	205	1	0.95597104	mRNA metabolism
GO:0050875	7018	97	0.956953091	cellular physiological process
GO:0000003	208	1	0.957960658	reproduction
GO:0006396	326	2	0.958696043	RNA processing
GO:0006066	213	1	0.961080276	alcohol metabolism
GO:0051234	1841	20	0.962024386	establishment of localization

GO:0007001	221	1	0.965600504	chromosome organization and biogenesis (sensu Eukaryota)
GO:0046907	448	3	0.966911374	intracellular transport
GO:0006810	1802	19	0.97063046	transport
GO:0051276	236	1	0.972717348	chromosome organization and biogenesis
GO:0050877	475	3	0.976008562	neurophysiological process
GO:0006512	255	1	0.979670423	ubiquitin cycle
GO:0006139	2328	25	0.982166958	nucleobase, nucleoside, nucleotide and nucleic acid metabolism
GO:0006259	489	2	0.995446019	DNA metabolism
GO:0005975	355	1	0.995723493	carbohydrate metabolism
GO:0006996	693	3	0.998525871	organelle organization and biogenesis
GO:0016043	842	4	0.999060152	cell organization and biogenesis
GO:0008150	9032	135	1	biological_process

20.3 GO: Molecular Function

GO ID	genes present in chip	genes present in tissues	p-value	GO term
GO:0004871	1837	45	0.000122810838227154	signal transducer activity
GO:0008367	9	3	0.000233350027434049	bacterial binding
GO:0004872	1060	29	0.000493901229258946	receptor activity
GO:0005529	103	7	0.000710016829986562	sugar binding
GO:0046983	170	9	0.000786164601293622	protein dimerization activity
GO:0003823	30	4	0.000857423863966536	antigen binding
GO:0035091	132	7	0.00298868106550828	phosphoinositide binding
GO:0008329	6	2	0.00299906691050927	pattern recognition receptor activity
GO:0005543	169	8	0.00309816717186389	phospholipid binding
GO:0016810	72	5	0.00379117097104806	hydrolase activity, acting on carbon-nitrogen (but not peptide) bonds
GO:0042834	7	2	0.00415896540019497	peptidoglycan binding
GO:0030246	181	8	0.00469668277723288	carbohydrate binding
GO:0004522	9	2	0.00699559905705462	pancreatic ribonuclease activity
GO:0016892	11	2	0.0104872218696011	endoribonuclease activity, producing 3'-phosphomonoesters
GO:0048503	92	5	0.0106009313208711	GPI anchor binding
GO:0008289	298	10	0.0111321686853177	lipid binding
GO:0008656	12	2	0.0124662696937899	caspase activator activity
GO:0001875	1	1	0.0144638926737234	lipopolysaccharide receptor activity
GO:0008383	1	1	0.0144638926737234	manganese superoxide dismutase activity
GO:0017113	1	1	0.0144638926737234	dihydropyrimidine dehydrogenase (NADP+) activity
GO:0051076	1	1	0.0144638926737234	Gram-positive bacterial binding
GO:0050528	1	1	0.0144638926737234	acyloxyacyl hydrolase activity
GO:0003933	1	1	0.0144638926737234	GTP cyclohydrolase activity
GO:0003934	1	1	0.0144638926737234	GTP cyclohydrolase I activity
GO:0004051	1	1	0.0144638926737234	arachidonate 5-lipoxygenase activity
GO:0016603	1	1	0.0144638926737234	glutaminyl-peptide cyclotransferase activity
GO:0004218	1	1	0.0144638926737234	cathepsin S activity
GO:0030881	1	1	0.0144638926737234	beta-2-microglobulin binding
GO:0030882	1	1	0.0144638926737234	lipid antigen binding
GO:0030884	1	1	0.0144638926737234	exogenous lipid antigen binding
GO:0004796	1	1	0.0144638926737234	thromboxane-A synthase activity
GO:0016894	13	2	0.0145944177828888	endonuclease activity, active with either ribo- or deoxyribonucleic acids and producing 3'-phosphomonoesters
GO:0015026	13	2	0.0145944177828888	coreceptor activity
GO:0030693	14	2	0.0168670035746484	caspase activity
GO:0016505	14	2	0.0168670035746484	apoptotic protease activator activity
GO:0004857	187	7	0.0187340369176129	enzyme inhibitor activity
GO:0016504	15	2	0.0192794664888421	protease activator activity
GO:0004725	75	4	0.0231036568235942	protein tyrosine phosphatase activity
GO:0004197	80	4	0.0284626909669421	cysteine-type endopeptidase activity
GO:0030676	2	1	0.028720075358318	Rac guanyl-nucleotide exchange factor activity
GO:0001530	2	1	0.028720075358318	lipopolysaccharide binding
GO:0004666	2	1	0.028720075358318	prostaglandin-endoperoxide synthase activity

GO:0008368	2	1	0.028720075358318	Gram-negative bacterial binding
GO:0045309	2	1	0.028720075358318	protein phosphorylated amino acid binding
GO:0048306	2	1	0.028720075358318	calcium-dependent protein binding
GO:0005153	2	1	0.028720075358318	interleukin-8 receptor binding
GO:0008934	2	1	0.028720075358318	inositol-1(or 4)-monophosphatase activity
GO:0003938	2	1	0.028720075358318	IMP dehydrogenase activity
GO:0004062	2	1	0.028720075358318	aryl sulfotransferase activity
GO:0004152	2	1	0.028720075358318	dihydroorotate dehydrogenase activity
GO:0004158	2	1	0.028720075358318	dihydroorotate oxidase activity
GO:0015266	2	1	0.028720075358318	protein channel activity
GO:0001784	2	1	0.028720075358318	phosphotyrosine binding
GO:0008233	399	11	0.0297286183015085	peptidase activity
GO:0005001	19	2	0.030240339462816	transmembrane receptor protein tyrosine phosphatase activity
GO:0043028	19	2	0.030240339462816	caspase regulator activity
GO:0019198	19	2	0.030240339462816	transmembrane receptor protein phosphatase activity
GO:0030234	509	13	0.0325871462018921	enzyme regulator activity
GO:0016814	21	2	0.0364486390770304	hydrolase activity, acting on carbon-nitrogen (but not peptide) bonds, in cyclic amidines
GO:0001871	88	4	0.0384597040859279	pattern binding
GO:0004601	22	2	0.0397207132332371	peroxidase activity
GO:0016684	22	2	0.0397207132332371	oxidoreductase activity, acting on peroxide as acceptor
GO:0004873	3	1	0.0427715094290327	asialoglycoprotein receptor activity
GO:0004668	3	1	0.0427715094290327	protein-arginine deiminase activity
GO:0015643	3	1	0.0427715094290327	toxin binding
GO:0005128	3	1	0.0427715094290327	erythropoietin receptor binding
GO:0001948	3	1	0.0427715094290327	glycoprotein binding
GO:0004944	3	1	0.0427715094290327	C5a anaphylatoxin receptor activity
GO:0003796	3	1	0.0427715094290327	lysozyme activity
GO:0019834	3	1	0.0427715094290327	phospholipase A2 inhibitor activity
GO:0016019	3	1	0.0427715094290327	peptidoglycan receptor activity
GO:0051219	3	1	0.0427715094290327	phosphoprotein binding
GO:0042608	3	1	0.0427715094290327	T cell receptor binding
GO:0005509	646	15	0.0462219853258075	calcium ion binding
GO:0004860	25	2	0.0501631441133264	protein kinase inhibitor activity
GO:0005488	7228	113	0.0524414079332191	binding
GO:0008234	98	4	0.0534497885947287	cysteine-type peptidase activity
GO:0005178	26	2	0.0538405489330967	integrin binding
GO:0008047	188	6	0.0550921922666403	enzyme activator activity
GO:0004866	99	4	0.0551008483194313	endopeptidase inhibitor activity
GO:0004942	4	1	0.0566211143460802	anaphylatoxin receptor activity
GO:0019238	4	1	0.0566211143460802	cyclohydrolase activity
GO:0030414	100	4	0.0567793397317332	protease inhibitor activity
GO:0004175	288	8	0.057346145825982	endopeptidase activity
GO:0004180	27	2	0.0576103855065922	carboxypeptidase activity
GO:0046982	61	3	0.0580771230501916	protein heterodimerization activity
GO:0004867	64	3	0.065200114053101	serine-type endopeptidase inhibitor activity
GO:0019210	29	2	0.0654138338238138	kinase inhibitor activity
GO:0019955	65	3	0.0676599589105939	cytokine binding

GO:0008301	5	1	0.0702717682435569	DNA bending activity
GO:0004960	5	1	0.0702717682435569	thromboxane receptor activity
GO:0004957	5	1	0.0702717682435569	prostaglandin E receptor activity
GO:0008243	5	1	0.0702717682435569	plasminogen activator activity
GO:0019863	5	1	0.0702717682435569	IgE binding
GO:0004465	5	1	0.0702717682435569	lipoprotein lipase activity
GO:0004185	5	1	0.0702717682435569	serine carboxypeptidase activity
GO:0008565	67	3	0.0727049370413273	protein transporter activity
GO:0004521	31	2	0.0735472171825977	endoribonuclease activity
GO:0016209	31	2	0.0735472171825977	antioxidant activity
GO:0030674	69	3	0.077913496138598	protein binding, bridging
GO:0005031	6	1	0.0837263085101668	tumor necrosis factor receptor activity
GO:0005484	6	1	0.0837263085101668	SNAP receptor activity
GO:0005154	6	1	0.0837263085101668	epidermal growth factor receptor binding
GO:0004982	6	1	0.0837263085101668	N-formyl peptide receptor activity
GO:0016165	6	1	0.0837263085101668	lipoxygenase activity
GO:0042708	6	1	0.0837263085101668	elastase activity
GO:0016721	6	1	0.0837263085101668	oxidoreductase activity, acting on superoxide radicals as acceptor
GO:0004784	6	1	0.0837263085101668	superoxide dismutase activity
GO:0005515	3709	62	0.0843165837062374	protein binding
GO:0016811	34	2	0.0863113826600888	hydrolase activity, acting on carbon-nitrogen (but not peptide) bonds, in linear amides
GO:0005506	212	6	0.0869733899941224	iron ion binding
GO:0008236	117	4	0.089405058385679	serine-type peptidase activity
GO:0051213	35	2	0.0907046354917691	dioxygenase activity
GO:0016701	35	2	0.0907046354917691	oxidoreductase activity, acting on single donors with incorporation of molecular oxygen
GO:0016702	35	2	0.0907046354917691	oxidoreductase activity, acting on single donors with incorporation of molecular oxygen, incorporation of two atoms of oxygen
GO:0004721	118	4	0.0915567362085695	phosphoprotein phosphatase activity
GO:0005035	7	1	0.0969875323618281	death receptor activity
GO:0043120	7	1	0.0969875323618281	tumor necrosis factor binding
GO:0016791	171	5	0.101988270930856	phosphoric monoester hydrolase activity
GO:0004859	8	1	0.110058197406308	phospholipase inhibitor activity
GO:0017017	8	1	0.110058197406308	MAP kinase phosphatase activity
GO:0004955	8	1	0.110058197406308	prostaglandin receptor activity
GO:0016813	8	1	0.110058197406308	hydrolase activity, acting on carbon-nitrogen (but not peptide) bonds, in linear amidines
GO:0016634	8	1	0.110058197406308	oxidoreductase activity, acting on the CH-CH group of donors, oxygen as acceptor
GO:0008009	40	2	0.113584500139326	chemokine activity
GO:0042379	40	2	0.113584500139326	chemokine receptor binding
GO:0016494	9	1	0.122941022199964	C-X-C chemokine receptor activity
GO:0004016	9	1	0.122941022199964	adenylate cyclase activity
GO:0001664	42	2	0.123114097790868	G-protein-coupled receptor binding
GO:0004888	694	14	0.128376130212496	transmembrane receptor activity
GO:0004953	10	1	0.135638686796734	icosanoid receptor activity
GO:0004954	10	1	0.135638686796734	prostanoid receptor activity
GO:0019958	10	1	0.135638686796734	C-X-C chemokine binding
GO:0016755	10	1	0.135638686796734	transferase activity, transferring amino-acyl groups
GO:0004716	10	1	0.135638686796734	receptor signaling protein tyrosine kinase activity
GO:0004540	45	2	0.137752103226293	ribonuclease activity

GO:0003779	189	5	0.138793519587671	actin binding
GO:0005102	528	11	0.142105561683927	receptor binding
GO:0003700	649	13	0.144950772525803	transcription factor activity
GO:0019887	47	2	0.147713399935643	protein kinase regulator activity
GO:0001653	94	3	0.154935490165415	peptide receptor activity
GO:0008528	94	3	0.154935490165415	peptide receptor activity, G-protein coupled
GO:0019865	12	1	0.160489066343599	immunoglobulin binding
GO:0045012	12	1	0.160489066343599	MHC class II receptor activity
GO:0004879	51	2	0.168046070637453	ligand-dependent nuclear receptor activity
GO:0016787	1424	25	0.172753263521687	hydrolase activity
GO:0005540	14	1	0.184630026812043	hyaluronic acid binding
GO:0016628	14	1	0.184630026812043	oxidoreductase activity, acting on the CH-CH group of donors, NAD or NADP as acceptor
GO:0004182	14	1	0.184630026812043	carboxypeptidase A activity
GO:0004252	105	3	0.194268934806028	serine-type endopeptidase activity
GO:0000146	15	1	0.196440781120843	microfilament motor activity
GO:0004715	15	1	0.196440781120843	non-membrane spanning protein tyrosine kinase activity
GO:0016788	446	9	0.196823214705351	hydrolase activity, acting on ester bonds
GO:0004713	161	4	0.204287827417124	protein-tyrosine kinase activity
GO:0019207	58	2	0.204624377463210	kinase regulator activity
GO:0016564	59	2	0.209925339495018	transcriptional repressor activity
GO:0005544	17	1	0.219555139116826	calcium-dependent phospholipid binding
GO:0016563	62	2	0.225906579122326	transcriptional activator activity
GO:0016849	18	1	0.230863558982626	phosphorus-oxygen lyase activity
GO:0004181	18	1	0.230863558982626	metallocarboxypeptidase activity
GO:0004298	18	1	0.230863558982626	threonine endopeptidase activity
GO:0005249	63	2	0.231254902919885	voltage-gated potassium channel activity
GO:0042578	228	5	0.234458479201967	phosphoric ester hydrolase activity
GO:0004519	65	2	0.241975050285434	endonuclease activity
GO:0004869	19	1	0.242009293400393	cysteine protease inhibitor activity
GO:0005164	20	1	0.252994665959808	tumor necrosis factor receptor binding
GO:0003690	20	1	0.252994665959808	double-stranded DNA binding
GO:0009975	20	1	0.252994665959808	cyclase activity
GO:0008238	68	2	0.258094359812588	exopeptidase activity
GO:0005125	181	4	0.266453916161209	cytokine activity
GO:0005244	124	3	0.266841196695969	voltage-gated ion channel activity
GO:0001637	22	1	0.274493455593028	G-protein chemoattractant receptor activity
GO:0004950	22	1	0.274493455593028	chemokine receptor activity
GO:0042277	127	3	0.278630056133074	peptide binding
GO:0005478	24	1	0.295377865969797	intracellular transporter activity
GO:0019956	24	1	0.295377865969797	chemokine binding
GO:0005057	132	3	0.298384321624303	receptor signaling protein activity
GO:0016712	25	1	0.30559514604715	oxidoreductase activity, acting on paired donors, with incorporation or reduction of molecular oxygen, reduced flavin or flavoprotein as one donor, and incorporation of one atom of oxygen
GO:0005539	77	2	0.306435990316473	glycosaminoglycan binding
GO:0008138	26	1	0.315665329931712	protein tyrosine/serine/threonine phosphatase activity
GO:0003725	26	1	0.315665329931712	double-stranded RNA binding
GO:0004437	26	1	0.315665329931712	inositol or phosphatidylinositol phosphatase activity

GO:0016491	509	9	0.316043200935853	oxidoreductase activity
GO:0030528	963	16	0.316874599249572	transcription regulator activity
GO:0030247	80	2	0.322450315387472	polysaccharide binding
GO:0004722	27	1	0.325590520101909	protein serine/threonine phosphatase activity
GO:0003697	28	1	0.335372789202806	single-stranded DNA binding
GO:0030955	86	2	0.354184833093194	potassium ion binding
GO:0016705	87	2	0.359426818937674	oxidoreductase activity, acting on paired donors, with incorporation or reduction of molecular oxygen
GO:0016860	31	1	0.363882357823785	intramolecular oxidoreductase activity
GO:0008146	32	1	0.373113087016247	sulfotransferase activity
GO:0005267	90	2	0.375058200463457	potassium channel activity
GO:0008092	279	5	0.378178711400878	cytoskeletal protein binding
GO:0016782	36	1	0.408724983373027	transferase activity, transferring sulfur-containing groups
GO:0016627	37	1	0.417309512809590	oxidoreductase activity, acting on the CH-CH group of donors
GO:0008134	293	5	0.418544853483302	transcription factor binding
GO:0005126	38	1	0.425770296398844	hematopoietin/interferon-class (D200-domain) cytokine receptor binding
GO:0005070	40	1	0.442327684715442	SH3/SH2 adaptor activity
GO:0009055	104	2	0.445725626036225	electron carrier activity
GO:0004221	41	1	0.450427756235206	ubiquitin thiolesterase activity
GO:0004518	105	2	0.450608280234869	nuclease activity
GO:0015036	42	1	0.458411016197262	disulfide oxidoreductase activity
GO:0004843	43	1	0.466279137027326	ubiquitin-specific protease activity
GO:0004672	447	7	0.470261660499372	protein kinase activity
GO:0008235	44	1	0.474033767380056	metalloexopeptidase activity
GO:0050660	44	1	0.474033767380056	FAD binding
GO:0005516	112	2	0.484094695999784	calmodulin binding
GO:0000287	251	4	0.493953497745442	magnesium ion binding
GO:0003682	47	1	0.496632852565664	chromatin binding
GO:0004896	48	1	0.503949543775474	hematopoietin/interferon-class (D200-domain) cytokine receptor activity
GO:0003707	48	1	0.503949543775474	steroid hormone receptor activity
GO:0005096	117	2	0.507236523437126	GTPase activator activity
GO:0016790	51	1	0.525272094361058	thiolester hydrolase activity
GO:0019901	54	1	0.54568445996641	protein kinase binding
GO:0015268	274	4	0.563421490815599	alpha-type channel activity
GO:0004553	59	1	0.577786486966372	hydrolase activity, hydrolyzing O-glycosyl compounds
GO:0016298	59	1	0.577786486966372	lipase activity
GO:0008201	60	1	0.583931336660386	heparin binding
GO:0019900	60	1	0.583931336660386	kinase binding
GO:0001584	282	4	0.586362865964091	rhodopsin-like receptor activity
GO:0015267	284	4	0.59199000967093	channel or pore class transporter activity
GO:0003713	140	2	0.604710281812226	transcription coactivator activity
GO:0042803	64	1	0.607635455325526	protein homodimerization activity
GO:0030695	219	3	0.617937910929247	GTPase regulator activity
GO:0008083	144	2	0.620111024001026	growth factor activity
GO:0031420	146	2	0.627638150823968	alkali metal ion binding
GO:0004497	71	1	0.645935819594039	monooxygenase activity
GO:0019899	151	2	0.645952963560553	enzyme binding
GO:0005489	152	2	0.649530098731577	electron transporter activity

GO:0003712	230	3	0.651029759742888	transcription cofactor activity
GO:0016773	532	7	0.655957162818005	phosphotransferase activity, alcohol group as acceptor
GO:0005201	73	1	0.656180252748525	extracellular matrix structural constituent
GO:0046906	74	1	0.661191567188819	tetrapyrrole binding
GO:0020037	74	1	0.661191567188819	heme binding
GO:0016301	609	8	0.662109286918021	kinase activity
GO:0042802	158	2	0.670397612147392	protein self binding
GO:0016798	77	1	0.675794541256039	hydrolase activity, acting on glycosyl bonds
GO:0016616	77	1	0.675794541256039	oxidoreductase activity, acting on the CH-OH group of donors, NAD or NADP as acceptor
GO:0016789	80	1	0.689772477935218	carboxylic ester hydrolase activity
GO:0003714	81	1	0.694297517364976	transcription corepressor activity
GO:0016614	83	1	0.703151943164793	oxidoreductase activity, acting on CH-OH group of donors
GO:0005216	252	3	0.71100585038819	ion channel activity
GO:0043169	2313	31	0.719064467654637	cation binding
GO:0050662	88	1	0.724189736912277	coenzyme binding
GO:0005179	90	1	0.732184231117909	hormone activity
GO:0016853	92	1	0.739948631777303	isomerase activity
GO:0030145	92	1	0.739948631777303	manganese ion binding
GO:0005085	93	1	0.743746609215628	guanyl-nucleotide exchange factor activity
GO:0004930	349	4	0.748917042888301	G-protein coupled receptor activity
GO:0046872	2495	33	0.755549024820571	metal ion binding
GO:0005261	188	2	0.760092496200583	cation channel activity
GO:0008415	98	1	0.761926099720428	acyltransferase activity
GO:0003774	98	1	0.761926099720428	motor activity
GO:0016747	98	1	0.761926099720428	transferase activity, transferring groups other than amino-acyl groups
GO:0048037	100	1	0.768834041472084	cofactor binding
GO:0016746	104	1	0.782058528755999	transferase activity, transferring acyl groups
GO:0003702	197	2	0.782573865833741	RNA polymerase II transcription factor activity
GO:0043167	2556	33	0.805588875060832	ion binding
GO:0015078	112	1	0.806295778051964	hydrogen ion transporter activity
GO:0016772	708	8	0.812999375886192	transferase activity, transferring phosphorus-containing groups
GO:0015077	121	1	0.830376561667145	monovalent inorganic cation transporter activity
GO:0016829	127	1	0.844754312712154	lyase activity
GO:0003824	3409	44	0.850826124273924	catalytic activity
GO:0004674	326	3	0.855826153883706	protein serine/threonine kinase activity
GO:0008237	133	1	0.857921448289604	metallopeptidase activity
GO:0005083	144	1	0.879247153722693	small GTPase regulator activity
GO:0005554	522	5	0.88036777336198	molecular function unknown
GO:0003723	436	4	0.881727847312603	RNA binding
GO:0003677	1384	15	0.914829209437128	DNA binding
GO:0005215	1030	10	0.938966265405113	transporter activity
GO:0046914	1596	17	0.93956704210157	transition metal ion binding
GO:0008324	421	3	0.946887755967346	cation transporter activity
GO:0015075	523	4	0.949175182726448	ion transporter activity
GO:0030554	1028	9	0.968381555753331	adenyl nucleotide binding
GO:0005198	488	3	0.975114027987638	structural molecule activity
GO:0005524	970	8	0.975652310866493	ATP binding

GO:0003676	1990	20	0.978717683285122	nucleic acid binding
GO:0016740	1169	10	0.979910733860483	transferase activity
GO:0005386	398	2	0.981009263223256	carrier activity
GO:0008270	1287	10	0.992977549682414	zinc ion binding
GO:0017076	1248	9	0.995771958500546	purine nucleotide binding
GO:0000166	1432	11	0.99578636182962	nucleotide binding
GO:0003674	9541	138	1	molecular_function

20.4 GO: Cellular Component

GO ID	genes present in chip	genes present in tissues	p-value	GO term
GO:0005887	1057	33	7.67944609652645e-06	integral to plasma membrane
GO:0031226	1062	33	8.49862882143092e-06	intrinsic to plasma membrane
GO:0005886	1472	39	4.43936113589347e-05	plasma membrane
GO:0001533	12	2	0.0120941178862536	cornified envelope
GO:0043020	1	1	0.0142410559221954	NADPH oxidase complex
GO:0005766	1	1	0.0142410559221954	primary lysosome
GO:0042582	1	1	0.0142410559221954	azurophil granule
GO:0016020	3514	62	0.0176988924370361	membrane
GO:0016021	2674	48	0.0339688221329886	integral to membrane
GO:0031224	2681	48	0.0355059052175181	intrinsic to membrane
GO:0008305	24	2	0.0452764714991278	integrin complex
GO:0005625	183	6	0.0463470488748152	soluble fraction
GO:0043235	58	3	0.0493782624371902	receptor complex
GO:0005764	107	4	0.0661162372324776	lysosome
GO:0000323	107	4	0.0661162372324776	lytic vacuole
GO:0005577	5	1	0.0692214541293766	fibrinogen complex
GO:0016461	5	1	0.0692214541293766	unconventional myosin
GO:0005576	949	19	0.0784718172955283	extracellular region
GO:0005773	120	4	0.0917558698373277	vacuole
GO:0008290	7	1	0.0955598757280653	F-actin capping protein complex
GO:0005615	385	9	0.0978122312918327	extracellular space
GO:0005925	8	1	0.108450505540979	focal adhesion
GO:0000228	42	2	0.119988505260946	nuclear chromosome
GO:0005924	9	1	0.121158882852192	cell-substrate adherens junction
GO:0016363	10	1	0.133687563590391	nuclear matrix
GO:0042101	10	1	0.133687563590391	T cell receptor complex
GO:0030055	11	1	0.146039068131760	cell-matrix junction
GO:0005769	13	1	0.170220455302300	early endosome
GO:0005626	15	1	0.193722514793853	insoluble fraction
GO:0005665	16	1	0.205224733615513	DNA-directed RNA polymerase II, core complex
GO:0009986	61	2	0.215545692180727	cell surface
GO:0005768	62	2	0.220792877611057	endosome
GO:0005839	18	1	0.227743135508295	proteasome core complex (sensu Eukaryota)
GO:0005912	18	1	0.227743135508295	adherens junction
GO:0030141	18	1	0.227743135508295	secretory granule
GO:0048471	22	1	0.270898668488154	perinuclear region
GO:0000793	23	1	0.281308356680371	condensed chromosome
GO:0030880	23	1	0.281308356680371	RNA polymerase complex
GO:0000267	698	12	0.290269224662073	cell fraction
GO:0005741	26	1	0.311661083338039	mitochondrial outer membrane
GO:0008021	27	1	0.321493354473728	synaptic vesicle
GO:0005938	28	1	0.331186306552675	cell cortex
GO:0001772	29	1	0.340741897712395	immunological synapse

GO:0019898	37	1	0.412467774236516	extrinsic to membrane
GO:0000502	41	1	0.445371448876244	proteasome complex (sensu Eukaryota)
GO:0016459	43	1	0.461131139228337	myosin
GO:0015629	183	3	0.485794299050738	actin cytoskeleton
GO:0005856	612	9	0.510535586663844	cytoskeleton
GO:0016327	50	1	0.512872107231872	apicolateral plasma membrane
GO:0016023	122	2	0.521484824088137	cytoplasmic membrane-bound vesicle
GO:0019867	52	1	0.526728179683695	outer membrane
GO:0031410	124	2	0.530251501320137	cytoplasmic vesicle
GO:0008076	53	1	0.533508894173957	voltage-gated potassium channel complex
GO:0005829	276	4	0.557150426765393	cytosol
GO:0005681	57	1	0.559681535163669	spliceosome complex
GO:0016591	58	1	0.565993792876357	DNA-directed RNA polymerase II, holoenzyme
GO:0030136	65	1	0.607735853538279	clathrin-coated vesicle
GO:0030135	79	1	0.679642059215274	coated vesicle
GO:0030054	93	1	0.73845444181374	cell junction
GO:0000785	100	1	0.76370814964217	chromatin
GO:0005694	203	2	0.78971147082791	chromosome
GO:0005623	7802	109	0.792986268197246	cell
GO:0005737	2530	32	0.816336740259896	cytoplasm
GO:0005740	119	1	0.820704789358938	mitochondrial membrane
GO:0043228	977	11	0.836168306698426	non-membrane-bound organelle
GO:0043232	977	11	0.836168306698426	intracellular non-membrane-bound organelle
GO:0005624	521	5	0.871718901187755	membrane fraction
GO:0008372	523	5	0.873840567475781	cellular component unknown
GO:0005578	303	2	0.933645266783735	extracellular matrix (sensu Metazoa)
GO:0031012	304	2	0.934441235459196	extracellular matrix
GO:0005794	195	1	0.940933688784375	Golgi apparatus
GO:0005654	221	1	0.9596946839769	nucleoplasm
GO:0030529	254	1	0.97522845259563	ribonucleoprotein complex
GO:0043234	1247	10	0.988506670850559	protein complex
GO:0005739	490	2	0.993874954049267	mitochondrion
GO:0005634	2704	26	0.995734481193046	nucleus
GO:0031090	377	1	0.996032481516386	organelle membrane
GO:0005783	483	1	0.999199446812674	endoplasmic reticulum
GO:0005622	5419	58	0.999858634188651	intracellular
GO:0043227	3955	33	0.999995635733952	membrane-bound organelle
GO:0043231	3955	33	0.999995635733952	intracellular membrane-bound organelle
GO:0043226	4515	40	0.999997213755648	organelle
GO:0043229	4515	40	0.999997213755648	intracellular organelle
GO:0005575	8637	123	1	cellular_component

21. BM-CD105Endothelial

21.1 BM-CD105Endothelial selective genes (126 genes)

Symbol	Description	Chromosome	Chromosome Location	LocusLink	Cytoband	UniGene
ERAF	erythroid associated factor	16	31446703	51327	16p11.2	Hs.274309
XK	X-linked Kx blood group (McLeod syndrome)	X	37430051	7504	Xp21.1	Hs.78919
MINPP1	multiple inositol polyphosphate histidine phosphatase, 1	10	89254632	9562	10q23	Hs.121260
DTL	denticleless homolog (Drosophila)	1	210275753	51514	NA	Hs.632496
KLF1	Kruppel-like factor 1 (erythroid)	19	-12856238	10661	19p13.13-p13.12	Hs.37860
HBD	hemoglobin, delta	11	-5210634	3045	11p15.5	Hs.591988
TOP2A	topoisomerase (DNA) II alpha 170kDa	17	-35798323	7153	17q21-q22	Hs.156346
MLF1IP	MLF1 interacting protein	4	-185852231	79682	4q35.1	Hs.481307 Hs.575032
CA2	carbonic anhydrase II	8	86563497	760	8q22	Hs.155097
AKR1C3	aldo-keto reductase family 1, member C3 (3-alpha hydroxysteroid dehydrogenase, type II)	10	5126567	8644	10p15-p14	Hs.78183
CD36	CD36 molecule (thrombospondin receptor)	7	80105892, 80069458	948	7q11.2	Hs.120949 Hs.633085
VCAM1	vascular cell adhesion molecule 1	1	100957884	7412	1p32-p31	Hs.109225
HMGB2	high-mobility group box 2	4	-174489362	3148	4q31	Hs.434953
GYPB	glycophorin B (MNS blood group)	4	-145136706	2994	4q28-q31	Hs.632594
NFE2	nuclear factor (erythroid-derived 2), 45kDa	12	-52972164	4778	12q13	Hs.75643
MYB	v-myb myeloblastosis viral oncogene homolog (avian)	6	135544145	4602	6q22-q23	Hs.531941
CPOX	coproporphyrinogen oxidase	3	-99780981	1371	3q12	Hs.476982
MYL4	myosin, light polypeptide 4, alkali, atrial, embryonic	17	42641426, 42641712	4635	17q21-qter	Hs.463300
AMMECR1	Alport syndrome, mental retardation, midface hypoplasia and elliptocytosis chromosomal region, gene 1	X	-109324070	9949	Xq22.3	Hs.481208
NMU	neuromedin U	4	-56156161	10874	4q12	Hs.418367
GYPA	glycophorin A (MNS blood group)	4	-145249906	2993	4q28.2-q31.1	Hs.434973
DLG7	discs, large homolog 7 (Drosophila)	14	-54684600	9787	14q22.3	Hs.77695
MCM5	MCM5 minichromosome maintenance deficient 5, cell division cycle 46 (S. cerevisiae)	22	34126127	4174	22q13.1	Hs.517582
PRC1	protein regulator of cytokinesis 1	15	-89310276	9055	15q26.1	Hs.567385
ALAS2	aminolevulinate, delta-, synthase 2 (sideroblastic/hypochromic anemia)	X	-55052213	212	Xp11.21	Hs.522666
PCNA	proliferating cell nuclear antigen	20	-5043599	5111	20pter-p12	Hs.147433
VRK1	vaccinia related kinase 1	14	96333436	7443	14q32	Hs.422662
NUSAP1	nucleolar and spindle associated protein 1	15	39412360	51203	15q15.1	Hs.615092
CDC20	CDC20 cell division cycle 20 homolog (S. cerevisiae)	1	43597214	991	1p34.1	Hs.524947
IGLL1	immunoglobulin lambda-like polypeptide 1	22	-22245313	3543	22q11.23	Hs.348935
APOBEC3B	apolipoprotein B mRNA editing enzyme, catalytic polypeptide-like 3B	22	37708350	9582	22q13.1-q13.2	Hs.226307
SPTA1	spectrin, alpha, erythrocytic 1 (elliptocytosis 2)	1	-156847119	6708	1q21	Hs.119825
LYZ	lysozyme (renal amyloidosis)	12	68028430	4069	12q15	Hs.524579

ZWINT	ZW10 interactor	10	-57787204	11130	10q21-q22	Hs.591363
CXCL12	chemokine (C-X-C motif) ligand 12 (stromal cell-derived factor 1)	10	-132569499	6387	10q11.1	Hs.522891
LMO2	LIM domain only 2 (rhombotin-like 1)	11	-33836698	4005	11p13	Hs.34560
SLC25A37	solute carrier family 25, member 37	8	23442307	51312	8p21.2	Hs.122514 Hs.596025
CA1	carbonic anhydrase I	8	-86427708	759	8q13-q22.1	Hs.23118
DNTT	deoxynucleotidyltransferase, terminal	10	98054074	1791	10q23-q24	Hs.534206
CKS2	CDC28 protein kinase regulatory subunit 2	9	91115932	1164	9q22	Hs.83758
TMEM14B	transmembrane protein 14B	6	10856035	81853	6p25.1-p23	Hs.273077
RRM2	ribonucleotide reductase M2 polypeptide	2	10180185	6241	2p25-p24	Hs.226390
HCAP-G	NA	4	17421622	64151	4p15.33	Hs.446201 Hs.567567
DBF4	DBF4 homolog (S. cerevisiae)	7	87343479	10926	7q21.3	Hs.485380
NBR1	neighbor of BRCA1 gene 1	17	38576771, 38576036, 38576023	4077	17q21.31	Hs.277721 Hs.373818 Hs.546264
SELENBP1	selenium binding protein 1	1	-149603404	8991	1q21-q22	Hs.632460
LXN	latexin	3	-159866899	56925	3q25.32	Hs.478067
TUBG1	tubulin, gamma 1	17	38015219	7283	17q21	Hs.279669
EPB42	erythrocyte membrane protein band 4.2	15	-41276719	2038	15q15-q21	Hs.368642
GYPE	glycophorin E	4	-290027384	2996	4q31.1	Hs.632594
TTK	TTK protein kinase	6	80771077	7272	6q13-q21	Hs.169840
RACGAP1	Rac GTPase activating protein 1	12	-48669214	29127	12q13.13	Hs.505469
S100A8	S100 calcium binding protein A8 (calgranulin A)	1	-151629132	6279	1q21	Hs.416073
CPVL	carboxypeptidase, vitellogenic-like	7	-29001773	54504	7p15-p14	Hs.233389
PSMC6	proteasome (prosome, macropain) 26S subunit, ATPase, 6	14	52243667	5706	14q22.1	Hs.156171 Hs.491779
HBQ1	hemoglobin, theta 1	16	170334	3049	16p13.3	Hs.247921
RNF138	ring finger protein 138	18	27925834, 27926644	51444	18q12.1	Hs.302408 Hs.501040
PSMD14	proteasome (prosome, macropain) 26S subunit, non-ATPase, 14	2	161873185	10213	2q24.2	Hs.567410
PRKAR2B	protein kinase, cAMP-dependent, regulatory, type II, beta	7	106472413	5577	7q22	Hs.433068
TFDP1	transcription factor Dp-1	13	113287056	7027	13q34	Hs.79353
HAT1	histone acetyltransferase 1	2	172487203	8520	2q31.2-q33.1	Hs.632532
TYMS	thymidylate synthetase	18	647650	7298	18p11.32	Hs.592338
GCLM	glutamate-cysteine ligase, modifier subunit	1	-94125177	2730	1p22.1	Hs.315562
LBR	lamin B receptor	1	-223655827	3930	1q42.1	Hs.435166
RHCE	Rh blood group, CcEe antigens	1	-25561327	6006	1p36.11	Hs.449968 Hs.643556
FAM117A	family with sequence similarity 117, member A	17	-45142687	81558	17q21.33	Hs.514308
BUB1B	BUB1 budding uninhibited by benzimidazoles 1 homolog beta (yeast)	15	38240529	701	15q15	Hs.631699
EZH2	enhancer of zeste homolog 2 (Drosophila)	7	-148135407	2146	7q35-q36	Hs.444082
NUDT22	nudix (nucleoside diphosphate linked moiety X)-type motif 22	11	63750337	84304	11q13.1	Hs.172847
MTHFD2	methylenetetrahydrofolate dehydrogenase (NADP+ dependent) 2, methenyltetrahydrofolate cyclohydrolase	2	74279197	10797	2p13.1	Hs.469030
RABGAP1L	RAB GTPase activating protein 1-like	1	173111306, 172395256	9910	1q24	Hs.585378 Hs.591475 Hs.615081

GFI1B	growth factor independent 1B (potential regulator of CDKN1A, translocated in CML)	9	134843918	8328	9q34.13	Hs.553160
PPBP	pro-platelet basic protein (chemokine (C-X-C motif) ligand 7)	4	-75071621	5473	4q12-q13	Hs.2164
PBK	PDZ binding kinase	8	-27723058	55872	8p21.2	Hs.104741
TYROBP	TYRO protein tyrosine kinase binding protein	19	-41087145	7305	19q13.1	Hs.515369
PAK1IP1	PAK1 interacting protein 1	6	10803173	55003	6p24.2	Hs.310231
SMC4	structural maintenance of chromosomes 4	3	161600117, 161600123, 161601040	10051	3q26.1	Hs.58992
PPOX	protoporphyrinogen oxidase	1	159402817	5498	1q22	Hs.517373
PAICS	phosphoribosylaminoimidazole carboxylase, phosphoribosylaminoimidazole succinocarboxamide synthetase	4	56996697	10606	4pter-q21	Hs.518774
GMNN	geminin, DNA replication inhibitor	6	24883142	51053	6p22.2	Hs.234896
MAD2L1	MAD2 mitotic arrest deficient-like 1 (yeast)	4	-121200039	4085	4q27	Hs.591697
FECH	ferrochelatase (protoporphyria)	18	-53366534	2235	18q21.3	Hs.465221
CCNE1	cyclin E1	19	34994740, 34995400	898	19q12	Hs.244723
EXOSC8	exosome component 8	13	36472916	11340	13q13.1	Hs.294041
S100A9	S100 calcium binding protein A9 (calgranulin B)	1	151596953	6280	1q21	Hs.112405
POLE2	polymerase (DNA directed), epsilon 2 (p59 subunit)	14	-49180030	5427	14q21-q22	Hs.162777
MCM6	MCM6 minichromosome maintenance deficient 6 (MIS5 homolog, S. pombe) (S. cerevisiae)	2	-136313673	4175	2q21	Hs.444118
DCK	deoxycytidine kinase	4	72078255	1633	4q13.3-q21.1	Hs.709
KIF4A	kinesin family member 4A	X	69426686	24137	Xq13.1	Hs.438712
FCN1	ficolin (collagen/fibrinogen domain containing) 1	9	-136941256	2219	9q34	Hs.440898
PRDX2	peroxiredoxin 2	19	-25541060	7001	19p13.2	Hs.631612
FOSB	FBJ murine osteosarcoma viral oncogene homolog B	19	50663092	2354	19q13.32	Hs.590958
NMI	N-myc (and STAT) interactor	2	-151835230	9111	2p24.3-q21.3	Hs.54483
NUDT1	nudix (nucleoside diphosphate linked moiety X)-type motif 1	7	2248382, 2248421, 2249065	4521	7p22	Hs.534331
HMBS	hydroxymethylbilane synthase	11	118460796, 118463906	3145	11q23.3	Hs.82609
NOLA1	nucleolar protein family A, member 1 (H/ACA small nucleolar RNPs)	4	110956114	54433	4q25	Hs.69851
SMC2	structural maintenance of chromosomes 2	9	105896651, 105896361	10592	9q31.1	Hs.119023
CETN3	centrin, EF-hand protein, 3 (CDC31 homolog, yeast)	5	-89725286	1070	5q14.3	Hs.591767
MTHFD1	methylenetetrahydrofolate dehydrogenase (NADP+ dependent) 1, methenyltetrahydrofolate cyclohydrolase, formyltetrahydrofolate synthetase	14	63924845	4522	14q24	Hs.632340
TRAK2	trafficking protein, kinesin binding 2	2	-201950175	66008	2q33	Hs.152774
MCM2	MCM2 minichromosome maintenance deficient 2, mitotin (S. cerevisiae)	3	128799942	4171	3q21	Hs.477481
BLVRB	biliverdin reductase B (flavin reductase (NADPH))	19	-45645540	645	19q13.1-q13.2	Hs.515785
REXO2	REX2, RNA exonuclease 2 homolog (S. cerevisiae)	11	113815386	25996	11q23.1-q23.2	Hs.7527
NAT13	N-acetyltransferase 13	3	-114920530	80218	3q13.2	Hs.269528
TAL1	T-cell acute lymphocytic leukemia 1	1	-47454550	6886	1p32	Hs.73828
CCNB2	cyclin B2	15	57184611	9133	15q22.2	Hs.194698

KCNH2	potassium voltage-gated channel, subfamily H (eag-related), member 2	7	-300550428	3757	7q35-q36	Hs.438823
UROD	uroporphyrinogen decarboxylase	1	45250416	7389	1p34	Hs.78601
PRPS2	phosphoribosyl pyrophosphate synthetase 2	X	12719413	5634	Xp22.3-p22.2	Hs.104123
PRPF40A	PRP40 pre-mRNA processing factor 40 homolog A (yeast)	2	NA	55660	2q23.3	Hs.591637 Hs.643580
NUCB2	nucleobindin 2	11	17254887	4925	11p15.1-p14	Hs.128686
AURKA	aurora kinase A	20	-54377851	6790	20q13.2-q13.3	Hs.250822
SHCBP1	SHC SH2-domain binding protein 1	16	-45171970	79801	16q11.2	Hs.123253
ATPIF1	ATPase inhibitory factor 1	1	28435197	93974	NA	Hs.590908
ALDH1A1	aldehyde dehydrogenase 1 family, member A1	9	-74705407	216	9q21.13	Hs.76392
KIAA0101	KIAA0101	15	-62444265	9768	15q22.31	Hs.81892
SSBP1	single-stranded DNA binding protein 1	7	141084644	6742	7q34	Hs.490394
TFRC	transferrin receptor (p90, CD71)	3	-197260746	7037	3q29	Hs.529618
ITGA4	integrin, alpha 4 (antigen CD49D, alpha 4 subunit of VLA-4 receptor)	2	182029863	3676	2q31.3	Hs.440955
TACSTD1	tumor-associated calcium signal transducer 1	2	47449970	4072	2p21	Hs.542050
SLC22A4	solute carrier family 22 (organic cation transporter), member 4	5	131658043	6583	5q31.1	Hs.310591 Hs.640104
RRM1	ribonucleotide reductase M1 polypeptide	11	4072586	6240	11p15.5	Hs.558393
CCNB1	cyclin B1	5	68498668	891	5q12	Hs.23960
MNDA	myeloid cell nuclear differentiation antigen	1	157067791	4332	1q22	Hs.153837
NUP37	nucleoporin 37kDa	12	-100992104	79023	12q23.2	Hs.444276
SNCA	synuclein, alpha (non A4 component of amyloid precursor)	4	-181731784	6622	4q21	Hs.271771

21.2 GO: Biological Process

GO ID	genes present in chip	genes present in tissues	p-value	GO term
GO:0007049	601	30	4.58E-12	cell cycle
GO:0000278	204	18	1.86E-11	mitotic cell cycle
GO:0007067	135	15	3.87E-11	mitosis
GO:0000087	137	15	4.79E-11	M phase of mitotic cell cycle
GO:0006260	140	14	7.52E-10	DNA replication
GO:0006783	11	6	9.98E-10	heme biosynthesis
GO:0000279	173	15	1.33E-09	M phase
GO:0046483	44	9	1.51E-09	heterocycle metabolism
GO:0007051	21	7	2.55E-09	spindle organization and biogenesis
GO:0006779	13	6	3.64E-09	porphyrin biosynthesis
GO:0051301	142	13	9.41E-09	cell division
GO:0042168	15	6	1.04E-08	heme metabolism
GO:0006778	18	6	3.75E-08	porphyrin metabolism
GO:0046148	20	6	7.69E-08	pigment biosynthesis
GO:0042440	24	6	2.57E-07	pigment metabolism
GO:0019748	26	6	4.31E-07	secondary metabolism
GO:0051188	85	9	5.88E-07	cofactor biosynthesis
GO:0048015	71	8	1.59E-06	phosphoinositide-mediated signaling
GO:0000226	52	7	2.18E-06	microtubule cytoskeleton organization and biogenesis
GO:0006261	65	7	1.00E-05	DNA-dependent DNA replication
GO:0006259	489	18	1.33E-05	DNA metabolism
GO:0051186	143	9	4.33E-05	cofactor metabolism
GO:0007017	112	8	4.78E-05	microtubule-based process
GO:0000074	394	15	5.16E-05	regulation of progression through cell cycle
GO:0000070	20	4	7.51E-05	mitotic sister chromatid segregation
GO:0000819	21	4	9.20E-05	sister chromatid segregation
GO:0006996	693	20	0.000136299	organelle organization and biogenesis
GO:0007076	11	3	0.000242104	mitotic chromosome condensation
GO:0030261	12	3	0.000320055	chromosome condensation
GO:0006730	30	4	0.000388333	one-carbon compound metabolism
GO:0007093	13	3	0.000412527	mitotic checkpoint
GO:0007052	14	3	0.000520565	mitotic spindle organization and biogenesis
GO:0007059	34	4	0.000633907	chromosome segregation
GO:0016043	842	21	0.00066859	cell organization and biogenesis
GO:0007010	305	11	0.000868079	cytoskeleton organization and biogenesis
GO:0006270	17	3	0.000947881	DNA replication initiation
GO:0019932	176	8	0.001054184	second-messenger-mediated signaling
GO:0007088	39	4	0.001074852	regulation of mitosis
GO:0009123	18	3	0.001127793	nucleoside monophosphate metabolism
GO:0009124	18	3	0.001127793	nucleoside monophosphate biosynthesis
GO:0009396	5	2	0.001333343	folic acid and derivative biosynthesis
GO:0009117	143	7	0.001422478	nucleotide metabolism
GO:0009262	7	2	0.002757321	deoxyribonucleotide metabolism

GO:0006760	8	2	0.003648325	folic acid and derivative metabolism
GO:0006268	9	2	0.00465488	DNA unwinding
GO:0050875	7018	93	0.006072022	cellular physiological process
GO:0015669	12	2	0.008340259	gas transport
GO:0015671	12	2	0.008340259	oxygen transport
GO:0006275	12	2	0.008340259	regulation of DNA replication
GO:0000075	37	3	0.009139403	cell cycle checkpoint
GO:0044237	5026	71	0.011082472	cellular metabolism
GO:0006826	14	2	0.011325181	iron ion transport
GO:0009076	1	1	0.01173605	histidine family amino acid biosynthesis
GO:0009162	1	1	0.01173605	deoxyribonucleoside monophosphate metabolism
GO:0009157	1	1	0.01173605	deoxyribonucleoside monophosphate biosynthesis
GO:0009176	1	1	0.01173605	pyrimidine deoxyribonucleoside monophosphate metabolism
GO:0009177	1	1	0.01173605	pyrimidine deoxyribonucleoside monophosphate biosynthesis
GO:0009118	1	1	0.01173605	regulation of nucleoside metabolism
GO:0045978	1	1	0.01173605	negative regulation of nucleoside metabolism
GO:0009221	1	1	0.01173605	pyrimidine deoxyribonucleotide biosynthesis
GO:0007108	1	1	0.01173605	cytokinesis, initiation of separation
GO:0000114	1	1	0.01173605	G1-specific transcription in mitotic cell cycle
GO:0000105	1	1	0.01173605	histidine biosynthesis
GO:0000212	1	1	0.01173605	meiotic spindle organization and biogenesis
GO:0046073	1	1	0.01173605	dTMP metabolism
GO:0051103	1	1	0.01173605	DNA ligation during DNA repair
GO:0006288	1	1	0.01173605	base-excision repair, DNA ligation
GO:0006231	1	1	0.01173605	dTMP biosynthesis
GO:0000920	1	1	0.01173605	cell separation during cytokinesis
GO:0000912	1	1	0.01173605	cytokinesis, formation of actomyosin apparatus
GO:0000915	1	1	0.01173605	cytokinesis, contractile ring formation
GO:0006139	2328	38	0.013433145	nucleobase, nucleoside, nucleotide and nucleic acid metabolism
GO:0051329	79	4	0.013740557	interphase of mitotic cell cycle
GO:0051325	82	4	0.015576018	interphase
GO:0006281	174	6	0.016398873	DNA repair
GO:0009161	17	2	0.016543133	ribonucleoside monophosphate metabolism
GO:0009156	17	2	0.016543133	ribonucleoside monophosphate biosynthesis
GO:0000910	18	2	0.01847005	cytokinesis
GO:0000096	20	2	0.022590978	sulfur amino acid metabolism
GO:0046653	2	1	0.023335649	tetrahydrofolate metabolism
GO:0008228	2	1	0.023335649	opsonization
GO:0045842	2	1	0.023335649	positive regulation of mitotic metaphase/anaphase transition
GO:0007079	2	1	0.023335649	mitotic chromosome movement towards spindle pole
GO:0000022	2	1	0.023335649	mitotic spindle elongation
GO:0009186	2	1	0.023335649	deoxyribonucleoside diphosphate metabolism
GO:0007405	2	1	0.023335649	neuroblast proliferation
GO:0009263	2	1	0.023335649	deoxyribonucleotide biosynthesis
GO:0051305	2	1	0.023335649	chromosome movement towards spindle pole
GO:0051231	2	1	0.023335649	spindle elongation
GO:0006475	2	1	0.023335649	internal protein amino acid acetylation

GO:0007582	7846	99	0.024423311	physiological process
GO:0006950	881	17	0.027119572	response to stress
GO:0009116	23	2	0.029405748	nucleoside metabolism
GO:0006974	200	6	0.030047716	response to DNA damage stimulus
GO:0044272	24	2	0.031837361	sulfur compound biosynthesis
GO:0009165	103	4	0.032719115	nucleotide biosynthesis
GO:0008360	25	2	0.034345403	regulation of cell shape
GO:0043249	3	1	0.034800369	erythrocyte maturation
GO:0009129	3	1	0.034800369	pyrimidine nucleoside monophosphate metabolism
GO:0009113	3	1	0.034800369	purine base biosynthesis
GO:0009130	3	1	0.034800369	pyrimidine nucleoside monophosphate biosynthesis
GO:0009132	3	1	0.034800369	nucleoside diphosphate metabolism
GO:0046040	3	1	0.034800369	IMP metabolism
GO:0006188	3	1	0.034800369	IMP biosynthesis
GO:0006189	3	1	0.034800369	'de novo' IMP biosynthesis
GO:0006266	3	1	0.034800369	DNA ligation
GO:0016998	3	1	0.034800369	cell wall catabolism
GO:0006220	26	2	0.03692778	pyrimidine nucleotide metabolism
GO:0009719	214	6	0.039816486	response to endogenous stimulus
GO:0008152	5262	71	0.040364286	metabolism
GO:0051052	29	2	0.045100566	regulation of DNA metabolism
GO:0008089	4	1	0.046131763	anterograde axon cargo transport
GO:0008156	4	1	0.046131763	negative regulation of DNA replication
GO:0006940	4	1	0.046131763	regulation of smooth muscle contraction
GO:0009086	4	1	0.046131763	methionine biosynthesis
GO:0007094	4	1	0.046131763	mitotic spindle checkpoint
GO:0009219	4	1	0.046131763	pyrimidine deoxyribonucleotide metabolism
GO:0006144	4	1	0.046131763	purine base metabolism
GO:0008283	473	10	0.050500799	cell proliferation
GO:0000041	31	2	0.050884084	transition metal ion transport
GO:0009108	72	3	0.052350042	coenzyme biosynthesis
GO:0006725	73	3	0.054143703	aromatic compound metabolism
GO:0043283	2240	34	0.054215492	biopolymer metabolism
GO:0008064	33	2	0.056917858	regulation of actin polymerization and/or depolymerization
GO:0006547	5	1	0.057331368	histidine metabolism
GO:0006534	5	1	0.057331368	cysteine metabolism
GO:0015695	5	1	0.057331368	organic cation transport
GO:0050000	5	1	0.057331368	chromosome localization
GO:0009075	5	1	0.057331368	histidine family amino acid metabolism
GO:0045840	5	1	0.057331368	positive regulation of mitosis
GO:0051053	5	1	0.057331368	negative regulation of DNA metabolism
GO:0051303	5	1	0.057331368	establishment of chromosome localization
GO:0020027	5	1	0.057331368	hemoglobin metabolism
GO:0006750	5	1	0.057331368	glutathione biosynthesis
GO:0051276	236	6	0.058877513	chromosome organization and biogenesis
GO:0042742	34	2	0.060024012	defense response to bacteria
GO:0030832	35	2	0.063187302	regulation of actin filament length

GO:0031032	6	1	0.068400702	actomyosin structure organization and biogenesis
GO:0000904	6	1	0.068400702	cellular morphogenesis during differentiation
GO:0008088	7	1	0.079341265	axon cargo transport
GO:0006555	7	1	0.079341265	methionine metabolism
GO:0045787	7	1	0.079341265	positive regulation of progression through cell cycle
GO:0009067	7	1	0.079341265	aspartate family amino acid biosynthesis
GO:0006265	7	1	0.079341265	DNA topological change
GO:0009613	458	9	0.088627799	response to pest, pathogen or parasite
GO:0007098	8	1	0.090154542	centrosome cycle
GO:0000097	8	1	0.090154542	sulfur amino acid biosynthesis
GO:0030071	8	1	0.090154542	regulation of mitotic metaphase/anaphase transition
GO:0007589	8	1	0.090154542	fluid secretion
GO:0046112	8	1	0.090154542	nucleobase biosynthesis
GO:0006749	8	1	0.090154542	glutathione metabolism
GO:0043207	463	9	0.093282187	response to external biotic stimulus
GO:0006979	44	2	0.093951401	response to oxidative stress
GO:0000086	44	2	0.093951401	G2/M transition of mitotic cell cycle
GO:0008154	45	2	0.097595525	actin polymerization and/or depolymerization
GO:0050878	94	3	0.09819098	regulation of body fluids
GO:0007091	9	1	0.100842	mitotic metaphase/anaphase transition
GO:0000160	9	1	0.100842	two-component signal transduction system (phosphorelay)
GO:0051297	9	1	0.100842	centrosome organization and biogenesis
GO:0031023	9	1	0.100842	microtubule organizing center organization and biogenesis
GO:0009611	341	7	0.105604233	response to wounding
GO:0006334	48	2	0.108760429	nucleosome assembly
GO:0006790	48	2	0.108760429	sulfur metabolism
GO:0006752	48	2	0.108760429	group transfer coenzyme metabolism
GO:0009892	217	5	0.11151438	negative regulation of metabolism
GO:0045934	159	4	0.116708737	negative regulation of nucleobase, nucleoside, nucleotide and nucleic acid metabolism
GO:0009617	51	2	0.120245333	response to bacteria
GO:0008015	103	3	0.12039568	circulation
GO:0009167	11	1	0.121845242	purine ribonucleoside monophosphate metabolism
GO:0009168	11	1	0.121845242	purine ribonucleoside monophosphate biosynthesis
GO:0009126	11	1	0.121845242	purine nucleoside monophosphate metabolism
GO:0009127	11	1	0.121845242	purine nucleoside monophosphate biosynthesis
GO:0051128	52	2	0.124138591	regulation of cell organization and biogenesis
GO:0006511	105	3	0.125564301	ubiquitin-dependent protein catabolism
GO:0019941	105	3	0.125564301	modification-dependent protein catabolism
GO:0000082	53	2	0.128062214	G1/S transition of mitotic cell cycle
GO:0009987	8200	100	0.131858502	cellular process
GO:0009112	12	1	0.132163876	nucleobase metabolism
GO:0031497	56	2	0.14000309	chromatin assembly
GO:0006081	13	1	0.142362394	aldehyde metabolism
GO:0030218	13	1	0.142362394	erythrocyte differentiation
GO:0006357	240	5	0.151277491	regulation of transcription from RNA polymerase II promoter
GO:0007018	59	2	0.152174381	microtubule-based movement
GO:0030705	59	2	0.152174381	cytoskeleton-dependent intracellular transport

GO:0019835	14	1	0.15244218	cytolysis
GO:0006800	61	2	0.160401657	oxygen and reactive oxygen species metabolism
GO:0000122	61	2	0.160401657	negative regulation of transcription from RNA polymerase II promoter
GO:0006164	61	2	0.160401657	purine nucleotide biosynthesis
GO:0006909	15	1	0.162404602	phagocytosis
GO:0009066	15	1	0.162404602	aspartate family amino acid metabolism
GO:0007020	15	1	0.162404602	microtubule nucleation
GO:0018149	15	1	0.162404602	peptide cross-linking
GO:0006879	15	1	0.162404602	iron ion homeostasis
GO:0031324	183	4	0.167764457	negative regulation of cellular metabolism
GO:0006163	63	2	0.168709469	purine nucleotide metabolism
GO:0006732	121	3	0.169548304	coenzyme metabolism
GO:0006473	16	1	0.172251016	protein amino acid acetylation
GO:0006284	16	1	0.172251016	base-excision repair
GO:0006954	185	4	0.172339925	inflammatory response
GO:0006364	64	2	0.172891022	rRNA processing
GO:0009260	65	2	0.177089686	ribonucleotide biosynthesis
GO:0016072	66	2	0.181304498	rRNA metabolism
GO:0015758	17	1	0.181982759	glucose transport
GO:0006692	17	1	0.181982759	prostanoid metabolism
GO:0006693	17	1	0.181982759	prostaglandin metabolism
GO:0044249	763	12	0.182525965	cellular biosynthesis
GO:0030036	126	3	0.18411952	actin cytoskeleton organization and biogenesis
GO:0009259	68	2	0.189778801	ribonucleotide metabolism
GO:0006323	193	4	0.191066267	DNA packaging
GO:0015749	18	1	0.191601153	monosaccharide transport
GO:0000080	18	1	0.191601153	G1 phase of mitotic cell cycle
GO:0008645	18	1	0.191601153	hexose transport
GO:0009416	18	1	0.191601153	response to light
GO:0006221	18	1	0.191601153	pyrimidine nucleotide biosynthesis
GO:0006939	19	1	0.201107508	smooth muscle contraction
GO:0006304	19	1	0.201107508	DNA modification
GO:0006936	132	3	0.202026383	muscle contraction
GO:0046916	20	1	0.210503114	transition metal ion homeostasis
GO:0048469	21	1	0.219789252	cell maturation
GO:0051016	21	1	0.219789252	barbed-end actin filament capping
GO:0051318	21	1	0.219789252	G1 phase
GO:0030029	139	3	0.223411436	actin filament-based process
GO:0051244	2319	31	0.228872512	regulation of cellular physiological process
GO:0006937	22	1	0.228967183	regulation of muscle contraction
GO:0030042	22	1	0.228967183	actin filament depolymerization
GO:0030834	22	1	0.228967183	regulation of actin filament depolymerization
GO:0030835	22	1	0.228967183	negative regulation of actin filament depolymerization
GO:0007242	879	13	0.22928179	intracellular signaling cascade
GO:0006366	426	7	0.233225776	transcription from RNA polymerase II promoter
GO:0050791	2407	32	0.23377157	regulation of physiological process
GO:0007046	79	2	0.237162248	ribosome biogenesis

GO:0008016	23	1	0.238038157	regulation of heart contraction rate
GO:0009069	23	1	0.238038157	serine family amino acid metabolism
GO:0007015	23	1	0.238038157	actin filament organization
GO:0042254	80	2	0.241512378	ribosome biogenesis and assembly
GO:0007599	83	2	0.254583377	hemostasis
GO:0007001	221	4	0.260945121	chromosome organization and biogenesis (sensu Eukaryota)
GO:0008643	26	1	0.264621615	carbohydrate transport
GO:0006968	87	2	0.272034933	cellular defense response
GO:0030097	87	2	0.272034933	hemopoiesis
GO:0030005	87	2	0.272034933	di-, tri-valent inorganic cation homeostasis
GO:0048534	88	2	0.276397869	hemopoietic or lymphoid organ development
GO:0007259	28	1	0.281831398	JAK-STAT cascade
GO:0006333	91	2	0.289477416	chromatin assembly or disassembly
GO:0030521	29	1	0.29028607	androgen receptor signaling pathway
GO:0006941	29	1	0.29028607	striated muscle contraction
GO:0006875	93	2	0.298183878	metal ion homeostasis
GO:0051129	30	1	0.298642138	negative regulation of cell organization and biogenesis
GO:0051261	30	1	0.298642138	protein depolymerization
GO:0050794	2488	32	0.303331602	regulation of cellular process
GO:0009607	857	12	0.303480342	response to biotic stimulus
GO:0009058	859	12	0.306293212	biosynthesis
GO:0007028	96	2	0.311213252	cytoplasm organization and biogenesis
GO:0045892	97	2	0.315546309	negative regulation of transcription, DNA-dependent
GO:0030003	98	2	0.319873542	cation homeostasis
GO:0006836	33	1	0.323130038	neurotransmitter transport
GO:0006690	33	1	0.323130038	icosanoid metabolism
GO:0009314	33	1	0.323130038	response to radiation
GO:0015674	99	2	0.324194494	di-, tri-valent inorganic cation transport
GO:0008652	34	1	0.331102948	amino acid biosynthesis
GO:0006935	101	2	0.332815779	chemotaxis
GO:0042330	101	2	0.332815779	taxis
GO:0006873	103	2	0.341406732	cell ion homeostasis
GO:0000079	36	1	0.346770729	regulation of cyclin dependent protein kinase activity
GO:0030099	37	1	0.354467739	myeloid cell differentiation
GO:0051258	37	1	0.354467739	protein polymerization
GO:0030518	38	1	0.362074901	steroid hormone receptor signaling pathway
GO:0030522	39	1	0.369593253	intracellular receptor-mediated signaling pathway
GO:0006325	186	3	0.373461764	establishment and/or maintenance of chromatin architecture
GO:0050801	111	2	0.375404414	ion homeostasis
GO:0048468	40	1	0.377023823	cell development
GO:0007588	40	1	0.377023823	excretion
GO:0006916	112	2	0.379606994	anti-apoptosis
GO:0006952	751	10	0.386680336	defense response
GO:0030001	269	4	0.388580981	metal ion transport
GO:0050896	1578	20	0.390640005	response to stimulus
GO:0006897	115	2	0.392144039	endocytosis
GO:0006865	43	1	0.398798933	amino acid transport

GO:0007126	44	1	0.405888411	meiosis
GO:0051321	44	1	0.405888411	meiotic cell cycle
GO:0051327	44	1	0.405888411	M phase of meiotic cell cycle
GO:0000398	119	2	0.408684252	nuclear mRNA splicing, via spliceosome
GO:0000375	119	2	0.408684252	RNA splicing, via transesterification reactions
GO:0000377	119	2	0.408684252	RNA splicing, via transesterification reactions with bulged adenosine as nucleophile
GO:0006955	684	9	0.411324464	immune response
GO:0007229	45	1	0.412895067	integrin-mediated signaling pathway
GO:0019752	359	5	0.413771757	carboxylic acid metabolism
GO:0050789	2689	33	0.415034176	regulation of biological process
GO:0006082	361	5	0.418454072	organic acid metabolism
GO:0019725	122	2	0.4209481	cell homeostasis
GO:0006468	444	6	0.422049444	protein amino acid phosphorylation
GO:0044238	4820	58	0.428435407	primary metabolism
GO:0006631	124	2	0.42905302	fatty acid metabolism
GO:0009309	49	1	0.440112488	amine biosynthesis
GO:0007586	49	1	0.440112488	digestion
GO:0044271	49	1	0.440112488	nitrogen compound biosynthesis
GO:0043066	128	2	0.445084522	negative regulation of apoptosis
GO:0009968	50	1	0.446719198	negative regulation of signal transduction
GO:0019219	1458	18	0.447384098	regulation of nucleobase, nucleoside, nucleotide and nucleic acid metabolism
GO:0043069	129	2	0.449054052	negative regulation of programmed cell death
GO:0019222	1629	20	0.450933663	regulation of metabolism
GO:0009605	963	12	0.458816336	response to external stimulus
GO:0015837	52	1	0.459701814	amine transport
GO:0051239	133	2	0.464773205	regulation of organismal physiological process
GO:0048523	640	8	0.480113344	negative regulation of cellular process
GO:0008380	137	2	0.480230053	RNA splicing
GO:0007218	56	1	0.484768516	neuropeptide signaling pathway
GO:0006817	57	1	0.490853023	phosphate transport
GO:0009152	58	1	0.496866347	purine ribonucleotide biosynthesis
GO:0051248	58	1	0.496866347	negative regulation of protein metabolism
GO:0009615	58	1	0.496866347	response to virus
GO:0015849	59	1	0.502809312	organic acid transport
GO:0046942	59	1	0.502809312	carboxylic acid transport
GO:0009150	60	1	0.508682733	purine ribonucleotide metabolism
GO:0009628	318	4	0.516111326	response to abiotic stimulus
GO:0051243	583	7	0.531519236	negative regulation of cellular physiological process
GO:0016481	151	2	0.532140155	negative regulation of transcription
GO:0006396	326	4	0.535869572	RNA processing
GO:0009790	65	1	0.537034586	embryonic development
GO:0006874	68	1	0.553261368	calcium ion homeostasis
GO:0042592	157	2	0.553297941	homeostasis
GO:0048519	688	8	0.564141021	negative regulation of biological process
GO:0043118	602	7	0.56629041	negative regulation of physiological process
GO:0031323	1566	18	0.579333081	regulation of cellular metabolism
GO:0016310	522	6	0.580682279	phosphorylation

GO:0006118	256	3	0.582342457	electron transport
GO:0006812	346	4	0.583468255	cation transport
GO:0006351	1407	16	0.596459005	transcription, DNA-dependent
GO:0045893	77	1	0.598637237	positive regulation of transcription, DNA-dependent
GO:0019735	77	1	0.598637237	antimicrobial humoral response (sensu Vertebrata)
GO:0019730	78	1	0.603388153	antimicrobial humoral response
GO:0007596	78	1	0.603388153	blood coagulation
GO:0006091	446	5	0.60603011	generation of precursor metabolites and energy
GO:0050817	79	1	0.608083357	coagulation
GO:0051246	174	2	0.60956273	regulation of protein metabolism
GO:0006814	80	1	0.612723496	sodium ion transport
GO:0006508	451	5	0.616019949	proteolysis and peptidolysis
GO:0044257	451	5	0.616019949	cellular protein catabolism
GO:0006397	180	2	0.628109278	mRNA processing
GO:0006520	181	2	0.631133928	amino acid metabolism
GO:0042060	85	1	0.635120351	wound healing
GO:0042221	183	2	0.637126452	response to chemical substance
GO:0000902	183	2	0.637126452	cellular morphogenesis
GO:0030163	463	5	0.639396992	protein catabolism
GO:0050954	86	1	0.639443279	sensory perception of mechanical stimulus
GO:0050982	86	1	0.639443279	detection of mechanical stimulus
GO:0007605	86	1	0.639443279	perception of sound
GO:0006355	1359	15	0.643944926	regulation of transcription, DNA-dependent
GO:0043285	474	5	0.660058266	biopolymer catabolism
GO:0006461	194	2	0.668741862	protein complex assembly
GO:0016070	391	4	0.679742034	RNA metabolism
GO:0045941	100	1	0.694896096	positive regulation of transcription
GO:0016071	205	2	0.698122463	mRNA metabolism
GO:0007417	101	1	0.698516899	central nervous system development
GO:0006350	1499	16	0.701541809	transcription
GO:0016064	103	1	0.705631294	humoral defense mechanism (sensu Vertebrata)
GO:0048232	105	1	0.71257935	male gamete generation
GO:0045935	105	1	0.71257935	positive regulation of nucleobase, nucleoside, nucleotide and nucleic acid metabolism
GO:0007283	105	1	0.71257935	spermatogenesis
GO:0015698	106	1	0.715992209	inorganic anion transport
GO:0006519	216	2	0.725338262	amino acid and derivative metabolism
GO:0009582	109	1	0.72599177	detection of abiotic stimulus
GO:0045449	1433	15	0.726043456	regulation of transcription
GO:0006811	515	5	0.730284993	ion transport
GO:0006813	111	1	0.732463576	potassium ion transport
GO:0015672	223	2	0.741569178	monovalent inorganic cation transport
GO:0045859	116	1	0.747988158	regulation of protein kinase activity
GO:0051338	117	1	0.750984261	regulation of transferase activity
GO:0008284	121	1	0.762619889	positive regulation of cell proliferation
GO:0051234	1841	19	0.771245726	establishment of localization
GO:0006793	641	6	0.772698374	phosphorus metabolism
GO:0006796	641	6	0.772698374	phosphate metabolism

GO:0007276	125	1	0.773716715	gametogenesis
GO:0006820	128	1	0.781700662	anion transport
GO:0051179	1857	19	0.784560592	localization
GO:0044265	563	5	0.798668982	cellular macromolecule catabolism
GO:0031325	136	1	0.801652649	positive regulation of cellular metabolism
GO:0006810	1802	18	0.812541045	transport
GO:0006959	141	1	0.813193914	humoral immune response
GO:0009057	575	5	0.813494182	macromolecule catabolism
GO:0050874	1531	15	0.815492794	organismal physiological process
GO:0045786	145	1	0.821945848	negative regulation of progression through cell cycle
GO:0009308	269	2	0.829147949	amine metabolism
GO:0019953	149	1	0.830291438	sexual reproduction
GO:0009893	149	1	0.830291438	positive regulation of metabolism
GO:0044248	700	6	0.840081963	cellular catabolism
GO:0006807	291	2	0.860848056	nitrogen compound metabolism
GO:0007155	518	4	0.864952739	cell adhesion
GO:0006457	169	1	0.866549524	protein folding
GO:0016192	296	2	0.867267387	vesicle-mediated transport
GO:0051242	411	3	0.86728112	positive regulation of cellular physiological process
GO:0016337	172	1	0.871281151	cell-cell adhesion
GO:0042981	306	2	0.879305759	regulation of apoptosis
GO:0043067	308	2	0.881590513	regulation of programmed cell death
GO:0043119	428	3	0.884732798	positive regulation of physiological process
GO:0009056	756	6	0.888401035	catabolism
GO:0043412	1160	10	0.889115551	biopolymer modification
GO:0006464	1087	9	0.904722322	protein modification
GO:0000004	465	3	0.915887232	biological process unknown
GO:0007267	465	3	0.915887232	cell-cell signaling
GO:0046903	208	1	0.916614844	secretion
GO:0000003	208	1	0.916614844	reproduction
GO:0050790	214	1	0.922448901	regulation of enzyme activity
GO:0048522	482	3	0.927481886	positive regulation of cellular process
GO:0009966	229	1	0.935324239	regulation of signal transduction
GO:0019538	2129	19	0.936004923	protein metabolism
GO:0008219	498	3	0.93705118	cell death
GO:0016265	501	3	0.938711796	death
GO:0044255	383	2	0.94335839	cellular lipid metabolism
GO:0007399	385	2	0.944489187	neurogenesis
GO:0007243	242	1	0.944754286	protein kinase cascade
GO:0007165	2168	19	0.947789328	signal transduction
GO:0006512	255	1	0.952820458	ubiquitin cycle
GO:0030154	409	2	0.956499554	cell differentiation
GO:0044267	2109	18	0.95713607	cellular protein metabolism
GO:0042127	265	1	0.958221066	regulation of cell proliferation
GO:0007610	269	1	0.96020561	behavior
GO:0048518	565	3	0.965837779	positive regulation of biological process
GO:0007600	287	1	0.968041103	sensory perception

GO:0046907	448	2	0.970924844	intracellular transport
GO:0044260	2298	19	0.974842509	cellular macromolecule metabolism
GO:0043170	2405	20	0.97641103	macromolecule metabolism
GO:0006629	469	2	0.976669411	lipid metabolism
GO:0009581	313	1	0.976735779	detection of external stimulus
GO:0006915	471	2	0.977155928	apoptosis
GO:0012501	473	2	0.977632719	programmed cell death
GO:0048513	872	5	0.980203722	organ development
GO:0007154	2730	23	0.981338856	cell communication
GO:0007186	491	2	0.981515387	G-protein coupled receptor protein signaling pathway
GO:0005975	355	1	0.986099022	carbohydrate metabolism
GO:0007275	1543	10	0.991414871	development
GO:0015031	439	1	0.995074244	protein transport
GO:0009653	1051	5	0.996027411	morphogenesis
GO:0045184	459	1	0.996158148	establishment of protein localization
GO:0008104	474	1	0.996812685	protein localization
GO:0050877	475	1	0.996852164	neurophysiological process
GO:0009887	807	3	0.9969297	organogenesis
GO:0007166	952	3	0.999356495	cell surface receptor linked signal transduction
GO:0008150	9032	106	1	biological_process

21.3 GO: Molecular Function

GO ID	genes present in chip	genes present in tissues	p-value	GO term
GO:0016725	2	2	0.000129332592406555	oxidoreductase activity, acting on CH2 groups
GO:0016728	2	2	0.000129332592406555	oxidoreductase activity, acting on CH2 groups, disulfide as acceptor
GO:0004488	2	2	0.000129332592406555	methylenetetrahydrofolate dehydrogenase (NADP+) activity
GO:0004477	2	2	0.000129332592406555	methenyltetrahydrofolate cyclohydrolase activity
GO:0004748	2	2	0.000129332592406555	ribonucleoside-diphosphate reductase activity
GO:0016646	11	3	0.00022389347485483	oxidoreductase activity, acting on the CH-NH group of donors, NAD or NADP as acceptor
GO:0004486	3	2	0.00038509630172171	methylenetetrahydrofolate dehydrogenase activity
GO:0019238	4	2	0.00076443802051253	cyclohydrolase activity
GO:0016627	37	4	0.000796310146417573	oxidoreductase activity, acting on the CH-CH group of donors
GO:0016645	18	3	0.00104457139381203	oxidoreductase activity, acting on the CH-NH group of donors
GO:0016634	8	2	0.00346217623255135	oxidoreductase activity, acting on the CH-CH group of donors, oxygen as acceptor
GO:0003887	28	3	0.00386001690032929	DNA-directed DNA polymerase activity
GO:0030554	1028	21	0.00570333144022306	adenyl nucleotide binding
GO:0008094	34	3	0.00671002089334689	DNA-dependent ATPase activity
GO:0005344	12	2	0.00792101518518785	oxygen transporter activity
GO:0016628	14	2	0.0107601524279605	oxidoreductase activity, acting on the CH-CH group of donors, NAD or NADP as acceptor
GO:0004853	1	1	0.0114243789959120	uroporphyrinogen decarboxylase activity
GO:0004638	1	1	0.0114243789959120	phosphoribosylaminoimidazole carboxylase activity
GO:0004639	1	1	0.0114243789959120	phosphoribosylaminoimidazolesuccinocarboxamide synthase activity
GO:0008413	1	1	0.0114243789959120	8-oxo-7,8-dihydroguanine triphosphatase activity
GO:0050355	1	1	0.0114243789959120	triphosphatase activity
GO:0047017	1	1	0.0114243789959120	prostaglandin-F synthase activity
GO:0042083	1	1	0.0114243789959120	5,10-methylenetetrahydrofolate-dependent methyltransferase activity
GO:0008513	1	1	0.0114243789959120	organic cation porter activity
GO:0030337	1	1	0.0114243789959120	DNA polymerase processivity factor activity
GO:0042602	1	1	0.0114243789959120	flavin reductase activity
GO:0016538	1	1	0.0114243789959120	cyclin-dependent protein kinase regulator activity
GO:0004325	1	1	0.0114243789959120	ferrochelatase activity
GO:0004329	1	1	0.0114243789959120	formate-tetrahydrofolate ligase activity
GO:0004418	1	1	0.0114243789959120	hydroxymethylbilane synthase activity
GO:0004446	1	1	0.0114243789959120	multiple inositol-polyphosphate phosphatase activity
GO:0004137	1	1	0.0114243789959120	deoxycytidine kinase activity
GO:0004799	1	1	0.0114243789959120	thymidylate synthase activity
GO:0004729	1	1	0.0114243789959120	protoporphyrinogen oxidase activity
GO:0005524	970	19	0.0132117129982571	ATP binding
GO:0004089	16	2	0.0139800893874154	carbonate dehydratase activity
GO:0016829	127	5	0.0150142643401759	lyase activity
GO:0043014	2	1	0.0227194253994182	alpha-tubulin binding
GO:0008379	2	1	0.0227194253994182	thioredoxin peroxidase activity
GO:0008198	2	1	0.0227194253994182	ferrous iron binding
GO:0004998	2	1	0.0227194253994182	transferrin receptor activity
GO:0047026	2	1	0.0227194253994182	3-alpha-hydroxysteroid dehydrogenase (A-specific) activity
GO:0003893	2	1	0.0227194253994182	epsilon DNA polymerase activity

GO:0003870	2	1	0.0227194253994182	5-aminolevulinate synthase activity
GO:0003912	2	1	0.0227194253994182	DNA nucleotidylexotransferase activity
GO:0004074	2	1	0.0227194253994182	biliverdin reductase activity
GO:0016749	2	1	0.0227194253994182	N-succinyltransferase activity
GO:0004357	2	1	0.0227194253994182	glutamate-cysteine ligase activity
GO:0004487	2	1	0.0227194253994182	methylenetetrahydrofolate dehydrogenase (NAD+) activity
GO:0004109	2	1	0.0227194253994182	coproporphyrinogen oxidase activity
GO:0001758	2	1	0.0227194253994182	retinal dehydrogenase activity
GO:0016814	21	2	0.0235764274899718	hydrolase activity, acting on carbon-nitrogen (but not peptide) bonds, in cyclic amidines
GO:0017076	1248	22	0.0238936219640091	purine nucleotide binding
GO:0008408	24	2	0.0303082496807488	3'-5' exonuclease activity
GO:0016831	24	2	0.0303082496807488	carboxy-lyase activity
GO:0016491	509	11	0.0305514430701976	oxidoreductase activity
GO:0003796	3	1	0.0338865899482665	lysozyme activity
GO:0047115	3	1	0.0338865899482665	trans-1,2-dihydrobenzene-1,2-diol dehydrogenase activity
GO:0016748	3	1	0.0338865899482665	succinyltransferase activity
GO:0016978	3	1	0.0338865899482665	lipoate-protein ligase B activity
GO:0005506	212	6	0.0343457158438138	iron ion binding
GO:0042802	158	5	0.0345433725560872	protein self binding
GO:0003697	28	2	0.0403065436819446	single-stranded DNA binding
GO:0003824	3409	48	0.044137062604611	catalytic activity
GO:0043015	4	1	0.0449273072575173	gamma-tubulin binding
GO:0005497	4	1	0.0449273072575173	androgen binding
GO:0019136	4	1	0.0449273072575173	deoxynucleoside kinase activity
GO:0042301	4	1	0.0449273072575173	phosphate binding
GO:0050811	4	1	0.0449273072575173	GABA receptor binding
GO:0003918	4	1	0.0449273072575173	DNA topoisomerase (ATP-hydrolyzing) activity
GO:0030492	4	1	0.0449273072575173	hemoglobin binding
GO:0016979	4	1	0.0449273072575173	lipoate-protein ligase activity
GO:0019825	31	2	0.04850559170517	oxygen binding
GO:0016830	31	2	0.04850559170517	carbon-carbon lyase activity
GO:0016410	33	2	0.0542780703768583	N-acyltransferase activity
GO:0008301	5	1	0.0558429959970507	DNA bending activity
GO:0048487	5	1	0.0558429959970507	beta-tubulin binding
GO:0005521	5	1	0.0558429959970507	lamin binding
GO:0004185	5	1	0.0558429959970507	serine carboxypeptidase activity
GO:0004749	5	1	0.0558429959970507	ribose phosphate diphosphokinase activity
GO:0016879	127	4	0.0573310515777338	ligase activity, forming carbon-nitrogen bonds
GO:0003916	6	1	0.0666350590671347	DNA topoisomerase activity
GO:0016778	6	1	0.0666350590671347	diphosphotransferase activity
GO:0004303	6	1	0.0666350590671347	estradiol 17-beta-dehydrogenase activity
GO:0016887	255	6	0.0716001430890079	ATPase activity
GO:0005200	86	3	0.0752097684637681	structural constituent of cytoskeleton
GO:0003713	140	4	0.0761880790274251	transcription coactivator activity
GO:0008009	40	2	0.0762009277680908	chemokine activity
GO:0004527	40	2	0.0762009277680908	exonuclease activity
GO:0042379	40	2	0.0762009277680908	chemokine receptor binding

GO:0004673	7	1	0.0773048837720828	protein histidine kinase activity
GO:0000155	7	1	0.0773048837720828	two-component sensor molecule activity
GO:0015101	7	1	0.0773048837720828	organic cation transporter activity
GO:0001664	42	2	0.0829038316215289	G-protein-coupled receptor binding
GO:0016836	43	2	0.0863210380742479	hydro-lyase activity
GO:0000166	1432	22	0.0863922782506284	nucleotide binding
GO:0008191	8	1	0.0878538419920163	metalloendopeptidase inhibitor activity
GO:0004029	8	1	0.0878538419920163	aldehyde dehydrogenase (NAD) activity
GO:0005099	8	1	0.0878538419920163	Ras GTPase activator activity
GO:0016779	95	3	0.0946708441662362	nucleotidyltransferase activity
GO:0017111	410	8	0.0966641139848202	nucleoside-triphosphatase activity
GO:0003993	9	1	0.0982832903527494	acid phosphatase activity
GO:0030552	9	1	0.0982832903527494	3',5'-cAMP binding
GO:0019887	47	2	0.100395649453071	protein kinase regulator activity
GO:0003677	1384	21	0.102702215701935	DNA binding
GO:0019843	10	1	0.108594570393827	rRNA binding
GO:0008603	10	1	0.108594570393827	cAMP-dependent protein kinase regulator activity
GO:0000175	10	1	0.108594570393827	3'-5'-exoribonuclease activity
GO:0016208	10	1	0.108594570393827	AMP binding
GO:0016775	10	1	0.108594570393827	phosphotransferase activity, nitrogenous group as acceptor
GO:0004033	10	1	0.108594570393827	aldo-keto reductase activity
GO:0016772	708	12	0.108732449353156	transferase activity, transferring phosphorus-containing groups
GO:0016835	50	2	0.111340370602751	carbon-oxygen lyase activity
GO:0016462	430	8	0.118278308904353	pyrophosphatase activity
GO:0008134	293	6	0.118500530762122	transcription factor binding
GO:0004532	11	1	0.118789008734722	exoribonuclease activity
GO:0005355	11	1	0.118789008734722	glucose transporter activity
GO:0019206	11	1	0.118789008734722	nucleoside kinase activity
GO:0016896	11	1	0.118789008734722	exoribonuclease activity, producing 5'-phosphomonoesters
GO:0016818	432	8	0.120573551020066	hydrolase activity, acting on acid anhydrides, in phosphorus-containing anhydrides
GO:0016817	433	8	0.121730154061228	hydrolase activity, acting on acid anhydrides
GO:0003712	230	5	0.122937963529723	transcription cofactor activity
GO:0004028	12	1	0.128867917239226	aldehyde dehydrogenase activity
GO:0042562	12	1	0.128867917239226	hormone binding
GO:0015145	12	1	0.128867917239226	monosaccharide transporter activity
GO:0015149	12	1	0.128867917239226	hexose transporter activity
GO:0046983	170	4	0.129828681578864	protein dimerization activity
GO:0005488	7228	88	0.133011320513979	binding
GO:0005251	13	1	0.138832593178036	delayed rectifier potassium channel activity
GO:0019207	58	2	0.141883404495381	kinase regulator activity
GO:0046982	61	2	0.153758620365497	protein heterodimerization activity
GO:0051015	15	1	0.158424364439075	actin filament binding
GO:0016229	15	1	0.158424364439075	steroid dehydrogenase activity
GO:0004468	16	1	0.168053982775852	lysine N-acetyltransferase activity
GO:0004402	16	1	0.168053982775852	histone acetyltransferase activity
GO:0016796	17	1	0.177574414888969	exonuclease activity, active with either ribo- or deoxyribonucleic acids and producing 5'-phosphomonoesters
GO:0008430	18	1	0.186986887461113	selenium binding

GO:0016620	18	1	0.186986887461113	oxidoreductase activity, acting on the aldehyde or oxo group of donors, NAD or NADP as acceptor
GO:0030551	18	1	0.186986887461113	cyclic nucleotide binding
GO:0050681	19	1	0.196292613520835	androgen receptor binding
GO:0016810	72	2	0.198701032397055	hydrolase activity, acting on carbon-nitrogen (but not peptide) bonds
GO:0003690	20	1	0.205492792593113	double-stranded DNA binding
GO:0046906	74	2	0.20704696892242	tetrapyrrole binding
GO:0020037	74	2	0.20704696892242	heme binding
GO:0004601	22	1	0.223581241249272	peroxidase activity
GO:0016684	22	1	0.223581241249272	oxidoreductase activity, acting on peroxide as acceptor
GO:0035258	23	1	0.232471843697411	steroid hormone receptor binding
GO:0005478	24	1	0.241261565176396	intracellular transporter activity
GO:0019200	24	1	0.241261565176396	carbohydrate kinase activity
GO:0016874	221	4	0.245896274648915	ligase activity
GO:0016903	25	1	0.249951539894876	oxidoreductase activity, acting on the aldehyde or oxo group of donors
GO:0003676	1990	26	0.251936601936912	nucleic acid binding
GO:0016740	1169	16	0.257222464830981	transferase activity
GO:0003704	26	1	0.258542889427395	specific RNA polymerase II transcription factor activity
GO:0016769	26	1	0.258542889427395	transferase activity, transferring nitrogenous groups
GO:0004437	26	1	0.258542889427395	inositol or phosphatidylinositol phosphatase activity
GO:0016301	609	9	0.259957035794377	kinase activity
GO:0050662	88	2	0.266201839657864	coenzyme binding
GO:0004180	27	1	0.267036722853819	carboxypeptidase activity
GO:0008080	28	1	0.275434136897222	N-acetyltransferase activity
GO:0051119	29	1	0.283736216060283	sugar transporter activity
GO:0003823	30	1	0.291944032760181	antigen binding
GO:0015144	30	1	0.291944032760181	carbohydrate transporter activity
GO:0016209	31	1	0.300058647462014	antioxidant activity
GO:0008415	98	2	0.308587864864112	acyltransferase activity
GO:0003774	98	2	0.308587864864112	motor activity
GO:0016747	98	2	0.308587864864112	transferase activity, transferring groups other than amino-acyl groups
GO:0008307	33	1	0.316012453761827	structural constituent of muscle
GO:0004674	326	5	0.316177003147907	protein serine/threonine kinase activity
GO:0048037	100	2	0.317022458235432	cofactor binding
GO:0016881	101	2	0.321231453114434	acid-amino acid ligase activity
GO:0019205	34	1	0.323853707710077	nucleobase, nucleoside, nucleotide kinase activity
GO:0016746	104	2	0.333820246851985	transferase activity, transferring acyl groups
GO:0005515	3709	45	0.335056082812866	protein binding
GO:0004518	105	2	0.33800245513025	nuclease activity
GO:0016765	37	1	0.346847006750761	transferase activity, transferring alkyl or aryl (other than methyl) groups
GO:0016407	39	1	0.361743707965709	acetyltransferase activity
GO:0005496	42	1	0.383459795458356	steroid binding
GO:0015631	42	1	0.383459795458356	tubulin binding
GO:0035257	42	1	0.383459795458356	nuclear hormone receptor binding
GO:0019992	42	1	0.383459795458356	diacylglycerol binding
GO:0005096	117	2	0.387491844401499	GTPase activator activity
GO:0051427	43	1	0.390534527777025	hormone receptor binding
GO:0050660	44	1	0.397528814623973	FAD binding

GO:0004672	447	6	0.403235536720316	protein kinase activity
GO:0003924	121	2	0.403645303089121	GTPase activity
GO:0004540	45	1	0.404443562355465	ribonuclease activity
GO:0003777	45	1	0.404443562355465	microtubule motor activity
GO:0016773	532	7	0.406525962851275	phosphotransferase activity, alcohol group as acceptor
GO:0015171	48	1	0.424719483330642	amino acid transporter activity
GO:0005509	646	8	0.459956393185665	calcium ion binding
GO:0016787	1424	17	0.462735465228863	hydrolase activity
GO:0005275	58	1	0.487491681031037	amine transporter activity
GO:0042623	229	3	0.488358938360223	ATPase activity, coupled
GO:0008083	144	2	0.492233359909584	growth factor activity
GO:0004553	59	1	0.493382581249071	hydrolase activity, hydrolyzing O-glycosyl compounds
GO:0046872	2495	29	0.493504092396083	metal ion binding
GO:0031420	146	2	0.499549047869467	alkali metal ion binding
GO:0016563	62	1	0.510655718211508	transcriptional activator activity
GO:0005249	63	1	0.51628273864773	voltage-gated potassium channel activity
GO:0030234	509	6	0.528611395026818	enzyme regulator activity
GO:0005342	66	1	0.532781974651091	organic acid transporter activity
GO:0046943	66	1	0.532781974651091	carboxylic acid transporter activity
GO:0008238	68	1	0.543470416208799	exopeptidase activity
GO:0030674	69	1	0.548723422081621	protein binding, bridging
GO:0000287	251	3	0.550333622069994	magnesium ion binding
GO:0031402	70	1	0.553916533039508	sodium ion binding
GO:0043167	2556	29	0.554135440067405	ion binding
GO:0043169	2313	26	0.575134361695845	cation binding
GO:0016791	171	2	0.585171593205172	phosphoric monoester hydrolase activity
GO:0016798	77	1	0.588647148696375	hydrolase activity, acting on glycosyl bonds
GO:0016616	77	1	0.588647148696375	oxidoreductase activity, acting on the CH-OH group of donors, NAD or NADP as acceptor
GO:0016614	83	1	0.616274332722897	oxidoreductase activity, acting on CH-OH group of donors
GO:0015293	83	1	0.616274332722897	symporter activity
GO:0005125	181	2	0.616301937018092	cytokine activity
GO:0008168	84	1	0.620696631066437	methyltransferase activity
GO:0030955	86	1	0.629390289796918	potassium ion binding
GO:0016741	87	1	0.633662786720465	transferase activity, transferring one-carbon groups
GO:0004857	187	2	0.634115713432959	enzyme inhibitor activity
GO:0008047	188	2	0.637021885063035	enzyme activator activity
GO:0005261	188	2	0.637021885063035	cation channel activity
GO:0005267	90	1	0.64618963389063	potassium channel activity
GO:0016853	92	1	0.654304109944418	isomerase activity
GO:0030145	92	1	0.654304109944418	manganese ion binding
GO:0030528	963	10	0.672554695855458	transcription regulator activity
GO:0004866	99	1	0.681279397551473	endopeptidase inhibitor activity
GO:0030414	100	1	0.684958760574867	protease inhibitor activity
GO:0005529	103	1	0.695746212910188	sugar binding
GO:0009055	104	1	0.69926005724085	electron carrier activity
GO:0030695	219	2	0.71842307879044	GTPase regulator activity
GO:0005554	522	5	0.719030704810837	molecular function unknown

GO:0005102	528	5	0.728402458752422	receptor binding
GO:0042578	228	2	0.739021566738103	phosphoric ester hydrolase activity
GO:0008236	117	1	0.741441570639556	serine-type peptidase activity
GO:0004721	118	1	0.74443211274485	phosphoprotein phosphatase activity
GO:0005215	1030	10	0.752769088571272	transporter activity
GO:0046914	1596	16	0.755238708221011	transition metal ion binding
GO:0016788	446	4	0.756460930981298	hydrolase activity, acting on ester bonds
GO:0005244	124	1	0.761669050115165	voltage-gated ion channel activity
GO:0051082	127	1	0.769850393855245	unfolded protein binding
GO:0005057	132	1	0.782872042155759	receptor signaling protein activity
GO:0005216	252	2	0.78780768487937	ion channel activity
GO:0005083	144	1	0.811220959783875	small GTPase regulator activity
GO:0005198	488	4	0.815616115306988	structural molecule activity
GO:0015290	149	1	0.821920778823898	electrochemical potential-driven transporter activity
GO:0015291	149	1	0.821920778823898	porter activity
GO:0015268	274	2	0.825359192971334	alpha-type channel activity
GO:0005489	152	1	0.828049905786595	electron transporter activity
GO:0008092	279	2	0.833024427953208	cytoskeletal protein binding
GO:0015267	284	2	0.840388046182194	channel or pore class transporter activity
GO:0004713	161	1	0.845210826368125	protein-tyrosine kinase activity
GO:0008324	421	3	0.86577872110297	cation transporter activity
GO:0003700	649	5	0.872008099998129	transcription factor activity
GO:0030246	181	1	0.877506832797053	carbohydrate binding
GO:0003779	189	1	0.888468072955803	actin binding
GO:0003702	197	1	0.898456646282416	RNA polymerase II transcription factor activity
GO:0005525	226	1	0.927781851075895	GTP binding
GO:0019001	235	1	0.935043970637075	guanyl nucleotide binding
GO:0008270	1287	10	0.93528920740584	zinc ion binding
GO:0015075	523	3	0.942837425096644	ion transporter activity
GO:0005386	398	2	0.945709253489345	carrier activity
GO:0008233	399	2	0.946240013577909	peptidase activity
GO:0003723	436	2	0.96276410138356	RNA binding
GO:0008289	298	1	0.96915590070677	lipid binding
GO:0004871	1837	13	0.985247879920675	signal transducer activity
GO:0004872	1060	5	0.995191009470646	receptor activity
GO:0004888	694	1	0.999746575795943	transmembrane receptor activity
GO:0003674	9541	109	1	molecular_function

21.4 GO: Cellular Component

GO ID	genes present in chip	genes present in tissues	p-value	GO term
GO:0005819	68	8	1.01513697040236e-06	spindle
GO:0005694	203	10	0.000113251647489173	chromosome
GO:0000793	23	4	0.000126509935398199	condensed chromosome
GO:0005833	10	3	0.000170379554021494	hemoglobin complex
GO:0015630	191	9	0.000351835308661110	microtubule cytoskeleton
GO:0000796	3	2	0.000395168650363217	condensin complex
GO:0005876	14	3	0.000499687253295501	spindle microtubule
GO:0005634	2704	47	0.000688355692084915	nucleus
GO:0005622	5419	78	0.000748749372105347	intracellular
GO:0043228	977	21	0.00350100572917195	non-membrane-bound organelle
GO:0043232	977	21	0.00350100572917195	intracellular non-membrane-bound organelle
GO:0043227	3955	58	0.00913900710144103	membrane-bound organelle
GO:0043231	3955	58	0.00913900710144103	intracellular membrane-bound organelle
GO:0043226	4515	64	0.0114682083312629	organelle
GO:0043229	4515	64	0.0114682083312629	intracellular organelle
GO:0009320	1	1	0.0115780942456878	phosphoribosylaminoimidazole carboxylase complex
GO:0005971	1	1	0.0115780942456878	ribonucleoside-diphosphate reductase complex
GO:0000228	42	3	0.0124705734685483	nuclear chromosome
GO:0000776	17	2	0.0161185623054063	kinetochore
GO:0031304	2	1	0.023023461380405	intrinsic to mitochondrial inner membrane
GO:0005827	2	1	0.023023461380405	polar microtubule
GO:0051233	2	1	0.023023461380405	spindle midzone
GO:0005739	490	11	0.0257755557590847	mitochondrion
GO:0043234	1247	22	0.0262371539845070	protein complex
GO:0005816	3	1	0.0343376077454274	spindle pole body
GO:0000775	27	2	0.038608306278255	chromosome, pericentric region
GO:0031229	4	1	0.0455220227588	intrinsic to nuclear inner membrane
GO:0005639	4	1	0.0455220227588	integral to nuclear inner membrane
GO:0005856	612	12	0.0495759569358517	cytoskeleton
GO:0005814	5	1	0.0565781791035368	centriole
GO:0005874	132	4	0.066671327201534	microtubule
GO:0005663	6	1	0.0675075329137369	DNA replication factor C complex
GO:0000930	6	1	0.0675075329137369	gamma-tubulin complex
GO:0005637	7	1	0.0783115239586488	nuclear inner membrane
GO:0000502	41	2	0.081342043309433	proteasome complex (sensu Eukaryota)
GO:0005737	2530	36	0.0868028201678636	cytoplasm
GO:0005813	43	2	0.0882729586945227	centrosome
GO:0008091	9	1	0.099549096095604	spectrin
GO:0005829	276	6	0.100345118366615	cytosol
GO:0000785	100	3	0.109405529698918	chromatin
GO:0005680	10	1	0.109985476530287	anaphase-promoting complex
GO:0016363	10	1	0.109985476530287	nuclear matrix
GO:0005952	10	1	0.109985476530287	cAMP-dependent protein kinase complex

GO:0005815	49	2	0.110036792242655	microtubule organizing center
GO:0000922	51	2	0.117578883366946	spindle pole
GO:0005732	12	1	0.130500306857942	small nucleolar ribonucleoprotein complex
GO:0030864	12	1	0.130500306857942	cortical actin cytoskeleton
GO:0005941	57	2	0.140926687030886	unlocalized protein complex
GO:0000152	14	1	0.150546891028431	nuclear ubiquitin ligase complex
GO:0030894	14	1	0.150546891028431	replisome
GO:0005859	15	1	0.160397907020216	muscle myosin
GO:0005657	15	1	0.160397907020216	replication fork
GO:0016460	15	1	0.160397907020216	myosin II
GO:0000178	15	1	0.160397907020216	exosome (RNase complex)
GO:0000794	15	1	0.160397907020216	condensed nuclear chromosome
GO:0030863	16	1	0.170135811137355	cortical cytoskeleton
GO:0048471	22	1	0.226258887944068	perinuclear region
GO:0008305	24	1	0.244118315460808	integrin complex
GO:0005793	24	1	0.244118315460808	ER-Golgi intermediate compartment
GO:0019866	89	2	0.275535950597695	inner membrane
GO:0005938	28	1	0.278621802796224	cell cortex
GO:0031300	90	2	0.279840256016096	intrinsic to organelle membrane
GO:0043292	40	1	0.373048943901818	contractile fiber
GO:0000786	40	1	0.373048943901818	nucleosome
GO:0000151	42	1	0.38755029933407	ubiquitin ligase complex
GO:0016459	43	1	0.394675950301678	myosin
GO:0008076	53	1	0.461568809966802	voltage-gated potassium channel complex
GO:0005615	385	5	0.462273336254299	extracellular space
GO:0005681	57	1	0.486227997511385	spliceosome complex
GO:0043235	58	1	0.492216016188409	receptor complex
GO:0005623	7802	91	0.495308556176609	cell
GO:0005768	62	1	0.515484935034161	endosome
GO:0005875	70	1	0.558901919665478	microtubule associated complex
GO:0005730	75	1	0.584057648501197	nucleolus
GO:0031301	76	1	0.588915653073712	integral to organelle membrane
GO:0005635	83	1	0.621384204034257	nuclear membrane
GO:0015629	183	2	0.629886797877701	actin cytoskeleton
GO:0005743	85	1	0.63018533984503	mitochondrial inner membrane
GO:0005667	91	1	0.655391580430808	transcription factor complex
GO:0005624	521	5	0.729148145212425	membrane fraction
GO:0008372	523	5	0.732268952190412	cellular component unknown
GO:0005886	1472	15	0.746758692096123	plasma membrane
GO:0005740	119	1	0.75227648394204	mitochondrial membrane
GO:0016023	122	1	0.760900165680698	cytoplasmic membrane-bound vesicle
GO:0031410	124	1	0.766483482627346	cytoplasmic vesicle
GO:0030529	254	2	0.798053024907082	ribonucleoprotein complex
GO:0005783	483	4	0.818190123668435	endoplasmic reticulum
GO:0005887	1057	9	0.877709385235515	integral to plasma membrane
GO:0031226	1062	9	0.881115874301765	intrinsic to plasma membrane
GO:0000267	698	5	0.915369887166074	cell fraction

GO:0005654	221	1	0.92626165282897	nucleoplasm
GO:0005576	949	7	0.93345454369373	extracellular region
GO:0031090	377	2	0.936909694703344	organelle membrane
GO:0012505	246	1	0.94533293961463	endomembrane system
GO:0031224	2681	18	0.998982610702124	intrinsic to membrane
GO:0016021	2674	17	0.999541217676317	integral to membrane
GO:0016020	3514	23	0.99994267046084	membrane
GO:0005575	8637	100	1	cellular_component

22. BM-CD34

22.1 BM-CD34 selective genes (105 genes)

Symbol	Description	Chromosome	Chromosome Location	LocusLink	Cytoband	UniGene
IGLL1	immunoglobulin lambda-like polypeptide 1	22	-22245313	3543	22q11.23	Hs.348935
MPO	myeloperoxidase	17	-53702215	4353	17q23.1	Hs.458272
MYB	v-myb myeloblastosis viral oncogene homolog (avian)	6	135544145	4602	6q22-q23	Hs.531941
AZU1	azurocidin 1 (cationic antimicrobial protein 37)	19	778830	566	19p13.3	Hs.72885
FCER1A	Fc fragment of IgE, high affinity I, receptor for, alpha polypeptide	1	157526127	2205	1q23	Hs.897
ELA2	elastase 2, neutrophil	19	803290	1991	19p13.3	Hs.99863
LYZ	lysozyme (renal amyloidosis)	12	68028430	4069	12q15	Hs.524579
DNTT	deoxynucleotidyltransferase, terminal	10	98054074	1791	10q23-q24	Hs.534206
DTL	denticleless homolog (Drosophila)	1	210275753	51514	NA	Hs.632496
NFE2	nuclear factor (erythroid-derived 2), 45kDa	12	-52972164	4778	12q13	Hs.75643
MS4A3	membrane-spanning 4-domains, subfamily A, member 3 (hematopoietic cell-specific)	11	59580676	932	11q12.1	Hs.99960
RNASE2	ribonuclease, RNase A family, 2 (liver, eosinophil-derived neurotoxin)	14	20493469	6036	14q24-q31	Hs.728
PPBP	pro-platelet basic protein (chemokine (C-X-C motif) ligand 7)	4	-75071621	5473	4q12-q13	Hs.2164
TOP2A	topoisomerase (DNA) II alpha 170kDa	17	-35798323	7153	17q21-q22	Hs.156346
MCM5	MCM5 minichromosome maintenance deficient 5, cell division cycle 46 (S. cerevisiae)	22	34126127	4174	22q13.1	Hs.517582
MLF1IP	MLF1 interacting protein	4	-185852231	79682	4q35.1	Hs.481307 Hs.575032
NUCB2	nucleobindin 2	11	17254887	4925	11p15.1-p14	Hs.128686
HMGB2	high-mobility group box 2	4	-174489362	3148	4q31	Hs.434953
PLAC8	placenta-specific 8	4	-84230866	51316	4q21.22	Hs.546392
MINPP1	multiple inositol polyphosphate histidine phosphatase, 1	10	89254632	9562	10q23	Hs.121260
KIT	v-kit Hardy-Zuckerman 4 feline sarcoma viral oncogene homolog	4	55218917	3815	4q11-q12	Hs.479754
ZWINT	ZW10 interactor	10	-57787204	11130	10q21-q22	Hs.591363
CLC	Charcot-Leyden crystal protein	19	-44913735	1178	19q13.1	Hs.889
CYTL1	cytokine-like 1	4	-5067216	54360	4p16-p15	Hs.13872
HBD	hemoglobin, delta	11	-5210634	3045	11p15.5	Hs.591988
GMFG	glia maturation factor, gamma	19	-44510841	9535	19q13.2	Hs.5210
LMO2	LIM domain only 2 (rhombotin-like 1)	11	-33836698	4005	11p13	Hs.34560
VPREB1	pre-B lymphocyte gene 1	22	20929199	7441	22q11.2|22q11.22	Hs.247979
NUSAP1	nucleolar and spindle associated protein 1	15	39412360	51203	15q15.1	Hs.615092
PROM1	prominin 1	4	-15578954	8842	4p15.32	Hs.479220
ZNF651	zinc finger protein 651	3	42675877	92999	3p22.1	Hs.409561

PCNA	proliferating cell nuclear antigen	20	-5043599	5111	20pter-p12	Hs.147433
VAMP8	vesicle-associated membrane protein 8 (endobrevin)	2	85658227	8673	2p12-p11.2	Hs.534373
KLF1	Kruppel-like factor 1 (erythroid)	19	-12856238	10661	19p13.13-p13.12	Hs.37860
PYCARD	PYD and CARD domain containing	16	-31120309	29108	16p12-p11.2	Hs.499094
TFEC	transcription factor EC	7	-115362437	22797	7q31.2	Hs.125962
RNF138	ring finger protein 138	18	27925834, 27926644	51444	18q12.1	Hs.302408 Hs.501040
DOCK2	dedicator of cytokinesis 2	5	168996870	1794	5q35.1	Hs.586174
PRC1	protein regulator of cytokinesis 1	15	-89310276	9055	15q26.1	Hs.567385
EREG	epiregulin	4	75449723	2069	4q13.3	Hs.115263
FOSB	FBJ murine osteosarcoma viral oncogene homolog B	19	50663092	2354	19q13.32	Hs.590958
NMI	N-myc (and STAT) interactor	2	-151835230	9111	2p24.3-q21.3	Hs.54483
XK	X-linked Kx blood group (McLeod syndrome)	X	37430051	7504	Xp21.1	Hs.78919
CKS2	CDC28 protein kinase regulatory subunit 2	9	91115932	1164	9q22	Hs.83758
EZH2	enhancer of zeste homolog 2 (Drosophila)	7	-148135407	2146	7q35-q36	Hs.444082
PRKAR2B	protein kinase, cAMP-dependent, regulatory, type II, beta	7	106472413	5577	7q22	Hs.433068
PF4	platelet factor 4 (chemokine (C-X-C motif) ligand 4)	4	-75065659	5196	4q12-q21	Hs.81564
VRK1	vaccinia related kinase 1	14	96333436	7443	14q32	Hs.422662
MYC	v-myc myelocytomatosis viral oncogene homolog (avian)	8	128817497	4609	8q24.12-q24.13	Hs.202453
TYMS	thymidylate synthetase	18	647650	7298	18p11.32	Hs.592338
ERG	v-ets erythroblastosis virus E26 oncogene like (avian)	21	-38675670	2078	21q22.3	Hs.473819
CD69	CD69 molecule	12	-9796352	969	12p13-p12	Hs.208854
SELL	selectin L (lymphocyte adhesion molecule 1)	1	-167926433	6402	1q23-q25	Hs.82848
ANKRD27	ankyrin repeat domain 27 (VPS9 domain)	19	-37779753	84079	19q13.11	Hs.59236
PRTN3	proteinase 3 (serine proteinase, neutrophil, Wegener granulomatosis autoantigen)	19	791984	5657	19p13.3	Hs.928
PAICS	phosphoribosylaminoimidazole carboxylase, phosphoribosylaminoimidazole succinocarboxamide synthetase	4	56996697	10606	4pter-q21	Hs.518774
FAM117A	family with sequence similarity 117, member A	17	-45142687	81558	17q21.33	Hs.514308
SPINK2	serine peptidase inhibitor, Kazal type 2 (acrosin-trypsin inhibitor)	4	-57370790	6691	4q12	Hs.98243
PSMC6	proteasome (prosome, macropain) 26S subunit, ATPase, 6	14	52243667	5706	14q22.1	Hs.156171 Hs.491779
MEST	mesoderm specific transcript homolog (mouse)	7	129919173, 129913281, 129918408	4232	7q32	Hs.270978
RACGAP1	Rac GTPase activating protein 1	12	-48669214	29127	12q13.13	Hs.505469
PRG1	proteoglycan 1, secretory granule	10	70517833	5552	10q22.1	Hs.1908
AKR1C3	aldo-keto reductase family 1, member C3 (3-alpha hydroxysteroid dehydrogenase, type II)	10	5126567	8644	10p15-p14	Hs.78183
ITGA4	integrin, alpha 4 (antigen CD49D, alpha 4 subunit of VLA-4 receptor)	2	182029863	3676	2q31.3	Hs.440955
DCK	deoxycytidine kinase	4	72078255	1633	4q13.3-q21.1	Hs.709
RP6-213H19.1	NA	X	130985316, 130984925	51765	Xq26.2	Hs.444247
PSMD14	proteasome (prosome, macropain) 26S subunit, non-ATPase, 14	2	161873185	10213	2q24.2	Hs.567410

RASGRP2	RAS guanyl releasing protein 2 (calcium and DAG-regulated)	11	-64250959	10235	11q13	Hs.99491
CTSG	cathepsin G	14	-24112564	1511	14q11.2	Hs.421724
PRPS2	phosphoribosyl pyrophosphate synthetase 2	X	12719413	5634	Xp22.3-p22.2	Hs.104123
RRM2	ribonucleotide reductase M2 polypeptide	2	10180185	6241	2p25-p24	Hs.226390
MTHFD2	methylenetetrahydrofolate dehydrogenase (NADP+ dependent) 2, methenyltetrahydrofolate cyclohydrolase	2	74279197	10797	2p13.1	Hs.469030
CCNB1IP1	cyclin B1 interacting protein 1	14	-39698736	57820	14q11.2	Hs.107003
DPCR1	diffuse panbronchiolitis critical region 1	6	31027537, 2367969, 2166477	135656	6p21.33	Hs.631993
HLA-DRA	major histocompatibility complex, class II, DR alpha	6	32515624	3122	6p21.3	Hs.520048
CSTA	cystatin A (stefin A)	3	123526700	1475	3q21	Hs.518198
EXOSC8	exosome component 8	13	36472916	11340	13q13.1	Hs.294041
BLNK	B-cell linker	10	-97941452	29760	10q23.2-q23.33	Hs.444049
CBFA2T3	core-binding factor, runt domain, alpha subunit 2, translocated to, 3	16	-87468767	863	16q24	Hs.513811
MNDA	myeloid cell nuclear differentiation antigen	1	157067791	4332	1q22	Hs.153837
HAT1	histone acetyltransferase 1	2	172487203	8520	2q31.2-q33.1	Hs.632532
HHEX	homeobox, hematopoietically expressed	10	94439679	3087	10q23.33	Hs.118651
IRF8	interferon regulatory factor 8	16	84490274	3394	16q24.1	Hs.137427
ITGA2B	integrin, alpha 2b (platelet glycoprotein IIb of IIb/IIIa complex, antigen CD41)	17	-39805075	3674	17q21.32	Hs.411312
DBF4	DBF4 homolog (S. cerevisiae)	7	87343479	10926	7q21.3	Hs.485380
BUB1B	BUB1 budding uninhibited by benzimidazoles 1 homolog beta (yeast)	15	38240529	701	15q15	Hs.631699
NA	NA	NA	NA	653991	NA	NA
PTPLA	protein tyrosine phosphatase-like (proline instead of catalytic arginine), member A	10	-17671965	9200	10p14-p13	Hs.114062
MCM6	MCM6 minichromosome maintenance deficient 6 (MIS5 homolog, S. pombe) (S. cerevisiae)	2	-136313673	4175	2q21	Hs.444118
NOLA1	nucleolar protein family A, member 1 (H/ACA small nucleolar RNPs)	4	110956114	54433	4q25	Hs.69851
KIAA0101	KIAA0101	15	-62444265	9768	15q22.31	Hs.81892
MTHFD1	methylenetetrahydrofolate dehydrogenase (NADP+ dependent) 1, methenyltetrahydrofolate cyclohydrolase, formyltetrahydrofolate synthetase	14	63924845	4522	14q24	Hs.632340
CD79B	CD79b molecule, immunoglobulin-associated beta	17	-59359830	974	17q23	Hs.89575
PNN	pinin, desmosome associated protein	14	38714150	5411	14q21.1	Hs.409965
RBM3	RNA binding motif (RNP1, RRM) protein 3	X	48317779	5935	Xp11.2	Hs.301404
LRRFIP1	leucine rich repeat (in FLII) interacting protein 1	2	238265667	9208	2q37.3	Hs.471779
PRG2	proteoglycan 2, bone marrow (natural killer cell activator, eosinophil granule major basic protein)	11	-56911410	5553	11q12	Hs.512633
NUDT22	nudix (nucleoside diphosphate linked moiety X)-type motif 22	11	63750337	84304	11q13.1	Hs.172847
HCAP-G	NA	4	17421622	64151	4p15.33	Hs.446201 Hs.567567
SYK	spleen tyrosine kinase	9	92603890	6850	9q22	Hs.371720
NMU	neuromedin U	4	-56156161	10874	4q12	Hs.418367

IMPA2	inositol(myo)-1(or 4)-monophosphatase 2	18	11971454	3613	18p11.2	Hs.367992
SERPINB1	serpin peptidase inhibitor, clade B (ovalbumin), member 1	6	-2778734	1992	6p25	Hs.381167
DDX3Y	DEAD (Asp-Glu-Ala-Asp) box polypeptide 3, Y-linked	Y	13526170	8653	Yq11	Hs.99120
PMAIP1	phorbol-12-myristate-13-acetate-induced protein 1	18	55718216	5366	18q21.32	Hs.96

22.2 GO: Biological Process

GO ID	genes present in chip	genes present in tissues	p-value	GO term
GO:0006952	751	25	1.19E-08	defense response
GO:0009607	857	25	1.62E-07	response to biotic stimulus
GO:0006955	684	22	2.08E-07	immune response
GO:0048015	71	8	2.89E-07	phosphoinositide-mediated signaling
GO:0006260	140	10	6.74E-07	DNA replication
GO:0030595	13	4	4.90E-06	immune cell chemotaxis
GO:0050900	14	4	6.81E-06	immune cell migration
GO:0050896	1578	31	2.10E-05	response to stimulus
GO:0006261	65	6	3.08E-05	DNA-dependent DNA replication
GO:0007049	601	17	3.53E-05	cell cycle
GO:0007051	21	4	3.87E-05	spindle organization and biogenesis
GO:0006935	101	7	4.29E-05	chemotaxis
GO:0042330	101	7	4.29E-05	taxis
GO:0001819	29	4	0.000145016	positive regulation of cytokine production
GO:0019932	176	8	0.000237777	second-messenger-mediated signaling
GO:0042742	34	4	0.000273197	defense response to bacteria
GO:0009613	458	13	0.000321327	response to pest, pathogen or parasite
GO:0043207	463	13	0.000356871	response to external biotic stimulus
GO:0006950	881	19	0.000432115	response to stress
GO:0001817	39	4	0.00046751	regulation of cytokine production
GO:0045073	4	2	0.000518791	regulation of chemokine biosynthesis
GO:0009123	18	3	0.000592723	nucleoside monophosphate metabolism
GO:0009124	18	3	0.000592723	nucleoside monophosphate biosynthesis
GO:0001816	42	4	0.000622709	cytokine production
GO:0043283	2240	35	0.000628186	biopolymer metabolism
GO:0042033	5	2	0.000859361	chemokine biosynthesis
GO:0009396	5	2	0.000859361	folic acid and derivative biosynthesis
GO:0050755	5	2	0.000859361	chemokine metabolism
GO:0042108	23	3	0.001243465	positive regulation of cytokine biosynthesis
GO:0009617	51	4	0.001303802	response to bacteria
GO:0000226	52	4	0.00140242	microtubule cytoskeleton organization and biogenesis
GO:0045727	25	3	0.001593135	positive regulation of protein biosynthesis
GO:0042221	183	7	0.001629961	response to chemical substance
GO:0006954	185	7	0.001734721	inflammatory response
GO:0009262	7	2	0.001782662	deoxyribonucleotide metabolism
GO:0050920	7	2	0.001782662	regulation of chemotaxis
GO:0031328	26	3	0.00178877	positive regulation of cellular biosynthesis
GO:0050778	56	4	0.001849201	positive regulation of immune response
GO:0006259	489	12	0.001968392	DNA metabolism
GO:0009117	143	6	0.002222992	nucleotide metabolism
GO:0051240	59	4	0.002243022	positive regulation of organismal physiological process
GO:0006760	8	2	0.002362368	folic acid and derivative metabolism
GO:0009891	30	3	0.002718597	positive regulation of biosynthesis

GO:0006139	2328	34	0.002742122	nucleobase, nucleoside, nucleotide and nucleic acid metabolism
GO:0050874	1531	25	0.002960393	organismal physiological process
GO:0006268	9	2	0.003018796	DNA unwinding
GO:0042035	33	3	0.003580005	regulation of cytokine biosynthesis
GO:0000074	394	10	0.003744999	regulation of progression through cell cycle
GO:0042089	36	3	0.004591013	cytokine biosynthesis
GO:0008283	473	11	0.004605225	cell proliferation
GO:0009611	341	9	0.004613042	response to wounding
GO:0042107	37	3	0.004962502	cytokine metabolism
GO:0006275	12	2	0.005433897	regulation of DNA replication
GO:0050776	77	4	0.005869415	regulation of immune response
GO:0044237	5026	59	0.006369021	cellular metabolism
GO:0051247	41	3	0.006627142	positive regulation of protein metabolism
GO:0046483	44	3	0.008068965	heterocycle metabolism
GO:0051239	133	5	0.008225846	regulation of organismal physiological process
GO:0016477	85	4	0.008298625	cell migration
GO:0007229	45	3	0.008587366	integrin-mediated signaling pathway
GO:0001556	1	1	0.009410983	oocyte maturation
GO:0045415	1	1	0.009410983	negative regulation of interleukin-8 biosynthesis
GO:0048160	1	1	0.009410983	primary follicle stage oogenesis
GO:0045740	1	1	0.009410983	positive regulation of DNA replication
GO:0045741	1	1	0.009410983	positive regulation of epidermal growth factor receptor activity
GO:0009076	1	1	0.009410983	histidine family amino acid biosynthesis
GO:0045653	1	1	0.009410983	negative regulation of megakaryocyte differentiation
GO:0050751	1	1	0.009410983	fractalkine biosynthesis
GO:0050752	1	1	0.009410983	regulation of fractalkine biosynthesis
GO:0050754	1	1	0.009410983	positive regulation of fractalkine biosynthesis
GO:0050756	1	1	0.009410983	fractalkine metabolism
GO:0050720	1	1	0.009410983	interleukin-1 beta biosynthesis
GO:0050722	1	1	0.009410983	regulation of interleukin-1 beta biosynthesis
GO:0050725	1	1	0.009410983	positive regulation of interleukin-1 beta biosynthesis
GO:0050728	1	1	0.009410983	negative regulation of inflammatory response
GO:0009162	1	1	0.009410983	deoxyribonucleoside monophosphate metabolism
GO:0009157	1	1	0.009410983	deoxyribonucleoside monophosphate biosynthesis
GO:0009176	1	1	0.009410983	pyrimidine deoxyribonucleoside monophosphate metabolism
GO:0009177	1	1	0.009410983	pyrimidine deoxyribonucleoside monophosphate biosynthesis
GO:0007143	1	1	0.009410983	female meiosis
GO:0009221	1	1	0.009410983	pyrimidine deoxyribonucleotide biosynthesis
GO:0042117	1	1	0.009410983	monocyte activation
GO:0007108	1	1	0.009410983	cytokinesis, initiation of separation
GO:0000105	1	1	0.009410983	histidine biosynthesis
GO:0046073	1	1	0.009410983	dTMP metabolism
GO:0042700	1	1	0.009410983	luteinizing hormone signaling pathway
GO:0051103	1	1	0.009410983	DNA ligation during DNA repair
GO:0050922	1	1	0.009410983	negative regulation of chemotaxis
GO:0006288	1	1	0.009410983	base-excision repair, DNA ligation
GO:0030728	1	1	0.009410983	ovulation

GO:0006231	1	1	0.009410983	dTMP biosynthesis
GO:0000920	1	1	0.009410983	cell separation during cytokinesis
GO:0000912	1	1	0.009410983	cytokinesis, formation of actomyosin apparatus
GO:0000915	1	1	0.009410983	cytokinesis, contractile ring formation
GO:0001774	1	1	0.009410983	microglial cell activation
GO:0045080	1	1	0.009410983	positive regulation of chemokine biosynthesis
GO:0051301	142	5	0.010741576	cell division
GO:0045596	17	2	0.010861027	negative regulation of cell differentiation
GO:0009161	17	2	0.010861027	ribonucleoside monophosphate metabolism
GO:0009156	17	2	0.010861027	ribonucleoside monophosphate biosynthesis
GO:0006270	17	2	0.010861027	DNA replication initiation
GO:0030216	18	2	0.012144565	keratinocyte differentiation
GO:0000910	18	2	0.012144565	cytokinesis
GO:0009165	103	4	0.015960936	nucleotide biosynthesis
GO:0019221	21	2	0.016368046	cytokine and chemokine mediated signaling pathway
GO:0009605	963	16	0.016532767	response to external stimulus
GO:0001550	2	1	0.018734432	ovarian cumulus expansion
GO:0001547	2	1	0.018734432	antral ovarian follicle growth
GO:0046653	2	1	0.018734432	tetrahydrofolate metabolism
GO:0045360	2	1	0.018734432	regulation of interleukin-1 biosynthesis
GO:0045362	2	1	0.018734432	positive regulation of interleukin-1 biosynthesis
GO:0045348	2	1	0.018734432	positive regulation of MHC class II biosynthesis
GO:0008347	2	1	0.018734432	glial cell migration
GO:0045123	2	1	0.018734432	cellular extravasation
GO:0048165	2	1	0.018734432	fused antrum stage oogenesis
GO:0048157	2	1	0.018734432	oogenesis (sensu Mammalia)
GO:0048146	2	1	0.018734432	positive regulation of fibroblast proliferation
GO:0048247	2	1	0.018734432	lymphocyte chemotaxis
GO:0042063	2	1	0.018734432	gliogenesis
GO:0000022	2	1	0.018734432	mitotic spindle elongation
GO:0045652	2	1	0.018734432	regulation of megakaryocyte differentiation
GO:0050829	2	1	0.018734432	defense response to Gram-negative bacteria
GO:0009186	2	1	0.018734432	deoxyribonucleoside diphosphate metabolism
GO:0007405	2	1	0.018734432	neuroblast proliferation
GO:0009263	2	1	0.018734432	deoxyribonucleotide biosynthesis
GO:0042222	2	1	0.018734432	interleukin-1 biosynthesis
GO:0051150	2	1	0.018734432	regulation of smooth muscle cell differentiation
GO:0051151	2	1	0.018734432	negative regulation of smooth muscle cell differentiation
GO:0051145	2	1	0.018734432	smooth muscle cell differentiation
GO:0051147	2	1	0.018734432	regulation of muscle cell differentiation
GO:0051148	2	1	0.018734432	negative regulation of muscle cell differentiation
GO:0051231	2	1	0.018734432	spindle elongation
GO:0006475	2	1	0.018734432	internal protein amino acid acetylation
GO:0007017	112	4	0.021047676	microtubule-based process
GO:0044238	4820	55	0.022228382	primary metabolism
GO:0008152	5262	59	0.022280627	metabolism
GO:0045765	25	2	0.022823107	regulation of angiogenesis

GO:0006220	26	2	0.024575969	pyrimidine nucleotide metabolism
GO:0006357	240	6	0.02529862	regulation of transcription from RNA polymerase II promoter
GO:0045342	3	1	0.027971151	MHC class II biosynthesis
GO:0045346	3	1	0.027971151	regulation of MHC class II biosynthesis
GO:0043114	3	1	0.027971151	regulation of vascular permeability
GO:0048246	3	1	0.027971151	macrophage chemotaxis
GO:0048599	3	1	0.027971151	oocyte development
GO:0009129	3	1	0.027971151	pyrimidine nucleoside monophosphate metabolism
GO:0009113	3	1	0.027971151	purine base biosynthesis
GO:0009130	3	1	0.027971151	pyrimidine nucleoside monophosphate biosynthesis
GO:0009132	3	1	0.027971151	nucleoside diphosphate metabolism
GO:0009299	3	1	0.027971151	mRNA transcription
GO:0007176	3	1	0.027971151	regulation of epidermal growth factor receptor activity
GO:0050680	3	1	0.027971151	negative regulation of epithelial cell proliferation
GO:0046040	3	1	0.027971151	IMP metabolism
GO:0042533	3	1	0.027971151	tumor necrosis factor-alpha biosynthesis
GO:0042534	3	1	0.027971151	regulation of tumor necrosis factor-alpha biosynthesis
GO:0042535	3	1	0.027971151	positive regulation of tumor necrosis factor-alpha biosynthesis
GO:0009994	3	1	0.027971151	oocyte differentiation
GO:0006188	3	1	0.027971151	IMP biosynthesis
GO:0006189	3	1	0.027971151	'de novo' IMP biosynthesis
GO:0030219	3	1	0.027971151	megakaryocyte differentiation
GO:0006266	3	1	0.027971151	DNA ligation
GO:0016998	3	1	0.027971151	cell wall catabolism
GO:0030851	3	1	0.027971151	granulocyte differentiation
GO:0045079	3	1	0.027971151	negative regulation of chemokine biosynthesis
GO:0031324	183	5	0.028843258	negative regulation of cellular metabolism
GO:0009628	318	7	0.029797917	response to abiotic stimulus
GO:0051052	29	2	0.030149918	regulation of DNA metabolism
GO:0006725	73	3	0.031214061	aromatic compound metabolism
GO:0006730	30	2	0.032109298	one-carbon compound metabolism
GO:0019735	77	3	0.035749505	antimicrobial humoral response (sensu Vertebrata)
GO:0048523	640	11	0.036855404	negative regulation of cellular process
GO:0019730	78	3	0.036933765	antimicrobial humoral response
GO:0045410	4	1	0.037121938	positive regulation of interleukin-6 biosynthesis
GO:0045414	4	1	0.037121938	regulation of interleukin-8 biosynthesis
GO:0045416	4	1	0.037121938	positive regulation of interleukin-8 biosynthesis
GO:0045408	4	1	0.037121938	regulation of interleukin-6 biosynthesis
GO:0048144	4	1	0.037121938	fibroblast proliferation
GO:0048145	4	1	0.037121938	regulation of fibroblast proliferation
GO:0006940	4	1	0.037121938	regulation of smooth muscle contraction
GO:0045785	4	1	0.037121938	positive regulation of cell adhesion
GO:0009086	4	1	0.037121938	methionine biosynthesis
GO:0050764	4	1	0.037121938	regulation of phagocytosis
GO:0050766	4	1	0.037121938	positive regulation of phagocytosis
GO:0009219	4	1	0.037121938	pyrimidine deoxyribonucleotide metabolism
GO:0042226	4	1	0.037121938	interleukin-6 biosynthesis

GO:0006144	4	1	0.037121938	purine base metabolism
GO:0045089	4	1	0.037121938	positive regulation of innate immune response
GO:0006793	641	11	0.037213643	phosphorus metabolism
GO:0006796	641	11	0.037213643	phosphate metabolism
GO:0007067	135	4	0.03818277	mitosis
GO:0000087	137	4	0.039962333	M phase of mitotic cell cycle
GO:0007610	269	6	0.040819207	behavior
GO:0000278	204	5	0.042939225	mitotic cell cycle
GO:0006928	205	5	0.043698166	cell motility
GO:0040011	205	5	0.043698166	locomotion
GO:0006547	5	1	0.046187582	histidine metabolism
GO:0048477	5	1	0.046187582	oogenesis
GO:0009075	5	1	0.046187582	histidine family amino acid metabolism
GO:0045840	5	1	0.046187582	positive regulation of mitosis
GO:0050727	5	1	0.046187582	regulation of inflammatory response
GO:0050716	5	1	0.046187582	positive regulation of interleukin-1 secretion
GO:0050718	5	1	0.046187582	positive regulation of interleukin-1 beta secretion
GO:0050701	5	1	0.046187582	interleukin-1 secretion
GO:0050702	5	1	0.046187582	interleukin-1 beta secretion
GO:0050704	5	1	0.046187582	regulation of interleukin-1 secretion
GO:0050706	5	1	0.046187582	regulation of interleukin-1 beta secretion
GO:0042228	5	1	0.046187582	interleukin-8 biosynthesis
GO:0050926	5	1	0.046187582	regulation of positive chemotaxis
GO:0050927	5	1	0.046187582	positive regulation of positive chemotaxis
GO:0050918	5	1	0.046187582	positive chemotaxis
GO:0050930	5	1	0.046187582	induction of positive chemotaxis
GO:0030236	5	1	0.046187582	anti-inflammatory response
GO:0045088	5	1	0.046187582	regulation of innate immune response
GO:0030593	5	1	0.046187582	neutrophil chemotaxis
GO:0051243	583	10	0.04632652	negative regulation of cellular physiological process
GO:0006366	426	8	0.046561542	transcription from RNA polymerase II promoter
GO:0007626	209	5	0.046814914	locomotory behavior
GO:0030099	37	2	0.047134635	myeloid cell differentiation
GO:0007088	39	2	0.051817927	regulation of mitosis
GO:0031323	1566	21	0.053158611	regulation of cellular metabolism
GO:0009892	217	5	0.053438813	negative regulation of metabolism
GO:0016481	151	4	0.053730366	negative regulation of transcription
GO:0051054	6	1	0.055168866	positive regulation of DNA metabolism
GO:0016478	6	1	0.055168866	negative regulation of translation
GO:0050921	6	1	0.055168866	positive regulation of chemotaxis
GO:0031032	6	1	0.055168866	actomyosin structure organization and biogenesis
GO:0043118	602	10	0.055400231	negative regulation of physiological process
GO:0016310	522	9	0.056011141	phosphorylation
GO:0006468	444	8	0.056811247	protein amino acid phosphorylation
GO:0048519	688	11	0.056957149	negative regulation of biological process
GO:0007582	7846	79	0.058307462	physiological process
GO:0006996	693	11	0.059407091	organelle organization and biogenesis

GO:0045934	159	4	0.062621465	negative regulation of nucleobase, nucleoside, nucleotide and nucleic acid metabolism
GO:0007165	2168	27	0.063012246	signal transduction
GO:0006555	7	1	0.064066567	methionine metabolism
GO:0045787	7	1	0.064066567	positive regulation of progression through cell cycle
GO:0009067	7	1	0.064066567	aspartate family amino acid biosynthesis
GO:0050715	7	1	0.064066567	positive regulation of cytokine secretion
GO:0050707	7	1	0.064066567	regulation of cytokine secretion
GO:0009411	7	1	0.064066567	response to UV
GO:0050663	7	1	0.064066567	cytokine secretion
GO:0050673	7	1	0.064066567	epithelial cell proliferation
GO:0050678	7	1	0.064066567	regulation of epithelial cell proliferation
GO:0042743	7	1	0.064066567	hydrogen peroxide metabolism
GO:0042744	7	1	0.064066567	hydrogen peroxide catabolism
GO:0006265	7	1	0.064066567	DNA topological change
GO:0007242	879	13	0.066514463	intracellular signaling cascade
GO:0007010	305	6	0.06712442	cytoskeleton organization and biogenesis
GO:0016064	103	3	0.072814322	humoral defense mechanism (sensu Vertebrata)
GO:0001541	8	1	0.072881453	ovarian follicle development
GO:0045807	8	1	0.072881453	positive regulation of endocytosis
GO:0000097	8	1	0.072881453	sulfur amino acid biosynthesis
GO:0009791	8	1	0.072881453	post-embryonic development
GO:0042542	8	1	0.072881453	response to hydrogen peroxide
GO:0042698	8	1	0.072881453	menstrual cycle
GO:0046112	8	1	0.072881453	nucleobase biosynthesis
GO:0006752	48	2	0.074756636	group transfer coenzyme metabolism
GO:0019222	1629	21	0.075288651	regulation of metabolism
GO:0050791	2407	29	0.077170228	regulation of physiological process
GO:0051093	49	2	0.077475898	negative regulation of development
GO:0043085	107	3	0.079609398	positive regulation of enzyme activity
GO:0006417	107	3	0.079609398	regulation of protein biosynthesis
GO:0000279	173	4	0.079930327	M phase
GO:0051244	2319	28	0.080869081	regulation of cellular physiological process
GO:0006281	174	4	0.081249881	DNA repair
GO:0001818	9	1	0.081614285	negative regulation of cytokine production
GO:0046545	9	1	0.081614285	development of primary female sexual characteristics
GO:0046660	9	1	0.081614285	female sex differentiation
GO:0045766	9	1	0.081614285	positive regulation of angiogenesis
GO:0042036	9	1	0.081614285	negative regulation of cytokine biosynthesis
GO:0008585	9	1	0.081614285	female gonad development
GO:0045638	9	1	0.081614285	negative regulation of myeloid cell differentiation
GO:0050714	9	1	0.081614285	positive regulation of protein secretion
GO:0007159	9	1	0.081614285	leukocyte adhesion
GO:0007281	9	1	0.081614285	germ cell development
GO:0019219	1458	19	0.082418527	regulation of nucleobase, nucleoside, nucleotide and nucleic acid metabolism
GO:0031326	111	3	0.08667162	regulation of cellular biosynthesis
GO:0045860	53	2	0.088654953	positive regulation of protein kinase activity
GO:0051347	54	2	0.09152127	positive regulation of transferase activity

GO:0045595	55	2	0.094414669	regulation of cell differentiation
GO:0045859	116	3	0.095860198	regulation of protein kinase activity
GO:0009889	116	3	0.095860198	regulation of biosynthesis
GO:0009056	756	11	0.09640735	catabolism
GO:0051338	117	3	0.097744448	regulation of transferase activity
GO:0007076	11	1	0.098836801	mitotic chromosome condensation
GO:0019884	11	1	0.098836801	antigen presentation, exogenous antigen
GO:0009167	11	1	0.098836801	purine ribonucleoside monophosphate metabolism
GO:0009168	11	1	0.098836801	purine ribonucleoside monophosphate biosynthesis
GO:0009126	11	1	0.098836801	purine nucleoside monophosphate metabolism
GO:0009127	11	1	0.098836801	purine nucleoside monophosphate biosynthesis
GO:0016525	11	1	0.098836801	negative regulation of angiogenesis
GO:0006350	1499	19	0.102016363	transcription
GO:0006351	1407	18	0.103312155	transcription, DNA-dependent
GO:0008284	121	3	0.105431185	positive regulation of cell proliferation
GO:0015669	12	1	0.107327974	gas transport
GO:0015671	12	1	0.107327974	oxygen transport
GO:0019886	12	1	0.107327974	antigen processing, exogenous antigen via MHC class II
GO:0050708	12	1	0.107327974	regulation of protein secretion
GO:0042327	12	1	0.107327974	positive regulation of phosphorylation
GO:0009112	12	1	0.107327974	nucleobase metabolism
GO:0045937	12	1	0.107327974	positive regulation of phosphate metabolism
GO:0030261	12	1	0.107327974	chromosome condensation
GO:0050794	2488	29	0.108876211	regulation of cellular process
GO:0000122	61	2	0.112302665	negative regulation of transcription from RNA polymerase II promoter
GO:0006164	61	2	0.112302665	purine nucleotide biosynthesis
GO:0007093	13	1	0.115740072	mitotic checkpoint
GO:0051047	13	1	0.115740072	positive regulation of secretion
GO:0000302	13	1	0.115740072	response to reactive oxygen species
GO:0030168	13	1	0.115740072	platelet activation
GO:0045449	1433	18	0.1177281	regulation of transcription
GO:0006163	63	2	0.118449354	purine nucleotide metabolism
GO:0006974	200	4	0.119222327	response to DNA damage stimulus
GO:0044248	700	10	0.120504353	cellular catabolism
GO:0006364	64	2	0.121554142	rRNA processing
GO:0007052	14	1	0.124073822	mitotic spindle organization and biogenesis
GO:0019835	14	1	0.124073822	cytolysis
GO:0001525	65	2	0.124679022	angiogenesis
GO:0009260	65	2	0.124679022	ribonucleotide biosynthesis
GO:0016072	66	2	0.127823351	rRNA metabolism
GO:0006508	451	7	0.131362905	proteolysis and peptidolysis
GO:0044257	451	7	0.131362905	cellular protein catabolism
GO:0006909	15	1	0.132329946	phagocytosis
GO:0009066	15	1	0.132329946	aspartate family amino acid metabolism
GO:0051050	15	1	0.132329946	positive regulation of transport
GO:0018149	15	1	0.132329946	peptide cross-linking
GO:0006879	15	1	0.132329946	iron ion homeostasis

GO:0001568	68	2	0.134167845	blood vessel development
GO:0048514	68	2	0.134167845	blood vessel morphogenesis
GO:0009259	68	2	0.134167845	ribonucleotide metabolism
GO:0031325	136	3	0.136232215	positive regulation of cellular metabolism
GO:0001944	69	2	0.137366777	vasculature development
GO:0045637	16	1	0.140509158	regulation of myeloid cell differentiation
GO:0050777	16	1	0.140509158	negative regulation of immune response
GO:0007157	16	1	0.140509158	heterophilic cell adhesion
GO:0007173	16	1	0.140509158	epidermal growth factor receptor signaling pathway
GO:0006473	16	1	0.140509158	protein amino acid acetylation
GO:0030100	16	1	0.140509158	regulation of endocytosis
GO:0006284	16	1	0.140509158	base-excision repair
GO:0050790	214	4	0.142362135	regulation of enzyme activity
GO:0009719	214	4	0.142362135	response to endogenous stimulus
GO:0030163	463	7	0.144797116	protein catabolism
GO:0009108	72	2	0.14706312	coenzyme biosynthesis
GO:0006959	141	3	0.147124645	humoral immune response
GO:0015758	17	1	0.148612166	glucose transport
GO:0006692	17	1	0.148612166	prostanoid metabolism
GO:0006693	17	1	0.148612166	prostaglandin metabolism
GO:0006904	17	1	0.148612166	vesicle docking during exocytosis
GO:0017148	17	1	0.148612166	negative regulation of protein biosynthesis
GO:0007292	17	1	0.148612166	female gamete generation
GO:0030574	17	1	0.148612166	collagen catabolism
GO:0045786	145	3	0.156039049	negative regulation of progression through cell cycle
GO:0015749	18	1	0.156639672	monosaccharide transport
GO:0031327	18	1	0.156639672	negative regulation of cellular biosynthesis
GO:0048278	18	1	0.156639672	vesicle docking
GO:0008645	18	1	0.156639672	hexose transport
GO:0006221	18	1	0.156639672	pyrimidine nucleotide biosynthesis
GO:0043285	474	7	0.157677886	biopolymer catabolism
GO:0050789	2689	30	0.158670737	regulation of biological process
GO:0044265	563	8	0.158883556	cellular macromolecule catabolism
GO:0006939	19	1	0.164592371	smooth muscle contraction
GO:0009890	19	1	0.164592371	negative regulation of biosynthesis
GO:0042692	19	1	0.164592371	muscle cell differentiation
GO:0006304	19	1	0.164592371	DNA modification
GO:0009893	149	3	0.165119738	positive regulation of metabolism
GO:0016043	842	11	0.165526127	cell organization and biogenesis
GO:0007046	79	2	0.170195567	ribosome biogenesis
GO:0009057	575	8	0.172340925	macromolecule catabolism
GO:0046916	20	1	0.172470951	transition metal ion homeostasis
GO:0000096	20	1	0.172470951	sulfur amino acid metabolism
GO:0000070	20	1	0.172470951	mitotic sister chromatid segregation
GO:0007205	20	1	0.172470951	protein kinase C activation
GO:0051241	20	1	0.172470951	negative regulation of organismal physiological process
GO:0042254	80	2	0.173550266	ribosome biogenesis and assembly

GO:0048469	21	1	0.180276097	cell maturation
GO:0000819	21	1	0.180276097	sister chromatid segregation
GO:0007599	83	2	0.183678192	hemostasis
GO:0006937	22	1	0.188008483	regulation of muscle contraction
GO:0051242	411	6	0.18968587	positive regulation of cellular physiological process
GO:0042060	85	2	0.190478483	wound healing
GO:0051188	85	2	0.190478483	cofactor biosynthesis
GO:0008406	23	1	0.19566878	gonad development
GO:0045137	23	1	0.19566878	development of primary sexual characteristics
GO:0009116	23	1	0.19566878	nucleoside metabolism
GO:0030097	87	2	0.197313066	hemopoiesis
GO:0030005	87	2	0.197313066	di-, tri-valent inorganic cation homeostasis
GO:0048534	88	2	0.200742083	hemopoietic or lymphoid organ development
GO:0006355	1359	16	0.201048863	regulation of transcription, DNA-dependent
GO:0051046	24	1	0.203257653	regulation of secretion
GO:0044272	24	1	0.203257653	sulfur compound biosynthesis
GO:0042325	25	1	0.210775759	regulation of phosphorylation
GO:0043119	428	6	0.21468476	positive regulation of physiological process
GO:0006875	93	2	0.217987022	metal ion homeostasis
GO:0008643	26	1	0.218223751	carbohydrate transport
GO:0009306	26	1	0.218223751	protein secretion
GO:0007498	26	1	0.218223751	mesoderm development
GO:0050878	94	2	0.221453051	regulation of body fluids
GO:0051246	174	3	0.224968367	regulation of protein metabolism
GO:0006919	27	1	0.225602274	caspase activation
GO:0007028	96	2	0.228398928	cytoplasm organization and biogenesis
GO:0045321	97	2	0.231878003	immune cell activation
GO:0045892	97	2	0.231878003	negative regulation of transcription, DNA-dependent
GO:0019220	28	1	0.232911969	regulation of phosphate metabolism
GO:0007259	28	1	0.232911969	JAK-STAT cascade
GO:0051174	28	1	0.232911969	regulation of phosphorus metabolism
GO:0030003	98	2	0.235360663	cation homeostasis
GO:0001775	98	2	0.235360663	cell activation
GO:0006470	99	2	0.238846538	protein amino acid dephosphorylation
GO:0048511	29	1	0.240153471	rhythmic process
GO:0042113	30	1	0.247327408	B cell activation
GO:0008015	103	2	0.252815004	circulation
GO:0006873	103	2	0.252815004	cell ion homeostasis
GO:0043406	31	1	0.254434403	positive regulation of MAPK activity
GO:0043280	31	1	0.254434403	positive regulation of caspase activity
GO:0043281	31	1	0.254434403	regulation of caspase activity
GO:0006511	105	2	0.259809364	ubiquitin-dependent protein catabolism
GO:0019941	105	2	0.259809364	modification-dependent protein catabolism
GO:0051345	32	1	0.261475074	positive regulation of hydrolase activity
GO:0050875	7018	69	0.265035082	cellular physiological process
GO:0006690	33	1	0.268450031	icosanoid metabolism
GO:0009314	33	1	0.268450031	response to radiation

GO:0030155	33	1	0.268450031	regulation of cell adhesion
GO:0006401	33	1	0.268450031	RNA catabolism
GO:0006323	193	3	0.272941104	DNA packaging
GO:0007059	34	1	0.275359882	chromosome segregation
GO:0008652	34	1	0.275359882	amino acid biosynthesis
GO:0006461	194	3	0.275502638	protein complex assembly
GO:0016311	110	2	0.277303727	dephosphorylation
GO:0050801	111	2	0.280801564	ion homeostasis
GO:0007548	35	1	0.282205227	sex differentiation
GO:0006916	112	2	0.28429836	anti-apoptosis
GO:0000079	36	1	0.28898666	regulation of cyclin dependent protein kinase activity
GO:0030333	36	1	0.28898666	antigen processing
GO:0043412	1160	13	0.293036626	biopolymer modification
GO:0000075	37	1	0.295704772	cell cycle checkpoint
GO:0007169	116	2	0.298269474	transmembrane receptor protein tyrosine kinase signaling pathway
GO:0048522	482	6	0.300557277	positive regulation of cellular process
GO:0016070	391	5	0.307108013	RNA metabolism
GO:0048468	40	1	0.315484995	cell development
GO:0006732	121	2	0.315677055	coenzyme metabolism
GO:0019725	122	2	0.319148514	cell homeostasis
GO:0019882	41	1	0.32195561	antigen presentation
GO:0006464	1087	12	0.323028868	protein modification
GO:0042981	306	4	0.325193809	regulation of apoptosis
GO:0044267	2109	22	0.328630642	cellular protein metabolism
GO:0043067	308	4	0.329491772	regulation of programmed cell death
GO:0007154	2730	28	0.32950862	cell communication
GO:0007276	125	2	0.329538477	gametogenesis
GO:0030036	126	2	0.332992901	actin cytoskeleton organization and biogenesis
GO:0006865	43	1	0.334716041	amino acid transport
GO:0043066	128	2	0.339887088	negative regulation of apoptosis
GO:0006979	44	1	0.341006967	response to oxidative stress
GO:0000086	44	1	0.341006967	G2/M transition of mitotic cell cycle
GO:0007126	44	1	0.341006967	meiosis
GO:0051321	44	1	0.341006967	meiotic cell cycle
GO:0051327	44	1	0.341006967	M phase of meiotic cell cycle
GO:0043069	129	2	0.343326428	negative regulation of programmed cell death
GO:0019538	2129	22	0.346541875	protein metabolism
GO:0051049	46	1	0.353412982	regulation of transport
GO:0006334	48	1	0.365588141	nucleosome assembly
GO:0006790	48	1	0.365588141	sulfur metabolism
GO:0006396	326	4	0.368285303	RNA processing
GO:0043405	49	1	0.371590479	regulation of MAPK activity
GO:0009309	49	1	0.371590479	amine biosynthesis
GO:0007586	49	1	0.371590479	digestion
GO:0044271	49	1	0.371590479	nitrogen compound biosynthesis
GO:0030029	139	2	0.377388549	actin filament-based process
GO:0007275	1543	16	0.377556973	development

GO:0051276	236	3	0.383932264	chromosome organization and biogenesis
GO:0015837	52	1	0.389262797	amine transport
GO:0051186	143	2	0.390819549	cofactor metabolism
GO:0000082	53	1	0.395043717	G1/S transition of mitotic cell cycle
GO:0008632	53	1	0.395043717	apoptotic program
GO:0045087	53	1	0.395043717	innate immune response
GO:0006887	55	1	0.406443813	exocytosis
GO:0007166	952	10	0.406682099	cell surface receptor linked signal transduction
GO:0043170	2405	24	0.408461378	macromolecule metabolism
GO:0019953	149	2	0.410726285	sexual reproduction
GO:0031497	56	1	0.412063984	chromatin assembly
GO:0007218	56	1	0.412063984	neuropeptide signaling pathway
GO:0007200	56	1	0.412063984	G-protein signaling, coupled to IP3 second messenger (phospholipase C activating)
GO:0009152	58	1	0.423147027	purine ribonucleotide biosynthesis
GO:0051248	58	1	0.423147027	negative regulation of protein metabolism
GO:0015849	59	1	0.428610867	organic acid transport
GO:0046942	59	1	0.428610867	carboxylic acid transport
GO:0044249	763	8	0.429359267	cellular biosynthesis
GO:0009150	60	1	0.434023558	purine ribonucleotide metabolism
GO:0051336	60	1	0.434023558	regulation of hydrolase activity
GO:0042592	157	2	0.436775258	homeostasis
GO:0006800	61	1	0.439385573	oxygen and reactive oxygen species metabolism
GO:0007050	61	1	0.439385573	cell cycle arrest
GO:0048609	61	1	0.439385573	reproductive organismal physiological process
GO:0048518	565	6	0.441485521	positive regulation of biological process
GO:0050876	62	1	0.444697381	reproductive physiological process
GO:0006445	64	1	0.455172228	regulation of translation
GO:0042127	265	3	0.457082317	regulation of cell proliferation
GO:0009790	65	1	0.460336185	embryonic development
GO:0007167	165	2	0.462205904	enzyme linked receptor protein signaling pathway
GO:0016042	67	1	0.470519422	lipid catabolism
GO:0050793	168	2	0.4715727	regulation of development
GO:0006874	68	1	0.475539595	calcium ion homeostasis
GO:0006644	73	1	0.499943465	phospholipid metabolism
GO:0044260	2298	22	0.504537592	cellular macromolecule metabolism
GO:0008219	498	5	0.507103232	cell death
GO:0016265	501	5	0.51250369	death
GO:0007596	78	1	0.523224532	blood coagulation
GO:0006325	186	2	0.525691978	establishment and/or maintenance of chromatin architecture
GO:0050817	79	1	0.527750544	coagulation
GO:0051329	79	1	0.527750544	interphase of mitotic cell cycle
GO:0046649	81	1	0.536675569	lymphocyte activation
GO:0051325	82	1	0.541075366	interphase
GO:0030154	409	4	0.541283248	cell differentiation
GO:0007155	518	5	0.542656167	cell adhesion
GO:0009967	85	1	0.554028518	positive regulation of signal transduction
GO:0006968	87	1	0.562462542	cellular defense response

GO:0009058	859	8	0.566918271	biosynthesis
GO:0006333	91	1	0.578860524	chromatin assembly or disassembly
GO:0048513	872	8	0.584582671	organ development
GO:0046903	208	2	0.586683466	secretion
GO:0000003	208	2	0.586683466	reproduction
GO:0009987	8200	77	0.617737656	cellular process
GO:0007001	221	2	0.619957358	chromosome organization and biogenesis (sensu Eukaryota)
GO:0048232	105	1	0.631612686	male gamete generation
GO:0045935	105	1	0.631612686	positive regulation of nucleobase, nucleoside, nucleotide and nucleic acid metabolism
GO:0007283	105	1	0.631612686	spermatogenesis
GO:0006643	106	1	0.635120351	membrane lipid metabolism
GO:0009887	807	7	0.645727283	organogenesis
GO:0006915	471	4	0.653979849	apoptosis
GO:0012501	473	4	0.657299707	programmed cell death
GO:0019752	359	3	0.662796879	carboxylic acid metabolism
GO:0006897	115	1	0.665240151	endocytosis
GO:0006082	361	3	0.666515703	organic acid metabolism
GO:0000398	119	1	0.677825083	nuclear mRNA splicing, via spliceosome
GO:0000375	119	1	0.677825083	RNA splicing, via transesterification reactions
GO:0000377	119	1	0.677825083	RNA splicing, via transesterification reactions with bulged adenosine as nucleophile
GO:0043037	122	1	0.686955865	translation
GO:0006631	124	1	0.692900491	fatty acid metabolism
GO:0006118	256	2	0.699368092	electron transport
GO:0006936	132	1	0.715583669	muscle contraction
GO:0008380	137	1	0.728911326	RNA splicing
GO:0008285	139	1	0.734067869	negative regulation of cell proliferation
GO:0006917	140	1	0.736609669	induction of apoptosis
GO:0012502	140	1	0.736609669	induction of programmed cell death
GO:0006412	407	3	0.744019065	protein biosynthesis
GO:0050953	149	1	0.75843182	sensory perception of light
GO:0007601	149	1	0.75843182	visual perception
GO:0043065	155	1	0.771977127	positive regulation of apoptosis
GO:0016192	296	2	0.772992566	vesicle-mediated transport
GO:0043068	156	1	0.774160516	positive regulation of programmed cell death
GO:0009888	158	1	0.778465498	histogenesis
GO:0045045	161	1	0.784771355	secretory pathway
GO:0016337	172	1	0.806411828	cell-cell adhesion
GO:0009059	464	3	0.819687464	macromolecule biosynthesis
GO:0006397	180	1	0.820785666	mRNA processing
GO:0006520	181	1	0.822506545	amino acid metabolism
GO:0016071	205	1	0.859243063	mRNA metabolism
GO:0006519	216	1	0.873462892	amino acid and derivative metabolism
GO:0009653	1051	7	0.879444783	morphogenesis
GO:0044255	383	2	0.881384161	cellular lipid metabolism
GO:0007399	385	2	0.883201874	neurogenesis
GO:0009966	229	1	0.888446873	regulation of signal transduction
GO:0007243	242	1	0.901674919	protein kinase cascade

GO:0006512	255	1	0.913350642	ubiquitin cycle
GO:0009308	269	1	0.924394752	amine metabolism
GO:0006091	446	2	0.9277686	generation of precursor metabolites and energy
GO:0007600	287	1	0.936572063	sensory perception
GO:0000004	465	2	0.93802766	biological process unknown
GO:0006807	291	1	0.93900279	nitrogen compound metabolism
GO:0006629	469	2	0.940006162	lipid metabolism
GO:0007186	491	2	0.949870484	G-protein coupled receptor protein signaling pathway
GO:0009581	313	1	0.950815089	detection of external stimulus
GO:0005975	355	1	0.967437801	carbohydrate metabolism
GO:0045184	459	1	0.988374649	establishment of protein localization
GO:0007267	465	1	0.989049505	cell-cell signaling
GO:0008104	474	1	0.989989841	protein localization
GO:0050877	475	1	0.990089265	neurophysiological process
GO:0051234	1841	8	0.998050692	establishment of localization
GO:0051179	1857	8	0.998268873	localization
GO:0006810	1802	7	0.999147043	transport
GO:0008150	9032	85	1	biological_process

22.3 GO: Molecular Function

GO ID	genes present in chip	genes present in tissues	p-value	GO term
GO:0004488	2	2	8.03110134634657e-05	methylenetetrahydrofolate dehydrogenase (NADP+) activity
GO:0004477	2	2	8.03110134634657e-05	methenyltetrahydrofolate cyclohydrolase activity
GO:0004486	3	2	0.000239518610129169	methylenetetrahydrofolate dehydrogenase activity
GO:0019238	4	2	0.000476226822366149	cyclohydrolase activity
GO:0042802	158	7	0.000537124335279596	protein self binding
GO:0016646	11	2	0.00418972066027776	oxidoreductase activity, acting on the CH-NH group of donors, NAD or NADP as acceptor
GO:0030246	181	6	0.00575623543270757	carbohydrate binding
GO:0003676	1990	28	0.00734524418870864	nucleic acid binding
GO:0019887	47	3	0.00861651085133779	protein kinase regulator activity
GO:0004638	1	1	0.00901373021695806	phosphoribosylaminoimidazole carboxylase activity
GO:0004639	1	1	0.00901373021695806	phosphoribosylaminoimidazolesuccinocarboxamide synthase activity
GO:0047017	1	1	0.00901373021695806	prostaglandin-F synthase activity
GO:0042083	1	1	0.00901373021695806	5,10-methylenetetrahydrofolate-dependent methyltransferase activity
GO:0030337	1	1	0.00901373021695806	DNA polymerase processivity factor activity
GO:0016538	1	1	0.00901373021695806	cyclin-dependent protein kinase regulator activity
GO:0004329	1	1	0.00901373021695806	formate-tetrahydrofolate ligase activity
GO:0004446	1	1	0.00901373021695806	multiple inositol-polyphosphate phosphatase activity
GO:0004137	1	1	0.00901373021695806	deoxycytidine kinase activity
GO:0004261	1	1	0.00901373021695806	cathepsin G activity
GO:0004799	1	1	0.00901373021695806	thymidylate synthase activity
GO:0003677	1384	21	0.00992822082525492	DNA binding
GO:0016645	18	2	0.0111881331394162	oxidoreductase activity, acting on the CH-NH group of donors
GO:0005529	103	4	0.0138407277337898	sugar binding
GO:0004252	105	4	0.0147639366922155	serine-type endopeptidase activity
GO:0016814	21	2	0.0150904317574965	hydrolase activity, acting on carbon-nitrogen (but not peptide) bonds, in cyclic amidines
GO:0019207	58	3	0.0152773523040730	kinase regulator activity
GO:0008201	60	3	0.0167270843647763	heparin binding
GO:0043014	2	1	0.0179471494204538	alpha-tubulin binding
GO:0030676	2	1	0.0179471494204538	Rac guanyl-nucleotide exchange factor activity
GO:0047026	2	1	0.0179471494204538	3-alpha-hydroxysteroid dehydrogenase (A-specific) activity
GO:0008934	2	1	0.0179471494204538	inositol-1(or 4)-monophosphatase activity
GO:0003912	2	1	0.0179471494204538	DNA nucleotidylexotransferase activity
GO:0016725	2	1	0.0179471494204538	oxidoreductase activity, acting on CH2 groups
GO:0016728	2	1	0.0179471494204538	oxidoreductase activity, acting on CH2 groups, disulfide as acceptor
GO:0004487	2	1	0.0179471494204538	methylenetetrahydrofolate dehydrogenase (NAD+) activity
GO:0004748	2	1	0.0179471494204538	ribonucleoside-diphosphate reductase activity
GO:0008236	117	4	0.0211206150523914	serine-type peptidase activity
GO:0004437	26	2	0.0226861570158885	inositol or phosphatidylinositol phosphatase activity
GO:0003887	28	2	0.0260819307021911	DNA-directed DNA polymerase activity
GO:0015643	3	1	0.0268009648256162	toxin binding
GO:0003796	3	1	0.0268009648256162	lysozyme activity
GO:0047115	3	1	0.0268009648256162	trans-1,2-dihydrobenzene-1,2-diol dehydrogenase activity
GO:0042608	3	1	0.0268009648256162	T cell receptor binding

GO:0016978	3	1	0.0268009648256162	lipoate-protein ligase B activity
GO:0005488	7228	73	0.0272347340142241	binding
GO:0003823	30	2	0.0296697994510363	antigen binding
GO:0005539	77	3	0.0320782689923180	glycosaminoglycan binding
GO:0030247	80	3	0.0353486376506015	polysaccharide binding
GO:0043015	4	1	0.035575877493365	gamma-tubulin binding
GO:0019136	4	1	0.035575877493365	deoxynucleoside kinase activity
GO:0042301	4	1	0.035575877493365	phosphate binding
GO:0003918	4	1	0.035575877493365	DNA topoisomerase (ATP-hydrolyzing) activity
GO:0016979	4	1	0.035575877493365	lipoate-protein ligase activity
GO:0008094	34	2	0.037390760307803	DNA-dependent ATPase activity
GO:0030234	509	9	0.039180091688237	enzyme regulator activity
GO:0008301	5	1	0.044272582383328	DNA bending activity
GO:0048487	5	1	0.044272582383328	beta-tubulin binding
GO:0019863	5	1	0.044272582383328	IgE binding
GO:0004749	5	1	0.044272582383328	ribose phosphate diphosphokinase activity
GO:0001871	88	3	0.04487684101318	pattern binding
GO:0008009	40	2	0.0502242672189512	chemokine activity
GO:0042379	40	2	0.0502242672189512	chemokine receptor binding
GO:0004622	6	1	0.0528917684062971	lysophospholipase activity
GO:0005154	6	1	0.0528917684062971	epidermal growth factor receptor binding
GO:0003916	6	1	0.0528917684062971	DNA topoisomerase activity
GO:0042708	6	1	0.0528917684062971	elastase activity
GO:0016778	6	1	0.0528917684062971	diphosphotransferase activity
GO:0004303	6	1	0.0528917684062971	estradiol 17-beta-dehydrogenase activity
GO:0001664	42	2	0.054807798263928	G-protein-coupled receptor binding
GO:0004866	99	3	0.0598380766235322	endopeptidase inhibitor activity
GO:0030414	100	3	0.0613013246035846	protease inhibitor activity
GO:0004540	45	2	0.0619467057395943	ribonuclease activity
GO:0003700	649	10	0.0660608461724506	transcription factor activity
GO:0046983	170	4	0.06718929010817	protein dimerization activity
GO:0030554	1028	14	0.0751976639001917	adenyl nucleotide binding
GO:0000287	251	5	0.0758848720966404	magnesium ion binding
GO:0004522	9	1	0.0782910127380442	pancreatic ribonuclease activity
GO:0008367	9	1	0.0782910127380442	bacterial binding
GO:0003993	9	1	0.0782910127380442	acid phosphatase activity
GO:0030552	9	1	0.0782910127380442	3',5'-cAMP binding
GO:0016787	1424	18	0.0822984891056686	hydrolase activity
GO:0019843	10	1	0.0866068932357914	rRNA binding
GO:0008603	10	1	0.0866068932357914	cAMP-dependent protein kinase regulator activity
GO:0000175	10	1	0.0866068932357914	3'-5'-exoribonuclease activity
GO:0016208	10	1	0.0866068932357914	AMP binding
GO:0004033	10	1	0.0866068932357914	aldo-keto reductase activity
GO:0004716	10	1	0.0866068932357914	receptor signaling protein tyrosine kinase activity
GO:0004857	187	4	0.0882191928182482	enzyme inhibitor activity
GO:0030528	963	13	0.0897608175971678	transcription regulator activity
GO:0005524	970	13	0.0937361867108663	ATP binding

GO:0004532	11	1	0.0948486104933425	exoribonuclease activity
GO:0005355	11	1	0.0948486104933425	glucose transporter activity
GO:0019206	11	1	0.0948486104933425	nucleoside kinase activity
GO:0016892	11	1	0.0948486104933425	endoribonuclease activity, producing 3'-phosphomonoesters
GO:0016896	11	1	0.0948486104933425	exoribonuclease activity, producing 5'-phosphomonoesters
GO:0003824	3409	37	0.0970380569735922	catalytic activity
GO:0005102	528	8	0.102458224028231	receptor binding
GO:0005344	12	1	0.103016818205576	oxygen transporter activity
GO:0019865	12	1	0.103016818205576	immunoglobulin binding
GO:0008656	12	1	0.103016818205576	caspase activator activity
GO:0015145	12	1	0.103016818205576	monosaccharide transporter activity
GO:0015149	12	1	0.103016818205576	hexose transporter activity
GO:0045012	12	1	0.103016818205576	MHC class II receptor activity
GO:0005021	12	1	0.103016818205576	vascular endothelial growth factor receptor activity
GO:0016772	708	10	0.103396402252151	transferase activity, transferring phosphorus-containing groups
GO:0016879	127	3	0.106732116693997	ligase activity, forming carbon-nitrogen bonds
GO:0016894	13	1	0.111112164373517	endonuclease activity, active with either ribo- or deoxyribonucleic acids and producing 3'-phosphomonoesters
GO:0004867	64	2	0.113248454497642	serine-type endopeptidase inhibitor activity
GO:0004175	288	5	0.118028243119511	endopeptidase activity
GO:0030693	14	1	0.119135291353353	caspase activity
GO:0004091	14	1	0.119135291353353	carboxylesterase activity
GO:0016505	14	1	0.119135291353353	apoptotic protease activator activity
GO:0016628	14	1	0.119135291353353	oxidoreductase activity, acting on the CH-CH group of donors, NAD or NADP as acceptor
GO:0004759	14	1	0.119135291353353	serine esterase activity
GO:0005506	212	4	0.124179482281895	iron ion binding
GO:0008134	293	5	0.124442101877526	transcription factor binding
GO:0016229	15	1	0.127086835905008	steroid dehydrogenase activity
GO:0016504	15	1	0.127086835905008	protease activator activity
GO:0004715	15	1	0.127086835905008	non-membrane spanning protein tyrosine kinase activity
GO:0030674	69	2	0.128130930847711	protein binding, bridging
GO:0005515	3709	39	0.130409223709656	protein binding
GO:0003713	140	3	0.132199044538255	transcription coactivator activity
GO:0003705	16	1	0.134967429240318	RNA polymerase II transcription factor activity, enhancer binding
GO:0004468	16	1	0.134967429240318	lysine N-acetyltransferase activity
GO:0004402	16	1	0.134967429240318	histone acetyltransferase activity
GO:0016810	72	2	0.137271318702584	hydrolase activity, acting on carbon-nitrogen (but not peptide) bonds
GO:0016874	221	4	0.138457138887233	ligase activity
GO:0008083	144	3	0.140432515851589	growth factor activity
GO:0016796	17	1	0.142777697070799	exonuclease activity, active with either ribo- or deoxyribonucleic acids and producing 5'-phosphomonoesters
GO:0030551	18	1	0.150518259654998	cyclic nucleotide binding
GO:0003712	230	4	0.153369423342865	transcription cofactor activity
GO:0043028	19	1	0.158189731845450	caspase regulator activity
GO:0004869	19	1	0.158189731845450	cysteine protease inhibitor activity
GO:0003690	20	1	0.165792723135231	double-stranded DNA binding
GO:0004713	161	3	0.177207066841198	protein-tyrosine kinase activity
GO:0004601	22	1	0.180795674464353	peroxidase activity
GO:0016684	22	1	0.180795674464353	oxidoreductase activity, acting on peroxide as acceptor

GO:0016301	609	8	0.182058614256199	kinase activity
GO:0008408	24	1	0.195531884142048	3'-5' exonuclease activity
GO:0019200	24	1	0.195531884142048	carbohydrate kinase activity
GO:0016831	24	1	0.195531884142048	carboxy-lyase activity
GO:0016887	255	4	0.197712194111856	ATPase activity
GO:0016791	171	3	0.199978404350899	phosphoric monoester hydrolase activity
GO:0004860	25	1	0.202801428952785	protein kinase inhibitor activity
GO:0005178	26	1	0.210006039830262	integrin binding
GO:0003725	26	1	0.210006039830262	double-stranded RNA binding
GO:0016779	95	2	0.211050851480961	nucleotidyltransferase activity
GO:0016788	446	6	0.213522509210144	hydrolase activity, acting on ester bonds
GO:0008080	28	1	0.224222747774608	N-acetyltransferase activity
GO:0003697	28	1	0.224222747774608	single-stranded DNA binding
GO:0017076	1248	14	0.229067662739865	purine nucleotide binding
GO:0019210	29	1	0.231235976376666	kinase inhibitor activity
GO:0051119	29	1	0.231235976376666	sugar transporter activity
GO:0015144	30	1	0.238186534201688	carbohydrate transporter activity
GO:0008047	188	3	0.240137219047977	enzyme activator activity
GO:0004518	105	2	0.244323880685734	nuclease activity
GO:0004521	31	1	0.245074974750385	endoribonuclease activity
GO:0019825	31	1	0.245074974750385	oxygen binding
GO:0016209	31	1	0.245074974750385	antioxidant activity
GO:0016830	31	1	0.245074974750385	carbon-carbon lyase activity
GO:0016410	33	1	0.258667693909492	N-acyltransferase activity
GO:0019205	34	1	0.265373055533786	nucleobase, nucleoside, nucleotide kinase activity
GO:0016627	37	1	0.285131547822592	oxidoreductase activity, acting on the CH-CH group of donors
GO:0008233	399	5	0.290796243019442	peptidase activity
GO:0016407	39	1	0.298011122208656	acetyltransferase activity
GO:0004527	40	1	0.304364631310955	exonuclease activity
GO:0000166	1432	15	0.305273625267032	nucleotide binding
GO:0015631	42	1	0.316901628501199	tubulin binding
GO:0019992	42	1	0.316901628501199	diacylglycerol binding
GO:0005057	132	2	0.334452077660474	receptor signaling protein activity
GO:0042578	228	3	0.338447714161423	phosphoric ester hydrolase activity
GO:0042623	229	3	0.340926896485276	ATPase activity, coupled
GO:0016773	532	6	0.346868920692777	phosphotransferase activity, alcohol group as acceptor
GO:0004620	47	1	0.347275481406635	phospholipase activity
GO:0003682	47	1	0.347275481406635	chromatin binding
GO:0015171	48	1	0.353188090275292	amino acid transporter activity
GO:0003723	436	5	0.35702166401608	RNA binding
GO:0016740	1169	12	0.36144520774956	transferase activity
GO:0004672	447	5	0.376939033814082	protein kinase activity
GO:0005275	58	1	0.409478375326103	amine transporter activity
GO:0004553	59	1	0.414833733305851	hydrolase activity, hydrolyzing O-glycosyl compounds
GO:0016298	59	1	0.414833733305851	lipase activity
GO:0016564	59	1	0.414833733305851	transcriptional repressor activity
GO:0004714	60	1	0.420141083963486	transmembrane receptor protein tyrosine kinase activity

GO:0046982	61	1	0.425400852635477	protein heterodimerization activity
GO:0016563	62	1	0.430613460934353	transcriptional activator activity
GO:0042803	64	1	0.440898864436215	protein homodimerization activity
GO:0004519	65	1	0.445972484533132	endonuclease activity
GO:0019955	65	1	0.445972484533132	cytokine binding
GO:0005342	66	1	0.451000594107863	organic acid transporter activity
GO:0046943	66	1	0.451000594107863	carboxylic acid transporter activity
GO:0019199	73	1	0.484956516496547	transmembrane receptor protein kinase activity
GO:0016491	509	5	0.488002884470739	oxidoreductase activity
GO:0005125	181	2	0.488004259500676	cytokine activity
GO:0046906	74	1	0.489634773740029	tetrapyrrole binding
GO:0020037	74	1	0.489634773740029	heme binding
GO:0004725	75	1	0.494271026983756	protein tyrosine phosphatase activity
GO:0004871	1837	17	0.495248259841742	signal transducer activity
GO:0016798	77	1	0.50341900915453	hydrolase activity, acting on glycosyl bonds
GO:0016616	77	1	0.50341900915453	oxidoreductase activity, acting on the CH-OH group of donors, NAD or NADP as acceptor
GO:0017111	410	4	0.508758435986814	nucleoside-triphosphatase activity
GO:0016789	80	1	0.516835165221171	carboxylic ester hydrolase activity
GO:0004197	80	1	0.516835165221171	cysteine-type endopeptidase activity
GO:0003714	81	1	0.521227108545447	transcription corepressor activity
GO:0016614	83	1	0.529892958159345	oxidoreductase activity, acting on CH-OH group of donors
GO:0003702	197	2	0.533369448280737	RNA polymerase II transcription factor activity
GO:0008168	84	1	0.534167562261511	methyltransferase activity
GO:0016741	87	1	0.546762214599788	transferase activity, transferring one-carbon groups
GO:0016462	430	4	0.546763841536163	pyrophosphatase activity
GO:0016818	432	4	0.550484200417964	hydrolase activity, acting on acid anhydrides, in phosphorus-containing anhydrides
GO:0016817	433	4	0.552338616319808	hydrolase activity, acting on acid anhydrides
GO:0016853	92	1	0.567009556855623	isomerase activity
GO:0004674	326	3	0.567571161463938	protein serine/threonine kinase activity
GO:0008026	93	1	0.570950416005842	ATP-dependent helicase activity
GO:0005085	93	1	0.570950416005842	guanyl-nucleotide exchange factor activity
GO:0008415	98	1	0.590129159581528	acyltransferase activity
GO:0008234	98	1	0.590129159581528	cysteine-type peptidase activity
GO:0016747	98	1	0.590129159581528	transferase activity, transferring groups other than amino-acyl groups
GO:0030695	219	2	0.591208303095678	GTPase regulator activity
GO:0016881	101	1	0.601227066356441	acid-amino acid ligase activity
GO:0004386	101	1	0.601227066356441	helicase activity
GO:0009055	104	1	0.612027879986468	electron carrier activity
GO:0016746	104	1	0.612027879986468	transferase activity, transferring acyl groups
GO:0005096	117	1	0.655585996607583	GTPase activator activity
GO:0004721	118	1	0.658728993667403	phosphoprotein phosphatase activity
GO:0016829	127	1	0.685770025075604	lyase activity
GO:0008092	279	2	0.721675541732783	cytoskeletal protein binding
GO:0005083	144	1	0.731197355366113	small GTPase regulator activity
GO:0005489	152	1	0.750265595492102	electron transporter activity
GO:0004872	1060	8	0.753509133963591	receptor activity
GO:0005261	188	1	0.820798825688958	cation channel activity

GO:0003779	189	1	0.822446564488354	actin binding
GO:0005509	646	4	0.843015216103829	calcium ion binding
GO:0005554	522	3	0.856947280425024	molecular function unknown
GO:0004888	694	4	0.8807505041268	transmembrane receptor activity
GO:0046872	2495	18	0.892823431665318	metal ion binding
GO:0005216	252	1	0.900981854274965	ion channel activity
GO:0043167	2556	18	0.915191249661221	ion binding
GO:0015268	274	1	0.919324101801648	alpha-type channel activity
GO:0015267	284	1	0.926509316777705	channel or pore class transporter activity
GO:0046914	1596	10	0.92764801824428	transition metal ion binding
GO:0008289	298	1	0.935517446514902	lipid binding
GO:0008270	1287	7	0.955326940787637	zinc ion binding
GO:0005215	1030	5	0.962639750548752	transporter activity
GO:0005386	398	1	0.974807682821566	carrier activity
GO:0008324	421	1	0.979734668196607	cation transporter activity
GO:0043169	2313	13	0.986285969840162	cation binding
GO:0005198	488	1	0.989283373172643	structural molecule activity
GO:0015075	523	1	0.992331747902732	ion transporter activity
GO:0003674	9541	86	1	molecular_function

22.4 GO: Cellular Component

GO ID	genes present in chip	genes present in tissues	p-value	GO term
GO:0005634	2704	37	0.00200298237912139	nucleus
GO:0005766	1	1	0.0090309135116371	primary lysosome
GO:0009320	1	1	0.0090309135116371	phosphoribosylaminoimidazole carboxylase complex
GO:0042582	1	1	0.0090309135116371	azurophil granule
GO:0043235	58	3	0.0153177154718659	receptor complex
GO:0051233	2	1	0.0179813059092840	spindle midzone
GO:0008305	24	2	0.019542586533047	integrin complex
GO:0043227	3955	44	0.0380316333593226	membrane-bound organelle
GO:0043231	3955	44	0.0380316333593226	intracellular membrane-bound organelle
GO:0000502	41	2	0.0526395529941625	proteasome complex (sensu Eukaryota)
GO:0005663	6	1	0.0529917462877538	DNA replication factor C complex
GO:0005764	107	3	0.072220069501132	lysosome
GO:0000323	107	3	0.072220069501132	lytic vacuole
GO:0005833	10	1	0.0867694491211822	hemoglobin complex
GO:0005680	10	1	0.0867694491211822	anaphase-promoting complex
GO:0016363	10	1	0.0867694491211822	nuclear matrix
GO:0005952	10	1	0.0867694491211822	cAMP-dependent protein kinase complex
GO:0042101	10	1	0.0867694491211822	T cell receptor complex
GO:0005941	57	2	0.0935255504432747	unlocalized protein complex
GO:0005622	5419	55	0.0939527447804501	intracellular
GO:0005773	120	3	0.0942055107316513	vacuole
GO:0001533	12	1	0.103209476193198	cornified envelope
GO:0005732	12	1	0.103209476193198	small nucleolar ribonucleoprotein complex
GO:0005694	203	4	0.110931811806718	chromosome
GO:0005769	13	1	0.111319581799800	early endosome
GO:0005876	14	1	0.119357275749198	spindle microtubule
GO:0000152	14	1	0.119357275749198	nuclear ubiquitin ligase complex
GO:0030894	14	1	0.119357275749198	replisome
GO:0005819	68	2	0.125457147627961	spindle
GO:0005657	15	1	0.127323196251525	replication fork
GO:0005626	15	1	0.127323196251525	insoluble fraction
GO:0000178	15	1	0.127323196251525	exosome (RNase complex)
GO:0043226	4515	46	0.140849704835197	organelle
GO:0043229	4515	46	0.140849704835197	intracellular organelle
GO:0000776	17	1	0.143042242045212	kinetochore
GO:0030141	18	1	0.150796616189118	secretory granule
GO:0048471	22	1	0.181127438992207	perinuclear region
GO:0000793	23	1	0.188541491198662	condensed chromosome
GO:0005793	24	1	0.195889269662383	ER-Golgi intermediate compartment
GO:0008021	27	1	0.217540785086343	synaptic vesicle
GO:0000775	27	1	0.217540785086343	chromosome, pericentric region
GO:0001772	29	1	0.231654346191212	immunological synapse
GO:0005576	949	11	0.233975900288296	extracellular region

GO:0005829	276	4	0.238502492501445	cytosol
GO:0015630	191	3	0.248168820334018	microtubule cytoskeleton
GO:0005615	385	5	0.267398772250931	extracellular space
GO:0016023	122	2	0.302024382317809	cytoplasmic membrane-bound vesicle
GO:0031410	124	2	0.308712365091036	cytoplasmic vesicle
GO:0000151	42	1	0.317459123398198	ubiquitin ligase complex
GO:0000228	42	1	0.317459123398198	nuclear chromosome
GO:0043234	1247	13	0.332832647059534	protein complex
GO:0005874	132	2	0.335331050353888	microtubule
GO:0043228	977	10	0.387526874116272	non-membrane-bound organelle
GO:0043232	977	10	0.387526874116272	intracellular non-membrane-bound organelle
GO:0005681	57	1	0.404760812064188	spliceosome complex
GO:0009986	61	1	0.426116181126840	cell surface
GO:0005768	62	1	0.431335740113793	endosome
GO:0030136	65	1	0.446714858191212	clathrin-coated vesicle
GO:0005856	612	6	0.479354382301836	cytoskeleton
GO:0005730	75	1	0.495072529861718	nucleolus
GO:0030135	79	1	0.513225358804614	coated vesicle
GO:0000785	100	1	0.598463770472316	chromatin
GO:0005887	1057	9	0.627091465148243	integral to plasma membrane
GO:0031226	1062	9	0.632944661777057	intrinsic to plasma membrane
GO:0005623	7802	70	0.660142792082322	cell
GO:0030529	254	2	0.673532504627993	ribonucleoprotein complex
GO:0005737	2530	21	0.717419478341558	cytoplasm
GO:0005886	1472	11	0.798369332160057	plasma membrane
GO:0005625	183	1	0.81325080637354	soluble fraction
GO:0005783	483	3	0.819737021423102	endoplasmic reticulum
GO:0005739	490	3	0.827314069344733	mitochondrion
GO:0005794	195	1	0.83292162796116	Golgi apparatus
GO:0008372	523	3	0.85952088370438	cellular component unknown
GO:0000267	698	4	0.88540626811026	cell fraction
GO:0005624	521	2	0.953789628826896	membrane fraction
GO:0016021	2674	13	0.99875131769183	integral to membrane
GO:0031224	2681	13	0.998810583923948	intrinsic to membrane
GO:0016020	3514	17	0.99987777077119	membrane
GO:0005575	8637	78	1	cellular_component

23. PB-BDCA4DentriticCells

23.1 PB-BDCA4DentriticCells selective genes (134 genes)

Symbol	Description	Chromosome	Chromosome Location	LocusLink	Cytoband	UniGene
GZMB	granzyme B (granzyme 2, cytotoxic T-lymphocyte-associated serine esterase 1)	14	-24170003	3002	14q11.2	Hs.1051
FCER1A	Fc fragment of IgE, high affinity I, receptor for, alpha polypeptide	1	157526127	2205	1q23	Hs.897
IGJ	immunoglobulin J polypeptide, linker protein for immunoglobulin alpha and mu polypeptides	4	-71740547	3512	4q21	Hs.643431
IGKV1-39	immunoglobulin kappa variable 1-39	2	NA	28930	2p12	NA
IGHA1	immunoglobulin heavy constant alpha 1	14	NA	3493	14q32.33	Hs.510635
P2RY14	purinergic receptor P2Y, G-protein coupled, 14	3	-152412596	9934	3q21-q25	Hs.2465
CLIC3	chloride intracellular channel 3	9	-139008880	9022	9q34.3	Hs.64746
IRF7	interferon regulatory factor 7	11	-602555	3665	11p15.5	Hs.166120
BLNK	B-cell linker	10	-97941452	29760	10q23.2-q23.33	Hs.444049
IGHV3-9	immunoglobulin heavy variable 3-9	14	NA	28451	14q32.33	NA
S100A9	S100 calcium binding protein A9 (calgranulin B)	1	151596953	6280	1q21	Hs.112405
LYZ	lysozyme (renal amyloidosis)	12	68028430	4069	12q15	Hs.524579
PLAC8	placenta-specific 8	4	-84230866	51316	4q21.22	Hs.546392
CTA-246H3.1	NA	22	24043887	91353	22q11.23	Hs.567636
RGS1	regulator of G-protein signalling 1	1	190811479	5996	1q31	Hs.75256
MNDA	myeloid cell nuclear differentiation antigen	1	157067791	4332	1q22	Hs.153837
FCN1	ficolin (collagen/fibrinogen domain containing) 1	9	-136941256	2219	9q34	Hs.440898
RABGAP1L	RAB GTPase activating protein 1-like	1	173111306, 172395256	9910	1q24	Hs.585378 Hs.591475 Hs.615081
RNASE6	ribonuclease, RNase A family, k6	14	20319049	6039	14q11.2	Hs.23262
TYROBP	TYRO protein tyrosine kinase binding protein	19	-41087145	7305	19q13.1	Hs.515369
DOCK2	dedicator of cytokinesis 2	5	168996870	1794	5q35.1	Hs.586174
ALOX5AP	arachidonate 5-lipoxygenase-activating protein	13	30207668	241	13q12	Hs.507658
DCK	deoxycytidine kinase	4	72078255	1633	4q13.3-q21.1	Hs.709
TLR7	toll-like receptor 7	X	12795122	51284	Xp22.3	Hs.443036
LILRA4	leukocyte immunoglobulin-like receptor, subfamily A (with TM domain), member 4	19	-59536504	23547	19q13.4	Hs.406708
EVI2B	ecotropic viral integration site 2B	17	-26654918	2124	17q11.2	Hs.5509
TCF4	transcription factor 4	18	-51045966	6925	18q21.1	Hs.569908
TNFRSF17	tumor necrosis factor receptor superfamily, member 17	16	11966464	608	16p13.1	Hs.2556
C13orf18	chromosome 13 open reading frame 18	13	-45815100	80183	13q14.12	Hs.98117
POU2AF1	POU domain, class 2, associating factor 1	11	-110728189	5450	11q23.1	Hs.128180
S100A8	S100 calcium binding protein A8 (calgranulin A)	1	-151629132	6279	1q21	Hs.416073
IRF8	interferon regulatory factor 8	16	84490274	3394	16q24.1	Hs.137427
SCT	secretin	11	-616312	6343	11p15.5	Hs.632324

PYCARD	PYD and CARD domain containing	16	-31120309	29108	16p12-p11.2	Hs.499094
FGL2	fibrinogen-like 2	7	-76660625	10875	7q11.23	Hs.520989
PTPRC	protein tyrosine phosphatase, receptor type, C	1	196874847	5788	1q31-q32	Hs.192039
UBE2J1	ubiquitin-conjugating enzyme E2, J1 (UBC6 homolog, yeast)	6	-90093063	51465	6q15	Hs.163776
IGHM	immunoglobulin heavy constant mu	14	NA	3507	14q32.33	Hs.510635
TCL1A	T-cell leukemia/lymphoma 1A	14	-95246057	8115	14q32.1	Hs.2484
VAMP8	vesicle-associated membrane protein 8 (endobrevin)	2	85658227	8673	2p12-p11.2	Hs.534373
ITGA4	integrin, alpha 4 (antigen CD49D, alpha 4 subunit of VLA-4 receptor)	2	182029863	3676	2q31.3	Hs.440955
OPN3	opsin 3 (encephalopsin, panopsin)	1	-239823075	23596	1q43	Hs.409081 Hs.534399
COTL1	coactosin-like 1 (Dictyostelium)	16	-83156706	23406	16q24.1	Hs.289092
CD302	CD302 molecule	2	-160333609	9936	2q24.2	Hs.130014
MCM5	MCM5 minichromosome maintenance deficient 5, cell division cycle 46 (S. cerevisiae)	22	34126127	4174	22q13.1	Hs.517582
PSCDBP	pleckstrin homology, Sec7 and coiled-coil domains, binding protein	2	-157979377	9595	2q11.2	Hs.270
DUSP5	dual specificity phosphatase 5	10	112247614	1847	10q25	Hs.2128
PTPRE	protein tyrosine phosphatase, receptor type, E	10	129595314, 129735823	5791	10q26	Hs.127022
IGHV3-23	immunoglobulin heavy variable 3-23	14	NA	28442	14q32.33	NA
CD36	CD36 molecule (thrombospondin receptor)	7	80105892, 80069458	948	7q11.2	Hs.120949 Hs.633085
PMAIP1	phorbol-12-myristate-13-acetate-induced protein 1	18	55718216	5366	18q21.32	Hs.96
CSTA	cystatin A (stefin A)	3	123526700	1475	3q21	Hs.518198
CD2AP	CD2-associated protein	6	47553898	23607	6p12	Hs.485518
CD37	CD37 molecule	19	54530488	951	19q13.3	Hs.166556
LTB	lymphotoxin beta (TNF superfamily, member 3)	6	-37446700	4050	6p21.3	Hs.376208
PPBP	pro-platelet basic protein (chemokine (C-X-C motif) ligand 7)	4	-75071621	5473	4q12-q13	Hs.2164
DPCR1	diffuse panbronchiolitis critical region 1	6	31027537, 2367969, 2166477	135656	6p21.33	Hs.631993
HLA-DRA	major histocompatibility complex, class II, DR alpha	6	32515624	3122	6p21.3	Hs.520048
CPVL	carboxypeptidase, vitellogenic-like	7	-29001773	54504	7p15-p14	Hs.233389
SELL	selectin L (lymphocyte adhesion molecule 1)	1	-167926433	6402	1q23-q25	Hs.82848
LYN	v-yes-1 Yamaguchi sarcoma viral related oncogene homolog	8	56954925	4067	8q13	Hs.491767 Hs.614775
PTPN6	protein tyrosine phosphatase, non-receptor type 6	12	6930703, 6926000	5777	12p13	Hs.63489
HCK	hemopoietic cell kinase	20	30103717	3055	20q11-q12	Hs.126521
NCF2	neutrophil cytosolic factor 2 (65kDa, chronic granulomatous disease, autosomal 2)	1	-181791320	4688	1q25	Hs.587558
ISG20	interferon stimulated exonuclease gene 20kDa	15	86983042	3669	15q26	Hs.459265
CSPG2	chondroitin sulfate proteoglycan 2 (versican)	5	82803338	1462	5q14.3	Hs.443681
CXCR4	chemokine (C-X-C motif) receptor 4	2	-136588389	7852	2q21	Hs.421986
HHEX	homeobox, hematopoietically expressed	10	94439679	3087	10q23.33	Hs.118651
NUCB2	nucleobindin 2	11	17254887	4925	11p15.1-p14	Hs.128686
CTSS	cathepsin S	1	-148969177	1520	1q21	Hs.181301
ADCY7	adenylate cyclase 7	16	48879322	113	16q12-q13	Hs.513578
LY96	lymphocyte antigen 96	8	75066143	23643	8q21.11	Hs.69328

GLIPR1	GLI pathogenesis-related 1 (glioma)	12	74160779	11010	12q21.2	Hs.205558 Hs.591034
SYK	spleen tyrosine kinase	9	92603890	6850	9q22	Hs.371720
TRIM22	tripartite motif-containing 22	11	5667663	10346	11p15	Hs.501778 Hs.621411
CYBB	cytochrome b-245, beta polypeptide (chronic granulomatous disease)	X	37524263	1536	Xp21.1	Hs.292356
GMFG	glia maturation factor, gamma	19	-44510841	9535	19q13.2	Hs.5210
BTAF1	BTAF1 RNA polymerase II, B-TFIID transcription factor-associated, 170kDa (Mot1 homolog, S. cerevisiae)	10	93673715	9044	10q22-q23	Hs.500526
CD1D	CD1d molecule	1	156416360	912	1q22-q23	Hs.1799 Hs.387539
HLA-DMA	major histocompatibility complex, class II, DM alpha	6	-41424089	3108	6p21.3	Hs.351279
SCAP2	src family associated phosphoprotein 2	7	-26673214	8935	7p21-p15	Hs.200770
IGSF6	immunoglobulin superfamily, member 6	16	-21560106	10261	16p12-p13	Hs.530902
PSMB10	proteasome (prosome, macropain) subunit, beta type, 10	16	-66525907	5699	16q22.1	Hs.9661
UGCG	UDP-glucose ceramide glucosyltransferase	9	113699026	7357	9q31	Hs.304249 Hs.593014
CBFA2T3	core-binding factor, runt domain, alpha subunit 2, translocated to, 3	16	-87468767	863	16q24	Hs.513811
RNF138	ring finger protein 138	18	27925834, 27926644	51444	18q12.1	Hs.302408 Hs.501040
GNA15	guanine nucleotide binding protein (G protein), alpha 15 (Gq class)	19	3087229	2769	19p13.3	Hs.73797
PRG1	proteoglycan 1, secretory granule	10	70517833	5552	10q22.1	Hs.1908
RASGRP2	RAS guanyl releasing protein 2 (calcium and DAG-regulated)	11	-64250959	10235	11q13	Hs.99491
RGS2	regulator of G-protein signalling 2, 24kDa	1	191044793	5997	1q31	Hs.78944
ZNF651	zinc finger protein 651	3	42675877	92999	3p22.1	Hs.409561
CASP1	caspase 1, apoptosis-related cysteine peptidase (interleukin 1, beta, convertase)	11	-104401451	834	11q23	Hs.2490
SIDT1	SID1 transmembrane family, member 1	3	114734182	54847	3q13.2	Hs.591291
KLF4	Kruppel-like factor 4 (gut)	9	-109286955	9314	9q31	Hs.376206
LGALS2	lectin, galactoside-binding, soluble, 2 (galectin 2)	22	-36296199	3957	22q12-q13|22q13.1	Hs.531776
C1orf38	chromosome 1 open reading frame 38	1	28071641	9473	1p35.3	Hs.10649
CD14	CD14 molecule	5	-139991500	929	5q22-q32|5q31.1	Hs.163867
MICAL1	microtubule associated monoxygenase, calponin and LIM domain containing 1	6	-109871962	64780	6q21	Hs.33476
FOSB	FBJ murine osteosarcoma viral oncogene homolog B	19	50663092	2354	19q13.32	Hs.590958
TNFRSF1B	tumor necrosis factor receptor superfamily, member 1B	1	12149646	7133	1p36.3-p36.2	Hs.256278
TNFAIP8	tumor necrosis factor, alpha-induced protein 8	5	118719628	25816	5q23.1	Hs.271955
NCF4	neutrophil cytosolic factor 4, 40kDa	22	35586990	4689	22q13.1	Hs.474781
IGKV4-1	immunoglobulin kappa variable 4-1	2	NA	28908	2p12	NA
MAN2B1	mannosidase, alpha, class 2B, member 1	19	-12618321	4125	19cen-q13.1	Hs.334626 Hs.356769
IGKC	immunoglobulin kappa constant	2	NA	3514	2p12	Hs.449621
LRRFIP1	leucine rich repeat (in FLII) interacting protein 1	2	238265667	9208	2q37.3	Hs.471779
KRT5	keratin 5 (epidermolysis bullosa simplex, Dowling-Meara/Kobner/Weber-Cockayne types)	12	-51194627	3852	12q12-q13	Hs.433845
KIAA0746	NA	4	-25358146	23231	4p15.2	Hs.479384
RBM3	RNA binding motif (RNP1, RRM) protein 3	X	48317779	5935	Xp11.2	Hs.301404

IGLV4-60	immunoglobulin lambda variable 4-60	22	NA	28785	22q11.2	NA
HCLS1	hematopoietic cell-specific Lyn substrate 1	3	-122832936	3059	3q13	Hs.14601
HMGB2	high-mobility group box 2	4	-174489362	3148	4q31	Hs.434953
ST6GALNAC4	ST6 (alpha-N-acetyl-neuraminyl-2,3-beta-galactosyl-1,3)-N-acetylgalactosaminide alpha-2,6-sialyltransferase 4	9	-129709986	27090	9q34	Hs.3972
TGFBI	transforming growth factor, beta-induced, 68kDa	5	135392596	7045	5q31	Hs.369397
BANK1	B-cell scaffold protein with ankyrin repeats 1	4	102930918	55024	4q24	Hs.480400
CAPG	capping protein (actin filament), gelsolin-like	2	-85475381	822	2p11.2	Hs.516155
ITGAM	integrin, alpha M (complement component 3 receptor 3 subunit)	16	31178788	3684	16p11.2	Hs.172631
CUTL2	cut-like 2 (Drosophila)	12	109956211	23316	12q24.11-q24.12	Hs.124953
CLEC4A	C-type lectin domain family 4, member A	12	8167494	50856	12p13	Hs.504657
FCHSD2	FCH and double SH3 domains 2	11	-72225438	9873	11q13.4	Hs.577053
HLA-DPA1	major histocompatibility complex, class II, DP alpha 1	6	-41773262	3113	6p21.3	Hs.347270
RAB11FIP1	RAB11 family interacting protein 1 (class I)	8	-75672971	80223	8p11.22	Hs.191179
CORO1A	coronin, actin binding protein, 1A	16	30102426	11151	16p11.2	Hs.415067
ARL4C	ADP-ribosylation factor-like 4C	2	-235066426	10123	2q37.1	Hs.111554
PLEKHF2	pleckstrin homology domain containing, family F (with FYVE domain) member 2	8	96215213	79666	8q22.1	Hs.29724
C6orf111	chromosome 6 open reading frame 111	6	-99954923	25957	6q16.3	Hs.591345
GCH1	GTP cyclohydrolase 1 (dopa-responsive dystonia)	14	-54378475	2643	14q22.1-q22.2	Hs.86724
IFI16	interferon, gamma-inducible protein 16	1	157246330	3428	1q22	Hs.380250
PSMD14	proteasome (prosome, macropain) 26S subunit, non-ATPase, 14	2	161873185	10213	2q24.2	Hs.567410
IL8	interleukin 8	4	74825138	3576	4q13-q21	Hs.624
S100A12	S100 calcium binding protein A12 (calgranulin C)	1	-151612807	6283	1q21	Hs.19413
LOC646082	NA	22	NA	646082	22q11.23	NA
EVI2A	ecotropic viral integration site 2A	17	-26668801	2123	17q11.2	Hs.591198
TFEC	transcription factor EC	7	-115362437	22797	7q31.2	Hs.125962

23.2 GO: Biological Process

GO ID	genes present in chip	genes present in tissues	p-value	GO term
GO:0006952	751	46	1.88E-22	defense response
GO:0006955	684	44	3.22E-22	immune response
GO:0009607	857	48	7.23E-22	response to biotic stimulus
GO:0050896	1578	54	4.47E-15	response to stimulus
GO:0043207	463	28	3.48E-13	response to external biotic stimulus
GO:0009613	458	27	1.85E-12	response to pest, pathogen or parasite
GO:0050874	1531	49	2.74E-12	organismal physiological process
GO:0019882	41	8	2.06E-08	antigen presentation
GO:0009605	963	31	1.22E-07	response to external stimulus
GO:0030333	36	7	1.69E-07	antigen processing
GO:0006950	881	29	2.22E-07	response to stress
GO:0006954	185	12	1.74E-06	inflammatory response
GO:0009611	341	16	2.28E-06	response to wounding
GO:0019883	21	5	3.72E-06	antigen presentation, endogenous antigen
GO:0019885	22	4	0.000115554	antigen processing, endogenous antigen via MHC class I
GO:0019884	11	3	0.000248922	antigen presentation, exogenous antigen
GO:0009617	51	5	0.000323592	response to bacteria
GO:0019886	12	3	0.00032904	antigen processing, exogenous antigen via MHC class II
GO:0042531	3	2	0.000413915	positive regulation of tyrosine phosphorylation of STAT protein
GO:0046427	3	2	0.000413915	positive regulation of JAK-STAT cascade
GO:0030595	13	3	0.000424073	immune cell chemotaxis
GO:0050900	14	3	0.00053509	immune cell migration
GO:0030097	87	6	0.000557455	hemopoiesis
GO:0048534	88	6	0.000592564	hemopoietic or lymphoid organ development
GO:0042742	34	4	0.000656778	defense response to bacteria
GO:0030099	37	4	0.000910192	myeloid cell differentiation
GO:0030593	5	2	0.001358438	neutrophil chemotaxis
GO:0006959	141	7	0.001384733	humoral immune response
GO:0019735	77	5	0.002134248	antimicrobial humoral response (sensu Vertebrata)
GO:0019730	78	5	0.002259266	antimicrobial humoral response
GO:0042509	7	2	0.0028088	regulation of tyrosine phosphorylation of STAT protein
GO:0046425	7	2	0.0028088	regulation of JAK-STAT cascade
GO:0018298	7	2	0.0028088	protein-chromophore linkage
GO:0006968	87	5	0.003636376	cellular defense response
GO:0050778	56	4	0.004262227	positive regulation of immune response
GO:0050731	9	2	0.004741089	positive regulation of peptidyl-tyrosine phosphorylation
GO:0009615	58	4	0.004835467	response to virus
GO:0051240	59	4	0.005140603	positive regulation of organismal physiological process
GO:0001934	10	2	0.00588071	positive regulation of protein amino acid phosphorylation
GO:0045764	10	2	0.00588071	positive regulation of amino acid metabolism
GO:0007260	10	2	0.00588071	tyrosine phosphorylation of STAT protein
GO:0030155	33	3	0.006812944	regulation of cell adhesion
GO:0016064	103	5	0.007425909	humoral defense mechanism (sensu Vertebrata)

GO:0042327	12	2	0.008492854	positive regulation of phosphorylation
GO:0045937	12	2	0.008492854	positive regulation of phosphate metabolism
GO:0016481	151	6	0.008952307	negative regulation of transcription
GO:0008283	473	12	0.009926698	cell proliferation
GO:0030218	13	2	0.009959904	erythrocyte differentiation
GO:0045934	159	6	0.01137373	negative regulation of nucleobase, nucleoside, nucleotide and nucleic acid metabolism
GO:0019835	14	2	0.011530707	cytolysis
GO:0009725	14	2	0.011530707	response to hormone stimulus
GO:0048003	1	1	0.011846767	antigen presentation, lipid antigen
GO:0048006	1	1	0.011846767	antigen presentation, endogenous lipid antigen
GO:0031342	1	1	0.011846767	negative regulation of cell killing
GO:0001911	1	1	0.011846767	negative regulation of immune cell mediated cytotoxicity
GO:0001913	1	1	0.011846767	T cell mediated cytotoxicity
GO:0001914	1	1	0.011846767	regulation of T cell mediated cytotoxicity
GO:0001915	1	1	0.011846767	negative regulation of T cell mediated cytotoxicity
GO:0050853	1	1	0.011846767	B cell receptor signaling pathway
GO:0051103	1	1	0.011846767	DNA ligation during DNA repair
GO:0000738	1	1	0.011846767	DNA catabolism, exonucleolytic
GO:0044416	1	1	0.011846767	induction of host defense response
GO:0006288	1	1	0.011846767	base-excision repair, DNA ligation
GO:0046730	1	1	0.011846767	viral induction of host immune response
GO:0046731	1	1	0.011846767	passive viral induction of host immune response
GO:0051247	41	3	0.01243355	positive regulation of protein metabolism
GO:0050776	77	4	0.013003546	regulation of immune response
GO:0006909	15	2	0.013202644	phagocytosis
GO:0050730	15	2	0.013202644	regulation of peptidyl-tyrosine phosphorylation
GO:0009892	217	7	0.014410198	negative regulation of metabolism
GO:0001932	16	2	0.014973145	regulation of protein amino acid phosphorylation
GO:0009595	16	2	0.014973145	detection of biotic stimulus
GO:0007229	45	3	0.015997728	integrin-mediated signaling pathway
GO:0006521	17	2	0.016839686	regulation of amino acid metabolism
GO:0016477	85	4	0.018113433	cell migration
GO:0009967	85	4	0.018113433	positive regulation of signal transduction
GO:0009966	229	7	0.018829995	regulation of signal transduction
GO:0006664	19	2	0.020851012	glycolipid metabolism
GO:0018108	19	2	0.020851012	peptidyl-tyrosine phosphorylation
GO:0031324	183	6	0.021364523	negative regulation of cellular metabolism
GO:0046513	2	1	0.023554484	ceramide biosynthesis
GO:0006517	2	1	0.023554484	protein deglycosylation
GO:0031341	2	1	0.023554484	regulation of cell killing
GO:0006678	2	1	0.023554484	glucosylceramide metabolism
GO:0006679	2	1	0.023554484	glucosylceramide biosynthesis
GO:0006922	2	1	0.023554484	cleavage of lamin
GO:0006923	2	1	0.023554484	cleavage of cytoskeletal proteins
GO:0001910	2	1	0.023554484	regulation of immune cell mediated cytotoxicity
GO:0048247	2	1	0.023554484	lymphocyte chemotaxis
GO:0008228	2	1	0.023554484	opsonization

GO:0019733	2	1	0.023554484	antibacterial humoral response (sensu Vertebrata)
GO:0007207	2	1	0.023554484	muscarinic acetylcholine receptor, phospholipase C activating pathway
GO:0042752	2	1	0.023554484	regulation of circadian rhythm
GO:0046476	2	1	0.023554484	glycosylceramide biosynthesis
GO:0045091	2	1	0.023554484	regulation of retroviral genome replication
GO:0007165	2168	35	0.025067867	signal transduction
GO:0018212	21	2	0.025217313	peptidyl-tyrosine modification
GO:0006461	194	6	0.027484451	protein complex assembly
GO:0045321	97	4	0.027854953	immune cell activation
GO:0045892	97	4	0.027854953	negative regulation of transcription, DNA-dependent
GO:0007154	2730	42	0.028190393	cell communication
GO:0001775	98	4	0.028783501	cell activation
GO:0006470	99	4	0.029730328	protein amino acid dephosphorylation
GO:0007166	952	18	0.030136609	cell surface receptor linked signal transduction
GO:0006935	101	4	0.031679018	chemotaxis
GO:0042330	101	4	0.031679018	taxis
GO:0048519	688	14	0.031954866	negative regulation of biological process
GO:0006928	205	6	0.034671473	cell motility
GO:0040011	205	6	0.034671473	locomotion
GO:0042325	25	2	0.034941017	regulation of phosphorylation
GO:0048002	3	1	0.035124769	antigen presentation, peptide antigen
GO:0048004	3	1	0.035124769	antigen presentation, endogenous peptide antigen
GO:0046520	3	1	0.035124769	sphingoid biosynthesis
GO:0006677	3	1	0.035124769	glycosylceramide metabolism
GO:0045350	3	1	0.035124769	interferon-beta biosynthesis
GO:0045356	3	1	0.035124769	positive regulation of interferon-alpha biosynthesis
GO:0045357	3	1	0.035124769	regulation of interferon-beta biosynthesis
GO:0045359	3	1	0.035124769	positive regulation of interferon-beta biosynthesis
GO:0006930	3	1	0.035124769	substrate-bound cell migration, cell extension
GO:0019731	3	1	0.035124769	antibacterial humoral response
GO:0050857	3	1	0.035124769	positive regulation of antigen receptor mediated signaling pathway
GO:0042119	3	1	0.035124769	neutrophil activation
GO:0042771	3	1	0.035124769	DNA damage response, signal transduction by p53 class mediator resulting in induction of apoptosis
GO:0007500	3	1	0.035124769	mesodermal cell fate determination
GO:0030157	3	1	0.035124769	pancreatic juice secretion
GO:0006266	3	1	0.035124769	DNA ligation
GO:0016998	3	1	0.035124769	cell wall catabolism
GO:0030851	3	1	0.035124769	granulocyte differentiation
GO:0045090	3	1	0.035124769	retroviral genome replication
GO:0000122	61	3	0.035444213	negative regulation of transcription from RNA polymerase II promoter
GO:0007626	209	6	0.037560527	locomotory behavior
GO:0007498	26	2	0.037565506	mesoderm development
GO:0044419	26	2	0.037565506	interaction between organisms
GO:0048523	640	13	0.038704013	negative regulation of cellular process
GO:0007610	269	7	0.040301615	behavior
GO:0016311	110	4	0.041363191	dephosphorylation
GO:0019220	28	2	0.04303189	regulation of phosphate metabolism

GO:0007259	28	2	0.04303189	JAK-STAT cascade
GO:0051174	28	2	0.04303189	regulation of phosphorus metabolism
GO:0001819	29	2	0.045869689	positive regulation of cytokine production
GO:0019048	4	1	0.046559219	virus-host interaction
GO:0045351	4	1	0.046559219	interferon type I biosynthesis
GO:0045354	4	1	0.046559219	regulation of interferon-alpha biosynthesis
GO:0045349	4	1	0.046559219	interferon-alpha biosynthesis
GO:0048333	4	1	0.046559219	mesodermal cell differentiation
GO:0045414	4	1	0.046559219	regulation of interleukin-8 biosynthesis
GO:0045416	4	1	0.046559219	positive regulation of interleukin-8 biosynthesis
GO:0050854	4	1	0.046559219	regulation of antigen receptor mediated signaling pathway
GO:0051282	4	1	0.046559219	regulation of sequestering of calcium ion
GO:0051283	4	1	0.046559219	negative regulation of sequestering of calcium ion
GO:0051209	4	1	0.046559219	release of sequestered calcium ion into cytoplasm
GO:0042541	4	1	0.046559219	hemoglobin biosynthesis
GO:0001710	4	1	0.046559219	mesodermal cell fate commitment
GO:0045089	4	1	0.046559219	positive regulation of innate immune response
GO:0042113	30	2	0.04877456	B cell activation
GO:0019932	176	5	0.057695026	second-messenger-mediated signaling
GO:0050716	5	1	0.057859415	positive regulation of interleukin-1 secretion
GO:0050718	5	1	0.057859415	positive regulation of interleukin-1 beta secretion
GO:0050701	5	1	0.057859415	interleukin-1 secretion
GO:0050702	5	1	0.057859415	interleukin-1 beta secretion
GO:0050704	5	1	0.057859415	regulation of interleukin-1 secretion
GO:0050706	5	1	0.057859415	regulation of interleukin-1 beta secretion
GO:0050852	5	1	0.057859415	T cell receptor signaling pathway
GO:0042228	5	1	0.057859415	interleukin-8 biosynthesis
GO:0050926	5	1	0.057859415	regulation of positive chemotaxis
GO:0050927	5	1	0.057859415	positive regulation of positive chemotaxis
GO:0050918	5	1	0.057859415	positive chemotaxis
GO:0050930	5	1	0.057859415	induction of positive chemotaxis
GO:0051208	5	1	0.057859415	sequestering of calcium ion
GO:0046146	5	1	0.057859415	tetrahydrobiopterin metabolism
GO:0020027	5	1	0.057859415	hemoglobin metabolism
GO:0006729	5	1	0.057859415	tetrahydrobiopterin biosynthesis
GO:0045069	5	1	0.057859415	regulation of viral genome replication
GO:0045088	5	1	0.057859415	regulation of innate immune response
GO:0006401	33	2	0.057872237	RNA catabolism
GO:0030098	34	2	0.061026208	lymphocyte differentiation
GO:0016032	35	2	0.064237834	viral life cycle
GO:0006357	240	6	0.065172184	regulation of transcription from RNA polymerase II promoter
GO:0042221	183	5	0.065951693	response to chemical substance
GO:0006929	6	1	0.069026917	substrate-bound cell migration
GO:0001909	6	1	0.069026917	immune cell mediated cytotoxicity
GO:0050851	6	1	0.069026917	antigen receptor-mediated signaling pathway
GO:0050921	6	1	0.069026917	positive regulation of chemotaxis
GO:0045058	6	1	0.069026917	T cell selection

GO:0008219	498	10	0.070271304	cell death
GO:0007204	37	2	0.070826967	elevation of cytoplasmic calcium ion concentration
GO:0051480	37	2	0.070826967	cytoplasmic calcium ion homeostasis
GO:0046649	81	3	0.071089361	lymphocyte activation
GO:0016265	501	10	0.072526591	death
GO:0051239	133	4	0.072910679	regulation of organismal physiological process
GO:0001817	39	2	0.077625799	regulation of cytokine production
GO:0048332	7	1	0.080063269	mesoderm morphogenesis
GO:0016045	7	1	0.080063269	detection of bacteria
GO:0050792	7	1	0.080063269	regulation of viral life cycle
GO:0050715	7	1	0.080063269	positive regulation of cytokine secretion
GO:0050707	7	1	0.080063269	regulation of cytokine secretion
GO:0050663	7	1	0.080063269	cytokine secretion
GO:0050920	7	1	0.080063269	regulation of chemotaxis
GO:0001704	7	1	0.080063269	formation of primary germ layer
GO:0001707	7	1	0.080063269	mesoderm formation
GO:0007155	518	10	0.086173772	cell adhesion
GO:0001816	42	2	0.088188231	cytokine production
GO:0006558	8	1	0.090969999	L-phenylalanine metabolism
GO:0006559	8	1	0.090969999	L-phenylalanine catabolism
GO:0006688	8	1	0.090969999	glycosphingolipid biosynthesis
GO:0001906	8	1	0.090969999	cell killing
GO:0007369	8	1	0.090969999	gastrulation
GO:0050832	8	1	0.090969999	defense response to fungi
GO:0009596	8	1	0.090969999	detection of pest, pathogen or parasite
GO:0006013	8	1	0.090969999	mannose metabolism
GO:0051238	8	1	0.090969999	sequestering of metal ion
GO:0045078	8	1	0.090969999	positive regulation of interferon-gamma biosynthesis
GO:0007242	879	15	0.094310744	intracellular signaling cascade
GO:0050714	9	1	0.101748612	positive regulation of protein secretion
GO:0007159	9	1	0.101748612	leukocyte adhesion
GO:0009247	9	1	0.101748612	glycolipid biosynthesis
GO:0042100	9	1	0.101748612	B cell proliferation
GO:0030330	9	1	0.101748612	DNA damage response, signal transduction by p53 class mediator
GO:0006268	9	1	0.101748612	DNA unwinding
GO:0006915	471	9	0.105528472	apoptosis
GO:0012501	473	9	0.107591406	programmed cell death
GO:0009719	214	5	0.109957951	response to endogenous stimulus
GO:0030154	409	8	0.11153309	cell differentiation
GO:0006516	10	1	0.112400601	glycoprotein catabolism
GO:0019438	10	1	0.112400601	aromatic compound biosynthesis
GO:0009074	10	1	0.112400601	aromatic amino acid family catabolism
GO:0008630	10	1	0.112400601	DNA damage response, signal transduction resulting in induction of apoptosis
GO:0042559	10	1	0.112400601	pteridine and derivative biosynthesis
GO:0009888	158	4	0.117679462	histogenesis
GO:0009968	50	2	0.118197099	negative regulation of signal transduction
GO:0018193	50	2	0.118197099	peptidyl-amino acid modification

GO:0006921	11	1	0.12292744	disassembly of cell structures during apoptosis
GO:0007193	11	1	0.12292744	G-protein signaling, adenylate cyclase inhibiting pathway
GO:0042558	11	1	0.12292744	pteridine and derivative metabolism
GO:0009620	11	1	0.12292744	response to fungi
GO:0030148	11	1	0.12292744	sphingolipid biosynthesis
GO:0006171	11	1	0.12292744	cAMP biosynthesis
GO:0045072	11	1	0.12292744	regulation of interferon-gamma biosynthesis
GO:0044248	700	12	0.124064533	cellular catabolism
GO:0007186	491	9	0.127184156	G-protein coupled receptor protein signaling pathway
GO:0044265	563	10	0.129434238	cellular macromolecule catabolism
GO:0008632	53	2	0.130031394	apoptotic program
GO:0006672	12	1	0.133330584	ceramide metabolism
GO:0019370	12	1	0.133330584	leukotriene biosynthesis
GO:0042095	12	1	0.133330584	interferon-gamma biosynthesis
GO:0050708	12	1	0.133330584	regulation of protein secretion
GO:0042102	12	1	0.133330584	positive regulation of T cell proliferation
GO:0001709	12	1	0.133330584	cell fate determination
GO:0043085	107	3	0.133490564	positive regulation of enzyme activity
GO:0006464	1087	17	0.140250653	protein modification
GO:0009057	575	10	0.14268541	macromolecule catabolism
GO:0008037	13	1	0.143611474	cell recognition
GO:0019439	13	1	0.143611474	aromatic compound catabolism
GO:0051047	13	1	0.143611474	positive regulation of secretion
GO:0046058	13	1	0.143611474	cAMP metabolism
GO:0009100	111	3	0.144392017	glycoprotein metabolism
GO:0044267	2109	30	0.149835193	cellular protein metabolism
GO:0051246	174	4	0.151333099	regulation of protein metabolism
GO:0051243	583	10	0.151905125	negative regulation of cellular physiological process
GO:0007582	7846	97	0.152492527	physiological process
GO:0046519	14	1	0.153771533	sphingoid metabolism
GO:0006691	14	1	0.153771533	leukotriene metabolism
GO:0007623	14	1	0.153771533	circadian rhythm
GO:0007602	14	1	0.153771533	phototransduction
GO:0006308	14	1	0.153771533	DNA catabolism
GO:0030224	14	1	0.153771533	monocyte differentiation
GO:0007188	59	2	0.154452762	G-protein signaling, coupled to cAMP nucleotide second messenger
GO:0019933	60	2	0.158606796	cAMP-mediated signaling
GO:0019538	2129	30	0.163162163	protein metabolism
GO:0006801	15	1	0.163812168	superoxide metabolism
GO:0006687	15	1	0.163812168	glycosphingolipid metabolism
GO:0045165	15	1	0.163812168	cell fate commitment
GO:0007162	15	1	0.163812168	negative regulation of cell adhesion
GO:0042129	15	1	0.163812168	regulation of T cell proliferation
GO:0009583	15	1	0.163812168	detection of light
GO:0018149	15	1	0.163812168	peptide cross-linking
GO:0006508	451	8	0.165263901	proteolysis and peptidolysis
GO:0044257	451	8	0.165263901	cellular protein catabolism

GO:0008284	121	3	0.172845612	positive regulation of cell proliferation
GO:0006809	16	1	0.17373477	nitric oxide biosynthesis
GO:0007157	16	1	0.17373477	heterophilic cell adhesion
GO:0007202	16	1	0.17373477	phospholipase C activation
GO:0006284	16	1	0.17373477	base-excision repair
GO:0046209	16	1	0.17373477	nitric oxide metabolism
GO:0043118	602	10	0.174992387	negative regulation of physiological process
GO:0009628	318	6	0.175250674	response to abiotic stimulus
GO:0006261	65	2	0.179670515	DNA-dependent DNA replication
GO:0030163	463	8	0.182533646	protein catabolism
GO:0009056	756	12	0.18275425	catabolism
GO:0015758	17	1	0.183540712	glucose transport
GO:0006904	17	1	0.183540712	vesicle docking during exocytosis
GO:0042098	17	1	0.183540712	T cell proliferation
GO:0019722	17	1	0.183540712	calcium-mediated signaling
GO:0006270	17	1	0.183540712	DNA replication initiation
GO:0043170	2405	33	0.187861731	macromolecule metabolism
GO:0006874	68	2	0.19250716	calcium ion homeostasis
GO:0015749	18	1	0.193231355	monosaccharide transport
GO:0019079	18	1	0.193231355	viral genome replication
GO:0031281	18	1	0.193231355	positive regulation of cyclase activity
GO:0048278	18	1	0.193231355	vesicle docking
GO:0045762	18	1	0.193231355	positive regulation of adenylate cyclase activity
GO:0009072	18	1	0.193231355	aromatic amino acid family metabolism
GO:0008645	18	1	0.193231355	hexose transport
GO:0009416	18	1	0.193231355	response to light
GO:0007190	18	1	0.193231355	adenylate cyclase activation
GO:0042133	18	1	0.193231355	neurotransmitter metabolism
GO:0050671	18	1	0.193231355	positive regulation of lymphocyte proliferation
GO:0051349	18	1	0.193231355	positive regulation of lyase activity
GO:0030216	18	1	0.193231355	keratinocyte differentiation
GO:0030217	18	1	0.193231355	T cell differentiation
GO:0043285	474	8	0.199038006	biopolymer catabolism
GO:0008544	70	2	0.201131681	epidermis development
GO:0042127	265	5	0.205459814	regulation of cell proliferation
GO:0048015	71	2	0.205461268	phosphoinositide-mediated signaling
GO:0043412	1160	17	0.207614256	biopolymer modification
GO:0008629	20	1	0.212272097	induction of apoptosis by intracellular signals
GO:0009190	20	1	0.212272097	cyclic nucleotide biosynthesis
GO:0044403	20	1	0.212272097	symbiosis, mutualism through parasitism
GO:0044404	20	1	0.212272097	symbiotic interaction between host and other organism
GO:0006725	73	2	0.214150782	aromatic compound metabolism
GO:0031325	136	3	0.218123869	positive regulation of cellular metabolism
GO:0019221	21	1	0.221624836	cytokine and chemokine mediated signaling pathway
GO:0051016	21	1	0.221624836	barbed-end actin filament capping
GO:0050870	21	1	0.221624836	positive regulation of T cell activation
GO:0048518	565	9	0.225525403	positive regulation of biological process

GO:0006917	140	3	0.230603218	induction of apoptosis
GO:0012502	140	3	0.230603218	induction of programmed cell death
GO:0007189	22	1	0.230867555	G-protein signaling, adenylate cyclase activating pathway
GO:0050670	22	1	0.230867555	regulation of lymphocyte proliferation
GO:0030042	22	1	0.230867555	actin filament depolymerization
GO:0042770	22	1	0.230867555	DNA damage response, signal transduction
GO:0006471	22	1	0.230867555	protein amino acid ADP-ribosylation
GO:0030834	22	1	0.230867555	regulation of actin filament depolymerization
GO:0030835	22	1	0.230867555	negative regulation of actin filament depolymerization
GO:0006805	23	1	0.240001537	xenobiotic metabolism
GO:0008277	23	1	0.240001537	regulation of G-protein coupled receptor protein signaling pathway
GO:0042108	23	1	0.240001537	positive regulation of cytokine biosynthesis
GO:0046456	23	1	0.240001537	icosanoid biosynthesis
GO:0006366	426	7	0.240314265	transcription from RNA polymerase II promoter
GO:0007398	79	2	0.240403548	ectoderm development
GO:0043119	428	7	0.243888451	positive regulation of physiological process
GO:0042088	24	1	0.249028047	T-helper 1 type immune response
GO:0051046	24	1	0.249028047	regulation of secretion
GO:0043283	2240	30	0.249083551	biopolymer metabolism
GO:0045727	25	1	0.25794834	positive regulation of protein biosynthesis
GO:0042087	25	1	0.25794834	cell-mediated immune response
GO:0051235	25	1	0.25794834	maintenance of localization
GO:0009893	149	3	0.259133965	positive regulation of metabolism
GO:0019058	26	1	0.266763654	viral infectious cycle
GO:0031328	26	1	0.266763654	positive regulation of cellular biosynthesis
GO:0008643	26	1	0.266763654	carbohydrate transport
GO:0009306	26	1	0.266763654	protein secretion
GO:0009410	26	1	0.266763654	response to xenobiotic stimulus
GO:0006220	26	1	0.266763654	pyrimidine nucleotide metabolism
GO:0006919	27	1	0.275475212	caspase activation
GO:0016066	27	1	0.275475212	cellular defense response (sensu Vertebrata)
GO:0009187	27	1	0.275475212	cyclic nucleotide metabolism
GO:0009792	27	1	0.275475212	embryonic development (sensu Metazoa)
GO:0030005	87	2	0.275615992	di-, tri-valent inorganic cation homeostasis
GO:0043065	155	3	0.278417953	positive regulation of apoptosis
GO:0043068	156	3	0.28164714	positive regulation of programmed cell death
GO:0050789	2689	35	0.283773291	regulation of biological process
GO:0046651	28	1	0.284084223	lymphocyte proliferation
GO:0051348	28	1	0.284084223	negative regulation of transferase activity
GO:0051251	28	1	0.284084223	positive regulation of lymphocyte activation
GO:0006469	28	1	0.284084223	negative regulation of protein kinase activity
GO:0048511	29	1	0.292591885	rhythmic process
GO:0050867	29	1	0.292591885	positive regulation of cell activation
GO:0042981	306	5	0.296621732	regulation of apoptosis
GO:0006486	92	2	0.297609587	protein amino acid glycosylation
GO:0009891	30	1	0.300999379	positive regulation of biosynthesis
GO:0051129	30	1	0.300999379	negative regulation of cell organization and biogenesis

GO:0051261	30	1	0.300999379	protein depolymerization
GO:0044260	2298	30	0.301108051	cellular macromolecule metabolism
GO:0043067	308	5	0.30126433	regulation of programmed cell death
GO:0043413	93	2	0.301998815	biopolymer glycosylation
GO:0006875	93	2	0.301998815	metal ion homeostasis
GO:0007187	94	2	0.306383635	G-protein signaling, coupled to cyclic nucleotide second messenger
GO:0043280	31	1	0.309307874	positive regulation of caspase activity
GO:0043281	31	1	0.309307874	regulation of caspase activity
GO:0050863	31	1	0.309307874	regulation of T cell activation
GO:0006367	31	1	0.309307874	transcription initiation from RNA polymerase II promoter
GO:0009101	96	2	0.315138029	glycoprotein biosynthesis
GO:0031279	32	1	0.317518523	regulation of cyclase activity
GO:0045761	32	1	0.317518523	regulation of adenylate cyclase activity
GO:0051345	32	1	0.317518523	positive regulation of hydrolase activity
GO:0051339	32	1	0.317518523	regulation of lyase activity
GO:0019935	98	2	0.323868856	cyclic-nucleotide-mediated signaling
GO:0030003	98	2	0.323868856	cation homeostasis
GO:0050794	2488	32	0.325117946	regulation of cellular process
GO:0006690	33	1	0.32563247	icosanoid metabolism
GO:0042035	33	1	0.32563247	regulation of cytokine biosynthesis
GO:0009314	33	1	0.32563247	response to radiation
GO:0008064	33	1	0.32563247	regulation of actin polymerization and/or depolymerization
GO:0006665	34	1	0.333650841	sphingolipid metabolism
GO:0048513	872	12	0.336611202	organ development
GO:0030832	35	1	0.341574753	regulation of actin filament length
GO:0006873	103	2	0.345568832	cell ion homeostasis
GO:0048522	482	7	0.345585924	positive regulation of cellular process
GO:0006793	641	9	0.348370934	phosphorus metabolism
GO:0006796	641	9	0.348370934	phosphate metabolism
GO:0042089	36	1	0.349405308	cytokine biosynthesis
GO:0044237	5026	62	0.351981448	cellular metabolism
GO:0006511	105	2	0.354189048	ubiquitin-dependent protein catabolism
GO:0019941	105	2	0.354189048	modification-dependent protein catabolism
GO:0042107	37	1	0.357143595	cytokine metabolism
GO:0006487	37	1	0.357143595	protein amino acid N-linked glycosylation
GO:0006643	106	2	0.358484753	membrane lipid metabolism
GO:0015698	106	2	0.358484753	inorganic anion transport
GO:0051242	411	6	0.360306143	positive regulation of cellular physiological process
GO:0006520	181	3	0.362892382	amino acid metabolism
GO:0006821	38	1	0.364790692	chloride transport
GO:0019222	1629	21	0.371599393	regulation of metabolism
GO:0050801	111	2	0.37980465	ion homeostasis
GO:0001505	41	1	0.387195424	regulation of neurotransmitter levels
GO:0051249	41	1	0.387195424	regulation of lymphocyte activation
GO:0031323	1566	20	0.393939783	regulation of cellular metabolism
GO:0009063	42	1	0.394488282	amino acid catabolism
GO:0006897	115	2	0.396650045	endocytosis

GO:0050865	43	1	0.401695152	regulation of cell activation
GO:0008154	45	1	0.415854928	actin polymerization and/or depolymerization
GO:0009310	45	1	0.415854928	amine catabolism
GO:0042110	45	1	0.415854928	T cell activation
GO:0019752	359	5	0.421691096	carboxylic acid metabolism
GO:0043086	46	1	0.42280981	negative regulation of enzyme activity
GO:0006974	200	3	0.423895077	response to DNA damage stimulus
GO:0019725	122	2	0.425618048	cell homeostasis
GO:0006082	361	5	0.426407311	organic acid metabolism
GO:0006633	47	1	0.429682651	fatty acid biosynthesis
GO:0006352	47	1	0.429682651	transcription initiation
GO:0044270	47	1	0.429682651	nitrogen compound catabolism
GO:0045449	1433	18	0.433146461	regulation of transcription
GO:0006631	124	2	0.433764923	fatty acid metabolism
GO:0006334	48	1	0.43647441	nucleosome assembly
GO:0030036	126	2	0.441850769	actin cytoskeleton organization and biogenesis
GO:0007586	49	1	0.443186035	digestion
GO:0046903	208	3	0.449033711	secretion
GO:0006820	128	2	0.44987393	anion transport
GO:0051128	52	1	0.462849417	regulation of cell organization and biogenesis
GO:0019219	1458	18	0.464437732	regulation of nucleobase, nucleoside, nucleotide and nucleic acid metabolism
GO:0050790	214	3	0.467604689	regulation of enzyme activity
GO:0045860	53	1	0.469249764	positive regulation of protein kinase activity
GO:0046467	53	1	0.469249764	membrane lipid biosynthesis
GO:0045087	53	1	0.469249764	innate immune response
GO:0006519	216	3	0.473735512	amino acid and derivative metabolism
GO:0016053	54	1	0.475574552	organic acid biosynthesis
GO:0051347	54	1	0.475574552	positive regulation of transferase activity
GO:0046394	54	1	0.475574552	carboxylic acid biosynthesis
GO:0006887	55	1	0.481824666	exocytosis
GO:0031497	56	1	0.488000979	chromatin assembly
GO:0007200	56	1	0.488000979	G-protein signaling, coupled to IP3 second messenger (phospholipase C activating)
GO:0008285	139	2	0.492816871	negative regulation of cell proliferation
GO:0030029	139	2	0.492816871	actin filament-based process
GO:0006817	57	1	0.494104354	phosphate transport
GO:0050791	2407	29	0.494625232	regulation of physiological process
GO:0006260	140	2	0.496617068	DNA replication
GO:0051248	58	1	0.500135645	negative regulation of protein metabolism
GO:0000074	394	5	0.502864103	regulation of progression through cell cycle
GO:0009117	143	2	0.50791007	nucleotide metabolism
GO:0009581	313	4	0.510978379	detection of external stimulus
GO:0051336	60	1	0.511985337	regulation of hydrolase activity
GO:0045786	145	2	0.515348008	negative regulation of progression through cell cycle
GO:0006350	1499	18	0.515515148	transcription
GO:0006800	61	1	0.517805396	oxygen and reactive oxygen species metabolism
GO:0007050	61	1	0.517805396	cell cycle arrest
GO:0050953	149	2	0.530002728	sensory perception of light

GO:0007601	149	2	0.530002728	visual perception
GO:0006364	64	1	0.534856169	rRNA processing
GO:0001525	65	1	0.540405945	angiogenesis
GO:0009790	65	1	0.540405945	embryonic development
GO:0016072	66	1	0.545890117	rRNA metabolism
GO:0044238	4820	57	0.54746876	primary metabolism
GO:0007243	242	3	0.550283934	protein kinase cascade
GO:0006355	1359	16	0.552694488	regulation of transcription, DNA-dependent
GO:0001568	68	1	0.55666471	blood vessel development
GO:0043123	68	1	0.55666471	positive regulation of I-kappaB kinase/NF-kappaB cascade
GO:0048514	68	1	0.55666471	blood vessel morphogenesis
GO:0042592	157	2	0.558410665	homeostasis
GO:0001944	69	1	0.561956641	vasculature development
GO:0008152	5262	62	0.567594562	metabolism
GO:0007275	1543	18	0.569208543	development
GO:0007049	601	7	0.57461862	cell cycle
GO:0051244	2319	27	0.579030138	regulation of cellular physiological process
GO:0043122	73	1	0.582505813	regulation of I-kappaB kinase/NF-kappaB cascade
GO:0006118	256	3	0.58875028	electron transport
GO:0007596	78	1	0.606853952	blood coagulation
GO:0006468	444	5	0.610381202	protein amino acid phosphorylation
GO:0007046	79	1	0.611552034	ribosome biogenesis
GO:0050817	79	1	0.611552034	coagulation
GO:0006351	1407	16	0.612376886	transcription, DNA-dependent
GO:0042254	80	1	0.616194493	ribosome biogenesis and assembly
GO:0008610	175	2	0.617831002	lipid biosynthesis
GO:0009308	269	3	0.622562548	amine metabolism
GO:0007599	83	1	0.629794615	hemostasis
GO:0042060	85	1	0.638595006	wound healing
GO:0000004	465	5	0.651437195	biological process unknown
GO:0006333	91	1	0.663771673	chromatin assembly or disassembly
GO:0044255	383	4	0.671024917	cellular lipid metabolism
GO:0006807	291	3	0.675414039	nitrogen compound metabolism
GO:0050878	94	1	0.675700384	regulation of body fluids
GO:0007028	96	1	0.683418951	cytoplasm organization and biogenesis
GO:0016192	296	3	0.686648288	vesicle-mediated transport
GO:0016070	391	4	0.686875652	RNA metabolism
GO:0007249	99	1	0.694656825	I-kappaB kinase/NF-kappaB cascade
GO:0007010	305	3	0.706144567	cytoskeleton organization and biogenesis
GO:0009165	103	1	0.709028109	nucleotide biosynthesis
GO:0006412	407	4	0.716971802	protein biosynthesis
GO:0006417	107	1	0.72272902	regulation of protein biosynthesis
GO:0009582	109	1	0.729337826	detection of abiotic stimulus
GO:0031326	111	1	0.735790547	regulation of cellular biosynthesis
GO:0006916	112	1	0.73895952	anti-apoptosis
GO:0019318	113	1	0.742090835	hexose metabolism
GO:0005996	115	1	0.748242259	monosaccharide metabolism

GO:0016310	522	5	0.748595528	phosphorylation
GO:0045859	116	1	0.751263239	regulation of protein kinase activity
GO:0007169	116	1	0.751263239	transmembrane receptor protein tyrosine kinase signaling pathway
GO:0009889	116	1	0.751263239	regulation of biosynthesis
GO:0051338	117	1	0.754248303	regulation of transferase activity
GO:0043066	128	1	0.784831103	negative regulation of apoptosis
GO:0043069	129	1	0.787416803	negative regulation of programmed cell death
GO:0007067	135	1	0.802297067	mitosis
GO:0000087	137	1	0.807024094	M phase of mitotic cell cycle
GO:0009059	464	4	0.807042534	macromolecule biosynthesis
GO:0007267	465	4	0.808392689	cell-cell signaling
GO:0009058	859	8	0.810294214	biosynthesis
GO:0044249	763	7	0.810335986	cellular biosynthesis
GO:0006629	469	4	0.81371782	lipid metabolism
GO:0006139	2328	24	0.816995064	nucleobase, nucleoside, nucleotide and nucleic acid metabolism
GO:0045045	161	1	0.855723027	secretory pathway
GO:0007600	287	2	0.859117333	sensory perception
GO:0007167	165	1	0.862560175	enzyme linked receptor protein signaling pathway
GO:0016337	172	1	0.873761505	cell-cell adhesion
GO:0000279	173	1	0.875286056	M phase
GO:0006281	174	1	0.876792365	DNA repair
GO:0006325	186	1	0.893521453	establishment and/or maintenance of chromatin architecture
GO:0007268	192	1	0.90102126	synaptic transmission
GO:0006323	193	1	0.902219306	DNA packaging
GO:0006396	326	2	0.903342781	RNA processing
GO:0006091	446	3	0.904427994	generation of precursor metabolites and energy
GO:0019226	198	1	0.907997409	transmission of nerve impulse
GO:0000278	204	1	0.914486142	mitotic cell cycle
GO:0006996	693	5	0.921399636	organelle organization and biogenesis
GO:0006066	213	1	0.923378541	alcohol metabolism
GO:0009887	807	6	0.925218673	organogenesis
GO:0050877	475	3	0.925820288	neurophysiological process
GO:0005975	355	2	0.92749564	carbohydrate metabolism
GO:0007001	221	1	0.930510104	chromosome organization and biogenesis (sensu Eukaryota)
GO:0006259	489	3	0.934509915	DNA metabolism
GO:0007264	227	1	0.935423477	small GTPase mediated signal transduction
GO:0051276	236	1	0.942155351	chromosome organization and biogenesis
GO:0006811	515	3	0.94822921	ion transport
GO:0006512	255	1	0.954168294	ubiquitin cycle
GO:0044262	265	1	0.959461422	cellular carbohydrate metabolism
GO:0050875	7018	76	0.959629217	cellular physiological process
GO:0009987	8200	92	0.964702431	cellular process
GO:0045184	459	2	0.975216915	establishment of protein localization
GO:0016043	842	5	0.976107282	cell organization and biogenesis
GO:0008104	474	2	0.978871452	protein localization
GO:0051179	1857	14	0.983753051	localization
GO:0009653	1051	6	0.989062832	morphogenesis

GO:0051234	1841	13	0.991102648	establishment of localization
GO:0015031	439	1	0.995316503	protein transport
GO:0006810	1802	11	0.997702791	transport
GO:0008150	9032	107	1	biological_process

23.3 GO: Molecular Function

GO ID	genes present in chip	genes present in tissues	p-value	GO term
GO:0003823	30	7	3.42963038489691e-08	antigen binding
GO:0004871	1837	43	7.30161213009664e-07	signal transducer activity
GO:0004872	1060	27	4.23742801284588e-05	receptor activity
GO:0030106	26	4	0.00019852232680938	MHC class I receptor activity
GO:0045012	12	3	0.000296045983309408	MHC class II receptor activity
GO:0005529	103	6	0.00112851322727185	sugar binding
GO:0004888	694	17	0.00214579253105804	transmembrane receptor activity
GO:0017124	12	2	0.00792101518518785	SH3 domain binding
GO:0008656	12	2	0.00792101518518785	caspase activator activity
GO:0004725	75	4	0.0105266848911115	protein tyrosine phosphatase activity
GO:0030693	14	2	0.0107601524279605	caspase activity
GO:0016505	14	2	0.0107601524279605	apoptotic protease activator activity
GO:0008310	1	1	0.0114243789959120	single-stranded DNA specific 3'-5' exodeoxyribonuclease activity
GO:0008120	1	1	0.0114243789959120	ceramide glucosyltransferase activity
GO:0008859	1	1	0.0114243789959120	exoribonuclease II activity
GO:0035197	1	1	0.0114243789959120	siRNA binding
GO:0047290	1	1	0.0114243789959120	(alpha-N-acetylneuraminyl-2,3-beta-galactosyl-1,3)-N-acetyl-galactosaminide 6-alpha-sialyltransferase activity
GO:0003933	1	1	0.0114243789959120	GTP cyclohydrolase activity
GO:0003934	1	1	0.0114243789959120	GTP cyclohydrolase I activity
GO:0004137	1	1	0.0114243789959120	deoxycytidine kinase activity
GO:0004278	1	1	0.0114243789959120	granzyme B activity
GO:0004218	1	1	0.0114243789959120	cathepsin S activity
GO:0030881	1	1	0.0114243789959120	beta-2-microglobulin binding
GO:0030882	1	1	0.0114243789959120	lipid antigen binding
GO:0030884	1	1	0.0114243789959120	exogenous lipid antigen binding
GO:0045029	1	1	0.0114243789959120	UDP-activated nucleotide receptor activity
GO:0016504	15	2	0.0123236886966552	protease activator activity
GO:0030246	181	6	0.0173886090083897	carbohydrate binding
GO:0005001	19	2	0.0194838183861057	transmembrane receptor protein tyrosine phosphatase activity
GO:0043028	19	2	0.0194838183861057	caspase regulator activity
GO:0019198	19	2	0.0194838183861057	transmembrane receptor protein phosphatase activity
GO:0008047	188	6	0.0205570161243295	enzyme activator activity
GO:0003690	20	2	0.0214892111475582	double-stranded DNA binding
GO:0030676	2	1	0.0227194253994182	Rac guanyl-nucleotide exchange factor activity
GO:0005153	2	1	0.0227194253994182	interleukin-8 receptor binding
GO:0008297	2	1	0.0227194253994182	single-stranded DNA specific exodeoxyribonuclease activity
GO:0015065	2	1	0.0227194253994182	uridine nucleotide receptor activity
GO:0016564	59	3	0.0297055836394545	transcriptional repressor activity
GO:0005128	3	1	0.0338865899482665	erythropoietin receptor binding
GO:0001948	3	1	0.0338865899482665	glycoprotein binding
GO:0008296	3	1	0.0338865899482665	3'-5'-exodeoxyribonuclease activity
GO:0003796	3	1	0.0338865899482665	lysozyme activity
GO:0016019	3	1	0.0338865899482665	peptidoglycan receptor activity

GO:0042608	3	1	0.0338865899482665	T cell receptor binding
GO:0005178	26	2	0.0351678015818809	integrin binding
GO:0003725	26	2	0.0351678015818809	double-stranded RNA binding
GO:0008020	4	1	0.0449273072575173	G-protein coupled photoreceptor activity
GO:0019136	4	1	0.0449273072575173	deoxynucleoside kinase activity
GO:0019238	4	1	0.0449273072575173	cyclohydrolase activity
GO:0004721	118	4	0.0459525812469213	phosphoprotein phosphatase activity
GO:0004559	5	1	0.0558429959970507	alpha-mannosidase activity
GO:0008301	5	1	0.0558429959970507	DNA bending activity
GO:0019863	5	1	0.0558429959970507	IgE binding
GO:0009881	5	1	0.0558429959970507	photoreceptor activity
GO:0004185	5	1	0.0558429959970507	serine carboxypeptidase activity
GO:0004197	80	3	0.063355191221721	cysteine-type endopeptidase activity
GO:0003700	649	12	0.0661568008392118	transcription factor activity
GO:0005031	6	1	0.0666350590671347	tumor necrosis factor receptor activity
GO:0008329	6	1	0.0666350590671347	pattern recognition receptor activity
GO:0008009	40	2	0.0762009277680908	chemokine activity
GO:0042379	40	2	0.0762009277680908	chemokine receptor binding
GO:0005035	7	1	0.0773048837720828	death receptor activity
GO:0004529	7	1	0.0773048837720828	exodeoxyribonuclease activity
GO:0043120	7	1	0.0773048837720828	tumor necrosis factor binding
GO:0042834	7	1	0.0773048837720828	peptidoglycan binding
GO:0016895	7	1	0.0773048837720828	exodeoxyribonuclease activity, producing 5'-phosphomonoesters
GO:0030528	963	16	0.0801959318348824	transcription regulator activity
GO:0001664	42	2	0.0829038316215289	G-protein-coupled receptor binding
GO:0017017	8	1	0.0878538419920163	MAP kinase phosphatase activity
GO:0035251	8	1	0.0878538419920163	UDP-glucosyltransferase activity
GO:0004540	45	2	0.0932799144382901	ribonuclease activity
GO:0046527	9	1	0.0982832903527494	glucosyltransferase activity
GO:0004522	9	1	0.0982832903527494	pancreatic ribonuclease activity
GO:0016494	9	1	0.0982832903527494	C-X-C chemokine receptor activity
GO:0004016	9	1	0.0982832903527494	adenylate cyclase activity
GO:0008234	98	3	0.101576002157364	cysteine-type peptidase activity
GO:0019958	10	1	0.108594570393827	C-X-C chemokine binding
GO:0000175	10	1	0.108594570393827	3'-5'-exoribonuclease activity
GO:0004716	10	1	0.108594570393827	receptor signaling protein tyrosine kinase activity
GO:0004532	11	1	0.118789008734722	exoribonuclease activity
GO:0005355	11	1	0.118789008734722	glucose transporter activity
GO:0019206	11	1	0.118789008734722	nucleoside kinase activity
GO:0003727	11	1	0.118789008734722	single-stranded RNA binding
GO:0016892	11	1	0.118789008734722	endoribonuclease activity, producing 3'-phosphomonoesters
GO:0016896	11	1	0.118789008734722	exoribonuclease activity, producing 5'-phosphomonoesters
GO:0019904	52	2	0.118802906976833	protein domain specific binding
GO:0030234	509	9	0.127175532116168	enzyme regulator activity
GO:0019865	12	1	0.128867917239226	immunoglobulin binding
GO:0016493	12	1	0.128867917239226	C-C chemokine receptor activity
GO:0019957	12	1	0.128867917239226	C-C chemokine binding

GO:0015145	12	1	0.128867917239226	monosaccharide transporter activity
GO:0015149	12	1	0.128867917239226	hexose transporter activity
GO:0046983	170	4	0.129828681578864	protein dimerization activity
GO:0016791	171	4	0.131837250040022	phosphoric monoester hydrolase activity
GO:0015923	13	1	0.138832593178036	mannosidase activity
GO:0016894	13	1	0.138832593178036	endonuclease activity, active with either ribo- or deoxyribonucleic acids and producing 3'-phosphomonoesters
GO:0015026	13	1	0.138832593178036	coreceptor activity
GO:0004553	59	2	0.145819468385063	hydrolase activity, hydrolyzing O-glycosyl compounds
GO:0008373	14	1	0.148684319389581	sialyltransferase activity
GO:0005540	14	1	0.148684319389581	hyaluronic acid binding
GO:0005102	528	9	0.148955462912093	receptor binding
GO:0005125	181	4	0.152613798759946	cytokine activity
GO:0005515	3709	48	0.155500427142843	protein binding
GO:0004864	15	1	0.158424364439075	protein phosphatase inhibitor activity
GO:0004715	15	1	0.158424364439075	non-membrane spanning protein tyrosine kinase activity
GO:0005247	16	1	0.168053982775852	voltage-gated chloride channel activity
GO:0003705	16	1	0.168053982775852	RNA polymerase II transcription factor activity, enhancer binding
GO:0019955	65	2	0.169877210004087	cytokine binding
GO:0003779	189	4	0.170077504660699	actin binding
GO:0019212	17	1	0.177574414888969	phosphatase inhibitor activity
GO:0016796	17	1	0.177574414888969	exonuclease activity, active with either ribo- or deoxyribonucleic acids and producing 5'-phosphomonoesters
GO:0030674	69	2	0.186266016856918	protein binding, bridging
GO:0016849	18	1	0.186986887461113	phosphorus-oxygen lyase activity
GO:0004298	18	1	0.186986887461113	threonine endopeptidase activity
GO:0003702	197	4	0.188212077202291	RNA polymerase II transcription factor activity
GO:0005488	7228	87	0.189949313054189	binding
GO:0005057	132	3	0.191558225357321	receptor signaling protein activity
GO:0004869	19	1	0.196292613520835	cysteine protease inhibitor activity
GO:0001614	20	1	0.205492792593113	purinergic nucleotide receptor activity
GO:0001608	20	1	0.205492792593113	nucleotide receptor activity, G-protein coupled
GO:0005164	20	1	0.205492792593113	tumor necrosis factor receptor binding
GO:0016502	20	1	0.205492792593113	nucleotide receptor activity
GO:0009975	20	1	0.205492792593113	cyclase activity
GO:0045028	20	1	0.205492792593113	purinergic nucleotide receptor activity, G-protein coupled
GO:0016814	21	1	0.21458861084827	hydrolase activity, acting on carbon-nitrogen (but not peptide) bonds, in cyclic amidines
GO:0016798	77	2	0.219634271292429	hydrolase activity, acting on glycosyl bonds
GO:0001637	22	1	0.223581241249272	G-protein chemoattractant receptor activity
GO:0004950	22	1	0.223581241249272	chemokine receptor activity
GO:0004175	288	5	0.232834765517494	endopeptidase activity
GO:0003714	81	2	0.236514466207673	transcription corepressor activity
GO:0008408	24	1	0.241261565176396	3'-5' exonuclease activity
GO:0019956	24	1	0.241261565176396	chemokine binding
GO:0004860	25	1	0.249951539894876	protein kinase inhibitor activity
GO:0004536	25	1	0.249951539894876	deoxyribonuclease activity
GO:0005200	86	2	0.257710026242442	structural constituent of cytoskeleton
GO:0008138	26	1	0.258542889427395	protein tyrosine/serine/threonine phosphatase activity
GO:0003704	26	1	0.258542889427395	specific RNA polymerase II transcription factor activity

GO:0042578	228	4	0.26344139981588	phosphoric ester hydrolase activity
GO:0001871	88	2	0.266201839657864	pattern binding
GO:0016787	1424	19	0.266694432244239	hydrolase activity
GO:0004180	27	1	0.267036722853819	carboxypeptidase activity
GO:0003697	28	1	0.275434136897222	single-stranded DNA binding
GO:0004713	161	3	0.279274464205580	protein-tyrosine kinase activity
GO:0019210	29	1	0.283736216060283	kinase inhibitor activity
GO:0051119	29	1	0.283736216060283	sugar transporter activity
GO:0015144	30	1	0.291944032760181	carbohydrate transporter activity
GO:0004521	31	1	0.300058647462014	endoribonuclease activity
GO:0008233	399	6	0.304907567264464	peptidase activity
GO:0019205	34	1	0.323853707710077	nucleobase, nucleoside, nucleotide kinase activity
GO:0008094	34	1	0.323853707710077	DNA-dependent ATPase activity
GO:0019888	35	1	0.331605884617577	protein phosphatase regulator activity
GO:0004518	105	2	0.33800245513025	nuclease activity
GO:0019208	36	1	0.339269987139845	phosphatase regulator activity
GO:0005126	38	1	0.354337923866098	hematopoietin/interferon-class (D200-domain) cytokine receptor binding
GO:0004857	187	3	0.360714325222795	enzyme inhibitor activity
GO:0005516	112	2	0.367043220127646	calmodulin binding
GO:0004527	40	1	0.369065317714366	exonuclease activity
GO:0005070	40	1	0.369065317714366	SH3/SH2 adaptor activity
GO:0008236	117	2	0.387491844401499	serine-type peptidase activity
GO:0005096	117	2	0.387491844401499	GTPase activator activity
GO:0005254	43	1	0.390534527777025	chloride channel activity
GO:0008092	279	4	0.395487225642378	cytoskeletal protein binding
GO:0050660	44	1	0.397528814623973	FAD binding
GO:0016788	446	6	0.401171847911997	hydrolase activity, acting on ester bonds
GO:0003924	121	2	0.403645303089121	GTPase activity
GO:0031404	45	1	0.404443562355465	chloride ion binding
GO:0043168	45	1	0.404443562355465	anion binding
GO:0005253	46	1	0.411279667210483	anion channel activity
GO:0003677	1384	17	0.413389835344998	DNA binding
GO:0005244	124	2	0.415629613157014	voltage-gated ion channel activity
GO:0019887	47	1	0.418038015422601	protein kinase regulator activity
GO:0008134	293	4	0.431686526354399	transcription factor binding
GO:0035091	132	2	0.446990195315317	phosphoinositide binding
GO:0030695	219	3	0.458890761960489	GTPase regulator activity
GO:0005509	646	8	0.459956393185665	calcium ion binding
GO:0019901	54	1	0.463241659958393	protein kinase binding
GO:0003713	140	2	0.477405230236845	transcription coactivator activity
GO:0019207	58	1	0.487491681031037	kinase regulator activity
GO:0003712	230	3	0.491264566446168	transcription cofactor activity
GO:0008083	144	2	0.492233359909584	growth factor activity
GO:0019900	60	1	0.499206384101196	kinase binding
GO:0046982	61	1	0.504963846830125	protein heterodimerization activity
GO:0016563	62	1	0.510655718211508	transcriptional activator activity
GO:0019899	151	2	0.517544692748956	enzyme binding

GO:0042803	64	1	0.521845640260665	protein homodimerization activity
GO:0004519	65	1	0.527345146983424	endonuclease activity
GO:0042802	158	2	0.542017508138201	protein self binding
GO:0008238	68	1	0.543470416208799	exopeptidase activity
GO:0004497	71	1	0.559050425753972	monooxygenase activity
GO:0016810	72	1	0.56412576932238	hydrolase activity, acting on carbon-nitrogen (but not peptide) bonds
GO:0016757	165	2	0.565629246059406	transferase activity, transferring glycosyl groups
GO:0005543	169	2	0.578729299714368	phospholipid binding
GO:0005539	77	1	0.588647148696375	glycosaminoglycan binding
GO:0008194	79	1	0.598068450198914	UDP-glycosyltransferase activity
GO:0030247	80	1	0.602698606500786	polysaccharide binding
GO:0003676	1990	22	0.607055190974335	nucleic acid binding
GO:0001584	282	3	0.629834766332027	rhodopsin-like receptor activity
GO:0004842	87	1	0.633662786720465	ubiquitin-protein ligase activity
GO:0050662	88	1	0.637886475767161	coenzyme binding
GO:0005179	90	1	0.64618963389063	hormone activity
GO:0048503	92	1	0.654304109944418	GPI anchor binding
GO:0030145	92	1	0.654304109944418	manganese ion binding
GO:0008026	93	1	0.658291923683021	ATP-dependent helicase activity
GO:0005085	93	1	0.658291923683021	guanyl-nucleotide exchange factor activity
GO:0001653	94	1	0.662234152759921	peptide receptor activity
GO:0008528	94	1	0.662234152759921	peptide receptor activity, G-protein coupled
GO:0008509	95	1	0.666131313482814	anion transporter activity
GO:0008289	298	3	0.666950347968797	lipid binding
GO:0004866	99	1	0.681279397551473	endopeptidase inhibitor activity
GO:0048037	100	1	0.684958760574867	cofactor binding
GO:0030414	100	1	0.684958760574867	protease inhibitor activity
GO:0016881	101	1	0.688596033649471	acid-amino acid ligase activity
GO:0004386	101	1	0.688596033649471	helicase activity
GO:0017111	410	4	0.69516285308848	nucleoside-triphosphatase activity
GO:0009055	104	1	0.69926005724085	electron carrier activity
GO:0004252	105	1	0.702733688030375	serine-type endopeptidase activity
GO:0016462	430	4	0.730455846228068	pyrophosphatase activity
GO:0016818	432	4	0.733814280424794	hydrolase activity, acting on acid anhydrides, in phosphorus-containing anhydrides
GO:0005525	226	2	0.734556926497163	GTP binding
GO:0016758	115	1	0.735356080584016	transferase activity, transferring hexosyl groups
GO:0016817	433	4	0.735481897581837	hydrolase activity, acting on acid anhydrides
GO:0003723	436	4	0.740438431282306	RNA binding
GO:0042623	229	2	0.741230048045397	ATPase activity, coupled
GO:0019001	235	2	0.754151527482983	guanyl nucleotide binding
GO:0004930	349	3	0.767111311349	G-protein coupled receptor activity
GO:0042277	127	1	0.769850393855245	peptide binding
GO:0016879	127	1	0.769850393855245	ligase activity, forming carbon-nitrogen bonds
GO:0016829	127	1	0.769850393855245	lyase activity
GO:0000287	251	2	0.785944507475128	magnesium ion binding
GO:0005216	252	2	0.78780768487937	ion channel activity
GO:0016887	255	2	0.793312774138987	ATPase activity

GO:0005083	144	1	0.811220959783875	small GTPase regulator activity
GO:0005198	488	4	0.815616115306988	structural molecule activity
GO:0015268	274	2	0.825359192971334	alpha-type channel activity
GO:0005489	152	1	0.828049905786595	electron transporter activity
GO:0016301	609	5	0.833619678630982	kinase activity
GO:0015267	284	2	0.840388046182194	channel or pore class transporter activity
GO:0016773	532	4	0.864569416052126	phosphotransferase activity, alcohol group as acceptor
GO:0004672	447	3	0.891227036860823	protein kinase activity
GO:0005506	212	1	0.914855834756091	iron ion binding
GO:0016772	708	5	0.91496067164771	transferase activity, transferring phosphorus-containing groups
GO:0016874	221	1	0.923405511757514	ligase activity
GO:0030554	1028	7	0.957009919775781	adenyl nucleotide binding
GO:0017076	1248	9	0.95734807085441	purine nucleotide binding
GO:0005524	970	6	0.971445344868158	ATP binding
GO:0003824	3409	30	0.973127111662843	catalytic activity
GO:0000166	1432	10	0.974206426775422	nucleotide binding
GO:0016491	509	2	0.982304777914029	oxidoreductase activity
GO:0005554	522	2	0.984538219001765	molecular function unknown
GO:0015075	523	2	0.984698298824817	ion transporter activity
GO:0016740	1169	7	0.984823505677205	transferase activity
GO:0008270	1287	8	0.985099154277336	zinc ion binding
GO:0043169	2313	17	0.98998973888165	cation binding
GO:0005386	398	1	0.990642026542462	carrier activity
GO:0046914	1596	10	0.991952389746352	transition metal ion binding
GO:0043167	2556	19	0.992238887934053	ion binding
GO:0046872	2495	18	0.994090353946078	metal ion binding
GO:0005215	1030	4	0.998230141736379	transporter activity
GO:0003674	9541	109	1	molecular_function

23.4 GO: Cellular Component

GO ID	genes present in chip	genes present in tissues	p-value	GO term
GO:0005886	1472	28	0.0124628230010878	plasma membrane
GO:0043020	1	1	0.012504341785342	NADPH oxidase complex
GO:0043235	58	3	0.0357195637797016	receptor complex
GO:0008305	24	2	0.0357537422837686	integrin complex
GO:0005764	107	4	0.044804335109624	lysosome
GO:0000323	107	4	0.044804335109624	lytic vacuole
GO:0005576	949	18	0.0464197373943046	extracellular region
GO:0016020	3514	53	0.0465960152570329	membrane
GO:0001772	29	2	0.0505255548320909	immunological synapse
GO:0005615	385	9	0.0513184404007308	extracellular space
GO:0005577	5	1	0.0609913246602207	fibrinogen complex
GO:0005773	120	4	0.0632021042603082	vacuole
GO:0005887	1057	19	0.0643796339788087	integral to plasma membrane
GO:0031226	1062	19	0.0668716431260329	intrinsic to plasma membrane
GO:0019898	37	2	0.0777609883537831	extrinsic to membrane
GO:0008290	7	1	0.0843426499789965	F-actin capping protein complex
GO:0000502	41	2	0.0927554743456226	proteasome complex (sensu Eukaryota)
GO:0005925	8	1	0.0958016295621095	focal adhesion
GO:0016605	8	1	0.0958016295621095	PML body
GO:0005765	9	1	0.107118517267207	lysosomal membrane
GO:0005924	9	1	0.107118517267207	cell-substrate adherens junction
GO:0042101	10	1	0.118295058775685	T cell receptor complex
GO:0030055	11	1	0.129332978522089	cell-matrix junction
GO:0005829	276	6	0.131435522126008	cytosol
GO:0001533	12	1	0.140233979950284	cornified envelope
GO:0016021	2674	39	0.144730739488720	integral to membrane
GO:0031224	2681	39	0.149009339795938	intrinsic to membrane
GO:0005769	13	1	0.150999745766559	early endosome
GO:0001726	14	1	0.161631938189705	ruffle
GO:0005626	15	1	0.172132199198114	insoluble fraction
GO:0005665	16	1	0.18250215077392	DNA-directed RNA polymerase II, core complex
GO:0016604	16	1	0.18250215077392	nuclear body
GO:0045111	65	2	0.195101970774035	intermediate filament cytoskeleton
GO:0005882	65	2	0.195101970774035	intermediate filament
GO:0005625	183	4	0.195771545189416	soluble fraction
GO:0005839	18	1	0.202857515019451	proteasome core complex (sensu Eukaryota)
GO:0005912	18	1	0.202857515019451	adherens junction
GO:0005834	21	1	0.232452666954198	heterotrimeric G-protein complex
GO:0005856	612	10	0.234262186096324	cytoskeleton
GO:0048471	22	1	0.242073733802962	perinuclear region
GO:0000793	23	1	0.251575305102936	condensed chromosome
GO:0030880	23	1	0.251575305102936	RNA polymerase complex
GO:0000267	698	11	0.254959441625815	cell fraction

GO:0031252	24	1	0.260958851312465	leading edge
GO:0005793	24	1	0.260958851312465	ER-Golgi intermediate compartment
GO:0019897	26	1	0.279377660879176	extrinsic to plasma membrane
GO:0008021	27	1	0.288415776385512	synaptic vesicle
GO:0005938	28	1	0.297341571524927	cell cortex
GO:0030173	29	1	0.306156429263957	integral to Golgi membrane
GO:0031228	35	1	0.356791236355498	intrinsic to Golgi membrane
GO:0015629	183	3	0.402119669104531	actin cytoskeleton
GO:0000228	42	1	0.411253695111349	nuclear chromosome
GO:0016327	50	1	0.467920227401951	apicolateral plasma membrane
GO:0016591	58	1	0.51917827752198	DNA-directed RNA polymerase II, holoenzyme
GO:0005654	221	3	0.525308750163513	nucleoplasm
GO:0005768	62	1	0.542941003217416	endosome
GO:0030136	65	1	0.559996007917482	clathrin-coated vesicle
GO:0000139	67	1	0.571014162285838	Golgi membrane
GO:0043228	977	12	0.572058840427294	non-membrane-bound organelle
GO:0043232	977	12	0.572058840427294	intracellular non-membrane-bound organelle
GO:0042995	70	1	0.58703084849532	cell projection
GO:0005730	75	1	0.612419046262106	nucleolus
GO:0031301	76	1	0.617307944066789	integral to organelle membrane
GO:0030135	79	1	0.631611030343774	coated vesicle
GO:0005624	521	6	0.641316527092155	membrane fraction
GO:0008372	523	6	0.64504346134286	cellular component unknown
GO:0005737	2530	30	0.67100986237087	cytoplasm
GO:0031300	90	1	0.679668527370419	intrinsic to organelle membrane
GO:0030054	93	1	0.691660255770154	cell junction
GO:0000785	100	1	0.717938926220023	chromatin
GO:0005783	483	5	0.729636576169688	endoplasmic reticulum
GO:0005792	108	1	0.74526356153847	microsome
GO:0042598	108	1	0.74526356153847	vesicular fraction
GO:0005623	7802	96	0.757501022684005	cell
GO:0016023	122	1	0.786916744654435	cytoplasmic membrane-bound vesicle
GO:0031410	124	1	0.792288061924968	cytoplasmic vesicle
GO:0005634	2704	29	0.867060250472846	nucleus
GO:0005578	303	2	0.897351605437922	extracellular matrix (sensu Metazoa)
GO:0031012	304	2	0.898404741090302	extracellular matrix
GO:0005794	195	1	0.916413841316647	Golgi apparatus
GO:0005694	203	1	0.92459834546268	chromosome
GO:0012505	246	1	0.956739005160625	endomembrane system
GO:0043234	1247	9	0.980894288887891	protein complex
GO:0005622	5419	57	0.98710606282982	intracellular
GO:0043226	4515	45	0.98981439970883	organelle
GO:0043229	4515	45	0.98981439970883	intracellular organelle
GO:0043227	3955	38	0.990448274373968	membrane-bound organelle
GO:0043231	3955	38	0.990448274373968	intracellular membrane-bound organelle
GO:0031090	377	1	0.992178759215207	organelle membrane
GO:0005575	8637	108	1	cellular_component

25. MedullaOblongata

25.1 MedullaOblongata selective genes (34 genes)

Symbol	Description	Chromosome	Chromosome Location	LocusLink	Cytoband	UniGene
ARPP-21	NA	3	35696119, 35658852, 35697432	10777	3p22.3	Hs.475902
PLP1	proteolipid protein 1 (Pelizaeus-Merzbacher disease, spastic paraplegia 2, uncomplicated)	X	102918409	5354	Xq22	Hs.1787
TMEM16C	transmembrane protein 16C	11	26309546	63982	11p14.2	Hs.91791
GRIA2	glutamate receptor, ionotropic, AMPA 2	4	158361296	2891	4q32-q33	Hs.32763
SNAP25	synaptosomal-associated protein, 25kDa	20	10147476	6616	20p12-p11.2	Hs.167317
DIRAS2	DIRAS family, GTP-binding RAS-like 2	9	-92411933	54769	9q22.2	Hs.165636
PCP4	Purkinje cell protein 4	21	40161216	5121	21q22.2	Hs.80296
GPM6A	glycoprotein M6A	4	-176791082	2823	4q34	Hs.75819
RTN1	reticulon 1	14	-59132446	6252	14q23.1	Hs.368626
SYT1	synaptotagmin I	12	78135413	6857	12cen-q21	Hs.310545
GFAP	glial fibrillary acidic protein	17	-40338518	2670	17q21	Hs.514227
GPR88	G protein-coupled receptor 88	1	100776280	54112	1p21.3	Hs.170053
GPR37	G protein-coupled receptor 37 (endothelin receptor type B-like)	7	-124173351	2861	7q31	Hs.406094
TAC1	tachykinin, precursor 1 (substance K, substance P, neurokinin 1, neurokinin 2, neuromedin L, neurokinin alpha, neuropeptide K, neuropeptide gamma)	7	97199310	6863	7q21-q22	Hs.2563
CCK	cholecystokinin	3	-42274321	885	3p22-p21.3	Hs.458426
STMN2	stathmin-like 2	8	80685934	11075	8q21.13	Hs.521651
VSNL1	visinin-like 1	2	17585287	7447	2p24.3	Hs.444212
MYT1L	myelin transcription factor 1-like	2	-1771891	23040	2p25.3	Hs.434418
DCAMKL1	doublecortin and CaM kinase-like 1	13	-35243477	9201	13q13	Hs.507755
MT3	metallothionein 3 (growth inhibitory factor (neurotrophic))	16	55180767	4504	16q13	Hs.73133
SCG5	secretogranin V (7B2 protein)	15	30721251	6447	15q13-q14	Hs.156540
DNM1	dynamin 1	9	130005483	1759	9q34	Hs.522413
KIAA1598	KIAA1598	10	-118634297	57698	10q25.3	Hs.501140
SPP1	secreted phosphoprotein 1 (osteopontin, bone sialoprotein I, early T-lymphocyte activation 1)	4	89115825	6696	4q21-q25	Hs.313
NAP1L3	nucleosome assembly protein 1-like 3	X	-92812589	4675	Xq21.3-q22	Hs.21365
TF	transferrin	3	134947924	7018	3q22.1	Hs.518267
GPM6B	glycoprotein M6B	X	-27401000	2824	Xp22.2	Hs.495710
S100B	S100 calcium binding protein, beta (neural)	21	-46842958	6285	21q22.3	Hs.422181
NRGN	neurogranin (protein kinase C substrate, RC3)	11	124114951	4900	11q24	Hs.524116
NEF3	neurofilament 3 (150kDa medium)	8	24827211	4741	8p21	Hs.458657 Hs.615287

CAMK2B	calcium/calmodulin-dependent protein kinase (CaM kinase) II beta	7	-44225421	816	22q12|7p14.3-p14.1	Hs.351887
NEFH	neurofilament, heavy polypeptide 200kDa	22	28206218	4744	22q12.2	Hs.198760
HPCA	hippocalcin	1	33124684	3208	1p35-p34.2	Hs.632391
CHN1	chimerin (chimaerin) 1	2	-175372337	1123	2q31-q32.1	Hs.380138

25.2 GO: Biological Process

GO ID	genes present in chip	genes present in tissues	p-value	GO term
GO:0007399	385	8	8.05E-06	neurogenesis
GO:0007268	192	5	0.00018846	synaptic transmission
GO:0019226	198	5	0.000217592	transmission of nerve impulse
GO:0046883	8	2	0.000220766	regulation of hormone secretion
GO:0007154	2730	17	0.000222218	cell communication
GO:0007267	465	7	0.000256226	cell-cell signaling
GO:0009887	807	9	0.000268395	organogenesis
GO:0046879	11	2	0.000431349	hormone secretion
GO:0048513	872	9	0.000480507	organ development
GO:0030182	13	2	0.000609568	neuron differentiation
GO:0050767	15	2	0.000817671	regulation of neurogenesis
GO:0030154	409	6	0.000883317	cell differentiation
GO:0007269	19	2	0.001322242	neurotransmitter secretion
GO:0045055	22	2	0.001776733	regulated secretory pathway
GO:0009653	1051	9	0.001869991	morphogenesis
GO:0051046	24	2	0.002115355	regulation of secretion
GO:0007275	1543	11	0.002199702	development
GO:0006875	93	3	0.002314572	metal ion homeostasis
GO:0030003	98	3	0.002687149	cation homeostasis
GO:0048151	1	1	0.002878654	hyperphosphorylation
GO:0019430	1	1	0.002878654	removal of superoxide radicals
GO:0048143	1	1	0.002878654	astrocyte activation
GO:0045917	1	1	0.002878654	positive regulation of complement activation
GO:0030449	1	1	0.002878654	regulation of complement activation
GO:0007417	101	3	0.002927508	central nervous system development
GO:0006873	103	3	0.003094899	cell ion homeostasis
GO:0007165	2168	13	0.00352683	signal transduction
GO:0050801	111	3	0.003823103	ion homeostasis
GO:0019725	122	3	0.004983225	cell homeostasis
GO:0048169	2	1	0.005749339	regulation of long-term neuronal synaptic plasticity
GO:0016081	2	1	0.005749339	synaptic vesicle docking during exocytosis
GO:0050774	2	1	0.005749339	negative regulation of dendrite morphogenesis
GO:0001505	41	2	0.006098881	regulation of neurotransmitter levels
GO:0031175	46	2	0.007630414	neurite morphogenesis
GO:0006334	48	2	0.008286788	nucleosome assembly
GO:0030502	3	1	0.008612076	negative regulation of bone mineralization
GO:0017157	3	1	0.008612076	regulation of exocytosis
GO:0050796	3	1	0.008612076	regulation of insulin secretion
GO:0050773	3	1	0.008612076	regulation of dendrite morphogenesis
GO:0007320	3	1	0.008612076	insemination
GO:0030279	3	1	0.008612076	negative regulation of ossification
GO:0046851	3	1	0.008612076	negative regulation of bone remodeling
GO:0016192	296	4	0.009560304	vesicle-mediated transport

GO:0042592	157	3	0.009992003	homeostasis
GO:0050877	475	5	0.010344301	neurophysiological process
GO:0006887	55	2	0.01077581	exocytosis
GO:0031497	56	2	0.011155263	chromatin assembly
GO:0007218	56	2	0.011155263	neuropeptide signaling pathway
GO:0001504	4	1	0.011466886	neurotransmitter uptake
GO:0048168	4	1	0.011466886	regulation of neuronal synaptic plasticity
GO:0050768	4	1	0.011466886	negative regulation of neurogenesis
GO:0016486	4	1	0.011466886	peptide hormone processing
GO:0050793	168	3	0.012005016	regulation of development
GO:0019098	5	1	0.014313792	reproductive behavior
GO:0005513	5	1	0.014313792	calcium ion sensing
GO:0016358	5	1	0.014313792	dendrite morphogenesis
GO:0007217	5	1	0.014313792	tachykinin signaling pathway
GO:0030073	5	1	0.014313792	insulin secretion
GO:0050926	5	1	0.014313792	regulation of positive chemotaxis
GO:0050927	5	1	0.014313792	positive regulation of positive chemotaxis
GO:0050918	5	1	0.014313792	positive chemotaxis
GO:0050930	5	1	0.014313792	induction of positive chemotaxis
GO:0007617	5	1	0.014313792	mating behavior
GO:0016079	6	1	0.017152813	synaptic vesicle exocytosis
GO:0030072	6	1	0.017152813	peptide hormone secretion
GO:0009593	6	1	0.017152813	detection of chemical substance
GO:0050921	6	1	0.017152813	positive regulation of chemotaxis
GO:0030500	7	1	0.01998397	regulation of bone mineralization
GO:0048167	7	1	0.01998397	regulation of synaptic plasticity
GO:0050920	7	1	0.01998397	regulation of chemotaxis
GO:0050776	77	2	0.020427171	regulation of immune response
GO:0046903	208	3	0.021193574	secretion
GO:0030278	8	1	0.022807285	regulation of ossification
GO:0046850	8	1	0.022807285	regulation of bone remodeling
GO:0030005	87	2	0.02566306	di-, tri-valent inorganic cation homeostasis
GO:0006333	91	2	0.027895402	chromatin assembly or disassembly
GO:0030282	10	1	0.02843047	bone mineralization
GO:0048489	11	1	0.031230383	synaptic vesicle transport
GO:0051260	11	1	0.031230383	protein homooligomerization
GO:0045321	97	2	0.031385729	immune cell activation
GO:0001775	98	2	0.031983616	cell activation
GO:0042102	12	1	0.034022535	positive regulation of T cell proliferation
GO:0030595	13	1	0.036806949	immune cell chemotaxis
GO:0009582	109	2	0.038853787	detection of abiotic stimulus
GO:0006826	14	1	0.039583645	iron ion transport
GO:0001666	14	1	0.039583645	response to hypoxia
GO:0050900	14	1	0.039583645	immune cell migration
GO:0007610	269	3	0.040986852	behavior
GO:0006801	15	1	0.042352643	superoxide metabolism
GO:0042129	15	1	0.042352643	regulation of T cell proliferation

GO:0006879	15	1	0.042352643	iron ion homeostasis
GO:0045637	16	1	0.045113964	regulation of myeloid cell differentiation
GO:0006904	17	1	0.047867629	vesicle docking during exocytosis
GO:0042098	17	1	0.047867629	T cell proliferation
GO:0007416	17	1	0.047867629	synaptogenesis
GO:0007611	17	1	0.047867629	learning and/or memory
GO:0007186	491	4	0.049886478	G-protein coupled receptor protein signaling pathway
GO:0048278	18	1	0.050613656	vesicle docking
GO:0050808	18	1	0.050613656	synapse organization and biogenesis
GO:0050671	18	1	0.050613656	positive regulation of lymphocyte proliferation
GO:0051239	133	2	0.055571	regulation of organismal physiological process
GO:0046916	20	1	0.056082885	transition metal ion homeostasis
GO:0051241	20	1	0.056082885	negative regulation of organismal physiological process
GO:0050870	21	1	0.058806125	positive regulation of T cell activation
GO:0050670	22	1	0.061521811	regulation of lymphocyte proliferation
GO:0016197	22	1	0.061521811	endosome transport
GO:0009628	318	3	0.061852492	response to abiotic stimulus
GO:0042088	24	1	0.066930596	T-helper 1 type immune response
GO:0007032	24	1	0.066930596	endosome organization and biogenesis
GO:0051259	24	1	0.066930596	protein oligomerization
GO:0045792	25	1	0.069623736	negative regulation of cell size
GO:0042087	25	1	0.069623736	cell-mediated immune response
GO:0030308	25	1	0.069623736	negative regulation of cell growth
GO:0016066	27	1	0.07498761	cellular defense response (sensu Vertebrata)
GO:0046651	28	1	0.077658384	lymphocyte proliferation
GO:0051251	28	1	0.077658384	positive regulation of lymphocyte activation
GO:0006898	28	1	0.077658384	receptor mediated endocytosis
GO:0045045	161	2	0.077673079	secretory pathway
GO:0050867	29	1	0.080321743	positive regulation of cell activation
GO:0045926	29	1	0.080321743	negative regulation of growth
GO:0006956	30	1	0.082977706	complement activation
GO:0000041	31	1	0.085626293	transition metal ion transport
GO:0050863	31	1	0.085626293	regulation of T cell activation
GO:0006836	33	1	0.090901417	neurotransmitter transport
GO:0042035	33	1	0.090901417	regulation of cytokine biosynthesis
GO:0006112	34	1	0.093527994	energy reserve metabolism
GO:0043062	35	1	0.096147273	extracellular structure organization and biogenesis
GO:0030198	35	1	0.096147273	extracellular matrix organization and biogenesis
GO:0042089	36	1	0.098759274	cytokine biosynthesis
GO:0006325	186	2	0.09936995	establishment and/or maintenance of chromatin architecture
GO:0016485	37	1	0.101364016	protein processing
GO:0042107	37	1	0.101364016	cytokine metabolism
GO:0030099	37	1	0.101364016	myeloid cell differentiation
GO:0001503	38	1	0.103961518	ossification
GO:0031214	38	1	0.103961518	biomineral formation
GO:0006323	193	2	0.1057285	DNA packaging
GO:0001817	39	1	0.106551801	regulation of cytokine production

GO:0006461	194	2	0.106646097	protein complex assembly
GO:0007409	40	1	0.109134882	axonogenesis
GO:0051249	41	1	0.111710782	regulation of lymphocyte activation
GO:0046849	41	1	0.111710782	bone remodeling
GO:0001816	42	1	0.11427952	cytokine production
GO:0050865	43	1	0.116841114	regulation of cell activation
GO:0042110	45	1	0.121942949	T cell activation
GO:0051049	46	1	0.124483228	regulation of transport
GO:0042445	46	1	0.124483228	hormone metabolism
GO:0006810	1802	8	0.129571918	transport
GO:0051093	49	1	0.132061735	negative regulation of development
GO:0007160	49	1	0.132061735	cell-matrix adhesion
GO:0007001	221	2	0.132210174	chromosome organization and biogenesis (sensu Eukaryota)
GO:0050874	1531	7	0.137662495	organismal physiological process
GO:0051234	1841	8	0.142275348	establishment of localization
GO:0051276	236	2	0.146988986	chromosome organization and biogenesis
GO:0045595	55	1	0.14702996	regulation of cell differentiation
GO:0051179	1857	8	0.147679444	localization
GO:0050778	56	1	0.149500409	positive regulation of immune response
GO:0008283	473	3	0.152852876	cell proliferation
GO:0051240	59	1	0.156870546	positive regulation of organismal physiological process
GO:0006800	61	1	0.161749811	oxygen and reactive oxygen species metabolism
GO:0048609	61	1	0.161749811	reproductive organismal physiological process
GO:0050876	62	1	0.164179251	reproductive physiological process
GO:0006874	68	1	0.178614331	calcium ion homeostasis
GO:0030001	269	2	0.18062593	metal ion transport
GO:0016310	522	3	0.187744689	phosphorylation
GO:0046649	81	1	0.209072547	lymphocyte activation
GO:0016477	85	1	0.218223751	cell migration
GO:0042981	306	2	0.219645702	regulation of apoptosis
GO:0016043	842	4	0.219788135	cell organization and biogenesis
GO:0043067	308	2	0.221781967	regulation of programmed cell death
GO:0006968	87	1	0.222761088	cellular defense response
GO:0030097	87	1	0.222761088	hemopoiesis
GO:0048534	88	1	0.225020251	hemopoietic or lymphoid organ development
GO:0009581	313	2	0.227131728	detection of external stimulus
GO:0001558	94	1	0.23844321	regulation of cell growth
GO:0007242	879	4	0.243220418	intracellular signaling cascade
GO:0015674	99	1	0.249457976	di-, tri-valent inorganic cation transport
GO:0006935	101	1	0.253820852	chemotaxis
GO:0042330	101	1	0.253820852	taxis
GO:0040008	104	1	0.260319472	regulation of growth
GO:0006812	346	2	0.262675816	cation transport
GO:0006417	107	1	0.266763654	regulation of protein biosynthesis
GO:0006813	111	1	0.275271969	potassium ion transport
GO:0031326	111	1	0.275271969	regulation of cellular biosynthesis
GO:0006916	112	1	0.277384168	anti-apoptosis

GO:0001501	113	1	0.279490447	skeletal development
GO:0006793	641	3	0.279731227	phosphorus metabolism
GO:0006796	641	3	0.279731227	phosphate metabolism
GO:0006897	115	1	0.28368531	endocytosis
GO:0009889	116	1	0.285773925	regulation of biosynthesis
GO:0007166	952	4	0.291185758	cell surface receptor linked signal transduction
GO:0008284	121	1	0.296129471	positive regulation of cell proliferation
GO:0009605	963	4	0.298568802	response to external stimulus
GO:0008361	123	1	0.300231134	regulation of cell size
GO:0016049	123	1	0.300231134	cell growth
GO:0043066	128	1	0.310384991	negative regulation of apoptosis
GO:0043069	129	1	0.312398692	negative regulation of programmed cell death
GO:0015980	132	1	0.318405927	energy derivation by oxidation of organic compounds
GO:0006996	693	3	0.321711398	organelle organization and biogenesis
GO:0051242	411	2	0.332846401	positive regulation of cellular physiological process
GO:0006917	140	1	0.334179532	induction of apoptosis
GO:0012502	140	1	0.334179532	induction of programmed cell death
GO:0006959	141	1	0.336126376	humoral immune response
GO:0043119	428	2	0.351017076	positive regulation of physiological process
GO:0040007	154	1	0.36094253	growth
GO:0043065	155	1	0.362814066	positive regulation of apoptosis
GO:0043068	156	1	0.364680331	positive regulation of programmed cell death
GO:0006468	444	2	0.36799194	protein amino acid phosphorylation
GO:0009888	158	1	0.368397106	histogenesis
GO:0006091	446	2	0.370103923	generation of precursor metabolites and energy
GO:0046907	448	2	0.372213565	intracellular transport
GO:0009613	458	2	0.382725196	response to pest, pathogen or parasite
GO:0043207	463	2	0.387957068	response to external biotic stimulus
GO:0006457	169	1	0.388468573	protein folding
GO:0006915	471	2	0.396292914	apoptosis
GO:0051246	174	1	0.397387873	regulation of protein metabolism
GO:0012501	473	2	0.398369881	programmed cell death
GO:0044267	2109	7	0.405757439	cellular protein metabolism
GO:0048522	482	2	0.407680194	positive regulation of cellular process
GO:0042221	183	1	0.413128354	response to chemical substance
GO:0000902	183	1	0.413128354	cellular morphogenesis
GO:0006259	489	2	0.414879325	DNA metabolism
GO:0019538	2129	7	0.416346978	protein metabolism
GO:0006954	185	1	0.416572157	inflammatory response
GO:0008219	498	2	0.424078583	cell death
GO:0016265	501	2	0.427130361	death
GO:0006811	515	2	0.441271367	ion transport
GO:0006928	205	1	0.449958427	cell motility
GO:0040011	205	1	0.449958427	locomotion
GO:0000003	208	1	0.454805128	reproduction
GO:0007626	209	1	0.456411549	locomotory behavior
GO:0006950	881	3	0.472560581	response to stress

GO:0015672	223	1	0.478429107	monovalent inorganic cation transport
GO:0007264	227	1	0.48456066	small GTPase mediated signal transduction
GO:0050896	1578	5	0.485824828	response to stimulus
GO:0048518	565	2	0.490306141	positive regulation of biological process
GO:0044260	2298	7	0.505681312	cellular macromolecule metabolism
GO:0051243	583	2	0.507350819	negative regulation of cellular physiological process
GO:0043118	602	2	0.524970711	negative regulation of physiological process
GO:0006118	256	1	0.526986976	electron transport
GO:0006886	257	1	0.528388336	intracellular protein transport
GO:0042127	265	1	0.539456404	regulation of cell proliferation
GO:0048523	640	2	0.559021	negative regulation of cellular process
GO:0043170	2405	7	0.560877284	macromolecule metabolism
GO:0050791	2407	7	0.561891052	regulation of physiological process
GO:0009987	8200	24	0.565954634	cellular process
GO:0006955	684	2	0.596396035	immune response
GO:0048519	688	2	0.599682438	negative regulation of biological process
GO:0006464	1087	3	0.620989434	protein modification
GO:0009611	341	1	0.632872417	response to wounding
GO:0006952	751	2	0.648970153	defense response
GO:0043412	1160	3	0.666973792	biopolymer modification
GO:0050789	2689	7	0.69500848	regulation of biological process
GO:0006412	407	1	0.698965899	protein biosynthesis
GO:0009607	857	2	0.721496255	response to biotic stimulus
GO:0015031	439	1	0.72673561	protein transport
GO:0045184	459	1	0.742822508	establishment of protein localization
GO:0009059	464	1	0.746699635	macromolecule biosynthesis
GO:0000004	465	1	0.747468287	biological process unknown
GO:0008104	474	1	0.754285967	protein localization
GO:0007155	518	1	0.785147697	cell adhesion
GO:0043283	2240	5	0.809859659	biopolymer metabolism
GO:0051244	2319	5	0.83582615	regulation of cellular physiological process
GO:0050794	2488	5	0.882380995	regulation of cellular process
GO:0044249	763	1	0.899549795	cellular biosynthesis
GO:0009058	859	1	0.925887563	biosynthesis
GO:0031323	1566	2	0.95453796	regulation of cellular metabolism
GO:0019222	1629	2	0.96202737	regulation of metabolism
GO:0006139	2328	3	0.978929277	nucleobase, nucleoside, nucleotide and nucleic acid metabolism
GO:0006355	1359	1	0.985680116	regulation of transcription, DNA-dependent
GO:0006351	1407	1	0.987839139	transcription, DNA-dependent
GO:0045449	1433	1	0.988874171	regulation of transcription
GO:0019219	1458	1	0.989789167	regulation of nucleobase, nucleoside, nucleotide and nucleic acid metabolism
GO:0044237	5026	9	0.990862238	cellular metabolism
GO:0006350	1499	1	0.991135202	transcription
GO:0044238	4820	8	0.994298563	primary metabolism
GO:0008152	5262	9	0.995767316	metabolism
GO:0050875	7018	14	0.998257101	cellular physiological process
GO:0007582	7846	15	0.999959325	physiological process

GO:0008150	9032	26	1 biological_process

25.3 GO: Molecular Function

GO ID	genes present in chip	genes present in tissues	p-value	GO term
GO:0016526	13	2	0.000681541629119627	G-protein coupled receptor activity, unknown ligand
GO:0048154	1	1	0.00303951367781119	S100 beta binding
GO:0017075	2	1	0.00607010635111804	syntaxin-1 binding
GO:0048155	2	1	0.00607010635111804	S100 alpha binding
GO:0048156	2	1	0.00607010635111804	tau protein binding
GO:0004971	3	1	0.00909180327069248	alpha-amino-3-hydroxy-5-methyl-4-isoxazole propionate selective glutamate receptor activity
GO:0008199	6	1	0.0181037709833499	ferric iron binding
GO:0050750	6	1	0.0181037709833499	low-density lipoprotein receptor binding
GO:0019905	6	1	0.0181037709833499	syntaxin binding
GO:0000149	6	1	0.0181037709833499	SNARE binding
GO:0015277	7	1	0.0210901360217846	kainate selective glutamate receptor activity
GO:0005545	9	1	0.0270365793528925	phosphatidylinositol binding
GO:0005200	86	2	0.0278523469169662	structural constituent of cytoskeleton
GO:0004685	10	1	0.0299967072587636	calcium- and calmodulin-dependent protein kinase activity
GO:0005179	90	2	0.0302929844792531	hormone activity
GO:0005525	226	3	0.0304646739200992	GTP binding
GO:0019001	235	3	0.0336524233644576	guanyl nucleotide binding
GO:0004871	1837	10	0.0388508975959020	signal transducer activity
GO:0004970	14	1	0.0417505028196800	ionotropic glutamate receptor activity
GO:0005234	14	1	0.0417505028196800	glutamate-gated ion channel activity
GO:0005516	112	2	0.0451335406547327	calmodulin binding
GO:0004683	17	1	0.0504754837262471	calmodulin regulated protein kinase activity
GO:0005198	488	4	0.058325426602792	structural molecule activity
GO:0005184	21	1	0.061989551503101	neuropeptide hormone activity
GO:0005554	522	4	0.0712027130691001	molecular function unknown
GO:0005102	528	4	0.0736192688171143	receptor binding
GO:0005178	26	1	0.0761926638941756	integrin binding
GO:0019210	29	1	0.0846146215346678	kinase inhibitor activity
GO:0008066	29	1	0.0846146215346678	glutamate receptor activity
GO:0016209	31	1	0.090188023666786	antioxidant activity
GO:0004857	187	2	0.109984464525661	enzyme inhibitor activity
GO:0008047	188	2	0.110964034087942	enzyme activator activity
GO:0005231	39	1	0.112155776825312	excitatory extracellular ligand-gated ion channel activity
GO:0005070	40	1	0.114865467676929	SH3/SH2 adaptor activity
GO:0019992	42	1	0.120260915226159	diacylglycerol binding
GO:0005509	646	4	0.129443240385883	calcium ion binding
GO:0005506	212	2	0.135142349256722	iron ion binding
GO:0005507	49	1	0.138896043103093	copper ion binding
GO:0005230	57	1	0.159726744012628	extracellular ligand-gated ion channel activity
GO:0019207	58	1	0.162296116052236	kinase regulator activity
GO:0042803	64	1	0.177553786608120	protein homodimerization activity
GO:0030674	69	1	0.190063225349034	protein binding, bridging
GO:0005515	3709	14	0.196866598922854	protein binding

GO:0030234	509	3	0.199581501724651	enzyme regulator activity
GO:0008092	279	2	0.207606151234539	cytoskeletal protein binding
GO:0001584	282	2	0.210964521268529	rhodopsin-like receptor activity
GO:0015276	84	1	0.226499100989747	ligand-gated ion channel activity
GO:0008289	298	2	0.228974427142830	lipid binding
GO:0005267	90	1	0.240625844988615	potassium channel activity
GO:0003774	98	1	0.259074497115236	motor activity
GO:0004674	326	2	0.260765480058330	protein serine/threonine kinase activity
GO:0009055	104	1	0.272626217410098	electron carrier activity
GO:0004930	349	2	0.286984546852706	G-protein coupled receptor activity
GO:0005096	117	1	0.301172210817654	GTPase activator activity
GO:0003924	121	1	0.309735814006717	GTPase activity
GO:0051082	127	1	0.322391522212158	unfolded protein binding
GO:0017076	1248	5	0.327497013378844	purine nucleotide binding
GO:0035091	132	1	0.332766535362573	phosphoinositide binding
GO:0005057	132	1	0.332766535362573	receptor signaling protein activity
GO:0004888	694	3	0.353942382966308	transmembrane receptor activity
GO:0008083	144	1	0.357044624079075	growth factor activity
GO:0005489	152	1	0.372753761610111	electron transporter activity
GO:0042802	158	1	0.384291749692265	protein self binding
GO:0004713	161	1	0.389983629142896	protein-tyrosine kinase activity
GO:0004672	447	2	0.396969339388289	protein kinase activity
GO:0005543	169	1	0.404914707933112	phospholipid binding
GO:0043169	2313	8	0.405480259344956	cation binding
GO:0046983	170	1	0.406756094346891	protein dimerization activity
GO:0005125	181	1	0.426651682086667	cytokine activity
GO:0005261	188	1	0.438975408937542	cation channel activity
GO:0003779	189	1	0.440714927075124	actin binding
GO:0000166	1432	5	0.445119795551733	nucleotide binding
GO:0016773	532	2	0.486512624031001	phosphotransferase activity, alcohol group as acceptor
GO:0030695	219	1	0.49054498551386	GTPase regulator activity
GO:0046872	2495	8	0.500463430648567	metal ion binding
GO:0043167	2556	8	0.531872339848995	ion binding
GO:0005216	252	1	0.540405765933189	ion channel activity
GO:0046914	1596	5	0.547367779592636	transition metal ion binding
GO:0016301	609	2	0.560709547912966	kinase activity
GO:0008270	1287	4	0.562639170434757	zinc ion binding
GO:0015268	274	1	0.570992169101039	alpha-type channel activity
GO:0015267	284	1	0.584236323960255	channel or pore class transporter activity
GO:0005215	1030	3	0.6196628237625	transporter activity
GO:0004872	1060	3	0.639937573233386	receptor activity
GO:0016772	708	2	0.645090459800396	transferase activity, transferring phosphorus-containing groups
GO:0005386	398	1	0.709902636880683	carrier activity
GO:0017111	410	1	0.720760114511073	nucleoside-triphosphatase activity
GO:0008324	421	1	0.73036734853351	cation transporter activity
GO:0016462	430	1	0.737989687376292	pyrophosphatase activity
GO:0016818	432	1	0.739655063779965	hydrolase activity, acting on acid anhydrides, in phosphorus-containing anhydrides

GO:0016817	433	1	0.74048391471315	hydrolase activity, acting on acid anhydrides
GO:0005488	7228	21	0.745126233809146	binding
GO:0015075	523	1	0.805508900438193	ion transporter activity
GO:0005524	970	2	0.809308864429156	ATP binding
GO:0030554	1028	2	0.835392386369224	adenyl nucleotide binding
GO:0003700	649	1	0.870756249373799	transcription factor activity
GO:0016740	1169	2	0.88633168776632	transferase activity
GO:0003677	1384	2	0.937394395478687	DNA binding
GO:0030528	963	1	0.954512386201661	transcription regulator activity
GO:0003676	1990	3	0.958509308638788	nucleic acid binding
GO:0016787	1424	1	0.990858895368779	hydrolase activity
GO:0003824	3409	3	0.99962014788581	catalytic activity
GO:0003674	9541	29	1	molecular_function

25.4 GO: Cellular Component

GO ID	genes present in chip	genes present in tissues	p-value	GO term
GO:0005883	5	2	9.35717573388069e-05	neurofilament
GO:0030424	7	2	0.000195743290159885	axon
GO:0016023	122	4	0.000517163301917347	cytoplasmic membrane-bound vesicle
GO:0031410	124	4	0.000550012139026646	cytoplasmic vesicle
GO:0045111	65	3	0.00104595220533430	intermediate filament cytoskeleton
GO:0005882	65	3	0.00104595220533430	intermediate filament
GO:0043005	17	2	0.00124345015854855	neuron projection
GO:0008021	27	2	0.00314797806775432	synaptic vesicle
GO:0030136	65	2	0.0173430136195965	clathrin-coated vesicle
GO:0005678	6	1	0.0186158831271888	chromatin assembly complex
GO:0042995	70	2	0.0199446741960584	cell projection
GO:0005576	949	7	0.0234254259373103	extracellular region
GO:0030135	79	2	0.0250113883567992	coated vesicle
GO:0030139	12	1	0.0368978197670319	endocytic vesicle
GO:0045202	98	2	0.0372130251206481	synapse
GO:0030141	18	1	0.0548515740923388	secretory granule
GO:0019717	19	1	0.057812359276177	synaptosome
GO:0005578	303	3	0.0672464166305052	extracellular matrix (sensu Metazoa)
GO:0031012	304	3	0.0677777908762573	extracellular matrix
GO:0016585	23	1	0.0695664336540501	chromatin remodeling complex
GO:0030176	26	1	0.0782891913606176	integral to endoplasmic reticulum membrane
GO:0031227	32	1	0.0954990691204732	intrinsic to endoplasmic reticulum membrane
GO:0005625	183	2	0.111058253002636	soluble fraction
GO:0005905	38	1	0.112399296666881	coated pit
GO:0000786	40	1	0.117964823837354	nucleosome
GO:0005856	612	4	0.120174189518068	cytoskeleton
GO:0009986	61	1	0.174410371539697	cell surface
GO:0045211	66	1	0.187327896202612	postsynaptic membrane
GO:0031301	76	1	0.212581548935726	integral to organelle membrane
GO:0005789	84	1	0.232238690898184	endoplasmic reticulum membrane
GO:0031300	90	1	0.246670547777276	intrinsic to organelle membrane
GO:0042175	90	1	0.246670547777276	nuclear envelope-endoplasmic reticulum network
GO:0000785	100	1	0.270144973216861	chromatin
GO:0016020	3514	13	0.273863446820201	membrane
GO:0016021	2674	10	0.310257106357837	integral to membrane
GO:0031224	2681	10	0.313554205807898	intrinsic to membrane
GO:0005615	385	2	0.340386974825891	extracellular space
GO:0005874	132	1	0.340623800910757	microtubule
GO:0043228	977	4	0.365370396736305	non-membrane-bound organelle
GO:0043232	977	4	0.365370396736305	intracellular non-membrane-bound organelle
GO:0000267	698	3	0.374458723839411	cell fraction
GO:0015630	191	1	0.453764026647797	microtubule cytoskeleton
GO:0005694	203	1	0.474367035812638	chromosome

GO:0005624	521	2	0.490829469252394	membrane fraction
GO:0008372	523	2	0.492899427367365	cellular component unknown
GO:0005886	1472	5	0.497717100277022	plasma membrane
GO:0005654	221	1	0.503874706258403	nucleoplasm
GO:0012505	246	1	0.542223877786968	endomembrane system
GO:0005887	1057	3	0.658913512388485	integral to plasma membrane
GO:0031226	1062	3	0.662221516167226	intrinsic to plasma membrane
GO:0031090	377	1	0.700873276309113	organelle membrane
GO:0005623	7802	24	0.738580840629743	cell
GO:0005783	483	1	0.789058222152118	endoplasmic reticulum
GO:0043226	4515	12	0.843451317642554	organelle
GO:0043229	4515	12	0.843451317642554	intracellular organelle
GO:0005737	2530	6	0.846733436838523	cytoplasm
GO:0043234	1247	2	0.917844678691263	protein complex
GO:0043227	3955	9	0.934166425717256	membrane-bound organelle
GO:0043231	3955	9	0.934166425717256	intracellular membrane-bound organelle
GO:0005622	5419	13	0.959693976606447	intracellular
GO:0005634	2704	4	0.985770219199574	nucleus
GO:0005575	8637	27	1	cellular_component

26. Pons

26.1 Pons selective genes (16 genes)

Symbol	Description	Chromosome	Chromosome Location	LocusLink	Cytoband	UniGene
ARPP-21	NA	3	35696119, 35658852, 35697432	10777	3p22.3	Hs.475902
PLP1	proteolipid protein 1 (Pelizaeus-Merzbacher disease, spastic paraplegia 2, uncomplicated)	X	102918409	5354	Xq22	Hs.1787
SNAP25	synaptosomal-associated protein, 25kDa	20	10147476	6616	20p12-p11.2	Hs.167317
PCP4	Purkinje cell protein 4	21	40161216	5121	21q22.2	Hs.80296
GRIA2	glutamate receptor, ionotropic, AMPA 2	4	158361296	2891	4q32-q33	Hs.32763
TMEM16C	transmembrane protein 16C	11	26309546	63982	11p14.2	Hs.91791
IL6	interleukin 6 (interferon, beta 2)	7	22733344	3569	7p21	Hs.512234
GPM6A	glycoprotein M6A	4	-176791082	2823	4q34	Hs.75819
GFAP	glial fibrillary acidic protein	17	-40338518	2670	17q21	Hs.514227
RTN1	reticulon 1	14	-59132446	6252	14q23.1	Hs.368626
STMN2	stathmin-like 2	8	80685934	11075	8q21.13	Hs.521651
DIRAS2	DIRAS family, GTP-binding RAS-like 2	9	-92411933	54769	9q22.2	Hs.165636
CCK	cholecystokinin	3	-42274321	885	3p22-p21.3	Hs.458426
MT3	metallothionein 3 (growth inhibitory factor (neurotrophic))	16	55180767	4504	16q13	Hs.73133
GPR88	G protein-coupled receptor 88	1	100776280	54112	1p21.3	Hs.170053
VSNL1	visinin-like 1	2	17585287	7447	2p24.3	Hs.444212

26.2 GO: Biological Process

GO ID	genes present in chip	genes present in tissues	p-value	GO term
GO:0030182	13	2	0.000104422	neuron differentiation
GO:0019430	1	1	0.001217892	removal of superoxide radicals
GO:0001780	1	1	0.001217892	neutrophil homeostasis
GO:0001781	1	1	0.001217892	programmed cell death, neutrophils
GO:0016081	2	1	0.002434435	synaptic vesicle docking during exocytosis
GO:0050774	2	1	0.002434435	negative regulation of dendrite morphogenesis
GO:0001776	2	1	0.002434435	immune cell homeostasis
GO:0050796	3	1	0.003649631	regulation of insulin secretion
GO:0050773	3	1	0.003649631	regulation of dendrite morphogenesis
GO:0045079	3	1	0.003649631	negative regulation of chemokine biosynthesis
GO:0007154	2730	8	0.004496674	cell communication
GO:0001504	4	1	0.004863482	neurotransmitter uptake
GO:0050768	4	1	0.004863482	negative regulation of neurogenesis
GO:0045073	4	1	0.004863482	regulation of chemokine biosynthesis
GO:0007165	2168	7	0.005912027	signal transduction
GO:0042033	5	1	0.006075987	chemokine biosynthesis
GO:0050755	5	1	0.006075987	chemokine metabolism
GO:0016358	5	1	0.006075987	dendrite morphogenesis
GO:0030073	5	1	0.006075987	insulin secretion
GO:0001894	5	1	0.006075987	tissue homeostasis
GO:0016079	6	1	0.00728715	synaptic vesicle exocytosis
GO:0030072	6	1	0.00728715	peptide hormone secretion
GO:0046883	8	1	0.009705451	regulation of hormone secretion
GO:0001818	9	1	0.010912592	negative regulation of cytokine production
GO:0042036	9	1	0.010912592	negative regulation of cytokine biosynthesis
GO:0048489	11	1	0.013322861	synaptic vesicle transport
GO:0046879	11	1	0.013322861	hormone secretion
GO:0042592	157	2	0.014898064	homeostasis
GO:0007267	465	3	0.016411238	cell-cell signaling
GO:0001666	14	1	0.016928256	response to hypoxia
GO:0006801	15	1	0.018127389	superoxide metabolism
GO:0050767	15	1	0.018127389	regulation of neurogenesis
GO:0050777	16	1	0.019325193	negative regulation of immune response
GO:0006904	17	1	0.020521669	vesicle docking during exocytosis
GO:0017148	17	1	0.020521669	negative regulation of protein biosynthesis
GO:0031327	18	1	0.021716817	negative regulation of cellular biosynthesis
GO:0048278	18	1	0.021716817	vesicle docking
GO:0007268	192	2	0.021794232	synaptic transmission
GO:0007269	19	1	0.02291064	neurotransmitter secretion
GO:0009890	19	1	0.02291064	negative regulation of biosynthesis
GO:0019226	198	2	0.023089121	transmission of nerve impulse
GO:0051241	20	1	0.024103137	negative regulation of organismal physiological process
GO:0045055	22	1	0.026484165	regulated secretory pathway

GO:0006953	23	1	0.027672697	acute-phase response
GO:0051046	24	1	0.02885991	regulation of secretion
GO:0045792	25	1	0.030045804	negative regulation of cell size
GO:0030308	25	1	0.030045804	negative regulation of cell growth
GO:0045926	29	1	0.034776231	negative regulation of growth
GO:0006836	33	1	0.039485675	neurotransmitter transport
GO:0042035	33	1	0.039485675	regulation of cytokine biosynthesis
GO:0042089	36	1	0.043004039	cytokine biosynthesis
GO:0042107	37	1	0.044174221	cytokine metabolism
GO:0001817	39	1	0.046510683	regulation of cytokine production
GO:0001505	41	1	0.048841952	regulation of neurotransmitter levels
GO:0001816	42	1	0.050005642	cytokine production
GO:0031175	46	1	0.054647471	neurite morphogenesis
GO:0051093	49	1	0.058115302	negative regulation of development
GO:0006887	55	1	0.065016292	exocytosis
GO:0051248	58	1	0.068449521	negative regulation of protein metabolism
GO:0006800	61	1	0.071871286	oxygen and reactive oxygen species metabolism
GO:0007399	385	2	0.077296646	neurogenesis
GO:0030154	409	2	0.085869951	cell differentiation
GO:0050776	77	1	0.089928495	regulation of immune response
GO:0007275	1543	4	0.102696193	development
GO:0006875	93	1	0.107665763	metal ion homeostasis
GO:0001558	94	1	0.108763836	regulation of cell growth
GO:0008283	473	2	0.110114047	cell proliferation
GO:0050877	475	2	0.110901201	neurophysiological process
GO:0030003	98	1	0.113143856	cation homeostasis
GO:0007417	101	1	0.116416019	central nervous system development
GO:0006873	103	1	0.118591359	cell ion homeostasis
GO:0040008	104	1	0.119677202	regulation of growth
GO:0006417	107	1	0.12292744	regulation of protein biosynthesis
GO:0006813	111	1	0.12724412	potassium ion transport
GO:0031326	111	1	0.12724412	regulation of cellular biosynthesis
GO:0050801	111	1	0.12724412	ion homeostasis
GO:0009889	116	1	0.132612807	regulation of biosynthesis
GO:0008284	121	1	0.137951454	positive regulation of cell proliferation
GO:0019725	122	1	0.139015592	cell homeostasis
GO:0008361	123	1	0.140078536	regulation of cell size
GO:0016049	123	1	0.140078536	cell growth
GO:0051239	133	1	0.15064255	regulation of organismal physiological process
GO:0051243	583	2	0.155603323	negative regulation of cellular physiological process
GO:0008285	139	1	0.156924189	negative regulation of cell proliferation
GO:0006959	141	1	0.159008664	humoral immune response
GO:0043118	602	2	0.163849243	negative regulation of physiological process
GO:0040007	154	1	0.172443947	growth
GO:0045045	161	1	0.179597229	secretory pathway
GO:0048523	640	2	0.180614636	negative regulation of cellular process
GO:0050793	168	1	0.186694255	regulation of development

GO:0051246	174	1	0.192732947	regulation of protein metabolism
GO:0031324	183	1	0.201714587	negative regulation of cellular metabolism
GO:0000902	183	1	0.201714587	cellular morphogenesis
GO:0048519	688	2	0.20223671	negative regulation of biological process
GO:0046903	208	1	0.22618902	secretion
GO:0009892	217	1	0.234831451	negative regulation of metabolism
GO:0015672	223	1	0.240544222	monovalent inorganic cation transport
GO:0007264	227	1	0.244331166	small GTPase mediated signal transduction
GO:0009887	807	2	0.257327433	organogenesis
GO:0006118	256	1	0.271277235	electron transport
GO:0042127	265	1	0.279460403	regulation of cell proliferation
GO:0050874	1531	3	0.282287886	organismal physiological process
GO:0030001	269	1	0.283070478	metal ion transport
GO:0048513	872	2	0.287916943	organ development
GO:0007242	879	2	0.291219858	intracellular signaling cascade
GO:0006950	881	2	0.292163701	response to stress
GO:0016192	296	1	0.307011571	vesicle-mediated transport
GO:0007166	952	2	0.325669283	cell surface receptor linked signal transduction
GO:0006812	346	1	0.349436898	cation transport
GO:0009653	1051	2	0.372097004	morphogenesis
GO:0006810	1802	3	0.380979257	transport
GO:0051234	1841	3	0.395329118	establishment of localization
GO:0006412	407	1	0.397992324	protein biosynthesis
GO:0051242	411	1	0.401058098	positive regulation of cellular physiological process
GO:0051179	1857	3	0.40121294	localization
GO:0043119	428	1	0.413929932	positive regulation of physiological process
GO:0006091	446	1	0.427284386	generation of precursor metabolites and energy
GO:0009613	458	1	0.436033027	response to pest, pathogen or parasite
GO:0043207	463	1	0.439642298	response to external biotic stimulus
GO:0009059	464	1	0.440361628	macromolecule biosynthesis
GO:0000004	465	1	0.441080118	biological process unknown
GO:0006915	471	1	0.445373477	apoptosis
GO:0012501	473	1	0.446797919	programmed cell death
GO:0048522	482	1	0.453166847	positive regulation of cellular process
GO:0007186	491	1	0.459469047	G-protein coupled receptor protein signaling pathway
GO:0008219	498	1	0.464325027	cell death
GO:0016265	501	1	0.466393994	death
GO:0006811	515	1	0.475953456	ion transport
GO:0048518	565	1	0.508837362	positive regulation of biological process
GO:0006955	684	1	0.579692865	immune response
GO:0050791	2407	3	0.594793398	regulation of physiological process
GO:0050896	1578	2	0.59748687	response to stimulus
GO:0006952	751	1	0.615366259	defense response
GO:0044249	763	1	0.621456782	cellular biosynthesis
GO:0016043	842	1	0.659413374	cell organization and biogenesis
GO:0009607	857	1	0.666216634	response to biotic stimulus
GO:0009058	859	1	0.667114339	biosynthesis

GO:0050789	2689	3	0.681510807	regulation of biological process
GO:0009605	963	1	0.710880244	response to external stimulus
GO:0051244	2319	2	0.816644492	regulation of cellular physiological process
GO:0050794	2488	2	0.850482245	regulation of cellular process
GO:0031323	1566	1	0.877032757	regulation of cellular metabolism
GO:0019222	1629	1	0.887984122	regulation of metabolism
GO:0009987	8200	9	0.926601329	cellular process
GO:0044267	2109	1	0.946444009	cellular protein metabolism
GO:0019538	2129	1	0.948122742	protein metabolism
GO:0044260	2298	1	0.960510857	cellular macromolecule metabolism
GO:0043170	2405	1	0.966894296	macromolecule metabolism
GO:0044237	5026	2	0.998076145	cellular metabolism
GO:0008152	5262	2	0.998910758	metabolism
GO:0050875	7018	4	0.999483421	cellular physiological process
GO:0044238	4820	1	0.999774722	primary metabolism
GO:0007582	7846	4	0.999990125	physiological process
GO:0008150	9032	11	1	biological_process

26.3 GO: Molecular Function

GO ID	genes present in chip	genes present in tissues	p-value	GO term
GO:0005138	3	1	0.00408248215626694	interleukin-6 receptor binding
GO:0004971	3	1	0.00408248215626694	alpha-amino-3-hydroxy-5-methyl-4-isoxazole propionate selective glutamate receptor activity
GO:0015277	7	1	0.00950186176156353	kainate selective glutamate receptor activity
GO:0016526	13	1	0.0175799085222755	G-protein coupled receptor activity, unknown ligand
GO:0004970	14	1	0.0189203221651399	ionotropic glutamate receptor activity
GO:0005234	14	1	0.0189203221651399	glutamate-gated ion channel activity
GO:0008066	29	1	0.0388250121976951	glutamate receptor activity
GO:0016209	31	1	0.0414506203171542	antioxidant activity
GO:0005126	38	1	0.050588201212024	hematopoietin/interferon-class (D200-domain) cytokine receptor binding
GO:0005231	39	1	0.0518869861624871	excitatory extracellular ligand-gated ion channel activity
GO:0005507	49	1	0.0647849359612526	copper ion binding
GO:0005230	57	1	0.074986469182564	extracellular ligand-gated ion channel activity
GO:0004871	1837	5	0.0865805414496634	signal transducer activity
GO:0015276	84	1	0.108663179225491	ligand-gated ion channel activity
GO:0005200	86	1	0.111112164373517	structural constituent of cytoskeleton
GO:0005179	90	1	0.115991511886262	hormone activity
GO:0005267	90	1	0.115991511886262	potassium channel activity
GO:0009055	104	1	0.132875199697607	electron carrier activity
GO:0005198	488	2	0.140449115946756	structural molecule activity
GO:0005554	522	2	0.156631883085397	molecular function unknown
GO:0005102	528	2	0.159529814658997	receptor binding
GO:0008083	144	1	0.179488168688739	growth factor activity
GO:0005489	152	1	0.188528573501108	electron transporter activity
GO:0005125	181	1	0.220534909405696	cytokine activity
GO:0005261	188	1	0.228083949498483	cation channel activity
GO:0004888	694	2	0.24325269096627	transmembrane receptor activity
GO:0005506	212	1	0.253457032184693	iron ion binding
GO:0005525	226	1	0.267900168964784	GTP binding
GO:0019001	235	1	0.277048367090188	guanyl nucleotide binding
GO:0005216	252	1	0.294041021532064	ion channel activity
GO:0015268	274	1	0.31548431729917	alpha-type channel activity
GO:0001584	282	1	0.323131637351956	rhodopsin-like receptor activity
GO:0015267	284	1	0.325031105113003	channel or pore class transporter activity
GO:0004930	349	1	0.384150441465452	G-protein coupled receptor activity
GO:0005215	1030	2	0.417286542927301	transporter activity
GO:0005386	398	1	0.425515892099646	carrier activity
GO:0004872	1060	2	0.432291533602076	receptor activity
GO:0008324	421	1	0.444034032059624	cation transporter activity
GO:0015075	523	1	0.519706994800568	ion transporter activity
GO:0005509	646	1	0.598284444452249	calcium ion binding
GO:0005515	3709	4	0.809949466574872	protein binding
GO:0017076	1248	1	0.838565557509774	purine nucleotide binding
GO:0008270	1287	1	0.848168015878051	zinc ion binding

GO:0043169	2313	2	0.860486727116516	cation binding
GO:0000166	1432	1	0.8794334947482	nucleotide binding
GO:0046872	2495	2	0.89127512944214	metal ion binding
GO:0043167	2556	2	0.900239337025935	ion binding
GO:0046914	1596	1	0.907575700648226	transition metal ion binding
GO:0005488	7228	8	0.929678022062054	binding
GO:0003676	1990	1	0.952312907082725	nucleic acid binding
GO:0003674	9541	13	1	molecular_function

26.4 GO: Cellular Component

GO ID	genes present in chip	genes present in tissues	p-value	GO term
GO:0019717	19	1	0.0303837540145840	synaptosome
GO:0005625	183	2	0.0343747947431459	soluble fraction
GO:0030176	26	1	0.0413600041605174	integral to endoplasmic reticulum membrane
GO:0008021	27	1	0.0429185873612792	synaptic vesicle
GO:0031227	32	1	0.0506762699833315	intrinsic to endoplasmic reticulum membrane
GO:0009986	61	1	0.0945314765141463	cell surface
GO:0000267	698	3	0.0979680994683056	cell fraction
GO:0045111	65	1	0.100430617865032	intermediate filament cytoskeleton
GO:0005882	65	1	0.100430617865032	intermediate filament
GO:0030136	65	1	0.100430617865032	clathrin-coated vesicle
GO:0045211	66	1	0.101899816575938	postsynaptic membrane
GO:0031301	76	1	0.116469755423438	integral to organelle membrane
GO:0030135	79	1	0.120797747549585	coated vesicle
GO:0005789	84	1	0.127967345708868	endoplasmic reticulum membrane
GO:0031300	90	1	0.136499193008033	intrinsic to organelle membrane
GO:0042175	90	1	0.136499193008033	nuclear envelope-endoplasmic reticulum network
GO:0045202	98	1	0.147754443585539	synapse
GO:0016020	3514	8	0.162858985365259	membrane
GO:0016023	122	1	0.180708160586629	cytoplasmic membrane-bound vesicle
GO:0031410	124	1	0.183400193219098	cytoplasmic vesicle
GO:0005576	949	3	0.193297163150096	extracellular region
GO:0005624	521	2	0.205298778790423	membrane fraction
GO:0008372	523	2	0.206505091481407	cellular component unknown
GO:0016021	2674	6	0.244152924121058	integral to membrane
GO:0031224	2681	6	0.246302068190529	intrinsic to membrane
GO:0012505	246	1	0.332921519374537	endomembrane system
GO:0005578	303	1	0.393680503427150	extracellular matrix (sensu Metazoa)
GO:0031012	304	1	0.394699038698571	extracellular matrix
GO:0031090	377	1	0.464905212695089	organelle membrane
GO:0005615	385	1	0.47212087950958	extracellular space
GO:0005783	483	1	0.553483316383632	endoplasmic reticulum
GO:0005856	612	1	0.64289135161834	cytoskeleton
GO:0005886	1472	2	0.716862972164652	plasma membrane
GO:0043228	977	1	0.813989425160123	non-membrane-bound organelle
GO:0043232	977	1	0.813989425160123	intracellular non-membrane-bound organelle
GO:0005887	1057	1	0.839435614333776	integral to plasma membrane
GO:0031226	1062	1	0.840913320207642	intrinsic to plasma membrane
GO:0005623	7802	12	0.853020384925633	cell
GO:0005737	2530	2	0.94704534890996	cytoplasm
GO:0043226	4515	3	0.996032812809068	organelle
GO:0043229	4515	3	0.996032812809068	intracellular organelle
GO:0043227	3955	2	0.997588443498243	membrane-bound organelle
GO:0043231	3955	2	0.997588443498243	intracellular membrane-bound organelle

GO:0005622	5419	4	0.998007694806662	intracellular
GO:0005575	8637	14	1	cellular_component

27. Appendix

27.1 Appendix selective genes (5 genes)

Symbol	Description	Chromosome	Chromosome Location	LocusLink	Cytoband	UniGene
FABP4	fatty acid binding protein 4, adipocyte	8	-82553489	2167	8q21	Hs.391561
IGHA1	immunoglobulin heavy constant alpha 1	14	NA	3493	14q32.33	Hs.510635
ACTG2	actin, gamma 2, smooth muscle, enteric	2	73973600	72	2p13.1	Hs.516105
ADH1B	alcohol dehydrogenase IB (class I), beta polypeptide	4	-100446551	125	4q21-q23	Hs.4
TAGLN	transgelin	11	116575249	6876	11q23.2	Hs.632099

27.2 GO: Biological Process

GO ID	genes present in chip	genes present in tissues	p-value	GO term
GO:0006067	4	1	0.001770597	ethanol metabolism
GO:0006069	4	1	0.001770597	ethanol oxidation
GO:0018298	7	1	0.003097	protein-chromophore linkage
GO:0019883	21	1	0.009269415	antigen presentation, endogenous antigen
GO:0019885	22	1	0.009709202	antigen processing, endogenous antigen via MHC class I
GO:0030333	36	1	0.015850862	antigen processing
GO:0019882	41	1	0.018037373	antigen presentation
GO:0007517	94	1	0.040991071	muscle development
GO:0006066	213	1	0.091061112	alcohol metabolism
GO:0006955	684	1	0.270256222	immune response
GO:0006952	751	1	0.293407129	defense response
GO:0009887	807	1	0.312330763	organogenesis
GO:0009607	857	1	0.32890334	response to biotic stimulus
GO:0048513	872	1	0.33381618	organ development
GO:0009653	1051	1	0.390385168	morphogenesis
GO:0006464	1087	1	0.401312201	protein modification
GO:0043412	1160	1	0.423018218	biopolymer modification
GO:0050874	1531	1	0.524355977	organismal physiological process
GO:0007275	1543	1	0.527393002	development
GO:0050896	1578	1	0.53616796	response to stimulus
GO:0006810	1802	1	0.589469389	transport
GO:0051234	1841	1	0.598257705	establishment of localization
GO:0051179	1857	1	0.601822042	localization
GO:0044267	2109	1	0.654893058	cellular protein metabolism
GO:0019538	2129	1	0.658864613	protein metabolism
GO:0043283	2240	1	0.680287353	biopolymer metabolism
GO:0044260	2298	1	0.691071305	cellular macromolecule metabolism
GO:0043170	2405	1	0.710247338	macromolecule metabolism
GO:0044237	5026	2	0.76713537	cellular metabolism
GO:0050875	7018	3	0.782977561	cellular physiological process
GO:0008152	5262	2	0.800223	metabolism
GO:0007582	7846	3	0.913805767	physiological process
GO:0044238	4820	1	0.952740682	primary metabolism
GO:0009987	8200	3	0.955160628	cellular process
GO:0008150	9032	4	1	biological_process

27.3 GO: Molecular Function

GO ID	genes present in chip	genes present in tissues	p-value	GO term
GO:0004024	4	1	0.00209489823087861	alcohol dehydrogenase activity, zinc-dependent
GO:0004022	8	1	0.00418628411098643	alcohol dehydrogenase activity
GO:0005504	17	1	0.00887908318924258	fatty acid binding
GO:0030106	26	1	0.0135541736151249	MHC class I receptor activity
GO:0003823	30	1	0.0156263204475630	antigen binding
GO:0016616	77	1	0.0397142721897678	oxidoreductase activity, acting on the CH-OH group of donors, NAD or NADP as acceptor
GO:0016614	83	1	0.0427550732591464	oxidoreductase activity, acting on CH-OH group of donors
GO:0005200	86	1	0.0442725823833281	structural constituent of cytoskeleton
GO:0009055	104	1	0.0533372728177547	electron carrier activity
GO:0005489	152	1	0.0771739518975045	electron transporter activity
GO:0003779	189	1	0.095218297020885	actin binding
GO:0008092	279	1	0.137933660140605	cytoskeletal protein binding
GO:0008289	298	1	0.146741534601651	lipid binding
GO:0005386	398	1	0.191920110613965	carrier activity
GO:0005198	488	1	0.230925205882841	structural molecule activity
GO:0016491	509	1	0.239805882110832	oxidoreductase activity
GO:0005488	7228	5	0.249445414322668	binding
GO:0004888	694	1	0.314551329509611	transmembrane receptor activity
GO:0005524	970	1	0.415026133574471	ATP binding
GO:0030554	1028	1	0.434557168589567	adenyl nucleotide binding
GO:0005215	1030	1	0.435221223533303	transporter activity
GO:0004872	1060	1	0.44510740906054	receptor activity
GO:0017076	1248	1	0.503955859330953	purine nucleotide binding
GO:0008270	1287	1	0.515513316588122	zinc ion binding
GO:0000166	1432	1	0.556609267013864	nucleotide binding
GO:0046914	1596	1	0.599678829125343	transition metal ion binding
GO:0005515	3709	2	0.643369949373103	protein binding
GO:0004871	1837	1	0.656834741002604	signal transducer activity
GO:0043169	2313	1	0.750554585677332	cation binding
GO:0046872	2495	1	0.780425211733226	metal ion binding
GO:0043167	2556	1	0.789769392346009	ion binding
GO:0003824	3409	1	0.890405667786476	catalytic activity
GO:0003674	9541	5	1	molecular_function

27.4 GO: Cellular Component

GO ID	genes present in chip	genes present in tissues	p-value	GO term
GO:0005884	17	1	0.0058938944683744	actin filament
GO:0015629	183	1	0.0622335186026003	actin cytoskeleton
GO:0005625	183	1	0.0622335186026003	soluble fraction
GO:0005856	612	1	0.197888287243506	cytoskeleton
GO:0000267	698	1	0.223403584507221	cell fraction
GO:0043228	977	1	0.302445240981168	non-membrane-bound organelle
GO:0043232	977	1	0.302445240981168	intracellular non-membrane-bound organelle
GO:0005737	2530	1	0.646546331900839	cytoplasm
GO:0016021	2674	1	0.67096809818503	integral to membrane
GO:0031224	2681	1	0.672125689694711	intrinsic to membrane
GO:0005622	5419	2	0.687008841674662	intracellular
GO:0005623	7802	3	0.737077121967901	cell
GO:0016020	3514	1	0.791368053158858	membrane
GO:0043226	4515	1	0.891339944420853	organelle
GO:0043229	4515	1	0.891339944420853	intracellular organelle
GO:0005575	8637	3	1	cellular_component

28. TrigeminalGanglion

28.1 TrigeminalGanglion selective genes (1 gene)

Symbol	Description	Chromosome	Chromosome Location	LocusLink	Cytoband	UniGene
NEFH	neurofilament, heavy polypeptide 200kDa	22	28206218	4744	22q12.2	Hs.198760

28.2 GO: Biological Process

GO ID	genes present in chip	genes present in tissues	p-value	GO term
GO:0006334	48	1	0.005314438	nucleosome assembly
GO:0031497	56	1	0.006200177	chromatin assembly
GO:0006333	91	1	0.010075288	chromatin assembly or disassembly
GO:0006325	186	1	0.020593446	establishment and/or maintenance of chromatin architecture
GO:0006323	193	1	0.021368468	DNA packaging
GO:0006461	194	1	0.021479185	protein complex assembly
GO:0007001	221	1	0.024468556	chromosome organization and biogenesis (sensu Eukaryota)
GO:0051276	236	1	0.026129318	chromosome organization and biogenesis
GO:0007399	385	1	0.042626218	neurogenesis
GO:0006259	489	1	0.054140833	DNA metabolism
GO:0006996	693	1	0.076727192	organelle organization and biogenesis
GO:0009887	807	1	0.089348981	organogenesis
GO:0016043	842	1	0.093224092	cell organization and biogenesis
GO:0048513	872	1	0.096545616	organ development
GO:0009653	1051	1	0.116364039	morphogenesis
GO:0007275	1543	1	0.170837024	development
GO:0044267	2109	1	0.2335031	cellular protein metabolism
GO:0019538	2129	1	0.235717449	protein metabolism
GO:0043283	2240	1	0.248007086	biopolymer metabolism
GO:0044260	2298	1	0.254428698	cellular macromolecule metabolism
GO:0006139	2328	1	0.257750221	nucleobase, nucleoside, nucleotide and nucleic acid metabolism
GO:0043170	2405	1	0.266275465	macromolecule metabolism
GO:0044238	4820	1	0.533658105	primary metabolism
GO:0044237	5026	1	0.556465899	cellular metabolism
GO:0008152	5262	1	0.582595217	metabolism
GO:0050875	7018	1	0.777015058	cellular physiological process
GO:0007582	7846	1	0.868689105	physiological process
GO:0009987	8200	1	0.907883082	cellular process
GO:0008150	9032	1	1	biological_process

28.3 GO: Molecular Function

GO ID	genes present in chip	genes present in tissues	p-value	GO term
GO:0005198	488	1	0.0511476784404141	structural molecule activity
GO:0005554	522	1	0.0547112462006071	molecular function unknown
GO:0003677	1384	1	0.145058170003144	DNA binding
GO:0003676	1990	1	0.208573524787757	nucleic acid binding
GO:0005488	7228	1	0.75757258149041	binding
GO:0003674	9541	1	1	molecular_function

28.4 GO: Cellular Component

GO ID	genes present in chip	genes present in tissues	p-value	GO term
GO:0005883	5	1	0.000578904712284833	neurofilament
GO:0030424	7	1	0.000810466597198967	axon
GO:0043005	17	1	0.0019682760217673	neuron projection
GO:0000786	40	1	0.00463123769827578	nucleosome
GO:0045111	65	1	0.0075257612596975	intermediate filament cytoskeleton
GO:0005882	65	1	0.0075257612596975	intermediate filament
GO:0042995	70	1	0.0081046659719819	cell projection
GO:0000785	100	1	0.0115780942456877	chromatin
GO:0005694	203	1	0.0235035313187463	chromosome
GO:0005856	612	1	0.0708579367836064	cytoskeleton
GO:0043228	977	1	0.113117980780364	non-membrane-bound organelle
GO:0043232	977	1	0.113117980780364	intracellular non-membrane-bound organelle
GO:0043234	1247	1	0.144378835243720	protein complex
GO:0005634	2704	1	0.313071668403381	nucleus
GO:0043227	3955	1	0.457913627416928	membrane-bound organelle
GO:0043231	3955	1	0.457913627416928	intracellular membrane-bound organelle
GO:0043226	4515	1	0.522750955192776	organelle
GO:0043229	4515	1	0.522750955192776	intracellular organelle
GO:0005622	5419	1	0.627416927173788	intracellular
GO:0005623	7802	1	0.903322913048512	cell
GO:0005575	8637	1	1	cellular_component

29. TemporalLobe

29.1 TemporalLobe selective genes (23 genes)

Symbol	Description	Chromosome	Chromosome Location	LocusLink	Cytoband	UniGene
CCK	cholecystokinin	3	-42274321	885	3p22-p21.3	Hs.458426
GRIA2	glutamate receptor, ionotropic, AMPA 2	4	158361296	2891	4q32-q33	Hs.32763
SNAP25	synaptosomal-associated protein, 25kDa	20	10147476	6616	20p12-p11.2	Hs.167317
MT3	metallothionein 3 (growth inhibitory factor (neurotrophic))	16	55180767	4504	16q13	Hs.73133
GPM6A	glycoprotein M6A	4	-176791082	2823	4q34	Hs.75819
SPP1	secreted phosphoprotein 1 (osteopontin, bone sialoprotein I, early T-lymphocyte activation 1)	4	89115825	6696	4q21-q25	Hs.313
RTN1	reticulon 1	14	-59132446	6252	14q23.1	Hs.368626
STMN2	stathmin-like 2	8	80685934	11075	8q21.13	Hs.521651
DIRAS2	DIRAS family, GTP-binding RAS-like 2	9	-92411933	54769	9q22.2	Hs.165636
PLP1	proteolipid protein 1 (Pelizaeus-Merzbacher disease, spastic paraplegia 2, uncomplicated)	X	102918409	5354	Xq22	Hs.1787
GFAP	glial fibrillary acidic protein	17	-40338518	2670	17q21	Hs.514227
VSNL1	visinin-like 1	2	17585287	7447	2p24.3	Hs.444212
DNM1	dynamin 1	9	130005483	1759	9q34	Hs.522413
EEF1A2	eukaryotic translation elongation factor 1 alpha 2	20	-61589809	1917	20q13.3	Hs.433839
NRGN	neurogranin (protein kinase C substrate, RC3)	11	124114951	4900	11q24	Hs.524116
SYT1	synaptotagmin I	12	78135413	6857	12cen-q21	Hs.310545
NAP1L3	nucleosome assembly protein 1-like 3	X	-92812589	4675	Xq21.3-q22	Hs.21365
DCAMKL1	doublecortin and CaM kinase-like 1	13	-35243477	9201	13q13	Hs.507755
CAMK2B	calcium/calmodulin-dependent protein kinase (CaM kinase) II beta	7	-44225421	816	22q12|7p14.3-p14.1	Hs.351887
SCG5	secretogranin V (7B2 protein)	15	30721251	6447	15q13-q14	Hs.156540
SH3GL2	SH3-domain GRB2-like 2	9	17569193	6456	9p22	Hs.75149
NEF3	neurofilament 3 (150kDa medium)	8	24827211	4741	8p21	Hs.458657 Hs.615287
GAP43	growth associated protein 43	3	116825141	2596	3q13.1-q13.2	Hs.134974

29.2 GO: Biological Process

GO ID	genes present in chip	genes present in tissues	p-value	GO term
GO:0007154	2730	15	4.72E-06	cell communication
GO:0046883	8	2	0.000104299	regulation of hormone secretion
GO:0007267	465	6	0.000196894	cell-cell signaling
GO:0046879	11	2	0.000204148	hormone secretion
[truncated: 503,580 more chars]
