# Supplementary material for: Physiology, Pathology and Relatedness of Human Tissues from Gene Expression Meta-Analysis
Source: PLoS One. 2008 Apr 2;3(4):e1880. doi: 10.1371/journal.pone.0001880 (PMC2268968; doi:10.1371/journal.pone.0001880)
Supplement: File S3 — (0.17 MB PDF) [file pone.0001880.s006.pdf]

D. Greco et al.
Supplemental Material Volume 2

Hierarchical Clustering (Figure 1 and 2)

Hierarchical clustering (HC) is a widely used method for grouping numeric entities according to their similarity. The similarity of objects can be measured in terms of statistical correlation or as a geometric distance measured in the euclidian space. The objects are visualized as leaves of a tree and their similarity is represented by their position with the respect of the root of the tree, which represents the cluster of all the objects together.
We have employed hierarchical clustering for visualizing the similarity of the 78 tissues analyzed. For this scope, we have computed the euclidian distance of all the tissue pairs. Distances are also shown as gray-scale heatmap, where the minimum distance between two tissues (identity) is represented by the color black, and the maximum distance as color white.

Principal Component Analysis (Figure 3 and 4)

Principal component analysis (PCA) is a linear multivariate data analysis method which can be used for data dimensionality reduction and visualization. It is based on maximizing the variance of the data projection. PCA was used here for visually revealing the potential data clusters. The steps involved in PCA are to compute the eigenvalues and eigenvectors of the covariance matrix of the data. Original data vectors are then projected into the principal components, i.e., eigenvectors with largest eigenvalues. For visualization purpose, we used three principal components. In PCA, the order of the principal components has interpretation as the first component explains the most of the data variance, the second component explains the most of the remaining variance and so on.

Curvilinear Component Analysis (Figure 5 and 6)

There are several algorithms which can be classified to the broad category of multi-dimensional scaling (MDS) methods. The projection from the original data space to the new space can be nonlinear. The general idea in these methods is to project the data from the originally high-dimensional space into the low-dimensional space so that the original pair-wise distances of data are preserved as carefully as possible in the new space. In this work we used the algorithm from Curvilinear Component Analysis. The dimensionality of the projection space was chosen to be three so that the visualization could be done conveniently. Since only distances are considered, the order of the output coordinates does not have similar meaning as in PCA.

Figure 1

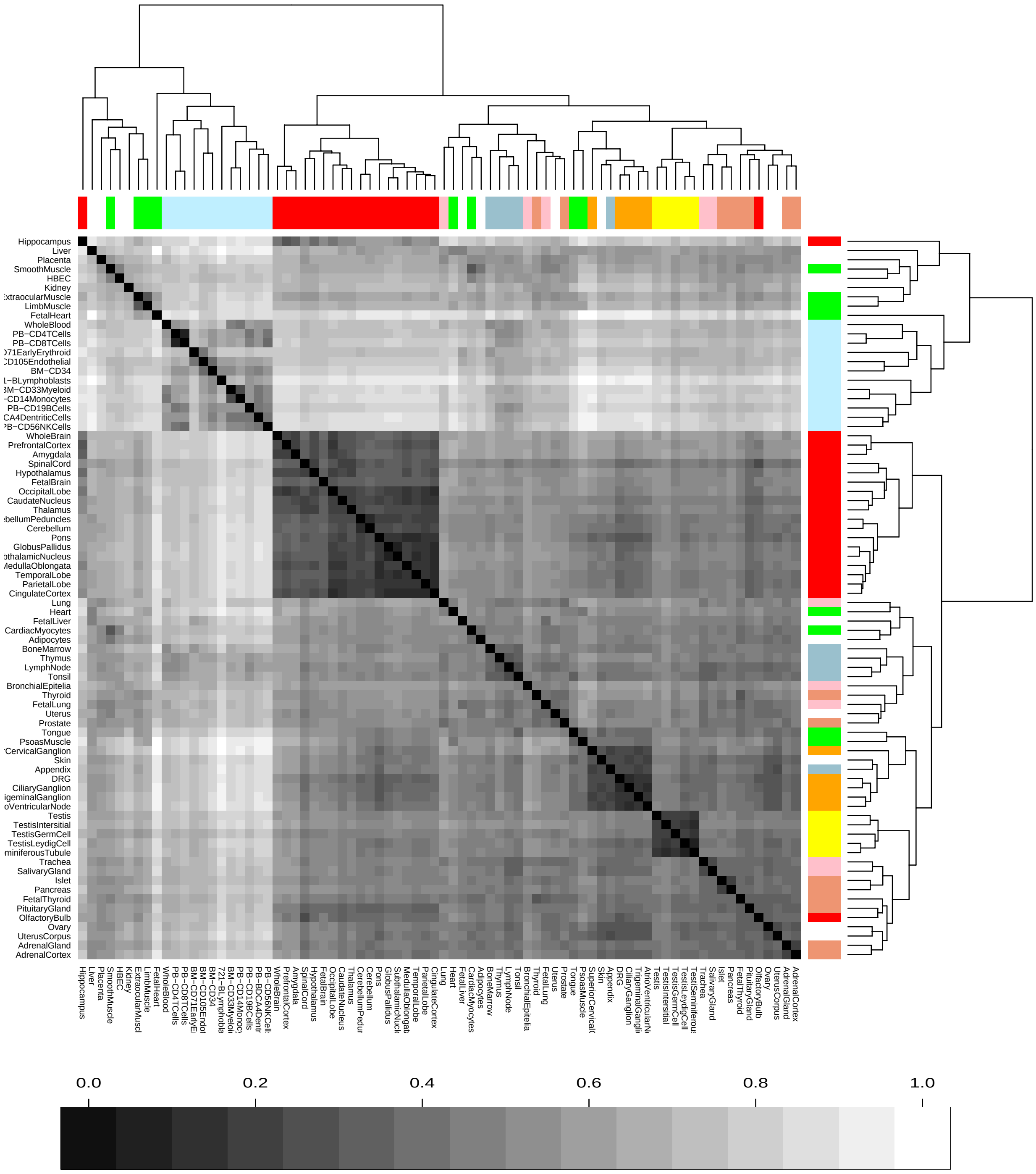
Hippocampus
Liver
Placenta
SmoothMuscle
HBEC
Kidney
xtraocularMuscle
LimbMuscle
FetalHeart
WholeBlood
PB−CD4TCells
PB−CD8TCells
71EarlyErythroid
CD105Endothelial
BM−CD34
L−BLymphoblasts
3M−CD33Myeloid
−CD14Monocytes
PB−CD19BCells
CA4DentriticCells
PB−CD56NKCells
WholeBrain
PrefrontalCortex
Amygdala
SpinalCord
Hypothalamus
FetalBrain
OccipitalLobe
CaudateNucleus
Thalamus
bellumPeduncles
Cerebellum
Pons
GlobusPallidus
othalamicNucleus
MedullaOblongata
TemporalLobe
ParietalLobe
CingulateCortex
Lung
Heart
FetalLiver
CardiacMyocytes
Adipocytes
BoneMarrow
Thymus
LymphNode
Tonsil
BronchialEpitelia
Thyroid
FetalLung
Uterus
Prostate
Tongue
PsoasMuscle
CervicalGanglion
Skin
Appendix
DRG
CiliaryGanglion
igeminalGanglion
oVentricularNode
Testis
TestisIntersitial
TestisGermCell
TestisLeydigCell
miniferousTubule
Trachea
SalivaryGland
Islet
Pancreas
FetalThyroid
PituitaryGland
OlfactoryBulb
Ovary
UterusCorpus
AdrenalGland
AdrenalCortex
0.0
0.2
0.4
0.6
0.8
1.0

Figure 2

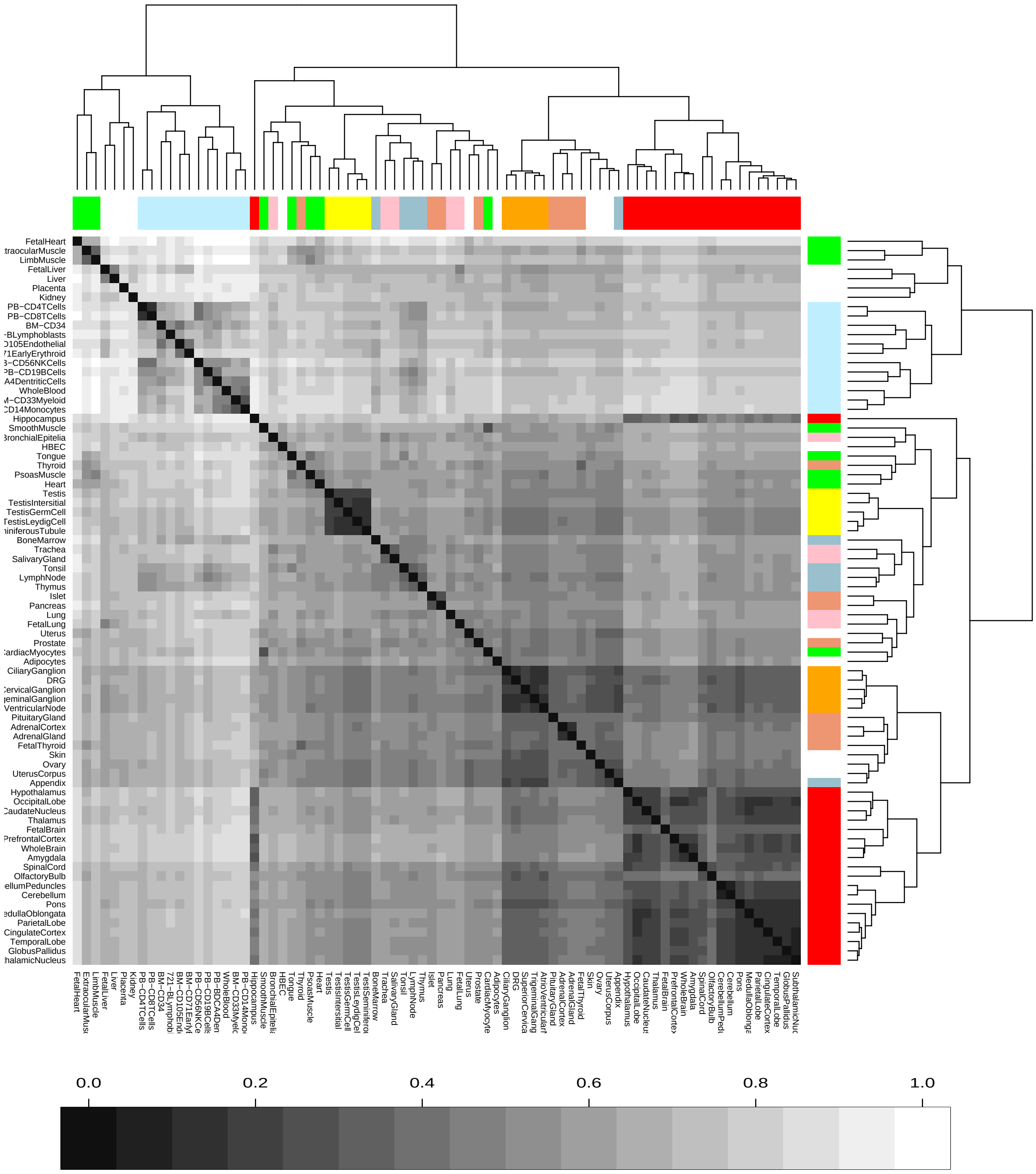

Figure 3

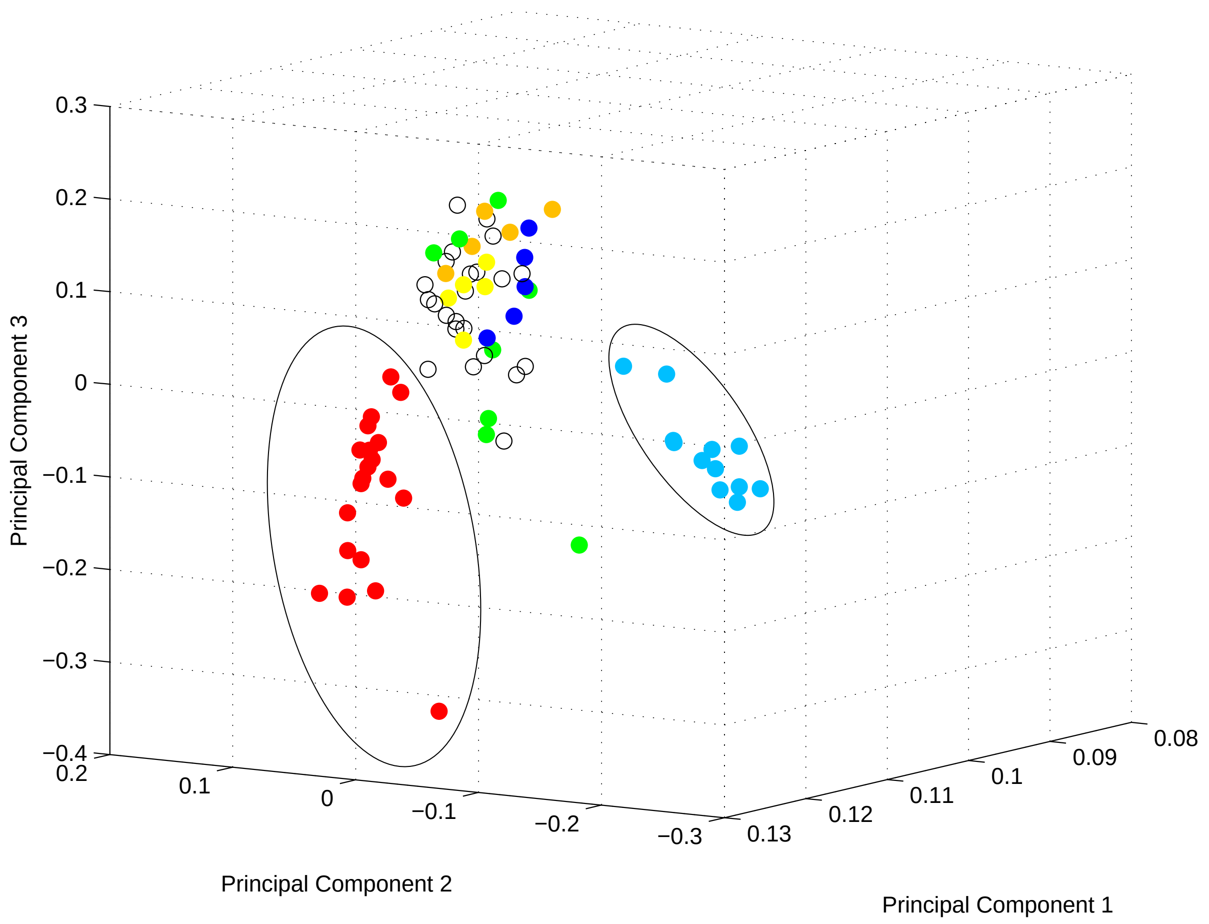
0.3
0.2
0.1
0
−0.1
−0.2
−0.3
−0.4
Principal Component 3
0.2
0.1
0
−0.1
−0.2
−0.3
Principal Component 2
0.13
0.12
0.11
0.1
0.09
0.08
Principal Component 1

Figure 4

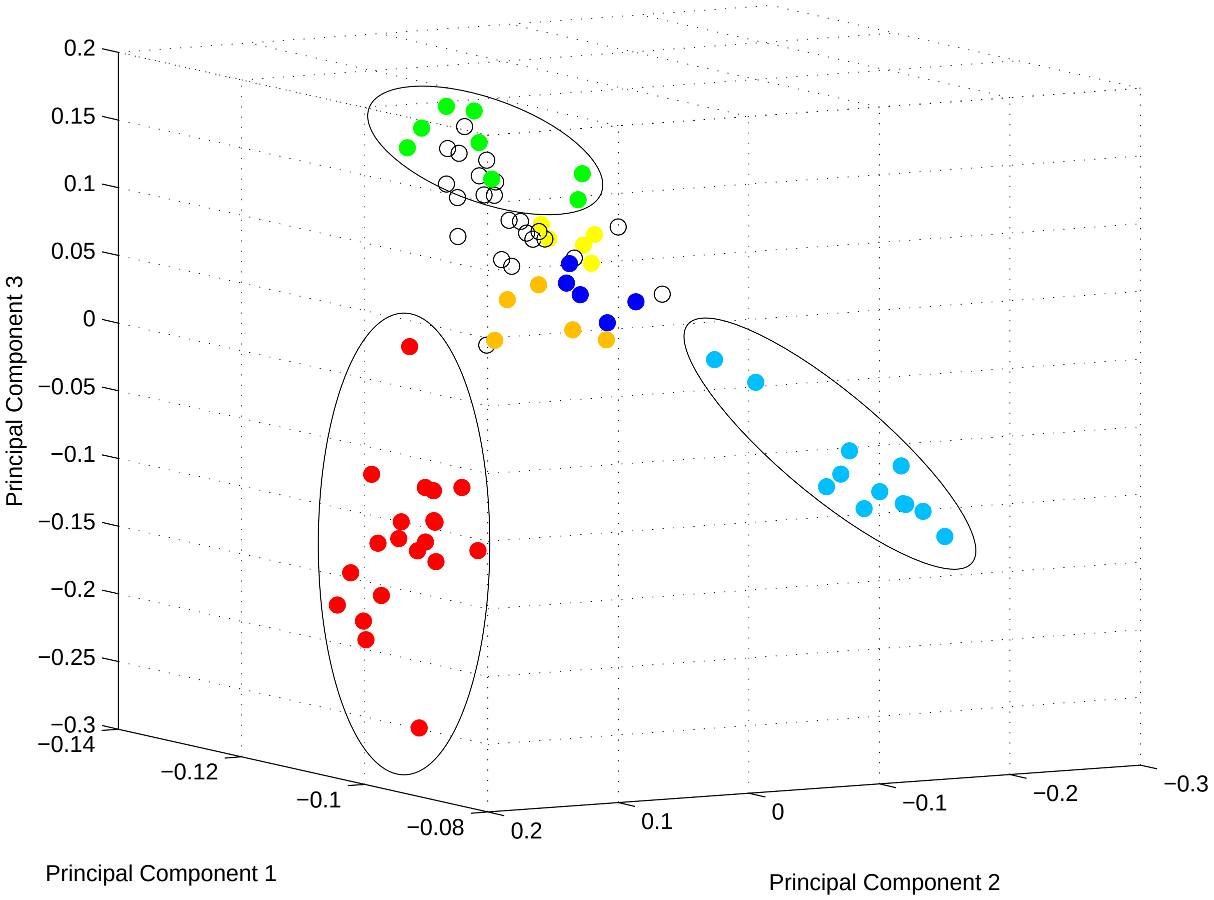

Principal Component 3
0.2
0.15
0.1
0.05
0
−0.05
−0.1
−0.15
−0.2
−0.25
−0.3
−0.14
−0.12
−0.1
−0.08
0.2
0.1
0
−0.1
−0.2
−0.3
Principal Component 1
Principal Component 2

Figure 5

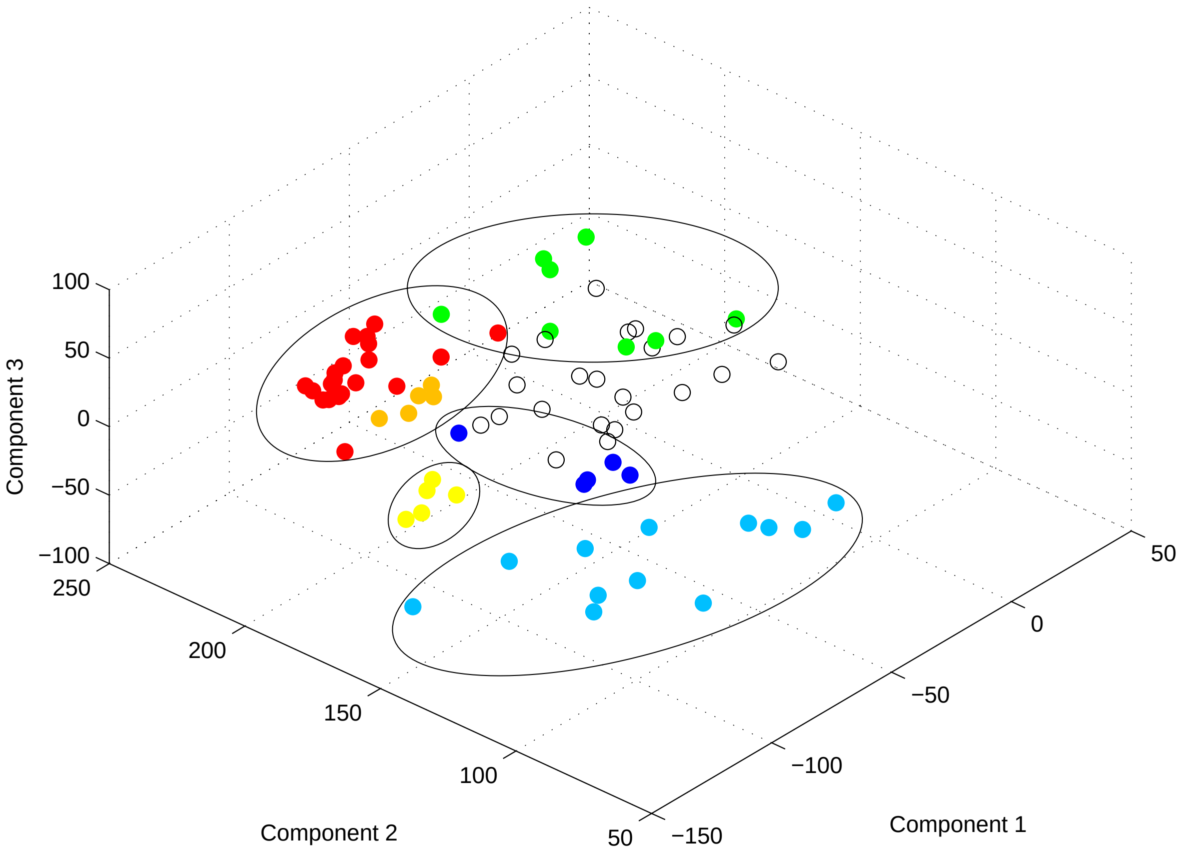

100
50
0
−50
−100
Component 3
250
200
150
100
50
Component 2
−150
−100
−50
0
50
Component 1

Figure 6

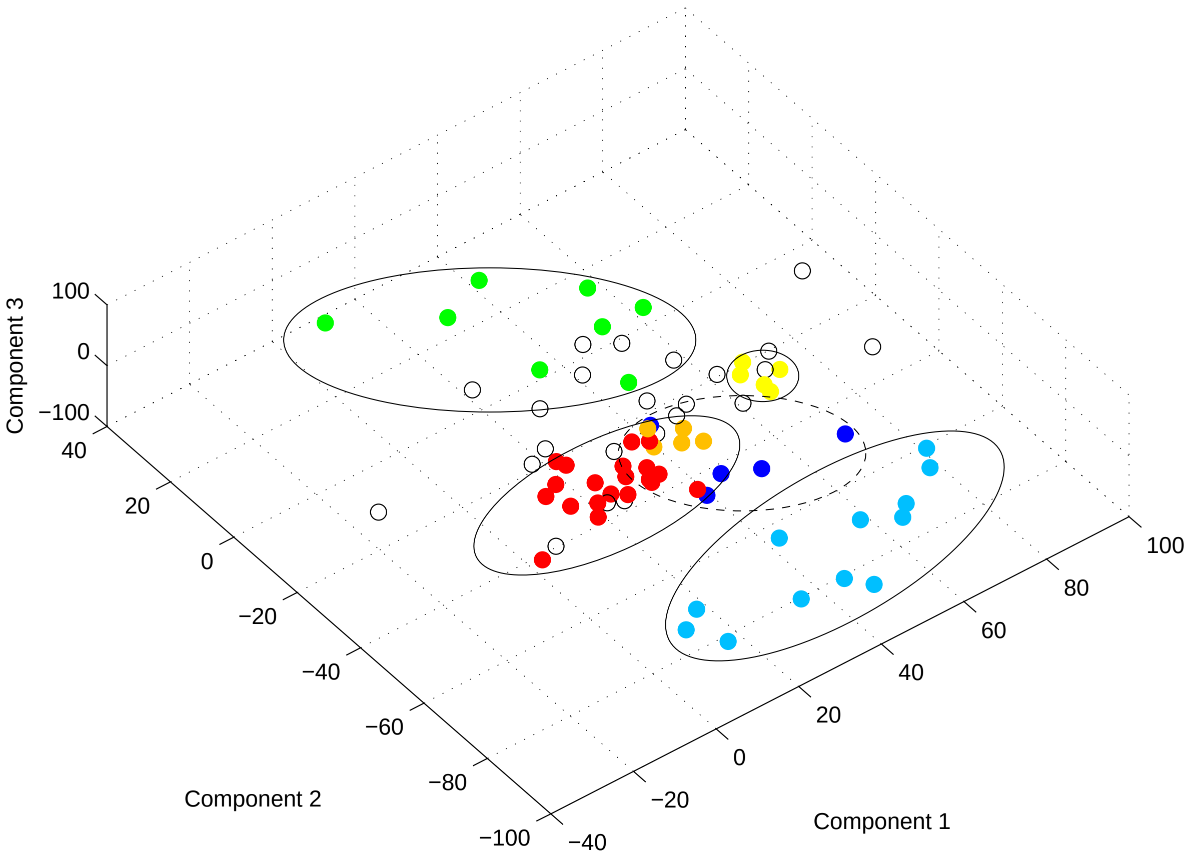
Component 3
100
0
−100
40
20
0
−20
−40
−60
−80
−100
Component 2
−40
−20
0
20
40
60
80
100
Component 1

Legends.

Figure 1 – Hierarchical clustering of tissues based on the expression of all the 12201 genes analyzed.
The color bars near by the trees indicate the tissue-color-code as in Table 1. Pair-wise Euclidian distances are shown as gray-scale heatmap between black (identity) to white. As shown by the hierarchical trees, the 78 tissues analyzed fall into two main branches. In the first one, immune cells (light blue) cluster together with liver, placenta, kidney, hippocampus, and with extraocular muscle, smooth muscle, limb muscle, and fetal heart (green). In the second main branch are placed the central nervous system (red) and peripheral nervous system tissues (orange), the glands (salmon), respiratory system tissues (pink)and muscle tissues heart, cardiac myocites, tongue, and psoas (green). Interestingly, olfactory bulb (red) is not together with all the other central nervous system tissues, but it clusters together with pituitary gland (salmon). Similarly, appendix (dark blue) is placed under the same branch of skin and the peripheral nervous tissues (orange).

Figure 2 – Hierarchical clustering of tissues based on the expression of the 1601 tissue-selective genes.
The color bars near by the trees indicate the tissue-color-code as in Table 1. Pair-wise Euclidian distances are shown as gray-scale heatmap between black (identity) to white. As shown by the hierarchical trees, the 78 tissues analyzed fall into two main branches. In the first one, immune cells (light blue) cluster is close to a cluster consisting of fetal heart, extraocular muscle, and limb muscle (green), and a second small cluster formed by fetal liver, liver, placenta, and kidney.
I the second bigger branch, central nervous tissues (red) cluster is close to other two smaller groups of tissues, consisted respectively of: peripheral nervous tissues (orange); appendix, uterus, ovary, skin, fetal thyroid and adrenal gland. In this last branch, adrenal gland tissues (salmon) show big similarity to peripheral nervous tissues (orange). Testis tissues (yellow) cluster together in a very compact way.

Figure 3 – Principal Component Analysis of tissues based on the expression of all 12201 genes analyzed.
Each tissue is represented as a spot in the three dimensional space, based on the first three principal components. The immune cells (light blue, circled) and the central nervous tissues (red, circled) are very well separated along the first and the second principal components, respectively. A minor degree of separation is shown by muscles (green), testis (yellow), and peripheral nervous tissues (orange) along the third component.

Figure 4 – Principal Component Analysis of tissues based on the expression of the 1601 tissue-selective genes.
Each tissue is represented as a spot in the three dimensional space, based on the first three principal components.
Central nervous tissues (red, circled) and peripheral nervous tissues (orange) are well separated from all the other tissues along the first principal component. Central nervous tissues (red) show clearer distinction as they are placed farther. On the second principal component, the immune cells (light blue, circled) and, less clearly, immune organs (dark blue) segregate from all the others. Muscle tissues (green, circled) and testis tissues (yellow) are segregated along the third principal component.

Figure 5 – Curvilinear Component Analysis of tissues based on the expression of all the 12201genes analyzed in the dataset.
Each tissue is represented as a spot in the three dimensional space.
The muscle tissues (green, circled) are segregated on the component 1, while immune cells (light blue, circled), immune tissues (dark blue, circled), testis (yellow, circled), peripheral nervous tissues (orange, circled), and central nervous tissues (red, circled) are well separated along the component 2.

Figure 6 – Curvilinear Component Analysis of tissues based on the expression of the 1601 tissue-selective genes.
Each tissue is represented as a spot in the three dimensional space.
The highlighted groups of tissues segregate clearly on the component 2, with immune cells (light blue, circled) and muscles (green, circled) to the two extremes, and central nervous tissues (red, circled) and testis (yellow, circled) in the middle. Testis (yellow) is shown distant from nervous tissues on the component 1.

Table 1

tissue	*tissue-color-code*	*tissue*	*tissue-color-code*
Hippocampus	red	Ovary	white
BronchialEpitelia	pink	Thyroid	lightsalmon2
LimbMuscle	green	Lung	pink
ExtraocularMuscle	green	FetalBrain	red
Kidney	white	Liver	white
SubthalamicNucleus	red	LymphNode	lightblue3
Skin	white	Amygdala	red
GlobusPallidus	red	Heart	green
CiliaryGanglion	orange	Uterus	white
AtrioVentricularNode	orange	Prostate	lightsalmon2
DRG	orange	Pancreas	lightsalmon2
Placenta	white	PrefrontalCortex	red
721-BLymphoblasts	lightblue1	CingulateCortex	red
PB-CD8TCells	lightblue1	Thymus	lightblue3
PB-CD4TCells	lightblue1	FetalLiver	white
BM-CD71EarlyErythroid	lightblue1	Testis	yellow
PB-CD14Monocytes	lightblue1	Trachea	pink
PB-CD56NKCells	lightblue1	AdrenalGland	lightsalmon2
PB-CD19BCells	lightblue1	SpinalCord	red
BM-CD33Myeloid	lightblue1	Cerebellum	red
BM-CD105Endothelial	lightblue1	PituitaryGland	lightsalmon2
BM-CD34	lightblue1	Thalamus	red
PB-BDCA4DentriticCells	lightblue1	BoneMarrow	lightblue3
SuperiorCervicalGanglion	orange	CardiacMyocytes	green
MedullaOblongata	red	FetalThyroid	lightsalmon2
Pons	red	OlfactoryBulb	red
Appendix	lightblue3	TestisGermCell	yellow
TrigeminalGanglion	orange	TestisIntersitial	yellow
TemporalLobe	red	TestisLeydigCell	yellow
Tongue	green	Hypothalamus	red
UterusCorpus	white	OccipitalLobe	red
PsoasMuscle	green	CerebellumPeduncles	red
FetalLung	pink	SmoothMuscle	green
ParietalLobe	red	CaudateNucleus	red
Tonsil	lightblue3	WholeBrain	red
WholeBlood	lightblue1	Islet	lightsalmon2
HBEC	white	Adipocytes	white
SalivaryGland	pink	TestiSeminiferousTubule	yellow
AdrenalCortex	lightsalmon2	FetalHeart	green
